# A generalized outlier method with an automated test protocol applied to proficiency testing schemes: utilizing Monte Carlo simulation for generating critical values for the extended Grubbs statistic

**DOI:** 10.1093/jaoacint/qsag036

**Published:** 2026-04-29

**Authors:** Tomas Nilsson, Simon Langer Sigaard

**Affiliations:** FOSS Analytical A/S, Technical Product Management, Nils Foss Allé 1, Hillerød 3400, Denmark; FOSS Analytical A/S, R&D: DataScience & MachineLearning, Nils Foss Allé 1, Hillerød 3400, Denmark

## Abstract

**Background:**

A generalized method for addressing outliers in proficiency testing (PT) schemes is presented, along with examples illustrating their impact on the analysis used for payment in the grain industry.

**Objective:**

Our aim is to enhance the accuracy and robustness of assigned values in the newly proposed automated testing protocol, and to provide added value to participants in PT schemes by enhancing the significance of outlier detection, enabling further investigation of the underlying causes.

**Methods:**

The Grubbs outlier detection method has been expanded to include 35 distinct tests for identifying single, double, and up to multiple-10 outliers. Monte Carlo simulations have been used to determine the critical values for each test, which are suitable for datasets containing up to 1000 observations. This research specifically focuses on the one-sided significance level of 1.25%, but six other levels are included. The application of this method is particularly relevant for analyzing PT scheme data within the grain industry.

**Results:**

The study discusses the challenges posed by extreme outlier data that may have identical values due to rounding practices. Additionally, another example from the world′s largest grain network in France shows a multiple-5 outlier in a large dataset of over 700 observations.

**Conclusion:**

This extended Grubbs test provides a more accurate determination of mean values and facilitates the identification of outlying observations, which may reveal significant insights when confirmed to be statistically distinct.

**Highlights:**

The importance of precise testing using the new protocol is underscored by the observation that even a minimal shift of 0.1% in protein content in barley could lead to financial implications amounting to millions of US dollars. Notably, a minor shift of 0.07% was observed when applying the quadruple outlier test that excluded the four highest observations from the PT scheme dataset.

## Introduction

Near-infrared (NIR) spectroscopy is widely used in the grain industry for payment purposes, particularly for measuring parameters such as moisture and protein content in barley. A shift in protein determination as minimal as 0.1% can have financial implications worth millions of US dollars. Consequently, it is imperative that NIR devices are meticulously validated against established reference methods and integrated into a comprehensive quality assurance framework. One such framework is the proficiency testing (PT) scheme, wherein analytical methodologies or instruments are assessed against assigned values derived from the testing material, typically calculated as the mean of observations after excluding outliers. Numerous outlier rejection methods exist, among which the Grubbs statistic is recognized as a powerful tool for identifying outlying observations in statistical assessments (1), such as those conducted in PT schemes. An essential component of PT schemes is acquiring a robust mean estimate, which often serves as the assigned value for the testing material. Moreover, it is crucial to obtain insights regarding outlying observations from participants. In this context, the Grubbs method presents distinct advantages over other methodologies commonly categorized as robust or accommodation methods (2). Accommodation techniques typically consist of utilizing the median, “trimming” data (exclusion of extreme values) or implementing “winsorization” (replacement of extreme values with neighboring data points). These methods tend to overlook or diminish the significance of outliers, thereby failing to address the issue directly. By employing the Grubbs method in PT schemes, researchers can gain a more profound understanding of the data; once outliers are identified, further investigation into their underlying causes can be pursued. In cases of significant asymmetric contamination from outliers, the mean value is more accurately estimated through outlier rejection methods, rather than through accommodation methods (3).

Grubbs introduced his formulation in 1950, wherein he derived the probability distribution for identifying the highest or lowest outlying observations and the two highest or the two lowest outliers (1). He established critical values based on a maximum of 25 observations. In this work, Grubbs also addressed the scenario in which both the highest and lowest values could simultaneously be outliers, though he did not derive distribution functions to ascertain critical values for this particular case. He remarked on the potential for extending his methodology to cases involving three outliers (three highest or three lowest) and four outliers (four highest or four lowest). In a subsequent paper (4), Grubbs presented numerous examples emphasizing statistical significance. Grubbs and Beck (5) extended his work with more observations to 147 and included additional percentage points for critical values. The issues concerning triple and quadruple outlier cases, initially alluded to by Grubbs in 1950, were addressed by Tietjen and Moore (6), who computed and tabulated several critical values for up to 50 observations. They defined two Grubbs-type statistics for identifying *k* outliers, up to a total of 10: the *L_4_* statistic (for multiple highest or lowest values) and the *E_4_* statistic (which considers the most extreme observations regardless of whether they are high or low). A limitation associated with the *E_4_* statistic was first identified by Rosner (7) and subsequently addressed by Hawkins (8), resulting in corrected calculations and critical values.

The utilization of Grubbs’ statistics in the evaluation step of our PT scheme is primarily justified by its inclusion in international standards, such as ISO 5725–2 (9). In 1988, Horwitz, representing the International Union of Pure and Applied Chemistry (IUPAC), formulated a protocol for PT schemes for Grubbs’ test (10). Subsequently, the IUPAC working committee revised the original protocol to adopt a 2.5% two-tailed percentage point for identifying outlying observations using Grubbs’ method (11), endorsed by numerous organizations, including AOAC INTERNATIONAL (12), the International Organization for Standardization (ISO), and IUPAC. However, the critical value tables only encompass data for observations up to 50, while omitting some intermediate ranges. This limitation renders the tables insufficient for our specific requirements. The paper by Mathiesen (13) is based on this standard by Horwitz (11), which serves as the foundation for an Excel macro. This has been utilized extensively for several years in our PT scheme. Nonetheless, as the number of observations has increased, the appropriateness of this macro has diminished.

Barnett and Lewis (2) conducted a comprehensive survey of various outlier detection methods. Their work encompasses the contributions of Grubbs and Beck (5) regarding single and double outliers, Tietjen and Moore (6) for triple and quadruple outliers, and the Dixon tests. The Dixon test is regarded as somewhat less rigorous than Grubbs’ test (14). Lemeshko and Lemeshko (15) addressed the highest and lowest double outliers and the three highest or three lowest triple outliers within the Grubbs framework. A significant advancement in extending both the Grubbs and Dixon tests, was presented in a series of papers by Verma et al. (16–18). This notable body of work established critical values for up to 33 variants of the Grubbs and Dixon tests to detect a maximum of four outliers simultaneously, accommodating datasets of up to 1000 observations through Monte Carlo (MC) simulations, along with artificial neural network (ANN) methodology for enhanced precision.

Despite the advancements documented in the aforementioned studies, they are insufficient for all observed scenarios in PT and validation schemes for the grain industry and possibly other business areas. We have, for example, found cases where higher-order outlier detection could enhance the PT scheme evaluation process. The main shortcomings are summarized as follows:

- Critical values for 2.5% significance level (two-sided), as recommended by IUPAC, are absent for many cases of multiple outlier testing.
- Critical values for all variants of multiple outliers (e.g. three highest and one lowest) cannot be found in the literature.
- Critical values for multiple outliers above quadruple cases are not tabulated for the number of observations above 50.
- A test protocol similar to that recommended by IUPAC has never been introduced that includes multiple outlier testing above double outliers.
- The correct handling of identical values has not been addressed before (as far as we know).

This paper addresses these gaps by extending the Grubbs method to account for higher-order outlier cases, including all variants with up to multiple-10 outliers, thereby providing a more comprehensive protocol for using Grubbs testing in PT schemes. Consequently, we propose a new testing protocol incorporating these higher-order tests, extending the IUPAC protocol (11) with the same significance level (2.5%) and a threshold of 2/9 for the maximum number of outliers to be rejected. However, we present critical values for seven significance levels in case other scenarios warrant them. Illustrative cases are given from our worldwide PT scheme and through data from a French validation scheme of more than 700 NIR instruments.

In our quality assurance program, we have implemented significant enhancements to the evaluation and reporting processes. A newly developed tool facilitates a more automated approach. The proposed protocol includes the Grubbs test as specified in ISO 5725–2 (9), incorporating the recommended significance level of 2.5% as determined by IUPAC (11) and extending its application to cases involving up to multiple-10 outliers. We conducted MC simulations to support this methodology to generate relevant critical values. This paper presents complete tables of Grubbs critical values for one-sided distributions at the significant percentage points of 0.1, 0.5, 1, 1.25, 2.5, 5, and 10%. The tables accommodate up to 1000 observations and are provided as supplementary material. They encompass 35 distinct cases comprising a total of 65 tests, as defined in the next section.

## Experimental

### Theory of Grubbs extended testing

The formulas for calculating Grubbs statistics are defined below in general terms to account for any number of outliers. In this discussion, we assume that the dataset, denoted as *x,* consists of *N* observations arranged in ascending order, with indices *i = 1, 2, …, N (*i.e. *x_1_ < x_2_ < … < x_N_)*. This discussion encompasses scenarios involving single and double outlying observations, as previously defined by Grubbs (1), explicitly defined. The new cases introduced in this comprehensive approach include any multiple-outlier configuration, exemplified by the double-outlier cases. The general expression for multiple outliers is used in the protocol to handle up to multiple-10 outlier scenarios, including all variants, such as the five highest and the five lowest simultaneously being rejected.

#### Grubbs’ test for single outlying observations

The Grubbs test for identifying a single highest observation, $x_{N}$, as an outlier, denoted as *GS(H)* in general terms with *H* for highest and *GS_N_* for index-specific notation, is defined according to Equations (1)–(3):


(1) $${GS(H)=GS}_{N}=\frac{\left(x_{N}-\bar{x}\right)}{s}$$

where the mean of the dataset is determined as presented in


(2) $$\bar{x}=\frac{1}{N}\sum_{i=1}^{N}x_{i}$$

and the SD of the dataset is defined as follows


(3) $$s=\sqrt{\frac{1}{N-1}\sum_{i=1}^{N}{\left(x_{i}-\bar{x}\right)}^{2}}$$

Conversely, the Grubbs test for identifying a single lowest observation, $x_{1}$, as an outlier, denoted *GS(L)* with *L* for lowest or *GS_1_* as index-specific notation, is defined in Equation (4), incorporating the mean and SD established in Equations (2) and (3), respectively.


(4) $${GS(L)=GS}_{1}=\frac{\left(\bar{x}-x_{1}\right)}{s}$$

#### Grubbs’ test for multiple outlying observations

A general expression for observations that are simultaneously outliers is formulated in Equation (5):


(5) $$\mathrm{GML}(\mathrm{nHkL})={\mathrm{GML}}_{k,n}=\frac{s_{k,n}^{2}}{s_{0}^{2}}$$

The notation GML stands for G for Grubbs, M for multiple and L for level 2 and higher. For the simplest cases of 2–4, the notation will be *GD*, *GT*, and *GQ* corresponding to double, triple and quadruple. For higher-multiple outlier cases, we will not use the latin nomenclature for multiples, even if it exists, but instead use notation such as *GM10* for multiple-10 outliers. Here, the indices *k* and *n* define the number of outliers at the low and high ends, respectively. For example, the case with the three highest and one lowest would be denoted *GQ(HHHL)* or *GQ_1, N-2, N-1, N_*, whereas the case with five highest and five lowest would use the terminology *GM10(5H5L)* or *GM10_5,5_*. The squared residual for all observations is characterized by


(6) $$s_{0}^{2}=\sum_{i=1}^{N}{\left(x_{i}-\bar{x}\right)}^{2}$$

Additionally, as outlined in Equation (7), a general formula for the squared residual excludes the outlying observations is given by


(7) $$s_{k,n}^{2}=\sum_{i=1-k}^{N-n}{\left(x_{i}-{\bar{x}}_{k,n}\right)}^{2}$$

The corresponding mean of the observations, which excludes the *k* and *n* outliers, is given by


(8) $${\bar{x}}_{k,n}=\frac{1}{N-(k+n)}\sum_{i=1-k}^{N-n}x_{i}$$

The expressions defined in Equations (5)–(8) will be used for all subsequent cases of the Grubbs statistic, simply applying the different number of outliers in the low and high end (i.e. for a specific number of *k* and *n*). The general expressions given in Equations (5)–(8) can now be applied to any number of multiple outlier cases, but we will limit the explicit expression to the case of double outliers for the sake of exemplifying it.

#### Grubbs’ test for double outlying observations

The procedure for assessing whether the two highest observations are simultaneously outliers is represented by the Grubbs double statistic, *GD(HH)*. This statistic is defined as the ratio of two squared residuals as detailed in Equations (5)–(8).


(9) $$GD(HH)={GD}_{N-1,N}=\frac{s_{N-1,N}^{2}}{s_{0}^{2}}\;$$

The example of Grubbs double *GD(HH)* translates to *k *= 0 and *n *= 2. The Grubbs double outlier test for the two lowest observations, denoted as *GD(LL)*, proceeds in a manner similar to Equation (10), with *k* = 2 and *n* = 0 applied to Equations (7) and (8).


(10) $$GD(LL)={GD}_{1,2}=\frac{s_{1,2}^{2}}{s_{0}^{2}}$$

The Grubbs double outlying test for the highest and lowest observations being simultaneously outliers, *GD(HL)*, is defined in Equation (11), with *k *= 1 and *n *= 1 applied to Equations (7) and (8).


(11) $$GD(HL)={GD}_{1,N}=\frac{s_{1,N}^{2}}{s_{0}^{2}}$$

### Protocol for statistical evaluation using the extended Grubbs test

A flow diagram illustrating the extended Grubbs test, which includes up to *L *= 10 levels in outlier tests, is presented in Figure 1. Cochran’s test is utilized to identify outliers among replicate observations from the same source (9), such as a specific laboratory within a PT scheme. However, the focus of this paper is on the Grubbs test. Thus, it is assumed that replicate data are not available, rendering the application of Cochran’s test unnecessary. The following is a detailed step-by-step description of the statistical evaluation process:

**Figure 1:**
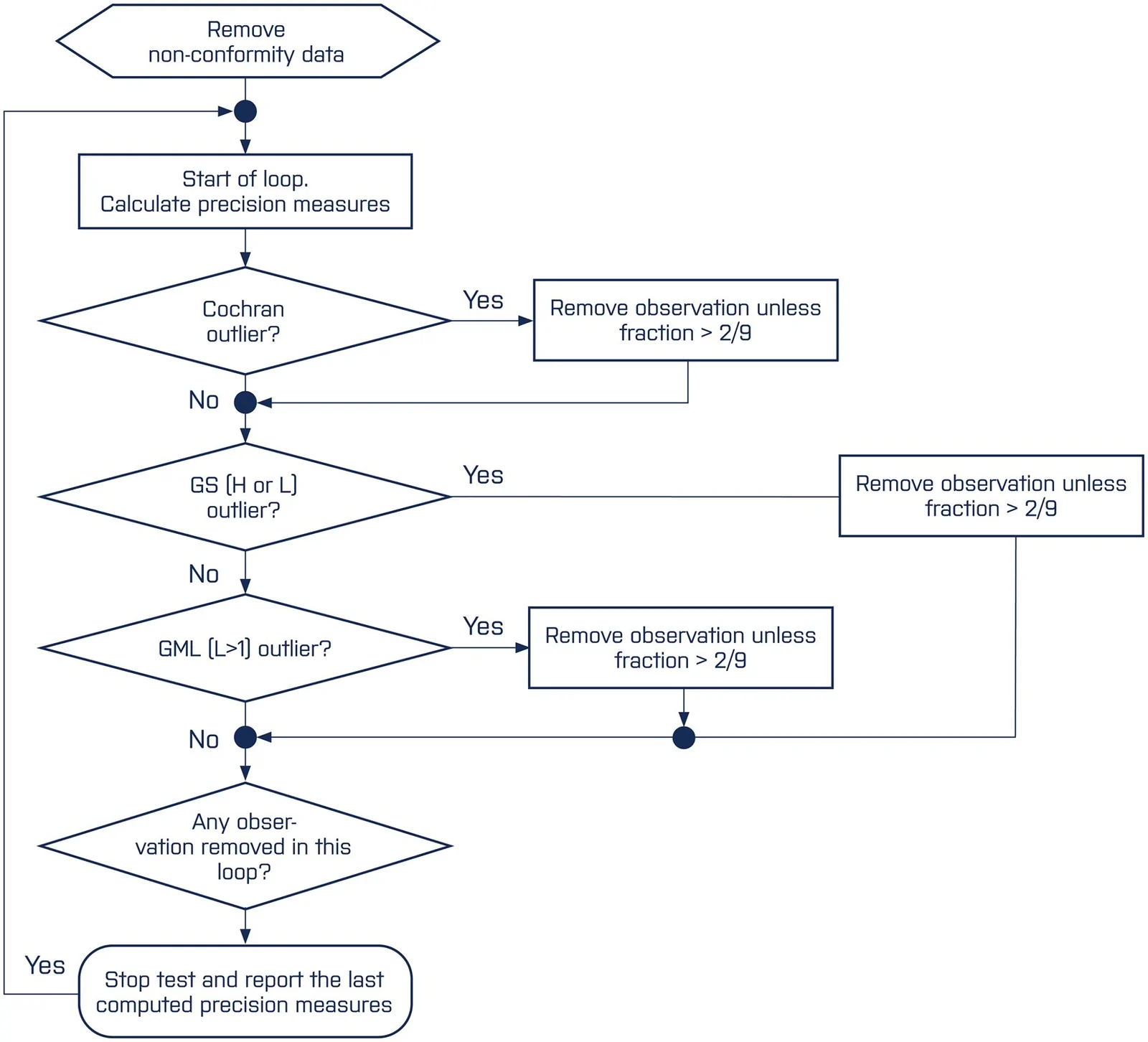
Flow diagram illustrating the statistical evaluation process in accordance with the IUPAC harmonized statistical procedure established in 1994. This diagram incorporates the outlier detection methodologies proposed by Cochran and Grubbs and has been extended to encompass the Grubbs’ multiple outlier tests starting after Grubbs’ single with *GM2* and ending with *GM10*. The most asymmetric cases come first in this iterative process.

1. Remove non-conformity data.
2. Calculate performance measures, such as the mean and SD.
3. In cases where replicate results for a specific measurement are available from all or nearly all laboratories, conduct Cochran’s outlier test. If such data are absent or insufficient, disregard the replicate data and forgo Cochran’s test.
4. Subsequently, apply Grubbs’ single outlier test. Should an outlier be identified, repeat the process until no further outliers remain. It is important to ensure that no more than 2/9 of the data points are removed as outliers.
5. If Cochran’s outlier test was performed prior to the Grubbs test and one or more outliers were removed by the Grubbs single test, reapply Cochran’s test before proceeding to the Grubbs double outlier test.
6. Once the Grubbs single outlier test and Cochran’s outlier test have been completed, assess the need for the Grubbs double outlier test, focusing on the two highest and two lowest values. If outliers are detected, eliminate them, and return to step (2).
7. Continue with the next Grubbs test and follow the same protocol outlined in step (6). If any outliers are removed for each test, revert to step (2). If no outliers are identified, proceed to the subsequent test, as illustrated in Figure 1. Currently, the program can handle up to 10 simultaneous outliers, but the protocol could be extended to handle even more if needed.
8. Upon completion of all outlier tests, the statistical values of repeatability, reproducibility, and the mean can be ascertained. These values can then be utilized for further analysis, including creating graphs and tables and calculating *z*-scores to evaluate laboratory performance within the PT scheme.

When discussing the testing order, it is important to note that asymmetric cases are assessed before symmetric cases. This implies that the Grubbs single-outlier test (high or low) and the double-outlier test (for either high–high or low–low) will precede the Grubbs double-outlier test for high–low scenarios. This rationale also extends to testing for triple, quadruple, and higher-order multiple outliers. Furthermore, when conducting tests such as the Grubbs single-highest or single-lowest, this process is executed in a single step, using the highest Grubbs statistic value obtained from *GS(H)* and *GS(L)* in the initial iteration. This approach is consistently employed in all *GML* levels whenever a shared distribution is present, exemplified by *GQ(HLLL)* and *GQ(HHHL)*.

### MATLAB program for Grubbs testing and reporting

A MATLAB program has been developed to implement the Grubbs test protocol according to the flow diagram shown in Figure 1. The program’s primary input is provided in Excel format. It consists of multiple sets of observations, typically including results from reference methods for grain parameters, such as protein and moisture content in barley. Additional observations may encompass NIR results obtained by each laboratory using their respective local equipment. The Grubbs critical values are incorporated as part of the input and are stored in MATLAB format. The program has the capability to generate a report in Word format automatically; however, while this feature is not the primary focus of the current paper, a limited version of the reporting function is available as supplementary material. It is important to emphasize that, despite increased automation in calculations, an expert assessment of the results—including the rejection of outliers—remains a critical component of the comprehensive evaluation process. That also means, for example, that if a laboratory shows a systematic error across most PT scheme materials relative to the calculated *z*-score tables, it should be corrected for or noted as laboratory outliers. The Grubbs test could be a good indicator for detecting this, but other measures, such as *z*-score evaluation or comparing mean values over all materials between the laboratories, are still part of the expert assessment.

In the context of outlier rejection methods, two crucial effects warrant attention, as articulated by Simonoff (3). One such effect is “masking,” where one outlier obscures another and goes undetected. In the case of multiple outlier detection, there is a potential risk of “swamping,” in which false outliers may be misidentified as part of a cluster. Our proposed protocol includes sequential testing to mitigate the risk of masking. Furthermore, adopting a moderate significance level of 2.5%, as recommended by IUPAC, is anticipated to reduce the risk of swamping when multiple outlier tests are implemented. Another concern is whether many iterations detect outliers, which could increase the overall risk of type-1 errors (i.e. misclassifying outliers). One way to deal with this problem is to apply the Bonferroni correction (19), where each iteration has a lower significance level, so that the accumulated error aligns with the overall level. However, it is difficult to determine the appropriate level, as the number of iterations is unknown. If those scenarios frequently occur and the expert assessment flags them as a risk, we propose applying a different significance level that terminates the iterative process earlier.

Much of the research on univariate outlier detection focuses on categorizing outlier types, identifying the underlying causes of their occurrence, and assessing whether the assumption of normality holds. Anscombe (20) characterized outliers with no meaningful context, such as measurement errors, as “spurious,” indicating they should be excluded unless the root causes can be identified and corrected. In the context of our PT scheme, we shall rectify any data errors whenever feasible; however, we will not differentiate between outlier types. A substantial body of research examines testing for inherent deviations, such as skewness and kurtosis (2), and for alternative distributions (15). While we also aim to identify the underlying causes of outliers, we assume that the data distribution within the PT scheme is normal, a requirement for the Grubbs test. We verify this assumption by applying normality tests, which indicate that any deviations are due to outliers. When the distribution is indeed non-normal, the simplest approach is to use the accommodation methodology; otherwise, a detailed investigation of the distribution is required to formulate a new protocol with specific statistics and critical values for that scenario.

### MC simulation for Grubbs’ critical values

MC simulations have been conducted in MATLAB for all 65 Grubbs’ tests to obtain the distributions needed to determine Grubbs’ critical values across a range of significance levels. For each sample size, *N*, up to 200 observations, the distributions for Grubbs’ cases up to quadruple outliers were simulated over 10 million runs, which were subsequently iterated 200 times. These iterations were averaged to derive critical values for various significance levels, specifically 0.1, 0.5, 1, 1.25, 2.5, 5, and 10%, applicable to one-sided distributions. These simulations took several months to complete. The simulations were extended to all other Grubbs’ cases with up to 10 outliers and to *N* up to 1000, to cover a broader range of scenarios. However, due to the time-consuming calculations, only 1 million runs and 10 iterations were performed, resulting in a precision of 1 significant figure lower than in the first set of simulations. The MATLAB code is available in this paper; users who wish to apply it to any of these cases can achieve higher precision.

A convergence study was conducted to determine the appropriate settings utilized in this research. The objective was to achieve a tolerance of 1e-5, corresponding to accurate values with at least four significant figures for Grubbs’ cases up to quadruple outliers and *N* = 200. Notably, the convergence process nearly reached 1e-6 (see the 1e-5 threshold as solid lines in Figure 2), thereby allowing us to assert that we possess accurate values to five significant figures. The average SD across iterations was 0.0007 for the different significance levels and sample sizes. The generated values were systematically compared against established tabulated values wherever feasible. In the research conducted by Verma et al. (18), a combination of MC simulation and ANN fitting was employed to obtain critical values with accuracies ranging from four to eight decimal places, contingent on the specific test and percentage point. In our comparison with these values for *GS(H)*, *GD(HH)*, *GT(HHH)*, and *GQ(HHHH)* across the available percentage points of 0.5, 1, 5, and 10%, we identified agreement within four or five significant figures. Therefore, the critical values tabulated in this paper for Grubbs’ cases up to quadruple outliers and *N* ≤ 200 are estimated to be accurate to four significant figures for percentage points ranging from 0.1 to 1.25% and at least three significant figures for percentage points from 2.5 to 10%. The critical values are presented with five significant figures to mitigate round-off errors within the estimated accuracy. Using Grubbs’ test for five to ten outliers, and for all cases with *N* from 200 to 1000, the convergence is well below the 1e-4 tolerance, as shown by the dotted vertical line in Figure 2. The precision for those scenarios yields critical values accurate to at least three significant figures. The reason for not pursuing the same precision for these cases is that it would have taken many months to complete, and it was not required for the cases we had in mind.

**Figure 2:**
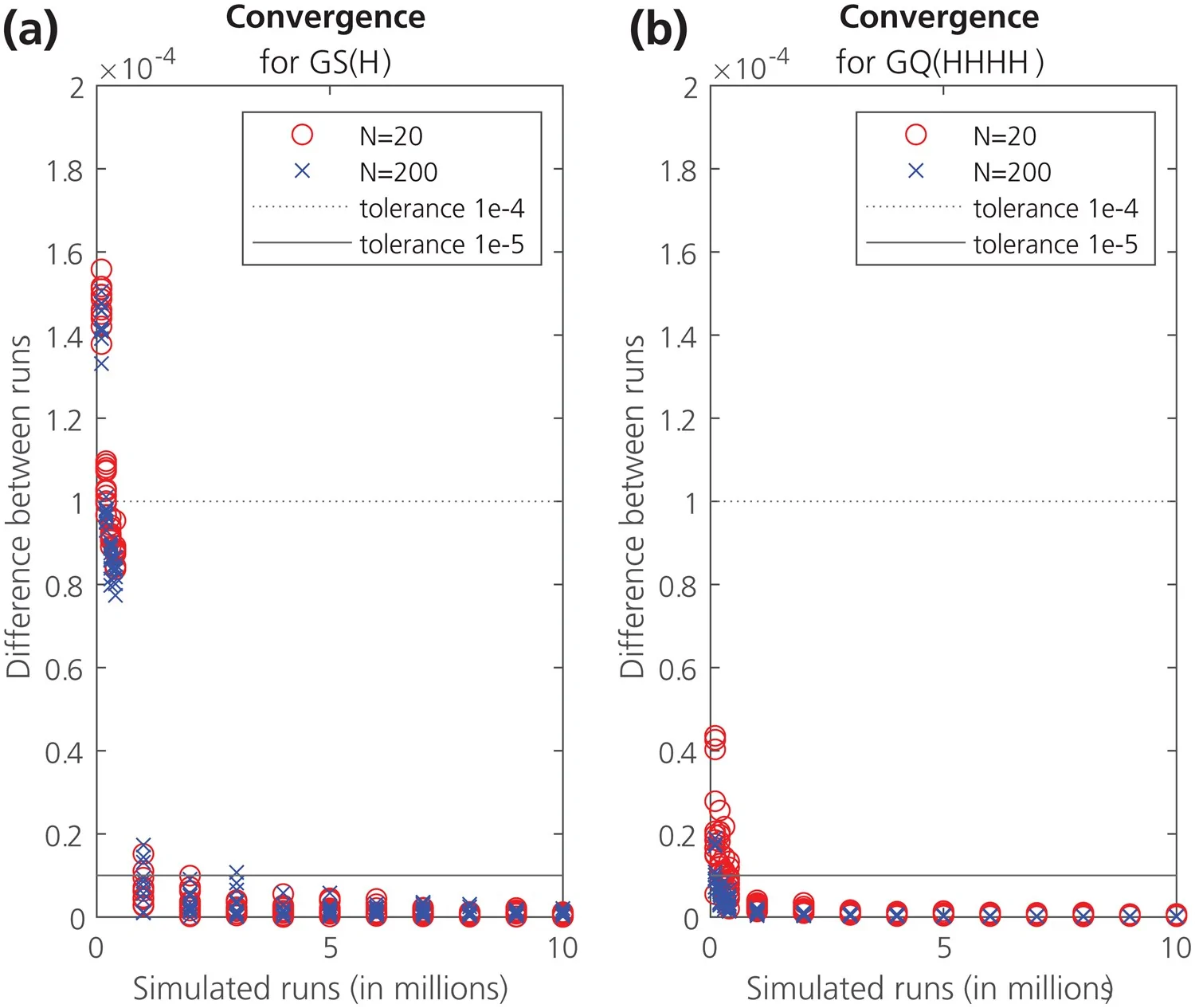
An illustration of the convergence study for the Grubbs’ outlier test is presented, highlighting the difference between runs with a 100-run gap. This analysis encompasses two cases: single *GS(H)* as shown in panel (a) and quadruple *GQ(HHHH)* as shown in panel (b). Examples are provided for sample sizes of *N* = 20 (circles) and *N* = 200 (crosses) at a significance level of 1.25%.

## Results and discussion

### Results of Grubbs’ critical values by Monte Carlo simulation

The comprehensive set of Grubbs’ critical values for up to 1000 observations, determined through MC simulations utilizing MATLAB, is available in the supplementary material. Furthermore, the critical values corresponding to a selection of observations used in the illustrative examples presented in this paper are detailed in the tables located in subsequent sections.

### Application to PT and validation scheme data

#### Grubbs’ extended test protocol applied to PT and validation scheme data

The efficacy of the extended Grubbs tests, alongside the tables of MC-simulated critical values, is demonstrated through examples derived from a worldwide PT scheme conducted in 2024 by one of the authors. The formulas in Equations (5)–(8) are general, which means that they could, in principle, be used to define any number of outliers. However, we have restricted ourselves to not go beyond the multiple-10 case simply because the application to the PT scheme discussed in this paper does not warrant higher degrees of outlier rejections. The occurrence of identical values represents one of several justifications for implementing higher-order multiple outlier testing methodologies. The findings from the PT scheme indicate a substantial number of observations categorized as Grubbs triple (*GT*) or Grubbs quadruple (*GQ*) outliers. In the barley protein PT scheme, which included both reference and NIR methods, a total of 50% of the datasets were identified as having outliers, with 14% of these attributed to *GT* or *GQ*. The PT scheme recorded the highest percentage of outliers related to oil content in rapeseed, with 63% of the datasets identified as having outliers, 41% of which originated from *GT* or *GQ* classifications. Two examples elucidating these findings are presented below. The normality of the distributions for these examples is tested using the approximate uniform order statistics medians and tabulated critical values by Filliben (21). A third example applies the protocol to a large dataset from the world’s largest grain network of NIR devices in France, revealing multiple outliers.

#### The issue with identical values in the Grubbs test

The initial example is presented in Figure 3, which includes a total of 56 observations of local NIR predictions of protein content in barley sample number B10. This dataset reveals several Grubbs single outliers in both the high and low extremes. The Grubbs outliers are designated with a prefix of Gn in the labels, where G denotes the Grubbs outlier, and n indicates the iteration number. A total of six iterations resulted in the detection of Grubbs’ single outliers, as detailed in Table 1.

**Figure 3:**
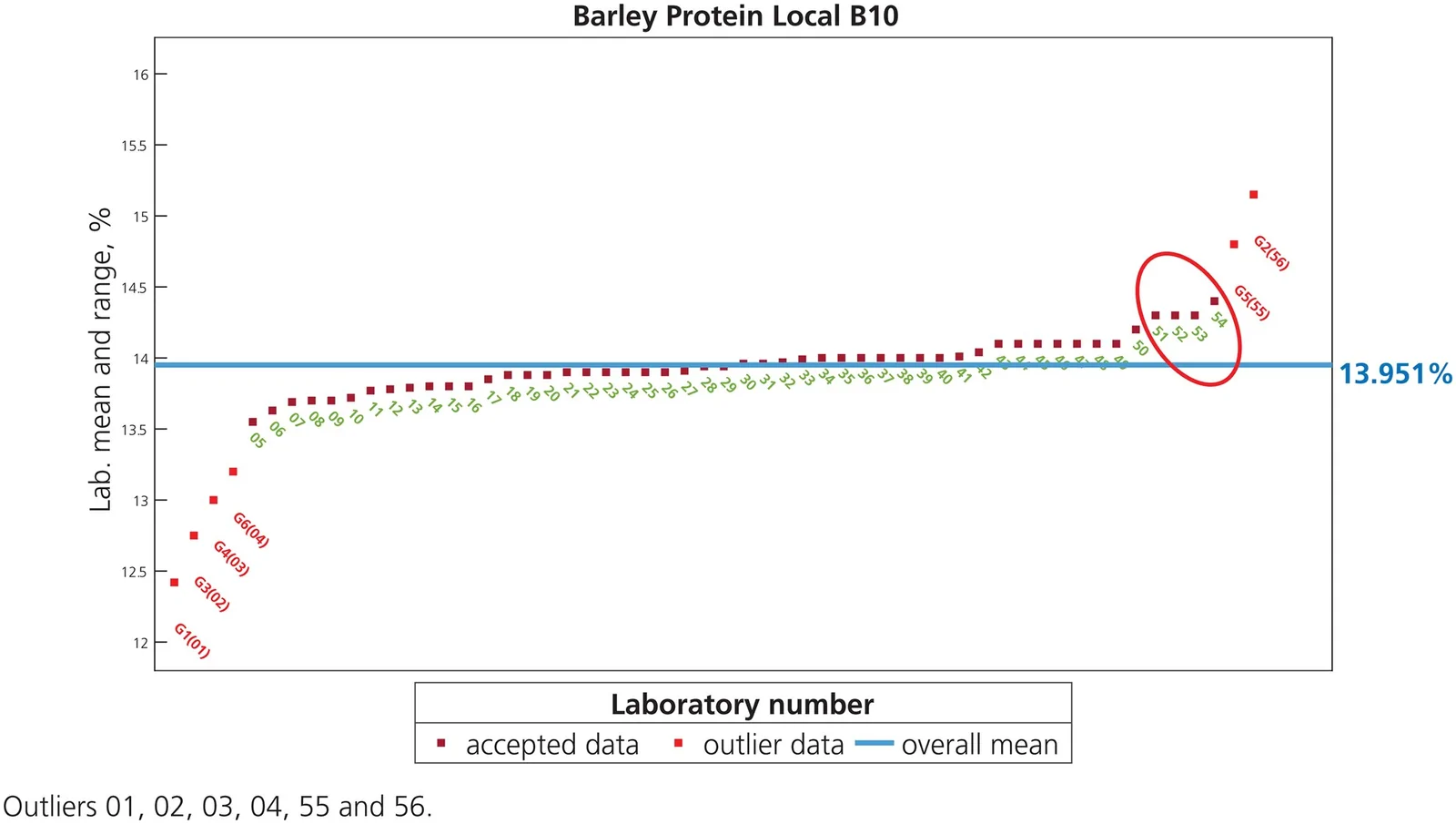
An illustration of the Grubbs test demonstrating improper management of identical values.

**Table 1:** Example presenting a comparative analysis of a MATLAB program and an Excel macro, elucidating the issue of identical observations obtained from the PT scheme for local NIR predictions of protein content in barley, specifically for sample B10.

Iteration	*N*	Test type	MATLAB program			Excel macro		
			G statistic	G critical	Outlier	G statistic	G critical	Outlier
1	56	*GS(L)*	3.7130	3.3337	Yes	12.9	11.1	Yes
2	55	*GS(H)*	3.4797	3.3265	Yes	11.3	11.1	Yes
3	54	*GS(L)*	3.7566	3.3190	Yes	13.8	11.1	Yes
4	53	*GS(L)*	3.5040	3.3115	Yes	12	11.1	Yes
5	52	*GS(H)*	3.6098	3.3037	Yes	13.2	11.1	Yes
6	51	*GS(L)*	3.6198	3.2957	Yes	13.5	11.1	Yes
7	50	*GS(H or L)*	2.5461	3.2875	No	6	11.1	No
7	50	*GD(HH)*	0.7790	0.67418	No	19.4	16.2	Yes

This table displays the calculated Grubbs statistic (referred to as G statistic) along with the relevant number of observations, *N*. It compares it against the Grubbs’ critical value (denoted as G critical), as derived from both the MATLAB program and the Excel macro developed by Mathiesen (13). It is important to note that the Grubbs statistic and critical values in the Excel macro are expressed as a percentage decrease, differing from the metrics employed in this study. Furthermore, critical values are available only for samples with *N* ≤ 50; however, these values are also applied to cases where *N* exceeds 50, as indicated in Table 1, where the value of 11.1 has been utilized for the initial six iterations. In this context, six Grubbs single outliers are systematically removed one at a time. After completing the sixth iteration, the MATLAB algorithm finds no further outliers. Conversely, the Excel macro, when assessing Grubbs double outliers, identifies observations that are encircled in Figure 3. The highest value corresponds to the observation labeled 54, while the next highest pertains to three identical values labeled 51–53. A significant limitation of this approach is that the *GD(HH)* statistic removes four observations and assesses the statistic against a critical value intended for a double-outlier test, which is inappropriate. The problem arises from using the MIN and MAX functions in Excel, which do not account for the number of observations included in that step. It could be argued that if one of the identical values were identified as an outlier, the other should be rejected too. This is logical, but only valid if the test is based on the correct statistics. The correct procedure would involve removing the highest observation and one of the identical values from the next-highest category, then comparing the result against the appropriate *GD* critical value. If this step is not automated, it is the expert performing the assessment’s responsibility to address it correctly. The methodology utilized effectively performs a quadruple-outlier test for the four highest observations, necessitating comparison against the correct *GQ* critical value, which is 0.5076 and even lower than the *GD* critical value. Notably, neither of these tests identifies any outliers using the MATLAB algorithm. This observation underscores a key finding of this study: the Grubbs tests should only use the exact number of rejections as stipulated by the test irrespective of the presence of identical values.

In Figure 4, a Q–Q plot is shown that illustrates the normality of the data for Example 1, which clearly shows deviating behavior. The calculated correlation coefficient is only 0.901, which is below critical values for both 1% and 5% significance levels (21). The failure of the normality test could arise from either of two possibilities: the data are inherently non-normal, or there are outliers caused by random errors that skew the test. This can be investigated further by testing the other PT scheme materials (at least 5, but 10 used in this scheme) for the exact same NIR devices. All of them pass the 1% significance level, and seven pass the 5% level. This confirms that the deviations from normality in Example 1 originate from outliers caused by random errors, hence the Grubbs test protocol is valid.

**Figure 4:**
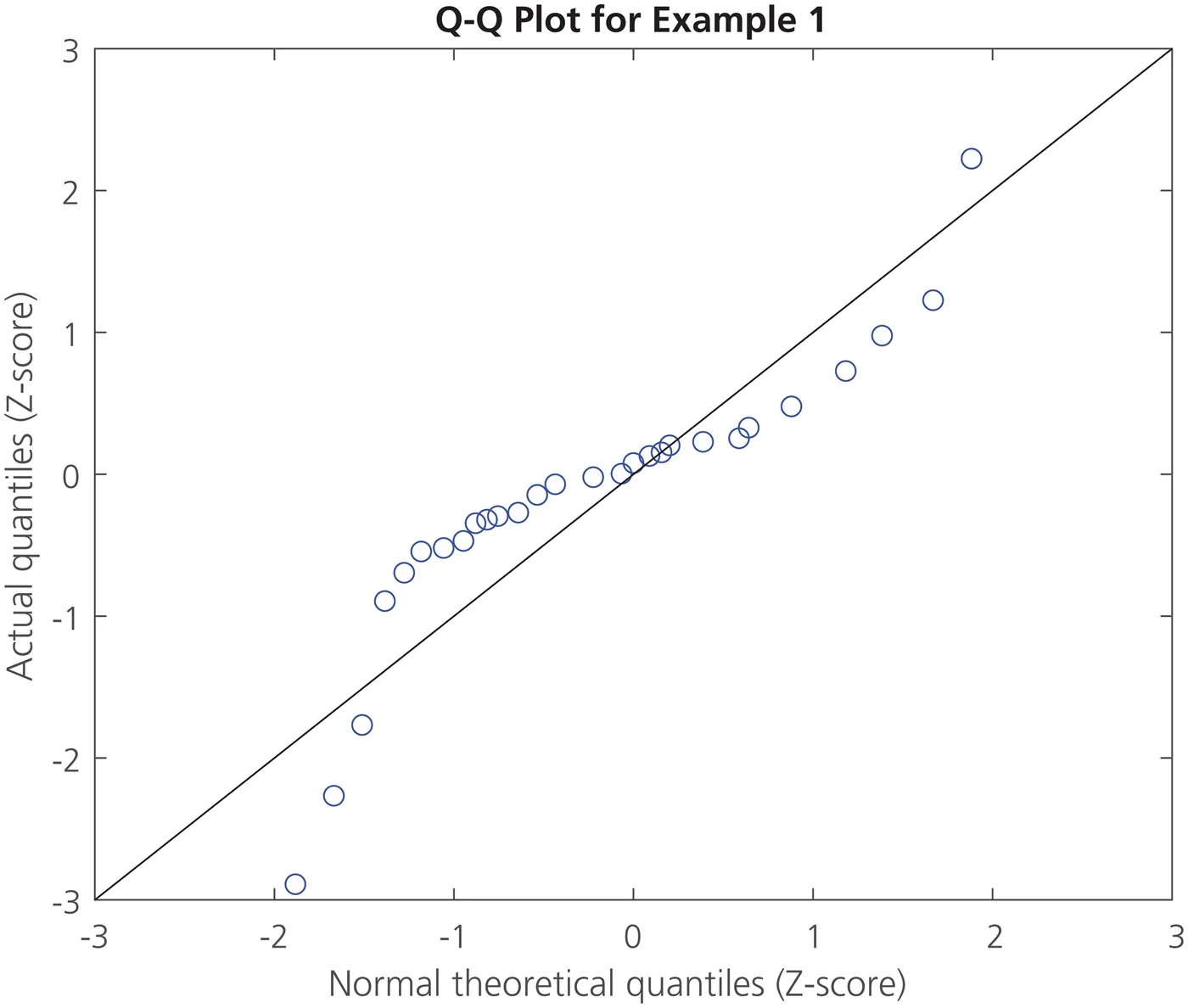
Test of normality illustrated with a Q–Q plot for Example 1.

#### Grubbs’ quadruple test for identifying the four highest outlying observations

Another set of 56 observations from the PT scheme of local NIR results for protein in barley Sample B9 is shown in Figure 5. The four outliers marked with G correspond to the four highest outlying observations, labeled 07, 26, 51, and 55, identified by the Grubbs quadruple test. The sequential testing process from Grubbs’ single (*GS*) to Grubbs’ quadruple (*GQ*) is detailed in Table 2, mirroring the format presented in Table 1. It is noteworthy that testing using the Excel macro was not conducted, as triple or quadruple testing is not incorporated within that tool. This instance exemplifies that identical values do not pose a significant issue and demonstrates that the occurrence of a quadruple outlying observation is relatively uncommon.

**Figure 5:**
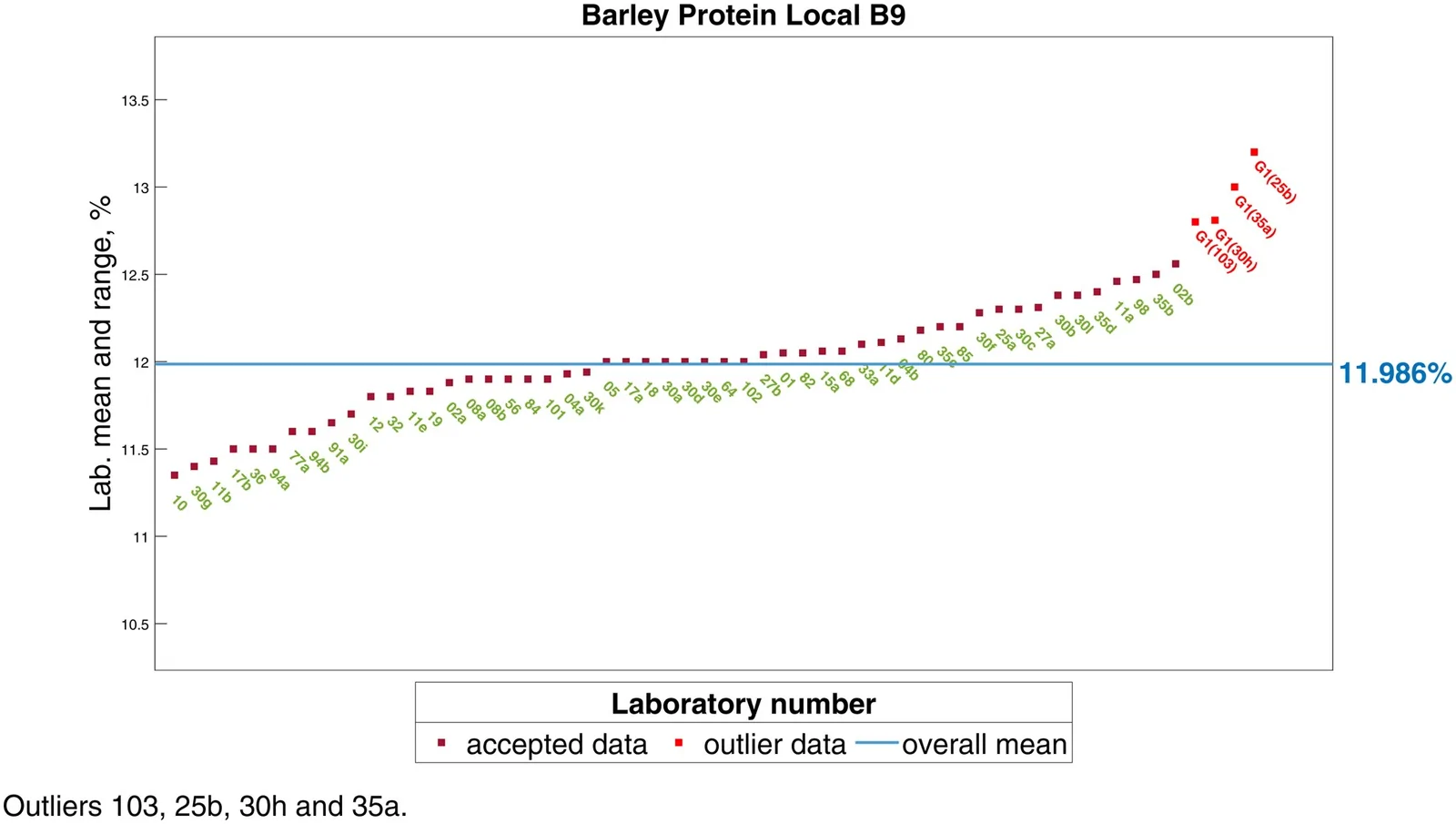
An illustration of Grubbs’ test demonstrating the impact of the quadruple outlier test *GQ(HHHH)*.

**Table 2:** Example of using a MATLAB program to demonstrate the occurrence of quadruple *GQ(HHHH)* outlying observations from the PT scheme through local NIR predictions of protein content in barley for Sample B9.

Iteration	*N*	Test type	G statistic	G critical	Outlier
1	56	*GS(H or L)*	2.9780	3.3337	No
1	56	*GD(HH or LL)*	0.7190	0.69988	No
1	56	*GD(HL)*	0.7772	0.68169	No
1	56	*GT(HHH or LLL)*	0.6400	0.62936	No
1	56	*GT(HLL or HHL)*	0.6634	0.60298	No
1	56	*GQ(HHHH or LLLL)*	0.5600	0.57205	Yes

The normality test for Example 2 is illustrated in Figure 6 as a Q–Q plot. The calculated correlation coefficient is 0.984 and passes both 1% and 5% levels of critical values (20) verifying the normality assumption.

**Figure 6:**
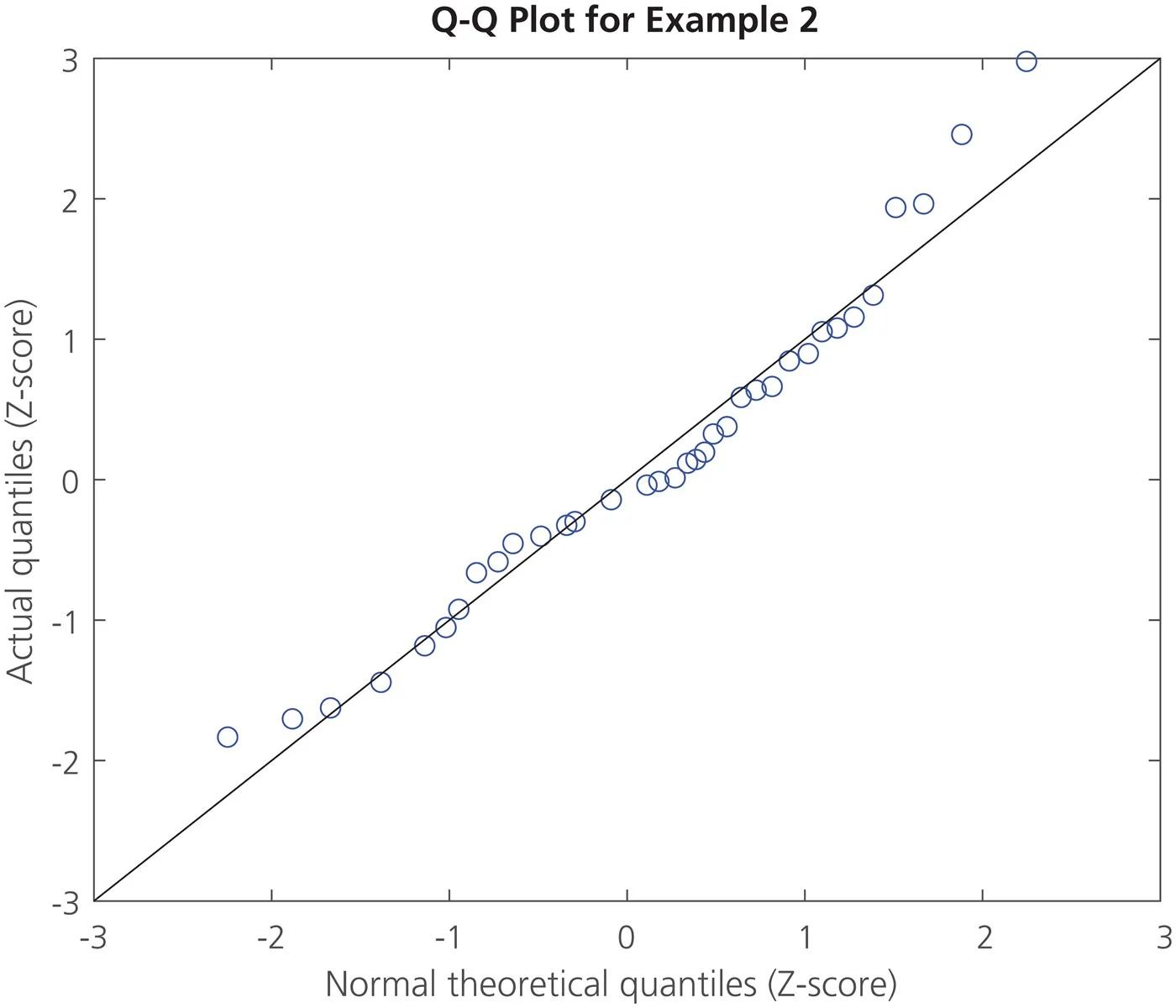
Test of normality illustrated with a Q–Q plot for Example 2.

Before removing outliers, the mean value was 12.055%, whereas after eliminating the four highest observations, the mean was adjusted to 11.986%. The difference between the two mean values is 0.069%, which, when rounded to two decimal points, amounts to 0.07%. This can be contrasted with a robust estimation using the median as the accommodation approach, which produced a value of 12.000%. This estimate closely aligns with the mean calculated using the proposed protocol with the Grubbs outlier test, indicating a low risk of the “swamping” effect, which signifies the removal of false outliers. Nonetheless, it is imperative to investigate the underlying causes to ensure that the removal of outliers is warranted.

Estimating the implications of a shift corresponding to a simplified scenario of 0.1% protein content in malting barley would be pertinent. The malting sector typically categorizes different grades based on protein content, which may vary among suppliers across various countries. Notably, one supplier in Denmark (22) has presented harvest conditions that allow for the recalculation of limits and reductions in terms of US dollars. For premium grade Pilsner malt, protein content should fall within the range 9.5 to 11%, reducing $1.17 per ton if the protein content is within the range 8.5 to 9.5% or 11.1 to 11.5%. It is essential to recognize that there is a significantly greater price reduction if the barley must be downgraded to feed quality, defined as having protein content below 8.4% or exceeding 11.5%. As reported in a UK newsletter by the Agriculture and Horticulture Development Board (AHDB; (23)), the price differential between feed and malt quality can reach as high as $102 per ton.

Three typical distributions of protein content in malting barley have been artificially constructed based on the aforementioned information, typical real harvest validation data in terms of mean and SD, and practical experience related to deviations. A normal distribution centered at the mean of the premium grade (10.25%) with a standard deviation of 0.7% is used to simulate a near-ideal harvest. A slightly distorted distribution with a shifted mean of 9.8% and a broader standard deviation of 0.9% is used as a second case. By incorporating a skew towards the lower end, amalgamated with the distribution utilized in the second case in a 75:25% relation, a third case was constructed.

Considering a country such as France, which handles approximately 11 million tons of barley annually, and applying the insights gleaned from Danish suppliers along with reports from the United Kingdom in a realistic yet hypothetical scenario, it becomes feasible to calculate the impact of a 0.1% shift in protein content based on the distributions constructed. The total cost in US dollars has been computed for the three scenarios, as shown in Table 3. The total cost for the nearly ideal scenario is estimated at approximately $34 million. Conversely, if distortions from this ideal scenario are accounted for, costs increase to $52 million and $58 million when considering the shifted/broadened distribution and the low-end skew, respectively. Although these scenarios are artificially constructed, they likely bear considerable resemblance to a realistic situation, underscoring the financial implications involved if proper validation and optimization of the payment devices employed in the grain industry are not conducted.

**Table 3:** An illustration demonstrating the value within the grain industry resulting from a minor shift associated with the implementation of quadruple *GQ(HHHH)* outlier testing is presented. The harvest distributions of protein content in malting barley were artificially generated, and the calculations are based on a total harvest of 11 million tons.

Cost levels	Cost ($/ton)	Normal		Shifted/broadened		Low-end skew	
		Frac. (%)^a^	Value ($)^b^	Frac. (%)^a^	Value ($)^b^	Frac. (%)^a^	Value ($)^b^
Protein 9.4–9.6%	1.17	6.4	0.8	8.4	0.8	7	0.9
Protein 10.9–11.1%	1.17	6.4	0.8	3.8	0.5	2.9	0.4
Protein 8.4–8.6%	102	0.5	5.5	3	33.8	3.9	44.2
Protein 11.4–11.6%	102	2.4	26.7	1.5	17.2	1.1	12.2
Total			34		52		58

a Fraction of the distribution for each cost level given as % and denoted Frac.

b Value is calculated for each cost level and reported in total as millions of $.

#### Grubbs’ multiple outlier test for five lowest outlying observations

In the present analysis, we examine a dataset comprising more than 700 observations derived from the validation scheme of local NIR predictions of protein content in barley for sample number B4. The data were generously shared by the French grain network Agroreso. This case is characterized by the presence of a Grubbs’ multiple outlier beyond quadruple outliers, identified as five extreme low observations, as illustrated in Figure 7.

**Figure 7:**
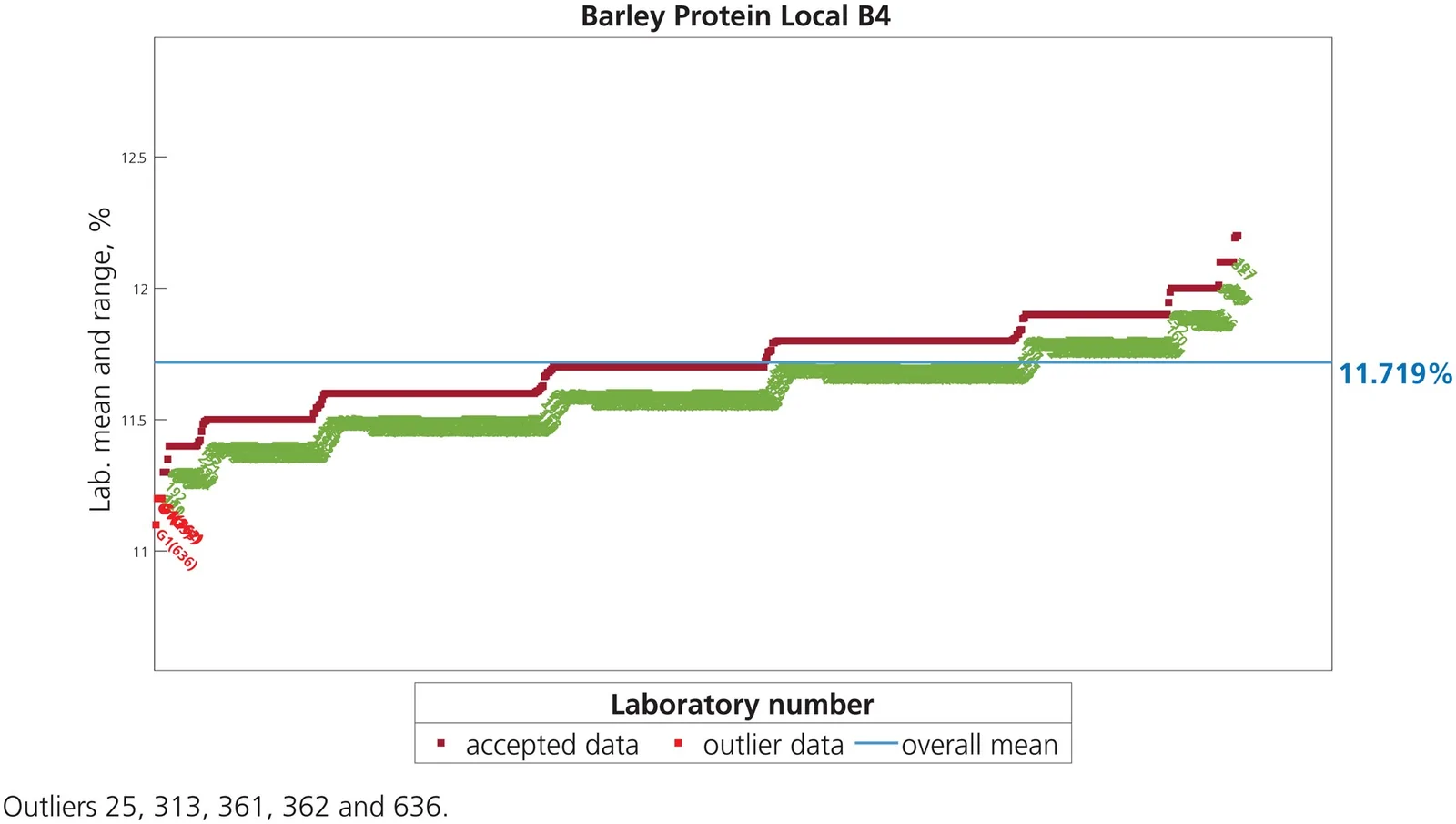
An illustration of Grubbs’ test demonstrating the impact of the multiple-5 outlier test *GM5(0H5L)* in a large dataset of 736 observations.

Table 4 details the detection of a Grubbs’ multiple-5 outlier at the lower end, *GM5(0H5L)*, in the first iteration. It is important to note that no intermediate Grubbs’ test results, ranging from single to quadruple outlier detection, yielded outlier identifications. Moreover, the critical values from Tietjen and Moore (6) only cover up to 50 observations, hence they cannot be compared against.

**Table 4:** Example utilizing a MATLAB program to demonstrate the occurrence of multiple 5 (0H5L) outlying observations from a French validation scheme by more than 700 local NIR predictions of protein in barley for Sample B4.

Iteration	*N*	Test type	G statistic	G critical	Outlier
1	736	*GS(H or L)*	3.7624	4.1217	No
1	736	*GD(HH or LL)*	0.9671	0.96244	No
1	736	*GD(HL)*	0.9688	0.96043	No
1	736	*GT(HHH or LLL)*	0.9535	0.95015	No
1	736	*GT(HLL or HHL)*	0.9552	0.94694	No
1	736	*GQ(HHHH or LLLL)*	0.9399	0.93911	No
1	736	*GQ(HHHL or HLLL)*	0.9417	0.93510	No
1	736	*GQ(HHLL)*	0.9433	0.93399	No
1	736	*GM5(5H0L or 0H5L)*	0.9262	0.92895	Yes

The mean value variance before and after the exclusion of outliers is approximately 0.004, which is statistically insignificant and certainly affected by the very large dataset. Nevertheless, the detection of these outlier observations signifies a noteworthy discrepancy when compared to the rest of the dataset. This prompts further investigation into the underlying causes of such differences. Potential factors may include discrepancies in adjustments against reference methods, or other influencing elements. We omit showing the Q–Q plot for this case, but it does show good linearity and a correlation coefficient of 0.9974, which is above the 1% significance level and slightly below the 5% level.

## Conclusions

The critical values for the Grubbs test reported in this paper were derived from extensive MC simulations. These values serve as a reference for the automated MATLAB program employed for Grubbs’ testing of observations from the PT and validation scheme discussed herein. Following rigorous convergence studies, we established good agreement with the tabulated values found in the literature. We have provided case studies from the PT scheme and a French validation scheme that demonstrate the value of higher-order Grubbs’ testing. This extended Grubbs’ testing allows for a more accurate determination of mean values and facilitates the identification of outlying observations that may uncover significant insights upon confirmation that these are statistically distinct. Our findings indicate that the higher-order Grubbs tests resolved issues associated with identical values and identified quadruple and higher outlying observations in unique non-identical cases. This includes instances not previously documented in the literature, such as scenarios involving the five lowest observations designated as outliers in a dataset comprising over 700 observations. Additionally, we discuss the implications of employing the quadruple outlier test in a real-world context drawn from the PT scheme for barley. Notably, excluding the four highest observations resulted in a mean shift of 0.07% protein content in barley, which could have substantial economic implications, potentially amounting to millions of US dollars in value. This underscores the critical importance of rigorously verifying analytical methods and devices in the grain industry, which we believe our newly proposed protocol will facilitate.

## Supplementary Material

qsag036_Supplementary_Data

## Data Availability

The data and program code that support the findings of this study are available in 4TU.ResearchData at DOI: 10.4121/ed95b71a-d5e4-4fb0-9d0b-adb08e651edf.
