# Supplementary material for: A generalized outlier method with an automated test protocol applied to proficiency testing schemes: utilizing Monte Carlo simulation for generating critical values for the extended Grubbs statistic
Source: J AOAC Int. 2026 Apr 29;109(6):974–84. doi: 10.1093/jaoacint/qsag036 (PMC13732683; doi:10.1093/jaoacint/qsag036)
Supplement: Supplementary file 1 [file qsag036_supplementary_data.zip › aoac-25-0307-File013.pdf]

Extended Grubbs Statistics Table for Single Outliers

Table 1 – *GS(H or L)*. Critical values of upper significance levels for the Grubbs single outlier test, applicable in situations where the highest or lowest observation is identified as outlier, denoted as *GS(H)* and *GS(L)*, respectively.

N	0.1%	0.5%	1%	1.25%	2.5%	5%	10%
3	1.1547	1.1547	1.1546	1.1546	1.1543	1.1531	1.1484
4	1.4992	1.4963	1.4925	1.4906	1.4813	1.4625	1.4250
5	1.7803	1.7637	1.7489	1.7424	1.7150	1.6714	1.6016
6	2.0107	1.9728	1.9443	1.9327	1.8871	1.8221	1.7289
7	2.2006	2.1391	2.0973	2.0811	2.0200	1.9381	1.8280
8	2.3586	2.2743	2.2208	2.2006	2.1266	2.0317	1.9089
9	2.4919	2.3868	2.3231	2.2996	2.2150	2.1096	1.9773
10	2.6059	2.4821	2.4097	2.3833	2.2900	2.1761	2.0362
11	2.7046	2.5641	2.4842	2.4554	2.3547	2.2339	2.0880
12	2.7909	2.6357	2.5494	2.5185	2.4115	2.2850	2.1341
13	2.8673	2.6990	2.6070	2.5743	2.4620	2.3305	2.1756
14	2.9354	2.7553	2.6584	2.6242	2.5073	2.3717	2.2132
15	2.9966	2.8061	2.7048	2.6692	2.5483	2.4090	2.2476
16	3.0520	2.8520	2.7469	2.7102	2.5857	2.4433	2.2793
17	3.1023	2.8939	2.7854	2.7475	2.6199	2.4748	2.3085
18	3.1483	2.9324	2.8208	2.7820	2.6516	2.5040	2.3358
19	3.1908	2.9679	2.8535	2.8138	2.6809	2.5312	2.3612
20	3.2300	3.0008	2.8838	2.8434	2.7082	2.5566	2.3850
21	3.2664	3.0313	2.9120	2.8709	2.7338	2.5804	2.4073
22	3.3002	3.0598	2.9384	2.8967	2.7577	2.6027	2.4284
23	3.3319	3.0865	2.9632	2.9209	2.7803	2.6239	2.4484
24	3.3616	3.1116	2.9866	2.9437	2.8015	2.6438	2.4673
25	3.3895	3.1353	3.0086	2.9653	2.8217	2.6628	2.4853
26	3.4158	3.1577	3.0295	2.9857	2.8408	2.6808	2.5024
27	3.4406	3.1788	3.0492	3.0050	2.8589	2.6979	2.5188
28	3.4640	3.1988	3.0680	3.0233	2.8762	2.7142	2.5344
29	3.4863	3.2179	3.0858	3.0409	2.8927	2.7299	2.5493
30	3.5074	3.2361	3.1029	3.0576	2.9084	2.7449	2.5637
31	3.5277	3.2535	3.1192	3.0736	2.9235	2.7592	2.5774
32	3.5468	3.2700	3.1348	3.0889	2.9380	2.7730	2.5907
33	3.5650	3.2858	3.1497	3.1035	2.9519	2.7863	2.6034
34	3.5825	3.3010	3.1640	3.1176	2.9652	2.7990	2.6157
35	3.5991	3.3156	3.1778	3.1311	2.9781	2.8113	2.6276
36	3.6152	3.3296	3.1910	3.1442	2.9905	2.8232	2.6390
37	3.6307	3.3431	3.2038	3.1567	3.0025	2.8347	2.6501
38	3.6455	3.3561	3.2162	3.1688	3.0140	2.8458	2.6609
39	3.6599	3.3687	3.2280	3.1805	3.0251	2.8565	2.6713
40	3.6736	3.3808	3.2395	3.1918	3.0360	2.8669	2.6814
41	3.6869	3.3924	3.2506	3.2028	3.0464	2.8770	2.6912
42	3.6997	3.4037	3.2613	3.2134	3.0566	2.8868	2.7007
43	3.7120	3.4146	3.2718	3.2236	3.0664	2.8962	2.7100
44	3.7239	3.4252	3.2818	3.2335	3.0759	2.9055	2.7189
45	3.7356	3.4354	3.2916	3.2432	3.0852	2.9144	2.7277
46	3.7468	3.4454	3.3011	3.2525	3.0942	2.9232	2.7362
47	3.7577	3.4551	3.3103	3.2616	3.1030	2.9317	2.7445
48	3.7681	3.4644	3.3193	3.2705	3.1115	2.9399	2.7526
49	3.7784	3.4736	3.3281	3.2791	3.1199	2.9480	2.7605
50	3.7884	3.4825	3.3366	3.2875	3.1280	2.9559	2.7683
51	3.7980	3.4912	3.3449	3.2957	3.1358	2.9636	2.7758
52	3.8074	3.4996	3.3529	3.3037	3.1436	2.9711	2.7832
53	3.8165	3.5077	3.3608	3.3115	3.1511	2.9784	2.7903
54	3.8254	3.5157	3.3685	3.3190	3.1585	2.9856	2.7974
55	3.8341	3.5235	3.3760	3.3265	3.1656	2.9926	2.8043
56	3.8426	3.5311	3.3833	3.3337	3.1726	2.9994	2.8110
57	3.8508	3.5385	3.3905	3.3408	3.1795	3.0061	2.8176
58	3.8588	3.5458	3.3975	3.3477	3.1862	3.0127	2.8241
59	3.8666	3.5529	3.4043	3.3544	3.1928	3.0191	2.8304
60	3.8742	3.5598	3.4110	3.3610	3.1992	3.0254	2.8366
61	3.8817	3.5666	3.4175	3.3675	3.2055	3.0316	2.8427
62	3.8889	3.5732	3.4239	3.3739	3.2117	3.0376	2.8486
63	3.8960	3.5797	3.4302	3.3801	3.2177	3.0436	2.8545
64	3.9030	3.5861	3.4363	3.3862	3.2237	3.0494	2.8603
65	3.9099	3.5924	3.4424	3.3922	3.2295	3.0551	2.8659
66	3.9165	3.5985	3.4483	3.3980	3.2352	3.0607	2.8715
67	3.9231	3.6044	3.4541	3.4038	3.2408	3.0662	2.8769
68	3.9294	3.6103	3.4598	3.4094	3.2463	3.0716	2.8823

N	0.1%	0.5%	1%	1.25%	2.5%	5%	10%
69	3.9358	3.6160	3.4654	3.4149	3.2517	3.0769	2.8875
70	3.9419	3.6217	3.4709	3.4203	3.2570	3.0821	2.8927
71	3.9479	3.6272	3.4762	3.4257	3.2622	3.0872	2.8978
72	3.9538	3.6326	3.4815	3.4309	3.2673	3.0923	2.9028
73	3.9596	3.6379	3.4867	3.4361	3.2724	3.0972	2.9077
74	3.9653	3.6432	3.4918	3.4411	3.2773	3.1021	2.9126
75	3.9708	3.6484	3.4968	3.4461	3.2822	3.1069	2.9174
76	3.9763	3.6535	3.5017	3.4510	3.2870	3.1117	2.9220
77	3.9817	3.6584	3.5065	3.4557	3.2917	3.1163	2.9267
78	3.9870	3.6633	3.5113	3.4605	3.2963	3.1209	2.9312
79	3.9921	3.6681	3.5160	3.4651	3.3009	3.1254	2.9357
80	3.9972	3.6728	3.5206	3.4697	3.3054	3.1299	2.9401
81	4.0023	3.6774	3.5251	3.4742	3.3098	3.1342	2.9445
82	4.0072	3.6820	3.5295	3.4786	3.3141	3.1385	2.9488
83	4.0120	3.6866	3.5339	3.4830	3.3184	3.1428	2.9530
84	4.0168	3.6910	3.5383	3.4873	3.3227	3.1470	2.9572
85	4.0215	3.6954	3.5425	3.4915	3.3268	3.1511	2.9613
86	4.0261	3.6997	3.5468	3.4957	3.3310	3.1552	2.9654
87	4.0306	3.7039	3.5509	3.4998	3.3350	3.1592	2.9694
88	4.0351	3.7080	3.5550	3.5039	3.3390	3.1632	2.9734
89	4.0395	3.7122	3.5590	3.5079	3.3430	3.1671	2.9773
90	4.0438	3.7162	3.5630	3.5119	3.3469	3.1710	2.9811
91	4.0481	3.7203	3.5669	3.5158	3.3507	3.1748	2.9850
92	4.0523	3.7242	3.5708	3.5196	3.3545	3.1785	2.9887
93	4.0564	3.7281	3.5746	3.5234	3.3582	3.1822	2.9924
94	4.0606	3.7319	3.5783	3.5271	3.3619	3.1859	2.9961
95	4.0646	3.7357	3.5820	3.5308	3.3656	3.1895	2.9997
96	4.0685	3.7394	3.5857	3.5345	3.3692	3.1931	3.0033
97	4.0724	3.7431	3.5893	3.5380	3.3727	3.1966	3.0068
98	4.0762	3.7467	3.5929	3.5416	3.3762	3.2001	3.0103
99	4.0801	3.7503	3.5964	3.5451	3.3797	3.2036	3.0138
100	4.0838	3.7538	3.5999	3.5485	3.3831	3.2070	3.0172
101	4.0876	3.7573	3.6033	3.5520	3.3865	3.2103	3.0206
102	4.0912	3.7608	3.6067	3.5554	3.3898	3.2137	3.0239
103	4.0949	3.7642	3.6101	3.5587	3.3931	3.2170	3.0272
104	4.0984	3.7676	3.6133	3.5620	3.3964	3.2202	3.0304
105	4.1019	3.7709	3.6166	3.5652	3.3996	3.2234	3.0337
106	4.1053	3.7742	3.6199	3.5684	3.4028	3.2266	3.0369
107	4.1087	3.7774	3.6231	3.5716	3.4060	3.2298	3.0400
108	4.1122	3.7806	3.6262	3.5748	3.4091	3.2329	3.0432
109	4.1155	3.7838	3.6293	3.5779	3.4122	3.2360	3.0462
110	4.1188	3.7869	3.6324	3.5810	3.4152	3.2390	3.0493
111	4.1221	3.7900	3.6355	3.5840	3.4182	3.2420	3.0523
112	4.1254	3.7931	3.6385	3.5870	3.4212	3.2450	3.0553
113	4.1285	3.7961	3.6415	3.5900	3.4241	3.2479	3.0583
114	4.1316	3.7991	3.6445	3.5929	3.4271	3.2508	3.0612
115	4.1347	3.8020	3.6474	3.5958	3.4300	3.2537	3.0641
116	4.1378	3.8049	3.6503	3.5987	3.4328	3.2566	3.0670
117	4.1408	3.8078	3.6531	3.6016	3.4356	3.2594	3.0698
118	4.1438	3.8107	3.6559	3.6044	3.4384	3.2622	3.0726
119	4.1468	3.8136	3.6587	3.6072	3.4412	3.2650	3.0754
120	4.1497	3.8164	3.6615	3.6100	3.4440	3.2677	3.0782
121	4.1526	3.8192	3.6643	3.6127	3.4467	3.2705	3.0809
122	4.1555	3.8219	3.6670	3.6154	3.4494	3.2732	3.0836
123	4.1584	3.8247	3.6697	3.6181	3.4521	3.2758	3.0863
124	4.1612	3.8273	3.6723	3.6207	3.4547	3.2785	3.0890
125	4.1639	3.8300	3.6750	3.6234	3.4573	3.2811	3.0916
126	4.1667	3.8326	3.6775	3.6259	3.4599	3.2837	3.0942
127	4.1695	3.8352	3.6801	3.6285	3.4625	3.2862	3.0968
128	4.1722	3.8378	3.6827	3.6311	3.4650	3.2888	3.0994
129	4.1748	3.8404	3.6852	3.6336	3.4675	3.2913	3.1019
130	4.1775	3.8430	3.6877	3.6361	3.4700	3.2938	3.1045
131	4.1801	3.8454	3.6902	3.6386	3.4725	3.2963	3.1070
132	4.1827	3.8480	3.6927	3.6410	3.4749	3.2988	3.1095
133	4.1852	3.8504	3.6951	3.6435	3.4774	3.3012	3.1119
134	4.1878	3.8528	3.6975	3.6459	3.4798	3.3036	3.1143
135	4.1903	3.8552	3.6999	3.6483	3.4822	3.3060	3.1168
136	4.1928	3.8576	3.7023	3.6506	3.4845	3.3084	3.1191
137	4.1953	3.8600	3.7046	3.6530	3.4869	3.3107	3.1215
138	4.1977	3.8624	3.7069	3.6553	3.4892	3.3130	3.1239
139	4.2002	3.8647	3.7093	3.6576	3.4915	3.3154	3.1262
140	4.2026	3.8670	3.7116	3.6599	3.4938	3.3177	3.1285
141	4.2050	3.8693	3.7138	3.6622	3.4960	3.3199	3.1308
142	4.2073	3.8716	3.7161	3.6644	3.4983	3.3222	3.1331

N	0.1%	0.5%	1%	1.25%	2.5%	5%	10%
143	4.2096	3.8738	3.7183	3.6667	3.5005	3.3244	3.1354
144	4.2119	3.8761	3.7205	3.6689	3.5027	3.3266	3.1376
145	4.2142	3.8783	3.7227	3.6711	3.5049	3.3288	3.1398
146	4.2165	3.8805	3.7249	3.6732	3.5071	3.3310	3.1420
147	4.2187	3.8827	3.7271	3.6754	3.5092	3.3332	3.1442
148	4.2210	3.8848	3.7292	3.6775	3.5114	3.3353	3.1464
149	4.2231	3.8870	3.7313	3.6796	3.5135	3.3375	3.1486
150	4.2253	3.8891	3.7334	3.6818	3.5156	3.3396	3.1507
151	4.2275	3.8912	3.7355	3.6838	3.5177	3.3417	3.1528
152	4.2296	3.8933	3.7376	3.6859	3.5197	3.3438	3.1549
153	4.2317	3.8953	3.7397	3.6880	3.5218	3.3458	3.1570
154	4.2338	3.8974	3.7417	3.6900	3.5238	3.3479	3.1591
155	4.2359	3.8994	3.7437	3.6920	3.5259	3.3499	3.1612
156	4.2380	3.9014	3.7457	3.6940	3.5279	3.3519	3.1632
157	4.2401	3.9034	3.7477	3.6960	3.5299	3.3539	3.1652
158	4.2421	3.9054	3.7497	3.6980	3.5318	3.3559	3.1673
159	4.2441	3.9074	3.7517	3.7000	3.5338	3.3579	3.1693
160	4.2461	3.9093	3.7536	3.7019	3.5358	3.3599	3.1713
161	4.2482	3.9112	3.7556	3.7039	3.5377	3.3618	3.1732
162	4.2501	3.9132	3.7575	3.7058	3.5396	3.3638	3.1752
163	4.2521	3.9151	3.7594	3.7077	3.5415	3.3657	3.1771
164	4.2540	3.9170	3.7613	3.7096	3.5434	3.3676	3.1791
165	4.2560	3.9189	3.7632	3.7115	3.5453	3.3695	3.1810
166	4.2579	3.9208	3.7650	3.7133	3.5471	3.3714	3.1829
167	4.2598	3.9226	3.7669	3.7152	3.5490	3.3733	3.1848
168	4.2617	3.9245	3.7687	3.7170	3.5508	3.3751	3.1867
169	4.2636	3.9263	3.7705	3.7188	3.5527	3.3769	3.1885
170	4.2655	3.9281	3.7723	3.7206	3.5545	3.3788	3.1904
171	4.2674	3.9299	3.7742	3.7224	3.5563	3.3806	3.1923
172	4.2692	3.9317	3.7759	3.7242	3.5581	3.3824	3.1941
173	4.2710	3.9335	3.7777	3.7260	3.5598	3.3842	3.1959
174	4.2728	3.9353	3.7795	3.7277	3.5616	3.3860	3.1977
175	4.2746	3.9370	3.7812	3.7295	3.5634	3.3877	3.1995
176	4.2764	3.9387	3.7829	3.7312	3.5651	3.3895	3.2013
177	4.2782	3.9405	3.7847	3.7329	3.5668	3.3912	3.2031
178	4.2799	3.9422	3.7864	3.7347	3.5686	3.3930	3.2048
179	4.2816	3.9439	3.7881	3.7364	3.5703	3.3947	3.2066
180	4.2834	3.9456	3.7898	3.7380	3.5720	3.3964	3.2083
181	4.2851	3.9473	3.7915	3.7397	3.5737	3.3981	3.2101
182	4.2869	3.9490	3.7931	3.7414	3.5754	3.3998	3.2118
183	4.2886	3.9506	3.7948	3.7431	3.5770	3.4015	3.2135
184	4.2903	3.9523	3.7964	3.7447	3.5787	3.4032	3.2152
185	4.2919	3.9539	3.7981	3.7464	3.5803	3.4048	3.2169
186	4.2936	3.9555	3.7997	3.7480	3.5819	3.4065	3.2185
187	4.2952	3.9572	3.8013	3.7496	3.5836	3.4081	3.2202
188	4.2969	3.9588	3.8029	3.7512	3.5852	3.4097	3.2219
189	4.2985	3.9604	3.8045	3.7528	3.5868	3.4114	3.2235
190	4.3001	3.9620	3.8061	3.7544	3.5884	3.4130	3.2251
191	4.3017	3.9636	3.8077	3.7560	3.5900	3.4146	3.2268
192	4.3033	3.9651	3.8092	3.7575	3.5916	3.4162	3.2284
193	4.3049	3.9667	3.8108	3.7591	3.5931	3.4178	3.2300
194	4.3065	3.9682	3.8123	3.7606	3.5947	3.4193	3.2316
195	4.3081	3.9697	3.8139	3.7622	3.5962	3.4209	3.2332
196	4.3096	3.9713	3.8154	3.7637	3.5978	3.4225	3.2348
197	4.3111	3.9728	3.8169	3.7652	3.5993	3.4240	3.2363
198	4.3126	3.9743	3.8184	3.7667	3.6008	3.4255	3.2379
199	4.3142	3.9758	3.8199	3.7682	3.6023	3.4271	3.2394
200	4.3156	3.9773	3.8214	3.7697	3.6038	3.4286	3.2410
201	4.3186	3.9797	3.8239	3.7721	3.6055	3.4303	3.2424
202	4.3201	3.9812	3.8253	3.7737	3.6071	3.4318	3.2439
203	4.3220	3.9827	3.8269	3.7752	3.6086	3.4333	3.2454
204	4.3230	3.9842	3.8282	3.7767	3.6100	3.4348	3.2470
205	4.3242	3.9861	3.8298	3.7780	3.6114	3.4362	3.2485
206	4.3259	3.9875	3.8314	3.7794	3.6128	3.4377	3.2500
207	4.3276	3.9888	3.8326	3.7811	3.6144	3.4390	3.2514
208	4.3290	3.9897	3.8342	3.7824	3.6157	3.4404	3.2529
209	4.3300	3.9911	3.8358	3.7840	3.6172	3.4420	3.2544
210	4.3303	3.9931	3.8369	3.7853	3.6185	3.4435	3.2558
211	4.3320	3.9943	3.8382	3.7867	3.6199	3.4449	3.2573
212	4.3340	3.9954	3.8396	3.7879	3.6213	3.4463	3.2587
213	4.3354	3.9968	3.8411	3.7894	3.6228	3.4477	3.2601
214	4.3371	3.9984	3.8425	3.7909	3.6242	3.4492	3.2616
215	4.3381	3.9996	3.8440	3.7920	3.6255	3.4506	3.2631
216	4.3399	4.0013	3.8454	3.7935	3.6270	3.4519	3.2645

N	0.1%	0.5%	1%	1.25%	2.5%	5%	10%
217	4.3408	4.0028	3.8467	3.7948	3.6283	3.4534	3.2659
218	4.3426	4.0037	3.8483	3.7960	3.6299	3.4548	3.2673
219	4.3436	4.0048	3.8495	3.7975	3.6314	3.4562	3.2687
220	4.3453	4.0067	3.8508	3.7990	3.6327	3.4576	3.2701
221	4.3473	4.0081	3.8522	3.8004	3.6340	3.4589	3.2714
222	4.3481	4.0092	3.8533	3.8017	3.6354	3.4603	3.2728
223	4.3496	4.0109	3.8547	3.8030	3.6368	3.4615	3.2741
224	4.3515	4.0120	3.8561	3.8042	3.6379	3.4629	3.2754
225	4.3518	4.0130	3.8571	3.8055	3.6392	3.4642	3.2768
226	4.3536	4.0146	3.8584	3.8069	3.6406	3.4656	3.2782
227	4.3550	4.0158	3.8598	3.8081	3.6420	3.4670	3.2796
228	4.3567	4.0172	3.8613	3.8094	3.6432	3.4682	3.2809
229	4.3584	4.0187	3.8624	3.8106	3.6444	3.4695	3.2824
230	4.3599	4.0198	3.8637	3.8119	3.6457	3.4708	3.2836
231	4.3615	4.0212	3.8649	3.8130	3.6472	3.4722	3.2849
232	4.3628	4.0226	3.8660	3.8144	3.6484	3.4735	3.2862
233	4.3636	4.0240	3.8676	3.8155	3.6498	3.4747	3.2876
234	4.3645	4.0251	3.8689	3.8167	3.6509	3.4760	3.2889
235	4.3661	4.0265	3.8700	3.8181	3.6521	3.4773	3.2903
236	4.3676	4.0273	3.8713	3.8193	3.6533	3.4786	3.2915
237	4.3696	4.0288	3.8726	3.8205	3.6547	3.4799	3.2928
238	4.3709	4.0297	3.8736	3.8219	3.6559	3.4812	3.2941
239	4.3719	4.0310	3.8746	3.8229	3.6570	3.4825	3.2954
240	4.3729	4.0320	3.8760	3.8242	3.6581	3.4837	3.2966
241	4.3741	4.0332	3.8773	3.8252	3.6594	3.4849	3.2979
242	4.3750	4.0345	3.8785	3.8265	3.6606	3.4861	3.2990
243	4.3761	4.0361	3.8796	3.8277	3.6617	3.4872	3.3002
244	4.3774	4.0373	3.8806	3.8288	3.6629	3.4885	3.3015
245	4.3783	4.0384	3.8820	3.8301	3.6642	3.4897	3.3027
246	4.3801	4.0395	3.8831	3.8314	3.6654	3.4910	3.3040
247	4.3811	4.0405	3.8844	3.8325	3.6665	3.4921	3.3052
248	4.3822	4.0420	3.8856	3.8336	3.6677	3.4933	3.3063
249	4.3829	4.0430	3.8867	3.8349	3.6688	3.4943	3.3076
250	4.3849	4.0443	3.8880	3.8360	3.6699	3.4956	3.3088
251	4.3860	4.0453	3.8892	3.8371	3.6712	3.4967	3.3101
252	4.3868	4.0466	3.8903	3.8382	3.6724	3.4979	3.3112
253	4.3881	4.0478	3.8915	3.8393	3.6736	3.4991	3.3124
254	4.3898	4.0493	3.8926	3.8402	3.6747	3.5002	3.3136
255	4.3906	4.0501	3.8937	3.8416	3.6760	3.5014	3.3148
256	4.3910	4.0511	3.8948	3.8426	3.6770	3.5025	3.3159
257	4.3914	4.0521	3.8960	3.8438	3.6780	3.5037	3.3171
258	4.3924	4.0530	3.8970	3.8448	3.6792	3.5048	3.3183
259	4.3941	4.0542	3.8979	3.8459	3.6803	3.5060	3.3194
260	4.3949	4.0559	3.8991	3.8469	3.6815	3.5071	3.3205
261	4.3961	4.0569	3.9002	3.8481	3.6826	3.5083	3.3216
262	4.3973	4.0581	3.9015	3.8494	3.6839	3.5094	3.3229
263	4.3986	4.0589	3.9026	3.8506	3.6851	3.5106	3.3240
264	4.3992	4.0600	3.9039	3.8516	3.6861	3.5116	3.3252
265	4.4000	4.0611	3.9048	3.8528	3.6871	3.5127	3.3262
266	4.4011	4.0618	3.9057	3.8538	3.6882	3.5137	3.3274
267	4.4027	4.0627	3.9068	3.8547	3.6892	3.5148	3.3285
268	4.4037	4.0638	3.9077	3.8557	3.6903	3.5159	3.3297
269	4.4047	4.0650	3.9087	3.8566	3.6913	3.5170	3.3308
270	4.4061	4.0662	3.9100	3.8578	3.6925	3.5181	3.3319
271	4.4076	4.0672	3.9109	3.8589	3.6934	3.5192	3.3330
272	4.4091	4.0683	3.9117	3.8600	3.6946	3.5203	3.3342
273	4.4102	4.0694	3.9131	3.8610	3.6956	3.5213	3.3353
274	4.4116	4.0703	3.9138	3.8620	3.6967	3.5224	3.3363
275	4.4125	4.0711	3.9149	3.8630	3.6978	3.5234	3.3374
276	4.4130	4.0722	3.9159	3.8642	3.6987	3.5244	3.3385
277	4.4144	4.0732	3.9169	3.8651	3.6998	3.5254	3.3396
278	4.4149	4.0743	3.9180	3.8660	3.7009	3.5265	3.3406
279	4.4150	4.0755	3.9190	3.8671	3.7019	3.5275	3.3417
280	4.4158	4.0763	3.9200	3.8680	3.7030	3.5286	3.3427
281	4.4165	4.0774	3.9209	3.8690	3.7040	3.5296	3.3438
282	4.4177	4.0782	3.9219	3.8700	3.7050	3.5306	3.3448
283	4.4187	4.0793	3.9229	3.8708	3.7059	3.5317	3.3459
284	4.4209	4.0801	3.9238	3.8717	3.7069	3.5327	3.3470
285	4.4216	4.0813	3.9248	3.8729	3.7079	3.5338	3.3481
286	4.4220	4.0820	3.9259	3.8737	3.7089	3.5348	3.3491
287	4.4232	4.0832	3.9266	3.8748	3.7100	3.5358	3.3501
288	4.4245	4.0839	3.9278	3.8759	3.7110	3.5368	3.3511
289	4.4254	4.0849	3.9286	3.8769	3.7119	3.5377	3.3521
290	4.4264	4.0859	3.9297	3.8780	3.7129	3.5388	3.3531

N	0.1%	0.5%	1%	1.25%	2.5%	5%	10%
291	4.4272	4.0868	3.9308	3.8791	3.7138	3.5397	3.3541
292	4.4279	4.0878	3.9319	3.8803	3.7148	3.5407	3.3552
293	4.4293	4.0884	3.9328	3.8811	3.7158	3.5418	3.3562
294	4.4301	4.0899	3.9337	3.8821	3.7167	3.5428	3.3572
295	4.4307	4.0910	3.9346	3.8830	3.7176	3.5439	3.3582
296	4.4314	4.0919	3.9356	3.8840	3.7186	3.5449	3.3592
297	4.4326	4.0927	3.9365	3.8849	3.7196	3.5458	3.3602
298	4.4326	4.0937	3.9374	3.8857	3.7205	3.5468	3.3612
299	4.4336	4.0947	3.9385	3.8868	3.7214	3.5477	3.3623
300	4.4350	4.0956	3.9395	3.8878	3.7224	3.5487	3.3633
301	4.4358	4.0965	3.9405	3.8888	3.7236	3.5497	3.3643
302	4.4367	4.0973	3.9412	3.8897	3.7245	3.5505	3.3653
303	4.4375	4.0982	3.9421	3.8905	3.7253	3.5515	3.3663
304	4.4388	4.0990	3.9431	3.8917	3.7262	3.5525	3.3672
305	4.4397	4.1001	3.9439	3.8924	3.7270	3.5534	3.3682
306	4.4408	4.1010	3.9449	3.8933	3.7280	3.5542	3.3691
307	4.4414	4.1020	3.9459	3.8943	3.7289	3.5551	3.3700
308	4.4424	4.1030	3.9468	3.8953	3.7300	3.5560	3.3711
309	4.4426	4.1039	3.9474	3.8961	3.7310	3.5569	3.3720
310	4.4429	4.1050	3.9486	3.8971	3.7318	3.5579	3.3730
311	4.4442	4.1060	3.9497	3.8981	3.7327	3.5588	3.3739
312	4.4451	4.1068	3.9507	3.8990	3.7336	3.5598	3.3749
313	4.4460	4.1077	3.9516	3.8998	3.7345	3.5607	3.3759
314	4.4464	4.1088	3.9525	3.9006	3.7354	3.5617	3.3768
315	4.4476	4.1097	3.9532	3.9016	3.7364	3.5626	3.3777
316	4.4482	4.1103	3.9542	3.9026	3.7373	3.5635	3.3787
317	4.4488	4.1110	3.9549	3.9032	3.7382	3.5644	3.3796
318	4.4497	4.1115	3.9557	3.9043	3.7391	3.5652	3.3804
319	4.4504	4.1126	3.9567	3.9051	3.7400	3.5662	3.3814
320	4.4508	4.1134	3.9575	3.9058	3.7409	3.5670	3.3824
321	4.4514	4.1139	3.9583	3.9066	3.7416	3.5679	3.3832
322	4.4526	4.1146	3.9591	3.9075	3.7425	3.5687	3.3841
323	4.4539	4.1156	3.9602	3.9085	3.7434	3.5697	3.3850
324	4.4555	4.1167	3.9611	3.9093	3.7443	3.5705	3.3858
325	4.4559	4.1173	3.9618	3.9102	3.7453	3.5714	3.3868
326	4.4567	4.1183	3.9627	3.9109	3.7460	3.5723	3.3877
327	4.4574	4.1191	3.9635	3.9118	3.7468	3.5732	3.3885
328	4.4589	4.1200	3.9645	3.9126	3.7476	3.5741	3.3894
329	4.4593	4.1208	3.9652	3.9135	3.7485	3.5749	3.3903
330	4.4604	4.1217	3.9658	3.9143	3.7495	3.5758	3.3912
331	4.4606	4.1227	3.9666	3.9152	3.7505	3.5766	3.3920
332	4.4617	4.1231	3.9673	3.9160	3.7511	3.5775	3.3929
333	4.4627	4.1240	3.9682	3.9169	3.7521	3.5783	3.3938
334	4.4631	4.1249	3.9691	3.9177	3.7529	3.5791	3.3947
335	4.4642	4.1255	3.9698	3.9186	3.7537	3.5800	3.3955
336	4.4650	4.1263	3.9708	3.9193	3.7544	3.5808	3.3963
337	4.4657	4.1270	3.9715	3.9201	3.7554	3.5817	3.3971
338	4.4660	4.1280	3.9721	3.9210	3.7561	3.5825	3.3980
339	4.4665	4.1288	3.9731	3.9216	3.7568	3.5833	3.3989
340	4.4672	4.1296	3.9741	3.9226	3.7577	3.5842	3.3997
341	4.4679	4.1304	3.9749	3.9233	3.7586	3.5849	3.4005
342	4.4687	4.1308	3.9755	3.9239	3.7594	3.5857	3.4014
343	4.4691	4.1316	3.9762	3.9244	3.7602	3.5865	3.4022
344	4.4700	4.1324	3.9770	3.9251	3.7610	3.5873	3.4030
345	4.4713	4.1331	3.9778	3.9258	3.7618	3.5881	3.4038
346	4.4717	4.1337	3.9787	3.9267	3.7626	3.5889	3.4047
347	4.4722	4.1345	3.9793	3.9274	3.7633	3.5897	3.4056
348	4.4734	4.1351	3.9800	3.9284	3.7642	3.5905	3.4064
349	4.4741	4.1359	3.9806	3.9290	3.7650	3.5913	3.4072
350	4.4745	4.1367	3.9815	3.9298	3.7658	3.5922	3.4080
351	4.4754	4.1373	3.9821	3.9305	3.7665	3.5930	3.4088
352	4.4759	4.1383	3.9830	3.9313	3.7673	3.5938	3.4096
353	4.4764	4.1391	3.9836	3.9320	3.7681	3.5946	3.4105
354	4.4774	4.1400	3.9846	3.9329	3.7689	3.5953	3.4113
355	4.4780	4.1407	3.9854	3.9336	3.7697	3.5962	3.4121
356	4.4796	4.1415	3.9862	3.9345	3.7704	3.5971	3.4129
357	4.4803	4.1422	3.9869	3.9351	3.7713	3.5978	3.4136
358	4.4811	4.1427	3.9876	3.9360	3.7719	3.5986	3.4145
359	4.4815	4.1435	3.9884	3.9368	3.7726	3.5995	3.4153
360	4.4827	4.1441	3.9893	3.9375	3.7734	3.6002	3.4161
361	4.4829	4.1451	3.9901	3.9381	3.7742	3.6009	3.4169
362	4.4835	4.1458	3.9906	3.9390	3.7750	3.6017	3.4177
363	4.4839	4.1462	3.9911	3.9398	3.7757	3.6025	3.4185
364	4.4844	4.1471	3.9918	3.9404	3.7764	3.6033	3.4192

N	0.1%	0.5%	1%	1.25%	2.5%	5%	10%
365	4.4854	4.1480	3.9926	3.9412	3.7772	3.6040	3.4201
366	4.4864	4.1488	3.9935	3.9421	3.7779	3.6048	3.4209
367	4.4867	4.1495	3.9944	3.9429	3.7787	3.6056	3.4217
368	4.4878	4.1501	3.9950	3.9437	3.7795	3.6064	3.4224
369	4.4884	4.1511	3.9959	3.9446	3.7803	3.6071	3.4232
370	4.4888	4.1519	3.9969	3.9453	3.7812	3.6078	3.4241
371	4.4904	4.1526	3.9973	3.9460	3.7819	3.6086	3.4248
372	4.4911	4.1534	3.9980	3.9467	3.7824	3.6093	3.4256
373	4.4914	4.1542	3.9988	3.9475	3.7832	3.6102	3.4263
374	4.4924	4.1549	3.9998	3.9482	3.7840	3.6110	3.4271
375	4.4932	4.1558	4.0005	3.9488	3.7846	3.6116	3.4279
376	4.4938	4.1565	4.0011	3.9497	3.7854	3.6124	3.4287
377	4.4945	4.1572	4.0018	3.9503	3.7862	3.6131	3.4294
378	4.4950	4.1580	4.0022	3.9510	3.7869	3.6138	3.4301
379	4.4960	4.1585	4.0027	3.9516	3.7876	3.6145	3.4309
380	4.4970	4.1590	4.0036	3.9522	3.7883	3.6152	3.4317
381	4.4977	4.1598	4.0043	3.9530	3.7890	3.6160	3.4324
382	4.4985	4.1603	4.0051	3.9538	3.7897	3.6167	3.4331
383	4.4988	4.1608	4.0057	3.9544	3.7904	3.6175	3.4338
384	4.4998	4.1613	4.0064	3.9552	3.7912	3.6182	3.4346
385	4.5004	4.1619	4.0070	3.9557	3.7919	3.6189	3.4353
386	4.5008	4.1628	4.0076	3.9565	3.7926	3.6197	3.4360
387	4.5023	4.1633	4.0084	3.9573	3.7933	3.6204	3.4368
388	4.5025	4.1640	4.0093	3.9580	3.7940	3.6211	3.4376
389	4.5036	4.1648	4.0098	3.9588	3.7948	3.6218	3.4383
390	4.5037	4.1657	4.0106	3.9595	3.7955	3.6225	3.4390
391	4.5043	4.1663	4.0113	3.9602	3.7962	3.6233	3.4398
392	4.5050	4.1667	4.0120	3.9610	3.7968	3.6240	3.4405
393	4.5065	4.1672	4.0126	3.9617	3.7976	3.6248	3.4413
394	4.5071	4.1681	4.0133	3.9622	3.7983	3.6255	3.4420
395	4.5079	4.1688	4.0139	3.9629	3.7989	3.6261	3.4427
396	4.5090	4.1695	4.0145	3.9638	3.7995	3.6269	3.4434
397	4.5092	4.1701	4.0154	3.9644	3.8002	3.6276	3.4442
398	4.5099	4.1710	4.0162	3.9650	3.8009	3.6283	3.4449
399	4.5101	4.1714	4.0169	3.9657	3.8017	3.6291	3.4456
400	4.5102	4.1722	4.0174	3.9664	3.8023	3.6297	3.4463
401	4.5113	4.1728	4.0181	3.9672	3.8029	3.6303	3.4470
402	4.5121	4.1735	4.0188	3.9678	3.8035	3.6310	3.4477
403	4.5125	4.1744	4.0193	3.9684	3.8042	3.6316	3.4484
404	4.5130	4.1748	4.0200	3.9689	3.8048	3.6322	3.4491
405	4.5140	4.1757	4.0207	3.9696	3.8056	3.6330	3.4498
406	4.5142	4.1767	4.0215	3.9703	3.8062	3.6337	3.4505
407	4.5149	4.1772	4.0222	3.9712	3.8067	3.6343	3.4512
408	4.5154	4.1778	4.0226	3.9717	3.8075	3.6350	3.4519
409	4.5155	4.1783	4.0233	3.9723	3.8082	3.6357	3.4526
410	4.5169	4.1789	4.0240	3.9731	3.8087	3.6363	3.4533
411	4.5178	4.1794	4.0246	3.9737	3.8094	3.6370	3.4540
412	4.5185	4.1799	4.0252	3.9743	3.8100	3.6377	3.4547
413	4.5188	4.1804	4.0258	3.9748	3.8107	3.6384	3.4555
414	4.5196	4.1809	4.0263	3.9755	3.8114	3.6391	3.4562
415	4.5202	4.1817	4.0270	3.9761	3.8119	3.6398	3.4568
416	4.5201	4.1823	4.0277	3.9766	3.8126	3.6404	3.4575
417	4.5211	4.1828	4.0284	3.9775	3.8133	3.6410	3.4582
418	4.5213	4.1836	4.0292	3.9779	3.8139	3.6417	3.4589
419	4.5223	4.1840	4.0296	3.9786	3.8145	3.6424	3.4596
420	4.5226	4.1845	4.0304	3.9793	3.8153	3.6430	3.4602
421	4.5234	4.1852	4.0309	3.9799	3.8160	3.6436	3.4609
422	4.5239	4.1858	4.0315	3.9805	3.8165	3.6443	3.4616
423	4.5242	4.1866	4.0323	3.9812	3.8173	3.6450	3.4622
424	4.5242	4.1874	4.0326	3.9818	3.8180	3.6456	3.4629
425	4.5249	4.1878	4.0333	3.9823	3.8185	3.6462	3.4635
426	4.5257	4.1885	4.0340	3.9831	3.8192	3.6469	3.4642
427	4.5263	4.1891	4.0348	3.9836	3.8199	3.6476	3.4648
428	4.5268	4.1896	4.0351	3.9842	3.8204	3.6482	3.4654
429	4.5272	4.1903	4.0356	3.9851	3.8211	3.6489	3.4661
430	4.5274	4.1908	4.0363	3.9856	3.8218	3.6496	3.4668
431	4.5276	4.1915	4.0368	3.9863	3.8224	3.6501	3.4674
432	4.5282	4.1921	4.0375	3.9869	3.8230	3.6509	3.4681
433	4.5288	4.1928	4.0382	3.9875	3.8236	3.6515	3.4687
434	4.5297	4.1933	4.0385	3.9881	3.8243	3.6520	3.4694
435	4.5303	4.1940	4.0393	3.9887	3.8248	3.6527	3.4701
436	4.5309	4.1945	4.0399	3.9892	3.8254	3.6534	3.4707
437	4.5317	4.1950	4.0407	3.9899	3.8261	3.6540	3.4713
438	4.5321	4.1955	4.0412	3.9905	3.8266	3.6546	3.4720

N	0.1%	0.5%	1%	1.25%	2.5%	5%	10%
439	4.5320	4.1960	4.0419	3.9910	3.8273	3.6552	3.4726
440	4.5328	4.1966	4.0425	3.9916	3.8279	3.6558	3.4733
441	4.5331	4.1972	4.0431	3.9923	3.8285	3.6565	3.4739
442	4.5339	4.1977	4.0438	3.9928	3.8291	3.6570	3.4745
443	4.5346	4.1980	4.0444	3.9933	3.8297	3.6577	3.4751
444	4.5355	4.1987	4.0449	3.9940	3.8303	3.6583	3.4757
445	4.5362	4.1993	4.0454	3.9946	3.8310	3.6589	3.4763
446	4.5368	4.1998	4.0460	3.9952	3.8315	3.6595	3.4769
447	4.5371	4.2004	4.0465	3.9956	3.8320	3.6601	3.4775
448	4.5370	4.2010	4.0469	3.9963	3.8326	3.6607	3.4781
449	4.5378	4.2018	4.0474	3.9967	3.8333	3.6613	3.4788
450	4.5381	4.2021	4.0480	3.9973	3.8339	3.6619	3.4794
451	4.5386	4.2029	4.0486	3.9978	3.8344	3.6625	3.4801
452	4.5392	4.2037	4.0491	3.9983	3.8352	3.6632	3.4806
453	4.5397	4.2039	4.0497	3.9990	3.8356	3.6638	3.4813
454	4.5403	4.2047	4.0503	3.9996	3.8362	3.6645	3.4819
455	4.5406	4.2054	4.0509	4.0001	3.8369	3.6651	3.4826
456	4.5415	4.2059	4.0517	4.0006	3.8375	3.6656	3.4832
457	4.5419	4.2064	4.0522	4.0013	3.8381	3.6662	3.4837
458	4.5424	4.2071	4.0527	4.0019	3.8387	3.6668	3.4844
459	4.5433	4.2073	4.0532	4.0025	3.8393	3.6674	3.4850
460	4.5438	4.2078	4.0539	4.0031	3.8399	3.6681	3.4856
461	4.5445	4.2085	4.0543	4.0037	3.8403	3.6686	3.4863
462	4.5447	4.2090	4.0547	4.0043	3.8409	3.6691	3.4869
463	4.5452	4.2095	4.0553	4.0048	3.8415	3.6697	3.4875
464	4.5463	4.2102	4.0559	4.0054	3.8422	3.6704	3.4880
465	4.5465	4.2106	4.0563	4.0059	3.8428	3.6710	3.4886
466	4.5470	4.2112	4.0569	4.0064	3.8434	3.6716	3.4893
467	4.5478	4.2117	4.0573	4.0069	3.8439	3.6721	3.4898
468	4.5484	4.2122	4.0581	4.0076	3.8444	3.6727	3.4904
469	4.5491	4.2128	4.0586	4.0080	3.8450	3.6732	3.4910
470	4.5497	4.2132	4.0589	4.0085	3.8455	3.6739	3.4916
471	4.5501	4.2136	4.0596	4.0090	3.8461	3.6745	3.4922
472	4.5507	4.2141	4.0601	4.0096	3.8467	3.6751	3.4928
473	4.5508	4.2147	4.0606	4.0101	3.8474	3.6756	3.4934
474	4.5519	4.2153	4.0613	4.0109	3.8479	3.6762	3.4940
475	4.5527	4.2161	4.0619	4.0114	3.8485	3.6768	3.4946
476	4.5527	4.2161	4.0624	4.0120	3.8491	3.6773	3.4952
477	4.5528	4.2169	4.0629	4.0125	3.8496	3.6780	3.4957
478	4.5533	4.2173	4.0635	4.0130	3.8502	3.6785	3.4963
479	4.5538	4.2179	4.0641	4.0135	3.8508	3.6791	3.4969
480	4.5539	4.2186	4.0646	4.0141	3.8513	3.6797	3.4975
481	4.5547	4.2192	4.0651	4.0145	3.8518	3.6802	3.4981
482	4.5555	4.2198	4.0657	4.0151	3.8524	3.6808	3.4987
483	4.5562	4.2203	4.0663	4.0157	3.8529	3.6814	3.4993
484	4.5564	4.2208	4.0666	4.0161	3.8534	3.6820	3.4998
485	4.5568	4.2210	4.0672	4.0165	3.8540	3.6825	3.5004
486	4.5575	4.2214	4.0677	4.0170	3.8545	3.6831	3.5010
487	4.5578	4.2220	4.0682	4.0175	3.8550	3.6836	3.5015
488	4.5584	4.2228	4.0686	4.0182	3.8555	3.6842	3.5021
489	4.5588	4.2235	4.0693	4.0187	3.8561	3.6848	3.5027
490	4.5588	4.2240	4.0699	4.0192	3.8565	3.6853	3.5033
491	4.5591	4.2244	4.0704	4.0198	3.8571	3.6859	3.5039
492	4.5593	4.2249	4.0710	4.0202	3.8577	3.6865	3.5044
493	4.5603	4.2252	4.0715	4.0209	3.8582	3.6871	3.5051
494	4.5607	4.2260	4.0721	4.0214	3.8588	3.6877	3.5056
495	4.5612	4.2262	4.0726	4.0220	3.8593	3.6882	3.5062
496	4.5615	4.2269	4.0732	4.0226	3.8598	3.6887	3.5068
497	4.5622	4.2275	4.0736	4.0230	3.8603	3.6892	3.5074
498	4.5626	4.2280	4.0740	4.0236	3.8609	3.6898	3.5079
499	4.5628	4.2286	4.0746	4.0239	3.8615	3.6903	3.5084
500	4.5629	4.2291	4.0751	4.0244	3.8620	3.6908	3.5090
501	4.5636	4.2295	4.0758	4.0250	3.8625	3.6914	3.5095
502	4.5647	4.2302	4.0763	4.0255	3.8630	3.6919	3.5101
503	4.5649	4.2307	4.0767	4.0259	3.8634	3.6925	3.5106
504	4.5653	4.2313	4.0772	4.0264	3.8639	3.6930	3.5112
505	4.5660	4.2318	4.0779	4.0269	3.8645	3.6936	3.5118
506	4.5667	4.2322	4.0782	4.0274	3.8650	3.6940	3.5124
507	4.5669	4.2330	4.0788	4.0280	3.8657	3.6946	3.5129
508	4.5678	4.2333	4.0793	4.0286	3.8662	3.6952	3.5134
509	4.5684	4.2336	4.0799	4.0290	3.8668	3.6957	3.5140
510	4.5685	4.2339	4.0803	4.0297	3.8673	3.6962	3.5145
511	4.5689	4.2345	4.0810	4.0301	3.8678	3.6968	3.5151
512	4.5697	4.2350	4.0814	4.0306	3.8684	3.6974	3.5156

N	0.1%	0.5%	1%	1.25%	2.5%	5%	10%
513	4.5704	4.2352	4.0819	4.0310	3.8689	3.6979	3.5162
514	4.5711	4.2356	4.0825	4.0316	3.8695	3.6985	3.5168
515	4.5712	4.2360	4.0829	4.0320	3.8699	3.6990	3.5173
516	4.5711	4.2366	4.0832	4.0325	3.8704	3.6995	3.5178
517	4.5717	4.2369	4.0838	4.0330	3.8709	3.7000	3.5184
518	4.5725	4.2374	4.0844	4.0334	3.8713	3.7005	3.5189
519	4.5729	4.2380	4.0848	4.0340	3.8719	3.7010	3.5195
520	4.5730	4.2387	4.0855	4.0346	3.8723	3.7015	3.5200
521	4.5730	4.2392	4.0859	4.0351	3.8728	3.7021	3.5206
522	4.5727	4.2393	4.0864	4.0356	3.8734	3.7025	3.5211
523	4.5738	4.2398	4.0869	4.0362	3.8739	3.7031	3.5216
524	4.5745	4.2406	4.0874	4.0366	3.8745	3.7036	3.5221
525	4.5748	4.2410	4.0878	4.0370	3.8750	3.7041	3.5226
526	4.5748	4.2416	4.0881	4.0376	3.8756	3.7047	3.5231
527	4.5754	4.2421	4.0887	4.0379	3.8759	3.7052	3.5237
528	4.5760	4.2426	4.0894	4.0385	3.8764	3.7057	3.5242
529	4.5771	4.2431	4.0898	4.0390	3.8768	3.7062	3.5247
530	4.5773	4.2435	4.0903	4.0395	3.8775	3.7067	3.5252
531	4.5777	4.2441	4.0907	4.0400	3.8778	3.7071	3.5258
532	4.5785	4.2445	4.0912	4.0405	3.8784	3.7077	3.5263
533	4.5787	4.2449	4.0916	4.0409	3.8790	3.7082	3.5268
534	4.5788	4.2452	4.0921	4.0413	3.8794	3.7087	3.5273
535	4.5795	4.2458	4.0926	4.0418	3.8799	3.7092	3.5278
536	4.5802	4.2463	4.0930	4.0422	3.8803	3.7097	3.5284
537	4.5802	4.2468	4.0934	4.0425	3.8808	3.7102	3.5289
538	4.5805	4.2474	4.0941	4.0431	3.8813	3.7107	3.5294
539	4.5806	4.2480	4.0947	4.0438	3.8819	3.7111	3.5299
540	4.5814	4.2485	4.0952	4.0443	3.8824	3.7116	3.5304
541	4.5820	4.2490	4.0956	4.0449	3.8828	3.7121	3.5309
542	4.5827	4.2495	4.0961	4.0453	3.8833	3.7125	3.5314
543	4.5829	4.2499	4.0966	4.0458	3.8837	3.7130	3.5319
544	4.5835	4.2503	4.0972	4.0462	3.8841	3.7135	3.5324
545	4.5836	4.2507	4.0977	4.0466	3.8846	3.7140	3.5329
546	4.5841	4.2512	4.0981	4.0470	3.8850	3.7146	3.5335
547	4.5848	4.2517	4.0983	4.0474	3.8855	3.7151	3.5340
548	4.5848	4.2523	4.0988	4.0480	3.8860	3.7156	3.5345
549	4.5849	4.2528	4.0993	4.0484	3.8864	3.7160	3.5350
550	4.5851	4.2532	4.0997	4.0489	3.8868	3.7165	3.5355
551	4.5852	4.2536	4.1002	4.0492	3.8873	3.7170	3.5360
552	4.5858	4.2539	4.1004	4.0497	3.8878	3.7176	3.5365
553	4.5862	4.2545	4.1009	4.0502	3.8882	3.7181	3.5369
554	4.5870	4.2548	4.1013	4.0507	3.8887	3.7186	3.5374
555	4.5873	4.2554	4.1018	4.0510	3.8892	3.7190	3.5379
556	4.5880	4.2559	4.1023	4.0515	3.8897	3.7195	3.5384
557	4.5886	4.2561	4.1026	4.0520	3.8902	3.7199	3.5388
558	4.5889	4.2567	4.1032	4.0525	3.8906	3.7204	3.5394
559	4.5889	4.2572	4.1036	4.0529	3.8911	3.7209	3.5399
560	4.5897	4.2576	4.1038	4.0532	3.8915	3.7213	3.5404
561	4.5900	4.2580	4.1043	4.0535	3.8920	3.7217	3.5408
562	4.5905	4.2586	4.1047	4.0541	3.8924	3.7222	3.5413
563	4.5908	4.2591	4.1051	4.0547	3.8929	3.7227	3.5418
564	4.5916	4.2595	4.1056	4.0553	3.8934	3.7232	3.5423
565	4.5923	4.2601	4.1061	4.0559	3.8939	3.7237	3.5428
566	4.5925	4.2606	4.1067	4.0564	3.8944	3.7241	3.5433
567	4.5935	4.2608	4.1072	4.0567	3.8948	3.7246	3.5438
568	4.5934	4.2610	4.1077	4.0570	3.8954	3.7250	3.5444
569	4.5937	4.2614	4.1082	4.0576	3.8958	3.7255	3.5448
570	4.5943	4.2619	4.1087	4.0580	3.8962	3.7259	3.5452
571	4.5944	4.2624	4.1091	4.0585	3.8967	3.7264	3.5458
572	4.5946	4.2628	4.1096	4.0589	3.8971	3.7269	3.5462
573	4.5954	4.2635	4.1100	4.0593	3.8976	3.7273	3.5467
574	4.5963	4.2639	4.1104	4.0597	3.8981	3.7278	3.5472
575	4.5972	4.2640	4.1107	4.0600	3.8985	3.7282	3.5476
576	4.5977	4.2646	4.1110	4.0604	3.8990	3.7287	3.5481
577	4.5977	4.2651	4.1114	4.0610	3.8994	3.7292	3.5486
578	4.5988	4.2654	4.1119	4.0615	3.8998	3.7296	3.5490
579	4.5993	4.2657	4.1124	4.0620	3.9002	3.7301	3.5495
580	4.6000	4.2662	4.1128	4.0625	3.9006	3.7305	3.5500
581	4.6003	4.2666	4.1133	4.0629	3.9010	3.7310	3.5504
582	4.6001	4.2669	4.1136	4.0633	3.9016	3.7315	3.5509
583	4.6004	4.2675	4.1141	4.0638	3.9019	3.7320	3.5514
584	4.6005	4.2680	4.1145	4.0643	3.9024	3.7324	3.5518
585	4.6007	4.2684	4.1150	4.0647	3.9028	3.7329	3.5523
586	4.6016	4.2690	4.1155	4.0650	3.9033	3.7333	3.5527

N	0.1%	0.5%	1%	1.25%	2.5%	5%	10%
587	4.6015	4.2693	4.1158	4.0654	3.9037	3.7337	3.5532
588	4.6019	4.2696	4.1162	4.0658	3.9041	3.7341	3.5536
589	4.6022	4.2701	4.1167	4.0662	3.9045	3.7346	3.5541
590	4.6028	4.2706	4.1171	4.0668	3.9050	3.7350	3.5546
591	4.6032	4.2708	4.1176	4.0671	3.9054	3.7355	3.5550
592	4.6035	4.2713	4.1179	4.0674	3.9058	3.7359	3.5554
593	4.6038	4.2717	4.1184	4.0678	3.9062	3.7364	3.5559
594	4.6042	4.2721	4.1189	4.0683	3.9067	3.7368	3.5564
595	4.6046	4.2724	4.1193	4.0688	3.9072	3.7373	3.5568
596	4.6052	4.2726	4.1198	4.0691	3.9076	3.7378	3.5573
597	4.6051	4.2730	4.1202	4.0694	3.9081	3.7382	3.5577
598	4.6057	4.2734	4.1206	4.0699	3.9086	3.7387	3.5581
599	4.6059	4.2738	4.1208	4.0704	3.9090	3.7392	3.5586
600	4.6060	4.2744	4.1213	4.0708	3.9095	3.7397	3.5590
601	4.6066	4.2749	4.1218	4.0710	3.9099	3.7400	3.5595
602	4.6066	4.2752	4.1220	4.0714	3.9103	3.7405	3.5599
603	4.6068	4.2756	4.1225	4.0719	3.9107	3.7410	3.5604
604	4.6070	4.2759	4.1228	4.0723	3.9111	3.7414	3.5609
605	4.6075	4.2763	4.1231	4.0726	3.9115	3.7419	3.5614
606	4.6079	4.2767	4.1236	4.0730	3.9120	3.7423	3.5618
607	4.6083	4.2770	4.1241	4.0734	3.9125	3.7427	3.5623
608	4.6086	4.2776	4.1246	4.0738	3.9129	3.7431	3.5627
609	4.6093	4.2782	4.1249	4.0744	3.9133	3.7436	3.5632
610	4.6101	4.2785	4.1252	4.0747	3.9138	3.7440	3.5636
611	4.6102	4.2788	4.1256	4.0753	3.9142	3.7445	3.5641
612	4.6105	4.2793	4.1261	4.0757	3.9147	3.7449	3.5646
613	4.6105	4.2798	4.1265	4.0759	3.9150	3.7454	3.5650
614	4.6110	4.2802	4.1270	4.0764	3.9154	3.7458	3.5655
615	4.6112	4.2807	4.1274	4.0768	3.9158	3.7462	3.5659
616	4.6115	4.2810	4.1278	4.0771	3.9163	3.7467	3.5664
617	4.6120	4.2814	4.1282	4.0775	3.9167	3.7470	3.5668
618	4.6119	4.2819	4.1286	4.0779	3.9171	3.7474	3.5672
619	4.6119	4.2822	4.1290	4.0783	3.9175	3.7479	3.5677
620	4.6126	4.2827	4.1294	4.0787	3.9179	3.7483	3.5681
621	4.6133	4.2830	4.1298	4.0792	3.9182	3.7488	3.5686
622	4.6138	4.2833	4.1301	4.0795	3.9188	3.7492	3.5690
623	4.6138	4.2837	4.1305	4.0799	3.9192	3.7496	3.5694
624	4.6144	4.2839	4.1310	4.0802	3.9196	3.7500	3.5698
625	4.6151	4.2846	4.1315	4.0807	3.9201	3.7504	3.5702
626	4.6153	4.2850	4.1318	4.0811	3.9206	3.7508	3.5707
627	4.6156	4.2853	4.1323	4.0815	3.9210	3.7512	3.5711
628	4.6161	4.2857	4.1327	4.0820	3.9214	3.7517	3.5715
629	4.6167	4.2861	4.1331	4.0823	3.9217	3.7521	3.5719
630	4.6171	4.2864	4.1334	4.0828	3.9222	3.7526	3.5724
631	4.6170	4.2868	4.1338	4.0832	3.9226	3.7530	3.5729
632	4.6175	4.2872	4.1342	4.0835	3.9230	3.7535	3.5733
633	4.6179	4.2876	4.1346	4.0838	3.9235	3.7539	3.5737
634	4.6185	4.2880	4.1350	4.0843	3.9239	3.7543	3.5742
635	4.6193	4.2884	4.1354	4.0845	3.9243	3.7547	3.5746
636	4.6197	4.2890	4.1357	4.0851	3.9248	3.7552	3.5750
637	4.6201	4.2894	4.1359	4.0856	3.9251	3.7556	3.5755
638	4.6204	4.2899	4.1364	4.0858	3.9254	3.7560	3.5759
639	4.6210	4.2903	4.1369	4.0863	3.9259	3.7564	3.5763
640	4.6215	4.2907	4.1372	4.0868	3.9262	3.7568	3.5768
641	4.6219	4.2910	4.1376	4.0871	3.9266	3.7572	3.5772
642	4.6225	4.2915	4.1381	4.0875	3.9270	3.7576	3.5776
643	4.6230	4.2917	4.1384	4.0879	3.9275	3.7580	3.5780
644	4.6235	4.2919	4.1388	4.0883	3.9278	3.7584	3.5784
645	4.6239	4.2922	4.1392	4.0888	3.9282	3.7588	3.5788
646	4.6242	4.2925	4.1395	4.0892	3.9285	3.7592	3.5792
647	4.6236	4.2929	4.1399	4.0895	3.9290	3.7596	3.5797
648	4.6245	4.2933	4.1403	4.0899	3.9294	3.7601	3.5800
649	4.6245	4.2936	4.1408	4.0903	3.9298	3.7605	3.5805
650	4.6252	4.2936	4.1411	4.0907	3.9302	3.7609	3.5809
651	4.6255	4.2943	4.1414	4.0911	3.9305	3.7613	3.5814
652	4.6257	4.2945	4.1419	4.0915	3.9310	3.7617	3.5818
653	4.6261	4.2948	4.1422	4.0918	3.9313	3.7621	3.5822
654	4.6265	4.2950	4.1427	4.0923	3.9317	3.7625	3.5826
655	4.6269	4.2954	4.1429	4.0927	3.9320	3.7629	3.5831
656	4.6278	4.2959	4.1434	4.0931	3.9324	3.7633	3.5835
657	4.6281	4.2962	4.1437	4.0936	3.9328	3.7638	3.5839
658	4.6285	4.2965	4.1442	4.0938	3.9332	3.7642	3.5843
659	4.6286	4.2971	4.1447	4.0942	3.9336	3.7646	3.5847
660	4.6291	4.2975	4.1448	4.0946	3.9339	3.7650	3.5852

N	0.1%	0.5%	1%	1.25%	2.5%	5%	10%
661	4.6292	4.2978	4.1451	4.0948	3.9343	3.7653	3.5855
662	4.6296	4.2982	4.1457	4.0953	3.9347	3.7658	3.5860
663	4.6302	4.2984	4.1461	4.0957	3.9350	3.7661	3.5864
664	4.6306	4.2987	4.1464	4.0960	3.9354	3.7666	3.5868
665	4.6307	4.2992	4.1468	4.0965	3.9358	3.7670	3.5872
666	4.6309	4.2996	4.1472	4.0968	3.9362	3.7673	3.5876
667	4.6310	4.3000	4.1476	4.0972	3.9366	3.7677	3.5880
668	4.6313	4.3005	4.1479	4.0976	3.9370	3.7682	3.5884
669	4.6315	4.3011	4.1482	4.0981	3.9374	3.7685	3.5888
670	4.6318	4.3014	4.1486	4.0983	3.9378	3.7690	3.5892
671	4.6325	4.3017	4.1490	4.0987	3.9381	3.7693	3.5896
672	4.6327	4.3020	4.1493	4.0991	3.9385	3.7697	3.5900
673	4.6332	4.3022	4.1498	4.0995	3.9388	3.7701	3.5905
674	4.6339	4.3027	4.1502	4.0999	3.9393	3.7705	3.5908
675	4.6345	4.3031	4.1506	4.1003	3.9397	3.7709	3.5912
676	4.6351	4.3035	4.1508	4.1006	3.9400	3.7713	3.5916
677	4.6353	4.3039	4.1511	4.1010	3.9404	3.7717	3.5920
678	4.6354	4.3043	4.1515	4.1014	3.9407	3.7720	3.5924
679	4.6359	4.3045	4.1520	4.1019	3.9411	3.7724	3.5928
680	4.6358	4.3049	4.1521	4.1021	3.9415	3.7727	3.5932
681	4.6362	4.3052	4.1526	4.1025	3.9419	3.7732	3.5936
682	4.6367	4.3055	4.1529	4.1028	3.9423	3.7735	3.5940
683	4.6367	4.3060	4.1532	4.1032	3.9427	3.7740	3.5945
684	4.6373	4.3063	4.1535	4.1035	3.9429	3.7744	3.5949
685	4.6376	4.3068	4.1540	4.1040	3.9434	3.7748	3.5953
686	4.6380	4.3070	4.1543	4.1043	3.9438	3.7751	3.5957
687	4.6381	4.3073	4.1546	4.1047	3.9442	3.7755	3.5961
688	4.6387	4.3078	4.1550	4.1050	3.9446	3.7760	3.5964
689	4.6392	4.3083	4.1554	4.1054	3.9450	3.7763	3.5968
690	4.6394	4.3086	4.1559	4.1056	3.9453	3.7767	3.5972
691	4.6399	4.3088	4.1562	4.1059	3.9457	3.7771	3.5976
692	4.6402	4.3089	4.1565	4.1064	3.9461	3.7775	3.5980
693	4.6405	4.3093	4.1568	4.1068	3.9465	3.7778	3.5984
694	4.6412	4.3098	4.1571	4.1071	3.9468	3.7782	3.5987
695	4.6417	4.3100	4.1576	4.1075	3.9472	3.7786	3.5991
696	4.6418	4.3104	4.1580	4.1079	3.9476	3.7789	3.5995
697	4.6422	4.3109	4.1585	4.1083	3.9479	3.7793	3.5999
698	4.6425	4.3111	4.1587	4.1086	3.9483	3.7797	3.6003
699	4.6428	4.3116	4.1590	4.1090	3.9486	3.7801	3.6006
700	4.6428	4.3119	4.1594	4.1093	3.9490	3.7804	3.6010
701	4.6431	4.3122	4.1596	4.1097	3.9493	3.7809	3.6014
702	4.6435	4.3125	4.1601	4.1100	3.9497	3.7813	3.6018
703	4.6440	4.3129	4.1605	4.1104	3.9501	3.7816	3.6022
704	4.6442	4.3134	4.1607	4.1106	3.9504	3.7820	3.6026
705	4.6446	4.3135	4.1611	4.1110	3.9508	3.7824	3.6030
706	4.6449	4.3138	4.1615	4.1114	3.9510	3.7827	3.6033
707	4.6450	4.3144	4.1617	4.1118	3.9513	3.7831	3.6037
708	4.6456	4.3147	4.1621	4.1120	3.9518	3.7835	3.6041
709	4.6464	4.3151	4.1624	4.1123	3.9522	3.7838	3.6045
710	4.6465	4.3155	4.1628	4.1127	3.9525	3.7842	3.6049
711	4.6470	4.3158	4.1630	4.1129	3.9529	3.7846	3.6053
712	4.6471	4.3161	4.1633	4.1134	3.9532	3.7850	3.6057
713	4.6482	4.3163	4.1637	4.1138	3.9536	3.7853	3.6060
714	4.6491	4.3167	4.1640	4.1141	3.9539	3.7858	3.6064
715	4.6491	4.3168	4.1642	4.1145	3.9543	3.7861	3.6068
716	4.6492	4.3173	4.1644	4.1147	3.9547	3.7865	3.6072
717	4.6496	4.3176	4.1647	4.1150	3.9549	3.7868	3.6075
718	4.6502	4.3180	4.1651	4.1153	3.9554	3.7871	3.6079
719	4.6505	4.3182	4.1655	4.1157	3.9557	3.7875	3.6083
720	4.6509	4.3186	4.1658	4.1160	3.9560	3.7879	3.6087
721	4.6508	4.3191	4.1662	4.1165	3.9564	3.7882	3.6090
722	4.6512	4.3196	4.1665	4.1168	3.9568	3.7887	3.6094
723	4.6515	4.3198	4.1667	4.1172	3.9572	3.7891	3.6098
724	4.6517	4.3202	4.1671	4.1175	3.9575	3.7894	3.6102
725	4.6523	4.3206	4.1676	4.1177	3.9578	3.7898	3.6105
726	4.6530	4.3207	4.1679	4.1181	3.9583	3.7902	3.6109
727	4.6532	4.3211	4.1683	4.1184	3.9586	3.7905	3.6112
728	4.6532	4.3216	4.1687	4.1188	3.9589	3.7909	3.6116
729	4.6537	4.3220	4.1690	4.1192	3.9592	3.7913	3.6120
730	4.6537	4.3225	4.1693	4.1196	3.9596	3.7917	3.6124
731	4.6540	4.3226	4.1696	4.1199	3.9599	3.7920	3.6128
732	4.6548	4.3230	4.1699	4.1202	3.9603	3.7923	3.6131
733	4.6554	4.3232	4.1704	4.1206	3.9607	3.7927	3.6135
734	4.6549	4.3237	4.1706	4.1210	3.9610	3.7931	3.6139

N	0.1%	0.5%	1%	1.25%	2.5%	5%	10%
735	4.6554	4.3240	4.1710	4.1213	3.9614	3.7935	3.6142
736	4.6556	4.3244	4.1714	4.1217	3.9617	3.7938	3.6146
737	4.6556	4.3246	4.1717	4.1222	3.9620	3.7941	3.6149
738	4.6561	4.3251	4.1720	4.1225	3.9622	3.7944	3.6153
739	4.6565	4.3253	4.1726	4.1229	3.9626	3.7947	3.6157
740	4.6570	4.3256	4.1728	4.1231	3.9630	3.7951	3.6161
741	4.6570	4.3261	4.1731	4.1234	3.9633	3.7954	3.6164
742	4.6577	4.3263	4.1735	4.1237	3.9637	3.7958	3.6168
743	4.6579	4.3268	4.1738	4.1242	3.9640	3.7962	3.6171
744	4.6583	4.3271	4.1743	4.1245	3.9644	3.7965	3.6175
745	4.6586	4.3276	4.1746	4.1248	3.9648	3.7969	3.6179
746	4.6587	4.3277	4.1748	4.1251	3.9651	3.7973	3.6182
747	4.6592	4.3280	4.1751	4.1255	3.9654	3.7976	3.6186
748	4.6594	4.3283	4.1755	4.1257	3.9658	3.7980	3.6190
749	4.6598	4.3286	4.1758	4.1261	3.9661	3.7983	3.6193
750	4.6600	4.3289	4.1762	4.1263	3.9664	3.7986	3.6196
751	4.6606	4.3292	4.1765	4.1267	3.9667	3.7989	3.6200
752	4.6606	4.3297	4.1769	4.1269	3.9670	3.7993	3.6204
753	4.6612	4.3301	4.1771	4.1272	3.9675	3.7997	3.6207
754	4.6618	4.3303	4.1774	4.1275	3.9678	3.8000	3.6211
755	4.6618	4.3304	4.1777	4.1279	3.9681	3.8003	3.6215
756	4.6618	4.3306	4.1781	4.1283	3.9684	3.8006	3.6218
757	4.6621	4.3309	4.1784	4.1286	3.9687	3.8010	3.6222
758	4.6624	4.3313	4.1787	4.1288	3.9690	3.8013	3.6225
759	4.6632	4.3316	4.1790	4.1291	3.9694	3.8016	3.6229
760	4.6630	4.3319	4.1794	4.1295	3.9697	3.8020	3.6232
761	4.6634	4.3321	4.1798	4.1296	3.9700	3.8023	3.6236
762	4.6636	4.3326	4.1801	4.1300	3.9704	3.8026	3.6239
763	4.6640	4.3327	4.1803	4.1304	3.9707	3.8030	3.6242
764	4.6647	4.3333	4.1807	4.1308	3.9711	3.8033	3.6246
765	4.6648	4.3336	4.1809	4.1311	3.9713	3.8037	3.6249
766	4.6646	4.3338	4.1813	4.1315	3.9717	3.8040	3.6253
767	4.6654	4.3340	4.1816	4.1318	3.9720	3.8044	3.6256
768	4.6656	4.3343	4.1819	4.1321	3.9723	3.8047	3.6260
769	4.6659	4.3347	4.1822	4.1324	3.9728	3.8051	3.6264
770	4.6661	4.3350	4.1826	4.1326	3.9730	3.8054	3.6267
771	4.6663	4.3354	4.1829	4.1330	3.9733	3.8058	3.6271
772	4.6668	4.3357	4.1833	4.1333	3.9736	3.8061	3.6274
773	4.6670	4.3362	4.1835	4.1336	3.9739	3.8064	3.6278
774	4.6673	4.3364	4.1839	4.1338	3.9742	3.8067	3.6281
775	4.6678	4.3367	4.1842	4.1342	3.9746	3.8070	3.6284
776	4.6684	4.3370	4.1846	4.1345	3.9749	3.8073	3.6288
777	4.6687	4.3372	4.1848	4.1348	3.9752	3.8076	3.6291
778	4.6688	4.3376	4.1851	4.1351	3.9756	3.8080	3.6294
779	4.6689	4.3379	4.1853	4.1354	3.9759	3.8083	3.6298
780	4.6691	4.3382	4.1856	4.1357	3.9761	3.8086	3.6301
781	4.6693	4.3387	4.1859	4.1360	3.9765	3.8089	3.6305
782	4.6692	4.3390	4.1862	4.1363	3.9768	3.8093	3.6309
783	4.6699	4.3391	4.1867	4.1367	3.9771	3.8096	3.6312
784	4.6704	4.3394	4.1870	4.1370	3.9775	3.8099	3.6316
785	4.6702	4.3398	4.1874	4.1372	3.9778	3.8102	3.6319
786	4.6707	4.3399	4.1876	4.1374	3.9780	3.8105	3.6322
787	4.6711	4.3405	4.1880	4.1377	3.9783	3.8109	3.6326
788	4.6712	4.3406	4.1882	4.1380	3.9787	3.8112	3.6329
789	4.6713	4.3410	4.1885	4.1384	3.9789	3.8116	3.6332
790	4.6715	4.3414	4.1887	4.1387	3.9792	3.8118	3.6335
791	4.6720	4.3413	4.1891	4.1390	3.9796	3.8122	3.6339
792	4.6723	4.3415	4.1892	4.1392	3.9799	3.8125	3.6343
793	4.6724	4.3418	4.1895	4.1396	3.9801	3.8128	3.6345
794	4.6727	4.3422	4.1898	4.1399	3.9804	3.8132	3.6349
795	4.6728	4.3425	4.1901	4.1403	3.9807	3.8135	3.6353
796	4.6733	4.3429	4.1905	4.1407	3.9810	3.8138	3.6356
797	4.6735	4.3432	4.1908	4.1410	3.9814	3.8141	3.6359
798	4.6738	4.3434	4.1910	4.1412	3.9817	3.8144	3.6362
799	4.6740	4.3438	4.1914	4.1415	3.9820	3.8148	3.6366
800	4.6741	4.3440	4.1916	4.1417	3.9822	3.8151	3.6369
801	4.6741	4.3444	4.1920	4.1420	3.9826	3.8154	3.6372
802	4.6744	4.3446	4.1923	4.1423	3.9829	3.8157	3.6376
803	4.6749	4.3449	4.1927	4.1425	3.9832	3.8161	3.6379
804	4.6755	4.3453	4.1930	4.1428	3.9835	3.8163	3.6382
805	4.6761	4.3455	4.1932	4.1432	3.9838	3.8167	3.6385
806	4.6761	4.3459	4.1935	4.1433	3.9841	3.8170	3.6389
807	4.6766	4.3461	4.1938	4.1438	3.9844	3.8173	3.6392
808	4.6765	4.3464	4.1940	4.1439	3.9847	3.8176	3.6395

N	0.1%	0.5%	1%	1.25%	2.5%	5%	10%
809	4.6769	4.3466	4.1943	4.1443	3.9850	3.8179	3.6399
810	4.6772	4.3469	4.1946	4.1446	3.9853	3.8183	3.6402
811	4.6775	4.3473	4.1949	4.1449	3.9857	3.8186	3.6406
812	4.6775	4.3476	4.1952	4.1452	3.9861	3.8189	3.6409
813	4.6778	4.3478	4.1956	4.1456	3.9864	3.8192	3.6412
814	4.6780	4.3480	4.1960	4.1459	3.9868	3.8195	3.6416
815	4.6783	4.3482	4.1963	4.1463	3.9871	3.8198	3.6419
816	4.6786	4.3485	4.1966	4.1466	3.9874	3.8202	3.6422
817	4.6786	4.3487	4.1968	4.1468	3.9878	3.8205	3.6426
818	4.6785	4.3491	4.1971	4.1473	3.9882	3.8208	3.6429
819	4.6792	4.3491	4.1973	4.1475	3.9884	3.8211	3.6433
820	4.6796	4.3495	4.1977	4.1479	3.9887	3.8214	3.6436
821	4.6792	4.3499	4.1979	4.1481	3.9890	3.8217	3.6439
822	4.6796	4.3501	4.1983	4.1484	3.9894	3.8221	3.6443
823	4.6798	4.3505	4.1985	4.1488	3.9897	3.8224	3.6446
824	4.6802	4.3507	4.1987	4.1492	3.9900	3.8227	3.6449
825	4.6804	4.3510	4.1989	4.1493	3.9902	3.8230	3.6453
826	4.6809	4.3515	4.1993	4.1497	3.9905	3.8234	3.6456
827	4.6814	4.3518	4.1997	4.1500	3.9909	3.8236	3.6459
828	4.6814	4.3520	4.2001	4.1504	3.9911	3.8239	3.6462
829	4.6818	4.3523	4.2005	4.1507	3.9914	3.8242	3.6465
830	4.6822	4.3525	4.2008	4.1509	3.9918	3.8246	3.6469
831	4.6826	4.3529	4.2010	4.1512	3.9920	3.8249	3.6472
832	4.6829	4.3531	4.2015	4.1514	3.9923	3.8253	3.6475
833	4.6835	4.3533	4.2017	4.1518	3.9927	3.8256	3.6478
834	4.6839	4.3537	4.2018	4.1520	3.9929	3.8259	3.6481
835	4.6839	4.3541	4.2021	4.1522	3.9933	3.8261	3.6484
836	4.6842	4.3544	4.2024	4.1525	3.9936	3.8264	3.6487
837	4.6845	4.3548	4.2027	4.1528	3.9939	3.8268	3.6490
838	4.6847	4.3550	4.2031	4.1531	3.9942	3.8271	3.6494
839	4.6850	4.3553	4.2034	4.1534	3.9944	3.8274	3.6496
840	4.6853	4.3556	4.2036	4.1538	3.9946	3.8277	3.6500
841	4.6856	4.3558	4.2039	4.1542	3.9950	3.8280	3.6503
842	4.6858	4.3561	4.2042	4.1544	3.9953	3.8283	3.6506
843	4.6862	4.3564	4.2045	4.1548	3.9957	3.8287	3.6509
844	4.6864	4.3566	4.2048	4.1551	3.9960	3.8290	3.6513
845	4.6866	4.3568	4.2050	4.1555	3.9963	3.8293	3.6516
846	4.6867	4.3572	4.2054	4.1558	3.9965	3.8296	3.6519
847	4.6874	4.3574	4.2059	4.1561	3.9968	3.8299	3.6522
848	4.6873	4.3577	4.2060	4.1564	3.9971	3.8302	3.6525
849	4.6875	4.3578	4.2062	4.1568	3.9973	3.8306	3.6528
850	4.6885	4.3581	4.2066	4.1569	3.9976	3.8308	3.6531
851	4.6887	4.3584	4.2069	4.1573	3.9979	3.8311	3.6535
852	4.6889	4.3585	4.2072	4.1575	3.9981	3.8314	3.6538
853	4.6890	4.3587	4.2074	4.1578	3.9985	3.8317	3.6541
854	4.6890	4.3590	4.2077	4.1581	3.9986	3.8320	3.6544
855	4.6893	4.3591	4.2080	4.1583	3.9990	3.8323	3.6547
856	4.6894	4.3594	4.2082	4.1588	3.9992	3.8326	3.6550
857	4.6897	4.3598	4.2085	4.1590	3.9995	3.8329	3.6553
858	4.6904	4.3602	4.2088	4.1593	3.9998	3.8332	3.6556
859	4.6907	4.3605	4.2090	4.1596	4.0000	3.8334	3.6559
860	4.6911	4.3606	4.2092	4.1598	4.0003	3.8338	3.6561
861	4.6912	4.3610	4.2095	4.1601	4.0005	3.8341	3.6565
862	4.6913	4.3614	4.2098	4.1603	4.0008	3.8344	3.6568
863	4.6916	4.3615	4.2100	4.1606	4.0012	3.8346	3.6571
864	4.6914	4.3619	4.2102	4.1608	4.0014	3.8349	3.6574
865	4.6916	4.3620	4.2106	4.1610	4.0018	3.8352	3.6577
866	4.6922	4.3623	4.2109	4.1613	4.0021	3.8355	3.6580
867	4.6924	4.3625	4.2112	4.1616	4.0024	3.8358	3.6583
868	4.6928	4.3627	4.2114	4.1619	4.0027	3.8361	3.6587
869	4.6931	4.3629	4.2116	4.1622	4.0030	3.8364	3.6590
870	4.6931	4.3633	4.2120	4.1625	4.0032	3.8367	3.6593
871	4.6932	4.3636	4.2122	4.1627	4.0035	3.8370	3.6596
872	4.6937	4.3639	4.2126	4.1630	4.0038	3.8373	3.6599
873	4.6939	4.3641	4.2129	4.1632	4.0041	3.8376	3.6602
874	4.6939	4.3643	4.2130	4.1636	4.0044	3.8379	3.6605
875	4.6941	4.3646	4.2133	4.1638	4.0046	3.8382	3.6608
876	4.6949	4.3648	4.2138	4.1640	4.0049	3.8385	3.6611
877	4.6953	4.3652	4.2141	4.1643	4.0052	3.8388	3.6613
878	4.6953	4.3656	4.2144	4.1645	4.0055	3.8391	3.6617
879	4.6958	4.3658	4.2146	4.1648	4.0057	3.8393	3.6620
880	4.6959	4.3660	4.2150	4.1651	4.0060	3.8396	3.6623
881	4.6965	4.3664	4.2152	4.1653	4.0062	3.8399	3.6625
882	4.6964	4.3665	4.2155	4.1656	4.0065	3.8402	3.6628

N	0.1%	0.5%	1%	1.25%	2.5%	5%	10%
883	4.6964	4.3667	4.2157	4.1661	4.0068	3.8405	3.6631
884	4.6965	4.3669	4.2160	4.1663	4.0071	3.8408	3.6634
885	4.6969	4.3674	4.2164	4.1666	4.0074	3.8411	3.6637
886	4.6973	4.3676	4.2167	4.1668	4.0077	3.8414	3.6640
887	4.6973	4.3680	4.2169	4.1671	4.0080	3.8417	3.6643
888	4.6975	4.3683	4.2172	4.1674	4.0083	3.8420	3.6646
889	4.6981	4.3686	4.2175	4.1676	4.0085	3.8423	3.6649
890	4.6983	4.3687	4.2176	4.1679	4.0088	3.8426	3.6652
891	4.6990	4.3689	4.2178	4.1683	4.0091	3.8429	3.6655
892	4.6987	4.3693	4.2180	4.1685	4.0094	3.8432	3.6657
893	4.6989	4.3696	4.2183	4.1687	4.0096	3.8435	3.6660
894	4.6992	4.3700	4.2186	4.1689	4.0099	3.8438	3.6663
895	4.6993	4.3703	4.2189	4.1693	4.0101	3.8441	3.6667
896	4.6992	4.3705	4.2192	4.1695	4.0105	3.8444	3.6669
897	4.6999	4.3707	4.2195	4.1699	4.0107	3.8446	3.6673
898	4.7000	4.3708	4.2197	4.1701	4.0109	3.8449	3.6675
899	4.7001	4.3710	4.2199	4.1703	4.0112	3.8452	3.6678
900	4.7004	4.3714	4.2200	4.1706	4.0115	3.8455	3.6681
901	4.7008	4.3717	4.2203	4.1709	4.0117	3.8458	3.6684
902	4.7014	4.3719	4.2205	4.1713	4.0120	3.8460	3.6687
903	4.7015	4.3721	4.2209	4.1715	4.0123	3.8463	3.6690
904	4.7021	4.3723	4.2211	4.1718	4.0126	3.8465	3.6692
905	4.7021	4.3726	4.2214	4.1721	4.0128	3.8468	3.6695
906	4.7025	4.3728	4.2216	4.1723	4.0131	3.8470	3.6698
907	4.7027	4.3733	4.2219	4.1727	4.0134	3.8474	3.6701
908	4.7029	4.3735	4.2222	4.1729	4.0136	3.8477	3.6704
909	4.7030	4.3738	4.2225	4.1732	4.0138	3.8479	3.6707
910	4.7033	4.3740	4.2228	4.1734	4.0141	3.8482	3.6710
911	4.7035	4.3743	4.2230	4.1736	4.0145	3.8485	3.6713
912	4.7041	4.3745	4.2233	4.1739	4.0148	3.8488	3.6716
913	4.7043	4.3749	4.2236	4.1741	4.0150	3.8491	3.6719
914	4.7046	4.3753	4.2239	4.1743	4.0153	3.8493	3.6722
915	4.7049	4.3756	4.2241	4.1746	4.0156	3.8496	3.6725
916	4.7049	4.3757	4.2244	4.1750	4.0159	3.8499	3.6728
917	4.7054	4.3761	4.2247	4.1751	4.0161	3.8502	3.6730
918	4.7054	4.3763	4.2249	4.1753	4.0163	3.8505	3.6733
919	4.7054	4.3766	4.2251	4.1756	4.0165	3.8507	3.6737
920	4.7057	4.3770	4.2255	4.1759	4.0169	3.8510	3.6739
921	4.7061	4.3772	4.2257	4.1762	4.0171	3.8513	3.6742
922	4.7063	4.3775	4.2261	4.1765	4.0174	3.8515	3.6745
923	4.7068	4.3776	4.2264	4.1768	4.0177	3.8518	3.6748
924	4.7068	4.3779	4.2265	4.1771	4.0180	3.8521	3.6751
925	4.7069	4.3780	4.2269	4.1773	4.0183	3.8525	3.6754
926	4.7076	4.3784	4.2272	4.1777	4.0186	3.8527	3.6757
927	4.7079	4.3786	4.2273	4.1779	4.0189	3.8530	3.6760
928	4.7081	4.3788	4.2275	4.1781	4.0192	3.8533	3.6762
929	4.7081	4.3791	4.2278	4.1784	4.0195	3.8535	3.6766
930	4.7085	4.3795	4.2281	4.1786	4.0197	3.8538	3.6768
931	4.7089	4.3795	4.2284	4.1789	4.0199	3.8540	3.6771
932	4.7090	4.3799	4.2286	4.1792	4.0201	3.8543	3.6774
933	4.7089	4.3800	4.2288	4.1794	4.0204	3.8546	3.6777
934	4.7089	4.3803	4.2291	4.1796	4.0207	3.8549	3.6780
935	4.7096	4.3805	4.2293	4.1799	4.0209	3.8552	3.6783
936	4.7099	4.3807	4.2296	4.1802	4.0212	3.8554	3.6786
937	4.7104	4.3808	4.2300	4.1805	4.0215	3.8557	3.6788
938	4.7109	4.3813	4.2302	4.1807	4.0217	3.8560	3.6791
939	4.7107	4.3815	4.2305	4.1809	4.0219	3.8563	3.6794
940	4.7106	4.3817	4.2308	4.1811	4.0221	3.8566	3.6796
941	4.7107	4.3820	4.2310	4.1814	4.0225	3.8569	3.6799
942	4.7109	4.3821	4.2310	4.1816	4.0228	3.8571	3.6802
943	4.7112	4.3824	4.2313	4.1819	4.0230	3.8573	3.6805
944	4.7114	4.3825	4.2316	4.1822	4.0232	3.8576	3.6808
945	4.7114	4.3826	4.2318	4.1824	4.0234	3.8579	3.6810
946	4.7120	4.3830	4.2321	4.1827	4.0237	3.8582	3.6813
947	4.7121	4.3834	4.2325	4.1830	4.0240	3.8585	3.6816
948	4.7124	4.3838	4.2327	4.1831	4.0243	3.8587	3.6819
949	4.7128	4.3839	4.2329	4.1834	4.0245	3.8590	3.6822
950	4.7131	4.3843	4.2331	4.1837	4.0247	3.8592	3.6824
951	4.7132	4.3846	4.2333	4.1839	4.0250	3.8595	3.6827
952	4.7133	4.3847	4.2336	4.1841	4.0253	3.8598	3.6829
953	4.7137	4.3848	4.2340	4.1844	4.0255	3.8600	3.6832
954	4.7138	4.3849	4.2342	4.1847	4.0257	3.8602	3.6835
955	4.7141	4.3851	4.2345	4.1849	4.0260	3.8604	3.6838
956	4.7143	4.3855	4.2348	4.1852	4.0263	3.8607	3.6841

N	0.1%	0.5%	1%	1.25%	2.5%	5%	10%
957	4.7145	4.3856	4.2349	4.1855	4.0266	3.8610	3.6843
958	4.7145	4.3861	4.2352	4.1858	4.0268	3.8613	3.6846
959	4.7147	4.3864	4.2355	4.1861	4.0271	3.8616	3.6849
960	4.7150	4.3865	4.2357	4.1863	4.0274	3.8619	3.6851
961	4.7153	4.3870	4.2360	4.1865	4.0277	3.8622	3.6854
962	4.7156	4.3871	4.2363	4.1868	4.0280	3.8624	3.6857
963	4.7159	4.3874	4.2364	4.1872	4.0282	3.8627	3.6859
964	4.7164	4.3877	4.2365	4.1874	4.0285	3.8629	3.6863
965	4.7161	4.3880	4.2369	4.1877	4.0287	3.8632	3.6866
966	4.7166	4.3881	4.2371	4.1880	4.0290	3.8634	3.6868
967	4.7172	4.3885	4.2375	4.1882	4.0293	3.8637	3.6871
968	4.7174	4.3888	4.2377	4.1884	4.0296	3.8640	3.6874
969	4.7177	4.3890	4.2380	4.1888	4.0298	3.8642	3.6877
970	4.7182	4.3893	4.2382	4.1890	4.0300	3.8645	3.6879
971	4.7181	4.3895	4.2385	4.1893	4.0303	3.8647	3.6882
972	4.7186	4.3896	4.2386	4.1895	4.0305	3.8650	3.6885
973	4.7189	4.3899	4.2390	4.1898	4.0308	3.8653	3.6888
974	4.7194	4.3903	4.2393	4.1900	4.0311	3.8655	3.6891
975	4.7196	4.3904	4.2395	4.1902	4.0312	3.8657	3.6893
976	4.7198	4.3907	4.2397	4.1904	4.0315	3.8660	3.6896
977	4.7201	4.3909	4.2400	4.1907	4.0317	3.8663	3.6899
978	4.7201	4.3912	4.2403	4.1909	4.0320	3.8666	3.6901
979	4.7206	4.3913	4.2406	4.1912	4.0323	3.8668	3.6904
980	4.7209	4.3916	4.2409	4.1914	4.0326	3.8671	3.6907
981	4.7211	4.3917	4.2411	4.1917	4.0328	3.8674	3.6909
982	4.7214	4.3920	4.2414	4.1919	4.0330	3.8676	3.6912
983	4.7220	4.3923	4.2416	4.1921	4.0333	3.8679	3.6914
984	4.7221	4.3926	4.2418	4.1924	4.0335	3.8681	3.6917
985	4.7227	4.3926	4.2420	4.1926	4.0338	3.8684	3.6919
986	4.7229	4.3929	4.2423	4.1929	4.0340	3.8686	3.6922
987	4.7231	4.3931	4.2425	4.1931	4.0343	3.8689	3.6924
988	4.7232	4.3934	4.2428	4.1932	4.0346	3.8691	3.6927
989	4.7231	4.3936	4.2429	4.1934	4.0348	3.8695	3.6930
990	4.7233	4.3938	4.2433	4.1936	4.0350	3.8697	3.6933
991	4.7235	4.3939	4.2434	4.1940	4.0353	3.8700	3.6936
992	4.7240	4.3942	4.2438	4.1942	4.0356	3.8702	3.6938
993	4.7240	4.3945	4.2441	4.1945	4.0359	3.8704	3.6941
994	4.7244	4.3947	4.2441	4.1947	4.0361	3.8706	3.6943
995	4.7245	4.3950	4.2444	4.1950	4.0363	3.8709	3.6946
996	4.7248	4.3951	4.2447	4.1952	4.0366	3.8711	3.6948
997	4.7252	4.3953	4.2449	4.1953	4.0367	3.8714	3.6951
998	4.7253	4.3955	4.2451	4.1956	4.0370	3.8716	3.6954
999	4.7256	4.3957	4.2453	4.1957	4.0373	3.8719	3.6956
1000	4.7262	4.3960	4.2456	4.1960	4.0375	3.8721	3.6960
