# Supplementary material for: A generalized outlier method with an automated test protocol applied to proficiency testing schemes: utilizing Monte Carlo simulation for generating critical values for the extended Grubbs statistic
Source: J AOAC Int. 2026 Apr 29;109(6):974–84. doi: 10.1093/jaoacint/qsag036 (PMC13732683; doi:10.1093/jaoacint/qsag036)
Supplement: Supplementary file 1 [file qsag036_supplementary_data.zip › aoac-25-0307-File014.pdf]

Extended Grubbs Statistics Tables for Double Outliers

Table 1 – *GD(HH or LL)*. Critical values of upper significance levels for the Grubbs double outlier test, applicable in situations where the two highest or two lowest observations are simultaneously identified as outliers, denoted as *GD(HH)* and *GD(LL)*, respectively.

N	0.1%	0.5%	1%	1.25%	2.5%	5%	10%
4	0.00000	0.00001	0.00003	0.00005	0.00019	0.00076	0.00311
5	0.00035	0.00176	0.00354	0.00444	0.00898	0.01829	0.03762
6	0.00390	0.01159	0.01859	0.02165	0.03488	0.05644	0.09206
7	0.01354	0.03080	0.04401	0.04939	0.07084	0.10204	0.14790
8	0.02908	0.05632	0.07506	0.08237	0.11013	0.14778	0.19940
9	0.04896	0.08510	0.10823	0.11698	0.14920	0.19089	0.24545
10	0.07151	0.11500	0.14145	0.15124	0.18645	0.23056	0.28635
11	0.09544	0.14485	0.17366	0.18417	0.22133	0.26671	0.32272
12	0.11989	0.17384	0.20436	0.21533	0.25366	0.29959	0.35518
13	0.14426	0.20166	0.23331	0.24459	0.28356	0.32952	0.38433
14	0.16815	0.22811	0.26050	0.27196	0.31118	0.35685	0.41062
15	0.19136	0.25315	0.28595	0.29748	0.33668	0.38183	0.43448
16	0.21369	0.27675	0.30979	0.32131	0.36028	0.40478	0.45622
17	0.23509	0.29902	0.33208	0.34355	0.38216	0.42592	0.47612
18	0.25562	0.32000	0.35294	0.36434	0.40249	0.44544	0.49443
19	0.27518	0.33977	0.37254	0.38381	0.42143	0.46355	0.51131
20	0.29386	0.35843	0.39090	0.40206	0.43910	0.48038	0.52695
21	0.31167	0.37609	0.40819	0.41918	0.45563	0.49605	0.54148
22	0.32868	0.39273	0.42446	0.43530	0.47112	0.51070	0.55502
23	0.34489	0.40848	0.43982	0.45049	0.48568	0.52443	0.56767
24	0.36034	0.42341	0.45432	0.46482	0.49938	0.53733	0.57952
25	0.37508	0.43755	0.46802	0.47835	0.51230	0.54945	0.59064
26	0.38918	0.45099	0.48101	0.49118	0.52451	0.56088	0.60112
27	0.40260	0.46377	0.49333	0.50333	0.53605	0.57168	0.61099
28	0.41546	0.47592	0.50504	0.51488	0.54698	0.58189	0.62032
29	0.42779	0.48750	0.51617	0.52584	0.55737	0.59157	0.62915
30	0.43953	0.49854	0.52677	0.53628	0.56723	0.60076	0.63753
31	0.45083	0.50908	0.53688	0.54622	0.57663	0.60949	0.64548
32	0.46168	0.51916	0.54652	0.55571	0.58558	0.61781	0.65304
33	0.47209	0.52881	0.55574	0.56477	0.59412	0.62573	0.66025
34	0.48206	0.53806	0.56456	0.57344	0.60227	0.63330	0.66712
35	0.49168	0.54692	0.57300	0.58175	0.61007	0.64053	0.67368
36	0.50092	0.55541	0.58110	0.58970	0.61755	0.64743	0.67995
37	0.50981	0.56356	0.58885	0.59731	0.62470	0.65406	0.68595
38	0.51836	0.57140	0.59631	0.60463	0.63157	0.66041	0.69171
39	0.52661	0.57894	0.60347	0.61167	0.63816	0.66650	0.69723
40	0.53455	0.58619	0.61037	0.61843	0.64450	0.67235	0.70252
41	0.54223	0.59318	0.61699	0.62494	0.65060	0.67798	0.70762
42	0.54964	0.59991	0.62339	0.63122	0.65646	0.68340	0.71251
43	0.55681	0.60641	0.62955	0.63725	0.66212	0.68861	0.71723
44	0.56372	0.61270	0.63550	0.64308	0.66757	0.69363	0.72177
45	0.57038	0.61876	0.64123	0.64872	0.67283	0.69848	0.72615
46	0.57687	0.62462	0.64679	0.65416	0.67791	0.70315	0.73037
47	0.58315	0.63029	0.65215	0.65941	0.68281	0.70767	0.73446
48	0.58921	0.63577	0.65734	0.66450	0.68756	0.71203	0.73840
49	0.59508	0.64109	0.66236	0.66942	0.69214	0.71626	0.74221
50	0.60076	0.64622	0.66722	0.67418	0.69658	0.72034	0.74590
51	0.60632	0.65122	0.67192	0.67880	0.70088	0.72430	0.74948
52	0.61171	0.65606	0.67649	0.68327	0.70506	0.72814	0.75294
53	0.61695	0.66075	0.68092	0.68761	0.70909	0.73185	0.75629
54	0.62202	0.66531	0.68522	0.69182	0.71302	0.73546	0.75954
55	0.62694	0.66973	0.68939	0.69591	0.71682	0.73896	0.76270
56	0.63175	0.67403	0.69344	0.69988	0.72052	0.74235	0.76576
57	0.63642	0.67822	0.69739	0.70374	0.72411	0.74565	0.76874
58	0.64097	0.68228	0.70122	0.70749	0.72760	0.74886	0.77163
59	0.64539	0.68623	0.70494	0.71114	0.73100	0.75197	0.77444
60	0.64969	0.69008	0.70857	0.71469	0.73430	0.75501	0.77718
61	0.65390	0.69382	0.71209	0.71814	0.73751	0.75796	0.77984
62	0.65800	0.69747	0.71553	0.72150	0.74064	0.76083	0.78244
63	0.66198	0.70103	0.71888	0.72478	0.74369	0.76363	0.78496
64	0.66588	0.70450	0.72214	0.72797	0.74666	0.76635	0.78742
65	0.66968	0.70788	0.72533	0.73109	0.74955	0.76901	0.78981
66	0.67339	0.71117	0.72843	0.73413	0.75238	0.77160	0.79215
67	0.67701	0.71439	0.73146	0.73709	0.75513	0.77413	0.79443
68	0.68053	0.71754	0.73441	0.73998	0.75782	0.77659	0.79665
69	0.68398	0.72061	0.73729	0.74281	0.76044	0.77900	0.79882

N	0.1%	0.5%	1%	1.25%	2.5%	5%	10%
70	0.68736	0.72360	0.74011	0.74556	0.76300	0.78134	0.80094
71	0.69067	0.72653	0.74286	0.74825	0.76550	0.78364	0.80301
72	0.69388	0.72939	0.74555	0.75089	0.76794	0.78588	0.80503
73	0.69703	0.73218	0.74818	0.75346	0.77033	0.78807	0.80700
74	0.70010	0.73491	0.75075	0.75597	0.77266	0.79021	0.80893
75	0.70311	0.73759	0.75326	0.75843	0.77494	0.79230	0.81082
76	0.70607	0.74020	0.75572	0.76083	0.77718	0.79435	0.81267
77	0.70896	0.74276	0.75812	0.76319	0.77936	0.79635	0.81447
78	0.71179	0.74527	0.76048	0.76549	0.78150	0.79832	0.81624
79	0.71457	0.74773	0.76278	0.76774	0.78359	0.80024	0.81797
80	0.71729	0.75013	0.76504	0.76995	0.78564	0.80212	0.81967
81	0.71994	0.75248	0.76724	0.77211	0.78765	0.80396	0.82133
82	0.72255	0.75479	0.76941	0.77423	0.78961	0.80576	0.82296
83	0.72511	0.75705	0.77153	0.77631	0.79154	0.80753	0.82455
84	0.72761	0.75926	0.77361	0.77835	0.79343	0.80926	0.82611
85	0.73006	0.76144	0.77565	0.78034	0.79528	0.81095	0.82764
86	0.73247	0.76357	0.77765	0.78229	0.79709	0.81262	0.82914
87	0.73484	0.76566	0.77961	0.78421	0.79887	0.81425	0.83061
88	0.73714	0.76771	0.78153	0.78609	0.80062	0.81585	0.83206
89	0.73942	0.76972	0.78342	0.78794	0.80233	0.81742	0.83347
90	0.74165	0.77169	0.78527	0.78975	0.80401	0.81896	0.83486
91	0.74384	0.77363	0.78709	0.79153	0.80566	0.82048	0.83623
92	0.74601	0.77553	0.78887	0.79327	0.80728	0.82196	0.83757
93	0.74812	0.77740	0.79063	0.79499	0.80887	0.82342	0.83888
94	0.75020	0.77923	0.79235	0.79667	0.81043	0.82485	0.84018
95	0.75224	0.78104	0.79404	0.79832	0.81197	0.82626	0.84144
96	0.75424	0.78280	0.79570	0.79995	0.81348	0.82764	0.84269
97	0.75621	0.78455	0.79733	0.80154	0.81495	0.82900	0.84392
98	0.75815	0.78626	0.79894	0.80311	0.81641	0.83033	0.84512
99	0.76005	0.78794	0.80051	0.80465	0.81784	0.83164	0.84631
100	0.76194	0.78959	0.80206	0.80617	0.81925	0.83293	0.84747
101	0.76378	0.79122	0.80359	0.80766	0.82063	0.83420	0.84861
102	0.76559	0.79281	0.80508	0.80912	0.82199	0.83545	0.84974
103	0.76737	0.79438	0.80656	0.81056	0.82332	0.83667	0.85084
104	0.76913	0.79592	0.80801	0.81198	0.82464	0.83788	0.85193
105	0.77085	0.79744	0.80943	0.81337	0.82593	0.83906	0.85301
106	0.77255	0.79894	0.81083	0.81475	0.82720	0.84023	0.85406
107	0.77421	0.80041	0.81222	0.81610	0.82846	0.84138	0.85510
108	0.77586	0.80186	0.81358	0.81743	0.82969	0.84251	0.85612
109	0.77748	0.80329	0.81491	0.81873	0.83090	0.84362	0.85712
110	0.77907	0.80469	0.81623	0.82002	0.83210	0.84472	0.85811
111	0.78064	0.80607	0.81752	0.82129	0.83327	0.84580	0.85909
112	0.78218	0.80744	0.81880	0.82254	0.83443	0.84686	0.86005
113	0.78371	0.80878	0.82006	0.82377	0.83557	0.84791	0.86099
114	0.78522	0.81010	0.82130	0.82498	0.83670	0.84894	0.86192
115	0.78669	0.81140	0.82252	0.82617	0.83781	0.84995	0.86284
116	0.78815	0.81269	0.82373	0.82735	0.83890	0.85096	0.86375
117	0.78959	0.81395	0.82491	0.82851	0.83997	0.85194	0.86464
118	0.79101	0.81520	0.82608	0.82965	0.84103	0.85291	0.86552
119	0.79240	0.81643	0.82723	0.83078	0.84207	0.85387	0.86638
120	0.79378	0.81764	0.82836	0.83189	0.84310	0.85482	0.86724
121	0.79513	0.81883	0.82948	0.83298	0.84412	0.85575	0.86808
122	0.79647	0.82001	0.83058	0.83406	0.84512	0.85667	0.86891
123	0.79779	0.82117	0.83167	0.83512	0.84610	0.85757	0.86973
124	0.79909	0.82231	0.83274	0.83617	0.84708	0.85846	0.87053
125	0.80037	0.82344	0.83380	0.83720	0.84804	0.85934	0.87133
126	0.80164	0.82455	0.83484	0.83823	0.84898	0.86021	0.87211
127	0.80289	0.82565	0.83587	0.83923	0.84992	0.86107	0.87289
128	0.80412	0.82673	0.83689	0.84022	0.85084	0.86192	0.87365
129	0.80533	0.82780	0.83789	0.84121	0.85175	0.86275	0.87441
130	0.80653	0.82885	0.83888	0.84217	0.85264	0.86357	0.87515
131	0.80772	0.82989	0.83986	0.84313	0.85353	0.86439	0.87589
132	0.80889	0.83092	0.84082	0.84407	0.85440	0.86519	0.87661
133	0.81004	0.83194	0.84177	0.84500	0.85526	0.86598	0.87733
134	0.81117	0.83294	0.84271	0.84591	0.85612	0.86676	0.87803
135	0.81230	0.83393	0.84363	0.84682	0.85696	0.86753	0.87873
136	0.81340	0.83491	0.84455	0.84771	0.85779	0.86829	0.87942
137	0.81450	0.83587	0.84545	0.84860	0.85861	0.86904	0.88010
138	0.81559	0.83682	0.84634	0.84947	0.85941	0.86979	0.88077
139	0.81665	0.83776	0.84722	0.85033	0.86021	0.87052	0.88144
140	0.81771	0.83868	0.84809	0.85118	0.86100	0.87125	0.88209
141	0.81875	0.83960	0.84895	0.85202	0.86178	0.87196	0.88274
142	0.81978	0.84051	0.84980	0.85285	0.86255	0.87267	0.88338
143	0.82079	0.84140	0.85064	0.85367	0.86331	0.87337	0.88401

N	0.1%	0.5%	1%	1.25%	2.5%	5%	10%
144	0.82180	0.84228	0.85147	0.85448	0.86407	0.87406	0.88464
145	0.82279	0.84316	0.85229	0.85528	0.86481	0.87474	0.88525
146	0.82378	0.84402	0.85310	0.85607	0.86554	0.87541	0.88586
147	0.82474	0.84487	0.85389	0.85685	0.86627	0.87608	0.88647
148	0.82571	0.84572	0.85468	0.85763	0.86698	0.87674	0.88706
149	0.82665	0.84655	0.85546	0.85839	0.86769	0.87739	0.88765
150	0.82759	0.84737	0.85623	0.85915	0.86839	0.87803	0.88823
151	0.82852	0.84818	0.85700	0.85989	0.86908	0.87866	0.88881
152	0.82943	0.84899	0.85775	0.86063	0.86977	0.87929	0.88938
153	0.83033	0.84979	0.85850	0.86136	0.87044	0.87992	0.88994
154	0.83123	0.85057	0.85923	0.86208	0.87111	0.88053	0.89049
155	0.83211	0.85135	0.85996	0.86279	0.87177	0.88114	0.89105
156	0.83299	0.85212	0.86068	0.86350	0.87243	0.88174	0.89159
157	0.83385	0.85288	0.86139	0.86419	0.87308	0.88233	0.89213
158	0.83471	0.85363	0.86210	0.86488	0.87372	0.88292	0.89266
159	0.83556	0.85437	0.86280	0.86556	0.87435	0.88350	0.89319
160	0.83639	0.85511	0.86349	0.86624	0.87497	0.88408	0.89371
161	0.83722	0.85583	0.86417	0.86690	0.87559	0.88465	0.89422
162	0.83803	0.85656	0.86484	0.86756	0.87620	0.88521	0.89473
163	0.83884	0.85727	0.86551	0.86822	0.87681	0.88576	0.89524
164	0.83964	0.85797	0.86617	0.86886	0.87741	0.88632	0.89574
165	0.84044	0.85867	0.86682	0.86950	0.87800	0.88686	0.89623
166	0.84122	0.85936	0.86747	0.87013	0.87859	0.88740	0.89672
167	0.84199	0.86004	0.86811	0.87076	0.87917	0.88793	0.89720
168	0.84276	0.86072	0.86874	0.87138	0.87975	0.88846	0.89768
169	0.84352	0.86138	0.86937	0.87199	0.88031	0.88898	0.89815
170	0.84427	0.86204	0.86999	0.87260	0.88088	0.88950	0.89862
171	0.84501	0.86270	0.87061	0.87320	0.88144	0.89001	0.89909
172	0.84575	0.86335	0.87121	0.87379	0.88199	0.89052	0.89955
173	0.84648	0.86399	0.87181	0.87438	0.88253	0.89102	0.90000
174	0.84721	0.86463	0.87241	0.87496	0.88308	0.89152	0.90045
175	0.84792	0.86525	0.87300	0.87554	0.88361	0.89201	0.90090
176	0.84863	0.86588	0.87358	0.87611	0.88414	0.89250	0.90134
177	0.84933	0.86649	0.87416	0.87668	0.88467	0.89298	0.90178
178	0.85002	0.86710	0.87473	0.87724	0.88518	0.89346	0.90221
179	0.85071	0.86771	0.87530	0.87779	0.88570	0.89393	0.90264
180	0.85139	0.86831	0.87586	0.87834	0.88621	0.89440	0.90306
181	0.85207	0.86890	0.87641	0.87888	0.88671	0.89486	0.90348
182	0.85273	0.86949	0.87696	0.87942	0.88721	0.89532	0.90390
183	0.85339	0.87007	0.87751	0.87995	0.88771	0.89578	0.90431
184	0.85404	0.87064	0.87805	0.88048	0.88820	0.89623	0.90472
185	0.85469	0.87121	0.87858	0.88100	0.88868	0.89668	0.90513
186	0.85534	0.87178	0.87911	0.88152	0.88916	0.89712	0.90553
187	0.85597	0.87234	0.87964	0.88203	0.88964	0.89756	0.90593
188	0.85661	0.87289	0.88016	0.88254	0.89011	0.89799	0.90632
189	0.85722	0.87344	0.88067	0.88304	0.89058	0.89842	0.90671
190	0.85784	0.87398	0.88118	0.88355	0.89104	0.89885	0.90710
191	0.85846	0.87452	0.88169	0.88404	0.89150	0.89927	0.90748
192	0.85907	0.87506	0.88219	0.88453	0.89196	0.89969	0.90786
193	0.85967	0.87558	0.88269	0.88502	0.89241	0.90010	0.90824
194	0.86026	0.87611	0.88318	0.88550	0.89286	0.90051	0.90861
195	0.86085	0.87663	0.88367	0.88597	0.89330	0.90092	0.90898
196	0.86144	0.87714	0.88415	0.88645	0.89374	0.90132	0.90935
197	0.86202	0.87765	0.88463	0.88692	0.89417	0.90173	0.90971
198	0.86260	0.87816	0.88510	0.88738	0.89460	0.90212	0.91007
199	0.86317	0.87866	0.88557	0.88784	0.89503	0.90251	0.91043
200	0.86373	0.87916	0.88604	0.88829	0.89545	0.90290	0.91078
201	0.86421	0.87962	0.88643	0.88871	0.89585	0.90328	0.91113
202	0.86474	0.88010	0.88689	0.88915	0.89627	0.90366	0.91147
203	0.86535	0.88059	0.88735	0.88960	0.89668	0.90404	0.91182
204	0.86589	0.88108	0.88781	0.89005	0.89709	0.90442	0.91216
205	0.86649	0.88154	0.88825	0.89048	0.89750	0.90479	0.91250
206	0.86703	0.88202	0.88870	0.89090	0.89790	0.90517	0.91284
207	0.86760	0.88249	0.88913	0.89133	0.89830	0.90554	0.91317
208	0.86811	0.88295	0.88957	0.89176	0.89870	0.90590	0.91350
209	0.86865	0.88341	0.89000	0.89218	0.89908	0.90625	0.91383
210	0.86917	0.88385	0.89043	0.89260	0.89948	0.90662	0.91415
211	0.86969	0.88431	0.89086	0.89302	0.89986	0.90697	0.91447
212	0.87017	0.88476	0.89127	0.89343	0.90025	0.90732	0.91479
213	0.87069	0.88522	0.89168	0.89383	0.90062	0.90767	0.91511
214	0.87123	0.88565	0.89208	0.89423	0.90100	0.90801	0.91542
215	0.87169	0.88608	0.89250	0.89463	0.90137	0.90835	0.91573
216	0.87222	0.88651	0.89292	0.89502	0.90174	0.90869	0.91605
217	0.87266	0.88695	0.89332	0.89542	0.90210	0.90903	0.91635

N	0.1%	0.5%	1%	1.25%	2.5%	5%	10%
218	0.87317	0.88738	0.89372	0.89581	0.90246	0.90937	0.91666
219	0.87365	0.88779	0.89411	0.89619	0.90283	0.90970	0.91695
220	0.87411	0.88822	0.89450	0.89657	0.90319	0.91002	0.91725
221	0.87458	0.88864	0.89490	0.89696	0.90354	0.91035	0.91755
222	0.87507	0.88906	0.89529	0.89735	0.90389	0.91067	0.91784
223	0.87553	0.88949	0.89567	0.89771	0.90424	0.91100	0.91813
224	0.87599	0.88988	0.89605	0.89809	0.90459	0.91132	0.91842
225	0.87646	0.89030	0.89643	0.89846	0.90494	0.91164	0.91872
226	0.87695	0.89068	0.89680	0.89884	0.90528	0.91195	0.91900
227	0.87737	0.89108	0.89718	0.89920	0.90561	0.91226	0.91928
228	0.87785	0.89148	0.89755	0.89957	0.90596	0.91257	0.91956
229	0.87827	0.89186	0.89791	0.89992	0.90629	0.91288	0.91984
230	0.87870	0.89225	0.89828	0.90028	0.90663	0.91319	0.92011
231	0.87916	0.89264	0.89864	0.90064	0.90695	0.91348	0.92039
232	0.87958	0.89302	0.89900	0.90098	0.90727	0.91379	0.92066
233	0.87995	0.89338	0.89936	0.90132	0.90760	0.91408	0.92093
234	0.88039	0.89376	0.89971	0.90168	0.90792	0.91438	0.92120
235	0.88082	0.89413	0.90007	0.90202	0.90824	0.91467	0.92147
236	0.88125	0.89451	0.90041	0.90235	0.90855	0.91496	0.92174
237	0.88165	0.89486	0.90075	0.90269	0.90886	0.91525	0.92200
238	0.88207	0.89522	0.90109	0.90302	0.90917	0.91553	0.92226
239	0.88245	0.89560	0.90143	0.90335	0.90949	0.91582	0.92252
240	0.88285	0.89596	0.90177	0.90368	0.90979	0.91611	0.92277
241	0.88325	0.89631	0.90209	0.90402	0.91010	0.91639	0.92303
242	0.88366	0.89667	0.90242	0.90435	0.91040	0.91667	0.92328
243	0.88404	0.89702	0.90276	0.90468	0.91070	0.91694	0.92353
244	0.88444	0.89738	0.90309	0.90500	0.91100	0.91722	0.92378
245	0.88482	0.89772	0.90341	0.90531	0.91130	0.91749	0.92404
246	0.88523	0.89807	0.90374	0.90562	0.91159	0.91776	0.92428
247	0.88560	0.89841	0.90407	0.90594	0.91188	0.91803	0.92453
248	0.88597	0.89875	0.90439	0.90626	0.91217	0.91830	0.92477
249	0.88637	0.89910	0.90471	0.90658	0.91246	0.91856	0.92501
250	0.88673	0.89943	0.90502	0.90688	0.91275	0.91882	0.92525
251	0.88712	0.89975	0.90534	0.90719	0.91304	0.91909	0.92549
252	0.88749	0.90009	0.90564	0.90749	0.91332	0.91935	0.92572
253	0.88787	0.90041	0.90594	0.90779	0.91360	0.91961	0.92596
254	0.88824	0.90073	0.90625	0.90809	0.91388	0.91986	0.92619
255	0.88858	0.90105	0.90655	0.90838	0.91414	0.92012	0.92642
256	0.88898	0.90137	0.90686	0.90867	0.91442	0.92037	0.92666
257	0.88934	0.90168	0.90715	0.90897	0.91469	0.92062	0.92688
258	0.88969	0.90200	0.90745	0.90925	0.91496	0.92086	0.92711
259	0.89005	0.90231	0.90774	0.90955	0.91523	0.92111	0.92734
260	0.89041	0.90263	0.90804	0.90983	0.91550	0.92136	0.92756
261	0.89077	0.90295	0.90833	0.91012	0.91576	0.92160	0.92778
262	0.89110	0.90326	0.90862	0.91041	0.91602	0.92184	0.92800
263	0.89146	0.90357	0.90891	0.91069	0.91628	0.92209	0.92822
264	0.89179	0.90387	0.90920	0.91096	0.91654	0.92232	0.92843
265	0.89214	0.90416	0.90948	0.91123	0.91679	0.92256	0.92865
266	0.89249	0.90446	0.90976	0.91151	0.91705	0.92280	0.92887
267	0.89282	0.90475	0.91004	0.91178	0.91731	0.92303	0.92908
268	0.89317	0.90505	0.91031	0.91206	0.91756	0.92326	0.92929
269	0.89350	0.90535	0.91059	0.91233	0.91782	0.92349	0.92950
270	0.89383	0.90563	0.91086	0.91260	0.91807	0.92372	0.92971
271	0.89415	0.90592	0.91113	0.91286	0.91831	0.92395	0.92992
272	0.89447	0.90621	0.91140	0.91313	0.91856	0.92418	0.93012
273	0.89478	0.90649	0.91168	0.91339	0.91880	0.92440	0.93033
274	0.89510	0.90679	0.91195	0.91366	0.91905	0.92463	0.93054
275	0.89544	0.90707	0.91221	0.91391	0.91929	0.92485	0.93074
276	0.89575	0.90735	0.91248	0.91417	0.91953	0.92508	0.93094
277	0.89605	0.90763	0.91273	0.91443	0.91977	0.92530	0.93114
278	0.89639	0.90791	0.91299	0.91468	0.92001	0.92551	0.93134
279	0.89669	0.90818	0.91325	0.91492	0.92024	0.92573	0.93154
280	0.89699	0.90846	0.91351	0.91517	0.92048	0.92595	0.93174
281	0.89730	0.90873	0.91378	0.91543	0.92071	0.92616	0.93193
282	0.89761	0.90899	0.91403	0.91567	0.92094	0.92637	0.93213
283	0.89793	0.90927	0.91429	0.91592	0.92117	0.92659	0.93232
284	0.89824	0.90953	0.91453	0.91617	0.92139	0.92679	0.93251
285	0.89854	0.90980	0.91479	0.91641	0.92162	0.92701	0.93270
286	0.89885	0.91007	0.91503	0.91665	0.92185	0.92722	0.93289
287	0.89916	0.91032	0.91528	0.91688	0.92207	0.92742	0.93308
288	0.89944	0.91059	0.91552	0.91712	0.92229	0.92762	0.93327
289	0.89975	0.91084	0.91576	0.91736	0.92251	0.92782	0.93345
290	0.90004	0.91109	0.91601	0.91760	0.92273	0.92803	0.93364
291	0.90031	0.91135	0.91624	0.91784	0.92295	0.92823	0.93382

N	0.1%	0.5%	1%	1.25%	2.5%	5%	10%
292	0.90059	0.91161	0.91648	0.91807	0.92317	0.92843	0.93400
293	0.90088	0.91186	0.91671	0.91830	0.92338	0.92862	0.93418
294	0.90120	0.91211	0.91695	0.91853	0.92359	0.92882	0.93436
295	0.90148	0.91236	0.91718	0.91877	0.92381	0.92902	0.93454
296	0.90178	0.91261	0.91741	0.91899	0.92402	0.92921	0.93472
297	0.90206	0.91286	0.91764	0.91922	0.92424	0.92941	0.93489
298	0.90232	0.91310	0.91787	0.91945	0.92444	0.92960	0.93507
299	0.90260	0.91335	0.91809	0.91967	0.92465	0.92980	0.93524
300	0.90288	0.91358	0.91833	0.91989	0.92486	0.92999	0.93542
301	0.90314	0.91382	0.91855	0.92011	0.92506	0.93017	0.93559
302	0.90344	0.91406	0.91878	0.92034	0.92527	0.93036	0.93576
303	0.90371	0.91429	0.91900	0.92056	0.92547	0.93055	0.93593
304	0.90399	0.91452	0.91922	0.92078	0.92568	0.93074	0.93610
305	0.90425	0.91476	0.91944	0.92100	0.92588	0.93092	0.93627
306	0.90451	0.91499	0.91966	0.92121	0.92608	0.93111	0.93644
307	0.90476	0.91523	0.91989	0.92142	0.92627	0.93129	0.93661
308	0.90503	0.91546	0.92010	0.92163	0.92647	0.93147	0.93677
309	0.90526	0.91567	0.92031	0.92184	0.92667	0.93166	0.93694
310	0.90555	0.91591	0.92053	0.92204	0.92686	0.93184	0.93710
311	0.90577	0.91613	0.92074	0.92225	0.92706	0.93201	0.93726
312	0.90603	0.91637	0.92095	0.92246	0.92725	0.93219	0.93742
313	0.90630	0.91660	0.92116	0.92267	0.92744	0.93237	0.93759
314	0.90655	0.91682	0.92138	0.92287	0.92764	0.93255	0.93775
315	0.90680	0.91705	0.92159	0.92307	0.92783	0.93272	0.93791
316	0.90705	0.91727	0.92180	0.92328	0.92801	0.93289	0.93806
317	0.90728	0.91750	0.92200	0.92348	0.92820	0.93307	0.93823
318	0.90754	0.91770	0.92221	0.92368	0.92838	0.93324	0.93838
319	0.90777	0.91792	0.92240	0.92388	0.92857	0.93341	0.93854
320	0.90805	0.91813	0.92260	0.92408	0.92875	0.93358	0.93869
321	0.90829	0.91836	0.92281	0.92427	0.92894	0.93375	0.93885
322	0.90854	0.91857	0.92300	0.92447	0.92912	0.93392	0.93900
323	0.90877	0.91878	0.92320	0.92467	0.92930	0.93409	0.93916
324	0.90901	0.91899	0.92340	0.92486	0.92948	0.93425	0.93931
325	0.90924	0.91920	0.92359	0.92505	0.92965	0.93441	0.93946
326	0.90951	0.91941	0.92378	0.92524	0.92983	0.93458	0.93961
327	0.90974	0.91960	0.92398	0.92543	0.93001	0.93475	0.93976
328	0.90996	0.91981	0.92418	0.92562	0.93018	0.93491	0.93991
329	0.91019	0.92001	0.92437	0.92581	0.93036	0.93507	0.94005
330	0.91042	0.92023	0.92457	0.92599	0.93053	0.93523	0.94020
331	0.91065	0.92042	0.92475	0.92617	0.93071	0.93539	0.94035
332	0.91087	0.92063	0.92494	0.92636	0.93088	0.93555	0.94049
333	0.91108	0.92083	0.92513	0.92654	0.93105	0.93571	0.94064
334	0.91133	0.92103	0.92532	0.92673	0.93122	0.93586	0.94078
335	0.91154	0.92122	0.92550	0.92691	0.93139	0.93602	0.94093
336	0.91173	0.92142	0.92568	0.92710	0.93156	0.93618	0.94107
337	0.91196	0.92162	0.92587	0.92727	0.93172	0.93634	0.94121
338	0.91220	0.92182	0.92605	0.92746	0.93189	0.93649	0.94135
339	0.91241	0.92201	0.92623	0.92764	0.93206	0.93664	0.94149
340	0.91264	0.92220	0.92641	0.92780	0.93223	0.93679	0.94163
341	0.91284	0.92240	0.92659	0.92799	0.93239	0.93695	0.94177
342	0.91307	0.92259	0.92678	0.92817	0.93255	0.93710	0.94191
343	0.91329	0.92278	0.92696	0.92834	0.93271	0.93725	0.94205
344	0.91352	0.92297	0.92713	0.92851	0.93287	0.93740	0.94218
345	0.91373	0.92315	0.92731	0.92868	0.93304	0.93755	0.94232
346	0.91393	0.92334	0.92749	0.92886	0.93320	0.93770	0.94245
347	0.91415	0.92353	0.92766	0.92903	0.93336	0.93785	0.94259
348	0.91434	0.92372	0.92783	0.92920	0.93351	0.93800	0.94272
349	0.91455	0.92390	0.92801	0.92937	0.93367	0.93814	0.94285
350	0.91477	0.92409	0.92817	0.92954	0.93383	0.93829	0.94299
351	0.91500	0.92427	0.92835	0.92970	0.93399	0.93843	0.94312
352	0.91518	0.92446	0.92851	0.92987	0.93415	0.93858	0.94325
353	0.91541	0.92464	0.92869	0.93004	0.93430	0.93872	0.94338
354	0.91561	0.92481	0.92886	0.93020	0.93446	0.93886	0.94351
355	0.91581	0.92499	0.92902	0.93037	0.93461	0.93900	0.94364
356	0.91602	0.92517	0.92919	0.93053	0.93476	0.93914	0.94377
357	0.91623	0.92535	0.92935	0.93069	0.93491	0.93928	0.94390
358	0.91643	0.92553	0.92952	0.93085	0.93506	0.93943	0.94402
359	0.91664	0.92571	0.92968	0.93101	0.93521	0.93956	0.94415
360	0.91681	0.92588	0.92985	0.93117	0.93536	0.93970	0.94428
361	0.91701	0.92606	0.93001	0.93133	0.93551	0.93984	0.94440
362	0.91720	0.92623	0.93018	0.93149	0.93566	0.93997	0.94452
363	0.91742	0.92639	0.93034	0.93165	0.93581	0.94011	0.94465
364	0.91762	0.92656	0.93050	0.93180	0.93595	0.94025	0.94477
365	0.91780	0.92673	0.93066	0.93196	0.93610	0.94038	0.94490

N	0.1%	0.5%	1%	1.25%	2.5%	5%	10%
366	0.91799	0.92689	0.93082	0.93212	0.93624	0.94052	0.94502
367	0.91817	0.92707	0.93097	0.93227	0.93639	0.94065	0.94514
368	0.91836	0.92723	0.93113	0.93242	0.93653	0.94078	0.94526
369	0.91855	0.92739	0.93128	0.93257	0.93667	0.94091	0.94538
370	0.91873	0.92756	0.93144	0.93273	0.93681	0.94104	0.94550
371	0.91893	0.92772	0.93159	0.93288	0.93695	0.94117	0.94562
372	0.91910	0.92788	0.93175	0.93303	0.93709	0.94130	0.94574
373	0.91930	0.92804	0.93190	0.93318	0.93723	0.94143	0.94586
374	0.91948	0.92820	0.93206	0.93333	0.93737	0.94156	0.94597
375	0.91968	0.92837	0.93221	0.93348	0.93751	0.94169	0.94609
376	0.91986	0.92851	0.93236	0.93362	0.93765	0.94182	0.94621
377	0.92002	0.92867	0.93251	0.93377	0.93779	0.94194	0.94632
378	0.92021	0.92883	0.93266	0.93392	0.93792	0.94207	0.94644
379	0.92038	0.92898	0.93281	0.93406	0.93806	0.94219	0.94655
380	0.92056	0.92914	0.93295	0.93421	0.93820	0.94232	0.94667
381	0.92075	0.92930	0.93311	0.93435	0.93833	0.94244	0.94678
382	0.92092	0.92946	0.93325	0.93450	0.93847	0.94257	0.94690
383	0.92109	0.92962	0.93340	0.93464	0.93860	0.94269	0.94701
384	0.92127	0.92978	0.93355	0.93479	0.93874	0.94281	0.94712
385	0.92147	0.92993	0.93369	0.93493	0.93887	0.94294	0.94723
386	0.92164	0.93008	0.93384	0.93507	0.93900	0.94306	0.94735
387	0.92181	0.93023	0.93398	0.93521	0.93913	0.94318	0.94746
388	0.92198	0.93039	0.93413	0.93535	0.93926	0.94330	0.94757
389	0.92214	0.93054	0.93427	0.93549	0.93939	0.94342	0.94768
390	0.92231	0.93069	0.93442	0.93563	0.93952	0.94354	0.94779
391	0.92248	0.93084	0.93456	0.93577	0.93965	0.94366	0.94789
392	0.92265	0.93099	0.93469	0.93591	0.93977	0.94378	0.94800
393	0.92281	0.93114	0.93484	0.93605	0.93990	0.94390	0.94811
394	0.92299	0.93128	0.93498	0.93618	0.94002	0.94402	0.94822
395	0.92315	0.93143	0.93511	0.93631	0.94016	0.94413	0.94833
396	0.92332	0.93157	0.93525	0.93645	0.94028	0.94424	0.94843
397	0.92349	0.93173	0.93538	0.93659	0.94041	0.94436	0.94854
398	0.92365	0.93187	0.93552	0.93672	0.94053	0.94448	0.94864
399	0.92381	0.93202	0.93566	0.93685	0.94065	0.94459	0.94875
400	0.92396	0.93216	0.93579	0.93698	0.94077	0.94471	0.94885
401	0.92412	0.93231	0.93592	0.93712	0.94090	0.94482	0.94896
402	0.92428	0.93245	0.93605	0.93725	0.94102	0.94493	0.94906
403	0.92447	0.93259	0.93620	0.93738	0.94115	0.94505	0.94916
404	0.92463	0.93274	0.93632	0.93751	0.94127	0.94516	0.94927
405	0.92479	0.93288	0.93645	0.93764	0.94139	0.94527	0.94937
406	0.92496	0.93302	0.93658	0.93776	0.94151	0.94538	0.94947
407	0.92510	0.93315	0.93672	0.93789	0.94163	0.94549	0.94957
408	0.92526	0.93329	0.93685	0.93802	0.94175	0.94560	0.94967
409	0.92541	0.93343	0.93698	0.93815	0.94187	0.94572	0.94977
410	0.92557	0.93357	0.93712	0.93828	0.94198	0.94582	0.94987
411	0.92573	0.93370	0.93724	0.93840	0.94210	0.94593	0.94997
412	0.92587	0.93383	0.93737	0.93853	0.94222	0.94604	0.95007
413	0.92603	0.93397	0.93750	0.93866	0.94233	0.94615	0.95017
414	0.92619	0.93410	0.93763	0.93878	0.94245	0.94625	0.95027
415	0.92634	0.93424	0.93775	0.93891	0.94257	0.94636	0.95036
416	0.92650	0.93437	0.93788	0.93903	0.94269	0.94647	0.95046
417	0.92665	0.93451	0.93800	0.93915	0.94280	0.94658	0.95056
418	0.92681	0.93464	0.93813	0.93927	0.94291	0.94668	0.95066
419	0.92694	0.93477	0.93826	0.93939	0.94303	0.94679	0.95075
420	0.92708	0.93490	0.93838	0.93952	0.94314	0.94689	0.95085
421	0.92723	0.93504	0.93850	0.93963	0.94325	0.94700	0.95095
422	0.92737	0.93517	0.93863	0.93975	0.94337	0.94710	0.95104
423	0.92751	0.93530	0.93875	0.93987	0.94348	0.94720	0.95113
424	0.92767	0.93543	0.93887	0.93998	0.94359	0.94731	0.95123
425	0.92781	0.93555	0.93899	0.94010	0.94370	0.94741	0.95132
426	0.92795	0.93568	0.93911	0.94022	0.94381	0.94751	0.95142
427	0.92809	0.93581	0.93923	0.94034	0.94392	0.94761	0.95151
428	0.92823	0.93594	0.93935	0.94046	0.94402	0.94771	0.95160
429	0.92839	0.93607	0.93947	0.94058	0.94413	0.94781	0.95169
430	0.92853	0.93620	0.93958	0.94069	0.94424	0.94791	0.95178
431	0.92867	0.93633	0.93971	0.94080	0.94435	0.94801	0.95187
432	0.92881	0.93646	0.93982	0.94092	0.94446	0.94811	0.95197
433	0.92895	0.93658	0.93994	0.94104	0.94456	0.94821	0.95206
434	0.92910	0.93670	0.94006	0.94116	0.94467	0.94831	0.95215
435	0.92925	0.93683	0.94018	0.94127	0.94477	0.94841	0.95224
436	0.92938	0.93695	0.94029	0.94138	0.94488	0.94851	0.95233
437	0.92953	0.93707	0.94040	0.94150	0.94498	0.94860	0.95242
438	0.92966	0.93719	0.94052	0.94161	0.94509	0.94870	0.95251
439	0.92980	0.93731	0.94064	0.94172	0.94519	0.94880	0.95260

N	0.1%	0.5%	1%	1.25%	2.5%	5%	10%
440	0.92995	0.93744	0.94075	0.94183	0.94530	0.94890	0.95268
441	0.93007	0.93755	0.94086	0.94194	0.94540	0.94899	0.95277
442	0.93021	0.93767	0.94098	0.94206	0.94550	0.94909	0.95286
443	0.93034	0.93779	0.94109	0.94217	0.94561	0.94918	0.95295
444	0.93049	0.93791	0.94120	0.94228	0.94571	0.94928	0.95303
445	0.93063	0.93803	0.94131	0.94239	0.94582	0.94937	0.95312
446	0.93075	0.93815	0.94142	0.94250	0.94592	0.94947	0.95320
447	0.93088	0.93827	0.94153	0.94261	0.94602	0.94956	0.95329
448	0.93101	0.93838	0.94165	0.94271	0.94612	0.94966	0.95338
449	0.93114	0.93850	0.94176	0.94282	0.94622	0.94975	0.95346
450	0.93128	0.93861	0.94187	0.94293	0.94632	0.94984	0.95355
451	0.93140	0.93873	0.94198	0.94304	0.94642	0.94993	0.95363
452	0.93153	0.93885	0.94208	0.94314	0.94652	0.95002	0.95372
453	0.93167	0.93896	0.94219	0.94325	0.94662	0.95012	0.95380
454	0.93181	0.93907	0.94230	0.94335	0.94671	0.95021	0.95388
455	0.93193	0.93919	0.94241	0.94346	0.94681	0.95030	0.95397
456	0.93205	0.93930	0.94251	0.94357	0.94691	0.95038	0.95405
457	0.93218	0.93942	0.94262	0.94367	0.94701	0.95047	0.95413
458	0.93230	0.93952	0.94272	0.94378	0.94711	0.95057	0.95421
459	0.93242	0.93963	0.94283	0.94388	0.94720	0.95065	0.95429
460	0.93255	0.93974	0.94293	0.94398	0.94730	0.95074	0.95438
461	0.93268	0.93986	0.94304	0.94408	0.94739	0.95083	0.95446
462	0.93280	0.93997	0.94315	0.94418	0.94749	0.95092	0.95454
463	0.93292	0.94008	0.94325	0.94428	0.94758	0.95101	0.95462
464	0.93305	0.94019	0.94335	0.94439	0.94768	0.95110	0.95470
465	0.93317	0.94031	0.94345	0.94449	0.94777	0.95118	0.95478
466	0.93329	0.94041	0.94356	0.94459	0.94787	0.95127	0.95486
467	0.93341	0.94051	0.94366	0.94469	0.94796	0.95136	0.95494
468	0.93353	0.94063	0.94376	0.94479	0.94805	0.95144	0.95502
469	0.93366	0.94074	0.94386	0.94489	0.94814	0.95153	0.95510
470	0.93378	0.94084	0.94396	0.94499	0.94824	0.95162	0.95517
471	0.93390	0.94095	0.94406	0.94509	0.94833	0.95170	0.95525
472	0.93403	0.94105	0.94416	0.94519	0.94842	0.95179	0.95533
473	0.93413	0.94116	0.94426	0.94528	0.94851	0.95187	0.95541
474	0.93425	0.94127	0.94436	0.94537	0.94860	0.95195	0.95548
475	0.93436	0.94137	0.94446	0.94547	0.94869	0.95204	0.95556
476	0.93449	0.94148	0.94456	0.94557	0.94878	0.95212	0.95564
477	0.93461	0.94159	0.94466	0.94566	0.94888	0.95221	0.95572
478	0.93473	0.94169	0.94475	0.94576	0.94897	0.95229	0.95579
479	0.93486	0.94180	0.94485	0.94586	0.94905	0.95237	0.95587
480	0.93496	0.94190	0.94495	0.94595	0.94914	0.95246	0.95594
481	0.93509	0.94200	0.94505	0.94605	0.94923	0.95254	0.95602
482	0.93521	0.94211	0.94515	0.94614	0.94932	0.95262	0.95609
483	0.93532	0.94221	0.94524	0.94623	0.94941	0.95270	0.95617
484	0.93542	0.94231	0.94534	0.94633	0.94949	0.95278	0.95624
485	0.93554	0.94241	0.94544	0.94642	0.94958	0.95286	0.95632
486	0.93565	0.94252	0.94553	0.94652	0.94967	0.95294	0.95639
487	0.93576	0.94262	0.94563	0.94661	0.94976	0.95303	0.95646
488	0.93587	0.94271	0.94572	0.94671	0.94984	0.95311	0.95654
489	0.93598	0.94281	0.94582	0.94680	0.94993	0.95318	0.95661
490	0.93611	0.94291	0.94591	0.94689	0.95001	0.95326	0.95668
491	0.93622	0.94301	0.94600	0.94698	0.95010	0.95334	0.95676
492	0.93633	0.94311	0.94609	0.94707	0.95018	0.95342	0.95683
493	0.93643	0.94321	0.94619	0.94716	0.95027	0.95350	0.95690
494	0.93655	0.94331	0.94628	0.94725	0.95035	0.95358	0.95697
495	0.93665	0.94340	0.94637	0.94734	0.95043	0.95366	0.95704
496	0.93676	0.94350	0.94646	0.94743	0.95052	0.95373	0.95712
497	0.93688	0.94360	0.94655	0.94752	0.95060	0.95381	0.95719
498	0.93700	0.94370	0.94664	0.94761	0.95068	0.95389	0.95726
499	0.93710	0.94379	0.94674	0.94770	0.95077	0.95397	0.95733
500	0.93721	0.94389	0.94683	0.94779	0.95085	0.95404	0.95740
501	0.93732	0.94399	0.94691	0.94788	0.95093	0.95412	0.95747
502	0.93742	0.94408	0.94700	0.94796	0.95101	0.95420	0.95754
503	0.93753	0.94418	0.94709	0.94805	0.95110	0.95427	0.95761
504	0.93763	0.94427	0.94718	0.94814	0.95118	0.95435	0.95767
505	0.93773	0.94436	0.94727	0.94822	0.95126	0.95442	0.95774
506	0.93785	0.94446	0.94736	0.94831	0.95134	0.95450	0.95781
507	0.93795	0.94455	0.94745	0.94840	0.95142	0.95457	0.95788
508	0.93806	0.94464	0.94754	0.94848	0.95150	0.95465	0.95795
509	0.93817	0.94474	0.94762	0.94857	0.95158	0.95472	0.95802
510	0.93828	0.94483	0.94771	0.94865	0.95166	0.95479	0.95808
511	0.93837	0.94492	0.94779	0.94874	0.95174	0.95487	0.95815
512	0.93847	0.94502	0.94788	0.94883	0.95182	0.95494	0.95822
513	0.93858	0.94510	0.94797	0.94891	0.95190	0.95501	0.95829

N	0.1%	0.5%	1%	1.25%	2.5%	5%	10%
514	0.93868	0.94520	0.94805	0.94899	0.95198	0.95509	0.95835
515	0.93878	0.94529	0.94814	0.94907	0.95206	0.95516	0.95842
516	0.93888	0.94538	0.94822	0.94916	0.95213	0.95523	0.95849
517	0.93899	0.94547	0.94831	0.94924	0.95221	0.95530	0.95855
518	0.93909	0.94556	0.94839	0.94933	0.95228	0.95538	0.95862
519	0.93918	0.94565	0.94847	0.94941	0.95236	0.95545	0.95869
520	0.93930	0.94573	0.94855	0.94949	0.95244	0.95552	0.95875
521	0.93939	0.94582	0.94864	0.94957	0.95252	0.95559	0.95882
522	0.93950	0.94591	0.94873	0.94965	0.95259	0.95566	0.95888
523	0.93960	0.94601	0.94881	0.94974	0.95267	0.95573	0.95895
524	0.93971	0.94610	0.94889	0.94982	0.95274	0.95580	0.95901
525	0.93982	0.94618	0.94897	0.94990	0.95282	0.95587	0.95907
526	0.93992	0.94626	0.94906	0.94998	0.95290	0.95594	0.95914
527	0.94001	0.94636	0.94914	0.95006	0.95297	0.95601	0.95920
528	0.94010	0.94644	0.94922	0.95014	0.95305	0.95608	0.95927
529	0.94019	0.94653	0.94930	0.95022	0.95312	0.95615	0.95933
530	0.94030	0.94661	0.94938	0.95030	0.95320	0.95622	0.95939
531	0.94040	0.94670	0.94946	0.95037	0.95327	0.95629	0.95946
532	0.94049	0.94678	0.94954	0.95045	0.95335	0.95636	0.95952
533	0.94059	0.94687	0.94962	0.95053	0.95342	0.95643	0.95958
534	0.94068	0.94695	0.94970	0.95061	0.95350	0.95649	0.95964
535	0.94078	0.94704	0.94978	0.95069	0.95357	0.95656	0.95971
536	0.94087	0.94713	0.94986	0.95077	0.95364	0.95663	0.95977
537	0.94097	0.94721	0.94994	0.95085	0.95372	0.95670	0.95983
538	0.94107	0.94729	0.95002	0.95093	0.95379	0.95676	0.95989
539	0.94117	0.94737	0.95010	0.95101	0.95386	0.95683	0.95995
540	0.94127	0.94746	0.95018	0.95109	0.95393	0.95689	0.96001
541	0.94135	0.94754	0.95026	0.95116	0.95400	0.95696	0.96007
542	0.94144	0.94763	0.95034	0.95124	0.95407	0.95703	0.96014
543	0.94154	0.94771	0.95041	0.95132	0.95414	0.95709	0.96020
544	0.94163	0.94779	0.95049	0.95139	0.95422	0.95716	0.96026
545	0.94172	0.94787	0.95057	0.95147	0.95429	0.95723	0.96032
546	0.94181	0.94796	0.95064	0.95154	0.95436	0.95729	0.96038
547	0.94191	0.94804	0.95072	0.95162	0.95443	0.95736	0.96044
548	0.94200	0.94813	0.95080	0.95169	0.95450	0.95742	0.96050
549	0.94210	0.94820	0.95088	0.95177	0.95457	0.95749	0.96055
550	0.94220	0.94828	0.95095	0.95184	0.95464	0.95755	0.96061
551	0.94229	0.94837	0.95103	0.95192	0.95470	0.95762	0.96067
552	0.94238	0.94845	0.95110	0.95199	0.95477	0.95768	0.96073
553	0.94248	0.94853	0.95118	0.95207	0.95484	0.95774	0.96079
554	0.94258	0.94861	0.95125	0.95214	0.95491	0.95781	0.96085
555	0.94267	0.94868	0.95133	0.95221	0.95498	0.95787	0.96091
556	0.94275	0.94876	0.95140	0.95228	0.95505	0.95794	0.96097
557	0.94284	0.94884	0.95148	0.95235	0.95512	0.95800	0.96103
558	0.94293	0.94892	0.95155	0.95243	0.95519	0.95806	0.96108
559	0.94302	0.94900	0.95162	0.95250	0.95525	0.95812	0.96114
560	0.94310	0.94908	0.95170	0.95257	0.95532	0.95818	0.96120
561	0.94321	0.94916	0.95177	0.95264	0.95539	0.95825	0.96126
562	0.94330	0.94924	0.95184	0.95271	0.95545	0.95831	0.96131
563	0.94337	0.94932	0.95192	0.95278	0.95552	0.95837	0.96137
564	0.94348	0.94939	0.95199	0.95286	0.95559	0.95843	0.96143
565	0.94356	0.94947	0.95206	0.95293	0.95566	0.95849	0.96148
566	0.94364	0.94954	0.95213	0.95300	0.95572	0.95856	0.96154
567	0.94373	0.94962	0.95220	0.95307	0.95579	0.95862	0.96159
568	0.94381	0.94970	0.95227	0.95314	0.95585	0.95868	0.96165
569	0.94390	0.94977	0.95234	0.95321	0.95592	0.95874	0.96171
570	0.94399	0.94984	0.95242	0.95328	0.95598	0.95880	0.96176
571	0.94408	0.94992	0.95249	0.95335	0.95605	0.95886	0.96182
572	0.94416	0.95000	0.95256	0.95342	0.95611	0.95892	0.96187
573	0.94424	0.95007	0.95263	0.95349	0.95618	0.95898	0.96193
574	0.94431	0.95015	0.95270	0.95356	0.95624	0.95904	0.96198
575	0.94439	0.95022	0.95277	0.95363	0.95630	0.95910	0.96204
576	0.94447	0.95030	0.95284	0.95369	0.95637	0.95916	0.96209
577	0.94455	0.95037	0.95291	0.95376	0.95643	0.95922	0.96215
578	0.94464	0.95044	0.95298	0.95383	0.95650	0.95927	0.96220
579	0.94472	0.95052	0.95305	0.95390	0.95656	0.95933	0.96225
580	0.94481	0.95059	0.95312	0.95397	0.95662	0.95939	0.96231
581	0.94489	0.95067	0.95319	0.95403	0.95669	0.95945	0.96236
582	0.94497	0.95074	0.95326	0.95410	0.95675	0.95951	0.96241
583	0.94505	0.95081	0.95333	0.95417	0.95682	0.95956	0.96247
584	0.94513	0.95088	0.95340	0.95423	0.95688	0.95962	0.96252
585	0.94521	0.95095	0.95346	0.95430	0.95694	0.95968	0.96257
586	0.94529	0.95102	0.95353	0.95437	0.95700	0.95974	0.96263
587	0.94537	0.95110	0.95360	0.95443	0.95706	0.95979	0.96268

N	0.1%	0.5%	1%	1.25%	2.5%	5%	10%
588	0.94546	0.95117	0.95367	0.95450	0.95713	0.95985	0.96273
589	0.94553	0.95124	0.95374	0.95456	0.95719	0.95991	0.96279
590	0.94561	0.95131	0.95380	0.95463	0.95725	0.95997	0.96284
591	0.94570	0.95138	0.95387	0.95469	0.95731	0.96002	0.96289
592	0.94579	0.95145	0.95394	0.95476	0.95737	0.96008	0.96294
593	0.94586	0.95152	0.95400	0.95482	0.95743	0.96013	0.96299
594	0.94595	0.95159	0.95407	0.95489	0.95749	0.96019	0.96304
595	0.94602	0.95166	0.95414	0.95495	0.95755	0.96025	0.96309
596	0.94610	0.95173	0.95420	0.95502	0.95761	0.96030	0.96315
597	0.94619	0.95180	0.95427	0.95508	0.95767	0.96036	0.96320
598	0.94626	0.95187	0.95433	0.95515	0.95773	0.96042	0.96325
599	0.94633	0.95194	0.95440	0.95521	0.95779	0.96047	0.96330
600	0.94642	0.95201	0.95446	0.95527	0.95785	0.96053	0.96335
601	0.94648	0.95207	0.95453	0.95534	0.95791	0.96058	0.96340
602	0.94655	0.95214	0.95459	0.95540	0.95797	0.96064	0.96345
603	0.94665	0.95220	0.95465	0.95546	0.95802	0.96069	0.96350
604	0.94672	0.95227	0.95472	0.95553	0.95808	0.96075	0.96355
605	0.94680	0.95234	0.95478	0.95559	0.95814	0.96080	0.96360
606	0.94688	0.95241	0.95485	0.95565	0.95820	0.96086	0.96365
607	0.94695	0.95247	0.95491	0.95572	0.95826	0.96091	0.96370
608	0.94704	0.95254	0.95497	0.95578	0.95832	0.96096	0.96375
609	0.94711	0.95261	0.95503	0.95584	0.95838	0.96102	0.96380
610	0.94719	0.95267	0.95510	0.95590	0.95844	0.96107	0.96385
611	0.94726	0.95274	0.95516	0.95596	0.95849	0.96112	0.96390
612	0.94734	0.95281	0.95522	0.95602	0.95855	0.96118	0.96394
613	0.94741	0.95288	0.95529	0.95608	0.95860	0.96123	0.96399
614	0.94748	0.95294	0.95535	0.95615	0.95866	0.96128	0.96404
615	0.94756	0.95301	0.95541	0.95621	0.95872	0.96133	0.96409
616	0.94763	0.95307	0.95547	0.95627	0.95878	0.96139	0.96414
617	0.94770	0.95314	0.95553	0.95633	0.95883	0.96144	0.96419
618	0.94778	0.95320	0.95559	0.95639	0.95889	0.96149	0.96423
619	0.94785	0.95326	0.95565	0.95645	0.95894	0.96154	0.96428
620	0.94793	0.95333	0.95572	0.95651	0.95900	0.96160	0.96433
621	0.94799	0.95339	0.95578	0.95657	0.95905	0.96165	0.96438
622	0.94807	0.95346	0.95584	0.95663	0.95911	0.96170	0.96442
623	0.94814	0.95352	0.95590	0.95669	0.95917	0.96175	0.96447
624	0.94821	0.95359	0.95596	0.95675	0.95922	0.96180	0.96452
625	0.94828	0.95365	0.95602	0.95680	0.95928	0.96185	0.96457
626	0.94835	0.95371	0.95608	0.95686	0.95933	0.96190	0.96461
627	0.94842	0.95378	0.95614	0.95692	0.95939	0.96195	0.96466
628	0.94849	0.95384	0.95620	0.95698	0.95944	0.96201	0.96471
629	0.94856	0.95390	0.95626	0.95704	0.95950	0.96206	0.96476
630	0.94863	0.95396	0.95632	0.95709	0.95955	0.96211	0.96480
631	0.94870	0.95403	0.95638	0.95715	0.95961	0.96216	0.96485
632	0.94876	0.95409	0.95644	0.95721	0.95966	0.96221	0.96490
633	0.94884	0.95415	0.95650	0.95727	0.95971	0.96226	0.96494
634	0.94892	0.95421	0.95656	0.95733	0.95977	0.96231	0.96499
635	0.94898	0.95428	0.95662	0.95739	0.95982	0.96236	0.96503
636	0.94905	0.95433	0.95667	0.95744	0.95988	0.96241	0.96508
637	0.94912	0.95439	0.95673	0.95750	0.95993	0.96246	0.96512
638	0.94919	0.95446	0.95679	0.95756	0.95999	0.96251	0.96517
639	0.94926	0.95452	0.95685	0.95762	0.96004	0.96256	0.96521
640	0.94932	0.95458	0.95691	0.95767	0.96009	0.96261	0.96526
641	0.94939	0.95464	0.95696	0.95773	0.96014	0.96265	0.96530
642	0.94946	0.95470	0.95702	0.95778	0.96020	0.96270	0.96535
643	0.94952	0.95476	0.95708	0.95784	0.96025	0.96275	0.96539
644	0.94960	0.95482	0.95714	0.95789	0.96030	0.96280	0.96544
645	0.94966	0.95488	0.95720	0.95795	0.96035	0.96285	0.96548
646	0.94973	0.95494	0.95725	0.95800	0.96040	0.96290	0.96553
647	0.94981	0.95500	0.95731	0.95806	0.96045	0.96294	0.96557
648	0.94987	0.95505	0.95737	0.95812	0.96050	0.96299	0.96562
649	0.94994	0.95512	0.95742	0.95817	0.96056	0.96304	0.96566
650	0.95000	0.95518	0.95748	0.95822	0.96061	0.96309	0.96570
651	0.95007	0.95524	0.95753	0.95828	0.96066	0.96314	0.96575
652	0.95012	0.95530	0.95759	0.95833	0.96071	0.96318	0.96579
653	0.95019	0.95536	0.95765	0.95839	0.96076	0.96323	0.96583
654	0.95026	0.95542	0.95771	0.95844	0.96081	0.96328	0.96588
655	0.95034	0.95547	0.95776	0.95850	0.96086	0.96332	0.96592
656	0.95040	0.95553	0.95782	0.95855	0.96091	0.96337	0.96596
657	0.95046	0.95559	0.95788	0.95860	0.96097	0.96342	0.96601
658	0.95053	0.95565	0.95793	0.95866	0.96102	0.96347	0.96605
659	0.95059	0.95571	0.95798	0.95871	0.96107	0.96351	0.96609
660	0.95066	0.95577	0.95803	0.95876	0.96112	0.96356	0.96613
661	0.95072	0.95582	0.95809	0.95882	0.96117	0.96360	0.96618

N	0.1%	0.5%	1%	1.25%	2.5%	5%	10%
662	0.95079	0.95588	0.95814	0.95887	0.96122	0.96365	0.96622
663	0.95085	0.95594	0.95820	0.95892	0.96127	0.96370	0.96626
664	0.95091	0.95599	0.95825	0.95898	0.96131	0.96374	0.96630
665	0.95099	0.95605	0.95831	0.95903	0.96136	0.96379	0.96635
666	0.95105	0.95610	0.95836	0.95908	0.96141	0.96383	0.96639
667	0.95111	0.95617	0.95841	0.95913	0.96147	0.96388	0.96643
668	0.95119	0.95622	0.95846	0.95918	0.96151	0.96392	0.96647
669	0.95124	0.95628	0.95852	0.95924	0.96156	0.96397	0.96651
670	0.95131	0.95633	0.95857	0.95929	0.96161	0.96402	0.96656
671	0.95137	0.95639	0.95862	0.95934	0.96166	0.96406	0.96660
672	0.95143	0.95644	0.95868	0.95940	0.96171	0.96410	0.96664
673	0.95150	0.95650	0.95873	0.95945	0.96176	0.96415	0.96668
674	0.95156	0.95655	0.95878	0.95950	0.96181	0.96420	0.96672
675	0.95162	0.95660	0.95883	0.95955	0.96185	0.96424	0.96676
676	0.95168	0.95666	0.95889	0.95960	0.96190	0.96428	0.96680
677	0.95174	0.95672	0.95894	0.95966	0.96195	0.96433	0.96684
678	0.95181	0.95677	0.95899	0.95970	0.96200	0.96437	0.96688
679	0.95187	0.95683	0.95904	0.95976	0.96204	0.96442	0.96693
680	0.95192	0.95688	0.95910	0.95981	0.96209	0.96446	0.96697
681	0.95198	0.95693	0.95915	0.95986	0.96214	0.96451	0.96701
682	0.95204	0.95699	0.95920	0.95991	0.96219	0.96455	0.96705
683	0.95211	0.95704	0.95925	0.95996	0.96223	0.96459	0.96709
684	0.95218	0.95710	0.95930	0.96001	0.96228	0.96464	0.96713
685	0.95224	0.95716	0.95935	0.96006	0.96233	0.96468	0.96717
686	0.95230	0.95721	0.95941	0.96011	0.96237	0.96472	0.96721
687	0.95235	0.95727	0.95946	0.96016	0.96242	0.96477	0.96725
688	0.95242	0.95732	0.95951	0.96021	0.96247	0.96481	0.96728
689	0.95248	0.95737	0.95956	0.96026	0.96251	0.96485	0.96732
690	0.95254	0.95743	0.95961	0.96031	0.96256	0.96490	0.96736
691	0.95260	0.95748	0.95966	0.96036	0.96261	0.96494	0.96740
692	0.95266	0.95753	0.95971	0.96041	0.96265	0.96498	0.96744
693	0.95271	0.95759	0.95976	0.96046	0.96270	0.96502	0.96748
694	0.95278	0.95763	0.95980	0.96050	0.96274	0.96507	0.96752
695	0.95283	0.95769	0.95985	0.96055	0.96279	0.96511	0.96756
696	0.95290	0.95774	0.95990	0.96060	0.96283	0.96515	0.96760
697	0.95296	0.95779	0.95995	0.96065	0.96288	0.96519	0.96764
698	0.95302	0.95784	0.96000	0.96070	0.96292	0.96523	0.96768
699	0.95308	0.95789	0.96005	0.96074	0.96297	0.96528	0.96771
700	0.95314	0.95795	0.96010	0.96079	0.96301	0.96532	0.96775
701	0.95318	0.95800	0.96015	0.96084	0.96306	0.96536	0.96779
702	0.95324	0.95805	0.96019	0.96088	0.96310	0.96540	0.96783
703	0.95330	0.95810	0.96024	0.96093	0.96315	0.96544	0.96787
704	0.95336	0.95815	0.96029	0.96098	0.96319	0.96548	0.96790
705	0.95342	0.95820	0.96034	0.96103	0.96324	0.96553	0.96794
706	0.95348	0.95826	0.96039	0.96107	0.96328	0.96557	0.96798
707	0.95354	0.95831	0.96044	0.96112	0.96332	0.96561	0.96802
708	0.95359	0.95836	0.96048	0.96117	0.96337	0.96565	0.96805
709	0.95365	0.95841	0.96053	0.96122	0.96341	0.96569	0.96809
710	0.95371	0.95846	0.96058	0.96126	0.96346	0.96573	0.96813
711	0.95377	0.95851	0.96063	0.96131	0.96350	0.96577	0.96817
712	0.95383	0.95857	0.96068	0.96135	0.96354	0.96581	0.96820
713	0.95389	0.95862	0.96072	0.96140	0.96359	0.96585	0.96824
714	0.95394	0.95867	0.96077	0.96145	0.96363	0.96589	0.96828
715	0.95400	0.95872	0.96082	0.96149	0.96367	0.96593	0.96832
716	0.95406	0.95877	0.96086	0.96154	0.96372	0.96597	0.96835
717	0.95412	0.95882	0.96091	0.96158	0.96376	0.96601	0.96839
718	0.95417	0.95886	0.96096	0.96163	0.96380	0.96605	0.96843
719	0.95423	0.95891	0.96100	0.96168	0.96384	0.96609	0.96846
720	0.95428	0.95896	0.96105	0.96172	0.96389	0.96613	0.96850
721	0.95434	0.95901	0.96110	0.96177	0.96393	0.96617	0.96853
722	0.95439	0.95906	0.96114	0.96181	0.96397	0.96621	0.96857
723	0.95445	0.95911	0.96119	0.96186	0.96402	0.96625	0.96861
724	0.95451	0.95915	0.96123	0.96190	0.96406	0.96629	0.96864
725	0.95456	0.95920	0.96128	0.96195	0.96410	0.96633	0.96868
726	0.95461	0.95925	0.96133	0.96199	0.96414	0.96636	0.96872
727	0.95467	0.95929	0.96137	0.96204	0.96419	0.96640	0.96875
728	0.95472	0.95934	0.96142	0.96208	0.96423	0.96644	0.96879
729	0.95478	0.95939	0.96146	0.96212	0.96427	0.96648	0.96882
730	0.95483	0.95944	0.96150	0.96217	0.96431	0.96652	0.96886
731	0.95489	0.95949	0.96155	0.96222	0.96435	0.96656	0.96889
732	0.95494	0.95954	0.96159	0.96226	0.96439	0.96660	0.96893
733	0.95499	0.95958	0.96164	0.96230	0.96444	0.96664	0.96896
734	0.95505	0.95963	0.96168	0.96235	0.96448	0.96667	0.96900
735	0.95510	0.95968	0.96173	0.96239	0.96452	0.96671	0.96903

N	0.1%	0.5%	1%	1.25%	2.5%	5%	10%
736	0.95515	0.95973	0.96177	0.96244	0.96456	0.96675	0.96907
737	0.95520	0.95978	0.96182	0.96248	0.96460	0.96679	0.96910
738	0.95525	0.95982	0.96187	0.96252	0.96464	0.96683	0.96914
739	0.95530	0.95987	0.96191	0.96257	0.96468	0.96686	0.96917
740	0.95536	0.95992	0.96195	0.96261	0.96472	0.96690	0.96921
741	0.95541	0.95997	0.96200	0.96265	0.96476	0.96694	0.96924
742	0.95546	0.96001	0.96204	0.96270	0.96480	0.96698	0.96928
743	0.95551	0.96006	0.96208	0.96274	0.96484	0.96701	0.96931
744	0.95556	0.96010	0.96213	0.96278	0.96488	0.96705	0.96935
745	0.95562	0.96015	0.96217	0.96283	0.96492	0.96709	0.96938
746	0.95567	0.96019	0.96221	0.96287	0.96496	0.96712	0.96942
747	0.95572	0.96024	0.96226	0.96291	0.96500	0.96716	0.96945
748	0.95577	0.96029	0.96230	0.96295	0.96504	0.96720	0.96949
749	0.95582	0.96033	0.96235	0.96300	0.96508	0.96723	0.96952
750	0.95587	0.96038	0.96239	0.96304	0.96512	0.96727	0.96955
751	0.95592	0.96042	0.96243	0.96308	0.96516	0.96731	0.96959
752	0.95597	0.96047	0.96248	0.96312	0.96520	0.96735	0.96962
753	0.95603	0.96052	0.96252	0.96317	0.96524	0.96738	0.96965
754	0.95608	0.96056	0.96256	0.96321	0.96528	0.96742	0.96969
755	0.95613	0.96060	0.96260	0.96325	0.96531	0.96746	0.96972
756	0.95618	0.96065	0.96264	0.96329	0.96536	0.96749	0.96976
757	0.95623	0.96070	0.96268	0.96333	0.96539	0.96753	0.96979
758	0.95628	0.96074	0.96273	0.96337	0.96543	0.96757	0.96982
759	0.95634	0.96079	0.96277	0.96342	0.96547	0.96760	0.96985
760	0.95639	0.96083	0.96281	0.96346	0.96551	0.96764	0.96989
761	0.95644	0.96088	0.96285	0.96350	0.96555	0.96767	0.96992
762	0.95649	0.96092	0.96290	0.96354	0.96559	0.96771	0.96995
763	0.95654	0.96097	0.96294	0.96358	0.96563	0.96774	0.96999
764	0.95658	0.96101	0.96298	0.96362	0.96566	0.96778	0.97002
765	0.95663	0.96106	0.96302	0.96367	0.96570	0.96781	0.97005
766	0.95668	0.96110	0.96306	0.96371	0.96574	0.96785	0.97009
767	0.95673	0.96114	0.96310	0.96375	0.96578	0.96788	0.97012
768	0.95679	0.96119	0.96315	0.96379	0.96582	0.96792	0.97015
769	0.95684	0.96123	0.96319	0.96383	0.96585	0.96796	0.97018
770	0.95689	0.96128	0.96323	0.96387	0.96589	0.96799	0.97021
771	0.95694	0.96132	0.96327	0.96391	0.96593	0.96803	0.97025
772	0.95699	0.96136	0.96331	0.96395	0.96597	0.96806	0.97028
773	0.95704	0.96141	0.96335	0.96399	0.96600	0.96810	0.97031
774	0.95708	0.96145	0.96339	0.96403	0.96604	0.96813	0.97034
775	0.95713	0.96149	0.96343	0.96407	0.96608	0.96817	0.97038
776	0.95718	0.96154	0.96347	0.96410	0.96612	0.96820	0.97041
777	0.95723	0.96158	0.96351	0.96415	0.96615	0.96823	0.97044
778	0.95727	0.96162	0.96355	0.96419	0.96619	0.96827	0.97047
779	0.95733	0.96167	0.96359	0.96422	0.96623	0.96830	0.97050
780	0.95737	0.96171	0.96363	0.96427	0.96627	0.96834	0.97053
781	0.95743	0.96176	0.96367	0.96430	0.96630	0.96837	0.97057
782	0.95748	0.96180	0.96371	0.96434	0.96634	0.96841	0.97060
783	0.95752	0.96184	0.96375	0.96438	0.96637	0.96844	0.97063
784	0.95757	0.96188	0.96379	0.96442	0.96641	0.96848	0.97066
785	0.95761	0.96192	0.96383	0.96446	0.96645	0.96851	0.97069
786	0.95766	0.96197	0.96387	0.96450	0.96649	0.96854	0.97072
787	0.95770	0.96201	0.96391	0.96454	0.96652	0.96858	0.97075
788	0.95775	0.96205	0.96395	0.96458	0.96656	0.96861	0.97078
789	0.95779	0.96209	0.96399	0.96461	0.96659	0.96865	0.97082
790	0.95785	0.96213	0.96403	0.96465	0.96663	0.96868	0.97085
791	0.95789	0.96217	0.96407	0.96469	0.96667	0.96871	0.97088
792	0.95794	0.96222	0.96411	0.96473	0.96670	0.96875	0.97091
793	0.95799	0.96226	0.96415	0.96477	0.96674	0.96878	0.97094
794	0.95804	0.96230	0.96419	0.96481	0.96677	0.96881	0.97097
795	0.95807	0.96234	0.96423	0.96485	0.96681	0.96885	0.97100
796	0.95813	0.96238	0.96426	0.96488	0.96684	0.96888	0.97103
797	0.95817	0.96242	0.96430	0.96492	0.96688	0.96891	0.97106
798	0.95822	0.96246	0.96434	0.96496	0.96692	0.96895	0.97109
799	0.95826	0.96250	0.96438	0.96500	0.96695	0.96898	0.97112
800	0.95830	0.96255	0.96442	0.96503	0.96699	0.96901	0.97115
801	0.95834	0.96259	0.96446	0.96507	0.96702	0.96904	0.97118
802	0.95839	0.96263	0.96449	0.96511	0.96706	0.96907	0.97121
803	0.95845	0.96267	0.96453	0.96515	0.96709	0.96911	0.97124
804	0.95849	0.96270	0.96457	0.96519	0.96713	0.96914	0.97127
805	0.95853	0.96275	0.96461	0.96522	0.96716	0.96917	0.97130
806	0.95858	0.96279	0.96465	0.96526	0.96720	0.96920	0.97133
807	0.95862	0.96283	0.96468	0.96530	0.96723	0.96924	0.97136
808	0.95867	0.96287	0.96472	0.96533	0.96727	0.96927	0.97139
809	0.95871	0.96291	0.96476	0.96537	0.96730	0.96930	0.97142

N	0.1%	0.5%	1%	1.25%	2.5%	5%	10%
810	0.95875	0.96294	0.96480	0.96541	0.96734	0.96933	0.97145
811	0.95881	0.96298	0.96483	0.96544	0.96737	0.96936	0.97148
812	0.95885	0.96302	0.96487	0.96548	0.96740	0.96940	0.97151
813	0.95889	0.96306	0.96491	0.96552	0.96744	0.96943	0.97154
814	0.95894	0.96310	0.96494	0.96555	0.96747	0.96946	0.97157
815	0.95898	0.96314	0.96498	0.96559	0.96751	0.96949	0.97160
816	0.95902	0.96318	0.96502	0.96563	0.96754	0.96952	0.97162
817	0.95906	0.96322	0.96506	0.96566	0.96757	0.96955	0.97165
818	0.95911	0.96326	0.96509	0.96570	0.96761	0.96959	0.97168
819	0.95916	0.96330	0.96513	0.96573	0.96764	0.96962	0.97171
820	0.95920	0.96334	0.96517	0.96577	0.96767	0.96965	0.97174
821	0.95924	0.96337	0.96520	0.96581	0.96771	0.96968	0.97177
822	0.95929	0.96341	0.96524	0.96584	0.96774	0.96971	0.97180
823	0.95933	0.96345	0.96528	0.96588	0.96777	0.96974	0.97183
824	0.95938	0.96349	0.96531	0.96591	0.96781	0.96977	0.97186
825	0.95942	0.96353	0.96535	0.96595	0.96784	0.96980	0.97188
826	0.95946	0.96356	0.96539	0.96598	0.96788	0.96983	0.97191
827	0.95950	0.96360	0.96542	0.96602	0.96791	0.96986	0.97194
828	0.95954	0.96364	0.96546	0.96605	0.96794	0.96990	0.97197
829	0.95959	0.96368	0.96549	0.96609	0.96798	0.96993	0.97200
830	0.95963	0.96371	0.96553	0.96612	0.96801	0.96996	0.97203
831	0.95967	0.96375	0.96556	0.96616	0.96804	0.96999	0.97205
832	0.95972	0.96379	0.96560	0.96619	0.96807	0.97002	0.97208
833	0.95976	0.96383	0.96564	0.96623	0.96810	0.97005	0.97211
834	0.95981	0.96387	0.96567	0.96626	0.96814	0.97008	0.97214
835	0.95984	0.96391	0.96571	0.96630	0.96817	0.97011	0.97217
836	0.95989	0.96394	0.96574	0.96633	0.96820	0.97014	0.97219
837	0.95993	0.96398	0.96578	0.96637	0.96824	0.97017	0.97222
838	0.95997	0.96402	0.96581	0.96640	0.96827	0.97020	0.97225
839	0.96002	0.96405	0.96585	0.96644	0.96830	0.97023	0.97228
840	0.96006	0.96409	0.96588	0.96647	0.96833	0.97026	0.97231
841	0.96010	0.96413	0.96592	0.96651	0.96836	0.97029	0.97233
842	0.96014	0.96417	0.96595	0.96654	0.96840	0.97032	0.97236
843	0.96018	0.96420	0.96599	0.96657	0.96843	0.97035	0.97239
844	0.96022	0.96424	0.96602	0.96661	0.96846	0.97038	0.97242
845	0.96026	0.96428	0.96606	0.96664	0.96849	0.97041	0.97244
846	0.96030	0.96431	0.96609	0.96668	0.96852	0.97044	0.97247
847	0.96034	0.96435	0.96613	0.96671	0.96856	0.97047	0.97250
848	0.96038	0.96439	0.96616	0.96674	0.96859	0.97050	0.97252
849	0.96043	0.96442	0.96620	0.96678	0.96862	0.97053	0.97255
850	0.96047	0.96446	0.96623	0.96681	0.96865	0.97056	0.97258
851	0.96051	0.96450	0.96626	0.96684	0.96868	0.97059	0.97261
852	0.96055	0.96453	0.96630	0.96688	0.96871	0.97062	0.97263
853	0.96060	0.96457	0.96633	0.96691	0.96874	0.97064	0.97266
854	0.96064	0.96461	0.96637	0.96695	0.96878	0.97067	0.97269
855	0.96068	0.96464	0.96640	0.96698	0.96881	0.97070	0.97271
856	0.96072	0.96468	0.96643	0.96701	0.96884	0.97073	0.97274
857	0.96076	0.96471	0.96647	0.96704	0.96887	0.97076	0.97277
858	0.96080	0.96475	0.96650	0.96708	0.96890	0.97079	0.97279
859	0.96084	0.96478	0.96654	0.96711	0.96893	0.97082	0.97282
860	0.96088	0.96482	0.96657	0.96714	0.96896	0.97085	0.97285
861	0.96092	0.96486	0.96660	0.96718	0.96899	0.97088	0.97287
862	0.96096	0.96489	0.96664	0.96721	0.96902	0.97091	0.97290
863	0.96100	0.96493	0.96667	0.96724	0.96905	0.97093	0.97292
864	0.96104	0.96496	0.96671	0.96727	0.96908	0.97096	0.97295
865	0.96108	0.96500	0.96674	0.96731	0.96911	0.97099	0.97298
866	0.96112	0.96503	0.96677	0.96734	0.96914	0.97102	0.97300
867	0.96116	0.96507	0.96680	0.96737	0.96917	0.97105	0.97303
868	0.96120	0.96511	0.96684	0.96741	0.96920	0.97108	0.97306
869	0.96123	0.96514	0.96687	0.96744	0.96923	0.97110	0.97308
870	0.96127	0.96517	0.96690	0.96747	0.96926	0.97113	0.97311
871	0.96131	0.96521	0.96693	0.96750	0.96929	0.97116	0.97313
872	0.96135	0.96524	0.96697	0.96753	0.96933	0.97119	0.97316
873	0.96140	0.96528	0.96700	0.96756	0.96936	0.97122	0.97319
874	0.96143	0.96531	0.96703	0.96760	0.96939	0.97124	0.97321
875	0.96147	0.96534	0.96707	0.96763	0.96941	0.97127	0.97324
876	0.96151	0.96538	0.96710	0.96766	0.96944	0.97130	0.97326
877	0.96155	0.96541	0.96713	0.96769	0.96948	0.97133	0.97329
878	0.96158	0.96545	0.96716	0.96772	0.96950	0.97136	0.97331
879	0.96163	0.96548	0.96719	0.96776	0.96953	0.97138	0.97334
880	0.96166	0.96551	0.96723	0.96779	0.96956	0.97141	0.97336
881	0.96170	0.96555	0.96726	0.96782	0.96959	0.97144	0.97339
882	0.96174	0.96558	0.96729	0.96785	0.96962	0.97147	0.97341
883	0.96178	0.96562	0.96732	0.96788	0.96965	0.97149	0.97344

N	0.1%	0.5%	1%	1.25%	2.5%	5%	10%
884	0.96181	0.96565	0.96736	0.96791	0.96968	0.97152	0.97347
885	0.96185	0.96568	0.96739	0.96794	0.96971	0.97155	0.97349
886	0.96189	0.96572	0.96742	0.96798	0.96974	0.97157	0.97352
887	0.96193	0.96575	0.96745	0.96801	0.96977	0.97160	0.97354
888	0.96197	0.96578	0.96748	0.96804	0.96980	0.97163	0.97357
889	0.96201	0.96582	0.96751	0.96807	0.96983	0.97166	0.97359
890	0.96204	0.96585	0.96755	0.96810	0.96986	0.97168	0.97361
891	0.96208	0.96589	0.96758	0.96813	0.96988	0.97171	0.97364
892	0.96212	0.96592	0.96761	0.96816	0.96991	0.97174	0.97366
893	0.96216	0.96595	0.96764	0.96819	0.96994	0.97176	0.97369
894	0.96219	0.96598	0.96767	0.96823	0.96997	0.97179	0.97371
895	0.96223	0.96602	0.96770	0.96825	0.97000	0.97182	0.97374
896	0.96227	0.96605	0.96773	0.96829	0.97003	0.97184	0.97376
897	0.96230	0.96608	0.96776	0.96832	0.97006	0.97187	0.97379
898	0.96234	0.96612	0.96779	0.96834	0.97009	0.97190	0.97381
899	0.96237	0.96615	0.96783	0.96838	0.97011	0.97192	0.97384
900	0.96241	0.96618	0.96786	0.96841	0.97014	0.97195	0.97386
901	0.96244	0.96622	0.96789	0.96844	0.97017	0.97198	0.97389
902	0.96248	0.96625	0.96792	0.96847	0.97020	0.97200	0.97391
903	0.96252	0.96628	0.96795	0.96850	0.97023	0.97203	0.97393
904	0.96256	0.96631	0.96798	0.96853	0.97026	0.97206	0.97396
905	0.96259	0.96635	0.96801	0.96856	0.97028	0.97208	0.97398
906	0.96263	0.96638	0.96804	0.96859	0.97031	0.97211	0.97401
907	0.96266	0.96641	0.96807	0.96862	0.97034	0.97214	0.97403
908	0.96270	0.96644	0.96810	0.96865	0.97037	0.97216	0.97405
909	0.96274	0.96647	0.96813	0.96868	0.97039	0.97219	0.97408
910	0.96278	0.96651	0.96816	0.96870	0.97042	0.97221	0.97410
911	0.96281	0.96654	0.96819	0.96873	0.97045	0.97224	0.97413
912	0.96285	0.96657	0.96822	0.96876	0.97048	0.97227	0.97415
913	0.96289	0.96660	0.96825	0.96879	0.97050	0.97229	0.97417
914	0.96292	0.96664	0.96828	0.96882	0.97053	0.97232	0.97420
915	0.96296	0.96667	0.96831	0.96885	0.97056	0.97234	0.97422
916	0.96300	0.96670	0.96834	0.96888	0.97059	0.97237	0.97424
917	0.96303	0.96673	0.96837	0.96891	0.97061	0.97239	0.97427
918	0.96308	0.96676	0.96840	0.96894	0.97064	0.97242	0.97429
919	0.96311	0.96679	0.96843	0.96897	0.97067	0.97244	0.97432
920	0.96314	0.96682	0.96846	0.96900	0.97070	0.97247	0.97434
921	0.96317	0.96686	0.96849	0.96903	0.97072	0.97249	0.97436
922	0.96321	0.96689	0.96852	0.96906	0.97075	0.97252	0.97439
923	0.96324	0.96692	0.96855	0.96908	0.97078	0.97255	0.97441
924	0.96328	0.96695	0.96858	0.96911	0.97081	0.97257	0.97443
925	0.96331	0.96698	0.96860	0.96914	0.97083	0.97260	0.97446
926	0.96335	0.96701	0.96863	0.96917	0.97086	0.97262	0.97448
927	0.96338	0.96704	0.96866	0.96920	0.97089	0.97265	0.97450
928	0.96341	0.96707	0.96869	0.96923	0.97091	0.97267	0.97453
929	0.96345	0.96711	0.96872	0.96926	0.97094	0.97269	0.97455
930	0.96348	0.96713	0.96875	0.96929	0.97097	0.97272	0.97457
931	0.96352	0.96717	0.96878	0.96931	0.97099	0.97275	0.97459
932	0.96355	0.96720	0.96881	0.96934	0.97102	0.97277	0.97462
933	0.96359	0.96723	0.96884	0.96937	0.97105	0.97279	0.97464
934	0.96362	0.96726	0.96887	0.96940	0.97107	0.97282	0.97466
935	0.96366	0.96729	0.96890	0.96943	0.97110	0.97284	0.97469
936	0.96369	0.96732	0.96892	0.96945	0.97113	0.97287	0.97471
937	0.96373	0.96735	0.96895	0.96948	0.97115	0.97289	0.97473
938	0.96376	0.96738	0.96898	0.96951	0.97118	0.97292	0.97475
939	0.96380	0.96741	0.96901	0.96954	0.97121	0.97294	0.97478
940	0.96383	0.96744	0.96904	0.96957	0.97123	0.97297	0.97480
941	0.96386	0.96747	0.96907	0.96960	0.97126	0.97299	0.97482
942	0.96390	0.96750	0.96910	0.96962	0.97128	0.97301	0.97484
943	0.96393	0.96753	0.96913	0.96965	0.97131	0.97304	0.97487
944	0.96396	0.96756	0.96915	0.96968	0.97134	0.97306	0.97489
945	0.96400	0.96759	0.96918	0.96971	0.97136	0.97309	0.97491
946	0.96403	0.96762	0.96921	0.96973	0.97139	0.97311	0.97493
947	0.96406	0.96765	0.96924	0.96976	0.97141	0.97314	0.97495
948	0.96410	0.96768	0.96927	0.96979	0.97144	0.97316	0.97498
949	0.96412	0.96771	0.96930	0.96982	0.97146	0.97318	0.97500
950	0.96416	0.96774	0.96932	0.96984	0.97149	0.97321	0.97502
951	0.96419	0.96777	0.96935	0.96987	0.97152	0.97323	0.97504
952	0.96422	0.96780	0.96938	0.96990	0.97154	0.97326	0.97507
953	0.96426	0.96783	0.96941	0.96992	0.97157	0.97328	0.97509
954	0.96429	0.96786	0.96944	0.96995	0.97159	0.97331	0.97511
955	0.96432	0.96788	0.96947	0.96998	0.97162	0.97333	0.97513
956	0.96436	0.96791	0.96949	0.97001	0.97164	0.97335	0.97515
957	0.96439	0.96794	0.96952	0.97003	0.97167	0.97337	0.97518

N	0.1%	0.5%	1%	1.25%	2.5%	5%	10%
958	0.96442	0.96797	0.96955	0.97006	0.97169	0.97340	0.97520
959	0.96446	0.96800	0.96957	0.97009	0.97172	0.97342	0.97522
960	0.96449	0.96803	0.96960	0.97012	0.97175	0.97345	0.97524
961	0.96453	0.96806	0.96963	0.97014	0.97177	0.97347	0.97526
962	0.96455	0.96809	0.96966	0.97017	0.97180	0.97349	0.97528
963	0.96458	0.96811	0.96968	0.97019	0.97182	0.97352	0.97531
964	0.96461	0.96814	0.96971	0.97022	0.97185	0.97354	0.97533
965	0.96465	0.96817	0.96974	0.97025	0.97187	0.97356	0.97535
966	0.96469	0.96820	0.96976	0.97027	0.97190	0.97359	0.97537
967	0.96472	0.96823	0.96979	0.97030	0.97192	0.97361	0.97539
968	0.96475	0.96826	0.96982	0.97033	0.97195	0.97363	0.97541
969	0.96478	0.96828	0.96984	0.97035	0.97197	0.97365	0.97543
970	0.96481	0.96831	0.96987	0.97038	0.97200	0.97368	0.97546
971	0.96484	0.96834	0.96990	0.97040	0.97202	0.97370	0.97548
972	0.96487	0.96837	0.96992	0.97043	0.97204	0.97372	0.97550
973	0.96490	0.96840	0.96995	0.97046	0.97207	0.97375	0.97552
974	0.96494	0.96843	0.96998	0.97048	0.97210	0.97377	0.97554
975	0.96497	0.96846	0.97000	0.97051	0.97212	0.97379	0.97556
976	0.96500	0.96848	0.97003	0.97054	0.97214	0.97382	0.97558
977	0.96503	0.96851	0.97005	0.97056	0.97217	0.97384	0.97560
978	0.96506	0.96854	0.97008	0.97059	0.97219	0.97386	0.97563
979	0.96509	0.96857	0.97011	0.97061	0.97222	0.97388	0.97565
980	0.96513	0.96859	0.97013	0.97064	0.97224	0.97391	0.97567
981	0.96516	0.96862	0.97016	0.97066	0.97226	0.97393	0.97569
982	0.96519	0.96865	0.97019	0.97069	0.97229	0.97395	0.97571
983	0.96522	0.96868	0.97021	0.97072	0.97231	0.97397	0.97573
984	0.96525	0.96870	0.97024	0.97074	0.97234	0.97400	0.97575
985	0.96528	0.96873	0.97027	0.97077	0.97236	0.97402	0.97577
986	0.96531	0.96876	0.97029	0.97080	0.97239	0.97404	0.97579
987	0.96534	0.96879	0.97032	0.97082	0.97241	0.97406	0.97581
988	0.96537	0.96881	0.97034	0.97085	0.97243	0.97409	0.97583
989	0.96540	0.96884	0.97037	0.97087	0.97246	0.97411	0.97585
990	0.96544	0.96887	0.97040	0.97090	0.97248	0.97413	0.97587
991	0.96547	0.96890	0.97042	0.97092	0.97251	0.97415	0.97589
992	0.96550	0.96892	0.97045	0.97095	0.97253	0.97418	0.97592
993	0.96553	0.96895	0.97048	0.97097	0.97255	0.97420	0.97594
994	0.96556	0.96898	0.97050	0.97100	0.97258	0.97422	0.97596
995	0.96559	0.96901	0.97053	0.97102	0.97260	0.97424	0.97598
996	0.96562	0.96904	0.97055	0.97105	0.97262	0.97426	0.97600
997	0.96565	0.96906	0.97058	0.97107	0.97265	0.97429	0.97602
998	0.96567	0.96909	0.97060	0.97110	0.97267	0.97431	0.97604
999	0.96570	0.96911	0.97063	0.97112	0.97269	0.97433	0.97606
1000	0.96574	0.96914	0.97065	0.97115	0.97272	0.97435	0.97608

Table 2 – *GD(HL)*. Critical values of upper significance levels for the Grubbs double outlier test, applicable in situations where the highest and lowest observations are simultaneously identified as outliers, denoted as *GD(HL)*.

N	0.1%	0.5%	1%	1.25%	2.5%	5%	10%
4	0.00000	0.00000	0.00002	0.00003	0.00011	0.00046	0.00189
5	0.00024	0.00122	0.00245	0.00308	0.00625	0.01277	0.02639
6	0.00293	0.00871	0.01398	0.01630	0.02630	0.04268	0.06985
7	0.01068	0.02435	0.03483	0.03912	0.05620	0.08110	0.11784
8	0.02380	0.04614	0.06155	0.06757	0.09045	0.12154	0.16425
9	0.04111	0.07154	0.09106	0.09845	0.12568	0.16096	0.20716
10	0.06127	0.09866	0.12138	0.12980	0.16014	0.19813	0.24622
11	0.08314	0.12620	0.15137	0.16055	0.19302	0.23267	0.28159
12	0.10576	0.15340	0.18036	0.19008	0.22398	0.26456	0.31363
13	0.12859	0.17982	0.20807	0.21814	0.25294	0.29395	0.34273
14	0.15125	0.20518	0.23436	0.24467	0.27998	0.32103	0.36925
15	0.17339	0.22942	0.25919	0.26964	0.30516	0.34602	0.39350
16	0.19489	0.25245	0.28260	0.29311	0.32863	0.36913	0.41575
17	0.21568	0.27434	0.30467	0.31518	0.35053	0.39054	0.43624
18	0.23568	0.29508	0.32543	0.33591	0.37100	0.41043	0.45518
19	0.25488	0.31470	0.34499	0.35541	0.39015	0.42896	0.47273
20	0.27331	0.33331	0.36344	0.37378	0.40810	0.44624	0.48906
21	0.29091	0.35093	0.38085	0.39108	0.42495	0.46242	0.50428
22	0.30776	0.36765	0.39729	0.40741	0.44080	0.47758	0.51851
23	0.32389	0.38352	0.41286	0.42285	0.45573	0.49183	0.53185
24	0.33928	0.39858	0.42760	0.43745	0.46983	0.50524	0.54437
25	0.35402	0.41291	0.44157	0.45128	0.48315	0.51788	0.55616
26	0.36816	0.42654	0.45484	0.46441	0.49575	0.52983	0.56728
27	0.38169	0.43952	0.46745	0.47688	0.50771	0.54114	0.57779

N	0.1%	0.5%	1%	1.25%	2.5%	5%	10%
28	0.39465	0.45192	0.47947	0.48876	0.51906	0.55187	0.58774
29	0.40705	0.46375	0.49091	0.50006	0.52985	0.56204	0.59717
30	0.41896	0.47505	0.50182	0.51083	0.54013	0.57172	0.60613
31	0.43040	0.48585	0.51224	0.52110	0.54993	0.58093	0.61465
32	0.44140	0.49619	0.52221	0.53094	0.55928	0.58971	0.62276
33	0.45196	0.50611	0.53175	0.54034	0.56821	0.59809	0.63050
34	0.46209	0.51561	0.54088	0.54934	0.57675	0.60610	0.63789
35	0.47188	0.52473	0.54963	0.55796	0.58493	0.61376	0.64495
36	0.48128	0.53348	0.55803	0.56624	0.59277	0.62110	0.65172
37	0.49034	0.54190	0.56610	0.57417	0.60028	0.62814	0.65819
38	0.49909	0.55000	0.57385	0.58180	0.60750	0.63488	0.66441
39	0.50750	0.55779	0.58130	0.58915	0.61444	0.64137	0.67037
40	0.51563	0.56530	0.58848	0.59621	0.62111	0.64760	0.67610
41	0.52351	0.57254	0.59540	0.60300	0.62753	0.65360	0.68161
42	0.53108	0.57953	0.60206	0.60957	0.63373	0.65937	0.68691
43	0.53843	0.58627	0.60851	0.61590	0.63970	0.66494	0.69202
44	0.54554	0.59280	0.61472	0.62201	0.64546	0.67030	0.69695
45	0.55241	0.59909	0.62072	0.62791	0.65102	0.67548	0.70170
46	0.55910	0.60518	0.62653	0.63362	0.65639	0.68048	0.70629
47	0.56554	0.61108	0.63214	0.63913	0.66158	0.68532	0.71072
48	0.57180	0.61678	0.63757	0.64447	0.66660	0.68999	0.71501
49	0.57786	0.62232	0.64283	0.64963	0.67146	0.69451	0.71915
50	0.58375	0.62768	0.64793	0.65464	0.67617	0.69889	0.72316
51	0.58946	0.63287	0.65287	0.65949	0.68072	0.70313	0.72705
52	0.59500	0.63792	0.65766	0.66420	0.68515	0.70724	0.73082
53	0.60040	0.64283	0.66231	0.66876	0.68944	0.71123	0.73447
54	0.60564	0.64758	0.66682	0.67320	0.69360	0.71510	0.73801
55	0.61075	0.65221	0.67121	0.67751	0.69765	0.71885	0.74145
56	0.61570	0.65670	0.67548	0.68169	0.70157	0.72250	0.74479
57	0.62052	0.66106	0.67962	0.68576	0.70539	0.72604	0.74804
58	0.62522	0.66532	0.68366	0.68972	0.70910	0.72949	0.75119
59	0.62980	0.66945	0.68757	0.69356	0.71271	0.73284	0.75426
60	0.63426	0.67347	0.69139	0.69730	0.71622	0.73610	0.75724
61	0.63862	0.67740	0.69510	0.70095	0.71964	0.73927	0.76015
62	0.64288	0.68123	0.69873	0.70451	0.72297	0.74236	0.76298
63	0.64701	0.68495	0.70225	0.70797	0.72622	0.74537	0.76573
64	0.65104	0.68859	0.70569	0.71134	0.72938	0.74831	0.76842
65	0.65498	0.69213	0.70905	0.71463	0.73247	0.75117	0.77103
66	0.65883	0.69559	0.71232	0.71784	0.73547	0.75396	0.77359
67	0.66259	0.69896	0.71551	0.72098	0.73841	0.75668	0.77607
68	0.66626	0.70226	0.71863	0.72404	0.74127	0.75933	0.77850
69	0.66983	0.70547	0.72168	0.72702	0.74407	0.76193	0.78088
70	0.67334	0.70861	0.72465	0.72994	0.74679	0.76446	0.78319
71	0.67676	0.71169	0.72756	0.73279	0.74946	0.76693	0.78545
72	0.68010	0.71469	0.73040	0.73557	0.75207	0.76935	0.78766
73	0.68338	0.71763	0.73317	0.73829	0.75462	0.77171	0.78982
74	0.68657	0.72050	0.73588	0.74095	0.75711	0.77402	0.79193
75	0.68971	0.72331	0.73854	0.74356	0.75955	0.77627	0.79400
76	0.69279	0.72606	0.74114	0.74610	0.76193	0.77848	0.79602
77	0.69578	0.72875	0.74368	0.74860	0.76427	0.78064	0.79800
78	0.69872	0.73139	0.74617	0.75104	0.76655	0.78276	0.79993
79	0.70162	0.73397	0.74861	0.75342	0.76879	0.78483	0.80183
80	0.70444	0.73649	0.75099	0.75577	0.77098	0.78686	0.80368
81	0.70721	0.73897	0.75333	0.75806	0.77312	0.78885	0.80550
82	0.70992	0.74140	0.75563	0.76031	0.77522	0.79079	0.80728
83	0.71258	0.74377	0.75787	0.76251	0.77728	0.79270	0.80902
84	0.71518	0.74610	0.76007	0.76467	0.77929	0.79457	0.81073
85	0.71773	0.74839	0.76223	0.76678	0.78127	0.79640	0.81241
86	0.72025	0.75063	0.76435	0.76886	0.78321	0.79820	0.81405
87	0.72271	0.75283	0.76642	0.77089	0.78512	0.79996	0.81566
88	0.72512	0.75498	0.76846	0.77289	0.78698	0.80169	0.81724
89	0.72750	0.75710	0.77046	0.77485	0.78882	0.80339	0.81879
90	0.72982	0.75918	0.77242	0.77677	0.79061	0.80505	0.82031
91	0.73211	0.76122	0.77434	0.77866	0.79238	0.80668	0.82181
92	0.73435	0.76322	0.77623	0.78051	0.79411	0.80829	0.82328
93	0.73657	0.76519	0.77809	0.78233	0.79581	0.80987	0.82472
94	0.73873	0.76712	0.77991	0.78411	0.79748	0.81141	0.82613
95	0.74085	0.76902	0.78170	0.78587	0.79912	0.81293	0.82752
96	0.74294	0.77088	0.78346	0.78759	0.80073	0.81443	0.82889
97	0.74499	0.77272	0.78519	0.78929	0.80231	0.81589	0.83023
98	0.74702	0.77452	0.78689	0.79096	0.80387	0.81734	0.83155
99	0.74901	0.77629	0.78856	0.79259	0.80540	0.81875	0.83285
100	0.75096	0.77803	0.79020	0.79421	0.80691	0.82015	0.83412
101	0.75289	0.77974	0.79182	0.79579	0.80839	0.82151	0.83537

N	0.1%	0.5%	1%	1.25%	2.5%	5%	10%
102	0.75478	0.78142	0.79341	0.79734	0.80984	0.82286	0.83661
103	0.75664	0.78308	0.79497	0.79887	0.81127	0.82419	0.83782
104	0.75846	0.78471	0.79651	0.80038	0.81268	0.82549	0.83901
105	0.76027	0.78632	0.79802	0.80186	0.81407	0.82678	0.84019
106	0.76203	0.78790	0.79951	0.80332	0.81543	0.82804	0.84134
107	0.76379	0.78945	0.80097	0.80476	0.81677	0.82928	0.84248
108	0.76551	0.79098	0.80241	0.80617	0.81809	0.83050	0.84360
109	0.76719	0.79248	0.80383	0.80756	0.81939	0.83171	0.84470
110	0.76886	0.79396	0.80523	0.80893	0.82067	0.83289	0.84578
111	0.77051	0.79542	0.80661	0.81028	0.82193	0.83406	0.84685
112	0.77212	0.79686	0.80796	0.81160	0.82317	0.83521	0.84790
113	0.77371	0.79828	0.80929	0.81291	0.82439	0.83634	0.84894
114	0.77527	0.79967	0.81061	0.81420	0.82560	0.83746	0.84996
115	0.77682	0.80105	0.81191	0.81547	0.82678	0.83855	0.85096
116	0.77834	0.80240	0.81318	0.81672	0.82795	0.83964	0.85195
117	0.77984	0.80373	0.81444	0.81795	0.82910	0.84070	0.85293
118	0.78132	0.80505	0.81568	0.81917	0.83024	0.84176	0.85389
119	0.78278	0.80634	0.81690	0.82036	0.83136	0.84279	0.85484
120	0.78422	0.80762	0.81811	0.82155	0.83246	0.84381	0.85578
121	0.78564	0.80888	0.81929	0.82271	0.83355	0.84482	0.85670
122	0.78703	0.81012	0.82047	0.82386	0.83462	0.84581	0.85761
123	0.78841	0.81135	0.82162	0.82499	0.83568	0.84679	0.85850
124	0.78977	0.81256	0.82276	0.82610	0.83672	0.84776	0.85939
125	0.79111	0.81375	0.82388	0.82720	0.83775	0.84871	0.86026
126	0.79244	0.81492	0.82499	0.82829	0.83876	0.84965	0.86112
127	0.79374	0.81608	0.82608	0.82936	0.83976	0.85058	0.86197
128	0.79503	0.81723	0.82716	0.83042	0.84075	0.85149	0.86280
129	0.79629	0.81835	0.82822	0.83146	0.84172	0.85239	0.86363
130	0.79755	0.81947	0.82927	0.83249	0.84269	0.85328	0.86444
131	0.79879	0.82056	0.83031	0.83350	0.84363	0.85416	0.86525
132	0.80000	0.82165	0.83133	0.83450	0.84457	0.85503	0.86604
133	0.80121	0.82272	0.83234	0.83549	0.84550	0.85589	0.86683
134	0.80240	0.82377	0.83334	0.83647	0.84641	0.85673	0.86760
135	0.80358	0.82482	0.83432	0.83743	0.84731	0.85757	0.86837
136	0.80474	0.82585	0.83529	0.83839	0.84820	0.85839	0.86912
137	0.80588	0.82687	0.83625	0.83932	0.84908	0.85920	0.86987
138	0.80702	0.82787	0.83720	0.84025	0.84994	0.86001	0.87060
139	0.80813	0.82886	0.83813	0.84117	0.85080	0.86080	0.87133
140	0.80924	0.82984	0.83906	0.84208	0.85165	0.86158	0.87205
141	0.81032	0.83081	0.83997	0.84297	0.85248	0.86236	0.87276
142	0.81140	0.83177	0.84087	0.84385	0.85331	0.86312	0.87346
143	0.81246	0.83271	0.84176	0.84473	0.85412	0.86388	0.87415
144	0.81352	0.83365	0.84264	0.84559	0.85493	0.86462	0.87483
145	0.81455	0.83457	0.84351	0.84644	0.85572	0.86536	0.87551
146	0.81558	0.83548	0.84437	0.84728	0.85651	0.86609	0.87617
147	0.81660	0.83638	0.84522	0.84811	0.85729	0.86681	0.87683
148	0.81760	0.83727	0.84606	0.84893	0.85806	0.86752	0.87749
149	0.81859	0.83815	0.84688	0.84975	0.85881	0.86823	0.87813
150	0.81957	0.83902	0.84770	0.85055	0.85956	0.86892	0.87877
151	0.82054	0.83988	0.84851	0.85134	0.86031	0.86961	0.87940
152	0.82149	0.84073	0.84931	0.85213	0.86104	0.87029	0.88002
153	0.82245	0.84157	0.85011	0.85290	0.86177	0.87096	0.88064
154	0.82337	0.84240	0.85089	0.85367	0.86248	0.87163	0.88125
155	0.82430	0.84322	0.85166	0.85443	0.86319	0.87228	0.88185
156	0.82522	0.84404	0.85243	0.85518	0.86389	0.87293	0.88244
157	0.82612	0.84484	0.85319	0.85592	0.86459	0.87358	0.88303
158	0.82702	0.84563	0.85394	0.85666	0.86527	0.87421	0.88362
159	0.82790	0.84642	0.85468	0.85738	0.86595	0.87484	0.88419
160	0.82878	0.84720	0.85541	0.85810	0.86662	0.87546	0.88476
161	0.82965	0.84797	0.85613	0.85881	0.86729	0.87608	0.88533
162	0.83050	0.84873	0.85685	0.85951	0.86794	0.87669	0.88588
163	0.83135	0.84948	0.85756	0.86021	0.86859	0.87729	0.88644
164	0.83219	0.85022	0.85826	0.86089	0.86923	0.87788	0.88698
165	0.83302	0.85096	0.85896	0.86157	0.86987	0.87847	0.88752
166	0.83384	0.85169	0.85964	0.86225	0.87050	0.87906	0.88806
167	0.83465	0.85241	0.86032	0.86291	0.87112	0.87963	0.88859
168	0.83546	0.85312	0.86100	0.86357	0.87174	0.88020	0.88911
169	0.83626	0.85383	0.86166	0.86423	0.87235	0.88077	0.88963
170	0.83704	0.85453	0.86232	0.86487	0.87295	0.88133	0.89014
171	0.83782	0.85522	0.86297	0.86551	0.87355	0.88188	0.89065
172	0.83860	0.85591	0.86362	0.86614	0.87414	0.88243	0.89115
173	0.83936	0.85658	0.86426	0.86677	0.87472	0.88298	0.89165
174	0.84012	0.85725	0.86489	0.86739	0.87530	0.88351	0.89214
175	0.84086	0.85792	0.86552	0.86800	0.87588	0.88405	0.89263

N	0.1%	0.5%	1%	1.25%	2.5%	5%	10%
176	0.84160	0.85858	0.86614	0.86861	0.87645	0.88457	0.89312
177	0.84234	0.85923	0.86675	0.86921	0.87701	0.88509	0.89359
178	0.84307	0.85987	0.86736	0.86981	0.87757	0.88561	0.89407
179	0.84379	0.86051	0.86796	0.87040	0.87812	0.88612	0.89454
180	0.84450	0.86114	0.86856	0.87098	0.87866	0.88663	0.89500
181	0.84521	0.86177	0.86915	0.87156	0.87921	0.88713	0.89546
182	0.84591	0.86239	0.86973	0.87213	0.87974	0.88763	0.89592
183	0.84660	0.86301	0.87031	0.87270	0.88027	0.88812	0.89637
184	0.84728	0.86361	0.87088	0.87326	0.88080	0.88861	0.89682
185	0.84796	0.86422	0.87145	0.87382	0.88132	0.88909	0.89726
186	0.84863	0.86481	0.87202	0.87437	0.88183	0.88957	0.89770
187	0.84930	0.86541	0.87257	0.87492	0.88234	0.89004	0.89813
188	0.84996	0.86599	0.87313	0.87546	0.88285	0.89051	0.89857
189	0.85062	0.86657	0.87367	0.87600	0.88335	0.89098	0.89899
190	0.85126	0.86714	0.87421	0.87653	0.88385	0.89144	0.89941
191	0.85190	0.86772	0.87475	0.87705	0.88434	0.89189	0.89983
192	0.85254	0.86828	0.87528	0.87758	0.88483	0.89235	0.90025
193	0.85317	0.86884	0.87581	0.87809	0.88531	0.89279	0.90066
194	0.85380	0.86940	0.87633	0.87860	0.88579	0.89324	0.90107
195	0.85442	0.86994	0.87685	0.87911	0.88626	0.89368	0.90147
196	0.85503	0.87049	0.87737	0.87961	0.88673	0.89412	0.90187
197	0.85564	0.87103	0.87787	0.88011	0.88720	0.89455	0.90227
198	0.85624	0.87156	0.87838	0.88061	0.88766	0.89498	0.90266
199	0.85684	0.87209	0.87888	0.88110	0.88812	0.89540	0.90305
200	0.85743	0.87262	0.87937	0.88158	0.88858	0.89582	0.90344
201	0.85791	0.87310	0.87981	0.88201	0.88900	0.89623	0.90383
202	0.85849	0.87361	0.88029	0.88249	0.88944	0.89664	0.90421
203	0.85909	0.87413	0.88077	0.88296	0.88988	0.89705	0.90458
204	0.85963	0.87465	0.88126	0.88344	0.89032	0.89746	0.90496
205	0.86021	0.87515	0.88173	0.88390	0.89075	0.89786	0.90532
206	0.86079	0.87566	0.88221	0.88438	0.89118	0.89827	0.90569
207	0.86135	0.87615	0.88268	0.88483	0.89161	0.89866	0.90606
208	0.86192	0.87664	0.88313	0.88528	0.89203	0.89905	0.90642
209	0.86250	0.87712	0.88360	0.88574	0.89246	0.89945	0.90677
210	0.86302	0.87762	0.88404	0.88618	0.89287	0.89983	0.90713
211	0.86357	0.87810	0.88450	0.88663	0.89327	0.90021	0.90748
212	0.86412	0.87857	0.88495	0.88706	0.89369	0.90059	0.90783
213	0.86463	0.87903	0.88538	0.88751	0.89410	0.90097	0.90817
214	0.86519	0.87949	0.88583	0.88793	0.89451	0.90134	0.90852
215	0.86570	0.87997	0.88627	0.88836	0.89491	0.90171	0.90886
216	0.86622	0.88042	0.88671	0.88878	0.89530	0.90208	0.90919
217	0.86673	0.88086	0.88714	0.88921	0.89570	0.90244	0.90953
218	0.86725	0.88130	0.88757	0.88962	0.89609	0.90281	0.90986
219	0.86774	0.88176	0.88798	0.89003	0.89648	0.90316	0.91020
220	0.86824	0.88220	0.88840	0.89045	0.89686	0.90352	0.91052
221	0.86874	0.88264	0.88880	0.89085	0.89724	0.90387	0.91084
222	0.86922	0.88308	0.88922	0.89125	0.89762	0.90422	0.91117
223	0.86970	0.88351	0.88963	0.89166	0.89800	0.90457	0.91149
224	0.87020	0.88394	0.89003	0.89205	0.89837	0.90492	0.91180
225	0.87069	0.88438	0.89045	0.89245	0.89873	0.90526	0.91212
226	0.87116	0.88479	0.89084	0.89284	0.89911	0.90560	0.91243
227	0.87168	0.88521	0.89124	0.89323	0.89946	0.90594	0.91274
228	0.87216	0.88563	0.89164	0.89361	0.89982	0.90628	0.91305
229	0.87261	0.88604	0.89203	0.89399	0.90018	0.90661	0.91336
230	0.87308	0.88644	0.89241	0.89437	0.90053	0.90694	0.91366
231	0.87350	0.88685	0.89279	0.89475	0.90088	0.90726	0.91396
232	0.87392	0.88726	0.89318	0.89512	0.90123	0.90759	0.91425
233	0.87439	0.88764	0.89355	0.89548	0.90158	0.90791	0.91455
234	0.87480	0.88804	0.89393	0.89585	0.90193	0.90823	0.91484
235	0.87525	0.88843	0.89430	0.89621	0.90226	0.90854	0.91513
236	0.87569	0.88882	0.89467	0.89657	0.90261	0.90886	0.91543
237	0.87612	0.88919	0.89503	0.89693	0.90294	0.90917	0.91571
238	0.87654	0.88957	0.89538	0.89730	0.90328	0.90948	0.91600
239	0.87694	0.88997	0.89575	0.89765	0.90361	0.90979	0.91628
240	0.87740	0.89035	0.89610	0.89800	0.90393	0.91010	0.91656
241	0.87782	0.89073	0.89646	0.89836	0.90426	0.91040	0.91684
242	0.87822	0.89111	0.89683	0.89870	0.90458	0.91070	0.91712
243	0.87866	0.89148	0.89718	0.89904	0.90490	0.91100	0.91739
244	0.87912	0.89185	0.89752	0.89938	0.90522	0.91129	0.91767
245	0.87949	0.89221	0.89787	0.89972	0.90554	0.91158	0.91794
246	0.87994	0.89257	0.89822	0.90006	0.90586	0.91188	0.91821
247	0.88034	0.89294	0.89856	0.90040	0.90617	0.91217	0.91848
248	0.88074	0.89328	0.89889	0.90074	0.90648	0.91246	0.91874
249	0.88117	0.89362	0.89923	0.90105	0.90679	0.91274	0.91900

N	0.1%	0.5%	1%	1.25%	2.5%	5%	10%
250	0.88157	0.89398	0.89956	0.90138	0.90710	0.91303	0.91927
251	0.88197	0.89432	0.89989	0.90171	0.90740	0.91332	0.91953
252	0.88236	0.89468	0.90022	0.90203	0.90771	0.91360	0.91979
253	0.88275	0.89502	0.90054	0.90235	0.90800	0.91387	0.92004
254	0.88309	0.89535	0.90087	0.90266	0.90830	0.91415	0.92030
255	0.88350	0.89570	0.90119	0.90298	0.90859	0.91443	0.92055
256	0.88388	0.89603	0.90152	0.90329	0.90889	0.91470	0.92080
257	0.88425	0.89637	0.90185	0.90360	0.90918	0.91497	0.92105
258	0.88462	0.89670	0.90216	0.90391	0.90947	0.91524	0.92130
259	0.88501	0.89705	0.90247	0.90422	0.90976	0.91550	0.92155
260	0.88537	0.89736	0.90278	0.90452	0.91004	0.91577	0.92179
261	0.88574	0.89768	0.90309	0.90482	0.91032	0.91603	0.92203
262	0.88612	0.89800	0.90339	0.90513	0.91061	0.91629	0.92227
263	0.88648	0.89833	0.90369	0.90543	0.91089	0.91655	0.92251
264	0.88683	0.89865	0.90400	0.90572	0.91117	0.91681	0.92274
265	0.88720	0.89898	0.90430	0.90601	0.91144	0.91707	0.92298
266	0.88757	0.89929	0.90460	0.90630	0.91172	0.91733	0.92321
267	0.88792	0.89962	0.90489	0.90659	0.91199	0.91757	0.92345
268	0.88824	0.89993	0.90519	0.90689	0.91226	0.91783	0.92368
269	0.88861	0.90024	0.90548	0.90718	0.91253	0.91807	0.92391
270	0.88897	0.90054	0.90577	0.90746	0.91280	0.91833	0.92414
271	0.88932	0.90085	0.90607	0.90775	0.91307	0.91858	0.92437
272	0.88965	0.90115	0.90635	0.90802	0.91333	0.91882	0.92459
273	0.88997	0.90146	0.90663	0.90830	0.91359	0.91906	0.92481
274	0.89035	0.90177	0.90691	0.90858	0.91385	0.91930	0.92504
275	0.89068	0.90206	0.90719	0.90885	0.91410	0.91954	0.92525
276	0.89103	0.90236	0.90747	0.90913	0.91436	0.91978	0.92547
277	0.89134	0.90266	0.90774	0.90940	0.91462	0.92002	0.92569
278	0.89168	0.90296	0.90802	0.90967	0.91488	0.92025	0.92591
279	0.89203	0.90326	0.90829	0.90994	0.91513	0.92049	0.92613
280	0.89233	0.90353	0.90857	0.91021	0.91538	0.92072	0.92634
281	0.89267	0.90382	0.90884	0.91047	0.91563	0.92095	0.92655
282	0.89300	0.90412	0.90911	0.91073	0.91588	0.92118	0.92677
283	0.89333	0.90439	0.90937	0.91100	0.91613	0.92141	0.92698
284	0.89361	0.90469	0.90964	0.91126	0.91636	0.92164	0.92718
285	0.89390	0.90496	0.90991	0.91152	0.91661	0.92186	0.92739
286	0.89426	0.90523	0.91018	0.91177	0.91686	0.92209	0.92760
287	0.89454	0.90551	0.91043	0.91203	0.91709	0.92231	0.92780
288	0.89484	0.90578	0.91069	0.91229	0.91733	0.92253	0.92801
289	0.89513	0.90605	0.91095	0.91253	0.91756	0.92275	0.92821
290	0.89543	0.90632	0.91121	0.91279	0.91780	0.92297	0.92841
291	0.89573	0.90659	0.91146	0.91304	0.91803	0.92319	0.92861
292	0.89601	0.90687	0.91171	0.91329	0.91827	0.92340	0.92880
293	0.89630	0.90714	0.91197	0.91354	0.91850	0.92362	0.92900
294	0.89658	0.90741	0.91222	0.91378	0.91873	0.92383	0.92920
295	0.89688	0.90767	0.91247	0.91402	0.91896	0.92404	0.92939
296	0.89716	0.90793	0.91271	0.91426	0.91918	0.92425	0.92959
297	0.89746	0.90820	0.91296	0.91451	0.91941	0.92446	0.92978
298	0.89777	0.90846	0.91321	0.91474	0.91963	0.92467	0.92997
299	0.89804	0.90872	0.91345	0.91499	0.91986	0.92488	0.93016
300	0.89833	0.90897	0.91369	0.91522	0.92008	0.92508	0.93035
301	0.89863	0.90923	0.91392	0.91546	0.92030	0.92529	0.93054
302	0.89892	0.90949	0.91416	0.91569	0.92052	0.92549	0.93073
303	0.89924	0.90974	0.91440	0.91591	0.92073	0.92569	0.93091
304	0.89951	0.90999	0.91463	0.91614	0.92095	0.92589	0.93110
305	0.89978	0.91024	0.91487	0.91637	0.92116	0.92609	0.93128
306	0.90005	0.91049	0.91510	0.91660	0.92138	0.92629	0.93146
307	0.90033	0.91073	0.91533	0.91683	0.92159	0.92649	0.93165
308	0.90060	0.91098	0.91556	0.91705	0.92179	0.92668	0.93183
309	0.90087	0.91123	0.91578	0.91728	0.92201	0.92688	0.93201
310	0.90116	0.91146	0.91601	0.91750	0.92221	0.92707	0.93219
311	0.90143	0.91171	0.91624	0.91773	0.92243	0.92727	0.93237
312	0.90168	0.91195	0.91646	0.91794	0.92263	0.92746	0.93254
313	0.90195	0.91219	0.91669	0.91816	0.92283	0.92765	0.93272
314	0.90221	0.91242	0.91691	0.91838	0.92304	0.92784	0.93289
315	0.90249	0.91266	0.91712	0.91860	0.92324	0.92803	0.93306
316	0.90274	0.91289	0.91734	0.91881	0.92344	0.92822	0.93324
317	0.90302	0.91312	0.91755	0.91902	0.92364	0.92840	0.93341
318	0.90326	0.91336	0.91777	0.91923	0.92383	0.92859	0.93358
319	0.90352	0.91358	0.91799	0.91944	0.92403	0.92877	0.93375
320	0.90379	0.91380	0.91821	0.91965	0.92423	0.92896	0.93392
321	0.90405	0.91404	0.91842	0.91986	0.92443	0.92914	0.93409
322	0.90431	0.91427	0.91863	0.92007	0.92462	0.92932	0.93426
323	0.90457	0.91449	0.91884	0.92028	0.92481	0.92950	0.93442

N	0.1%	0.5%	1%	1.25%	2.5%	5%	10%
324	0.90483	0.91470	0.91906	0.92049	0.92500	0.92968	0.93459
325	0.90504	0.91493	0.91926	0.92069	0.92520	0.92986	0.93475
326	0.90529	0.91515	0.91947	0.92089	0.92539	0.93004	0.93492
327	0.90555	0.91537	0.91968	0.92110	0.92558	0.93021	0.93508
328	0.90581	0.91558	0.91988	0.92130	0.92577	0.93039	0.93524
329	0.90604	0.91579	0.92008	0.92150	0.92596	0.93056	0.93540
330	0.90627	0.91600	0.92028	0.92170	0.92614	0.93074	0.93556
331	0.90652	0.91622	0.92049	0.92189	0.92633	0.93091	0.93572
332	0.90678	0.91644	0.92069	0.92209	0.92651	0.93108	0.93587
333	0.90702	0.91665	0.92089	0.92228	0.92669	0.93125	0.93603
334	0.90724	0.91686	0.92110	0.92248	0.92688	0.93142	0.93619
335	0.90751	0.91707	0.92129	0.92267	0.92706	0.93159	0.93635
336	0.90773	0.91727	0.92149	0.92287	0.92723	0.93176	0.93650
337	0.90798	0.91749	0.92169	0.92306	0.92742	0.93193	0.93666
338	0.90823	0.91770	0.92188	0.92325	0.92759	0.93209	0.93681
339	0.90845	0.91790	0.92208	0.92344	0.92777	0.93226	0.93696
340	0.90868	0.91811	0.92227	0.92363	0.92795	0.93242	0.93711
341	0.90890	0.91831	0.92246	0.92382	0.92812	0.93259	0.93726
342	0.90913	0.91851	0.92265	0.92401	0.92830	0.93275	0.93742
343	0.90936	0.91871	0.92284	0.92419	0.92848	0.93291	0.93756
344	0.90958	0.91890	0.92303	0.92438	0.92865	0.93307	0.93772
345	0.90980	0.91912	0.92321	0.92457	0.92882	0.93324	0.93786
346	0.91001	0.91932	0.92340	0.92475	0.92900	0.93340	0.93801
347	0.91021	0.91951	0.92359	0.92494	0.92917	0.93355	0.93816
348	0.91046	0.91970	0.92378	0.92512	0.92934	0.93371	0.93830
349	0.91068	0.91990	0.92396	0.92530	0.92950	0.93387	0.93845
350	0.91090	0.92009	0.92414	0.92547	0.92968	0.93402	0.93859
351	0.91113	0.92029	0.92433	0.92566	0.92984	0.93418	0.93873
352	0.91134	0.92048	0.92451	0.92583	0.93001	0.93433	0.93887
353	0.91154	0.92067	0.92469	0.92601	0.93017	0.93449	0.93902
354	0.91174	0.92086	0.92487	0.92619	0.93034	0.93464	0.93916
355	0.91196	0.92106	0.92504	0.92636	0.93050	0.93479	0.93930
356	0.91219	0.92124	0.92522	0.92654	0.93067	0.93494	0.93944
357	0.91240	0.92142	0.92540	0.92671	0.93083	0.93510	0.93958
358	0.91261	0.92161	0.92558	0.92688	0.93099	0.93524	0.93972
359	0.91281	0.92179	0.92576	0.92705	0.93115	0.93539	0.93985
360	0.91301	0.92198	0.92593	0.92723	0.93131	0.93554	0.93999
361	0.91321	0.92216	0.92610	0.92740	0.93147	0.93569	0.94013
362	0.91342	0.92235	0.92628	0.92757	0.93163	0.93584	0.94026
363	0.91362	0.92254	0.92645	0.92773	0.93179	0.93598	0.94040
364	0.91383	0.92271	0.92662	0.92790	0.93194	0.93613	0.94053
365	0.91403	0.92290	0.92679	0.92806	0.93210	0.93627	0.94067
366	0.91423	0.92307	0.92696	0.92823	0.93225	0.93642	0.94080
367	0.91442	0.92325	0.92713	0.92840	0.93241	0.93656	0.94093
368	0.91459	0.92343	0.92728	0.92856	0.93256	0.93670	0.94107
369	0.91478	0.92360	0.92745	0.92872	0.93271	0.93684	0.94120
370	0.91498	0.92377	0.92762	0.92888	0.93287	0.93699	0.94133
371	0.91519	0.92395	0.92778	0.92904	0.93302	0.93713	0.94146
372	0.91538	0.92413	0.92795	0.92921	0.93317	0.93727	0.94159
373	0.91558	0.92430	0.92811	0.92936	0.93332	0.93741	0.94172
374	0.91579	0.92446	0.92827	0.92952	0.93347	0.93755	0.94184
375	0.91598	0.92464	0.92844	0.92968	0.93362	0.93768	0.94197
376	0.91615	0.92480	0.92859	0.92984	0.93376	0.93782	0.94210
377	0.91636	0.92497	0.92876	0.92999	0.93391	0.93796	0.94222
378	0.91656	0.92514	0.92892	0.93015	0.93405	0.93809	0.94235
379	0.91673	0.92531	0.92907	0.93030	0.93420	0.93823	0.94248
380	0.91693	0.92548	0.92924	0.93046	0.93434	0.93837	0.94260
381	0.91713	0.92564	0.92939	0.93061	0.93449	0.93850	0.94272
382	0.91730	0.92580	0.92955	0.93076	0.93463	0.93863	0.94285
383	0.91749	0.92597	0.92971	0.93092	0.93478	0.93877	0.94297
384	0.91770	0.92613	0.92986	0.93107	0.93492	0.93890	0.94309
385	0.91787	0.92629	0.93002	0.93122	0.93506	0.93903	0.94321
386	0.91803	0.92646	0.93017	0.93137	0.93520	0.93916	0.94333
387	0.91822	0.92662	0.93032	0.93153	0.93534	0.93929	0.94345
388	0.91840	0.92677	0.93047	0.93168	0.93548	0.93942	0.94358
389	0.91858	0.92693	0.93063	0.93183	0.93562	0.93955	0.94369
390	0.91875	0.92710	0.93078	0.93197	0.93576	0.93968	0.94381
391	0.91892	0.92725	0.93092	0.93212	0.93590	0.93981	0.94393
392	0.91912	0.92741	0.93107	0.93227	0.93603	0.93993	0.94405
393	0.91929	0.92756	0.93122	0.93241	0.93617	0.94006	0.94416
394	0.91947	0.92772	0.93137	0.93256	0.93631	0.94019	0.94428
395	0.91965	0.92787	0.93152	0.93270	0.93644	0.94032	0.94440
396	0.91982	0.92803	0.93166	0.93284	0.93658	0.94044	0.94451
397	0.91999	0.92818	0.93181	0.93299	0.93671	0.94057	0.94463

N	0.1%	0.5%	1%	1.25%	2.5%	5%	10%
398	0.92016	0.92834	0.93196	0.93313	0.93684	0.94069	0.94474
399	0.92033	0.92848	0.93210	0.93327	0.93697	0.94081	0.94485
400	0.92048	0.92863	0.93224	0.93341	0.93711	0.94094	0.94497
401	0.92065	0.92878	0.93238	0.93355	0.93724	0.94106	0.94508
402	0.92082	0.92894	0.93253	0.93369	0.93737	0.94118	0.94519
403	0.92100	0.92909	0.93267	0.93383	0.93750	0.94131	0.94531
404	0.92117	0.92924	0.93281	0.93397	0.93763	0.94143	0.94542
405	0.92132	0.92938	0.93295	0.93411	0.93776	0.94155	0.94553
406	0.92150	0.92953	0.93309	0.93424	0.93789	0.94167	0.94564
407	0.92167	0.92968	0.93323	0.93438	0.93802	0.94179	0.94575
408	0.92184	0.92982	0.93336	0.93452	0.93815	0.94191	0.94586
409	0.92200	0.92997	0.93350	0.93465	0.93827	0.94202	0.94597
410	0.92217	0.93012	0.93364	0.93479	0.93840	0.94214	0.94608
411	0.92234	0.93026	0.93378	0.93492	0.93852	0.94226	0.94619
412	0.92250	0.93040	0.93391	0.93506	0.93865	0.94237	0.94629
413	0.92266	0.93055	0.93405	0.93519	0.93877	0.94249	0.94640
414	0.92283	0.93070	0.93418	0.93532	0.93890	0.94261	0.94651
415	0.92298	0.93084	0.93432	0.93545	0.93902	0.94272	0.94661
416	0.92314	0.93098	0.93446	0.93558	0.93915	0.94284	0.94672
417	0.92329	0.93112	0.93459	0.93571	0.93927	0.94295	0.94683
418	0.92346	0.93126	0.93472	0.93585	0.93939	0.94306	0.94693
419	0.92361	0.93140	0.93485	0.93598	0.93951	0.94318	0.94704
420	0.92378	0.93154	0.93498	0.93611	0.93963	0.94329	0.94714
421	0.92394	0.93168	0.93512	0.93623	0.93975	0.94341	0.94724
422	0.92409	0.93182	0.93525	0.93636	0.93988	0.94352	0.94735
423	0.92425	0.93196	0.93538	0.93649	0.93999	0.94363	0.94745
424	0.92440	0.93210	0.93551	0.93662	0.94011	0.94374	0.94755
425	0.92457	0.93223	0.93564	0.93674	0.94023	0.94385	0.94765
426	0.92472	0.93237	0.93577	0.93687	0.94035	0.94396	0.94775
427	0.92487	0.93250	0.93590	0.93700	0.94046	0.94407	0.94785
428	0.92503	0.93263	0.93603	0.93712	0.94058	0.94418	0.94796
429	0.92517	0.93276	0.93615	0.93724	0.94070	0.94429	0.94806
430	0.92534	0.93289	0.93628	0.93737	0.94081	0.94440	0.94816
431	0.92547	0.93303	0.93640	0.93749	0.94093	0.94451	0.94826
432	0.92560	0.93316	0.93652	0.93761	0.94105	0.94461	0.94835
433	0.92576	0.93330	0.93665	0.93773	0.94116	0.94472	0.94845
434	0.92590	0.93343	0.93677	0.93785	0.94127	0.94483	0.94855
435	0.92604	0.93356	0.93690	0.93797	0.94139	0.94493	0.94865
436	0.92619	0.93370	0.93702	0.93809	0.94150	0.94504	0.94874
437	0.92634	0.93382	0.93714	0.93821	0.94161	0.94514	0.94884
438	0.92646	0.93395	0.93726	0.93833	0.94172	0.94525	0.94894
439	0.92661	0.93407	0.93738	0.93845	0.94184	0.94535	0.94904
440	0.92675	0.93420	0.93750	0.93857	0.94195	0.94545	0.94913
441	0.92691	0.93433	0.93762	0.93869	0.94206	0.94556	0.94923
442	0.92707	0.93446	0.93774	0.93881	0.94217	0.94566	0.94932
443	0.92722	0.93458	0.93786	0.93892	0.94228	0.94576	0.94942
444	0.92735	0.93471	0.93798	0.93904	0.94239	0.94587	0.94951
445	0.92750	0.93483	0.93810	0.93916	0.94250	0.94596	0.94960
446	0.92764	0.93496	0.93822	0.93927	0.94261	0.94607	0.94970
447	0.92777	0.93508	0.93834	0.93939	0.94272	0.94617	0.94979
448	0.92792	0.93520	0.93845	0.93950	0.94282	0.94627	0.94989
449	0.92805	0.93533	0.93857	0.93962	0.94293	0.94637	0.94998
450	0.92819	0.93545	0.93869	0.93973	0.94304	0.94647	0.95007
451	0.92834	0.93558	0.93880	0.93984	0.94314	0.94657	0.95016
452	0.92846	0.93570	0.93892	0.93995	0.94325	0.94667	0.95025
453	0.92860	0.93581	0.93903	0.94006	0.94336	0.94677	0.95034
454	0.92873	0.93594	0.93914	0.94018	0.94346	0.94686	0.95044
455	0.92886	0.93606	0.93925	0.94029	0.94357	0.94696	0.95053
456	0.92899	0.93618	0.93937	0.94040	0.94367	0.94706	0.95062
457	0.92911	0.93630	0.93948	0.94051	0.94377	0.94715	0.95070
458	0.92924	0.93642	0.93959	0.94061	0.94387	0.94725	0.95079
459	0.92938	0.93654	0.93969	0.94072	0.94398	0.94735	0.95088
460	0.92951	0.93666	0.93981	0.94083	0.94408	0.94744	0.95097
461	0.92964	0.93677	0.93992	0.94095	0.94418	0.94754	0.95106
462	0.92977	0.93689	0.94003	0.94106	0.94428	0.94763	0.95115
463	0.92990	0.93702	0.94014	0.94116	0.94439	0.94773	0.95123
464	0.93004	0.93714	0.94025	0.94127	0.94449	0.94782	0.95132
465	0.93016	0.93725	0.94036	0.94138	0.94459	0.94792	0.95141
466	0.93030	0.93737	0.94047	0.94149	0.94469	0.94801	0.95149
467	0.93042	0.93748	0.94059	0.94160	0.94479	0.94810	0.95158
468	0.93055	0.93759	0.94069	0.94170	0.94489	0.94820	0.95167
469	0.93069	0.93771	0.94080	0.94181	0.94499	0.94829	0.95175
470	0.93082	0.93782	0.94091	0.94192	0.94509	0.94838	0.95184
471	0.93095	0.93793	0.94101	0.94202	0.94519	0.94848	0.95192

N	0.1%	0.5%	1%	1.25%	2.5%	5%	10%
472	0.93108	0.93804	0.94112	0.94212	0.94528	0.94857	0.95200
473	0.93122	0.93816	0.94123	0.94223	0.94538	0.94866	0.95209
474	0.93134	0.93827	0.94133	0.94232	0.94548	0.94875	0.95217
475	0.93146	0.93838	0.94144	0.94243	0.94557	0.94884	0.95225
476	0.93158	0.93850	0.94154	0.94253	0.94567	0.94893	0.95234
477	0.93171	0.93861	0.94165	0.94264	0.94577	0.94902	0.95242
478	0.93185	0.93871	0.94175	0.94273	0.94586	0.94911	0.95250
479	0.93196	0.93882	0.94186	0.94284	0.94596	0.94920	0.95259
480	0.93209	0.93893	0.94196	0.94294	0.94605	0.94929	0.95267
481	0.93219	0.93904	0.94206	0.94304	0.94615	0.94937	0.95275
482	0.93231	0.93915	0.94216	0.94314	0.94624	0.94946	0.95283
483	0.93243	0.93925	0.94226	0.94324	0.94634	0.94955	0.95291
484	0.93256	0.93937	0.94236	0.94334	0.94643	0.94964	0.95300
485	0.93268	0.93947	0.94247	0.94344	0.94652	0.94972	0.95308
486	0.93280	0.93957	0.94257	0.94354	0.94661	0.94981	0.95316
487	0.93292	0.93968	0.94267	0.94364	0.94671	0.94990	0.95324
488	0.93305	0.93978	0.94277	0.94374	0.94680	0.94999	0.95332
489	0.93315	0.93989	0.94286	0.94384	0.94689	0.95007	0.95339
490	0.93328	0.93999	0.94296	0.94393	0.94699	0.95015	0.95347
491	0.93339	0.94010	0.94306	0.94403	0.94708	0.95024	0.95355
492	0.93351	0.94020	0.94316	0.94412	0.94717	0.95032	0.95363
493	0.93363	0.94031	0.94326	0.94422	0.94726	0.95041	0.95371
494	0.93376	0.94042	0.94335	0.94432	0.94735	0.95049	0.95379
495	0.93387	0.94052	0.94345	0.94442	0.94744	0.95057	0.95386
496	0.93399	0.94062	0.94355	0.94451	0.94752	0.95066	0.95394
497	0.93411	0.94073	0.94365	0.94461	0.94761	0.95074	0.95402
498	0.93423	0.94083	0.94374	0.94470	0.94770	0.95083	0.95410
499	0.93434	0.94093	0.94384	0.94479	0.94779	0.95091	0.95417
500	0.93448	0.94103	0.94394	0.94489	0.94788	0.95099	0.95425
501	0.93459	0.94113	0.94404	0.94498	0.94797	0.95107	0.95433
502	0.93469	0.94123	0.94413	0.94508	0.94806	0.95116	0.95440
503	0.93481	0.94133	0.94422	0.94517	0.94814	0.95124	0.95448
504	0.93492	0.94144	0.94432	0.94526	0.94823	0.95132	0.95455
505	0.93502	0.94153	0.94441	0.94536	0.94832	0.95140	0.95463
506	0.93514	0.94163	0.94451	0.94545	0.94840	0.95148	0.95470
507	0.93526	0.94173	0.94460	0.94554	0.94849	0.95156	0.95478
508	0.93535	0.94183	0.94470	0.94563	0.94858	0.95164	0.95485
509	0.93548	0.94193	0.94479	0.94572	0.94866	0.95172	0.95492
510	0.93559	0.94203	0.94488	0.94581	0.94875	0.95180	0.95500
511	0.93570	0.94212	0.94498	0.94590	0.94883	0.95188	0.95507
512	0.93581	0.94222	0.94507	0.94599	0.94891	0.95196	0.95514
513	0.93591	0.94231	0.94516	0.94608	0.94900	0.95204	0.95521
514	0.93603	0.94241	0.94525	0.94617	0.94908	0.95211	0.95529
515	0.93614	0.94251	0.94535	0.94626	0.94917	0.95219	0.95536
516	0.93624	0.94260	0.94543	0.94635	0.94925	0.95227	0.95543
517	0.93636	0.94269	0.94553	0.94644	0.94934	0.95235	0.95550
518	0.93646	0.94279	0.94562	0.94653	0.94942	0.95242	0.95558
519	0.93656	0.94289	0.94570	0.94662	0.94950	0.95250	0.95565
520	0.93666	0.94298	0.94579	0.94670	0.94959	0.95258	0.95572
521	0.93677	0.94308	0.94588	0.94679	0.94967	0.95265	0.95579
522	0.93687	0.94317	0.94597	0.94688	0.94975	0.95273	0.95586
523	0.93697	0.94326	0.94606	0.94696	0.94983	0.95281	0.95593
524	0.93708	0.94336	0.94615	0.94705	0.94991	0.95288	0.95600
525	0.93718	0.94345	0.94624	0.94714	0.94999	0.95296	0.95607
526	0.93729	0.94355	0.94632	0.94722	0.95007	0.95304	0.95614
527	0.93740	0.94363	0.94641	0.94731	0.95015	0.95311	0.95621
528	0.93750	0.94372	0.94649	0.94739	0.95023	0.95319	0.95628
529	0.93759	0.94382	0.94658	0.94748	0.95031	0.95326	0.95635
530	0.93770	0.94391	0.94667	0.94756	0.95039	0.95333	0.95641
531	0.93779	0.94400	0.94675	0.94765	0.95047	0.95341	0.95648
532	0.93788	0.94409	0.94684	0.94773	0.95055	0.95348	0.95655
533	0.93798	0.94418	0.94693	0.94781	0.95063	0.95355	0.95662
534	0.93808	0.94427	0.94701	0.94790	0.95071	0.95363	0.95669
535	0.93819	0.94436	0.94709	0.94798	0.95079	0.95370	0.95675
536	0.93829	0.94445	0.94718	0.94806	0.95086	0.95377	0.95682
537	0.93839	0.94455	0.94726	0.94815	0.95094	0.95385	0.95689
538	0.93850	0.94463	0.94735	0.94823	0.95102	0.95392	0.95695
539	0.93859	0.94472	0.94743	0.94831	0.95109	0.95399	0.95702
540	0.93869	0.94481	0.94751	0.94839	0.95117	0.95406	0.95709
541	0.93878	0.94489	0.94759	0.94847	0.95125	0.95413	0.95715
542	0.93888	0.94498	0.94768	0.94855	0.95133	0.95421	0.95722
543	0.93898	0.94507	0.94776	0.94863	0.95140	0.95428	0.95728
544	0.93907	0.94516	0.94785	0.94872	0.95148	0.95435	0.95735
545	0.93918	0.94525	0.94793	0.94880	0.95156	0.95442	0.95742

N	0.1%	0.5%	1%	1.25%	2.5%	5%	10%
546	0.93926	0.94533	0.94801	0.94888	0.95163	0.95449	0.95748
547	0.93936	0.94542	0.94809	0.94896	0.95171	0.95456	0.95754
548	0.93946	0.94550	0.94817	0.94904	0.95178	0.95463	0.95761
549	0.93956	0.94558	0.94825	0.94912	0.95186	0.95469	0.95767
550	0.93967	0.94567	0.94833	0.94920	0.95193	0.95476	0.95774
551	0.93977	0.94575	0.94841	0.94928	0.95201	0.95483	0.95780
552	0.93986	0.94584	0.94849	0.94936	0.95208	0.95490	0.95787
553	0.93995	0.94592	0.94857	0.94944	0.95215	0.95497	0.95793
554	0.94005	0.94600	0.94865	0.94951	0.95223	0.95504	0.95799
555	0.94015	0.94609	0.94873	0.94959	0.95230	0.95511	0.95806
556	0.94024	0.94617	0.94881	0.94967	0.95237	0.95518	0.95812
557	0.94033	0.94626	0.94889	0.94975	0.95245	0.95524	0.95818
558	0.94042	0.94634	0.94897	0.94983	0.95252	0.95531	0.95825
559	0.94052	0.94642	0.94904	0.94990	0.95259	0.95538	0.95831
560	0.94061	0.94651	0.94913	0.94998	0.95266	0.95544	0.95837
561	0.94070	0.94658	0.94921	0.95005	0.95273	0.95551	0.95843
562	0.94080	0.94667	0.94928	0.95013	0.95281	0.95558	0.95849
563	0.94088	0.94675	0.94936	0.95021	0.95288	0.95565	0.95855
564	0.94098	0.94683	0.94944	0.95028	0.95295	0.95571	0.95862
565	0.94107	0.94691	0.94951	0.95036	0.95302	0.95578	0.95868
566	0.94115	0.94699	0.94959	0.95044	0.95309	0.95584	0.95874
567	0.94124	0.94708	0.94967	0.95051	0.95316	0.95591	0.95880
568	0.94132	0.94716	0.94974	0.95058	0.95323	0.95598	0.95886
569	0.94142	0.94724	0.94982	0.95066	0.95330	0.95604	0.95892
570	0.94150	0.94732	0.94989	0.95074	0.95337	0.95611	0.95898
571	0.94159	0.94740	0.94997	0.95081	0.95344	0.95617	0.95904
572	0.94167	0.94748	0.95005	0.95088	0.95351	0.95624	0.95910
573	0.94177	0.94755	0.95012	0.95095	0.95358	0.95630	0.95916
574	0.94185	0.94763	0.95019	0.95102	0.95365	0.95636	0.95922
575	0.94195	0.94771	0.95027	0.95110	0.95372	0.95643	0.95928
576	0.94203	0.94778	0.95034	0.95117	0.95379	0.95649	0.95934
577	0.94212	0.94786	0.95041	0.95124	0.95385	0.95655	0.95940
578	0.94220	0.94794	0.95048	0.95131	0.95392	0.95662	0.95946
579	0.94229	0.94802	0.95056	0.95138	0.95399	0.95668	0.95951
580	0.94237	0.94809	0.95063	0.95146	0.95406	0.95674	0.95957
581	0.94246	0.94817	0.95070	0.95153	0.95412	0.95680	0.95963
582	0.94255	0.94825	0.95077	0.95160	0.95419	0.95687	0.95969
583	0.94263	0.94833	0.95085	0.95167	0.95426	0.95693	0.95975
584	0.94271	0.94840	0.95092	0.95174	0.95433	0.95699	0.95980
585	0.94280	0.94848	0.95099	0.95181	0.95439	0.95705	0.95986
586	0.94289	0.94855	0.95106	0.95188	0.95446	0.95712	0.95992
587	0.94298	0.94863	0.95113	0.95196	0.95452	0.95718	0.95998
588	0.94305	0.94871	0.95120	0.95202	0.95459	0.95724	0.96003
589	0.94314	0.94878	0.95127	0.95210	0.95465	0.95730	0.96009
590	0.94322	0.94886	0.95134	0.95217	0.95472	0.95736	0.96015
591	0.94330	0.94893	0.95141	0.95223	0.95478	0.95742	0.96020
592	0.94340	0.94901	0.95149	0.95231	0.95485	0.95748	0.96026
593	0.94348	0.94908	0.95156	0.95237	0.95491	0.95754	0.96031
594	0.94356	0.94916	0.95163	0.95244	0.95498	0.95761	0.96037
595	0.94364	0.94923	0.95169	0.95251	0.95504	0.95767	0.96042
596	0.94373	0.94931	0.95177	0.95258	0.95511	0.95773	0.96048
597	0.94381	0.94938	0.95183	0.95264	0.95517	0.95779	0.96054
598	0.94389	0.94946	0.95190	0.95271	0.95523	0.95785	0.96059
599	0.94397	0.94953	0.95197	0.95278	0.95530	0.95790	0.96065
600	0.94405	0.94961	0.95204	0.95285	0.95536	0.95796	0.96070
601	0.94413	0.94967	0.95211	0.95291	0.95543	0.95802	0.96076
602	0.94422	0.94975	0.95218	0.95298	0.95549	0.95808	0.96081
603	0.94430	0.94982	0.95224	0.95305	0.95555	0.95814	0.96086
604	0.94439	0.94989	0.95231	0.95311	0.95561	0.95820	0.96092
605	0.94446	0.94996	0.95238	0.95318	0.95568	0.95826	0.96097
606	0.94454	0.95003	0.95245	0.95324	0.95574	0.95832	0.96103
607	0.94462	0.95011	0.95251	0.95331	0.95580	0.95838	0.96108
608	0.94470	0.95018	0.95258	0.95338	0.95586	0.95843	0.96113
609	0.94477	0.95025	0.95265	0.95344	0.95592	0.95849	0.96119
610	0.94485	0.95032	0.95271	0.95351	0.95598	0.95855	0.96124
611	0.94493	0.95038	0.95278	0.95357	0.95605	0.95860	0.96129
612	0.94501	0.95046	0.95285	0.95363	0.95611	0.95866	0.96135
613	0.94508	0.95053	0.95291	0.95370	0.95617	0.95872	0.96140
614	0.94516	0.95059	0.95298	0.95376	0.95623	0.95877	0.96145
615	0.94524	0.95066	0.95304	0.95382	0.95629	0.95883	0.96151
616	0.94530	0.95073	0.95311	0.95389	0.95635	0.95889	0.96156
617	0.94539	0.95080	0.95318	0.95396	0.95641	0.95895	0.96161
618	0.94546	0.95087	0.95324	0.95402	0.95647	0.95900	0.96166
619	0.94553	0.95094	0.95330	0.95408	0.95653	0.95906	0.96171

N	0.1%	0.5%	1%	1.25%	2.5%	5%	10%
620	0.94561	0.95101	0.95337	0.95415	0.95659	0.95911	0.96177
621	0.94569	0.95108	0.95344	0.95421	0.95665	0.95917	0.96182
622	0.94576	0.95114	0.95350	0.95427	0.95671	0.95922	0.96187
623	0.94584	0.95121	0.95356	0.95434	0.95677	0.95928	0.96192
624	0.94590	0.95128	0.95363	0.95440	0.95683	0.95933	0.96197
625	0.94598	0.95134	0.95369	0.95446	0.95689	0.95939	0.96202
626	0.94605	0.95141	0.95375	0.95452	0.95694	0.95944	0.96207
627	0.94613	0.95148	0.95382	0.95458	0.95700	0.95950	0.96212
628	0.94622	0.95155	0.95388	0.95465	0.95706	0.95955	0.96217
629	0.94628	0.95161	0.95394	0.95471	0.95712	0.95961	0.96222
630	0.94635	0.95168	0.95401	0.95477	0.95718	0.95966	0.96228
631	0.94643	0.95174	0.95407	0.95483	0.95724	0.95972	0.96232
632	0.94649	0.95181	0.95413	0.95489	0.95729	0.95977	0.96237
633	0.94657	0.95187	0.95419	0.95495	0.95735	0.95982	0.96242
634	0.94664	0.95193	0.95426	0.95501	0.95741	0.95988	0.96247
635	0.94671	0.95200	0.95432	0.95508	0.95746	0.95993	0.96252
636	0.94678	0.95206	0.95438	0.95514	0.95752	0.95998	0.96257
637	0.94687	0.95212	0.95444	0.95520	0.95758	0.96004	0.96262
638	0.94694	0.95219	0.95450	0.95526	0.95763	0.96009	0.96267
639	0.94701	0.95226	0.95457	0.95532	0.95769	0.96014	0.96272
640	0.94708	0.95232	0.95463	0.95538	0.95775	0.96020	0.96277
641	0.94716	0.95239	0.95469	0.95544	0.95781	0.96025	0.96282
642	0.94723	0.95245	0.95475	0.95549	0.95786	0.96030	0.96287
643	0.94729	0.95251	0.95481	0.95555	0.95792	0.96035	0.96292
644	0.94737	0.95258	0.95486	0.95562	0.95797	0.96040	0.96296
645	0.94743	0.95264	0.95492	0.95567	0.95803	0.96046	0.96301
646	0.94751	0.95271	0.95498	0.95573	0.95808	0.96051	0.96306
647	0.94758	0.95277	0.95504	0.95579	0.95814	0.96056	0.96311
648	0.94765	0.95283	0.95510	0.95585	0.95819	0.96061	0.96316
649	0.94771	0.95290	0.95516	0.95591	0.95825	0.96066	0.96320
650	0.94779	0.95296	0.95522	0.95596	0.95830	0.96071	0.96325
651	0.94786	0.95302	0.95528	0.95602	0.95836	0.96077	0.96330
652	0.94793	0.95309	0.95534	0.95608	0.95841	0.96082	0.96335
653	0.94800	0.95315	0.95540	0.95614	0.95847	0.96087	0.96339
654	0.94808	0.95321	0.95546	0.95620	0.95852	0.96092	0.96344
655	0.94815	0.95327	0.95552	0.95626	0.95857	0.96097	0.96349
656	0.94821	0.95334	0.95558	0.95631	0.95863	0.96102	0.96353
657	0.94827	0.95340	0.95564	0.95637	0.95868	0.96107	0.96358
658	0.94835	0.95346	0.95570	0.95643	0.95874	0.96112	0.96363
659	0.94842	0.95352	0.95575	0.95648	0.95879	0.96117	0.96367
660	0.94848	0.95358	0.95581	0.95654	0.95884	0.96122	0.96372
661	0.94855	0.95364	0.95587	0.95660	0.95889	0.96127	0.96377
662	0.94862	0.95370	0.95593	0.95665	0.95895	0.96132	0.96381
663	0.94868	0.95376	0.95598	0.95671	0.95900	0.96137	0.96386
664	0.94876	0.95382	0.95604	0.95676	0.95905	0.96142	0.96390
665	0.94882	0.95388	0.95610	0.95682	0.95911	0.96147	0.96395
666	0.94890	0.95394	0.95616	0.95688	0.95916	0.96152	0.96399
667	0.94896	0.95400	0.95621	0.95693	0.95921	0.96157	0.96404
668	0.94903	0.95406	0.95627	0.95699	0.95926	0.96161	0.96408
669	0.94909	0.95412	0.95633	0.95704	0.95931	0.96166	0.96413
670	0.94915	0.95418	0.95638	0.95710	0.95937	0.96171	0.96417
671	0.94922	0.95424	0.95644	0.95715	0.95942	0.96176	0.96422
672	0.94929	0.95429	0.95649	0.95721	0.95947	0.96181	0.96426
673	0.94936	0.95435	0.95655	0.95726	0.95952	0.96186	0.96431
674	0.94942	0.95441	0.95660	0.95732	0.95957	0.96190	0.96435
675	0.94948	0.95447	0.95666	0.95737	0.95962	0.96195	0.96440
676	0.94955	0.95453	0.95672	0.95743	0.95967	0.96200	0.96444
677	0.94962	0.95459	0.95677	0.95748	0.95973	0.96205	0.96448
678	0.94968	0.95465	0.95683	0.95754	0.95978	0.96209	0.96453
679	0.94975	0.95471	0.95689	0.95759	0.95983	0.96214	0.96457
680	0.94982	0.95476	0.95694	0.95764	0.95988	0.96219	0.96462
681	0.94987	0.95482	0.95699	0.95770	0.95993	0.96223	0.96466
682	0.94994	0.95487	0.95705	0.95775	0.95998	0.96228	0.96470
683	0.95001	0.95493	0.95710	0.95780	0.96003	0.96233	0.96475
684	0.95007	0.95499	0.95715	0.95786	0.96008	0.96238	0.96479
685	0.95014	0.95505	0.95721	0.95791	0.96013	0.96242	0.96483
686	0.95021	0.95510	0.95726	0.95796	0.96018	0.96247	0.96488
687	0.95028	0.95516	0.95732	0.95802	0.96023	0.96252	0.96492
688	0.95034	0.95521	0.95737	0.95807	0.96028	0.96256	0.96496
689	0.95040	0.95527	0.95742	0.95812	0.96032	0.96261	0.96500
690	0.95046	0.95533	0.95747	0.95817	0.96038	0.96265	0.96505
691	0.95052	0.95538	0.95753	0.95822	0.96042	0.96270	0.96509
692	0.95059	0.95544	0.95758	0.95828	0.96047	0.96275	0.96513
693	0.95064	0.95549	0.95763	0.95833	0.96052	0.96279	0.96518

N	0.1%	0.5%	1%	1.25%	2.5%	5%	10%
694	0.95071	0.95555	0.95768	0.95838	0.96057	0.96284	0.96522
695	0.95077	0.95560	0.95773	0.95843	0.96062	0.96288	0.96526
696	0.95083	0.95566	0.95779	0.95848	0.96067	0.96293	0.96530
697	0.95089	0.95572	0.95784	0.95853	0.96071	0.96297	0.96534
698	0.95095	0.95577	0.95789	0.95858	0.96076	0.96302	0.96538
699	0.95101	0.95583	0.95794	0.95863	0.96081	0.96306	0.96543
700	0.95107	0.95588	0.95799	0.95868	0.96086	0.96311	0.96547
701	0.95112	0.95593	0.95805	0.95874	0.96091	0.96315	0.96551
702	0.95118	0.95599	0.95810	0.95878	0.96096	0.96320	0.96555
703	0.95125	0.95604	0.95815	0.95883	0.96100	0.96324	0.96559
704	0.95131	0.95610	0.95820	0.95888	0.96105	0.96328	0.96563
705	0.95137	0.95615	0.95825	0.95894	0.96110	0.96333	0.96567
706	0.95143	0.95620	0.95830	0.95899	0.96115	0.96337	0.96572
707	0.95148	0.95625	0.95835	0.95904	0.96119	0.96342	0.96576
708	0.95156	0.95631	0.95840	0.95909	0.96124	0.96346	0.96580
709	0.95161	0.95636	0.95845	0.95913	0.96129	0.96350	0.96584
710	0.95167	0.95642	0.95850	0.95919	0.96133	0.96355	0.96588
711	0.95173	0.95647	0.95855	0.95924	0.96138	0.96359	0.96592
712	0.95179	0.95652	0.95861	0.95928	0.96142	0.96363	0.96596
713	0.95185	0.95658	0.95866	0.95933	0.96147	0.96368	0.96600
714	0.95190	0.95663	0.95871	0.95938	0.96152	0.96372	0.96604
715	0.95196	0.95668	0.95876	0.95943	0.96156	0.96376	0.96608
716	0.95202	0.95673	0.95881	0.95948	0.96161	0.96381	0.96612
717	0.95208	0.95679	0.95886	0.95953	0.96166	0.96385	0.96616
718	0.95215	0.95684	0.95891	0.95958	0.96170	0.96389	0.96620
719	0.95220	0.95690	0.95896	0.95963	0.96175	0.96394	0.96623
720	0.95226	0.95695	0.95901	0.95968	0.96179	0.96398	0.96627
721	0.95231	0.95700	0.95906	0.95973	0.96184	0.96402	0.96631
722	0.95237	0.95705	0.95911	0.95977	0.96188	0.96406	0.96635
723	0.95243	0.95710	0.95915	0.95982	0.96193	0.96410	0.96639
724	0.95249	0.95715	0.95920	0.95987	0.96197	0.96415	0.96643
725	0.95254	0.95720	0.95925	0.95991	0.96202	0.96419	0.96647
726	0.95261	0.95726	0.95930	0.95996	0.96206	0.96423	0.96651
727	0.95266	0.95730	0.95935	0.96001	0.96211	0.96427	0.96655
728	0.95272	0.95735	0.95940	0.96006	0.96215	0.96431	0.96659
729	0.95278	0.95741	0.95944	0.96011	0.96220	0.96435	0.96662
730	0.95283	0.95745	0.95949	0.96015	0.96224	0.96440	0.96666
731	0.95289	0.95750	0.95954	0.96020	0.96228	0.96444	0.96670
732	0.95295	0.95755	0.95958	0.96024	0.96233	0.96448	0.96674
733	0.95300	0.95761	0.95963	0.96029	0.96237	0.96452	0.96678
734	0.95306	0.95766	0.95968	0.96034	0.96242	0.96456	0.96682
735	0.95312	0.95771	0.95973	0.96038	0.96246	0.96460	0.96685
736	0.95317	0.95776	0.95977	0.96043	0.96250	0.96464	0.96689
737	0.95323	0.95781	0.95982	0.96048	0.96255	0.96468	0.96693
738	0.95329	0.95786	0.95987	0.96052	0.96259	0.96473	0.96697
739	0.95335	0.95790	0.95991	0.96057	0.96263	0.96477	0.96701
740	0.95341	0.95795	0.95996	0.96061	0.96268	0.96481	0.96704
741	0.95346	0.95801	0.96001	0.96066	0.96272	0.96485	0.96708
742	0.95352	0.95805	0.96005	0.96070	0.96276	0.96489	0.96712
743	0.95357	0.95810	0.96010	0.96075	0.96280	0.96493	0.96715
744	0.95363	0.95815	0.96014	0.96079	0.96285	0.96497	0.96719
745	0.95368	0.95820	0.96019	0.96084	0.96289	0.96501	0.96723
746	0.95374	0.95824	0.96024	0.96088	0.96293	0.96505	0.96727
747	0.95379	0.95829	0.96028	0.96093	0.96298	0.96509	0.96730
748	0.95384	0.95834	0.96033	0.96098	0.96302	0.96513	0.96734
749	0.95389	0.95839	0.96038	0.96102	0.96306	0.96517	0.96738
750	0.95395	0.95844	0.96042	0.96107	0.96310	0.96521	0.96741
751	0.95400	0.95849	0.96047	0.96111	0.96315	0.96525	0.96745
752	0.95406	0.95854	0.96051	0.96115	0.96319	0.96529	0.96749
753	0.95411	0.95858	0.96056	0.96120	0.96323	0.96532	0.96752
754	0.95415	0.95863	0.96060	0.96124	0.96327	0.96536	0.96756
755	0.95421	0.95868	0.96065	0.96129	0.96331	0.96540	0.96759
756	0.95426	0.95873	0.96069	0.96133	0.96335	0.96544	0.96763
757	0.95432	0.95877	0.96074	0.96137	0.96340	0.96548	0.96767
758	0.95437	0.95882	0.96078	0.96142	0.96343	0.96552	0.96770
759	0.95443	0.95887	0.96083	0.96146	0.96348	0.96556	0.96774
760	0.95448	0.95891	0.96087	0.96150	0.96352	0.96560	0.96777
761	0.95453	0.95896	0.96092	0.96155	0.96356	0.96563	0.96781
762	0.95458	0.95901	0.96096	0.96159	0.96360	0.96567	0.96785
763	0.95464	0.95906	0.96100	0.96164	0.96364	0.96571	0.96788
764	0.95469	0.95910	0.96105	0.96168	0.96368	0.96575	0.96792
765	0.95474	0.95915	0.96109	0.96172	0.96372	0.96579	0.96795
766	0.95479	0.95919	0.96114	0.96177	0.96376	0.96583	0.96799
767	0.95485	0.95924	0.96118	0.96181	0.96380	0.96586	0.96802

N	0.1%	0.5%	1%	1.25%	2.5%	5%	10%
768	0.95490	0.95928	0.96123	0.96185	0.96384	0.96590	0.96806
769	0.95494	0.95933	0.96127	0.96190	0.96388	0.96594	0.96809
770	0.95499	0.95937	0.96131	0.96194	0.96392	0.96598	0.96813
771	0.95505	0.95942	0.96135	0.96198	0.96396	0.96601	0.96816
772	0.95511	0.95946	0.96140	0.96203	0.96401	0.96605	0.96820
773	0.95516	0.95951	0.96144	0.96207	0.96404	0.96609	0.96823
774	0.95521	0.95955	0.96148	0.96211	0.96408	0.96612	0.96826
775	0.95526	0.95960	0.96152	0.96215	0.96412	0.96616	0.96830
776	0.95531	0.95964	0.96157	0.96219	0.96416	0.96620	0.96833
777	0.95536	0.95969	0.96161	0.96223	0.96420	0.96624	0.96837
778	0.95540	0.95973	0.96165	0.96228	0.96424	0.96627	0.96840
779	0.95546	0.95978	0.96170	0.96232	0.96428	0.96631	0.96844
780	0.95551	0.95982	0.96174	0.96236	0.96432	0.96635	0.96847
781	0.95556	0.95987	0.96178	0.96240	0.96436	0.96638	0.96850
782	0.95561	0.95991	0.96182	0.96244	0.96440	0.96642	0.96854
783	0.95566	0.95996	0.96187	0.96248	0.96444	0.96646	0.96857
784	0.95571	0.96000	0.96191	0.96252	0.96448	0.96649	0.96861
785	0.95576	0.96005	0.96195	0.96257	0.96452	0.96653	0.96864
786	0.95581	0.96009	0.96199	0.96261	0.96455	0.96657	0.96867
787	0.95586	0.96013	0.96203	0.96265	0.96459	0.96660	0.96871
788	0.95591	0.96018	0.96208	0.96269	0.96463	0.96664	0.96874
789	0.95596	0.96022	0.96212	0.96273	0.96467	0.96667	0.96877
790	0.95601	0.96027	0.96216	0.96277	0.96471	0.96671	0.96881
791	0.95606	0.96031	0.96220	0.96281	0.96475	0.96675	0.96884
792	0.95610	0.96035	0.96224	0.96285	0.96478	0.96678	0.96888
793	0.95615	0.96040	0.96228	0.96289	0.96482	0.96682	0.96891
794	0.95620	0.96044	0.96233	0.96293	0.96486	0.96685	0.96894
795	0.95625	0.96048	0.96237	0.96297	0.96490	0.96689	0.96898
796	0.95630	0.96053	0.96241	0.96301	0.96494	0.96692	0.96901
797	0.95635	0.96057	0.96245	0.96305	0.96497	0.96696	0.96904
798	0.95639	0.96061	0.96249	0.96309	0.96501	0.96699	0.96907
799	0.95644	0.96066	0.96253	0.96313	0.96505	0.96703	0.96911
800	0.95649	0.96070	0.96257	0.96317	0.96509	0.96707	0.96914
801	0.95653	0.96074	0.96261	0.96321	0.96512	0.96710	0.96917
802	0.95658	0.96078	0.96265	0.96325	0.96516	0.96714	0.96920
803	0.95663	0.96082	0.96269	0.96329	0.96520	0.96717	0.96924
804	0.95668	0.96087	0.96273	0.96333	0.96524	0.96720	0.96927
805	0.95673	0.96091	0.96277	0.96337	0.96527	0.96724	0.96930
806	0.95678	0.96096	0.96281	0.96341	0.96531	0.96727	0.96933
807	0.95682	0.96100	0.96285	0.96345	0.96535	0.96731	0.96937
808	0.95686	0.96104	0.96289	0.96349	0.96539	0.96734	0.96940
809	0.95690	0.96108	0.96293	0.96353	0.96542	0.96738	0.96943
810	0.95695	0.96112	0.96297	0.96356	0.96546	0.96741	0.96946
811	0.95700	0.96116	0.96301	0.96360	0.96549	0.96745	0.96949
812	0.95704	0.96120	0.96305	0.96364	0.96553	0.96748	0.96952
813	0.95709	0.96125	0.96309	0.96368	0.96557	0.96751	0.96956
814	0.95713	0.96129	0.96313	0.96372	0.96560	0.96755	0.96959
815	0.95718	0.96133	0.96316	0.96376	0.96564	0.96758	0.96962
816	0.95722	0.96137	0.96320	0.96379	0.96568	0.96762	0.96965
817	0.95726	0.96141	0.96324	0.96383	0.96571	0.96765	0.96968
818	0.95731	0.96145	0.96328	0.96387	0.96575	0.96768	0.96971
819	0.95736	0.96149	0.96332	0.96391	0.96578	0.96772	0.96974
820	0.95740	0.96153	0.96336	0.96395	0.96582	0.96775	0.96978
821	0.95745	0.96157	0.96340	0.96398	0.96585	0.96778	0.96981
822	0.95749	0.96161	0.96343	0.96402	0.96589	0.96782	0.96984
823	0.95754	0.96166	0.96347	0.96406	0.96592	0.96785	0.96987
824	0.95758	0.96169	0.96351	0.96410	0.96596	0.96788	0.96990
825	0.95762	0.96173	0.96355	0.96413	0.96600	0.96792	0.96993
826	0.95767	0.96178	0.96359	0.96417	0.96603	0.96795	0.96996
827	0.95772	0.96182	0.96362	0.96421	0.96607	0.96798	0.96999
828	0.95777	0.96186	0.96366	0.96424	0.96610	0.96802	0.97002
829	0.95782	0.96190	0.96370	0.96428	0.96614	0.96805	0.97005
830	0.95786	0.96194	0.96374	0.96432	0.96617	0.96808	0.97008
831	0.95790	0.96198	0.96377	0.96436	0.96621	0.96812	0.97011
832	0.95795	0.96202	0.96381	0.96439	0.96624	0.96815	0.97014
833	0.95800	0.96206	0.96385	0.96443	0.96628	0.96818	0.97018
834	0.95804	0.96210	0.96389	0.96447	0.96631	0.96821	0.97020
835	0.95809	0.96214	0.96392	0.96451	0.96635	0.96824	0.97024
836	0.95813	0.96218	0.96396	0.96454	0.96638	0.96828	0.97027
837	0.95817	0.96222	0.96400	0.96458	0.96642	0.96831	0.97030
838	0.95821	0.96226	0.96404	0.96462	0.96645	0.96834	0.97033
839	0.95826	0.96229	0.96407	0.96465	0.96648	0.96837	0.97036
840	0.95830	0.96233	0.96411	0.96469	0.96652	0.96841	0.97039
841	0.95834	0.96237	0.96415	0.96473	0.96655	0.96844	0.97041

N	0.1%	0.5%	1%	1.25%	2.5%	5%	10%
842	0.95838	0.96241	0.96419	0.96476	0.96659	0.96847	0.97044
843	0.95843	0.96245	0.96422	0.96480	0.96662	0.96850	0.97047
844	0.95848	0.96249	0.96426	0.96483	0.96666	0.96853	0.97050
845	0.95852	0.96253	0.96430	0.96487	0.96669	0.96856	0.97053
846	0.95856	0.96257	0.96433	0.96491	0.96672	0.96860	0.97056
847	0.95860	0.96260	0.96437	0.96494	0.96676	0.96863	0.97059
848	0.95865	0.96264	0.96441	0.96498	0.96679	0.96866	0.97062
849	0.95869	0.96268	0.96444	0.96501	0.96682	0.96869	0.97065
850	0.95874	0.96272	0.96448	0.96505	0.96686	0.96872	0.97068
851	0.95878	0.96275	0.96451	0.96508	0.96689	0.96875	0.97071
852	0.95882	0.96279	0.96455	0.96512	0.96693	0.96878	0.97074
853	0.95886	0.96283	0.96459	0.96516	0.96696	0.96882	0.97077
854	0.95890	0.96287	0.96462	0.96519	0.96699	0.96885	0.97080
855	0.95894	0.96291	0.96466	0.96523	0.96702	0.96888	0.97082
856	0.95899	0.96294	0.96469	0.96526	0.96706	0.96891	0.97085
857	0.95903	0.96298	0.96473	0.96530	0.96709	0.96894	0.97088
858	0.95908	0.96302	0.96477	0.96533	0.96712	0.96897	0.97091
859	0.95911	0.96306	0.96480	0.96537	0.96716	0.96900	0.97094
860	0.95916	0.96309	0.96484	0.96540	0.96719	0.96903	0.97097
861	0.95920	0.96313	0.96487	0.96544	0.96722	0.96906	0.97100
862	0.95923	0.96317	0.96491	0.96547	0.96725	0.96909	0.97102
863	0.95928	0.96321	0.96494	0.96551	0.96729	0.96912	0.97105
864	0.95932	0.96324	0.96498	0.96554	0.96732	0.96915	0.97108
865	0.95936	0.96328	0.96502	0.96557	0.96735	0.96918	0.97111
866	0.95941	0.96332	0.96505	0.96561	0.96739	0.96922	0.97114
867	0.95945	0.96335	0.96508	0.96564	0.96742	0.96925	0.97117
868	0.95948	0.96339	0.96512	0.96568	0.96745	0.96928	0.97120
869	0.95953	0.96343	0.96515	0.96571	0.96748	0.96931	0.97122
870	0.95957	0.96347	0.96519	0.96575	0.96751	0.96934	0.97125
871	0.95961	0.96350	0.96522	0.96578	0.96755	0.96937	0.97128
872	0.95965	0.96354	0.96525	0.96581	0.96758	0.96940	0.97131
873	0.95969	0.96358	0.96529	0.96585	0.96761	0.96943	0.97133
874	0.95973	0.96361	0.96532	0.96588	0.96764	0.96946	0.97136
875	0.95977	0.96365	0.96536	0.96592	0.96767	0.96949	0.97139
876	0.95982	0.96368	0.96539	0.96595	0.96771	0.96952	0.97142
877	0.95985	0.96372	0.96543	0.96598	0.96774	0.96955	0.97145
878	0.95990	0.96376	0.96546	0.96601	0.96777	0.96958	0.97147
879	0.95994	0.96379	0.96550	0.96605	0.96780	0.96961	0.97150
880	0.95997	0.96383	0.96553	0.96608	0.96783	0.96964	0.97153
881	0.96001	0.96387	0.96556	0.96611	0.96786	0.96966	0.97156
882	0.96006	0.96390	0.96560	0.96615	0.96790	0.96969	0.97158
883	0.96011	0.96394	0.96563	0.96618	0.96793	0.96972	0.97161
884	0.96014	0.96397	0.96566	0.96621	0.96796	0.96975	0.97164
885	0.96018	0.96401	0.96570	0.96625	0.96799	0.96978	0.97166
886	0.96022	0.96404	0.96573	0.96628	0.96802	0.96981	0.97169
887	0.96026	0.96408	0.96577	0.96631	0.96805	0.96984	0.97172
888	0.96030	0.96412	0.96580	0.96635	0.96808	0.96987	0.97175
889	0.96034	0.96415	0.96583	0.96638	0.96811	0.96990	0.97177
890	0.96038	0.96419	0.96586	0.96641	0.96814	0.96993	0.97180
891	0.96042	0.96422	0.96590	0.96645	0.96818	0.96995	0.97183
892	0.96046	0.96426	0.96593	0.96648	0.96821	0.96998	0.97185
893	0.96050	0.96429	0.96597	0.96651	0.96824	0.97001	0.97188
894	0.96054	0.96433	0.96600	0.96654	0.96827	0.97004	0.97191
895	0.96058	0.96436	0.96603	0.96657	0.96830	0.97007	0.97193
896	0.96062	0.96440	0.96606	0.96661	0.96833	0.97010	0.97196
897	0.96066	0.96443	0.96610	0.96664	0.96836	0.97013	0.97199
898	0.96070	0.96446	0.96613	0.96667	0.96839	0.97016	0.97201
899	0.96074	0.96450	0.96616	0.96670	0.96842	0.97018	0.97204
900	0.96078	0.96453	0.96620	0.96673	0.96845	0.97021	0.97206
901	0.96082	0.96457	0.96623	0.96677	0.96848	0.97024	0.97209
902	0.96086	0.96460	0.96626	0.96680	0.96851	0.97027	0.97212
903	0.96090	0.96464	0.96629	0.96683	0.96854	0.97030	0.97214
904	0.96093	0.96467	0.96632	0.96686	0.96857	0.97032	0.97217
905	0.96097	0.96470	0.96635	0.96689	0.96860	0.97035	0.97220
906	0.96101	0.96474	0.96639	0.96692	0.96863	0.97038	0.97222
907	0.96104	0.96477	0.96642	0.96696	0.96866	0.97041	0.97225
908	0.96108	0.96480	0.96645	0.96699	0.96869	0.97044	0.97227
909	0.96112	0.96484	0.96648	0.96702	0.96872	0.97046	0.97230
910	0.96116	0.96487	0.96652	0.96705	0.96875	0.97049	0.97233
911	0.96119	0.96490	0.96655	0.96708	0.96878	0.97052	0.97235
912	0.96123	0.96494	0.96658	0.96711	0.96881	0.97055	0.97238
913	0.96126	0.96497	0.96661	0.96715	0.96884	0.97057	0.97240
914	0.96131	0.96501	0.96664	0.96718	0.96887	0.97060	0.97243
915	0.96135	0.96504	0.96667	0.96721	0.96889	0.97063	0.97245

N	0.1%	0.5%	1%	1.25%	2.5%	5%	10%
916	0.96139	0.96507	0.96671	0.96724	0.96892	0.97066	0.97248
917	0.96142	0.96511	0.96673	0.96727	0.96895	0.97068	0.97251
918	0.96146	0.96514	0.96677	0.96730	0.96898	0.97071	0.97253
919	0.96150	0.96517	0.96680	0.96733	0.96901	0.97074	0.97256
920	0.96154	0.96520	0.96683	0.96736	0.96904	0.97077	0.97258
921	0.96158	0.96524	0.96686	0.96739	0.96907	0.97079	0.97261
922	0.96161	0.96527	0.96689	0.96742	0.96910	0.97082	0.97263
923	0.96165	0.96530	0.96692	0.96745	0.96913	0.97085	0.97266
924	0.96168	0.96533	0.96696	0.96748	0.96915	0.97087	0.97268
925	0.96172	0.96537	0.96699	0.96751	0.96918	0.97090	0.97271
926	0.96176	0.96540	0.96702	0.96754	0.96921	0.97093	0.97273
927	0.96179	0.96543	0.96705	0.96758	0.96924	0.97096	0.97276
928	0.96183	0.96546	0.96708	0.96761	0.96927	0.97098	0.97278
929	0.96186	0.96549	0.96711	0.96764	0.96930	0.97101	0.97281
930	0.96190	0.96553	0.96714	0.96767	0.96933	0.97104	0.97283
931	0.96193	0.96556	0.96717	0.96770	0.96935	0.97106	0.97286
932	0.96197	0.96559	0.96720	0.96773	0.96938	0.97109	0.97288
933	0.96200	0.96563	0.96724	0.96776	0.96941	0.97111	0.97291
934	0.96204	0.96566	0.96726	0.96778	0.96944	0.97114	0.97293
935	0.96208	0.96569	0.96729	0.96781	0.96947	0.97117	0.97295
936	0.96212	0.96572	0.96733	0.96784	0.96950	0.97119	0.97298
937	0.96216	0.96575	0.96736	0.96788	0.96952	0.97122	0.97300
938	0.96219	0.96579	0.96739	0.96790	0.96955	0.97125	0.97303
939	0.96222	0.96582	0.96741	0.96794	0.96958	0.97127	0.97305
940	0.96226	0.96585	0.96744	0.96796	0.96961	0.97130	0.97308
941	0.96229	0.96588	0.96748	0.96799	0.96963	0.97132	0.97310
942	0.96233	0.96592	0.96751	0.96802	0.96966	0.97135	0.97313
943	0.96236	0.96595	0.96754	0.96805	0.96969	0.97138	0.97315
944	0.96240	0.96598	0.96757	0.96808	0.96972	0.97140	0.97317
945	0.96243	0.96601	0.96760	0.96811	0.96975	0.97143	0.97320
946	0.96247	0.96604	0.96763	0.96814	0.96977	0.97145	0.97322
947	0.96250	0.96607	0.96766	0.96817	0.96980	0.97148	0.97325
948	0.96254	0.96611	0.96768	0.96820	0.96983	0.97151	0.97327
949	0.96257	0.96614	0.96771	0.96823	0.96986	0.97153	0.97329
950	0.96260	0.96617	0.96774	0.96826	0.96988	0.97156	0.97332
951	0.96264	0.96620	0.96777	0.96829	0.96991	0.97158	0.97334
952	0.96267	0.96623	0.96780	0.96831	0.96994	0.97161	0.97336
953	0.96271	0.96626	0.96783	0.96834	0.96997	0.97163	0.97339
954	0.96274	0.96629	0.96786	0.96837	0.96999	0.97166	0.97341
955	0.96278	0.96632	0.96789	0.96840	0.97002	0.97168	0.97344
956	0.96281	0.96635	0.96792	0.96843	0.97005	0.97171	0.97346
957	0.96285	0.96638	0.96795	0.96846	0.97008	0.97174	0.97348
958	0.96288	0.96642	0.96798	0.96849	0.97010	0.97176	0.97351
959	0.96291	0.96645	0.96801	0.96852	0.97013	0.97179	0.97353
960	0.96294	0.96648	0.96804	0.96855	0.97016	0.97181	0.97355
961	0.96298	0.96651	0.96807	0.96857	0.97018	0.97184	0.97358
962	0.96302	0.96654	0.96810	0.96860	0.97021	0.97186	0.97360
963	0.96305	0.96657	0.96812	0.96863	0.97023	0.97189	0.97362
964	0.96308	0.96660	0.96815	0.96866	0.97026	0.97191	0.97365
965	0.96312	0.96663	0.96818	0.96869	0.97029	0.97194	0.97367
966	0.96315	0.96666	0.96821	0.96871	0.97031	0.97196	0.97369
967	0.96318	0.96669	0.96824	0.96874	0.97034	0.97199	0.97372
968	0.96322	0.96672	0.96827	0.96877	0.97037	0.97201	0.97374
969	0.96326	0.96675	0.96830	0.96880	0.97039	0.97203	0.97376
970	0.96329	0.96678	0.96832	0.96882	0.97042	0.97206	0.97378
971	0.96332	0.96681	0.96835	0.96885	0.97044	0.97208	0.97381
972	0.96335	0.96684	0.96838	0.96888	0.97047	0.97211	0.97383
973	0.96339	0.96686	0.96841	0.96891	0.97050	0.97213	0.97385
974	0.96342	0.96690	0.96844	0.96894	0.97052	0.97216	0.97388
975	0.96346	0.96693	0.96847	0.96896	0.97055	0.97218	0.97390
976	0.96349	0.96695	0.96849	0.96899	0.97057	0.97221	0.97392
977	0.96352	0.96698	0.96852	0.96902	0.97060	0.97223	0.97394
978	0.96355	0.96701	0.96855	0.96905	0.97063	0.97225	0.97397
979	0.96358	0.96704	0.96858	0.96907	0.97065	0.97228	0.97399
980	0.96361	0.96707	0.96861	0.96910	0.97068	0.97230	0.97401
981	0.96365	0.96710	0.96863	0.96913	0.97070	0.97233	0.97404
982	0.96368	0.96713	0.96866	0.96916	0.97073	0.97235	0.97406
983	0.96371	0.96716	0.96869	0.96918	0.97076	0.97238	0.97408
984	0.96375	0.96719	0.96872	0.96921	0.97078	0.97240	0.97410
985	0.96378	0.96722	0.96875	0.96924	0.97081	0.97243	0.97413
986	0.96381	0.96725	0.96877	0.96926	0.97083	0.97245	0.97415
987	0.96385	0.96728	0.96880	0.96929	0.97086	0.97247	0.97417
988	0.96388	0.96731	0.96883	0.96932	0.97088	0.97250	0.97419
989	0.96391	0.96734	0.96886	0.96935	0.97091	0.97252	0.97421

N	0.1%	0.5%	1%	1.25%	2.5%	5%	10%
990	0.96394	0.96737	0.96888	0.96938	0.97093	0.97255	0.97424
991	0.96398	0.96740	0.96891	0.96940	0.97096	0.97257	0.97426
992	0.96400	0.96742	0.96894	0.96943	0.97099	0.97259	0.97428
993	0.96404	0.96745	0.96897	0.96946	0.97101	0.97262	0.97430
994	0.96407	0.96748	0.96899	0.96948	0.97104	0.97264	0.97432
995	0.96410	0.96751	0.96902	0.96951	0.97106	0.97266	0.97435
996	0.96413	0.96754	0.96905	0.96954	0.97109	0.97269	0.97437
997	0.96417	0.96757	0.96907	0.96956	0.97111	0.97271	0.97439
998	0.96420	0.96760	0.96910	0.96959	0.97114	0.97273	0.97441
999	0.96423	0.96763	0.96913	0.96962	0.97116	0.97276	0.97443
1000	0.96426	0.96766	0.96916	0.96964	0.97119	0.97278	0.97445
