# Supplementary material for: A generalized outlier method with an automated test protocol applied to proficiency testing schemes: utilizing Monte Carlo simulation for generating critical values for the extended Grubbs statistic
Source: J AOAC Int. 2026 Apr 29;109(6):974–84. doi: 10.1093/jaoacint/qsag036 (PMC13732683; doi:10.1093/jaoacint/qsag036)
Supplement: Supplementary file 1 [file qsag036_supplementary_data.zip › aoac-25-0307-File015.pdf]

Extended Grubbs Statistics Tables for Triple Outliers

Table 1 – *GT(HHH or LLL)*. Critical values of upper significance levels for the Grubbs triple outlier test, applicable in situations where the three highest or three lowest observations are simultaneously identified as outliers, denoted as *GT(HHH)* and *GT(LLL)*, respectively.

N	0.1%	0.5%	1%	1.25%	2.5%	5%	10%
5	0.00000	0.00000	0.00001	0.00002	0.00009	0.00037	0.00152
6	0.00018	0.00092	0.00187	0.00235	0.00479	0.00987	0.02066
7	0.00219	0.00656	0.01059	0.01237	0.02013	0.03305	0.05504
8	0.00801	0.01846	0.02660	0.02995	0.04347	0.06360	0.09417
9	0.01802	0.03541	0.04761	0.05242	0.07093	0.09669	0.13321
10	0.03152	0.05562	0.07138	0.07742	0.09993	0.12982	0.17030
11	0.04752	0.07766	0.09638	0.10338	0.12895	0.16181	0.20482
12	0.06524	0.10053	0.12160	0.12937	0.15725	0.19215	0.23673
13	0.08395	0.12355	0.14648	0.15483	0.18438	0.22068	0.26613
14	0.10313	0.14627	0.17063	0.17942	0.21020	0.24739	0.29323
15	0.12242	0.16844	0.19390	0.20300	0.23462	0.27235	0.31823
16	0.14160	0.18990	0.21618	0.22551	0.25768	0.29566	0.34135
17	0.16041	0.21057	0.23744	0.24693	0.27944	0.31746	0.36278
18	0.17880	0.23039	0.25769	0.26727	0.29994	0.33785	0.38269
19	0.19670	0.24938	0.27694	0.28658	0.31928	0.35697	0.40124
20	0.21405	0.26753	0.29526	0.30491	0.33753	0.37493	0.41856
21	0.23081	0.28491	0.31267	0.32231	0.35478	0.39179	0.43477
22	0.24703	0.30147	0.32924	0.33884	0.37111	0.40769	0.44997
23	0.26266	0.31731	0.34501	0.35456	0.38655	0.42269	0.46427
24	0.27768	0.33244	0.36002	0.36952	0.40120	0.43686	0.47774
25	0.29216	0.34692	0.37433	0.38374	0.41509	0.45027	0.49044
26	0.30610	0.36077	0.38798	0.39730	0.42831	0.46298	0.50246
27	0.31954	0.37403	0.40100	0.41024	0.44088	0.47505	0.51384
28	0.33248	0.38669	0.41346	0.42259	0.45287	0.48652	0.52463
29	0.34494	0.39885	0.42535	0.43439	0.46428	0.49744	0.53489
30	0.35692	0.41050	0.43674	0.44568	0.47519	0.50784	0.54464
31	0.36848	0.42168	0.44766	0.45649	0.48562	0.51778	0.55394
32	0.37963	0.43243	0.45812	0.46685	0.49560	0.52727	0.56281
33	0.39041	0.44275	0.46816	0.47678	0.50514	0.53634	0.57128
34	0.40078	0.45268	0.47781	0.48632	0.51430	0.54502	0.57938
35	0.41082	0.46223	0.48709	0.49549	0.52308	0.55335	0.58714
36	0.42050	0.47144	0.49600	0.50430	0.53152	0.56133	0.59457
37	0.42986	0.48031	0.50457	0.51278	0.53963	0.56900	0.60171
38	0.43887	0.48887	0.51285	0.52094	0.54743	0.57638	0.60856
39	0.44764	0.49710	0.52082	0.52881	0.55495	0.58347	0.61514
40	0.45610	0.50508	0.52850	0.53639	0.56219	0.59030	0.62148
41	0.46427	0.51278	0.53593	0.54372	0.56917	0.59687	0.62758
42	0.47220	0.52022	0.54309	0.55078	0.57591	0.60322	0.63346
43	0.47991	0.52742	0.55002	0.55762	0.58241	0.60935	0.63913
44	0.48734	0.53439	0.55672	0.56422	0.58870	0.61526	0.64460
45	0.49458	0.54113	0.56320	0.57062	0.59477	0.62097	0.64988
46	0.50158	0.54767	0.56948	0.57680	0.60066	0.62649	0.65499
47	0.50838	0.55400	0.57557	0.58280	0.60635	0.63184	0.65993
48	0.51502	0.56015	0.58146	0.58861	0.61186	0.63701	0.66471
49	0.52143	0.56611	0.58718	0.59424	0.61721	0.64202	0.66934
50	0.52766	0.57190	0.59272	0.59970	0.62238	0.64688	0.67382
51	0.53375	0.57752	0.59811	0.60501	0.62741	0.65159	0.67817
52	0.53966	0.58299	0.60334	0.61016	0.63229	0.65617	0.68239
53	0.54543	0.58829	0.60842	0.61516	0.63704	0.66061	0.68648
54	0.55101	0.59346	0.61337	0.62003	0.64164	0.66492	0.69045
55	0.55644	0.59848	0.61817	0.62475	0.64612	0.66911	0.69432
56	0.56175	0.60336	0.62284	0.62936	0.65047	0.67318	0.69807
57	0.56692	0.60812	0.62739	0.63383	0.65470	0.67714	0.70172
58	0.57197	0.61276	0.63182	0.63819	0.65882	0.68100	0.70527
59	0.57688	0.61728	0.63614	0.64244	0.66283	0.68475	0.70872
60	0.58169	0.62168	0.64035	0.64657	0.66674	0.68841	0.71209
61	0.58637	0.62598	0.64444	0.65060	0.67055	0.69196	0.71537
62	0.59095	0.63017	0.64843	0.65453	0.67426	0.69543	0.71856
63	0.59542	0.63425	0.65233	0.65836	0.67788	0.69881	0.72167
64	0.59978	0.63823	0.65613	0.66210	0.68141	0.70211	0.72471
65	0.60403	0.64213	0.65984	0.66576	0.68485	0.70533	0.72767
66	0.60820	0.64593	0.66347	0.66933	0.68822	0.70847	0.73056
67	0.61226	0.64965	0.66702	0.67281	0.69150	0.71154	0.73338
68	0.61625	0.65328	0.67048	0.67621	0.69471	0.71453	0.73613
69	0.62014	0.65683	0.67386	0.67953	0.69785	0.71746	0.73882
70	0.62395	0.66030	0.67716	0.68278	0.70091	0.72032	0.74145

N	0.1%	0.5%	1%	1.25%	2.5%	5%	10%
71	0.62769	0.66370	0.68040	0.68596	0.70391	0.72311	0.74402
72	0.63133	0.66702	0.68356	0.68906	0.70683	0.72584	0.74653
73	0.63489	0.67027	0.68665	0.69210	0.70970	0.72851	0.74899
74	0.63838	0.67345	0.68968	0.69508	0.71250	0.73113	0.75139
75	0.64180	0.67657	0.69264	0.69799	0.71525	0.73369	0.75375
76	0.64517	0.67961	0.69554	0.70084	0.71793	0.73619	0.75605
77	0.64846	0.68260	0.69838	0.70363	0.72056	0.73864	0.75830
78	0.65170	0.68552	0.70117	0.70637	0.72314	0.74105	0.76051
79	0.65485	0.68839	0.70389	0.70905	0.72566	0.74340	0.76267
80	0.65796	0.69120	0.70657	0.71168	0.72814	0.74571	0.76479
81	0.66099	0.69396	0.70919	0.71425	0.73056	0.74796	0.76687
82	0.66396	0.69666	0.71176	0.71678	0.73294	0.75018	0.76890
83	0.66688	0.69931	0.71428	0.71925	0.73527	0.75235	0.77090
84	0.66975	0.70192	0.71675	0.72168	0.73756	0.75448	0.77285
85	0.67257	0.70447	0.71918	0.72406	0.73980	0.75657	0.77477
86	0.67533	0.70697	0.72155	0.72640	0.74200	0.75862	0.77666
87	0.67804	0.70944	0.72389	0.72869	0.74416	0.76063	0.77850
88	0.68071	0.71185	0.72618	0.73094	0.74627	0.76260	0.78032
89	0.68331	0.71422	0.72844	0.73316	0.74836	0.76454	0.78210
90	0.68589	0.71655	0.73065	0.73533	0.75040	0.76645	0.78385
91	0.68841	0.71883	0.73282	0.73746	0.75241	0.76832	0.78556
92	0.69090	0.72108	0.73495	0.73956	0.75438	0.77015	0.78725
93	0.69334	0.72328	0.73705	0.74162	0.75631	0.77196	0.78891
94	0.69574	0.72546	0.73911	0.74364	0.75822	0.77373	0.79053
95	0.69809	0.72759	0.74113	0.74563	0.76009	0.77547	0.79213
96	0.70042	0.72969	0.74312	0.74758	0.76193	0.77718	0.79371
97	0.70270	0.73175	0.74508	0.74950	0.76373	0.77887	0.79525
98	0.70494	0.73378	0.74701	0.75139	0.76551	0.78052	0.79677
99	0.70715	0.73577	0.74890	0.75326	0.76726	0.78215	0.79827
100	0.70932	0.73774	0.75076	0.75508	0.76897	0.78375	0.79974
101	0.71146	0.73966	0.75259	0.75688	0.77067	0.78532	0.80118
102	0.71357	0.74156	0.75439	0.75865	0.77233	0.78687	0.80261
103	0.71564	0.74343	0.75617	0.76039	0.77397	0.78840	0.80401
104	0.71768	0.74527	0.75791	0.76211	0.77558	0.78990	0.80538
105	0.71969	0.74708	0.75963	0.76379	0.77717	0.79138	0.80674
106	0.72167	0.74886	0.76132	0.76545	0.77873	0.79283	0.80808
107	0.72361	0.75062	0.76299	0.76709	0.78027	0.79426	0.80939
108	0.72552	0.75235	0.76463	0.76870	0.78178	0.79567	0.81069
109	0.72742	0.75405	0.76625	0.77029	0.78327	0.79706	0.81196
110	0.72928	0.75573	0.76784	0.77185	0.78474	0.79843	0.81322
111	0.73111	0.75738	0.76941	0.77339	0.78618	0.79977	0.81445
112	0.73291	0.75901	0.77095	0.77491	0.78761	0.80110	0.81567
113	0.73469	0.76062	0.77247	0.77640	0.78902	0.80241	0.81688
114	0.73645	0.76220	0.77397	0.77788	0.79040	0.80369	0.81806
115	0.73818	0.76375	0.77545	0.77933	0.79176	0.80497	0.81923
116	0.73988	0.76529	0.77691	0.78076	0.79311	0.80622	0.82038
117	0.74157	0.76680	0.77835	0.78217	0.79443	0.80745	0.82151
118	0.74323	0.76830	0.77976	0.78356	0.79574	0.80867	0.82263
119	0.74487	0.76977	0.78116	0.78493	0.79703	0.80987	0.82373
120	0.74648	0.77122	0.78254	0.78628	0.79830	0.81105	0.82482
121	0.74807	0.77266	0.78390	0.78762	0.79955	0.81222	0.82589
122	0.74964	0.77407	0.78524	0.78893	0.80079	0.81337	0.82695
123	0.75119	0.77546	0.78656	0.79023	0.80201	0.81450	0.82799
124	0.75272	0.77684	0.78786	0.79151	0.80321	0.81562	0.82902
125	0.75423	0.77820	0.78915	0.79277	0.80440	0.81673	0.83004
126	0.75572	0.77954	0.79042	0.79402	0.80557	0.81782	0.83104
127	0.75718	0.78086	0.79167	0.79525	0.80673	0.81890	0.83203
128	0.75863	0.78216	0.79291	0.79646	0.80787	0.81996	0.83300
129	0.76005	0.78345	0.79413	0.79766	0.80899	0.82101	0.83397
130	0.76146	0.78472	0.79534	0.79885	0.81011	0.82204	0.83492
131	0.76286	0.78598	0.79653	0.80002	0.81120	0.82306	0.83586
132	0.76424	0.78722	0.79770	0.80117	0.81229	0.82407	0.83679
133	0.76560	0.78844	0.79886	0.80231	0.81336	0.82507	0.83770
134	0.76695	0.78965	0.80001	0.80343	0.81441	0.82605	0.83861
135	0.76827	0.79085	0.80114	0.80454	0.81546	0.82702	0.83950
136	0.76958	0.79203	0.80225	0.80564	0.81649	0.82798	0.84038
137	0.77088	0.79319	0.80336	0.80672	0.81751	0.82893	0.84125
138	0.77216	0.79434	0.80445	0.80779	0.81851	0.82987	0.84211
139	0.77342	0.79548	0.80553	0.80885	0.81951	0.83079	0.84296
140	0.77467	0.79660	0.80659	0.80990	0.82049	0.83171	0.84380
141	0.77590	0.79771	0.80764	0.81093	0.82146	0.83261	0.84463
142	0.77712	0.79881	0.80868	0.81195	0.82242	0.83350	0.84545
143	0.77833	0.79989	0.80971	0.81296	0.82336	0.83438	0.84626
144	0.77952	0.80096	0.81072	0.81395	0.82430	0.83525	0.84706

N	0.1%	0.5%	1%	1.25%	2.5%	5%	10%
145	0.78070	0.80201	0.81173	0.81494	0.82523	0.83612	0.84786
146	0.78186	0.80306	0.81272	0.81591	0.82614	0.83697	0.84864
147	0.78301	0.80410	0.81370	0.81687	0.82704	0.83781	0.84941
148	0.78415	0.80512	0.81467	0.81782	0.82794	0.83864	0.85018
149	0.78527	0.80613	0.81563	0.81876	0.82882	0.83946	0.85094
150	0.78639	0.80713	0.81657	0.81969	0.82969	0.84028	0.85168
151	0.78749	0.80812	0.81751	0.82061	0.83056	0.84108	0.85242
152	0.78858	0.80910	0.81843	0.82152	0.83141	0.84188	0.85315
153	0.78965	0.81006	0.81935	0.82242	0.83226	0.84266	0.85388
154	0.79071	0.81102	0.82026	0.82331	0.83309	0.84344	0.85459
155	0.79176	0.81197	0.82115	0.82419	0.83392	0.84421	0.85530
156	0.79281	0.81290	0.82204	0.82506	0.83474	0.84497	0.85600
157	0.79384	0.81383	0.82292	0.82592	0.83555	0.84573	0.85670
158	0.79485	0.81474	0.82378	0.82677	0.83634	0.84647	0.85738
159	0.79587	0.81565	0.82464	0.82761	0.83714	0.84721	0.85806
160	0.79686	0.81654	0.82549	0.82845	0.83792	0.84794	0.85873
161	0.79785	0.81743	0.82633	0.82927	0.83869	0.84866	0.85939
162	0.79883	0.81831	0.82716	0.83009	0.83946	0.84937	0.86005
163	0.79980	0.81918	0.82798	0.83090	0.84022	0.85008	0.86070
164	0.80075	0.82004	0.82880	0.83169	0.84097	0.85078	0.86134
165	0.80170	0.82088	0.82960	0.83248	0.84171	0.85147	0.86198
166	0.80263	0.82172	0.83040	0.83327	0.84245	0.85216	0.86261
167	0.80355	0.82255	0.83119	0.83404	0.84318	0.85284	0.86323
168	0.80448	0.82338	0.83197	0.83481	0.84390	0.85351	0.86385
169	0.80538	0.82420	0.83274	0.83557	0.84461	0.85417	0.86446
170	0.80628	0.82500	0.83351	0.83632	0.84532	0.85483	0.86507
171	0.80717	0.82581	0.83427	0.83706	0.84602	0.85548	0.86567
172	0.80805	0.82660	0.83502	0.83780	0.84671	0.85613	0.86626
173	0.80892	0.82738	0.83576	0.83853	0.84740	0.85677	0.86685
174	0.80979	0.82816	0.83650	0.83925	0.84807	0.85740	0.86743
175	0.81065	0.82893	0.83723	0.83997	0.84875	0.85803	0.86801
176	0.81149	0.82969	0.83795	0.84068	0.84941	0.85865	0.86858
177	0.81233	0.83044	0.83866	0.84138	0.85007	0.85926	0.86915
178	0.81316	0.83119	0.83937	0.84207	0.85073	0.85987	0.86971
179	0.81398	0.83193	0.84007	0.84276	0.85137	0.86047	0.87026
180	0.81480	0.83266	0.84077	0.84344	0.85201	0.86107	0.87081
181	0.81561	0.83339	0.84146	0.84412	0.85265	0.86166	0.87135
182	0.81641	0.83411	0.84214	0.84479	0.85328	0.86224	0.87189
183	0.81720	0.83482	0.84281	0.84545	0.85390	0.86282	0.87243
184	0.81799	0.83553	0.84348	0.84611	0.85452	0.86340	0.87296
185	0.81877	0.83623	0.84414	0.84676	0.85513	0.86397	0.87348
186	0.81954	0.83692	0.84480	0.84740	0.85573	0.86453	0.87400
187	0.82031	0.83761	0.84545	0.84804	0.85633	0.86509	0.87452
188	0.82106	0.83829	0.84609	0.84867	0.85693	0.86565	0.87503
189	0.82182	0.83896	0.84673	0.84930	0.85752	0.86620	0.87553
190	0.82256	0.83963	0.84737	0.84992	0.85810	0.86674	0.87603
191	0.82329	0.84029	0.84799	0.85054	0.85868	0.86728	0.87653
192	0.82403	0.84095	0.84862	0.85115	0.85925	0.86781	0.87702
193	0.82475	0.84160	0.84923	0.85175	0.85982	0.86834	0.87751
194	0.82547	0.84224	0.84984	0.85235	0.86039	0.86887	0.87800
195	0.82618	0.84288	0.85045	0.85295	0.86094	0.86939	0.87848
196	0.82688	0.84351	0.85105	0.85354	0.86150	0.86991	0.87895
197	0.82758	0.84414	0.85164	0.85412	0.86205	0.87042	0.87942
198	0.82828	0.84476	0.85223	0.85470	0.86259	0.87093	0.87989
199	0.82896	0.84538	0.85282	0.85527	0.86313	0.87143	0.88035
200	0.82964	0.84599	0.85340	0.85584	0.86367	0.87193	0.88081
201	0.83035	0.84659	0.85395	0.85638	0.86419	0.87239	0.88125
202	0.83101	0.84719	0.85451	0.85694	0.86471	0.87288	0.88171
203	0.83164	0.84779	0.85507	0.85750	0.86522	0.87337	0.88215
204	0.83234	0.84839	0.85563	0.85804	0.86574	0.87386	0.88260
205	0.83299	0.84897	0.85617	0.85859	0.86625	0.87434	0.88304
206	0.83368	0.84956	0.85673	0.85913	0.86675	0.87481	0.88348
207	0.83431	0.85013	0.85729	0.85966	0.86726	0.87528	0.88391
208	0.83497	0.85071	0.85784	0.86021	0.86776	0.87575	0.88434
209	0.83554	0.85127	0.85837	0.86072	0.86825	0.87621	0.88477
210	0.83615	0.85183	0.85889	0.86125	0.86874	0.87667	0.88519
211	0.83678	0.85241	0.85943	0.86175	0.86923	0.87713	0.88560
212	0.83740	0.85296	0.85996	0.86226	0.86971	0.87758	0.88602
213	0.83801	0.85348	0.86048	0.86279	0.87019	0.87802	0.88643
214	0.83866	0.85402	0.86100	0.86331	0.87067	0.87847	0.88684
215	0.83925	0.85456	0.86150	0.86380	0.87114	0.87891	0.88725
216	0.83983	0.85510	0.86202	0.86430	0.87161	0.87935	0.88765
217	0.84049	0.85564	0.86251	0.86477	0.87207	0.87978	0.88805
218	0.84110	0.85615	0.86301	0.86527	0.87254	0.88021	0.88845

N	0.1%	0.5%	1%	1.25%	2.5%	5%	10%
219	0.84165	0.85666	0.86350	0.86577	0.87299	0.88064	0.88884
220	0.84221	0.85718	0.86398	0.86624	0.87345	0.88106	0.88923
221	0.84279	0.85770	0.86448	0.86672	0.87389	0.88148	0.88962
222	0.84332	0.85821	0.86497	0.86721	0.87434	0.88189	0.88999
223	0.84390	0.85872	0.86545	0.86767	0.87479	0.88230	0.89038
224	0.84446	0.85922	0.86593	0.86815	0.87524	0.88271	0.89076
225	0.84498	0.85973	0.86640	0.86862	0.87567	0.88312	0.89113
226	0.84555	0.86021	0.86687	0.86908	0.87611	0.88352	0.89150
227	0.84606	0.86072	0.86733	0.86954	0.87653	0.88393	0.89187
228	0.84660	0.86120	0.86780	0.86999	0.87695	0.88433	0.89224
229	0.84714	0.86167	0.86826	0.87043	0.87737	0.88472	0.89261
230	0.84769	0.86215	0.86873	0.87088	0.87781	0.88511	0.89297
231	0.84824	0.86262	0.86918	0.87133	0.87822	0.88550	0.89333
232	0.84872	0.86309	0.86962	0.87177	0.87863	0.88588	0.89368
233	0.84924	0.86358	0.87007	0.87221	0.87904	0.88627	0.89403
234	0.84978	0.86404	0.87052	0.87265	0.87946	0.88665	0.89438
235	0.85029	0.86451	0.87097	0.87308	0.87986	0.88702	0.89473
236	0.85082	0.86496	0.87141	0.87349	0.88026	0.88739	0.89508
237	0.85130	0.86543	0.87184	0.87392	0.88066	0.88776	0.89542
238	0.85180	0.86588	0.87227	0.87435	0.88106	0.88814	0.89576
239	0.85233	0.86633	0.87268	0.87477	0.88145	0.88851	0.89609
240	0.85284	0.86679	0.87311	0.87518	0.88183	0.88887	0.89643
241	0.85333	0.86723	0.87351	0.87559	0.88222	0.88923	0.89676
242	0.85385	0.86768	0.87393	0.87600	0.88260	0.88959	0.89709
243	0.85431	0.86811	0.87436	0.87641	0.88299	0.88995	0.89742
244	0.85480	0.86855	0.87477	0.87681	0.88337	0.89030	0.89775
245	0.85525	0.86898	0.87518	0.87721	0.88375	0.89065	0.89807
246	0.85573	0.86941	0.87558	0.87761	0.88412	0.89100	0.89840
247	0.85619	0.86982	0.87599	0.87799	0.88450	0.89135	0.89872
248	0.85666	0.87024	0.87639	0.87839	0.88487	0.89169	0.89904
249	0.85713	0.87067	0.87679	0.87879	0.88524	0.89203	0.89935
250	0.85761	0.87110	0.87718	0.87917	0.88560	0.89237	0.89967
251	0.85804	0.87149	0.87757	0.87956	0.88596	0.89271	0.89997
252	0.85851	0.87188	0.87796	0.87994	0.88633	0.89305	0.90028
253	0.85897	0.87230	0.87834	0.88032	0.88669	0.89338	0.90059
254	0.85943	0.87269	0.87872	0.88070	0.88703	0.89371	0.90089
255	0.85991	0.87310	0.87910	0.88107	0.88739	0.89404	0.90120
256	0.86031	0.87351	0.87949	0.88145	0.88774	0.89436	0.90150
257	0.86073	0.87389	0.87986	0.88181	0.88808	0.89468	0.90180
258	0.86117	0.87429	0.88021	0.88218	0.88843	0.89500	0.90210
259	0.86160	0.87468	0.88059	0.88254	0.88878	0.89532	0.90239
260	0.86201	0.87505	0.88094	0.88289	0.88912	0.89564	0.90268
261	0.86245	0.87544	0.88131	0.88326	0.88945	0.89595	0.90297
262	0.86285	0.87582	0.88169	0.88362	0.88978	0.89627	0.90326
263	0.86327	0.87621	0.88205	0.88397	0.89012	0.89657	0.90355
264	0.86370	0.87657	0.88240	0.88432	0.89045	0.89688	0.90383
265	0.86410	0.87693	0.88276	0.88466	0.89078	0.89719	0.90412
266	0.86452	0.87731	0.88310	0.88500	0.89110	0.89749	0.90440
267	0.86493	0.87769	0.88346	0.88536	0.89143	0.89780	0.90468
268	0.86534	0.87806	0.88382	0.88570	0.89174	0.89809	0.90496
269	0.86574	0.87843	0.88418	0.88604	0.89206	0.89840	0.90523
270	0.86616	0.87878	0.88451	0.88639	0.89238	0.89869	0.90550
271	0.86654	0.87915	0.88485	0.88673	0.89269	0.89899	0.90578
272	0.86695	0.87951	0.88518	0.88706	0.89300	0.89928	0.90605
273	0.86734	0.87987	0.88552	0.88740	0.89331	0.89958	0.90631
274	0.86773	0.88023	0.88586	0.88772	0.89363	0.89987	0.90658
275	0.86818	0.88057	0.88619	0.88805	0.89393	0.90016	0.90685
276	0.86854	0.88092	0.88653	0.88838	0.89425	0.90044	0.90711
277	0.86889	0.88127	0.88686	0.88870	0.89454	0.90073	0.90738
278	0.86927	0.88162	0.88719	0.88903	0.89485	0.90101	0.90764
279	0.86965	0.88195	0.88751	0.88934	0.89515	0.90129	0.90790
280	0.87005	0.88230	0.88783	0.88966	0.89545	0.90157	0.90816
281	0.87039	0.88265	0.88815	0.88997	0.89575	0.90185	0.90841
282	0.87077	0.88300	0.88846	0.89028	0.89605	0.90212	0.90867
283	0.87119	0.88333	0.88878	0.89060	0.89634	0.90240	0.90892
284	0.87153	0.88366	0.88909	0.89091	0.89663	0.90267	0.90917
285	0.87190	0.88398	0.88941	0.89122	0.89691	0.90294	0.90942
286	0.87227	0.88430	0.88972	0.89152	0.89720	0.90321	0.90967
287	0.87262	0.88462	0.89003	0.89183	0.89748	0.90348	0.90992
288	0.87300	0.88494	0.89033	0.89213	0.89777	0.90374	0.91016
289	0.87336	0.88527	0.89063	0.89242	0.89806	0.90401	0.91041
290	0.87373	0.88560	0.89094	0.89272	0.89835	0.90427	0.91065
291	0.87408	0.88592	0.89125	0.89302	0.89862	0.90453	0.91089
292	0.87444	0.88623	0.89156	0.89332	0.89890	0.90479	0.91113

N	0.1%	0.5%	1%	1.25%	2.5%	5%	10%
293	0.87477	0.88656	0.89186	0.89360	0.89918	0.90505	0.91137
294	0.87512	0.88687	0.89215	0.89390	0.89946	0.90530	0.91160
295	0.87548	0.88718	0.89244	0.89418	0.89972	0.90556	0.91184
296	0.87582	0.88749	0.89274	0.89447	0.89999	0.90581	0.91207
297	0.87617	0.88781	0.89304	0.89476	0.90026	0.90606	0.91230
298	0.87652	0.88812	0.89331	0.89505	0.90053	0.90631	0.91254
299	0.87687	0.88842	0.89360	0.89533	0.90080	0.90656	0.91276
300	0.87720	0.88872	0.89389	0.89561	0.90106	0.90681	0.91299
301	0.87758	0.88902	0.89418	0.89588	0.90133	0.90706	0.91322
302	0.87792	0.88932	0.89447	0.89617	0.90159	0.90730	0.91345
303	0.87823	0.88960	0.89475	0.89645	0.90185	0.90755	0.91367
304	0.87856	0.88989	0.89503	0.89672	0.90211	0.90779	0.91389
305	0.87887	0.89018	0.89530	0.89699	0.90237	0.90803	0.91412
306	0.87921	0.89047	0.89559	0.89726	0.90263	0.90827	0.91434
307	0.87953	0.89077	0.89585	0.89753	0.90288	0.90851	0.91456
308	0.87988	0.89105	0.89613	0.89780	0.90313	0.90875	0.91478
309	0.88018	0.89134	0.89640	0.89806	0.90338	0.90898	0.91499
310	0.88050	0.89163	0.89666	0.89832	0.90363	0.90922	0.91521
311	0.88079	0.89191	0.89693	0.89860	0.90388	0.90945	0.91542
312	0.88112	0.89220	0.89721	0.89885	0.90413	0.90969	0.91564
313	0.88145	0.89247	0.89747	0.89912	0.90437	0.90991	0.91585
314	0.88172	0.89275	0.89773	0.89938	0.90461	0.91014	0.91606
315	0.88203	0.89303	0.89800	0.89964	0.90485	0.91037	0.91628
316	0.88231	0.89331	0.89826	0.89990	0.90509	0.91060	0.91648
317	0.88266	0.89360	0.89853	0.90015	0.90534	0.91083	0.91669
318	0.88295	0.89387	0.89878	0.90040	0.90557	0.91105	0.91690
319	0.88323	0.89412	0.89903	0.90065	0.90581	0.91127	0.91710
320	0.88354	0.89439	0.89928	0.90090	0.90605	0.91149	0.91731
321	0.88385	0.89466	0.89954	0.90116	0.90629	0.91171	0.91751
322	0.88414	0.89494	0.89979	0.90139	0.90651	0.91192	0.91771
323	0.88439	0.89521	0.90004	0.90164	0.90675	0.91214	0.91791
324	0.88471	0.89546	0.90028	0.90189	0.90698	0.91235	0.91811
325	0.88502	0.89573	0.90053	0.90213	0.90721	0.91257	0.91831
326	0.88532	0.89599	0.90078	0.90237	0.90744	0.91278	0.91851
327	0.88563	0.89625	0.90103	0.90261	0.90767	0.91300	0.91871
328	0.88587	0.89650	0.90127	0.90286	0.90790	0.91321	0.91890
329	0.88617	0.89676	0.90151	0.90309	0.90812	0.91342	0.91910
330	0.88644	0.89700	0.90175	0.90333	0.90834	0.91363	0.91929
331	0.88673	0.89726	0.90199	0.90356	0.90857	0.91384	0.91949
332	0.88701	0.89752	0.90223	0.90380	0.90879	0.91404	0.91967
333	0.88729	0.89777	0.90247	0.90404	0.90901	0.91425	0.91986
334	0.88758	0.89803	0.90272	0.90427	0.90923	0.91445	0.92005
335	0.88781	0.89829	0.90295	0.90451	0.90945	0.91466	0.92024
336	0.88810	0.89853	0.90318	0.90473	0.90967	0.91486	0.92043
337	0.88837	0.89878	0.90342	0.90496	0.90988	0.91506	0.92062
338	0.88867	0.89903	0.90364	0.90520	0.91010	0.91526	0.92081
339	0.88894	0.89926	0.90388	0.90542	0.91031	0.91546	0.92099
340	0.88921	0.89951	0.90411	0.90565	0.91052	0.91566	0.92118
341	0.88946	0.89974	0.90434	0.90587	0.91073	0.91586	0.92136
342	0.88973	0.89997	0.90457	0.90610	0.91094	0.91606	0.92154
343	0.88999	0.90021	0.90479	0.90632	0.91115	0.91626	0.92173
344	0.89025	0.90044	0.90503	0.90654	0.91136	0.91645	0.92191
345	0.89051	0.90068	0.90525	0.90676	0.91157	0.91664	0.92208
346	0.89077	0.90093	0.90547	0.90698	0.91178	0.91683	0.92226
347	0.89105	0.90116	0.90569	0.90719	0.91199	0.91703	0.92244
348	0.89132	0.90139	0.90590	0.90741	0.91219	0.91722	0.92261
349	0.89156	0.90162	0.90612	0.90762	0.91239	0.91741	0.92279
350	0.89181	0.90184	0.90635	0.90784	0.91259	0.91760	0.92297
351	0.89206	0.90207	0.90656	0.90806	0.91280	0.91779	0.92315
352	0.89232	0.90230	0.90679	0.90827	0.91299	0.91797	0.92332
353	0.89260	0.90254	0.90700	0.90848	0.91319	0.91816	0.92349
354	0.89284	0.90276	0.90721	0.90869	0.91339	0.91834	0.92366
355	0.89308	0.90298	0.90743	0.90890	0.91359	0.91853	0.92383
356	0.89333	0.90319	0.90764	0.90910	0.91379	0.91871	0.92400
357	0.89357	0.90343	0.90786	0.90931	0.91398	0.91889	0.92417
358	0.89382	0.90364	0.90808	0.90952	0.91418	0.91907	0.92434
359	0.89407	0.90387	0.90828	0.90972	0.91437	0.91925	0.92450
360	0.89431	0.90407	0.90849	0.90993	0.91457	0.91944	0.92467
361	0.89455	0.90430	0.90869	0.91013	0.91476	0.91962	0.92483
362	0.89480	0.90452	0.90890	0.91033	0.91495	0.91979	0.92500
363	0.89505	0.90474	0.90911	0.91053	0.91514	0.91997	0.92516
364	0.89528	0.90495	0.90931	0.91073	0.91533	0.92015	0.92533
365	0.89551	0.90516	0.90952	0.91094	0.91552	0.92033	0.92549
366	0.89575	0.90537	0.90971	0.91114	0.91570	0.92050	0.92565

N	0.1%	0.5%	1%	1.25%	2.5%	5%	10%
367	0.89596	0.90559	0.90991	0.91133	0.91589	0.92067	0.92581
368	0.89619	0.90580	0.91011	0.91153	0.91607	0.92085	0.92597
369	0.89644	0.90601	0.91031	0.91173	0.91625	0.92102	0.92613
370	0.89666	0.90622	0.91050	0.91192	0.91644	0.92119	0.92629
371	0.89690	0.90643	0.91070	0.91212	0.91662	0.92136	0.92645
372	0.89712	0.90664	0.91090	0.91231	0.91680	0.92153	0.92660
373	0.89735	0.90684	0.91109	0.91249	0.91698	0.92170	0.92676
374	0.89759	0.90705	0.91129	0.91269	0.91716	0.92187	0.92692
375	0.89783	0.90725	0.91148	0.91288	0.91734	0.92203	0.92707
376	0.89806	0.90745	0.91167	0.91306	0.91752	0.92220	0.92723
377	0.89829	0.90766	0.91186	0.91325	0.91770	0.92237	0.92738
378	0.89851	0.90786	0.91205	0.91343	0.91788	0.92253	0.92753
379	0.89874	0.90806	0.91224	0.91362	0.91806	0.92269	0.92768
380	0.89897	0.90826	0.91243	0.91381	0.91823	0.92286	0.92784
381	0.89918	0.90846	0.91262	0.91400	0.91841	0.92302	0.92799
382	0.89938	0.90865	0.91282	0.91418	0.91858	0.92318	0.92814
383	0.89962	0.90885	0.91300	0.91436	0.91875	0.92334	0.92829
384	0.89980	0.90904	0.91318	0.91455	0.91892	0.92351	0.92844
385	0.90001	0.90923	0.91337	0.91473	0.91909	0.92366	0.92859
386	0.90025	0.90942	0.91355	0.91491	0.91926	0.92382	0.92874
387	0.90046	0.90961	0.91373	0.91508	0.91943	0.92398	0.92888
388	0.90068	0.90980	0.91392	0.91527	0.91960	0.92414	0.92903
389	0.90090	0.90999	0.91410	0.91544	0.91976	0.92430	0.92918
390	0.90110	0.91018	0.91427	0.91562	0.91993	0.92446	0.92932
391	0.90134	0.91037	0.91445	0.91579	0.92010	0.92461	0.92946
392	0.90157	0.91057	0.91462	0.91596	0.92027	0.92476	0.92961
393	0.90176	0.91075	0.91480	0.91614	0.92043	0.92492	0.92975
394	0.90197	0.91094	0.91498	0.91632	0.92059	0.92507	0.92989
395	0.90217	0.91112	0.91516	0.91649	0.92076	0.92523	0.93004
396	0.90238	0.91130	0.91533	0.91667	0.92092	0.92538	0.93018
397	0.90258	0.91149	0.91550	0.91684	0.92108	0.92553	0.93032
398	0.90278	0.91167	0.91568	0.91701	0.92124	0.92568	0.93046
399	0.90298	0.91186	0.91585	0.91718	0.92140	0.92583	0.93059
400	0.90318	0.91204	0.91603	0.91734	0.92156	0.92598	0.93073
401	0.90338	0.91221	0.91620	0.91751	0.92172	0.92613	0.93087
402	0.90358	0.91239	0.91637	0.91768	0.92188	0.92628	0.93101
403	0.90379	0.91257	0.91655	0.91785	0.92204	0.92643	0.93114
404	0.90400	0.91274	0.91672	0.91801	0.92220	0.92657	0.93128
405	0.90421	0.91292	0.91688	0.91818	0.92235	0.92672	0.93142
406	0.90439	0.91310	0.91705	0.91835	0.92250	0.92687	0.93155
407	0.90460	0.91327	0.91722	0.91852	0.92266	0.92701	0.93168
408	0.90478	0.91345	0.91739	0.91868	0.92281	0.92716	0.93182
409	0.90499	0.91363	0.91755	0.91884	0.92296	0.92730	0.93195
410	0.90521	0.91381	0.91772	0.91901	0.92312	0.92744	0.93208
411	0.90538	0.91398	0.91788	0.91916	0.92327	0.92758	0.93222
412	0.90557	0.91416	0.91804	0.91932	0.92342	0.92773	0.93235
413	0.90576	0.91432	0.91820	0.91949	0.92357	0.92787	0.93248
414	0.90594	0.91450	0.91837	0.91965	0.92372	0.92801	0.93261
415	0.90612	0.91467	0.91853	0.91981	0.92387	0.92815	0.93274
416	0.90630	0.91485	0.91869	0.91997	0.92403	0.92829	0.93287
417	0.90648	0.91500	0.91885	0.92012	0.92417	0.92843	0.93300
418	0.90669	0.91517	0.91901	0.92028	0.92432	0.92857	0.93313
419	0.90687	0.91535	0.91917	0.92043	0.92447	0.92871	0.93325
420	0.90704	0.91552	0.91933	0.92059	0.92461	0.92884	0.93338
421	0.90724	0.91568	0.91949	0.92075	0.92476	0.92898	0.93351
422	0.90742	0.91585	0.91965	0.92090	0.92491	0.92912	0.93363
423	0.90761	0.91601	0.91980	0.92105	0.92505	0.92925	0.93376
424	0.90778	0.91616	0.91995	0.92121	0.92519	0.92939	0.93388
425	0.90797	0.91633	0.92011	0.92136	0.92533	0.92952	0.93401
426	0.90816	0.91649	0.92026	0.92151	0.92547	0.92965	0.93413
427	0.90833	0.91665	0.92042	0.92165	0.92562	0.92978	0.93426
428	0.90851	0.91682	0.92057	0.92181	0.92576	0.92992	0.93438
429	0.90869	0.91697	0.92072	0.92195	0.92590	0.93005	0.93450
430	0.90886	0.91713	0.92087	0.92210	0.92604	0.93018	0.93462
431	0.90905	0.91729	0.92103	0.92225	0.92618	0.93031	0.93474
432	0.90921	0.91744	0.92117	0.92240	0.92632	0.93044	0.93486
433	0.90939	0.91760	0.92132	0.92255	0.92646	0.93057	0.93499
434	0.90957	0.91777	0.92148	0.92269	0.92660	0.93070	0.93511
435	0.90976	0.91792	0.92163	0.92284	0.92674	0.93083	0.93523
436	0.90994	0.91808	0.92177	0.92299	0.92687	0.93096	0.93535
437	0.91011	0.91824	0.92193	0.92313	0.92701	0.93109	0.93547
438	0.91028	0.91839	0.92207	0.92328	0.92714	0.93121	0.93558
439	0.91045	0.91854	0.92222	0.92342	0.92728	0.93134	0.93570
440	0.91061	0.91870	0.92236	0.92356	0.92741	0.93147	0.93582

N	0.1%	0.5%	1%	1.25%	2.5%	5%	10%
441	0.91077	0.91886	0.92250	0.92370	0.92754	0.93159	0.93594
442	0.91095	0.91901	0.92265	0.92384	0.92768	0.93172	0.93605
443	0.91111	0.91916	0.92279	0.92399	0.92781	0.93184	0.93617
444	0.91126	0.91931	0.92294	0.92413	0.92795	0.93197	0.93628
445	0.91144	0.91947	0.92308	0.92427	0.92808	0.93209	0.93640
446	0.91160	0.91962	0.92322	0.92441	0.92821	0.93222	0.93651
447	0.91177	0.91977	0.92336	0.92455	0.92834	0.93234	0.93663
448	0.91194	0.91992	0.92350	0.92469	0.92847	0.93246	0.93674
449	0.91211	0.92006	0.92364	0.92482	0.92860	0.93258	0.93685
450	0.91229	0.92021	0.92378	0.92496	0.92873	0.93271	0.93697
451	0.91245	0.92035	0.92392	0.92510	0.92886	0.93283	0.93708
452	0.91258	0.92050	0.92406	0.92524	0.92899	0.93294	0.93719
453	0.91276	0.92064	0.92420	0.92538	0.92912	0.93306	0.93730
454	0.91293	0.92079	0.92434	0.92551	0.92925	0.93318	0.93741
455	0.91308	0.92094	0.92448	0.92565	0.92937	0.93330	0.93752
456	0.91325	0.92108	0.92461	0.92579	0.92950	0.93342	0.93763
457	0.91340	0.92122	0.92475	0.92592	0.92962	0.93354	0.93774
458	0.91354	0.92137	0.92489	0.92605	0.92975	0.93365	0.93785
459	0.91372	0.92150	0.92502	0.92619	0.92988	0.93377	0.93796
460	0.91388	0.92164	0.92516	0.92631	0.93000	0.93389	0.93807
461	0.91405	0.92179	0.92529	0.92644	0.93013	0.93401	0.93818
462	0.91419	0.92193	0.92542	0.92658	0.93025	0.93412	0.93828
463	0.91435	0.92207	0.92555	0.92670	0.93037	0.93424	0.93839
464	0.91450	0.92221	0.92569	0.92683	0.93049	0.93435	0.93850
465	0.91466	0.92234	0.92582	0.92696	0.93062	0.93447	0.93860
466	0.91479	0.92248	0.92595	0.92709	0.93074	0.93458	0.93871
467	0.91494	0.92262	0.92607	0.92722	0.93086	0.93469	0.93881
468	0.91509	0.92275	0.92621	0.92735	0.93098	0.93480	0.93892
469	0.91525	0.92289	0.92634	0.92747	0.93110	0.93492	0.93902
470	0.91540	0.92303	0.92647	0.92760	0.93122	0.93503	0.93913
471	0.91556	0.92316	0.92660	0.92773	0.93134	0.93514	0.93923
472	0.91569	0.92330	0.92673	0.92786	0.93146	0.93525	0.93933
473	0.91585	0.92344	0.92686	0.92798	0.93158	0.93536	0.93944
474	0.91600	0.92357	0.92698	0.92811	0.93170	0.93548	0.93954
475	0.91615	0.92371	0.92711	0.92823	0.93182	0.93559	0.93964
476	0.91631	0.92384	0.92724	0.92836	0.93193	0.93570	0.93975
477	0.91644	0.92398	0.92736	0.92848	0.93206	0.93581	0.93985
478	0.91660	0.92411	0.92749	0.92860	0.93217	0.93592	0.93995
479	0.91676	0.92424	0.92762	0.92873	0.93229	0.93603	0.94005
480	0.91691	0.92437	0.92773	0.92885	0.93240	0.93614	0.94015
481	0.91706	0.92450	0.92786	0.92897	0.93252	0.93624	0.94025
482	0.91720	0.92464	0.92798	0.92910	0.93263	0.93635	0.94035
483	0.91733	0.92477	0.92811	0.92922	0.93274	0.93646	0.94045
484	0.91748	0.92489	0.92823	0.92934	0.93286	0.93657	0.94055
485	0.91763	0.92502	0.92836	0.92946	0.93297	0.93667	0.94065
486	0.91778	0.92516	0.92848	0.92958	0.93308	0.93678	0.94074
487	0.91793	0.92528	0.92860	0.92970	0.93320	0.93688	0.94084
488	0.91806	0.92541	0.92872	0.92982	0.93331	0.93699	0.94094
489	0.91820	0.92553	0.92885	0.92993	0.93342	0.93709	0.94104
490	0.91834	0.92566	0.92896	0.93006	0.93353	0.93720	0.94113
491	0.91847	0.92579	0.92908	0.93017	0.93364	0.93730	0.94123
492	0.91861	0.92591	0.92920	0.93029	0.93375	0.93741	0.94132
493	0.91876	0.92604	0.92931	0.93040	0.93386	0.93751	0.94142
494	0.91889	0.92616	0.92943	0.93052	0.93397	0.93761	0.94152
495	0.91901	0.92628	0.92955	0.93063	0.93408	0.93772	0.94161
496	0.91915	0.92640	0.92967	0.93075	0.93419	0.93782	0.94171
497	0.91929	0.92653	0.92979	0.93086	0.93430	0.93792	0.94180
498	0.91943	0.92665	0.92991	0.93098	0.93441	0.93802	0.94190
499	0.91955	0.92677	0.93002	0.93109	0.93452	0.93812	0.94199
500	0.91968	0.92690	0.93014	0.93120	0.93463	0.93822	0.94208
501	0.91980	0.92702	0.93025	0.93132	0.93473	0.93832	0.94218
502	0.91994	0.92714	0.93037	0.93143	0.93484	0.93842	0.94227
503	0.92007	0.92726	0.93049	0.93154	0.93494	0.93852	0.94236
504	0.92021	0.92738	0.93060	0.93166	0.93505	0.93862	0.94245
505	0.92035	0.92750	0.93071	0.93177	0.93515	0.93872	0.94255
506	0.92049	0.92761	0.93083	0.93188	0.93526	0.93882	0.94264
507	0.92061	0.92773	0.93094	0.93199	0.93536	0.93892	0.94273
508	0.92075	0.92785	0.93105	0.93210	0.93547	0.93902	0.94282
509	0.92090	0.92798	0.93117	0.93222	0.93557	0.93911	0.94291
510	0.92102	0.92810	0.93128	0.93232	0.93568	0.93921	0.94300
511	0.92115	0.92822	0.93140	0.93243	0.93578	0.93931	0.94309
512	0.92128	0.92834	0.93150	0.93254	0.93588	0.93940	0.94318
513	0.92140	0.92845	0.93161	0.93264	0.93599	0.93950	0.94327
514	0.92153	0.92857	0.93173	0.93275	0.93609	0.93959	0.94336

N	0.1%	0.5%	1%	1.25%	2.5%	5%	10%
515	0.92165	0.92868	0.93184	0.93287	0.93619	0.93969	0.94345
516	0.92178	0.92879	0.93195	0.93297	0.93629	0.93978	0.94354
517	0.92191	0.92890	0.93206	0.93308	0.93639	0.93988	0.94362
518	0.92205	0.92902	0.93216	0.93319	0.93649	0.93997	0.94371
519	0.92217	0.92913	0.93228	0.93329	0.93660	0.94007	0.94380
520	0.92229	0.92924	0.93238	0.93340	0.93669	0.94016	0.94389
521	0.92243	0.92935	0.93249	0.93350	0.93679	0.94026	0.94397
522	0.92256	0.92947	0.93260	0.93361	0.93689	0.94035	0.94406
523	0.92269	0.92958	0.93271	0.93372	0.93700	0.94044	0.94415
524	0.92280	0.92969	0.93281	0.93382	0.93709	0.94054	0.94423
525	0.92291	0.92980	0.93292	0.93392	0.93719	0.94063	0.94432
526	0.92304	0.92991	0.93302	0.93402	0.93729	0.94072	0.94441
527	0.92316	0.93002	0.93313	0.93413	0.93738	0.94082	0.94449
528	0.92328	0.93013	0.93323	0.93424	0.93748	0.94090	0.94458
529	0.92340	0.93024	0.93334	0.93434	0.93758	0.94100	0.94466
530	0.92352	0.93035	0.93344	0.93444	0.93768	0.94109	0.94475
531	0.92366	0.93045	0.93355	0.93454	0.93778	0.94118	0.94483
532	0.92378	0.93056	0.93365	0.93465	0.93787	0.94127	0.94491
533	0.92391	0.93067	0.93376	0.93475	0.93797	0.94136	0.94500
534	0.92403	0.93078	0.93386	0.93485	0.93807	0.94145	0.94508
535	0.92414	0.93089	0.93396	0.93495	0.93816	0.94154	0.94516
536	0.92425	0.93100	0.93407	0.93505	0.93826	0.94163	0.94524
537	0.92438	0.93111	0.93418	0.93515	0.93835	0.94172	0.94533
538	0.92450	0.93122	0.93428	0.93525	0.93844	0.94181	0.94541
539	0.92464	0.93132	0.93438	0.93535	0.93853	0.94190	0.94549
540	0.92475	0.93143	0.93448	0.93546	0.93863	0.94198	0.94557
541	0.92486	0.93153	0.93458	0.93556	0.93872	0.94207	0.94565
542	0.92498	0.93164	0.93468	0.93565	0.93882	0.94216	0.94574
543	0.92510	0.93175	0.93478	0.93575	0.93891	0.94224	0.94582
544	0.92523	0.93186	0.93488	0.93585	0.93900	0.94233	0.94590
545	0.92533	0.93196	0.93498	0.93595	0.93910	0.94242	0.94598
546	0.92545	0.93207	0.93508	0.93605	0.93919	0.94251	0.94606
547	0.92557	0.93218	0.93518	0.93614	0.93928	0.94259	0.94614
548	0.92569	0.93228	0.93528	0.93624	0.93937	0.94268	0.94622
549	0.92579	0.93238	0.93538	0.93634	0.93946	0.94276	0.94630
550	0.92591	0.93249	0.93547	0.93643	0.93955	0.94285	0.94637
551	0.92603	0.93259	0.93557	0.93653	0.93964	0.94293	0.94645
552	0.92614	0.93269	0.93567	0.93663	0.93973	0.94302	0.94653
553	0.92625	0.93279	0.93576	0.93672	0.93982	0.94310	0.94661
554	0.92637	0.93289	0.93585	0.93682	0.93991	0.94319	0.94669
555	0.92648	0.93300	0.93595	0.93691	0.94000	0.94327	0.94677
556	0.92659	0.93309	0.93605	0.93700	0.94009	0.94335	0.94685
557	0.92670	0.93320	0.93614	0.93710	0.94018	0.94343	0.94692
558	0.92682	0.93330	0.93624	0.93719	0.94027	0.94352	0.94700
559	0.92692	0.93339	0.93633	0.93728	0.94035	0.94360	0.94708
560	0.92704	0.93350	0.93643	0.93738	0.94044	0.94368	0.94716
561	0.92715	0.93359	0.93652	0.93747	0.94053	0.94376	0.94723
562	0.92727	0.93369	0.93661	0.93756	0.94062	0.94384	0.94731
563	0.92738	0.93380	0.93670	0.93765	0.94070	0.94393	0.94738
564	0.92748	0.93389	0.93679	0.93775	0.94079	0.94401	0.94746
565	0.92760	0.93399	0.93689	0.93784	0.94088	0.94409	0.94753
566	0.92770	0.93409	0.93698	0.93793	0.94096	0.94417	0.94761
567	0.92781	0.93418	0.93707	0.93802	0.94105	0.94425	0.94768
568	0.92792	0.93428	0.93716	0.93811	0.94114	0.94433	0.94776
569	0.92803	0.93438	0.93726	0.93820	0.94122	0.94441	0.94783
570	0.92813	0.93448	0.93735	0.93829	0.94131	0.94449	0.94791
571	0.92825	0.93457	0.93744	0.93838	0.94139	0.94457	0.94798
572	0.92836	0.93467	0.93753	0.93847	0.94148	0.94465	0.94806
573	0.92846	0.93477	0.93762	0.93856	0.94156	0.94473	0.94813
574	0.92856	0.93486	0.93771	0.93865	0.94165	0.94481	0.94820
575	0.92867	0.93495	0.93780	0.93874	0.94173	0.94489	0.94827
576	0.92876	0.93505	0.93789	0.93882	0.94182	0.94497	0.94835
577	0.92887	0.93515	0.93798	0.93891	0.94190	0.94505	0.94842
578	0.92898	0.93524	0.93807	0.93900	0.94198	0.94513	0.94849
579	0.92908	0.93534	0.93816	0.93909	0.94207	0.94520	0.94857
580	0.92917	0.93543	0.93825	0.93918	0.94215	0.94528	0.94864
581	0.92927	0.93553	0.93834	0.93927	0.94223	0.94536	0.94871
582	0.92938	0.93561	0.93842	0.93936	0.94231	0.94544	0.94878
583	0.92947	0.93570	0.93851	0.93944	0.94240	0.94551	0.94885
584	0.92958	0.93579	0.93860	0.93953	0.94248	0.94559	0.94892
585	0.92968	0.93588	0.93869	0.93961	0.94256	0.94566	0.94899
586	0.92978	0.93597	0.93877	0.93970	0.94264	0.94574	0.94907
587	0.92988	0.93607	0.93886	0.93978	0.94272	0.94582	0.94914
588	0.92998	0.93615	0.93895	0.93987	0.94280	0.94589	0.94921

N	0.1%	0.5%	1%	1.25%	2.5%	5%	10%
589	0.93008	0.93625	0.93903	0.93995	0.94288	0.94597	0.94928
590	0.93018	0.93634	0.93912	0.94004	0.94296	0.94604	0.94935
591	0.93030	0.93643	0.93921	0.94012	0.94304	0.94612	0.94942
592	0.93040	0.93652	0.93929	0.94021	0.94312	0.94619	0.94949
593	0.93050	0.93661	0.93938	0.94029	0.94320	0.94627	0.94956
594	0.93061	0.93670	0.93946	0.94038	0.94328	0.94634	0.94962
595	0.93070	0.93679	0.93955	0.94046	0.94336	0.94641	0.94969
596	0.93079	0.93688	0.93963	0.94055	0.94344	0.94649	0.94976
597	0.93090	0.93696	0.93972	0.94062	0.94351	0.94656	0.94983
598	0.93099	0.93705	0.93980	0.94070	0.94359	0.94664	0.94990
599	0.93109	0.93714	0.93988	0.94079	0.94367	0.94671	0.94997
600	0.93118	0.93723	0.93997	0.94087	0.94375	0.94678	0.95003
601	0.93129	0.93732	0.94005	0.94095	0.94382	0.94685	0.95010
602	0.93137	0.93740	0.94013	0.94103	0.94390	0.94693	0.95017
603	0.93146	0.93749	0.94022	0.94111	0.94398	0.94700	0.95024
604	0.93156	0.93758	0.94030	0.94119	0.94405	0.94707	0.95030
605	0.93165	0.93767	0.94038	0.94127	0.94413	0.94714	0.95037
606	0.93176	0.93775	0.94047	0.94136	0.94421	0.94721	0.95044
607	0.93185	0.93784	0.94055	0.94144	0.94428	0.94728	0.95050
608	0.93195	0.93793	0.94063	0.94152	0.94436	0.94735	0.95057
609	0.93204	0.93801	0.94071	0.94160	0.94443	0.94743	0.95063
610	0.93213	0.93810	0.94079	0.94167	0.94451	0.94750	0.95070
611	0.93222	0.93818	0.94087	0.94175	0.94458	0.94757	0.95076
612	0.93233	0.93827	0.94095	0.94183	0.94466	0.94764	0.95083
613	0.93242	0.93835	0.94103	0.94191	0.94473	0.94770	0.95089
614	0.93251	0.93844	0.94111	0.94199	0.94481	0.94778	0.95096
615	0.93261	0.93852	0.94119	0.94207	0.94488	0.94785	0.95102
616	0.93270	0.93860	0.94127	0.94215	0.94495	0.94792	0.95109
617	0.93280	0.93869	0.94135	0.94222	0.94503	0.94799	0.95115
618	0.93290	0.93876	0.94143	0.94230	0.94510	0.94805	0.95122
619	0.93299	0.93885	0.94151	0.94238	0.94517	0.94812	0.95128
620	0.93307	0.93893	0.94159	0.94246	0.94525	0.94819	0.95134
621	0.93315	0.93901	0.94167	0.94254	0.94532	0.94826	0.95141
622	0.93325	0.93910	0.94175	0.94261	0.94540	0.94833	0.95147
623	0.93335	0.93918	0.94183	0.94269	0.94547	0.94840	0.95154
624	0.93344	0.93926	0.94190	0.94277	0.94554	0.94847	0.95160
625	0.93351	0.93934	0.94198	0.94285	0.94561	0.94854	0.95166
626	0.93360	0.93943	0.94206	0.94292	0.94568	0.94860	0.95173
627	0.93369	0.93951	0.94214	0.94300	0.94576	0.94867	0.95179
628	0.93377	0.93959	0.94221	0.94307	0.94583	0.94874	0.95185
629	0.93387	0.93967	0.94229	0.94315	0.94590	0.94881	0.95192
630	0.93395	0.93975	0.94237	0.94323	0.94597	0.94887	0.95198
631	0.93404	0.93983	0.94244	0.94330	0.94604	0.94894	0.95204
632	0.93413	0.93991	0.94252	0.94338	0.94611	0.94901	0.95210
633	0.93422	0.93999	0.94259	0.94345	0.94618	0.94907	0.95216
634	0.93432	0.94007	0.94267	0.94353	0.94625	0.94914	0.95222
635	0.93440	0.94015	0.94275	0.94360	0.94632	0.94920	0.95228
636	0.93449	0.94023	0.94283	0.94367	0.94639	0.94927	0.95235
637	0.93458	0.94031	0.94290	0.94375	0.94646	0.94933	0.95241
638	0.93467	0.94039	0.94298	0.94382	0.94653	0.94940	0.95247
639	0.93476	0.94046	0.94305	0.94390	0.94660	0.94946	0.95253
640	0.93484	0.94054	0.94312	0.94397	0.94667	0.94953	0.95259
641	0.93492	0.94062	0.94320	0.94404	0.94674	0.94959	0.95265
642	0.93502	0.94069	0.94327	0.94412	0.94681	0.94966	0.95271
643	0.93510	0.94077	0.94334	0.94419	0.94687	0.94972	0.95277
644	0.93519	0.94085	0.94342	0.94426	0.94694	0.94978	0.95283
645	0.93527	0.94093	0.94349	0.94433	0.94701	0.94985	0.95289
646	0.93536	0.94100	0.94356	0.94440	0.94708	0.94991	0.95295
647	0.93545	0.94108	0.94364	0.94448	0.94715	0.94997	0.95301
648	0.93553	0.94115	0.94371	0.94455	0.94722	0.95004	0.95307
649	0.93562	0.94123	0.94378	0.94462	0.94728	0.95010	0.95312
650	0.93569	0.94131	0.94385	0.94469	0.94735	0.95016	0.95318
651	0.93578	0.94139	0.94392	0.94476	0.94741	0.95023	0.95324
652	0.93586	0.94147	0.94399	0.94484	0.94748	0.95029	0.95330
653	0.93594	0.94153	0.94407	0.94490	0.94755	0.95035	0.95336
654	0.93602	0.94161	0.94414	0.94497	0.94762	0.95042	0.95342
655	0.93611	0.94169	0.94421	0.94505	0.94768	0.95048	0.95347
656	0.93619	0.94176	0.94428	0.94511	0.94775	0.95054	0.95353
657	0.93626	0.94184	0.94436	0.94519	0.94781	0.95060	0.95359
658	0.93636	0.94191	0.94442	0.94526	0.94788	0.95066	0.95365
659	0.93643	0.94199	0.94449	0.94532	0.94794	0.95072	0.95371
660	0.93652	0.94206	0.94457	0.94539	0.94801	0.95079	0.95376
661	0.93659	0.94213	0.94464	0.94546	0.94807	0.95085	0.95382
662	0.93667	0.94220	0.94471	0.94553	0.94814	0.95091	0.95388

N	0.1%	0.5%	1%	1.25%	2.5%	5%	10%
663	0.93675	0.94228	0.94478	0.94560	0.94821	0.95097	0.95393
664	0.93684	0.94235	0.94485	0.94566	0.94827	0.95103	0.95399
665	0.93692	0.94243	0.94491	0.94573	0.94833	0.95109	0.95405
666	0.93701	0.94250	0.94498	0.94580	0.94840	0.95115	0.95410
667	0.93709	0.94257	0.94505	0.94587	0.94846	0.95121	0.95416
668	0.93717	0.94264	0.94512	0.94593	0.94852	0.95127	0.95422
669	0.93724	0.94271	0.94519	0.94600	0.94859	0.95133	0.95427
670	0.93733	0.94278	0.94526	0.94607	0.94865	0.95139	0.95433
671	0.93742	0.94286	0.94533	0.94614	0.94872	0.95145	0.95438
672	0.93749	0.94293	0.94540	0.94620	0.94878	0.95151	0.95444
673	0.93757	0.94300	0.94546	0.94627	0.94884	0.95157	0.95449
674	0.93765	0.94307	0.94553	0.94634	0.94891	0.95163	0.95455
675	0.93773	0.94313	0.94560	0.94640	0.94897	0.95169	0.95460
676	0.93780	0.94321	0.94567	0.94647	0.94904	0.95175	0.95466
677	0.93787	0.94328	0.94573	0.94654	0.94910	0.95181	0.95471
678	0.93796	0.94335	0.94580	0.94661	0.94916	0.95187	0.95477
679	0.93804	0.94342	0.94587	0.94667	0.94922	0.95193	0.95482
680	0.93812	0.94349	0.94594	0.94674	0.94928	0.95198	0.95488
681	0.93819	0.94356	0.94600	0.94680	0.94935	0.95204	0.95493
682	0.93827	0.94363	0.94607	0.94687	0.94941	0.95210	0.95498
683	0.93835	0.94370	0.94614	0.94693	0.94947	0.95216	0.95504
684	0.93843	0.94377	0.94620	0.94700	0.94953	0.95221	0.95509
685	0.93851	0.94384	0.94627	0.94706	0.94959	0.95227	0.95514
686	0.93859	0.94391	0.94634	0.94713	0.94965	0.95233	0.95520
687	0.93867	0.94397	0.94640	0.94719	0.94971	0.95239	0.95525
688	0.93874	0.94404	0.94647	0.94726	0.94977	0.95244	0.95530
689	0.93882	0.94411	0.94653	0.94732	0.94983	0.95250	0.95536
690	0.93890	0.94418	0.94660	0.94738	0.94990	0.95256	0.95541
691	0.93898	0.94424	0.94666	0.94745	0.94996	0.95261	0.95546
692	0.93905	0.94431	0.94672	0.94751	0.95002	0.95267	0.95552
693	0.93912	0.94438	0.94679	0.94758	0.95008	0.95273	0.95557
694	0.93920	0.94444	0.94685	0.94764	0.95014	0.95278	0.95562
695	0.93926	0.94451	0.94692	0.94770	0.95020	0.95284	0.95567
696	0.93934	0.94458	0.94698	0.94777	0.95026	0.95290	0.95573
697	0.93941	0.94465	0.94704	0.94783	0.95032	0.95295	0.95578
698	0.93949	0.94472	0.94710	0.94789	0.95038	0.95301	0.95583
699	0.93957	0.94478	0.94717	0.94795	0.95044	0.95306	0.95588
700	0.93964	0.94485	0.94723	0.94802	0.95049	0.95312	0.95593
701	0.93971	0.94492	0.94729	0.94808	0.95055	0.95317	0.95598
702	0.93979	0.94498	0.94735	0.94814	0.95061	0.95323	0.95603
703	0.93987	0.94505	0.94742	0.94820	0.95067	0.95328	0.95609
704	0.93994	0.94512	0.94748	0.94826	0.95073	0.95334	0.95614
705	0.94001	0.94518	0.94754	0.94833	0.95079	0.95339	0.95619
706	0.94009	0.94524	0.94761	0.94839	0.95085	0.95345	0.95624
707	0.94015	0.94531	0.94767	0.94845	0.95090	0.95350	0.95629
708	0.94023	0.94538	0.94773	0.94851	0.95096	0.95356	0.95634
709	0.94030	0.94544	0.94779	0.94857	0.95102	0.95361	0.95639
710	0.94038	0.94551	0.94785	0.94863	0.95108	0.95367	0.95644
711	0.94045	0.94557	0.94791	0.94869	0.95113	0.95372	0.95649
712	0.94052	0.94563	0.94798	0.94875	0.95119	0.95377	0.95654
713	0.94060	0.94570	0.94804	0.94881	0.95125	0.95383	0.95659
714	0.94066	0.94576	0.94810	0.94887	0.95130	0.95388	0.95664
715	0.94074	0.94583	0.94816	0.94893	0.95136	0.95393	0.95669
716	0.94080	0.94589	0.94822	0.94899	0.95142	0.95398	0.95674
717	0.94087	0.94595	0.94828	0.94905	0.95147	0.95404	0.95679
718	0.94095	0.94602	0.94834	0.94911	0.95153	0.95409	0.95684
719	0.94101	0.94608	0.94840	0.94917	0.95159	0.95414	0.95689
720	0.94107	0.94614	0.94846	0.94922	0.95164	0.95420	0.95694
721	0.94114	0.94620	0.94852	0.94928	0.95170	0.95425	0.95698
722	0.94121	0.94627	0.94858	0.94934	0.95176	0.95430	0.95703
723	0.94128	0.94633	0.94864	0.94940	0.95181	0.95435	0.95708
724	0.94135	0.94639	0.94870	0.94946	0.95186	0.95441	0.95713
725	0.94142	0.94646	0.94876	0.94952	0.95192	0.95446	0.95718
726	0.94148	0.94652	0.94882	0.94958	0.95197	0.95451	0.95723
727	0.94155	0.94659	0.94888	0.94964	0.95203	0.95456	0.95728
728	0.94162	0.94665	0.94894	0.94969	0.95208	0.95461	0.95732
729	0.94169	0.94671	0.94899	0.94975	0.95214	0.95467	0.95737
730	0.94176	0.94677	0.94905	0.94981	0.95219	0.95472	0.95742
731	0.94182	0.94683	0.94911	0.94986	0.95225	0.95477	0.95747
732	0.94189	0.94689	0.94917	0.94992	0.95230	0.95482	0.95751
733	0.94197	0.94695	0.94923	0.94998	0.95236	0.95487	0.95756
734	0.94203	0.94701	0.94929	0.95004	0.95241	0.95492	0.95761
735	0.94208	0.94707	0.94934	0.95010	0.95247	0.95497	0.95766
736	0.94215	0.94714	0.94940	0.95015	0.95252	0.95502	0.95770

N	0.1%	0.5%	1%	1.25%	2.5%	5%	10%
737	0.94222	0.94720	0.94946	0.95021	0.95257	0.95507	0.95775
738	0.94230	0.94726	0.94952	0.95026	0.95263	0.95512	0.95780
739	0.94236	0.94732	0.94958	0.95032	0.95268	0.95517	0.95784
740	0.94242	0.94738	0.94963	0.95037	0.95273	0.95522	0.95789
741	0.94249	0.94743	0.94969	0.95043	0.95279	0.95527	0.95794
742	0.94255	0.94749	0.94975	0.95049	0.95284	0.95532	0.95798
743	0.94262	0.94755	0.94981	0.95054	0.95289	0.95537	0.95803
744	0.94268	0.94761	0.94986	0.95060	0.95295	0.95542	0.95808
745	0.94275	0.94767	0.94992	0.95065	0.95300	0.95547	0.95812
746	0.94282	0.94773	0.94998	0.95071	0.95305	0.95552	0.95817
747	0.94288	0.94779	0.95003	0.95076	0.95311	0.95557	0.95821
748	0.94295	0.94785	0.95009	0.95082	0.95316	0.95562	0.95826
749	0.94302	0.94791	0.95015	0.95088	0.95321	0.95567	0.95831
750	0.94309	0.94797	0.95021	0.95093	0.95326	0.95572	0.95835
751	0.94316	0.94803	0.95026	0.95099	0.95331	0.95576	0.95840
752	0.94323	0.94808	0.95031	0.95104	0.95337	0.95581	0.95844
753	0.94329	0.94815	0.95037	0.95110	0.95342	0.95586	0.95849
754	0.94335	0.94820	0.95042	0.95115	0.95347	0.95591	0.95853
755	0.94341	0.94826	0.95048	0.95121	0.95352	0.95596	0.95858
756	0.94347	0.94832	0.95054	0.95126	0.95357	0.95601	0.95862
757	0.94355	0.94838	0.95059	0.95132	0.95362	0.95605	0.95867
758	0.94361	0.94844	0.95065	0.95137	0.95368	0.95610	0.95871
759	0.94366	0.94850	0.95070	0.95143	0.95372	0.95615	0.95876
760	0.94373	0.94855	0.95076	0.95148	0.95378	0.95620	0.95880
761	0.94380	0.94861	0.95081	0.95153	0.95383	0.95625	0.95885
762	0.94385	0.94867	0.95086	0.95158	0.95388	0.95629	0.95889
763	0.94392	0.94873	0.95092	0.95164	0.95393	0.95634	0.95893
764	0.94398	0.94878	0.95097	0.95169	0.95398	0.95639	0.95898
765	0.94404	0.94884	0.95103	0.95174	0.95403	0.95644	0.95902
766	0.94411	0.94890	0.95108	0.95179	0.95408	0.95648	0.95907
767	0.94417	0.94896	0.95113	0.95185	0.95413	0.95653	0.95911
768	0.94423	0.94901	0.95119	0.95190	0.95418	0.95658	0.95915
769	0.94430	0.94907	0.95124	0.95195	0.95423	0.95662	0.95920
770	0.94436	0.94913	0.95130	0.95201	0.95428	0.95667	0.95924
771	0.94443	0.94918	0.95135	0.95206	0.95433	0.95672	0.95928
772	0.94449	0.94924	0.95141	0.95211	0.95438	0.95676	0.95933
773	0.94455	0.94929	0.95146	0.95216	0.95443	0.95681	0.95937
774	0.94461	0.94935	0.95151	0.95222	0.95448	0.95686	0.95941
775	0.94467	0.94940	0.95156	0.95227	0.95453	0.95690	0.95946
776	0.94474	0.94945	0.95161	0.95232	0.95458	0.95695	0.95950
777	0.94479	0.94951	0.95167	0.95237	0.95463	0.95699	0.95954
778	0.94485	0.94956	0.95172	0.95242	0.95467	0.95704	0.95958
779	0.94491	0.94962	0.95177	0.95247	0.95472	0.95709	0.95963
780	0.94498	0.94968	0.95182	0.95253	0.95477	0.95713	0.95967
781	0.94503	0.94973	0.95187	0.95258	0.95482	0.95718	0.95971
782	0.94510	0.94979	0.95192	0.95263	0.95487	0.95722	0.95976
783	0.94516	0.94984	0.95197	0.95268	0.95492	0.95727	0.95980
784	0.94522	0.94989	0.95202	0.95273	0.95497	0.95731	0.95984
785	0.94528	0.94994	0.95208	0.95278	0.95501	0.95736	0.95988
786	0.94533	0.95000	0.95213	0.95283	0.95506	0.95740	0.95992
787	0.94540	0.95005	0.95218	0.95288	0.95511	0.95745	0.95997
788	0.94545	0.95011	0.95223	0.95293	0.95516	0.95749	0.96001
789	0.94551	0.95017	0.95228	0.95298	0.95520	0.95754	0.96005
790	0.94557	0.95022	0.95234	0.95303	0.95525	0.95758	0.96009
791	0.94563	0.95028	0.95239	0.95309	0.95530	0.95763	0.96013
792	0.94570	0.95033	0.95244	0.95314	0.95535	0.95767	0.96017
793	0.94576	0.95038	0.95249	0.95319	0.95539	0.95772	0.96021
794	0.94581	0.95043	0.95254	0.95323	0.95544	0.95776	0.96026
795	0.94587	0.95049	0.95259	0.95328	0.95549	0.95780	0.96030
796	0.94593	0.95054	0.95264	0.95333	0.95553	0.95785	0.96034
797	0.94599	0.95059	0.95269	0.95338	0.95558	0.95789	0.96038
798	0.94604	0.95065	0.95274	0.95343	0.95563	0.95794	0.96042
799	0.94610	0.95070	0.95279	0.95348	0.95567	0.95798	0.96046
800	0.94616	0.95075	0.95284	0.95353	0.95572	0.95802	0.96050
801	0.94622	0.95080	0.95289	0.95358	0.95577	0.95807	0.96054
802	0.94628	0.95086	0.95294	0.95363	0.95581	0.95811	0.96058
803	0.94634	0.95091	0.95299	0.95368	0.95586	0.95815	0.96062
804	0.94639	0.95096	0.95303	0.95372	0.95591	0.95820	0.96066
805	0.94646	0.95101	0.95309	0.95377	0.95595	0.95824	0.96070
806	0.94651	0.95106	0.95314	0.95382	0.95600	0.95828	0.96074
807	0.94657	0.95111	0.95318	0.95387	0.95604	0.95833	0.96078
808	0.94662	0.95116	0.95323	0.95392	0.95609	0.95837	0.96082
809	0.94668	0.95121	0.95329	0.95396	0.95613	0.95841	0.96086
810	0.94673	0.95127	0.95333	0.95401	0.95618	0.95845	0.96090

N	0.1%	0.5%	1%	1.25%	2.5%	5%	10%
811	0.94679	0.95131	0.95338	0.95406	0.95622	0.95850	0.96094
812	0.94684	0.95137	0.95343	0.95411	0.95627	0.95854	0.96098
813	0.94691	0.95142	0.95348	0.95416	0.95631	0.95858	0.96102
814	0.94696	0.95147	0.95353	0.95420	0.95636	0.95862	0.96106
815	0.94701	0.95152	0.95357	0.95425	0.95640	0.95867	0.96110
816	0.94707	0.95157	0.95362	0.95430	0.95645	0.95871	0.96114
817	0.94712	0.95162	0.95367	0.95434	0.95649	0.95875	0.96118
818	0.94718	0.95167	0.95372	0.95439	0.95654	0.95879	0.96122
819	0.94723	0.95173	0.95376	0.95444	0.95658	0.95883	0.96126
820	0.94730	0.95178	0.95381	0.95448	0.95663	0.95887	0.96129
821	0.94735	0.95182	0.95386	0.95453	0.95667	0.95892	0.96133
822	0.94741	0.95187	0.95391	0.95458	0.95671	0.95896	0.96137
823	0.94746	0.95192	0.95395	0.95462	0.95676	0.95900	0.96141
824	0.94751	0.95197	0.95400	0.95467	0.95680	0.95904	0.96145
825	0.94757	0.95202	0.95405	0.95472	0.95685	0.95908	0.96149
826	0.94762	0.95207	0.95410	0.95476	0.95689	0.95912	0.96153
827	0.94767	0.95212	0.95414	0.95481	0.95693	0.95916	0.96156
828	0.94773	0.95217	0.95419	0.95485	0.95698	0.95920	0.96160
829	0.94779	0.95222	0.95423	0.95490	0.95702	0.95924	0.96164
830	0.94784	0.95227	0.95428	0.95494	0.95706	0.95928	0.96168
831	0.94789	0.95231	0.95433	0.95499	0.95711	0.95932	0.96172
832	0.94795	0.95236	0.95437	0.95504	0.95715	0.95937	0.96175
833	0.94800	0.95241	0.95442	0.95508	0.95719	0.95941	0.96179
834	0.94806	0.95246	0.95447	0.95513	0.95724	0.95945	0.96183
835	0.94810	0.95251	0.95451	0.95517	0.95728	0.95949	0.96187
836	0.94816	0.95256	0.95456	0.95522	0.95732	0.95953	0.96191
837	0.94822	0.95261	0.95460	0.95526	0.95736	0.95957	0.96194
838	0.94827	0.95266	0.95465	0.95531	0.95741	0.95961	0.96198
839	0.94833	0.95270	0.95469	0.95535	0.95745	0.95965	0.96202
840	0.94838	0.95275	0.95474	0.95540	0.95749	0.95969	0.96206
841	0.94843	0.95280	0.95479	0.95544	0.95753	0.95973	0.96209
842	0.94849	0.95284	0.95483	0.95549	0.95758	0.95977	0.96213
843	0.94854	0.95289	0.95488	0.95553	0.95762	0.95980	0.96217
844	0.94860	0.95294	0.95492	0.95558	0.95766	0.95984	0.96220
845	0.94865	0.95299	0.95497	0.95562	0.95770	0.95988	0.96224
846	0.94870	0.95303	0.95501	0.95567	0.95774	0.95992	0.96228
847	0.94875	0.95308	0.95506	0.95571	0.95778	0.95996	0.96231
848	0.94880	0.95313	0.95510	0.95575	0.95783	0.96000	0.96235
849	0.94886	0.95318	0.95514	0.95579	0.95787	0.96004	0.96239
850	0.94891	0.95322	0.95519	0.95584	0.95791	0.96008	0.96242
851	0.94895	0.95327	0.95523	0.95588	0.95795	0.96012	0.96246
852	0.94900	0.95331	0.95528	0.95592	0.95799	0.96016	0.96249
853	0.94906	0.95336	0.95532	0.95597	0.95803	0.96020	0.96253
854	0.94910	0.95340	0.95537	0.95601	0.95808	0.96024	0.96257
855	0.94916	0.95345	0.95541	0.95605	0.95812	0.96028	0.96260
856	0.94921	0.95350	0.95545	0.95609	0.95816	0.96031	0.96264
857	0.94926	0.95354	0.95550	0.95614	0.95820	0.96035	0.96267
858	0.94931	0.95359	0.95554	0.95618	0.95824	0.96039	0.96271
859	0.94937	0.95364	0.95559	0.95622	0.95828	0.96043	0.96275
860	0.94942	0.95369	0.95563	0.95627	0.95832	0.96047	0.96278
861	0.94947	0.95373	0.95567	0.95631	0.95836	0.96051	0.96282
862	0.94952	0.95378	0.95571	0.95635	0.95840	0.96054	0.96285
863	0.94957	0.95383	0.95576	0.95640	0.95844	0.96058	0.96289
864	0.94962	0.95387	0.95580	0.95644	0.95848	0.96062	0.96292
865	0.94968	0.95392	0.95585	0.95648	0.95852	0.96066	0.96296
866	0.94973	0.95396	0.95589	0.95653	0.95856	0.96070	0.96300
867	0.94978	0.95400	0.95593	0.95657	0.95860	0.96073	0.96303
868	0.94982	0.95404	0.95597	0.95661	0.95864	0.96077	0.96307
869	0.94987	0.95409	0.95602	0.95665	0.95868	0.96081	0.96310
870	0.94992	0.95414	0.95606	0.95669	0.95872	0.96085	0.96313
871	0.94997	0.95418	0.95610	0.95674	0.95876	0.96088	0.96317
872	0.95002	0.95423	0.95614	0.95678	0.95880	0.96092	0.96320
873	0.95007	0.95427	0.95619	0.95682	0.95884	0.96096	0.96324
874	0.95012	0.95431	0.95623	0.95686	0.95888	0.96100	0.96327
875	0.95016	0.95436	0.95627	0.95690	0.95892	0.96103	0.96331
876	0.95022	0.95440	0.95631	0.95694	0.95896	0.96107	0.96334
877	0.95026	0.95445	0.95635	0.95699	0.95900	0.96111	0.96338
878	0.95031	0.95449	0.95639	0.95703	0.95904	0.96114	0.96341
879	0.95037	0.95453	0.95643	0.95707	0.95907	0.96118	0.96345
880	0.95041	0.95458	0.95648	0.95711	0.95912	0.96122	0.96348
881	0.95046	0.95462	0.95652	0.95715	0.95915	0.96125	0.96351
882	0.95050	0.95467	0.95656	0.95719	0.95919	0.96129	0.96355
883	0.95055	0.95471	0.95660	0.95723	0.95923	0.96133	0.96358
884	0.95060	0.95475	0.95665	0.95727	0.95927	0.96136	0.96362

N	0.1%	0.5%	1%	1.25%	2.5%	5%	10%
885	0.95064	0.95479	0.95669	0.95731	0.95931	0.96140	0.96365
886	0.95069	0.95484	0.95673	0.95735	0.95935	0.96144	0.96368
887	0.95075	0.95488	0.95677	0.95740	0.95938	0.96147	0.96372
888	0.95080	0.95492	0.95681	0.95743	0.95942	0.96151	0.96375
889	0.95085	0.95497	0.95685	0.95748	0.95946	0.96154	0.96379
890	0.95090	0.95501	0.95689	0.95752	0.95950	0.96158	0.96382
891	0.95094	0.95506	0.95693	0.95756	0.95954	0.96161	0.96385
892	0.95099	0.95510	0.95698	0.95760	0.95958	0.96165	0.96389
893	0.95104	0.95514	0.95702	0.95764	0.95961	0.96169	0.96392
894	0.95109	0.95518	0.95706	0.95768	0.95965	0.96172	0.96395
895	0.95113	0.95523	0.95710	0.95772	0.95969	0.96176	0.96399
896	0.95118	0.95527	0.95714	0.95776	0.95973	0.96179	0.96402
897	0.95122	0.95531	0.95718	0.95780	0.95977	0.96183	0.96405
898	0.95127	0.95536	0.95722	0.95784	0.95980	0.96187	0.96409
899	0.95132	0.95540	0.95726	0.95787	0.95984	0.96190	0.96412
900	0.95136	0.95544	0.95730	0.95792	0.95988	0.96194	0.96415
901	0.95141	0.95549	0.95734	0.95795	0.95992	0.96197	0.96419
902	0.95145	0.95553	0.95738	0.95799	0.95995	0.96201	0.96422
903	0.95150	0.95557	0.95742	0.95803	0.95999	0.96204	0.96425
904	0.95155	0.95561	0.95746	0.95807	0.96003	0.96208	0.96428
905	0.95159	0.95565	0.95750	0.95811	0.96007	0.96211	0.96432
906	0.95164	0.95569	0.95754	0.95815	0.96010	0.96215	0.96435
907	0.95168	0.95573	0.95758	0.95819	0.96014	0.96218	0.96438
908	0.95173	0.95577	0.95762	0.95823	0.96018	0.96221	0.96441
909	0.95178	0.95581	0.95766	0.95827	0.96021	0.96225	0.96445
910	0.95182	0.95586	0.95770	0.95831	0.96025	0.96228	0.96448
911	0.95187	0.95590	0.95774	0.95834	0.96029	0.96232	0.96451
912	0.95192	0.95594	0.95778	0.95838	0.96032	0.96235	0.96454
913	0.95196	0.95598	0.95781	0.95842	0.96036	0.96239	0.96458
914	0.95201	0.95602	0.95785	0.95846	0.96040	0.96242	0.96461
915	0.95205	0.95606	0.95789	0.95850	0.96043	0.96246	0.96464
916	0.95210	0.95610	0.95793	0.95854	0.96047	0.96249	0.96467
917	0.95214	0.95615	0.95797	0.95857	0.96050	0.96252	0.96470
918	0.95219	0.95618	0.95801	0.95861	0.96054	0.96256	0.96473
919	0.95223	0.95622	0.95805	0.95865	0.96058	0.96259	0.96477
920	0.95228	0.95626	0.95809	0.95869	0.96061	0.96263	0.96480
921	0.95232	0.95631	0.95812	0.95873	0.96065	0.96266	0.96483
922	0.95237	0.95635	0.95816	0.95876	0.96068	0.96269	0.96486
923	0.95241	0.95639	0.95820	0.95880	0.96072	0.96273	0.96489
924	0.95245	0.95643	0.95824	0.95884	0.96076	0.96276	0.96492
925	0.95249	0.95646	0.95828	0.95888	0.96079	0.96280	0.96495
926	0.95253	0.95650	0.95831	0.95892	0.96083	0.96283	0.96499
927	0.95258	0.95655	0.95835	0.95895	0.96086	0.96286	0.96502
928	0.95262	0.95658	0.95839	0.95899	0.96090	0.96290	0.96505
929	0.95267	0.95662	0.95843	0.95903	0.96093	0.96293	0.96508
930	0.95271	0.95666	0.95846	0.95907	0.96097	0.96296	0.96511
931	0.95276	0.95670	0.95850	0.95910	0.96100	0.96300	0.96514
932	0.95280	0.95675	0.95854	0.95914	0.96104	0.96303	0.96517
933	0.95284	0.95678	0.95858	0.95918	0.96107	0.96306	0.96520
934	0.95289	0.95682	0.95862	0.95921	0.96111	0.96309	0.96523
935	0.95293	0.95686	0.95865	0.95925	0.96114	0.96313	0.96526
936	0.95297	0.95690	0.95869	0.95929	0.96118	0.96316	0.96529
937	0.95302	0.95694	0.95873	0.95933	0.96121	0.96319	0.96533
938	0.95306	0.95698	0.95876	0.95936	0.96125	0.96323	0.96536
939	0.95310	0.95702	0.95880	0.95940	0.96128	0.96326	0.96539
940	0.95314	0.95706	0.95884	0.95944	0.96132	0.96329	0.96542
941	0.95318	0.95709	0.95887	0.95947	0.96135	0.96332	0.96545
942	0.95323	0.95713	0.95891	0.95951	0.96139	0.96336	0.96548
943	0.95327	0.95718	0.95895	0.95955	0.96142	0.96339	0.96551
944	0.95331	0.95721	0.95899	0.95958	0.96146	0.96342	0.96554
945	0.95335	0.95725	0.95902	0.95962	0.96149	0.96346	0.96557
946	0.95339	0.95729	0.95906	0.95965	0.96153	0.96349	0.96560
947	0.95344	0.95733	0.95910	0.95969	0.96156	0.96352	0.96563
948	0.95348	0.95736	0.95914	0.95973	0.96159	0.96355	0.96566
949	0.95352	0.95741	0.95917	0.95976	0.96163	0.96358	0.96569
950	0.95356	0.95744	0.95921	0.95980	0.96166	0.96362	0.96572
951	0.95361	0.95748	0.95925	0.95983	0.96170	0.96365	0.96575
952	0.95365	0.95752	0.95928	0.95987	0.96173	0.96368	0.96578
953	0.95370	0.95756	0.95932	0.95990	0.96176	0.96371	0.96581
954	0.95374	0.95760	0.95936	0.95994	0.96180	0.96374	0.96584
955	0.95379	0.95764	0.95939	0.95998	0.96183	0.96378	0.96587
956	0.95383	0.95768	0.95943	0.96001	0.96186	0.96381	0.96590
957	0.95386	0.95771	0.95946	0.96005	0.96190	0.96384	0.96593
958	0.95390	0.95775	0.95950	0.96008	0.96193	0.96387	0.96595

N	0.1%	0.5%	1%	1.25%	2.5%	5%	10%
959	0.95395	0.95779	0.95954	0.96012	0.96196	0.96390	0.96598
960	0.95399	0.95782	0.95957	0.96015	0.96200	0.96393	0.96601
961	0.95403	0.95786	0.95961	0.96019	0.96203	0.96396	0.96604
962	0.95407	0.95790	0.95964	0.96022	0.96206	0.96400	0.96607
963	0.95411	0.95793	0.95968	0.96026	0.96210	0.96403	0.96610
964	0.95415	0.95797	0.95972	0.96029	0.96213	0.96406	0.96613
965	0.95420	0.95801	0.95975	0.96033	0.96216	0.96409	0.96616
966	0.95423	0.95804	0.95978	0.96036	0.96220	0.96412	0.96619
967	0.95427	0.95808	0.95982	0.96039	0.96223	0.96415	0.96622
968	0.95431	0.95812	0.95985	0.96043	0.96226	0.96418	0.96625
969	0.95435	0.95815	0.95989	0.96046	0.96229	0.96421	0.96628
970	0.95439	0.95819	0.95992	0.96050	0.96232	0.96424	0.96630
971	0.95443	0.95823	0.95996	0.96053	0.96236	0.96427	0.96633
972	0.95447	0.95827	0.95999	0.96057	0.96239	0.96431	0.96636
973	0.95451	0.95830	0.96003	0.96060	0.96242	0.96434	0.96639
974	0.95455	0.95834	0.96006	0.96063	0.96245	0.96437	0.96642
975	0.95459	0.95837	0.96010	0.96067	0.96249	0.96440	0.96645
976	0.95463	0.95841	0.96013	0.96070	0.96252	0.96443	0.96648
977	0.95467	0.95845	0.96017	0.96074	0.96255	0.96446	0.96650
978	0.95471	0.95848	0.96020	0.96077	0.96258	0.96449	0.96653
979	0.95475	0.95852	0.96024	0.96080	0.96262	0.96452	0.96656
980	0.95479	0.95855	0.96027	0.96084	0.96265	0.96455	0.96659
981	0.95482	0.95859	0.96031	0.96087	0.96268	0.96458	0.96662
982	0.95486	0.95863	0.96034	0.96090	0.96271	0.96461	0.96664
983	0.95491	0.95866	0.96037	0.96094	0.96274	0.96464	0.96667
984	0.95495	0.95870	0.96041	0.96097	0.96277	0.96467	0.96670
985	0.95498	0.95873	0.96044	0.96100	0.96281	0.96470	0.96673
986	0.95502	0.95877	0.96047	0.96104	0.96284	0.96473	0.96676
987	0.95506	0.95881	0.96051	0.96107	0.96287	0.96476	0.96679
988	0.95510	0.95884	0.96054	0.96110	0.96290	0.96479	0.96681
989	0.95515	0.95888	0.96058	0.96114	0.96293	0.96482	0.96684
990	0.95518	0.95891	0.96061	0.96117	0.96297	0.96485	0.96687
991	0.95522	0.95895	0.96065	0.96120	0.96300	0.96488	0.96690
992	0.95526	0.95899	0.96068	0.96124	0.96303	0.96491	0.96693
993	0.95529	0.95902	0.96071	0.96127	0.96306	0.96494	0.96695
994	0.95533	0.95905	0.96075	0.96130	0.96309	0.96497	0.96698
995	0.95537	0.95909	0.96078	0.96134	0.96312	0.96500	0.96701
996	0.95541	0.95913	0.96081	0.96137	0.96315	0.96503	0.96704
997	0.95544	0.95916	0.96085	0.96141	0.96318	0.96506	0.96706
998	0.95548	0.95920	0.96088	0.96144	0.96322	0.96509	0.96709
999	0.95551	0.95923	0.96092	0.96147	0.96325	0.96511	0.96712
1000	0.95555	0.95927	0.96095	0.96150	0.96328	0.96514	0.96714

Table 2 – *GT(HHL or HLL)*. Critical values of upper significance levels for the Grubb triple outlier test, applicable in situations where the two highest and one lowest or one highest and two lowest observations are simultaneously identified as outliers, denoted as *GT(HHL)* and *GT(HLL)*, respectively.

N	0.1%	0.5%	1%	1.25%	2.5%	5%	10%
5	0.00000	0.00000	0.00001	0.00001	0.00005	0.00018	0.00076
6	0.00011	0.00055	0.00111	0.00139	0.00285	0.00589	0.01241
7	0.00144	0.00432	0.00699	0.00817	0.01332	0.02195	0.03672
8	0.00565	0.01306	0.01884	0.02122	0.03085	0.04524	0.06719
9	0.01336	0.02631	0.03540	0.03900	0.05285	0.07216	0.09958
10	0.02424	0.04287	0.05505	0.05972	0.07716	0.10035	0.13174
11	0.03766	0.06159	0.07645	0.08203	0.10239	0.12853	0.16272
12	0.05288	0.08153	0.09865	0.10497	0.12763	0.15597	0.19210
13	0.06929	0.10205	0.12101	0.12792	0.15236	0.18232	0.21974
14	0.08646	0.12268	0.14314	0.15050	0.17630	0.20744	0.24566
15	0.10399	0.14311	0.16477	0.17250	0.19931	0.23124	0.26993
16	0.12156	0.16317	0.18574	0.19374	0.22130	0.25377	0.29264
17	0.13907	0.18268	0.20595	0.21416	0.24226	0.27503	0.31393
18	0.15638	0.20158	0.22538	0.23374	0.26219	0.29512	0.33388
19	0.17333	0.21978	0.24402	0.25249	0.28116	0.31410	0.35262
20	0.18991	0.23735	0.26188	0.27042	0.29919	0.33206	0.37024
21	0.20604	0.25427	0.27898	0.28754	0.31634	0.34904	0.38684
22	0.22172	0.27052	0.29533	0.30390	0.33263	0.36514	0.40249
23	0.23686	0.28610	0.31096	0.31953	0.34818	0.38041	0.41729
24	0.25158	0.30107	0.32593	0.33447	0.36297	0.39489	0.43130
25	0.26581	0.31546	0.34027	0.34878	0.37707	0.40867	0.44457
26	0.27950	0.32927	0.35398	0.36244	0.39051	0.42177	0.45717
27	0.29282	0.34255	0.36712	0.37552	0.40335	0.43425	0.46914
28	0.30566	0.35530	0.37973	0.38806	0.41564	0.44615	0.48053
29	0.31804	0.36754	0.39182	0.40008	0.42737	0.45752	0.49139

N	0.1%	0.5%	1%	1.25%	2.5%	5%	10%
30	0.33001	0.37935	0.40344	0.41163	0.43863	0.46838	0.50176
31	0.34159	0.39069	0.41458	0.42270	0.44940	0.47878	0.51165
32	0.35280	0.40161	0.42531	0.43334	0.45973	0.48873	0.52111
33	0.36363	0.41213	0.43562	0.44356	0.46966	0.49827	0.53018
34	0.37407	0.42228	0.44554	0.45340	0.47919	0.50743	0.53886
35	0.38419	0.43205	0.45509	0.46287	0.48835	0.51622	0.54719
36	0.39398	0.44147	0.46428	0.47198	0.49717	0.52467	0.55519
37	0.40350	0.45058	0.47316	0.48078	0.50566	0.53280	0.56286
38	0.41265	0.45937	0.48173	0.48926	0.51384	0.54061	0.57026
39	0.42157	0.46788	0.48999	0.49743	0.52173	0.54816	0.57737
40	0.43019	0.47608	0.49797	0.50532	0.52934	0.55542	0.58422
41	0.43855	0.48405	0.50567	0.51295	0.53669	0.56244	0.59083
42	0.44663	0.49174	0.51314	0.52034	0.54379	0.56920	0.59720
43	0.45449	0.49919	0.52037	0.52748	0.55066	0.57574	0.60335
44	0.46215	0.50642	0.52738	0.53441	0.55731	0.58207	0.60931
45	0.46957	0.51343	0.53414	0.54110	0.56373	0.58818	0.61505
46	0.47676	0.52022	0.54071	0.54759	0.56995	0.59410	0.62062
47	0.48375	0.52680	0.54709	0.55388	0.57598	0.59984	0.62600
48	0.49055	0.53319	0.55326	0.55999	0.58183	0.60539	0.63121
49	0.49716	0.53939	0.55927	0.56593	0.58750	0.61077	0.63626
50	0.50359	0.54543	0.56509	0.57167	0.59301	0.61599	0.64116
51	0.50986	0.55129	0.57075	0.57726	0.59835	0.62107	0.64592
52	0.51595	0.55701	0.57625	0.58269	0.60355	0.62599	0.65053
53	0.52187	0.56255	0.58161	0.58798	0.60860	0.63078	0.65501
54	0.52765	0.56795	0.58681	0.59312	0.61351	0.63542	0.65937
55	0.53328	0.57321	0.59188	0.59812	0.61828	0.63995	0.66359
56	0.53879	0.57833	0.59681	0.60298	0.62294	0.64435	0.66771
57	0.54416	0.58333	0.60161	0.60773	0.62746	0.64862	0.67171
58	0.54941	0.58818	0.60629	0.61233	0.63186	0.65279	0.67561
59	0.55451	0.59293	0.61084	0.61682	0.63615	0.65685	0.67940
60	0.55946	0.59755	0.61529	0.62121	0.64033	0.66080	0.68310
61	0.56429	0.60206	0.61962	0.62548	0.64441	0.66466	0.68670
62	0.56904	0.60648	0.62385	0.62965	0.64838	0.66841	0.69021
63	0.57369	0.61077	0.62799	0.63373	0.65226	0.67207	0.69363
64	0.57822	0.61496	0.63202	0.63770	0.65605	0.67565	0.69697
65	0.58265	0.61906	0.63596	0.64159	0.65974	0.67914	0.70023
66	0.58700	0.62306	0.63980	0.64538	0.66335	0.68255	0.70341
67	0.59120	0.62698	0.64356	0.64908	0.66688	0.68588	0.70652
68	0.59535	0.63081	0.64724	0.65271	0.67032	0.68913	0.70955
69	0.59942	0.63456	0.65083	0.65624	0.67369	0.69231	0.71252
70	0.60338	0.63822	0.65434	0.65970	0.67698	0.69541	0.71542
71	0.60727	0.64180	0.65777	0.66308	0.68020	0.69845	0.71825
72	0.61106	0.64530	0.66114	0.66640	0.68335	0.70142	0.72102
73	0.61479	0.64873	0.66442	0.66964	0.68643	0.70433	0.72373
74	0.61844	0.65208	0.66764	0.67281	0.68945	0.70717	0.72639
75	0.62200	0.65538	0.67080	0.67592	0.69240	0.70996	0.72898
76	0.62550	0.65862	0.67388	0.67896	0.69530	0.71268	0.73152
77	0.62895	0.66177	0.67691	0.68194	0.69813	0.71535	0.73401
78	0.63232	0.66487	0.67988	0.68487	0.70090	0.71797	0.73645
79	0.63563	0.66790	0.68278	0.68773	0.70362	0.72053	0.73883
80	0.63887	0.67088	0.68564	0.69053	0.70629	0.72304	0.74117
81	0.64206	0.67380	0.68843	0.69328	0.70890	0.72551	0.74347
82	0.64518	0.67666	0.69117	0.69598	0.71146	0.72792	0.74572
83	0.64823	0.67947	0.69386	0.69863	0.71397	0.73029	0.74792
84	0.65124	0.68223	0.69650	0.70123	0.71644	0.73261	0.75009
85	0.65421	0.68493	0.69908	0.70377	0.71886	0.73488	0.75221
86	0.65710	0.68759	0.70162	0.70627	0.72123	0.73712	0.75429
87	0.65993	0.69020	0.70412	0.70874	0.72356	0.73931	0.75634
88	0.66274	0.69276	0.70657	0.71115	0.72585	0.74147	0.75834
89	0.66548	0.69528	0.70898	0.71352	0.72810	0.74358	0.76031
90	0.66817	0.69775	0.71133	0.71584	0.73031	0.74566	0.76224
91	0.67081	0.70018	0.71366	0.71812	0.73248	0.74770	0.76414
92	0.67343	0.70257	0.71594	0.72036	0.73460	0.74970	0.76601
93	0.67599	0.70491	0.71817	0.72257	0.73669	0.75167	0.76784
94	0.67853	0.70721	0.72037	0.72474	0.73875	0.75361	0.76965
95	0.68099	0.70948	0.72254	0.72687	0.74077	0.75551	0.77142
96	0.68342	0.71170	0.72467	0.72897	0.74276	0.75738	0.77316
97	0.68583	0.71390	0.72676	0.73103	0.74471	0.75922	0.77487
98	0.68818	0.71606	0.72883	0.73306	0.74663	0.76102	0.77655
99	0.69050	0.71818	0.73085	0.73504	0.74852	0.76280	0.77821
100	0.69279	0.72027	0.73284	0.73700	0.75038	0.76455	0.77983
101	0.69503	0.72232	0.73480	0.73894	0.75221	0.76627	0.78144
102	0.69724	0.72434	0.73673	0.74083	0.75401	0.76796	0.78301
103	0.69942	0.72633	0.73864	0.74270	0.75578	0.76963	0.78456

N	0.1%	0.5%	1%	1.25%	2.5%	5%	10%
104	0.70158	0.72828	0.74050	0.74455	0.75752	0.77127	0.78609
105	0.70369	0.73021	0.74234	0.74636	0.75924	0.77288	0.78759
106	0.70576	0.73211	0.74416	0.74814	0.76093	0.77447	0.78907
107	0.70781	0.73398	0.74594	0.74990	0.76259	0.77604	0.79053
108	0.70982	0.73582	0.74769	0.75162	0.76423	0.77758	0.79196
109	0.71181	0.73764	0.74942	0.75332	0.76585	0.77910	0.79337
110	0.71377	0.73942	0.75112	0.75501	0.76743	0.78059	0.79477
111	0.71570	0.74118	0.75281	0.75666	0.76900	0.78206	0.79614
112	0.71761	0.74292	0.75447	0.75829	0.77054	0.78351	0.79749
113	0.71950	0.74463	0.75610	0.75990	0.77206	0.78495	0.79882
114	0.72133	0.74632	0.75771	0.76148	0.77356	0.78635	0.80013
115	0.72315	0.74798	0.75930	0.76304	0.77504	0.78774	0.80142
116	0.72496	0.74963	0.76086	0.76458	0.77650	0.78911	0.80269
117	0.72673	0.75124	0.76240	0.76610	0.77793	0.79046	0.80395
118	0.72849	0.75283	0.76392	0.76759	0.77935	0.79180	0.80519
119	0.73021	0.75440	0.76542	0.76906	0.78075	0.79311	0.80641
120	0.73190	0.75595	0.76690	0.77052	0.78212	0.79440	0.80762
121	0.73358	0.75748	0.76836	0.77195	0.78348	0.79568	0.80881
122	0.73522	0.75899	0.76979	0.77337	0.78482	0.79694	0.80998
123	0.73685	0.76048	0.77122	0.77477	0.78614	0.79818	0.81113
124	0.73848	0.76195	0.77261	0.77615	0.78745	0.79941	0.81227
125	0.74007	0.76339	0.77399	0.77750	0.78873	0.80062	0.81340
126	0.74164	0.76482	0.77536	0.77884	0.79000	0.80181	0.81451
127	0.74319	0.76623	0.77670	0.78016	0.79125	0.80299	0.81560
128	0.74472	0.76762	0.77803	0.78147	0.79249	0.80415	0.81669
129	0.74624	0.76899	0.77934	0.78276	0.79371	0.80530	0.81775
130	0.74773	0.77035	0.78063	0.78403	0.79491	0.80643	0.81881
131	0.74921	0.77169	0.78191	0.78529	0.79610	0.80755	0.81985
132	0.75067	0.77301	0.78317	0.78653	0.79728	0.80865	0.82088
133	0.75210	0.77431	0.78441	0.78775	0.79844	0.80974	0.82189
134	0.75352	0.77560	0.78564	0.78896	0.79959	0.81082	0.82289
135	0.75493	0.77687	0.78686	0.79016	0.80072	0.81188	0.82388
136	0.75631	0.77813	0.78806	0.79134	0.80184	0.81293	0.82486
137	0.75768	0.77937	0.78925	0.79250	0.80294	0.81397	0.82582
138	0.75904	0.78060	0.79042	0.79366	0.80403	0.81500	0.82678
139	0.76037	0.78181	0.79157	0.79480	0.80511	0.81601	0.82772
140	0.76169	0.78301	0.79272	0.79592	0.80618	0.81701	0.82865
141	0.76300	0.78420	0.79385	0.79703	0.80723	0.81800	0.82957
142	0.76428	0.78537	0.79496	0.79813	0.80827	0.81897	0.83048
143	0.76555	0.78653	0.79607	0.79921	0.80929	0.81994	0.83138
144	0.76681	0.78767	0.79716	0.80029	0.81031	0.82089	0.83227
145	0.76806	0.78880	0.79824	0.80135	0.81131	0.82184	0.83314
146	0.76928	0.78992	0.79930	0.80240	0.81231	0.82277	0.83401
147	0.77050	0.79103	0.80035	0.80343	0.81329	0.82369	0.83487
148	0.77171	0.79212	0.80140	0.80446	0.81426	0.82460	0.83572
149	0.77289	0.79320	0.80242	0.80547	0.81521	0.82550	0.83656
150	0.77408	0.79427	0.80344	0.80647	0.81616	0.82639	0.83738
151	0.77523	0.79532	0.80445	0.80746	0.81710	0.82727	0.83820
152	0.77639	0.79637	0.80544	0.80844	0.81803	0.82815	0.83901
153	0.77752	0.79740	0.80643	0.80941	0.81894	0.82901	0.83981
154	0.77864	0.79842	0.80740	0.81037	0.81985	0.82986	0.84061
155	0.77974	0.79944	0.80837	0.81132	0.82075	0.83070	0.84139
156	0.78086	0.80044	0.80932	0.81225	0.82164	0.83154	0.84217
157	0.78194	0.80143	0.81026	0.81318	0.82251	0.83236	0.84294
158	0.78302	0.80240	0.81119	0.81410	0.82338	0.83318	0.84369
159	0.78407	0.80337	0.81212	0.81500	0.82424	0.83398	0.84445
160	0.78514	0.80432	0.81303	0.81590	0.82509	0.83478	0.84519
161	0.78619	0.80527	0.81393	0.81679	0.82593	0.83557	0.84593
162	0.78722	0.80621	0.81483	0.81767	0.82676	0.83635	0.84665
163	0.78825	0.80714	0.81571	0.81854	0.82759	0.83713	0.84737
164	0.78925	0.80806	0.81659	0.81940	0.82840	0.83789	0.84808
165	0.79025	0.80897	0.81745	0.82026	0.82921	0.83865	0.84879
166	0.79124	0.80986	0.81831	0.82110	0.83001	0.83940	0.84949
167	0.79223	0.81075	0.81916	0.82193	0.83080	0.84015	0.85018
168	0.79320	0.81163	0.82000	0.82276	0.83158	0.84088	0.85086
169	0.79416	0.81251	0.82083	0.82357	0.83235	0.84161	0.85154
170	0.79511	0.81337	0.82165	0.82438	0.83312	0.84233	0.85221
171	0.79605	0.81422	0.82247	0.82519	0.83388	0.84304	0.85288
172	0.79698	0.81507	0.82328	0.82598	0.83463	0.84375	0.85353
173	0.79791	0.81591	0.82408	0.82676	0.83537	0.84445	0.85419
174	0.79882	0.81674	0.82487	0.82755	0.83611	0.84514	0.85483
175	0.79972	0.81756	0.82565	0.82832	0.83684	0.84583	0.85547
176	0.80062	0.81838	0.82642	0.82908	0.83756	0.84650	0.85610
177	0.80151	0.81919	0.82719	0.82983	0.83828	0.84718	0.85673

N	0.1%	0.5%	1%	1.25%	2.5%	5%	10%
178	0.80239	0.81998	0.82795	0.83058	0.83898	0.84784	0.85735
179	0.80326	0.82078	0.82871	0.83132	0.83969	0.84850	0.85796
180	0.80413	0.82156	0.82946	0.83206	0.84038	0.84916	0.85857
181	0.80499	0.82234	0.83020	0.83279	0.84107	0.84980	0.85917
182	0.80583	0.82311	0.83093	0.83351	0.84175	0.85044	0.85977
183	0.80667	0.82387	0.83165	0.83422	0.84243	0.85108	0.86036
184	0.80750	0.82462	0.83237	0.83493	0.84310	0.85171	0.86095
185	0.80832	0.82537	0.83309	0.83563	0.84376	0.85233	0.86153
186	0.80914	0.82611	0.83379	0.83632	0.84442	0.85295	0.86210
187	0.80996	0.82685	0.83449	0.83701	0.84507	0.85356	0.86267
188	0.81075	0.82757	0.83518	0.83770	0.84571	0.85417	0.86324
189	0.81154	0.82829	0.83587	0.83837	0.84635	0.85477	0.86380
190	0.81233	0.82901	0.83655	0.83904	0.84699	0.85537	0.86435
191	0.81312	0.82972	0.83723	0.83970	0.84762	0.85596	0.86490
192	0.81390	0.83042	0.83790	0.84036	0.84824	0.85654	0.86545
193	0.81466	0.83111	0.83856	0.84101	0.84886	0.85712	0.86599
194	0.81542	0.83180	0.83922	0.84166	0.84947	0.85770	0.86652
195	0.81617	0.83249	0.83987	0.84230	0.85007	0.85827	0.86705
196	0.81692	0.83316	0.84051	0.84294	0.85068	0.85883	0.86758
197	0.81765	0.83384	0.84115	0.84356	0.85127	0.85939	0.86810
198	0.81838	0.83450	0.84179	0.84419	0.85186	0.85995	0.86862
199	0.81911	0.83516	0.84242	0.84481	0.85245	0.86050	0.86913
200	0.81983	0.83582	0.84304	0.84542	0.85303	0.86104	0.86964
201	0.82048	0.83645	0.84361	0.84601	0.85358	0.86155	0.87013
202	0.82120	0.83710	0.84424	0.84660	0.85414	0.86210	0.87064
203	0.82191	0.83773	0.84486	0.84721	0.85471	0.86263	0.87113
204	0.82262	0.83837	0.84546	0.84781	0.85527	0.86315	0.87162
205	0.82335	0.83898	0.84607	0.84840	0.85583	0.86368	0.87211
206	0.82402	0.83960	0.84666	0.84898	0.85638	0.86420	0.87259
207	0.82470	0.84023	0.84726	0.84957	0.85692	0.86471	0.87307
208	0.82536	0.84084	0.84784	0.85014	0.85747	0.86522	0.87355
209	0.82607	0.84144	0.84842	0.85071	0.85801	0.86572	0.87402
210	0.82671	0.84204	0.84898	0.85126	0.85853	0.86622	0.87448
211	0.82733	0.84265	0.84955	0.85183	0.85907	0.86672	0.87494
212	0.82799	0.84323	0.85011	0.85238	0.85960	0.86721	0.87540
213	0.82858	0.84383	0.85067	0.85293	0.86012	0.86769	0.87585
214	0.82927	0.84440	0.85122	0.85347	0.86064	0.86818	0.87631
215	0.82995	0.84497	0.85177	0.85401	0.86115	0.86867	0.87676
216	0.83061	0.84553	0.85231	0.85455	0.86165	0.86915	0.87720
217	0.83126	0.84610	0.85286	0.85507	0.86216	0.86962	0.87765
218	0.83190	0.84667	0.85339	0.85560	0.86266	0.87010	0.87809
219	0.83250	0.84723	0.85392	0.85612	0.86315	0.87056	0.87852
220	0.83312	0.84779	0.85446	0.85664	0.86365	0.87102	0.87895
221	0.83362	0.84834	0.85496	0.85716	0.86414	0.87148	0.87938
222	0.83426	0.84888	0.85549	0.85766	0.86463	0.87193	0.87980
223	0.83482	0.84944	0.85601	0.85816	0.86510	0.87238	0.88022
224	0.83544	0.84998	0.85653	0.85867	0.86558	0.87283	0.88065
225	0.83605	0.85051	0.85704	0.85916	0.86606	0.87329	0.88107
226	0.83660	0.85103	0.85754	0.85967	0.86653	0.87373	0.88148
227	0.83714	0.85156	0.85803	0.86018	0.86700	0.87416	0.88189
228	0.83770	0.85207	0.85853	0.86066	0.86747	0.87460	0.88230
229	0.83826	0.85259	0.85903	0.86115	0.86792	0.87503	0.88270
230	0.83882	0.85310	0.85952	0.86163	0.86838	0.87546	0.88309
231	0.83941	0.85360	0.86001	0.86210	0.86882	0.87589	0.88349
232	0.83994	0.85410	0.86049	0.86259	0.86927	0.87631	0.88388
233	0.84048	0.85459	0.86096	0.86306	0.86972	0.87673	0.88427
234	0.84098	0.85509	0.86144	0.86353	0.87017	0.87715	0.88466
235	0.84154	0.85557	0.86191	0.86398	0.87059	0.87757	0.88505
236	0.84207	0.85607	0.86237	0.86444	0.87103	0.87798	0.88543
237	0.84263	0.85655	0.86282	0.86489	0.87146	0.87838	0.88581
238	0.84316	0.85703	0.86328	0.86534	0.87189	0.87879	0.88618
239	0.84371	0.85751	0.86374	0.86579	0.87232	0.87919	0.88656
240	0.84422	0.85800	0.86420	0.86624	0.87274	0.87959	0.88693
241	0.84474	0.85848	0.86465	0.86669	0.87317	0.87999	0.88729
242	0.84530	0.85894	0.86510	0.86714	0.87358	0.88038	0.88767
243	0.84581	0.85939	0.86554	0.86757	0.87401	0.88077	0.88803
244	0.84636	0.85986	0.86599	0.86801	0.87441	0.88116	0.88839
245	0.84689	0.86032	0.86642	0.86844	0.87482	0.88154	0.88875
246	0.84736	0.86078	0.86686	0.86887	0.87523	0.88193	0.88911
247	0.84789	0.86124	0.86730	0.86931	0.87564	0.88231	0.88946
248	0.84838	0.86168	0.86773	0.86972	0.87603	0.88269	0.88981
249	0.84888	0.86214	0.86815	0.87015	0.87643	0.88306	0.89016
250	0.84937	0.86257	0.86857	0.87055	0.87682	0.88344	0.89050
251	0.84987	0.86300	0.86899	0.87096	0.87722	0.88381	0.89085

N	0.1%	0.5%	1%	1.25%	2.5%	5%	10%
252	0.85032	0.86345	0.86941	0.87138	0.87761	0.88417	0.89119
253	0.85079	0.86388	0.86983	0.87178	0.87799	0.88454	0.89153
254	0.85128	0.86431	0.87025	0.87219	0.87837	0.88489	0.89186
255	0.85179	0.86475	0.87066	0.87259	0.87875	0.88525	0.89219
256	0.85224	0.86516	0.87107	0.87299	0.87913	0.88561	0.89253
257	0.85271	0.86559	0.87147	0.87338	0.87950	0.88596	0.89286
258	0.85312	0.86602	0.87187	0.87378	0.87988	0.88631	0.89319
259	0.85362	0.86644	0.87227	0.87417	0.88025	0.88666	0.89352
260	0.85406	0.86686	0.87267	0.87455	0.88062	0.88701	0.89384
261	0.85454	0.86726	0.87305	0.87493	0.88099	0.88735	0.89416
262	0.85496	0.86766	0.87343	0.87532	0.88135	0.88770	0.89448
263	0.85540	0.86808	0.87381	0.87569	0.88171	0.88804	0.89480
264	0.85583	0.86849	0.87421	0.87608	0.88206	0.88838	0.89511
265	0.85625	0.86888	0.87459	0.87645	0.88242	0.88871	0.89543
266	0.85673	0.86928	0.87498	0.87683	0.88277	0.88904	0.89574
267	0.85713	0.86968	0.87535	0.87720	0.88313	0.88937	0.89605
268	0.85758	0.87007	0.87571	0.87757	0.88348	0.88970	0.89635
269	0.85803	0.87047	0.87609	0.87794	0.88383	0.89003	0.89666
270	0.85843	0.87086	0.87646	0.87830	0.88417	0.89036	0.89696
271	0.85887	0.87124	0.87683	0.87866	0.88451	0.89068	0.89726
272	0.85931	0.87162	0.87720	0.87902	0.88485	0.89100	0.89756
273	0.85975	0.87200	0.87756	0.87937	0.88519	0.89132	0.89786
274	0.86016	0.87239	0.87792	0.87973	0.88554	0.89164	0.89815
275	0.86059	0.87275	0.87827	0.88007	0.88586	0.89195	0.89844
276	0.86098	0.87314	0.87863	0.88043	0.88620	0.89227	0.89874
277	0.86141	0.87353	0.87898	0.88078	0.88653	0.89257	0.89903
278	0.86181	0.87389	0.87934	0.88113	0.88686	0.89289	0.89932
279	0.86223	0.87426	0.87968	0.88147	0.88719	0.89320	0.89960
280	0.86263	0.87461	0.88003	0.88181	0.88751	0.89350	0.89988
281	0.86304	0.87498	0.88038	0.88216	0.88783	0.89380	0.90017
282	0.86345	0.87533	0.88073	0.88250	0.88815	0.89411	0.90045
283	0.86385	0.87569	0.88108	0.88284	0.88847	0.89441	0.90073
284	0.86423	0.87606	0.88141	0.88317	0.88879	0.89470	0.90101
285	0.86462	0.87640	0.88175	0.88350	0.88910	0.89499	0.90129
286	0.86502	0.87676	0.88208	0.88383	0.88941	0.89529	0.90156
287	0.86539	0.87710	0.88241	0.88416	0.88972	0.89558	0.90183
288	0.86577	0.87745	0.88275	0.88448	0.89003	0.89586	0.90210
289	0.86615	0.87781	0.88308	0.88480	0.89033	0.89616	0.90237
290	0.86653	0.87815	0.88341	0.88514	0.89064	0.89644	0.90264
291	0.86689	0.87850	0.88373	0.88546	0.89094	0.89673	0.90291
292	0.86730	0.87885	0.88405	0.88578	0.89124	0.89701	0.90317
293	0.86764	0.87919	0.88438	0.88609	0.89154	0.89729	0.90343
294	0.86801	0.87952	0.88470	0.88641	0.89184	0.89757	0.90369
295	0.86835	0.87984	0.88503	0.88673	0.89214	0.89784	0.90395
296	0.86869	0.88018	0.88534	0.88703	0.89242	0.89812	0.90421
297	0.86906	0.88051	0.88566	0.88735	0.89272	0.89839	0.90446
298	0.86943	0.88084	0.88596	0.88765	0.89302	0.89867	0.90472
299	0.86982	0.88116	0.88627	0.88795	0.89330	0.89894	0.90497
300	0.87017	0.88149	0.88657	0.88826	0.89359	0.89921	0.90523
301	0.87055	0.88182	0.88688	0.88855	0.89388	0.89948	0.90548
302	0.87089	0.88214	0.88719	0.88885	0.89416	0.89974	0.90573
303	0.87122	0.88245	0.88749	0.88915	0.89444	0.90001	0.90598
304	0.87157	0.88276	0.88778	0.88944	0.89471	0.90027	0.90622
305	0.87194	0.88308	0.88809	0.88974	0.89500	0.90053	0.90647
306	0.87226	0.88340	0.88839	0.89003	0.89527	0.90080	0.90671
307	0.87264	0.88370	0.88869	0.89032	0.89555	0.90106	0.90695
308	0.87295	0.88401	0.88898	0.89061	0.89583	0.90132	0.90719
309	0.87330	0.88431	0.88928	0.89090	0.89610	0.90158	0.90743
310	0.87363	0.88462	0.88956	0.89119	0.89636	0.90183	0.90767
311	0.87395	0.88492	0.88986	0.89147	0.89664	0.90209	0.90790
312	0.87432	0.88524	0.89014	0.89176	0.89690	0.90234	0.90814
313	0.87464	0.88552	0.89043	0.89204	0.89717	0.90260	0.90837
314	0.87495	0.88584	0.89072	0.89232	0.89743	0.90284	0.90860
315	0.87529	0.88613	0.89100	0.89261	0.89769	0.90309	0.90884
316	0.87562	0.88643	0.89130	0.89288	0.89796	0.90334	0.90907
317	0.87594	0.88672	0.89157	0.89315	0.89822	0.90358	0.90930
318	0.87625	0.88702	0.89184	0.89342	0.89848	0.90383	0.90953
319	0.87656	0.88731	0.89212	0.89370	0.89873	0.90407	0.90975
320	0.87690	0.88759	0.89240	0.89396	0.89899	0.90431	0.90998
321	0.87724	0.88788	0.89268	0.89424	0.89925	0.90454	0.91020
322	0.87753	0.88817	0.89295	0.89451	0.89950	0.90478	0.91042
323	0.87786	0.88845	0.89322	0.89477	0.89975	0.90502	0.91065
324	0.87815	0.88873	0.89348	0.89503	0.90001	0.90526	0.91087
325	0.87845	0.88902	0.89375	0.89529	0.90026	0.90549	0.91108

N	0.1%	0.5%	1%	1.25%	2.5%	5%	10%
326	0.87876	0.88929	0.89401	0.89556	0.90051	0.90572	0.91130
327	0.87905	0.88956	0.89428	0.89581	0.90075	0.90596	0.91152
328	0.87934	0.88985	0.89454	0.89607	0.90100	0.90619	0.91173
329	0.87964	0.89013	0.89481	0.89633	0.90124	0.90641	0.91195
330	0.87994	0.89039	0.89507	0.89658	0.90148	0.90664	0.91216
331	0.88026	0.89066	0.89532	0.89684	0.90173	0.90687	0.91238
332	0.88056	0.89094	0.89558	0.89709	0.90197	0.90710	0.91259
333	0.88084	0.89120	0.89584	0.89734	0.90220	0.90732	0.91280
334	0.88114	0.89147	0.89609	0.89759	0.90244	0.90755	0.91301
335	0.88143	0.89174	0.89634	0.89784	0.90268	0.90777	0.91321
336	0.88173	0.89201	0.89659	0.89809	0.90291	0.90799	0.91342
337	0.88204	0.89227	0.89684	0.89834	0.90315	0.90821	0.91363
338	0.88233	0.89254	0.89710	0.89858	0.90338	0.90843	0.91384
339	0.88262	0.89280	0.89734	0.89883	0.90361	0.90865	0.91404
340	0.88291	0.89306	0.89759	0.89907	0.90384	0.90886	0.91424
341	0.88318	0.89331	0.89784	0.89931	0.90407	0.90908	0.91444
342	0.88347	0.89357	0.89808	0.89955	0.90430	0.90930	0.91465
343	0.88376	0.89383	0.89832	0.89979	0.90453	0.90951	0.91484
344	0.88407	0.89408	0.89857	0.90003	0.90475	0.90973	0.91504
345	0.88435	0.89434	0.89881	0.90026	0.90498	0.90994	0.91524
346	0.88462	0.89459	0.89905	0.90050	0.90520	0.91015	0.91543
347	0.88490	0.89484	0.89929	0.90074	0.90543	0.91036	0.91563
348	0.88517	0.89510	0.89952	0.90098	0.90565	0.91057	0.91583
349	0.88544	0.89535	0.89976	0.90121	0.90587	0.91077	0.91602
350	0.88572	0.89559	0.89999	0.90144	0.90608	0.91098	0.91621
351	0.88598	0.89585	0.90024	0.90168	0.90630	0.91118	0.91640
352	0.88624	0.89608	0.90046	0.90190	0.90652	0.91138	0.91659
353	0.88652	0.89633	0.90069	0.90213	0.90674	0.91159	0.91678
354	0.88677	0.89657	0.90093	0.90235	0.90696	0.91179	0.91697
355	0.88703	0.89681	0.90116	0.90258	0.90717	0.91200	0.91716
356	0.88729	0.89704	0.90138	0.90281	0.90738	0.91220	0.91735
357	0.88756	0.89728	0.90161	0.90303	0.90760	0.91240	0.91753
358	0.88781	0.89752	0.90184	0.90325	0.90781	0.91259	0.91772
359	0.88807	0.89775	0.90206	0.90347	0.90802	0.91279	0.91790
360	0.88834	0.89798	0.90228	0.90370	0.90822	0.91299	0.91809
361	0.88858	0.89822	0.90250	0.90391	0.90843	0.91319	0.91827
362	0.88887	0.89845	0.90272	0.90413	0.90864	0.91338	0.91845
363	0.88912	0.89868	0.90295	0.90435	0.90885	0.91358	0.91863
364	0.88936	0.89891	0.90317	0.90457	0.90905	0.91377	0.91881
365	0.88961	0.89914	0.90338	0.90478	0.90926	0.91396	0.91899
366	0.88984	0.89937	0.90360	0.90500	0.90946	0.91415	0.91917
367	0.89008	0.89960	0.90382	0.90522	0.90966	0.91434	0.91935
368	0.89033	0.89981	0.90404	0.90542	0.90986	0.91453	0.91952
369	0.89058	0.90003	0.90424	0.90563	0.91005	0.91472	0.91970
370	0.89084	0.90026	0.90446	0.90584	0.91025	0.91490	0.91987
371	0.89107	0.90049	0.90467	0.90605	0.91045	0.91509	0.92005
372	0.89132	0.90070	0.90488	0.90626	0.91064	0.91527	0.92022
373	0.89157	0.90092	0.90508	0.90646	0.91084	0.91546	0.92039
374	0.89181	0.90114	0.90529	0.90667	0.91103	0.91564	0.92056
375	0.89205	0.90136	0.90550	0.90687	0.91123	0.91582	0.92073
376	0.89230	0.90157	0.90571	0.90707	0.91142	0.91600	0.92090
377	0.89255	0.90179	0.90592	0.90727	0.91162	0.91618	0.92107
378	0.89277	0.90200	0.90612	0.90748	0.91181	0.91636	0.92124
379	0.89301	0.90221	0.90632	0.90767	0.91200	0.91654	0.92141
380	0.89324	0.90243	0.90653	0.90788	0.91219	0.91672	0.92157
381	0.89347	0.90264	0.90673	0.90807	0.91238	0.91690	0.92174
382	0.89370	0.90286	0.90693	0.90827	0.91256	0.91708	0.92191
383	0.89392	0.90307	0.90713	0.90847	0.91275	0.91725	0.92207
384	0.89417	0.90327	0.90733	0.90867	0.91294	0.91743	0.92224
385	0.89441	0.90348	0.90753	0.90887	0.91312	0.91760	0.92240
386	0.89463	0.90369	0.90773	0.90906	0.91331	0.91778	0.92256
387	0.89485	0.90390	0.90793	0.90925	0.91349	0.91795	0.92272
388	0.89509	0.90411	0.90812	0.90945	0.91368	0.91812	0.92288
389	0.89533	0.90431	0.90832	0.90964	0.91386	0.91829	0.92304
390	0.89555	0.90450	0.90852	0.90983	0.91404	0.91846	0.92320
391	0.89576	0.90471	0.90871	0.91002	0.91422	0.91863	0.92336
392	0.89599	0.90491	0.90890	0.91021	0.91440	0.91880	0.92351
393	0.89620	0.90511	0.90909	0.91040	0.91458	0.91897	0.92367
394	0.89643	0.90532	0.90928	0.91059	0.91476	0.91914	0.92383
395	0.89668	0.90551	0.90947	0.91077	0.91493	0.91931	0.92398
396	0.89688	0.90571	0.90966	0.91096	0.91511	0.91947	0.92414
397	0.89708	0.90590	0.90984	0.91114	0.91528	0.91964	0.92429
398	0.89729	0.90610	0.91003	0.91133	0.91546	0.91980	0.92444
399	0.89752	0.90629	0.91022	0.91152	0.91563	0.91996	0.92460

N	0.1%	0.5%	1%	1.25%	2.5%	5%	10%
400	0.89773	0.90649	0.91041	0.91170	0.91580	0.92013	0.92475
401	0.89797	0.90668	0.91059	0.91188	0.91597	0.92029	0.92490
402	0.89817	0.90686	0.91078	0.91206	0.91615	0.92045	0.92505
403	0.89837	0.90706	0.91097	0.91225	0.91632	0.92061	0.92520
404	0.89859	0.90725	0.91115	0.91242	0.91649	0.92077	0.92535
405	0.89881	0.90744	0.91133	0.91260	0.91666	0.92093	0.92550
406	0.89901	0.90763	0.91151	0.91278	0.91683	0.92109	0.92565
407	0.89920	0.90783	0.91169	0.91296	0.91699	0.92125	0.92580
408	0.89942	0.90801	0.91187	0.91313	0.91716	0.92140	0.92595
409	0.89962	0.90820	0.91205	0.91331	0.91733	0.92156	0.92609
410	0.89984	0.90839	0.91223	0.91348	0.91749	0.92171	0.92624
411	0.90003	0.90857	0.91240	0.91365	0.91766	0.92187	0.92638
412	0.90023	0.90876	0.91257	0.91383	0.91782	0.92202	0.92653
413	0.90042	0.90894	0.91274	0.91400	0.91798	0.92218	0.92667
414	0.90064	0.90913	0.91292	0.91417	0.91814	0.92233	0.92681
415	0.90085	0.90931	0.91309	0.91434	0.91830	0.92248	0.92695
416	0.90103	0.90950	0.91327	0.91451	0.91847	0.92263	0.92710
417	0.90121	0.90969	0.91344	0.91468	0.91862	0.92279	0.92724
418	0.90144	0.90987	0.91361	0.91485	0.91878	0.92294	0.92738
419	0.90164	0.91003	0.91378	0.91502	0.91894	0.92309	0.92752
420	0.90184	0.91021	0.91395	0.91518	0.91910	0.92324	0.92766
421	0.90202	0.91040	0.91412	0.91535	0.91926	0.92339	0.92780
422	0.90223	0.91057	0.91429	0.91552	0.91942	0.92353	0.92794
423	0.90241	0.91074	0.91446	0.91568	0.91958	0.92368	0.92808
424	0.90261	0.91091	0.91462	0.91585	0.91973	0.92383	0.92822
425	0.90278	0.91109	0.91479	0.91601	0.91989	0.92397	0.92835
426	0.90299	0.91126	0.91496	0.91618	0.92004	0.92412	0.92849
427	0.90318	0.91143	0.91512	0.91633	0.92020	0.92426	0.92862
428	0.90336	0.91160	0.91529	0.91650	0.92035	0.92441	0.92876
429	0.90357	0.91178	0.91545	0.91666	0.92050	0.92455	0.92889
430	0.90375	0.91194	0.91561	0.91682	0.92065	0.92470	0.92903
431	0.90393	0.91211	0.91577	0.91698	0.92081	0.92484	0.92916
432	0.90415	0.91228	0.91594	0.91714	0.92095	0.92498	0.92929
433	0.90433	0.91245	0.91610	0.91730	0.92110	0.92512	0.92943
434	0.90451	0.91262	0.91625	0.91745	0.92125	0.92527	0.92956
435	0.90469	0.91278	0.91641	0.91762	0.92140	0.92541	0.92969
436	0.90486	0.91296	0.91657	0.91777	0.92155	0.92555	0.92982
437	0.90505	0.91312	0.91673	0.91793	0.92170	0.92569	0.92995
438	0.90525	0.91327	0.91689	0.91808	0.92185	0.92582	0.93008
439	0.90542	0.91343	0.91705	0.91824	0.92199	0.92596	0.93021
440	0.90559	0.91359	0.91720	0.91839	0.92214	0.92610	0.93034
441	0.90578	0.91376	0.91736	0.91854	0.92228	0.92624	0.93046
442	0.90594	0.91392	0.91752	0.91870	0.92243	0.92637	0.93059
443	0.90611	0.91408	0.91767	0.91885	0.92257	0.92651	0.93072
444	0.90628	0.91424	0.91783	0.91900	0.92272	0.92665	0.93085
445	0.90646	0.91441	0.91798	0.91916	0.92286	0.92678	0.93097
446	0.90663	0.91456	0.91814	0.91931	0.92301	0.92692	0.93110
447	0.90682	0.91472	0.91829	0.91946	0.92315	0.92705	0.93122
448	0.90700	0.91489	0.91844	0.91960	0.92329	0.92718	0.93135
449	0.90718	0.91505	0.91859	0.91975	0.92343	0.92731	0.93147
450	0.90736	0.91520	0.91874	0.91990	0.92357	0.92745	0.93160
451	0.90752	0.91536	0.91889	0.92005	0.92371	0.92758	0.93172
452	0.90770	0.91552	0.91904	0.92020	0.92385	0.92771	0.93184
453	0.90786	0.91567	0.91919	0.92034	0.92399	0.92784	0.93197
454	0.90805	0.91583	0.91934	0.92048	0.92413	0.92797	0.93209
455	0.90824	0.91598	0.91948	0.92063	0.92426	0.92810	0.93221
456	0.90840	0.91613	0.91964	0.92077	0.92440	0.92823	0.93233
457	0.90858	0.91629	0.91978	0.92092	0.92454	0.92836	0.93245
458	0.90874	0.91644	0.91993	0.92106	0.92467	0.92849	0.93257
459	0.90892	0.91660	0.92007	0.92120	0.92481	0.92861	0.93269
460	0.90909	0.91674	0.92022	0.92134	0.92494	0.92874	0.93281
461	0.90926	0.91690	0.92036	0.92149	0.92508	0.92887	0.93293
462	0.90941	0.91705	0.92050	0.92163	0.92521	0.92900	0.93304
463	0.90957	0.91719	0.92065	0.92177	0.92535	0.92913	0.93316
464	0.90974	0.91734	0.92079	0.92190	0.92548	0.92925	0.93328
465	0.90990	0.91749	0.92093	0.92204	0.92561	0.92937	0.93340
466	0.91007	0.91765	0.92107	0.92219	0.92574	0.92950	0.93351
467	0.91023	0.91780	0.92121	0.92233	0.92587	0.92962	0.93363
468	0.91038	0.91793	0.92135	0.92246	0.92600	0.92974	0.93374
469	0.91053	0.91809	0.92149	0.92260	0.92613	0.92986	0.93386
470	0.91071	0.91823	0.92163	0.92274	0.92626	0.92999	0.93397
471	0.91086	0.91837	0.92178	0.92288	0.92639	0.93011	0.93409
472	0.91102	0.91852	0.92191	0.92301	0.92652	0.93023	0.93420
473	0.91116	0.91867	0.92205	0.92315	0.92665	0.93035	0.93431

N	0.1%	0.5%	1%	1.25%	2.5%	5%	10%
474	0.91132	0.91881	0.92218	0.92328	0.92678	0.93047	0.93443
475	0.91147	0.91895	0.92232	0.92342	0.92690	0.93059	0.93454
476	0.91163	0.91910	0.92246	0.92355	0.92703	0.93071	0.93465
477	0.91180	0.91924	0.92260	0.92369	0.92715	0.93083	0.93476
478	0.91195	0.91938	0.92273	0.92382	0.92728	0.93095	0.93487
479	0.91208	0.91952	0.92286	0.92395	0.92741	0.93107	0.93499
480	0.91225	0.91967	0.92300	0.92408	0.92753	0.93119	0.93510
481	0.91241	0.91981	0.92313	0.92421	0.92766	0.93130	0.93521
482	0.91257	0.91994	0.92326	0.92434	0.92778	0.93142	0.93532
483	0.91272	0.92008	0.92339	0.92447	0.92791	0.93154	0.93543
484	0.91288	0.92022	0.92352	0.92460	0.92803	0.93166	0.93554
485	0.91302	0.92035	0.92365	0.92472	0.92815	0.93177	0.93564
486	0.91319	0.92049	0.92378	0.92485	0.92828	0.93189	0.93575
487	0.91333	0.92063	0.92392	0.92498	0.92839	0.93200	0.93586
488	0.91349	0.92076	0.92405	0.92511	0.92852	0.93212	0.93597
489	0.91363	0.92090	0.92417	0.92523	0.92864	0.93223	0.93607
490	0.91377	0.92103	0.92430	0.92536	0.92876	0.93234	0.93618
491	0.91392	0.92117	0.92443	0.92549	0.92888	0.93245	0.93628
492	0.91408	0.92130	0.92456	0.92561	0.92900	0.93257	0.93639
493	0.91423	0.92144	0.92468	0.92574	0.92912	0.93268	0.93650
494	0.91439	0.92157	0.92481	0.92586	0.92923	0.93279	0.93660
495	0.91453	0.92172	0.92494	0.92599	0.92936	0.93290	0.93671
496	0.91468	0.92184	0.92506	0.92611	0.92947	0.93301	0.93681
497	0.91483	0.92198	0.92519	0.92624	0.92959	0.93313	0.93691
498	0.91497	0.92211	0.92531	0.92636	0.92971	0.93324	0.93702
499	0.91512	0.92224	0.92544	0.92648	0.92983	0.93335	0.93712
500	0.91525	0.92237	0.92556	0.92661	0.92994	0.93346	0.93722
501	0.91540	0.92250	0.92569	0.92673	0.93006	0.93357	0.93732
502	0.91553	0.92263	0.92581	0.92685	0.93017	0.93367	0.93743
503	0.91567	0.92276	0.92594	0.92697	0.93029	0.93378	0.93753
504	0.91581	0.92289	0.92606	0.92710	0.93040	0.93389	0.93763
505	0.91595	0.92302	0.92618	0.92721	0.93052	0.93400	0.93773
506	0.91608	0.92315	0.92630	0.92734	0.93063	0.93411	0.93783
507	0.91622	0.92328	0.92642	0.92746	0.93075	0.93421	0.93793
508	0.91637	0.92341	0.92655	0.92758	0.93086	0.93432	0.93803
509	0.91651	0.92353	0.92667	0.92770	0.93097	0.93443	0.93813
510	0.91666	0.92366	0.92679	0.92781	0.93108	0.93453	0.93823
511	0.91679	0.92378	0.92690	0.92793	0.93119	0.93464	0.93833
512	0.91694	0.92391	0.92702	0.92804	0.93131	0.93474	0.93843
513	0.91707	0.92403	0.92714	0.92816	0.93142	0.93485	0.93852
514	0.91720	0.92415	0.92726	0.92828	0.93153	0.93495	0.93862
515	0.91735	0.92428	0.92738	0.92840	0.93164	0.93506	0.93872
516	0.91748	0.92440	0.92750	0.92851	0.93175	0.93516	0.93881
517	0.91760	0.92452	0.92761	0.92863	0.93186	0.93526	0.93891
518	0.91774	0.92463	0.92773	0.92874	0.93197	0.93537	0.93901
519	0.91788	0.92476	0.92784	0.92885	0.93208	0.93547	0.93910
520	0.91801	0.92488	0.92796	0.92897	0.93219	0.93557	0.93920
521	0.91814	0.92501	0.92808	0.92908	0.93230	0.93567	0.93930
522	0.91827	0.92513	0.92819	0.92920	0.93240	0.93577	0.93939
523	0.91840	0.92525	0.92831	0.92932	0.93251	0.93588	0.93949
524	0.91853	0.92537	0.92842	0.92943	0.93262	0.93598	0.93958
525	0.91865	0.92549	0.92853	0.92954	0.93273	0.93608	0.93967
526	0.91877	0.92561	0.92865	0.92965	0.93283	0.93618	0.93977
527	0.91890	0.92573	0.92876	0.92976	0.93294	0.93628	0.93986
528	0.91903	0.92584	0.92887	0.92987	0.93304	0.93638	0.93995
529	0.91916	0.92596	0.92898	0.92998	0.93315	0.93648	0.94005
530	0.91929	0.92608	0.92910	0.93009	0.93325	0.93658	0.94014
531	0.91941	0.92619	0.92921	0.93020	0.93336	0.93667	0.94023
532	0.91955	0.92631	0.92932	0.93031	0.93346	0.93677	0.94033
533	0.91968	0.92643	0.92943	0.93042	0.93356	0.93687	0.94042
534	0.91980	0.92654	0.92954	0.93053	0.93367	0.93697	0.94051
535	0.91992	0.92666	0.92965	0.93064	0.93377	0.93706	0.94060
536	0.92005	0.92677	0.92976	0.93075	0.93387	0.93716	0.94069
537	0.92019	0.92689	0.92987	0.93085	0.93397	0.93726	0.94078
538	0.92032	0.92700	0.92998	0.93096	0.93407	0.93735	0.94087
539	0.92044	0.92711	0.93009	0.93107	0.93417	0.93745	0.94096
540	0.92057	0.92722	0.93020	0.93117	0.93428	0.93754	0.94105
541	0.92070	0.92734	0.93031	0.93128	0.93438	0.93764	0.94114
542	0.92080	0.92745	0.93042	0.93139	0.93448	0.93774	0.94123
543	0.92094	0.92757	0.93053	0.93149	0.93458	0.93783	0.94131
544	0.92107	0.92768	0.93064	0.93159	0.93468	0.93793	0.94140
545	0.92118	0.92778	0.93074	0.93170	0.93478	0.93802	0.94149
546	0.92131	0.92789	0.93085	0.93180	0.93488	0.93811	0.94158
547	0.92143	0.92800	0.93095	0.93191	0.93498	0.93820	0.94167

N	0.1%	0.5%	1%	1.25%	2.5%	5%	10%
548	0.92156	0.92811	0.93106	0.93202	0.93508	0.93830	0.94175
549	0.92167	0.92823	0.93116	0.93212	0.93518	0.93839	0.94184
550	0.92181	0.92834	0.93127	0.93222	0.93528	0.93848	0.94193
551	0.92194	0.92845	0.93137	0.93232	0.93537	0.93858	0.94201
552	0.92206	0.92856	0.93148	0.93243	0.93547	0.93867	0.94210
553	0.92218	0.92867	0.93158	0.93253	0.93557	0.93876	0.94219
554	0.92231	0.92878	0.93168	0.93264	0.93566	0.93886	0.94227
555	0.92242	0.92888	0.93178	0.93274	0.93576	0.93895	0.94236
556	0.92254	0.92899	0.93188	0.93284	0.93586	0.93904	0.94244
557	0.92266	0.92910	0.93199	0.93294	0.93595	0.93913	0.94253
558	0.92278	0.92920	0.93209	0.93304	0.93605	0.93922	0.94261
559	0.92292	0.92932	0.93219	0.93314	0.93614	0.93931	0.94270
560	0.92303	0.92943	0.93230	0.93324	0.93624	0.93940	0.94278
561	0.92314	0.92953	0.93240	0.93334	0.93634	0.93948	0.94287
562	0.92325	0.92963	0.93249	0.93344	0.93643	0.93957	0.94295
563	0.92337	0.92974	0.93259	0.93354	0.93652	0.93966	0.94303
564	0.92349	0.92984	0.93270	0.93363	0.93662	0.93975	0.94311
565	0.92359	0.92995	0.93280	0.93373	0.93671	0.93984	0.94320
566	0.92371	0.93005	0.93289	0.93383	0.93680	0.93993	0.94328
567	0.92383	0.93015	0.93299	0.93393	0.93690	0.94002	0.94336
568	0.92394	0.93025	0.93309	0.93402	0.93699	0.94010	0.94344
569	0.92405	0.93036	0.93319	0.93412	0.93708	0.94019	0.94352
570	0.92415	0.93046	0.93329	0.93421	0.93717	0.94028	0.94361
571	0.92428	0.93056	0.93339	0.93431	0.93726	0.94036	0.94369
572	0.92439	0.93066	0.93349	0.93441	0.93736	0.94045	0.94377
573	0.92449	0.93077	0.93358	0.93450	0.93745	0.94054	0.94385
574	0.92461	0.93086	0.93368	0.93460	0.93754	0.94062	0.94393
575	0.92472	0.93096	0.93378	0.93470	0.93763	0.94071	0.94401
576	0.92483	0.93107	0.93388	0.93479	0.93772	0.94079	0.94409
577	0.92494	0.93116	0.93398	0.93489	0.93781	0.94088	0.94417
578	0.92505	0.93127	0.93407	0.93498	0.93790	0.94096	0.94425
579	0.92517	0.93137	0.93416	0.93507	0.93799	0.94105	0.94433
580	0.92528	0.93148	0.93426	0.93517	0.93808	0.94113	0.94441
581	0.92539	0.93157	0.93436	0.93526	0.93817	0.94122	0.94449
582	0.92549	0.93167	0.93445	0.93535	0.93825	0.94130	0.94456
583	0.92559	0.93177	0.93455	0.93545	0.93834	0.94138	0.94464
584	0.92571	0.93187	0.93464	0.93554	0.93843	0.94146	0.94472
585	0.92582	0.93197	0.93474	0.93564	0.93852	0.94155	0.94480
586	0.92591	0.93207	0.93483	0.93573	0.93860	0.94163	0.94487
587	0.92602	0.93217	0.93492	0.93582	0.93869	0.94171	0.94495
588	0.92613	0.93227	0.93502	0.93591	0.93878	0.94179	0.94503
589	0.92623	0.93236	0.93511	0.93600	0.93887	0.94187	0.94511
590	0.92634	0.93246	0.93520	0.93609	0.93895	0.94196	0.94518
591	0.92644	0.93255	0.93529	0.93618	0.93904	0.94204	0.94526
592	0.92655	0.93265	0.93538	0.93628	0.93912	0.94212	0.94533
593	0.92666	0.93275	0.93547	0.93636	0.93921	0.94220	0.94541
594	0.92677	0.93284	0.93556	0.93646	0.93930	0.94228	0.94548
595	0.92688	0.93293	0.93565	0.93655	0.93938	0.94236	0.94556
596	0.92698	0.93303	0.93574	0.93663	0.93947	0.94244	0.94563
597	0.92708	0.93313	0.93583	0.93672	0.93955	0.94252	0.94571
598	0.92717	0.93322	0.93592	0.93681	0.93963	0.94260	0.94579
599	0.92727	0.93332	0.93601	0.93690	0.93972	0.94268	0.94586
600	0.92737	0.93341	0.93610	0.93699	0.93980	0.94276	0.94593
601	0.92747	0.93350	0.93619	0.93708	0.93988	0.94284	0.94601
602	0.92757	0.93360	0.93628	0.93716	0.93997	0.94291	0.94608
603	0.92767	0.93369	0.93637	0.93725	0.94005	0.94299	0.94615
604	0.92777	0.93379	0.93646	0.93733	0.94013	0.94307	0.94623
605	0.92788	0.93388	0.93655	0.93742	0.94022	0.94315	0.94630
606	0.92799	0.93397	0.93664	0.93751	0.94030	0.94323	0.94637
607	0.92811	0.93406	0.93672	0.93760	0.94038	0.94331	0.94645
608	0.92820	0.93415	0.93681	0.93768	0.94046	0.94338	0.94652
609	0.92830	0.93425	0.93690	0.93777	0.94055	0.94346	0.94659
610	0.92840	0.93434	0.93698	0.93785	0.94063	0.94354	0.94666
611	0.92851	0.93443	0.93707	0.93794	0.94071	0.94361	0.94674
612	0.92861	0.93451	0.93715	0.93803	0.94079	0.94369	0.94681
613	0.92869	0.93461	0.93724	0.93811	0.94087	0.94377	0.94688
614	0.92878	0.93469	0.93733	0.93819	0.94095	0.94384	0.94695
615	0.92887	0.93478	0.93741	0.93828	0.94103	0.94392	0.94702
616	0.92898	0.93487	0.93750	0.93837	0.94111	0.94400	0.94709
617	0.92908	0.93496	0.93758	0.93845	0.94119	0.94407	0.94716
618	0.92918	0.93505	0.93767	0.93853	0.94127	0.94415	0.94723
619	0.92928	0.93513	0.93775	0.93861	0.94134	0.94422	0.94731
620	0.92937	0.93522	0.93784	0.93869	0.94143	0.94430	0.94737
621	0.92947	0.93531	0.93793	0.93878	0.94151	0.94437	0.94744

N	0.1%	0.5%	1%	1.25%	2.5%	5%	10%
622	0.92956	0.93540	0.93801	0.93886	0.94159	0.94445	0.94751
623	0.92965	0.93548	0.93809	0.93894	0.94166	0.94452	0.94758
624	0.92974	0.93558	0.93817	0.93903	0.94174	0.94459	0.94765
625	0.92984	0.93567	0.93825	0.93911	0.94182	0.94467	0.94772
626	0.92993	0.93575	0.93834	0.93919	0.94190	0.94474	0.94779
627	0.93003	0.93584	0.93842	0.93927	0.94197	0.94481	0.94786
628	0.93012	0.93592	0.93850	0.93935	0.94205	0.94489	0.94793
629	0.93021	0.93601	0.93859	0.93943	0.94213	0.94496	0.94800
630	0.93031	0.93610	0.93867	0.93951	0.94220	0.94504	0.94806
631	0.93041	0.93618	0.93875	0.93959	0.94228	0.94511	0.94813
632	0.93050	0.93626	0.93883	0.93967	0.94236	0.94518	0.94820
633	0.93059	0.93635	0.93891	0.93975	0.94244	0.94525	0.94827
634	0.93069	0.93643	0.93899	0.93983	0.94251	0.94532	0.94833
635	0.93078	0.93652	0.93908	0.93991	0.94258	0.94539	0.94840
636	0.93088	0.93660	0.93916	0.93999	0.94266	0.94547	0.94847
637	0.93098	0.93669	0.93923	0.94007	0.94273	0.94554	0.94853
638	0.93108	0.93677	0.93931	0.94015	0.94281	0.94561	0.94860
639	0.93117	0.93685	0.93939	0.94023	0.94288	0.94568	0.94867
640	0.93125	0.93693	0.93947	0.94031	0.94296	0.94575	0.94873
641	0.93135	0.93701	0.93955	0.94039	0.94304	0.94582	0.94880
642	0.93143	0.93710	0.93964	0.94046	0.94311	0.94589	0.94886
643	0.93152	0.93718	0.93971	0.94054	0.94318	0.94596	0.94893
644	0.93161	0.93726	0.93979	0.94062	0.94326	0.94603	0.94899
645	0.93170	0.93735	0.93987	0.94069	0.94333	0.94610	0.94906
646	0.93178	0.93743	0.93995	0.94077	0.94340	0.94617	0.94912
647	0.93187	0.93751	0.94002	0.94085	0.94348	0.94624	0.94919
648	0.93197	0.93759	0.94010	0.94092	0.94355	0.94631	0.94925
649	0.93205	0.93767	0.94018	0.94100	0.94362	0.94637	0.94932
650	0.93214	0.93776	0.94026	0.94108	0.94369	0.94644	0.94938
651	0.93222	0.93784	0.94034	0.94115	0.94376	0.94651	0.94945
652	0.93232	0.93792	0.94041	0.94123	0.94384	0.94658	0.94951
653	0.93240	0.93801	0.94049	0.94131	0.94391	0.94665	0.94957
654	0.93249	0.93808	0.94057	0.94139	0.94398	0.94672	0.94964
655	0.93258	0.93816	0.94065	0.94146	0.94406	0.94679	0.94970
656	0.93267	0.93824	0.94072	0.94154	0.94413	0.94685	0.94977
657	0.93275	0.93832	0.94079	0.94161	0.94420	0.94692	0.94983
658	0.93284	0.93840	0.94087	0.94169	0.94427	0.94699	0.94989
659	0.93293	0.93847	0.94095	0.94176	0.94434	0.94705	0.94995
660	0.93302	0.93855	0.94102	0.94184	0.94441	0.94712	0.95002
661	0.93310	0.93863	0.94110	0.94191	0.94448	0.94719	0.95008
662	0.93320	0.93870	0.94118	0.94198	0.94455	0.94725	0.95014
663	0.93329	0.93878	0.94125	0.94206	0.94462	0.94732	0.95020
664	0.93338	0.93886	0.94132	0.94213	0.94469	0.94738	0.95027
665	0.93346	0.93894	0.94140	0.94220	0.94476	0.94745	0.95033
666	0.93355	0.93902	0.94147	0.94227	0.94483	0.94752	0.95039
667	0.93364	0.93910	0.94155	0.94235	0.94490	0.94758	0.95045
668	0.93372	0.93918	0.94162	0.94242	0.94497	0.94765	0.95051
669	0.93380	0.93925	0.94169	0.94249	0.94504	0.94771	0.95057
670	0.93388	0.93933	0.94177	0.94256	0.94511	0.94778	0.95063
671	0.93397	0.93941	0.94184	0.94264	0.94518	0.94784	0.95069
672	0.93405	0.93949	0.94191	0.94271	0.94524	0.94791	0.95076
673	0.93414	0.93956	0.94199	0.94278	0.94531	0.94797	0.95082
674	0.93422	0.93964	0.94206	0.94285	0.94538	0.94803	0.95088
675	0.93430	0.93971	0.94213	0.94292	0.94545	0.94810	0.95094
676	0.93437	0.93979	0.94220	0.94300	0.94552	0.94816	0.95100
677	0.93446	0.93987	0.94227	0.94307	0.94558	0.94823	0.95106
678	0.93454	0.93994	0.94235	0.94314	0.94565	0.94829	0.95112
679	0.93463	0.94002	0.94242	0.94321	0.94572	0.94835	0.95117
680	0.93471	0.94010	0.94249	0.94328	0.94579	0.94842	0.95123
681	0.93479	0.94017	0.94256	0.94335	0.94585	0.94848	0.95129
682	0.93488	0.94024	0.94263	0.94342	0.94592	0.94854	0.95135
683	0.93497	0.94032	0.94271	0.94349	0.94598	0.94860	0.95141
684	0.93505	0.94040	0.94278	0.94356	0.94605	0.94867	0.95147
685	0.93513	0.94047	0.94285	0.94363	0.94611	0.94873	0.95153
686	0.93521	0.94055	0.94292	0.94370	0.94618	0.94879	0.95158
687	0.93528	0.94062	0.94299	0.94377	0.94625	0.94885	0.95164
688	0.93537	0.94070	0.94306	0.94384	0.94631	0.94892	0.95170
689	0.93546	0.94077	0.94313	0.94391	0.94638	0.94898	0.95176
690	0.93553	0.94084	0.94320	0.94398	0.94644	0.94904	0.95182
691	0.93561	0.94091	0.94327	0.94404	0.94651	0.94910	0.95188
692	0.93570	0.94099	0.94334	0.94411	0.94657	0.94916	0.95193
693	0.93578	0.94106	0.94341	0.94418	0.94664	0.94922	0.95199
694	0.93585	0.94113	0.94347	0.94425	0.94670	0.94928	0.95205
695	0.93593	0.94120	0.94354	0.94431	0.94677	0.94934	0.95211

N	0.1%	0.5%	1%	1.25%	2.5%	5%	10%
696	0.93600	0.94127	0.94361	0.94438	0.94683	0.94941	0.95216
697	0.93608	0.94135	0.94368	0.94445	0.94689	0.94947	0.95222
698	0.93617	0.94142	0.94375	0.94451	0.94696	0.94953	0.95228
699	0.93624	0.94149	0.94382	0.94458	0.94702	0.94959	0.95233
700	0.93632	0.94156	0.94388	0.94465	0.94709	0.94964	0.95239
701	0.93638	0.94163	0.94395	0.94472	0.94715	0.94971	0.95244
702	0.93647	0.94170	0.94401	0.94479	0.94721	0.94977	0.95250
703	0.93655	0.94177	0.94408	0.94485	0.94727	0.94982	0.95256
704	0.93663	0.94184	0.94415	0.94492	0.94734	0.94988	0.95261
705	0.93670	0.94191	0.94421	0.94498	0.94740	0.94994	0.95267
706	0.93677	0.94198	0.94428	0.94505	0.94746	0.95000	0.95272
707	0.93685	0.94205	0.94435	0.94511	0.94752	0.95006	0.95278
708	0.93693	0.94212	0.94441	0.94518	0.94759	0.95012	0.95283
709	0.93701	0.94219	0.94448	0.94524	0.94765	0.95018	0.95289
710	0.93709	0.94226	0.94455	0.94531	0.94771	0.95024	0.95294
711	0.93717	0.94232	0.94461	0.94537	0.94777	0.95030	0.95300
712	0.93723	0.94239	0.94468	0.94544	0.94783	0.95035	0.95305
713	0.93732	0.94246	0.94474	0.94550	0.94790	0.95041	0.95311
714	0.93740	0.94253	0.94481	0.94557	0.94796	0.95047	0.95316
715	0.93747	0.94260	0.94487	0.94563	0.94802	0.95053	0.95322
716	0.93754	0.94267	0.94494	0.94569	0.94808	0.95058	0.95327
717	0.93762	0.94274	0.94501	0.94576	0.94814	0.95064	0.95332
718	0.93769	0.94281	0.94507	0.94583	0.94820	0.95070	0.95338
719	0.93777	0.94287	0.94514	0.94589	0.94826	0.95076	0.95343
720	0.93783	0.94294	0.94520	0.94595	0.94832	0.95081	0.95348
721	0.93791	0.94301	0.94526	0.94601	0.94838	0.95087	0.95354
722	0.93799	0.94307	0.94533	0.94608	0.94844	0.95093	0.95359
723	0.93806	0.94314	0.94539	0.94614	0.94850	0.95098	0.95364
724	0.93814	0.94320	0.94546	0.94620	0.94856	0.95104	0.95370
725	0.93820	0.94327	0.94552	0.94626	0.94862	0.95110	0.95375
726	0.93828	0.94334	0.94559	0.94633	0.94868	0.95115	0.95380
727	0.93835	0.94341	0.94565	0.94639	0.94874	0.95121	0.95385
728	0.93842	0.94347	0.94571	0.94645	0.94880	0.95126	0.95391
729	0.93850	0.94354	0.94577	0.94651	0.94886	0.95132	0.95396
730	0.93857	0.94361	0.94584	0.94657	0.94892	0.95137	0.95401
731	0.93864	0.94367	0.94590	0.94663	0.94898	0.95143	0.95406
732	0.93871	0.94374	0.94596	0.94670	0.94904	0.95149	0.95412
733	0.93878	0.94381	0.94602	0.94676	0.94910	0.95154	0.95417
734	0.93886	0.94387	0.94608	0.94682	0.94915	0.95160	0.95422
735	0.93893	0.94393	0.94615	0.94688	0.94921	0.95165	0.95427
736	0.93899	0.94400	0.94621	0.94694	0.94927	0.95171	0.95432
737	0.93907	0.94406	0.94627	0.94700	0.94933	0.95176	0.95438
738	0.93913	0.94412	0.94633	0.94706	0.94938	0.95182	0.95443
739	0.93921	0.94419	0.94640	0.94712	0.94944	0.95187	0.95448
740	0.93929	0.94425	0.94646	0.94719	0.94950	0.95193	0.95453
741	0.93935	0.94432	0.94652	0.94725	0.94956	0.95198	0.95458
742	0.93941	0.94438	0.94658	0.94731	0.94961	0.95203	0.95463
743	0.93948	0.94445	0.94664	0.94737	0.94967	0.95209	0.95468
744	0.93956	0.94451	0.94670	0.94742	0.94972	0.95214	0.95473
745	0.93964	0.94457	0.94676	0.94748	0.94978	0.95220	0.95478
746	0.93970	0.94464	0.94682	0.94754	0.94984	0.95225	0.95483
747	0.93978	0.94470	0.94688	0.94761	0.94990	0.95230	0.95488
748	0.93985	0.94476	0.94694	0.94767	0.94995	0.95236	0.95493
749	0.93993	0.94482	0.94700	0.94772	0.95001	0.95241	0.95498
750	0.94000	0.94488	0.94706	0.94778	0.95007	0.95246	0.95503
751	0.94007	0.94495	0.94712	0.94784	0.95012	0.95252	0.95508
752	0.94012	0.94501	0.94718	0.94790	0.95018	0.95257	0.95513
753	0.94020	0.94507	0.94724	0.94796	0.95023	0.95262	0.95518
754	0.94026	0.94513	0.94730	0.94802	0.95029	0.95268	0.95523
755	0.94034	0.94520	0.94736	0.94807	0.95034	0.95273	0.95528
756	0.94041	0.94526	0.94742	0.94813	0.95040	0.95278	0.95533
757	0.94048	0.94532	0.94748	0.94819	0.95046	0.95283	0.95538
758	0.94055	0.94539	0.94754	0.94825	0.95051	0.95288	0.95543
759	0.94062	0.94545	0.94760	0.94831	0.95057	0.95294	0.95548
760	0.94068	0.94551	0.94765	0.94836	0.95062	0.95299	0.95553
761	0.94075	0.94557	0.94771	0.94842	0.95068	0.95304	0.95557
762	0.94081	0.94563	0.94777	0.94848	0.95073	0.95309	0.95562
763	0.94087	0.94569	0.94782	0.94853	0.95079	0.95314	0.95567
764	0.94094	0.94575	0.94788	0.94859	0.95084	0.95319	0.95572
765	0.94100	0.94581	0.94794	0.94865	0.95089	0.95325	0.95577
766	0.94107	0.94587	0.94800	0.94870	0.95095	0.95330	0.95581
767	0.94114	0.94593	0.94806	0.94876	0.95100	0.95335	0.95586
768	0.94121	0.94599	0.94811	0.94882	0.95105	0.95340	0.95591
769	0.94128	0.94605	0.94817	0.94887	0.95111	0.95345	0.95596

N	0.1%	0.5%	1%	1.25%	2.5%	5%	10%
770	0.94134	0.94611	0.94823	0.94893	0.95116	0.95350	0.95600
771	0.94142	0.94617	0.94828	0.94899	0.95122	0.95355	0.95605
772	0.94148	0.94623	0.94834	0.94904	0.95127	0.95360	0.95610
773	0.94154	0.94629	0.94839	0.94910	0.95132	0.95365	0.95614
774	0.94161	0.94635	0.94845	0.94915	0.95137	0.95370	0.95619
775	0.94168	0.94641	0.94851	0.94921	0.95143	0.95375	0.95624
776	0.94174	0.94646	0.94856	0.94926	0.95148	0.95380	0.95629
777	0.94180	0.94652	0.94862	0.94932	0.95153	0.95385	0.95633
778	0.94186	0.94658	0.94868	0.94937	0.95159	0.95390	0.95638
779	0.94192	0.94664	0.94873	0.94943	0.95164	0.95395	0.95643
780	0.94199	0.94670	0.94879	0.94948	0.95169	0.95400	0.95647
781	0.94205	0.94676	0.94884	0.94954	0.95174	0.95405	0.95652
782	0.94213	0.94682	0.94890	0.94959	0.95179	0.95410	0.95657
783	0.94219	0.94687	0.94895	0.94965	0.95185	0.95414	0.95661
784	0.94225	0.94693	0.94901	0.94970	0.95190	0.95419	0.95666
785	0.94231	0.94699	0.94906	0.94976	0.95195	0.95424	0.95670
786	0.94237	0.94705	0.94912	0.94981	0.95200	0.95429	0.95675
787	0.94244	0.94710	0.94918	0.94986	0.95205	0.95434	0.95679
788	0.94251	0.94716	0.94923	0.94992	0.95210	0.95439	0.95684
789	0.94257	0.94722	0.94929	0.94997	0.95215	0.95444	0.95689
790	0.94264	0.94727	0.94934	0.95002	0.95221	0.95449	0.95693
791	0.94270	0.94733	0.94940	0.95008	0.95226	0.95453	0.95698
792	0.94275	0.94739	0.94945	0.95013	0.95231	0.95458	0.95702
793	0.94282	0.94745	0.94951	0.95018	0.95236	0.95463	0.95707
794	0.94288	0.94750	0.94956	0.95024	0.95241	0.95468	0.95711
795	0.94295	0.94756	0.94961	0.95029	0.95246	0.95473	0.95716
796	0.94301	0.94762	0.94967	0.95034	0.95251	0.95477	0.95720
797	0.94307	0.94767	0.94972	0.95040	0.95256	0.95482	0.95725
798	0.94313	0.94773	0.94977	0.95045	0.95261	0.95487	0.95729
799	0.94318	0.94778	0.94983	0.95050	0.95266	0.95492	0.95734
800	0.94325	0.94784	0.94988	0.95055	0.95271	0.95496	0.95738
801	0.94330	0.94789	0.94993	0.95060	0.95276	0.95501	0.95742
802	0.94337	0.94795	0.94999	0.95066	0.95281	0.95506	0.95747
803	0.94342	0.94800	0.95004	0.95071	0.95286	0.95510	0.95751
804	0.94348	0.94806	0.95009	0.95076	0.95291	0.95515	0.95756
805	0.94354	0.94811	0.95015	0.95081	0.95296	0.95520	0.95760
806	0.94360	0.94817	0.95020	0.95086	0.95301	0.95525	0.95764
807	0.94366	0.94822	0.95025	0.95092	0.95306	0.95529	0.95769
808	0.94372	0.94828	0.95030	0.95097	0.95310	0.95534	0.95773
809	0.94379	0.94833	0.95036	0.95102	0.95315	0.95538	0.95777
810	0.94385	0.94839	0.95041	0.95107	0.95320	0.95543	0.95782
811	0.94391	0.94845	0.95046	0.95112	0.95325	0.95548	0.95786
812	0.94397	0.94850	0.95051	0.95117	0.95330	0.95552	0.95790
813	0.94403	0.94855	0.95056	0.95123	0.95335	0.95557	0.95795
814	0.94409	0.94861	0.95061	0.95128	0.95340	0.95561	0.95799
815	0.94415	0.94866	0.95067	0.95133	0.95344	0.95566	0.95803
816	0.94422	0.94871	0.95072	0.95138	0.95349	0.95571	0.95807
817	0.94428	0.94877	0.95077	0.95143	0.95354	0.95575	0.95812
818	0.94434	0.94882	0.95082	0.95148	0.95359	0.95580	0.95816
819	0.94440	0.94887	0.95087	0.95153	0.95363	0.95584	0.95820
820	0.94446	0.94893	0.95092	0.95158	0.95368	0.95588	0.95825
821	0.94452	0.94898	0.95097	0.95163	0.95373	0.95593	0.95829
822	0.94458	0.94903	0.95102	0.95168	0.95378	0.95597	0.95833
823	0.94464	0.94909	0.95107	0.95172	0.95382	0.95602	0.95837
824	0.94469	0.94914	0.95112	0.95178	0.95387	0.95606	0.95841
825	0.94475	0.94919	0.95117	0.95183	0.95392	0.95611	0.95846
826	0.94481	0.94924	0.95122	0.95188	0.95397	0.95615	0.95850
827	0.94487	0.94930	0.95128	0.95193	0.95401	0.95620	0.95854
828	0.94492	0.94935	0.95132	0.95197	0.95406	0.95624	0.95858
829	0.94499	0.94940	0.95137	0.95202	0.95411	0.95629	0.95862
830	0.94504	0.94945	0.95142	0.95207	0.95415	0.95633	0.95866
831	0.94509	0.94950	0.95147	0.95212	0.95420	0.95638	0.95870
832	0.94515	0.94955	0.95152	0.95217	0.95425	0.95642	0.95875
833	0.94521	0.94960	0.95157	0.95222	0.95429	0.95646	0.95879
834	0.94527	0.94966	0.95163	0.95227	0.95434	0.95651	0.95883
835	0.94532	0.94971	0.95168	0.95232	0.95438	0.95655	0.95887
836	0.94538	0.94976	0.95173	0.95237	0.95443	0.95660	0.95891
837	0.94543	0.94981	0.95178	0.95242	0.95447	0.95664	0.95895
838	0.94549	0.94986	0.95183	0.95246	0.95452	0.95668	0.95899
839	0.94555	0.94991	0.95188	0.95251	0.95457	0.95673	0.95903
840	0.94561	0.94996	0.95192	0.95256	0.95461	0.95677	0.95907
841	0.94566	0.95001	0.95197	0.95261	0.95466	0.95681	0.95911
842	0.94572	0.95006	0.95202	0.95266	0.95470	0.95685	0.95915
843	0.94577	0.95011	0.95207	0.95270	0.95475	0.95690	0.95920

N	0.1%	0.5%	1%	1.25%	2.5%	5%	10%
844	0.94583	0.95016	0.95212	0.95275	0.95479	0.95694	0.95924
845	0.94588	0.95021	0.95216	0.95280	0.95484	0.95698	0.95928
846	0.94594	0.95027	0.95221	0.95285	0.95488	0.95703	0.95932
847	0.94599	0.95032	0.95226	0.95289	0.95493	0.95707	0.95935
848	0.94604	0.95037	0.95231	0.95294	0.95497	0.95711	0.95940
849	0.94610	0.95042	0.95236	0.95299	0.95502	0.95715	0.95944
850	0.94615	0.95047	0.95241	0.95304	0.95506	0.95719	0.95948
851	0.94621	0.95051	0.95245	0.95308	0.95510	0.95724	0.95952
852	0.94626	0.95057	0.95250	0.95313	0.95515	0.95728	0.95956
853	0.94631	0.95062	0.95255	0.95318	0.95520	0.95732	0.95959
854	0.94637	0.95066	0.95260	0.95322	0.95524	0.95736	0.95963
855	0.94642	0.95071	0.95264	0.95327	0.95528	0.95741	0.95967
856	0.94648	0.95076	0.95269	0.95331	0.95533	0.95745	0.95971
857	0.94653	0.95081	0.95274	0.95336	0.95537	0.95749	0.95975
858	0.94658	0.95086	0.95278	0.95341	0.95542	0.95753	0.95979
859	0.94663	0.95091	0.95283	0.95345	0.95546	0.95757	0.95983
860	0.94670	0.95096	0.95288	0.95350	0.95550	0.95761	0.95987
861	0.94674	0.95101	0.95293	0.95355	0.95555	0.95765	0.95991
862	0.94680	0.95106	0.95297	0.95359	0.95559	0.95770	0.95995
863	0.94685	0.95111	0.95302	0.95364	0.95563	0.95774	0.95999
864	0.94690	0.95116	0.95306	0.95368	0.95568	0.95778	0.96002
865	0.94695	0.95120	0.95311	0.95373	0.95572	0.95782	0.96006
866	0.94701	0.95125	0.95315	0.95377	0.95576	0.95786	0.96010
867	0.94706	0.95130	0.95320	0.95382	0.95581	0.95790	0.96014
868	0.94712	0.95135	0.95325	0.95386	0.95585	0.95794	0.96018
869	0.94717	0.95140	0.95329	0.95391	0.95589	0.95798	0.96022
870	0.94721	0.95144	0.95334	0.95395	0.95594	0.95802	0.96025
871	0.94726	0.95150	0.95338	0.95400	0.95598	0.95806	0.96029
872	0.94731	0.95154	0.95343	0.95404	0.95602	0.95811	0.96033
873	0.94737	0.95159	0.95347	0.95409	0.95606	0.95814	0.96037
874	0.94742	0.95164	0.95352	0.95413	0.95611	0.95819	0.96041
875	0.94747	0.95169	0.95356	0.95418	0.95615	0.95823	0.96044
876	0.94753	0.95174	0.95360	0.95422	0.95619	0.95826	0.96048
877	0.94758	0.95178	0.95365	0.95426	0.95623	0.95831	0.96052
878	0.94762	0.95182	0.95369	0.95431	0.95627	0.95835	0.96056
879	0.94767	0.95187	0.95374	0.95435	0.95632	0.95838	0.96059
880	0.94773	0.95192	0.95378	0.95440	0.95636	0.95842	0.96063
881	0.94777	0.95196	0.95383	0.95444	0.95640	0.95846	0.96067
882	0.94783	0.95201	0.95387	0.95449	0.95644	0.95850	0.96071
883	0.94788	0.95206	0.95392	0.95453	0.95648	0.95854	0.96074
884	0.94793	0.95210	0.95397	0.95457	0.95653	0.95858	0.96078
885	0.94798	0.95214	0.95401	0.95462	0.95657	0.95862	0.96082
886	0.94803	0.95219	0.95405	0.95466	0.95661	0.95866	0.96085
887	0.94808	0.95224	0.95410	0.95470	0.95665	0.95870	0.96089
888	0.94813	0.95228	0.95414	0.95475	0.95669	0.95874	0.96093
889	0.94818	0.95233	0.95419	0.95479	0.95673	0.95878	0.96097
890	0.94824	0.95237	0.95423	0.95483	0.95678	0.95882	0.96100
891	0.94829	0.95242	0.95427	0.95488	0.95682	0.95886	0.96104
892	0.94835	0.95246	0.95432	0.95492	0.95686	0.95890	0.96108
893	0.94840	0.95251	0.95436	0.95496	0.95690	0.95893	0.96111
894	0.94845	0.95255	0.95441	0.95500	0.95694	0.95897	0.96115
895	0.94849	0.95260	0.95445	0.95505	0.95698	0.95901	0.96118
896	0.94855	0.95265	0.95449	0.95509	0.95702	0.95905	0.96122
897	0.94860	0.95269	0.95454	0.95513	0.95706	0.95909	0.96126
898	0.94865	0.95274	0.95458	0.95517	0.95710	0.95913	0.96129
899	0.94870	0.95279	0.95462	0.95522	0.95714	0.95917	0.96133
900	0.94875	0.95283	0.95467	0.95526	0.95718	0.95920	0.96136
901	0.94879	0.95287	0.95471	0.95530	0.95722	0.95924	0.96140
902	0.94884	0.95292	0.95475	0.95534	0.95726	0.95928	0.96143
903	0.94890	0.95296	0.95479	0.95539	0.95730	0.95932	0.96147
904	0.94895	0.95301	0.95484	0.95543	0.95734	0.95936	0.96151
905	0.94899	0.95305	0.95488	0.95547	0.95738	0.95939	0.96154
906	0.94904	0.95309	0.95492	0.95551	0.95742	0.95943	0.96158
907	0.94908	0.95314	0.95496	0.95555	0.95746	0.95947	0.96161
908	0.94913	0.95318	0.95500	0.95560	0.95750	0.95950	0.96165
909	0.94919	0.95322	0.95505	0.95564	0.95754	0.95954	0.96168
910	0.94924	0.95327	0.95509	0.95568	0.95758	0.95958	0.96172
911	0.94929	0.95331	0.95513	0.95572	0.95762	0.95962	0.96175
912	0.94933	0.95335	0.95517	0.95576	0.95766	0.95965	0.96179
913	0.94938	0.95340	0.95521	0.95580	0.95770	0.95969	0.96182
914	0.94944	0.95344	0.95526	0.95584	0.95774	0.95973	0.96186
915	0.94949	0.95349	0.95530	0.95589	0.95778	0.95977	0.96189
916	0.94953	0.95353	0.95534	0.95593	0.95782	0.95980	0.96193
917	0.94958	0.95358	0.95538	0.95597	0.95785	0.95984	0.96196

N	0.1%	0.5%	1%	1.25%	2.5%	5%	10%
918	0.94963	0.95362	0.95542	0.95601	0.95789	0.95988	0.96200
919	0.94967	0.95366	0.95546	0.95605	0.95793	0.95991	0.96203
920	0.94972	0.95370	0.95550	0.95609	0.95797	0.95995	0.96206
921	0.94976	0.95374	0.95555	0.95613	0.95801	0.95999	0.96210
922	0.94982	0.95378	0.95559	0.95617	0.95805	0.96002	0.96213
923	0.94986	0.95383	0.95563	0.95621	0.95809	0.96006	0.96217
924	0.94990	0.95387	0.95567	0.95625	0.95813	0.96010	0.96220
925	0.94995	0.95391	0.95571	0.95629	0.95816	0.96013	0.96224
926	0.94999	0.95395	0.95575	0.95633	0.95820	0.96017	0.96227
927	0.95005	0.95400	0.95579	0.95637	0.95824	0.96020	0.96230
928	0.95009	0.95404	0.95583	0.95641	0.95828	0.96024	0.96234
929	0.95013	0.95408	0.95587	0.95645	0.95832	0.96028	0.96237
930	0.95018	0.95412	0.95591	0.95649	0.95835	0.96031	0.96241
931	0.95022	0.95417	0.95595	0.95653	0.95839	0.96035	0.96244
932	0.95027	0.95421	0.95599	0.95657	0.95843	0.96038	0.96247
933	0.95032	0.95425	0.95603	0.95661	0.95847	0.96042	0.96251
934	0.95036	0.95430	0.95607	0.95665	0.95851	0.96046	0.96254
935	0.95041	0.95434	0.95611	0.95669	0.95854	0.96049	0.96257
936	0.95046	0.95438	0.95615	0.95673	0.95858	0.96053	0.96261
937	0.95050	0.95442	0.95619	0.95677	0.95862	0.96056	0.96264
938	0.95054	0.95446	0.95623	0.95681	0.95866	0.96060	0.96267
939	0.95059	0.95450	0.95627	0.95685	0.95869	0.96063	0.96271
940	0.95063	0.95454	0.95631	0.95689	0.95873	0.96067	0.96274
941	0.95068	0.95459	0.95635	0.95693	0.95877	0.96070	0.96277
942	0.95073	0.95463	0.95639	0.95697	0.95881	0.96074	0.96281
943	0.95077	0.95467	0.95643	0.95701	0.95884	0.96077	0.96284
944	0.95082	0.95471	0.95647	0.95704	0.95888	0.96081	0.96287
945	0.95086	0.95475	0.95651	0.95708	0.95892	0.96084	0.96291
946	0.95091	0.95479	0.95655	0.95712	0.95895	0.96088	0.96294
947	0.95095	0.95483	0.95659	0.95716	0.95899	0.96091	0.96297
948	0.95100	0.95488	0.95663	0.95720	0.95903	0.96095	0.96300
949	0.95104	0.95492	0.95667	0.95724	0.95906	0.96098	0.96304
950	0.95109	0.95496	0.95670	0.95728	0.95910	0.96102	0.96307
951	0.95114	0.95500	0.95674	0.95731	0.95914	0.96105	0.96310
952	0.95118	0.95504	0.95678	0.95735	0.95917	0.96109	0.96313
953	0.95122	0.95508	0.95682	0.95739	0.95921	0.96112	0.96317
954	0.95126	0.95512	0.95686	0.95743	0.95924	0.96115	0.96320
955	0.95130	0.95516	0.95690	0.95747	0.95928	0.96119	0.96323
956	0.95135	0.95520	0.95693	0.95750	0.95932	0.96122	0.96326
957	0.95140	0.95524	0.95697	0.95754	0.95935	0.96126	0.96330
958	0.95144	0.95528	0.95701	0.95758	0.95939	0.96129	0.96333
959	0.95149	0.95532	0.95705	0.95762	0.95942	0.96133	0.96336
960	0.95153	0.95536	0.95709	0.95766	0.95946	0.96136	0.96339
961	0.95157	0.95540	0.95713	0.95769	0.95950	0.96139	0.96343
962	0.95161	0.95544	0.95716	0.95773	0.95953	0.96143	0.96346
963	0.95165	0.95548	0.95720	0.95777	0.95957	0.96146	0.96349
964	0.95169	0.95552	0.95724	0.95781	0.95960	0.96149	0.96352
965	0.95173	0.95556	0.95728	0.95785	0.95964	0.96153	0.96355
966	0.95178	0.95559	0.95732	0.95788	0.95967	0.96156	0.96358
967	0.95182	0.95563	0.95736	0.95792	0.95971	0.96159	0.96361
968	0.95187	0.95568	0.95739	0.95795	0.95975	0.96163	0.96365
969	0.95191	0.95572	0.95743	0.95799	0.95978	0.96166	0.96368
970	0.95196	0.95575	0.95747	0.95803	0.95982	0.96169	0.96371
971	0.95200	0.95579	0.95750	0.95806	0.95985	0.96173	0.96374
972	0.95205	0.95583	0.95754	0.95810	0.95989	0.96176	0.96377
973	0.95209	0.95587	0.95758	0.95814	0.95992	0.96179	0.96380
974	0.95213	0.95591	0.95762	0.95817	0.95996	0.96183	0.96383
975	0.95217	0.95595	0.95765	0.95821	0.95999	0.96186	0.96387
976	0.95221	0.95599	0.95769	0.95825	0.96002	0.96189	0.96390
977	0.95225	0.95603	0.95773	0.95829	0.96006	0.96192	0.96393
978	0.95229	0.95606	0.95776	0.95832	0.96009	0.96196	0.96396
979	0.95234	0.95610	0.95780	0.95836	0.96013	0.96199	0.96399
980	0.95238	0.95614	0.95784	0.95840	0.96016	0.96202	0.96402
981	0.95242	0.95618	0.95788	0.95843	0.96020	0.96206	0.96405
982	0.95247	0.95622	0.95791	0.95847	0.96023	0.96209	0.96408
983	0.95251	0.95626	0.95795	0.95850	0.96027	0.96212	0.96411
984	0.95255	0.95629	0.95799	0.95854	0.96030	0.96215	0.96414
985	0.95259	0.95633	0.95802	0.95858	0.96034	0.96219	0.96417
986	0.95263	0.95637	0.95806	0.95861	0.96037	0.96222	0.96420
987	0.95268	0.95641	0.95810	0.95865	0.96041	0.96225	0.96423
988	0.95271	0.95644	0.95813	0.95868	0.96044	0.96228	0.96426
989	0.95275	0.95649	0.95817	0.95872	0.96047	0.96232	0.96429
990	0.95280	0.95652	0.95820	0.95875	0.96051	0.96235	0.96432
991	0.95284	0.95656	0.95824	0.95879	0.96054	0.96238	0.96435

N	0.1%	0.5%	1%	1.25%	2.5%	5%	10%
992	0.95288	0.95660	0.95828	0.95883	0.96058	0.96241	0.96438
993	0.95292	0.95664	0.95831	0.95886	0.96061	0.96244	0.96441
994	0.95296	0.95668	0.95835	0.95890	0.96064	0.96248	0.96444
995	0.95300	0.95672	0.95839	0.95893	0.96068	0.96251	0.96447
996	0.95305	0.95676	0.95842	0.95897	0.96071	0.96254	0.96450
997	0.95309	0.95679	0.95846	0.95900	0.96074	0.96257	0.96453
998	0.95313	0.95683	0.95849	0.95904	0.96078	0.96260	0.96456
999	0.95318	0.95687	0.95853	0.95907	0.96081	0.96263	0.96459
1000	0.95322	0.95690	0.95856	0.95911	0.96084	0.96267	0.96462
