# Supplementary material for: A generalized outlier method with an automated test protocol applied to proficiency testing schemes: utilizing Monte Carlo simulation for generating critical values for the extended Grubbs statistic
Source: J AOAC Int. 2026 Apr 29;109(6):974–84. doi: 10.1093/jaoacint/qsag036 (PMC13732683; doi:10.1093/jaoacint/qsag036)
Supplement: Supplementary file 1 [file qsag036_supplementary_data.zip › aoac-25-0307-File016.pdf]

Extended Grubbs Statistics Tables for Quadruple Outliers

Table 1 – *GQ(HHHH or LLLL)*. Critical values of upper significance levels for the Grubbs quadruple outlier test, applicable in situations where the four highest or four lowest observations are simultaneously identified as outliers, denoted as *GQ(HHHH)* and *GQ(LLLL)*, respectively.

N	0.1%	0.5%	1%	1.25%	2.5%	5%	10%
6	0.00000	0.00000	0.00001	0.00001	0.00006	0.00023	0.00093
7	0.00011	0.00058	0.00117	0.00148	0.00303	0.00631	0.01337
8	0.00143	0.00429	0.00695	0.00814	0.01338	0.02216	0.03740
9	0.00539	0.01256	0.01817	0.02050	0.02999	0.04430	0.06650
10	0.01254	0.02481	0.03356	0.03702	0.05047	0.06951	0.09706
11	0.02244	0.03992	0.05150	0.05598	0.07288	0.09573	0.12728
12	0.03449	0.05698	0.07116	0.07650	0.09620	0.12196	0.15641
13	0.04817	0.07515	0.09149	0.09754	0.11958	0.14750	0.18412
14	0.06305	0.09379	0.11195	0.11861	0.14239	0.17221	0.21022
15	0.07864	0.11264	0.13224	0.13942	0.16466	0.19572	0.23474
16	0.09462	0.13149	0.15238	0.15986	0.18617	0.21813	0.25768
17	0.11050	0.15015	0.17185	0.17955	0.20676	0.23936	0.27928
18	0.12662	0.16822	0.19070	0.19877	0.22654	0.25950	0.29953
19	0.14306	0.18596	0.20909	0.21722	0.24537	0.27859	0.31866
20	0.15822	0.20285	0.22652	0.23479	0.26332	0.29672	0.33667
21	0.17398	0.21929	0.24330	0.25178	0.28049	0.31387	0.35363
22	0.18880	0.23511	0.25943	0.26792	0.29685	0.33020	0.36965
23	0.20352	0.25036	0.27494	0.28357	0.31240	0.34570	0.38471
24	0.21784	0.26537	0.29005	0.29858	0.32738	0.36042	0.39908
25	0.23164	0.27955	0.30431	0.31293	0.34167	0.37444	0.41266
26	0.24491	0.29340	0.31803	0.32671	0.35522	0.38772	0.42554
27	0.25774	0.30653	0.33129	0.33986	0.36826	0.40041	0.43781
28	0.27033	0.31921	0.34395	0.35248	0.38064	0.41253	0.44949
29	0.28260	0.33144	0.35610	0.36455	0.39256	0.42419	0.46061
30	0.29450	0.34324	0.36785	0.37616	0.40400	0.43523	0.47124
31	0.30620	0.35472	0.37911	0.38738	0.41505	0.44589	0.48134
32	0.31724	0.36568	0.38984	0.39811	0.42551	0.45609	0.49103
33	0.32766	0.37634	0.40036	0.40856	0.43561	0.46589	0.50032
34	0.33822	0.38665	0.41041	0.41851	0.44536	0.47524	0.50919
35	0.34875	0.39640	0.42009	0.42809	0.45472	0.48424	0.51769
36	0.35877	0.40604	0.42933	0.43733	0.46363	0.49289	0.52588
37	0.36804	0.41528	0.43846	0.44633	0.47240	0.50122	0.53377
38	0.37730	0.42426	0.44721	0.45498	0.48078	0.50926	0.54139
39	0.38637	0.43290	0.45568	0.46336	0.48885	0.51700	0.54870
40	0.39497	0.44121	0.46374	0.47139	0.49668	0.52451	0.55575
41	0.40338	0.44938	0.47166	0.47927	0.50421	0.53170	0.56255
42	0.41159	0.45737	0.47942	0.48694	0.51150	0.53870	0.56910
43	0.41970	0.46493	0.48679	0.49421	0.51852	0.54539	0.57542
44	0.42744	0.47233	0.49395	0.50127	0.52535	0.55189	0.58154
45	0.43491	0.47947	0.50091	0.50813	0.53201	0.55817	0.58750
46	0.44222	0.48636	0.50763	0.51485	0.53839	0.56427	0.59322
47	0.44951	0.49319	0.51422	0.52133	0.54468	0.57020	0.59877
48	0.45643	0.49969	0.52052	0.52755	0.55063	0.57595	0.60416
49	0.46306	0.50616	0.52673	0.53363	0.55649	0.58152	0.60939
50	0.46958	0.51230	0.53274	0.53968	0.56217	0.58694	0.61445
51	0.47600	0.51835	0.53864	0.54545	0.56769	0.59214	0.61935
52	0.48231	0.52428	0.54433	0.55107	0.57313	0.59724	0.62411
53	0.48843	0.52998	0.54989	0.55651	0.57835	0.60219	0.62873
54	0.49427	0.53556	0.55522	0.56183	0.58345	0.60701	0.63321
55	0.50013	0.54103	0.56047	0.56699	0.58844	0.61175	0.63763
56	0.50546	0.54634	0.56561	0.57212	0.59324	0.61631	0.64190
57	0.51092	0.55148	0.57061	0.57702	0.59796	0.62075	0.64606
58	0.51628	0.55659	0.57551	0.58182	0.60252	0.62509	0.65008
59	0.52177	0.56155	0.58023	0.58649	0.60698	0.62931	0.65401
60	0.52685	0.56636	0.58487	0.59110	0.61132	0.63342	0.65787
61	0.53189	0.57090	0.58932	0.59554	0.61554	0.63741	0.66161
62	0.53686	0.57551	0.59376	0.59984	0.61970	0.64136	0.66527
63	0.54151	0.57992	0.59805	0.60404	0.62374	0.64515	0.66881
64	0.54616	0.58434	0.60226	0.60816	0.62768	0.64886	0.67229
65	0.55054	0.58869	0.60638	0.61225	0.63153	0.65252	0.67570
66	0.55508	0.59270	0.61033	0.61619	0.63528	0.65608	0.67902
67	0.55958	0.59679	0.61421	0.62002	0.63897	0.65954	0.68223
68	0.56391	0.60077	0.61800	0.62383	0.64257	0.66292	0.68540
69	0.56813	0.60467	0.62173	0.62746	0.64609	0.66623	0.68848
70	0.57221	0.60845	0.62540	0.63104	0.64952	0.66949	0.69148
71	0.57625	0.61222	0.62893	0.63452	0.65284	0.67264	0.69444

N	0.1%	0.5%	1%	1.25%	2.5%	5%	10%
72	0.58030	0.61583	0.63244	0.63800	0.65614	0.67574	0.69732
73	0.58416	0.61941	0.63595	0.64143	0.65934	0.67876	0.70015
74	0.58787	0.62291	0.63926	0.64471	0.66251	0.68175	0.70291
75	0.59166	0.62633	0.64257	0.64798	0.66558	0.68465	0.70562
76	0.59538	0.62970	0.64580	0.65119	0.66860	0.68750	0.70828
77	0.59878	0.63298	0.64897	0.65432	0.67157	0.69029	0.71086
78	0.60234	0.63617	0.65207	0.65735	0.67448	0.69300	0.71341
79	0.60591	0.63937	0.65511	0.66032	0.67731	0.69571	0.71592
80	0.60921	0.64242	0.65810	0.66328	0.68014	0.69833	0.71836
81	0.61246	0.64551	0.66100	0.66616	0.68287	0.70092	0.72076
82	0.61563	0.64843	0.66387	0.66897	0.68555	0.70346	0.72312
83	0.61868	0.65145	0.66667	0.67173	0.68818	0.70594	0.72543
84	0.62192	0.65428	0.66942	0.67445	0.69076	0.70837	0.72770
85	0.62509	0.65712	0.67212	0.67712	0.69329	0.71078	0.72994
86	0.62803	0.65993	0.67479	0.67974	0.69579	0.71313	0.73211
87	0.63105	0.66259	0.67741	0.68232	0.69822	0.71543	0.73425
88	0.63397	0.66532	0.67995	0.68485	0.70064	0.71771	0.73634
89	0.63680	0.66795	0.68250	0.68737	0.70302	0.71992	0.73840
90	0.63962	0.67059	0.68497	0.68978	0.70534	0.72210	0.74043
91	0.64234	0.67308	0.68746	0.69220	0.70763	0.72424	0.74243
92	0.64490	0.67560	0.68987	0.69459	0.70987	0.72636	0.74439
93	0.64770	0.67809	0.69224	0.69688	0.71208	0.72844	0.74630
94	0.65035	0.68048	0.69453	0.69916	0.71423	0.73048	0.74821
95	0.65287	0.68281	0.69679	0.70138	0.71637	0.73247	0.75007
96	0.65537	0.68524	0.69900	0.70358	0.71847	0.73444	0.75192
97	0.65786	0.68747	0.70118	0.70575	0.72053	0.73640	0.75372
98	0.66035	0.68983	0.70337	0.70789	0.72255	0.73829	0.75550
99	0.66276	0.69198	0.70554	0.71002	0.72453	0.74017	0.75723
100	0.66512	0.69420	0.70766	0.71208	0.72650	0.74202	0.75895
101	0.66749	0.69631	0.70974	0.71411	0.72845	0.74385	0.76064
102	0.66990	0.69842	0.71173	0.71610	0.73036	0.74563	0.76231
103	0.67231	0.70054	0.71372	0.71806	0.73225	0.74737	0.76395
104	0.67459	0.70267	0.71570	0.72001	0.73408	0.74909	0.76556
105	0.67693	0.70469	0.71762	0.72191	0.73588	0.75079	0.76714
106	0.67901	0.70673	0.71956	0.72379	0.73768	0.75249	0.76870
107	0.68119	0.70866	0.72146	0.72567	0.73944	0.75413	0.77024
108	0.68338	0.71065	0.72331	0.72751	0.74117	0.75577	0.77175
109	0.68564	0.71261	0.72511	0.72932	0.74286	0.75737	0.77324
110	0.68756	0.71448	0.72693	0.73109	0.74455	0.75895	0.77472
111	0.68967	0.71633	0.72873	0.73282	0.74621	0.76051	0.77616
112	0.69165	0.71816	0.73045	0.73456	0.74783	0.76205	0.77759
113	0.69354	0.71998	0.73218	0.73627	0.74943	0.76356	0.77901
114	0.69549	0.72177	0.73389	0.73794	0.75103	0.76506	0.78039
115	0.69737	0.72349	0.73556	0.73958	0.75258	0.76653	0.78174
116	0.69923	0.72521	0.73721	0.74121	0.75413	0.76799	0.78310
117	0.70107	0.72691	0.73882	0.74279	0.75565	0.76941	0.78442
118	0.70299	0.72860	0.74044	0.74439	0.75715	0.77080	0.78573
119	0.70486	0.73025	0.74205	0.74596	0.75863	0.77221	0.78702
120	0.70672	0.73190	0.74360	0.74751	0.76009	0.77359	0.78830
121	0.70852	0.73354	0.74515	0.74903	0.76153	0.77495	0.78956
122	0.71019	0.73513	0.74669	0.75056	0.76296	0.77629	0.79080
123	0.71186	0.73673	0.74821	0.75205	0.76436	0.77760	0.79202
124	0.71357	0.73824	0.74966	0.75350	0.76576	0.77890	0.79323
125	0.71533	0.73979	0.75118	0.75496	0.76712	0.78019	0.79442
126	0.71693	0.74133	0.75263	0.75639	0.76848	0.78146	0.79560
127	0.71852	0.74286	0.75407	0.75781	0.76981	0.78272	0.79676
128	0.72004	0.74433	0.75549	0.75921	0.77112	0.78395	0.79791
129	0.72165	0.74582	0.75689	0.76056	0.77242	0.78518	0.79904
130	0.72335	0.74722	0.75825	0.76191	0.77370	0.78638	0.80016
131	0.72491	0.74861	0.75958	0.76323	0.77497	0.78757	0.80127
132	0.72639	0.75003	0.76093	0.76455	0.77621	0.78874	0.80237
133	0.72792	0.75140	0.76224	0.76584	0.77744	0.78990	0.80345
134	0.72949	0.75277	0.76357	0.76714	0.77868	0.79105	0.80451
135	0.73089	0.75415	0.76485	0.76840	0.77988	0.79218	0.80556
136	0.73244	0.75549	0.76614	0.76970	0.78108	0.79329	0.80659
137	0.73385	0.75684	0.76739	0.77094	0.78227	0.79439	0.80762
138	0.73526	0.75816	0.76865	0.77217	0.78343	0.79549	0.80864
139	0.73664	0.75946	0.76988	0.77337	0.78460	0.79656	0.80964
140	0.73807	0.76078	0.77113	0.77459	0.78573	0.79764	0.81063
141	0.73955	0.76204	0.77233	0.77577	0.78685	0.79869	0.81161
142	0.74101	0.76329	0.77352	0.77694	0.78797	0.79973	0.81258
143	0.74229	0.76453	0.77468	0.77810	0.78905	0.80078	0.81353
144	0.74355	0.76573	0.77589	0.77924	0.79014	0.80179	0.81448
145	0.74491	0.76695	0.77705	0.78039	0.79122	0.80279	0.81541

N	0.1%	0.5%	1%	1.25%	2.5%	5%	10%
146	0.74626	0.76815	0.77820	0.78150	0.79229	0.80378	0.81634
147	0.74747	0.76933	0.77929	0.78261	0.79333	0.80475	0.81725
148	0.74875	0.77051	0.78040	0.78369	0.79435	0.80573	0.81815
149	0.75008	0.77163	0.78152	0.78478	0.79537	0.80670	0.81905
150	0.75129	0.77276	0.78263	0.78585	0.79639	0.80765	0.81993
151	0.75249	0.77387	0.78369	0.78693	0.79740	0.80859	0.82080
152	0.75378	0.77498	0.78476	0.78799	0.79838	0.80952	0.82168
153	0.75498	0.77612	0.78582	0.78902	0.79936	0.81045	0.82253
154	0.75620	0.77721	0.78685	0.79006	0.80034	0.81135	0.82338
155	0.75741	0.77830	0.78788	0.79106	0.80132	0.81227	0.82421
156	0.75854	0.77936	0.78889	0.79207	0.80227	0.81317	0.82506
157	0.75964	0.78040	0.78991	0.79306	0.80321	0.81404	0.82587
158	0.76079	0.78143	0.79090	0.79405	0.80416	0.81492	0.82668
159	0.76197	0.78248	0.79189	0.79502	0.80506	0.81578	0.82748
160	0.76317	0.78349	0.79285	0.79598	0.80597	0.81663	0.82827
161	0.76426	0.78451	0.79382	0.79691	0.80687	0.81748	0.82906
162	0.76537	0.78551	0.79477	0.79787	0.80776	0.81832	0.82984
163	0.76644	0.78652	0.79572	0.79880	0.80864	0.81915	0.83062
164	0.76755	0.78751	0.79668	0.79974	0.80952	0.81998	0.83137
165	0.76863	0.78851	0.79759	0.80063	0.81037	0.82079	0.83212
166	0.76972	0.78943	0.79853	0.80152	0.81123	0.82159	0.83287
167	0.77070	0.79039	0.79944	0.80242	0.81208	0.82238	0.83361
168	0.77168	0.79134	0.80034	0.80330	0.81293	0.82317	0.83435
169	0.77273	0.79227	0.80122	0.80418	0.81376	0.82395	0.83507
170	0.77372	0.79317	0.80211	0.80505	0.81458	0.82473	0.83578
171	0.77475	0.79409	0.80298	0.80591	0.81537	0.82550	0.83649
172	0.77574	0.79499	0.80384	0.80676	0.81619	0.82625	0.83720
173	0.77672	0.79590	0.80470	0.80760	0.81699	0.82700	0.83790
174	0.77767	0.79679	0.80554	0.80843	0.81778	0.82776	0.83859
175	0.77867	0.79766	0.80637	0.80927	0.81856	0.82849	0.83928
176	0.77957	0.79853	0.80719	0.81007	0.81934	0.82923	0.83995
177	0.78063	0.79940	0.80805	0.81088	0.82011	0.82995	0.84062
178	0.78153	0.80024	0.80885	0.81170	0.82088	0.83067	0.84129
179	0.78243	0.80112	0.80966	0.81249	0.82164	0.83137	0.84194
180	0.78335	0.80198	0.81047	0.81330	0.82238	0.83207	0.84260
181	0.78424	0.80281	0.81127	0.81406	0.82311	0.83277	0.84325
182	0.78511	0.80363	0.81204	0.81484	0.82384	0.83345	0.84389
183	0.78609	0.80446	0.81283	0.81561	0.82457	0.83413	0.84452
184	0.78697	0.80528	0.81360	0.81636	0.82529	0.83481	0.84515
185	0.78786	0.80611	0.81437	0.81711	0.82600	0.83547	0.84578
186	0.78869	0.80691	0.81514	0.81788	0.82671	0.83614	0.84640
187	0.78957	0.80768	0.81590	0.81861	0.82742	0.83681	0.84701
188	0.79042	0.80843	0.81665	0.81936	0.82811	0.83746	0.84761
189	0.79131	0.80923	0.81741	0.82010	0.82879	0.83811	0.84822
190	0.79218	0.80999	0.81813	0.82083	0.82947	0.83875	0.84882
191	0.79307	0.81073	0.81885	0.82155	0.83015	0.83938	0.84940
192	0.79387	0.81150	0.81957	0.82225	0.83082	0.84001	0.84999
193	0.79468	0.81226	0.82029	0.82295	0.83149	0.84063	0.85058
194	0.79550	0.81301	0.82101	0.82364	0.83214	0.84125	0.85115
195	0.79627	0.81373	0.82170	0.82432	0.83281	0.84187	0.85172
196	0.79713	0.81446	0.82239	0.82500	0.83346	0.84248	0.85229
197	0.79794	0.81520	0.82309	0.82568	0.83410	0.84309	0.85285
198	0.79863	0.81591	0.82379	0.82635	0.83474	0.84368	0.85341
199	0.79948	0.81665	0.82447	0.82703	0.83536	0.84428	0.85396
200	0.80028	0.81735	0.82514	0.82770	0.83600	0.84487	0.85451
201	0.80103	0.81806	0.82580	0.82835	0.83663	0.84546	0.85506
202	0.80180	0.81874	0.82647	0.82902	0.83725	0.84604	0.85559
203	0.80250	0.81943	0.82712	0.82966	0.83785	0.84661	0.85612
204	0.80332	0.82012	0.82777	0.83031	0.83846	0.84717	0.85665
205	0.80408	0.82078	0.82843	0.83094	0.83906	0.84775	0.85718
206	0.80482	0.82144	0.82905	0.83157	0.83966	0.84831	0.85771
207	0.80559	0.82213	0.82969	0.83220	0.84025	0.84887	0.85822
208	0.80630	0.82278	0.83033	0.83282	0.84084	0.84941	0.85874
209	0.80701	0.82344	0.83095	0.83341	0.84140	0.84996	0.85925
210	0.80771	0.82407	0.83155	0.83402	0.84199	0.85050	0.85975
211	0.80838	0.82473	0.83217	0.83462	0.84256	0.85104	0.86025
212	0.80907	0.82539	0.83278	0.83523	0.84313	0.85158	0.86075
213	0.80976	0.82601	0.83338	0.83582	0.84369	0.85211	0.86123
214	0.81049	0.82665	0.83399	0.83641	0.84425	0.85262	0.86172
215	0.81118	0.82726	0.83457	0.83699	0.84479	0.85315	0.86221
216	0.81188	0.82786	0.83517	0.83758	0.84534	0.85367	0.86269
217	0.81251	0.82851	0.83576	0.83816	0.84589	0.85418	0.86317
218	0.81315	0.82909	0.83634	0.83873	0.84643	0.85470	0.86365
219	0.81384	0.82970	0.83691	0.83929	0.84697	0.85520	0.86411

N	0.1%	0.5%	1%	1.25%	2.5%	5%	10%
220	0.81449	0.83029	0.83748	0.83985	0.84751	0.85570	0.86458
221	0.81518	0.83089	0.83806	0.84042	0.84804	0.85619	0.86504
222	0.81579	0.83147	0.83863	0.84098	0.84856	0.85668	0.86550
223	0.81646	0.83205	0.83918	0.84155	0.84909	0.85717	0.86595
224	0.81709	0.83266	0.83975	0.84209	0.84961	0.85766	0.86641
225	0.81771	0.83323	0.84030	0.84264	0.85012	0.85814	0.86686
226	0.81837	0.83380	0.84086	0.84317	0.85063	0.85863	0.86731
227	0.81902	0.83438	0.84140	0.84371	0.85113	0.85910	0.86775
228	0.81964	0.83494	0.84195	0.84425	0.85165	0.85957	0.86819
229	0.82024	0.83547	0.84248	0.84476	0.85214	0.86004	0.86862
230	0.82084	0.83605	0.84301	0.84529	0.85263	0.86051	0.86905
231	0.82148	0.83658	0.84353	0.84580	0.85312	0.86097	0.86948
232	0.82209	0.83715	0.84405	0.84632	0.85361	0.86142	0.86991
233	0.82267	0.83770	0.84456	0.84683	0.85409	0.86188	0.87033
234	0.82324	0.83823	0.84508	0.84733	0.85457	0.86233	0.87075
235	0.82384	0.83877	0.84558	0.84784	0.85505	0.86277	0.87116
236	0.82442	0.83929	0.84610	0.84834	0.85552	0.86322	0.87158
237	0.82496	0.83984	0.84660	0.84884	0.85599	0.86366	0.87199
238	0.82553	0.84038	0.84711	0.84933	0.85645	0.86410	0.87239
239	0.82615	0.84088	0.84760	0.84981	0.85693	0.86453	0.87279
240	0.82672	0.84140	0.84811	0.85031	0.85738	0.86496	0.87320
241	0.82725	0.84191	0.84858	0.85078	0.85784	0.86539	0.87360
242	0.82785	0.84243	0.84908	0.85127	0.85829	0.86582	0.87399
243	0.82842	0.84291	0.84956	0.85175	0.85874	0.86624	0.87439
244	0.82894	0.84343	0.85004	0.85222	0.85920	0.86666	0.87478
245	0.82952	0.84393	0.85052	0.85268	0.85964	0.86708	0.87517
246	0.83008	0.84444	0.85099	0.85316	0.86008	0.86750	0.87556
247	0.83062	0.84493	0.85146	0.85361	0.86053	0.86791	0.87593
248	0.83118	0.84539	0.85194	0.85409	0.86097	0.86832	0.87632
249	0.83175	0.84588	0.85240	0.85454	0.86140	0.86872	0.87670
250	0.83226	0.84636	0.85285	0.85499	0.86183	0.86912	0.87707
251	0.83277	0.84684	0.85330	0.85543	0.86225	0.86952	0.87744
252	0.83333	0.84732	0.85374	0.85588	0.86267	0.86993	0.87782
253	0.83381	0.84780	0.85419	0.85633	0.86310	0.87032	0.87818
254	0.83429	0.84826	0.85464	0.85677	0.86352	0.87071	0.87855
255	0.83479	0.84873	0.85509	0.85719	0.86393	0.87111	0.87891
256	0.83530	0.84920	0.85555	0.85764	0.86435	0.87149	0.87927
257	0.83584	0.84966	0.85599	0.85808	0.86475	0.87187	0.87963
258	0.83636	0.85013	0.85642	0.85851	0.86515	0.87226	0.87999
259	0.83684	0.85059	0.85685	0.85893	0.86556	0.87264	0.88034
260	0.83734	0.85102	0.85728	0.85934	0.86596	0.87301	0.88069
261	0.83784	0.85146	0.85770	0.85977	0.86636	0.87338	0.88104
262	0.83828	0.85190	0.85813	0.86018	0.86676	0.87376	0.88139
263	0.83878	0.85235	0.85855	0.86061	0.86716	0.87412	0.88173
264	0.83926	0.85278	0.85897	0.86102	0.86755	0.87449	0.88207
265	0.83975	0.85322	0.85939	0.86143	0.86794	0.87486	0.88241
266	0.84021	0.85364	0.85980	0.86184	0.86831	0.87522	0.88275
267	0.84063	0.85408	0.86022	0.86225	0.86870	0.87558	0.88309
268	0.84115	0.85451	0.86064	0.86265	0.86908	0.87594	0.88342
269	0.84161	0.85494	0.86104	0.86305	0.86945	0.87629	0.88375
270	0.84208	0.85538	0.86144	0.86344	0.86983	0.87665	0.88408
271	0.84252	0.85579	0.86185	0.86383	0.87021	0.87700	0.88441
272	0.84302	0.85621	0.86225	0.86422	0.87058	0.87735	0.88474
273	0.84346	0.85661	0.86263	0.86462	0.87094	0.87770	0.88506
274	0.84391	0.85703	0.86303	0.86500	0.87132	0.87804	0.88538
275	0.84439	0.85744	0.86341	0.86537	0.87168	0.87839	0.88570
276	0.84481	0.85785	0.86380	0.86577	0.87204	0.87874	0.88602
277	0.84527	0.85826	0.86418	0.86615	0.87241	0.87907	0.88633
278	0.84567	0.85867	0.86456	0.86653	0.87276	0.87940	0.88665
279	0.84615	0.85906	0.86495	0.86690	0.87311	0.87974	0.88696
280	0.84659	0.85946	0.86533	0.86726	0.87347	0.88007	0.88727
281	0.84702	0.85985	0.86571	0.86764	0.87382	0.88040	0.88758
282	0.84747	0.86023	0.86609	0.86801	0.87416	0.88073	0.88788
283	0.84792	0.86064	0.86646	0.86839	0.87451	0.88105	0.88819
284	0.84834	0.86102	0.86683	0.86875	0.87486	0.88138	0.88849
285	0.84881	0.86140	0.86720	0.86911	0.87520	0.88170	0.88879
286	0.84920	0.86178	0.86756	0.86947	0.87555	0.88203	0.88909
287	0.84962	0.86217	0.86793	0.86982	0.87589	0.88234	0.88939
288	0.85002	0.86255	0.86828	0.87018	0.87622	0.88266	0.88968
289	0.85047	0.86292	0.86864	0.87054	0.87656	0.88298	0.88998
290	0.85089	0.86330	0.86900	0.87089	0.87689	0.88329	0.89027
291	0.85132	0.86368	0.86935	0.87123	0.87722	0.88360	0.89056
292	0.85172	0.86404	0.86972	0.87160	0.87755	0.88391	0.89085
293	0.85213	0.86440	0.87006	0.87194	0.87788	0.88422	0.89114

N	0.1%	0.5%	1%	1.25%	2.5%	5%	10%
294	0.85252	0.86478	0.87042	0.87228	0.87820	0.88452	0.89142
295	0.85290	0.86514	0.87076	0.87262	0.87853	0.88483	0.89170
296	0.85330	0.86550	0.87111	0.87296	0.87886	0.88513	0.89199
297	0.85367	0.86587	0.87146	0.87330	0.87917	0.88543	0.89227
298	0.85411	0.86623	0.87179	0.87363	0.87949	0.88574	0.89254
299	0.85449	0.86659	0.87213	0.87397	0.87981	0.88603	0.89282
300	0.85488	0.86694	0.87247	0.87429	0.88012	0.88633	0.89310
301	0.85526	0.86728	0.87281	0.87462	0.88043	0.88662	0.89337
302	0.85565	0.86764	0.87314	0.87495	0.88074	0.88692	0.89364
303	0.85605	0.86798	0.87347	0.87528	0.88105	0.88721	0.89391
304	0.85643	0.86831	0.87379	0.87559	0.88136	0.88750	0.89418
305	0.85680	0.86867	0.87412	0.87592	0.88167	0.88779	0.89444
306	0.85716	0.86902	0.87445	0.87624	0.88198	0.88807	0.89471
307	0.85752	0.86936	0.87478	0.87655	0.88228	0.88836	0.89498
308	0.85792	0.86969	0.87510	0.87686	0.88258	0.88865	0.89524
309	0.85828	0.87004	0.87540	0.87718	0.88287	0.88893	0.89551
310	0.85864	0.87035	0.87573	0.87749	0.88316	0.88921	0.89577
311	0.85898	0.87067	0.87604	0.87780	0.88346	0.88948	0.89602
312	0.85934	0.87100	0.87636	0.87812	0.88376	0.88976	0.89628
313	0.85971	0.87133	0.87668	0.87842	0.88405	0.89004	0.89654
314	0.86006	0.87165	0.87698	0.87872	0.88434	0.89031	0.89679
315	0.86042	0.87198	0.87730	0.87904	0.88463	0.89058	0.89705
316	0.86074	0.87232	0.87760	0.87934	0.88491	0.89085	0.89730
317	0.86110	0.87264	0.87791	0.87963	0.88520	0.89112	0.89755
318	0.86147	0.87296	0.87820	0.87993	0.88549	0.89139	0.89780
319	0.86180	0.87327	0.87850	0.88022	0.88577	0.89165	0.89805
320	0.86215	0.87359	0.87881	0.88053	0.88605	0.89191	0.89829
321	0.86250	0.87391	0.87910	0.88081	0.88633	0.89217	0.89854
322	0.86283	0.87420	0.87939	0.88110	0.88660	0.89244	0.89878
323	0.86319	0.87451	0.87968	0.88140	0.88688	0.89270	0.89903
324	0.86354	0.87481	0.87998	0.88168	0.88715	0.89296	0.89927
325	0.86387	0.87512	0.88027	0.88197	0.88742	0.89321	0.89951
326	0.86423	0.87543	0.88055	0.88225	0.88769	0.89347	0.89975
327	0.86455	0.87574	0.88084	0.88254	0.88797	0.89372	0.89998
328	0.86487	0.87603	0.88113	0.88282	0.88824	0.89398	0.90022
329	0.86525	0.87634	0.88141	0.88310	0.88850	0.89423	0.90046
330	0.86555	0.87663	0.88171	0.88337	0.88876	0.89448	0.90069
331	0.86589	0.87693	0.88200	0.88366	0.88903	0.89473	0.90092
332	0.86619	0.87722	0.88228	0.88394	0.88929	0.89498	0.90115
333	0.86649	0.87751	0.88256	0.88421	0.88955	0.89523	0.90138
334	0.86683	0.87781	0.88283	0.88448	0.88981	0.89547	0.90161
335	0.86713	0.87810	0.88310	0.88476	0.89007	0.89571	0.90184
336	0.86746	0.87839	0.88339	0.88504	0.89034	0.89596	0.90207
337	0.86776	0.87868	0.88367	0.88531	0.89059	0.89620	0.90229
338	0.86811	0.87897	0.88394	0.88558	0.89085	0.89645	0.90252
339	0.86843	0.87924	0.88421	0.88584	0.89110	0.89669	0.90274
340	0.86872	0.87954	0.88448	0.88611	0.89136	0.89692	0.90296
341	0.86904	0.87983	0.88475	0.88637	0.89161	0.89716	0.90319
342	0.86934	0.88010	0.88502	0.88664	0.89185	0.89740	0.90341
343	0.86965	0.88037	0.88528	0.88690	0.89211	0.89764	0.90363
344	0.86995	0.88065	0.88555	0.88716	0.89236	0.89786	0.90385
345	0.87025	0.88093	0.88581	0.88742	0.89260	0.89810	0.90406
346	0.87058	0.88120	0.88608	0.88768	0.89285	0.89832	0.90427
347	0.87088	0.88148	0.88634	0.88794	0.89310	0.89856	0.90449
348	0.87120	0.88175	0.88659	0.88819	0.89334	0.89879	0.90470
349	0.87148	0.88201	0.88686	0.88845	0.89358	0.89901	0.90492
350	0.87173	0.88228	0.88712	0.88870	0.89382	0.89924	0.90513
351	0.87202	0.88255	0.88737	0.88896	0.89406	0.89947	0.90534
352	0.87231	0.88282	0.88763	0.88921	0.89429	0.89970	0.90555
353	0.87260	0.88309	0.88789	0.88946	0.89453	0.89992	0.90576
354	0.87289	0.88335	0.88814	0.88971	0.89477	0.90014	0.90597
355	0.87315	0.88361	0.88839	0.88996	0.89500	0.90036	0.90617
356	0.87342	0.88388	0.88864	0.89021	0.89523	0.90058	0.90638
357	0.87370	0.88414	0.88890	0.89046	0.89547	0.90080	0.90658
358	0.87400	0.88441	0.88915	0.89070	0.89570	0.90102	0.90678
359	0.87428	0.88467	0.88940	0.89095	0.89593	0.90123	0.90699
360	0.87457	0.88492	0.88964	0.89119	0.89615	0.90145	0.90719
361	0.87484	0.88517	0.88988	0.89143	0.89638	0.90166	0.90739
362	0.87511	0.88544	0.89012	0.89166	0.89661	0.90187	0.90759
363	0.87542	0.88569	0.89037	0.89191	0.89684	0.90208	0.90779
364	0.87570	0.88594	0.89061	0.89214	0.89707	0.90230	0.90798
365	0.87597	0.88619	0.89084	0.89238	0.89729	0.90251	0.90818
366	0.87626	0.88645	0.89108	0.89261	0.89752	0.90272	0.90838
367	0.87654	0.88670	0.89133	0.89285	0.89774	0.90292	0.90857

N	0.1%	0.5%	1%	1.25%	2.5%	5%	10%
368	0.87679	0.88694	0.89156	0.89309	0.89796	0.90313	0.90877
369	0.87707	0.88720	0.89179	0.89332	0.89817	0.90334	0.90896
370	0.87732	0.88744	0.89203	0.89354	0.89839	0.90355	0.90915
371	0.87763	0.88768	0.89226	0.89377	0.89861	0.90375	0.90934
372	0.87787	0.88792	0.89250	0.89400	0.89882	0.90396	0.90953
373	0.87814	0.88816	0.89273	0.89422	0.89904	0.90416	0.90972
374	0.87839	0.88841	0.89295	0.89445	0.89925	0.90436	0.90991
375	0.87863	0.88864	0.89318	0.89467	0.89947	0.90456	0.91010
376	0.87890	0.88888	0.89341	0.89489	0.89968	0.90477	0.91028
377	0.87915	0.88911	0.89363	0.89511	0.89989	0.90496	0.91047
378	0.87940	0.88935	0.89385	0.89533	0.90010	0.90516	0.91066
379	0.87968	0.88958	0.89408	0.89555	0.90031	0.90536	0.91084
380	0.87992	0.88981	0.89430	0.89577	0.90052	0.90555	0.91103
381	0.88018	0.89005	0.89452	0.89599	0.90073	0.90575	0.91121
382	0.88040	0.89028	0.89474	0.89620	0.90093	0.90594	0.91139
383	0.88065	0.89051	0.89496	0.89642	0.90114	0.90614	0.91157
384	0.88089	0.89074	0.89518	0.89664	0.90134	0.90633	0.91175
385	0.88116	0.89097	0.89540	0.89685	0.90154	0.90652	0.91193
386	0.88143	0.89119	0.89562	0.89706	0.90175	0.90672	0.91211
387	0.88169	0.89141	0.89583	0.89728	0.90195	0.90691	0.91228
388	0.88194	0.89164	0.89605	0.89750	0.90215	0.90709	0.91246
389	0.88220	0.89187	0.89627	0.89771	0.90235	0.90729	0.91264
390	0.88244	0.89210	0.89649	0.89792	0.90255	0.90747	0.91282
391	0.88270	0.89231	0.89669	0.89812	0.90275	0.90766	0.91299
392	0.88293	0.89254	0.89690	0.89833	0.90295	0.90785	0.91316
393	0.88318	0.89276	0.89712	0.89853	0.90314	0.90804	0.91334
394	0.88344	0.89297	0.89734	0.89874	0.90334	0.90822	0.91351
395	0.88367	0.89319	0.89754	0.89895	0.90354	0.90840	0.91368
396	0.88391	0.89340	0.89775	0.89916	0.90373	0.90859	0.91385
397	0.88417	0.89362	0.89795	0.89936	0.90392	0.90877	0.91402
398	0.88440	0.89384	0.89816	0.89956	0.90411	0.90895	0.91419
399	0.88465	0.89406	0.89836	0.89976	0.90430	0.90913	0.91436
400	0.88489	0.89427	0.89857	0.89996	0.90449	0.90931	0.91453
401	0.88512	0.89449	0.89877	0.90016	0.90468	0.90949	0.91469
402	0.88534	0.89469	0.89897	0.90037	0.90487	0.90967	0.91486
403	0.88561	0.89491	0.89917	0.90057	0.90506	0.90985	0.91503
404	0.88585	0.89512	0.89938	0.90077	0.90525	0.91003	0.91519
405	0.88606	0.89533	0.89957	0.90096	0.90544	0.91020	0.91536
406	0.88630	0.89555	0.89977	0.90115	0.90562	0.91038	0.91552
407	0.88655	0.89575	0.89997	0.90135	0.90581	0.91055	0.91568
408	0.88676	0.89596	0.90017	0.90154	0.90599	0.91073	0.91584
409	0.88699	0.89617	0.90036	0.90174	0.90618	0.91090	0.91601
410	0.88722	0.89638	0.90056	0.90193	0.90636	0.91107	0.91617
411	0.88744	0.89657	0.90076	0.90212	0.90654	0.91124	0.91633
412	0.88766	0.89677	0.90095	0.90231	0.90672	0.91141	0.91649
413	0.88788	0.89697	0.90114	0.90250	0.90690	0.91157	0.91665
414	0.88807	0.89718	0.90134	0.90269	0.90708	0.91175	0.91680
415	0.88831	0.89739	0.90153	0.90288	0.90726	0.91191	0.91696
416	0.88853	0.89759	0.90171	0.90307	0.90744	0.91208	0.91712
417	0.88875	0.89778	0.90190	0.90326	0.90762	0.91225	0.91727
418	0.88899	0.89798	0.90209	0.90344	0.90780	0.91242	0.91743
419	0.88919	0.89818	0.90227	0.90363	0.90798	0.91258	0.91758
420	0.88939	0.89837	0.90246	0.90381	0.90815	0.91275	0.91774
421	0.88961	0.89856	0.90265	0.90399	0.90833	0.91291	0.91789
422	0.88981	0.89876	0.90284	0.90418	0.90850	0.91308	0.91804
423	0.89005	0.89895	0.90303	0.90435	0.90867	0.91324	0.91820
424	0.89025	0.89914	0.90321	0.90453	0.90885	0.91340	0.91835
425	0.89045	0.89932	0.90339	0.90472	0.90901	0.91356	0.91850
426	0.89067	0.89952	0.90357	0.90490	0.90918	0.91373	0.91865
427	0.89087	0.89971	0.90376	0.90508	0.90935	0.91388	0.91880
428	0.89107	0.89990	0.90393	0.90526	0.90952	0.91404	0.91895
429	0.89130	0.90010	0.90411	0.90544	0.90969	0.91420	0.91910
430	0.89151	0.90029	0.90429	0.90561	0.90986	0.91436	0.91925
431	0.89170	0.90048	0.90447	0.90579	0.91002	0.91452	0.91940
432	0.89191	0.90065	0.90465	0.90596	0.91019	0.91467	0.91954
433	0.89210	0.90084	0.90483	0.90614	0.91035	0.91483	0.91969
434	0.89232	0.90102	0.90500	0.90631	0.91052	0.91499	0.91983
435	0.89251	0.90121	0.90518	0.90648	0.91068	0.91514	0.91998
436	0.89272	0.90140	0.90536	0.90666	0.91085	0.91530	0.92012
437	0.89294	0.90158	0.90553	0.90683	0.91100	0.91545	0.92027
438	0.89314	0.90176	0.90571	0.90700	0.91117	0.91560	0.92041
439	0.89334	0.90194	0.90588	0.90717	0.91133	0.91576	0.92055
440	0.89353	0.90212	0.90606	0.90734	0.91149	0.91591	0.92070
441	0.89372	0.90230	0.90623	0.90751	0.91165	0.91606	0.92084

N	0.1%	0.5%	1%	1.25%	2.5%	5%	10%
442	0.89389	0.90248	0.90640	0.90768	0.91181	0.91621	0.92098
443	0.89408	0.90265	0.90657	0.90785	0.91197	0.91636	0.92112
444	0.89427	0.90283	0.90674	0.90802	0.91213	0.91651	0.92126
445	0.89448	0.90302	0.90691	0.90819	0.91229	0.91667	0.92140
446	0.89467	0.90319	0.90708	0.90835	0.91244	0.91681	0.92154
447	0.89487	0.90336	0.90724	0.90852	0.91260	0.91696	0.92168
448	0.89508	0.90354	0.90741	0.90868	0.91276	0.91711	0.92182
449	0.89529	0.90370	0.90758	0.90885	0.91292	0.91725	0.92195
450	0.89546	0.90388	0.90774	0.90901	0.91307	0.91740	0.92209
451	0.89565	0.90406	0.90791	0.90917	0.91322	0.91754	0.92223
452	0.89584	0.90424	0.90807	0.90934	0.91338	0.91769	0.92237
453	0.89602	0.90441	0.90824	0.90950	0.91353	0.91783	0.92250
454	0.89621	0.90457	0.90840	0.90966	0.91368	0.91798	0.92264
455	0.89640	0.90474	0.90857	0.90982	0.91384	0.91812	0.92277
456	0.89658	0.90492	0.90873	0.90998	0.91399	0.91826	0.92290
457	0.89676	0.90509	0.90889	0.91014	0.91414	0.91840	0.92303
458	0.89693	0.90527	0.90905	0.91030	0.91429	0.91855	0.92317
459	0.89713	0.90543	0.90921	0.91045	0.91444	0.91869	0.92330
460	0.89731	0.90560	0.90938	0.91061	0.91459	0.91883	0.92343
461	0.89749	0.90576	0.90953	0.91078	0.91474	0.91897	0.92356
462	0.89768	0.90593	0.90969	0.91093	0.91489	0.91911	0.92369
463	0.89788	0.90609	0.90984	0.91108	0.91504	0.91925	0.92382
464	0.89806	0.90625	0.91000	0.91123	0.91518	0.91939	0.92395
465	0.89825	0.90642	0.91016	0.91139	0.91533	0.91953	0.92408
466	0.89841	0.90658	0.91031	0.91154	0.91548	0.91966	0.92421
467	0.89859	0.90676	0.91047	0.91170	0.91562	0.91980	0.92434
468	0.89877	0.90692	0.91062	0.91185	0.91577	0.91994	0.92446
469	0.89896	0.90707	0.91078	0.91200	0.91591	0.92007	0.92459
470	0.89914	0.90723	0.91093	0.91215	0.91605	0.92021	0.92472
471	0.89932	0.90740	0.91108	0.91231	0.91620	0.92034	0.92485
472	0.89948	0.90757	0.91124	0.91246	0.91634	0.92048	0.92497
473	0.89965	0.90772	0.91138	0.91260	0.91648	0.92061	0.92510
474	0.89982	0.90788	0.91154	0.91275	0.91662	0.92075	0.92523
475	0.90001	0.90804	0.91169	0.91290	0.91677	0.92088	0.92535
476	0.90018	0.90819	0.91184	0.91305	0.91691	0.92101	0.92548
477	0.90037	0.90836	0.91199	0.91320	0.91705	0.92115	0.92560
478	0.90056	0.90851	0.91214	0.91334	0.91719	0.92128	0.92572
479	0.90073	0.90867	0.91229	0.91349	0.91733	0.92141	0.92584
480	0.90089	0.90882	0.91243	0.91363	0.91747	0.92154	0.92597
481	0.90108	0.90898	0.91258	0.91378	0.91761	0.92167	0.92609
482	0.90123	0.90913	0.91272	0.91392	0.91774	0.92180	0.92621
483	0.90142	0.90929	0.91287	0.91407	0.91788	0.92193	0.92633
484	0.90159	0.90944	0.91302	0.91421	0.91802	0.92206	0.92645
485	0.90176	0.90959	0.91317	0.91436	0.91815	0.92219	0.92657
486	0.90193	0.90974	0.91331	0.91450	0.91829	0.92232	0.92669
487	0.90211	0.90990	0.91346	0.91464	0.91842	0.92245	0.92681
488	0.90227	0.91004	0.91360	0.91478	0.91856	0.92257	0.92693
489	0.90244	0.91019	0.91374	0.91492	0.91869	0.92270	0.92704
490	0.90260	0.91035	0.91389	0.91506	0.91882	0.92282	0.92716
491	0.90274	0.91049	0.91403	0.91521	0.91895	0.92295	0.92728
492	0.90290	0.91064	0.91417	0.91535	0.91909	0.92308	0.92740
493	0.90307	0.91079	0.91431	0.91549	0.91922	0.92320	0.92751
494	0.90323	0.91093	0.91445	0.91563	0.91935	0.92332	0.92763
495	0.90339	0.91108	0.91460	0.91577	0.91948	0.92345	0.92775
496	0.90355	0.91123	0.91474	0.91590	0.91961	0.92357	0.92786
497	0.90369	0.91137	0.91488	0.91604	0.91975	0.92370	0.92798
498	0.90386	0.91152	0.91502	0.91618	0.91987	0.92382	0.92809
499	0.90402	0.91166	0.91516	0.91631	0.92000	0.92394	0.92821
500	0.90417	0.91181	0.91530	0.91644	0.92013	0.92406	0.92832
501	0.90432	0.91195	0.91543	0.91658	0.92026	0.92418	0.92843
502	0.90447	0.91209	0.91557	0.91671	0.92039	0.92430	0.92854
503	0.90463	0.91224	0.91571	0.91685	0.92051	0.92443	0.92866
504	0.90480	0.91239	0.91584	0.91699	0.92064	0.92454	0.92877
505	0.90493	0.91252	0.91598	0.91712	0.92077	0.92467	0.92888
506	0.90508	0.91267	0.91612	0.91725	0.92090	0.92479	0.92899
507	0.90524	0.91281	0.91625	0.91738	0.92103	0.92490	0.92910
508	0.90541	0.91294	0.91638	0.91751	0.92115	0.92502	0.92922
509	0.90554	0.91309	0.91652	0.91765	0.92127	0.92514	0.92933
510	0.90569	0.91323	0.91665	0.91778	0.92140	0.92526	0.92944
511	0.90585	0.91337	0.91678	0.91791	0.92152	0.92537	0.92954
512	0.90598	0.91351	0.91691	0.91804	0.92164	0.92549	0.92966
513	0.90614	0.91364	0.91704	0.91817	0.92177	0.92560	0.92976
514	0.90630	0.91378	0.91717	0.91830	0.92189	0.92572	0.92987
515	0.90646	0.91393	0.91731	0.91843	0.92202	0.92584	0.92998

N	0.1%	0.5%	1%	1.25%	2.5%	5%	10%
516	0.90661	0.91406	0.91743	0.91856	0.92214	0.92595	0.93009
517	0.90677	0.91419	0.91756	0.91869	0.92226	0.92606	0.93020
518	0.90692	0.91433	0.91769	0.91882	0.92238	0.92618	0.93031
519	0.90707	0.91446	0.91783	0.91895	0.92250	0.92629	0.93041
520	0.90722	0.91459	0.91795	0.91907	0.92262	0.92641	0.93052
521	0.90736	0.91472	0.91808	0.91920	0.92274	0.92652	0.93062
522	0.90750	0.91487	0.91821	0.91933	0.92286	0.92664	0.93073
523	0.90765	0.91500	0.91834	0.91945	0.92298	0.92675	0.93084
524	0.90780	0.91513	0.91846	0.91958	0.92310	0.92686	0.93094
525	0.90794	0.91527	0.91859	0.91971	0.92322	0.92697	0.93104
526	0.90807	0.91539	0.91872	0.91983	0.92333	0.92708	0.93115
527	0.90823	0.91552	0.91884	0.91995	0.92345	0.92719	0.93125
528	0.90838	0.91565	0.91897	0.92007	0.92357	0.92730	0.93135
529	0.90853	0.91578	0.91909	0.92020	0.92369	0.92741	0.93146
530	0.90866	0.91591	0.91922	0.92032	0.92380	0.92752	0.93156
531	0.90880	0.91604	0.91934	0.92044	0.92392	0.92763	0.93166
532	0.90893	0.91616	0.91947	0.92057	0.92403	0.92774	0.93176
533	0.90908	0.91629	0.91958	0.92068	0.92415	0.92785	0.93187
534	0.90922	0.91642	0.91971	0.92081	0.92426	0.92796	0.93197
535	0.90936	0.91655	0.91984	0.92093	0.92438	0.92807	0.93207
536	0.90950	0.91668	0.91996	0.92105	0.92449	0.92818	0.93217
537	0.90963	0.91681	0.92009	0.92117	0.92461	0.92829	0.93227
538	0.90978	0.91694	0.92021	0.92129	0.92472	0.92839	0.93237
539	0.90991	0.91706	0.92033	0.92141	0.92484	0.92850	0.93247
540	0.91005	0.91719	0.92045	0.92153	0.92495	0.92861	0.93257
541	0.91019	0.91732	0.92057	0.92165	0.92506	0.92871	0.93267
542	0.91033	0.91744	0.92069	0.92177	0.92518	0.92882	0.93277
543	0.91047	0.91757	0.92081	0.92189	0.92529	0.92892	0.93287
544	0.91062	0.91769	0.92093	0.92201	0.92540	0.92903	0.93296
545	0.91075	0.91782	0.92105	0.92212	0.92551	0.92914	0.93306
546	0.91088	0.91794	0.92117	0.92224	0.92562	0.92924	0.93316
547	0.91102	0.91807	0.92129	0.92235	0.92573	0.92934	0.93326
548	0.91116	0.91819	0.92140	0.92247	0.92584	0.92945	0.93335
549	0.91129	0.91832	0.92152	0.92259	0.92595	0.92955	0.93345
550	0.91142	0.91844	0.92164	0.92270	0.92605	0.92965	0.93355
551	0.91156	0.91856	0.92175	0.92282	0.92617	0.92976	0.93364
552	0.91170	0.91868	0.92187	0.92293	0.92628	0.92986	0.93374
553	0.91185	0.91880	0.92199	0.92304	0.92638	0.92996	0.93384
554	0.91198	0.91892	0.92210	0.92316	0.92649	0.93006	0.93393
555	0.91210	0.91905	0.92221	0.92327	0.92659	0.93016	0.93403
556	0.91223	0.91916	0.92233	0.92337	0.92671	0.93026	0.93412
557	0.91236	0.91928	0.92244	0.92349	0.92681	0.93036	0.93422
558	0.91249	0.91940	0.92256	0.92360	0.92692	0.93046	0.93431
559	0.91263	0.91952	0.92267	0.92371	0.92703	0.93056	0.93440
560	0.91276	0.91964	0.92279	0.92382	0.92713	0.93067	0.93450
561	0.91288	0.91975	0.92290	0.92393	0.92724	0.93076	0.93459
562	0.91302	0.91987	0.92301	0.92405	0.92734	0.93086	0.93468
563	0.91316	0.91999	0.92312	0.92415	0.92744	0.93096	0.93478
564	0.91329	0.92010	0.92322	0.92426	0.92755	0.93106	0.93487
565	0.91342	0.92021	0.92334	0.92437	0.92765	0.93116	0.93496
566	0.91353	0.92033	0.92345	0.92448	0.92776	0.93126	0.93505
567	0.91367	0.92045	0.92356	0.92459	0.92786	0.93135	0.93515
568	0.91378	0.92057	0.92367	0.92470	0.92797	0.93145	0.93524
569	0.91391	0.92068	0.92378	0.92480	0.92807	0.93155	0.93533
570	0.91404	0.92079	0.92388	0.92491	0.92817	0.93165	0.93542
571	0.91416	0.92091	0.92399	0.92502	0.92828	0.93174	0.93551
572	0.91429	0.92102	0.92410	0.92513	0.92838	0.93184	0.93560
573	0.91442	0.92113	0.92421	0.92524	0.92848	0.93193	0.93569
574	0.91454	0.92124	0.92431	0.92534	0.92858	0.93203	0.93578
575	0.91466	0.92135	0.92442	0.92544	0.92868	0.93213	0.93587
576	0.91479	0.92147	0.92453	0.92554	0.92878	0.93222	0.93595
577	0.91491	0.92159	0.92464	0.92565	0.92888	0.93232	0.93605
578	0.91504	0.92170	0.92475	0.92576	0.92898	0.93241	0.93613
579	0.91517	0.92181	0.92486	0.92586	0.92908	0.93250	0.93622
580	0.91528	0.92192	0.92497	0.92597	0.92918	0.93260	0.93631
581	0.91539	0.92203	0.92508	0.92607	0.92928	0.93269	0.93640
582	0.91551	0.92214	0.92518	0.92617	0.92938	0.93278	0.93648
583	0.91564	0.92225	0.92528	0.92628	0.92948	0.93288	0.93657
584	0.91576	0.92235	0.92538	0.92638	0.92958	0.93297	0.93666
585	0.91586	0.92246	0.92549	0.92648	0.92968	0.93306	0.93674
586	0.91598	0.92257	0.92559	0.92659	0.92977	0.93316	0.93683
587	0.91610	0.92269	0.92569	0.92669	0.92987	0.93325	0.93692
588	0.91622	0.92279	0.92580	0.92679	0.92997	0.93334	0.93700
589	0.91633	0.92289	0.92590	0.92689	0.93006	0.93343	0.93709

N	0.1%	0.5%	1%	1.25%	2.5%	5%	10%
590	0.91645	0.92300	0.92600	0.92699	0.93016	0.93352	0.93718
591	0.91657	0.92311	0.92610	0.92709	0.93025	0.93361	0.93726
592	0.91669	0.92321	0.92620	0.92719	0.93035	0.93370	0.93735
593	0.91681	0.92332	0.92631	0.92729	0.93045	0.93379	0.93743
594	0.91693	0.92342	0.92641	0.92739	0.93054	0.93388	0.93751
595	0.91704	0.92353	0.92651	0.92749	0.93064	0.93397	0.93760
596	0.91715	0.92364	0.92661	0.92759	0.93073	0.93406	0.93768
597	0.91726	0.92374	0.92671	0.92769	0.93083	0.93415	0.93777
598	0.91737	0.92385	0.92681	0.92779	0.93092	0.93424	0.93785
599	0.91748	0.92396	0.92691	0.92789	0.93101	0.93432	0.93793
600	0.91761	0.92406	0.92701	0.92799	0.93111	0.93441	0.93801
601	0.91772	0.92417	0.92711	0.92809	0.93120	0.93450	0.93810
602	0.91784	0.92427	0.92721	0.92818	0.93129	0.93459	0.93818
603	0.91795	0.92437	0.92731	0.92828	0.93138	0.93468	0.93826
604	0.91806	0.92447	0.92741	0.92838	0.93148	0.93476	0.93834
605	0.91818	0.92458	0.92751	0.92848	0.93157	0.93485	0.93842
606	0.91830	0.92468	0.92760	0.92857	0.93166	0.93494	0.93851
607	0.91840	0.92478	0.92770	0.92867	0.93175	0.93503	0.93859
608	0.91851	0.92489	0.92780	0.92877	0.93184	0.93512	0.93867
609	0.91862	0.92499	0.92790	0.92886	0.93194	0.93520	0.93875
610	0.91873	0.92509	0.92800	0.92896	0.93203	0.93529	0.93883
611	0.91884	0.92519	0.92809	0.92905	0.93212	0.93537	0.93891
612	0.91896	0.92530	0.92819	0.92915	0.93221	0.93545	0.93899
613	0.91907	0.92540	0.92829	0.92924	0.93229	0.93554	0.93907
614	0.91917	0.92549	0.92838	0.92934	0.93238	0.93562	0.93915
615	0.91928	0.92559	0.92848	0.92943	0.93247	0.93571	0.93923
616	0.91939	0.92569	0.92857	0.92953	0.93256	0.93579	0.93931
617	0.91950	0.92579	0.92867	0.92962	0.93265	0.93588	0.93938
618	0.91961	0.92589	0.92876	0.92971	0.93274	0.93596	0.93946
619	0.91972	0.92599	0.92886	0.92980	0.93283	0.93604	0.93954
620	0.91983	0.92609	0.92895	0.92990	0.93292	0.93613	0.93962
621	0.91993	0.92619	0.92904	0.92999	0.93301	0.93621	0.93970
622	0.92004	0.92629	0.92913	0.93008	0.93310	0.93629	0.93977
623	0.92014	0.92639	0.92923	0.93017	0.93319	0.93638	0.93985
624	0.92025	0.92648	0.92932	0.93026	0.93327	0.93646	0.93993
625	0.92036	0.92658	0.92942	0.93036	0.93336	0.93654	0.94001
626	0.92047	0.92668	0.92951	0.93045	0.93345	0.93662	0.94008
627	0.92058	0.92678	0.92960	0.93054	0.93353	0.93671	0.94016
628	0.92068	0.92687	0.92969	0.93063	0.93362	0.93679	0.94024
629	0.92079	0.92696	0.92978	0.93072	0.93371	0.93687	0.94031
630	0.92091	0.92706	0.92988	0.93080	0.93379	0.93695	0.94039
631	0.92101	0.92716	0.92997	0.93089	0.93388	0.93703	0.94047
632	0.92112	0.92725	0.93006	0.93099	0.93396	0.93711	0.94054
633	0.92122	0.92735	0.93015	0.93108	0.93405	0.93719	0.94062
634	0.92132	0.92744	0.93024	0.93117	0.93413	0.93727	0.94069
635	0.92142	0.92754	0.93033	0.93125	0.93422	0.93735	0.94077
636	0.92153	0.92763	0.93042	0.93134	0.93430	0.93743	0.94084
637	0.92163	0.92772	0.93051	0.93143	0.93439	0.93751	0.94091
638	0.92172	0.92782	0.93060	0.93152	0.93447	0.93759	0.94099
639	0.92184	0.92791	0.93069	0.93160	0.93455	0.93767	0.94106
640	0.92193	0.92801	0.93078	0.93169	0.93464	0.93775	0.94114
641	0.92204	0.92810	0.93086	0.93178	0.93472	0.93783	0.94121
642	0.92214	0.92819	0.93095	0.93187	0.93480	0.93791	0.94129
643	0.92224	0.92828	0.93104	0.93195	0.93488	0.93798	0.94136
644	0.92235	0.92838	0.93113	0.93204	0.93497	0.93806	0.94143
645	0.92244	0.92847	0.93122	0.93212	0.93505	0.93814	0.94151
646	0.92251	0.92856	0.93130	0.93221	0.93513	0.93822	0.94158
647	0.92263	0.92865	0.93139	0.93230	0.93521	0.93830	0.94165
648	0.92274	0.92874	0.93147	0.93239	0.93529	0.93837	0.94172
649	0.92285	0.92884	0.93156	0.93247	0.93537	0.93845	0.94179
650	0.92293	0.92893	0.93164	0.93256	0.93545	0.93853	0.94187
651	0.92302	0.92902	0.93173	0.93264	0.93554	0.93861	0.94194
652	0.92312	0.92911	0.93182	0.93273	0.93562	0.93868	0.94201
653	0.92321	0.92920	0.93191	0.93281	0.93570	0.93876	0.94208
654	0.92332	0.92928	0.93199	0.93290	0.93578	0.93883	0.94215
655	0.92342	0.92938	0.93208	0.93298	0.93586	0.93891	0.94222
656	0.92353	0.92947	0.93216	0.93306	0.93594	0.93898	0.94229
657	0.92361	0.92956	0.93225	0.93315	0.93602	0.93906	0.94236
658	0.92370	0.92964	0.93233	0.93323	0.93610	0.93913	0.94243
659	0.92380	0.92973	0.93242	0.93331	0.93618	0.93921	0.94250
660	0.92392	0.92982	0.93251	0.93340	0.93625	0.93928	0.94257
661	0.92400	0.92990	0.93259	0.93349	0.93634	0.93936	0.94265
662	0.92410	0.92999	0.93267	0.93357	0.93641	0.93943	0.94271
663	0.92419	0.93007	0.93275	0.93365	0.93649	0.93951	0.94278

N	0.1%	0.5%	1%	1.25%	2.5%	5%	10%
664	0.92429	0.93016	0.93284	0.93373	0.93657	0.93958	0.94285
665	0.92438	0.93025	0.93292	0.93381	0.93665	0.93965	0.94292
666	0.92448	0.93033	0.93300	0.93389	0.93672	0.93973	0.94299
667	0.92457	0.93041	0.93308	0.93397	0.93680	0.93980	0.94306
668	0.92468	0.93051	0.93317	0.93405	0.93688	0.93987	0.94313
669	0.92477	0.93059	0.93325	0.93413	0.93696	0.93995	0.94320
670	0.92487	0.93067	0.93334	0.93422	0.93704	0.94002	0.94326
671	0.92495	0.93076	0.93342	0.93430	0.93711	0.94009	0.94333
672	0.92506	0.93084	0.93350	0.93438	0.93719	0.94017	0.94340
673	0.92516	0.93093	0.93358	0.93446	0.93727	0.94024	0.94347
674	0.92524	0.93101	0.93366	0.93454	0.93734	0.94031	0.94353
675	0.92534	0.93109	0.93374	0.93462	0.93742	0.94038	0.94360
676	0.92543	0.93117	0.93382	0.93470	0.93749	0.94045	0.94367
677	0.92552	0.93126	0.93390	0.93478	0.93757	0.94053	0.94374
678	0.92561	0.93135	0.93399	0.93486	0.93765	0.94060	0.94380
679	0.92570	0.93143	0.93407	0.93494	0.93772	0.94067	0.94387
680	0.92579	0.93151	0.93415	0.93502	0.93780	0.94074	0.94394
681	0.92587	0.93160	0.93423	0.93509	0.93787	0.94081	0.94400
682	0.92597	0.93168	0.93431	0.93517	0.93794	0.94088	0.94407
683	0.92607	0.93176	0.93439	0.93525	0.93802	0.94095	0.94413
684	0.92617	0.93185	0.93447	0.93533	0.93809	0.94102	0.94420
685	0.92625	0.93194	0.93455	0.93541	0.93816	0.94109	0.94426
686	0.92633	0.93201	0.93462	0.93548	0.93824	0.94116	0.94433
687	0.92642	0.93209	0.93470	0.93556	0.93831	0.94123	0.94440
688	0.92650	0.93218	0.93478	0.93564	0.93839	0.94130	0.94446
689	0.92658	0.93226	0.93486	0.93572	0.93846	0.94137	0.94452
690	0.92668	0.93234	0.93494	0.93579	0.93853	0.94144	0.94459
691	0.92677	0.93242	0.93501	0.93587	0.93860	0.94151	0.94465
692	0.92686	0.93250	0.93509	0.93595	0.93868	0.94158	0.94472
693	0.92694	0.93259	0.93517	0.93602	0.93875	0.94164	0.94478
694	0.92703	0.93266	0.93525	0.93610	0.93882	0.94171	0.94485
695	0.92711	0.93274	0.93532	0.93617	0.93890	0.94178	0.94491
696	0.92721	0.93283	0.93540	0.93625	0.93897	0.94185	0.94497
697	0.92731	0.93291	0.93548	0.93632	0.93904	0.94191	0.94504
698	0.92738	0.93298	0.93555	0.93640	0.93911	0.94198	0.94510
699	0.92747	0.93306	0.93563	0.93647	0.93918	0.94205	0.94516
700	0.92756	0.93314	0.93571	0.93655	0.93925	0.94212	0.94523
701	0.92765	0.93322	0.93578	0.93662	0.93932	0.94218	0.94529
702	0.92775	0.93330	0.93586	0.93670	0.93939	0.94225	0.94535
703	0.92784	0.93338	0.93593	0.93677	0.93946	0.94232	0.94542
704	0.92792	0.93346	0.93601	0.93685	0.93953	0.94238	0.94548
705	0.92800	0.93353	0.93608	0.93692	0.93960	0.94245	0.94554
706	0.92808	0.93361	0.93616	0.93699	0.93967	0.94252	0.94560
707	0.92817	0.93369	0.93623	0.93707	0.93974	0.94258	0.94567
708	0.92826	0.93377	0.93630	0.93714	0.93981	0.94265	0.94573
709	0.92834	0.93385	0.93638	0.93721	0.93988	0.94271	0.94579
710	0.92843	0.93392	0.93645	0.93729	0.93995	0.94278	0.94585
711	0.92851	0.93400	0.93653	0.93736	0.94002	0.94285	0.94591
712	0.92859	0.93408	0.93660	0.93743	0.94009	0.94291	0.94598
713	0.92868	0.93416	0.93668	0.93750	0.94016	0.94298	0.94604
714	0.92876	0.93423	0.93675	0.93758	0.94023	0.94304	0.94610
715	0.92885	0.93431	0.93682	0.93764	0.94030	0.94311	0.94616
716	0.92892	0.93438	0.93689	0.93772	0.94036	0.94317	0.94622
717	0.92901	0.93446	0.93697	0.93779	0.94043	0.94323	0.94628
718	0.92909	0.93453	0.93704	0.93786	0.94050	0.94330	0.94634
719	0.92917	0.93461	0.93711	0.93793	0.94057	0.94336	0.94640
720	0.92925	0.93469	0.93718	0.93800	0.94063	0.94343	0.94646
721	0.92933	0.93476	0.93725	0.93807	0.94070	0.94349	0.94652
722	0.92941	0.93484	0.93733	0.93814	0.94077	0.94355	0.94658
723	0.92949	0.93492	0.93740	0.93821	0.94084	0.94362	0.94664
724	0.92958	0.93499	0.93747	0.93828	0.94091	0.94368	0.94670
725	0.92967	0.93507	0.93754	0.93835	0.94097	0.94374	0.94676
726	0.92974	0.93514	0.93761	0.93842	0.94104	0.94381	0.94682
727	0.92983	0.93522	0.93768	0.93849	0.94110	0.94387	0.94688
728	0.92991	0.93529	0.93775	0.93856	0.94117	0.94393	0.94694
729	0.93000	0.93536	0.93782	0.93863	0.94124	0.94399	0.94699
730	0.93007	0.93543	0.93789	0.93870	0.94130	0.94406	0.94705
731	0.93015	0.93551	0.93796	0.93877	0.94137	0.94412	0.94711
732	0.93024	0.93558	0.93803	0.93884	0.94144	0.94418	0.94717
733	0.93032	0.93565	0.93810	0.93891	0.94150	0.94424	0.94723
734	0.93040	0.93572	0.93817	0.93898	0.94156	0.94430	0.94729
735	0.93048	0.93580	0.93824	0.93905	0.94163	0.94437	0.94734
736	0.93056	0.93587	0.93831	0.93911	0.94169	0.94443	0.94740
737	0.93065	0.93594	0.93838	0.93918	0.94176	0.94449	0.94746

N	0.1%	0.5%	1%	1.25%	2.5%	5%	10%
738	0.93073	0.93601	0.93845	0.93925	0.94182	0.94455	0.94752
739	0.93079	0.93608	0.93852	0.93932	0.94189	0.94461	0.94758
740	0.93087	0.93615	0.93858	0.93939	0.94195	0.94467	0.94763
741	0.93094	0.93622	0.93865	0.93946	0.94202	0.94473	0.94769
742	0.93102	0.93629	0.93872	0.93952	0.94208	0.94479	0.94775
743	0.93110	0.93636	0.93879	0.93959	0.94214	0.94486	0.94780
744	0.93118	0.93643	0.93886	0.93965	0.94221	0.94492	0.94786
745	0.93126	0.93650	0.93893	0.93972	0.94227	0.94497	0.94792
746	0.93133	0.93657	0.93900	0.93979	0.94234	0.94504	0.94797
747	0.93142	0.93665	0.93907	0.93985	0.94240	0.94509	0.94803
748	0.93149	0.93672	0.93913	0.93992	0.94246	0.94515	0.94808
749	0.93157	0.93679	0.93920	0.93999	0.94253	0.94521	0.94814
750	0.93166	0.93686	0.93927	0.94006	0.94259	0.94527	0.94820
751	0.93173	0.93693	0.93934	0.94012	0.94265	0.94533	0.94825
752	0.93180	0.93700	0.93941	0.94019	0.94271	0.94539	0.94831
753	0.93187	0.93707	0.93947	0.94025	0.94278	0.94545	0.94836
754	0.93195	0.93714	0.93954	0.94032	0.94284	0.94551	0.94842
755	0.93203	0.93721	0.93960	0.94039	0.94290	0.94557	0.94847
756	0.93212	0.93728	0.93967	0.94045	0.94296	0.94563	0.94853
757	0.93219	0.93735	0.93974	0.94051	0.94303	0.94569	0.94858
758	0.93225	0.93742	0.93980	0.94058	0.94309	0.94575	0.94864
759	0.93233	0.93749	0.93987	0.94064	0.94315	0.94580	0.94869
760	0.93240	0.93756	0.93993	0.94070	0.94321	0.94586	0.94875
761	0.93248	0.93762	0.94000	0.94077	0.94327	0.94592	0.94880
762	0.93255	0.93769	0.94006	0.94083	0.94333	0.94598	0.94886
763	0.93263	0.93776	0.94013	0.94090	0.94339	0.94604	0.94891
764	0.93270	0.93783	0.94019	0.94097	0.94345	0.94609	0.94897
765	0.93278	0.93789	0.94026	0.94103	0.94351	0.94615	0.94902
766	0.93286	0.93797	0.94033	0.94109	0.94358	0.94621	0.94907
767	0.93293	0.93804	0.94039	0.94116	0.94364	0.94627	0.94913
768	0.93300	0.93810	0.94045	0.94122	0.94370	0.94632	0.94918
769	0.93308	0.93817	0.94051	0.94128	0.94376	0.94638	0.94923
770	0.93315	0.93824	0.94058	0.94134	0.94382	0.94644	0.94929
771	0.93322	0.93830	0.94064	0.94141	0.94388	0.94650	0.94934
772	0.93329	0.93837	0.94071	0.94147	0.94394	0.94655	0.94939
773	0.93337	0.93844	0.94077	0.94153	0.94400	0.94661	0.94945
774	0.93344	0.93850	0.94083	0.94159	0.94406	0.94666	0.94950
775	0.93351	0.93857	0.94089	0.94166	0.94412	0.94672	0.94955
776	0.93359	0.93863	0.94096	0.94172	0.94418	0.94678	0.94960
777	0.93366	0.93870	0.94102	0.94178	0.94424	0.94683	0.94966
778	0.93372	0.93876	0.94109	0.94184	0.94429	0.94689	0.94971
779	0.93380	0.93883	0.94115	0.94190	0.94435	0.94694	0.94976
780	0.93386	0.93889	0.94121	0.94197	0.94441	0.94700	0.94981
781	0.93394	0.93896	0.94127	0.94203	0.94447	0.94705	0.94987
782	0.93402	0.93903	0.94133	0.94209	0.94453	0.94711	0.94992
783	0.93408	0.93909	0.94139	0.94215	0.94459	0.94716	0.94997
784	0.93415	0.93916	0.94145	0.94221	0.94465	0.94722	0.95002
785	0.93421	0.93922	0.94152	0.94227	0.94470	0.94727	0.95007
786	0.93428	0.93929	0.94158	0.94233	0.94476	0.94733	0.95012
787	0.93435	0.93936	0.94164	0.94239	0.94482	0.94738	0.95018
788	0.93442	0.93942	0.94170	0.94245	0.94488	0.94744	0.95023
789	0.93449	0.93948	0.94176	0.94251	0.94494	0.94749	0.95028
790	0.93455	0.93955	0.94182	0.94257	0.94500	0.94755	0.95033
791	0.93461	0.93961	0.94189	0.94263	0.94505	0.94760	0.95038
792	0.93469	0.93968	0.94195	0.94270	0.94511	0.94766	0.95043
793	0.93476	0.93974	0.94201	0.94275	0.94517	0.94771	0.95048
794	0.93484	0.93980	0.94207	0.94281	0.94522	0.94777	0.95053
795	0.93490	0.93986	0.94213	0.94288	0.94528	0.94782	0.95058
796	0.93497	0.93992	0.94219	0.94293	0.94534	0.94787	0.95063
797	0.93504	0.93998	0.94225	0.94299	0.94539	0.94793	0.95068
798	0.93511	0.94005	0.94231	0.94305	0.94545	0.94798	0.95073
799	0.93518	0.94011	0.94237	0.94311	0.94551	0.94803	0.95078
800	0.93525	0.94017	0.94243	0.94317	0.94557	0.94809	0.95083
801	0.93532	0.94023	0.94249	0.94323	0.94562	0.94814	0.95088
802	0.93538	0.94030	0.94255	0.94329	0.94568	0.94819	0.95093
803	0.93545	0.94036	0.94261	0.94335	0.94573	0.94825	0.95098
804	0.93552	0.94042	0.94267	0.94341	0.94579	0.94830	0.95103
805	0.93559	0.94048	0.94273	0.94347	0.94584	0.94835	0.95108
806	0.93565	0.94054	0.94279	0.94352	0.94590	0.94840	0.95113
807	0.93572	0.94060	0.94284	0.94358	0.94595	0.94846	0.95118
808	0.93579	0.94066	0.94290	0.94364	0.94601	0.94851	0.95123
809	0.93585	0.94073	0.94296	0.94370	0.94606	0.94856	0.95128
810	0.93592	0.94079	0.94302	0.94375	0.94612	0.94861	0.95133
811	0.93597	0.94085	0.94308	0.94381	0.94617	0.94866	0.95137

N	0.1%	0.5%	1%	1.25%	2.5%	5%	10%
812	0.93604	0.94091	0.94313	0.94387	0.94623	0.94872	0.95142
813	0.93612	0.94097	0.94319	0.94392	0.94628	0.94877	0.95147
814	0.93619	0.94103	0.94325	0.94398	0.94634	0.94882	0.95152
815	0.93625	0.94109	0.94331	0.94404	0.94639	0.94887	0.95157
816	0.93632	0.94116	0.94336	0.94409	0.94645	0.94892	0.95162
817	0.93638	0.94122	0.94342	0.94415	0.94650	0.94897	0.95166
818	0.93645	0.94128	0.94348	0.94421	0.94655	0.94902	0.95171
819	0.93651	0.94134	0.94354	0.94426	0.94661	0.94907	0.95176
820	0.93658	0.94140	0.94359	0.94432	0.94666	0.94912	0.95181
821	0.93665	0.94146	0.94365	0.94438	0.94671	0.94917	0.95185
822	0.93672	0.94152	0.94371	0.94443	0.94677	0.94922	0.95190
823	0.93679	0.94157	0.94376	0.94449	0.94682	0.94928	0.95195
824	0.93685	0.94164	0.94382	0.94455	0.94687	0.94933	0.95200
825	0.93692	0.94170	0.94388	0.94460	0.94692	0.94938	0.95204
826	0.93698	0.94176	0.94393	0.94466	0.94698	0.94943	0.95209
827	0.93704	0.94181	0.94399	0.94471	0.94703	0.94948	0.95214
828	0.93711	0.94187	0.94405	0.94477	0.94708	0.94953	0.95219
829	0.93716	0.94193	0.94410	0.94482	0.94714	0.94958	0.95223
830	0.93722	0.94199	0.94416	0.94488	0.94719	0.94963	0.95228
831	0.93729	0.94205	0.94421	0.94493	0.94724	0.94967	0.95233
832	0.93735	0.94211	0.94427	0.94499	0.94729	0.94972	0.95237
833	0.93742	0.94217	0.94432	0.94504	0.94735	0.94978	0.95242
834	0.93748	0.94223	0.94438	0.94510	0.94740	0.94983	0.95246
835	0.93755	0.94229	0.94443	0.94515	0.94745	0.94988	0.95251
836	0.93761	0.94235	0.94449	0.94521	0.94750	0.94992	0.95256
837	0.93766	0.94241	0.94455	0.94526	0.94755	0.94997	0.95260
838	0.93774	0.94246	0.94460	0.94532	0.94760	0.95002	0.95265
839	0.93778	0.94252	0.94466	0.94537	0.94766	0.95007	0.95269
840	0.93785	0.94257	0.94471	0.94543	0.94771	0.95012	0.95274
841	0.93792	0.94263	0.94477	0.94548	0.94776	0.95017	0.95279
842	0.93798	0.94268	0.94482	0.94553	0.94781	0.95022	0.95283
843	0.93804	0.94274	0.94487	0.94559	0.94786	0.95026	0.95288
844	0.93810	0.94280	0.94493	0.94564	0.94791	0.95031	0.95292
845	0.93817	0.94286	0.94498	0.94570	0.94796	0.95036	0.95297
846	0.93823	0.94291	0.94504	0.94575	0.94801	0.95041	0.95301
847	0.93829	0.94297	0.94509	0.94580	0.94806	0.95046	0.95306
848	0.93836	0.94303	0.94515	0.94586	0.94811	0.95051	0.95310
849	0.93841	0.94308	0.94520	0.94591	0.94816	0.95055	0.95315
850	0.93848	0.94314	0.94525	0.94596	0.94821	0.95060	0.95319
851	0.93854	0.94320	0.94531	0.94601	0.94826	0.95065	0.95324
852	0.93860	0.94325	0.94536	0.94607	0.94832	0.95070	0.95328
853	0.93866	0.94331	0.94541	0.94612	0.94837	0.95074	0.95333
854	0.93872	0.94336	0.94547	0.94617	0.94842	0.95079	0.95337
855	0.93878	0.94341	0.94552	0.94622	0.94847	0.95084	0.95341
856	0.93884	0.94347	0.94557	0.94627	0.94852	0.95089	0.95346
857	0.93890	0.94352	0.94562	0.94633	0.94856	0.95093	0.95350
858	0.93895	0.94359	0.94568	0.94638	0.94861	0.95098	0.95355
859	0.93902	0.94364	0.94573	0.94643	0.94866	0.95103	0.95359
860	0.93907	0.94370	0.94578	0.94648	0.94871	0.95107	0.95364
861	0.93913	0.94375	0.94583	0.94653	0.94876	0.95112	0.95368
862	0.93920	0.94380	0.94589	0.94659	0.94881	0.95117	0.95372
863	0.93926	0.94386	0.94594	0.94664	0.94886	0.95121	0.95377
864	0.93931	0.94391	0.94600	0.94669	0.94891	0.95126	0.95381
865	0.93938	0.94396	0.94605	0.94674	0.94896	0.95131	0.95385
866	0.93945	0.94402	0.94610	0.94679	0.94900	0.95135	0.95390
867	0.93951	0.94407	0.94615	0.94684	0.94905	0.95140	0.95394
868	0.93956	0.94412	0.94620	0.94689	0.94910	0.95144	0.95398
869	0.93962	0.94417	0.94625	0.94694	0.94915	0.95149	0.95402
870	0.93968	0.94423	0.94630	0.94699	0.94920	0.95153	0.95407
871	0.93974	0.94428	0.94635	0.94704	0.94925	0.95158	0.95411
872	0.93980	0.94433	0.94641	0.94709	0.94929	0.95163	0.95415
873	0.93986	0.94438	0.94646	0.94714	0.94934	0.95167	0.95420
874	0.93991	0.94444	0.94651	0.94719	0.94939	0.95172	0.95424
875	0.93998	0.94449	0.94656	0.94724	0.94944	0.95176	0.95428
876	0.94004	0.94454	0.94661	0.94729	0.94949	0.95181	0.95432
877	0.94009	0.94459	0.94666	0.94734	0.94953	0.95185	0.95437
878	0.94015	0.94464	0.94671	0.94739	0.94958	0.95190	0.95441
879	0.94021	0.94470	0.94676	0.94744	0.94963	0.95194	0.95445
880	0.94027	0.94475	0.94681	0.94749	0.94968	0.95199	0.95449
881	0.94033	0.94480	0.94686	0.94754	0.94972	0.95203	0.95453
882	0.94039	0.94485	0.94691	0.94759	0.94977	0.95208	0.95458
883	0.94043	0.94491	0.94696	0.94764	0.94982	0.95212	0.95462
884	0.94050	0.94496	0.94701	0.94769	0.94986	0.95217	0.95466
885	0.94055	0.94501	0.94706	0.94774	0.94991	0.95221	0.95470

N	0.1%	0.5%	1%	1.25%	2.5%	5%	10%
886	0.94060	0.94506	0.94711	0.94779	0.94996	0.95225	0.95474
887	0.94066	0.94512	0.94716	0.94784	0.95000	0.95230	0.95478
888	0.94073	0.94517	0.94721	0.94789	0.95005	0.95234	0.95483
889	0.94079	0.94522	0.94726	0.94793	0.95010	0.95239	0.95487
890	0.94084	0.94527	0.94731	0.94798	0.95014	0.95243	0.95491
891	0.94090	0.94533	0.94736	0.94803	0.95019	0.95247	0.95495
892	0.94096	0.94538	0.94741	0.94808	0.95023	0.95252	0.95499
893	0.94101	0.94542	0.94745	0.94813	0.95028	0.95256	0.95503
894	0.94106	0.94547	0.94750	0.94818	0.95033	0.95261	0.95507
895	0.94113	0.94552	0.94755	0.94823	0.95037	0.95265	0.95511
896	0.94118	0.94557	0.94760	0.94828	0.95042	0.95269	0.95515
897	0.94124	0.94562	0.94765	0.94832	0.95046	0.95274	0.95520
898	0.94129	0.94567	0.94770	0.94837	0.95051	0.95278	0.95524
899	0.94135	0.94572	0.94775	0.94842	0.95055	0.95282	0.95528
900	0.94141	0.94578	0.94779	0.94847	0.95060	0.95287	0.95532
901	0.94146	0.94583	0.94784	0.94851	0.95064	0.95291	0.95536
902	0.94152	0.94588	0.94789	0.94856	0.95069	0.95295	0.95540
903	0.94157	0.94593	0.94794	0.94861	0.95074	0.95300	0.95544
904	0.94162	0.94598	0.94799	0.94865	0.95078	0.95304	0.95548
905	0.94168	0.94603	0.94803	0.94870	0.95083	0.95308	0.95552
906	0.94173	0.94608	0.94808	0.94875	0.95087	0.95312	0.95556
907	0.94179	0.94613	0.94813	0.94880	0.95092	0.95316	0.95560
908	0.94185	0.94618	0.94818	0.94884	0.95096	0.95321	0.95564
909	0.94190	0.94622	0.94822	0.94889	0.95101	0.95325	0.95568
910	0.94195	0.94627	0.94827	0.94893	0.95105	0.95329	0.95572
911	0.94200	0.94632	0.94832	0.94898	0.95110	0.95333	0.95576
912	0.94205	0.94637	0.94837	0.94903	0.95114	0.95338	0.95580
913	0.94211	0.94642	0.94842	0.94907	0.95118	0.95342	0.95584
914	0.94216	0.94647	0.94846	0.94912	0.95123	0.95346	0.95588
915	0.94222	0.94652	0.94851	0.94917	0.95127	0.95350	0.95592
916	0.94227	0.94657	0.94856	0.94921	0.95132	0.95354	0.95595
917	0.94232	0.94661	0.94860	0.94926	0.95136	0.95358	0.95599
918	0.94239	0.94666	0.94865	0.94930	0.95141	0.95363	0.95603
919	0.94244	0.94671	0.94870	0.94935	0.95145	0.95367	0.95607
920	0.94249	0.94676	0.94875	0.94940	0.95149	0.95371	0.95611
921	0.94254	0.94681	0.94879	0.94944	0.95154	0.95375	0.95615
922	0.94259	0.94686	0.94884	0.94949	0.95158	0.95379	0.95619
923	0.94265	0.94691	0.94888	0.94954	0.95162	0.95383	0.95623
924	0.94269	0.94695	0.94893	0.94958	0.95167	0.95387	0.95627
925	0.94275	0.94700	0.94898	0.94963	0.95171	0.95391	0.95631
926	0.94280	0.94705	0.94902	0.94967	0.95175	0.95395	0.95634
927	0.94286	0.94709	0.94907	0.94972	0.95179	0.95399	0.95638
928	0.94292	0.94714	0.94911	0.94976	0.95184	0.95404	0.95642
929	0.94297	0.94719	0.94916	0.94981	0.95188	0.95408	0.95646
930	0.94302	0.94724	0.94920	0.94985	0.95192	0.95412	0.95650
931	0.94307	0.94728	0.94925	0.94989	0.95196	0.95416	0.95653
932	0.94312	0.94733	0.94929	0.94994	0.95201	0.95420	0.95657
933	0.94317	0.94738	0.94934	0.94998	0.95205	0.95424	0.95661
934	0.94323	0.94743	0.94938	0.95003	0.95209	0.95428	0.95665
935	0.94328	0.94747	0.94943	0.95007	0.95214	0.95432	0.95669
936	0.94333	0.94752	0.94948	0.95012	0.95218	0.95436	0.95672
937	0.94337	0.94757	0.94952	0.95016	0.95222	0.95440	0.95676
938	0.94343	0.94762	0.94956	0.95020	0.95226	0.95444	0.95680
939	0.94348	0.94766	0.94961	0.95025	0.95231	0.95448	0.95684
940	0.94353	0.94771	0.94965	0.95029	0.95235	0.95452	0.95687
941	0.94359	0.94776	0.94970	0.95034	0.95239	0.95456	0.95691
942	0.94363	0.94781	0.94974	0.95038	0.95243	0.95460	0.95695
943	0.94368	0.94785	0.94979	0.95042	0.95247	0.95464	0.95699
944	0.94373	0.94790	0.94983	0.95047	0.95251	0.95468	0.95702
945	0.94378	0.94795	0.94988	0.95051	0.95256	0.95472	0.95706
946	0.94383	0.94799	0.94992	0.95056	0.95260	0.95476	0.95710
947	0.94389	0.94804	0.94996	0.95060	0.95264	0.95479	0.95713
948	0.94394	0.94808	0.95001	0.95064	0.95268	0.95483	0.95717
949	0.94399	0.94813	0.95005	0.95069	0.95272	0.95487	0.95721
950	0.94404	0.94817	0.95009	0.95073	0.95276	0.95491	0.95724
951	0.94409	0.94822	0.95014	0.95077	0.95280	0.95495	0.95728
952	0.94414	0.94826	0.95018	0.95082	0.95285	0.95499	0.95732
953	0.94419	0.94831	0.95023	0.95086	0.95289	0.95503	0.95736
954	0.94424	0.94835	0.95027	0.95090	0.95293	0.95507	0.95739
955	0.94429	0.94840	0.95031	0.95095	0.95297	0.95511	0.95743
956	0.94433	0.94845	0.95036	0.95099	0.95301	0.95515	0.95747
957	0.94438	0.94849	0.95040	0.95104	0.95305	0.95519	0.95750
958	0.94443	0.94854	0.95045	0.95108	0.95309	0.95523	0.95754
959	0.94447	0.94858	0.95049	0.95112	0.95313	0.95526	0.95757

N	0.1%	0.5%	1%	1.25%	2.5%	5%	10%
960	0.94452	0.94862	0.95053	0.95116	0.95317	0.95530	0.95761
961	0.94456	0.94867	0.95058	0.95121	0.95321	0.95534	0.95765
962	0.94461	0.94871	0.95062	0.95125	0.95325	0.95538	0.95768
963	0.94466	0.94875	0.95066	0.95129	0.95329	0.95542	0.95772
964	0.94471	0.94880	0.95070	0.95133	0.95334	0.95545	0.95775
965	0.94476	0.94884	0.95075	0.95137	0.95337	0.95549	0.95779
966	0.94480	0.94889	0.95079	0.95141	0.95342	0.95553	0.95783
967	0.94485	0.94893	0.95083	0.95146	0.95345	0.95557	0.95786
968	0.94489	0.94898	0.95087	0.95150	0.95349	0.95561	0.95790
969	0.94495	0.94902	0.95092	0.95154	0.95353	0.95564	0.95793
970	0.94499	0.94907	0.95096	0.95158	0.95357	0.95568	0.95797
971	0.94504	0.94911	0.95100	0.95162	0.95361	0.95572	0.95800
972	0.94508	0.94916	0.95104	0.95166	0.95365	0.95576	0.95804
973	0.94512	0.94920	0.95108	0.95170	0.95369	0.95579	0.95807
974	0.94517	0.94924	0.95112	0.95175	0.95373	0.95583	0.95811
975	0.94522	0.94929	0.95116	0.95179	0.95377	0.95587	0.95814
976	0.94526	0.94933	0.95120	0.95183	0.95381	0.95591	0.95818
977	0.94531	0.94937	0.95124	0.95187	0.95385	0.95594	0.95821
978	0.94536	0.94942	0.95129	0.95191	0.95389	0.95598	0.95825
979	0.94540	0.94946	0.95133	0.95195	0.95392	0.95602	0.95828
980	0.94545	0.94951	0.95137	0.95199	0.95396	0.95606	0.95832
981	0.94550	0.94955	0.95141	0.95203	0.95400	0.95609	0.95835
982	0.94554	0.94959	0.95145	0.95207	0.95404	0.95613	0.95839
983	0.94559	0.94964	0.95149	0.95211	0.95408	0.95617	0.95842
984	0.94564	0.94968	0.95154	0.95215	0.95412	0.95620	0.95846
985	0.94569	0.94972	0.95158	0.95220	0.95416	0.95624	0.95849
986	0.94574	0.94977	0.95162	0.95224	0.95420	0.95628	0.95853
987	0.94578	0.94981	0.95166	0.95228	0.95424	0.95631	0.95856
988	0.94583	0.94985	0.95170	0.95232	0.95427	0.95635	0.95860
989	0.94588	0.94989	0.95174	0.95236	0.95431	0.95639	0.95863
990	0.94593	0.94993	0.95178	0.95240	0.95435	0.95642	0.95866
991	0.94597	0.94998	0.95182	0.95244	0.95439	0.95646	0.95870
992	0.94602	0.95002	0.95186	0.95248	0.95443	0.95650	0.95873
993	0.94606	0.95006	0.95190	0.95252	0.95446	0.95653	0.95877
994	0.94611	0.95011	0.95194	0.95256	0.95450	0.95657	0.95880
995	0.94616	0.95015	0.95198	0.95260	0.95454	0.95660	0.95883
996	0.94620	0.95019	0.95202	0.95264	0.95458	0.95664	0.95887
997	0.94624	0.95024	0.95206	0.95268	0.95462	0.95668	0.95890
998	0.94629	0.95028	0.95210	0.95272	0.95465	0.95671	0.95894
999	0.94633	0.95032	0.95214	0.95276	0.95469	0.95675	0.95897
1000	0.94637	0.95036	0.95218	0.95280	0.95473	0.95678	0.95900

Table 2 – *GQ(HHHL or HLLL)*. Critical values of upper significance levels for the Grubbs quadruple outlier test, applicable in situations where the three highest and one lowest or one highest and three lowest observations are simultaneously identified as outliers, denoted as *GQ(HHHL)* and *GQ(HLLL)*, respectively.

N	0.1%	0.5%	1%	1.25%	2.5%	5%	10%
6	0.00000	0.00000	0.00000	0.00001	0.00002	0.00010	0.00042
7	0.00006	0.00031	0.00064	0.00081	0.00166	0.00345	0.00735
8	0.00086	0.00261	0.00426	0.00499	0.00820	0.01362	0.02308
9	0.00354	0.00828	0.01202	0.01356	0.01989	0.02945	0.04436
10	0.00870	0.01732	0.02344	0.02588	0.03538	0.04880	0.06831
11	0.01631	0.02906	0.03759	0.04085	0.05327	0.07002	0.09322
12	0.02619	0.04298	0.05365	0.05775	0.07264	0.09212	0.11813
13	0.03729	0.05816	0.07083	0.07551	0.09257	0.11425	0.14255
14	0.04994	0.07425	0.08861	0.09386	0.11263	0.13612	0.16611
15	0.06343	0.09082	0.10662	0.11237	0.13268	0.15751	0.18873
16	0.07747	0.10767	0.12472	0.13079	0.15225	0.17827	0.21035
17	0.09181	0.12450	0.14252	0.14902	0.17143	0.19816	0.23096
18	0.10634	0.14109	0.15993	0.16668	0.18986	0.21730	0.25057
19	0.12109	0.15746	0.17705	0.18400	0.20773	0.23560	0.26918
20	0.13547	0.17333	0.19361	0.20071	0.22486	0.25316	0.28692
21	0.14969	0.18907	0.20970	0.21694	0.24143	0.27003	0.30378
22	0.16403	0.20438	0.22523	0.23252	0.25743	0.28605	0.31977
23	0.17795	0.21911	0.24025	0.24763	0.27265	0.30140	0.33499
24	0.19184	0.23347	0.25475	0.26221	0.28737	0.31609	0.34961
25	0.20507	0.24739	0.26878	0.27628	0.30154	0.33022	0.36347
26	0.21798	0.26074	0.28242	0.28992	0.31508	0.34364	0.37670
27	0.23044	0.27375	0.29543	0.30292	0.32805	0.35652	0.38928
28	0.24288	0.28624	0.30805	0.31550	0.34061	0.36892	0.40136
29	0.25481	0.29826	0.32013	0.32763	0.35272	0.38083	0.41295
30	0.26639	0.31004	0.33188	0.33940	0.36434	0.39220	0.42402
31	0.27772	0.32149	0.34319	0.35070	0.37554	0.40314	0.43462

N	0.1%	0.5%	1%	1.25%	2.5%	5%	10%
32	0.28842	0.33241	0.35419	0.36165	0.38627	0.41366	0.44484
33	0.29942	0.34317	0.36481	0.37224	0.39662	0.42381	0.45461
34	0.30969	0.35359	0.37505	0.38240	0.40658	0.43353	0.46402
35	0.31998	0.36355	0.38487	0.39220	0.41623	0.44295	0.47309
36	0.32952	0.37308	0.39448	0.40168	0.42558	0.45199	0.48177
37	0.33935	0.38256	0.40369	0.41088	0.43463	0.46073	0.49016
38	0.34868	0.39165	0.41261	0.41968	0.44322	0.46912	0.49828
39	0.35748	0.40037	0.42127	0.42832	0.45163	0.47725	0.50611
40	0.36648	0.40901	0.42964	0.43668	0.45976	0.48516	0.51365
41	0.37511	0.41727	0.43783	0.44476	0.46762	0.49273	0.52092
42	0.38323	0.42543	0.44571	0.45261	0.47531	0.50008	0.52798
43	0.39139	0.43319	0.45332	0.46020	0.48267	0.50720	0.53477
44	0.39919	0.44085	0.46084	0.46753	0.48978	0.51416	0.54136
45	0.40680	0.44829	0.46798	0.47467	0.49676	0.52083	0.54775
46	0.41379	0.45553	0.47501	0.48165	0.50353	0.52733	0.55392
47	0.42114	0.46249	0.48182	0.48843	0.51004	0.53364	0.55992
48	0.42825	0.46925	0.48848	0.49500	0.51642	0.53975	0.56573
49	0.43503	0.47590	0.49492	0.50141	0.52264	0.54569	0.57140
50	0.44204	0.48225	0.50116	0.50766	0.52867	0.55147	0.57687
51	0.44860	0.48848	0.50727	0.51370	0.53451	0.55708	0.58216
52	0.45482	0.49456	0.51320	0.51960	0.54021	0.56258	0.58739
53	0.46117	0.50045	0.51899	0.52527	0.54577	0.56783	0.59240
54	0.46722	0.50627	0.52463	0.53084	0.55110	0.57301	0.59731
55	0.47337	0.51187	0.53015	0.53627	0.55638	0.57805	0.60209
56	0.47898	0.51750	0.53547	0.54159	0.56147	0.58293	0.60676
57	0.48468	0.52291	0.54076	0.54679	0.56643	0.58771	0.61129
58	0.49019	0.52806	0.54584	0.55182	0.57131	0.59238	0.61573
59	0.49568	0.53316	0.55082	0.55680	0.57605	0.59691	0.62000
60	0.50093	0.53824	0.55574	0.56162	0.58066	0.60134	0.62419
61	0.50604	0.54311	0.56046	0.56629	0.58516	0.60565	0.62827
62	0.51130	0.54788	0.56512	0.57088	0.58959	0.60986	0.63226
63	0.51600	0.55257	0.56961	0.57537	0.59392	0.61396	0.63616
64	0.52091	0.55718	0.57407	0.57970	0.59815	0.61799	0.63994
65	0.52557	0.56163	0.57844	0.58401	0.60221	0.62192	0.64366
66	0.53038	0.56590	0.58260	0.58818	0.60624	0.62576	0.64728
67	0.53486	0.57020	0.58669	0.59228	0.61017	0.62948	0.65082
68	0.53930	0.57438	0.59080	0.59628	0.61397	0.63318	0.65430
69	0.54357	0.57844	0.59474	0.60016	0.61774	0.63674	0.65766
70	0.54779	0.58248	0.59867	0.60401	0.62145	0.64026	0.66096
71	0.55215	0.58646	0.60242	0.60779	0.62505	0.64368	0.66422
72	0.55631	0.59022	0.60614	0.61149	0.62858	0.64703	0.66739
73	0.56040	0.59401	0.60984	0.61507	0.63200	0.65032	0.67050
74	0.56419	0.59765	0.61340	0.61859	0.63537	0.65354	0.67353
75	0.56797	0.60133	0.61694	0.62207	0.63872	0.65670	0.67651
76	0.57187	0.60489	0.62035	0.62543	0.64200	0.65979	0.67944
77	0.57572	0.60835	0.62367	0.62872	0.64517	0.66282	0.68229
78	0.57920	0.61170	0.62693	0.63197	0.64826	0.66580	0.68509
79	0.58304	0.61501	0.63011	0.63511	0.65135	0.66872	0.68785
80	0.58648	0.61837	0.63328	0.63821	0.65434	0.67158	0.69054
81	0.59016	0.62162	0.63637	0.64130	0.65727	0.67441	0.69318
82	0.59353	0.62482	0.63938	0.64430	0.66015	0.67713	0.69578
83	0.59682	0.62791	0.64244	0.64725	0.66296	0.67983	0.69833
84	0.60007	0.63090	0.64535	0.65014	0.66575	0.68247	0.70082
85	0.60333	0.63392	0.64824	0.65300	0.66847	0.68507	0.70328
86	0.60646	0.63686	0.65107	0.65581	0.67117	0.68763	0.70568
87	0.60958	0.63979	0.65382	0.65855	0.67382	0.69013	0.70804
88	0.61262	0.64270	0.65664	0.66130	0.67642	0.69260	0.71035
89	0.61566	0.64540	0.65935	0.66398	0.67898	0.69502	0.71263
90	0.61860	0.64815	0.66200	0.66659	0.68146	0.69740	0.71486
91	0.62159	0.65085	0.66458	0.66918	0.68393	0.69975	0.71706
92	0.62433	0.65353	0.66717	0.67170	0.68634	0.70204	0.71922
93	0.62722	0.65618	0.66971	0.67419	0.68872	0.70430	0.72134
94	0.63000	0.65874	0.67212	0.67660	0.69106	0.70651	0.72344
95	0.63267	0.66126	0.67454	0.67898	0.69335	0.70868	0.72549
96	0.63532	0.66379	0.67694	0.68135	0.69561	0.71085	0.72750
97	0.63804	0.66619	0.67930	0.68367	0.69784	0.71295	0.72948
98	0.64059	0.66862	0.68161	0.68595	0.70004	0.71505	0.73144
99	0.64315	0.67099	0.68392	0.68823	0.70222	0.71709	0.73338
100	0.64559	0.67334	0.68619	0.69046	0.70432	0.71910	0.73527
101	0.64799	0.67565	0.68838	0.69264	0.70641	0.72109	0.73714
102	0.65051	0.67792	0.69053	0.69482	0.70847	0.72304	0.73898
103	0.65293	0.68014	0.69270	0.69693	0.71051	0.72498	0.74078
104	0.65532	0.68236	0.69481	0.69899	0.71249	0.72685	0.74256
105	0.65770	0.68451	0.69690	0.70104	0.71447	0.72871	0.74430

N	0.1%	0.5%	1%	1.25%	2.5%	5%	10%
106	0.65996	0.68666	0.69899	0.70310	0.71639	0.73053	0.74602
107	0.66238	0.68875	0.70099	0.70508	0.71829	0.73233	0.74773
108	0.66461	0.69087	0.70302	0.70706	0.72017	0.73414	0.74939
109	0.66683	0.69287	0.70501	0.70898	0.72202	0.73589	0.75105
110	0.66897	0.69491	0.70690	0.71090	0.72383	0.73760	0.75267
111	0.67106	0.69689	0.70882	0.71277	0.72562	0.73931	0.75428
112	0.67321	0.69888	0.71070	0.71460	0.72739	0.74100	0.75586
113	0.67522	0.70080	0.71254	0.71645	0.72912	0.74265	0.75742
114	0.67732	0.70267	0.71438	0.71829	0.73084	0.74428	0.75896
115	0.67924	0.70455	0.71618	0.72006	0.73253	0.74588	0.76045
116	0.68131	0.70640	0.71793	0.72180	0.73420	0.74746	0.76195
117	0.68326	0.70820	0.71974	0.72355	0.73585	0.74902	0.76342
118	0.68518	0.71005	0.72144	0.72525	0.73749	0.75056	0.76486
119	0.68712	0.71183	0.72317	0.72692	0.73911	0.75208	0.76630
120	0.68900	0.71358	0.72485	0.72858	0.74069	0.75357	0.76771
121	0.69096	0.71529	0.72651	0.73024	0.74224	0.75507	0.76910
122	0.69284	0.71704	0.72814	0.73185	0.74380	0.75654	0.77047
123	0.69467	0.71871	0.72975	0.73343	0.74530	0.75798	0.77183
124	0.69645	0.72038	0.73137	0.73502	0.74679	0.75942	0.77317
125	0.69826	0.72206	0.73294	0.73657	0.74830	0.76081	0.77449
126	0.70002	0.72366	0.73452	0.73809	0.74975	0.76221	0.77580
127	0.70165	0.72524	0.73605	0.73962	0.75119	0.76359	0.77708
128	0.70334	0.72682	0.73757	0.74112	0.75262	0.76493	0.77835
129	0.70503	0.72837	0.73907	0.74260	0.75404	0.76626	0.77960
130	0.70663	0.72992	0.74052	0.74404	0.75542	0.76758	0.78082
131	0.70831	0.73144	0.74198	0.74547	0.75680	0.76889	0.78205
132	0.70996	0.73295	0.74341	0.74689	0.75816	0.77016	0.78325
133	0.71149	0.73443	0.74482	0.74830	0.75949	0.77144	0.78444
134	0.71308	0.73590	0.74627	0.74969	0.76082	0.77268	0.78563
135	0.71466	0.73735	0.74765	0.75107	0.76212	0.77392	0.78680
136	0.71632	0.73877	0.74902	0.75243	0.76341	0.77516	0.78795
137	0.71789	0.74017	0.75036	0.75377	0.76469	0.77638	0.78909
138	0.71933	0.74155	0.75173	0.75508	0.76596	0.77758	0.79021
139	0.72084	0.74292	0.75303	0.75639	0.76720	0.77876	0.79131
140	0.72232	0.74430	0.75436	0.75769	0.76844	0.77991	0.79242
141	0.72375	0.74565	0.75565	0.75897	0.76966	0.78107	0.79349
142	0.72524	0.74700	0.75691	0.76021	0.77085	0.78222	0.79456
143	0.72664	0.74832	0.75818	0.76148	0.77204	0.78334	0.79563
144	0.72805	0.74963	0.75946	0.76271	0.77322	0.78445	0.79668
145	0.72944	0.75091	0.76070	0.76394	0.77439	0.78554	0.79771
146	0.73089	0.75220	0.76193	0.76516	0.77554	0.78663	0.79874
147	0.73218	0.75346	0.76315	0.76633	0.77668	0.78771	0.79975
148	0.73357	0.75473	0.76433	0.76750	0.77780	0.78877	0.80074
149	0.73485	0.75594	0.76552	0.76868	0.77892	0.78983	0.80173
150	0.73622	0.75718	0.76668	0.76982	0.78002	0.79087	0.80271
151	0.73753	0.75835	0.76782	0.77096	0.78111	0.79190	0.80368
152	0.73896	0.75957	0.76895	0.77209	0.78218	0.79292	0.80464
153	0.74020	0.76073	0.77008	0.77322	0.78325	0.79394	0.80559
154	0.74146	0.76190	0.77124	0.77434	0.78431	0.79494	0.80653
155	0.74270	0.76306	0.77236	0.77543	0.78536	0.79593	0.80746
156	0.74386	0.76421	0.77343	0.77652	0.78639	0.79691	0.80838
157	0.74512	0.76534	0.77452	0.77757	0.78740	0.79787	0.80929
158	0.74631	0.76645	0.77557	0.77862	0.78841	0.79884	0.81018
159	0.74752	0.76757	0.77665	0.77967	0.78942	0.79978	0.81107
160	0.74864	0.76864	0.77770	0.78071	0.79042	0.80073	0.81196
161	0.74984	0.76971	0.77877	0.78174	0.79139	0.80165	0.81283
162	0.75104	0.77078	0.77979	0.78273	0.79236	0.80257	0.81369
163	0.75217	0.77187	0.78081	0.78376	0.79332	0.80347	0.81453
164	0.75336	0.77293	0.78182	0.78476	0.79426	0.80436	0.81537
165	0.75444	0.77397	0.78280	0.78575	0.79519	0.80526	0.81621
166	0.75558	0.77502	0.78378	0.78673	0.79611	0.80614	0.81703
167	0.75672	0.77600	0.78478	0.78769	0.79704	0.80701	0.81785
168	0.75781	0.77703	0.78573	0.78863	0.79796	0.80788	0.81866
169	0.75889	0.77803	0.78670	0.78958	0.79886	0.80872	0.81947
170	0.75999	0.77903	0.78764	0.79051	0.79975	0.80957	0.82026
171	0.76104	0.78001	0.78859	0.79143	0.80064	0.81041	0.82105
172	0.76211	0.78098	0.78954	0.79237	0.80150	0.81123	0.82183
173	0.76318	0.78195	0.79047	0.79329	0.80238	0.81207	0.82260
174	0.76422	0.78293	0.79141	0.79419	0.80324	0.81288	0.82337
175	0.76529	0.78388	0.79230	0.79510	0.80410	0.81369	0.82412
176	0.76632	0.78481	0.79318	0.79598	0.80494	0.81449	0.82488
177	0.76732	0.78575	0.79408	0.79687	0.80578	0.81528	0.82562
178	0.76834	0.78665	0.79495	0.79773	0.80662	0.81606	0.82635
179	0.76936	0.78758	0.79582	0.79860	0.80744	0.81683	0.82708

N	0.1%	0.5%	1%	1.25%	2.5%	5%	10%
180	0.77033	0.78848	0.79671	0.79945	0.80825	0.81760	0.82780
181	0.77131	0.78935	0.79755	0.80030	0.80905	0.81836	0.82852
182	0.77224	0.79025	0.79839	0.80112	0.80984	0.81912	0.82923
183	0.77324	0.79113	0.79921	0.80194	0.81062	0.81987	0.82993
184	0.77416	0.79201	0.80007	0.80276	0.81140	0.82060	0.83062
185	0.77509	0.79288	0.80091	0.80358	0.81218	0.82134	0.83132
186	0.77607	0.79372	0.80173	0.80438	0.81296	0.82207	0.83200
187	0.77700	0.79456	0.80255	0.80519	0.81373	0.82280	0.83268
188	0.77798	0.79542	0.80336	0.80599	0.81449	0.82352	0.83335
189	0.77893	0.79625	0.80415	0.80680	0.81523	0.82423	0.83401
190	0.77975	0.79707	0.80495	0.80756	0.81597	0.82493	0.83467
191	0.78072	0.79789	0.80573	0.80835	0.81671	0.82563	0.83533
192	0.78158	0.79868	0.80648	0.80910	0.81743	0.82632	0.83597
193	0.78242	0.79948	0.80725	0.80985	0.81816	0.82701	0.83662
194	0.78326	0.80026	0.80803	0.81062	0.81889	0.82768	0.83726
195	0.78415	0.80104	0.80879	0.81136	0.81960	0.82836	0.83789
196	0.78499	0.80185	0.80952	0.81209	0.82030	0.82902	0.83852
197	0.78580	0.80262	0.81029	0.81283	0.82100	0.82969	0.83914
198	0.78667	0.80337	0.81102	0.81356	0.82169	0.83034	0.83976
199	0.78747	0.80413	0.81175	0.81427	0.82238	0.83099	0.84036
200	0.78831	0.80489	0.81248	0.81499	0.82305	0.83165	0.84097
201	0.78911	0.80564	0.81320	0.81571	0.82372	0.83228	0.84158
202	0.78988	0.80638	0.81392	0.81639	0.82439	0.83292	0.84217
203	0.79067	0.80713	0.81461	0.81710	0.82507	0.83355	0.84276
204	0.79147	0.80786	0.81532	0.81780	0.82573	0.83417	0.84335
205	0.79226	0.80861	0.81603	0.81847	0.82638	0.83479	0.84393
206	0.79303	0.80933	0.81672	0.81915	0.82703	0.83542	0.84450
207	0.79380	0.81004	0.81740	0.81983	0.82767	0.83602	0.84507
208	0.79457	0.81076	0.81807	0.82050	0.82830	0.83662	0.84564
209	0.79530	0.81146	0.81875	0.82116	0.82894	0.83721	0.84621
210	0.79611	0.81213	0.81941	0.82182	0.82956	0.83781	0.84677
211	0.79684	0.81281	0.82007	0.82248	0.83019	0.83840	0.84732
212	0.79757	0.81349	0.82073	0.82313	0.83081	0.83898	0.84786
213	0.79828	0.81418	0.82137	0.82376	0.83143	0.83956	0.84841
214	0.79910	0.81487	0.82202	0.82440	0.83202	0.84014	0.84895
215	0.79982	0.81552	0.82266	0.82502	0.83262	0.84071	0.84949
216	0.80057	0.81617	0.82331	0.82567	0.83323	0.84127	0.85002
217	0.80133	0.81684	0.82393	0.82630	0.83382	0.84183	0.85054
218	0.80197	0.81748	0.82456	0.82690	0.83440	0.84239	0.85107
219	0.80270	0.81812	0.82518	0.82752	0.83498	0.84294	0.85159
220	0.80342	0.81879	0.82581	0.82812	0.83556	0.84349	0.85211
221	0.80410	0.81940	0.82641	0.82872	0.83614	0.84403	0.85261
222	0.80475	0.82005	0.82701	0.82932	0.83671	0.84457	0.85312
223	0.80544	0.82068	0.82762	0.82991	0.83727	0.84510	0.85363
224	0.80618	0.82134	0.82820	0.83049	0.83784	0.84564	0.85413
225	0.80686	0.82196	0.82880	0.83108	0.83840	0.84617	0.85462
226	0.80748	0.82257	0.82939	0.83167	0.83895	0.84670	0.85512
227	0.80814	0.82317	0.82998	0.83225	0.83950	0.84722	0.85561
228	0.80879	0.82376	0.83055	0.83283	0.84004	0.84774	0.85610
229	0.80945	0.82436	0.83112	0.83339	0.84058	0.84825	0.85658
230	0.81008	0.82498	0.83170	0.83396	0.84112	0.84876	0.85706
231	0.81069	0.82555	0.83228	0.83452	0.84165	0.84927	0.85753
232	0.81136	0.82613	0.83284	0.83506	0.84219	0.84977	0.85800
233	0.81200	0.82672	0.83339	0.83561	0.84271	0.85026	0.85847
234	0.81263	0.82731	0.83395	0.83615	0.84323	0.85076	0.85894
235	0.81325	0.82787	0.83449	0.83670	0.84374	0.85125	0.85940
236	0.81385	0.82844	0.83504	0.83725	0.84426	0.85174	0.85986
237	0.81451	0.82901	0.83558	0.83777	0.84477	0.85222	0.86030
238	0.81509	0.82955	0.83611	0.83831	0.84527	0.85271	0.86075
239	0.81568	0.83012	0.83664	0.83883	0.84578	0.85318	0.86120
240	0.81626	0.83068	0.83717	0.83935	0.84628	0.85365	0.86164
241	0.81690	0.83124	0.83770	0.83987	0.84677	0.85412	0.86209
242	0.81751	0.83178	0.83823	0.84039	0.84727	0.85459	0.86252
243	0.81811	0.83231	0.83876	0.84090	0.84776	0.85506	0.86296
244	0.81867	0.83285	0.83928	0.84142	0.84824	0.85552	0.86339
245	0.81925	0.83339	0.83979	0.84191	0.84872	0.85598	0.86382
246	0.81981	0.83394	0.84031	0.84242	0.84920	0.85643	0.86425
247	0.82035	0.83446	0.84081	0.84292	0.84968	0.85688	0.86467
248	0.82091	0.83498	0.84132	0.84341	0.85015	0.85732	0.86509
249	0.82149	0.83550	0.84181	0.84391	0.85062	0.85776	0.86551
250	0.82205	0.83602	0.84231	0.84439	0.85109	0.85821	0.86593
251	0.82262	0.83653	0.84280	0.84488	0.85156	0.85865	0.86634
252	0.82316	0.83705	0.84329	0.84536	0.85202	0.85909	0.86675
253	0.82371	0.83757	0.84378	0.84583	0.85248	0.85953	0.86716

N	0.1%	0.5%	1%	1.25%	2.5%	5%	10%
254	0.82426	0.83805	0.84426	0.84630	0.85293	0.85996	0.86756
255	0.82479	0.83855	0.84473	0.84678	0.85339	0.86038	0.86796
256	0.82538	0.83904	0.84521	0.84725	0.85383	0.86081	0.86836
257	0.82589	0.83953	0.84567	0.84772	0.85427	0.86123	0.86876
258	0.82642	0.84002	0.84613	0.84817	0.85471	0.86165	0.86915
259	0.82693	0.84050	0.84660	0.84863	0.85515	0.86207	0.86955
260	0.82746	0.84098	0.84707	0.84910	0.85558	0.86248	0.86994
261	0.82799	0.84146	0.84752	0.84955	0.85601	0.86289	0.87032
262	0.82851	0.84193	0.84798	0.85000	0.85644	0.86330	0.87070
263	0.82903	0.84239	0.84845	0.85045	0.85687	0.86371	0.87108
264	0.82957	0.84287	0.84889	0.85088	0.85730	0.86410	0.87146
265	0.83006	0.84334	0.84935	0.85133	0.85772	0.86450	0.87184
266	0.83058	0.84380	0.84979	0.85178	0.85813	0.86490	0.87221
267	0.83107	0.84427	0.85023	0.85221	0.85855	0.86530	0.87258
268	0.83160	0.84474	0.85068	0.85265	0.85896	0.86568	0.87295
269	0.83208	0.84519	0.85113	0.85308	0.85937	0.86607	0.87331
270	0.83257	0.84564	0.85155	0.85351	0.85978	0.86646	0.87368
271	0.83311	0.84607	0.85198	0.85393	0.86019	0.86685	0.87404
272	0.83358	0.84653	0.85241	0.85436	0.86060	0.86723	0.87440
273	0.83406	0.84698	0.85284	0.85478	0.86099	0.86762	0.87476
274	0.83454	0.84741	0.85327	0.85520	0.86139	0.86800	0.87511
275	0.83503	0.84784	0.85368	0.85561	0.86179	0.86837	0.87547
276	0.83549	0.84830	0.85410	0.85603	0.86218	0.86874	0.87582
277	0.83598	0.84872	0.85452	0.85645	0.86257	0.86912	0.87616
278	0.83647	0.84916	0.85494	0.85686	0.86297	0.86949	0.87651
279	0.83693	0.84959	0.85535	0.85726	0.86335	0.86985	0.87685
280	0.83741	0.85002	0.85575	0.85766	0.86374	0.87022	0.87720
281	0.83789	0.85044	0.85616	0.85807	0.86411	0.87058	0.87754
282	0.83834	0.85085	0.85657	0.85846	0.86449	0.87094	0.87787
283	0.83882	0.85128	0.85698	0.85886	0.86488	0.87130	0.87821
284	0.83932	0.85170	0.85737	0.85925	0.86524	0.87164	0.87854
285	0.83974	0.85210	0.85777	0.85964	0.86561	0.87200	0.87888
286	0.84019	0.85253	0.85817	0.86003	0.86599	0.87235	0.87921
287	0.84063	0.85293	0.85855	0.86042	0.86635	0.87270	0.87953
288	0.84109	0.85334	0.85894	0.86080	0.86671	0.87304	0.87986
289	0.84151	0.85375	0.85934	0.86118	0.86708	0.87339	0.88018
290	0.84194	0.85415	0.85971	0.86156	0.86744	0.87373	0.88050
291	0.84238	0.85456	0.86010	0.86194	0.86780	0.87407	0.88082
292	0.84285	0.85495	0.86048	0.86231	0.86816	0.87441	0.88114
293	0.84324	0.85535	0.86086	0.86268	0.86851	0.87475	0.88145
294	0.84367	0.85574	0.86123	0.86306	0.86887	0.87508	0.88177
295	0.84408	0.85612	0.86160	0.86342	0.86922	0.87541	0.88208
296	0.84450	0.85652	0.86197	0.86379	0.86957	0.87573	0.88239
297	0.84491	0.85692	0.86234	0.86415	0.86992	0.87606	0.88270
298	0.84532	0.85730	0.86271	0.86451	0.87027	0.87639	0.88301
299	0.84576	0.85768	0.86308	0.86487	0.87061	0.87671	0.88332
300	0.84615	0.85806	0.86344	0.86522	0.87095	0.87704	0.88362
301	0.84657	0.85843	0.86381	0.86559	0.87129	0.87735	0.88393
302	0.84700	0.85882	0.86416	0.86595	0.87162	0.87767	0.88422
303	0.84739	0.85918	0.86451	0.86630	0.87196	0.87800	0.88452
304	0.84782	0.85955	0.86487	0.86664	0.87229	0.87831	0.88482
305	0.84820	0.85992	0.86522	0.86698	0.87262	0.87863	0.88511
306	0.84861	0.86029	0.86558	0.86733	0.87295	0.87894	0.88541
307	0.84902	0.86065	0.86593	0.86767	0.87328	0.87925	0.88570
308	0.84940	0.86101	0.86627	0.86801	0.87361	0.87956	0.88599
309	0.84977	0.86137	0.86661	0.86835	0.87393	0.87987	0.88628
310	0.85017	0.86171	0.86696	0.86868	0.87425	0.88018	0.88657
311	0.85054	0.86207	0.86730	0.86901	0.87457	0.88048	0.88685
312	0.85093	0.86242	0.86764	0.86935	0.87489	0.88078	0.88714
313	0.85128	0.86277	0.86797	0.86969	0.87521	0.88108	0.88742
314	0.85164	0.86312	0.86832	0.87002	0.87553	0.88138	0.88770
315	0.85204	0.86347	0.86865	0.87034	0.87584	0.88167	0.88798
316	0.85241	0.86382	0.86898	0.87067	0.87615	0.88197	0.88826
317	0.85278	0.86417	0.86930	0.87099	0.87645	0.88226	0.88853
318	0.85316	0.86451	0.86964	0.87132	0.87676	0.88255	0.88881
319	0.85355	0.86485	0.86997	0.87164	0.87706	0.88284	0.88908
320	0.85393	0.86519	0.87030	0.87197	0.87737	0.88312	0.88935
321	0.85434	0.86553	0.87062	0.87229	0.87768	0.88341	0.88962
322	0.85468	0.86587	0.87094	0.87261	0.87797	0.88370	0.88989
323	0.85507	0.86619	0.87125	0.87292	0.87827	0.88398	0.89016
324	0.85542	0.86652	0.87157	0.87323	0.87857	0.88426	0.89042
325	0.85576	0.86685	0.87189	0.87354	0.87887	0.88454	0.89069
326	0.85609	0.86716	0.87220	0.87386	0.87916	0.88482	0.89095
327	0.85645	0.86749	0.87251	0.87417	0.87946	0.88510	0.89121

N	0.1%	0.5%	1%	1.25%	2.5%	5%	10%
328	0.85679	0.86782	0.87281	0.87447	0.87975	0.88538	0.89147
329	0.85717	0.86814	0.87311	0.87477	0.88004	0.88565	0.89173
330	0.85748	0.86845	0.87342	0.87508	0.88033	0.88593	0.89198
331	0.85785	0.86877	0.87373	0.87537	0.88062	0.88620	0.89224
332	0.85817	0.86909	0.87403	0.87568	0.88090	0.88648	0.89249
333	0.85852	0.86941	0.87433	0.87598	0.88119	0.88675	0.89275
334	0.85886	0.86972	0.87464	0.87627	0.88147	0.88701	0.89300
335	0.85920	0.87003	0.87494	0.87657	0.88176	0.88728	0.89325
336	0.85953	0.87034	0.87524	0.87686	0.88204	0.88755	0.89350
337	0.85988	0.87065	0.87554	0.87715	0.88232	0.88781	0.89375
338	0.86021	0.87095	0.87583	0.87745	0.88260	0.88807	0.89400
339	0.86055	0.87125	0.87612	0.87773	0.88287	0.88833	0.89424
340	0.86091	0.87156	0.87641	0.87801	0.88314	0.88859	0.89449
341	0.86125	0.87187	0.87671	0.87830	0.88342	0.88885	0.89474
342	0.86159	0.87218	0.87699	0.87858	0.88369	0.88911	0.89498
343	0.86192	0.87248	0.87728	0.87887	0.88396	0.88937	0.89522
344	0.86224	0.87277	0.87756	0.87914	0.88423	0.88962	0.89546
345	0.86256	0.87307	0.87785	0.87943	0.88450	0.88988	0.89570
346	0.86289	0.87336	0.87813	0.87971	0.88477	0.89013	0.89594
347	0.86317	0.87366	0.87841	0.87998	0.88504	0.89038	0.89617
348	0.86348	0.87395	0.87870	0.88026	0.88530	0.89063	0.89641
349	0.86381	0.87425	0.87898	0.88053	0.88556	0.89088	0.89664
350	0.86412	0.87452	0.87926	0.88080	0.88582	0.89113	0.89687
351	0.86445	0.87481	0.87954	0.88108	0.88608	0.89138	0.89711
352	0.86474	0.87510	0.87981	0.88135	0.88634	0.89162	0.89734
353	0.86507	0.87539	0.88009	0.88163	0.88660	0.89187	0.89756
354	0.86538	0.87567	0.88035	0.88189	0.88685	0.89211	0.89779
355	0.86569	0.87596	0.88063	0.88216	0.88711	0.89235	0.89802
356	0.86598	0.87624	0.88090	0.88243	0.88736	0.89259	0.89825
357	0.86627	0.87652	0.88117	0.88269	0.88762	0.89283	0.89847
358	0.86656	0.87680	0.88144	0.88296	0.88787	0.89307	0.89869
359	0.86687	0.87708	0.88170	0.88322	0.88812	0.89330	0.89892
360	0.86719	0.87735	0.88196	0.88348	0.88837	0.89354	0.89914
361	0.86748	0.87762	0.88223	0.88374	0.88862	0.89378	0.89936
362	0.86779	0.87789	0.88249	0.88399	0.88886	0.89401	0.89958
363	0.86810	0.87816	0.88276	0.88426	0.88911	0.89424	0.89980
364	0.86839	0.87844	0.88301	0.88451	0.88936	0.89447	0.90002
365	0.86865	0.87870	0.88327	0.88477	0.88960	0.89470	0.90023
366	0.86895	0.87897	0.88353	0.88503	0.88984	0.89493	0.90045
367	0.86924	0.87924	0.88378	0.88528	0.89008	0.89516	0.90066
368	0.86952	0.87949	0.88403	0.88553	0.89032	0.89538	0.90088
369	0.86981	0.87976	0.88429	0.88578	0.89056	0.89561	0.90109
370	0.87010	0.88002	0.88454	0.88602	0.89079	0.89583	0.90130
371	0.87036	0.88029	0.88479	0.88628	0.89103	0.89606	0.90151
372	0.87065	0.88055	0.88505	0.88652	0.89126	0.89628	0.90172
373	0.87093	0.88081	0.88529	0.88677	0.89149	0.89650	0.90193
374	0.87122	0.88107	0.88553	0.88702	0.89173	0.89672	0.90214
375	0.87151	0.88132	0.88578	0.88726	0.89195	0.89694	0.90234
376	0.87177	0.88158	0.88603	0.88749	0.89218	0.89715	0.90255
377	0.87206	0.88184	0.88627	0.88773	0.89241	0.89737	0.90275
378	0.87234	0.88208	0.88651	0.88797	0.89264	0.89759	0.90296
379	0.87262	0.88234	0.88676	0.88820	0.89287	0.89781	0.90316
380	0.87289	0.88258	0.88699	0.88844	0.89310	0.89802	0.90336
381	0.87315	0.88284	0.88723	0.88868	0.89332	0.89824	0.90356
382	0.87341	0.88308	0.88747	0.88891	0.89355	0.89845	0.90376
383	0.87368	0.88333	0.88770	0.88915	0.89376	0.89866	0.90396
384	0.87395	0.88357	0.88794	0.88938	0.89398	0.89888	0.90416
385	0.87423	0.88382	0.88817	0.88961	0.89421	0.89908	0.90436
386	0.87450	0.88407	0.88841	0.88984	0.89443	0.89929	0.90455
387	0.87477	0.88431	0.88864	0.89007	0.89465	0.89950	0.90475
388	0.87504	0.88455	0.88887	0.89030	0.89487	0.89971	0.90494
389	0.87532	0.88480	0.88910	0.89053	0.89509	0.89991	0.90514
390	0.87558	0.88504	0.88933	0.89075	0.89531	0.90012	0.90533
391	0.87585	0.88527	0.88955	0.89098	0.89552	0.90032	0.90552
392	0.87609	0.88551	0.88978	0.89120	0.89574	0.90052	0.90571
393	0.87635	0.88574	0.89001	0.89142	0.89595	0.90073	0.90590
394	0.87663	0.88597	0.89023	0.89165	0.89617	0.90093	0.90609
395	0.87686	0.88621	0.89047	0.89187	0.89638	0.90113	0.90628
396	0.87710	0.88644	0.89069	0.89209	0.89659	0.90133	0.90647
397	0.87735	0.88667	0.89091	0.89231	0.89680	0.90153	0.90665
398	0.87761	0.88690	0.89114	0.89253	0.89700	0.90173	0.90684
399	0.87788	0.88713	0.89136	0.89275	0.89721	0.90192	0.90702
400	0.87813	0.88736	0.89158	0.89297	0.89742	0.90212	0.90720
401	0.87837	0.88758	0.89180	0.89319	0.89762	0.90231	0.90739

N	0.1%	0.5%	1%	1.25%	2.5%	5%	10%
402	0.87862	0.88781	0.89202	0.89340	0.89783	0.90251	0.90757
403	0.87889	0.88804	0.89224	0.89362	0.89803	0.90270	0.90776
404	0.87912	0.88826	0.89245	0.89383	0.89824	0.90289	0.90794
405	0.87936	0.88849	0.89266	0.89404	0.89844	0.90308	0.90812
406	0.87960	0.88871	0.89287	0.89425	0.89864	0.90328	0.90830
407	0.87984	0.88894	0.89309	0.89446	0.89884	0.90346	0.90848
408	0.88009	0.88917	0.89331	0.89468	0.89904	0.90365	0.90866
409	0.88032	0.88939	0.89352	0.89488	0.89924	0.90384	0.90883
410	0.88055	0.88960	0.89373	0.89509	0.89944	0.90403	0.90901
411	0.88079	0.88982	0.89394	0.89530	0.89963	0.90422	0.90919
412	0.88105	0.89004	0.89414	0.89551	0.89983	0.90440	0.90936
413	0.88127	0.89026	0.89435	0.89571	0.90002	0.90459	0.90953
414	0.88151	0.89047	0.89456	0.89591	0.90022	0.90477	0.90971
415	0.88174	0.89068	0.89477	0.89611	0.90041	0.90496	0.90988
416	0.88198	0.89090	0.89497	0.89631	0.90060	0.90514	0.91005
417	0.88220	0.89112	0.89518	0.89652	0.90080	0.90532	0.91022
418	0.88243	0.89133	0.89539	0.89672	0.90099	0.90550	0.91040
419	0.88265	0.89154	0.89559	0.89692	0.90118	0.90569	0.91057
420	0.88290	0.89175	0.89579	0.89712	0.90137	0.90587	0.91074
421	0.88314	0.89196	0.89599	0.89732	0.90156	0.90604	0.91091
422	0.88336	0.89217	0.89619	0.89752	0.90175	0.90622	0.91108
423	0.88359	0.89239	0.89639	0.89771	0.90193	0.90640	0.91124
424	0.88380	0.89259	0.89658	0.89790	0.90212	0.90658	0.91141
425	0.88400	0.89280	0.89678	0.89810	0.90230	0.90675	0.91158
426	0.88424	0.89301	0.89698	0.89830	0.90249	0.90693	0.91174
427	0.88448	0.89321	0.89717	0.89849	0.90267	0.90711	0.91191
428	0.88469	0.89341	0.89737	0.89868	0.90285	0.90728	0.91207
429	0.88490	0.89361	0.89757	0.89887	0.90304	0.90745	0.91224
430	0.88511	0.89382	0.89776	0.89906	0.90322	0.90762	0.91240
431	0.88531	0.89401	0.89795	0.89925	0.90339	0.90780	0.91256
432	0.88555	0.89422	0.89814	0.89943	0.90358	0.90797	0.91272
433	0.88578	0.89441	0.89833	0.89963	0.90376	0.90814	0.91288
434	0.88599	0.89462	0.89852	0.89981	0.90393	0.90831	0.91304
435	0.88621	0.89482	0.89871	0.90000	0.90411	0.90848	0.91320
436	0.88644	0.89502	0.89891	0.90019	0.90430	0.90865	0.91336
437	0.88664	0.89522	0.89909	0.90038	0.90447	0.90881	0.91352
438	0.88686	0.89542	0.89928	0.90056	0.90464	0.90898	0.91368
439	0.88708	0.89561	0.89947	0.90075	0.90482	0.90915	0.91383
440	0.88728	0.89579	0.89965	0.90093	0.90500	0.90931	0.91399
441	0.88749	0.89598	0.89984	0.90112	0.90517	0.90948	0.91414
442	0.88770	0.89617	0.90003	0.90131	0.90535	0.90964	0.91430
443	0.88791	0.89636	0.90021	0.90149	0.90552	0.90981	0.91446
444	0.88810	0.89655	0.90039	0.90167	0.90569	0.90997	0.91461
445	0.88831	0.89675	0.90058	0.90185	0.90586	0.91013	0.91476
446	0.88852	0.89695	0.90075	0.90203	0.90603	0.91030	0.91492
447	0.88873	0.89713	0.90094	0.90221	0.90620	0.91046	0.91507
448	0.88893	0.89732	0.90112	0.90238	0.90637	0.91062	0.91522
449	0.88914	0.89751	0.90130	0.90256	0.90654	0.91079	0.91537
450	0.88935	0.89769	0.90147	0.90273	0.90671	0.91095	0.91552
451	0.88956	0.89788	0.90165	0.90290	0.90687	0.91110	0.91567
452	0.88974	0.89806	0.90183	0.90308	0.90704	0.91126	0.91582
453	0.88995	0.89825	0.90201	0.90325	0.90721	0.91142	0.91596
454	0.89016	0.89843	0.90219	0.90342	0.90738	0.91158	0.91611
455	0.89035	0.89861	0.90236	0.90360	0.90754	0.91173	0.91626
456	0.89056	0.89880	0.90254	0.90377	0.90770	0.91189	0.91641
457	0.89074	0.89899	0.90271	0.90395	0.90787	0.91204	0.91655
458	0.89095	0.89916	0.90288	0.90412	0.90803	0.91220	0.91670
459	0.89115	0.89934	0.90305	0.90429	0.90819	0.91235	0.91684
460	0.89134	0.89952	0.90322	0.90446	0.90836	0.91251	0.91699
461	0.89153	0.89970	0.90340	0.90462	0.90852	0.91266	0.91713
462	0.89172	0.89987	0.90356	0.90479	0.90868	0.91281	0.91727
463	0.89191	0.90005	0.90373	0.90495	0.90884	0.91296	0.91742
464	0.89210	0.90022	0.90390	0.90512	0.90900	0.91311	0.91756
465	0.89229	0.90041	0.90407	0.90529	0.90916	0.91326	0.91770
466	0.89248	0.90058	0.90424	0.90546	0.90931	0.91341	0.91784
467	0.89267	0.90076	0.90440	0.90562	0.90947	0.91356	0.91799
468	0.89288	0.90093	0.90457	0.90578	0.90963	0.91371	0.91813
469	0.89306	0.90110	0.90473	0.90594	0.90978	0.91386	0.91827
470	0.89325	0.90127	0.90490	0.90611	0.90994	0.91400	0.91841
471	0.89347	0.90144	0.90506	0.90627	0.91009	0.91415	0.91855
472	0.89364	0.90162	0.90523	0.90644	0.91025	0.91430	0.91868
473	0.89381	0.90179	0.90539	0.90660	0.91040	0.91444	0.91882
474	0.89398	0.90197	0.90556	0.90676	0.91055	0.91459	0.91896
475	0.89416	0.90213	0.90572	0.90692	0.91071	0.91473	0.91909

N	0.1%	0.5%	1%	1.25%	2.5%	5%	10%
476	0.89434	0.90230	0.90588	0.90708	0.91086	0.91488	0.91923
477	0.89453	0.90245	0.90604	0.90723	0.91101	0.91502	0.91937
478	0.89472	0.90263	0.90620	0.90739	0.91116	0.91517	0.91950
479	0.89491	0.90279	0.90636	0.90755	0.91131	0.91531	0.91963
480	0.89509	0.90296	0.90652	0.90771	0.91146	0.91545	0.91977
481	0.89526	0.90312	0.90668	0.90787	0.91162	0.91560	0.91990
482	0.89543	0.90329	0.90683	0.90802	0.91176	0.91574	0.92004
483	0.89562	0.90345	0.90700	0.90817	0.91191	0.91588	0.92017
484	0.89579	0.90362	0.90715	0.90833	0.91206	0.91602	0.92030
485	0.89599	0.90378	0.90730	0.90848	0.91220	0.91616	0.92043
486	0.89615	0.90394	0.90747	0.90864	0.91235	0.91630	0.92057
487	0.89634	0.90411	0.90762	0.90879	0.91250	0.91644	0.92070
488	0.89651	0.90426	0.90777	0.90895	0.91264	0.91658	0.92083
489	0.89668	0.90443	0.90793	0.90909	0.91279	0.91671	0.92096
490	0.89686	0.90459	0.90808	0.90925	0.91293	0.91685	0.92109
491	0.89702	0.90474	0.90823	0.90940	0.91308	0.91699	0.92121
492	0.89721	0.90491	0.90838	0.90955	0.91322	0.91713	0.92134
493	0.89739	0.90507	0.90854	0.90969	0.91336	0.91726	0.92147
494	0.89757	0.90522	0.90869	0.90985	0.91350	0.91740	0.92160
495	0.89773	0.90538	0.90884	0.90999	0.91365	0.91753	0.92173
496	0.89792	0.90555	0.90899	0.91014	0.91379	0.91767	0.92185
497	0.89806	0.90570	0.90915	0.91029	0.91393	0.91780	0.92198
498	0.89823	0.90586	0.90929	0.91043	0.91407	0.91793	0.92210
499	0.89841	0.90601	0.90944	0.91058	0.91421	0.91807	0.92223
500	0.89858	0.90617	0.90959	0.91073	0.91435	0.91820	0.92235
501	0.89875	0.90632	0.90973	0.91087	0.91449	0.91833	0.92248
502	0.89890	0.90648	0.90988	0.91102	0.91463	0.91846	0.92260
503	0.89909	0.90663	0.91003	0.91117	0.91477	0.91859	0.92272
504	0.89926	0.90678	0.91018	0.91131	0.91491	0.91873	0.92285
505	0.89943	0.90694	0.91033	0.91145	0.91504	0.91886	0.92297
506	0.89960	0.90708	0.91047	0.91160	0.91518	0.91899	0.92309
507	0.89976	0.90724	0.91061	0.91174	0.91532	0.91912	0.92321
508	0.89991	0.90738	0.91076	0.91189	0.91546	0.91925	0.92334
509	0.90008	0.90754	0.91090	0.91203	0.91559	0.91938	0.92346
510	0.90025	0.90769	0.91105	0.91217	0.91573	0.91950	0.92358
511	0.90041	0.90784	0.91119	0.91231	0.91586	0.91963	0.92370
512	0.90056	0.90798	0.91134	0.91244	0.91600	0.91976	0.92382
513	0.90071	0.90813	0.91148	0.91258	0.91613	0.91988	0.92393
514	0.90089	0.90827	0.91162	0.91271	0.91626	0.92001	0.92406
515	0.90104	0.90842	0.91176	0.91286	0.91640	0.92013	0.92418
516	0.90121	0.90857	0.91190	0.91300	0.91653	0.92026	0.92429
517	0.90136	0.90871	0.91204	0.91314	0.91666	0.92038	0.92441
518	0.90149	0.90886	0.91218	0.91327	0.91679	0.92051	0.92453
519	0.90167	0.90900	0.91232	0.91341	0.91693	0.92063	0.92465
520	0.90182	0.90914	0.91245	0.91355	0.91705	0.92076	0.92476
521	0.90198	0.90929	0.91259	0.91369	0.91718	0.92088	0.92488
522	0.90214	0.90943	0.91273	0.91383	0.91731	0.92100	0.92499
523	0.90230	0.90957	0.91287	0.91396	0.91744	0.92112	0.92511
524	0.90245	0.90971	0.91301	0.91410	0.91757	0.92125	0.92522
525	0.90260	0.90986	0.91314	0.91423	0.91770	0.92137	0.92534
526	0.90275	0.91000	0.91328	0.91436	0.91782	0.92149	0.92545
527	0.90290	0.91014	0.91342	0.91449	0.91795	0.92161	0.92557
528	0.90306	0.91028	0.91355	0.91463	0.91807	0.92173	0.92568
529	0.90321	0.91042	0.91368	0.91476	0.91820	0.92185	0.92580
530	0.90336	0.91056	0.91381	0.91489	0.91832	0.92197	0.92591
531	0.90352	0.91070	0.91394	0.91502	0.91845	0.92209	0.92602
532	0.90367	0.91084	0.91408	0.91515	0.91858	0.92221	0.92613
533	0.90382	0.91098	0.91421	0.91528	0.91870	0.92232	0.92624
534	0.90397	0.91112	0.91434	0.91542	0.91883	0.92245	0.92636
535	0.90411	0.91125	0.91447	0.91555	0.91896	0.92256	0.92647
536	0.90426	0.91139	0.91461	0.91568	0.91908	0.92268	0.92658
537	0.90442	0.91153	0.91474	0.91581	0.91920	0.92279	0.92669
538	0.90457	0.91167	0.91487	0.91593	0.91932	0.92291	0.92680
539	0.90473	0.91180	0.91500	0.91606	0.91944	0.92303	0.92691
540	0.90488	0.91193	0.91512	0.91619	0.91957	0.92314	0.92702
541	0.90503	0.91207	0.91525	0.91632	0.91969	0.92326	0.92713
542	0.90517	0.91219	0.91538	0.91645	0.91981	0.92337	0.92723
543	0.90532	0.91233	0.91551	0.91657	0.91993	0.92349	0.92734
544	0.90546	0.91246	0.91564	0.91670	0.92005	0.92360	0.92745
545	0.90561	0.91260	0.91577	0.91683	0.92017	0.92372	0.92756
546	0.90575	0.91273	0.91590	0.91695	0.92029	0.92383	0.92767
547	0.90589	0.91286	0.91602	0.91707	0.92041	0.92394	0.92777
548	0.90603	0.91298	0.91615	0.91720	0.92053	0.92406	0.92788
549	0.90618	0.91312	0.91628	0.91732	0.92065	0.92417	0.92799

N	0.1%	0.5%	1%	1.25%	2.5%	5%	10%
550	0.90633	0.91325	0.91640	0.91744	0.92076	0.92428	0.92809
551	0.90647	0.91338	0.91653	0.91757	0.92088	0.92439	0.92820
552	0.90661	0.91351	0.91665	0.91769	0.92100	0.92451	0.92830
553	0.90674	0.91364	0.91678	0.91781	0.92111	0.92462	0.92841
554	0.90687	0.91377	0.91690	0.91793	0.92123	0.92473	0.92851
555	0.90701	0.91390	0.91703	0.91806	0.92135	0.92484	0.92861
556	0.90715	0.91403	0.91715	0.91818	0.92147	0.92495	0.92872
557	0.90730	0.91416	0.91727	0.91830	0.92158	0.92506	0.92882
558	0.90744	0.91428	0.91739	0.91842	0.92170	0.92517	0.92893
559	0.90759	0.91441	0.91751	0.91854	0.92181	0.92528	0.92903
560	0.90774	0.91454	0.91764	0.91866	0.92192	0.92538	0.92913
561	0.90788	0.91467	0.91775	0.91878	0.92204	0.92549	0.92924
562	0.90800	0.91479	0.91787	0.91890	0.92215	0.92560	0.92934
563	0.90814	0.91493	0.91800	0.91902	0.92226	0.92571	0.92944
564	0.90828	0.91505	0.91812	0.91914	0.92237	0.92581	0.92954
565	0.90842	0.91516	0.91823	0.91925	0.92249	0.92592	0.92964
566	0.90856	0.91529	0.91835	0.91937	0.92260	0.92603	0.92974
567	0.90869	0.91541	0.91847	0.91948	0.92271	0.92614	0.92984
568	0.90882	0.91553	0.91859	0.91960	0.92282	0.92624	0.92994
569	0.90897	0.91566	0.91871	0.91972	0.92294	0.92635	0.93004
570	0.90910	0.91578	0.91883	0.91983	0.92305	0.92645	0.93014
571	0.90923	0.91590	0.91895	0.91995	0.92316	0.92656	0.93024
572	0.90937	0.91603	0.91906	0.92006	0.92327	0.92666	0.93034
573	0.90951	0.91615	0.91918	0.92018	0.92338	0.92677	0.93044
574	0.90962	0.91627	0.91930	0.92030	0.92349	0.92687	0.93053
575	0.90976	0.91639	0.91940	0.92041	0.92359	0.92697	0.93063
576	0.90989	0.91651	0.91952	0.92053	0.92370	0.92708	0.93073
577	0.91001	0.91662	0.91964	0.92064	0.92381	0.92718	0.93083
578	0.91014	0.91675	0.91975	0.92075	0.92392	0.92728	0.93092
579	0.91026	0.91686	0.91987	0.92086	0.92402	0.92739	0.93102
580	0.91038	0.91698	0.91998	0.92098	0.92413	0.92749	0.93112
581	0.91052	0.91710	0.92009	0.92109	0.92424	0.92759	0.93121
582	0.91065	0.91722	0.92021	0.92120	0.92435	0.92769	0.93131
583	0.91078	0.91734	0.92032	0.92131	0.92445	0.92779	0.93140
584	0.91092	0.91746	0.92043	0.92142	0.92456	0.92789	0.93150
585	0.91105	0.91758	0.92055	0.92153	0.92466	0.92799	0.93159
586	0.91117	0.91770	0.92066	0.92164	0.92477	0.92809	0.93168
587	0.91129	0.91782	0.92077	0.92175	0.92487	0.92819	0.93178
588	0.91143	0.91793	0.92089	0.92186	0.92498	0.92829	0.93187
589	0.91155	0.91804	0.92099	0.92197	0.92508	0.92839	0.93196
590	0.91168	0.91816	0.92110	0.92208	0.92519	0.92849	0.93206
591	0.91181	0.91828	0.92122	0.92219	0.92529	0.92859	0.93215
592	0.91192	0.91839	0.92133	0.92230	0.92539	0.92869	0.93225
593	0.91205	0.91850	0.92144	0.92241	0.92550	0.92879	0.93234
594	0.91217	0.91861	0.92154	0.92252	0.92560	0.92888	0.93243
595	0.91230	0.91872	0.92166	0.92262	0.92570	0.92898	0.93252
596	0.91242	0.91884	0.92176	0.92273	0.92581	0.92908	0.93261
597	0.91252	0.91895	0.92187	0.92283	0.92591	0.92917	0.93271
598	0.91265	0.91906	0.92198	0.92294	0.92601	0.92927	0.93280
599	0.91277	0.91917	0.92209	0.92305	0.92611	0.92937	0.93289
600	0.91289	0.91929	0.92220	0.92315	0.92621	0.92946	0.93298
601	0.91303	0.91940	0.92230	0.92326	0.92631	0.92956	0.93307
602	0.91313	0.91951	0.92241	0.92336	0.92641	0.92965	0.93316
603	0.91325	0.91962	0.92252	0.92346	0.92651	0.92975	0.93325
604	0.91338	0.91974	0.92263	0.92357	0.92661	0.92984	0.93334
605	0.91350	0.91985	0.92273	0.92368	0.92671	0.92994	0.93343
606	0.91361	0.91996	0.92283	0.92378	0.92681	0.93003	0.93352
607	0.91373	0.92006	0.92294	0.92388	0.92691	0.93013	0.93361
608	0.91387	0.92017	0.92305	0.92399	0.92701	0.93022	0.93369
609	0.91399	0.92028	0.92315	0.92409	0.92711	0.93032	0.93378
610	0.91411	0.92039	0.92325	0.92419	0.92720	0.93041	0.93387
611	0.91422	0.92050	0.92336	0.92429	0.92730	0.93050	0.93396
612	0.91434	0.92061	0.92346	0.92439	0.92740	0.93060	0.93405
613	0.91446	0.92071	0.92357	0.92449	0.92750	0.93069	0.93413
614	0.91457	0.92082	0.92367	0.92459	0.92760	0.93078	0.93422
615	0.91468	0.92093	0.92377	0.92470	0.92769	0.93087	0.93431
616	0.91480	0.92103	0.92387	0.92480	0.92779	0.93096	0.93439
617	0.91492	0.92114	0.92397	0.92490	0.92789	0.93106	0.93448
618	0.91503	0.92124	0.92407	0.92500	0.92798	0.93114	0.93457
619	0.91515	0.92135	0.92418	0.92510	0.92808	0.93124	0.93465
620	0.91527	0.92145	0.92427	0.92520	0.92817	0.93133	0.93474
621	0.91538	0.92157	0.92438	0.92529	0.92827	0.93142	0.93482
622	0.91549	0.92167	0.92448	0.92539	0.92836	0.93151	0.93491
623	0.91560	0.92177	0.92458	0.92549	0.92846	0.93160	0.93499

N	0.1%	0.5%	1%	1.25%	2.5%	5%	10%
624	0.91571	0.92188	0.92468	0.92559	0.92855	0.93169	0.93508
625	0.91582	0.92198	0.92477	0.92569	0.92865	0.93178	0.93516
626	0.91593	0.92209	0.92487	0.92579	0.92874	0.93187	0.93525
627	0.91605	0.92219	0.92498	0.92589	0.92883	0.93195	0.93533
628	0.91615	0.92229	0.92507	0.92599	0.92893	0.93204	0.93542
629	0.91626	0.92239	0.92517	0.92609	0.92902	0.93213	0.93550
630	0.91638	0.92250	0.92527	0.92618	0.92911	0.93222	0.93558
631	0.91649	0.92260	0.92537	0.92628	0.92920	0.93231	0.93566
632	0.91659	0.92270	0.92547	0.92638	0.92930	0.93240	0.93575
633	0.91670	0.92280	0.92556	0.92647	0.92939	0.93248	0.93583
634	0.91680	0.92290	0.92566	0.92657	0.92948	0.93257	0.93591
635	0.91691	0.92300	0.92576	0.92667	0.92957	0.93266	0.93599
636	0.91703	0.92310	0.92586	0.92676	0.92966	0.93275	0.93608
637	0.91714	0.92320	0.92595	0.92686	0.92976	0.93283	0.93616
638	0.91726	0.92330	0.92605	0.92695	0.92984	0.93292	0.93624
639	0.91736	0.92340	0.92614	0.92704	0.92994	0.93300	0.93632
640	0.91747	0.92350	0.92624	0.92714	0.93003	0.93309	0.93640
641	0.91758	0.92360	0.92634	0.92723	0.93012	0.93317	0.93648
642	0.91769	0.92370	0.92643	0.92733	0.93021	0.93326	0.93656
643	0.91780	0.92379	0.92652	0.92742	0.93030	0.93334	0.93664
644	0.91791	0.92389	0.92662	0.92751	0.93039	0.93343	0.93672
645	0.91801	0.92399	0.92671	0.92760	0.93047	0.93351	0.93680
646	0.91811	0.92409	0.92681	0.92770	0.93056	0.93360	0.93688
647	0.91821	0.92418	0.92690	0.92779	0.93065	0.93368	0.93696
648	0.91832	0.92429	0.92699	0.92788	0.93074	0.93376	0.93704
649	0.91843	0.92439	0.92708	0.92798	0.93083	0.93385	0.93712
650	0.91853	0.92449	0.92717	0.92807	0.93091	0.93393	0.93719
651	0.91864	0.92458	0.92727	0.92816	0.93100	0.93402	0.93727
652	0.91874	0.92468	0.92736	0.92825	0.93109	0.93410	0.93735
653	0.91885	0.92478	0.92745	0.92834	0.93118	0.93418	0.93743
654	0.91895	0.92488	0.92754	0.92843	0.93126	0.93426	0.93751
655	0.91905	0.92497	0.92763	0.92852	0.93135	0.93434	0.93759
656	0.91915	0.92506	0.92772	0.92861	0.93144	0.93443	0.93766
657	0.91926	0.92516	0.92781	0.92870	0.93152	0.93451	0.93774
658	0.91937	0.92526	0.92790	0.92880	0.93161	0.93459	0.93782
659	0.91946	0.92535	0.92799	0.92888	0.93169	0.93467	0.93789
660	0.91957	0.92544	0.92809	0.92897	0.93178	0.93476	0.93797
661	0.91966	0.92553	0.92818	0.92906	0.93186	0.93483	0.93805
662	0.91977	0.92563	0.92827	0.92915	0.93195	0.93492	0.93812
663	0.91987	0.92572	0.92836	0.92924	0.93203	0.93500	0.93820
664	0.91999	0.92581	0.92844	0.92932	0.93212	0.93507	0.93828
665	0.92009	0.92591	0.92854	0.92941	0.93220	0.93515	0.93835
666	0.92019	0.92600	0.92863	0.92949	0.93228	0.93524	0.93843
667	0.92029	0.92609	0.92872	0.92958	0.93237	0.93532	0.93850
668	0.92038	0.92618	0.92880	0.92967	0.93245	0.93540	0.93858
669	0.92049	0.92628	0.92889	0.92976	0.93253	0.93547	0.93865
670	0.92058	0.92637	0.92898	0.92984	0.93262	0.93555	0.93873
671	0.92068	0.92646	0.92906	0.92993	0.93270	0.93563	0.93880
672	0.92077	0.92654	0.92915	0.93002	0.93279	0.93571	0.93888
673	0.92088	0.92663	0.92924	0.93010	0.93287	0.93579	0.93895
674	0.92098	0.92672	0.92932	0.93019	0.93295	0.93586	0.93902
675	0.92109	0.92681	0.92941	0.93027	0.93303	0.93594	0.93910
676	0.92118	0.92691	0.92950	0.93036	0.93311	0.93602	0.93917
677	0.92127	0.92700	0.92958	0.93044	0.93319	0.93610	0.93924
678	0.92137	0.92709	0.92967	0.93053	0.93327	0.93618	0.93932
679	0.92147	0.92718	0.92976	0.93061	0.93336	0.93625	0.93939
680	0.92156	0.92728	0.92984	0.93070	0.93344	0.93633	0.93946
681	0.92165	0.92736	0.92993	0.93078	0.93352	0.93641	0.93953
682	0.92174	0.92746	0.93001	0.93087	0.93360	0.93648	0.93961
683	0.92186	0.92754	0.93010	0.93095	0.93368	0.93656	0.93968
684	0.92195	0.92763	0.93018	0.93103	0.93376	0.93663	0.93975
685	0.92205	0.92772	0.93027	0.93112	0.93384	0.93671	0.93982
686	0.92214	0.92781	0.93035	0.93120	0.93392	0.93679	0.93989
687	0.92224	0.92790	0.93044	0.93128	0.93400	0.93686	0.93996
688	0.92235	0.92798	0.93052	0.93137	0.93408	0.93694	0.94004
689	0.92243	0.92806	0.93061	0.93145	0.93416	0.93701	0.94011
690	0.92252	0.92815	0.93069	0.93153	0.93424	0.93709	0.94018
691	0.92262	0.92825	0.93078	0.93161	0.93432	0.93716	0.94025
692	0.92271	0.92833	0.93086	0.93170	0.93439	0.93723	0.94032
693	0.92281	0.92842	0.93094	0.93178	0.93447	0.93731	0.94039
694	0.92290	0.92851	0.93102	0.93186	0.93455	0.93739	0.94046
695	0.92298	0.92859	0.93110	0.93194	0.93463	0.93746	0.94053
696	0.92309	0.92868	0.93118	0.93202	0.93471	0.93753	0.94060
697	0.92319	0.92877	0.93127	0.93210	0.93478	0.93761	0.94067

N	0.1%	0.5%	1%	1.25%	2.5%	5%	10%
698	0.92328	0.92885	0.93135	0.93218	0.93486	0.93768	0.94074
699	0.92338	0.92894	0.93143	0.93226	0.93494	0.93775	0.94081
700	0.92347	0.92902	0.93151	0.93234	0.93501	0.93783	0.94088
701	0.92356	0.92910	0.93159	0.93243	0.93509	0.93790	0.94094
702	0.92366	0.92919	0.93167	0.93250	0.93516	0.93797	0.94101
703	0.92375	0.92927	0.93175	0.93258	0.93524	0.93804	0.94108
704	0.92384	0.92936	0.93183	0.93266	0.93531	0.93811	0.94115
705	0.92394	0.92943	0.93191	0.93274	0.93539	0.93819	0.94122
706	0.92402	0.92952	0.93199	0.93282	0.93547	0.93826	0.94129
707	0.92411	0.92960	0.93207	0.93290	0.93554	0.93833	0.94135
708	0.92421	0.92968	0.93215	0.93297	0.93562	0.93840	0.94142
709	0.92430	0.92977	0.93223	0.93306	0.93569	0.93847	0.94149
710	0.92439	0.92985	0.93231	0.93314	0.93577	0.93855	0.94155
711	0.92449	0.92994	0.93239	0.93321	0.93584	0.93862	0.94162
712	0.92458	0.93001	0.93247	0.93329	0.93592	0.93869	0.94169
713	0.92467	0.93010	0.93256	0.93337	0.93599	0.93876	0.94176
714	0.92475	0.93017	0.93263	0.93345	0.93607	0.93883	0.94182
715	0.92483	0.93025	0.93271	0.93352	0.93614	0.93890	0.94189
716	0.92493	0.93034	0.93279	0.93360	0.93622	0.93897	0.94195
717	0.92503	0.93042	0.93287	0.93368	0.93629	0.93904	0.94202
718	0.92512	0.93050	0.93294	0.93376	0.93636	0.93911	0.94209
719	0.92520	0.93059	0.93302	0.93383	0.93643	0.93918	0.94215
720	0.92529	0.93067	0.93310	0.93391	0.93651	0.93925	0.94222
721	0.92537	0.93075	0.93318	0.93399	0.93658	0.93932	0.94228
722	0.92546	0.93083	0.93325	0.93406	0.93666	0.93939	0.94235
723	0.92556	0.93091	0.93333	0.93414	0.93673	0.93946	0.94241
724	0.92563	0.93099	0.93340	0.93421	0.93680	0.93953	0.94248
725	0.92572	0.93107	0.93348	0.93429	0.93687	0.93959	0.94254
726	0.92581	0.93114	0.93356	0.93436	0.93694	0.93966	0.94261
727	0.92590	0.93123	0.93364	0.93444	0.93702	0.93973	0.94267
728	0.92598	0.93130	0.93371	0.93451	0.93709	0.93980	0.94273
729	0.92607	0.93138	0.93378	0.93458	0.93716	0.93987	0.94280
730	0.92616	0.93146	0.93386	0.93466	0.93723	0.93993	0.94286
731	0.92624	0.93154	0.93394	0.93473	0.93730	0.94000	0.94293
732	0.92633	0.93162	0.93401	0.93481	0.93737	0.94007	0.94299
733	0.92641	0.93170	0.93409	0.93488	0.93744	0.94014	0.94305
734	0.92649	0.93177	0.93416	0.93495	0.93751	0.94020	0.94312
735	0.92658	0.93185	0.93424	0.93503	0.93759	0.94027	0.94318
736	0.92665	0.93193	0.93432	0.93510	0.93766	0.94034	0.94324
737	0.92673	0.93201	0.93439	0.93517	0.93772	0.94040	0.94331
738	0.92681	0.93208	0.93446	0.93525	0.93779	0.94047	0.94337
739	0.92690	0.93216	0.93454	0.93532	0.93786	0.94054	0.94343
740	0.92698	0.93224	0.93461	0.93539	0.93794	0.94060	0.94350
741	0.92706	0.93232	0.93468	0.93547	0.93801	0.94067	0.94356
742	0.92714	0.93240	0.93476	0.93554	0.93807	0.94073	0.94362
743	0.92723	0.93247	0.93483	0.93561	0.93814	0.94080	0.94368
744	0.92730	0.93254	0.93490	0.93568	0.93821	0.94087	0.94374
745	0.92739	0.93262	0.93497	0.93575	0.93828	0.94093	0.94381
746	0.92748	0.93270	0.93504	0.93582	0.93835	0.94100	0.94387
747	0.92756	0.93277	0.93511	0.93590	0.93842	0.94106	0.94393
748	0.92764	0.93285	0.93519	0.93597	0.93849	0.94113	0.94399
749	0.92772	0.93292	0.93526	0.93604	0.93855	0.94119	0.94405
750	0.92780	0.93300	0.93533	0.93611	0.93862	0.94126	0.94411
751	0.92788	0.93307	0.93541	0.93619	0.93869	0.94132	0.94417
752	0.92797	0.93314	0.93548	0.93626	0.93876	0.94139	0.94423
753	0.92805	0.93321	0.93555	0.93632	0.93883	0.94145	0.94429
754	0.92814	0.93329	0.93562	0.93639	0.93889	0.94152	0.94435
755	0.92821	0.93336	0.93569	0.93646	0.93896	0.94158	0.94441
756	0.92829	0.93344	0.93576	0.93653	0.93902	0.94164	0.94447
757	0.92837	0.93351	0.93583	0.93660	0.93909	0.94171	0.94453
758	0.92846	0.93358	0.93590	0.93667	0.93916	0.94177	0.94459
759	0.92853	0.93366	0.93597	0.93674	0.93922	0.94183	0.94465
760	0.92862	0.93373	0.93604	0.93681	0.93929	0.94190	0.94471
761	0.92869	0.93381	0.93611	0.93688	0.93936	0.94196	0.94477
762	0.92878	0.93388	0.93618	0.93695	0.93942	0.94202	0.94483
763	0.92886	0.93395	0.93625	0.93702	0.93949	0.94208	0.94489
764	0.92893	0.93402	0.93632	0.93709	0.93955	0.94215	0.94495
765	0.92901	0.93410	0.93639	0.93716	0.93962	0.94221	0.94501
766	0.92909	0.93417	0.93646	0.93723	0.93968	0.94227	0.94507
767	0.92917	0.93424	0.93653	0.93729	0.93975	0.94233	0.94512
768	0.92925	0.93431	0.93660	0.93736	0.93981	0.94239	0.94518
769	0.92933	0.93439	0.93667	0.93743	0.93988	0.94246	0.94524
770	0.92941	0.93446	0.93674	0.93750	0.93994	0.94252	0.94530
771	0.92949	0.93453	0.93681	0.93757	0.94001	0.94258	0.94536

N	0.1%	0.5%	1%	1.25%	2.5%	5%	10%
772	0.92956	0.93460	0.93688	0.93763	0.94007	0.94264	0.94542
773	0.92963	0.93467	0.93694	0.93770	0.94014	0.94270	0.94548
774	0.92972	0.93474	0.93701	0.93777	0.94020	0.94276	0.94553
775	0.92981	0.93481	0.93708	0.93783	0.94026	0.94282	0.94559
776	0.92987	0.93488	0.93715	0.93790	0.94033	0.94288	0.94565
777	0.92995	0.93495	0.93721	0.93797	0.94039	0.94294	0.94570
778	0.93002	0.93502	0.93728	0.93803	0.94045	0.94300	0.94576
779	0.93010	0.93509	0.93735	0.93810	0.94052	0.94306	0.94582
780	0.93018	0.93515	0.93742	0.93817	0.94058	0.94313	0.94588
781	0.93024	0.93523	0.93748	0.93823	0.94064	0.94319	0.94593
782	0.93032	0.93530	0.93755	0.93830	0.94070	0.94325	0.94599
783	0.93039	0.93537	0.93762	0.93837	0.94077	0.94331	0.94604
784	0.93046	0.93544	0.93769	0.93843	0.94083	0.94337	0.94610
785	0.93054	0.93550	0.93775	0.93850	0.94089	0.94343	0.94616
786	0.93062	0.93557	0.93782	0.93856	0.94096	0.94349	0.94621
787	0.93070	0.93564	0.93789	0.93863	0.94102	0.94354	0.94627
788	0.93077	0.93571	0.93796	0.93869	0.94108	0.94360	0.94633
789	0.93085	0.93578	0.93802	0.93876	0.94114	0.94366	0.94638
790	0.93092	0.93585	0.93809	0.93882	0.94120	0.94372	0.94644
791	0.93099	0.93592	0.93815	0.93889	0.94127	0.94378	0.94649
792	0.93106	0.93598	0.93822	0.93896	0.94133	0.94384	0.94655
793	0.93113	0.93605	0.93828	0.93902	0.94139	0.94390	0.94660
794	0.93120	0.93612	0.93835	0.93908	0.94145	0.94396	0.94666
795	0.93127	0.93619	0.93841	0.93915	0.94151	0.94402	0.94671
796	0.93136	0.93625	0.93848	0.93921	0.94157	0.94407	0.94677
797	0.93142	0.93632	0.93854	0.93928	0.94163	0.94413	0.94682
798	0.93150	0.93639	0.93861	0.93934	0.94169	0.94419	0.94688
799	0.93158	0.93645	0.93867	0.93940	0.94175	0.94425	0.94694
800	0.93165	0.93652	0.93873	0.93947	0.94181	0.94430	0.94699
801	0.93171	0.93659	0.93880	0.93953	0.94188	0.94436	0.94704
802	0.93179	0.93666	0.93886	0.93959	0.94194	0.94442	0.94710
803	0.93187	0.93673	0.93892	0.93965	0.94200	0.94448	0.94715
804	0.93194	0.93680	0.93899	0.93972	0.94206	0.94453	0.94721
805	0.93202	0.93686	0.93905	0.93978	0.94212	0.94459	0.94726
806	0.93208	0.93692	0.93911	0.93984	0.94218	0.94465	0.94731
807	0.93215	0.93699	0.93918	0.93990	0.94224	0.94470	0.94737
808	0.93223	0.93705	0.93924	0.93996	0.94230	0.94476	0.94742
809	0.93229	0.93712	0.93930	0.94003	0.94236	0.94482	0.94747
810	0.93236	0.93718	0.93937	0.94009	0.94242	0.94487	0.94753
811	0.93243	0.93725	0.93943	0.94015	0.94247	0.94493	0.94758
812	0.93251	0.93732	0.93949	0.94021	0.94253	0.94499	0.94763
813	0.93258	0.93738	0.93955	0.94027	0.94259	0.94504	0.94769
814	0.93265	0.93745	0.93961	0.94033	0.94265	0.94510	0.94774
815	0.93271	0.93751	0.93968	0.94039	0.94271	0.94515	0.94779
816	0.93279	0.93758	0.93974	0.94045	0.94277	0.94521	0.94784
817	0.93286	0.93764	0.93980	0.94052	0.94283	0.94526	0.94790
818	0.93294	0.93770	0.93986	0.94058	0.94288	0.94532	0.94795
819	0.93301	0.93777	0.93993	0.94064	0.94294	0.94537	0.94800
820	0.93307	0.93783	0.93999	0.94070	0.94300	0.94543	0.94805
821	0.93314	0.93789	0.94005	0.94076	0.94306	0.94548	0.94810
822	0.93321	0.93796	0.94011	0.94082	0.94311	0.94554	0.94816
823	0.93327	0.93802	0.94017	0.94088	0.94317	0.94559	0.94821
824	0.93335	0.93808	0.94023	0.94094	0.94323	0.94565	0.94826
825	0.93342	0.93814	0.94029	0.94100	0.94329	0.94570	0.94831
826	0.93348	0.93821	0.94035	0.94106	0.94334	0.94576	0.94836
827	0.93355	0.93827	0.94041	0.94112	0.94340	0.94581	0.94841
828	0.93363	0.93833	0.94048	0.94118	0.94346	0.94586	0.94847
829	0.93370	0.93839	0.94054	0.94124	0.94351	0.94592	0.94852
830	0.93376	0.93846	0.94060	0.94130	0.94357	0.94597	0.94857
831	0.93383	0.93852	0.94066	0.94136	0.94362	0.94603	0.94862
832	0.93389	0.93859	0.94072	0.94142	0.94368	0.94608	0.94867
833	0.93396	0.93865	0.94078	0.94147	0.94374	0.94613	0.94872
834	0.93403	0.93871	0.94084	0.94153	0.94379	0.94619	0.94877
835	0.93410	0.93877	0.94090	0.94159	0.94385	0.94624	0.94882
836	0.93416	0.93883	0.94095	0.94165	0.94390	0.94629	0.94887
837	0.93423	0.93890	0.94101	0.94171	0.94396	0.94635	0.94892
838	0.93430	0.93896	0.94107	0.94177	0.94402	0.94640	0.94897
839	0.93436	0.93902	0.94113	0.94183	0.94407	0.94645	0.94902
840	0.93443	0.93908	0.94119	0.94188	0.94413	0.94651	0.94907
841	0.93450	0.93914	0.94124	0.94194	0.94418	0.94656	0.94912
842	0.93456	0.93920	0.94130	0.94200	0.94424	0.94661	0.94917
843	0.93462	0.93926	0.94136	0.94206	0.94429	0.94666	0.94922
844	0.93469	0.93932	0.94142	0.94212	0.94435	0.94672	0.94927
845	0.93476	0.93938	0.94148	0.94217	0.94440	0.94677	0.94932

N	0.1%	0.5%	1%	1.25%	2.5%	5%	10%
846	0.93484	0.93944	0.94154	0.94223	0.94446	0.94682	0.94937
847	0.93490	0.93950	0.94160	0.94229	0.94451	0.94687	0.94942
848	0.93496	0.93956	0.94166	0.94234	0.94457	0.94692	0.94947
849	0.93502	0.93962	0.94171	0.94240	0.94462	0.94697	0.94952
850	0.93508	0.93968	0.94177	0.94246	0.94468	0.94702	0.94957
851	0.93514	0.93974	0.94183	0.94252	0.94473	0.94708	0.94961
852	0.93522	0.93980	0.94189	0.94257	0.94478	0.94713	0.94966
853	0.93527	0.93986	0.94194	0.94263	0.94484	0.94718	0.94971
854	0.93534	0.93992	0.94200	0.94268	0.94489	0.94723	0.94976
855	0.93540	0.93998	0.94206	0.94274	0.94494	0.94728	0.94981
856	0.93546	0.94004	0.94211	0.94279	0.94500	0.94733	0.94986
857	0.93553	0.94010	0.94217	0.94285	0.94505	0.94738	0.94990
858	0.93559	0.94016	0.94223	0.94291	0.94510	0.94743	0.94995
859	0.93566	0.94022	0.94229	0.94297	0.94516	0.94749	0.95000
860	0.93573	0.94028	0.94234	0.94302	0.94521	0.94754	0.95005
861	0.93578	0.94034	0.94240	0.94308	0.94526	0.94759	0.95010
862	0.93584	0.94039	0.94246	0.94313	0.94532	0.94764	0.95014
863	0.93591	0.94045	0.94251	0.94319	0.94537	0.94769	0.95019
864	0.93596	0.94051	0.94257	0.94324	0.94542	0.94774	0.95024
865	0.93602	0.94057	0.94262	0.94330	0.94547	0.94779	0.95029
866	0.93609	0.94062	0.94268	0.94335	0.94553	0.94784	0.95033
867	0.93615	0.94068	0.94273	0.94341	0.94558	0.94789	0.95038
868	0.93621	0.94073	0.94279	0.94346	0.94563	0.94794	0.95043
869	0.93627	0.94079	0.94284	0.94352	0.94568	0.94799	0.95048
870	0.93634	0.94085	0.94290	0.94357	0.94574	0.94804	0.95052
871	0.93641	0.94090	0.94295	0.94363	0.94579	0.94809	0.95057
872	0.93647	0.94096	0.94301	0.94368	0.94584	0.94814	0.95062
873	0.93652	0.94102	0.94306	0.94373	0.94589	0.94818	0.95066
874	0.93658	0.94107	0.94311	0.94379	0.94594	0.94823	0.95071
875	0.93664	0.94113	0.94317	0.94384	0.94599	0.94828	0.95076
876	0.93670	0.94119	0.94322	0.94389	0.94604	0.94833	0.95080
877	0.93677	0.94124	0.94328	0.94395	0.94609	0.94838	0.95085
878	0.93684	0.94130	0.94333	0.94400	0.94615	0.94843	0.95089
879	0.93690	0.94136	0.94338	0.94406	0.94620	0.94848	0.95094
880	0.93696	0.94141	0.94344	0.94411	0.94625	0.94853	0.95099
881	0.93702	0.94147	0.94349	0.94416	0.94630	0.94857	0.95103
882	0.93708	0.94153	0.94355	0.94422	0.94635	0.94862	0.95108
883	0.93713	0.94158	0.94360	0.94427	0.94640	0.94867	0.95112
884	0.93719	0.94164	0.94365	0.94432	0.94645	0.94872	0.95117
885	0.93725	0.94169	0.94371	0.94437	0.94650	0.94877	0.95121
886	0.93731	0.94175	0.94376	0.94443	0.94655	0.94881	0.95126
887	0.93736	0.94180	0.94382	0.94448	0.94660	0.94886	0.95130
888	0.93742	0.94186	0.94387	0.94453	0.94665	0.94891	0.95135
889	0.93748	0.94191	0.94392	0.94458	0.94670	0.94896	0.95139
890	0.93754	0.94197	0.94398	0.94463	0.94675	0.94900	0.95144
891	0.93761	0.94203	0.94403	0.94469	0.94680	0.94905	0.95148
892	0.93767	0.94208	0.94408	0.94474	0.94685	0.94910	0.95153
893	0.93773	0.94214	0.94413	0.94479	0.94690	0.94915	0.95157
894	0.93779	0.94219	0.94419	0.94484	0.94695	0.94919	0.95162
895	0.93785	0.94225	0.94424	0.94490	0.94700	0.94924	0.95166
896	0.93791	0.94231	0.94429	0.94495	0.94705	0.94929	0.95171
897	0.93797	0.94236	0.94434	0.94500	0.94710	0.94933	0.95175
898	0.93803	0.94242	0.94440	0.94505	0.94715	0.94938	0.95180
899	0.93809	0.94247	0.94445	0.94510	0.94720	0.94943	0.95184
900	0.93815	0.94253	0.94450	0.94515	0.94724	0.94947	0.95188
901	0.93821	0.94258	0.94455	0.94520	0.94729	0.94952	0.95193
902	0.93827	0.94264	0.94460	0.94525	0.94734	0.94957	0.95197
903	0.93833	0.94269	0.94465	0.94530	0.94739	0.94961	0.95202
904	0.93838	0.94274	0.94470	0.94535	0.94744	0.94966	0.95206
905	0.93844	0.94280	0.94475	0.94540	0.94749	0.94971	0.95210
906	0.93849	0.94285	0.94480	0.94545	0.94754	0.94975	0.95215
907	0.93855	0.94290	0.94485	0.94550	0.94759	0.94980	0.95219
908	0.93860	0.94295	0.94491	0.94555	0.94763	0.94984	0.95223
909	0.93866	0.94300	0.94496	0.94560	0.94768	0.94989	0.95228
910	0.93872	0.94306	0.94501	0.94566	0.94773	0.94993	0.95232
911	0.93878	0.94311	0.94506	0.94571	0.94778	0.94998	0.95236
912	0.93883	0.94316	0.94511	0.94576	0.94782	0.95003	0.95241
913	0.93888	0.94321	0.94516	0.94581	0.94787	0.95007	0.95245
914	0.93894	0.94326	0.94521	0.94586	0.94792	0.95011	0.95249
915	0.93901	0.94332	0.94526	0.94591	0.94797	0.95016	0.95253
916	0.93907	0.94337	0.94531	0.94595	0.94801	0.95021	0.95258
917	0.93913	0.94342	0.94536	0.94601	0.94806	0.95025	0.95262
918	0.93918	0.94347	0.94541	0.94606	0.94811	0.95030	0.95266
919	0.93924	0.94353	0.94546	0.94611	0.94816	0.95034	0.95271

N	0.1%	0.5%	1%	1.25%	2.5%	5%	10%
920	0.93930	0.94358	0.94551	0.94615	0.94820	0.95039	0.95275
921	0.93936	0.94363	0.94556	0.94620	0.94825	0.95043	0.95279
922	0.93942	0.94368	0.94561	0.94625	0.94830	0.95048	0.95283
923	0.93947	0.94373	0.94566	0.94630	0.94834	0.95052	0.95287
924	0.93951	0.94378	0.94571	0.94635	0.94839	0.95056	0.95291
925	0.93957	0.94384	0.94576	0.94640	0.94844	0.95061	0.95296
926	0.93963	0.94389	0.94581	0.94645	0.94848	0.95065	0.95300
927	0.93968	0.94394	0.94586	0.94650	0.94853	0.95070	0.95304
928	0.93974	0.94399	0.94591	0.94654	0.94857	0.95074	0.95308
929	0.93979	0.94404	0.94596	0.94659	0.94862	0.95078	0.95312
930	0.93985	0.94409	0.94600	0.94664	0.94867	0.95083	0.95317
931	0.93991	0.94414	0.94605	0.94669	0.94871	0.95087	0.95321
932	0.93996	0.94419	0.94610	0.94674	0.94876	0.95092	0.95325
933	0.94001	0.94424	0.94615	0.94678	0.94881	0.95096	0.95329
934	0.94007	0.94429	0.94620	0.94683	0.94885	0.95100	0.95333
935	0.94012	0.94434	0.94624	0.94688	0.94890	0.95105	0.95337
936	0.94017	0.94439	0.94629	0.94693	0.94894	0.95109	0.95341
937	0.94022	0.94444	0.94634	0.94697	0.94899	0.95113	0.95346
938	0.94028	0.94449	0.94639	0.94702	0.94904	0.95118	0.95350
939	0.94033	0.94454	0.94644	0.94707	0.94908	0.95122	0.95354
940	0.94039	0.94459	0.94649	0.94711	0.94913	0.95126	0.95358
941	0.94044	0.94464	0.94653	0.94716	0.94917	0.95131	0.95362
942	0.94050	0.94469	0.94658	0.94721	0.94922	0.95135	0.95366
943	0.94054	0.94474	0.94663	0.94726	0.94926	0.95139	0.95370
944	0.94059	0.94479	0.94668	0.94730	0.94931	0.95144	0.95374
945	0.94064	0.94484	0.94672	0.94735	0.94935	0.95148	0.95378
946	0.94069	0.94489	0.94677	0.94740	0.94940	0.95152	0.95382
947	0.94075	0.94494	0.94682	0.94744	0.94944	0.95156	0.95386
948	0.94080	0.94499	0.94687	0.94749	0.94949	0.95161	0.95390
949	0.94086	0.94504	0.94691	0.94754	0.94953	0.95165	0.95395
950	0.94091	0.94509	0.94696	0.94759	0.94958	0.95169	0.95398
951	0.94097	0.94514	0.94701	0.94763	0.94962	0.95174	0.95402
952	0.94102	0.94519	0.94706	0.94768	0.94966	0.95178	0.95407
953	0.94107	0.94523	0.94710	0.94773	0.94971	0.95182	0.95410
954	0.94113	0.94528	0.94715	0.94777	0.94975	0.95186	0.95414
955	0.94118	0.94532	0.94720	0.94782	0.94980	0.95190	0.95418
956	0.94123	0.94537	0.94724	0.94786	0.94984	0.95194	0.95422
957	0.94128	0.94542	0.94729	0.94791	0.94988	0.95199	0.95426
958	0.94133	0.94546	0.94733	0.94795	0.94993	0.95203	0.95430
959	0.94139	0.94551	0.94738	0.94800	0.94997	0.95207	0.95434
960	0.94144	0.94556	0.94742	0.94804	0.95002	0.95211	0.95438
961	0.94149	0.94561	0.94747	0.94809	0.95006	0.95215	0.95442
962	0.94154	0.94565	0.94752	0.94814	0.95010	0.95219	0.95446
963	0.94158	0.94570	0.94756	0.94818	0.95014	0.95224	0.95450
964	0.94164	0.94575	0.94761	0.94822	0.95019	0.95228	0.95454
965	0.94168	0.94579	0.94765	0.94827	0.95023	0.95232	0.95458
966	0.94174	0.94584	0.94770	0.94832	0.95027	0.95236	0.95461
967	0.94179	0.94589	0.94774	0.94836	0.95032	0.95240	0.95465
968	0.94185	0.94594	0.94779	0.94840	0.95036	0.95244	0.95469
969	0.94190	0.94599	0.94784	0.94845	0.95040	0.95248	0.95473
970	0.94194	0.94603	0.94788	0.94849	0.95044	0.95252	0.95477
971	0.94199	0.94608	0.94793	0.94854	0.95049	0.95256	0.95481
972	0.94205	0.94613	0.94797	0.94858	0.95053	0.95260	0.95484
973	0.94210	0.94617	0.94802	0.94863	0.95057	0.95264	0.95488
974	0.94215	0.94622	0.94806	0.94867	0.95062	0.95269	0.95492
975	0.94220	0.94627	0.94810	0.94872	0.95066	0.95273	0.95496
976	0.94224	0.94632	0.94815	0.94876	0.95070	0.95277	0.95500
977	0.94229	0.94636	0.94820	0.94880	0.95074	0.95281	0.95504
978	0.94234	0.94641	0.94824	0.94885	0.95078	0.95285	0.95507
979	0.94239	0.94645	0.94828	0.94889	0.95083	0.95289	0.95511
980	0.94245	0.94650	0.94833	0.94893	0.95087	0.95293	0.95515
981	0.94250	0.94655	0.94838	0.94898	0.95091	0.95297	0.95519
982	0.94255	0.94659	0.94842	0.94902	0.95095	0.95301	0.95523
983	0.94260	0.94664	0.94846	0.94907	0.95099	0.95305	0.95526
984	0.94266	0.94668	0.94851	0.94911	0.95104	0.95309	0.95530
985	0.94271	0.94673	0.94855	0.94915	0.95108	0.95313	0.95534
986	0.94276	0.94677	0.94859	0.94920	0.95112	0.95317	0.95538
987	0.94280	0.94682	0.94864	0.94924	0.95116	0.95320	0.95541
988	0.94285	0.94686	0.94868	0.94928	0.95120	0.95324	0.95545
989	0.94290	0.94691	0.94873	0.94933	0.95124	0.95328	0.95549
990	0.94295	0.94696	0.94877	0.94937	0.95129	0.95332	0.95553
991	0.94300	0.94701	0.94881	0.94941	0.95133	0.95336	0.95556
992	0.94305	0.94705	0.94886	0.94946	0.95137	0.95340	0.95560
993	0.94310	0.94710	0.94890	0.94950	0.95141	0.95344	0.95564

N	0.1%	0.5%	1%	1.25%	2.5%	5%	10%
994	0.94315	0.94714	0.94895	0.94954	0.95145	0.95348	0.95567
995	0.94320	0.94719	0.94899	0.94959	0.95149	0.95352	0.95571
996	0.94325	0.94723	0.94903	0.94963	0.95153	0.95356	0.95575
997	0.94330	0.94727	0.94907	0.94967	0.95157	0.95360	0.95578
998	0.94334	0.94732	0.94912	0.94971	0.95161	0.95363	0.95582
999	0.94339	0.94736	0.94916	0.94975	0.95165	0.95367	0.95586
1000	0.94343	0.94741	0.94920	0.94979	0.95169	0.95371	0.95589

Table 3 – *GQ(HHLL)*. Critical values of upper significance levels for the Grubbs quadruple outlier test, applicable in situations where the two highest and two lowest observations are simultaneously identified as outliers, denoted as *GQ(HHLL)*.

N	0.1%	0.5%	1%	1.25%	2.5%	5%	10%
6	0.00000	0.00000	0.00000	0.00000	0.00002	0.00008	0.00034
7	0.00005	0.00027	0.00055	0.00069	0.00142	0.00295	0.00628
8	0.00076	0.00228	0.00373	0.00436	0.00718	0.01197	0.02032
9	0.00315	0.00740	0.01076	0.01215	0.01780	0.02637	0.03977
10	0.00791	0.01572	0.02127	0.02350	0.03210	0.04433	0.06207
11	0.01497	0.02668	0.03451	0.03755	0.04892	0.06429	0.08561
12	0.02401	0.03976	0.04966	0.05345	0.06726	0.08533	0.10943
13	0.03474	0.05427	0.06599	0.07037	0.08637	0.10661	0.13297
14	0.04680	0.06963	0.08305	0.08796	0.10572	0.12775	0.15583
15	0.05956	0.08553	0.10033	0.10574	0.12498	0.14847	0.17788
16	0.07294	0.10164	0.11790	0.12373	0.14399	0.16863	0.19902
17	0.08693	0.11802	0.13535	0.14149	0.16272	0.18813	0.21925
18	0.10100	0.13422	0.15241	0.15880	0.18081	0.20691	0.23859
19	0.11524	0.15036	0.16907	0.17570	0.19829	0.22502	0.25705
20	0.12952	0.16598	0.18539	0.19215	0.21537	0.24236	0.27464
21	0.14364	0.18141	0.20126	0.20814	0.23172	0.25901	0.29133
22	0.15730	0.19636	0.21670	0.22373	0.24755	0.27492	0.30730
23	0.17090	0.21105	0.23152	0.23866	0.26270	0.29024	0.32252
24	0.18471	0.22517	0.24600	0.25319	0.27732	0.30482	0.33704
25	0.19807	0.23885	0.25983	0.26707	0.29136	0.31890	0.35095
26	0.21103	0.25241	0.27326	0.28056	0.30481	0.33234	0.36422
27	0.22360	0.26510	0.28630	0.29355	0.31786	0.34524	0.37692
28	0.23579	0.27763	0.29891	0.30610	0.33029	0.35756	0.38902
29	0.24765	0.28965	0.31086	0.31814	0.34231	0.36953	0.40067
30	0.25904	0.30133	0.32248	0.32976	0.35392	0.38092	0.41181
31	0.27025	0.31268	0.33378	0.34109	0.36516	0.39197	0.42253
32	0.28091	0.32369	0.34481	0.35203	0.37591	0.40251	0.43279
33	0.29163	0.33424	0.35541	0.36257	0.38629	0.41271	0.44271
34	0.30192	0.34448	0.36563	0.37279	0.39639	0.42255	0.45222
35	0.31202	0.35454	0.37564	0.38270	0.40608	0.43206	0.46137
36	0.32183	0.36442	0.38521	0.39225	0.41545	0.44117	0.47022
37	0.33145	0.37391	0.39447	0.40154	0.42448	0.44996	0.47872
38	0.34059	0.38296	0.40349	0.41047	0.43327	0.45847	0.48692
39	0.34967	0.39178	0.41226	0.41919	0.44177	0.46672	0.49484
40	0.35841	0.40038	0.42076	0.42756	0.44999	0.47473	0.50251
41	0.36724	0.40869	0.42887	0.43565	0.45792	0.48244	0.50991
42	0.37537	0.41684	0.43683	0.44352	0.46564	0.48993	0.51708
43	0.38330	0.42457	0.44456	0.45121	0.47310	0.49715	0.52404
44	0.39119	0.43223	0.45205	0.45861	0.48030	0.50414	0.53074
45	0.39908	0.43970	0.45927	0.46584	0.48732	0.51090	0.53720
46	0.40656	0.44684	0.46633	0.47279	0.49411	0.51749	0.54351
47	0.41390	0.45397	0.47314	0.47957	0.50077	0.52390	0.54965
48	0.42091	0.46083	0.47980	0.48620	0.50716	0.53007	0.55556
49	0.42793	0.46751	0.48632	0.49265	0.51341	0.53607	0.56131
50	0.43477	0.47392	0.49261	0.49896	0.51951	0.54193	0.56691
51	0.44111	0.48024	0.49883	0.50510	0.52542	0.54761	0.57233
52	0.44772	0.48648	0.50485	0.51106	0.53120	0.55320	0.57761
53	0.45405	0.49239	0.51070	0.51685	0.53676	0.55858	0.58273
54	0.46004	0.49815	0.51637	0.52248	0.54224	0.56385	0.58773
55	0.46610	0.50390	0.52189	0.52796	0.54759	0.56898	0.59264
56	0.47179	0.50956	0.52739	0.53334	0.55278	0.57399	0.59738
57	0.47754	0.51494	0.53266	0.53856	0.55781	0.57885	0.60198
58	0.48307	0.52024	0.53775	0.54360	0.56279	0.58358	0.60650
59	0.48851	0.52548	0.54280	0.54863	0.56760	0.58821	0.61089
60	0.49401	0.53049	0.54772	0.55352	0.57231	0.59270	0.61515
61	0.49929	0.53543	0.55255	0.55830	0.57690	0.59709	0.61930
62	0.50447	0.54021	0.55730	0.56294	0.58141	0.60136	0.62337
63	0.50933	0.54499	0.56181	0.56744	0.58575	0.60555	0.62735
64	0.51432	0.54966	0.56627	0.57188	0.59002	0.60964	0.63123
65	0.51915	0.55415	0.57069	0.57623	0.59421	0.61362	0.63502

N	0.1%	0.5%	1%	1.25%	2.5%	5%	10%
66	0.52390	0.55850	0.57497	0.58046	0.59829	0.61753	0.63873
67	0.52858	0.56287	0.57912	0.58462	0.60229	0.62136	0.64236
68	0.53286	0.56714	0.58329	0.58872	0.60619	0.62509	0.64591
69	0.53721	0.57130	0.58733	0.59267	0.61005	0.62872	0.64937
70	0.54159	0.57549	0.59126	0.59656	0.61377	0.63231	0.65275
71	0.54590	0.57944	0.59514	0.60036	0.61743	0.63582	0.65607
72	0.54997	0.58335	0.59888	0.60406	0.62101	0.63925	0.65931
73	0.55394	0.58720	0.60258	0.60768	0.62450	0.64262	0.66250
74	0.55775	0.59094	0.60620	0.61130	0.62794	0.64590	0.66563
75	0.56168	0.59458	0.60977	0.61482	0.63132	0.64912	0.66868
76	0.56544	0.59821	0.61324	0.61823	0.63461	0.65230	0.67167
77	0.56932	0.60166	0.61661	0.62160	0.63784	0.65538	0.67459
78	0.57291	0.60521	0.61996	0.62490	0.64104	0.65841	0.67744
79	0.57675	0.60860	0.62325	0.62814	0.64418	0.66139	0.68027
80	0.58024	0.61185	0.62647	0.63133	0.64724	0.66431	0.68302
81	0.58371	0.61509	0.62964	0.63447	0.65020	0.66718	0.68572
82	0.58726	0.61828	0.63274	0.63753	0.65315	0.66998	0.68838
83	0.59069	0.62143	0.63574	0.64052	0.65602	0.67274	0.69099
84	0.59391	0.62449	0.63870	0.64347	0.65886	0.67544	0.69355
85	0.59711	0.62763	0.64162	0.64637	0.66167	0.67809	0.69607
86	0.60037	0.63055	0.64454	0.64922	0.66442	0.68070	0.69852
87	0.60345	0.63348	0.64736	0.65204	0.66713	0.68325	0.70094
88	0.60651	0.63643	0.65019	0.65485	0.66978	0.68576	0.70331
89	0.60953	0.63928	0.65293	0.65757	0.67235	0.68825	0.70565
90	0.61257	0.64211	0.65564	0.66023	0.67491	0.69066	0.70793
91	0.61555	0.64485	0.65831	0.66284	0.67743	0.69306	0.71018
92	0.61846	0.64751	0.66089	0.66540	0.67989	0.69540	0.71240
93	0.62122	0.65018	0.66345	0.66791	0.68232	0.69773	0.71456
94	0.62404	0.65275	0.66595	0.67042	0.68465	0.70000	0.71672
95	0.62685	0.65531	0.66842	0.67283	0.68701	0.70223	0.71882
96	0.62964	0.65784	0.67089	0.67522	0.68933	0.70443	0.72090
97	0.63226	0.66033	0.67329	0.67760	0.69160	0.70659	0.72294
98	0.63489	0.66279	0.67565	0.67997	0.69383	0.70872	0.72494
99	0.63740	0.66523	0.67795	0.68226	0.69603	0.71080	0.72692
100	0.64007	0.66757	0.68025	0.68452	0.69821	0.71286	0.72886
101	0.64266	0.66988	0.68248	0.68674	0.70035	0.71490	0.73077
102	0.64515	0.67218	0.68471	0.68894	0.70243	0.71688	0.73265
103	0.64749	0.67442	0.68691	0.69110	0.70449	0.71887	0.73449
104	0.64996	0.67668	0.68904	0.69322	0.70652	0.72077	0.73632
105	0.65227	0.67884	0.69116	0.69529	0.70853	0.72269	0.73811
106	0.65453	0.68105	0.69325	0.69735	0.71051	0.72456	0.73988
107	0.65702	0.68319	0.69534	0.69939	0.71247	0.72642	0.74163
108	0.65935	0.68532	0.69739	0.70142	0.71439	0.72827	0.74334
109	0.66150	0.68737	0.69940	0.70337	0.71627	0.73005	0.74504
110	0.66366	0.68941	0.70130	0.70530	0.71813	0.73182	0.74670
111	0.66589	0.69141	0.70328	0.70725	0.71996	0.73353	0.74834
112	0.66792	0.69341	0.70524	0.70911	0.72176	0.73524	0.74996
113	0.67009	0.69540	0.70709	0.71098	0.72353	0.73692	0.75155
114	0.67218	0.69736	0.70894	0.71281	0.72527	0.73859	0.75312
115	0.67411	0.69920	0.71077	0.71462	0.72701	0.74025	0.75468
116	0.67614	0.70105	0.71260	0.71640	0.72871	0.74187	0.75621
117	0.67809	0.70288	0.71439	0.71816	0.73038	0.74347	0.75771
118	0.68017	0.70477	0.71615	0.71990	0.73205	0.74504	0.75919
119	0.68208	0.70663	0.71788	0.72162	0.73368	0.74661	0.76066
120	0.68408	0.70843	0.71961	0.72330	0.73529	0.74814	0.76211
121	0.68601	0.71017	0.72129	0.72497	0.73690	0.74966	0.76353
122	0.68784	0.71194	0.72298	0.72665	0.73846	0.75115	0.76494
123	0.68975	0.71363	0.72465	0.72825	0.74002	0.75263	0.76632
124	0.69158	0.71536	0.72630	0.72987	0.74158	0.75410	0.76770
125	0.69345	0.71704	0.72790	0.73145	0.74308	0.75552	0.76905
126	0.69518	0.71867	0.72946	0.73301	0.74457	0.75693	0.77039
127	0.69686	0.72031	0.73100	0.73455	0.74605	0.75835	0.77171
128	0.69858	0.72194	0.73256	0.73607	0.74751	0.75972	0.77301
129	0.70042	0.72353	0.73408	0.73757	0.74893	0.76109	0.77430
130	0.70200	0.72508	0.73558	0.73906	0.75036	0.76245	0.77557
131	0.70367	0.72667	0.73707	0.74054	0.75175	0.76378	0.77682
132	0.70534	0.72819	0.73854	0.74196	0.75313	0.76510	0.77805
133	0.70704	0.72967	0.73998	0.74341	0.75449	0.76640	0.77928
134	0.70872	0.73115	0.74141	0.74485	0.75583	0.76767	0.78050
135	0.71028	0.73259	0.74284	0.74623	0.75716	0.76895	0.78170
136	0.71186	0.73405	0.74422	0.74759	0.75847	0.77019	0.78288
137	0.71339	0.73550	0.74560	0.74896	0.75978	0.77143	0.78403
138	0.71493	0.73692	0.74694	0.75029	0.76108	0.77265	0.78519
139	0.71643	0.73830	0.74832	0.75161	0.76234	0.77385	0.78633

N	0.1%	0.5%	1%	1.25%	2.5%	5%	10%
140	0.71795	0.73969	0.74962	0.75294	0.76360	0.77505	0.78745
141	0.71942	0.74106	0.75095	0.75422	0.76484	0.77622	0.78855
142	0.72089	0.74241	0.75226	0.75552	0.76607	0.77739	0.78965
143	0.72235	0.74375	0.75357	0.75678	0.76730	0.77855	0.79073
144	0.72384	0.74508	0.75484	0.75806	0.76851	0.77968	0.79181
145	0.72524	0.74639	0.75608	0.75930	0.76970	0.78081	0.79288
146	0.72660	0.74766	0.75735	0.76053	0.77086	0.78193	0.79393
147	0.72796	0.74893	0.75858	0.76177	0.77204	0.78304	0.79497
148	0.72932	0.75018	0.75980	0.76296	0.77318	0.78412	0.79599
149	0.73066	0.75145	0.76101	0.76414	0.77433	0.78520	0.79700
150	0.73201	0.75271	0.76220	0.76532	0.77545	0.78627	0.79801
151	0.73336	0.75393	0.76338	0.76646	0.77656	0.78732	0.79900
152	0.73469	0.75514	0.76453	0.76760	0.77765	0.78837	0.79999
153	0.73607	0.75634	0.76567	0.76875	0.77875	0.78941	0.80097
154	0.73738	0.75754	0.76681	0.76989	0.77981	0.79043	0.80193
155	0.73865	0.75873	0.76794	0.77103	0.78089	0.79144	0.80288
156	0.73990	0.75988	0.76905	0.77211	0.78193	0.79243	0.80381
157	0.74119	0.76102	0.77016	0.77319	0.78298	0.79342	0.80475
158	0.74249	0.76218	0.77125	0.77427	0.78402	0.79440	0.80568
159	0.74367	0.76329	0.77235	0.77536	0.78504	0.79537	0.80658
160	0.74483	0.76445	0.77343	0.77641	0.78606	0.79633	0.80748
161	0.74604	0.76556	0.77450	0.77746	0.78705	0.79728	0.80838
162	0.74727	0.76664	0.77557	0.77851	0.78805	0.79821	0.80925
163	0.74831	0.76773	0.77660	0.77952	0.78904	0.79915	0.81013
164	0.74951	0.76879	0.77763	0.78056	0.78999	0.80007	0.81099
165	0.75066	0.76986	0.77865	0.78157	0.79097	0.80098	0.81185
166	0.75180	0.77090	0.77968	0.78255	0.79191	0.80187	0.81270
167	0.75290	0.77194	0.78065	0.78353	0.79284	0.80277	0.81355
168	0.75400	0.77296	0.78164	0.78451	0.79377	0.80365	0.81437
169	0.75514	0.77399	0.78262	0.78547	0.79469	0.80453	0.81520
170	0.75622	0.77498	0.78357	0.78643	0.79560	0.80539	0.81601
171	0.75730	0.77598	0.78453	0.78738	0.79651	0.80625	0.81681
172	0.75837	0.77699	0.78547	0.78832	0.79740	0.80711	0.81761
173	0.75943	0.77798	0.78643	0.78926	0.79830	0.80795	0.81841
174	0.76052	0.77895	0.78735	0.79017	0.79918	0.80879	0.81920
175	0.76154	0.77992	0.78827	0.79109	0.80005	0.80961	0.81998
176	0.76254	0.78086	0.78921	0.79200	0.80091	0.81043	0.82074
177	0.76357	0.78182	0.79011	0.79288	0.80178	0.81124	0.82151
178	0.76462	0.78276	0.79101	0.79377	0.80261	0.81204	0.82226
179	0.76566	0.78368	0.79190	0.79466	0.80345	0.81284	0.82301
180	0.76668	0.78457	0.79281	0.79554	0.80429	0.81362	0.82375
181	0.76761	0.78546	0.79368	0.79640	0.80511	0.81440	0.82449
182	0.76856	0.78637	0.79456	0.79726	0.80593	0.81517	0.82522
183	0.76957	0.78730	0.79539	0.79809	0.80675	0.81594	0.82594
184	0.77051	0.78817	0.79625	0.79894	0.80755	0.81669	0.82665
185	0.77143	0.78904	0.79709	0.79976	0.80834	0.81744	0.82736
186	0.77242	0.78992	0.79794	0.80058	0.80912	0.81819	0.82807
187	0.77338	0.79077	0.79877	0.80140	0.80990	0.81894	0.82876
188	0.77434	0.79164	0.79957	0.80220	0.81067	0.81967	0.82945
189	0.77521	0.79249	0.80038	0.80301	0.81144	0.82039	0.83013
190	0.77609	0.79333	0.80120	0.80380	0.81220	0.82111	0.83080
191	0.77696	0.79417	0.80198	0.80460	0.81296	0.82182	0.83147
192	0.77788	0.79498	0.80276	0.80536	0.81371	0.82253	0.83214
193	0.77875	0.79580	0.80355	0.80613	0.81445	0.82323	0.83279
194	0.77965	0.79660	0.80432	0.80689	0.81518	0.82392	0.83345
195	0.78050	0.79742	0.80510	0.80766	0.81591	0.82462	0.83410
196	0.78144	0.79818	0.80588	0.80841	0.81663	0.82530	0.83474
197	0.78227	0.79900	0.80663	0.80919	0.81736	0.82599	0.83538
198	0.78313	0.79976	0.80739	0.80991	0.81806	0.82665	0.83602
199	0.78396	0.80055	0.80814	0.81065	0.81876	0.82731	0.83664
200	0.78480	0.80133	0.80887	0.81138	0.81945	0.82797	0.83726
201	0.78556	0.80209	0.80962	0.81210	0.82013	0.82863	0.83787
202	0.78644	0.80284	0.81035	0.81282	0.82082	0.82928	0.83849
203	0.78726	0.80361	0.81107	0.81354	0.82150	0.82993	0.83910
204	0.78806	0.80436	0.81179	0.81424	0.82218	0.83057	0.83970
205	0.78888	0.80509	0.81250	0.81494	0.82285	0.83120	0.84029
206	0.78967	0.80581	0.81320	0.81564	0.82351	0.83182	0.84089
207	0.79048	0.80657	0.81390	0.81633	0.82416	0.83245	0.84147
208	0.79123	0.80729	0.81459	0.81701	0.82482	0.83307	0.84206
209	0.79206	0.80799	0.81528	0.81769	0.82546	0.83369	0.84263
210	0.79279	0.80870	0.81595	0.81836	0.82610	0.83429	0.84321
211	0.79357	0.80942	0.81662	0.81903	0.82674	0.83489	0.84378
212	0.79437	0.81014	0.81730	0.81969	0.82738	0.83549	0.84434
213	0.79509	0.81083	0.81796	0.82035	0.82799	0.83608	0.84489

N	0.1%	0.5%	1%	1.25%	2.5%	5%	10%
214	0.79587	0.81153	0.81863	0.82100	0.82860	0.83667	0.84545
215	0.79655	0.81220	0.81929	0.82164	0.82922	0.83726	0.84599
216	0.79733	0.81287	0.81994	0.82228	0.82984	0.83784	0.84654
217	0.79805	0.81354	0.82057	0.82291	0.83044	0.83842	0.84708
218	0.79879	0.81419	0.82121	0.82354	0.83103	0.83899	0.84762
219	0.79952	0.81485	0.82185	0.82416	0.83163	0.83955	0.84815
220	0.80022	0.81550	0.82247	0.82478	0.83222	0.84011	0.84868
221	0.80093	0.81616	0.82309	0.82541	0.83281	0.84067	0.84921
222	0.80159	0.81680	0.82372	0.82602	0.83340	0.84122	0.84972
223	0.80226	0.81745	0.82432	0.82663	0.83398	0.84177	0.85024
224	0.80299	0.81808	0.82494	0.82722	0.83455	0.84231	0.85075
225	0.80367	0.81871	0.82554	0.82782	0.83513	0.84286	0.85127
226	0.80435	0.81932	0.82613	0.82842	0.83570	0.84339	0.85177
227	0.80502	0.81992	0.82673	0.82902	0.83626	0.84393	0.85227
228	0.80568	0.82056	0.82734	0.82960	0.83681	0.84445	0.85278
229	0.80637	0.82115	0.82792	0.83018	0.83737	0.84498	0.85327
230	0.80701	0.82176	0.82850	0.83076	0.83791	0.84550	0.85376
231	0.80767	0.82236	0.82909	0.83132	0.83845	0.84602	0.85425
232	0.80830	0.82296	0.82967	0.83189	0.83900	0.84653	0.85474
233	0.80893	0.82354	0.83024	0.83245	0.83954	0.84705	0.85521
234	0.80958	0.82413	0.83081	0.83300	0.84007	0.84756	0.85569
235	0.81019	0.82473	0.83136	0.83355	0.84060	0.84807	0.85616
236	0.81082	0.82530	0.83191	0.83410	0.84112	0.84856	0.85663
237	0.81147	0.82587	0.83246	0.83464	0.84164	0.84906	0.85709
238	0.81209	0.82645	0.83301	0.83518	0.84217	0.84955	0.85755
239	0.81272	0.82703	0.83356	0.83573	0.84268	0.85004	0.85801
240	0.81328	0.82759	0.83409	0.83626	0.84318	0.85051	0.85847
241	0.81395	0.82816	0.83464	0.83680	0.84369	0.85099	0.85892
242	0.81453	0.82872	0.83517	0.83733	0.84419	0.85147	0.85937
243	0.81509	0.82927	0.83570	0.83785	0.84469	0.85194	0.85982
244	0.81571	0.82983	0.83622	0.83837	0.84519	0.85241	0.86026
245	0.81630	0.83039	0.83675	0.83889	0.84569	0.85288	0.86070
246	0.81693	0.83095	0.83727	0.83940	0.84617	0.85335	0.86114
247	0.81753	0.83148	0.83779	0.83990	0.84667	0.85381	0.86158
248	0.81808	0.83200	0.83829	0.84042	0.84714	0.85427	0.86201
249	0.81864	0.83253	0.83881	0.84090	0.84761	0.85473	0.86244
250	0.81921	0.83308	0.83930	0.84141	0.84809	0.85518	0.86287
251	0.81977	0.83359	0.83981	0.84190	0.84857	0.85563	0.86329
252	0.82034	0.83411	0.84032	0.84238	0.84903	0.85608	0.86371
253	0.82092	0.83463	0.84082	0.84287	0.84950	0.85652	0.86412
254	0.82146	0.83513	0.84130	0.84336	0.84996	0.85696	0.86454
255	0.82202	0.83566	0.84179	0.84384	0.85043	0.85740	0.86495
256	0.82259	0.83616	0.84228	0.84431	0.85087	0.85783	0.86536
257	0.82313	0.83665	0.84275	0.84478	0.85133	0.85826	0.86577
258	0.82364	0.83714	0.84324	0.84526	0.85178	0.85869	0.86617
259	0.82418	0.83764	0.84372	0.84573	0.85224	0.85911	0.86657
260	0.82475	0.83812	0.84419	0.84619	0.85267	0.85954	0.86697
261	0.82528	0.83862	0.84465	0.84665	0.85311	0.85996	0.86736
262	0.82583	0.83912	0.84511	0.84713	0.85356	0.86037	0.86775
263	0.82633	0.83959	0.84559	0.84758	0.85399	0.86079	0.86814
264	0.82685	0.84006	0.84605	0.84804	0.85442	0.86120	0.86853
265	0.82733	0.84053	0.84651	0.84849	0.85485	0.86160	0.86892
266	0.82791	0.84102	0.84696	0.84894	0.85529	0.86201	0.86930
267	0.82841	0.84148	0.84741	0.84939	0.85572	0.86241	0.86968
268	0.82893	0.84196	0.84785	0.84983	0.85614	0.86281	0.87006
269	0.82945	0.84242	0.84831	0.85028	0.85656	0.86321	0.87043
270	0.82993	0.84287	0.84874	0.85071	0.85697	0.86361	0.87081
271	0.83042	0.84332	0.84918	0.85114	0.85739	0.86400	0.87118
272	0.83092	0.84379	0.84963	0.85157	0.85780	0.86440	0.87155
273	0.83139	0.84424	0.85008	0.85201	0.85821	0.86478	0.87191
274	0.83187	0.84469	0.85050	0.85243	0.85862	0.86517	0.87227
275	0.83234	0.84513	0.85092	0.85286	0.85903	0.86556	0.87263
276	0.83283	0.84559	0.85136	0.85328	0.85942	0.86594	0.87300
277	0.83331	0.84605	0.85178	0.85370	0.85983	0.86632	0.87335
278	0.83380	0.84647	0.85221	0.85411	0.86023	0.86670	0.87371
279	0.83429	0.84692	0.85264	0.85453	0.86061	0.86707	0.87406
280	0.83477	0.84735	0.85305	0.85493	0.86101	0.86744	0.87441
281	0.83527	0.84778	0.85348	0.85535	0.86140	0.86781	0.87477
282	0.83577	0.84822	0.85388	0.85575	0.86179	0.86818	0.87512
283	0.83624	0.84865	0.85430	0.85616	0.86217	0.86855	0.87546
284	0.83670	0.84908	0.85469	0.85656	0.86256	0.86891	0.87580
285	0.83717	0.84949	0.85510	0.85697	0.86294	0.86927	0.87613
286	0.83759	0.84991	0.85550	0.85736	0.86331	0.86963	0.87647
287	0.83805	0.85033	0.85590	0.85775	0.86369	0.86999	0.87681

N	0.1%	0.5%	1%	1.25%	2.5%	5%	10%
288	0.83850	0.85073	0.85630	0.85814	0.86406	0.87034	0.87714
289	0.83891	0.85114	0.85670	0.85853	0.86443	0.87069	0.87748
290	0.83935	0.85154	0.85708	0.85892	0.86480	0.87104	0.87781
291	0.83978	0.85197	0.85748	0.85931	0.86517	0.87139	0.87813
292	0.84022	0.85237	0.85787	0.85969	0.86554	0.87174	0.87846
293	0.84070	0.85277	0.85826	0.86007	0.86589	0.87208	0.87878
294	0.84115	0.85316	0.85865	0.86045	0.86626	0.87242	0.87911
295	0.84159	0.85357	0.85902	0.86083	0.86662	0.87276	0.87943
296	0.84201	0.85395	0.85941	0.86119	0.86697	0.87310	0.87975
297	0.84239	0.85436	0.85979	0.86157	0.86734	0.87345	0.88006
298	0.84282	0.85476	0.86016	0.86193	0.86769	0.87378	0.88037
299	0.84323	0.85514	0.86053	0.86230	0.86803	0.87411	0.88069
300	0.84364	0.85554	0.86089	0.86266	0.86838	0.87444	0.88100
301	0.84408	0.85592	0.86125	0.86303	0.86873	0.87477	0.88130
302	0.84450	0.85629	0.86162	0.86340	0.86907	0.87510	0.88161
303	0.84491	0.85668	0.86199	0.86376	0.86941	0.87542	0.88192
304	0.84531	0.85704	0.86235	0.86411	0.86975	0.87574	0.88222
305	0.84573	0.85742	0.86270	0.86447	0.87009	0.87606	0.88253
306	0.84611	0.85779	0.86307	0.86483	0.87043	0.87638	0.88283
307	0.84650	0.85816	0.86342	0.86518	0.87076	0.87670	0.88313
308	0.84689	0.85854	0.86376	0.86552	0.87109	0.87701	0.88342
309	0.84730	0.85890	0.86412	0.86587	0.87142	0.87733	0.88372
310	0.84773	0.85926	0.86446	0.86622	0.87175	0.87764	0.88402
311	0.84813	0.85961	0.86483	0.86656	0.87208	0.87795	0.88431
312	0.84855	0.85997	0.86518	0.86691	0.87240	0.87826	0.88460
313	0.84892	0.86032	0.86552	0.86724	0.87273	0.87856	0.88489
314	0.84932	0.86069	0.86585	0.86758	0.87306	0.87887	0.88518
315	0.84969	0.86104	0.86620	0.86792	0.87337	0.87917	0.88547
316	0.85006	0.86140	0.86653	0.86826	0.87370	0.87948	0.88575
317	0.85045	0.86175	0.86687	0.86858	0.87401	0.87977	0.88603
318	0.85084	0.86210	0.86721	0.86890	0.87432	0.88007	0.88631
319	0.85118	0.86245	0.86755	0.86924	0.87464	0.88037	0.88659
320	0.85157	0.86280	0.86788	0.86955	0.87495	0.88066	0.88687
321	0.85194	0.86314	0.86821	0.86989	0.87526	0.88096	0.88715
322	0.85232	0.86347	0.86853	0.87022	0.87557	0.88126	0.88742
323	0.85270	0.86382	0.86886	0.87054	0.87588	0.88155	0.88770
324	0.85307	0.86417	0.86919	0.87086	0.87618	0.88184	0.88797
325	0.85343	0.86450	0.86951	0.87117	0.87649	0.88212	0.88824
326	0.85380	0.86483	0.86983	0.87149	0.87679	0.88240	0.88851
327	0.85414	0.86518	0.87016	0.87180	0.87708	0.88269	0.88878
328	0.85449	0.86551	0.87048	0.87212	0.87738	0.88298	0.88904
329	0.85484	0.86583	0.87079	0.87243	0.87768	0.88326	0.88931
330	0.85519	0.86616	0.87110	0.87273	0.87797	0.88354	0.88958
331	0.85555	0.86649	0.87140	0.87304	0.87826	0.88382	0.88984
332	0.85592	0.86681	0.87172	0.87335	0.87855	0.88409	0.89010
333	0.85627	0.86714	0.87202	0.87365	0.87884	0.88437	0.89036
334	0.85660	0.86746	0.87233	0.87396	0.87913	0.88465	0.89062
335	0.85692	0.86777	0.87264	0.87426	0.87942	0.88492	0.89088
336	0.85729	0.86809	0.87295	0.87456	0.87970	0.88520	0.89113
337	0.85763	0.86840	0.87325	0.87486	0.87999	0.88547	0.89139
338	0.85798	0.86872	0.87354	0.87516	0.88027	0.88574	0.89164
339	0.85833	0.86902	0.87385	0.87545	0.88055	0.88600	0.89190
340	0.85865	0.86934	0.87416	0.87575	0.88083	0.88627	0.89215
341	0.85899	0.86965	0.87445	0.87604	0.88112	0.88653	0.89240
342	0.85934	0.86996	0.87475	0.87633	0.88139	0.88679	0.89265
343	0.85966	0.87024	0.87504	0.87662	0.88167	0.88706	0.89289
344	0.85999	0.87056	0.87533	0.87691	0.88195	0.88732	0.89314
345	0.86031	0.87086	0.87562	0.87720	0.88222	0.88758	0.89338
346	0.86063	0.87115	0.87591	0.87749	0.88249	0.88784	0.89362
347	0.86095	0.87145	0.87620	0.87777	0.88276	0.88810	0.89387
348	0.86131	0.87175	0.87648	0.87805	0.88303	0.88835	0.89411
349	0.86161	0.87204	0.87678	0.87834	0.88329	0.88861	0.89435
350	0.86193	0.87236	0.87707	0.87862	0.88356	0.88886	0.89458
351	0.86227	0.87264	0.87736	0.87890	0.88383	0.88912	0.89482
352	0.86260	0.87294	0.87764	0.87917	0.88409	0.88937	0.89506
353	0.86290	0.87323	0.87791	0.87944	0.88436	0.88962	0.89529
354	0.86321	0.87353	0.87819	0.87972	0.88463	0.88987	0.89553
355	0.86354	0.87381	0.87847	0.87999	0.88488	0.89011	0.89576
356	0.86385	0.87409	0.87875	0.88026	0.88514	0.89036	0.89599
357	0.86414	0.87437	0.87902	0.88053	0.88540	0.89060	0.89622
358	0.86447	0.87466	0.87929	0.88080	0.88566	0.89085	0.89645
359	0.86480	0.87494	0.87955	0.88107	0.88591	0.89109	0.89668
360	0.86511	0.87522	0.87983	0.88133	0.88617	0.89133	0.89691
361	0.86537	0.87549	0.88009	0.88160	0.88642	0.89157	0.89713

N	0.1%	0.5%	1%	1.25%	2.5%	5%	10%
362	0.86571	0.87576	0.88037	0.88186	0.88667	0.89181	0.89736
363	0.86599	0.87605	0.88063	0.88213	0.88693	0.89204	0.89758
364	0.86629	0.87631	0.88090	0.88238	0.88718	0.89228	0.89781
365	0.86658	0.87660	0.88116	0.88265	0.88742	0.89251	0.89803
366	0.86687	0.87687	0.88141	0.88290	0.88767	0.89275	0.89825
367	0.86718	0.87715	0.88168	0.88317	0.88792	0.89298	0.89847
368	0.86745	0.87742	0.88194	0.88342	0.88816	0.89321	0.89869
369	0.86774	0.87768	0.88220	0.88367	0.88840	0.89344	0.89891
370	0.86803	0.87795	0.88245	0.88392	0.88864	0.89367	0.89913
371	0.86833	0.87822	0.88270	0.88418	0.88888	0.89390	0.89934
372	0.86862	0.87849	0.88296	0.88443	0.88912	0.89412	0.89956
373	0.86890	0.87875	0.88321	0.88468	0.88936	0.89435	0.89977
374	0.86919	0.87902	0.88346	0.88493	0.88960	0.89458	0.89998
375	0.86948	0.87927	0.88371	0.88518	0.88984	0.89480	0.90020
376	0.86977	0.87953	0.88396	0.88542	0.89007	0.89502	0.90041
377	0.87004	0.87980	0.88421	0.88567	0.89030	0.89525	0.90061
378	0.87031	0.88006	0.88446	0.88591	0.89053	0.89547	0.90082
379	0.87063	0.88032	0.88471	0.88615	0.89077	0.89569	0.90103
380	0.87090	0.88057	0.88496	0.88640	0.89100	0.89590	0.90124
381	0.87118	0.88083	0.88519	0.88663	0.89123	0.89612	0.90144
382	0.87144	0.88109	0.88544	0.88686	0.89147	0.89634	0.90165
383	0.87173	0.88134	0.88568	0.88710	0.89170	0.89656	0.90185
384	0.87201	0.88159	0.88591	0.88734	0.89192	0.89677	0.90205
385	0.87230	0.88183	0.88616	0.88758	0.89215	0.89698	0.90225
386	0.87257	0.88209	0.88640	0.88781	0.89238	0.89720	0.90246
387	0.87282	0.88233	0.88664	0.88805	0.89260	0.89741	0.90266
388	0.87309	0.88256	0.88687	0.88828	0.89282	0.89762	0.90286
389	0.87336	0.88281	0.88710	0.88851	0.89305	0.89783	0.90306
390	0.87364	0.88306	0.88734	0.88874	0.89327	0.89805	0.90325
391	0.87393	0.88329	0.88757	0.88897	0.89349	0.89826	0.90345
392	0.87419	0.88354	0.88780	0.88920	0.89370	0.89846	0.90365
393	0.87445	0.88378	0.88803	0.88942	0.89392	0.89867	0.90384
394	0.87469	0.88402	0.88827	0.88965	0.89414	0.89887	0.90404
395	0.87497	0.88425	0.88850	0.88988	0.89435	0.89907	0.90423
396	0.87522	0.88449	0.88872	0.89010	0.89457	0.89928	0.90442
397	0.87547	0.88472	0.88895	0.89033	0.89478	0.89948	0.90462
398	0.87573	0.88496	0.88917	0.89055	0.89499	0.89969	0.90481
399	0.87598	0.88520	0.88939	0.89078	0.89521	0.89988	0.90500
400	0.87625	0.88543	0.88961	0.89099	0.89542	0.90009	0.90518
401	0.87649	0.88567	0.88984	0.89121	0.89563	0.90029	0.90537
402	0.87676	0.88590	0.89006	0.89143	0.89584	0.90049	0.90556
403	0.87699	0.88612	0.89028	0.89165	0.89605	0.90069	0.90575
404	0.87724	0.88636	0.89050	0.89187	0.89626	0.90089	0.90593
405	0.87748	0.88660	0.89072	0.89209	0.89646	0.90108	0.90612
406	0.87772	0.88682	0.89094	0.89231	0.89667	0.90128	0.90630
407	0.87797	0.88704	0.89115	0.89251	0.89687	0.90147	0.90648
408	0.87820	0.88728	0.89137	0.89273	0.89708	0.90167	0.90667
409	0.87847	0.88749	0.89159	0.89294	0.89728	0.90186	0.90685
410	0.87870	0.88772	0.89179	0.89315	0.89748	0.90206	0.90703
411	0.87895	0.88794	0.89202	0.89337	0.89768	0.90225	0.90721
412	0.87920	0.88816	0.89223	0.89357	0.89788	0.90244	0.90739
413	0.87944	0.88838	0.89244	0.89379	0.89807	0.90263	0.90757
414	0.87970	0.88860	0.89265	0.89399	0.89828	0.90282	0.90775
415	0.87993	0.88882	0.89286	0.89420	0.89847	0.90301	0.90792
416	0.88016	0.88904	0.89307	0.89440	0.89867	0.90319	0.90810
417	0.88042	0.88925	0.89328	0.89461	0.89887	0.90338	0.90828
418	0.88066	0.88947	0.89349	0.89482	0.89907	0.90356	0.90845
419	0.88088	0.88968	0.89370	0.89503	0.89926	0.90375	0.90863
420	0.88109	0.88989	0.89391	0.89523	0.89945	0.90393	0.90880
421	0.88132	0.89010	0.89411	0.89543	0.89965	0.90412	0.90897
422	0.88157	0.89031	0.89431	0.89563	0.89984	0.90430	0.90915
423	0.88182	0.89052	0.89452	0.89583	0.90003	0.90448	0.90932
424	0.88202	0.89073	0.89472	0.89603	0.90022	0.90466	0.90949
425	0.88225	0.89094	0.89492	0.89622	0.90041	0.90485	0.90966
426	0.88248	0.89116	0.89512	0.89642	0.90060	0.90502	0.90982
427	0.88271	0.89136	0.89532	0.89662	0.90079	0.90520	0.90999
428	0.88295	0.89156	0.89552	0.89682	0.90098	0.90538	0.91016
429	0.88318	0.89177	0.89572	0.89701	0.90117	0.90556	0.91033
430	0.88341	0.89198	0.89592	0.89720	0.90135	0.90573	0.91050
431	0.88361	0.89219	0.89611	0.89740	0.90154	0.90591	0.91066
432	0.88383	0.89239	0.89631	0.89759	0.90172	0.90609	0.91083
433	0.88406	0.89259	0.89650	0.89779	0.90190	0.90626	0.91099
434	0.88430	0.89279	0.89670	0.89798	0.90209	0.90644	0.91116
435	0.88451	0.89300	0.89689	0.89817	0.90227	0.90661	0.91132

N	0.1%	0.5%	1%	1.25%	2.5%	5%	10%
436	0.88474	0.89320	0.89709	0.89836	0.90245	0.90678	0.91148
437	0.88494	0.89340	0.89728	0.89855	0.90264	0.90695	0.91164
438	0.88515	0.89359	0.89746	0.89874	0.90282	0.90712	0.91180
439	0.88536	0.89379	0.89766	0.89893	0.90299	0.90729	0.91197
440	0.88557	0.89400	0.89785	0.89912	0.90317	0.90746	0.91212
441	0.88577	0.89420	0.89803	0.89930	0.90334	0.90763	0.91228
442	0.88597	0.89439	0.89823	0.89948	0.90352	0.90780	0.91244
443	0.88620	0.89458	0.89841	0.89967	0.90370	0.90797	0.91260
444	0.88642	0.89478	0.89860	0.89985	0.90388	0.90814	0.91276
445	0.88663	0.89498	0.89879	0.90004	0.90405	0.90830	0.91291
446	0.88684	0.89518	0.89898	0.90023	0.90423	0.90847	0.91307
447	0.88704	0.89537	0.89916	0.90041	0.90440	0.90863	0.91323
448	0.88726	0.89556	0.89934	0.90059	0.90457	0.90880	0.91338
449	0.88748	0.89575	0.89953	0.90077	0.90475	0.90896	0.91354
450	0.88772	0.89595	0.89970	0.90095	0.90492	0.90913	0.91369
451	0.88789	0.89614	0.89989	0.90113	0.90509	0.90929	0.91384
452	0.88809	0.89633	0.90007	0.90130	0.90526	0.90945	0.91400
453	0.88830	0.89651	0.90025	0.90148	0.90543	0.90961	0.91415
454	0.88850	0.89670	0.90043	0.90165	0.90560	0.90977	0.91430
455	0.88870	0.89689	0.90062	0.90184	0.90577	0.90993	0.91445
456	0.88889	0.89708	0.90079	0.90201	0.90594	0.91009	0.91460
457	0.88909	0.89726	0.90097	0.90218	0.90611	0.91025	0.91475
458	0.88930	0.89745	0.90115	0.90236	0.90627	0.91041	0.91490
459	0.88950	0.89763	0.90132	0.90253	0.90643	0.91057	0.91505
460	0.88971	0.89781	0.90150	0.90271	0.90660	0.91072	0.91519
461	0.88991	0.89800	0.90167	0.90288	0.90677	0.91088	0.91534
462	0.89011	0.89819	0.90185	0.90306	0.90693	0.91103	0.91549
463	0.89031	0.89836	0.90202	0.90322	0.90709	0.91119	0.91563
464	0.89050	0.89854	0.90219	0.90339	0.90725	0.91135	0.91578
465	0.89070	0.89871	0.90236	0.90356	0.90742	0.91150	0.91593
466	0.89090	0.89890	0.90253	0.90374	0.90758	0.91166	0.91607
467	0.89107	0.89908	0.90271	0.90391	0.90774	0.91181	0.91621
468	0.89128	0.89925	0.90288	0.90407	0.90790	0.91196	0.91636
469	0.89146	0.89943	0.90304	0.90424	0.90806	0.91211	0.91650
470	0.89166	0.89961	0.90322	0.90440	0.90822	0.91226	0.91664
471	0.89185	0.89978	0.90338	0.90457	0.90838	0.91241	0.91679
472	0.89203	0.89996	0.90355	0.90473	0.90854	0.91256	0.91693
473	0.89222	0.90014	0.90371	0.90490	0.90869	0.91271	0.91707
474	0.89242	0.90031	0.90389	0.90506	0.90885	0.91286	0.91721
475	0.89259	0.90048	0.90405	0.90522	0.90900	0.91301	0.91735
476	0.89278	0.90067	0.90421	0.90539	0.90916	0.91316	0.91749
477	0.89295	0.90083	0.90438	0.90555	0.90932	0.91331	0.91763
478	0.89312	0.90100	0.90454	0.90571	0.90947	0.91345	0.91777
479	0.89331	0.90117	0.90471	0.90587	0.90962	0.91360	0.91791
480	0.89350	0.90134	0.90487	0.90603	0.90978	0.91375	0.91804
481	0.89370	0.90151	0.90503	0.90619	0.90993	0.91389	0.91818
482	0.89388	0.90168	0.90519	0.90635	0.91008	0.91403	0.91831
483	0.89405	0.90185	0.90536	0.90651	0.91023	0.91418	0.91845
484	0.89423	0.90201	0.90551	0.90667	0.91039	0.91432	0.91859
485	0.89441	0.90217	0.90567	0.90683	0.91054	0.91447	0.91872
486	0.89459	0.90233	0.90584	0.90698	0.91069	0.91461	0.91886
487	0.89476	0.90250	0.90599	0.90714	0.91084	0.91475	0.91899
488	0.89494	0.90266	0.90615	0.90729	0.91099	0.91489	0.91913
489	0.89510	0.90282	0.90630	0.90745	0.91113	0.91503	0.91926
490	0.89528	0.90299	0.90646	0.90761	0.91128	0.91518	0.91939
491	0.89544	0.90314	0.90662	0.90776	0.91142	0.91532	0.91952
492	0.89563	0.90331	0.90677	0.90792	0.91157	0.91545	0.91965
493	0.89582	0.90347	0.90692	0.90807	0.91172	0.91560	0.91978
494	0.89599	0.90365	0.90708	0.90822	0.91186	0.91573	0.91991
495	0.89618	0.90380	0.90723	0.90838	0.91201	0.91587	0.92004
496	0.89635	0.90397	0.90739	0.90853	0.91215	0.91601	0.92017
497	0.89651	0.90412	0.90754	0.90868	0.91230	0.91615	0.92030
498	0.89669	0.90428	0.90769	0.90883	0.91244	0.91628	0.92043
499	0.89685	0.90443	0.90784	0.90898	0.91258	0.91642	0.92056
500	0.89701	0.90459	0.90800	0.90913	0.91273	0.91655	0.92069
501	0.89720	0.90475	0.90815	0.90928	0.91287	0.91669	0.92082
502	0.89736	0.90491	0.90830	0.90943	0.91301	0.91682	0.92095
503	0.89754	0.90507	0.90845	0.90958	0.91315	0.91696	0.92107
504	0.89772	0.90523	0.90860	0.90972	0.91329	0.91709	0.92120
505	0.89788	0.90538	0.90875	0.90987	0.91343	0.91723	0.92133
506	0.89804	0.90553	0.90890	0.91002	0.91357	0.91736	0.92145
507	0.89821	0.90569	0.90905	0.91016	0.91371	0.91749	0.92158
508	0.89837	0.90584	0.90920	0.91031	0.91385	0.91763	0.92170
509	0.89855	0.90599	0.90934	0.91045	0.91399	0.91775	0.92182

N	0.1%	0.5%	1%	1.25%	2.5%	5%	10%
510	0.89870	0.90615	0.90949	0.91059	0.91413	0.91789	0.92195
511	0.89886	0.90629	0.90964	0.91074	0.91427	0.91802	0.92207
512	0.89902	0.90645	0.90978	0.91088	0.91440	0.91815	0.92219
513	0.89919	0.90660	0.90993	0.91102	0.91454	0.91828	0.92232
514	0.89934	0.90675	0.91007	0.91117	0.91467	0.91841	0.92244
515	0.89951	0.90690	0.91021	0.91131	0.91481	0.91853	0.92256
516	0.89967	0.90705	0.91035	0.91145	0.91495	0.91866	0.92268
517	0.89983	0.90719	0.91050	0.91159	0.91508	0.91879	0.92280
518	0.89998	0.90734	0.91064	0.91173	0.91522	0.91892	0.92292
519	0.90015	0.90748	0.91078	0.91187	0.91535	0.91904	0.92304
520	0.90032	0.90763	0.91093	0.91200	0.91548	0.91917	0.92316
521	0.90047	0.90777	0.91106	0.91215	0.91561	0.91930	0.92328
522	0.90064	0.90792	0.91120	0.91229	0.91575	0.91942	0.92340
523	0.90080	0.90807	0.91135	0.91242	0.91588	0.91955	0.92352
524	0.90094	0.90821	0.91148	0.91256	0.91601	0.91967	0.92364
525	0.90111	0.90836	0.91163	0.91270	0.91614	0.91980	0.92376
526	0.90126	0.90850	0.91177	0.91284	0.91627	0.91992	0.92387
527	0.90141	0.90865	0.91191	0.91297	0.91640	0.92004	0.92399
528	0.90156	0.90880	0.91204	0.91311	0.91653	0.92017	0.92410
529	0.90172	0.90893	0.91218	0.91325	0.91666	0.92029	0.92422
530	0.90189	0.90908	0.91232	0.91338	0.91679	0.92041	0.92433
531	0.90204	0.90921	0.91245	0.91351	0.91692	0.92053	0.92445
532	0.90219	0.90935	0.91259	0.91365	0.91704	0.92065	0.92457
533	0.90234	0.90949	0.91272	0.91379	0.91717	0.92077	0.92468
534	0.90249	0.90963	0.91286	0.91392	0.91730	0.92089	0.92479
535	0.90265	0.90976	0.91299	0.91405	0.91743	0.92102	0.92491
536	0.90282	0.90991	0.91312	0.91418	0.91755	0.92114	0.92502
537	0.90297	0.91004	0.91326	0.91431	0.91767	0.92126	0.92513
538	0.90313	0.91018	0.91339	0.91445	0.91780	0.92137	0.92525
539	0.90328	0.91032	0.91352	0.91458	0.91793	0.92149	0.92536
540	0.90342	0.91045	0.91366	0.91471	0.91806	0.92161	0.92547
541	0.90356	0.91059	0.91379	0.91484	0.91818	0.92173	0.92558
542	0.90370	0.91073	0.91392	0.91496	0.91830	0.92184	0.92569
543	0.90386	0.91086	0.91405	0.91509	0.91843	0.92196	0.92580
544	0.90400	0.91101	0.91418	0.91522	0.91855	0.92208	0.92591
545	0.90415	0.91113	0.91430	0.91535	0.91867	0.92220	0.92602
546	0.90430	0.91127	0.91444	0.91548	0.91879	0.92231	0.92613
547	0.90444	0.91141	0.91457	0.91560	0.91891	0.92243	0.92624
548	0.90458	0.91154	0.91469	0.91573	0.91904	0.92254	0.92635
549	0.90472	0.91168	0.91482	0.91586	0.91916	0.92266	0.92646
550	0.90485	0.91181	0.91494	0.91598	0.91928	0.92278	0.92657
551	0.90501	0.91195	0.91507	0.91611	0.91940	0.92289	0.92668
552	0.90516	0.91207	0.91520	0.91623	0.91952	0.92300	0.92679
553	0.90531	0.91220	0.91532	0.91636	0.91964	0.92312	0.92689
554	0.90545	0.91234	0.91545	0.91648	0.91976	0.92323	0.92700
555	0.90559	0.91247	0.91558	0.91660	0.91988	0.92335	0.92711
556	0.90573	0.91261	0.91571	0.91673	0.92000	0.92346	0.92721
557	0.90589	0.91274	0.91583	0.91686	0.92012	0.92357	0.92732
558	0.90602	0.91287	0.91596	0.91698	0.92023	0.92368	0.92742
559	0.90617	0.91300	0.91608	0.91710	0.92035	0.92379	0.92753
560	0.90632	0.91313	0.91621	0.91722	0.92047	0.92390	0.92763
561	0.90647	0.91326	0.91633	0.91734	0.92058	0.92402	0.92774
562	0.90661	0.91338	0.91645	0.91746	0.92070	0.92413	0.92784
563	0.90675	0.91351	0.91657	0.91759	0.92081	0.92424	0.92795
564	0.90689	0.91365	0.91669	0.91771	0.92093	0.92435	0.92805
565	0.90702	0.91377	0.91681	0.91783	0.92104	0.92446	0.92815
566	0.90714	0.91390	0.91693	0.91795	0.92116	0.92457	0.92826
567	0.90727	0.91402	0.91705	0.91807	0.92127	0.92468	0.92836
568	0.90741	0.91414	0.91718	0.91819	0.92139	0.92478	0.92846
569	0.90755	0.91427	0.91729	0.91830	0.92150	0.92489	0.92857
570	0.90768	0.91439	0.91741	0.91842	0.92161	0.92500	0.92867
571	0.90781	0.91451	0.91753	0.91854	0.92173	0.92511	0.92877
572	0.90794	0.91464	0.91765	0.91866	0.92184	0.92521	0.92887
573	0.90808	0.91476	0.91777	0.91877	0.92195	0.92532	0.92897
574	0.90821	0.91488	0.91789	0.91889	0.92206	0.92543	0.92907
575	0.90834	0.91501	0.91800	0.91900	0.92217	0.92553	0.92917
576	0.90847	0.91513	0.91812	0.91912	0.92228	0.92564	0.92927
577	0.90860	0.91525	0.91824	0.91924	0.92239	0.92574	0.92937
578	0.90874	0.91537	0.91836	0.91935	0.92250	0.92585	0.92947
579	0.90887	0.91550	0.91847	0.91947	0.92261	0.92595	0.92957
580	0.90901	0.91562	0.91858	0.91958	0.92272	0.92605	0.92967
581	0.90913	0.91574	0.91870	0.91969	0.92283	0.92616	0.92977
582	0.90927	0.91586	0.91881	0.91981	0.92294	0.92626	0.92986
583	0.90940	0.91598	0.91893	0.91993	0.92305	0.92636	0.92996

N	0.1%	0.5%	1%	1.25%	2.5%	5%	10%
584	0.90952	0.91609	0.91905	0.92004	0.92316	0.92647	0.93006
585	0.90965	0.91622	0.91916	0.92015	0.92327	0.92657	0.93015
586	0.90978	0.91633	0.91928	0.92026	0.92337	0.92667	0.93025
587	0.90991	0.91645	0.91939	0.92038	0.92348	0.92677	0.93035
588	0.91005	0.91656	0.91950	0.92049	0.92359	0.92688	0.93044
589	0.91017	0.91669	0.91962	0.92060	0.92369	0.92698	0.93054
590	0.91029	0.91681	0.91973	0.92071	0.92380	0.92708	0.93064
591	0.91041	0.91692	0.91984	0.92082	0.92391	0.92718	0.93073
592	0.91055	0.91704	0.91995	0.92093	0.92401	0.92728	0.93082
593	0.91066	0.91716	0.92006	0.92104	0.92412	0.92738	0.93092
594	0.91079	0.91728	0.92017	0.92115	0.92422	0.92748	0.93101
595	0.91092	0.91739	0.92028	0.92126	0.92433	0.92758	0.93111
596	0.91104	0.91750	0.92040	0.92137	0.92443	0.92768	0.93120
597	0.91117	0.91761	0.92051	0.92148	0.92454	0.92778	0.93129
598	0.91130	0.91773	0.92062	0.92158	0.92464	0.92788	0.93139
599	0.91143	0.91785	0.92073	0.92169	0.92475	0.92797	0.93148
600	0.91155	0.91796	0.92084	0.92180	0.92485	0.92807	0.93157
601	0.91167	0.91807	0.92095	0.92191	0.92495	0.92817	0.93167
602	0.91179	0.91818	0.92106	0.92201	0.92506	0.92827	0.93176
603	0.91192	0.91830	0.92117	0.92212	0.92516	0.92836	0.93185
604	0.91203	0.91841	0.92127	0.92222	0.92526	0.92846	0.93194
605	0.91215	0.91853	0.92139	0.92233	0.92536	0.92856	0.93204
606	0.91228	0.91864	0.92150	0.92243	0.92546	0.92865	0.93213
607	0.91240	0.91875	0.92160	0.92254	0.92556	0.92875	0.93222
608	0.91251	0.91886	0.92171	0.92265	0.92566	0.92885	0.93231
609	0.91264	0.91897	0.92181	0.92275	0.92576	0.92894	0.93240
610	0.91276	0.91908	0.92192	0.92286	0.92586	0.92904	0.93249
611	0.91287	0.91919	0.92202	0.92296	0.92596	0.92913	0.93258
612	0.91299	0.91930	0.92213	0.92306	0.92606	0.92923	0.93267
613	0.91310	0.91940	0.92223	0.92317	0.92616	0.92932	0.93276
614	0.91322	0.91951	0.92234	0.92327	0.92626	0.92942	0.93285
615	0.91334	0.91962	0.92244	0.92338	0.92636	0.92951	0.93294
616	0.91346	0.91972	0.92254	0.92348	0.92646	0.92961	0.93303
617	0.91358	0.91983	0.92265	0.92358	0.92656	0.92970	0.93311
618	0.91370	0.91994	0.92275	0.92368	0.92666	0.92979	0.93320
619	0.91381	0.92005	0.92285	0.92379	0.92676	0.92989	0.93329
620	0.91394	0.92015	0.92296	0.92389	0.92685	0.92998	0.93338
621	0.91405	0.92026	0.92306	0.92399	0.92695	0.93008	0.93347
622	0.91417	0.92037	0.92317	0.92409	0.92704	0.93017	0.93355
623	0.91429	0.92047	0.92327	0.92419	0.92714	0.93026	0.93364
624	0.91440	0.92058	0.92337	0.92429	0.92724	0.93035	0.93373
625	0.91451	0.92069	0.92347	0.92439	0.92733	0.93044	0.93381
626	0.91462	0.92079	0.92357	0.92449	0.92743	0.93054	0.93390
627	0.91473	0.92090	0.92368	0.92459	0.92752	0.93063	0.93399
628	0.91485	0.92100	0.92378	0.92469	0.92762	0.93072	0.93407
629	0.91496	0.92110	0.92388	0.92479	0.92772	0.93081	0.93416
630	0.91505	0.92121	0.92398	0.92489	0.92781	0.93090	0.93424
631	0.91517	0.92131	0.92408	0.92499	0.92791	0.93099	0.93433
632	0.91529	0.92141	0.92418	0.92509	0.92800	0.93108	0.93441
633	0.91541	0.92152	0.92428	0.92519	0.92809	0.93117	0.93450
634	0.91552	0.92162	0.92438	0.92528	0.92819	0.93126	0.93458
635	0.91563	0.92172	0.92448	0.92538	0.92828	0.93135	0.93466
636	0.91574	0.92182	0.92457	0.92548	0.92837	0.93143	0.93475
637	0.91586	0.92192	0.92467	0.92557	0.92847	0.93152	0.93483
638	0.91596	0.92203	0.92477	0.92567	0.92856	0.93161	0.93491
639	0.91608	0.92213	0.92486	0.92577	0.92865	0.93170	0.93500
640	0.91619	0.92223	0.92496	0.92586	0.92874	0.93179	0.93508
641	0.91629	0.92233	0.92506	0.92596	0.92884	0.93187	0.93516
642	0.91640	0.92243	0.92515	0.92606	0.92893	0.93196	0.93525
643	0.91651	0.92253	0.92524	0.92615	0.92902	0.93205	0.93533
644	0.91663	0.92262	0.92534	0.92624	0.92911	0.93213	0.93541
645	0.91674	0.92272	0.92544	0.92634	0.92920	0.93222	0.93549
646	0.91683	0.92283	0.92553	0.92643	0.92929	0.93231	0.93557
647	0.91695	0.92292	0.92563	0.92653	0.92938	0.93239	0.93565
648	0.91706	0.92303	0.92572	0.92662	0.92947	0.93248	0.93574
649	0.91716	0.92312	0.92582	0.92671	0.92956	0.93256	0.93582
650	0.91727	0.92322	0.92591	0.92680	0.92965	0.93265	0.93590
651	0.91738	0.92332	0.92601	0.92690	0.92973	0.93273	0.93598
652	0.91749	0.92341	0.92610	0.92699	0.92982	0.93282	0.93606
653	0.91762	0.92351	0.92620	0.92709	0.92991	0.93290	0.93614
654	0.91771	0.92360	0.92629	0.92718	0.93000	0.93299	0.93622
655	0.91782	0.92370	0.92639	0.92727	0.93009	0.93307	0.93630
656	0.91791	0.92379	0.92648	0.92736	0.93018	0.93315	0.93638
657	0.91802	0.92389	0.92657	0.92745	0.93026	0.93324	0.93645

N	0.1%	0.5%	1%	1.25%	2.5%	5%	10%
658	0.91813	0.92399	0.92667	0.92754	0.93035	0.93332	0.93653
659	0.91823	0.92408	0.92676	0.92764	0.93044	0.93340	0.93661
660	0.91833	0.92418	0.92684	0.92773	0.93053	0.93349	0.93669
661	0.91843	0.92428	0.92694	0.92782	0.93061	0.93357	0.93677
662	0.91854	0.92437	0.92703	0.92790	0.93070	0.93365	0.93685
663	0.91865	0.92447	0.92712	0.92799	0.93078	0.93373	0.93692
664	0.91875	0.92456	0.92721	0.92808	0.93087	0.93382	0.93700
665	0.91885	0.92466	0.92730	0.92817	0.93096	0.93390	0.93708
666	0.91895	0.92475	0.92740	0.92826	0.93104	0.93398	0.93716
667	0.91906	0.92485	0.92749	0.92835	0.93113	0.93406	0.93723
668	0.91915	0.92494	0.92758	0.92844	0.93121	0.93414	0.93731
669	0.91926	0.92504	0.92767	0.92852	0.93130	0.93422	0.93739
670	0.91936	0.92513	0.92775	0.92861	0.93138	0.93430	0.93746
671	0.91945	0.92522	0.92784	0.92870	0.93147	0.93438	0.93754
672	0.91955	0.92531	0.92793	0.92879	0.93155	0.93446	0.93762
673	0.91966	0.92541	0.92802	0.92888	0.93164	0.93454	0.93769
674	0.91977	0.92550	0.92811	0.92897	0.93172	0.93462	0.93777
675	0.91986	0.92559	0.92820	0.92905	0.93181	0.93470	0.93784
676	0.91996	0.92569	0.92828	0.92914	0.93189	0.93478	0.93792
677	0.92005	0.92578	0.92837	0.92923	0.93197	0.93486	0.93799
678	0.92016	0.92587	0.92846	0.92932	0.93206	0.93494	0.93807
679	0.92027	0.92596	0.92855	0.92940	0.93214	0.93502	0.93814
680	0.92038	0.92606	0.92864	0.92949	0.93222	0.93510	0.93821
681	0.92047	0.92615	0.92873	0.92958	0.93230	0.93518	0.93829
682	0.92057	0.92624	0.92881	0.92966	0.93238	0.93525	0.93836
683	0.92066	0.92633	0.92890	0.92975	0.93247	0.93533	0.93844
684	0.92076	0.92642	0.92899	0.92983	0.93255	0.93541	0.93851
685	0.92086	0.92651	0.92907	0.92992	0.93263	0.93549	0.93858
686	0.92095	0.92660	0.92916	0.93000	0.93271	0.93557	0.93866
687	0.92104	0.92669	0.92924	0.93009	0.93279	0.93564	0.93873
688	0.92114	0.92678	0.92933	0.93017	0.93287	0.93572	0.93880
689	0.92123	0.92687	0.92942	0.93026	0.93295	0.93580	0.93887
690	0.92133	0.92695	0.92950	0.93034	0.93303	0.93587	0.93894
691	0.92143	0.92704	0.92958	0.93042	0.93311	0.93595	0.93902
692	0.92153	0.92713	0.92967	0.93051	0.93319	0.93603	0.93909
693	0.92162	0.92722	0.92976	0.93059	0.93327	0.93610	0.93916
694	0.92172	0.92731	0.92984	0.93067	0.93335	0.93618	0.93923
695	0.92181	0.92739	0.92992	0.93076	0.93343	0.93625	0.93930
696	0.92191	0.92748	0.93001	0.93084	0.93351	0.93633	0.93938
697	0.92201	0.92757	0.93009	0.93092	0.93359	0.93640	0.93945
698	0.92209	0.92766	0.93017	0.93100	0.93367	0.93648	0.93952
699	0.92219	0.92774	0.93026	0.93109	0.93375	0.93655	0.93959
700	0.92229	0.92783	0.93034	0.93117	0.93382	0.93663	0.93966
701	0.92237	0.92792	0.93042	0.93125	0.93390	0.93670	0.93973
702	0.92247	0.92800	0.93051	0.93134	0.93398	0.93677	0.93980
703	0.92256	0.92809	0.93059	0.93142	0.93406	0.93685	0.93987
704	0.92265	0.92817	0.93067	0.93150	0.93414	0.93692	0.93994
705	0.92276	0.92826	0.93075	0.93158	0.93422	0.93700	0.94001
706	0.92284	0.92835	0.93084	0.93166	0.93429	0.93707	0.94008
707	0.92294	0.92843	0.93092	0.93174	0.93437	0.93714	0.94015
708	0.92303	0.92852	0.93100	0.93182	0.93444	0.93722	0.94022
709	0.92313	0.92860	0.93108	0.93190	0.93452	0.93729	0.94029
710	0.92322	0.92868	0.93117	0.93198	0.93460	0.93736	0.94035
711	0.92331	0.92877	0.93125	0.93206	0.93467	0.93743	0.94042
712	0.92341	0.92885	0.93133	0.93214	0.93475	0.93751	0.94049
713	0.92349	0.92894	0.93141	0.93222	0.93483	0.93758	0.94056
714	0.92356	0.92902	0.93148	0.93230	0.93490	0.93765	0.94063
715	0.92366	0.92911	0.93156	0.93238	0.93498	0.93772	0.94070
716	0.92375	0.92919	0.93165	0.93246	0.93505	0.93779	0.94076
717	0.92383	0.92927	0.93173	0.93254	0.93513	0.93786	0.94083
718	0.92393	0.92935	0.93181	0.93262	0.93520	0.93794	0.94090
719	0.92402	0.92944	0.93189	0.93270	0.93528	0.93801	0.94097
720	0.92410	0.92952	0.93196	0.93277	0.93535	0.93808	0.94104
721	0.92420	0.92960	0.93205	0.93285	0.93543	0.93815	0.94110
722	0.92428	0.92969	0.93212	0.93293	0.93550	0.93822	0.94117
723	0.92437	0.92976	0.93220	0.93300	0.93557	0.93829	0.94124
724	0.92445	0.92985	0.93228	0.93308	0.93565	0.93836	0.94130
725	0.92455	0.92993	0.93236	0.93316	0.93572	0.93843	0.94137
726	0.92464	0.93001	0.93244	0.93324	0.93579	0.93850	0.94143
727	0.92474	0.93009	0.93252	0.93331	0.93587	0.93857	0.94150
728	0.92483	0.93017	0.93259	0.93338	0.93594	0.93864	0.94157
729	0.92491	0.93025	0.93267	0.93346	0.93601	0.93871	0.94163
730	0.92499	0.93033	0.93275	0.93354	0.93608	0.93878	0.94170
731	0.92509	0.93041	0.93282	0.93361	0.93616	0.93885	0.94176

N	0.1%	0.5%	1%	1.25%	2.5%	5%	10%
732	0.92518	0.93049	0.93290	0.93369	0.93623	0.93892	0.94183
733	0.92526	0.93057	0.93297	0.93376	0.93630	0.93899	0.94189
734	0.92535	0.93064	0.93305	0.93384	0.93637	0.93905	0.94196
735	0.92544	0.93072	0.93312	0.93391	0.93645	0.93912	0.94202
736	0.92553	0.93080	0.93320	0.93399	0.93652	0.93919	0.94209
737	0.92561	0.93088	0.93328	0.93406	0.93659	0.93926	0.94215
738	0.92570	0.93095	0.93335	0.93414	0.93666	0.93933	0.94222
739	0.92579	0.93103	0.93343	0.93421	0.93674	0.93939	0.94228
740	0.92587	0.93112	0.93350	0.93429	0.93681	0.93946	0.94235
741	0.92595	0.93119	0.93358	0.93436	0.93688	0.93953	0.94241
742	0.92605	0.93127	0.93365	0.93443	0.93695	0.93960	0.94247
743	0.92613	0.93135	0.93373	0.93451	0.93702	0.93967	0.94254
744	0.92622	0.93143	0.93380	0.93458	0.93709	0.93973	0.94260
745	0.92630	0.93150	0.93387	0.93465	0.93716	0.93980	0.94266
746	0.92638	0.93158	0.93395	0.93472	0.93723	0.93987	0.94272
747	0.92647	0.93166	0.93402	0.93480	0.93730	0.93993	0.94279
748	0.92656	0.93173	0.93410	0.93487	0.93737	0.94000	0.94285
749	0.92664	0.93181	0.93417	0.93494	0.93744	0.94007	0.94291
750	0.92672	0.93189	0.93424	0.93501	0.93751	0.94013	0.94297
751	0.92680	0.93196	0.93432	0.93509	0.93758	0.94020	0.94304
752	0.92689	0.93204	0.93439	0.93516	0.93765	0.94026	0.94310
753	0.92697	0.93212	0.93446	0.93523	0.93771	0.94033	0.94316
754	0.92704	0.93219	0.93454	0.93530	0.93778	0.94039	0.94322
755	0.92713	0.93227	0.93461	0.93537	0.93785	0.94046	0.94328
756	0.92720	0.93234	0.93468	0.93544	0.93792	0.94052	0.94335
757	0.92728	0.93242	0.93476	0.93552	0.93799	0.94059	0.94341
758	0.92735	0.93249	0.93483	0.93559	0.93806	0.94065	0.94347
759	0.92744	0.93257	0.93490	0.93566	0.93812	0.94072	0.94353
760	0.92752	0.93265	0.93497	0.93572	0.93819	0.94078	0.94359
761	0.92760	0.93272	0.93504	0.93580	0.93826	0.94085	0.94365
762	0.92769	0.93279	0.93511	0.93587	0.93832	0.94091	0.94371
763	0.92776	0.93287	0.93518	0.93594	0.93839	0.94097	0.94377
764	0.92785	0.93294	0.93525	0.93601	0.93846	0.94104	0.94383
765	0.92793	0.93301	0.93532	0.93608	0.93852	0.94110	0.94389
766	0.92801	0.93308	0.93539	0.93615	0.93859	0.94117	0.94395
767	0.92809	0.93316	0.93547	0.93622	0.93866	0.94123	0.94401
768	0.92818	0.93323	0.93554	0.93629	0.93872	0.94129	0.94407
769	0.92826	0.93331	0.93560	0.93636	0.93879	0.94136	0.94413
770	0.92834	0.93338	0.93567	0.93643	0.93886	0.94142	0.94419
771	0.92841	0.93345	0.93574	0.93649	0.93892	0.94148	0.94425
772	0.92849	0.93352	0.93581	0.93656	0.93899	0.94154	0.94431
773	0.92856	0.93359	0.93588	0.93663	0.93905	0.94161	0.94437
774	0.92864	0.93366	0.93595	0.93670	0.93912	0.94167	0.94443
775	0.92872	0.93374	0.93602	0.93676	0.93918	0.94173	0.94449
776	0.92880	0.93380	0.93609	0.93683	0.93925	0.94179	0.94455
777	0.92887	0.93388	0.93616	0.93690	0.93931	0.94186	0.94461
778	0.92895	0.93395	0.93623	0.93697	0.93938	0.94192	0.94466
779	0.92903	0.93402	0.93629	0.93703	0.93944	0.94198	0.94472
780	0.92910	0.93410	0.93636	0.93710	0.93951	0.94204	0.94478
781	0.92918	0.93417	0.93643	0.93717	0.93957	0.94210	0.94484
782	0.92925	0.93424	0.93650	0.93724	0.93963	0.94216	0.94490
783	0.92933	0.93431	0.93657	0.93731	0.93970	0.94222	0.94495
784	0.92941	0.93438	0.93663	0.93737	0.93976	0.94228	0.94501
785	0.92948	0.93445	0.93670	0.93744	0.93982	0.94235	0.94507
786	0.92956	0.93452	0.93677	0.93751	0.93989	0.94241	0.94513
787	0.92963	0.93459	0.93683	0.93757	0.93995	0.94247	0.94518
788	0.92971	0.93466	0.93690	0.93764	0.94002	0.94253	0.94524
789	0.92978	0.93473	0.93697	0.93771	0.94008	0.94259	0.94530
790	0.92986	0.93480	0.93704	0.93777	0.94014	0.94265	0.94536
791	0.92994	0.93487	0.93710	0.93784	0.94021	0.94271	0.94541
792	0.93001	0.93493	0.93717	0.93791	0.94027	0.94277	0.94547
793	0.93010	0.93500	0.93724	0.93797	0.94033	0.94283	0.94553
794	0.93017	0.93507	0.93730	0.93804	0.94039	0.94289	0.94558
795	0.93024	0.93515	0.93737	0.93810	0.94046	0.94295	0.94564
796	0.93031	0.93522	0.93744	0.93817	0.94052	0.94301	0.94570
797	0.93038	0.93528	0.93750	0.93824	0.94058	0.94307	0.94575
798	0.93047	0.93535	0.93757	0.93830	0.94064	0.94313	0.94581
799	0.93055	0.93542	0.93763	0.93836	0.94071	0.94319	0.94586
800	0.93062	0.93549	0.93770	0.93843	0.94077	0.94324	0.94592
801	0.93069	0.93555	0.93776	0.93849	0.94083	0.94330	0.94598
802	0.93076	0.93562	0.93783	0.93856	0.94089	0.94336	0.94603
803	0.93083	0.93569	0.93789	0.93862	0.94095	0.94342	0.94609
804	0.93090	0.93575	0.93796	0.93868	0.94101	0.94348	0.94614
805	0.93098	0.93582	0.93802	0.93875	0.94107	0.94354	0.94620

N	0.1%	0.5%	1%	1.25%	2.5%	5%	10%
806	0.93105	0.93589	0.93808	0.93881	0.94113	0.94360	0.94625
807	0.93113	0.93596	0.93815	0.93887	0.94119	0.94365	0.94631
808	0.93120	0.93602	0.93821	0.93894	0.94125	0.94371	0.94636
809	0.93127	0.93609	0.93828	0.93900	0.94131	0.94377	0.94642
810	0.93135	0.93615	0.93834	0.93907	0.94137	0.94383	0.94647
811	0.93142	0.93622	0.93841	0.93913	0.94143	0.94388	0.94652
812	0.93149	0.93628	0.93847	0.93919	0.94150	0.94394	0.94658
813	0.93156	0.93635	0.93853	0.93925	0.94156	0.94400	0.94663
814	0.93164	0.93641	0.93860	0.93931	0.94162	0.94405	0.94669
815	0.93170	0.93648	0.93866	0.93938	0.94167	0.94411	0.94674
816	0.93177	0.93655	0.93872	0.93944	0.94174	0.94417	0.94679
817	0.93184	0.93661	0.93878	0.93950	0.94180	0.94423	0.94685
818	0.93191	0.93668	0.93884	0.93956	0.94185	0.94428	0.94690
819	0.93199	0.93674	0.93891	0.93963	0.94191	0.94434	0.94695
820	0.93206	0.93681	0.93897	0.93969	0.94197	0.94439	0.94701
821	0.93213	0.93687	0.93903	0.93975	0.94203	0.94445	0.94706
822	0.93219	0.93694	0.93910	0.93981	0.94209	0.94451	0.94711
823	0.93227	0.93700	0.93916	0.93987	0.94215	0.94456	0.94717
824	0.93234	0.93706	0.93922	0.93993	0.94221	0.94462	0.94722
825	0.93241	0.93712	0.93928	0.93999	0.94226	0.94467	0.94727
826	0.93248	0.93719	0.93934	0.94006	0.94232	0.94473	0.94732
827	0.93256	0.93726	0.93941	0.94012	0.94238	0.94478	0.94738
828	0.93262	0.93732	0.93947	0.94018	0.94244	0.94484	0.94743
829	0.93269	0.93738	0.93953	0.94024	0.94250	0.94490	0.94748
830	0.93276	0.93744	0.93959	0.94030	0.94255	0.94495	0.94753
831	0.93282	0.93751	0.93965	0.94035	0.94261	0.94500	0.94759
832	0.93290	0.93757	0.93971	0.94042	0.94267	0.94506	0.94764
833	0.93296	0.93764	0.93978	0.94047	0.94273	0.94511	0.94769
834	0.93303	0.93770	0.93984	0.94053	0.94278	0.94517	0.94774
835	0.93310	0.93777	0.93990	0.94059	0.94284	0.94522	0.94779
836	0.93317	0.93783	0.93996	0.94065	0.94290	0.94528	0.94784
837	0.93324	0.93789	0.94001	0.94071	0.94295	0.94533	0.94789
838	0.93331	0.93795	0.94008	0.94077	0.94301	0.94538	0.94794
839	0.93338	0.93802	0.94013	0.94083	0.94307	0.94544	0.94800
840	0.93344	0.93808	0.94020	0.94089	0.94312	0.94549	0.94805
841	0.93351	0.93814	0.94025	0.94095	0.94318	0.94555	0.94810
842	0.93358	0.93820	0.94031	0.94101	0.94324	0.94560	0.94815
843	0.93365	0.93826	0.94037	0.94107	0.94329	0.94565	0.94820
844	0.93371	0.93832	0.94043	0.94112	0.94335	0.94571	0.94825
845	0.93377	0.93838	0.94049	0.94119	0.94341	0.94576	0.94830
846	0.93385	0.93845	0.94055	0.94124	0.94346	0.94581	0.94835
847	0.93392	0.93851	0.94061	0.94130	0.94352	0.94587	0.94840
848	0.93398	0.93857	0.94067	0.94136	0.94357	0.94592	0.94845
849	0.93405	0.93863	0.94073	0.94142	0.94363	0.94597	0.94850
850	0.93410	0.93869	0.94078	0.94147	0.94368	0.94603	0.94855
851	0.93417	0.93875	0.94084	0.94153	0.94374	0.94608	0.94860
852	0.93423	0.93881	0.94090	0.94159	0.94379	0.94613	0.94865
853	0.93429	0.93888	0.94096	0.94165	0.94385	0.94618	0.94870
854	0.93436	0.93893	0.94102	0.94170	0.94390	0.94623	0.94875
855	0.93443	0.93900	0.94108	0.94176	0.94396	0.94629	0.94880
856	0.93449	0.93906	0.94113	0.94182	0.94401	0.94634	0.94885
857	0.93455	0.93912	0.94119	0.94188	0.94407	0.94639	0.94890
858	0.93462	0.93918	0.94125	0.94193	0.94412	0.94644	0.94895
859	0.93469	0.93924	0.94131	0.94199	0.94418	0.94649	0.94900
860	0.93475	0.93930	0.94137	0.94205	0.94423	0.94655	0.94905
861	0.93482	0.93936	0.94142	0.94210	0.94428	0.94660	0.94910
862	0.93488	0.93942	0.94148	0.94216	0.94434	0.94665	0.94914
863	0.93494	0.93948	0.94154	0.94222	0.94439	0.94670	0.94919
864	0.93500	0.93954	0.94159	0.94227	0.94445	0.94675	0.94924
865	0.93506	0.93960	0.94165	0.94233	0.94450	0.94680	0.94929
866	0.93513	0.93965	0.94171	0.94238	0.94455	0.94686	0.94934
867	0.93519	0.93971	0.94176	0.94244	0.94461	0.94691	0.94939
868	0.93525	0.93977	0.94182	0.94249	0.94466	0.94696	0.94944
869	0.93531	0.93983	0.94188	0.94255	0.94471	0.94701	0.94948
870	0.93538	0.93989	0.94193	0.94260	0.94477	0.94706	0.94953
871	0.93545	0.93995	0.94199	0.94266	0.94482	0.94711	0.94958
872	0.93551	0.94001	0.94204	0.94272	0.94488	0.94716	0.94963
873	0.93557	0.94006	0.94210	0.94277	0.94493	0.94721	0.94967
874	0.93564	0.94012	0.94215	0.94283	0.94498	0.94726	0.94972
875	0.93569	0.94018	0.94221	0.94288	0.94503	0.94731	0.94977
876	0.93576	0.94024	0.94226	0.94293	0.94508	0.94736	0.94982
877	0.93582	0.94029	0.94232	0.94299	0.94514	0.94741	0.94986
878	0.93588	0.94035	0.94238	0.94305	0.94519	0.94746	0.94991
879	0.93595	0.94041	0.94243	0.94310	0.94524	0.94751	0.94996

N	0.1%	0.5%	1%	1.25%	2.5%	5%	10%
880	0.93601	0.94047	0.94248	0.94315	0.94529	0.94756	0.95001
881	0.93606	0.94052	0.94254	0.94321	0.94534	0.94761	0.95005
882	0.93612	0.94058	0.94259	0.94326	0.94539	0.94766	0.95010
883	0.93618	0.94064	0.94265	0.94331	0.94545	0.94771	0.95015
884	0.93624	0.94069	0.94270	0.94337	0.94550	0.94775	0.95019
885	0.93631	0.94076	0.94275	0.94342	0.94555	0.94780	0.95024
886	0.93636	0.94081	0.94281	0.94347	0.94560	0.94785	0.95029
887	0.93643	0.94086	0.94286	0.94353	0.94565	0.94790	0.95033
888	0.93648	0.94092	0.94292	0.94358	0.94570	0.94795	0.95038
889	0.93655	0.94097	0.94297	0.94363	0.94575	0.94800	0.95042
890	0.93661	0.94103	0.94303	0.94369	0.94581	0.94805	0.95047
891	0.93667	0.94109	0.94308	0.94374	0.94586	0.94810	0.95052
892	0.93672	0.94115	0.94314	0.94379	0.94591	0.94814	0.95056
893	0.93679	0.94120	0.94319	0.94384	0.94596	0.94819	0.95061
894	0.93685	0.94126	0.94324	0.94390	0.94601	0.94824	0.95065
895	0.93692	0.94131	0.94330	0.94395	0.94606	0.94829	0.95070
896	0.93697	0.94136	0.94335	0.94400	0.94611	0.94834	0.95075
897	0.93704	0.94142	0.94340	0.94406	0.94616	0.94838	0.95079
898	0.93709	0.94147	0.94346	0.94411	0.94621	0.94843	0.95084
899	0.93716	0.94153	0.94351	0.94416	0.94626	0.94848	0.95088
900	0.93721	0.94159	0.94356	0.94421	0.94631	0.94853	0.95093
901	0.93726	0.94164	0.94361	0.94427	0.94636	0.94858	0.95097
902	0.93733	0.94170	0.94367	0.94432	0.94641	0.94862	0.95102
903	0.93739	0.94175	0.94372	0.94437	0.94646	0.94867	0.95106
904	0.93745	0.94180	0.94377	0.94442	0.94651	0.94872	0.95111
905	0.93751	0.94186	0.94383	0.94447	0.94656	0.94876	0.95115
906	0.93756	0.94191	0.94388	0.94452	0.94660	0.94881	0.95119
907	0.93762	0.94197	0.94393	0.94457	0.94666	0.94886	0.95124
908	0.93767	0.94202	0.94398	0.94463	0.94670	0.94891	0.95128
909	0.93774	0.94207	0.94403	0.94468	0.94675	0.94895	0.95133
910	0.93780	0.94213	0.94408	0.94473	0.94680	0.94900	0.95137
911	0.93786	0.94219	0.94414	0.94478	0.94685	0.94905	0.95142
912	0.93792	0.94224	0.94419	0.94483	0.94690	0.94909	0.95146
913	0.93797	0.94229	0.94424	0.94488	0.94695	0.94914	0.95150
914	0.93804	0.94235	0.94429	0.94493	0.94700	0.94918	0.95155
915	0.93809	0.94240	0.94434	0.94498	0.94705	0.94923	0.95159
916	0.93815	0.94245	0.94439	0.94503	0.94710	0.94928	0.95164
917	0.93821	0.94251	0.94445	0.94508	0.94714	0.94932	0.95168
918	0.93826	0.94256	0.94449	0.94513	0.94719	0.94937	0.95172
919	0.93832	0.94261	0.94455	0.94519	0.94724	0.94941	0.95177
920	0.93837	0.94266	0.94460	0.94524	0.94729	0.94946	0.95181
921	0.93843	0.94271	0.94465	0.94528	0.94733	0.94950	0.95185
922	0.93849	0.94277	0.94470	0.94533	0.94738	0.94955	0.95190
923	0.93854	0.94282	0.94475	0.94538	0.94743	0.94959	0.95194
924	0.93860	0.94287	0.94480	0.94544	0.94748	0.94964	0.95198
925	0.93866	0.94292	0.94485	0.94548	0.94753	0.94969	0.95203
926	0.93871	0.94298	0.94490	0.94554	0.94757	0.94973	0.95207
927	0.93877	0.94303	0.94495	0.94559	0.94762	0.94978	0.95211
928	0.93882	0.94308	0.94500	0.94563	0.94767	0.94982	0.95215
929	0.93888	0.94313	0.94505	0.94568	0.94771	0.94987	0.95220
930	0.93894	0.94318	0.94510	0.94573	0.94776	0.94991	0.95224
931	0.93900	0.94323	0.94515	0.94578	0.94781	0.94996	0.95228
932	0.93905	0.94328	0.94520	0.94583	0.94785	0.95000	0.95232
933	0.93910	0.94333	0.94525	0.94588	0.94790	0.95004	0.95237
934	0.93916	0.94338	0.94530	0.94593	0.94795	0.95009	0.95241
935	0.93922	0.94343	0.94534	0.94598	0.94800	0.95013	0.95245
936	0.93927	0.94348	0.94539	0.94602	0.94804	0.95018	0.95249
937	0.93932	0.94354	0.94544	0.94607	0.94809	0.95022	0.95253
938	0.93938	0.94359	0.94549	0.94612	0.94813	0.95027	0.95258
939	0.93944	0.94364	0.94554	0.94617	0.94818	0.95031	0.95262
940	0.93949	0.94369	0.94559	0.94622	0.94823	0.95036	0.95266
941	0.93954	0.94374	0.94564	0.94627	0.94827	0.95040	0.95270
942	0.93960	0.94379	0.94569	0.94631	0.94832	0.95044	0.95274
943	0.93965	0.94384	0.94574	0.94636	0.94836	0.95049	0.95278
944	0.93971	0.94389	0.94579	0.94641	0.94841	0.95053	0.95283
945	0.93976	0.94394	0.94583	0.94646	0.94845	0.95058	0.95287
946	0.93981	0.94399	0.94588	0.94651	0.94850	0.95062	0.95291
947	0.93987	0.94404	0.94593	0.94655	0.94855	0.95066	0.95295
948	0.93992	0.94409	0.94598	0.94660	0.94859	0.95071	0.95299
949	0.93998	0.94414	0.94603	0.94665	0.94864	0.95075	0.95303
950	0.94003	0.94419	0.94607	0.94670	0.94868	0.95079	0.95307
951	0.94008	0.94424	0.94612	0.94674	0.94873	0.95083	0.95311
952	0.94013	0.94429	0.94617	0.94679	0.94877	0.95088	0.95316
953	0.94019	0.94434	0.94622	0.94684	0.94882	0.95092	0.95320

N	0.1%	0.5%	1%	1.25%	2.5%	5%	10%
954	0.94024	0.94439	0.94626	0.94688	0.94886	0.95096	0.95324
955	0.94030	0.94444	0.94631	0.94693	0.94891	0.95101	0.95328
956	0.94035	0.94449	0.94636	0.94698	0.94895	0.95105	0.95332
957	0.94041	0.94453	0.94640	0.94702	0.94900	0.95109	0.95336
958	0.94046	0.94459	0.94645	0.94707	0.94904	0.95114	0.95340
959	0.94051	0.94463	0.94650	0.94712	0.94909	0.95118	0.95344
960	0.94056	0.94468	0.94655	0.94716	0.94913	0.95122	0.95348
961	0.94061	0.94473	0.94659	0.94721	0.94918	0.95126	0.95352
962	0.94066	0.94478	0.94664	0.94725	0.94922	0.95130	0.95356
963	0.94072	0.94483	0.94668	0.94730	0.94927	0.95135	0.95360
964	0.94077	0.94488	0.94673	0.94734	0.94931	0.95139	0.95364
965	0.94082	0.94493	0.94678	0.94739	0.94935	0.95143	0.95368
966	0.94088	0.94498	0.94682	0.94744	0.94940	0.95147	0.95372
967	0.94093	0.94502	0.94687	0.94748	0.94944	0.95151	0.95376
968	0.94099	0.94507	0.94692	0.94753	0.94949	0.95156	0.95380
969	0.94104	0.94512	0.94696	0.94757	0.94953	0.95160	0.95384
970	0.94110	0.94517	0.94701	0.94762	0.94957	0.95164	0.95388
971	0.94114	0.94522	0.94706	0.94767	0.94962	0.95168	0.95391
972	0.94120	0.94526	0.94710	0.94771	0.94966	0.95172	0.95395
973	0.94125	0.94531	0.94715	0.94776	0.94971	0.95176	0.95399
974	0.94130	0.94536	0.94720	0.94780	0.94975	0.95181	0.95403
975	0.94135	0.94541	0.94725	0.94785	0.94979	0.95185	0.95407
976	0.94139	0.94546	0.94729	0.94789	0.94983	0.95189	0.95411
977	0.94145	0.94551	0.94733	0.94794	0.94988	0.95193	0.95415
978	0.94150	0.94555	0.94738	0.94798	0.94992	0.95197	0.95419
979	0.94155	0.94560	0.94743	0.94803	0.94996	0.95201	0.95423
980	0.94160	0.94565	0.94747	0.94807	0.95001	0.95205	0.95427
981	0.94166	0.94569	0.94752	0.94812	0.95005	0.95209	0.95431
982	0.94170	0.94574	0.94756	0.94816	0.95009	0.95213	0.95434
983	0.94176	0.94579	0.94761	0.94820	0.95013	0.95217	0.95438
984	0.94181	0.94584	0.94765	0.94825	0.95018	0.95221	0.95442
985	0.94187	0.94588	0.94770	0.94830	0.95022	0.95225	0.95446
986	0.94191	0.94593	0.94775	0.94834	0.95026	0.95229	0.95450
987	0.94196	0.94597	0.94779	0.94838	0.95030	0.95234	0.95454
988	0.94201	0.94602	0.94783	0.94843	0.95035	0.95238	0.95457
989	0.94206	0.94607	0.94788	0.94847	0.95039	0.95242	0.95461
990	0.94212	0.94611	0.94792	0.94851	0.95043	0.95246	0.95465
991	0.94217	0.94616	0.94797	0.94856	0.95047	0.95250	0.95469
992	0.94221	0.94621	0.94801	0.94860	0.95051	0.95254	0.95473
993	0.94226	0.94625	0.94806	0.94865	0.95055	0.95258	0.95476
994	0.94231	0.94630	0.94810	0.94869	0.95060	0.95262	0.95480
995	0.94236	0.94635	0.94815	0.94873	0.95064	0.95266	0.95484
996	0.94241	0.94639	0.94819	0.94878	0.95068	0.95270	0.95488
997	0.94246	0.94644	0.94823	0.94882	0.95072	0.95274	0.95491
998	0.94251	0.94648	0.94828	0.94886	0.95076	0.95278	0.95495
999	0.94255	0.94653	0.94832	0.94891	0.95081	0.95282	0.95499
1000	0.94260	0.94657	0.94836	0.94895	0.95085	0.95285	0.95503
