# Supplementary material for: A generalized outlier method with an automated test protocol applied to proficiency testing schemes: utilizing Monte Carlo simulation for generating critical values for the extended Grubbs statistic
Source: J AOAC Int. 2026 Apr 29;109(6):974–84. doi: 10.1093/jaoacint/qsag036 (PMC13732683; doi:10.1093/jaoacint/qsag036)
Supplement: Supplementary file 1 [file qsag036_supplementary_data.zip › aoac-25-0307-File017.pdf]

Extended Grubbs Statistics Tables for Multiple-5 Outliers

Table 1 – *GM5(5H0L or 0H5L)*. Critical values of upper significance levels for the Grubbs multiple-5 outlier test, applicable in situations where the five highest or five lowest observations are simultaneously identified as outliers, denoted as *GM5(5H0L)* and *GM5(0H5L)*, respectively.

N	0.1%	0.5%	1%	1.25%	2.5%	5%	10%
7	0.00000	0.00000	0.00001	0.00001	0.00004	0.00016	0.00064
8	0.00008	0.00041	0.00084	0.00105	0.00215	0.00448	0.00954
9	0.00103	0.00310	0.00502	0.00588	0.00967	0.01611	0.02746
10	0.00395	0.00918	0.01337	0.01511	0.02219	0.03306	0.05012
11	0.00921	0.01853	0.02518	0.02784	0.03819	0.05298	0.07477
12	0.01683	0.03035	0.03942	0.04290	0.05619	0.07434	0.09993
13	0.02639	0.04401	0.05525	0.05952	0.07536	0.09631	0.12475
14	0.03749	0.05896	0.07210	0.07699	0.09496	0.11813	0.14886
15	0.04966	0.07466	0.08945	0.09490	0.11457	0.13956	0.17196
16	0.06258	0.09065	0.10682	0.11272	0.13392	0.16036	0.19398
17	0.07604	0.10672	0.12422	0.13053	0.15292	0.18034	0.21503
18	0.08997	0.12309	0.14133	0.14803	0.17132	0.19968	0.23503
19	0.10389	0.13898	0.15820	0.16516	0.18917	0.21816	0.25393
20	0.11786	0.15471	0.17458	0.18164	0.20635	0.23587	0.27197
21	0.13199	0.17000	0.19048	0.19779	0.22295	0.25276	0.28910
22	0.14592	0.18486	0.20600	0.21347	0.23900	0.26903	0.30530
23	0.15929	0.19940	0.22096	0.22852	0.25438	0.28456	0.32075
24	0.17228	0.21370	0.23543	0.24314	0.26906	0.29932	0.33542
25	0.18534	0.22748	0.24948	0.25717	0.28321	0.31347	0.34943
26	0.19790	0.24095	0.26297	0.27069	0.29690	0.32701	0.36270
27	0.21026	0.25375	0.27593	0.28373	0.30998	0.33999	0.37543
28	0.22261	0.26624	0.28863	0.29639	0.32251	0.35245	0.38757
29	0.23446	0.27835	0.30064	0.30850	0.33459	0.36438	0.39923
30	0.24625	0.28996	0.31237	0.32016	0.34628	0.37585	0.41038
31	0.25741	0.30140	0.32379	0.33160	0.35745	0.38681	0.42107
32	0.26830	0.31244	0.33478	0.34246	0.36823	0.39744	0.43127
33	0.27906	0.32313	0.34536	0.35298	0.37866	0.40761	0.44110
34	0.28933	0.33339	0.35557	0.36326	0.38872	0.41739	0.45052
35	0.29937	0.34338	0.36551	0.37313	0.39835	0.42679	0.45964
36	0.30927	0.35309	0.37510	0.38260	0.40767	0.43591	0.46836
37	0.31849	0.36248	0.38446	0.39189	0.41674	0.44471	0.47677
38	0.32753	0.37166	0.39335	0.40073	0.42548	0.45323	0.48487
39	0.33662	0.38051	0.40192	0.40935	0.43398	0.46138	0.49272
40	0.34513	0.38902	0.41042	0.41775	0.44215	0.46936	0.50030
41	0.35358	0.39734	0.41865	0.42593	0.45009	0.47697	0.50760
42	0.36208	0.40542	0.42657	0.43382	0.45779	0.48438	0.51465
43	0.37039	0.41328	0.43434	0.44147	0.46525	0.49156	0.52146
44	0.37810	0.42092	0.44177	0.44887	0.47242	0.49853	0.52804
45	0.38570	0.42834	0.44900	0.45611	0.47942	0.50525	0.53446
46	0.39309	0.43557	0.45605	0.46317	0.48618	0.51173	0.54069
47	0.40027	0.44266	0.46302	0.46990	0.49279	0.51807	0.54671
48	0.40760	0.44948	0.46969	0.47654	0.49917	0.52417	0.55250
49	0.41435	0.45615	0.47613	0.48287	0.50544	0.53019	0.55818
50	0.42113	0.46255	0.48240	0.48914	0.51149	0.53603	0.56367
51	0.42787	0.46886	0.48859	0.49530	0.51739	0.54169	0.56900
52	0.43435	0.47501	0.49460	0.50129	0.52308	0.54717	0.57420
53	0.44064	0.48095	0.50042	0.50702	0.52868	0.55249	0.57926
54	0.44674	0.48683	0.50611	0.51262	0.53410	0.55768	0.58419
55	0.45274	0.49253	0.51167	0.51816	0.53941	0.56275	0.58895
56	0.45849	0.49802	0.51707	0.52353	0.54459	0.56768	0.59358
57	0.46417	0.50361	0.52236	0.52871	0.54961	0.57251	0.59812
58	0.46974	0.50890	0.52756	0.53386	0.55451	0.57720	0.60254
59	0.47507	0.51412	0.53261	0.53884	0.55932	0.58177	0.60687
60	0.48040	0.51916	0.53752	0.54367	0.56397	0.58620	0.61106
61	0.48581	0.52412	0.54235	0.54849	0.56854	0.59062	0.61517
62	0.49092	0.52890	0.54701	0.55308	0.57296	0.59484	0.61919
63	0.49605	0.53367	0.55159	0.55760	0.57734	0.59900	0.62309
64	0.50078	0.53827	0.55608	0.56195	0.58155	0.60302	0.62689
65	0.50558	0.54278	0.56042	0.56634	0.58570	0.60695	0.63062
66	0.51035	0.54711	0.56473	0.57062	0.58980	0.61083	0.63426
67	0.51492	0.55144	0.56890	0.57474	0.59377	0.61461	0.63785
68	0.51949	0.55570	0.57302	0.57881	0.59764	0.61830	0.64131
69	0.52386	0.55993	0.57706	0.58277	0.60143	0.62194	0.64473
70	0.52813	0.56405	0.58093	0.58660	0.60519	0.62551	0.64807
71	0.53231	0.56813	0.58481	0.59041	0.60887	0.62898	0.65131
72	0.53634	0.57198	0.58859	0.59416	0.61245	0.63235	0.65451

N	0.1%	0.5%	1%	1.25%	2.5%	5%	10%
73	0.54044	0.57571	0.59229	0.59783	0.61593	0.63568	0.65763
74	0.54457	0.57952	0.59591	0.60141	0.61937	0.63892	0.66069
75	0.54857	0.58313	0.59951	0.60493	0.62271	0.64211	0.66371
76	0.55228	0.58674	0.60294	0.60837	0.62601	0.64525	0.66664
77	0.55615	0.59027	0.60637	0.61173	0.62922	0.64832	0.66951
78	0.55978	0.59377	0.60976	0.61505	0.63237	0.65133	0.67234
79	0.56353	0.59714	0.61307	0.61830	0.63553	0.65427	0.67510
80	0.56709	0.60049	0.61624	0.62146	0.63858	0.65716	0.67782
81	0.57060	0.60377	0.61939	0.62457	0.64155	0.65997	0.68048
82	0.57406	0.60698	0.62248	0.62766	0.64445	0.66276	0.68309
83	0.57738	0.61012	0.62558	0.63067	0.64738	0.66550	0.68566
84	0.58073	0.61328	0.62857	0.63366	0.65019	0.66819	0.68818
85	0.58385	0.61638	0.63147	0.63658	0.65296	0.67084	0.69064
86	0.58709	0.61937	0.63437	0.63943	0.65570	0.67344	0.69308
87	0.59006	0.62233	0.63721	0.64220	0.65839	0.67597	0.69545
88	0.59324	0.62524	0.63997	0.64494	0.66101	0.67847	0.69779
89	0.59645	0.62811	0.64266	0.64762	0.66357	0.68093	0.70007
90	0.59937	0.63088	0.64538	0.65023	0.66610	0.68334	0.70234
91	0.60234	0.63367	0.64802	0.65286	0.66864	0.68571	0.70456
92	0.60539	0.63628	0.65065	0.65545	0.67108	0.68805	0.70675
93	0.60825	0.63901	0.65325	0.65800	0.67353	0.69034	0.70887
94	0.61113	0.64166	0.65580	0.66052	0.67590	0.69259	0.71099
95	0.61405	0.64419	0.65825	0.66296	0.67823	0.69480	0.71305
96	0.61664	0.64672	0.66073	0.66537	0.68052	0.69699	0.71509
97	0.61936	0.64918	0.66313	0.66775	0.68279	0.69912	0.71710
98	0.62201	0.65162	0.66551	0.67012	0.68502	0.70122	0.71908
99	0.62446	0.65406	0.66783	0.67241	0.68721	0.70329	0.72103
100	0.62704	0.65647	0.67015	0.67464	0.68938	0.70534	0.72296
101	0.62967	0.65882	0.67241	0.67690	0.69151	0.70735	0.72484
102	0.63220	0.66114	0.67463	0.67908	0.69360	0.70932	0.72668
103	0.63472	0.66346	0.67681	0.68122	0.69566	0.71128	0.72853
104	0.63714	0.66567	0.67896	0.68334	0.69770	0.71321	0.73032
105	0.63956	0.66791	0.68106	0.68544	0.69968	0.71508	0.73211
106	0.64200	0.67009	0.68314	0.68750	0.70169	0.71695	0.73385
107	0.64424	0.67228	0.68524	0.68952	0.70364	0.71879	0.73557
108	0.64659	0.67439	0.68728	0.69155	0.70555	0.72061	0.73726
109	0.64895	0.67650	0.68927	0.69355	0.70743	0.72240	0.73893
110	0.65120	0.67854	0.69123	0.69547	0.70928	0.72416	0.74058
111	0.65333	0.68060	0.69319	0.69739	0.71109	0.72590	0.74219
112	0.65534	0.68254	0.69510	0.69930	0.71291	0.72761	0.74379
113	0.65743	0.68453	0.69702	0.70118	0.71472	0.72931	0.74537
114	0.65965	0.68647	0.69887	0.70301	0.71648	0.73096	0.74693
115	0.66177	0.68832	0.70069	0.70481	0.71821	0.73258	0.74847
116	0.66376	0.69019	0.70249	0.70663	0.71993	0.73420	0.74999
117	0.66578	0.69209	0.70431	0.70840	0.72158	0.73580	0.75147
118	0.66778	0.69389	0.70610	0.71014	0.72324	0.73737	0.75295
119	0.66971	0.69572	0.70784	0.71188	0.72488	0.73893	0.75440
120	0.67182	0.69752	0.70956	0.71354	0.72649	0.74046	0.75583
121	0.67380	0.69930	0.71125	0.71522	0.72809	0.74197	0.75725
122	0.67558	0.70106	0.71293	0.71687	0.72966	0.74347	0.75864
123	0.67747	0.70276	0.71459	0.71852	0.73120	0.74493	0.76002
124	0.67934	0.70446	0.71622	0.72011	0.73273	0.74639	0.76138
125	0.68119	0.70613	0.71783	0.72170	0.73427	0.74782	0.76272
126	0.68293	0.70779	0.71943	0.72327	0.73576	0.74924	0.76405
127	0.68465	0.70944	0.72099	0.72484	0.73723	0.75067	0.76536
128	0.68644	0.71109	0.72256	0.72634	0.73871	0.75204	0.76666
129	0.68810	0.71266	0.72407	0.72786	0.74014	0.75341	0.76793
130	0.68989	0.71424	0.72555	0.72935	0.74154	0.75475	0.76919
131	0.69166	0.71582	0.72707	0.73084	0.74295	0.75610	0.77045
132	0.69332	0.71734	0.72856	0.73230	0.74435	0.75741	0.77167
133	0.69494	0.71885	0.73002	0.73375	0.74574	0.75871	0.77290
134	0.69660	0.72036	0.73148	0.73517	0.74709	0.76000	0.77410
135	0.69823	0.72188	0.73291	0.73658	0.74845	0.76126	0.77528
136	0.69982	0.72331	0.73430	0.73795	0.74977	0.76252	0.77645
137	0.70139	0.72476	0.73569	0.73932	0.75108	0.76374	0.77762
138	0.70289	0.72621	0.73707	0.74068	0.75239	0.76496	0.77876
139	0.70448	0.72759	0.73845	0.74205	0.75366	0.76617	0.77990
140	0.70605	0.72902	0.73980	0.74335	0.75493	0.76736	0.78101
141	0.70755	0.73045	0.74112	0.74468	0.75619	0.76854	0.78211
142	0.70909	0.73181	0.74243	0.74596	0.75743	0.76971	0.78321
143	0.71050	0.73315	0.74375	0.74728	0.75866	0.77087	0.78430
144	0.71187	0.73451	0.74503	0.74855	0.75985	0.77201	0.78536
145	0.71338	0.73588	0.74630	0.74981	0.76106	0.77314	0.78642
146	0.71490	0.73719	0.74758	0.75106	0.76223	0.77427	0.78747

N	0.1%	0.5%	1%	1.25%	2.5%	5%	10%
147	0.71633	0.73853	0.74884	0.75227	0.76341	0.77537	0.78850
148	0.71771	0.73980	0.75008	0.75349	0.76455	0.77646	0.78952
149	0.71902	0.74107	0.75130	0.75468	0.76570	0.77753	0.79053
150	0.72036	0.74234	0.75252	0.75589	0.76684	0.77860	0.79152
151	0.72177	0.74356	0.75371	0.75704	0.76794	0.77966	0.79251
152	0.72314	0.74482	0.75492	0.75822	0.76904	0.78072	0.79348
153	0.72449	0.74608	0.75610	0.75937	0.77015	0.78175	0.79445
154	0.72581	0.74729	0.75724	0.76050	0.77123	0.78279	0.79541
155	0.72713	0.74850	0.75840	0.76165	0.77232	0.78380	0.79636
156	0.72844	0.74967	0.75952	0.76278	0.77338	0.78480	0.79730
157	0.72962	0.75084	0.76062	0.76387	0.77443	0.78579	0.79823
158	0.73089	0.75198	0.76171	0.76497	0.77548	0.78678	0.79915
159	0.73219	0.75312	0.76281	0.76605	0.77650	0.78774	0.80006
160	0.73343	0.75429	0.76391	0.76713	0.77751	0.78870	0.80096
161	0.73465	0.75539	0.76498	0.76819	0.77853	0.78966	0.80185
162	0.73581	0.75647	0.76604	0.76923	0.77953	0.79059	0.80274
163	0.73704	0.75756	0.76708	0.77028	0.78052	0.79153	0.80361
164	0.73821	0.75863	0.76812	0.77129	0.78149	0.79245	0.80448
165	0.73942	0.75967	0.76916	0.77230	0.78245	0.79337	0.80533
166	0.74058	0.76079	0.77018	0.77330	0.78342	0.79429	0.80619
167	0.74167	0.76185	0.77119	0.77429	0.78439	0.79519	0.80703
168	0.74277	0.76289	0.77217	0.77526	0.78532	0.79608	0.80786
169	0.74391	0.76394	0.77316	0.77624	0.78625	0.79696	0.80869
170	0.74502	0.76501	0.77413	0.77722	0.78718	0.79783	0.80950
171	0.74604	0.76599	0.77511	0.77817	0.78808	0.79869	0.81031
172	0.74717	0.76700	0.77608	0.77914	0.78899	0.79953	0.81111
173	0.74825	0.76802	0.77703	0.78009	0.78989	0.80039	0.81190
174	0.74934	0.76901	0.77799	0.78101	0.79077	0.80124	0.81269
175	0.75039	0.76997	0.77893	0.78195	0.79165	0.80207	0.81347
176	0.75151	0.77093	0.77987	0.78286	0.79252	0.80289	0.81424
177	0.75245	0.77190	0.78080	0.78377	0.79339	0.80371	0.81500
178	0.75347	0.77285	0.78171	0.78468	0.79424	0.80452	0.81576
179	0.75455	0.77381	0.78262	0.78557	0.79508	0.80531	0.81652
180	0.75552	0.77474	0.78352	0.78645	0.79593	0.80611	0.81726
181	0.75656	0.77564	0.78441	0.78734	0.79676	0.80689	0.81799
182	0.75751	0.77659	0.78528	0.78821	0.79759	0.80767	0.81872
183	0.75862	0.77750	0.78616	0.78907	0.79843	0.80845	0.81944
184	0.75956	0.77840	0.78702	0.78993	0.79924	0.80921	0.82015
185	0.76053	0.77929	0.78785	0.79076	0.80004	0.80996	0.82086
186	0.76149	0.78018	0.78870	0.79160	0.80082	0.81072	0.82157
187	0.76240	0.78104	0.78955	0.79243	0.80162	0.81146	0.82226
188	0.76330	0.78192	0.79037	0.79325	0.80240	0.81220	0.82295
189	0.76422	0.78277	0.79121	0.79405	0.80317	0.81293	0.82364
190	0.76521	0.78362	0.79204	0.79485	0.80394	0.81366	0.82432
191	0.76612	0.78444	0.79285	0.79567	0.80471	0.81437	0.82500
192	0.76706	0.78529	0.79366	0.79645	0.80545	0.81509	0.82566
193	0.76796	0.78610	0.79447	0.79722	0.80620	0.81579	0.82632
194	0.76885	0.78693	0.79525	0.79798	0.80694	0.81650	0.82697
195	0.76979	0.78778	0.79603	0.79876	0.80767	0.81720	0.82763
196	0.77062	0.78862	0.79680	0.79953	0.80840	0.81789	0.82828
197	0.77151	0.78939	0.79755	0.80030	0.80912	0.81857	0.82892
198	0.77237	0.79018	0.79833	0.80106	0.80984	0.81924	0.82955
199	0.77319	0.79099	0.79909	0.80180	0.81056	0.81992	0.83018
200	0.77402	0.79178	0.79985	0.80254	0.81127	0.82058	0.83080
201	0.77483	0.79255	0.80058	0.80329	0.81195	0.82125	0.83142
202	0.77576	0.79331	0.80134	0.80400	0.81264	0.82190	0.83204
203	0.77662	0.79408	0.80206	0.80473	0.81333	0.82256	0.83265
204	0.77745	0.79484	0.80279	0.80544	0.81401	0.82320	0.83325
205	0.77828	0.79557	0.80351	0.80613	0.81469	0.82385	0.83385
206	0.77909	0.79634	0.80422	0.80686	0.81536	0.82448	0.83445
207	0.77987	0.79709	0.80494	0.80756	0.81603	0.82510	0.83504
208	0.78070	0.79781	0.80564	0.80825	0.81669	0.82573	0.83562
209	0.78150	0.79856	0.80634	0.80893	0.81735	0.82636	0.83620
210	0.78226	0.79927	0.80704	0.80962	0.81799	0.82697	0.83678
211	0.78305	0.79999	0.80773	0.81030	0.81864	0.82758	0.83735
212	0.78380	0.80071	0.80840	0.81098	0.81928	0.82818	0.83792
213	0.78456	0.80142	0.80908	0.81163	0.81992	0.82879	0.83848
214	0.78539	0.80212	0.80976	0.81232	0.82055	0.82938	0.83903
215	0.78611	0.80283	0.81044	0.81297	0.82117	0.82997	0.83959
216	0.78690	0.80351	0.81111	0.81362	0.82180	0.83055	0.84013
217	0.78762	0.80418	0.81177	0.81429	0.82242	0.83114	0.84068
218	0.78833	0.80488	0.81243	0.81494	0.82303	0.83172	0.84122
219	0.78912	0.80559	0.81307	0.81559	0.82364	0.83229	0.84175
220	0.78986	0.80624	0.81370	0.81621	0.82425	0.83286	0.84229

N	0.1%	0.5%	1%	1.25%	2.5%	5%	10%
221	0.79059	0.80688	0.81434	0.81683	0.82484	0.83342	0.84282
222	0.79127	0.80755	0.81497	0.81746	0.82544	0.83399	0.84334
223	0.79195	0.80820	0.81561	0.81808	0.82602	0.83455	0.84386
224	0.79261	0.80885	0.81623	0.81869	0.82660	0.83510	0.84438
225	0.79333	0.80950	0.81685	0.81929	0.82718	0.83564	0.84489
226	0.79401	0.81015	0.81746	0.81991	0.82776	0.83619	0.84541
227	0.79471	0.81077	0.81806	0.82047	0.82832	0.83673	0.84591
228	0.79539	0.81138	0.81866	0.82107	0.82889	0.83727	0.84641
229	0.79612	0.81199	0.81926	0.82166	0.82946	0.83780	0.84691
230	0.79676	0.81260	0.81987	0.82226	0.83001	0.83833	0.84741
231	0.79744	0.81322	0.82048	0.82285	0.83056	0.83886	0.84790
232	0.79810	0.81385	0.82105	0.82342	0.83113	0.83938	0.84839
233	0.79877	0.81446	0.82163	0.82400	0.83167	0.83989	0.84887
234	0.79940	0.81504	0.82220	0.82457	0.83222	0.84040	0.84936
235	0.80004	0.81565	0.82276	0.82515	0.83275	0.84092	0.84983
236	0.80071	0.81626	0.82334	0.82571	0.83328	0.84142	0.85031
237	0.80135	0.81688	0.82391	0.82627	0.83382	0.84192	0.85078
238	0.80201	0.81746	0.82450	0.82682	0.83435	0.84242	0.85125
239	0.80262	0.81805	0.82505	0.82738	0.83486	0.84292	0.85172
240	0.80324	0.81862	0.82559	0.82793	0.83538	0.84341	0.85218
241	0.80387	0.81920	0.82615	0.82846	0.83591	0.84390	0.85263
242	0.80451	0.81978	0.82671	0.82901	0.83642	0.84438	0.85308
243	0.80510	0.82033	0.82726	0.82954	0.83693	0.84486	0.85354
244	0.80575	0.82089	0.82780	0.83008	0.83743	0.84534	0.85398
245	0.80638	0.82145	0.82833	0.83059	0.83793	0.84582	0.85443
246	0.80703	0.82200	0.82885	0.83112	0.83844	0.84630	0.85486
247	0.80764	0.82255	0.82939	0.83164	0.83893	0.84676	0.85530
248	0.80823	0.82310	0.82991	0.83217	0.83943	0.84723	0.85574
249	0.80883	0.82364	0.83043	0.83268	0.83991	0.84769	0.85617
250	0.80944	0.82417	0.83094	0.83319	0.84039	0.84815	0.85660
251	0.81003	0.82472	0.83145	0.83369	0.84088	0.84860	0.85703
252	0.81063	0.82524	0.83197	0.83420	0.84135	0.84906	0.85745
253	0.81122	0.82578	0.83245	0.83469	0.84183	0.84951	0.85788
254	0.81176	0.82631	0.83296	0.83519	0.84231	0.84996	0.85830
255	0.81235	0.82685	0.83347	0.83570	0.84279	0.85040	0.85871
256	0.81288	0.82738	0.83398	0.83619	0.84326	0.85084	0.85913
257	0.81342	0.82790	0.83446	0.83667	0.84373	0.85128	0.85953
258	0.81398	0.82840	0.83495	0.83714	0.84420	0.85172	0.85994
259	0.81453	0.82889	0.83544	0.83761	0.84466	0.85214	0.86035
260	0.81506	0.82939	0.83591	0.83809	0.84510	0.85257	0.86076
261	0.81557	0.82989	0.83640	0.83858	0.84555	0.85300	0.86116
262	0.81619	0.83041	0.83687	0.83904	0.84599	0.85342	0.86155
263	0.81671	0.83090	0.83733	0.83951	0.84644	0.85384	0.86195
264	0.81724	0.83140	0.83780	0.83996	0.84688	0.85427	0.86234
265	0.81777	0.83189	0.83827	0.84042	0.84732	0.85468	0.86273
266	0.81833	0.83237	0.83874	0.84089	0.84776	0.85510	0.86311
267	0.81885	0.83286	0.83921	0.84134	0.84820	0.85551	0.86350
268	0.81941	0.83335	0.83967	0.84181	0.84863	0.85592	0.86389
269	0.81993	0.83381	0.84015	0.84227	0.84906	0.85633	0.86426
270	0.82046	0.83430	0.84060	0.84271	0.84949	0.85673	0.86464
271	0.82096	0.83477	0.84106	0.84315	0.84991	0.85713	0.86502
272	0.82148	0.83523	0.84150	0.84359	0.85033	0.85754	0.86539
273	0.82199	0.83571	0.84195	0.84403	0.85075	0.85793	0.86576
274	0.82247	0.83616	0.84238	0.84446	0.85117	0.85832	0.86613
275	0.82297	0.83662	0.84283	0.84489	0.85158	0.85871	0.86649
276	0.82348	0.83707	0.84327	0.84533	0.85200	0.85911	0.86686
277	0.82397	0.83753	0.84372	0.84578	0.85241	0.85949	0.86722
278	0.82442	0.83798	0.84416	0.84619	0.85280	0.85987	0.86758
279	0.82495	0.83844	0.84458	0.84661	0.85321	0.86025	0.86794
280	0.82539	0.83889	0.84500	0.84703	0.85362	0.86063	0.86829
281	0.82588	0.83934	0.84542	0.84745	0.85402	0.86101	0.86864
282	0.82637	0.83976	0.84584	0.84786	0.85442	0.86138	0.86900
283	0.82686	0.84022	0.84627	0.84828	0.85481	0.86176	0.86935
284	0.82736	0.84065	0.84669	0.84869	0.85520	0.86213	0.86969
285	0.82779	0.84109	0.84710	0.84910	0.85558	0.86250	0.87004
286	0.82827	0.84151	0.84752	0.84951	0.85597	0.86286	0.87038
287	0.82875	0.84194	0.84794	0.84992	0.85636	0.86322	0.87072
288	0.82923	0.84239	0.84834	0.85032	0.85674	0.86358	0.87106
289	0.82972	0.84280	0.84874	0.85071	0.85713	0.86395	0.87140
290	0.83017	0.84323	0.84914	0.85111	0.85751	0.86430	0.87174
291	0.83061	0.84366	0.84954	0.85151	0.85788	0.86466	0.87207
292	0.83110	0.84406	0.84995	0.85190	0.85826	0.86502	0.87240
293	0.83155	0.84446	0.85035	0.85229	0.85862	0.86537	0.87273
294	0.83197	0.84486	0.85074	0.85267	0.85899	0.86571	0.87306

N	0.1%	0.5%	1%	1.25%	2.5%	5%	10%
295	0.83242	0.84527	0.85113	0.85307	0.85935	0.86606	0.87339
296	0.83283	0.84567	0.85153	0.85346	0.85972	0.86641	0.87371
297	0.83329	0.84609	0.85191	0.85383	0.86008	0.86675	0.87403
298	0.83376	0.84647	0.85229	0.85421	0.86044	0.86709	0.87435
299	0.83419	0.84688	0.85267	0.85458	0.86080	0.86744	0.87467
300	0.83460	0.84727	0.85305	0.85496	0.86116	0.86777	0.87498
301	0.83506	0.84767	0.85343	0.85533	0.86152	0.86811	0.87530
302	0.83549	0.84806	0.85380	0.85571	0.86187	0.86844	0.87561
303	0.83591	0.84842	0.85417	0.85607	0.86221	0.86877	0.87592
304	0.83634	0.84882	0.85454	0.85644	0.86256	0.86910	0.87623
305	0.83675	0.84920	0.85492	0.85680	0.86290	0.86943	0.87654
306	0.83716	0.84958	0.85529	0.85717	0.86325	0.86975	0.87685
307	0.83761	0.84996	0.85566	0.85752	0.86359	0.87007	0.87715
308	0.83803	0.85034	0.85603	0.85788	0.86393	0.87040	0.87745
309	0.83845	0.85072	0.85639	0.85824	0.86427	0.87072	0.87775
310	0.83883	0.85111	0.85675	0.85860	0.86461	0.87104	0.87805
311	0.83927	0.85148	0.85710	0.85894	0.86494	0.87135	0.87835
312	0.83968	0.85185	0.85747	0.85930	0.86528	0.87167	0.87865
313	0.84009	0.85222	0.85781	0.85965	0.86561	0.87199	0.87894
314	0.84050	0.85258	0.85816	0.86000	0.86594	0.87229	0.87924
315	0.84088	0.85295	0.85851	0.86035	0.86627	0.87261	0.87953
316	0.84127	0.85330	0.85885	0.86068	0.86659	0.87292	0.87982
317	0.84169	0.85366	0.85919	0.86102	0.86692	0.87322	0.88011
318	0.84208	0.85402	0.85953	0.86135	0.86724	0.87353	0.88040
319	0.84249	0.85439	0.85988	0.86169	0.86756	0.87383	0.88068
320	0.84289	0.85474	0.86022	0.86203	0.86788	0.87414	0.88097
321	0.84329	0.85509	0.86057	0.86237	0.86820	0.87444	0.88125
322	0.84362	0.85544	0.86090	0.86269	0.86852	0.87474	0.88153
323	0.84403	0.85580	0.86124	0.86302	0.86884	0.87504	0.88181
324	0.84443	0.85615	0.86157	0.86335	0.86915	0.87534	0.88209
325	0.84481	0.85649	0.86190	0.86367	0.86947	0.87563	0.88237
326	0.84517	0.85683	0.86222	0.86400	0.86977	0.87592	0.88264
327	0.84557	0.85720	0.86254	0.86433	0.87008	0.87622	0.88292
328	0.84593	0.85754	0.86287	0.86464	0.87038	0.87650	0.88319
329	0.84631	0.85786	0.86320	0.86496	0.87069	0.87679	0.88346
330	0.84666	0.85821	0.86352	0.86528	0.87099	0.87708	0.88373
331	0.84700	0.85854	0.86384	0.86560	0.87130	0.87737	0.88399
332	0.84738	0.85887	0.86415	0.86591	0.87159	0.87765	0.88426
333	0.84773	0.85921	0.86448	0.86622	0.87189	0.87793	0.88453
334	0.84806	0.85953	0.86479	0.86653	0.87218	0.87821	0.88479
335	0.84845	0.85986	0.86511	0.86683	0.87248	0.87849	0.88506
336	0.84879	0.86020	0.86542	0.86714	0.87278	0.87877	0.88531
337	0.84915	0.86052	0.86574	0.86746	0.87307	0.87905	0.88557
338	0.84947	0.86084	0.86604	0.86777	0.87336	0.87933	0.88583
339	0.84980	0.86117	0.86635	0.86808	0.87364	0.87960	0.88609
340	0.85014	0.86149	0.86666	0.86838	0.87393	0.87988	0.88635
341	0.85048	0.86179	0.86697	0.86868	0.87422	0.88015	0.88660
342	0.85084	0.86211	0.86727	0.86897	0.87451	0.88042	0.88685
343	0.85115	0.86242	0.86757	0.86928	0.87479	0.88068	0.88710
344	0.85149	0.86275	0.86787	0.86958	0.87507	0.88095	0.88736
345	0.85185	0.86306	0.86818	0.86988	0.87536	0.88122	0.88760
346	0.85218	0.86337	0.86846	0.87017	0.87563	0.88148	0.88785
347	0.85253	0.86368	0.86877	0.87047	0.87592	0.88175	0.88810
348	0.85286	0.86398	0.86907	0.87075	0.87619	0.88201	0.88834
349	0.85317	0.86430	0.86937	0.87105	0.87647	0.88227	0.88859
350	0.85353	0.86460	0.86965	0.87134	0.87675	0.88253	0.88883
351	0.85387	0.86490	0.86994	0.87162	0.87703	0.88279	0.88907
352	0.85419	0.86520	0.87022	0.87190	0.87729	0.88305	0.88932
353	0.85450	0.86550	0.87051	0.87218	0.87756	0.88330	0.88956
354	0.85483	0.86581	0.87080	0.87246	0.87783	0.88356	0.88980
355	0.85515	0.86611	0.87109	0.87275	0.87809	0.88381	0.89004
356	0.85550	0.86639	0.87137	0.87303	0.87836	0.88406	0.89028
357	0.85582	0.86669	0.87165	0.87331	0.87863	0.88432	0.89051
358	0.85615	0.86697	0.87194	0.87358	0.87889	0.88456	0.89074
359	0.85649	0.86726	0.87221	0.87386	0.87916	0.88481	0.89098
360	0.85679	0.86756	0.87249	0.87413	0.87942	0.88506	0.89121
361	0.85710	0.86785	0.87276	0.87440	0.87968	0.88531	0.89145
362	0.85744	0.86814	0.87304	0.87467	0.87994	0.88555	0.89168
363	0.85774	0.86841	0.87331	0.87494	0.88020	0.88579	0.89190
364	0.85804	0.86869	0.87357	0.87520	0.88045	0.88604	0.89213
365	0.85834	0.86897	0.87385	0.87547	0.88071	0.88628	0.89236
366	0.85866	0.86924	0.87412	0.87574	0.88096	0.88652	0.89258
367	0.85895	0.86953	0.87439	0.87601	0.88121	0.88676	0.89281
368	0.85926	0.86980	0.87466	0.87627	0.88147	0.88700	0.89303

N	0.1%	0.5%	1%	1.25%	2.5%	5%	10%
369	0.85956	0.87008	0.87493	0.87654	0.88172	0.88724	0.89325
370	0.85985	0.87036	0.87519	0.87680	0.88197	0.88748	0.89347
371	0.86019	0.87063	0.87546	0.87706	0.88222	0.88771	0.89369
372	0.86045	0.87091	0.87572	0.87731	0.88247	0.88795	0.89391
373	0.86074	0.87118	0.87598	0.87757	0.88271	0.88818	0.89414
374	0.86105	0.87146	0.87625	0.87783	0.88296	0.88841	0.89435
375	0.86135	0.87173	0.87651	0.87809	0.88320	0.88864	0.89457
376	0.86168	0.87201	0.87677	0.87834	0.88344	0.88887	0.89479
377	0.86199	0.87228	0.87702	0.87859	0.88369	0.88909	0.89500
378	0.86229	0.87254	0.87728	0.87885	0.88392	0.88932	0.89521
379	0.86255	0.87281	0.87754	0.87910	0.88416	0.88955	0.89542
380	0.86285	0.87308	0.87779	0.87935	0.88440	0.88978	0.89564
381	0.86312	0.87334	0.87804	0.87960	0.88464	0.89000	0.89585
382	0.86340	0.87360	0.87829	0.87984	0.88487	0.89022	0.89606
383	0.86368	0.87387	0.87854	0.88009	0.88511	0.89045	0.89626
384	0.86396	0.87413	0.87879	0.88035	0.88534	0.89067	0.89647
385	0.86423	0.87439	0.87904	0.88059	0.88558	0.89089	0.89668
386	0.86452	0.87465	0.87929	0.88083	0.88581	0.89111	0.89689
387	0.86479	0.87491	0.87953	0.88107	0.88604	0.89132	0.89709
388	0.86506	0.87516	0.87978	0.88131	0.88626	0.89154	0.89730
389	0.86537	0.87541	0.88002	0.88155	0.88649	0.89176	0.89750
390	0.86562	0.87567	0.88026	0.88179	0.88672	0.89197	0.89770
391	0.86591	0.87592	0.88050	0.88203	0.88695	0.89219	0.89790
392	0.86619	0.87617	0.88074	0.88227	0.88717	0.89240	0.89810
393	0.86646	0.87642	0.88098	0.88250	0.88739	0.89262	0.89830
394	0.86674	0.87667	0.88122	0.88273	0.88761	0.89283	0.89850
395	0.86699	0.87692	0.88146	0.88297	0.88784	0.89304	0.89870
396	0.86728	0.87716	0.88169	0.88320	0.88806	0.89325	0.89890
397	0.86754	0.87741	0.88192	0.88344	0.88827	0.89346	0.89910
398	0.86780	0.87767	0.88216	0.88367	0.88850	0.89367	0.89929
399	0.86807	0.87791	0.88240	0.88390	0.88872	0.89388	0.89949
400	0.86832	0.87815	0.88263	0.88414	0.88893	0.89408	0.89968
401	0.86859	0.87838	0.88286	0.88436	0.88915	0.89429	0.89988
402	0.86885	0.87862	0.88309	0.88459	0.88937	0.89450	0.90007
403	0.86911	0.87885	0.88333	0.88482	0.88958	0.89470	0.90026
404	0.86936	0.87910	0.88356	0.88504	0.88981	0.89490	0.90045
405	0.86962	0.87933	0.88379	0.88527	0.89002	0.89511	0.90064
406	0.86987	0.87957	0.88402	0.88549	0.89024	0.89531	0.90084
407	0.87013	0.87980	0.88425	0.88572	0.89045	0.89551	0.90102
408	0.87038	0.88004	0.88447	0.88594	0.89066	0.89571	0.90121
409	0.87066	0.88026	0.88470	0.88616	0.89087	0.89591	0.90140
410	0.87092	0.88050	0.88492	0.88637	0.89107	0.89611	0.90158
411	0.87115	0.88074	0.88514	0.88659	0.89128	0.89630	0.90177
412	0.87140	0.88096	0.88536	0.88680	0.89149	0.89650	0.90195
413	0.87164	0.88120	0.88558	0.88703	0.89169	0.89670	0.90214
414	0.87190	0.88142	0.88581	0.88724	0.89190	0.89689	0.90232
415	0.87211	0.88164	0.88602	0.88746	0.89210	0.89708	0.90250
416	0.87234	0.88187	0.88623	0.88767	0.89231	0.89728	0.90268
417	0.87259	0.88209	0.88644	0.88788	0.89251	0.89747	0.90286
418	0.87281	0.88232	0.88666	0.88809	0.89272	0.89766	0.90304
419	0.87306	0.88255	0.88687	0.88830	0.89292	0.89785	0.90322
420	0.87330	0.88276	0.88708	0.88851	0.89311	0.89804	0.90340
421	0.87354	0.88298	0.88729	0.88872	0.89331	0.89823	0.90358
422	0.87379	0.88320	0.88751	0.88893	0.89351	0.89842	0.90375
423	0.87401	0.88343	0.88771	0.88914	0.89370	0.89861	0.90393
424	0.87426	0.88364	0.88792	0.88934	0.89390	0.89879	0.90411
425	0.87451	0.88387	0.88814	0.88955	0.89411	0.89898	0.90428
426	0.87475	0.88409	0.88835	0.88976	0.89430	0.89916	0.90445
427	0.87498	0.88430	0.88855	0.88996	0.89449	0.89935	0.90463
428	0.87520	0.88452	0.88876	0.89016	0.89469	0.89954	0.90480
429	0.87544	0.88475	0.88897	0.89037	0.89488	0.89972	0.90497
430	0.87569	0.88497	0.88917	0.89057	0.89507	0.89990	0.90514
431	0.87590	0.88518	0.88938	0.89076	0.89526	0.90008	0.90531
432	0.87612	0.88539	0.88957	0.89097	0.89546	0.90026	0.90548
433	0.87637	0.88560	0.88978	0.89117	0.89565	0.90045	0.90565
434	0.87659	0.88581	0.88998	0.89137	0.89584	0.90062	0.90582
435	0.87682	0.88602	0.89018	0.89157	0.89603	0.90080	0.90599
436	0.87703	0.88622	0.89038	0.89176	0.89621	0.90098	0.90615
437	0.87726	0.88643	0.89058	0.89196	0.89640	0.90116	0.90632
438	0.87752	0.88663	0.89077	0.89216	0.89659	0.90134	0.90649
439	0.87773	0.88683	0.89098	0.89235	0.89678	0.90151	0.90665
440	0.87797	0.88703	0.89117	0.89255	0.89696	0.90168	0.90682
441	0.87821	0.88723	0.89137	0.89274	0.89714	0.90186	0.90698
442	0.87843	0.88744	0.89156	0.89293	0.89733	0.90203	0.90714

N	0.1%	0.5%	1%	1.25%	2.5%	5%	10%
443	0.87865	0.88764	0.89175	0.89311	0.89751	0.90221	0.90731
444	0.87888	0.88784	0.89194	0.89331	0.89770	0.90238	0.90747
445	0.87908	0.88805	0.89213	0.89350	0.89787	0.90255	0.90763
446	0.87930	0.88824	0.89233	0.89369	0.89805	0.90272	0.90779
447	0.87951	0.88844	0.89252	0.89388	0.89823	0.90289	0.90795
448	0.87972	0.88864	0.89271	0.89407	0.89841	0.90306	0.90811
449	0.87995	0.88884	0.89289	0.89425	0.89858	0.90323	0.90827
450	0.88018	0.88904	0.89308	0.89444	0.89876	0.90339	0.90843
451	0.88039	0.88924	0.89328	0.89462	0.89894	0.90356	0.90859
452	0.88061	0.88943	0.89347	0.89481	0.89912	0.90373	0.90874
453	0.88081	0.88963	0.89365	0.89499	0.89929	0.90389	0.90890
454	0.88104	0.88982	0.89384	0.89518	0.89946	0.90406	0.90906
455	0.88124	0.89002	0.89402	0.89536	0.89964	0.90423	0.90921
456	0.88145	0.89021	0.89421	0.89554	0.89981	0.90439	0.90937
457	0.88165	0.89039	0.89439	0.89572	0.89999	0.90456	0.90952
458	0.88185	0.89059	0.89458	0.89591	0.90016	0.90472	0.90968
459	0.88209	0.89077	0.89476	0.89609	0.90033	0.90488	0.90983
460	0.88228	0.89096	0.89494	0.89627	0.90050	0.90505	0.90998
461	0.88249	0.89113	0.89513	0.89645	0.90067	0.90521	0.91013
462	0.88269	0.89133	0.89531	0.89662	0.90085	0.90537	0.91029
463	0.88291	0.89152	0.89549	0.89680	0.90101	0.90553	0.91044
464	0.88311	0.89170	0.89567	0.89698	0.90118	0.90569	0.91059
465	0.88331	0.89190	0.89585	0.89715	0.90135	0.90585	0.91074
466	0.88352	0.89209	0.89602	0.89732	0.90152	0.90600	0.91089
467	0.88371	0.89227	0.89620	0.89750	0.90169	0.90616	0.91104
468	0.88391	0.89246	0.89638	0.89767	0.90185	0.90632	0.91118
469	0.88411	0.89265	0.89655	0.89785	0.90202	0.90648	0.91133
470	0.88430	0.89283	0.89673	0.89802	0.90218	0.90663	0.91148
471	0.88451	0.89301	0.89691	0.89820	0.90234	0.90678	0.91162
472	0.88470	0.89319	0.89708	0.89837	0.90251	0.90694	0.91177
473	0.88490	0.89337	0.89725	0.89854	0.90267	0.90709	0.91192
474	0.88509	0.89355	0.89742	0.89870	0.90283	0.90725	0.91206
475	0.88527	0.89373	0.89759	0.89888	0.90299	0.90740	0.91221
476	0.88546	0.89391	0.89777	0.89905	0.90316	0.90756	0.91236
477	0.88565	0.89409	0.89794	0.89922	0.90332	0.90771	0.91250
478	0.88585	0.89426	0.89811	0.89938	0.90348	0.90786	0.91264
479	0.88604	0.89444	0.89827	0.89955	0.90364	0.90801	0.91278
480	0.88624	0.89462	0.89844	0.89972	0.90380	0.90817	0.91293
481	0.88644	0.89480	0.89861	0.89989	0.90396	0.90832	0.91307
482	0.88663	0.89498	0.89879	0.90006	0.90411	0.90847	0.91321
483	0.88680	0.89515	0.89895	0.90022	0.90427	0.90861	0.91335
484	0.88701	0.89533	0.89912	0.90038	0.90443	0.90876	0.91349
485	0.88720	0.89550	0.89928	0.90055	0.90459	0.90891	0.91363
486	0.88739	0.89566	0.89944	0.90071	0.90474	0.90906	0.91377
487	0.88758	0.89584	0.89961	0.90087	0.90490	0.90921	0.91390
488	0.88778	0.89600	0.89978	0.90104	0.90505	0.90935	0.91404
489	0.88793	0.89616	0.89994	0.90119	0.90521	0.90950	0.91418
490	0.88812	0.89633	0.90010	0.90135	0.90536	0.90964	0.91432
491	0.88831	0.89650	0.90027	0.90151	0.90551	0.90979	0.91445
492	0.88850	0.89666	0.90042	0.90167	0.90567	0.90993	0.91459
493	0.88870	0.89683	0.90058	0.90183	0.90581	0.91008	0.91473
494	0.88887	0.89700	0.90074	0.90199	0.90597	0.91022	0.91486
495	0.88905	0.89717	0.90090	0.90214	0.90612	0.91036	0.91499
496	0.88922	0.89734	0.90106	0.90230	0.90627	0.91051	0.91513
497	0.88940	0.89751	0.90123	0.90246	0.90642	0.91065	0.91526
498	0.88958	0.89768	0.90138	0.90262	0.90657	0.91079	0.91539
499	0.88977	0.89784	0.90154	0.90278	0.90672	0.91093	0.91553
500	0.88995	0.89800	0.90171	0.90293	0.90687	0.91107	0.91566
501	0.89015	0.89817	0.90186	0.90309	0.90701	0.91121	0.91579
502	0.89032	0.89833	0.90202	0.90324	0.90716	0.91135	0.91592
503	0.89050	0.89850	0.90218	0.90340	0.90731	0.91149	0.91605
504	0.89069	0.89866	0.90233	0.90355	0.90745	0.91163	0.91618
505	0.89084	0.89882	0.90249	0.90370	0.90760	0.91177	0.91631
506	0.89102	0.89898	0.90264	0.90385	0.90775	0.91190	0.91644
507	0.89120	0.89914	0.90279	0.90400	0.90789	0.91204	0.91657
508	0.89138	0.89930	0.90295	0.90416	0.90803	0.91218	0.91670
509	0.89155	0.89947	0.90310	0.90431	0.90818	0.91232	0.91683
510	0.89173	0.89963	0.90326	0.90446	0.90832	0.91245	0.91696
511	0.89190	0.89978	0.90342	0.90461	0.90847	0.91259	0.91709
512	0.89208	0.89995	0.90357	0.90475	0.90861	0.91272	0.91721
513	0.89225	0.90010	0.90372	0.90490	0.90875	0.91285	0.91734
514	0.89240	0.90026	0.90387	0.90505	0.90889	0.91299	0.91746
515	0.89256	0.90041	0.90402	0.90520	0.90903	0.91312	0.91759
516	0.89276	0.90057	0.90416	0.90535	0.90917	0.91326	0.91771

N	0.1%	0.5%	1%	1.25%	2.5%	5%	10%
517	0.89293	0.90072	0.90431	0.90550	0.90931	0.91339	0.91784
518	0.89309	0.90088	0.90446	0.90564	0.90945	0.91353	0.91796
519	0.89326	0.90103	0.90461	0.90579	0.90959	0.91365	0.91809
520	0.89342	0.90118	0.90475	0.90593	0.90973	0.91379	0.91821
521	0.89359	0.90133	0.90490	0.90608	0.90987	0.91392	0.91833
522	0.89375	0.90149	0.90505	0.90622	0.91001	0.91405	0.91846
523	0.89393	0.90165	0.90519	0.90636	0.91015	0.91418	0.91858
524	0.89409	0.90181	0.90534	0.90651	0.91029	0.91431	0.91870
525	0.89426	0.90196	0.90548	0.90665	0.91042	0.91443	0.91882
526	0.89441	0.90211	0.90563	0.90679	0.91055	0.91456	0.91894
527	0.89458	0.90226	0.90577	0.90693	0.91069	0.91469	0.91906
528	0.89473	0.90240	0.90591	0.90707	0.91082	0.91482	0.91918
529	0.89490	0.90255	0.90605	0.90721	0.91095	0.91495	0.91930
530	0.89505	0.90270	0.90620	0.90734	0.91109	0.91508	0.91942
531	0.89521	0.90285	0.90634	0.90749	0.91123	0.91520	0.91954
532	0.89536	0.90299	0.90648	0.90763	0.91136	0.91533	0.91966
533	0.89554	0.90315	0.90662	0.90776	0.91149	0.91545	0.91978
534	0.89570	0.90330	0.90676	0.90790	0.91162	0.91558	0.91990
535	0.89585	0.90344	0.90690	0.90804	0.91176	0.91570	0.92001
536	0.89603	0.90358	0.90704	0.90818	0.91189	0.91583	0.92013
537	0.89618	0.90373	0.90718	0.90832	0.91202	0.91595	0.92025
538	0.89634	0.90387	0.90732	0.90846	0.91215	0.91607	0.92036
539	0.89650	0.90402	0.90746	0.90860	0.91228	0.91620	0.92048
540	0.89665	0.90416	0.90759	0.90873	0.91241	0.91632	0.92060
541	0.89680	0.90431	0.90773	0.90886	0.91254	0.91644	0.92071
542	0.89694	0.90446	0.90786	0.90900	0.91267	0.91657	0.92082
543	0.89710	0.90459	0.90800	0.90914	0.91279	0.91669	0.92094
544	0.89726	0.90473	0.90814	0.90927	0.91292	0.91681	0.92105
545	0.89742	0.90487	0.90828	0.90940	0.91305	0.91693	0.92117
546	0.89757	0.90501	0.90841	0.90953	0.91317	0.91705	0.92128
547	0.89773	0.90515	0.90855	0.90966	0.91330	0.91717	0.92140
548	0.89787	0.90528	0.90868	0.90979	0.91342	0.91729	0.92151
549	0.89803	0.90542	0.90881	0.90993	0.91355	0.91741	0.92162
550	0.89817	0.90557	0.90894	0.91006	0.91368	0.91753	0.92173
551	0.89833	0.90571	0.90907	0.91019	0.91380	0.91765	0.92184
552	0.89849	0.90584	0.90920	0.91032	0.91392	0.91777	0.92195
553	0.89863	0.90598	0.90935	0.91046	0.91405	0.91788	0.92207
554	0.89879	0.90611	0.90947	0.91058	0.91417	0.91800	0.92218
555	0.89894	0.90625	0.90961	0.91071	0.91430	0.91812	0.92228
556	0.89908	0.90639	0.90974	0.91084	0.91442	0.91823	0.92239
557	0.89925	0.90653	0.90987	0.91097	0.91454	0.91835	0.92250
558	0.89939	0.90666	0.91000	0.91110	0.91467	0.91846	0.92261
559	0.89954	0.90680	0.91013	0.91123	0.91479	0.91858	0.92272
560	0.89969	0.90693	0.91026	0.91136	0.91491	0.91870	0.92283
561	0.89982	0.90707	0.91039	0.91148	0.91503	0.91881	0.92294
562	0.89995	0.90720	0.91052	0.91162	0.91515	0.91893	0.92305
563	0.90011	0.90734	0.91065	0.91174	0.91527	0.91904	0.92316
564	0.90026	0.90748	0.91078	0.91187	0.91539	0.91916	0.92326
565	0.90040	0.90761	0.91090	0.91199	0.91551	0.91927	0.92337
566	0.90056	0.90774	0.91102	0.91212	0.91563	0.91938	0.92348
567	0.90071	0.90788	0.91115	0.91224	0.91575	0.91949	0.92358
568	0.90086	0.90801	0.91127	0.91236	0.91586	0.91961	0.92369
569	0.90100	0.90814	0.91140	0.91249	0.91598	0.91972	0.92380
570	0.90115	0.90827	0.91153	0.91261	0.91610	0.91983	0.92390
571	0.90128	0.90840	0.91165	0.91273	0.91622	0.91994	0.92401
572	0.90142	0.90853	0.91178	0.91286	0.91634	0.92005	0.92411
573	0.90156	0.90866	0.91190	0.91298	0.91645	0.92016	0.92421
574	0.90170	0.90878	0.91203	0.91311	0.91657	0.92028	0.92432
575	0.90183	0.90890	0.91215	0.91323	0.91669	0.92039	0.92442
576	0.90196	0.90903	0.91227	0.91335	0.91680	0.92050	0.92452
577	0.90211	0.90915	0.91239	0.91347	0.91692	0.92061	0.92463
578	0.90225	0.90928	0.91251	0.91359	0.91703	0.92071	0.92473
579	0.90238	0.90941	0.91264	0.91371	0.91715	0.92083	0.92483
580	0.90251	0.90953	0.91276	0.91384	0.91726	0.92094	0.92494
581	0.90266	0.90965	0.91289	0.91396	0.91737	0.92104	0.92504
582	0.90280	0.90979	0.91301	0.91408	0.91749	0.92115	0.92514
583	0.90293	0.90991	0.91313	0.91419	0.91760	0.92126	0.92524
584	0.90307	0.91003	0.91325	0.91431	0.91771	0.92136	0.92534
585	0.90321	0.91016	0.91337	0.91443	0.91783	0.92147	0.92544
586	0.90334	0.91028	0.91349	0.91455	0.91794	0.92158	0.92554
587	0.90346	0.91040	0.91361	0.91466	0.91805	0.92168	0.92564
588	0.90359	0.91053	0.91373	0.91478	0.91817	0.92179	0.92574
589	0.90373	0.91066	0.91385	0.91490	0.91828	0.92189	0.92584
590	0.90387	0.91078	0.91397	0.91501	0.91839	0.92200	0.92594

N	0.1%	0.5%	1%	1.25%	2.5%	5%	10%
591	0.90399	0.91091	0.91408	0.91513	0.91850	0.92211	0.92604
592	0.90412	0.91103	0.91419	0.91524	0.91861	0.92221	0.92614
593	0.90427	0.91116	0.91431	0.91536	0.91872	0.92231	0.92624
594	0.90440	0.91128	0.91443	0.91547	0.91883	0.92242	0.92633
595	0.90455	0.91140	0.91454	0.91558	0.91894	0.92252	0.92643
596	0.90468	0.91152	0.91466	0.91570	0.91905	0.92263	0.92653
597	0.90480	0.91164	0.91478	0.91581	0.91916	0.92273	0.92663
598	0.90494	0.91176	0.91489	0.91593	0.91927	0.92283	0.92672
599	0.90507	0.91187	0.91500	0.91605	0.91937	0.92294	0.92682
600	0.90520	0.91200	0.91512	0.91616	0.91948	0.92304	0.92692
601	0.90531	0.91211	0.91523	0.91627	0.91959	0.92314	0.92701
602	0.90543	0.91223	0.91535	0.91638	0.91970	0.92324	0.92711
603	0.90556	0.91235	0.91546	0.91649	0.91980	0.92334	0.92721
604	0.90568	0.91246	0.91557	0.91660	0.91991	0.92344	0.92730
605	0.90580	0.91258	0.91569	0.91671	0.92001	0.92354	0.92740
606	0.90594	0.91269	0.91580	0.91682	0.92012	0.92364	0.92749
607	0.90606	0.91281	0.91591	0.91693	0.92022	0.92374	0.92758
608	0.90618	0.91292	0.91602	0.91704	0.92033	0.92384	0.92768
609	0.90630	0.91304	0.91613	0.91715	0.92044	0.92394	0.92777
610	0.90641	0.91315	0.91625	0.91727	0.92054	0.92405	0.92787
611	0.90656	0.91327	0.91636	0.91738	0.92064	0.92415	0.92796
612	0.90668	0.91339	0.91647	0.91749	0.92075	0.92424	0.92805
613	0.90679	0.91350	0.91658	0.91760	0.92085	0.92434	0.92815
614	0.90691	0.91361	0.91669	0.91770	0.92096	0.92444	0.92824
615	0.90705	0.91373	0.91680	0.91781	0.92106	0.92454	0.92833
616	0.90717	0.91384	0.91691	0.91792	0.92116	0.92464	0.92842
617	0.90730	0.91395	0.91702	0.91803	0.92127	0.92473	0.92851
618	0.90742	0.91407	0.91713	0.91814	0.92137	0.92483	0.92860
619	0.90754	0.91418	0.91724	0.91825	0.92148	0.92493	0.92870
620	0.90767	0.91430	0.91735	0.91835	0.92158	0.92502	0.92879
621	0.90779	0.91441	0.91746	0.91846	0.92168	0.92512	0.92888
622	0.90791	0.91452	0.91756	0.91856	0.92178	0.92522	0.92897
623	0.90803	0.91464	0.91767	0.91867	0.92188	0.92531	0.92906
624	0.90815	0.91475	0.91778	0.91878	0.92198	0.92541	0.92915
625	0.90827	0.91486	0.91788	0.91888	0.92208	0.92550	0.92924
626	0.90839	0.91497	0.91799	0.91899	0.92218	0.92560	0.92933
627	0.90851	0.91508	0.91809	0.91909	0.92228	0.92569	0.92942
628	0.90862	0.91519	0.91820	0.91920	0.92238	0.92579	0.92951
629	0.90874	0.91530	0.91831	0.91930	0.92248	0.92588	0.92960
630	0.90886	0.91540	0.91841	0.91940	0.92258	0.92598	0.92969
631	0.90897	0.91552	0.91852	0.91951	0.92267	0.92607	0.92978
632	0.90911	0.91562	0.91862	0.91961	0.92277	0.92616	0.92986
633	0.90922	0.91573	0.91873	0.91972	0.92287	0.92626	0.92995
634	0.90933	0.91584	0.91883	0.91982	0.92297	0.92635	0.93004
635	0.90945	0.91595	0.91893	0.91992	0.92306	0.92644	0.93013
636	0.90958	0.91606	0.91904	0.92002	0.92316	0.92653	0.93021
637	0.90970	0.91616	0.91914	0.92012	0.92326	0.92662	0.93030
638	0.90981	0.91627	0.91924	0.92022	0.92336	0.92671	0.93038
639	0.90994	0.91638	0.91935	0.92032	0.92346	0.92681	0.93047
640	0.91005	0.91649	0.91944	0.92042	0.92355	0.92690	0.93056
641	0.91015	0.91659	0.91955	0.92052	0.92365	0.92699	0.93064
642	0.91026	0.91670	0.91965	0.92062	0.92374	0.92708	0.93073
643	0.91037	0.91680	0.91975	0.92072	0.92384	0.92717	0.93081
644	0.91048	0.91691	0.91985	0.92082	0.92393	0.92726	0.93090
645	0.91060	0.91701	0.91995	0.92092	0.92403	0.92735	0.93098
646	0.91073	0.91712	0.92005	0.92101	0.92412	0.92744	0.93107
647	0.91083	0.91722	0.92014	0.92111	0.92422	0.92753	0.93115
648	0.91094	0.91733	0.92025	0.92122	0.92431	0.92762	0.93124
649	0.91106	0.91743	0.92035	0.92131	0.92440	0.92771	0.93132
650	0.91116	0.91753	0.92045	0.92141	0.92450	0.92780	0.93140
651	0.91128	0.91764	0.92055	0.92151	0.92459	0.92789	0.93149
652	0.91139	0.91774	0.92064	0.92161	0.92468	0.92798	0.93157
653	0.91151	0.91784	0.92075	0.92171	0.92477	0.92806	0.93165
654	0.91162	0.91794	0.92084	0.92180	0.92487	0.92815	0.93174
655	0.91174	0.91805	0.92094	0.92190	0.92496	0.92824	0.93182
656	0.91186	0.91814	0.92104	0.92200	0.92505	0.92833	0.93190
657	0.91197	0.91825	0.92113	0.92209	0.92515	0.92841	0.93198
658	0.91208	0.91835	0.92123	0.92219	0.92524	0.92850	0.93207
659	0.91220	0.91845	0.92133	0.92229	0.92533	0.92859	0.93215
660	0.91230	0.91854	0.92142	0.92238	0.92542	0.92868	0.93223
661	0.91241	0.91865	0.92152	0.92248	0.92551	0.92876	0.93231
662	0.91251	0.91875	0.92162	0.92257	0.92560	0.92885	0.93239
663	0.91262	0.91885	0.92172	0.92267	0.92569	0.92893	0.93247
664	0.91274	0.91895	0.92181	0.92276	0.92578	0.92902	0.93255

N	0.1%	0.5%	1%	1.25%	2.5%	5%	10%
665	0.91285	0.91905	0.92191	0.92286	0.92587	0.92911	0.93264
666	0.91296	0.91915	0.92200	0.92295	0.92596	0.92919	0.93272
667	0.91306	0.91925	0.92210	0.92304	0.92605	0.92927	0.93280
668	0.91316	0.91935	0.92219	0.92314	0.92614	0.92936	0.93288
669	0.91327	0.91945	0.92228	0.92323	0.92623	0.92944	0.93295
670	0.91337	0.91955	0.92238	0.92332	0.92632	0.92953	0.93303
671	0.91347	0.91964	0.92247	0.92341	0.92641	0.92961	0.93311
672	0.91357	0.91975	0.92256	0.92350	0.92649	0.92970	0.93319
673	0.91368	0.91984	0.92266	0.92359	0.92659	0.92978	0.93327
674	0.91378	0.91994	0.92275	0.92369	0.92668	0.92986	0.93335
675	0.91389	0.92004	0.92285	0.92378	0.92676	0.92995	0.93343
676	0.91401	0.92014	0.92294	0.92387	0.92685	0.93003	0.93350
677	0.91411	0.92024	0.92303	0.92396	0.92694	0.93011	0.93358
678	0.91421	0.92033	0.92312	0.92405	0.92702	0.93019	0.93366
679	0.91431	0.92042	0.92322	0.92414	0.92711	0.93028	0.93374
680	0.91440	0.92052	0.92331	0.92423	0.92719	0.93036	0.93382
681	0.91451	0.92062	0.92340	0.92432	0.92728	0.93044	0.93389
682	0.91462	0.92071	0.92349	0.92441	0.92737	0.93052	0.93397
683	0.91472	0.92080	0.92358	0.92450	0.92746	0.93061	0.93405
684	0.91483	0.92090	0.92367	0.92459	0.92754	0.93069	0.93412
685	0.91495	0.92099	0.92376	0.92468	0.92762	0.93077	0.93420
686	0.91505	0.92109	0.92385	0.92477	0.92771	0.93085	0.93428
687	0.91515	0.92118	0.92394	0.92485	0.92779	0.93093	0.93435
688	0.91526	0.92128	0.92404	0.92494	0.92788	0.93101	0.93443
689	0.91534	0.92137	0.92412	0.92503	0.92796	0.93109	0.93450
690	0.91545	0.92146	0.92421	0.92512	0.92805	0.93117	0.93458
691	0.91555	0.92155	0.92430	0.92521	0.92813	0.93125	0.93465
692	0.91565	0.92164	0.92439	0.92530	0.92821	0.93133	0.93473
693	0.91575	0.92173	0.92447	0.92538	0.92830	0.93141	0.93480
694	0.91585	0.92182	0.92456	0.92547	0.92838	0.93149	0.93488
695	0.91595	0.92192	0.92465	0.92556	0.92846	0.93157	0.93495
696	0.91604	0.92201	0.92474	0.92565	0.92855	0.93165	0.93503
697	0.91614	0.92211	0.92483	0.92574	0.92863	0.93173	0.93510
698	0.91623	0.92220	0.92491	0.92582	0.92871	0.93181	0.93518
699	0.91634	0.92228	0.92500	0.92591	0.92879	0.93189	0.93525
700	0.91643	0.92237	0.92509	0.92600	0.92888	0.93196	0.93532
701	0.91653	0.92246	0.92518	0.92608	0.92896	0.93204	0.93540
702	0.91664	0.92256	0.92527	0.92617	0.92904	0.93212	0.93547
703	0.91674	0.92265	0.92535	0.92625	0.92913	0.93220	0.93554
704	0.91683	0.92275	0.92544	0.92634	0.92921	0.93227	0.93562
705	0.91694	0.92283	0.92553	0.92643	0.92929	0.93235	0.93569
706	0.91703	0.92293	0.92562	0.92651	0.92937	0.93243	0.93576
707	0.91714	0.92301	0.92570	0.92659	0.92945	0.93250	0.93584
708	0.91723	0.92310	0.92578	0.92668	0.92953	0.93258	0.93591
709	0.91733	0.92319	0.92587	0.92676	0.92961	0.93266	0.93598
710	0.91742	0.92328	0.92595	0.92685	0.92969	0.93273	0.93605
711	0.91751	0.92337	0.92603	0.92693	0.92977	0.93281	0.93612
712	0.91759	0.92345	0.92612	0.92701	0.92985	0.93289	0.93619
713	0.91769	0.92354	0.92621	0.92709	0.92993	0.93296	0.93626
714	0.91779	0.92363	0.92629	0.92718	0.93001	0.93304	0.93633
715	0.91789	0.92372	0.92637	0.92726	0.93008	0.93311	0.93641
716	0.91798	0.92380	0.92646	0.92734	0.93016	0.93319	0.93648
717	0.91808	0.92389	0.92654	0.92743	0.93024	0.93326	0.93655
718	0.91817	0.92397	0.92662	0.92751	0.93032	0.93334	0.93662
719	0.91827	0.92406	0.92670	0.92759	0.93040	0.93341	0.93669
720	0.91836	0.92415	0.92678	0.92767	0.93048	0.93348	0.93676
721	0.91846	0.92423	0.92687	0.92775	0.93056	0.93356	0.93683
722	0.91856	0.92432	0.92695	0.92783	0.93064	0.93363	0.93690
723	0.91864	0.92440	0.92703	0.92791	0.93071	0.93371	0.93697
724	0.91873	0.92449	0.92711	0.92799	0.93079	0.93378	0.93704
725	0.91883	0.92458	0.92719	0.92807	0.93087	0.93385	0.93711
726	0.91893	0.92466	0.92727	0.92815	0.93095	0.93393	0.93718
727	0.91902	0.92474	0.92735	0.92823	0.93102	0.93400	0.93724
728	0.91911	0.92483	0.92744	0.92831	0.93110	0.93407	0.93731
729	0.91921	0.92491	0.92752	0.92839	0.93117	0.93415	0.93738
730	0.91930	0.92500	0.92760	0.92847	0.93125	0.93422	0.93745
731	0.91939	0.92509	0.92768	0.92855	0.93132	0.93429	0.93752
732	0.91949	0.92517	0.92776	0.92863	0.93140	0.93437	0.93759
733	0.91957	0.92525	0.92785	0.92871	0.93148	0.93444	0.93766
734	0.91966	0.92534	0.92793	0.92879	0.93155	0.93451	0.93772
735	0.91975	0.92543	0.92801	0.92887	0.93163	0.93458	0.93779
736	0.91984	0.92551	0.92809	0.92895	0.93170	0.93465	0.93786
737	0.91993	0.92559	0.92816	0.92903	0.93178	0.93472	0.93793
738	0.92002	0.92567	0.92824	0.92911	0.93185	0.93480	0.93799

N	0.1%	0.5%	1%	1.25%	2.5%	5%	10%
739	0.92010	0.92575	0.92833	0.92919	0.93193	0.93487	0.93806
740	0.92019	0.92584	0.92840	0.92926	0.93200	0.93494	0.93813
741	0.92027	0.92592	0.92848	0.92934	0.93208	0.93501	0.93819
742	0.92036	0.92600	0.92856	0.92942	0.93215	0.93508	0.93826
743	0.92046	0.92608	0.92864	0.92949	0.93222	0.93515	0.93833
744	0.92053	0.92616	0.92872	0.92957	0.93230	0.93522	0.93839
745	0.92062	0.92624	0.92879	0.92965	0.93237	0.93529	0.93846
746	0.92071	0.92632	0.92887	0.92973	0.93245	0.93536	0.93852
747	0.92080	0.92641	0.92895	0.92980	0.93252	0.93543	0.93859
748	0.92088	0.92649	0.92903	0.92988	0.93259	0.93550	0.93865
749	0.92097	0.92657	0.92910	0.92995	0.93267	0.93557	0.93872
750	0.92105	0.92665	0.92918	0.93003	0.93274	0.93564	0.93878
751	0.92115	0.92673	0.92926	0.93010	0.93281	0.93570	0.93885
752	0.92124	0.92681	0.92934	0.93018	0.93288	0.93577	0.93892
753	0.92132	0.92690	0.92942	0.93026	0.93296	0.93584	0.93898
754	0.92141	0.92698	0.92949	0.93033	0.93303	0.93591	0.93905
755	0.92149	0.92705	0.92957	0.93041	0.93310	0.93598	0.93911
756	0.92157	0.92713	0.92964	0.93048	0.93317	0.93605	0.93917
757	0.92167	0.92721	0.92972	0.93056	0.93324	0.93611	0.93924
758	0.92175	0.92729	0.92980	0.93063	0.93331	0.93618	0.93930
759	0.92183	0.92736	0.92987	0.93070	0.93339	0.93625	0.93937
760	0.92192	0.92744	0.92994	0.93078	0.93346	0.93632	0.93943
761	0.92200	0.92752	0.93002	0.93085	0.93353	0.93639	0.93949
762	0.92209	0.92760	0.93009	0.93093	0.93360	0.93645	0.93956
763	0.92217	0.92768	0.93017	0.93100	0.93367	0.93652	0.93962
764	0.92225	0.92775	0.93025	0.93108	0.93374	0.93659	0.93968
765	0.92234	0.92783	0.93032	0.93115	0.93381	0.93665	0.93975
766	0.92242	0.92791	0.93039	0.93122	0.93388	0.93672	0.93981
767	0.92250	0.92799	0.93046	0.93129	0.93395	0.93678	0.93987
768	0.92258	0.92806	0.93054	0.93137	0.93402	0.93685	0.93994
769	0.92266	0.92814	0.93061	0.93144	0.93409	0.93692	0.94000
770	0.92274	0.92821	0.93068	0.93151	0.93416	0.93698	0.94006
771	0.92283	0.92829	0.93076	0.93158	0.93423	0.93705	0.94012
772	0.92291	0.92836	0.93083	0.93166	0.93430	0.93712	0.94018
773	0.92299	0.92844	0.93090	0.93173	0.93437	0.93718	0.94025
774	0.92308	0.92851	0.93098	0.93180	0.93444	0.93725	0.94031
775	0.92316	0.92859	0.93105	0.93188	0.93450	0.93731	0.94037
776	0.92324	0.92866	0.93112	0.93195	0.93457	0.93738	0.94043
777	0.92333	0.92874	0.93120	0.93202	0.93464	0.93744	0.94049
778	0.92341	0.92882	0.93127	0.93209	0.93471	0.93751	0.94055
779	0.92349	0.92889	0.93134	0.93216	0.93478	0.93757	0.94062
780	0.92357	0.92896	0.93141	0.93223	0.93484	0.93763	0.94068
781	0.92365	0.92904	0.93148	0.93230	0.93491	0.93770	0.94074
782	0.92373	0.92912	0.93156	0.93237	0.93498	0.93776	0.94080
783	0.92381	0.92919	0.93163	0.93244	0.93505	0.93783	0.94086
784	0.92389	0.92926	0.93170	0.93251	0.93511	0.93789	0.94092
785	0.92398	0.92934	0.93177	0.93258	0.93518	0.93796	0.94098
786	0.92405	0.92941	0.93184	0.93265	0.93525	0.93802	0.94104
787	0.92414	0.92949	0.93191	0.93272	0.93531	0.93808	0.94110
788	0.92421	0.92956	0.93198	0.93279	0.93538	0.93815	0.94116
789	0.92429	0.92963	0.93205	0.93286	0.93544	0.93821	0.94122
790	0.92438	0.92971	0.93212	0.93292	0.93551	0.93827	0.94128
791	0.92446	0.92978	0.93219	0.93299	0.93558	0.93833	0.94134
792	0.92454	0.92986	0.93226	0.93307	0.93564	0.93840	0.94140
793	0.92461	0.92993	0.93233	0.93314	0.93571	0.93846	0.94146
794	0.92468	0.93000	0.93240	0.93321	0.93578	0.93852	0.94151
795	0.92476	0.93007	0.93247	0.93327	0.93584	0.93859	0.94157
796	0.92484	0.93014	0.93253	0.93334	0.93591	0.93865	0.94163
797	0.92491	0.93022	0.93260	0.93341	0.93597	0.93871	0.94169
798	0.92499	0.93029	0.93268	0.93348	0.93604	0.93877	0.94175
799	0.92507	0.93036	0.93274	0.93355	0.93610	0.93883	0.94181
800	0.92515	0.93042	0.93281	0.93361	0.93617	0.93889	0.94187
801	0.92523	0.93049	0.93288	0.93368	0.93623	0.93896	0.94193
802	0.92530	0.93057	0.93295	0.93375	0.93630	0.93902	0.94198
803	0.92538	0.93064	0.93301	0.93382	0.93636	0.93908	0.94204
804	0.92546	0.93071	0.93308	0.93388	0.93642	0.93914	0.94210
805	0.92554	0.93078	0.93316	0.93395	0.93649	0.93920	0.94216
806	0.92562	0.93086	0.93322	0.93402	0.93655	0.93926	0.94221
807	0.92569	0.93093	0.93329	0.93408	0.93661	0.93932	0.94227
808	0.92577	0.93100	0.93336	0.93415	0.93668	0.93938	0.94233
809	0.92584	0.93107	0.93343	0.93422	0.93674	0.93944	0.94239
810	0.92591	0.93114	0.93349	0.93428	0.93681	0.93950	0.94244
811	0.92600	0.93121	0.93356	0.93435	0.93687	0.93956	0.94250
812	0.92607	0.93128	0.93363	0.93441	0.93693	0.93963	0.94256

N	0.1%	0.5%	1%	1.25%	2.5%	5%	10%
813	0.92614	0.93135	0.93369	0.93448	0.93700	0.93968	0.94261
814	0.92622	0.93142	0.93376	0.93454	0.93706	0.93974	0.94267
815	0.92630	0.93149	0.93382	0.93461	0.93712	0.93980	0.94272
816	0.92638	0.93156	0.93389	0.93468	0.93718	0.93986	0.94278
817	0.92645	0.93163	0.93396	0.93474	0.93725	0.93992	0.94284
818	0.92652	0.93170	0.93402	0.93481	0.93731	0.93998	0.94289
819	0.92661	0.93177	0.93409	0.93487	0.93737	0.94004	0.94295
820	0.92668	0.93183	0.93415	0.93493	0.93743	0.94010	0.94300
821	0.92675	0.93190	0.93422	0.93500	0.93749	0.94016	0.94306
822	0.92683	0.93196	0.93428	0.93507	0.93755	0.94021	0.94312
823	0.92690	0.93203	0.93435	0.93513	0.93762	0.94027	0.94317
824	0.92697	0.93210	0.93442	0.93519	0.93768	0.94033	0.94323
825	0.92705	0.93217	0.93448	0.93526	0.93774	0.94039	0.94328
826	0.92712	0.93223	0.93455	0.93532	0.93780	0.94045	0.94334
827	0.92719	0.93230	0.93461	0.93539	0.93786	0.94051	0.94339
828	0.92727	0.93237	0.93468	0.93545	0.93792	0.94057	0.94345
829	0.92734	0.93243	0.93474	0.93551	0.93798	0.94062	0.94350
830	0.92741	0.93250	0.93481	0.93557	0.93804	0.94068	0.94356
831	0.92748	0.93257	0.93487	0.93564	0.93810	0.94074	0.94361
832	0.92755	0.93264	0.93494	0.93570	0.93816	0.94079	0.94367
833	0.92763	0.93270	0.93500	0.93577	0.93822	0.94085	0.94372
834	0.92769	0.93277	0.93506	0.93583	0.93828	0.94091	0.94377
835	0.92777	0.93283	0.93513	0.93589	0.93834	0.94097	0.94383
836	0.92784	0.93290	0.93519	0.93596	0.93841	0.94102	0.94388
837	0.92791	0.93296	0.93525	0.93602	0.93846	0.94108	0.94394
838	0.92799	0.93303	0.93532	0.93608	0.93853	0.94114	0.94399
839	0.92807	0.93309	0.93538	0.93614	0.93859	0.94120	0.94404
840	0.92813	0.93316	0.93544	0.93620	0.93865	0.94125	0.94410
841	0.92820	0.93323	0.93551	0.93627	0.93871	0.94131	0.94415
842	0.92827	0.93329	0.93557	0.93633	0.93876	0.94137	0.94420
843	0.92835	0.93335	0.93563	0.93639	0.93883	0.94142	0.94426
844	0.92842	0.93342	0.93570	0.93645	0.93888	0.94148	0.94431
845	0.92849	0.93348	0.93576	0.93651	0.93894	0.94153	0.94436
846	0.92856	0.93355	0.93582	0.93658	0.93900	0.94159	0.94442
847	0.92862	0.93361	0.93589	0.93664	0.93906	0.94165	0.94447
848	0.92869	0.93368	0.93595	0.93670	0.93912	0.94170	0.94452
849	0.92876	0.93374	0.93601	0.93676	0.93918	0.94176	0.94457
850	0.92884	0.93380	0.93607	0.93682	0.93923	0.94181	0.94462
851	0.92891	0.93387	0.93613	0.93688	0.93929	0.94187	0.94468
852	0.92898	0.93393	0.93619	0.93694	0.93935	0.94192	0.94473
853	0.92904	0.93400	0.93626	0.93700	0.93941	0.94198	0.94478
854	0.92911	0.93406	0.93632	0.93706	0.93947	0.94203	0.94483
855	0.92918	0.93412	0.93638	0.93712	0.93952	0.94209	0.94489
856	0.92926	0.93419	0.93644	0.93718	0.93958	0.94214	0.94494
857	0.92932	0.93425	0.93650	0.93724	0.93964	0.94220	0.94499
858	0.92938	0.93431	0.93656	0.93730	0.93970	0.94225	0.94504
859	0.92946	0.93438	0.93662	0.93736	0.93976	0.94231	0.94509
860	0.92953	0.93444	0.93668	0.93742	0.93981	0.94236	0.94514
861	0.92960	0.93450	0.93674	0.93748	0.93987	0.94242	0.94520
862	0.92967	0.93457	0.93680	0.93754	0.93992	0.94247	0.94525
863	0.92973	0.93463	0.93686	0.93760	0.93998	0.94252	0.94530
864	0.92980	0.93469	0.93692	0.93766	0.94004	0.94258	0.94535
865	0.92987	0.93475	0.93698	0.93772	0.94010	0.94263	0.94540
866	0.92995	0.93482	0.93704	0.93778	0.94015	0.94269	0.94545
867	0.93001	0.93488	0.93710	0.93784	0.94021	0.94274	0.94550
868	0.93008	0.93494	0.93716	0.93790	0.94027	0.94279	0.94555
869	0.93015	0.93500	0.93722	0.93795	0.94032	0.94285	0.94560
870	0.93022	0.93506	0.93728	0.93801	0.94038	0.94290	0.94565
871	0.93028	0.93512	0.93734	0.93807	0.94043	0.94295	0.94570
872	0.93035	0.93518	0.93740	0.93813	0.94049	0.94301	0.94575
873	0.93041	0.93524	0.93746	0.93819	0.94054	0.94306	0.94580
874	0.93048	0.93531	0.93752	0.93824	0.94060	0.94311	0.94585
875	0.93054	0.93537	0.93758	0.93830	0.94066	0.94317	0.94590
876	0.93060	0.93542	0.93764	0.93836	0.94071	0.94322	0.94595
877	0.93067	0.93549	0.93770	0.93842	0.94077	0.94327	0.94600
878	0.93074	0.93555	0.93775	0.93848	0.94082	0.94332	0.94605
879	0.93081	0.93561	0.93781	0.93853	0.94088	0.94338	0.94610
880	0.93087	0.93567	0.93787	0.93859	0.94093	0.94343	0.94615
881	0.93094	0.93573	0.93793	0.93865	0.94099	0.94348	0.94620
882	0.93099	0.93579	0.93799	0.93871	0.94104	0.94353	0.94625
883	0.93106	0.93585	0.93804	0.93877	0.94110	0.94358	0.94630
884	0.93112	0.93591	0.93810	0.93882	0.94115	0.94364	0.94635
885	0.93119	0.93597	0.93816	0.93888	0.94120	0.94369	0.94640
886	0.93126	0.93603	0.93822	0.93894	0.94126	0.94374	0.94645

N	0.1%	0.5%	1%	1.25%	2.5%	5%	10%
887	0.93132	0.93609	0.93827	0.93899	0.94132	0.94379	0.94649
888	0.93139	0.93615	0.93833	0.93905	0.94137	0.94384	0.94654
889	0.93146	0.93622	0.93839	0.93911	0.94142	0.94389	0.94659
890	0.93153	0.93628	0.93845	0.93916	0.94148	0.94394	0.94664
891	0.93158	0.93634	0.93850	0.93922	0.94153	0.94400	0.94669
892	0.93164	0.93639	0.93856	0.93927	0.94158	0.94405	0.94674
893	0.93170	0.93646	0.93861	0.93933	0.94163	0.94410	0.94679
894	0.93177	0.93651	0.93867	0.93938	0.94169	0.94415	0.94683
895	0.93183	0.93657	0.93873	0.93944	0.94174	0.94420	0.94688
896	0.93189	0.93663	0.93879	0.93950	0.94179	0.94425	0.94693
897	0.93195	0.93669	0.93884	0.93955	0.94185	0.94430	0.94698
898	0.93202	0.93674	0.93890	0.93961	0.94190	0.94435	0.94702
899	0.93208	0.93680	0.93895	0.93966	0.94195	0.94440	0.94707
900	0.93214	0.93686	0.93901	0.93972	0.94201	0.94445	0.94712
901	0.93220	0.93692	0.93907	0.93978	0.94206	0.94450	0.94717
902	0.93227	0.93698	0.93912	0.93983	0.94211	0.94455	0.94721
903	0.93232	0.93703	0.93918	0.93989	0.94216	0.94460	0.94726
904	0.93239	0.93709	0.93923	0.93994	0.94222	0.94465	0.94731
905	0.93246	0.93715	0.93929	0.93999	0.94227	0.94470	0.94735
906	0.93252	0.93721	0.93934	0.94005	0.94232	0.94475	0.94740
907	0.93258	0.93727	0.93939	0.94010	0.94237	0.94480	0.94745
908	0.93264	0.93732	0.93945	0.94016	0.94242	0.94485	0.94749
909	0.93271	0.93738	0.93950	0.94021	0.94248	0.94490	0.94754
910	0.93277	0.93744	0.93956	0.94027	0.94253	0.94495	0.94759
911	0.93283	0.93749	0.93961	0.94032	0.94258	0.94500	0.94763
912	0.93289	0.93755	0.93967	0.94037	0.94263	0.94504	0.94768
913	0.93295	0.93761	0.93972	0.94043	0.94268	0.94509	0.94772
914	0.93302	0.93767	0.93978	0.94048	0.94273	0.94514	0.94777
915	0.93308	0.93772	0.93983	0.94053	0.94278	0.94519	0.94782
916	0.93313	0.93778	0.93988	0.94058	0.94283	0.94524	0.94786
917	0.93319	0.93783	0.93994	0.94064	0.94289	0.94529	0.94791
918	0.93325	0.93789	0.93999	0.94069	0.94294	0.94534	0.94795
919	0.93331	0.93794	0.94004	0.94074	0.94299	0.94538	0.94800
920	0.93337	0.93800	0.94010	0.94080	0.94304	0.94543	0.94805
921	0.93344	0.93806	0.94015	0.94085	0.94309	0.94548	0.94809
922	0.93350	0.93811	0.94020	0.94090	0.94314	0.94553	0.94814
923	0.93356	0.93817	0.94026	0.94095	0.94319	0.94558	0.94818
924	0.93362	0.93822	0.94031	0.94101	0.94324	0.94563	0.94823
925	0.93368	0.93828	0.94036	0.94106	0.94329	0.94567	0.94827
926	0.93375	0.93834	0.94042	0.94111	0.94334	0.94572	0.94832
927	0.93380	0.93839	0.94047	0.94116	0.94339	0.94577	0.94836
928	0.93386	0.93845	0.94052	0.94122	0.94344	0.94582	0.94841
929	0.93392	0.93850	0.94057	0.94127	0.94349	0.94586	0.94845
930	0.93398	0.93856	0.94062	0.94132	0.94354	0.94591	0.94850
931	0.93404	0.93861	0.94068	0.94137	0.94359	0.94596	0.94854
932	0.93410	0.93866	0.94073	0.94142	0.94364	0.94600	0.94859
933	0.93415	0.93872	0.94078	0.94147	0.94369	0.94605	0.94863
934	0.93421	0.93877	0.94084	0.94153	0.94374	0.94610	0.94867
935	0.93427	0.93882	0.94089	0.94158	0.94379	0.94614	0.94872
936	0.93433	0.93888	0.94094	0.94163	0.94384	0.94619	0.94876
937	0.93439	0.93893	0.94099	0.94168	0.94389	0.94624	0.94881
938	0.93446	0.93899	0.94105	0.94173	0.94394	0.94628	0.94885
939	0.93452	0.93904	0.94110	0.94178	0.94398	0.94633	0.94889
940	0.93458	0.93909	0.94115	0.94183	0.94403	0.94638	0.94894
941	0.93464	0.93915	0.94120	0.94189	0.94408	0.94642	0.94898
942	0.93469	0.93920	0.94125	0.94194	0.94413	0.94647	0.94903
943	0.93475	0.93925	0.94130	0.94199	0.94418	0.94652	0.94907
944	0.93481	0.93930	0.94136	0.94204	0.94423	0.94656	0.94911
945	0.93487	0.93935	0.94140	0.94209	0.94428	0.94661	0.94916
946	0.93492	0.93941	0.94146	0.94214	0.94432	0.94665	0.94920
947	0.93498	0.93946	0.94151	0.94219	0.94437	0.94670	0.94924
948	0.93503	0.93951	0.94156	0.94224	0.94442	0.94674	0.94929
949	0.93508	0.93957	0.94161	0.94229	0.94447	0.94679	0.94933
950	0.93514	0.93962	0.94166	0.94234	0.94451	0.94684	0.94937
951	0.93520	0.93967	0.94171	0.94239	0.94456	0.94688	0.94941
952	0.93525	0.93973	0.94176	0.94244	0.94461	0.94693	0.94946
953	0.93530	0.93978	0.94181	0.94248	0.94466	0.94697	0.94950
954	0.93537	0.93983	0.94186	0.94253	0.94471	0.94702	0.94954
955	0.93543	0.93989	0.94191	0.94258	0.94476	0.94706	0.94959
956	0.93548	0.93994	0.94196	0.94263	0.94480	0.94711	0.94963
957	0.93554	0.93999	0.94201	0.94268	0.94485	0.94715	0.94967
958	0.93559	0.94004	0.94206	0.94273	0.94490	0.94720	0.94971
959	0.93565	0.94009	0.94211	0.94278	0.94494	0.94724	0.94976
960	0.93570	0.94014	0.94216	0.94283	0.94499	0.94729	0.94980

N	0.1%	0.5%	1%	1.25%	2.5%	5%	10%
961	0.93576	0.94020	0.94221	0.94288	0.94504	0.94733	0.94984
962	0.93581	0.94025	0.94226	0.94292	0.94508	0.94738	0.94988
963	0.93587	0.94030	0.94231	0.94297	0.94513	0.94742	0.94992
964	0.93593	0.94035	0.94236	0.94302	0.94518	0.94747	0.94997
965	0.93598	0.94040	0.94241	0.94307	0.94522	0.94751	0.95001
966	0.93603	0.94045	0.94246	0.94312	0.94527	0.94756	0.95005
967	0.93609	0.94050	0.94251	0.94317	0.94532	0.94760	0.95009
968	0.93614	0.94055	0.94256	0.94322	0.94537	0.94764	0.95013
969	0.93619	0.94060	0.94261	0.94327	0.94541	0.94769	0.95017
970	0.93625	0.94066	0.94265	0.94332	0.94546	0.94773	0.95021
971	0.93631	0.94071	0.94270	0.94336	0.94550	0.94778	0.95026
972	0.93636	0.94076	0.94275	0.94341	0.94555	0.94782	0.95030
973	0.93642	0.94081	0.94280	0.94346	0.94560	0.94786	0.95034
974	0.93647	0.94086	0.94285	0.94351	0.94564	0.94790	0.95038
975	0.93653	0.94091	0.94290	0.94356	0.94569	0.94795	0.95042
976	0.93659	0.94096	0.94295	0.94360	0.94573	0.94799	0.95046
977	0.93664	0.94101	0.94300	0.94365	0.94578	0.94804	0.95050
978	0.93669	0.94106	0.94304	0.94370	0.94583	0.94808	0.95054
979	0.93675	0.94111	0.94309	0.94375	0.94587	0.94812	0.95058
980	0.93680	0.94116	0.94314	0.94379	0.94591	0.94817	0.95062
981	0.93685	0.94121	0.94318	0.94384	0.94596	0.94821	0.95067
982	0.93691	0.94126	0.94323	0.94389	0.94600	0.94825	0.95071
983	0.93696	0.94131	0.94328	0.94394	0.94605	0.94829	0.95075
984	0.93702	0.94136	0.94333	0.94398	0.94610	0.94834	0.95079
985	0.93708	0.94141	0.94337	0.94403	0.94614	0.94838	0.95083
986	0.93713	0.94146	0.94342	0.94407	0.94619	0.94842	0.95087
987	0.93719	0.94150	0.94347	0.94412	0.94623	0.94847	0.95091
988	0.93725	0.94155	0.94352	0.94417	0.94628	0.94851	0.95095
989	0.93729	0.94160	0.94356	0.94421	0.94632	0.94855	0.95099
990	0.93734	0.94165	0.94361	0.94426	0.94637	0.94860	0.95103
991	0.93740	0.94170	0.94366	0.94431	0.94641	0.94864	0.95107
992	0.93746	0.94175	0.94370	0.94435	0.94646	0.94868	0.95111
993	0.93751	0.94180	0.94375	0.94440	0.94650	0.94872	0.95115
994	0.93756	0.94185	0.94380	0.94444	0.94654	0.94876	0.95119
995	0.93761	0.94189	0.94384	0.94449	0.94659	0.94881	0.95123
996	0.93766	0.94194	0.94389	0.94454	0.94663	0.94885	0.95127
997	0.93771	0.94199	0.94394	0.94458	0.94667	0.94889	0.95131
998	0.93776	0.94204	0.94398	0.94463	0.94672	0.94893	0.95135
999	0.93781	0.94209	0.94403	0.94468	0.94676	0.94897	0.95138
1000	0.93787	0.94214	0.94408	0.94472	0.94680	0.94902	0.95142

Table 2 – *GM5(4H1L or 1H4L)*. Critical values of upper significance levels for the Grubbs multiple-5 outlier test, applicable in situations where the four highest and one lowest or one highest and four lowest observations are simultaneously identified as outliers, denoted as *GM5(4H1L)* and *GM5(1H4L)*, respectively.

N	0.1%	0.5%	1%	1.25%	2.5%	5%	10%
7	0.00000	0.00000	0.00000	0.00000	0.00002	0.00006	0.00027
8	0.00004	0.00021	0.00042	0.00053	0.00110	0.00230	0.00492
9	0.00058	0.00178	0.00290	0.00340	0.00562	0.00941	0.01608
10	0.00246	0.00577	0.00841	0.00950	0.01404	0.02093	0.03186
11	0.00619	0.01235	0.01686	0.01864	0.02564	0.03566	0.05039
12	0.01174	0.02128	0.02769	0.03017	0.03949	0.05232	0.07035
13	0.01916	0.03200	0.04023	0.04336	0.05490	0.07015	0.09086
14	0.02802	0.04412	0.05404	0.05774	0.07120	0.08852	0.11144
15	0.03805	0.05715	0.06859	0.07282	0.08793	0.10703	0.13171
16	0.04886	0.07087	0.08362	0.08830	0.10486	0.12539	0.15151
17	0.06060	0.08489	0.09885	0.10395	0.12169	0.14350	0.17075
18	0.07265	0.09933	0.11421	0.11961	0.13843	0.16117	0.18932
19	0.08493	0.11372	0.12951	0.13521	0.15484	0.17833	0.20715
20	0.09759	0.12807	0.14467	0.15052	0.17085	0.19492	0.22435
21	0.11031	0.14224	0.15939	0.16547	0.18634	0.21104	0.24084
22	0.12296	0.15613	0.17390	0.18013	0.20144	0.22654	0.25664
23	0.13533	0.16980	0.18804	0.19446	0.21611	0.24148	0.27178
24	0.14778	0.18322	0.20181	0.20827	0.23039	0.25592	0.28629
25	0.16022	0.19635	0.21525	0.22188	0.24411	0.26978	0.30027
26	0.17215	0.20918	0.22836	0.23491	0.25736	0.28324	0.31367
27	0.18408	0.22152	0.24089	0.24758	0.27015	0.29608	0.32653
28	0.19601	0.23382	0.25310	0.25980	0.28257	0.30853	0.33888
29	0.20751	0.24552	0.26487	0.27170	0.29454	0.32049	0.35072
30	0.21830	0.25690	0.27649	0.28334	0.30612	0.33207	0.36216
31	0.22903	0.26805	0.28770	0.29454	0.31737	0.34323	0.37314

N	0.1%	0.5%	1%	1.25%	2.5%	5%	10%
32	0.23970	0.27903	0.29859	0.30537	0.32829	0.35398	0.38368
33	0.25004	0.28950	0.30924	0.31602	0.33874	0.36440	0.39390
34	0.26005	0.29974	0.31950	0.32636	0.34900	0.37444	0.40375
35	0.26994	0.30976	0.32950	0.33627	0.35888	0.38415	0.41322
36	0.27952	0.31940	0.33919	0.34598	0.36844	0.39352	0.42232
37	0.28884	0.32879	0.34849	0.35531	0.37770	0.40263	0.43114
38	0.29781	0.33800	0.35769	0.36440	0.38662	0.41138	0.43965
39	0.30686	0.34683	0.36650	0.37316	0.39532	0.41989	0.44791
40	0.31555	0.35551	0.37506	0.38174	0.40371	0.42811	0.45591
41	0.32419	0.36397	0.38343	0.39003	0.41190	0.43609	0.46366
42	0.33250	0.37203	0.39154	0.39814	0.41978	0.44386	0.47114
43	0.34053	0.38010	0.39937	0.40593	0.42751	0.45136	0.47842
44	0.34866	0.38794	0.40701	0.41353	0.43493	0.45866	0.48545
45	0.35624	0.39553	0.41453	0.42099	0.44215	0.46573	0.49228
46	0.36404	0.40284	0.42177	0.42816	0.44920	0.47261	0.49891
47	0.37153	0.41007	0.42876	0.43521	0.45608	0.47925	0.50532
48	0.37883	0.41708	0.43568	0.44202	0.46280	0.48573	0.51156
49	0.38549	0.42400	0.44244	0.44871	0.46928	0.49204	0.51763
50	0.39232	0.43070	0.44893	0.45516	0.47562	0.49820	0.52352
51	0.39919	0.43709	0.45537	0.46154	0.48184	0.50421	0.52927
52	0.40595	0.44351	0.46165	0.46773	0.48783	0.51002	0.53486
53	0.41221	0.44968	0.46764	0.47369	0.49367	0.51570	0.54028
54	0.41842	0.45570	0.47351	0.47954	0.49944	0.52125	0.54563
55	0.42463	0.46166	0.47931	0.48529	0.50499	0.52666	0.55079
56	0.43048	0.46735	0.48496	0.49091	0.51046	0.53186	0.55579
57	0.43635	0.47296	0.49047	0.49640	0.51579	0.53699	0.56071
58	0.44212	0.47859	0.49595	0.50174	0.52096	0.54199	0.56546
59	0.44773	0.48397	0.50112	0.50694	0.52602	0.54684	0.57013
60	0.45323	0.48925	0.50625	0.51200	0.53097	0.55163	0.57468
61	0.45859	0.49437	0.51124	0.51697	0.53582	0.55633	0.57912
62	0.46389	0.49935	0.51614	0.52186	0.54051	0.56085	0.58349
63	0.46895	0.50421	0.52096	0.52664	0.54513	0.56530	0.58770
64	0.47406	0.50902	0.52561	0.53125	0.54963	0.56963	0.59185
65	0.47895	0.51368	0.53023	0.53584	0.55407	0.57388	0.59591
66	0.48380	0.51827	0.53482	0.54033	0.55836	0.57800	0.59987
67	0.48854	0.52290	0.53922	0.54469	0.56258	0.58205	0.60375
68	0.49331	0.52737	0.54354	0.54896	0.56672	0.58603	0.60754
69	0.49793	0.53170	0.54782	0.55313	0.57073	0.58994	0.61124
70	0.50246	0.53601	0.55197	0.55728	0.57469	0.59375	0.61488
71	0.50685	0.54024	0.55599	0.56130	0.57860	0.59749	0.61844
72	0.51112	0.54433	0.55998	0.56526	0.58241	0.60114	0.62188
73	0.51524	0.54836	0.56388	0.56911	0.58617	0.60470	0.62528
74	0.51930	0.55219	0.56770	0.57287	0.58984	0.60820	0.62862
75	0.52341	0.55610	0.57142	0.57659	0.59343	0.61164	0.63189
76	0.52732	0.55989	0.57512	0.58026	0.59692	0.61502	0.63510
77	0.53125	0.56361	0.57872	0.58378	0.60037	0.61835	0.63824
78	0.53533	0.56726	0.58223	0.58730	0.60377	0.62159	0.64133
79	0.53905	0.57087	0.58571	0.59076	0.60709	0.62477	0.64435
80	0.54279	0.57440	0.58912	0.59411	0.61034	0.62788	0.64734
81	0.54643	0.57781	0.59243	0.59741	0.61352	0.63095	0.65025
82	0.55004	0.58114	0.59575	0.60069	0.61668	0.63395	0.65312
83	0.55343	0.58450	0.59894	0.60388	0.61978	0.63692	0.65592
84	0.55695	0.58773	0.60217	0.60704	0.62283	0.63981	0.65868
85	0.56039	0.59092	0.60532	0.61013	0.62579	0.64268	0.66140
86	0.56366	0.59409	0.60845	0.61321	0.62874	0.64547	0.66405
87	0.56702	0.59713	0.61147	0.61622	0.63160	0.64822	0.66666
88	0.57030	0.60031	0.61445	0.61910	0.63443	0.65094	0.66923
89	0.57331	0.60333	0.61733	0.62198	0.63723	0.65360	0.67174
90	0.57644	0.60628	0.62014	0.62479	0.63996	0.65622	0.67422
91	0.57963	0.60920	0.62299	0.62758	0.64265	0.65881	0.67666
92	0.58276	0.61207	0.62578	0.63033	0.64527	0.66135	0.67904
93	0.58572	0.61490	0.62851	0.63306	0.64789	0.66383	0.68140
94	0.58873	0.61766	0.63122	0.63570	0.65044	0.66627	0.68372
95	0.59162	0.62039	0.63389	0.63832	0.65294	0.66868	0.68600
96	0.59456	0.62309	0.63652	0.64093	0.65544	0.67105	0.68827
97	0.59735	0.62574	0.63903	0.64345	0.65786	0.67336	0.69048
98	0.60020	0.62829	0.64154	0.64595	0.66029	0.67566	0.69265
99	0.60276	0.63086	0.64401	0.64839	0.66264	0.67791	0.69478
100	0.60539	0.63337	0.64645	0.65079	0.66494	0.68014	0.69688
101	0.60811	0.63591	0.64884	0.65317	0.66724	0.68234	0.69896
102	0.61084	0.63835	0.65124	0.65556	0.66949	0.68447	0.70099
103	0.61349	0.64078	0.65359	0.65785	0.67170	0.68660	0.70300
104	0.61596	0.64311	0.65588	0.66015	0.67388	0.68868	0.70497
105	0.61845	0.64550	0.65814	0.66238	0.67606	0.69074	0.70692

N	0.1%	0.5%	1%	1.25%	2.5%	5%	10%
106	0.62107	0.64780	0.66038	0.66459	0.67816	0.69277	0.70884
107	0.62338	0.65010	0.66258	0.66679	0.68027	0.69479	0.71073
108	0.62592	0.65238	0.66478	0.66894	0.68234	0.69676	0.71259
109	0.62837	0.65457	0.66694	0.67106	0.68438	0.69870	0.71444
110	0.63060	0.65679	0.66904	0.67315	0.68639	0.70062	0.71624
111	0.63303	0.65892	0.67111	0.67518	0.68837	0.70249	0.71804
112	0.63524	0.66104	0.67317	0.67723	0.69031	0.70435	0.71982
113	0.63758	0.66312	0.67519	0.67919	0.69224	0.70622	0.72156
114	0.63977	0.66518	0.67715	0.68114	0.69413	0.70800	0.72327
115	0.64185	0.66719	0.67914	0.68307	0.69599	0.70980	0.72495
116	0.64414	0.66920	0.68107	0.68502	0.69785	0.71154	0.72661
117	0.64622	0.67120	0.68298	0.68690	0.69966	0.71327	0.72825
118	0.64827	0.67316	0.68486	0.68877	0.70144	0.71499	0.72987
119	0.65041	0.67510	0.68672	0.69060	0.70322	0.71668	0.73146
120	0.65243	0.67703	0.68857	0.69243	0.70496	0.71835	0.73305
121	0.65449	0.67894	0.69041	0.69424	0.70669	0.72000	0.73461
122	0.65651	0.68079	0.69219	0.69602	0.70841	0.72163	0.73614
123	0.65851	0.68261	0.69399	0.69776	0.71008	0.72324	0.73766
124	0.66057	0.68443	0.69574	0.69950	0.71174	0.72482	0.73915
125	0.66231	0.68627	0.69746	0.70122	0.71338	0.72637	0.74064
126	0.66427	0.68803	0.69914	0.70292	0.71499	0.72791	0.74210
127	0.66611	0.68977	0.70085	0.70459	0.71659	0.72944	0.74354
128	0.66801	0.69146	0.70253	0.70622	0.71817	0.73096	0.74496
129	0.66970	0.69313	0.70418	0.70784	0.71974	0.73245	0.74637
130	0.67158	0.69481	0.70579	0.70945	0.72126	0.73393	0.74776
131	0.67334	0.69654	0.70740	0.71102	0.72279	0.73537	0.74914
132	0.67510	0.69817	0.70898	0.71261	0.72428	0.73680	0.75050
133	0.67683	0.69981	0.71055	0.71414	0.72577	0.73822	0.75184
134	0.67867	0.70141	0.71209	0.71568	0.72725	0.73962	0.75316
135	0.68033	0.70300	0.71362	0.71721	0.72869	0.74101	0.75448
136	0.68204	0.70453	0.71515	0.71868	0.73011	0.74237	0.75577
137	0.68369	0.70614	0.71664	0.72016	0.73152	0.74372	0.75705
138	0.68532	0.70769	0.71811	0.72161	0.73292	0.74504	0.75831
139	0.68692	0.70919	0.71959	0.72306	0.73430	0.74636	0.75955
140	0.68855	0.71069	0.72103	0.72450	0.73567	0.74767	0.76078
141	0.69014	0.71217	0.72246	0.72591	0.73703	0.74896	0.76198
142	0.69165	0.71362	0.72387	0.72734	0.73839	0.75023	0.76319
143	0.69324	0.71509	0.72530	0.72872	0.73971	0.75150	0.76438
144	0.69481	0.71654	0.72669	0.73007	0.74102	0.75275	0.76556
145	0.69633	0.71798	0.72805	0.73144	0.74232	0.75399	0.76673
146	0.69786	0.71937	0.72941	0.73278	0.74360	0.75520	0.76788
147	0.69933	0.72076	0.73076	0.73408	0.74486	0.75641	0.76902
148	0.70081	0.72217	0.73208	0.73540	0.74610	0.75760	0.77014
149	0.70233	0.72347	0.73340	0.73667	0.74734	0.75878	0.77126
150	0.70373	0.72483	0.73466	0.73796	0.74857	0.75994	0.77235
151	0.70518	0.72611	0.73596	0.73923	0.74978	0.76110	0.77345
152	0.70657	0.72744	0.73722	0.74050	0.75098	0.76224	0.77453
153	0.70788	0.72879	0.73847	0.74172	0.75217	0.76336	0.77559
154	0.70935	0.73004	0.73972	0.74296	0.75335	0.76448	0.77665
155	0.71073	0.73137	0.74097	0.74418	0.75453	0.76558	0.77770
156	0.71201	0.73263	0.74219	0.74537	0.75568	0.76667	0.77873
157	0.71342	0.73386	0.74338	0.74656	0.75681	0.76776	0.77976
158	0.71479	0.73511	0.74456	0.74774	0.75794	0.76884	0.78077
159	0.71609	0.73630	0.74574	0.74890	0.75905	0.76990	0.78177
160	0.71739	0.73751	0.74690	0.75005	0.76014	0.77095	0.78277
161	0.71873	0.73869	0.74807	0.75119	0.76124	0.77198	0.78375
162	0.71992	0.73987	0.74920	0.75233	0.76233	0.77302	0.78471
163	0.72121	0.74108	0.75034	0.75343	0.76340	0.77404	0.78568
164	0.72254	0.74224	0.75147	0.75456	0.76445	0.77505	0.78663
165	0.72371	0.74340	0.75257	0.75564	0.76550	0.77605	0.78758
166	0.72499	0.74453	0.75366	0.75672	0.76653	0.77704	0.78852
167	0.72614	0.74565	0.75476	0.75779	0.76758	0.77803	0.78945
168	0.72737	0.74677	0.75580	0.75884	0.76860	0.77900	0.79037
169	0.72855	0.74788	0.75691	0.75988	0.76959	0.77996	0.79128
170	0.72978	0.74898	0.75797	0.76092	0.77059	0.78091	0.79218
171	0.73086	0.75006	0.75900	0.76196	0.77158	0.78185	0.79306
172	0.73210	0.75117	0.76005	0.76298	0.77256	0.78279	0.79395
173	0.73324	0.75224	0.76108	0.76399	0.77353	0.78372	0.79483
174	0.73439	0.75332	0.76209	0.76502	0.77448	0.78464	0.79569
175	0.73546	0.75437	0.76311	0.76600	0.77543	0.78555	0.79655
176	0.73657	0.75538	0.76409	0.76700	0.77637	0.78644	0.79740
177	0.73767	0.75644	0.76510	0.76797	0.77731	0.78733	0.79823
178	0.73876	0.75745	0.76609	0.76895	0.77825	0.78822	0.79907
179	0.73985	0.75845	0.76705	0.76990	0.77916	0.78910	0.79990

N	0.1%	0.5%	1%	1.25%	2.5%	5%	10%
180	0.74089	0.75946	0.76801	0.77085	0.78008	0.78997	0.80072
181	0.74201	0.76042	0.76897	0.77181	0.78099	0.79082	0.80153
182	0.74305	0.76142	0.76991	0.77275	0.78188	0.79167	0.80234
183	0.74410	0.76238	0.77086	0.77367	0.78276	0.79252	0.80313
184	0.74515	0.76335	0.77178	0.77461	0.78364	0.79335	0.80392
185	0.74617	0.76433	0.77270	0.77549	0.78451	0.79419	0.80469
186	0.74716	0.76528	0.77362	0.77640	0.78538	0.79500	0.80546
187	0.74809	0.76619	0.77451	0.77727	0.78622	0.79581	0.80624
188	0.74911	0.76716	0.77540	0.77816	0.78706	0.79661	0.80700
189	0.75019	0.76807	0.77630	0.77903	0.78790	0.79741	0.80775
190	0.75116	0.76895	0.77717	0.77990	0.78873	0.79821	0.80850
191	0.75209	0.76988	0.77805	0.78078	0.78957	0.79899	0.80925
192	0.75304	0.77076	0.77891	0.78163	0.79039	0.79977	0.80999
193	0.75393	0.77167	0.77976	0.78246	0.79120	0.80055	0.81072
194	0.75493	0.77253	0.78061	0.78331	0.79200	0.80132	0.81144
195	0.75583	0.77342	0.78145	0.78416	0.79279	0.80207	0.81216
196	0.75686	0.77429	0.78230	0.78497	0.79358	0.80283	0.81288
197	0.75775	0.77516	0.78314	0.78579	0.79438	0.80356	0.81358
198	0.75874	0.77602	0.78396	0.78662	0.79515	0.80430	0.81427
199	0.75960	0.77688	0.78478	0.78742	0.79591	0.80504	0.81497
200	0.76050	0.77770	0.78559	0.78821	0.79668	0.80577	0.81565
201	0.76147	0.77855	0.78639	0.78901	0.79745	0.80648	0.81634
202	0.76237	0.77936	0.78719	0.78981	0.79820	0.80720	0.81701
203	0.76331	0.78016	0.78798	0.79057	0.79894	0.80792	0.81769
204	0.76419	0.78099	0.78878	0.79134	0.79968	0.80861	0.81836
205	0.76505	0.78180	0.78954	0.79210	0.80041	0.80932	0.81902
206	0.76591	0.78260	0.79031	0.79286	0.80114	0.81001	0.81968
207	0.76673	0.78339	0.79108	0.79361	0.80186	0.81069	0.82032
208	0.76763	0.78417	0.79183	0.79437	0.80259	0.81138	0.82097
209	0.76844	0.78497	0.79259	0.79512	0.80329	0.81206	0.82161
210	0.76929	0.78573	0.79333	0.79586	0.80400	0.81273	0.82225
211	0.77017	0.78649	0.79407	0.79660	0.80470	0.81339	0.82288
212	0.77095	0.78726	0.79482	0.79732	0.80540	0.81406	0.82349
213	0.77177	0.78802	0.79554	0.79804	0.80608	0.81471	0.82411
214	0.77260	0.78878	0.79626	0.79875	0.80677	0.81536	0.82472
215	0.77346	0.78954	0.79697	0.79944	0.80745	0.81602	0.82534
216	0.77426	0.79029	0.79768	0.80016	0.80812	0.81666	0.82594
217	0.77504	0.79101	0.79839	0.80086	0.80878	0.81729	0.82654
218	0.77577	0.79175	0.79911	0.80156	0.80945	0.81792	0.82714
219	0.77652	0.79247	0.79980	0.80224	0.81012	0.81854	0.82773
220	0.77727	0.79319	0.80049	0.80293	0.81077	0.81916	0.82832
221	0.77801	0.79391	0.80119	0.80359	0.81141	0.81978	0.82890
222	0.77884	0.79461	0.80188	0.80428	0.81205	0.82038	0.82948
223	0.77957	0.79530	0.80256	0.80493	0.81270	0.82100	0.83005
224	0.78030	0.79599	0.80323	0.80559	0.81332	0.82159	0.83062
225	0.78106	0.79667	0.80389	0.80625	0.81394	0.82219	0.83119
226	0.78180	0.79737	0.80455	0.80690	0.81457	0.82279	0.83175
227	0.78248	0.79805	0.80520	0.80755	0.81519	0.82338	0.83231
228	0.78321	0.79874	0.80584	0.80819	0.81580	0.82396	0.83286
229	0.78390	0.79942	0.80648	0.80882	0.81641	0.82454	0.83341
230	0.78465	0.80007	0.80713	0.80946	0.81703	0.82512	0.83395
231	0.78538	0.80074	0.80776	0.81010	0.81762	0.82569	0.83449
232	0.78610	0.80140	0.80841	0.81073	0.81822	0.82626	0.83503
233	0.78681	0.80204	0.80902	0.81133	0.81881	0.82683	0.83556
234	0.78750	0.80267	0.80964	0.81195	0.81940	0.82739	0.83609
235	0.78818	0.80332	0.81027	0.81258	0.81999	0.82794	0.83662
236	0.78890	0.80396	0.81088	0.81319	0.82058	0.82849	0.83714
237	0.78957	0.80459	0.81148	0.81379	0.82115	0.82903	0.83765
238	0.79027	0.80522	0.81209	0.81438	0.82171	0.82958	0.83817
239	0.79094	0.80584	0.81271	0.81499	0.82228	0.83012	0.83868
240	0.79159	0.80647	0.81331	0.81558	0.82285	0.83065	0.83919
241	0.79229	0.80709	0.81389	0.81617	0.82341	0.83119	0.83969
242	0.79295	0.80769	0.81449	0.81675	0.82397	0.83172	0.84019
243	0.79362	0.80831	0.81506	0.81733	0.82453	0.83224	0.84069
244	0.79426	0.80891	0.81565	0.81790	0.82507	0.83277	0.84118
245	0.79488	0.80953	0.81622	0.81846	0.82562	0.83328	0.84167
246	0.79555	0.81015	0.81680	0.81902	0.82616	0.83379	0.84216
247	0.79623	0.81074	0.81738	0.81958	0.82670	0.83430	0.84264
248	0.79684	0.81131	0.81794	0.82014	0.82723	0.83482	0.84312
249	0.79746	0.81190	0.81850	0.82068	0.82776	0.83532	0.84359
250	0.79812	0.81247	0.81907	0.82122	0.82829	0.83582	0.84407
251	0.79872	0.81306	0.81962	0.82178	0.82882	0.83632	0.84454
252	0.79930	0.81362	0.82017	0.82233	0.82934	0.83682	0.84500
253	0.79994	0.81418	0.82072	0.82288	0.82986	0.83731	0.84547

N	0.1%	0.5%	1%	1.25%	2.5%	5%	10%
254	0.80055	0.81474	0.82124	0.82340	0.83036	0.83780	0.84593
255	0.80117	0.81531	0.82179	0.82393	0.83087	0.83828	0.84639
256	0.80181	0.81587	0.82233	0.82448	0.83137	0.83877	0.84685
257	0.80237	0.81640	0.82288	0.82500	0.83187	0.83924	0.84730
258	0.80295	0.81695	0.82340	0.82552	0.83237	0.83972	0.84775
259	0.80355	0.81749	0.82393	0.82605	0.83288	0.84020	0.84819
260	0.80413	0.81804	0.82446	0.82656	0.83337	0.84066	0.84864
261	0.80470	0.81857	0.82497	0.82707	0.83385	0.84113	0.84908
262	0.80528	0.81911	0.82546	0.82758	0.83434	0.84159	0.84951
263	0.80585	0.81964	0.82599	0.82807	0.83482	0.84205	0.84995
264	0.80639	0.82017	0.82649	0.82857	0.83530	0.84250	0.85038
265	0.80697	0.82069	0.82699	0.82906	0.83578	0.84296	0.85081
266	0.80757	0.82120	0.82750	0.82956	0.83626	0.84341	0.85124
267	0.80807	0.82174	0.82800	0.83006	0.83673	0.84386	0.85166
268	0.80861	0.82225	0.82849	0.83054	0.83720	0.84431	0.85208
269	0.80920	0.82275	0.82898	0.83103	0.83766	0.84475	0.85250
270	0.80977	0.82326	0.82947	0.83151	0.83812	0.84519	0.85291
271	0.81034	0.82376	0.82997	0.83199	0.83858	0.84563	0.85333
272	0.81085	0.82428	0.83044	0.83247	0.83904	0.84608	0.85374
273	0.81139	0.82476	0.83093	0.83294	0.83950	0.84651	0.85415
274	0.81197	0.82525	0.83141	0.83342	0.83995	0.84693	0.85455
275	0.81250	0.82576	0.83188	0.83389	0.84040	0.84736	0.85495
276	0.81302	0.82625	0.83235	0.83435	0.84085	0.84778	0.85536
277	0.81356	0.82674	0.83280	0.83481	0.84129	0.84821	0.85575
278	0.81409	0.82722	0.83327	0.83527	0.84174	0.84863	0.85615
279	0.81465	0.82770	0.83375	0.83573	0.84218	0.84904	0.85654
280	0.81516	0.82818	0.83420	0.83620	0.84261	0.84946	0.85693
281	0.81566	0.82866	0.83465	0.83664	0.84304	0.84986	0.85732
282	0.81617	0.82913	0.83511	0.83709	0.84347	0.85028	0.85771
283	0.81667	0.82961	0.83557	0.83753	0.84390	0.85068	0.85810
284	0.81721	0.83008	0.83601	0.83797	0.84432	0.85108	0.85848
285	0.81769	0.83054	0.83646	0.83841	0.84475	0.85148	0.85886
286	0.81821	0.83100	0.83691	0.83886	0.84517	0.85189	0.85924
287	0.81867	0.83146	0.83735	0.83929	0.84557	0.85228	0.85961
288	0.81916	0.83192	0.83778	0.83974	0.84600	0.85267	0.85998
289	0.81964	0.83237	0.83821	0.84017	0.84641	0.85307	0.86035
290	0.82017	0.83284	0.83865	0.84059	0.84682	0.85346	0.86072
291	0.82063	0.83328	0.83907	0.84101	0.84722	0.85385	0.86109
292	0.82113	0.83372	0.83950	0.84144	0.84762	0.85423	0.86145
293	0.82160	0.83416	0.83993	0.84186	0.84802	0.85462	0.86181
294	0.82210	0.83459	0.84036	0.84227	0.84842	0.85500	0.86217
295	0.82256	0.83504	0.84079	0.84269	0.84882	0.85537	0.86253
296	0.82300	0.83546	0.84122	0.84311	0.84922	0.85576	0.86289
297	0.82346	0.83590	0.84164	0.84353	0.84961	0.85613	0.86324
298	0.82393	0.83632	0.84205	0.84394	0.85000	0.85650	0.86359
299	0.82437	0.83674	0.84245	0.84434	0.85039	0.85687	0.86394
300	0.82483	0.83717	0.84286	0.84475	0.85077	0.85724	0.86429
301	0.82529	0.83758	0.84328	0.84514	0.85115	0.85760	0.86464
302	0.82575	0.83800	0.84368	0.84553	0.85153	0.85796	0.86499
303	0.82619	0.83843	0.84408	0.84592	0.85192	0.85833	0.86532
304	0.82663	0.83884	0.84447	0.84632	0.85229	0.85868	0.86566
305	0.82710	0.83925	0.84487	0.84671	0.85267	0.85904	0.86600
306	0.82754	0.83966	0.84527	0.84710	0.85305	0.85940	0.86634
307	0.82800	0.84008	0.84565	0.84750	0.85342	0.85975	0.86668
308	0.82843	0.84049	0.84604	0.84789	0.85378	0.86011	0.86701
309	0.82890	0.84089	0.84645	0.84827	0.85415	0.86045	0.86734
310	0.82929	0.84129	0.84682	0.84866	0.85452	0.86080	0.86767
311	0.82973	0.84170	0.84722	0.84904	0.85488	0.86115	0.86800
312	0.83017	0.84209	0.84759	0.84941	0.85525	0.86150	0.86832
313	0.83062	0.84248	0.84797	0.84978	0.85561	0.86184	0.86865
314	0.83103	0.84288	0.84835	0.85015	0.85597	0.86218	0.86897
315	0.83143	0.84327	0.84873	0.85052	0.85633	0.86252	0.86929
316	0.83184	0.84366	0.84909	0.85089	0.85669	0.86286	0.86961
317	0.83229	0.84405	0.84947	0.85126	0.85704	0.86319	0.86993
318	0.83266	0.84443	0.84984	0.85163	0.85739	0.86352	0.87024
319	0.83310	0.84482	0.85022	0.85199	0.85773	0.86386	0.87056
320	0.83351	0.84519	0.85058	0.85235	0.85808	0.86419	0.87087
321	0.83395	0.84557	0.85094	0.85272	0.85842	0.86452	0.87118
322	0.83433	0.84594	0.85131	0.85308	0.85877	0.86485	0.87149
323	0.83474	0.84632	0.85167	0.85345	0.85911	0.86518	0.87179
324	0.83518	0.84670	0.85202	0.85380	0.85945	0.86549	0.87209
325	0.83559	0.84705	0.85238	0.85415	0.85979	0.86582	0.87240
326	0.83600	0.84743	0.85274	0.85450	0.86013	0.86613	0.87270
327	0.83637	0.84780	0.85309	0.85484	0.86046	0.86645	0.87300

N	0.1%	0.5%	1%	1.25%	2.5%	5%	10%
328	0.83676	0.84817	0.85344	0.85518	0.86079	0.86677	0.87330
329	0.83714	0.84854	0.85378	0.85553	0.86112	0.86708	0.87360
330	0.83750	0.84890	0.85413	0.85588	0.86144	0.86739	0.87389
331	0.83792	0.84925	0.85448	0.85622	0.86177	0.86770	0.87419
332	0.83831	0.84960	0.85482	0.85655	0.86210	0.86801	0.87448
333	0.83870	0.84995	0.85515	0.85690	0.86242	0.86832	0.87477
334	0.83907	0.85031	0.85550	0.85723	0.86274	0.86862	0.87506
335	0.83945	0.85067	0.85584	0.85756	0.86307	0.86892	0.87535
336	0.83984	0.85102	0.85618	0.85789	0.86339	0.86923	0.87563
337	0.84023	0.85136	0.85652	0.85822	0.86370	0.86953	0.87592
338	0.84059	0.85172	0.85684	0.85855	0.86402	0.86983	0.87621
339	0.84094	0.85206	0.85717	0.85888	0.86433	0.87013	0.87649
340	0.84129	0.85241	0.85751	0.85921	0.86464	0.87043	0.87677
341	0.84169	0.85275	0.85784	0.85953	0.86495	0.87072	0.87705
342	0.84204	0.85310	0.85818	0.85986	0.86526	0.87102	0.87733
343	0.84241	0.85344	0.85850	0.86018	0.86556	0.87131	0.87760
344	0.84281	0.85378	0.85881	0.86051	0.86588	0.87160	0.87788
345	0.84316	0.85411	0.85914	0.86082	0.86618	0.87190	0.87815
346	0.84353	0.85445	0.85946	0.86114	0.86648	0.87219	0.87842
347	0.84391	0.85478	0.85977	0.86146	0.86679	0.87247	0.87870
348	0.84425	0.85510	0.86010	0.86177	0.86709	0.87276	0.87897
349	0.84460	0.85544	0.86041	0.86207	0.86738	0.87304	0.87923
350	0.84496	0.85576	0.86073	0.86239	0.86768	0.87333	0.87950
351	0.84533	0.85610	0.86105	0.86270	0.86798	0.87361	0.87976
352	0.84565	0.85641	0.86136	0.86300	0.86827	0.87389	0.88003
353	0.84601	0.85673	0.86167	0.86331	0.86856	0.87417	0.88029
354	0.84635	0.85705	0.86198	0.86361	0.86885	0.87444	0.88056
355	0.84667	0.85736	0.86228	0.86393	0.86914	0.87472	0.88082
356	0.84703	0.85768	0.86259	0.86423	0.86944	0.87500	0.88108
357	0.84736	0.85799	0.86288	0.86452	0.86972	0.87527	0.88134
358	0.84771	0.85831	0.86319	0.86481	0.87001	0.87554	0.88160
359	0.84806	0.85863	0.86349	0.86510	0.87030	0.87581	0.88185
360	0.84841	0.85894	0.86379	0.86540	0.87058	0.87608	0.88211
361	0.84872	0.85925	0.86409	0.86569	0.87086	0.87635	0.88236
362	0.84907	0.85957	0.86438	0.86599	0.87114	0.87662	0.88261
363	0.84941	0.85987	0.86468	0.86628	0.87142	0.87688	0.88286
364	0.84973	0.86018	0.86498	0.86657	0.87170	0.87715	0.88311
365	0.85008	0.86048	0.86526	0.86686	0.87198	0.87742	0.88336
366	0.85041	0.86078	0.86556	0.86715	0.87225	0.87768	0.88361
367	0.85073	0.86108	0.86584	0.86743	0.87252	0.87794	0.88386
368	0.85104	0.86138	0.86613	0.86772	0.87279	0.87820	0.88410
369	0.85137	0.86169	0.86643	0.86800	0.87306	0.87846	0.88435
370	0.85168	0.86198	0.86671	0.86828	0.87333	0.87871	0.88459
371	0.85198	0.86228	0.86700	0.86856	0.87360	0.87897	0.88483
372	0.85228	0.86258	0.86728	0.86884	0.87387	0.87923	0.88508
373	0.85259	0.86289	0.86756	0.86913	0.87413	0.87948	0.88532
374	0.85293	0.86317	0.86784	0.86940	0.87440	0.87973	0.88555
375	0.85325	0.86347	0.86813	0.86968	0.87467	0.87998	0.88579
376	0.85356	0.86376	0.86841	0.86995	0.87493	0.88024	0.88602
377	0.85386	0.86404	0.86868	0.87022	0.87519	0.88049	0.88626
378	0.85418	0.86432	0.86897	0.87049	0.87545	0.88073	0.88649
379	0.85449	0.86462	0.86924	0.87077	0.87571	0.88098	0.88673
380	0.85479	0.86489	0.86951	0.87103	0.87597	0.88123	0.88696
381	0.85508	0.86518	0.86978	0.87130	0.87622	0.88147	0.88719
382	0.85539	0.86545	0.87006	0.87157	0.87648	0.88172	0.88743
383	0.85572	0.86573	0.87032	0.87184	0.87673	0.88196	0.88766
384	0.85602	0.86600	0.87059	0.87210	0.87698	0.88220	0.88788
385	0.85633	0.86628	0.87085	0.87236	0.87723	0.88244	0.88811
386	0.85664	0.86657	0.87112	0.87262	0.87748	0.88268	0.88834
387	0.85693	0.86685	0.87138	0.87289	0.87774	0.88292	0.88856
388	0.85719	0.86712	0.87164	0.87315	0.87798	0.88315	0.88879
389	0.85749	0.86738	0.87190	0.87341	0.87823	0.88338	0.88901
390	0.85778	0.86766	0.87216	0.87367	0.87847	0.88362	0.88923
391	0.85810	0.86793	0.87242	0.87392	0.87872	0.88386	0.88945
392	0.85838	0.86820	0.87268	0.87418	0.87897	0.88409	0.88967
393	0.85870	0.86847	0.87294	0.87443	0.87921	0.88432	0.88989
394	0.85897	0.86874	0.87320	0.87468	0.87945	0.88455	0.89011
395	0.85925	0.86900	0.87345	0.87493	0.87969	0.88478	0.89033
396	0.85955	0.86925	0.87371	0.87520	0.87994	0.88501	0.89054
397	0.85982	0.86952	0.87396	0.87544	0.88017	0.88524	0.89076
398	0.86009	0.86978	0.87421	0.87570	0.88042	0.88547	0.89097
399	0.86037	0.87004	0.87447	0.87595	0.88066	0.88569	0.89119
400	0.86064	0.87029	0.87472	0.87619	0.88089	0.88592	0.89140
401	0.86095	0.87055	0.87497	0.87643	0.88113	0.88614	0.89161

N	0.1%	0.5%	1%	1.25%	2.5%	5%	10%
402	0.86122	0.87081	0.87521	0.87668	0.88136	0.88637	0.89182
403	0.86152	0.87107	0.87546	0.87692	0.88159	0.88659	0.89203
404	0.86178	0.87133	0.87571	0.87716	0.88183	0.88681	0.89224
405	0.86206	0.87159	0.87595	0.87741	0.88206	0.88704	0.89245
406	0.86234	0.87184	0.87619	0.87765	0.88228	0.88726	0.89266
407	0.86258	0.87209	0.87643	0.87789	0.88251	0.88747	0.89287
408	0.86286	0.87235	0.87667	0.87812	0.88275	0.88769	0.89307
409	0.86312	0.87260	0.87691	0.87836	0.88297	0.88791	0.89327
410	0.86338	0.87284	0.87714	0.87859	0.88320	0.88812	0.89348
411	0.86367	0.87310	0.87738	0.87882	0.88342	0.88834	0.89368
412	0.86394	0.87333	0.87763	0.87906	0.88364	0.88855	0.89389
413	0.86419	0.87358	0.87786	0.87929	0.88387	0.88876	0.89408
414	0.86446	0.87383	0.87810	0.87951	0.88409	0.88897	0.89428
415	0.86472	0.87407	0.87834	0.87975	0.88431	0.88918	0.89449
416	0.86499	0.87431	0.87856	0.87998	0.88453	0.88939	0.89468
417	0.86526	0.87455	0.87880	0.88020	0.88475	0.88960	0.89488
418	0.86549	0.87479	0.87903	0.88044	0.88497	0.88981	0.89508
419	0.86575	0.87502	0.87927	0.88067	0.88519	0.89002	0.89527
420	0.86602	0.87527	0.87948	0.88089	0.88540	0.89022	0.89547
421	0.86628	0.87550	0.87972	0.88112	0.88562	0.89043	0.89567
422	0.86652	0.87574	0.87994	0.88134	0.88584	0.89063	0.89586
423	0.86676	0.87597	0.88017	0.88157	0.88605	0.89084	0.89605
424	0.86702	0.87621	0.88039	0.88180	0.88626	0.89104	0.89625
425	0.86728	0.87646	0.88062	0.88201	0.88647	0.89124	0.89644
426	0.86752	0.87670	0.88085	0.88223	0.88669	0.89145	0.89663
427	0.86778	0.87693	0.88107	0.88245	0.88690	0.89165	0.89682
428	0.86805	0.87716	0.88129	0.88267	0.88711	0.89185	0.89701
429	0.86830	0.87738	0.88151	0.88289	0.88731	0.89205	0.89720
430	0.86854	0.87761	0.88173	0.88311	0.88752	0.89225	0.89739
431	0.86879	0.87784	0.88195	0.88332	0.88773	0.89244	0.89758
432	0.86906	0.87807	0.88217	0.88353	0.88793	0.89264	0.89776
433	0.86928	0.87830	0.88239	0.88375	0.88814	0.89283	0.89795
434	0.86953	0.87851	0.88261	0.88396	0.88835	0.89303	0.89813
435	0.86977	0.87874	0.88283	0.88417	0.88855	0.89323	0.89832
436	0.86999	0.87896	0.88304	0.88439	0.88876	0.89342	0.89850
437	0.87024	0.87918	0.88326	0.88461	0.88896	0.89361	0.89868
438	0.87051	0.87940	0.88346	0.88481	0.88917	0.89380	0.89886
439	0.87072	0.87962	0.88368	0.88503	0.88937	0.89399	0.89904
440	0.87097	0.87984	0.88389	0.88523	0.88956	0.89418	0.89922
441	0.87121	0.88005	0.88410	0.88545	0.88976	0.89437	0.89940
442	0.87145	0.88027	0.88431	0.88566	0.88996	0.89457	0.89958
443	0.87169	0.88049	0.88452	0.88586	0.89016	0.89475	0.89976
444	0.87191	0.88069	0.88472	0.88607	0.89036	0.89494	0.89994
445	0.87215	0.88091	0.88494	0.88627	0.89056	0.89513	0.90012
446	0.87239	0.88113	0.88514	0.88647	0.89075	0.89531	0.90029
447	0.87261	0.88135	0.88535	0.88668	0.89095	0.89550	0.90047
448	0.87282	0.88156	0.88556	0.88688	0.89114	0.89568	0.90064
449	0.87306	0.88177	0.88576	0.88708	0.89133	0.89586	0.90081
450	0.87329	0.88198	0.88596	0.88728	0.89152	0.89605	0.90099
451	0.87350	0.88220	0.88617	0.88749	0.89172	0.89623	0.90116
452	0.87373	0.88240	0.88636	0.88769	0.89191	0.89641	0.90133
453	0.87395	0.88260	0.88656	0.88788	0.89210	0.89659	0.90151
454	0.87418	0.88281	0.88676	0.88808	0.89228	0.89677	0.90167
455	0.87440	0.88303	0.88696	0.88827	0.89248	0.89695	0.90184
456	0.87462	0.88323	0.88716	0.88847	0.89266	0.89713	0.90201
457	0.87484	0.88344	0.88736	0.88867	0.89285	0.89731	0.90218
458	0.87507	0.88364	0.88756	0.88886	0.89303	0.89749	0.90235
459	0.87527	0.88384	0.88775	0.88905	0.89322	0.89766	0.90252
460	0.87550	0.88405	0.88795	0.88924	0.89340	0.89784	0.90268
461	0.87574	0.88425	0.88815	0.88944	0.89359	0.89802	0.90285
462	0.87596	0.88446	0.88834	0.88963	0.89377	0.89819	0.90302
463	0.87617	0.88465	0.88854	0.88982	0.89395	0.89837	0.90318
464	0.87640	0.88485	0.88873	0.89001	0.89414	0.89854	0.90335
465	0.87661	0.88506	0.88892	0.89020	0.89432	0.89871	0.90351
466	0.87683	0.88526	0.88912	0.89039	0.89450	0.89889	0.90368
467	0.87705	0.88545	0.88930	0.89057	0.89468	0.89906	0.90384
468	0.87727	0.88565	0.88949	0.89076	0.89486	0.89923	0.90400
469	0.87746	0.88584	0.88968	0.89095	0.89504	0.89940	0.90416
470	0.87767	0.88605	0.88987	0.89114	0.89522	0.89957	0.90433
471	0.87787	0.88623	0.89006	0.89132	0.89540	0.89974	0.90449
472	0.87807	0.88643	0.89025	0.89151	0.89557	0.89991	0.90465
473	0.87830	0.88661	0.89044	0.89169	0.89575	0.90008	0.90481
474	0.87849	0.88681	0.89062	0.89187	0.89593	0.90025	0.90496
475	0.87870	0.88700	0.89080	0.89206	0.89610	0.90041	0.90512

N	0.1%	0.5%	1%	1.25%	2.5%	5%	10%
476	0.87892	0.88719	0.89099	0.89224	0.89628	0.90058	0.90528
477	0.87914	0.88737	0.89117	0.89242	0.89645	0.90074	0.90543
478	0.87934	0.88756	0.89135	0.89260	0.89662	0.90091	0.90559
479	0.87956	0.88775	0.89154	0.89279	0.89680	0.90108	0.90575
480	0.87976	0.88794	0.89172	0.89297	0.89697	0.90124	0.90590
481	0.87997	0.88814	0.89190	0.89315	0.89714	0.90140	0.90606
482	0.88017	0.88832	0.89208	0.89333	0.89731	0.90157	0.90621
483	0.88037	0.88851	0.89226	0.89351	0.89748	0.90173	0.90637
484	0.88057	0.88869	0.89243	0.89368	0.89765	0.90189	0.90652
485	0.88078	0.88888	0.89261	0.89386	0.89782	0.90205	0.90667
486	0.88099	0.88906	0.89279	0.89404	0.89799	0.90221	0.90682
487	0.88118	0.88925	0.89297	0.89421	0.89816	0.90237	0.90697
488	0.88138	0.88943	0.89314	0.89438	0.89832	0.90253	0.90712
489	0.88157	0.88961	0.89332	0.89455	0.89848	0.90269	0.90727
490	0.88178	0.88979	0.89350	0.89472	0.89865	0.90285	0.90742
491	0.88197	0.88997	0.89367	0.89489	0.89881	0.90301	0.90757
492	0.88216	0.89015	0.89384	0.89506	0.89898	0.90317	0.90772
493	0.88235	0.89033	0.89402	0.89524	0.89915	0.90332	0.90787
494	0.88253	0.89051	0.89420	0.89541	0.89931	0.90348	0.90801
495	0.88273	0.89069	0.89438	0.89558	0.89948	0.90363	0.90816
496	0.88291	0.89087	0.89454	0.89575	0.89964	0.90379	0.90831
497	0.88309	0.89105	0.89471	0.89593	0.89980	0.90394	0.90845
498	0.88329	0.89123	0.89488	0.89609	0.89996	0.90410	0.90860
499	0.88349	0.89140	0.89506	0.89626	0.90013	0.90425	0.90875
500	0.88368	0.89158	0.89523	0.89643	0.90029	0.90441	0.90889
501	0.88386	0.89176	0.89540	0.89660	0.90044	0.90456	0.90903
502	0.88405	0.89193	0.89556	0.89677	0.90060	0.90471	0.90918
503	0.88424	0.89211	0.89573	0.89693	0.90076	0.90486	0.90932
504	0.88443	0.89229	0.89590	0.89710	0.90092	0.90501	0.90947
505	0.88462	0.89246	0.89606	0.89726	0.90108	0.90516	0.90961
506	0.88481	0.89264	0.89623	0.89743	0.90123	0.90531	0.90975
507	0.88500	0.89281	0.89640	0.89759	0.90139	0.90546	0.90989
508	0.88518	0.89298	0.89656	0.89775	0.90155	0.90561	0.91003
509	0.88537	0.89315	0.89673	0.89791	0.90170	0.90576	0.91017
510	0.88556	0.89332	0.89689	0.89807	0.90185	0.90591	0.91031
511	0.88574	0.89349	0.89705	0.89823	0.90201	0.90605	0.91045
512	0.88593	0.89366	0.89722	0.89839	0.90216	0.90620	0.91059
513	0.88612	0.89384	0.89738	0.89855	0.90232	0.90634	0.91072
514	0.88630	0.89400	0.89754	0.89871	0.90247	0.90649	0.91086
515	0.88647	0.89416	0.89770	0.89887	0.90262	0.90663	0.91100
516	0.88665	0.89433	0.89786	0.89903	0.90277	0.90678	0.91114
517	0.88683	0.89449	0.89802	0.89919	0.90292	0.90692	0.91127
518	0.88700	0.89466	0.89818	0.89935	0.90307	0.90707	0.91141
519	0.88718	0.89483	0.89834	0.89950	0.90322	0.90721	0.91155
520	0.88736	0.89499	0.89849	0.89965	0.90337	0.90735	0.91168
521	0.88754	0.89516	0.89865	0.89981	0.90352	0.90750	0.91181
522	0.88771	0.89532	0.89881	0.89996	0.90367	0.90764	0.91195
523	0.88790	0.89549	0.89897	0.90012	0.90382	0.90778	0.91209
524	0.88807	0.89565	0.89913	0.90027	0.90397	0.90792	0.91222
525	0.88824	0.89582	0.89929	0.90043	0.90412	0.90806	0.91235
526	0.88841	0.89598	0.89944	0.90058	0.90426	0.90820	0.91249
527	0.88858	0.89614	0.89960	0.90073	0.90441	0.90834	0.91262
528	0.88877	0.89629	0.89975	0.90088	0.90456	0.90848	0.91275
529	0.88893	0.89645	0.89990	0.90103	0.90470	0.90862	0.91288
530	0.88911	0.89661	0.90005	0.90118	0.90485	0.90876	0.91301
531	0.88929	0.89677	0.90020	0.90133	0.90498	0.90889	0.91314
532	0.88946	0.89693	0.90035	0.90148	0.90513	0.90903	0.91327
533	0.88965	0.89708	0.90050	0.90163	0.90527	0.90917	0.91340
534	0.88982	0.89724	0.90065	0.90178	0.90542	0.90930	0.91353
535	0.88997	0.89739	0.90080	0.90193	0.90556	0.90944	0.91366
536	0.89016	0.89755	0.90095	0.90207	0.90570	0.90957	0.91379
537	0.89033	0.89770	0.90110	0.90222	0.90585	0.90971	0.91391
538	0.89049	0.89786	0.90125	0.90237	0.90599	0.90984	0.91404
539	0.89066	0.89802	0.90140	0.90252	0.90613	0.90998	0.91417
540	0.89083	0.89817	0.90155	0.90267	0.90627	0.91011	0.91429
541	0.89098	0.89833	0.90170	0.90281	0.90641	0.91024	0.91442
542	0.89113	0.89848	0.90185	0.90296	0.90655	0.91037	0.91454
543	0.89129	0.89864	0.90199	0.90310	0.90669	0.91051	0.91467
544	0.89146	0.89878	0.90214	0.90325	0.90683	0.91064	0.91479
545	0.89162	0.89894	0.90228	0.90339	0.90697	0.91077	0.91492
546	0.89179	0.89908	0.90242	0.90353	0.90710	0.91090	0.91504
547	0.89194	0.89923	0.90257	0.90368	0.90724	0.91103	0.91517
548	0.89209	0.89938	0.90272	0.90382	0.90737	0.91116	0.91529
549	0.89226	0.89953	0.90286	0.90396	0.90751	0.91129	0.91541

N	0.1%	0.5%	1%	1.25%	2.5%	5%	10%
550	0.89241	0.89969	0.90300	0.90410	0.90765	0.91142	0.91553
551	0.89257	0.89983	0.90315	0.90425	0.90778	0.91155	0.91566
552	0.89275	0.89997	0.90328	0.90439	0.90792	0.91168	0.91578
553	0.89290	0.90013	0.90343	0.90453	0.90805	0.91181	0.91590
554	0.89307	0.90027	0.90357	0.90467	0.90818	0.91194	0.91602
555	0.89322	0.90043	0.90371	0.90481	0.90832	0.91207	0.91614
556	0.89337	0.90058	0.90385	0.90495	0.90845	0.91219	0.91626
557	0.89352	0.90072	0.90399	0.90508	0.90858	0.91232	0.91638
558	0.89369	0.90086	0.90413	0.90522	0.90871	0.91244	0.91650
559	0.89385	0.90101	0.90427	0.90536	0.90884	0.91257	0.91662
560	0.89401	0.90115	0.90441	0.90550	0.90898	0.91270	0.91674
561	0.89417	0.90129	0.90455	0.90563	0.90911	0.91282	0.91686
562	0.89433	0.90143	0.90469	0.90577	0.90924	0.91294	0.91698
563	0.89449	0.90158	0.90483	0.90590	0.90937	0.91307	0.91710
564	0.89463	0.90172	0.90496	0.90604	0.90950	0.91319	0.91721
565	0.89476	0.90186	0.90511	0.90617	0.90963	0.91331	0.91733
566	0.89491	0.90201	0.90524	0.90631	0.90976	0.91344	0.91745
567	0.89505	0.90215	0.90538	0.90644	0.90988	0.91356	0.91756
568	0.89520	0.90229	0.90551	0.90657	0.91001	0.91368	0.91768
569	0.89535	0.90243	0.90564	0.90671	0.91014	0.91381	0.91779
570	0.89551	0.90257	0.90578	0.90684	0.91026	0.91393	0.91791
571	0.89566	0.90270	0.90591	0.90697	0.91039	0.91405	0.91802
572	0.89581	0.90285	0.90604	0.90711	0.91052	0.91417	0.91814
573	0.89595	0.90297	0.90617	0.90724	0.91065	0.91429	0.91825
574	0.89609	0.90312	0.90631	0.90736	0.91077	0.91441	0.91836
575	0.89624	0.90326	0.90644	0.90750	0.91089	0.91453	0.91848
576	0.89640	0.90339	0.90657	0.90762	0.91102	0.91465	0.91859
577	0.89654	0.90352	0.90670	0.90775	0.91115	0.91477	0.91870
578	0.89670	0.90366	0.90683	0.90789	0.91127	0.91489	0.91881
579	0.89684	0.90380	0.90696	0.90802	0.91140	0.91501	0.91893
580	0.89699	0.90393	0.90709	0.90815	0.91152	0.91512	0.91904
581	0.89712	0.90407	0.90722	0.90827	0.91164	0.91524	0.91915
582	0.89728	0.90420	0.90735	0.90840	0.91177	0.91536	0.91926
583	0.89741	0.90434	0.90748	0.90853	0.91189	0.91547	0.91937
584	0.89757	0.90447	0.90761	0.90866	0.91201	0.91559	0.91948
585	0.89770	0.90461	0.90774	0.90878	0.91213	0.91571	0.91959
586	0.89786	0.90474	0.90787	0.90891	0.91225	0.91582	0.91970
587	0.89799	0.90488	0.90800	0.90904	0.91237	0.91594	0.91981
588	0.89815	0.90501	0.90813	0.90916	0.91250	0.91605	0.91992
589	0.89829	0.90514	0.90826	0.90929	0.91262	0.91617	0.92003
590	0.89842	0.90527	0.90839	0.90941	0.91274	0.91628	0.92014
591	0.89857	0.90540	0.90851	0.90954	0.91286	0.91639	0.92025
592	0.89871	0.90554	0.90864	0.90966	0.91298	0.91651	0.92035
593	0.89884	0.90567	0.90876	0.90979	0.91310	0.91662	0.92046
594	0.89899	0.90580	0.90889	0.90991	0.91322	0.91673	0.92057
595	0.89912	0.90593	0.90902	0.91003	0.91333	0.91684	0.92068
596	0.89927	0.90606	0.90915	0.91016	0.91345	0.91696	0.92078
597	0.89941	0.90619	0.90927	0.91028	0.91357	0.91707	0.92089
598	0.89953	0.90631	0.90939	0.91040	0.91369	0.91718	0.92099
599	0.89966	0.90644	0.90951	0.91052	0.91380	0.91729	0.92110
600	0.89981	0.90657	0.90964	0.91065	0.91392	0.91740	0.92120
601	0.89994	0.90670	0.90976	0.91077	0.91403	0.91751	0.92131
602	0.90008	0.90682	0.90988	0.91089	0.91415	0.91763	0.92141
603	0.90021	0.90695	0.91000	0.91101	0.91427	0.91774	0.92152
604	0.90035	0.90708	0.91012	0.91113	0.91438	0.91785	0.92162
605	0.90046	0.90720	0.91024	0.91124	0.91449	0.91796	0.92173
606	0.90060	0.90732	0.91036	0.91136	0.91461	0.91806	0.92183
607	0.90074	0.90745	0.91048	0.91148	0.91472	0.91817	0.92193
608	0.90088	0.90757	0.91060	0.91160	0.91484	0.91828	0.92203
609	0.90103	0.90769	0.91072	0.91172	0.91495	0.91839	0.92214
610	0.90117	0.90781	0.91084	0.91184	0.91506	0.91850	0.92224
611	0.90129	0.90793	0.91096	0.91195	0.91517	0.91861	0.92234
612	0.90142	0.90806	0.91108	0.91207	0.91529	0.91872	0.92244
613	0.90156	0.90818	0.91120	0.91219	0.91540	0.91882	0.92254
614	0.90168	0.90830	0.91132	0.91230	0.91551	0.91893	0.92265
615	0.90181	0.90842	0.91144	0.91242	0.91562	0.91903	0.92275
616	0.90193	0.90854	0.91155	0.91254	0.91573	0.91914	0.92285
617	0.90208	0.90866	0.91167	0.91265	0.91584	0.91924	0.92295
618	0.90223	0.90878	0.91179	0.91277	0.91596	0.91935	0.92305
619	0.90235	0.90890	0.91191	0.91289	0.91607	0.91946	0.92315
620	0.90248	0.90902	0.91202	0.91300	0.91618	0.91956	0.92325
621	0.90261	0.90915	0.91214	0.91312	0.91629	0.91967	0.92335
622	0.90274	0.90927	0.91225	0.91323	0.91640	0.91977	0.92344
623	0.90288	0.90939	0.91236	0.91334	0.91650	0.91987	0.92354

N	0.1%	0.5%	1%	1.25%	2.5%	5%	10%
624	0.90301	0.90951	0.91248	0.91345	0.91661	0.91998	0.92364
625	0.90313	0.90962	0.91259	0.91357	0.91672	0.92008	0.92374
626	0.90325	0.90975	0.91270	0.91368	0.91683	0.92018	0.92384
627	0.90337	0.90986	0.91282	0.91379	0.91694	0.92028	0.92394
628	0.90349	0.90999	0.91293	0.91391	0.91704	0.92039	0.92403
629	0.90363	0.91010	0.91305	0.91402	0.91715	0.92049	0.92413
630	0.90375	0.91021	0.91316	0.91413	0.91726	0.92059	0.92423
631	0.90388	0.91033	0.91328	0.91423	0.91736	0.92069	0.92432
632	0.90401	0.91045	0.91339	0.91434	0.91747	0.92079	0.92442
633	0.90412	0.91056	0.91350	0.91446	0.91757	0.92090	0.92452
634	0.90424	0.91067	0.91361	0.91457	0.91768	0.92100	0.92461
635	0.90438	0.91079	0.91372	0.91468	0.91778	0.92110	0.92470
636	0.90450	0.91091	0.91383	0.91478	0.91789	0.92120	0.92480
637	0.90462	0.91102	0.91394	0.91489	0.91799	0.92130	0.92490
638	0.90475	0.91114	0.91405	0.91500	0.91810	0.92140	0.92499
639	0.90488	0.91125	0.91416	0.91511	0.91820	0.92150	0.92509
640	0.90500	0.91136	0.91426	0.91522	0.91830	0.92159	0.92518
641	0.90513	0.91147	0.91437	0.91533	0.91841	0.92169	0.92527
642	0.90526	0.91159	0.91448	0.91543	0.91851	0.92179	0.92537
643	0.90538	0.91171	0.91459	0.91554	0.91861	0.92189	0.92546
644	0.90550	0.91182	0.91470	0.91565	0.91871	0.92199	0.92555
645	0.90562	0.91193	0.91481	0.91575	0.91881	0.92208	0.92565
646	0.90573	0.91204	0.91491	0.91586	0.91892	0.92218	0.92574
647	0.90586	0.91215	0.91502	0.91597	0.91902	0.92228	0.92583
648	0.90596	0.91225	0.91513	0.91608	0.91912	0.92238	0.92592
649	0.90609	0.91237	0.91524	0.91618	0.91922	0.92247	0.92601
650	0.90621	0.91248	0.91534	0.91629	0.91932	0.92257	0.92610
651	0.90633	0.91259	0.91545	0.91639	0.91942	0.92267	0.92619
652	0.90644	0.91270	0.91555	0.91649	0.91953	0.92276	0.92629
653	0.90656	0.91280	0.91566	0.91660	0.91963	0.92286	0.92638
654	0.90669	0.91291	0.91576	0.91670	0.91973	0.92295	0.92647
655	0.90682	0.91302	0.91586	0.91681	0.91983	0.92305	0.92656
656	0.90693	0.91313	0.91597	0.91691	0.91992	0.92314	0.92665
657	0.90705	0.91324	0.91607	0.91702	0.92002	0.92324	0.92674
658	0.90716	0.91335	0.91618	0.91712	0.92012	0.92333	0.92683
659	0.90727	0.91346	0.91628	0.91722	0.92022	0.92343	0.92692
660	0.90738	0.91357	0.91639	0.91732	0.92032	0.92352	0.92701
661	0.90748	0.91367	0.91649	0.91742	0.92041	0.92362	0.92709
662	0.90760	0.91378	0.91660	0.91752	0.92052	0.92371	0.92718
663	0.90772	0.91388	0.91670	0.91763	0.92061	0.92380	0.92727
664	0.90783	0.91399	0.91680	0.91773	0.92071	0.92389	0.92736
665	0.90793	0.91410	0.91691	0.91783	0.92081	0.92399	0.92745
666	0.90805	0.91420	0.91701	0.91793	0.92090	0.92408	0.92753
667	0.90816	0.91430	0.91711	0.91803	0.92100	0.92417	0.92762
668	0.90828	0.91441	0.91721	0.91813	0.92110	0.92426	0.92771
669	0.90839	0.91451	0.91731	0.91823	0.92119	0.92435	0.92779
670	0.90852	0.91462	0.91742	0.91833	0.92129	0.92444	0.92788
671	0.90863	0.91473	0.91752	0.91843	0.92138	0.92454	0.92797
672	0.90875	0.91483	0.91762	0.91852	0.92148	0.92463	0.92806
673	0.90885	0.91494	0.91772	0.91862	0.92157	0.92472	0.92814
674	0.90896	0.91504	0.91781	0.91872	0.92167	0.92481	0.92823
675	0.90906	0.91514	0.91791	0.91882	0.92176	0.92489	0.92831
676	0.90918	0.91525	0.91801	0.91892	0.92186	0.92499	0.92840
677	0.90929	0.91535	0.91811	0.91902	0.92195	0.92508	0.92848
678	0.90940	0.91546	0.91821	0.91912	0.92204	0.92517	0.92857
679	0.90951	0.91556	0.91832	0.91922	0.92214	0.92525	0.92865
680	0.90961	0.91566	0.91841	0.91931	0.92223	0.92534	0.92874
681	0.90972	0.91576	0.91851	0.91941	0.92232	0.92543	0.92882
682	0.90982	0.91586	0.91861	0.91951	0.92242	0.92552	0.92890
683	0.90994	0.91596	0.91871	0.91961	0.92251	0.92561	0.92899
684	0.91005	0.91606	0.91880	0.91971	0.92260	0.92570	0.92907
685	0.91016	0.91616	0.91890	0.91980	0.92270	0.92578	0.92916
686	0.91028	0.91626	0.91900	0.91989	0.92279	0.92587	0.92924
687	0.91038	0.91637	0.91909	0.91999	0.92288	0.92596	0.92932
688	0.91048	0.91647	0.91919	0.92009	0.92297	0.92604	0.92940
689	0.91059	0.91656	0.91929	0.92018	0.92306	0.92613	0.92948
690	0.91069	0.91666	0.91938	0.92027	0.92315	0.92622	0.92957
691	0.91081	0.91676	0.91948	0.92037	0.92324	0.92630	0.92965
692	0.91092	0.91686	0.91957	0.92046	0.92333	0.92639	0.92973
693	0.91104	0.91696	0.91967	0.92056	0.92342	0.92648	0.92981
694	0.91114	0.91706	0.91976	0.92065	0.92351	0.92656	0.92989
695	0.91124	0.91716	0.91986	0.92074	0.92360	0.92665	0.92997
696	0.91135	0.91726	0.91995	0.92084	0.92369	0.92673	0.93005
697	0.91145	0.91736	0.92005	0.92093	0.92378	0.92682	0.93014

N	0.1%	0.5%	1%	1.25%	2.5%	5%	10%
698	0.91156	0.91745	0.92014	0.92102	0.92387	0.92690	0.93022
699	0.91168	0.91755	0.92023	0.92112	0.92396	0.92699	0.93030
700	0.91178	0.91764	0.92033	0.92121	0.92405	0.92708	0.93038
701	0.91188	0.91774	0.92042	0.92130	0.92413	0.92716	0.93046
702	0.91199	0.91784	0.92051	0.92139	0.92422	0.92725	0.93054
703	0.91210	0.91794	0.92060	0.92148	0.92431	0.92733	0.93062
704	0.91221	0.91804	0.92069	0.92157	0.92440	0.92741	0.93070
705	0.91231	0.91813	0.92078	0.92167	0.92448	0.92749	0.93077
706	0.91241	0.91822	0.92088	0.92175	0.92457	0.92758	0.93085
707	0.91251	0.91831	0.92097	0.92185	0.92466	0.92766	0.93093
708	0.91262	0.91841	0.92106	0.92194	0.92474	0.92775	0.93101
709	0.91273	0.91851	0.92116	0.92203	0.92483	0.92783	0.93109
710	0.91284	0.91861	0.92125	0.92212	0.92492	0.92791	0.93117
711	0.91294	0.91870	0.92134	0.92221	0.92500	0.92799	0.93125
712	0.91304	0.91880	0.92143	0.92230	0.92509	0.92807	0.93133
713	0.91314	0.91889	0.92152	0.92239	0.92517	0.92816	0.93140
714	0.91324	0.91898	0.92161	0.92247	0.92526	0.92824	0.93148
715	0.91334	0.91907	0.92170	0.92256	0.92535	0.92832	0.93156
716	0.91344	0.91917	0.92179	0.92265	0.92543	0.92840	0.93164
717	0.91355	0.91926	0.92188	0.92274	0.92552	0.92848	0.93171
718	0.91365	0.91935	0.92197	0.92283	0.92560	0.92856	0.93179
719	0.91374	0.91944	0.92205	0.92292	0.92569	0.92865	0.93187
720	0.91385	0.91953	0.92214	0.92301	0.92577	0.92872	0.93194
721	0.91394	0.91963	0.92223	0.92310	0.92586	0.92881	0.93202
722	0.91405	0.91972	0.92232	0.92318	0.92594	0.92888	0.93209
723	0.91415	0.91981	0.92241	0.92327	0.92602	0.92896	0.93217
724	0.91424	0.91990	0.92250	0.92335	0.92611	0.92904	0.93225
725	0.91434	0.91999	0.92258	0.92344	0.92619	0.92912	0.93232
726	0.91443	0.92008	0.92267	0.92353	0.92627	0.92920	0.93240
727	0.91454	0.92017	0.92276	0.92362	0.92636	0.92928	0.93247
728	0.91463	0.92026	0.92285	0.92370	0.92644	0.92936	0.93255
729	0.91474	0.92035	0.92294	0.92379	0.92652	0.92944	0.93262
730	0.91483	0.92044	0.92302	0.92387	0.92660	0.92952	0.93269
731	0.91494	0.92053	0.92311	0.92396	0.92668	0.92960	0.93277
732	0.91504	0.92062	0.92320	0.92405	0.92676	0.92967	0.93284
733	0.91513	0.92071	0.92328	0.92413	0.92685	0.92975	0.93292
734	0.91521	0.92080	0.92337	0.92422	0.92693	0.92983	0.93299
735	0.91530	0.92089	0.92345	0.92430	0.92701	0.92991	0.93307
736	0.91540	0.92098	0.92354	0.92439	0.92709	0.92999	0.93314
737	0.91550	0.92107	0.92363	0.92447	0.92717	0.93006	0.93321
738	0.91559	0.92115	0.92371	0.92455	0.92725	0.93014	0.93329
739	0.91568	0.92124	0.92380	0.92463	0.92733	0.93022	0.93336
740	0.91577	0.92133	0.92388	0.92472	0.92741	0.93029	0.93343
741	0.91586	0.92142	0.92396	0.92480	0.92749	0.93037	0.93350
742	0.91597	0.92150	0.92405	0.92489	0.92758	0.93045	0.93357
743	0.91606	0.92159	0.92413	0.92497	0.92765	0.93052	0.93365
744	0.91615	0.92168	0.92421	0.92506	0.92773	0.93060	0.93372
745	0.91624	0.92177	0.92430	0.92513	0.92781	0.93067	0.93379
746	0.91633	0.92185	0.92438	0.92522	0.92789	0.93075	0.93386
747	0.91642	0.92194	0.92447	0.92530	0.92797	0.93083	0.93393
748	0.91652	0.92202	0.92455	0.92538	0.92805	0.93090	0.93400
749	0.91662	0.92211	0.92463	0.92546	0.92813	0.93098	0.93408
750	0.91671	0.92220	0.92471	0.92554	0.92821	0.93105	0.93415
751	0.91680	0.92228	0.92479	0.92562	0.92828	0.93113	0.93422
752	0.91690	0.92237	0.92487	0.92571	0.92836	0.93120	0.93429
753	0.91698	0.92246	0.92496	0.92578	0.92844	0.93128	0.93436
754	0.91707	0.92254	0.92503	0.92587	0.92852	0.93135	0.93443
755	0.91717	0.92263	0.92512	0.92595	0.92859	0.93142	0.93450
756	0.91725	0.92271	0.92520	0.92603	0.92867	0.93150	0.93457
757	0.91735	0.92279	0.92528	0.92611	0.92875	0.93157	0.93464
758	0.91744	0.92287	0.92536	0.92619	0.92883	0.93164	0.93471
759	0.91753	0.92296	0.92544	0.92627	0.92890	0.93172	0.93478
760	0.91763	0.92304	0.92552	0.92635	0.92898	0.93179	0.93485
761	0.91772	0.92312	0.92560	0.92643	0.92906	0.93187	0.93492
762	0.91781	0.92321	0.92568	0.92651	0.92913	0.93194	0.93499
763	0.91789	0.92329	0.92576	0.92659	0.92921	0.93201	0.93505
764	0.91798	0.92337	0.92584	0.92666	0.92928	0.93208	0.93512
765	0.91806	0.92345	0.92593	0.92674	0.92936	0.93215	0.93519
766	0.91816	0.92354	0.92600	0.92682	0.92944	0.93222	0.93526
767	0.91824	0.92362	0.92608	0.92690	0.92951	0.93230	0.93533
768	0.91834	0.92371	0.92616	0.92698	0.92959	0.93237	0.93540
769	0.91842	0.92379	0.92624	0.92705	0.92966	0.93244	0.93547
770	0.91850	0.92386	0.92632	0.92713	0.92974	0.93251	0.93553
771	0.91859	0.92395	0.92640	0.92721	0.92981	0.93258	0.93560

N	0.1%	0.5%	1%	1.25%	2.5%	5%	10%
772	0.91867	0.92403	0.92647	0.92729	0.92989	0.93265	0.93567
773	0.91877	0.92411	0.92655	0.92736	0.92996	0.93272	0.93574
774	0.91886	0.92419	0.92663	0.92744	0.93004	0.93280	0.93580
775	0.91895	0.92428	0.92671	0.92752	0.93011	0.93287	0.93587
776	0.91903	0.92435	0.92678	0.92759	0.93018	0.93294	0.93594
777	0.91912	0.92444	0.92686	0.92767	0.93026	0.93301	0.93600
778	0.91920	0.92452	0.92694	0.92774	0.93033	0.93308	0.93607
779	0.91929	0.92460	0.92702	0.92782	0.93040	0.93315	0.93614
780	0.91937	0.92468	0.92710	0.92789	0.93048	0.93322	0.93620
781	0.91946	0.92476	0.92717	0.92797	0.93055	0.93329	0.93627
782	0.91954	0.92483	0.92725	0.92804	0.93062	0.93336	0.93633
783	0.91963	0.92491	0.92733	0.92812	0.93069	0.93343	0.93640
784	0.91971	0.92499	0.92740	0.92819	0.93077	0.93349	0.93647
785	0.91979	0.92507	0.92748	0.92827	0.93084	0.93356	0.93653
786	0.91987	0.92515	0.92755	0.92835	0.93091	0.93363	0.93660
787	0.91996	0.92523	0.92763	0.92842	0.93098	0.93370	0.93666
788	0.92004	0.92531	0.92770	0.92850	0.93105	0.93377	0.93673
789	0.92013	0.92539	0.92778	0.92857	0.93112	0.93384	0.93679
790	0.92022	0.92547	0.92786	0.92865	0.93120	0.93391	0.93686
791	0.92031	0.92555	0.92793	0.92872	0.93127	0.93397	0.93692
792	0.92040	0.92563	0.92801	0.92879	0.93134	0.93404	0.93699
793	0.92047	0.92571	0.92808	0.92887	0.93141	0.93411	0.93705
794	0.92057	0.92578	0.92816	0.92894	0.93148	0.93418	0.93712
795	0.92065	0.92586	0.92823	0.92901	0.93155	0.93425	0.93718
796	0.92073	0.92594	0.92831	0.92909	0.93162	0.93431	0.93724
797	0.92080	0.92602	0.92838	0.92916	0.93169	0.93438	0.93731
798	0.92089	0.92609	0.92846	0.92924	0.93177	0.93445	0.93737
799	0.92097	0.92617	0.92853	0.92931	0.93183	0.93452	0.93743
800	0.92105	0.92625	0.92860	0.92938	0.93190	0.93458	0.93750
801	0.92112	0.92632	0.92868	0.92945	0.93197	0.93465	0.93756
802	0.92122	0.92640	0.92875	0.92952	0.93204	0.93472	0.93762
803	0.92130	0.92647	0.92882	0.92960	0.93211	0.93478	0.93769
804	0.92137	0.92655	0.92890	0.92967	0.93218	0.93485	0.93775
805	0.92146	0.92662	0.92897	0.92974	0.93225	0.93491	0.93781
806	0.92155	0.92670	0.92904	0.92982	0.93232	0.93498	0.93787
807	0.92163	0.92677	0.92912	0.92989	0.93239	0.93504	0.93794
808	0.92171	0.92685	0.92919	0.92996	0.93246	0.93511	0.93800
809	0.92178	0.92692	0.92926	0.93003	0.93253	0.93517	0.93806
810	0.92187	0.92700	0.92933	0.93010	0.93259	0.93524	0.93812
811	0.92196	0.92707	0.92940	0.93017	0.93266	0.93531	0.93819
812	0.92204	0.92715	0.92948	0.93024	0.93273	0.93537	0.93825
813	0.92212	0.92722	0.92955	0.93031	0.93280	0.93543	0.93831
814	0.92218	0.92729	0.92962	0.93039	0.93287	0.93550	0.93837
815	0.92227	0.92737	0.92969	0.93046	0.93293	0.93557	0.93843
816	0.92235	0.92744	0.92976	0.93053	0.93300	0.93563	0.93849
817	0.92242	0.92752	0.92983	0.93060	0.93307	0.93569	0.93855
818	0.92251	0.92759	0.92990	0.93067	0.93313	0.93576	0.93862
819	0.92259	0.92766	0.92997	0.93074	0.93320	0.93582	0.93868
820	0.92268	0.92774	0.93005	0.93081	0.93327	0.93588	0.93874
821	0.92275	0.92781	0.93012	0.93088	0.93334	0.93595	0.93880
822	0.92282	0.92787	0.93019	0.93095	0.93340	0.93601	0.93886
823	0.92290	0.92795	0.93026	0.93102	0.93347	0.93607	0.93892
824	0.92298	0.92802	0.93033	0.93109	0.93353	0.93614	0.93898
825	0.92306	0.92809	0.93040	0.93115	0.93360	0.93620	0.93904
826	0.92314	0.92816	0.93046	0.93122	0.93367	0.93626	0.93910
827	0.92321	0.92823	0.93053	0.93129	0.93373	0.93633	0.93916
828	0.92329	0.92831	0.93060	0.93136	0.93380	0.93639	0.93922
829	0.92337	0.92838	0.93067	0.93143	0.93386	0.93645	0.93928
830	0.92344	0.92844	0.93074	0.93150	0.93392	0.93651	0.93934
831	0.92351	0.92852	0.93081	0.93156	0.93399	0.93658	0.93940
832	0.92359	0.92859	0.93088	0.93163	0.93405	0.93664	0.93945
833	0.92367	0.92866	0.93095	0.93169	0.93412	0.93670	0.93951
834	0.92375	0.92873	0.93102	0.93176	0.93418	0.93676	0.93957
835	0.92384	0.92881	0.93108	0.93183	0.93425	0.93682	0.93963
836	0.92391	0.92888	0.93115	0.93190	0.93431	0.93689	0.93969
837	0.92399	0.92895	0.93122	0.93197	0.93438	0.93695	0.93975
838	0.92407	0.92902	0.93129	0.93203	0.93444	0.93701	0.93981
839	0.92415	0.92909	0.93136	0.93210	0.93451	0.93707	0.93986
840	0.92423	0.92916	0.93143	0.93217	0.93457	0.93713	0.93992
841	0.92430	0.92923	0.93149	0.93224	0.93463	0.93719	0.93998
842	0.92438	0.92930	0.93156	0.93231	0.93470	0.93725	0.94004
843	0.92446	0.92937	0.93163	0.93237	0.93476	0.93732	0.94010
844	0.92453	0.92944	0.93169	0.93244	0.93483	0.93738	0.94015
845	0.92461	0.92951	0.93176	0.93251	0.93489	0.93744	0.94021

N	0.1%	0.5%	1%	1.25%	2.5%	5%	10%
846	0.92468	0.92958	0.93183	0.93257	0.93495	0.93750	0.94027
847	0.92475	0.92965	0.93189	0.93264	0.93501	0.93756	0.94033
848	0.92483	0.92971	0.93196	0.93270	0.93508	0.93762	0.94038
849	0.92490	0.92978	0.93203	0.93276	0.93514	0.93768	0.94044
850	0.92497	0.92985	0.93209	0.93283	0.93520	0.93774	0.94050
851	0.92505	0.92991	0.93215	0.93290	0.93527	0.93780	0.94055
852	0.92511	0.92998	0.93222	0.93296	0.93533	0.93786	0.94061
853	0.92519	0.93005	0.93229	0.93302	0.93539	0.93792	0.94067
854	0.92526	0.93012	0.93235	0.93309	0.93545	0.93798	0.94072
855	0.92533	0.93019	0.93242	0.93315	0.93552	0.93804	0.94078
856	0.92541	0.93025	0.93249	0.93322	0.93558	0.93810	0.94084
857	0.92549	0.93032	0.93255	0.93328	0.93564	0.93816	0.94089
858	0.92556	0.93039	0.93262	0.93335	0.93570	0.93821	0.94095
859	0.92564	0.93046	0.93268	0.93342	0.93576	0.93827	0.94100
860	0.92570	0.93053	0.93275	0.93348	0.93582	0.93833	0.94106
861	0.92577	0.93060	0.93281	0.93355	0.93589	0.93839	0.94112
862	0.92584	0.93066	0.93288	0.93361	0.93595	0.93845	0.94117
863	0.92591	0.93073	0.93294	0.93367	0.93601	0.93851	0.94123
864	0.92598	0.93079	0.93301	0.93374	0.93607	0.93857	0.94128
865	0.92605	0.93086	0.93307	0.93380	0.93613	0.93863	0.94134
866	0.92613	0.93093	0.93313	0.93386	0.93619	0.93869	0.94139
867	0.92620	0.93100	0.93319	0.93392	0.93625	0.93874	0.94145
868	0.92627	0.93106	0.93326	0.93399	0.93631	0.93880	0.94150
869	0.92634	0.93112	0.93333	0.93405	0.93637	0.93886	0.94156
870	0.92642	0.93119	0.93339	0.93411	0.93643	0.93892	0.94161
871	0.92649	0.93126	0.93345	0.93418	0.93649	0.93897	0.94167
872	0.92656	0.93132	0.93351	0.93424	0.93655	0.93903	0.94172
873	0.92663	0.93139	0.93358	0.93430	0.93661	0.93909	0.94178
874	0.92670	0.93146	0.93364	0.93436	0.93667	0.93915	0.94183
875	0.92677	0.93152	0.93370	0.93442	0.93673	0.93920	0.94188
876	0.92684	0.93159	0.93377	0.93448	0.93679	0.93926	0.94194
877	0.92691	0.93165	0.93383	0.93455	0.93685	0.93932	0.94199
878	0.92698	0.93172	0.93389	0.93461	0.93691	0.93937	0.94205
879	0.92705	0.93178	0.93395	0.93467	0.93697	0.93943	0.94210
880	0.92711	0.93184	0.93401	0.93473	0.93703	0.93949	0.94215
881	0.92719	0.93191	0.93408	0.93479	0.93709	0.93954	0.94221
882	0.92725	0.93197	0.93414	0.93485	0.93715	0.93960	0.94226
883	0.92734	0.93203	0.93420	0.93491	0.93721	0.93966	0.94232
884	0.92741	0.93210	0.93426	0.93497	0.93727	0.93971	0.94237
885	0.92747	0.93217	0.93432	0.93503	0.93732	0.93977	0.94242
886	0.92755	0.93223	0.93438	0.93509	0.93738	0.93982	0.94247
887	0.92761	0.93230	0.93444	0.93516	0.93744	0.93988	0.94253
888	0.92768	0.93236	0.93451	0.93522	0.93750	0.93993	0.94258
889	0.92774	0.93242	0.93456	0.93528	0.93756	0.93999	0.94263
890	0.92781	0.93249	0.93463	0.93534	0.93761	0.94005	0.94269
891	0.92788	0.93255	0.93469	0.93540	0.93767	0.94010	0.94274
892	0.92795	0.93261	0.93475	0.93546	0.93773	0.94016	0.94279
893	0.92802	0.93267	0.93481	0.93552	0.93779	0.94021	0.94284
894	0.92808	0.93274	0.93487	0.93558	0.93784	0.94027	0.94290
895	0.92815	0.93280	0.93493	0.93564	0.93790	0.94032	0.94295
896	0.92822	0.93286	0.93499	0.93570	0.93796	0.94037	0.94300
897	0.92829	0.93292	0.93505	0.93576	0.93802	0.94043	0.94305
898	0.92837	0.93298	0.93511	0.93582	0.93807	0.94048	0.94310
899	0.92843	0.93304	0.93517	0.93587	0.93813	0.94054	0.94316
900	0.92849	0.93311	0.93523	0.93593	0.93818	0.94059	0.94321
901	0.92855	0.93317	0.93529	0.93599	0.93824	0.94065	0.94326
902	0.92862	0.93323	0.93535	0.93605	0.93830	0.94070	0.94331
903	0.92869	0.93329	0.93541	0.93611	0.93836	0.94076	0.94336
904	0.92876	0.93336	0.93547	0.93617	0.93841	0.94081	0.94341
905	0.92884	0.93342	0.93553	0.93623	0.93847	0.94086	0.94346
906	0.92889	0.93348	0.93559	0.93628	0.93853	0.94092	0.94351
907	0.92895	0.93354	0.93565	0.93634	0.93858	0.94097	0.94356
908	0.92902	0.93360	0.93571	0.93640	0.93864	0.94102	0.94361
909	0.92908	0.93366	0.93577	0.93646	0.93869	0.94108	0.94366
910	0.92915	0.93372	0.93582	0.93652	0.93875	0.94113	0.94371
911	0.92921	0.93378	0.93588	0.93658	0.93880	0.94118	0.94376
912	0.92928	0.93384	0.93594	0.93664	0.93886	0.94123	0.94381
913	0.92934	0.93390	0.93600	0.93669	0.93892	0.94129	0.94386
914	0.92941	0.93396	0.93605	0.93675	0.93897	0.94134	0.94391
915	0.92947	0.93402	0.93611	0.93681	0.93903	0.94139	0.94396
916	0.92953	0.93408	0.93617	0.93686	0.93908	0.94144	0.94402
917	0.92960	0.93414	0.93623	0.93692	0.93914	0.94150	0.94406
918	0.92967	0.93420	0.93628	0.93698	0.93919	0.94155	0.94411
919	0.92974	0.93425	0.93634	0.93703	0.93925	0.94160	0.94416

N	0.1%	0.5%	1%	1.25%	2.5%	5%	10%
920	0.92980	0.93432	0.93640	0.93709	0.93930	0.94165	0.94421
921	0.92986	0.93438	0.93646	0.93715	0.93936	0.94171	0.94426
922	0.92993	0.93444	0.93652	0.93721	0.93941	0.94176	0.94431
923	0.92999	0.93449	0.93657	0.93726	0.93946	0.94181	0.94436
924	0.93005	0.93455	0.93663	0.93732	0.93952	0.94186	0.94441
925	0.93011	0.93462	0.93669	0.93737	0.93957	0.94191	0.94446
926	0.93017	0.93468	0.93674	0.93743	0.93962	0.94197	0.94451
927	0.93023	0.93473	0.93680	0.93748	0.93968	0.94202	0.94456
928	0.93029	0.93479	0.93685	0.93754	0.93973	0.94207	0.94460
929	0.93036	0.93485	0.93691	0.93759	0.93978	0.94212	0.94465
930	0.93041	0.93490	0.93696	0.93765	0.93984	0.94217	0.94470
931	0.93048	0.93497	0.93702	0.93770	0.93989	0.94222	0.94475
932	0.93054	0.93503	0.93708	0.93775	0.93994	0.94227	0.94480
933	0.93061	0.93508	0.93713	0.93781	0.94000	0.94232	0.94485
934	0.93068	0.93515	0.93719	0.93787	0.94005	0.94237	0.94490
935	0.93074	0.93521	0.93724	0.93792	0.94010	0.94242	0.94494
936	0.93081	0.93526	0.93730	0.93798	0.94015	0.94247	0.94499
937	0.93087	0.93533	0.93736	0.93803	0.94021	0.94252	0.94504
938	0.93093	0.93538	0.93741	0.93808	0.94026	0.94258	0.94509
939	0.93100	0.93544	0.93747	0.93814	0.94031	0.94262	0.94513
940	0.93106	0.93550	0.93752	0.93819	0.94036	0.94267	0.94518
941	0.93112	0.93555	0.93758	0.93825	0.94042	0.94273	0.94523
942	0.93118	0.93561	0.93763	0.93831	0.94047	0.94278	0.94528
943	0.93125	0.93567	0.93769	0.93836	0.94052	0.94282	0.94532
944	0.93130	0.93572	0.93774	0.93841	0.94057	0.94287	0.94537
945	0.93136	0.93578	0.93780	0.93847	0.94063	0.94292	0.94542
946	0.93141	0.93584	0.93785	0.93852	0.94068	0.94297	0.94546
947	0.93147	0.93589	0.93791	0.93857	0.94073	0.94302	0.94551
948	0.93153	0.93595	0.93796	0.93863	0.94078	0.94307	0.94556
949	0.93159	0.93600	0.93801	0.93868	0.94083	0.94312	0.94561
950	0.93164	0.93606	0.93807	0.93873	0.94088	0.94317	0.94565
951	0.93170	0.93612	0.93812	0.93879	0.94093	0.94322	0.94570
952	0.93176	0.93618	0.93818	0.93884	0.94099	0.94327	0.94575
953	0.93181	0.93623	0.93823	0.93889	0.94104	0.94332	0.94579
954	0.93187	0.93629	0.93828	0.93895	0.94109	0.94337	0.94584
955	0.93193	0.93634	0.93834	0.93900	0.94114	0.94342	0.94588
956	0.93199	0.93640	0.93839	0.93906	0.94119	0.94346	0.94593
957	0.93205	0.93645	0.93845	0.93911	0.94124	0.94351	0.94598
958	0.93211	0.93651	0.93850	0.93916	0.94129	0.94356	0.94603
959	0.93217	0.93657	0.93856	0.93922	0.94134	0.94361	0.94607
960	0.93222	0.93662	0.93861	0.93927	0.94139	0.94366	0.94612
961	0.93229	0.93668	0.93866	0.93932	0.94144	0.94371	0.94616
962	0.93235	0.93673	0.93871	0.93937	0.94149	0.94375	0.94621
963	0.93241	0.93679	0.93877	0.93943	0.94154	0.94380	0.94625
964	0.93247	0.93684	0.93882	0.93948	0.94160	0.94385	0.94630
965	0.93253	0.93690	0.93887	0.93953	0.94165	0.94390	0.94634
966	0.93258	0.93695	0.93893	0.93958	0.94170	0.94394	0.94639
967	0.93265	0.93700	0.93898	0.93963	0.94175	0.94399	0.94643
968	0.93271	0.93706	0.93903	0.93969	0.94180	0.94404	0.94648
969	0.93276	0.93711	0.93908	0.93974	0.94184	0.94409	0.94653
970	0.93282	0.93718	0.93913	0.93979	0.94189	0.94413	0.94657
971	0.93289	0.93723	0.93919	0.93984	0.94194	0.94418	0.94662
972	0.93294	0.93728	0.93924	0.93989	0.94199	0.94423	0.94666
973	0.93300	0.93734	0.93929	0.93994	0.94204	0.94428	0.94671
974	0.93306	0.93739	0.93934	0.94000	0.94209	0.94432	0.94675
975	0.93312	0.93744	0.93940	0.94005	0.94214	0.94437	0.94679
976	0.93318	0.93749	0.93945	0.94010	0.94219	0.94442	0.94684
977	0.93324	0.93755	0.93950	0.94015	0.94224	0.94446	0.94688
978	0.93329	0.93760	0.93955	0.94020	0.94229	0.94451	0.94693
979	0.93334	0.93765	0.93960	0.94025	0.94234	0.94456	0.94697
980	0.93340	0.93771	0.93965	0.94030	0.94238	0.94460	0.94702
981	0.93346	0.93776	0.93971	0.94035	0.94243	0.94465	0.94706
982	0.93352	0.93781	0.93976	0.94040	0.94248	0.94470	0.94710
983	0.93358	0.93786	0.93981	0.94045	0.94253	0.94474	0.94715
984	0.93364	0.93792	0.93986	0.94050	0.94258	0.94479	0.94719
985	0.93371	0.93797	0.93991	0.94055	0.94263	0.94483	0.94724
986	0.93377	0.93802	0.93996	0.94060	0.94267	0.94488	0.94728
987	0.93383	0.93808	0.94001	0.94066	0.94272	0.94493	0.94732
988	0.93388	0.93813	0.94006	0.94070	0.94277	0.94497	0.94737
989	0.93393	0.93818	0.94011	0.94075	0.94282	0.94502	0.94741
990	0.93399	0.93823	0.94016	0.94080	0.94287	0.94506	0.94745
991	0.93405	0.93828	0.94021	0.94085	0.94292	0.94511	0.94750
992	0.93411	0.93833	0.94026	0.94090	0.94297	0.94516	0.94754
993	0.93417	0.93839	0.94031	0.94095	0.94301	0.94520	0.94758

N	0.1%	0.5%	1%	1.25%	2.5%	5%	10%
994	0.93423	0.93844	0.94036	0.94100	0.94306	0.94525	0.94763
995	0.93428	0.93849	0.94041	0.94105	0.94311	0.94529	0.94767
996	0.93433	0.93854	0.94046	0.94110	0.94316	0.94534	0.94771
997	0.93439	0.93859	0.94051	0.94115	0.94320	0.94538	0.94776
998	0.93444	0.93864	0.94056	0.94120	0.94325	0.94543	0.94780
999	0.93450	0.93870	0.94061	0.94125	0.94330	0.94547	0.94784
1000	0.93456	0.93875	0.94066	0.94130	0.94334	0.94552	0.94789

Table 3 – *GM5(3H2L or 2H3L)*. Critical values of upper significance levels for the Grubbs multiple-5 outlier test, applicable in situations where the three highest and two lowest or two highest and three lowest observations are simultaneously identified as outliers, denoted as *GM5(3H2L)* and *GM5(2H3L)*, respectively.

N	0.1%	0.5%	1%	1.25%	2.5%	5%	10%
7	0.00000	0.00000	0.00000	0.00000	0.00001	0.00005	0.00020
8	0.00003	0.00016	0.00033	0.00042	0.00086	0.00180	0.00385
9	0.00047	0.00144	0.00236	0.00277	0.00456	0.00766	0.01313
10	0.00206	0.00485	0.00707	0.00798	0.01176	0.01758	0.02676
11	0.00527	0.01061	0.01442	0.01596	0.02196	0.03054	0.04323
12	0.01029	0.01851	0.02406	0.02623	0.03442	0.04560	0.06131
13	0.01698	0.02827	0.03553	0.03827	0.04843	0.06192	0.08022
14	0.02493	0.03931	0.04813	0.05144	0.06343	0.07896	0.09942
15	0.03421	0.05137	0.06165	0.06543	0.07909	0.09626	0.11853
16	0.04435	0.06415	0.07576	0.07999	0.09502	0.11366	0.13739
17	0.05522	0.07746	0.09027	0.09486	0.11109	0.13092	0.15579
18	0.06654	0.09120	0.10487	0.10981	0.12706	0.14779	0.17369
19	0.07834	0.10495	0.11948	0.12468	0.14271	0.16437	0.19102
20	0.09048	0.11849	0.13396	0.13936	0.15814	0.18052	0.20775
21	0.10260	0.13227	0.14816	0.15381	0.17326	0.19616	0.22389
22	0.11469	0.14567	0.16225	0.16802	0.18797	0.21137	0.23941
23	0.12647	0.15887	0.17595	0.18197	0.20232	0.22605	0.25444
24	0.13866	0.17194	0.18949	0.19557	0.21622	0.24029	0.26878
25	0.15067	0.18481	0.20264	0.20881	0.22979	0.25403	0.28262
26	0.16237	0.19733	0.21545	0.22165	0.24290	0.26728	0.29599
27	0.17405	0.20952	0.22784	0.23418	0.25562	0.28003	0.30882
28	0.18543	0.22150	0.23989	0.24632	0.26789	0.29238	0.32110
29	0.19659	0.23310	0.25176	0.25821	0.27973	0.30435	0.33302
30	0.20742	0.24430	0.26314	0.26960	0.29124	0.31586	0.34445
31	0.21821	0.25536	0.27429	0.28074	0.30242	0.32705	0.35549
32	0.22849	0.26624	0.28511	0.29155	0.31329	0.33783	0.36614
33	0.23873	0.27670	0.29564	0.30212	0.32380	0.34825	0.37640
34	0.24865	0.28692	0.30592	0.31242	0.33398	0.35831	0.38633
35	0.25861	0.29685	0.31584	0.32233	0.34390	0.36809	0.39590
36	0.26821	0.30655	0.32542	0.33191	0.35351	0.37755	0.40517
37	0.27771	0.31593	0.33488	0.34129	0.36272	0.38671	0.41411
38	0.28663	0.32498	0.34395	0.35036	0.37174	0.39555	0.42274
39	0.29555	0.33391	0.35281	0.35922	0.38048	0.40415	0.43113
40	0.30427	0.34265	0.36145	0.36787	0.38898	0.41246	0.43924
41	0.31290	0.35110	0.36984	0.37622	0.39722	0.42057	0.44708
42	0.32101	0.35929	0.37795	0.38424	0.40522	0.42845	0.45474
43	0.32899	0.36734	0.38587	0.39224	0.41300	0.43601	0.46214
44	0.33704	0.37521	0.39362	0.39989	0.42056	0.44339	0.46930
45	0.34493	0.38276	0.40111	0.40733	0.42790	0.45062	0.47624
46	0.35251	0.39011	0.40843	0.41459	0.43498	0.45758	0.48299
47	0.35994	0.39740	0.41551	0.42170	0.44200	0.46436	0.48957
48	0.36725	0.40458	0.42249	0.42864	0.44874	0.47092	0.49596
49	0.37440	0.41134	0.42931	0.43540	0.45534	0.47739	0.50216
50	0.38117	0.41818	0.43589	0.44194	0.46179	0.48360	0.50820
51	0.38798	0.42467	0.44233	0.44837	0.46807	0.48969	0.51408
52	0.39464	0.43111	0.44867	0.45459	0.47418	0.49562	0.51977
53	0.40098	0.43724	0.45477	0.46061	0.48010	0.50141	0.52534
54	0.40750	0.44336	0.46069	0.46661	0.48596	0.50708	0.53078
55	0.41350	0.44931	0.46659	0.47245	0.49163	0.51257	0.53608
56	0.41947	0.45521	0.47228	0.47813	0.49719	0.51798	0.54126
57	0.42546	0.46085	0.47792	0.48368	0.50257	0.52322	0.54631
58	0.43128	0.46641	0.48331	0.48907	0.50787	0.52834	0.55122
59	0.43705	0.47183	0.48869	0.49435	0.51298	0.53334	0.55598
60	0.44245	0.47714	0.49388	0.49953	0.51803	0.53818	0.56065
61	0.44776	0.48233	0.49903	0.50461	0.52297	0.54294	0.56520
62	0.45308	0.48745	0.50398	0.50959	0.52781	0.54759	0.56965
63	0.45815	0.49255	0.50888	0.51441	0.53248	0.55215	0.57401
64	0.46338	0.49741	0.51364	0.51916	0.53711	0.55659	0.57827

N	0.1%	0.5%	1%	1.25%	2.5%	5%	10%
65	0.46844	0.50218	0.51830	0.52377	0.54166	0.56093	0.58244
66	0.47319	0.50690	0.52291	0.52831	0.54603	0.56517	0.58651
67	0.47806	0.51146	0.52743	0.53279	0.55032	0.56935	0.59050
68	0.48286	0.51600	0.53187	0.53714	0.55455	0.57343	0.59441
69	0.48754	0.52047	0.53616	0.54144	0.55867	0.57742	0.59824
70	0.49200	0.52482	0.54042	0.54565	0.56273	0.58131	0.60196
71	0.49647	0.52910	0.54457	0.54977	0.56671	0.58512	0.60562
72	0.50071	0.53324	0.54857	0.55375	0.57060	0.58888	0.60920
73	0.50491	0.53732	0.55256	0.55772	0.57443	0.59255	0.61272
74	0.50913	0.54134	0.55646	0.56157	0.57813	0.59617	0.61617
75	0.51335	0.54527	0.56033	0.56542	0.58181	0.59969	0.61954
76	0.51743	0.54911	0.56411	0.56912	0.58538	0.60315	0.62286
77	0.52135	0.55285	0.56775	0.57271	0.58894	0.60655	0.62610
78	0.52532	0.55661	0.57136	0.57630	0.59240	0.60990	0.62927
79	0.52912	0.56028	0.57491	0.57981	0.59578	0.61316	0.63238
80	0.53288	0.56382	0.57841	0.58325	0.59912	0.61637	0.63546
81	0.53668	0.56729	0.58185	0.58664	0.60239	0.61952	0.63846
82	0.54029	0.57081	0.58516	0.58997	0.60558	0.62264	0.64141
83	0.54380	0.57423	0.58845	0.59320	0.60875	0.62567	0.64431
84	0.54721	0.57750	0.59170	0.59645	0.61188	0.62866	0.64716
85	0.55074	0.58075	0.59488	0.59960	0.61496	0.63158	0.64996
86	0.55421	0.58403	0.59802	0.60273	0.61796	0.63446	0.65269
87	0.55742	0.58722	0.60109	0.60578	0.62094	0.63729	0.65538
88	0.56080	0.59042	0.60416	0.60880	0.62384	0.64005	0.65802
89	0.56417	0.59343	0.60711	0.61172	0.62668	0.64279	0.66063
90	0.56738	0.59647	0.61003	0.61465	0.62949	0.64547	0.66320
91	0.57065	0.59946	0.61293	0.61752	0.63225	0.64813	0.66572
92	0.57370	0.60238	0.61579	0.62033	0.63498	0.65072	0.66819
93	0.57678	0.60522	0.61860	0.62311	0.63761	0.65329	0.67062
94	0.57976	0.60806	0.62135	0.62580	0.64023	0.65580	0.67302
95	0.58271	0.61079	0.62402	0.62849	0.64282	0.65829	0.67539
96	0.58568	0.61356	0.62671	0.63110	0.64536	0.66071	0.67771
97	0.58839	0.61630	0.62934	0.63368	0.64787	0.66311	0.67999
98	0.59134	0.61895	0.63192	0.63626	0.65033	0.66549	0.68223
99	0.59416	0.62159	0.63445	0.63878	0.65276	0.66783	0.68443
100	0.59690	0.62419	0.63697	0.64124	0.65515	0.67012	0.68661
101	0.59967	0.62669	0.63942	0.64368	0.65753	0.67238	0.68876
102	0.60236	0.62912	0.64184	0.64610	0.65984	0.67462	0.69086
103	0.60487	0.63163	0.64425	0.64847	0.66212	0.67680	0.69296
104	0.60754	0.63412	0.64661	0.65081	0.66435	0.67896	0.69500
105	0.61007	0.63651	0.64894	0.65312	0.66659	0.68108	0.69701
106	0.61257	0.63888	0.65125	0.65539	0.66876	0.68317	0.69899
107	0.61505	0.64121	0.65352	0.65759	0.67094	0.68524	0.70095
108	0.61744	0.64355	0.65582	0.65982	0.67309	0.68729	0.70287
109	0.61982	0.64580	0.65799	0.66200	0.67517	0.68928	0.70478
110	0.62221	0.64805	0.66011	0.66412	0.67722	0.69125	0.70666
111	0.62463	0.65029	0.66226	0.66626	0.67927	0.69320	0.70851
112	0.62695	0.65245	0.66437	0.66837	0.68124	0.69512	0.71033
113	0.62932	0.65462	0.66644	0.67043	0.68321	0.69702	0.71213
114	0.63152	0.65669	0.66850	0.67245	0.68515	0.69888	0.71390
115	0.63374	0.65877	0.67053	0.67444	0.68709	0.70071	0.71564
116	0.63582	0.66082	0.67250	0.67638	0.68897	0.70252	0.71737
117	0.63812	0.66288	0.67446	0.67834	0.69084	0.70431	0.71907
118	0.64030	0.66484	0.67642	0.68026	0.69268	0.70608	0.72075
119	0.64227	0.66688	0.67835	0.68215	0.69448	0.70784	0.72240
120	0.64429	0.66885	0.68024	0.68404	0.69630	0.70955	0.72403
121	0.64647	0.67083	0.68212	0.68589	0.69808	0.71124	0.72565
122	0.64841	0.67273	0.68395	0.68772	0.69984	0.71292	0.72724
123	0.65056	0.67461	0.68580	0.68953	0.70158	0.71457	0.72881
124	0.65249	0.67650	0.68759	0.69132	0.70329	0.71622	0.73037
125	0.65458	0.67834	0.68935	0.69305	0.70498	0.71783	0.73189
126	0.65647	0.68015	0.69109	0.69478	0.70663	0.71943	0.73341
127	0.65838	0.68195	0.69284	0.69648	0.70829	0.72099	0.73490
128	0.66032	0.68371	0.69455	0.69818	0.70990	0.72255	0.73638
129	0.66211	0.68547	0.69625	0.69984	0.71151	0.72409	0.73785
130	0.66388	0.68723	0.69793	0.70151	0.71311	0.72560	0.73928
131	0.66573	0.68890	0.69958	0.70314	0.71465	0.72710	0.74070
132	0.66758	0.69053	0.70120	0.70473	0.71620	0.72858	0.74210
133	0.66927	0.69216	0.70279	0.70633	0.71774	0.73005	0.74349
134	0.67102	0.69388	0.70436	0.70790	0.71923	0.73149	0.74486
135	0.67281	0.69550	0.70597	0.70945	0.72072	0.73291	0.74621
136	0.67456	0.69709	0.70752	0.71097	0.72217	0.73432	0.74755
137	0.67627	0.69871	0.70904	0.71248	0.72362	0.73571	0.74887
138	0.67800	0.70026	0.71053	0.71397	0.72509	0.73709	0.75019

N	0.1%	0.5%	1%	1.25%	2.5%	5%	10%
139	0.67966	0.70181	0.71204	0.71543	0.72649	0.73844	0.75148
140	0.68130	0.70336	0.71354	0.71689	0.72791	0.73978	0.75276
141	0.68293	0.70491	0.71500	0.71834	0.72931	0.74110	0.75401
142	0.68449	0.70638	0.71645	0.71980	0.73069	0.74243	0.75526
143	0.68602	0.70787	0.71787	0.72122	0.73206	0.74374	0.75650
144	0.68770	0.70933	0.71929	0.72261	0.73342	0.74503	0.75772
145	0.68917	0.71078	0.72068	0.72401	0.73475	0.74630	0.75892
146	0.69070	0.71220	0.72208	0.72539	0.73606	0.74756	0.76011
147	0.69222	0.71361	0.72345	0.72674	0.73738	0.74880	0.76129
148	0.69371	0.71503	0.72481	0.72807	0.73867	0.75002	0.76245
149	0.69523	0.71641	0.72616	0.72942	0.73995	0.75123	0.76360
150	0.69670	0.71780	0.72752	0.73073	0.74121	0.75245	0.76474
151	0.69818	0.71914	0.72880	0.73202	0.74246	0.75364	0.76588
152	0.69967	0.72047	0.73007	0.73330	0.74369	0.75483	0.76701
153	0.70117	0.72184	0.73139	0.73457	0.74492	0.75599	0.76812
154	0.70257	0.72319	0.73265	0.73583	0.74613	0.75714	0.76921
155	0.70401	0.72447	0.73393	0.73708	0.74735	0.75829	0.77029
156	0.70540	0.72577	0.73516	0.73831	0.74852	0.75942	0.77136
157	0.70671	0.72704	0.73637	0.73951	0.74969	0.76054	0.77243
158	0.70814	0.72832	0.73761	0.74073	0.75083	0.76165	0.77348
159	0.70948	0.72961	0.73882	0.74192	0.75199	0.76274	0.77452
160	0.71080	0.73083	0.74002	0.74310	0.75311	0.76382	0.77555
161	0.71211	0.73208	0.74123	0.74429	0.75424	0.76489	0.77657
162	0.71339	0.73327	0.74238	0.74545	0.75536	0.76595	0.77757
163	0.71472	0.73447	0.74355	0.74660	0.75646	0.76701	0.77858
164	0.71607	0.73564	0.74471	0.74774	0.75754	0.76805	0.77956
165	0.71727	0.73681	0.74583	0.74886	0.75863	0.76908	0.78054
166	0.71846	0.73799	0.74697	0.74999	0.75970	0.77010	0.78151
167	0.71969	0.73915	0.74806	0.75108	0.76075	0.77111	0.78246
168	0.72093	0.74028	0.74920	0.75217	0.76182	0.77212	0.78342
169	0.72223	0.74143	0.75028	0.75325	0.76285	0.77311	0.78436
170	0.72341	0.74254	0.75136	0.75432	0.76389	0.77409	0.78528
171	0.72460	0.74367	0.75246	0.75539	0.76492	0.77508	0.78620
172	0.72582	0.74479	0.75350	0.75644	0.76592	0.77604	0.78712
173	0.72701	0.74591	0.75458	0.75749	0.76693	0.77700	0.78803
174	0.72810	0.74701	0.75562	0.75853	0.76793	0.77795	0.78892
175	0.72921	0.74809	0.75665	0.75954	0.76890	0.77888	0.78980
176	0.73031	0.74914	0.75768	0.76054	0.76988	0.77981	0.79069
177	0.73147	0.75021	0.75873	0.76155	0.77084	0.78073	0.79156
178	0.73264	0.75125	0.75972	0.76256	0.77180	0.78165	0.79241
179	0.73375	0.75226	0.76069	0.76356	0.77273	0.78255	0.79328
180	0.73485	0.75330	0.76170	0.76453	0.77369	0.78345	0.79412
181	0.73596	0.75429	0.76268	0.76550	0.77462	0.78433	0.79496
182	0.73708	0.75530	0.76367	0.76645	0.77554	0.78521	0.79580
183	0.73818	0.75629	0.76463	0.76742	0.77644	0.78608	0.79662
184	0.73925	0.75730	0.76557	0.76837	0.77735	0.78695	0.79743
185	0.74024	0.75826	0.76651	0.76928	0.77824	0.78780	0.79824
186	0.74127	0.75922	0.76747	0.77022	0.77913	0.78866	0.79905
187	0.74235	0.76017	0.76840	0.77115	0.78001	0.78950	0.79984
188	0.74334	0.76112	0.76933	0.77207	0.78087	0.79034	0.80063
189	0.74437	0.76205	0.77025	0.77296	0.78173	0.79115	0.80141
190	0.74535	0.76303	0.77115	0.77386	0.78261	0.79197	0.80219
191	0.74636	0.76393	0.77204	0.77473	0.78346	0.79279	0.80296
192	0.74732	0.76485	0.77293	0.77563	0.78430	0.79360	0.80373
193	0.74831	0.76576	0.77382	0.77649	0.78514	0.79440	0.80449
194	0.74930	0.76665	0.77469	0.77736	0.78596	0.79519	0.80524
195	0.75026	0.76757	0.77557	0.77821	0.78679	0.79597	0.80597
196	0.75127	0.76846	0.77644	0.77907	0.78760	0.79674	0.80671
197	0.75218	0.76933	0.77728	0.77992	0.78842	0.79752	0.80744
198	0.75308	0.77022	0.77813	0.78074	0.78921	0.79827	0.80816
199	0.75399	0.77110	0.77896	0.78157	0.79001	0.79903	0.80888
200	0.75495	0.77197	0.77979	0.78240	0.79080	0.79979	0.80960
201	0.75585	0.77280	0.78062	0.78320	0.79158	0.80053	0.81031
202	0.75675	0.77368	0.78144	0.78402	0.79236	0.80128	0.81100
203	0.75772	0.77454	0.78226	0.78484	0.79313	0.80201	0.81170
204	0.75862	0.77538	0.78307	0.78563	0.79388	0.80273	0.81238
205	0.75943	0.77620	0.78386	0.78640	0.79464	0.80346	0.81307
206	0.76041	0.77703	0.78466	0.78719	0.79539	0.80417	0.81375
207	0.76125	0.77785	0.78545	0.78799	0.79613	0.80488	0.81442
208	0.76215	0.77864	0.78624	0.78877	0.79687	0.80558	0.81509
209	0.76300	0.77944	0.78701	0.78952	0.79761	0.80627	0.81576
210	0.76386	0.78023	0.78779	0.79028	0.79832	0.80697	0.81641
211	0.76469	0.78101	0.78854	0.79103	0.79905	0.80766	0.81707
212	0.76556	0.78183	0.78931	0.79178	0.79977	0.80834	0.81771

N	0.1%	0.5%	1%	1.25%	2.5%	5%	10%
213	0.76639	0.78261	0.79005	0.79252	0.80048	0.80901	0.81835
214	0.76729	0.78338	0.79079	0.79325	0.80119	0.80968	0.81899
215	0.76807	0.78415	0.79153	0.79397	0.80189	0.81035	0.81962
216	0.76891	0.78491	0.79226	0.79471	0.80259	0.81101	0.82025
217	0.76971	0.78565	0.79300	0.79542	0.80329	0.81167	0.82087
218	0.77053	0.78643	0.79372	0.79613	0.80397	0.81232	0.82149
219	0.77134	0.78714	0.79443	0.79683	0.80465	0.81297	0.82210
220	0.77215	0.78789	0.79513	0.79753	0.80533	0.81361	0.82271
221	0.77295	0.78862	0.79585	0.79824	0.80600	0.81425	0.82332
222	0.77374	0.78932	0.79655	0.79892	0.80666	0.81489	0.82391
223	0.77455	0.79004	0.79724	0.79960	0.80732	0.81551	0.82450
224	0.77531	0.79076	0.79793	0.80027	0.80798	0.81614	0.82510
225	0.77603	0.79149	0.79861	0.80096	0.80862	0.81676	0.82568
226	0.77682	0.79215	0.79929	0.80163	0.80926	0.81738	0.82626
227	0.77757	0.79286	0.79994	0.80230	0.80989	0.81799	0.82684
228	0.77832	0.79358	0.80061	0.80295	0.81052	0.81860	0.82741
229	0.77901	0.79426	0.80128	0.80361	0.81115	0.81920	0.82798
230	0.77973	0.79495	0.80195	0.80425	0.81177	0.81979	0.82855
231	0.78048	0.79563	0.80260	0.80491	0.81240	0.82039	0.82911
232	0.78119	0.79629	0.80324	0.80554	0.81302	0.82096	0.82966
233	0.78191	0.79693	0.80389	0.80618	0.81363	0.82155	0.83022
234	0.78259	0.79759	0.80453	0.80682	0.81423	0.82212	0.83076
235	0.78330	0.79824	0.80515	0.80743	0.81484	0.82270	0.83131
236	0.78400	0.79890	0.80580	0.80806	0.81544	0.82328	0.83185
237	0.78472	0.79955	0.80642	0.80868	0.81603	0.82384	0.83238
238	0.78545	0.80020	0.80704	0.80930	0.81662	0.82440	0.83291
239	0.78611	0.80085	0.80766	0.80991	0.81721	0.82496	0.83344
240	0.78681	0.80149	0.80828	0.81053	0.81778	0.82552	0.83396
241	0.78747	0.80216	0.80888	0.81113	0.81836	0.82607	0.83449
242	0.78815	0.80277	0.80947	0.81172	0.81893	0.82662	0.83500
243	0.78883	0.80341	0.81009	0.81232	0.81951	0.82716	0.83552
244	0.78954	0.80404	0.81068	0.81292	0.82007	0.82770	0.83603
245	0.79019	0.80465	0.81127	0.81351	0.82063	0.82823	0.83654
246	0.79084	0.80527	0.81187	0.81407	0.82119	0.82876	0.83704
247	0.79149	0.80588	0.81245	0.81465	0.82174	0.82929	0.83755
248	0.79214	0.80646	0.81302	0.81522	0.82229	0.82982	0.83805
249	0.79279	0.80705	0.81361	0.81579	0.82284	0.83034	0.83854
250	0.79343	0.80766	0.81417	0.81635	0.82338	0.83086	0.83903
251	0.79406	0.80823	0.81474	0.81692	0.82391	0.83137	0.83952
252	0.79470	0.80884	0.81530	0.81747	0.82445	0.83188	0.84001
253	0.79534	0.80942	0.81587	0.81804	0.82498	0.83239	0.84049
254	0.79597	0.80998	0.81641	0.81858	0.82551	0.83290	0.84097
255	0.79659	0.81055	0.81696	0.81913	0.82603	0.83340	0.84144
256	0.79720	0.81112	0.81753	0.81968	0.82655	0.83389	0.84191
257	0.79779	0.81170	0.81808	0.82022	0.82707	0.83438	0.84238
258	0.79840	0.81228	0.81862	0.82075	0.82759	0.83487	0.84284
259	0.79903	0.81283	0.81917	0.82128	0.82810	0.83537	0.84330
260	0.79965	0.81338	0.81971	0.82181	0.82861	0.83585	0.84376
261	0.80021	0.81394	0.82023	0.82234	0.82912	0.83633	0.84421
262	0.80081	0.81450	0.82076	0.82286	0.82962	0.83680	0.84467
263	0.80142	0.81503	0.82129	0.82339	0.83011	0.83728	0.84512
264	0.80200	0.81558	0.82181	0.82391	0.83061	0.83775	0.84557
265	0.80258	0.81611	0.82233	0.82442	0.83110	0.83822	0.84601
266	0.80319	0.81665	0.82286	0.82492	0.83159	0.83869	0.84645
267	0.80373	0.81718	0.82337	0.82542	0.83208	0.83915	0.84689
268	0.80425	0.81773	0.82389	0.82592	0.83256	0.83961	0.84733
269	0.80485	0.81825	0.82439	0.82643	0.83304	0.84007	0.84777
270	0.80546	0.81875	0.82490	0.82692	0.83352	0.84052	0.84820
271	0.80600	0.81927	0.82537	0.82742	0.83399	0.84097	0.84863
272	0.80655	0.81978	0.82589	0.82792	0.83447	0.84143	0.84905
273	0.80710	0.82032	0.82638	0.82841	0.83494	0.84188	0.84947
274	0.80759	0.82083	0.82686	0.82889	0.83540	0.84232	0.84990
275	0.80818	0.82134	0.82736	0.82936	0.83586	0.84276	0.85032
276	0.80871	0.82184	0.82784	0.82984	0.83632	0.84319	0.85073
277	0.80929	0.82235	0.82833	0.83033	0.83678	0.84363	0.85115
278	0.80982	0.82284	0.82881	0.83081	0.83723	0.84407	0.85155
279	0.81038	0.82334	0.82928	0.83127	0.83769	0.84451	0.85196
280	0.81087	0.82382	0.82976	0.83173	0.83813	0.84494	0.85237
281	0.81139	0.82432	0.83024	0.83220	0.83858	0.84536	0.85277
282	0.81193	0.82481	0.83070	0.83266	0.83902	0.84578	0.85317
283	0.81245	0.82529	0.83118	0.83312	0.83945	0.84620	0.85357
284	0.81294	0.82577	0.83163	0.83358	0.83989	0.84661	0.85396
285	0.81350	0.82624	0.83208	0.83403	0.84033	0.84703	0.85436
286	0.81399	0.82672	0.83256	0.83447	0.84076	0.84744	0.85475

N	0.1%	0.5%	1%	1.25%	2.5%	5%	10%
287	0.81452	0.82719	0.83301	0.83492	0.84118	0.84785	0.85514
288	0.81498	0.82767	0.83345	0.83538	0.84161	0.84826	0.85553
289	0.81547	0.82814	0.83391	0.83582	0.84204	0.84866	0.85591
290	0.81596	0.82860	0.83435	0.83626	0.84247	0.84906	0.85629
291	0.81646	0.82905	0.83479	0.83670	0.84289	0.84947	0.85667
292	0.81693	0.82953	0.83523	0.83715	0.84331	0.84986	0.85705
293	0.81743	0.82997	0.83566	0.83758	0.84373	0.85026	0.85743
294	0.81792	0.83042	0.83611	0.83801	0.84414	0.85065	0.85780
295	0.81842	0.83087	0.83654	0.83845	0.84455	0.85104	0.85817
296	0.81889	0.83131	0.83697	0.83887	0.84495	0.85143	0.85854
297	0.81935	0.83176	0.83740	0.83930	0.84537	0.85182	0.85891
298	0.81980	0.83220	0.83782	0.83972	0.84577	0.85221	0.85927
299	0.82027	0.83265	0.83825	0.84013	0.84617	0.85259	0.85963
300	0.82076	0.83309	0.83866	0.84055	0.84657	0.85297	0.85999
301	0.82124	0.83352	0.83908	0.84096	0.84696	0.85336	0.86035
302	0.82170	0.83395	0.83950	0.84135	0.84736	0.85373	0.86070
303	0.82216	0.83436	0.83991	0.84176	0.84775	0.85410	0.86106
304	0.82264	0.83478	0.84032	0.84216	0.84814	0.85447	0.86141
305	0.82310	0.83520	0.84074	0.84258	0.84852	0.85484	0.86176
306	0.82355	0.83564	0.84114	0.84298	0.84891	0.85521	0.86211
307	0.82402	0.83607	0.84155	0.84338	0.84929	0.85558	0.86246
308	0.82447	0.83649	0.84195	0.84379	0.84967	0.85594	0.86280
309	0.82494	0.83691	0.84235	0.84418	0.85005	0.85630	0.86314
310	0.82536	0.83732	0.84275	0.84457	0.85043	0.85666	0.86349
311	0.82580	0.83773	0.84316	0.84496	0.85081	0.85702	0.86382
312	0.82624	0.83814	0.84355	0.84535	0.85119	0.85738	0.86416
313	0.82668	0.83853	0.84394	0.84574	0.85156	0.85773	0.86449
314	0.82714	0.83894	0.84433	0.84613	0.85193	0.85808	0.86483
315	0.82754	0.83934	0.84472	0.84652	0.85230	0.85843	0.86516
316	0.82796	0.83974	0.84512	0.84689	0.85266	0.85878	0.86549
317	0.82838	0.84012	0.84549	0.84728	0.85302	0.85913	0.86582
318	0.82883	0.84052	0.84588	0.84765	0.85339	0.85947	0.86614
319	0.82928	0.84091	0.84626	0.84803	0.85375	0.85981	0.86647
320	0.82968	0.84131	0.84664	0.84840	0.85410	0.86016	0.86679
321	0.83009	0.84170	0.84701	0.84877	0.85446	0.86050	0.86712
322	0.83053	0.84209	0.84738	0.84913	0.85481	0.86083	0.86744
323	0.83092	0.84248	0.84776	0.84950	0.85516	0.86117	0.86776
324	0.83136	0.84287	0.84813	0.84986	0.85551	0.86150	0.86807
325	0.83173	0.84326	0.84849	0.85022	0.85585	0.86184	0.86839
326	0.83214	0.84364	0.84886	0.85059	0.85620	0.86216	0.86870
327	0.83257	0.84403	0.84922	0.85095	0.85654	0.86249	0.86900
328	0.83293	0.84439	0.84957	0.85130	0.85688	0.86281	0.86931
329	0.83334	0.84475	0.84994	0.85166	0.85722	0.86314	0.86962
330	0.83375	0.84512	0.85030	0.85201	0.85756	0.86346	0.86993
331	0.83413	0.84549	0.85065	0.85236	0.85789	0.86379	0.87023
332	0.83453	0.84586	0.85101	0.85270	0.85823	0.86410	0.87054
333	0.83493	0.84621	0.85136	0.85306	0.85856	0.86443	0.87084
334	0.83529	0.84657	0.85171	0.85341	0.85889	0.86474	0.87114
335	0.83570	0.84695	0.85205	0.85376	0.85922	0.86506	0.87144
336	0.83614	0.84730	0.85241	0.85410	0.85955	0.86538	0.87174
337	0.83653	0.84766	0.85275	0.85444	0.85987	0.86569	0.87203
338	0.83689	0.84802	0.85311	0.85478	0.86019	0.86600	0.87233
339	0.83728	0.84837	0.85345	0.85511	0.86052	0.86630	0.87263
340	0.83765	0.84873	0.85378	0.85545	0.86085	0.86661	0.87291
341	0.83804	0.84908	0.85413	0.85580	0.86117	0.86692	0.87321
342	0.83843	0.84942	0.85446	0.85612	0.86149	0.86722	0.87350
343	0.83876	0.84977	0.85479	0.85645	0.86181	0.86752	0.87378
344	0.83915	0.85011	0.85512	0.85677	0.86212	0.86782	0.87407
345	0.83952	0.85045	0.85546	0.85711	0.86243	0.86812	0.87435
346	0.83986	0.85079	0.85579	0.85744	0.86275	0.86842	0.87463
347	0.84023	0.85114	0.85613	0.85776	0.86306	0.86872	0.87491
348	0.84059	0.85148	0.85645	0.85808	0.86337	0.86901	0.87519
349	0.84097	0.85183	0.85678	0.85841	0.86368	0.86931	0.87547
350	0.84134	0.85216	0.85710	0.85873	0.86399	0.86960	0.87575
351	0.84172	0.85250	0.85743	0.85905	0.86429	0.86989	0.87602
352	0.84206	0.85282	0.85774	0.85937	0.86459	0.87018	0.87630
353	0.84242	0.85316	0.85806	0.85968	0.86489	0.87047	0.87657
354	0.84274	0.85350	0.85838	0.85999	0.86520	0.87076	0.87684
355	0.84312	0.85382	0.85870	0.86031	0.86549	0.87104	0.87711
356	0.84346	0.85413	0.85902	0.86061	0.86579	0.87133	0.87738
357	0.84383	0.85445	0.85934	0.86092	0.86609	0.87161	0.87765
358	0.84414	0.85478	0.85964	0.86125	0.86639	0.87189	0.87791
359	0.84452	0.85510	0.85995	0.86155	0.86667	0.87217	0.87817
360	0.84487	0.85543	0.86025	0.86185	0.86697	0.87245	0.87844

N	0.1%	0.5%	1%	1.25%	2.5%	5%	10%
361	0.84519	0.85574	0.86057	0.86216	0.86726	0.87273	0.87870
362	0.84553	0.85605	0.86087	0.86246	0.86755	0.87300	0.87896
363	0.84589	0.85636	0.86118	0.86276	0.86784	0.87328	0.87922
364	0.84623	0.85668	0.86147	0.86306	0.86812	0.87356	0.87948
365	0.84656	0.85699	0.86177	0.86335	0.86841	0.87382	0.87974
366	0.84690	0.85730	0.86208	0.86365	0.86868	0.87410	0.88000
367	0.84723	0.85761	0.86238	0.86394	0.86897	0.87436	0.88026
368	0.84754	0.85792	0.86267	0.86423	0.86925	0.87463	0.88051
369	0.84786	0.85823	0.86297	0.86452	0.86953	0.87490	0.88076
370	0.84822	0.85853	0.86327	0.86482	0.86981	0.87516	0.88102
371	0.84854	0.85885	0.86356	0.86511	0.87009	0.87543	0.88127
372	0.84887	0.85915	0.86385	0.86539	0.87036	0.87569	0.88152
373	0.84919	0.85945	0.86415	0.86567	0.87064	0.87595	0.88177
374	0.84952	0.85975	0.86444	0.86596	0.87091	0.87622	0.88201
375	0.84984	0.86006	0.86472	0.86624	0.87118	0.87648	0.88226
376	0.85016	0.86035	0.86501	0.86653	0.87145	0.87674	0.88250
377	0.85047	0.86064	0.86529	0.86681	0.87172	0.87699	0.88275
378	0.85081	0.86093	0.86557	0.86709	0.87199	0.87725	0.88299
379	0.85113	0.86123	0.86586	0.86736	0.87225	0.87750	0.88323
380	0.85145	0.86151	0.86614	0.86765	0.87252	0.87776	0.88347
381	0.85177	0.86181	0.86641	0.86792	0.87278	0.87801	0.88371
382	0.85208	0.86208	0.86670	0.86820	0.87304	0.87827	0.88395
383	0.85238	0.86238	0.86697	0.86846	0.87331	0.87851	0.88419
384	0.85269	0.86266	0.86725	0.86874	0.87357	0.87876	0.88443
385	0.85303	0.86295	0.86751	0.86901	0.87383	0.87901	0.88466
386	0.85333	0.86323	0.86779	0.86928	0.87409	0.87926	0.88490
387	0.85362	0.86351	0.86806	0.86955	0.87435	0.87950	0.88512
388	0.85393	0.86378	0.86833	0.86982	0.87461	0.87975	0.88536
389	0.85423	0.86406	0.86860	0.87008	0.87486	0.88000	0.88559
390	0.85452	0.86434	0.86886	0.87035	0.87512	0.88024	0.88582
391	0.85484	0.86463	0.86913	0.87061	0.87537	0.88048	0.88605
392	0.85511	0.86490	0.86939	0.87087	0.87562	0.88072	0.88628
393	0.85541	0.86517	0.86965	0.87113	0.87587	0.88096	0.88650
394	0.85571	0.86545	0.86991	0.87139	0.87612	0.88119	0.88673
395	0.85600	0.86572	0.87018	0.87165	0.87637	0.88143	0.88695
396	0.85629	0.86599	0.87044	0.87192	0.87662	0.88167	0.88717
397	0.85657	0.86626	0.87070	0.87217	0.87686	0.88190	0.88740
398	0.85686	0.86654	0.87095	0.87242	0.87711	0.88214	0.88762
399	0.85714	0.86681	0.87123	0.87268	0.87735	0.88237	0.88784
400	0.85742	0.86707	0.87148	0.87293	0.87760	0.88260	0.88807
401	0.85772	0.86733	0.87173	0.87318	0.87784	0.88284	0.88828
402	0.85801	0.86759	0.87200	0.87343	0.87808	0.88307	0.88850
403	0.85832	0.86785	0.87225	0.87369	0.87832	0.88330	0.88872
404	0.85859	0.86812	0.87250	0.87393	0.87857	0.88353	0.88894
405	0.85888	0.86838	0.87275	0.87418	0.87880	0.88375	0.88916
406	0.85914	0.86864	0.87300	0.87443	0.87904	0.88398	0.88937
407	0.85942	0.86889	0.87325	0.87467	0.87928	0.88420	0.88958
408	0.85969	0.86916	0.87349	0.87493	0.87951	0.88443	0.88980
409	0.85999	0.86942	0.87373	0.87517	0.87974	0.88466	0.89001
410	0.86027	0.86967	0.87399	0.87541	0.87997	0.88488	0.89022
411	0.86051	0.86993	0.87423	0.87565	0.88020	0.88510	0.89043
412	0.86078	0.87018	0.87447	0.87589	0.88044	0.88532	0.89064
413	0.86106	0.87044	0.87472	0.87613	0.88067	0.88554	0.89084
414	0.86135	0.87069	0.87495	0.87637	0.88090	0.88576	0.89105
415	0.86161	0.87094	0.87520	0.87660	0.88113	0.88598	0.89126
416	0.86189	0.87118	0.87543	0.87684	0.88135	0.88619	0.89146
417	0.86215	0.87143	0.87567	0.87708	0.88159	0.88641	0.89167
418	0.86242	0.87167	0.87591	0.87732	0.88181	0.88662	0.89187
419	0.86269	0.87192	0.87614	0.87755	0.88203	0.88684	0.89208
420	0.86296	0.87217	0.87638	0.87778	0.88225	0.88705	0.89228
421	0.86323	0.87240	0.87661	0.87801	0.88248	0.88726	0.89248
422	0.86350	0.87265	0.87685	0.87824	0.88270	0.88747	0.89268
423	0.86376	0.87289	0.87709	0.87847	0.88292	0.88768	0.89288
424	0.86400	0.87312	0.87732	0.87870	0.88315	0.88789	0.89308
425	0.86426	0.87337	0.87754	0.87892	0.88337	0.88810	0.89327
426	0.86453	0.87360	0.87777	0.87915	0.88359	0.88831	0.89347
427	0.86478	0.87384	0.87800	0.87938	0.88381	0.88852	0.89367
428	0.86502	0.87407	0.87823	0.87960	0.88402	0.88872	0.89387
429	0.86529	0.87430	0.87845	0.87982	0.88423	0.88893	0.89406
430	0.86554	0.87455	0.87868	0.88005	0.88445	0.88913	0.89426
431	0.86580	0.87478	0.87891	0.88027	0.88466	0.88934	0.89445
432	0.86608	0.87502	0.87913	0.88049	0.88487	0.88954	0.89464
433	0.86632	0.87525	0.87936	0.88071	0.88509	0.88975	0.89483
434	0.86658	0.87548	0.87959	0.88094	0.88530	0.88995	0.89503

N	0.1%	0.5%	1%	1.25%	2.5%	5%	10%
435	0.86683	0.87571	0.87981	0.88116	0.88551	0.89015	0.89522
436	0.86707	0.87595	0.88004	0.88138	0.88572	0.89035	0.89540
437	0.86732	0.87619	0.88026	0.88160	0.88592	0.89054	0.89560
438	0.86757	0.87641	0.88048	0.88181	0.88613	0.89074	0.89578
439	0.86779	0.87664	0.88069	0.88203	0.88634	0.89094	0.89597
440	0.86805	0.87686	0.88091	0.88224	0.88655	0.89114	0.89616
441	0.86829	0.87709	0.88113	0.88246	0.88675	0.89133	0.89634
442	0.86852	0.87731	0.88134	0.88267	0.88696	0.89153	0.89653
443	0.86875	0.87754	0.88156	0.88289	0.88717	0.89172	0.89672
444	0.86901	0.87775	0.88178	0.88310	0.88737	0.89192	0.89690
445	0.86926	0.87797	0.88200	0.88331	0.88757	0.89211	0.89708
446	0.86949	0.87819	0.88221	0.88353	0.88777	0.89230	0.89726
447	0.86973	0.87841	0.88242	0.88374	0.88797	0.89250	0.89745
448	0.86998	0.87863	0.88263	0.88394	0.88817	0.89269	0.89762
449	0.87021	0.87885	0.88284	0.88415	0.88837	0.89287	0.89781
450	0.87043	0.87907	0.88305	0.88435	0.88857	0.89307	0.89798
451	0.87065	0.87929	0.88326	0.88457	0.88877	0.89325	0.89816
452	0.87090	0.87951	0.88347	0.88477	0.88896	0.89344	0.89834
453	0.87113	0.87972	0.88367	0.88497	0.88916	0.89363	0.89852
454	0.87135	0.87994	0.88389	0.88518	0.88936	0.89382	0.89869
455	0.87158	0.88015	0.88409	0.88538	0.88955	0.89400	0.89887
456	0.87180	0.88036	0.88429	0.88558	0.88974	0.89419	0.89904
457	0.87202	0.88057	0.88449	0.88579	0.88994	0.89437	0.89922
458	0.87227	0.88079	0.88469	0.88598	0.89013	0.89455	0.89939
459	0.87249	0.88100	0.88489	0.88618	0.89032	0.89474	0.89956
460	0.87271	0.88121	0.88509	0.88638	0.89051	0.89492	0.89973
461	0.87295	0.88142	0.88530	0.88658	0.89070	0.89510	0.89991
462	0.87317	0.88163	0.88550	0.88678	0.89090	0.89528	0.90008
463	0.87339	0.88183	0.88569	0.88697	0.89108	0.89546	0.90025
464	0.87361	0.88205	0.88589	0.88716	0.89127	0.89564	0.90042
465	0.87380	0.88225	0.88609	0.88736	0.89145	0.89582	0.90059
466	0.87402	0.88246	0.88629	0.88755	0.89164	0.89600	0.90076
467	0.87424	0.88267	0.88649	0.88774	0.89183	0.89617	0.90093
468	0.87446	0.88287	0.88668	0.88793	0.89201	0.89635	0.90110
469	0.87468	0.88308	0.88687	0.88813	0.89219	0.89653	0.90126
470	0.87489	0.88327	0.88706	0.88833	0.89238	0.89670	0.90143
471	0.87511	0.88347	0.88725	0.88851	0.89256	0.89688	0.90160
472	0.87532	0.88366	0.88744	0.88869	0.89274	0.89705	0.90176
473	0.87556	0.88385	0.88764	0.88889	0.89293	0.89723	0.90193
474	0.87579	0.88405	0.88783	0.88908	0.89311	0.89740	0.90209
475	0.87598	0.88426	0.88802	0.88927	0.89329	0.89757	0.90225
476	0.87618	0.88445	0.88820	0.88945	0.89347	0.89774	0.90242
477	0.87641	0.88465	0.88840	0.88964	0.89365	0.89791	0.90258
478	0.87662	0.88485	0.88858	0.88982	0.89382	0.89808	0.90274
479	0.87684	0.88505	0.88877	0.89001	0.89400	0.89825	0.90290
480	0.87704	0.88524	0.88897	0.89019	0.89418	0.89842	0.90306
481	0.87726	0.88544	0.88915	0.89038	0.89436	0.89859	0.90322
482	0.87748	0.88562	0.88934	0.89056	0.89454	0.89876	0.90338
483	0.87768	0.88582	0.88952	0.89075	0.89471	0.89893	0.90354
484	0.87788	0.88601	0.88970	0.89093	0.89489	0.89909	0.90370
485	0.87808	0.88620	0.88988	0.89111	0.89506	0.89926	0.90385
486	0.87832	0.88639	0.89007	0.89129	0.89523	0.89943	0.90401
487	0.87852	0.88658	0.89025	0.89147	0.89541	0.89959	0.90417
488	0.87871	0.88677	0.89043	0.89165	0.89558	0.89976	0.90433
489	0.87889	0.88695	0.89061	0.89183	0.89574	0.89992	0.90448
490	0.87910	0.88714	0.89079	0.89201	0.89591	0.90008	0.90464
491	0.87930	0.88732	0.89097	0.89218	0.89609	0.90025	0.90479
492	0.87951	0.88751	0.89115	0.89236	0.89626	0.90041	0.90494
493	0.87971	0.88769	0.89133	0.89254	0.89643	0.90057	0.90510
494	0.87988	0.88788	0.89151	0.89271	0.89660	0.90073	0.90525
495	0.88008	0.88806	0.89169	0.89289	0.89677	0.90090	0.90540
496	0.88028	0.88824	0.89186	0.89306	0.89693	0.90105	0.90556
497	0.88047	0.88843	0.89204	0.89324	0.89710	0.90121	0.90571
498	0.88068	0.88860	0.89221	0.89341	0.89726	0.90137	0.90585
499	0.88086	0.88879	0.89239	0.89359	0.89743	0.90153	0.90601
500	0.88106	0.88897	0.89257	0.89376	0.89760	0.90169	0.90616
501	0.88124	0.88915	0.89274	0.89394	0.89776	0.90185	0.90631
502	0.88143	0.88933	0.89291	0.89411	0.89793	0.90200	0.90645
503	0.88164	0.88951	0.89308	0.89428	0.89809	0.90216	0.90660
504	0.88181	0.88969	0.89326	0.89445	0.89826	0.90231	0.90675
505	0.88201	0.88986	0.89343	0.89462	0.89842	0.90247	0.90690
506	0.88221	0.89005	0.89361	0.89479	0.89858	0.90263	0.90705
507	0.88239	0.89022	0.89377	0.89496	0.89874	0.90278	0.90719
508	0.88259	0.89039	0.89394	0.89513	0.89890	0.90293	0.90734

N	0.1%	0.5%	1%	1.25%	2.5%	5%	10%
509	0.88279	0.89056	0.89411	0.89529	0.89906	0.90308	0.90748
510	0.88296	0.89074	0.89428	0.89545	0.89922	0.90324	0.90763
511	0.88314	0.89092	0.89444	0.89562	0.89938	0.90339	0.90777
512	0.88333	0.89109	0.89461	0.89578	0.89954	0.90354	0.90791
513	0.88350	0.89126	0.89478	0.89595	0.89969	0.90369	0.90806
514	0.88368	0.89144	0.89495	0.89611	0.89985	0.90384	0.90820
515	0.88390	0.89161	0.89511	0.89627	0.90001	0.90399	0.90834
516	0.88406	0.89178	0.89528	0.89643	0.90016	0.90414	0.90848
517	0.88425	0.89195	0.89544	0.89660	0.90032	0.90429	0.90862
518	0.88444	0.89211	0.89561	0.89676	0.90048	0.90444	0.90877
519	0.88462	0.89228	0.89577	0.89692	0.90063	0.90458	0.90890
520	0.88481	0.89245	0.89593	0.89708	0.90079	0.90473	0.90905
521	0.88499	0.89262	0.89610	0.89724	0.90094	0.90488	0.90918
522	0.88518	0.89280	0.89626	0.89740	0.90109	0.90503	0.90932
523	0.88536	0.89297	0.89642	0.89756	0.90125	0.90517	0.90946
524	0.88553	0.89313	0.89659	0.89773	0.90140	0.90532	0.90960
525	0.88573	0.89329	0.89674	0.89788	0.90155	0.90546	0.90974
526	0.88589	0.89346	0.89690	0.89804	0.90170	0.90561	0.90988
527	0.88607	0.89362	0.89706	0.89819	0.90185	0.90575	0.91001
528	0.88624	0.89379	0.89722	0.89835	0.90200	0.90589	0.91015
529	0.88640	0.89396	0.89738	0.89851	0.90215	0.90604	0.91028
530	0.88659	0.89412	0.89754	0.89866	0.90230	0.90618	0.91042
531	0.88676	0.89428	0.89769	0.89882	0.90245	0.90632	0.91055
532	0.88696	0.89443	0.89785	0.89897	0.90260	0.90646	0.91068
533	0.88713	0.89460	0.89800	0.89912	0.90274	0.90660	0.91082
534	0.88729	0.89475	0.89816	0.89928	0.90289	0.90674	0.91095
535	0.88747	0.89492	0.89832	0.89943	0.90304	0.90688	0.91108
536	0.88766	0.89507	0.89847	0.89957	0.90318	0.90702	0.91122
537	0.88782	0.89523	0.89862	0.89972	0.90333	0.90716	0.91135
538	0.88799	0.89539	0.89878	0.89988	0.90347	0.90730	0.91148
539	0.88815	0.89555	0.89893	0.90003	0.90362	0.90744	0.91161
540	0.88832	0.89570	0.89908	0.90018	0.90376	0.90758	0.91174
541	0.88848	0.89586	0.89923	0.90033	0.90391	0.90771	0.91187
542	0.88865	0.89602	0.89939	0.90048	0.90405	0.90785	0.91200
543	0.88882	0.89617	0.89954	0.90062	0.90419	0.90799	0.91213
544	0.88899	0.89633	0.89968	0.90077	0.90434	0.90813	0.91226
545	0.88916	0.89648	0.89983	0.90092	0.90448	0.90826	0.91239
546	0.88932	0.89664	0.89998	0.90107	0.90462	0.90840	0.91252
547	0.88949	0.89679	0.90013	0.90122	0.90476	0.90853	0.91265
548	0.88966	0.89694	0.90028	0.90136	0.90490	0.90866	0.91278
549	0.88982	0.89709	0.90042	0.90151	0.90504	0.90880	0.91290
550	0.88999	0.89725	0.90057	0.90166	0.90518	0.90893	0.91303
551	0.89015	0.89741	0.90072	0.90180	0.90532	0.90907	0.91316
552	0.89031	0.89756	0.90087	0.90194	0.90546	0.90920	0.91328
553	0.89047	0.89771	0.90101	0.90209	0.90560	0.90933	0.91341
554	0.89064	0.89787	0.90116	0.90224	0.90574	0.90947	0.91353
555	0.89078	0.89801	0.90130	0.90238	0.90588	0.90959	0.91366
556	0.89095	0.89816	0.90145	0.90252	0.90601	0.90973	0.91378
557	0.89112	0.89832	0.90159	0.90266	0.90615	0.90986	0.91391
558	0.89130	0.89846	0.90173	0.90280	0.90629	0.90999	0.91403
559	0.89145	0.89861	0.90188	0.90294	0.90642	0.91012	0.91415
560	0.89161	0.89876	0.90202	0.90309	0.90656	0.91025	0.91428
561	0.89177	0.89891	0.90217	0.90323	0.90669	0.91037	0.91440
562	0.89192	0.89905	0.90231	0.90337	0.90683	0.91050	0.91452
563	0.89208	0.89920	0.90245	0.90351	0.90696	0.91063	0.91464
564	0.89224	0.89935	0.90259	0.90365	0.90709	0.91076	0.91476
565	0.89238	0.89950	0.90273	0.90379	0.90722	0.91088	0.91488
566	0.89253	0.89965	0.90288	0.90393	0.90736	0.91101	0.91500
567	0.89268	0.89979	0.90302	0.90406	0.90749	0.91114	0.91512
568	0.89285	0.89993	0.90315	0.90420	0.90762	0.91126	0.91524
569	0.89299	0.90007	0.90329	0.90434	0.90775	0.91139	0.91536
570	0.89315	0.90022	0.90343	0.90447	0.90789	0.91152	0.91548
571	0.89332	0.90036	0.90356	0.90460	0.90801	0.91164	0.91560
572	0.89349	0.90051	0.90370	0.90474	0.90814	0.91177	0.91572
573	0.89364	0.90065	0.90384	0.90488	0.90827	0.91189	0.91584
574	0.89378	0.90078	0.90398	0.90501	0.90841	0.91201	0.91595
575	0.89395	0.90092	0.90411	0.90515	0.90853	0.91214	0.91607
576	0.89409	0.90106	0.90424	0.90528	0.90867	0.91226	0.91619
577	0.89424	0.90121	0.90438	0.90542	0.90879	0.91238	0.91630
578	0.89440	0.90134	0.90451	0.90555	0.90892	0.91251	0.91642
579	0.89454	0.90148	0.90464	0.90568	0.90905	0.91263	0.91654
580	0.89469	0.90162	0.90477	0.90581	0.90918	0.91275	0.91665
581	0.89484	0.90175	0.90491	0.90595	0.90930	0.91287	0.91677
582	0.89499	0.90190	0.90504	0.90608	0.90943	0.91299	0.91688

N	0.1%	0.5%	1%	1.25%	2.5%	5%	10%
583	0.89514	0.90204	0.90517	0.90621	0.90956	0.91311	0.91700
584	0.89529	0.90218	0.90531	0.90634	0.90968	0.91323	0.91711
585	0.89544	0.90231	0.90544	0.90647	0.90981	0.91335	0.91722
586	0.89557	0.90245	0.90557	0.90660	0.90993	0.91347	0.91734
587	0.89573	0.90259	0.90570	0.90673	0.91006	0.91359	0.91745
588	0.89587	0.90273	0.90583	0.90687	0.91018	0.91371	0.91756
589	0.89603	0.90286	0.90596	0.90699	0.91031	0.91382	0.91768
590	0.89616	0.90300	0.90609	0.90712	0.91043	0.91394	0.91779
591	0.89631	0.90314	0.90623	0.90725	0.91055	0.91406	0.91790
592	0.89645	0.90327	0.90635	0.90738	0.91068	0.91418	0.91801
593	0.89660	0.90340	0.90648	0.90751	0.91080	0.91430	0.91812
594	0.89676	0.90354	0.90661	0.90763	0.91092	0.91441	0.91823
595	0.89690	0.90367	0.90674	0.90776	0.91104	0.91453	0.91834
596	0.89703	0.90379	0.90687	0.90789	0.91116	0.91464	0.91845
597	0.89717	0.90393	0.90699	0.90801	0.91128	0.91476	0.91856
598	0.89732	0.90406	0.90712	0.90814	0.91140	0.91488	0.91867
599	0.89746	0.90419	0.90724	0.90826	0.91152	0.91499	0.91878
600	0.89759	0.90432	0.90737	0.90838	0.91164	0.91511	0.91889
601	0.89773	0.90445	0.90749	0.90851	0.91176	0.91522	0.91900
602	0.89787	0.90458	0.90762	0.90864	0.91188	0.91534	0.91910
603	0.89801	0.90471	0.90774	0.90876	0.91200	0.91545	0.91921
604	0.89815	0.90483	0.90787	0.90888	0.91211	0.91556	0.91932
605	0.89829	0.90496	0.90799	0.90900	0.91223	0.91567	0.91943
606	0.89841	0.90510	0.90811	0.90912	0.91235	0.91579	0.91953
607	0.89855	0.90522	0.90824	0.90925	0.91247	0.91590	0.91964
608	0.89870	0.90534	0.90836	0.90937	0.91258	0.91601	0.91975
609	0.89883	0.90547	0.90848	0.90949	0.91270	0.91612	0.91985
610	0.89897	0.90561	0.90861	0.90961	0.91281	0.91623	0.91996
611	0.89910	0.90573	0.90873	0.90973	0.91293	0.91634	0.92006
612	0.89925	0.90586	0.90885	0.90985	0.91305	0.91645	0.92017
613	0.89937	0.90598	0.90897	0.90997	0.91316	0.91657	0.92028
614	0.89951	0.90610	0.90910	0.91009	0.91328	0.91668	0.92038
615	0.89965	0.90623	0.90921	0.91021	0.91339	0.91679	0.92048
616	0.89978	0.90636	0.90933	0.91033	0.91351	0.91689	0.92059
617	0.89993	0.90648	0.90945	0.91045	0.91362	0.91700	0.92069
618	0.90006	0.90660	0.90958	0.91057	0.91374	0.91711	0.92079
619	0.90019	0.90672	0.90970	0.91068	0.91385	0.91722	0.92090
620	0.90031	0.90684	0.90982	0.91080	0.91396	0.91733	0.92100
621	0.90043	0.90697	0.90994	0.91092	0.91408	0.91744	0.92110
622	0.90057	0.90709	0.91005	0.91104	0.91419	0.91755	0.92121
623	0.90071	0.90721	0.91017	0.91115	0.91430	0.91766	0.92131
624	0.90083	0.90733	0.91029	0.91127	0.91441	0.91776	0.92141
625	0.90097	0.90745	0.91040	0.91138	0.91452	0.91787	0.92151
626	0.90111	0.90757	0.91052	0.91150	0.91464	0.91797	0.92161
627	0.90124	0.90769	0.91064	0.91161	0.91475	0.91808	0.92171
628	0.90137	0.90781	0.91076	0.91173	0.91486	0.91819	0.92181
629	0.90150	0.90794	0.91087	0.91185	0.91497	0.91829	0.92191
630	0.90163	0.90805	0.91098	0.91196	0.91508	0.91840	0.92201
631	0.90176	0.90817	0.91110	0.91207	0.91519	0.91850	0.92211
632	0.90187	0.90829	0.91121	0.91219	0.91530	0.91861	0.92221
633	0.90200	0.90841	0.91133	0.91230	0.91541	0.91871	0.92231
634	0.90214	0.90853	0.91144	0.91241	0.91552	0.91881	0.92241
635	0.90227	0.90865	0.91156	0.91252	0.91562	0.91892	0.92251
636	0.90240	0.90877	0.91167	0.91263	0.91573	0.91902	0.92261
637	0.90253	0.90889	0.91179	0.91274	0.91584	0.91912	0.92270
638	0.90265	0.90900	0.91190	0.91286	0.91595	0.91922	0.92280
639	0.90278	0.90912	0.91201	0.91297	0.91606	0.91933	0.92290
640	0.90291	0.90923	0.91212	0.91308	0.91616	0.91943	0.92300
641	0.90303	0.90934	0.91223	0.91319	0.91627	0.91953	0.92310
642	0.90316	0.90946	0.91235	0.91330	0.91637	0.91963	0.92319
643	0.90328	0.90957	0.91246	0.91341	0.91648	0.91973	0.92329
644	0.90342	0.90969	0.91257	0.91352	0.91658	0.91984	0.92338
645	0.90354	0.90980	0.91268	0.91363	0.91669	0.91994	0.92348
646	0.90366	0.90991	0.91279	0.91374	0.91679	0.92004	0.92357
647	0.90379	0.91002	0.91290	0.91384	0.91690	0.92014	0.92367
648	0.90391	0.91014	0.91301	0.91395	0.91700	0.92024	0.92376
649	0.90403	0.91026	0.91312	0.91406	0.91711	0.92034	0.92386
650	0.90416	0.91037	0.91323	0.91417	0.91721	0.92044	0.92395
651	0.90429	0.91049	0.91333	0.91428	0.91732	0.92053	0.92405
652	0.90441	0.91060	0.91344	0.91439	0.91742	0.92063	0.92414
653	0.90454	0.91071	0.91356	0.91449	0.91752	0.92073	0.92423
654	0.90465	0.91083	0.91366	0.91460	0.91763	0.92083	0.92433
655	0.90477	0.91093	0.91377	0.91471	0.91773	0.92093	0.92442
656	0.90487	0.91105	0.91388	0.91481	0.91783	0.92103	0.92451

N	0.1%	0.5%	1%	1.25%	2.5%	5%	10%
657	0.90500	0.91116	0.91399	0.91492	0.91793	0.92113	0.92461
658	0.90513	0.91127	0.91410	0.91502	0.91804	0.92122	0.92470
659	0.90524	0.91138	0.91420	0.91513	0.91814	0.92132	0.92479
660	0.90537	0.91149	0.91431	0.91523	0.91823	0.92142	0.92488
661	0.90548	0.91160	0.91441	0.91534	0.91833	0.92152	0.92497
662	0.90560	0.91171	0.91452	0.91544	0.91844	0.92161	0.92507
663	0.90572	0.91182	0.91462	0.91555	0.91854	0.92171	0.92516
664	0.90582	0.91193	0.91473	0.91565	0.91864	0.92180	0.92525
665	0.90594	0.91204	0.91483	0.91576	0.91874	0.92190	0.92534
666	0.90605	0.91214	0.91494	0.91586	0.91884	0.92200	0.92543
667	0.90618	0.91225	0.91505	0.91596	0.91894	0.92209	0.92552
668	0.90628	0.91236	0.91515	0.91606	0.91903	0.92218	0.92561
669	0.90639	0.91247	0.91525	0.91616	0.91913	0.92228	0.92570
670	0.90652	0.91257	0.91536	0.91627	0.91923	0.92237	0.92579
671	0.90665	0.91269	0.91546	0.91637	0.91933	0.92247	0.92588
672	0.90677	0.91279	0.91556	0.91648	0.91943	0.92256	0.92597
673	0.90688	0.91290	0.91566	0.91658	0.91953	0.92265	0.92606
674	0.90699	0.91301	0.91576	0.91668	0.91962	0.92275	0.92615
675	0.90710	0.91312	0.91587	0.91678	0.91972	0.92284	0.92623
676	0.90721	0.91322	0.91597	0.91688	0.91982	0.92293	0.92632
677	0.90733	0.91333	0.91607	0.91698	0.91992	0.92302	0.92641
678	0.90745	0.91343	0.91618	0.91709	0.92001	0.92312	0.92650
679	0.90756	0.91354	0.91628	0.91719	0.92011	0.92321	0.92659
680	0.90768	0.91364	0.91638	0.91729	0.92020	0.92330	0.92667
681	0.90779	0.91375	0.91649	0.91739	0.92030	0.92339	0.92676
682	0.90791	0.91385	0.91658	0.91748	0.92040	0.92349	0.92684
683	0.90802	0.91396	0.91668	0.91759	0.92049	0.92358	0.92693
684	0.90813	0.91406	0.91678	0.91769	0.92059	0.92367	0.92702
685	0.90824	0.91417	0.91688	0.91778	0.92068	0.92376	0.92710
686	0.90836	0.91427	0.91698	0.91788	0.92077	0.92385	0.92719
687	0.90846	0.91437	0.91708	0.91798	0.92087	0.92394	0.92728
688	0.90857	0.91448	0.91718	0.91808	0.92096	0.92403	0.92736
689	0.90868	0.91458	0.91729	0.91818	0.92106	0.92412	0.92745
690	0.90878	0.91468	0.91738	0.91827	0.92115	0.92421	0.92753
691	0.90889	0.91478	0.91748	0.91837	0.92125	0.92430	0.92761
692	0.90899	0.91488	0.91758	0.91846	0.92134	0.92438	0.92770
693	0.90911	0.91498	0.91768	0.91856	0.92143	0.92447	0.92778
694	0.90922	0.91509	0.91777	0.91866	0.92152	0.92456	0.92787
695	0.90932	0.91519	0.91787	0.91876	0.92162	0.92465	0.92795
696	0.90943	0.91529	0.91796	0.91886	0.92171	0.92474	0.92804
697	0.90953	0.91539	0.91806	0.91895	0.92180	0.92482	0.92812
698	0.90965	0.91549	0.91816	0.91904	0.92189	0.92491	0.92820
699	0.90976	0.91559	0.91825	0.91914	0.92198	0.92500	0.92829
700	0.90986	0.91569	0.91835	0.91924	0.92207	0.92509	0.92837
701	0.90997	0.91579	0.91845	0.91933	0.92216	0.92517	0.92845
702	0.91007	0.91589	0.91855	0.91942	0.92225	0.92526	0.92853
703	0.91018	0.91599	0.91864	0.91952	0.92235	0.92535	0.92862
704	0.91029	0.91609	0.91874	0.91962	0.92244	0.92543	0.92870
705	0.91039	0.91618	0.91883	0.91971	0.92252	0.92552	0.92878
706	0.91049	0.91628	0.91892	0.91980	0.92261	0.92560	0.92886
707	0.91060	0.91637	0.91902	0.91989	0.92270	0.92569	0.92894
708	0.91071	0.91647	0.91911	0.91999	0.92279	0.92578	0.92903
709	0.91081	0.91657	0.91921	0.92008	0.92288	0.92586	0.92911
710	0.91092	0.91667	0.91930	0.92018	0.92297	0.92594	0.92919
711	0.91102	0.91676	0.91939	0.92027	0.92306	0.92603	0.92927
712	0.91113	0.91686	0.91948	0.92036	0.92315	0.92611	0.92935
713	0.91122	0.91695	0.91958	0.92045	0.92324	0.92620	0.92943
714	0.91133	0.91705	0.91967	0.92054	0.92332	0.92628	0.92951
715	0.91143	0.91714	0.91977	0.92064	0.92341	0.92637	0.92959
716	0.91154	0.91724	0.91986	0.92073	0.92350	0.92645	0.92967
717	0.91164	0.91734	0.91995	0.92082	0.92359	0.92653	0.92975
718	0.91175	0.91743	0.92004	0.92091	0.92367	0.92662	0.92983
719	0.91184	0.91753	0.92013	0.92100	0.92376	0.92670	0.92991
720	0.91195	0.91763	0.92023	0.92109	0.92385	0.92678	0.92998
721	0.91205	0.91773	0.92032	0.92118	0.92393	0.92687	0.93006
722	0.91216	0.91782	0.92041	0.92127	0.92402	0.92695	0.93014
723	0.91225	0.91791	0.92050	0.92135	0.92411	0.92703	0.93022
724	0.91235	0.91800	0.92059	0.92145	0.92419	0.92711	0.93030
725	0.91245	0.91810	0.92068	0.92153	0.92427	0.92719	0.93037
726	0.91255	0.91819	0.92077	0.92163	0.92436	0.92728	0.93045
727	0.91265	0.91828	0.92086	0.92171	0.92444	0.92736	0.93053
728	0.91275	0.91837	0.92095	0.92180	0.92453	0.92744	0.93061
729	0.91286	0.91847	0.92104	0.92189	0.92461	0.92752	0.93068
730	0.91296	0.91856	0.92113	0.92198	0.92470	0.92760	0.93076

N	0.1%	0.5%	1%	1.25%	2.5%	5%	10%
731	0.91306	0.91866	0.92122	0.92206	0.92478	0.92768	0.93084
732	0.91317	0.91875	0.92131	0.92215	0.92487	0.92776	0.93092
733	0.91326	0.91884	0.92140	0.92224	0.92495	0.92784	0.93099
734	0.91336	0.91893	0.92149	0.92233	0.92504	0.92792	0.93107
735	0.91346	0.91902	0.92157	0.92241	0.92512	0.92800	0.93115
736	0.91356	0.91912	0.92166	0.92250	0.92520	0.92808	0.93122
737	0.91366	0.91921	0.92175	0.92259	0.92529	0.92816	0.93130
738	0.91376	0.91929	0.92184	0.92267	0.92537	0.92824	0.93137
739	0.91387	0.91939	0.92192	0.92276	0.92545	0.92832	0.93145
740	0.91396	0.91948	0.92201	0.92285	0.92554	0.92840	0.93152
741	0.91406	0.91957	0.92210	0.92293	0.92562	0.92848	0.93160
742	0.91416	0.91965	0.92219	0.92302	0.92570	0.92856	0.93167
743	0.91425	0.91974	0.92227	0.92310	0.92578	0.92864	0.93175
744	0.91435	0.91983	0.92235	0.92318	0.92586	0.92871	0.93182
745	0.91444	0.91991	0.92244	0.92327	0.92595	0.92879	0.93189
746	0.91452	0.92001	0.92252	0.92335	0.92603	0.92887	0.93197
747	0.91463	0.92010	0.92261	0.92344	0.92611	0.92895	0.93204
748	0.91471	0.92018	0.92270	0.92353	0.92619	0.92902	0.93212
749	0.91481	0.92027	0.92278	0.92361	0.92627	0.92910	0.93219
750	0.91491	0.92036	0.92287	0.92370	0.92635	0.92918	0.93226
751	0.91500	0.92045	0.92296	0.92378	0.92643	0.92925	0.93234
752	0.91509	0.92054	0.92304	0.92386	0.92651	0.92933	0.93241
753	0.91518	0.92062	0.92312	0.92395	0.92659	0.92941	0.93248
754	0.91527	0.92071	0.92321	0.92403	0.92667	0.92949	0.93255
755	0.91537	0.92080	0.92329	0.92411	0.92675	0.92956	0.93263
756	0.91546	0.92089	0.92337	0.92420	0.92683	0.92964	0.93270
757	0.91555	0.92098	0.92346	0.92428	0.92691	0.92971	0.93277
758	0.91563	0.92105	0.92354	0.92437	0.92699	0.92979	0.93284
759	0.91573	0.92114	0.92363	0.92445	0.92707	0.92987	0.93291
760	0.91583	0.92123	0.92371	0.92453	0.92715	0.92994	0.93299
761	0.91593	0.92132	0.92379	0.92461	0.92723	0.93002	0.93306
762	0.91601	0.92140	0.92388	0.92469	0.92731	0.93009	0.93313
763	0.91611	0.92149	0.92396	0.92477	0.92738	0.93017	0.93320
764	0.91619	0.92157	0.92404	0.92485	0.92746	0.93024	0.93327
765	0.91628	0.92166	0.92412	0.92494	0.92754	0.93032	0.93334
766	0.91637	0.92174	0.92420	0.92502	0.92762	0.93039	0.93341
767	0.91646	0.92182	0.92428	0.92510	0.92769	0.93046	0.93348
768	0.91655	0.92191	0.92437	0.92518	0.92777	0.93054	0.93355
769	0.91665	0.92199	0.92445	0.92526	0.92785	0.93061	0.93362
770	0.91673	0.92208	0.92453	0.92534	0.92793	0.93069	0.93370
771	0.91682	0.92216	0.92460	0.92542	0.92800	0.93076	0.93377
772	0.91691	0.92225	0.92468	0.92550	0.92808	0.93083	0.93384
773	0.91699	0.92233	0.92477	0.92557	0.92816	0.93090	0.93390
774	0.91710	0.92241	0.92485	0.92565	0.92823	0.93098	0.93397
775	0.91719	0.92250	0.92493	0.92573	0.92831	0.93105	0.93404
776	0.91729	0.92258	0.92500	0.92581	0.92838	0.93112	0.93411
777	0.91737	0.92266	0.92508	0.92589	0.92846	0.93119	0.93418
778	0.91746	0.92275	0.92516	0.92597	0.92853	0.93127	0.93425
779	0.91756	0.92283	0.92524	0.92605	0.92861	0.93134	0.93432
780	0.91765	0.92291	0.92532	0.92613	0.92868	0.93141	0.93438
781	0.91775	0.92300	0.92540	0.92620	0.92876	0.93148	0.93445
782	0.91784	0.92308	0.92548	0.92628	0.92883	0.93156	0.93452
783	0.91792	0.92317	0.92555	0.92636	0.92891	0.93163	0.93459
784	0.91801	0.92325	0.92563	0.92643	0.92898	0.93170	0.93466
785	0.91809	0.92333	0.92571	0.92651	0.92905	0.93177	0.93472
786	0.91818	0.92341	0.92579	0.92659	0.92913	0.93184	0.93479
787	0.91827	0.92349	0.92587	0.92667	0.92920	0.93191	0.93486
788	0.91835	0.92357	0.92595	0.92674	0.92928	0.93198	0.93493
789	0.91844	0.92365	0.92602	0.92682	0.92935	0.93205	0.93499
790	0.91852	0.92373	0.92610	0.92690	0.92943	0.93212	0.93506
791	0.91860	0.92381	0.92618	0.92697	0.92950	0.93219	0.93513
792	0.91868	0.92389	0.92626	0.92705	0.92958	0.93226	0.93519
793	0.91877	0.92397	0.92634	0.92712	0.92965	0.93233	0.93526
794	0.91886	0.92405	0.92641	0.92720	0.92972	0.93241	0.93533
795	0.91894	0.92413	0.92649	0.92728	0.92979	0.93247	0.93539
796	0.91903	0.92421	0.92657	0.92735	0.92987	0.93254	0.93546
797	0.91911	0.92429	0.92664	0.92742	0.92994	0.93261	0.93553
798	0.91920	0.92437	0.92672	0.92750	0.93001	0.93268	0.93559
799	0.91929	0.92444	0.92679	0.92757	0.93008	0.93275	0.93566
800	0.91937	0.92452	0.92687	0.92765	0.93015	0.93282	0.93572
801	0.91945	0.92460	0.92694	0.92773	0.93022	0.93289	0.93579
802	0.91953	0.92468	0.92702	0.92780	0.93030	0.93296	0.93585
803	0.91962	0.92475	0.92710	0.92787	0.93037	0.93303	0.93592
804	0.91970	0.92483	0.92717	0.92795	0.93044	0.93310	0.93598

N	0.1%	0.5%	1%	1.25%	2.5%	5%	10%
805	0.91979	0.92490	0.92725	0.92802	0.93051	0.93316	0.93605
806	0.91987	0.92498	0.92732	0.92809	0.93058	0.93323	0.93611
807	0.91995	0.92506	0.92740	0.92817	0.93065	0.93330	0.93618
808	0.92003	0.92514	0.92747	0.92824	0.93072	0.93337	0.93624
809	0.92011	0.92522	0.92754	0.92831	0.93079	0.93343	0.93631
810	0.92019	0.92529	0.92762	0.92839	0.93086	0.93350	0.93637
811	0.92027	0.92537	0.92769	0.92846	0.93093	0.93357	0.93643
812	0.92036	0.92544	0.92777	0.92853	0.93100	0.93363	0.93650
813	0.92044	0.92552	0.92784	0.92860	0.93107	0.93370	0.93656
814	0.92052	0.92559	0.92791	0.92868	0.93114	0.93377	0.93662
815	0.92061	0.92567	0.92798	0.92875	0.93121	0.93384	0.93669
816	0.92069	0.92574	0.92806	0.92882	0.93128	0.93390	0.93675
817	0.92077	0.92582	0.92814	0.92890	0.93135	0.93397	0.93681
818	0.92086	0.92590	0.92821	0.92897	0.93142	0.93403	0.93687
819	0.92094	0.92597	0.92828	0.92904	0.93149	0.93410	0.93694
820	0.92101	0.92605	0.92835	0.92911	0.93155	0.93417	0.93700
821	0.92110	0.92612	0.92842	0.92918	0.93162	0.93423	0.93706
822	0.92117	0.92620	0.92849	0.92925	0.93169	0.93430	0.93712
823	0.92125	0.92627	0.92856	0.92932	0.93176	0.93436	0.93719
824	0.92134	0.92635	0.92864	0.92939	0.93183	0.93443	0.93725
825	0.92142	0.92642	0.92871	0.92947	0.93190	0.93449	0.93731
826	0.92151	0.92650	0.92878	0.92954	0.93197	0.93456	0.93737
827	0.92158	0.92657	0.92885	0.92961	0.93203	0.93462	0.93743
828	0.92166	0.92664	0.92892	0.92968	0.93210	0.93469	0.93750
829	0.92175	0.92672	0.92899	0.92975	0.93217	0.93475	0.93756
830	0.92182	0.92679	0.92906	0.92982	0.93223	0.93482	0.93762
831	0.92190	0.92686	0.92913	0.92989	0.93230	0.93488	0.93768
832	0.92198	0.92694	0.92920	0.92996	0.93236	0.93495	0.93774
833	0.92205	0.92701	0.92927	0.93003	0.93243	0.93501	0.93780
834	0.92213	0.92708	0.92934	0.93009	0.93250	0.93507	0.93786
835	0.92221	0.92715	0.92941	0.93017	0.93257	0.93514	0.93792
836	0.92229	0.92722	0.92948	0.93023	0.93263	0.93520	0.93798
837	0.92237	0.92730	0.92955	0.93030	0.93270	0.93527	0.93805
838	0.92245	0.92737	0.92962	0.93037	0.93277	0.93533	0.93811
839	0.92253	0.92744	0.92969	0.93044	0.93283	0.93539	0.93817
840	0.92261	0.92751	0.92976	0.93051	0.93290	0.93545	0.93823
841	0.92269	0.92758	0.92983	0.93058	0.93297	0.93552	0.93829
842	0.92277	0.92766	0.92990	0.93065	0.93303	0.93558	0.93835
843	0.92284	0.92773	0.92997	0.93072	0.93310	0.93564	0.93841
844	0.92292	0.92780	0.93004	0.93078	0.93316	0.93570	0.93847
845	0.92300	0.92787	0.93011	0.93085	0.93323	0.93577	0.93853
846	0.92308	0.92794	0.93018	0.93092	0.93329	0.93583	0.93859
847	0.92316	0.92802	0.93025	0.93098	0.93336	0.93589	0.93865
848	0.92322	0.92808	0.93031	0.93105	0.93342	0.93595	0.93870
849	0.92330	0.92816	0.93038	0.93111	0.93349	0.93601	0.93876
850	0.92337	0.92823	0.93045	0.93118	0.93355	0.93607	0.93882
851	0.92345	0.92830	0.93052	0.93125	0.93361	0.93614	0.93888
852	0.92352	0.92837	0.93059	0.93132	0.93368	0.93620	0.93894
853	0.92360	0.92844	0.93065	0.93138	0.93374	0.93626	0.93900
854	0.92368	0.92851	0.93072	0.93145	0.93381	0.93632	0.93906
855	0.92375	0.92857	0.93078	0.93152	0.93387	0.93638	0.93911
856	0.92383	0.92864	0.93085	0.93158	0.93394	0.93644	0.93917
857	0.92390	0.92872	0.93092	0.93165	0.93400	0.93650	0.93923
858	0.92398	0.92879	0.93099	0.93171	0.93406	0.93657	0.93929
859	0.92405	0.92886	0.93106	0.93178	0.93412	0.93663	0.93935
860	0.92412	0.92893	0.93112	0.93184	0.93419	0.93669	0.93940
861	0.92419	0.92899	0.93119	0.93191	0.93425	0.93675	0.93946
862	0.92427	0.92906	0.93126	0.93197	0.93432	0.93681	0.93952
863	0.92434	0.92913	0.93132	0.93204	0.93438	0.93687	0.93958
864	0.92441	0.92920	0.93139	0.93211	0.93444	0.93693	0.93963
865	0.92448	0.92927	0.93145	0.93217	0.93451	0.93699	0.93969
866	0.92455	0.92934	0.93152	0.93224	0.93457	0.93705	0.93975
867	0.92463	0.92941	0.93159	0.93230	0.93463	0.93711	0.93980
868	0.92471	0.92947	0.93165	0.93237	0.93469	0.93717	0.93986
869	0.92478	0.92954	0.93172	0.93243	0.93476	0.93723	0.93992
870	0.92484	0.92961	0.93178	0.93250	0.93482	0.93729	0.93997
871	0.92492	0.92968	0.93185	0.93256	0.93488	0.93734	0.94003
872	0.92499	0.92974	0.93191	0.93263	0.93494	0.93740	0.94009
873	0.92506	0.92981	0.93198	0.93269	0.93500	0.93746	0.94014
874	0.92514	0.92987	0.93204	0.93275	0.93507	0.93752	0.94020
875	0.92521	0.92994	0.93211	0.93282	0.93512	0.93758	0.94026
876	0.92527	0.93001	0.93217	0.93288	0.93519	0.93764	0.94031
877	0.92534	0.93007	0.93223	0.93294	0.93525	0.93770	0.94037
878	0.92542	0.93014	0.93230	0.93301	0.93531	0.93775	0.94042

N	0.1%	0.5%	1%	1.25%	2.5%	5%	10%
879	0.92549	0.93021	0.93236	0.93307	0.93537	0.93781	0.94048
880	0.92556	0.93027	0.93243	0.93313	0.93543	0.93787	0.94053
881	0.92563	0.93034	0.93249	0.93319	0.93549	0.93793	0.94059
882	0.92570	0.93041	0.93255	0.93326	0.93555	0.93799	0.94064
883	0.92577	0.93047	0.93262	0.93332	0.93561	0.93804	0.94070
884	0.92584	0.93053	0.93268	0.93338	0.93567	0.93810	0.94075
885	0.92590	0.93060	0.93274	0.93344	0.93573	0.93816	0.94081
886	0.92598	0.93066	0.93281	0.93350	0.93579	0.93822	0.94086
887	0.92605	0.93073	0.93287	0.93357	0.93585	0.93828	0.94092
888	0.92612	0.93079	0.93293	0.93363	0.93591	0.93833	0.94097
889	0.92619	0.93086	0.93300	0.93369	0.93597	0.93839	0.94103
890	0.92627	0.93092	0.93306	0.93375	0.93603	0.93845	0.94108
891	0.92633	0.93099	0.93312	0.93382	0.93609	0.93850	0.94114
892	0.92640	0.93105	0.93318	0.93388	0.93615	0.93856	0.94119
893	0.92648	0.93112	0.93324	0.93394	0.93621	0.93861	0.94124
894	0.92655	0.93118	0.93330	0.93400	0.93627	0.93867	0.94130
895	0.92661	0.93125	0.93337	0.93406	0.93633	0.93873	0.94135
896	0.92668	0.93131	0.93343	0.93412	0.93639	0.93879	0.94140
897	0.92675	0.93137	0.93349	0.93419	0.93644	0.93884	0.94146
898	0.92682	0.93144	0.93356	0.93425	0.93650	0.93890	0.94151
899	0.92689	0.93150	0.93362	0.93431	0.93656	0.93895	0.94156
900	0.92695	0.93157	0.93368	0.93437	0.93662	0.93901	0.94162
901	0.92702	0.93163	0.93374	0.93443	0.93668	0.93907	0.94167
902	0.92708	0.93170	0.93380	0.93449	0.93673	0.93912	0.94172
903	0.92714	0.93176	0.93386	0.93455	0.93679	0.93918	0.94177
904	0.92721	0.93182	0.93392	0.93461	0.93685	0.93923	0.94183
905	0.92729	0.93189	0.93398	0.93467	0.93691	0.93929	0.94188
906	0.92735	0.93195	0.93404	0.93473	0.93697	0.93934	0.94193
907	0.92742	0.93201	0.93410	0.93479	0.93702	0.93940	0.94198
908	0.92748	0.93207	0.93416	0.93485	0.93708	0.93945	0.94204
909	0.92755	0.93213	0.93423	0.93491	0.93714	0.93951	0.94209
910	0.92762	0.93219	0.93429	0.93497	0.93720	0.93956	0.94214
911	0.92769	0.93226	0.93434	0.93503	0.93725	0.93962	0.94219
912	0.92775	0.93232	0.93441	0.93509	0.93731	0.93967	0.94224
913	0.92782	0.93239	0.93446	0.93515	0.93737	0.93973	0.94230
914	0.92788	0.93244	0.93452	0.93521	0.93742	0.93978	0.94235
915	0.92795	0.93251	0.93458	0.93527	0.93748	0.93983	0.94240
916	0.92802	0.93257	0.93464	0.93533	0.93754	0.93989	0.94245
917	0.92808	0.93263	0.93470	0.93538	0.93759	0.93994	0.94250
918	0.92814	0.93270	0.93476	0.93544	0.93765	0.93999	0.94255
919	0.92822	0.93276	0.93482	0.93550	0.93770	0.94005	0.94261
920	0.92828	0.93282	0.93488	0.93556	0.93776	0.94010	0.94266
921	0.92834	0.93288	0.93494	0.93562	0.93782	0.94016	0.94271
922	0.92841	0.93294	0.93500	0.93568	0.93787	0.94021	0.94276
923	0.92848	0.93300	0.93506	0.93574	0.93793	0.94026	0.94281
924	0.92855	0.93306	0.93511	0.93579	0.93798	0.94032	0.94286
925	0.92861	0.93312	0.93517	0.93585	0.93804	0.94037	0.94291
926	0.92868	0.93318	0.93523	0.93591	0.93809	0.94042	0.94296
927	0.92874	0.93324	0.93529	0.93596	0.93815	0.94048	0.94301
928	0.92880	0.93330	0.93534	0.93602	0.93820	0.94053	0.94306
929	0.92887	0.93336	0.93540	0.93608	0.93826	0.94058	0.94311
930	0.92893	0.93342	0.93546	0.93613	0.93831	0.94063	0.94316
931	0.92899	0.93348	0.93552	0.93619	0.93837	0.94069	0.94321
932	0.92906	0.93354	0.93558	0.93625	0.93842	0.94074	0.94326
933	0.92913	0.93360	0.93563	0.93630	0.93848	0.94079	0.94331
934	0.92919	0.93366	0.93569	0.93636	0.93853	0.94084	0.94336
935	0.92926	0.93372	0.93575	0.93642	0.93859	0.94089	0.94341
936	0.92932	0.93378	0.93581	0.93647	0.93864	0.94095	0.94346
937	0.92939	0.93384	0.93586	0.93653	0.93869	0.94100	0.94351
938	0.92945	0.93390	0.93592	0.93659	0.93875	0.94105	0.94356
939	0.92952	0.93395	0.93598	0.93664	0.93880	0.94110	0.94361
940	0.92957	0.93401	0.93604	0.93670	0.93886	0.94116	0.94366
941	0.92964	0.93407	0.93609	0.93676	0.93891	0.94121	0.94371
942	0.92971	0.93413	0.93615	0.93681	0.93896	0.94126	0.94376
943	0.92977	0.93419	0.93621	0.93687	0.93902	0.94131	0.94380
944	0.92982	0.93425	0.93626	0.93693	0.93907	0.94136	0.94385
945	0.92989	0.93431	0.93632	0.93698	0.93913	0.94141	0.94390
946	0.92995	0.93437	0.93638	0.93703	0.93918	0.94146	0.94395
947	0.93001	0.93442	0.93643	0.93709	0.93923	0.94152	0.94400
948	0.93008	0.93448	0.93649	0.93715	0.93929	0.94157	0.94405
949	0.93014	0.93454	0.93654	0.93720	0.93934	0.94162	0.94410
950	0.93020	0.93460	0.93660	0.93726	0.93939	0.94167	0.94415
951	0.93026	0.93466	0.93665	0.93731	0.93945	0.94172	0.94420
952	0.93031	0.93471	0.93671	0.93736	0.93950	0.94177	0.94424

N	0.1%	0.5%	1%	1.25%	2.5%	5%	10%
953	0.93038	0.93477	0.93676	0.93742	0.93955	0.94182	0.94429
954	0.93044	0.93483	0.93682	0.93748	0.93960	0.94187	0.94434
955	0.93051	0.93489	0.93688	0.93753	0.93966	0.94192	0.94439
956	0.93056	0.93494	0.93693	0.93759	0.93971	0.94197	0.94443
957	0.93062	0.93500	0.93698	0.93764	0.93976	0.94202	0.94448
958	0.93067	0.93505	0.93704	0.93770	0.93981	0.94207	0.94453
959	0.93074	0.93511	0.93709	0.93775	0.93986	0.94212	0.94458
960	0.93080	0.93517	0.93715	0.93781	0.93992	0.94217	0.94462
961	0.93086	0.93523	0.93720	0.93786	0.93997	0.94222	0.94467
962	0.93093	0.93528	0.93726	0.93791	0.94002	0.94227	0.94472
963	0.93099	0.93534	0.93731	0.93797	0.94007	0.94232	0.94476
964	0.93105	0.93540	0.93736	0.93802	0.94012	0.94237	0.94481
965	0.93111	0.93546	0.93742	0.93807	0.94017	0.94241	0.94486
966	0.93117	0.93551	0.93747	0.93813	0.94022	0.94246	0.94491
967	0.93123	0.93557	0.93753	0.93818	0.94028	0.94251	0.94495
968	0.93130	0.93562	0.93758	0.93823	0.94033	0.94256	0.94500
969	0.93136	0.93568	0.93763	0.93828	0.94038	0.94261	0.94504
970	0.93142	0.93573	0.93769	0.93834	0.94043	0.94266	0.94509
971	0.93148	0.93579	0.93774	0.93839	0.94048	0.94271	0.94514
972	0.93153	0.93585	0.93780	0.93844	0.94053	0.94276	0.94518
973	0.93160	0.93590	0.93785	0.93850	0.94058	0.94280	0.94523
974	0.93165	0.93596	0.93790	0.93855	0.94063	0.94285	0.94528
975	0.93171	0.93601	0.93796	0.93860	0.94068	0.94290	0.94532
976	0.93177	0.93607	0.93801	0.93865	0.94073	0.94295	0.94537
977	0.93184	0.93613	0.93807	0.93871	0.94078	0.94300	0.94541
978	0.93190	0.93618	0.93812	0.93876	0.94083	0.94305	0.94546
979	0.93195	0.93623	0.93817	0.93881	0.94088	0.94309	0.94551
980	0.93200	0.93629	0.93822	0.93886	0.94093	0.94314	0.94555
981	0.93206	0.93635	0.93828	0.93891	0.94098	0.94319	0.94560
982	0.93212	0.93640	0.93833	0.93896	0.94103	0.94324	0.94564
983	0.93218	0.93646	0.93838	0.93902	0.94108	0.94329	0.94569
984	0.93224	0.93651	0.93843	0.93907	0.94113	0.94333	0.94573
985	0.93230	0.93656	0.93849	0.93912	0.94118	0.94338	0.94578
986	0.93236	0.93662	0.93854	0.93917	0.94123	0.94343	0.94582
987	0.93243	0.93667	0.93859	0.93922	0.94128	0.94348	0.94587
988	0.93248	0.93672	0.93864	0.93928	0.94133	0.94352	0.94591
989	0.93254	0.93678	0.93869	0.93933	0.94138	0.94357	0.94596
990	0.93260	0.93683	0.93874	0.93938	0.94143	0.94362	0.94600
991	0.93265	0.93688	0.93880	0.93943	0.94148	0.94367	0.94605
992	0.93271	0.93693	0.93885	0.93948	0.94153	0.94371	0.94609
993	0.93277	0.93699	0.93890	0.93953	0.94157	0.94376	0.94614
994	0.93283	0.93704	0.93895	0.93958	0.94162	0.94381	0.94618
995	0.93289	0.93709	0.93900	0.93963	0.94167	0.94385	0.94623
996	0.93294	0.93714	0.93905	0.93968	0.94172	0.94390	0.94627
997	0.93299	0.93720	0.93911	0.93974	0.94177	0.94395	0.94632
998	0.93306	0.93725	0.93916	0.93978	0.94182	0.94399	0.94636
999	0.93311	0.93730	0.93921	0.93984	0.94187	0.94404	0.94640
1000	0.93317	0.93735	0.93926	0.93989	0.94191	0.94409	0.94645
