# Supplementary material for: A generalized outlier method with an automated test protocol applied to proficiency testing schemes: utilizing Monte Carlo simulation for generating critical values for the extended Grubbs statistic
Source: J AOAC Int. 2026 Apr 29;109(6):974–84. doi: 10.1093/jaoacint/qsag036 (PMC13732683; doi:10.1093/jaoacint/qsag036)
Supplement: Supplementary file 1 [file qsag036_supplementary_data.zip › aoac-25-0307-File018.pdf]

Extended Grubbs Statistics Tables for Multiple-6 Outliers

Table 1 – *GM6(6H0L or 0H6L)*. Critical values of upper significance levels for the Grubbs multiple-6 outlier test, applicable in situations where the six highest or six lowest observations are simultaneously identified as outliers, denoted as *GM6(6H0L)* and *GM6(0H6L)*, respectively.

N	0.1%	0.5%	1%	1.25%	2.5%	5%	10%
8	0.00000	0.00000	0.00000	0.00001	0.00003	0.00012	0.00048
9	0.00006	0.00031	0.00063	0.00079	0.00162	0.00338	0.00722
10	0.00078	0.00235	0.00384	0.00450	0.00741	0.01240	0.02124
11	0.00303	0.00710	0.01038	0.01172	0.01731	0.02586	0.03951
12	0.00715	0.01447	0.01977	0.02186	0.03016	0.04211	0.05989
13	0.01323	0.02411	0.03138	0.03421	0.04501	0.05994	0.08118
14	0.02112	0.03532	0.04449	0.04803	0.06106	0.07856	0.10259
15	0.03013	0.04779	0.05872	0.06280	0.07785	0.09745	0.12371
16	0.04033	0.06105	0.07351	0.07817	0.09485	0.11622	0.14429
17	0.05132	0.07494	0.08869	0.09374	0.11186	0.13466	0.16413
18	0.06284	0.08914	0.10396	0.10939	0.12867	0.15271	0.18331
19	0.07490	0.10329	0.11916	0.12498	0.14520	0.17018	0.20173
20	0.08712	0.11756	0.13422	0.14021	0.16138	0.18719	0.21931
21	0.09957	0.13156	0.14907	0.15536	0.17717	0.20354	0.23618
22	0.11212	0.14543	0.16349	0.17007	0.19250	0.21941	0.25232
23	0.12434	0.15912	0.17766	0.18441	0.20738	0.23457	0.26769
24	0.13667	0.17246	0.19156	0.19832	0.22174	0.24916	0.28246
25	0.14892	0.18545	0.20504	0.21197	0.23548	0.26319	0.29662
26	0.16070	0.19827	0.21817	0.22514	0.24896	0.27671	0.31013
27	0.17243	0.21067	0.23088	0.23781	0.26192	0.28972	0.32305
28	0.18374	0.22287	0.24322	0.25026	0.27430	0.30226	0.33544
29	0.19502	0.23463	0.25508	0.26225	0.28642	0.31431	0.34734
30	0.20612	0.24597	0.26669	0.27382	0.29806	0.32591	0.35879
31	0.21703	0.25729	0.27795	0.28511	0.30933	0.33711	0.36980
32	0.22740	0.26823	0.28892	0.29600	0.32020	0.34790	0.38037
33	0.23787	0.27872	0.29940	0.30661	0.33074	0.35826	0.39050
34	0.24798	0.28894	0.30962	0.31691	0.34091	0.36831	0.40032
35	0.25781	0.29894	0.31967	0.32682	0.35073	0.37801	0.40978
36	0.26772	0.30869	0.32931	0.33639	0.36023	0.38736	0.41884
37	0.27680	0.31803	0.33866	0.34576	0.36947	0.39640	0.42764
38	0.28599	0.32719	0.34778	0.35482	0.37846	0.40521	0.43609
39	0.29500	0.33607	0.35652	0.36363	0.38710	0.41368	0.44427
40	0.30370	0.34465	0.36520	0.37217	0.39556	0.42194	0.45220
41	0.31208	0.35315	0.37351	0.38049	0.40372	0.42986	0.45989
42	0.32042	0.36132	0.38157	0.38855	0.41164	0.43758	0.46732
43	0.32855	0.36919	0.38946	0.39646	0.41936	0.44503	0.47450
44	0.33632	0.37698	0.39718	0.40404	0.42685	0.45227	0.48146
45	0.34427	0.38461	0.40460	0.41144	0.43402	0.45934	0.48824
46	0.35169	0.39204	0.41184	0.41870	0.44103	0.46615	0.49478
47	0.35892	0.39927	0.41897	0.42568	0.44796	0.47280	0.50115
48	0.36609	0.40630	0.42584	0.43254	0.45459	0.47918	0.50729
49	0.37295	0.41304	0.43245	0.43908	0.46111	0.48546	0.51331
50	0.37970	0.41962	0.43900	0.44560	0.46743	0.49158	0.51913
51	0.38649	0.42614	0.44538	0.45195	0.47359	0.49755	0.52480
52	0.39318	0.43258	0.45162	0.45816	0.47957	0.50333	0.53034
53	0.39955	0.43867	0.45764	0.46412	0.48539	0.50896	0.53570
54	0.40597	0.44471	0.46355	0.46994	0.49107	0.51448	0.54095
55	0.41201	0.45059	0.46930	0.47562	0.49665	0.51980	0.54603
56	0.41792	0.45631	0.47495	0.48130	0.50208	0.52502	0.55099
57	0.42390	0.46200	0.48049	0.48674	0.50733	0.53011	0.55584
58	0.42963	0.46753	0.48589	0.49211	0.51253	0.53505	0.56055
59	0.43514	0.47294	0.49112	0.49731	0.51759	0.53990	0.56517
60	0.44058	0.47815	0.49630	0.50242	0.52248	0.54466	0.56967
61	0.44601	0.48331	0.50132	0.50741	0.52729	0.54931	0.57405
62	0.45115	0.48831	0.50623	0.51224	0.53196	0.55382	0.57836
63	0.45647	0.49327	0.51102	0.51700	0.53658	0.55823	0.58253
64	0.46154	0.49806	0.51571	0.52163	0.54109	0.56253	0.58663
65	0.46629	0.50274	0.52029	0.52619	0.54549	0.56674	0.59062
66	0.47119	0.50736	0.52480	0.53065	0.54979	0.57085	0.59452
67	0.47605	0.51198	0.52917	0.53498	0.55394	0.57490	0.59836
68	0.48087	0.51642	0.53349	0.53930	0.55808	0.57884	0.60210
69	0.48536	0.52072	0.53778	0.54351	0.56213	0.58271	0.60577
70	0.48977	0.52505	0.54192	0.54758	0.56610	0.58650	0.60938
71	0.49403	0.52926	0.54599	0.55162	0.57002	0.59022	0.61287
72	0.49831	0.53332	0.54999	0.55556	0.57380	0.59382	0.61630
73	0.50258	0.53727	0.55385	0.55946	0.57753	0.59738	0.61968

N	0.1%	0.5%	1%	1.25%	2.5%	5%	10%
74	0.50681	0.54130	0.55769	0.56321	0.58117	0.60086	0.62298
75	0.51093	0.54518	0.56149	0.56695	0.58474	0.60428	0.62622
76	0.51488	0.54899	0.56516	0.57055	0.58824	0.60764	0.62939
77	0.51900	0.55270	0.56877	0.57408	0.59170	0.61093	0.63249
78	0.52276	0.55643	0.57234	0.57761	0.59507	0.61419	0.63556
79	0.52655	0.56002	0.57577	0.58109	0.59842	0.61735	0.63855
80	0.53033	0.56352	0.57919	0.58445	0.60167	0.62044	0.64149
81	0.53396	0.56693	0.58250	0.58781	0.60486	0.62348	0.64437
82	0.53756	0.57035	0.58587	0.59104	0.60799	0.62648	0.64720
83	0.54109	0.57367	0.58913	0.59428	0.61110	0.62941	0.64998
84	0.54464	0.57701	0.59227	0.59738	0.61409	0.63230	0.65270
85	0.54792	0.58025	0.59543	0.60048	0.61707	0.63516	0.65537
86	0.55128	0.58340	0.59849	0.60352	0.61998	0.63796	0.65803
87	0.55468	0.58652	0.60153	0.60648	0.62285	0.64069	0.66060
88	0.55790	0.58959	0.60444	0.60939	0.62569	0.64338	0.66313
89	0.56118	0.59263	0.60732	0.61227	0.62841	0.64602	0.66562
90	0.56420	0.59559	0.61021	0.61511	0.63115	0.64862	0.66808
91	0.56730	0.59852	0.61303	0.61790	0.63383	0.65118	0.67051
92	0.57054	0.60132	0.61584	0.62066	0.63646	0.65369	0.67287
93	0.57349	0.60417	0.61858	0.62337	0.63910	0.65619	0.67517
94	0.57649	0.60699	0.62130	0.62606	0.64164	0.65862	0.67746
95	0.57947	0.60975	0.62394	0.62870	0.64415	0.66099	0.67972
96	0.58224	0.61249	0.62657	0.63125	0.64661	0.66338	0.68193
97	0.58515	0.61510	0.62911	0.63377	0.64906	0.66568	0.68414
98	0.58787	0.61774	0.63165	0.63630	0.65145	0.66795	0.68628
99	0.59054	0.62031	0.63411	0.63874	0.65379	0.67021	0.68841
100	0.59322	0.62285	0.63656	0.64116	0.65613	0.67241	0.69050
101	0.59601	0.62538	0.63902	0.64360	0.65843	0.67459	0.69256
102	0.59879	0.62786	0.64138	0.64594	0.66068	0.67672	0.69457
103	0.60141	0.63030	0.64374	0.64825	0.66288	0.67884	0.69658
104	0.60400	0.63270	0.64605	0.65051	0.66508	0.68092	0.69855
105	0.60649	0.63505	0.64830	0.65276	0.66721	0.68297	0.70048
106	0.60901	0.63736	0.65053	0.65498	0.66938	0.68500	0.70239
107	0.61155	0.63963	0.65277	0.65715	0.67149	0.68701	0.70427
108	0.61398	0.64196	0.65495	0.65933	0.67356	0.68897	0.70612
109	0.61630	0.64419	0.65713	0.66148	0.67558	0.69091	0.70794
110	0.61873	0.64640	0.65929	0.66357	0.67758	0.69283	0.70975
111	0.62105	0.64856	0.66132	0.66563	0.67955	0.69471	0.71150
112	0.62333	0.65068	0.66337	0.66764	0.68150	0.69658	0.71325
113	0.62553	0.65284	0.66541	0.66966	0.68344	0.69842	0.71498
114	0.62773	0.65483	0.66744	0.67163	0.68533	0.70022	0.71668
115	0.63001	0.65684	0.66942	0.67357	0.68721	0.70200	0.71836
116	0.63219	0.65885	0.67138	0.67553	0.68905	0.70376	0.72002
117	0.63439	0.66090	0.67331	0.67741	0.69085	0.70548	0.72164
118	0.63655	0.66283	0.67521	0.67932	0.69266	0.70719	0.72327
119	0.63854	0.66474	0.67707	0.68117	0.69446	0.70888	0.72485
120	0.64070	0.66668	0.67892	0.68300	0.69620	0.71055	0.72642
121	0.64281	0.66864	0.68074	0.68479	0.69793	0.71219	0.72797
122	0.64480	0.67053	0.68254	0.68656	0.69963	0.71380	0.72950
123	0.64678	0.67240	0.68433	0.68832	0.70130	0.71542	0.73100
124	0.64869	0.67419	0.68610	0.69005	0.70297	0.71699	0.73250
125	0.65072	0.67600	0.68780	0.69175	0.70461	0.71857	0.73398
126	0.65261	0.67777	0.68948	0.69346	0.70624	0.72010	0.73543
127	0.65450	0.67955	0.69120	0.69516	0.70784	0.72165	0.73687
128	0.65645	0.68127	0.69290	0.69682	0.70943	0.72314	0.73829
129	0.65820	0.68297	0.69457	0.69846	0.71098	0.72463	0.73969
130	0.66006	0.68466	0.69618	0.70007	0.71252	0.72611	0.74107
131	0.66187	0.68636	0.69783	0.70166	0.71404	0.72756	0.74244
132	0.66372	0.68808	0.69941	0.70322	0.71556	0.72899	0.74380
133	0.66550	0.68968	0.70100	0.70479	0.71707	0.73042	0.74514
134	0.66728	0.69126	0.70257	0.70633	0.71856	0.73182	0.74646
135	0.66896	0.69286	0.70411	0.70786	0.72002	0.73320	0.74777
136	0.67065	0.69447	0.70563	0.70937	0.72146	0.73458	0.74905
137	0.67233	0.69606	0.70714	0.71086	0.72288	0.73592	0.75031
138	0.67396	0.69757	0.70863	0.71232	0.72428	0.73725	0.75158
139	0.67566	0.69910	0.71013	0.71380	0.72569	0.73858	0.75282
140	0.67730	0.70064	0.71158	0.71524	0.72708	0.73989	0.75404
141	0.67883	0.70216	0.71302	0.71665	0.72844	0.74118	0.75526
142	0.68042	0.70366	0.71444	0.71806	0.72980	0.74246	0.75647
143	0.68201	0.70512	0.71584	0.71945	0.73111	0.74372	0.75767
144	0.68351	0.70654	0.71725	0.72082	0.73242	0.74499	0.75885
145	0.68516	0.70801	0.71865	0.72220	0.73371	0.74621	0.76000
146	0.68673	0.70943	0.72004	0.72355	0.73500	0.74745	0.76116
147	0.68826	0.71082	0.72139	0.72489	0.73628	0.74867	0.76229

N	0.1%	0.5%	1%	1.25%	2.5%	5%	10%
148	0.68971	0.71221	0.72273	0.72621	0.73752	0.74986	0.76341
149	0.69121	0.71357	0.72402	0.72751	0.73877	0.75102	0.76453
150	0.69266	0.71496	0.72532	0.72880	0.74001	0.75220	0.76563
151	0.69410	0.71633	0.72661	0.73006	0.74123	0.75336	0.76672
152	0.69552	0.71766	0.72792	0.73133	0.74244	0.75451	0.76779
153	0.69697	0.71900	0.72919	0.73259	0.74365	0.75564	0.76885
154	0.69842	0.72029	0.73040	0.73379	0.74483	0.75677	0.76992
155	0.69983	0.72159	0.73164	0.73503	0.74601	0.75788	0.77096
156	0.70119	0.72285	0.73289	0.73625	0.74718	0.75897	0.77199
157	0.70256	0.72410	0.73409	0.73744	0.74832	0.76006	0.77302
158	0.70389	0.72537	0.73528	0.73861	0.74946	0.76113	0.77403
159	0.70530	0.72661	0.73647	0.73983	0.75058	0.76221	0.77504
160	0.70667	0.72784	0.73766	0.74100	0.75169	0.76326	0.77603
161	0.70789	0.72906	0.73882	0.74214	0.75280	0.76431	0.77701
162	0.70919	0.73022	0.74000	0.74328	0.75389	0.76534	0.77798
163	0.71057	0.73139	0.74112	0.74440	0.75497	0.76637	0.77896
164	0.71182	0.73258	0.74226	0.74552	0.75604	0.76739	0.77991
165	0.71316	0.73374	0.74337	0.74662	0.75710	0.76839	0.78086
166	0.71434	0.73488	0.74450	0.74772	0.75815	0.76940	0.78180
167	0.71551	0.73602	0.74558	0.74878	0.75919	0.77038	0.78273
168	0.71673	0.73716	0.74667	0.74986	0.76022	0.77136	0.78365
169	0.71797	0.73832	0.74775	0.75092	0.76125	0.77234	0.78456
170	0.71918	0.73946	0.74883	0.75197	0.76226	0.77329	0.78546
171	0.72035	0.74054	0.74988	0.75302	0.76323	0.77423	0.78635
172	0.72147	0.74165	0.75097	0.75407	0.76424	0.77517	0.78724
173	0.72261	0.74274	0.75200	0.75508	0.76522	0.77611	0.78811
174	0.72379	0.74379	0.75306	0.75611	0.76619	0.77702	0.78898
175	0.72488	0.74485	0.75406	0.75711	0.76714	0.77794	0.78984
176	0.72602	0.74590	0.75504	0.75811	0.76809	0.77885	0.79069
177	0.72707	0.74695	0.75603	0.75910	0.76904	0.77975	0.79153
178	0.72823	0.74797	0.75705	0.76010	0.76998	0.78064	0.79238
179	0.72943	0.74902	0.75807	0.76106	0.77091	0.78152	0.79320
180	0.73049	0.75003	0.75903	0.76203	0.77184	0.78238	0.79403
181	0.73163	0.75103	0.75999	0.76299	0.77275	0.78326	0.79484
182	0.73268	0.75202	0.76093	0.76394	0.77366	0.78412	0.79565
183	0.73373	0.75301	0.76190	0.76487	0.77457	0.78497	0.79645
184	0.73473	0.75399	0.76283	0.76579	0.77545	0.78581	0.79724
185	0.73576	0.75495	0.76375	0.76671	0.77633	0.78663	0.79802
186	0.73680	0.75591	0.76470	0.76763	0.77721	0.78746	0.79880
187	0.73778	0.75685	0.76561	0.76853	0.77807	0.78829	0.79957
188	0.73876	0.75782	0.76650	0.76943	0.77894	0.78910	0.80034
189	0.73980	0.75873	0.76741	0.77032	0.77979	0.78991	0.80110
190	0.74079	0.75964	0.76832	0.77122	0.78063	0.79071	0.80184
191	0.74183	0.76056	0.76920	0.77210	0.78147	0.79150	0.80259
192	0.74281	0.76146	0.77008	0.77296	0.78229	0.79228	0.80333
193	0.74381	0.76236	0.77095	0.77381	0.78312	0.79305	0.80406
194	0.74477	0.76328	0.77181	0.77466	0.78392	0.79383	0.80479
195	0.74574	0.76418	0.77267	0.77551	0.78472	0.79461	0.80551
196	0.74675	0.76507	0.77352	0.77634	0.78552	0.79537	0.80623
197	0.74771	0.76593	0.77435	0.77718	0.78630	0.79612	0.80693
198	0.74865	0.76682	0.77518	0.77801	0.78710	0.79686	0.80763
199	0.74954	0.76768	0.77602	0.77882	0.78787	0.79761	0.80833
200	0.75050	0.76852	0.77685	0.77963	0.78865	0.79834	0.80902
201	0.75140	0.76937	0.77766	0.78043	0.78941	0.79908	0.80971
202	0.75233	0.77022	0.77848	0.78121	0.79018	0.79980	0.81039
203	0.75320	0.77105	0.77927	0.78201	0.79093	0.80052	0.81107
204	0.75406	0.77186	0.78006	0.78281	0.79167	0.80123	0.81173
205	0.75498	0.77267	0.78083	0.78358	0.79242	0.80194	0.81241
206	0.75584	0.77348	0.78163	0.78434	0.79316	0.80265	0.81307
207	0.75673	0.77429	0.78241	0.78512	0.79389	0.80334	0.81372
208	0.75757	0.77510	0.78318	0.78588	0.79460	0.80403	0.81437
209	0.75852	0.77590	0.78396	0.78664	0.79533	0.80472	0.81501
210	0.75932	0.77668	0.78472	0.78739	0.79604	0.80540	0.81565
211	0.76014	0.77746	0.78548	0.78813	0.79676	0.80607	0.81629
212	0.76097	0.77824	0.78623	0.78886	0.79746	0.80673	0.81691
213	0.76186	0.77903	0.78698	0.78960	0.79816	0.80740	0.81754
214	0.76266	0.77979	0.78772	0.79034	0.79886	0.80806	0.81815
215	0.76348	0.78054	0.78843	0.79107	0.79955	0.80871	0.81877
216	0.76430	0.78129	0.78917	0.79179	0.80023	0.80936	0.81938
217	0.76514	0.78204	0.78989	0.79250	0.80091	0.81000	0.81998
218	0.76593	0.78281	0.79062	0.79320	0.80158	0.81064	0.82059
219	0.76677	0.78354	0.79132	0.79390	0.80226	0.81128	0.82118
220	0.76751	0.78426	0.79200	0.79459	0.80293	0.81191	0.82178
221	0.76833	0.78497	0.79271	0.79527	0.80358	0.81254	0.82237

N	0.1%	0.5%	1%	1.25%	2.5%	5%	10%
222	0.76908	0.78569	0.79341	0.79595	0.80424	0.81316	0.82295
223	0.76985	0.78640	0.79407	0.79662	0.80488	0.81378	0.82352
224	0.77058	0.78709	0.79476	0.79729	0.80552	0.81439	0.82410
225	0.77135	0.78778	0.79544	0.79796	0.80616	0.81499	0.82467
226	0.77213	0.78850	0.79612	0.79862	0.80679	0.81559	0.82524
227	0.77285	0.78919	0.79677	0.79927	0.80741	0.81619	0.82579
228	0.77358	0.78987	0.79743	0.79992	0.80803	0.81678	0.82635
229	0.77437	0.79054	0.79808	0.80057	0.80867	0.81737	0.82691
230	0.77506	0.79124	0.79875	0.80123	0.80928	0.81795	0.82747
231	0.77577	0.79190	0.79940	0.80187	0.80989	0.81854	0.82802
232	0.77651	0.79257	0.80003	0.80250	0.81049	0.81911	0.82856
233	0.77725	0.79323	0.80068	0.80313	0.81111	0.81968	0.82910
234	0.77796	0.79390	0.80133	0.80376	0.81171	0.82024	0.82963
235	0.77863	0.79456	0.80193	0.80439	0.81230	0.82081	0.83016
236	0.77934	0.79521	0.80257	0.80501	0.81289	0.82137	0.83069
237	0.78000	0.79587	0.80318	0.80560	0.81347	0.82193	0.83121
238	0.78074	0.79652	0.80381	0.80622	0.81406	0.82247	0.83173
239	0.78140	0.79716	0.80443	0.80684	0.81463	0.82303	0.83225
240	0.78207	0.79777	0.80505	0.80744	0.81521	0.82357	0.83276
241	0.78274	0.79842	0.80565	0.80803	0.81578	0.82411	0.83327
242	0.78346	0.79904	0.80623	0.80863	0.81634	0.82465	0.83378
243	0.78411	0.79966	0.80685	0.80922	0.81690	0.82519	0.83428
244	0.78477	0.80028	0.80745	0.80980	0.81746	0.82572	0.83477
245	0.78541	0.80091	0.80804	0.81039	0.81801	0.82625	0.83527
246	0.78610	0.80152	0.80862	0.81097	0.81856	0.82677	0.83576
247	0.78680	0.80211	0.80920	0.81154	0.81910	0.82729	0.83625
248	0.78744	0.80272	0.80977	0.81212	0.81965	0.82782	0.83673
249	0.78809	0.80330	0.81034	0.81268	0.82019	0.82833	0.83722
250	0.78875	0.80389	0.81091	0.81324	0.82073	0.82883	0.83770
251	0.78941	0.80447	0.81146	0.81378	0.82125	0.82933	0.83817
252	0.79005	0.80505	0.81202	0.81433	0.82178	0.82984	0.83864
253	0.79065	0.80562	0.81256	0.81488	0.82230	0.83034	0.83911
254	0.79124	0.80621	0.81312	0.81543	0.82284	0.83083	0.83958
255	0.79188	0.80679	0.81368	0.81598	0.82336	0.83133	0.84004
256	0.79257	0.80736	0.81423	0.81652	0.82388	0.83182	0.84050
257	0.79318	0.80792	0.81477	0.81705	0.82439	0.83230	0.84096
258	0.79370	0.80847	0.81530	0.81758	0.82490	0.83278	0.84141
259	0.79431	0.80902	0.81585	0.81812	0.82541	0.83326	0.84187
260	0.79489	0.80958	0.81637	0.81863	0.82590	0.83374	0.84232
261	0.79547	0.81014	0.81691	0.81915	0.82640	0.83420	0.84277
262	0.79611	0.81069	0.81743	0.81966	0.82689	0.83467	0.84321
263	0.79668	0.81124	0.81794	0.82017	0.82739	0.83515	0.84365
264	0.79728	0.81177	0.81846	0.82068	0.82788	0.83561	0.84409
265	0.79785	0.81230	0.81896	0.82119	0.82836	0.83607	0.84452
266	0.79844	0.81284	0.81948	0.82169	0.82885	0.83653	0.84495
267	0.79902	0.81338	0.82000	0.82220	0.82933	0.83699	0.84539
268	0.79958	0.81390	0.82050	0.82271	0.82980	0.83744	0.84581
269	0.80013	0.81444	0.82100	0.82321	0.83028	0.83789	0.84624
270	0.80075	0.81496	0.82151	0.82370	0.83075	0.83834	0.84665
271	0.80128	0.81548	0.82199	0.82420	0.83123	0.83879	0.84708
272	0.80184	0.81599	0.82250	0.82468	0.83169	0.83923	0.84749
273	0.80240	0.81650	0.82299	0.82515	0.83216	0.83967	0.84791
274	0.80296	0.81699	0.82348	0.82563	0.83262	0.84011	0.84832
275	0.80350	0.81750	0.82395	0.82612	0.83308	0.84054	0.84872
276	0.80405	0.81801	0.82443	0.82659	0.83353	0.84097	0.84914
277	0.80459	0.81851	0.82492	0.82707	0.83398	0.84139	0.84954
278	0.80515	0.81900	0.82540	0.82753	0.83443	0.84182	0.84993
279	0.80569	0.81950	0.82587	0.82800	0.83487	0.84225	0.85033
280	0.80621	0.82000	0.82633	0.82846	0.83532	0.84267	0.85073
281	0.80671	0.82049	0.82680	0.82892	0.83576	0.84309	0.85113
282	0.80722	0.82098	0.82728	0.82938	0.83620	0.84350	0.85152
283	0.80777	0.82147	0.82775	0.82984	0.83663	0.84392	0.85191
284	0.80830	0.82195	0.82821	0.83029	0.83707	0.84433	0.85229
285	0.80882	0.82244	0.82865	0.83074	0.83749	0.84474	0.85268
286	0.80933	0.82291	0.82912	0.83119	0.83792	0.84514	0.85306
287	0.80985	0.82337	0.82956	0.83165	0.83836	0.84555	0.85345
288	0.81034	0.82385	0.83002	0.83209	0.83878	0.84595	0.85382
289	0.81085	0.82431	0.83047	0.83253	0.83920	0.84635	0.85420
290	0.81134	0.82478	0.83091	0.83297	0.83962	0.84675	0.85457
291	0.81183	0.82524	0.83135	0.83340	0.84003	0.84714	0.85495
292	0.81230	0.82569	0.83180	0.83383	0.84045	0.84754	0.85531
293	0.81280	0.82613	0.83223	0.83427	0.84086	0.84793	0.85568
294	0.81327	0.82658	0.83266	0.83470	0.84127	0.84832	0.85605
295	0.81377	0.82703	0.83310	0.83514	0.84168	0.84870	0.85641

N	0.1%	0.5%	1%	1.25%	2.5%	5%	10%
296	0.81427	0.82749	0.83353	0.83555	0.84209	0.84909	0.85678
297	0.81479	0.82793	0.83395	0.83596	0.84248	0.84947	0.85714
298	0.81526	0.82835	0.83437	0.83638	0.84288	0.84985	0.85750
299	0.81576	0.82878	0.83479	0.83680	0.84328	0.85023	0.85785
300	0.81622	0.82923	0.83522	0.83721	0.84367	0.85060	0.85820
301	0.81669	0.82966	0.83563	0.83761	0.84406	0.85097	0.85856
302	0.81716	0.83009	0.83606	0.83803	0.84445	0.85135	0.85891
303	0.81761	0.83052	0.83646	0.83843	0.84483	0.85171	0.85925
304	0.81807	0.83092	0.83687	0.83883	0.84523	0.85208	0.85960
305	0.81851	0.83136	0.83728	0.83923	0.84561	0.85244	0.85994
306	0.81900	0.83179	0.83770	0.83964	0.84599	0.85281	0.86028
307	0.81944	0.83221	0.83811	0.84003	0.84637	0.85317	0.86062
308	0.81989	0.83263	0.83851	0.84043	0.84675	0.85353	0.86096
309	0.82034	0.83303	0.83889	0.84082	0.84712	0.85389	0.86130
310	0.82081	0.83345	0.83930	0.84121	0.84750	0.85424	0.86163
311	0.82125	0.83385	0.83969	0.84161	0.84787	0.85460	0.86197
312	0.82170	0.83426	0.84009	0.84199	0.84824	0.85495	0.86230
313	0.82214	0.83466	0.84047	0.84238	0.84861	0.85530	0.86263
314	0.82259	0.83505	0.84085	0.84276	0.84898	0.85564	0.86296
315	0.82302	0.83547	0.84125	0.84315	0.84934	0.85599	0.86329
316	0.82347	0.83587	0.84163	0.84352	0.84970	0.85634	0.86361
317	0.82391	0.83626	0.84200	0.84390	0.85006	0.85668	0.86393
318	0.82431	0.83667	0.84238	0.84426	0.85041	0.85702	0.86426
319	0.82472	0.83706	0.84275	0.84464	0.85077	0.85736	0.86458
320	0.82517	0.83744	0.84313	0.84501	0.85113	0.85770	0.86490
321	0.82560	0.83782	0.84350	0.84538	0.85148	0.85804	0.86521
322	0.82602	0.83821	0.84387	0.84574	0.85183	0.85837	0.86552
323	0.82646	0.83861	0.84425	0.84611	0.85218	0.85870	0.86584
324	0.82687	0.83898	0.84461	0.84648	0.85253	0.85903	0.86615
325	0.82727	0.83937	0.84498	0.84685	0.85287	0.85936	0.86646
326	0.82768	0.83975	0.84534	0.84721	0.85321	0.85969	0.86677
327	0.82808	0.84014	0.84570	0.84756	0.85355	0.86001	0.86708
328	0.82850	0.84051	0.84605	0.84792	0.85389	0.86033	0.86738
329	0.82892	0.84089	0.84641	0.84827	0.85423	0.86065	0.86768
330	0.82928	0.84124	0.84677	0.84862	0.85457	0.86097	0.86798
331	0.82971	0.84160	0.84711	0.84897	0.85491	0.86129	0.86828
332	0.83012	0.84199	0.84747	0.84930	0.85524	0.86160	0.86858
333	0.83052	0.84236	0.84783	0.84965	0.85556	0.86192	0.86888
334	0.83089	0.84271	0.84818	0.85000	0.85589	0.86223	0.86917
335	0.83129	0.84308	0.84852	0.85034	0.85622	0.86255	0.86946
336	0.83168	0.84344	0.84887	0.85069	0.85655	0.86286	0.86975
337	0.83204	0.84380	0.84921	0.85103	0.85687	0.86316	0.87004
338	0.83243	0.84416	0.84956	0.85137	0.85720	0.86347	0.87033
339	0.83280	0.84451	0.84991	0.85170	0.85752	0.86377	0.87062
340	0.83319	0.84486	0.85025	0.85204	0.85784	0.86408	0.87091
341	0.83355	0.84522	0.85059	0.85238	0.85817	0.86438	0.87119
342	0.83391	0.84557	0.85092	0.85271	0.85848	0.86468	0.87148
343	0.83427	0.84592	0.85125	0.85304	0.85880	0.86498	0.87176
344	0.83463	0.84626	0.85159	0.85337	0.85911	0.86528	0.87204
345	0.83505	0.84662	0.85192	0.85370	0.85942	0.86558	0.87232
346	0.83540	0.84697	0.85226	0.85402	0.85973	0.86587	0.87259
347	0.83575	0.84730	0.85258	0.85436	0.86004	0.86616	0.87287
348	0.83613	0.84765	0.85291	0.85468	0.86035	0.86646	0.87315
349	0.83648	0.84798	0.85323	0.85499	0.86066	0.86675	0.87342
350	0.83688	0.84832	0.85356	0.85531	0.86096	0.86704	0.87369
351	0.83723	0.84865	0.85388	0.85564	0.86126	0.86733	0.87397
352	0.83755	0.84898	0.85420	0.85594	0.86157	0.86761	0.87424
353	0.83791	0.84932	0.85452	0.85625	0.86187	0.86790	0.87451
354	0.83829	0.84965	0.85483	0.85656	0.86217	0.86818	0.87478
355	0.83866	0.84995	0.85516	0.85688	0.86247	0.86846	0.87504
356	0.83900	0.85028	0.85546	0.85720	0.86277	0.86875	0.87531
357	0.83936	0.85059	0.85577	0.85750	0.86306	0.86902	0.87557
358	0.83970	0.85090	0.85608	0.85780	0.86336	0.86931	0.87583
359	0.84007	0.85123	0.85639	0.85811	0.86365	0.86959	0.87609
360	0.84039	0.85156	0.85669	0.85840	0.86394	0.86986	0.87635
361	0.84076	0.85187	0.85700	0.85871	0.86422	0.87013	0.87661
362	0.84111	0.85218	0.85730	0.85901	0.86451	0.87041	0.87687
363	0.84143	0.85249	0.85760	0.85931	0.86480	0.87068	0.87713
364	0.84177	0.85280	0.85789	0.85960	0.86508	0.87095	0.87738
365	0.84214	0.85311	0.85819	0.85989	0.86536	0.87122	0.87764
366	0.84248	0.85342	0.85848	0.86019	0.86565	0.87149	0.87789
367	0.84278	0.85373	0.85879	0.86049	0.86594	0.87176	0.87814
368	0.84314	0.85404	0.85908	0.86079	0.86622	0.87203	0.87840
369	0.84347	0.85435	0.85938	0.86107	0.86650	0.87229	0.87864

N	0.1%	0.5%	1%	1.25%	2.5%	5%	10%
370	0.84377	0.85465	0.85968	0.86136	0.86677	0.87255	0.87889
371	0.84410	0.85496	0.85996	0.86164	0.86704	0.87282	0.87914
372	0.84442	0.85527	0.86026	0.86193	0.86732	0.87308	0.87939
373	0.84475	0.85558	0.86055	0.86221	0.86759	0.87334	0.87963
374	0.84507	0.85588	0.86084	0.86251	0.86787	0.87360	0.87988
375	0.84541	0.85617	0.86113	0.86279	0.86814	0.87386	0.88012
376	0.84573	0.85648	0.86142	0.86307	0.86841	0.87411	0.88036
377	0.84605	0.85678	0.86170	0.86334	0.86867	0.87436	0.88060
378	0.84640	0.85706	0.86199	0.86362	0.86894	0.87462	0.88084
379	0.84670	0.85735	0.86227	0.86390	0.86921	0.87487	0.88108
380	0.84702	0.85763	0.86256	0.86418	0.86947	0.87513	0.88132
381	0.84730	0.85794	0.86283	0.86446	0.86974	0.87538	0.88156
382	0.84763	0.85823	0.86312	0.86474	0.87000	0.87563	0.88179
383	0.84793	0.85852	0.86339	0.86501	0.87026	0.87588	0.88202
384	0.84823	0.85880	0.86367	0.86528	0.87052	0.87612	0.88226
385	0.84855	0.85909	0.86396	0.86555	0.87077	0.87636	0.88249
386	0.84884	0.85938	0.86422	0.86582	0.87104	0.87661	0.88272
387	0.84915	0.85966	0.86450	0.86609	0.87130	0.87686	0.88295
388	0.84946	0.85995	0.86476	0.86636	0.87155	0.87710	0.88318
389	0.84977	0.86022	0.86503	0.86662	0.87180	0.87734	0.88341
390	0.85006	0.86050	0.86530	0.86688	0.87205	0.87758	0.88364
391	0.85037	0.86079	0.86556	0.86715	0.87231	0.87782	0.88387
392	0.85069	0.86107	0.86583	0.86741	0.87256	0.87806	0.88409
393	0.85100	0.86135	0.86609	0.86767	0.87281	0.87830	0.88431
394	0.85129	0.86163	0.86635	0.86793	0.87305	0.87853	0.88454
395	0.85159	0.86190	0.86662	0.86819	0.87330	0.87877	0.88476
396	0.85188	0.86218	0.86688	0.86845	0.87355	0.87900	0.88498
397	0.85216	0.86245	0.86714	0.86871	0.87379	0.87924	0.88521
398	0.85246	0.86271	0.86740	0.86897	0.87403	0.87948	0.88543
399	0.85277	0.86298	0.86766	0.86924	0.87428	0.87971	0.88565
400	0.85306	0.86324	0.86792	0.86948	0.87452	0.87994	0.88586
401	0.85333	0.86351	0.86818	0.86974	0.87476	0.88017	0.88608
402	0.85365	0.86378	0.86844	0.86999	0.87501	0.88040	0.88630
403	0.85395	0.86404	0.86870	0.87024	0.87525	0.88063	0.88651
404	0.85423	0.86431	0.86895	0.87049	0.87548	0.88085	0.88673
405	0.85452	0.86456	0.86920	0.87074	0.87572	0.88108	0.88694
406	0.85480	0.86482	0.86945	0.87099	0.87596	0.88130	0.88715
407	0.85507	0.86508	0.86971	0.87123	0.87619	0.88153	0.88737
408	0.85536	0.86534	0.86996	0.87148	0.87643	0.88175	0.88758
409	0.85562	0.86560	0.87020	0.87173	0.87667	0.88197	0.88778
410	0.85588	0.86585	0.87045	0.87198	0.87689	0.88219	0.88799
411	0.85616	0.86611	0.87070	0.87221	0.87713	0.88241	0.88820
412	0.85641	0.86635	0.87095	0.87245	0.87736	0.88264	0.88841
413	0.85669	0.86661	0.87119	0.87269	0.87759	0.88285	0.88861
414	0.85696	0.86686	0.87143	0.87293	0.87782	0.88307	0.88882
415	0.85722	0.86712	0.87168	0.87317	0.87805	0.88329	0.88902
416	0.85749	0.86736	0.87191	0.87341	0.87828	0.88350	0.88923
417	0.85775	0.86761	0.87215	0.87364	0.87850	0.88372	0.88943
418	0.85800	0.86786	0.87239	0.87388	0.87873	0.88394	0.88963
419	0.85827	0.86810	0.87262	0.87412	0.87895	0.88415	0.88984
420	0.85852	0.86835	0.87286	0.87435	0.87917	0.88436	0.89004
421	0.85880	0.86860	0.87310	0.87458	0.87940	0.88457	0.89023
422	0.85905	0.86884	0.87333	0.87482	0.87962	0.88478	0.89043
423	0.85933	0.86908	0.87355	0.87504	0.87983	0.88499	0.89063
424	0.85960	0.86931	0.87378	0.87526	0.88005	0.88520	0.89083
425	0.85987	0.86956	0.87402	0.87549	0.88028	0.88541	0.89102
426	0.86012	0.86981	0.87425	0.87573	0.88050	0.88562	0.89122
427	0.86037	0.87006	0.87448	0.87595	0.88071	0.88582	0.89141
428	0.86063	0.87031	0.87471	0.87618	0.88093	0.88603	0.89161
429	0.86091	0.87054	0.87495	0.87640	0.88114	0.88624	0.89180
430	0.86115	0.87078	0.87518	0.87663	0.88136	0.88644	0.89199
431	0.86139	0.87101	0.87540	0.87685	0.88157	0.88664	0.89219
432	0.86168	0.87125	0.87562	0.87708	0.88178	0.88684	0.89238
433	0.86192	0.87149	0.87585	0.87730	0.88199	0.88704	0.89256
434	0.86219	0.87172	0.87607	0.87751	0.88220	0.88725	0.89275
435	0.86244	0.87194	0.87630	0.87774	0.88241	0.88744	0.89294
436	0.86269	0.87218	0.87652	0.87796	0.88262	0.88764	0.89313
437	0.86293	0.87240	0.87674	0.87817	0.88283	0.88784	0.89332
438	0.86319	0.87263	0.87696	0.87839	0.88304	0.88804	0.89350
439	0.86344	0.87286	0.87717	0.87861	0.88325	0.88824	0.89369
440	0.86370	0.87307	0.87739	0.87882	0.88346	0.88843	0.89387
441	0.86394	0.87330	0.87761	0.87904	0.88366	0.88863	0.89406
442	0.86417	0.87351	0.87782	0.87925	0.88386	0.88882	0.89424
443	0.86442	0.87373	0.87803	0.87946	0.88407	0.88901	0.89442

N	0.1%	0.5%	1%	1.25%	2.5%	5%	10%
444	0.86466	0.87395	0.87825	0.87967	0.88427	0.88921	0.89461
445	0.86490	0.87417	0.87847	0.87989	0.88447	0.88940	0.89479
446	0.86513	0.87440	0.87867	0.88010	0.88467	0.88959	0.89497
447	0.86539	0.87462	0.87889	0.88030	0.88487	0.88978	0.89515
448	0.86562	0.87485	0.87910	0.88051	0.88507	0.88997	0.89533
449	0.86584	0.87507	0.87931	0.88071	0.88527	0.89016	0.89551
450	0.86610	0.87529	0.87952	0.88093	0.88546	0.89035	0.89569
451	0.86632	0.87550	0.87974	0.88113	0.88566	0.89054	0.89587
452	0.86655	0.87572	0.87995	0.88134	0.88586	0.89073	0.89604
453	0.86679	0.87593	0.88015	0.88154	0.88606	0.89091	0.89622
454	0.86704	0.87615	0.88037	0.88175	0.88625	0.89110	0.89639
455	0.86725	0.87636	0.88056	0.88196	0.88645	0.89128	0.89657
456	0.86748	0.87657	0.88076	0.88216	0.88664	0.89147	0.89674
457	0.86772	0.87678	0.88097	0.88237	0.88683	0.89166	0.89692
458	0.86793	0.87699	0.88118	0.88257	0.88703	0.89184	0.89709
459	0.86817	0.87720	0.88138	0.88277	0.88722	0.89202	0.89726
460	0.86839	0.87742	0.88157	0.88297	0.88741	0.89220	0.89744
461	0.86861	0.87762	0.88178	0.88318	0.88760	0.89238	0.89761
462	0.86884	0.87783	0.88197	0.88337	0.88779	0.89256	0.89778
463	0.86908	0.87804	0.88217	0.88357	0.88798	0.89274	0.89795
464	0.86930	0.87825	0.88238	0.88377	0.88817	0.89291	0.89812
465	0.86953	0.87846	0.88257	0.88396	0.88835	0.89310	0.89829
466	0.86972	0.87867	0.88277	0.88415	0.88854	0.89328	0.89846
467	0.86994	0.87887	0.88297	0.88434	0.88873	0.89345	0.89862
468	0.87017	0.87908	0.88316	0.88454	0.88892	0.89363	0.89879
469	0.87038	0.87927	0.88336	0.88474	0.88911	0.89380	0.89895
470	0.87060	0.87948	0.88356	0.88493	0.88929	0.89398	0.89912
471	0.87083	0.87968	0.88375	0.88513	0.88948	0.89415	0.89929
472	0.87103	0.87987	0.88395	0.88531	0.88966	0.89432	0.89945
473	0.87125	0.88009	0.88414	0.88550	0.88984	0.89450	0.89962
474	0.87146	0.88028	0.88433	0.88568	0.89002	0.89467	0.89978
475	0.87167	0.88048	0.88452	0.88587	0.89020	0.89484	0.89994
476	0.87189	0.88068	0.88472	0.88606	0.89038	0.89501	0.90011
477	0.87210	0.88087	0.88491	0.88625	0.89056	0.89518	0.90027
478	0.87233	0.88107	0.88509	0.88643	0.89074	0.89535	0.90043
479	0.87253	0.88127	0.88528	0.88662	0.89092	0.89553	0.90059
480	0.87276	0.88147	0.88547	0.88681	0.89110	0.89569	0.90075
481	0.87296	0.88166	0.88566	0.88700	0.89127	0.89587	0.90091
482	0.87317	0.88186	0.88585	0.88719	0.89144	0.89603	0.90107
483	0.87337	0.88206	0.88604	0.88738	0.89161	0.89620	0.90122
484	0.87356	0.88224	0.88622	0.88755	0.89179	0.89636	0.90138
485	0.87379	0.88243	0.88641	0.88774	0.89197	0.89653	0.90154
486	0.87401	0.88262	0.88659	0.88792	0.89214	0.89670	0.90169
487	0.87422	0.88282	0.88677	0.88810	0.89231	0.89686	0.90185
488	0.87442	0.88300	0.88695	0.88828	0.89249	0.89703	0.90201
489	0.87461	0.88319	0.88713	0.88845	0.89266	0.89719	0.90216
490	0.87481	0.88337	0.88732	0.88863	0.89283	0.89735	0.90231
491	0.87501	0.88356	0.88750	0.88880	0.89300	0.89752	0.90246
492	0.87523	0.88374	0.88768	0.88898	0.89318	0.89768	0.90262
493	0.87543	0.88394	0.88786	0.88915	0.89334	0.89784	0.90277
494	0.87562	0.88413	0.88803	0.88933	0.89351	0.89800	0.90292
495	0.87582	0.88432	0.88821	0.88951	0.89369	0.89816	0.90307
496	0.87602	0.88450	0.88839	0.88968	0.89385	0.89832	0.90322
497	0.87620	0.88469	0.88857	0.88985	0.89402	0.89848	0.90337
498	0.87640	0.88488	0.88875	0.89003	0.89419	0.89864	0.90352
499	0.87660	0.88506	0.88892	0.89021	0.89435	0.89880	0.90367
500	0.87682	0.88523	0.88909	0.89038	0.89452	0.89895	0.90382
501	0.87701	0.88542	0.88926	0.89055	0.89469	0.89911	0.90397
502	0.87719	0.88560	0.88944	0.89072	0.89485	0.89927	0.90412
503	0.87739	0.88579	0.88961	0.89089	0.89502	0.89942	0.90427
504	0.87761	0.88596	0.88978	0.89106	0.89518	0.89958	0.90441
505	0.87781	0.88614	0.88996	0.89124	0.89534	0.89973	0.90456
506	0.87799	0.88633	0.89014	0.89141	0.89551	0.89989	0.90470
507	0.87821	0.88651	0.89030	0.89158	0.89567	0.90004	0.90485
508	0.87840	0.88667	0.89048	0.89175	0.89583	0.90020	0.90499
509	0.87859	0.88685	0.89065	0.89192	0.89599	0.90035	0.90514
510	0.87877	0.88703	0.89082	0.89208	0.89615	0.90050	0.90528
511	0.87895	0.88721	0.89099	0.89225	0.89631	0.90066	0.90543
512	0.87914	0.88739	0.89116	0.89242	0.89647	0.90081	0.90557
513	0.87933	0.88756	0.89133	0.89258	0.89662	0.90096	0.90571
514	0.87952	0.88773	0.89150	0.89275	0.89678	0.90111	0.90585
515	0.87970	0.88790	0.89167	0.89291	0.89694	0.90126	0.90599
516	0.87988	0.88808	0.89183	0.89307	0.89710	0.90141	0.90614
517	0.88008	0.88825	0.89200	0.89324	0.89725	0.90156	0.90628

N	0.1%	0.5%	1%	1.25%	2.5%	5%	10%
518	0.88027	0.88841	0.89216	0.89340	0.89741	0.90171	0.90642
519	0.88043	0.88859	0.89233	0.89357	0.89756	0.90185	0.90656
520	0.88065	0.88875	0.89249	0.89373	0.89772	0.90200	0.90670
521	0.88083	0.88893	0.89265	0.89389	0.89788	0.90215	0.90683
522	0.88102	0.88911	0.89281	0.89405	0.89803	0.90230	0.90697
523	0.88121	0.88928	0.89297	0.89421	0.89818	0.90244	0.90711
524	0.88140	0.88945	0.89314	0.89437	0.89834	0.90259	0.90725
525	0.88157	0.88961	0.89330	0.89453	0.89848	0.90273	0.90738
526	0.88175	0.88978	0.89345	0.89469	0.89864	0.90287	0.90752
527	0.88193	0.88995	0.89361	0.89484	0.89879	0.90302	0.90766
528	0.88211	0.89012	0.89377	0.89499	0.89894	0.90317	0.90779
529	0.88229	0.89028	0.89393	0.89515	0.89909	0.90331	0.90793
530	0.88248	0.89045	0.89409	0.89531	0.89924	0.90345	0.90806
531	0.88265	0.89061	0.89425	0.89547	0.89939	0.90359	0.90820
532	0.88284	0.89077	0.89441	0.89563	0.89953	0.90373	0.90833
533	0.88301	0.89094	0.89456	0.89578	0.89969	0.90387	0.90846
534	0.88319	0.89110	0.89472	0.89593	0.89984	0.90401	0.90860
535	0.88337	0.89127	0.89487	0.89608	0.89999	0.90415	0.90873
536	0.88357	0.89143	0.89503	0.89624	0.90013	0.90429	0.90886
537	0.88373	0.89159	0.89519	0.89639	0.90028	0.90443	0.90899
538	0.88389	0.89175	0.89534	0.89654	0.90043	0.90457	0.90912
539	0.88407	0.89191	0.89549	0.89669	0.90057	0.90471	0.90925
540	0.88424	0.89206	0.89564	0.89684	0.90072	0.90485	0.90939
541	0.88440	0.89222	0.89579	0.89700	0.90086	0.90498	0.90952
542	0.88457	0.89238	0.89594	0.89714	0.90100	0.90512	0.90965
543	0.88474	0.89254	0.89610	0.89730	0.90115	0.90526	0.90978
544	0.88491	0.89270	0.89625	0.89744	0.90129	0.90540	0.90990
545	0.88507	0.89286	0.89640	0.89759	0.90143	0.90553	0.91003
546	0.88524	0.89301	0.89655	0.89775	0.90157	0.90567	0.91016
547	0.88542	0.89317	0.89669	0.89789	0.90171	0.90580	0.91029
548	0.88557	0.89332	0.89684	0.89803	0.90186	0.90593	0.91042
549	0.88574	0.89347	0.89699	0.89818	0.90199	0.90607	0.91054
550	0.88591	0.89362	0.89714	0.89832	0.90213	0.90620	0.91067
551	0.88608	0.89378	0.89729	0.89847	0.90228	0.90634	0.91080
552	0.88626	0.89393	0.89744	0.89862	0.90241	0.90647	0.91092
553	0.88642	0.89409	0.89759	0.89877	0.90256	0.90660	0.91105
554	0.88661	0.89424	0.89774	0.89892	0.90269	0.90673	0.91117
555	0.88677	0.89439	0.89788	0.89907	0.90283	0.90687	0.91129
556	0.88692	0.89455	0.89804	0.89921	0.90297	0.90700	0.91142
557	0.88708	0.89470	0.89818	0.89935	0.90310	0.90713	0.91154
558	0.88725	0.89484	0.89832	0.89949	0.90324	0.90726	0.91167
559	0.88743	0.89499	0.89847	0.89964	0.90338	0.90739	0.91179
560	0.88759	0.89515	0.89861	0.89978	0.90352	0.90752	0.91191
561	0.88774	0.89529	0.89876	0.89992	0.90365	0.90765	0.91203
562	0.88791	0.89544	0.89891	0.90006	0.90378	0.90778	0.91216
563	0.88808	0.89560	0.89905	0.90020	0.90392	0.90790	0.91228
564	0.88824	0.89574	0.89920	0.90034	0.90406	0.90803	0.91240
565	0.88841	0.89588	0.89933	0.90048	0.90419	0.90816	0.91252
566	0.88856	0.89603	0.89948	0.90062	0.90432	0.90829	0.91264
567	0.88874	0.89618	0.89962	0.90076	0.90445	0.90841	0.91276
568	0.88887	0.89633	0.89976	0.90089	0.90459	0.90854	0.91288
569	0.88902	0.89647	0.89991	0.90103	0.90472	0.90867	0.91300
570	0.88919	0.89662	0.90004	0.90117	0.90485	0.90879	0.91312
571	0.88934	0.89676	0.90018	0.90131	0.90498	0.90892	0.91324
572	0.88949	0.89691	0.90032	0.90145	0.90512	0.90905	0.91335
573	0.88965	0.89705	0.90046	0.90158	0.90524	0.90917	0.91347
574	0.88980	0.89720	0.90060	0.90172	0.90537	0.90929	0.91359
575	0.88995	0.89734	0.90073	0.90185	0.90550	0.90941	0.91370
576	0.89012	0.89748	0.90087	0.90199	0.90563	0.90954	0.91382
577	0.89029	0.89762	0.90100	0.90212	0.90576	0.90966	0.91394
578	0.89045	0.89776	0.90114	0.90225	0.90589	0.90979	0.91405
579	0.89060	0.89790	0.90127	0.90239	0.90602	0.90991	0.91417
580	0.89074	0.89804	0.90141	0.90253	0.90615	0.91004	0.91428
581	0.89088	0.89817	0.90155	0.90266	0.90627	0.91016	0.91440
582	0.89102	0.89831	0.90168	0.90280	0.90640	0.91028	0.91451
583	0.89119	0.89845	0.90182	0.90293	0.90653	0.91040	0.91463
584	0.89134	0.89860	0.90196	0.90306	0.90666	0.91052	0.91474
585	0.89149	0.89874	0.90209	0.90319	0.90678	0.91064	0.91485
586	0.89163	0.89887	0.90222	0.90332	0.90691	0.91076	0.91497
587	0.89179	0.89901	0.90235	0.90345	0.90703	0.91088	0.91508
588	0.89195	0.89914	0.90248	0.90359	0.90716	0.91100	0.91519
589	0.89211	0.89929	0.90262	0.90372	0.90728	0.91111	0.91531
590	0.89225	0.89942	0.90275	0.90385	0.90740	0.91123	0.91542
591	0.89240	0.89956	0.90288	0.90398	0.90753	0.91135	0.91553

N	0.1%	0.5%	1%	1.25%	2.5%	5%	10%
592	0.89254	0.89970	0.90301	0.90410	0.90765	0.91147	0.91564
593	0.89268	0.89984	0.90314	0.90423	0.90778	0.91159	0.91575
594	0.89283	0.89998	0.90327	0.90436	0.90790	0.91170	0.91586
595	0.89299	0.90011	0.90340	0.90449	0.90802	0.91182	0.91597
596	0.89313	0.90024	0.90353	0.90462	0.90815	0.91194	0.91608
597	0.89327	0.90037	0.90366	0.90475	0.90827	0.91205	0.91619
598	0.89341	0.90051	0.90378	0.90487	0.90839	0.91216	0.91630
599	0.89356	0.90064	0.90391	0.90500	0.90851	0.91228	0.91641
600	0.89369	0.90076	0.90404	0.90513	0.90863	0.91240	0.91652
601	0.89382	0.90090	0.90416	0.90525	0.90875	0.91251	0.91663
602	0.89398	0.90103	0.90429	0.90538	0.90887	0.91263	0.91674
603	0.89411	0.90116	0.90442	0.90550	0.90899	0.91274	0.91684
604	0.89425	0.90129	0.90455	0.90563	0.90911	0.91285	0.91695
605	0.89437	0.90142	0.90467	0.90575	0.90923	0.91297	0.91706
606	0.89451	0.90154	0.90480	0.90587	0.90935	0.91308	0.91717
607	0.89466	0.90167	0.90492	0.90599	0.90947	0.91319	0.91727
608	0.89479	0.90180	0.90505	0.90612	0.90958	0.91331	0.91738
609	0.89492	0.90192	0.90517	0.90625	0.90970	0.91342	0.91748
610	0.89506	0.90206	0.90530	0.90637	0.90982	0.91353	0.91759
611	0.89520	0.90219	0.90543	0.90649	0.90993	0.91364	0.91770
612	0.89534	0.90232	0.90555	0.90661	0.91005	0.91375	0.91780
613	0.89549	0.90245	0.90567	0.90674	0.91017	0.91386	0.91791
614	0.89562	0.90257	0.90580	0.90686	0.91029	0.91398	0.91801
615	0.89577	0.90271	0.90592	0.90698	0.91040	0.91408	0.91811
616	0.89591	0.90284	0.90604	0.90710	0.91052	0.91419	0.91822
617	0.89604	0.90296	0.90616	0.90722	0.91063	0.91430	0.91832
618	0.89618	0.90310	0.90628	0.90734	0.91075	0.91441	0.91843
619	0.89632	0.90321	0.90641	0.90746	0.91087	0.91453	0.91853
620	0.89644	0.90335	0.90653	0.90758	0.91098	0.91463	0.91863
621	0.89659	0.90347	0.90664	0.90770	0.91110	0.91474	0.91873
622	0.89672	0.90359	0.90676	0.90782	0.91121	0.91485	0.91884
623	0.89685	0.90371	0.90688	0.90794	0.91132	0.91495	0.91894
624	0.89698	0.90384	0.90700	0.90806	0.91144	0.91506	0.91904
625	0.89712	0.90396	0.90712	0.90817	0.91155	0.91517	0.91914
626	0.89724	0.90408	0.90724	0.90829	0.91166	0.91528	0.91924
627	0.89739	0.90420	0.90736	0.90841	0.91177	0.91539	0.91934
628	0.89753	0.90433	0.90748	0.90852	0.91188	0.91549	0.91945
629	0.89767	0.90445	0.90760	0.90864	0.91200	0.91560	0.91955
630	0.89779	0.90457	0.90771	0.90875	0.91211	0.91570	0.91965
631	0.89792	0.90470	0.90783	0.90886	0.91222	0.91581	0.91974
632	0.89805	0.90482	0.90795	0.90898	0.91233	0.91591	0.91984
633	0.89817	0.90493	0.90806	0.90909	0.91244	0.91602	0.91994
634	0.89829	0.90505	0.90817	0.90921	0.91255	0.91612	0.92004
635	0.89842	0.90517	0.90829	0.90933	0.91265	0.91622	0.92014
636	0.89853	0.90530	0.90840	0.90944	0.91276	0.91633	0.92024
637	0.89867	0.90542	0.90852	0.90955	0.91287	0.91643	0.92034
638	0.89879	0.90554	0.90864	0.90966	0.91298	0.91654	0.92044
639	0.89891	0.90566	0.90875	0.90978	0.91309	0.91664	0.92053
640	0.89904	0.90578	0.90887	0.90989	0.91320	0.91674	0.92063
641	0.89916	0.90589	0.90898	0.91001	0.91330	0.91685	0.92072
642	0.89928	0.90600	0.90910	0.91012	0.91341	0.91695	0.92082
643	0.89940	0.90612	0.90921	0.91023	0.91352	0.91705	0.92092
644	0.89954	0.90624	0.90932	0.91033	0.91362	0.91715	0.92102
645	0.89968	0.90635	0.90943	0.91045	0.91373	0.91725	0.92111
646	0.89979	0.90647	0.90953	0.91056	0.91384	0.91735	0.92121
647	0.89991	0.90659	0.90965	0.91067	0.91394	0.91745	0.92130
648	0.90003	0.90670	0.90976	0.91078	0.91405	0.91756	0.92140
649	0.90017	0.90681	0.90988	0.91089	0.91415	0.91765	0.92149
650	0.90028	0.90693	0.90998	0.91100	0.91426	0.91775	0.92159
651	0.90040	0.90705	0.91010	0.91111	0.91436	0.91785	0.92168
652	0.90052	0.90716	0.91021	0.91122	0.91446	0.91795	0.92178
653	0.90067	0.90727	0.91032	0.91133	0.91457	0.91805	0.92187
654	0.90078	0.90739	0.91043	0.91144	0.91467	0.91815	0.92196
655	0.90092	0.90750	0.91054	0.91154	0.91478	0.91825	0.92206
656	0.90106	0.90762	0.91065	0.91165	0.91488	0.91835	0.92215
657	0.90118	0.90773	0.91075	0.91176	0.91498	0.91845	0.92224
658	0.90129	0.90785	0.91086	0.91187	0.91509	0.91854	0.92234
659	0.90141	0.90796	0.91097	0.91197	0.91519	0.91864	0.92243
660	0.90154	0.90807	0.91108	0.91208	0.91530	0.91874	0.92252
661	0.90167	0.90818	0.91119	0.91219	0.91540	0.91884	0.92261
662	0.90179	0.90829	0.91129	0.91229	0.91550	0.91894	0.92271
663	0.90191	0.90840	0.91141	0.91240	0.91560	0.91903	0.92280
664	0.90203	0.90852	0.91152	0.91251	0.91570	0.91913	0.92289
665	0.90215	0.90863	0.91162	0.91261	0.91580	0.91922	0.92298

N	0.1%	0.5%	1%	1.25%	2.5%	5%	10%
666	0.90225	0.90873	0.91173	0.91271	0.91590	0.91932	0.92307
667	0.90237	0.90884	0.91184	0.91282	0.91600	0.91942	0.92316
668	0.90249	0.90895	0.91194	0.91292	0.91610	0.91951	0.92325
669	0.90262	0.90906	0.91205	0.91302	0.91620	0.91961	0.92334
670	0.90273	0.90917	0.91215	0.91313	0.91630	0.91970	0.92343
671	0.90285	0.90928	0.91225	0.91323	0.91640	0.91980	0.92352
672	0.90296	0.90939	0.91236	0.91334	0.91650	0.91989	0.92361
673	0.90307	0.90950	0.91246	0.91344	0.91660	0.91999	0.92370
674	0.90320	0.90960	0.91256	0.91355	0.91670	0.92008	0.92378
675	0.90331	0.90971	0.91267	0.91365	0.91680	0.92017	0.92387
676	0.90343	0.90982	0.91277	0.91375	0.91689	0.92027	0.92396
677	0.90354	0.90993	0.91287	0.91385	0.91699	0.92036	0.92405
678	0.90365	0.91004	0.91298	0.91395	0.91709	0.92046	0.92414
679	0.90377	0.91014	0.91308	0.91406	0.91719	0.92055	0.92423
680	0.90387	0.91024	0.91319	0.91416	0.91728	0.92064	0.92431
681	0.90398	0.91035	0.91329	0.91426	0.91738	0.92073	0.92440
682	0.90409	0.91046	0.91339	0.91436	0.91748	0.92083	0.92449
683	0.90420	0.91056	0.91349	0.91446	0.91757	0.92092	0.92458
684	0.90432	0.91067	0.91360	0.91456	0.91767	0.92101	0.92466
685	0.90445	0.91078	0.91370	0.91466	0.91777	0.92110	0.92475
686	0.90457	0.91088	0.91380	0.91476	0.91786	0.92119	0.92484
687	0.90468	0.91099	0.91390	0.91486	0.91796	0.92128	0.92492
688	0.90479	0.91109	0.91400	0.91496	0.91805	0.92137	0.92501
689	0.90490	0.91120	0.91410	0.91505	0.91815	0.92146	0.92509
690	0.90502	0.91130	0.91420	0.91515	0.91824	0.92156	0.92518
691	0.90512	0.91140	0.91430	0.91525	0.91834	0.92165	0.92526
692	0.90524	0.91150	0.91440	0.91535	0.91843	0.92173	0.92535
693	0.90535	0.91161	0.91449	0.91545	0.91853	0.92183	0.92543
694	0.90546	0.91171	0.91460	0.91554	0.91862	0.92191	0.92552
695	0.90558	0.91182	0.91470	0.91565	0.91871	0.92200	0.92560
696	0.90568	0.91192	0.91479	0.91574	0.91880	0.92209	0.92569
697	0.90579	0.91203	0.91489	0.91584	0.91890	0.92218	0.92577
698	0.90590	0.91213	0.91499	0.91594	0.91899	0.92227	0.92585
699	0.90601	0.91222	0.91508	0.91603	0.91908	0.92236	0.92594
700	0.90613	0.91233	0.91518	0.91613	0.91918	0.92244	0.92602
701	0.90624	0.91243	0.91528	0.91623	0.91927	0.92253	0.92610
702	0.90634	0.91253	0.91538	0.91632	0.91936	0.92262	0.92619
703	0.90644	0.91264	0.91547	0.91642	0.91945	0.92271	0.92627
704	0.90656	0.91273	0.91557	0.91651	0.91954	0.92279	0.92635
705	0.90667	0.91284	0.91567	0.91661	0.91964	0.92288	0.92644
706	0.90678	0.91294	0.91577	0.91670	0.91973	0.92297	0.92652
707	0.90690	0.91304	0.91586	0.91680	0.91982	0.92305	0.92660
708	0.90700	0.91314	0.91596	0.91689	0.91991	0.92314	0.92668
709	0.90710	0.91324	0.91605	0.91699	0.92000	0.92323	0.92676
710	0.90720	0.91334	0.91615	0.91708	0.92009	0.92331	0.92684
711	0.90731	0.91344	0.91624	0.91717	0.92018	0.92340	0.92692
712	0.90741	0.91353	0.91634	0.91727	0.92026	0.92348	0.92700
713	0.90751	0.91364	0.91643	0.91736	0.92035	0.92357	0.92709
714	0.90762	0.91373	0.91653	0.91745	0.92044	0.92365	0.92717
715	0.90773	0.91383	0.91662	0.91755	0.92053	0.92374	0.92725
716	0.90784	0.91392	0.91671	0.91764	0.92062	0.92382	0.92733
717	0.90795	0.91402	0.91681	0.91773	0.92071	0.92391	0.92741
718	0.90806	0.91412	0.91690	0.91782	0.92080	0.92399	0.92749
719	0.90817	0.91421	0.91699	0.91791	0.92089	0.92407	0.92757
720	0.90827	0.91431	0.91708	0.91800	0.92098	0.92416	0.92765
721	0.90837	0.91440	0.91718	0.91810	0.92106	0.92425	0.92772
722	0.90848	0.91450	0.91727	0.91819	0.92115	0.92433	0.92780
723	0.90858	0.91460	0.91736	0.91828	0.92124	0.92441	0.92788
724	0.90869	0.91469	0.91745	0.91837	0.92132	0.92449	0.92796
725	0.90880	0.91479	0.91754	0.91846	0.92141	0.92458	0.92804
726	0.90890	0.91489	0.91763	0.91855	0.92149	0.92466	0.92812
727	0.90899	0.91499	0.91772	0.91864	0.92158	0.92474	0.92819
728	0.90909	0.91508	0.91781	0.91873	0.92167	0.92482	0.92827
729	0.90919	0.91517	0.91790	0.91882	0.92175	0.92491	0.92835
730	0.90928	0.91527	0.91799	0.91890	0.92184	0.92499	0.92843
731	0.90938	0.91536	0.91808	0.91899	0.92192	0.92507	0.92850
732	0.90948	0.91546	0.91817	0.91909	0.92201	0.92515	0.92858
733	0.90958	0.91555	0.91827	0.91918	0.92210	0.92523	0.92866
734	0.90968	0.91565	0.91835	0.91927	0.92218	0.92532	0.92874
735	0.90977	0.91574	0.91844	0.91935	0.92227	0.92539	0.92881
736	0.90988	0.91583	0.91853	0.91944	0.92235	0.92548	0.92889
737	0.90999	0.91592	0.91862	0.91953	0.92243	0.92556	0.92897
738	0.91010	0.91601	0.91871	0.91962	0.92251	0.92564	0.92904
739	0.91020	0.91610	0.91880	0.91971	0.92260	0.92572	0.92912

N	0.1%	0.5%	1%	1.25%	2.5%	5%	10%
740	0.91029	0.91620	0.91889	0.91979	0.92268	0.92580	0.92919
741	0.91039	0.91629	0.91897	0.91988	0.92277	0.92588	0.92927
742	0.91048	0.91638	0.91906	0.91997	0.92285	0.92596	0.92934
743	0.91058	0.91647	0.91915	0.92005	0.92293	0.92603	0.92942
744	0.91066	0.91656	0.91924	0.92014	0.92302	0.92611	0.92949
745	0.91075	0.91665	0.91933	0.92023	0.92310	0.92619	0.92957
746	0.91085	0.91674	0.91942	0.92032	0.92318	0.92627	0.92964
747	0.91095	0.91684	0.91951	0.92040	0.92327	0.92635	0.92972
748	0.91105	0.91692	0.91959	0.92048	0.92335	0.92643	0.92979
749	0.91115	0.91701	0.91968	0.92057	0.92343	0.92650	0.92987
750	0.91123	0.91710	0.91976	0.92066	0.92351	0.92658	0.92994
751	0.91135	0.91719	0.91985	0.92074	0.92359	0.92666	0.93001
752	0.91145	0.91728	0.91994	0.92082	0.92367	0.92674	0.93009
753	0.91154	0.91737	0.92002	0.92091	0.92375	0.92682	0.93016
754	0.91162	0.91746	0.92011	0.92099	0.92384	0.92689	0.93023
755	0.91173	0.91755	0.92019	0.92108	0.92391	0.92697	0.93031
756	0.91182	0.91764	0.92028	0.92116	0.92399	0.92705	0.93038
757	0.91192	0.91772	0.92036	0.92124	0.92407	0.92713	0.93045
758	0.91202	0.91781	0.92045	0.92133	0.92416	0.92720	0.93052
759	0.91212	0.91789	0.92053	0.92141	0.92424	0.92728	0.93060
760	0.91221	0.91798	0.92061	0.92150	0.92432	0.92736	0.93067
761	0.91231	0.91807	0.92070	0.92158	0.92440	0.92743	0.93074
762	0.91241	0.91815	0.92078	0.92166	0.92448	0.92751	0.93081
763	0.91249	0.91824	0.92087	0.92174	0.92455	0.92758	0.93088
764	0.91258	0.91833	0.92095	0.92183	0.92463	0.92766	0.93096
765	0.91267	0.91842	0.92103	0.92191	0.92471	0.92773	0.93103
766	0.91275	0.91851	0.92111	0.92199	0.92479	0.92781	0.93110
767	0.91285	0.91859	0.92120	0.92207	0.92487	0.92788	0.93117
768	0.91293	0.91868	0.92128	0.92215	0.92495	0.92796	0.93124
769	0.91304	0.91876	0.92136	0.92224	0.92503	0.92803	0.93131
770	0.91313	0.91884	0.92144	0.92232	0.92511	0.92811	0.93138
771	0.91322	0.91893	0.92153	0.92240	0.92518	0.92818	0.93145
772	0.91331	0.91901	0.92161	0.92248	0.92526	0.92826	0.93152
773	0.91341	0.91910	0.92169	0.92256	0.92534	0.92833	0.93159
774	0.91349	0.91919	0.92177	0.92264	0.92542	0.92840	0.93166
775	0.91358	0.91927	0.92185	0.92272	0.92549	0.92848	0.93173
776	0.91368	0.91935	0.92194	0.92280	0.92557	0.92855	0.93180
777	0.91376	0.91943	0.92202	0.92288	0.92565	0.92862	0.93187
778	0.91387	0.91952	0.92210	0.92296	0.92572	0.92869	0.93194
779	0.91397	0.91960	0.92218	0.92304	0.92580	0.92877	0.93201
780	0.91406	0.91969	0.92226	0.92312	0.92587	0.92884	0.93208
781	0.91415	0.91977	0.92234	0.92320	0.92595	0.92891	0.93215
782	0.91424	0.91985	0.92242	0.92328	0.92603	0.92899	0.93222
783	0.91433	0.91994	0.92250	0.92336	0.92610	0.92906	0.93229
784	0.91442	0.92002	0.92258	0.92343	0.92618	0.92913	0.93235
785	0.91450	0.92010	0.92265	0.92351	0.92625	0.92920	0.93242
786	0.91458	0.92019	0.92273	0.92359	0.92633	0.92928	0.93249
787	0.91467	0.92026	0.92282	0.92367	0.92640	0.92935	0.93256
788	0.91476	0.92034	0.92289	0.92374	0.92648	0.92942	0.93263
789	0.91485	0.92042	0.92297	0.92382	0.92655	0.92949	0.93270
790	0.91495	0.92050	0.92305	0.92390	0.92663	0.92956	0.93276
791	0.91503	0.92059	0.92313	0.92397	0.92670	0.92963	0.93283
792	0.91511	0.92067	0.92320	0.92405	0.92678	0.92970	0.93290
793	0.91520	0.92076	0.92329	0.92413	0.92685	0.92977	0.93296
794	0.91529	0.92084	0.92336	0.92421	0.92693	0.92984	0.93303
795	0.91537	0.92092	0.92344	0.92428	0.92700	0.92991	0.93310
796	0.91545	0.92100	0.92352	0.92436	0.92707	0.92998	0.93317
797	0.91553	0.92108	0.92360	0.92444	0.92715	0.93005	0.93323
798	0.91562	0.92116	0.92368	0.92452	0.92722	0.93012	0.93330
799	0.91572	0.92123	0.92376	0.92459	0.92729	0.93019	0.93336
800	0.91580	0.92132	0.92383	0.92467	0.92736	0.93026	0.93343
801	0.91589	0.92140	0.92391	0.92475	0.92744	0.93033	0.93350
802	0.91597	0.92148	0.92399	0.92482	0.92751	0.93040	0.93356
803	0.91606	0.92156	0.92406	0.92489	0.92758	0.93047	0.93363
804	0.91614	0.92164	0.92414	0.92497	0.92765	0.93054	0.93369
805	0.91623	0.92172	0.92422	0.92505	0.92772	0.93060	0.93376
806	0.91632	0.92180	0.92430	0.92512	0.92780	0.93067	0.93382
807	0.91641	0.92188	0.92437	0.92520	0.92787	0.93074	0.93389
808	0.91649	0.92196	0.92445	0.92527	0.92794	0.93081	0.93395
809	0.91657	0.92204	0.92452	0.92534	0.92801	0.93088	0.93402
810	0.91666	0.92211	0.92460	0.92542	0.92808	0.93095	0.93408
811	0.91674	0.92220	0.92467	0.92549	0.92815	0.93102	0.93415
812	0.91683	0.92227	0.92475	0.92557	0.92823	0.93108	0.93421
813	0.91691	0.92236	0.92482	0.92564	0.92830	0.93115	0.93428

N	0.1%	0.5%	1%	1.25%	2.5%	5%	10%
814	0.91699	0.92243	0.92490	0.92572	0.92837	0.93122	0.93434
815	0.91707	0.92251	0.92497	0.92579	0.92844	0.93129	0.93440
816	0.91715	0.92259	0.92505	0.92586	0.92851	0.93135	0.93447
817	0.91723	0.92267	0.92512	0.92593	0.92858	0.93142	0.93453
818	0.91731	0.92274	0.92519	0.92601	0.92865	0.93149	0.93459
819	0.91740	0.92282	0.92527	0.92608	0.92872	0.93155	0.93466
820	0.91749	0.92290	0.92534	0.92615	0.92879	0.93162	0.93472
821	0.91758	0.92297	0.92541	0.92622	0.92886	0.93169	0.93478
822	0.91766	0.92304	0.92549	0.92630	0.92893	0.93175	0.93485
823	0.91774	0.92312	0.92556	0.92637	0.92900	0.93182	0.93491
824	0.91782	0.92319	0.92563	0.92644	0.92907	0.93189	0.93497
825	0.91791	0.92327	0.92571	0.92651	0.92914	0.93195	0.93504
826	0.91798	0.92335	0.92578	0.92659	0.92921	0.93202	0.93510
827	0.91805	0.92342	0.92585	0.92665	0.92928	0.93208	0.93516
828	0.91814	0.92350	0.92592	0.92673	0.92935	0.93215	0.93522
829	0.91821	0.92357	0.92600	0.92680	0.92941	0.93222	0.93528
830	0.91831	0.92365	0.92607	0.92687	0.92948	0.93228	0.93535
831	0.91840	0.92372	0.92614	0.92694	0.92955	0.93234	0.93541
832	0.91847	0.92379	0.92621	0.92701	0.92961	0.93241	0.93547
833	0.91855	0.92387	0.92629	0.92709	0.92968	0.93248	0.93553
834	0.91864	0.92394	0.92636	0.92716	0.92975	0.93254	0.93559
835	0.91872	0.92401	0.92643	0.92723	0.92982	0.93261	0.93565
836	0.91880	0.92409	0.92650	0.92730	0.92988	0.93267	0.93571
837	0.91888	0.92416	0.92657	0.92737	0.92995	0.93273	0.93578
838	0.91896	0.92424	0.92664	0.92744	0.93002	0.93280	0.93584
839	0.91905	0.92431	0.92671	0.92751	0.93009	0.93286	0.93590
840	0.91913	0.92439	0.92679	0.92758	0.93016	0.93293	0.93596
841	0.91920	0.92445	0.92686	0.92765	0.93022	0.93299	0.93602
842	0.91928	0.92453	0.92693	0.92772	0.93029	0.93306	0.93608
843	0.91936	0.92460	0.92700	0.92779	0.93036	0.93312	0.93614
844	0.91945	0.92467	0.92707	0.92786	0.93042	0.93318	0.93620
845	0.91953	0.92475	0.92713	0.92793	0.93049	0.93325	0.93626
846	0.91960	0.92482	0.92721	0.92800	0.93056	0.93331	0.93632
847	0.91967	0.92489	0.92728	0.92807	0.93062	0.93337	0.93638
848	0.91975	0.92496	0.92734	0.92813	0.93069	0.93343	0.93644
849	0.91982	0.92503	0.92741	0.92820	0.93076	0.93350	0.93650
850	0.91990	0.92510	0.92748	0.92827	0.93082	0.93356	0.93656
851	0.91998	0.92517	0.92755	0.92834	0.93089	0.93362	0.93662
852	0.92007	0.92525	0.92762	0.92841	0.93095	0.93368	0.93668
853	0.92014	0.92532	0.92769	0.92848	0.93102	0.93375	0.93674
854	0.92022	0.92539	0.92776	0.92855	0.93108	0.93381	0.93679
855	0.92029	0.92546	0.92783	0.92861	0.93115	0.93387	0.93685
856	0.92036	0.92553	0.92790	0.92868	0.93121	0.93393	0.93691
857	0.92043	0.92560	0.92796	0.92875	0.93128	0.93399	0.93697
858	0.92050	0.92568	0.92803	0.92882	0.93134	0.93406	0.93703
859	0.92059	0.92575	0.92810	0.92889	0.93141	0.93412	0.93709
860	0.92065	0.92582	0.92817	0.92895	0.93147	0.93418	0.93715
861	0.92073	0.92589	0.92824	0.92902	0.93154	0.93424	0.93720
862	0.92081	0.92596	0.92831	0.92909	0.93160	0.93430	0.93726
863	0.92089	0.92603	0.92837	0.92915	0.93166	0.93436	0.93732
864	0.92096	0.92610	0.92844	0.92922	0.93172	0.93442	0.93738
865	0.92102	0.92617	0.92851	0.92928	0.93179	0.93449	0.93744
866	0.92111	0.92624	0.92858	0.92935	0.93185	0.93455	0.93750
867	0.92118	0.92631	0.92864	0.92942	0.93191	0.93461	0.93755
868	0.92125	0.92638	0.92871	0.92949	0.93198	0.93467	0.93761
869	0.92133	0.92644	0.92877	0.92955	0.93204	0.93473	0.93767
870	0.92141	0.92651	0.92884	0.92962	0.93211	0.93479	0.93772
871	0.92148	0.92658	0.92891	0.92968	0.93217	0.93485	0.93778
872	0.92155	0.92665	0.92897	0.92975	0.93223	0.93491	0.93784
873	0.92162	0.92672	0.92904	0.92981	0.93229	0.93497	0.93789
874	0.92170	0.92679	0.92910	0.92988	0.93236	0.93503	0.93795
875	0.92177	0.92686	0.92917	0.92994	0.93242	0.93509	0.93801
876	0.92184	0.92693	0.92923	0.93001	0.93248	0.93515	0.93806
877	0.92192	0.92699	0.92930	0.93007	0.93255	0.93521	0.93812
878	0.92199	0.92706	0.92937	0.93013	0.93261	0.93526	0.93818
879	0.92207	0.92712	0.92943	0.93020	0.93267	0.93532	0.93823
880	0.92213	0.92719	0.92950	0.93027	0.93273	0.93538	0.93829
881	0.92221	0.92727	0.92956	0.93033	0.93280	0.93544	0.93834
882	0.92227	0.92733	0.92962	0.93039	0.93286	0.93550	0.93840
883	0.92235	0.92740	0.92969	0.93046	0.93292	0.93556	0.93846
884	0.92242	0.92747	0.92976	0.93052	0.93298	0.93562	0.93851
885	0.92249	0.92754	0.92982	0.93059	0.93304	0.93567	0.93857
886	0.92257	0.92760	0.92989	0.93065	0.93310	0.93573	0.93862
887	0.92264	0.92767	0.92995	0.93071	0.93316	0.93579	0.93868

N	0.1%	0.5%	1%	1.25%	2.5%	5%	10%
888	0.92272	0.92774	0.93002	0.93078	0.93322	0.93585	0.93873
889	0.92278	0.92781	0.93008	0.93084	0.93328	0.93591	0.93879
890	0.92286	0.92787	0.93014	0.93091	0.93335	0.93597	0.93884
891	0.92293	0.92794	0.93021	0.93097	0.93340	0.93603	0.93890
892	0.92300	0.92801	0.93027	0.93103	0.93347	0.93608	0.93895
893	0.92306	0.92807	0.93033	0.93109	0.93353	0.93614	0.93901
894	0.92313	0.92814	0.93040	0.93115	0.93358	0.93620	0.93906
895	0.92320	0.92820	0.93046	0.93122	0.93364	0.93625	0.93912
896	0.92327	0.92827	0.93052	0.93128	0.93370	0.93631	0.93917
897	0.92335	0.92834	0.93059	0.93134	0.93376	0.93637	0.93923
898	0.92342	0.92840	0.93065	0.93140	0.93382	0.93642	0.93928
899	0.92349	0.92846	0.93071	0.93146	0.93388	0.93648	0.93933
900	0.92356	0.92853	0.93078	0.93152	0.93394	0.93654	0.93939
901	0.92364	0.92859	0.93084	0.93158	0.93400	0.93660	0.93944
902	0.92370	0.92866	0.93090	0.93165	0.93406	0.93665	0.93949
903	0.92377	0.92872	0.93096	0.93171	0.93412	0.93671	0.93955
904	0.92384	0.92879	0.93103	0.93177	0.93418	0.93676	0.93960
905	0.92391	0.92885	0.93109	0.93183	0.93424	0.93682	0.93965
906	0.92398	0.92892	0.93115	0.93190	0.93430	0.93688	0.93971
907	0.92405	0.92898	0.93121	0.93196	0.93436	0.93693	0.93976
908	0.92412	0.92904	0.93127	0.93202	0.93441	0.93699	0.93981
909	0.92418	0.92911	0.93133	0.93208	0.93447	0.93704	0.93987
910	0.92425	0.92917	0.93140	0.93214	0.93453	0.93710	0.93992
911	0.92432	0.92923	0.93146	0.93220	0.93459	0.93716	0.93997
912	0.92439	0.92930	0.93152	0.93226	0.93465	0.93721	0.94002
913	0.92446	0.92936	0.93158	0.93232	0.93471	0.93726	0.94008
914	0.92453	0.92942	0.93164	0.93238	0.93476	0.93732	0.94013
915	0.92460	0.92948	0.93170	0.93244	0.93482	0.93738	0.94018
916	0.92467	0.92955	0.93176	0.93250	0.93488	0.93743	0.94023
917	0.92474	0.92961	0.93182	0.93256	0.93493	0.93749	0.94029
918	0.92480	0.92967	0.93188	0.93262	0.93499	0.93754	0.94034
919	0.92487	0.92973	0.93194	0.93267	0.93505	0.93760	0.94039
920	0.92494	0.92980	0.93200	0.93273	0.93511	0.93765	0.94044
921	0.92501	0.92986	0.93206	0.93280	0.93516	0.93770	0.94049
922	0.92508	0.92992	0.93212	0.93286	0.93522	0.93776	0.94054
923	0.92515	0.92998	0.93218	0.93291	0.93528	0.93781	0.94059
924	0.92521	0.93004	0.93224	0.93297	0.93534	0.93787	0.94065
925	0.92528	0.93011	0.93230	0.93303	0.93539	0.93792	0.94070
926	0.92535	0.93017	0.93236	0.93309	0.93545	0.93798	0.94075
927	0.92541	0.93023	0.93242	0.93315	0.93550	0.93803	0.94080
928	0.92548	0.93030	0.93248	0.93321	0.93556	0.93808	0.94085
929	0.92554	0.93035	0.93254	0.93327	0.93562	0.93814	0.94090
930	0.92561	0.93042	0.93260	0.93332	0.93567	0.93819	0.94095
931	0.92568	0.93048	0.93266	0.93338	0.93573	0.93824	0.94100
932	0.92574	0.93054	0.93272	0.93344	0.93578	0.93830	0.94105
933	0.92581	0.93060	0.93278	0.93350	0.93584	0.93835	0.94110
934	0.92589	0.93066	0.93283	0.93356	0.93590	0.93840	0.94115
935	0.92594	0.93072	0.93290	0.93361	0.93595	0.93845	0.94120
936	0.92601	0.93078	0.93296	0.93367	0.93601	0.93851	0.94125
937	0.92608	0.93084	0.93301	0.93373	0.93606	0.93856	0.94130
938	0.92614	0.93090	0.93307	0.93379	0.93612	0.93861	0.94135
939	0.92620	0.93096	0.93313	0.93384	0.93617	0.93866	0.94140
940	0.92627	0.93102	0.93319	0.93390	0.93623	0.93872	0.94145
941	0.92634	0.93108	0.93324	0.93396	0.93628	0.93877	0.94150
942	0.92640	0.93114	0.93330	0.93402	0.93634	0.93882	0.94155
943	0.92646	0.93120	0.93336	0.93407	0.93639	0.93887	0.94160
944	0.92653	0.93126	0.93342	0.93413	0.93645	0.93893	0.94165
945	0.92660	0.93132	0.93348	0.93419	0.93650	0.93898	0.94170
946	0.92666	0.93138	0.93353	0.93424	0.93656	0.93903	0.94175
947	0.92672	0.93143	0.93359	0.93430	0.93661	0.93908	0.94180
948	0.92678	0.93150	0.93365	0.93436	0.93666	0.93913	0.94185
949	0.92685	0.93156	0.93371	0.93441	0.93672	0.93918	0.94190
950	0.92691	0.93161	0.93376	0.93447	0.93677	0.93924	0.94194
951	0.92697	0.93167	0.93382	0.93452	0.93683	0.93929	0.94199
952	0.92705	0.93173	0.93387	0.93458	0.93688	0.93934	0.94204
953	0.92710	0.93179	0.93393	0.93464	0.93694	0.93939	0.94209
954	0.92716	0.93185	0.93399	0.93469	0.93699	0.93944	0.94214
955	0.92723	0.93191	0.93404	0.93475	0.93704	0.93949	0.94219
956	0.92729	0.93196	0.93410	0.93480	0.93710	0.93954	0.94224
957	0.92736	0.93202	0.93416	0.93486	0.93715	0.93960	0.94228
958	0.92742	0.93208	0.93421	0.93491	0.93720	0.93965	0.94233
959	0.92748	0.93214	0.93427	0.93497	0.93726	0.93970	0.94238
960	0.92754	0.93221	0.93432	0.93503	0.93731	0.93975	0.94243
961	0.92760	0.93226	0.93438	0.93508	0.93736	0.93980	0.94248

N	0.1%	0.5%	1%	1.25%	2.5%	5%	10%
962	0.92766	0.93232	0.93444	0.93514	0.93741	0.93985	0.94252
963	0.92773	0.93238	0.93449	0.93519	0.93747	0.93990	0.94257
964	0.92778	0.93243	0.93455	0.93525	0.93752	0.93995	0.94262
965	0.92785	0.93249	0.93461	0.93530	0.93757	0.94000	0.94267
966	0.92791	0.93255	0.93466	0.93536	0.93763	0.94005	0.94271
967	0.92797	0.93261	0.93472	0.93541	0.93768	0.94010	0.94276
968	0.92803	0.93266	0.93477	0.93547	0.93773	0.94015	0.94281
969	0.92809	0.93272	0.93482	0.93552	0.93778	0.94020	0.94286
970	0.92816	0.93278	0.93488	0.93557	0.93783	0.94025	0.94290
971	0.92822	0.93283	0.93493	0.93563	0.93789	0.94030	0.94295
972	0.92828	0.93289	0.93499	0.93568	0.93794	0.94035	0.94300
973	0.92834	0.93295	0.93504	0.93574	0.93799	0.94040	0.94304
974	0.92840	0.93300	0.93510	0.93579	0.93804	0.94045	0.94309
975	0.92846	0.93306	0.93515	0.93584	0.93809	0.94050	0.94314
976	0.92853	0.93311	0.93521	0.93590	0.93814	0.94055	0.94318
977	0.92859	0.93317	0.93526	0.93595	0.93819	0.94059	0.94323
978	0.92865	0.93323	0.93531	0.93600	0.93825	0.94064	0.94328
979	0.92871	0.93328	0.93537	0.93606	0.93830	0.94069	0.94332
980	0.92877	0.93334	0.93542	0.93611	0.93835	0.94074	0.94337
981	0.92883	0.93339	0.93547	0.93616	0.93840	0.94079	0.94341
982	0.92888	0.93345	0.93552	0.93621	0.93845	0.94084	0.94346
983	0.92895	0.93350	0.93558	0.93627	0.93850	0.94089	0.94351
984	0.92900	0.93356	0.93563	0.93632	0.93855	0.94093	0.94355
985	0.92907	0.93362	0.93569	0.93637	0.93860	0.94098	0.94360
986	0.92914	0.93367	0.93574	0.93643	0.93866	0.94103	0.94364
987	0.92919	0.93373	0.93579	0.93648	0.93871	0.94108	0.94369
988	0.92925	0.93378	0.93585	0.93653	0.93876	0.94113	0.94374
989	0.92931	0.93384	0.93590	0.93659	0.93881	0.94118	0.94378
990	0.92937	0.93389	0.93595	0.93664	0.93886	0.94123	0.94383
991	0.92943	0.93395	0.93601	0.93669	0.93890	0.94127	0.94387
992	0.92949	0.93400	0.93606	0.93674	0.93896	0.94132	0.94392
993	0.92955	0.93405	0.93611	0.93680	0.93901	0.94137	0.94396
994	0.92960	0.93411	0.93616	0.93685	0.93906	0.94142	0.94401
995	0.92966	0.93416	0.93622	0.93690	0.93911	0.94146	0.94405
996	0.92972	0.93422	0.93627	0.93695	0.93916	0.94151	0.94410
997	0.92978	0.93427	0.93632	0.93700	0.93921	0.94156	0.94414
998	0.92984	0.93432	0.93637	0.93705	0.93926	0.94161	0.94419
999	0.92990	0.93438	0.93642	0.93710	0.93930	0.94165	0.94423
1000	0.92995	0.93443	0.93647	0.93716	0.93935	0.94170	0.94428

Table 2 – *GM6(5H1L or 1H5L)*. Critical values of upper significance levels for the Grubbs multiple-6 outlier test, applicable in situations where the five highest and one lowest or one highest and five lowest observations are simultaneously identified as outliers, denoted as *GM6(5H1L)* and *GM6(1H5L)*, respectively.

N	0.1%	0.5%	1%	1.25%	2.5%	5%	10%
8	0.00000	0.00000	0.00000	0.00000	0.00001	0.00005	0.00019
9	0.00003	0.00015	0.00031	0.00038	0.00079	0.00166	0.00357
10	0.00042	0.00131	0.00213	0.00250	0.00414	0.00696	0.01198
11	0.00183	0.00432	0.00631	0.00714	0.01056	0.01584	0.02428
12	0.00465	0.00940	0.01284	0.01423	0.01965	0.02746	0.03911
13	0.00903	0.01640	0.02141	0.02335	0.03074	0.04097	0.05550
14	0.01484	0.02502	0.03154	0.03405	0.04338	0.05573	0.07269
15	0.02209	0.03494	0.04289	0.04590	0.05691	0.07115	0.09029
16	0.03015	0.04573	0.05508	0.05854	0.07109	0.08699	0.10787
17	0.03935	0.05726	0.06786	0.07172	0.08560	0.10294	0.12536
18	0.04904	0.06936	0.08105	0.08530	0.10029	0.11890	0.14252
19	0.05954	0.08187	0.09440	0.09896	0.11502	0.13461	0.15926
20	0.07019	0.09430	0.10786	0.11268	0.12960	0.15006	0.17553
21	0.08091	0.10691	0.12119	0.12627	0.14397	0.16515	0.19129
22	0.09200	0.11946	0.13441	0.13973	0.15807	0.17987	0.20656
23	0.10307	0.13200	0.14753	0.15303	0.17186	0.19414	0.22129
24	0.11444	0.14434	0.16039	0.16606	0.18537	0.20808	0.23556
25	0.12559	0.15662	0.17313	0.17890	0.19853	0.22156	0.24929
26	0.13665	0.16870	0.18549	0.19129	0.21133	0.23468	0.26256
27	0.14756	0.18054	0.19751	0.20350	0.22373	0.24734	0.27537
28	0.15862	0.19209	0.20930	0.21534	0.23581	0.25958	0.28774
29	0.16945	0.20325	0.22079	0.22689	0.24757	0.27146	0.29965
30	0.17981	0.21427	0.23195	0.23810	0.25900	0.28295	0.31114
31	0.19019	0.22502	0.24283	0.24907	0.27009	0.29409	0.32224
32	0.20033	0.23565	0.25353	0.25976	0.28090	0.30489	0.33299
33	0.21033	0.24590	0.26405	0.27029	0.29139	0.31537	0.34338

N	0.1%	0.5%	1%	1.25%	2.5%	5%	10%
34	0.22008	0.25593	0.27412	0.28048	0.30153	0.32555	0.35345
35	0.22975	0.26582	0.28403	0.29040	0.31143	0.33535	0.36314
36	0.23879	0.27548	0.29371	0.30002	0.32104	0.34488	0.37251
37	0.24797	0.28479	0.30312	0.30938	0.33040	0.35412	0.38159
38	0.25682	0.29392	0.31216	0.31847	0.33938	0.36307	0.39035
39	0.26562	0.30270	0.32099	0.32725	0.34822	0.37177	0.39889
40	0.27406	0.31126	0.32962	0.33594	0.35677	0.38021	0.40716
41	0.28254	0.31978	0.33811	0.34434	0.36513	0.38844	0.41515
42	0.29066	0.32797	0.34629	0.35255	0.37321	0.39639	0.42295
43	0.29895	0.33601	0.35423	0.36046	0.38109	0.40415	0.43052
44	0.30682	0.34387	0.36201	0.36821	0.38873	0.41168	0.43782
45	0.31435	0.35150	0.36963	0.37584	0.39616	0.41896	0.44496
46	0.32204	0.35895	0.37695	0.38312	0.40342	0.42608	0.45187
47	0.32961	0.36622	0.38419	0.39025	0.41046	0.43300	0.45862
48	0.33684	0.37334	0.39118	0.39722	0.41739	0.43973	0.46516
49	0.34396	0.38030	0.39801	0.40402	0.42407	0.44630	0.47154
50	0.35087	0.38714	0.40475	0.41078	0.43065	0.45271	0.47773
51	0.35757	0.39381	0.41133	0.41730	0.43704	0.45895	0.48375
52	0.36443	0.40028	0.41776	0.42369	0.44330	0.46505	0.48964
53	0.37085	0.40664	0.42395	0.42985	0.44934	0.47095	0.49537
54	0.37714	0.41277	0.43007	0.43596	0.45531	0.47678	0.50097
55	0.38320	0.41889	0.43604	0.44187	0.46111	0.48243	0.50645
56	0.38918	0.42486	0.44188	0.44761	0.46678	0.48794	0.51173
57	0.39505	0.43061	0.44760	0.45331	0.47231	0.49331	0.51691
58	0.40105	0.43633	0.45322	0.45888	0.47772	0.49856	0.52198
59	0.40692	0.44192	0.45865	0.46430	0.48297	0.50367	0.52694
60	0.41243	0.44729	0.46396	0.46961	0.48818	0.50872	0.53177
61	0.41803	0.45274	0.46920	0.47483	0.49326	0.51361	0.53649
62	0.42343	0.45783	0.47429	0.47986	0.49818	0.51843	0.54109
63	0.42865	0.46287	0.47930	0.48488	0.50303	0.52311	0.54559
64	0.43370	0.46781	0.48415	0.48966	0.50778	0.52770	0.55002
65	0.43881	0.47276	0.48892	0.49442	0.51244	0.53216	0.55431
66	0.44385	0.47753	0.49363	0.49911	0.51699	0.53656	0.55852
67	0.44890	0.48228	0.49830	0.50369	0.52142	0.54086	0.56265
68	0.45364	0.48691	0.50285	0.50817	0.52577	0.54507	0.56671
69	0.45832	0.49145	0.50723	0.51256	0.53002	0.54922	0.57068
70	0.46291	0.49593	0.51160	0.51690	0.53424	0.55326	0.57455
71	0.46758	0.50030	0.51586	0.52114	0.53831	0.55722	0.57834
72	0.47201	0.50458	0.52007	0.52527	0.54234	0.56110	0.58205
73	0.47612	0.50877	0.52415	0.52933	0.54631	0.56489	0.58570
74	0.48040	0.51287	0.52815	0.53333	0.55019	0.56861	0.58927
75	0.48448	0.51692	0.53211	0.53722	0.55402	0.57229	0.59278
76	0.48873	0.52086	0.53592	0.54100	0.55772	0.57587	0.59622
77	0.49286	0.52478	0.53971	0.54481	0.56138	0.57940	0.59959
78	0.49708	0.52866	0.54348	0.54853	0.56500	0.58287	0.60289
79	0.50098	0.53240	0.54717	0.55215	0.56854	0.58628	0.60614
80	0.50491	0.53604	0.55077	0.55574	0.57195	0.58960	0.60934
81	0.50863	0.53963	0.55427	0.55925	0.57533	0.59288	0.61247
82	0.51246	0.54315	0.55777	0.56269	0.57869	0.59609	0.61555
83	0.51601	0.54668	0.56115	0.56602	0.58198	0.59925	0.61856
84	0.51966	0.55015	0.56454	0.56938	0.58521	0.60237	0.62154
85	0.52313	0.55349	0.56782	0.57267	0.58839	0.60541	0.62446
86	0.52663	0.55684	0.57110	0.57593	0.59150	0.60843	0.62733
87	0.52992	0.56012	0.57428	0.57906	0.59455	0.61137	0.63013
88	0.53358	0.56338	0.57746	0.58219	0.59755	0.61427	0.63291
89	0.53686	0.56657	0.58055	0.58522	0.60053	0.61712	0.63562
90	0.54006	0.56974	0.58356	0.58827	0.60341	0.61992	0.63830
91	0.54334	0.57282	0.58654	0.59126	0.60629	0.62271	0.64094
92	0.54669	0.57586	0.58951	0.59415	0.60912	0.62543	0.64351
93	0.54981	0.57883	0.59244	0.59700	0.61188	0.62809	0.64605
94	0.55289	0.58183	0.59527	0.59981	0.61463	0.63072	0.64854
95	0.55598	0.58472	0.59808	0.60262	0.61731	0.63331	0.65102
96	0.55901	0.58754	0.60089	0.60536	0.61997	0.63586	0.65346
97	0.56196	0.59033	0.60356	0.60805	0.62258	0.63837	0.65585
98	0.56496	0.59304	0.60622	0.61070	0.62517	0.64084	0.65820
99	0.56771	0.59577	0.60889	0.61333	0.62768	0.64326	0.66052
100	0.57057	0.59844	0.61151	0.61588	0.63015	0.64565	0.66280
101	0.57337	0.60108	0.61409	0.61845	0.63261	0.64802	0.66504
102	0.57611	0.60369	0.61662	0.62095	0.63502	0.65032	0.66725
103	0.57872	0.60624	0.61912	0.62344	0.63740	0.65261	0.66944
104	0.58143	0.60875	0.62155	0.62590	0.63976	0.65486	0.67158
105	0.58408	0.61127	0.62401	0.62828	0.64209	0.65708	0.67369
106	0.58671	0.61375	0.62640	0.63063	0.64435	0.65927	0.67578
107	0.58934	0.61619	0.62874	0.63299	0.64662	0.66145	0.67782

N	0.1%	0.5%	1%	1.25%	2.5%	5%	10%
108	0.59181	0.61861	0.63109	0.63529	0.64886	0.66356	0.67986
109	0.59447	0.62098	0.63338	0.63754	0.65104	0.66566	0.68184
110	0.59699	0.62329	0.63563	0.63976	0.65320	0.66772	0.68381
111	0.59938	0.62553	0.63784	0.64196	0.65532	0.66976	0.68576
112	0.60182	0.62778	0.64003	0.64412	0.65741	0.67178	0.68767
113	0.60416	0.63002	0.64221	0.64625	0.65948	0.67377	0.68957
114	0.60661	0.63223	0.64433	0.64836	0.66153	0.67573	0.69143
115	0.60889	0.63440	0.64642	0.65044	0.66352	0.67765	0.69327
116	0.61118	0.63652	0.64852	0.65251	0.66553	0.67955	0.69507
117	0.61346	0.63867	0.65057	0.65453	0.66749	0.68143	0.69686
118	0.61571	0.64074	0.65261	0.65653	0.66944	0.68328	0.69862
119	0.61794	0.64283	0.65456	0.65853	0.67134	0.68512	0.70038
120	0.62007	0.64486	0.65658	0.66052	0.67323	0.68690	0.70210
121	0.62217	0.64687	0.65851	0.66245	0.67509	0.68870	0.70380
122	0.62431	0.64886	0.66046	0.66435	0.67692	0.69047	0.70547
123	0.62634	0.65084	0.66236	0.66620	0.67873	0.69221	0.70711
124	0.62849	0.65282	0.66423	0.66806	0.68054	0.69392	0.70874
125	0.63055	0.65471	0.66605	0.66987	0.68230	0.69563	0.71036
126	0.63253	0.65656	0.66790	0.67171	0.68407	0.69731	0.71196
127	0.63455	0.65846	0.66970	0.67351	0.68577	0.69897	0.71354
128	0.63657	0.66035	0.67150	0.67529	0.68748	0.70061	0.71509
129	0.63846	0.66213	0.67326	0.67705	0.68918	0.70222	0.71663
130	0.64038	0.66396	0.67500	0.67877	0.69084	0.70383	0.71814
131	0.64227	0.66575	0.67674	0.68047	0.69248	0.70541	0.71965
132	0.64419	0.66748	0.67847	0.68212	0.69410	0.70696	0.72114
133	0.64607	0.66925	0.68014	0.68380	0.69571	0.70850	0.72260
134	0.64790	0.67101	0.68180	0.68546	0.69731	0.71002	0.72404
135	0.64971	0.67270	0.68346	0.68708	0.69887	0.71154	0.72549
136	0.65151	0.67436	0.68508	0.68868	0.70044	0.71303	0.72690
137	0.65326	0.67605	0.68671	0.69029	0.70194	0.71450	0.72829
138	0.65503	0.67772	0.68827	0.69188	0.70346	0.71595	0.72967
139	0.65680	0.67931	0.68986	0.69342	0.70498	0.71737	0.73103
140	0.65854	0.68094	0.69142	0.69498	0.70646	0.71879	0.73237
141	0.66019	0.68254	0.69299	0.69649	0.70793	0.72020	0.73371
142	0.66187	0.68409	0.69451	0.69802	0.70938	0.72158	0.73502
143	0.66356	0.68566	0.69603	0.69951	0.71081	0.72296	0.73633
144	0.66524	0.68719	0.69752	0.70100	0.71223	0.72431	0.73761
145	0.66686	0.68873	0.69905	0.70247	0.71363	0.72567	0.73889
146	0.66851	0.69026	0.70050	0.70392	0.71503	0.72701	0.74016
147	0.67011	0.69177	0.70195	0.70535	0.71639	0.72833	0.74141
148	0.67175	0.69322	0.70337	0.70675	0.71775	0.72962	0.74263
149	0.67324	0.69468	0.70480	0.70816	0.71909	0.73090	0.74384
150	0.67481	0.69613	0.70619	0.70956	0.72044	0.73217	0.74505
151	0.67633	0.69756	0.70757	0.71092	0.72175	0.73344	0.74625
152	0.67777	0.69899	0.70898	0.71228	0.72306	0.73468	0.74743
153	0.67927	0.70041	0.71032	0.71359	0.72434	0.73591	0.74860
154	0.68076	0.70180	0.71166	0.71492	0.72562	0.73714	0.74977
155	0.68229	0.70319	0.71301	0.71627	0.72687	0.73835	0.75091
156	0.68376	0.70455	0.71431	0.71756	0.72813	0.73955	0.75205
157	0.68514	0.70587	0.71560	0.71886	0.72938	0.74073	0.75317
158	0.68666	0.70722	0.71687	0.72011	0.73062	0.74188	0.75428
159	0.68811	0.70852	0.71816	0.72139	0.73182	0.74305	0.75539
160	0.68952	0.70982	0.71944	0.72263	0.73302	0.74419	0.75648
161	0.69092	0.71112	0.72070	0.72386	0.73421	0.74534	0.75756
162	0.69223	0.71241	0.72192	0.72510	0.73540	0.74647	0.75863
163	0.69358	0.71365	0.72315	0.72630	0.73654	0.74757	0.75969
164	0.69494	0.71492	0.72436	0.72754	0.73770	0.74868	0.76073
165	0.69629	0.71618	0.72558	0.72871	0.73885	0.74978	0.76178
166	0.69753	0.71742	0.72677	0.72990	0.73998	0.75086	0.76281
167	0.69883	0.71862	0.72793	0.73105	0.74111	0.75194	0.76382
168	0.70016	0.71983	0.72910	0.73220	0.74222	0.75300	0.76484
169	0.70151	0.72107	0.73027	0.73333	0.74333	0.75404	0.76584
170	0.70271	0.72224	0.73143	0.73446	0.74442	0.75510	0.76683
171	0.70391	0.72343	0.73256	0.73558	0.74550	0.75611	0.76781
172	0.70520	0.72460	0.73368	0.73669	0.74656	0.75713	0.76878
173	0.70641	0.72576	0.73481	0.73782	0.74761	0.75816	0.76974
174	0.70761	0.72688	0.73591	0.73892	0.74867	0.75916	0.77069
175	0.70889	0.72801	0.73702	0.74001	0.74970	0.76016	0.77163
176	0.70996	0.72913	0.73810	0.74106	0.75072	0.76114	0.77257
177	0.71116	0.73028	0.73917	0.74212	0.75174	0.76212	0.77350
178	0.71231	0.73137	0.74025	0.74317	0.75277	0.76309	0.77442
179	0.71350	0.73247	0.74129	0.74423	0.75376	0.76404	0.77532
180	0.71470	0.73354	0.74232	0.74527	0.75476	0.76500	0.77623
181	0.71587	0.73459	0.74338	0.74627	0.75575	0.76594	0.77712

N	0.1%	0.5%	1%	1.25%	2.5%	5%	10%
182	0.71703	0.73567	0.74441	0.74731	0.75674	0.76687	0.77801
183	0.71815	0.73672	0.74543	0.74833	0.75770	0.76779	0.77889
184	0.71927	0.73775	0.74643	0.74931	0.75866	0.76870	0.77976
185	0.72042	0.73878	0.74742	0.75030	0.75959	0.76962	0.78062
186	0.72147	0.73983	0.74843	0.75128	0.76053	0.77052	0.78147
187	0.72250	0.74082	0.74939	0.75225	0.76148	0.77142	0.78231
188	0.72356	0.74183	0.75037	0.75322	0.76239	0.77230	0.78315
189	0.72463	0.74285	0.75134	0.75417	0.76330	0.77317	0.78399
190	0.72572	0.74384	0.75230	0.75512	0.76421	0.77404	0.78481
191	0.72676	0.74482	0.75326	0.75607	0.76513	0.77490	0.78563
192	0.72778	0.74579	0.75419	0.75699	0.76602	0.77576	0.78644
193	0.72877	0.74678	0.75513	0.75792	0.76691	0.77661	0.78724
194	0.72979	0.74772	0.75605	0.75883	0.76779	0.77745	0.78804
195	0.73085	0.74870	0.75696	0.75973	0.76864	0.77828	0.78884
196	0.73185	0.74963	0.75789	0.76064	0.76952	0.77912	0.78962
197	0.73286	0.75057	0.75878	0.76153	0.77037	0.77993	0.79041
198	0.73385	0.75150	0.75968	0.76240	0.77122	0.78073	0.79117
199	0.73478	0.75243	0.76057	0.76327	0.77207	0.78155	0.79194
200	0.73575	0.75333	0.76145	0.76416	0.77291	0.78235	0.79269
201	0.73671	0.75425	0.76231	0.76503	0.77375	0.78313	0.79345
202	0.73776	0.75513	0.76318	0.76589	0.77458	0.78392	0.79420
203	0.73877	0.75604	0.76406	0.76673	0.77540	0.78471	0.79494
204	0.73970	0.75694	0.76491	0.76757	0.77620	0.78548	0.79568
205	0.74067	0.75780	0.76575	0.76840	0.77701	0.78626	0.79640
206	0.74157	0.75867	0.76659	0.76922	0.77781	0.78701	0.79713
207	0.74243	0.75954	0.76744	0.77005	0.77859	0.78778	0.79785
208	0.74344	0.76039	0.76826	0.77087	0.77938	0.78852	0.79856
209	0.74432	0.76124	0.76909	0.77169	0.78016	0.78927	0.79926
210	0.74525	0.76208	0.76989	0.77249	0.78093	0.79001	0.79997
211	0.74618	0.76289	0.77070	0.77329	0.78170	0.79074	0.80066
212	0.74711	0.76372	0.77150	0.77409	0.78247	0.79147	0.80135
213	0.74797	0.76455	0.77230	0.77486	0.78322	0.79219	0.80204
214	0.74883	0.76537	0.77308	0.77566	0.78397	0.79290	0.80272
215	0.74967	0.76618	0.77386	0.77643	0.78471	0.79363	0.80339
216	0.75055	0.76700	0.77465	0.77719	0.78544	0.79434	0.80406
217	0.75135	0.76781	0.77542	0.77796	0.78617	0.79503	0.80472
218	0.75219	0.76860	0.77619	0.77871	0.78691	0.79573	0.80538
219	0.75302	0.76938	0.77695	0.77947	0.78763	0.79642	0.80604
220	0.75385	0.77017	0.77772	0.78021	0.78835	0.79710	0.80669
221	0.75472	0.77094	0.77847	0.78095	0.78906	0.79777	0.80733
222	0.75556	0.77173	0.77922	0.78169	0.78976	0.79845	0.80798
223	0.75640	0.77250	0.77995	0.78241	0.79045	0.79912	0.80861
224	0.75715	0.77327	0.78067	0.78313	0.79114	0.79979	0.80924
225	0.75794	0.77402	0.78139	0.78386	0.79183	0.80044	0.80986
226	0.75876	0.77476	0.78210	0.78458	0.79251	0.80110	0.81048
227	0.75952	0.77552	0.78282	0.78529	0.79321	0.80175	0.81110
228	0.76028	0.77624	0.78355	0.78599	0.79387	0.80239	0.81171
229	0.76105	0.77700	0.78426	0.78667	0.79454	0.80304	0.81232
230	0.76185	0.77770	0.78496	0.78738	0.79521	0.80368	0.81292
231	0.76261	0.77842	0.78565	0.78806	0.79587	0.80431	0.81352
232	0.76333	0.77913	0.78635	0.78875	0.79652	0.80493	0.81412
233	0.76412	0.77981	0.78705	0.78942	0.79717	0.80556	0.81471
234	0.76489	0.78052	0.78772	0.79010	0.79782	0.80617	0.81530
235	0.76563	0.78123	0.78842	0.79077	0.79845	0.80678	0.81588
236	0.76642	0.78192	0.78908	0.79145	0.79910	0.80739	0.81646
237	0.76712	0.78262	0.78975	0.79211	0.79974	0.80800	0.81703
238	0.76792	0.78331	0.79040	0.79275	0.80036	0.80860	0.81759
239	0.76863	0.78399	0.79106	0.79342	0.80100	0.80920	0.81816
240	0.76934	0.78466	0.79171	0.79406	0.80161	0.80978	0.81872
241	0.77006	0.78535	0.79236	0.79470	0.80223	0.81037	0.81928
242	0.77078	0.78604	0.79301	0.79534	0.80285	0.81095	0.81983
243	0.77149	0.78669	0.79364	0.79597	0.80345	0.81153	0.82039
244	0.77219	0.78734	0.79428	0.79659	0.80405	0.81210	0.82094
245	0.77292	0.78801	0.79491	0.79722	0.80466	0.81268	0.82148
246	0.77362	0.78866	0.79554	0.79783	0.80525	0.81324	0.82202
247	0.77437	0.78929	0.79618	0.79844	0.80584	0.81381	0.82255
248	0.77503	0.78994	0.79679	0.79906	0.80643	0.81437	0.82308
249	0.77569	0.79055	0.79740	0.79966	0.80701	0.81493	0.82360
250	0.77637	0.79119	0.79801	0.80026	0.80760	0.81548	0.82413
251	0.77701	0.79180	0.79862	0.80087	0.80818	0.81603	0.82465
252	0.77772	0.79244	0.79922	0.80147	0.80875	0.81658	0.82517
253	0.77837	0.79307	0.79983	0.80206	0.80931	0.81713	0.82568
254	0.77906	0.79367	0.80041	0.80266	0.80988	0.81766	0.82620
255	0.77973	0.79428	0.80099	0.80324	0.81044	0.81820	0.82671

N	0.1%	0.5%	1%	1.25%	2.5%	5%	10%
256	0.78034	0.79489	0.80159	0.80383	0.81101	0.81874	0.82721
257	0.78100	0.79548	0.80218	0.80441	0.81155	0.81926	0.82771
258	0.78163	0.79607	0.80276	0.80498	0.81210	0.81979	0.82821
259	0.78229	0.79667	0.80333	0.80553	0.81265	0.82032	0.82871
260	0.78293	0.79727	0.80390	0.80610	0.81318	0.82083	0.82921
261	0.78355	0.79785	0.80447	0.80667	0.81372	0.82135	0.82969
262	0.78421	0.79845	0.80503	0.80722	0.81426	0.82186	0.83018
263	0.78485	0.79904	0.80559	0.80777	0.81479	0.82237	0.83066
264	0.78548	0.79961	0.80614	0.80832	0.81532	0.82287	0.83113
265	0.78613	0.80018	0.80667	0.80886	0.81584	0.82337	0.83161
266	0.78673	0.80077	0.80723	0.80941	0.81636	0.82387	0.83209
267	0.78730	0.80134	0.80778	0.80995	0.81688	0.82437	0.83256
268	0.78791	0.80191	0.80832	0.81049	0.81740	0.82485	0.83303
269	0.78851	0.80245	0.80887	0.81102	0.81792	0.82534	0.83349
270	0.78914	0.80302	0.80941	0.81155	0.81843	0.82583	0.83395
271	0.78972	0.80357	0.80995	0.81209	0.81893	0.82632	0.83441
272	0.79030	0.80411	0.81049	0.81260	0.81945	0.82681	0.83487
273	0.79090	0.80465	0.81102	0.81313	0.81994	0.82728	0.83533
274	0.79150	0.80519	0.81154	0.81364	0.82044	0.82775	0.83578
275	0.79207	0.80573	0.81207	0.81417	0.82093	0.82823	0.83623
276	0.79267	0.80628	0.81259	0.81467	0.82142	0.82869	0.83667
277	0.79322	0.80681	0.81310	0.81518	0.82191	0.82917	0.83711
278	0.79380	0.80734	0.81362	0.81569	0.82239	0.82962	0.83755
279	0.79436	0.80787	0.81413	0.81620	0.82288	0.83009	0.83799
280	0.79492	0.80839	0.81463	0.81669	0.82335	0.83055	0.83842
281	0.79553	0.80888	0.81514	0.81719	0.82383	0.83100	0.83885
282	0.79611	0.80941	0.81563	0.81769	0.82431	0.83146	0.83929
283	0.79661	0.80994	0.81613	0.81819	0.82478	0.83191	0.83972
284	0.79716	0.81045	0.81663	0.81868	0.82524	0.83235	0.84014
285	0.79770	0.81097	0.81712	0.81916	0.82571	0.83279	0.84056
286	0.79825	0.81148	0.81763	0.81964	0.82617	0.83324	0.84098
287	0.79879	0.81198	0.81811	0.82012	0.82664	0.83368	0.84140
288	0.79934	0.81248	0.81857	0.82060	0.82710	0.83411	0.84181
289	0.79987	0.81298	0.81906	0.82107	0.82755	0.83455	0.84223
290	0.80039	0.81346	0.81954	0.82153	0.82800	0.83498	0.84264
291	0.80092	0.81397	0.82001	0.82201	0.82845	0.83541	0.84304
292	0.80148	0.81446	0.82049	0.82247	0.82889	0.83584	0.84345
293	0.80199	0.81493	0.82095	0.82294	0.82933	0.83627	0.84385
294	0.80249	0.81540	0.82140	0.82340	0.82978	0.83669	0.84425
295	0.80302	0.81588	0.82188	0.82387	0.83022	0.83711	0.84465
296	0.80351	0.81638	0.82233	0.82432	0.83066	0.83753	0.84504
297	0.80400	0.81686	0.82278	0.82477	0.83109	0.83794	0.84544
298	0.80452	0.81731	0.82324	0.82522	0.83153	0.83835	0.84583
299	0.80500	0.81779	0.82370	0.82566	0.83196	0.83877	0.84622
300	0.80551	0.81826	0.82415	0.82610	0.83239	0.83917	0.84661
301	0.80605	0.81870	0.82459	0.82654	0.83281	0.83958	0.84699
302	0.80654	0.81917	0.82504	0.82697	0.83324	0.83998	0.84738
303	0.80704	0.81962	0.82549	0.82740	0.83365	0.84037	0.84776
304	0.80751	0.82008	0.82592	0.82784	0.83407	0.84077	0.84814
305	0.80801	0.82053	0.82635	0.82828	0.83448	0.84117	0.84852
306	0.80843	0.82100	0.82678	0.82871	0.83491	0.84156	0.84889
307	0.80893	0.82144	0.82722	0.82914	0.83532	0.84195	0.84926
308	0.80942	0.82189	0.82764	0.82956	0.83573	0.84234	0.84963
309	0.80988	0.82234	0.82807	0.82999	0.83614	0.84274	0.85000
310	0.81034	0.82277	0.82849	0.83041	0.83654	0.84312	0.85037
311	0.81084	0.82322	0.82891	0.83083	0.83694	0.84351	0.85073
312	0.81131	0.82366	0.82934	0.83124	0.83734	0.84389	0.85110
313	0.81181	0.82409	0.82975	0.83166	0.83774	0.84427	0.85146
314	0.81226	0.82453	0.83016	0.83206	0.83814	0.84465	0.85182
315	0.81273	0.82496	0.83058	0.83247	0.83853	0.84503	0.85217
316	0.81316	0.82538	0.83098	0.83287	0.83893	0.84541	0.85253
317	0.81366	0.82581	0.83139	0.83329	0.83931	0.84578	0.85288
318	0.81408	0.82622	0.83180	0.83369	0.83970	0.84615	0.85323
319	0.81453	0.82665	0.83221	0.83409	0.84008	0.84652	0.85358
320	0.81497	0.82706	0.83262	0.83449	0.84047	0.84689	0.85393
321	0.81543	0.82749	0.83301	0.83488	0.84085	0.84725	0.85427
322	0.81588	0.82791	0.83341	0.83528	0.84123	0.84761	0.85461
323	0.81631	0.82833	0.83382	0.83568	0.84160	0.84797	0.85495
324	0.81677	0.82874	0.83421	0.83606	0.84198	0.84833	0.85529
325	0.81722	0.82915	0.83461	0.83645	0.84236	0.84868	0.85563
326	0.81767	0.82955	0.83500	0.83683	0.84272	0.84904	0.85597
327	0.81811	0.82994	0.83540	0.83723	0.84309	0.84939	0.85630
328	0.81853	0.83033	0.83579	0.83760	0.84346	0.84973	0.85664
329	0.81895	0.83073	0.83617	0.83798	0.84382	0.85008	0.85696

N	0.1%	0.5%	1%	1.25%	2.5%	5%	10%
330	0.81939	0.83113	0.83655	0.83836	0.84418	0.85043	0.85729
331	0.81978	0.83150	0.83693	0.83873	0.84455	0.85079	0.85762
332	0.82024	0.83190	0.83731	0.83910	0.84490	0.85113	0.85795
333	0.82067	0.83229	0.83768	0.83948	0.84527	0.85147	0.85827
334	0.82106	0.83269	0.83807	0.83985	0.84563	0.85181	0.85859
335	0.82147	0.83307	0.83844	0.84022	0.84598	0.85215	0.85891
336	0.82186	0.83346	0.83880	0.84059	0.84633	0.85249	0.85923
337	0.82230	0.83385	0.83918	0.84095	0.84669	0.85282	0.85956
338	0.82272	0.83422	0.83954	0.84131	0.84704	0.85315	0.85987
339	0.82314	0.83459	0.83991	0.84168	0.84738	0.85348	0.86019
340	0.82351	0.83496	0.84027	0.84204	0.84773	0.85381	0.86050
341	0.82389	0.83534	0.84064	0.84240	0.84807	0.85414	0.86081
342	0.82431	0.83571	0.84101	0.84276	0.84841	0.85446	0.86112
343	0.82472	0.83611	0.84136	0.84312	0.84875	0.85479	0.86143
344	0.82511	0.83648	0.84171	0.84348	0.84910	0.85512	0.86174
345	0.82552	0.83685	0.84206	0.84382	0.84944	0.85544	0.86204
346	0.82592	0.83722	0.84242	0.84417	0.84977	0.85576	0.86235
347	0.82630	0.83757	0.84278	0.84452	0.85011	0.85608	0.86265
348	0.82666	0.83792	0.84313	0.84486	0.85043	0.85640	0.86295
349	0.82706	0.83828	0.84349	0.84522	0.85077	0.85672	0.86325
350	0.82747	0.83865	0.84384	0.84556	0.85110	0.85703	0.86355
351	0.82784	0.83902	0.84418	0.84589	0.85143	0.85734	0.86385
352	0.82824	0.83937	0.84452	0.84623	0.85176	0.85765	0.86414
353	0.82862	0.83972	0.84487	0.84657	0.85208	0.85797	0.86444
354	0.82902	0.84008	0.84521	0.84691	0.85240	0.85828	0.86473
355	0.82938	0.84043	0.84554	0.84724	0.85272	0.85858	0.86502
356	0.82976	0.84079	0.84588	0.84757	0.85304	0.85889	0.86531
357	0.83013	0.84113	0.84622	0.84790	0.85336	0.85919	0.86560
358	0.83048	0.84147	0.84655	0.84824	0.85368	0.85950	0.86589
359	0.83086	0.84181	0.84689	0.84856	0.85399	0.85980	0.86618
360	0.83123	0.84215	0.84722	0.84889	0.85430	0.86010	0.86646
361	0.83160	0.84250	0.84755	0.84921	0.85462	0.86040	0.86675
362	0.83193	0.84284	0.84788	0.84954	0.85493	0.86070	0.86703
363	0.83231	0.84318	0.84820	0.84986	0.85523	0.86100	0.86731
364	0.83266	0.84352	0.84852	0.85017	0.85554	0.86129	0.86758
365	0.83300	0.84386	0.84885	0.85049	0.85585	0.86159	0.86786
366	0.83334	0.84420	0.84917	0.85081	0.85615	0.86187	0.86813
367	0.83371	0.84453	0.84949	0.85112	0.85646	0.86217	0.86841
368	0.83406	0.84486	0.84980	0.85144	0.85676	0.86245	0.86868
369	0.83440	0.84520	0.85012	0.85175	0.85706	0.86274	0.86896
370	0.83475	0.84552	0.85044	0.85207	0.85736	0.86303	0.86923
371	0.83509	0.84584	0.85075	0.85239	0.85766	0.86331	0.86951
372	0.83547	0.84617	0.85106	0.85269	0.85795	0.86359	0.86977
373	0.83582	0.84650	0.85137	0.85300	0.85825	0.86388	0.87004
374	0.83618	0.84682	0.85169	0.85331	0.85854	0.86417	0.87031
375	0.83654	0.84713	0.85199	0.85361	0.85883	0.86444	0.87058
376	0.83687	0.84746	0.85230	0.85391	0.85912	0.86472	0.87084
377	0.83722	0.84777	0.85261	0.85421	0.85941	0.86500	0.87111
378	0.83757	0.84808	0.85291	0.85452	0.85970	0.86527	0.87137
379	0.83790	0.84840	0.85322	0.85482	0.85999	0.86555	0.87163
380	0.83822	0.84870	0.85352	0.85512	0.86028	0.86583	0.87189
381	0.83856	0.84901	0.85382	0.85541	0.86056	0.86609	0.87215
382	0.83890	0.84932	0.85412	0.85570	0.86085	0.86636	0.87240
383	0.83924	0.84963	0.85442	0.85600	0.86113	0.86663	0.87266
384	0.83954	0.84994	0.85471	0.85630	0.86141	0.86691	0.87292
385	0.83985	0.85024	0.85499	0.85659	0.86169	0.86717	0.87317
386	0.84018	0.85056	0.85529	0.85687	0.86197	0.86744	0.87342
387	0.84052	0.85087	0.85558	0.85717	0.86225	0.86771	0.87367
388	0.84081	0.85117	0.85588	0.85746	0.86253	0.86797	0.87393
389	0.84114	0.85147	0.85616	0.85774	0.86280	0.86823	0.87418
390	0.84145	0.85176	0.85645	0.85803	0.86307	0.86849	0.87442
391	0.84178	0.85206	0.85675	0.85832	0.86334	0.86876	0.87466
392	0.84209	0.85236	0.85704	0.85859	0.86362	0.86901	0.87491
393	0.84243	0.85266	0.85732	0.85887	0.86389	0.86927	0.87516
394	0.84277	0.85295	0.85761	0.85916	0.86415	0.86953	0.87540
395	0.84307	0.85325	0.85789	0.85944	0.86442	0.86979	0.87565
396	0.84338	0.85354	0.85818	0.85972	0.86469	0.87004	0.87589
397	0.84368	0.85382	0.85846	0.85999	0.86496	0.87029	0.87613
398	0.84401	0.85411	0.85874	0.86027	0.86522	0.87054	0.87637
399	0.84434	0.85440	0.85901	0.86055	0.86548	0.87079	0.87661
400	0.84462	0.85468	0.85929	0.86082	0.86574	0.87105	0.87685
401	0.84495	0.85496	0.85956	0.86109	0.86601	0.87130	0.87708
402	0.84528	0.85525	0.85983	0.86136	0.86627	0.87155	0.87732
403	0.84557	0.85554	0.86010	0.86163	0.86653	0.87180	0.87756

N	0.1%	0.5%	1%	1.25%	2.5%	5%	10%
404	0.84588	0.85583	0.86038	0.86190	0.86679	0.87205	0.87779
405	0.84618	0.85610	0.86065	0.86216	0.86704	0.87229	0.87802
406	0.84648	0.85637	0.86093	0.86244	0.86730	0.87253	0.87826
407	0.84676	0.85666	0.86120	0.86270	0.86755	0.87278	0.87849
408	0.84706	0.85693	0.86147	0.86296	0.86781	0.87302	0.87872
409	0.84737	0.85721	0.86173	0.86322	0.86806	0.87326	0.87895
410	0.84767	0.85747	0.86198	0.86347	0.86831	0.87350	0.87918
411	0.84799	0.85775	0.86225	0.86374	0.86856	0.87374	0.87940
412	0.84827	0.85803	0.86251	0.86399	0.86881	0.87398	0.87963
413	0.84854	0.85830	0.86277	0.86426	0.86906	0.87421	0.87986
414	0.84882	0.85856	0.86303	0.86452	0.86931	0.87445	0.88008
415	0.84912	0.85882	0.86329	0.86478	0.86955	0.87468	0.88030
416	0.84941	0.85910	0.86354	0.86503	0.86980	0.87492	0.88052
417	0.84974	0.85937	0.86380	0.86528	0.87004	0.87515	0.88074
418	0.85001	0.85963	0.86407	0.86554	0.87028	0.87538	0.88097
419	0.85029	0.85990	0.86432	0.86579	0.87052	0.87561	0.88119
420	0.85056	0.86017	0.86457	0.86604	0.87077	0.87585	0.88141
421	0.85086	0.86042	0.86483	0.86629	0.87101	0.87607	0.88162
422	0.85112	0.86068	0.86508	0.86654	0.87125	0.87630	0.88184
423	0.85140	0.86094	0.86532	0.86678	0.87149	0.87653	0.88206
424	0.85168	0.86120	0.86558	0.86703	0.87173	0.87676	0.88228
425	0.85195	0.86146	0.86583	0.86727	0.87197	0.87698	0.88249
426	0.85224	0.86172	0.86608	0.86752	0.87221	0.87721	0.88270
427	0.85251	0.86197	0.86632	0.86777	0.87243	0.87743	0.88292
428	0.85282	0.86222	0.86657	0.86801	0.87267	0.87766	0.88313
429	0.85307	0.86249	0.86681	0.86825	0.87290	0.87788	0.88334
430	0.85333	0.86274	0.86706	0.86849	0.87313	0.87810	0.88355
431	0.85358	0.86299	0.86730	0.86873	0.87336	0.87832	0.88376
432	0.85388	0.86323	0.86754	0.86897	0.87359	0.87853	0.88397
433	0.85414	0.86348	0.86779	0.86921	0.87382	0.87876	0.88418
434	0.85442	0.86373	0.86802	0.86945	0.87405	0.87897	0.88439
435	0.85470	0.86397	0.86826	0.86968	0.87428	0.87919	0.88459
436	0.85496	0.86422	0.86851	0.86992	0.87450	0.87941	0.88480
437	0.85524	0.86446	0.86875	0.87015	0.87473	0.87962	0.88500
438	0.85551	0.86471	0.86898	0.87039	0.87495	0.87984	0.88520
439	0.85576	0.86494	0.86922	0.87062	0.87518	0.88005	0.88541
440	0.85601	0.86519	0.86945	0.87086	0.87540	0.88026	0.88561
441	0.85626	0.86543	0.86969	0.87108	0.87562	0.88047	0.88581
442	0.85653	0.86566	0.86991	0.87131	0.87584	0.88068	0.88601
443	0.85677	0.86590	0.87015	0.87154	0.87606	0.88090	0.88621
444	0.85705	0.86614	0.87038	0.87177	0.87628	0.88111	0.88641
445	0.85731	0.86638	0.87060	0.87200	0.87650	0.88131	0.88661
446	0.85754	0.86662	0.87084	0.87223	0.87671	0.88152	0.88680
447	0.85780	0.86686	0.87106	0.87246	0.87694	0.88173	0.88700
448	0.85804	0.86709	0.87129	0.87268	0.87715	0.88193	0.88720
449	0.85829	0.86733	0.87152	0.87290	0.87736	0.88213	0.88739
450	0.85855	0.86757	0.87175	0.87313	0.87758	0.88234	0.88759
451	0.85880	0.86780	0.87197	0.87336	0.87779	0.88254	0.88778
452	0.85904	0.86802	0.87219	0.87358	0.87800	0.88275	0.88797
453	0.85930	0.86825	0.87241	0.87380	0.87821	0.88295	0.88816
454	0.85953	0.86848	0.87264	0.87401	0.87842	0.88316	0.88836
455	0.85979	0.86870	0.87286	0.87423	0.87864	0.88336	0.88855
456	0.86005	0.86894	0.87308	0.87444	0.87884	0.88356	0.88874
457	0.86031	0.86916	0.87330	0.87467	0.87906	0.88376	0.88893
458	0.86053	0.86939	0.87351	0.87488	0.87927	0.88395	0.88912
459	0.86078	0.86961	0.87372	0.87509	0.87947	0.88415	0.88930
460	0.86103	0.86984	0.87394	0.87531	0.87967	0.88435	0.88949
461	0.86127	0.87007	0.87416	0.87552	0.87987	0.88454	0.88968
462	0.86150	0.87029	0.87437	0.87573	0.88008	0.88474	0.88987
463	0.86175	0.87052	0.87459	0.87594	0.88028	0.88493	0.89005
464	0.86199	0.87074	0.87480	0.87616	0.88049	0.88513	0.89023
465	0.86222	0.87097	0.87501	0.87637	0.88069	0.88533	0.89042
466	0.86246	0.87119	0.87522	0.87658	0.88089	0.88552	0.89060
467	0.86269	0.87140	0.87544	0.87678	0.88110	0.88571	0.89078
468	0.86291	0.87162	0.87565	0.87699	0.88129	0.88590	0.89096
469	0.86316	0.87185	0.87586	0.87719	0.88149	0.88609	0.89115
470	0.86338	0.87206	0.87607	0.87741	0.88169	0.88628	0.89133
471	0.86363	0.87227	0.87627	0.87761	0.88189	0.88647	0.89151
472	0.86386	0.87248	0.87648	0.87781	0.88209	0.88666	0.89169
473	0.86409	0.87269	0.87669	0.87801	0.88228	0.88685	0.89186
474	0.86432	0.87290	0.87688	0.87821	0.88248	0.88703	0.89204
475	0.86454	0.87312	0.87709	0.87842	0.88267	0.88722	0.89222
476	0.86476	0.87333	0.87731	0.87862	0.88286	0.88740	0.89240
477	0.86499	0.87354	0.87751	0.87882	0.88306	0.88759	0.89257

N	0.1%	0.5%	1%	1.25%	2.5%	5%	10%
478	0.86522	0.87374	0.87771	0.87902	0.88325	0.88777	0.89274
479	0.86545	0.87395	0.87792	0.87923	0.88344	0.88796	0.89292
480	0.86568	0.87416	0.87812	0.87943	0.88363	0.88814	0.89309
481	0.86590	0.87437	0.87832	0.87963	0.88383	0.88832	0.89327
482	0.86611	0.87458	0.87853	0.87983	0.88402	0.88850	0.89344
483	0.86633	0.87479	0.87873	0.88002	0.88421	0.88869	0.89361
484	0.86654	0.87499	0.87892	0.88022	0.88440	0.88887	0.89378
485	0.86677	0.87520	0.87912	0.88041	0.88459	0.88905	0.89395
486	0.86699	0.87540	0.87932	0.88061	0.88478	0.88923	0.89413
487	0.86721	0.87561	0.87951	0.88080	0.88497	0.88941	0.89430
488	0.86743	0.87581	0.87971	0.88099	0.88515	0.88959	0.89446
489	0.86764	0.87601	0.87990	0.88119	0.88533	0.88977	0.89463
490	0.86785	0.87622	0.88010	0.88137	0.88552	0.88994	0.89480
491	0.86808	0.87642	0.88029	0.88157	0.88570	0.89012	0.89497
492	0.86832	0.87662	0.88048	0.88176	0.88588	0.89030	0.89513
493	0.86853	0.87681	0.88067	0.88195	0.88607	0.89047	0.89530
494	0.86873	0.87700	0.88087	0.88214	0.88625	0.89065	0.89546
495	0.86895	0.87721	0.88106	0.88233	0.88643	0.89082	0.89563
496	0.86915	0.87741	0.88124	0.88252	0.88661	0.89099	0.89579
497	0.86939	0.87761	0.88143	0.88270	0.88679	0.89117	0.89596
498	0.86960	0.87782	0.88162	0.88289	0.88698	0.89134	0.89612
499	0.86981	0.87802	0.88181	0.88308	0.88715	0.89151	0.89628
500	0.87001	0.87822	0.88201	0.88326	0.88733	0.89168	0.89645
501	0.87023	0.87841	0.88220	0.88345	0.88751	0.89185	0.89661
502	0.87044	0.87860	0.88238	0.88364	0.88769	0.89202	0.89677
503	0.87065	0.87880	0.88257	0.88382	0.88787	0.89219	0.89693
504	0.87086	0.87899	0.88275	0.88400	0.88805	0.89236	0.89709
505	0.87107	0.87918	0.88294	0.88419	0.88822	0.89253	0.89725
506	0.87127	0.87937	0.88313	0.88437	0.88839	0.89269	0.89741
507	0.87146	0.87956	0.88331	0.88455	0.88857	0.89286	0.89757
508	0.87168	0.87975	0.88350	0.88474	0.88874	0.89303	0.89773
509	0.87190	0.87994	0.88368	0.88492	0.88891	0.89319	0.89789
510	0.87209	0.88013	0.88386	0.88510	0.88909	0.89336	0.89804
511	0.87229	0.88031	0.88404	0.88527	0.88926	0.89352	0.89820
512	0.87248	0.88050	0.88422	0.88545	0.88943	0.89369	0.89836
513	0.87268	0.88069	0.88440	0.88563	0.88960	0.89385	0.89851
514	0.87287	0.88087	0.88458	0.88581	0.88977	0.89401	0.89867
515	0.87307	0.88105	0.88476	0.88598	0.88994	0.89417	0.89882
516	0.87329	0.88122	0.88493	0.88616	0.89011	0.89433	0.89897
517	0.87349	0.88142	0.88511	0.88633	0.89028	0.89449	0.89913
518	0.87368	0.88161	0.88529	0.88650	0.89044	0.89466	0.89928
519	0.87388	0.88178	0.88547	0.88668	0.89062	0.89482	0.89943
520	0.87407	0.88197	0.88564	0.88685	0.89079	0.89498	0.89958
521	0.87426	0.88214	0.88582	0.88702	0.89095	0.89514	0.89973
522	0.87446	0.88233	0.88600	0.88720	0.89112	0.89530	0.89989
523	0.87464	0.88251	0.88617	0.88737	0.89128	0.89545	0.90003
524	0.87483	0.88269	0.88634	0.88755	0.89144	0.89561	0.90018
525	0.87502	0.88287	0.88651	0.88772	0.89161	0.89577	0.90033
526	0.87522	0.88305	0.88669	0.88789	0.89177	0.89592	0.90048
527	0.87542	0.88323	0.88686	0.88806	0.89194	0.89608	0.90063
528	0.87561	0.88341	0.88702	0.88823	0.89210	0.89624	0.90078
529	0.87578	0.88358	0.88719	0.88839	0.89226	0.89639	0.90093
530	0.87597	0.88376	0.88736	0.88856	0.89242	0.89654	0.90108
531	0.87615	0.88393	0.88752	0.88873	0.89258	0.89670	0.90122
532	0.87635	0.88411	0.88770	0.88890	0.89274	0.89685	0.90137
533	0.87655	0.88429	0.88787	0.88906	0.89290	0.89701	0.90151
534	0.87673	0.88446	0.88803	0.88922	0.89306	0.89716	0.90166
535	0.87691	0.88464	0.88820	0.88939	0.89322	0.89731	0.90180
536	0.87709	0.88482	0.88836	0.88955	0.89338	0.89746	0.90194
537	0.87726	0.88498	0.88853	0.88972	0.89354	0.89761	0.90209
538	0.87743	0.88516	0.88870	0.88989	0.89370	0.89776	0.90223
539	0.87761	0.88533	0.88887	0.89005	0.89385	0.89791	0.90237
540	0.87778	0.88550	0.88903	0.89021	0.89401	0.89806	0.90252
541	0.87798	0.88567	0.88920	0.89037	0.89416	0.89821	0.90266
542	0.87816	0.88584	0.88936	0.89053	0.89432	0.89836	0.90280
543	0.87833	0.88600	0.88953	0.89069	0.89447	0.89851	0.90294
544	0.87852	0.88617	0.88969	0.89085	0.89463	0.89866	0.90308
545	0.87869	0.88634	0.88985	0.89101	0.89478	0.89880	0.90322
546	0.87887	0.88650	0.89001	0.89117	0.89494	0.89895	0.90336
547	0.87905	0.88667	0.89017	0.89133	0.89509	0.89910	0.90350
548	0.87923	0.88683	0.89033	0.89149	0.89524	0.89924	0.90364
549	0.87942	0.88700	0.89049	0.89165	0.89540	0.89939	0.90378
550	0.87958	0.88716	0.89065	0.89180	0.89555	0.89953	0.90391
551	0.87977	0.88732	0.89081	0.89196	0.89570	0.89968	0.90405

N	0.1%	0.5%	1%	1.25%	2.5%	5%	10%
552	0.87997	0.88749	0.89097	0.89212	0.89585	0.89982	0.90419
553	0.88015	0.88766	0.89113	0.89227	0.89600	0.89997	0.90432
554	0.88031	0.88782	0.89128	0.89243	0.89615	0.90011	0.90446
555	0.88049	0.88799	0.89144	0.89259	0.89630	0.90026	0.90459
556	0.88066	0.88815	0.89159	0.89274	0.89644	0.90040	0.90473
557	0.88084	0.88831	0.89175	0.89289	0.89659	0.90054	0.90487
558	0.88101	0.88847	0.89190	0.89305	0.89674	0.90068	0.90500
559	0.88118	0.88863	0.89206	0.89320	0.89688	0.90082	0.90513
560	0.88135	0.88879	0.89221	0.89336	0.89703	0.90096	0.90527
561	0.88151	0.88894	0.89237	0.89351	0.89718	0.90110	0.90540
562	0.88168	0.88911	0.89253	0.89366	0.89732	0.90124	0.90553
563	0.88187	0.88928	0.89268	0.89381	0.89747	0.90138	0.90566
564	0.88200	0.88944	0.89284	0.89396	0.89762	0.90152	0.90580
565	0.88218	0.88958	0.89299	0.89412	0.89776	0.90166	0.90593
566	0.88235	0.88975	0.89314	0.89426	0.89790	0.90180	0.90606
567	0.88251	0.88990	0.89330	0.89442	0.89804	0.90193	0.90619
568	0.88267	0.89006	0.89344	0.89456	0.89818	0.90207	0.90632
569	0.88284	0.89022	0.89359	0.89471	0.89833	0.90221	0.90645
570	0.88301	0.89037	0.89374	0.89486	0.89847	0.90235	0.90658
571	0.88317	0.89053	0.89389	0.89500	0.89861	0.90248	0.90670
572	0.88333	0.89068	0.89404	0.89515	0.89875	0.90261	0.90683
573	0.88347	0.89083	0.89419	0.89530	0.89889	0.90275	0.90696
574	0.88364	0.89097	0.89434	0.89544	0.89904	0.90288	0.90709
575	0.88380	0.89112	0.89449	0.89559	0.89917	0.90302	0.90722
576	0.88398	0.89128	0.89463	0.89573	0.89932	0.90315	0.90734
577	0.88415	0.89143	0.89478	0.89588	0.89945	0.90328	0.90747
578	0.88431	0.89159	0.89493	0.89603	0.89959	0.90342	0.90760
579	0.88447	0.89174	0.89507	0.89617	0.89973	0.90355	0.90772
580	0.88465	0.89189	0.89522	0.89631	0.89987	0.90368	0.90785
581	0.88480	0.89204	0.89536	0.89646	0.90000	0.90381	0.90797
582	0.88496	0.89219	0.89550	0.89660	0.90014	0.90394	0.90810
583	0.88512	0.89233	0.89565	0.89674	0.90028	0.90407	0.90822
584	0.88527	0.89248	0.89580	0.89688	0.90041	0.90420	0.90835
585	0.88543	0.89263	0.89594	0.89702	0.90055	0.90434	0.90847
586	0.88560	0.89278	0.89608	0.89717	0.90068	0.90446	0.90859
587	0.88575	0.89293	0.89622	0.89731	0.90082	0.90459	0.90872
588	0.88590	0.89308	0.89637	0.89745	0.90095	0.90472	0.90884
589	0.88607	0.89323	0.89651	0.89759	0.90109	0.90485	0.90896
590	0.88623	0.89337	0.89665	0.89773	0.90122	0.90498	0.90908
591	0.88638	0.89351	0.89679	0.89787	0.90136	0.90510	0.90920
592	0.88653	0.89366	0.89693	0.89800	0.90149	0.90523	0.90933
593	0.88669	0.89381	0.89707	0.89814	0.90162	0.90536	0.90945
594	0.88685	0.89396	0.89721	0.89828	0.90176	0.90549	0.90957
595	0.88699	0.89411	0.89735	0.89842	0.90189	0.90561	0.90969
596	0.88714	0.89425	0.89749	0.89856	0.90202	0.90574	0.90981
597	0.88729	0.89439	0.89763	0.89870	0.90215	0.90586	0.90993
598	0.88744	0.89453	0.89777	0.89883	0.90228	0.90599	0.91005
599	0.88759	0.89467	0.89791	0.89896	0.90241	0.90611	0.91016
600	0.88774	0.89481	0.89804	0.89910	0.90254	0.90623	0.91028
601	0.88789	0.89495	0.89818	0.89923	0.90267	0.90636	0.91040
602	0.88803	0.89509	0.89831	0.89937	0.90280	0.90648	0.91052
603	0.88818	0.89523	0.89844	0.89950	0.90293	0.90661	0.91064
604	0.88833	0.89537	0.89858	0.89963	0.90306	0.90673	0.91076
605	0.88847	0.89552	0.89872	0.89977	0.90318	0.90685	0.91087
606	0.88863	0.89565	0.89885	0.89989	0.90331	0.90697	0.91099
607	0.88879	0.89579	0.89898	0.90003	0.90344	0.90710	0.91110
608	0.88894	0.89592	0.89911	0.90016	0.90357	0.90722	0.91122
609	0.88908	0.89606	0.89925	0.90030	0.90369	0.90734	0.91134
610	0.88924	0.89620	0.89938	0.90042	0.90382	0.90746	0.91145
611	0.88939	0.89633	0.89952	0.90056	0.90395	0.90759	0.91157
612	0.88951	0.89647	0.89965	0.90069	0.90408	0.90771	0.91168
613	0.88965	0.89662	0.89978	0.90082	0.90420	0.90782	0.91179
614	0.88981	0.89675	0.89991	0.90095	0.90432	0.90794	0.91191
615	0.88996	0.89688	0.90004	0.90108	0.90445	0.90806	0.91202
616	0.89011	0.89702	0.90017	0.90121	0.90457	0.90818	0.91213
617	0.89026	0.89715	0.90030	0.90134	0.90470	0.90830	0.91225
618	0.89042	0.89728	0.90043	0.90147	0.90482	0.90842	0.91236
619	0.89056	0.89742	0.90056	0.90160	0.90495	0.90854	0.91247
620	0.89071	0.89756	0.90069	0.90172	0.90507	0.90865	0.91258
621	0.89086	0.89769	0.90082	0.90185	0.90519	0.90877	0.91269
622	0.89100	0.89782	0.90095	0.90198	0.90532	0.90889	0.91281
623	0.89114	0.89796	0.90108	0.90210	0.90544	0.90901	0.91292
624	0.89127	0.89809	0.90120	0.90223	0.90556	0.90912	0.91303
625	0.89141	0.89822	0.90133	0.90235	0.90568	0.90924	0.91314

N	0.1%	0.5%	1%	1.25%	2.5%	5%	10%
626	0.89156	0.89836	0.90146	0.90248	0.90580	0.90935	0.91325
627	0.89169	0.89848	0.90159	0.90261	0.90592	0.90947	0.91336
628	0.89183	0.89862	0.90171	0.90273	0.90604	0.90959	0.91347
629	0.89197	0.89875	0.90184	0.90286	0.90616	0.90970	0.91358
630	0.89211	0.89888	0.90196	0.90298	0.90628	0.90981	0.91368
631	0.89225	0.89901	0.90209	0.90311	0.90640	0.90993	0.91379
632	0.89239	0.89914	0.90221	0.90323	0.90651	0.91004	0.91390
633	0.89252	0.89927	0.90234	0.90335	0.90663	0.91016	0.91401
634	0.89266	0.89940	0.90246	0.90347	0.90675	0.91027	0.91412
635	0.89281	0.89952	0.90258	0.90360	0.90686	0.91038	0.91423
636	0.89295	0.89965	0.90270	0.90372	0.90698	0.91050	0.91433
637	0.89308	0.89978	0.90282	0.90383	0.90710	0.91061	0.91444
638	0.89322	0.89991	0.90295	0.90396	0.90722	0.91072	0.91454
639	0.89335	0.90004	0.90307	0.90408	0.90733	0.91083	0.91465
640	0.89349	0.90016	0.90319	0.90420	0.90745	0.91094	0.91476
641	0.89362	0.90028	0.90331	0.90432	0.90756	0.91105	0.91486
642	0.89376	0.90041	0.90343	0.90444	0.90768	0.91117	0.91497
643	0.89390	0.90053	0.90355	0.90456	0.90779	0.91127	0.91507
644	0.89402	0.90066	0.90368	0.90468	0.90790	0.91138	0.91518
645	0.89415	0.90077	0.90379	0.90480	0.90802	0.91149	0.91528
646	0.89428	0.90089	0.90392	0.90492	0.90813	0.91160	0.91539
647	0.89442	0.90102	0.90403	0.90504	0.90825	0.91171	0.91549
648	0.89454	0.90115	0.90416	0.90516	0.90836	0.91182	0.91559
649	0.89468	0.90128	0.90427	0.90527	0.90848	0.91193	0.91570
650	0.89481	0.90140	0.90439	0.90539	0.90859	0.91204	0.91580
651	0.89495	0.90152	0.90452	0.90551	0.90871	0.91214	0.91590
652	0.89507	0.90164	0.90463	0.90563	0.90882	0.91225	0.91600
653	0.89521	0.90177	0.90475	0.90574	0.90893	0.91236	0.91611
654	0.89533	0.90189	0.90486	0.90586	0.90904	0.91247	0.91621
655	0.89547	0.90201	0.90498	0.90597	0.90915	0.91258	0.91631
656	0.89559	0.90213	0.90510	0.90609	0.90927	0.91268	0.91641
657	0.89573	0.90225	0.90521	0.90621	0.90937	0.91279	0.91651
658	0.89585	0.90237	0.90534	0.90632	0.90949	0.91289	0.91661
659	0.89599	0.90249	0.90545	0.90643	0.90960	0.91300	0.91671
660	0.89612	0.90261	0.90556	0.90655	0.90971	0.91310	0.91681
661	0.89624	0.90272	0.90568	0.90667	0.90981	0.91321	0.91691
662	0.89637	0.90284	0.90580	0.90678	0.90992	0.91332	0.91701
663	0.89650	0.90296	0.90591	0.90689	0.91003	0.91342	0.91711
664	0.89662	0.90308	0.90603	0.90701	0.91014	0.91353	0.91721
665	0.89675	0.90320	0.90614	0.90712	0.91025	0.91363	0.91731
666	0.89687	0.90332	0.90626	0.90723	0.91036	0.91373	0.91741
667	0.89700	0.90344	0.90637	0.90734	0.91047	0.91383	0.91751
668	0.89711	0.90355	0.90649	0.90745	0.91057	0.91394	0.91761
669	0.89723	0.90367	0.90659	0.90756	0.91068	0.91404	0.91771
670	0.89737	0.90379	0.90670	0.90767	0.91079	0.91414	0.91780
671	0.89749	0.90391	0.90681	0.90779	0.91090	0.91424	0.91790
672	0.89762	0.90403	0.90693	0.90790	0.91100	0.91435	0.91800
673	0.89774	0.90414	0.90704	0.90801	0.91111	0.91445	0.91810
674	0.89786	0.90426	0.90715	0.90812	0.91122	0.91455	0.91819
675	0.89797	0.90437	0.90726	0.90823	0.91132	0.91465	0.91829
676	0.89811	0.90448	0.90738	0.90834	0.91143	0.91475	0.91838
677	0.89823	0.90460	0.90749	0.90845	0.91153	0.91485	0.91848
678	0.89835	0.90472	0.90760	0.90856	0.91164	0.91495	0.91858
679	0.89848	0.90483	0.90771	0.90867	0.91174	0.91505	0.91867
680	0.89859	0.90495	0.90782	0.90878	0.91185	0.91515	0.91877
681	0.89871	0.90505	0.90793	0.90889	0.91195	0.91525	0.91886
682	0.89883	0.90516	0.90804	0.90900	0.91205	0.91535	0.91896
683	0.89896	0.90528	0.90815	0.90910	0.91216	0.91545	0.91905
684	0.89909	0.90539	0.90827	0.90921	0.91226	0.91555	0.91915
685	0.89921	0.90550	0.90837	0.90932	0.91236	0.91565	0.91924
686	0.89933	0.90561	0.90847	0.90942	0.91247	0.91575	0.91933
687	0.89946	0.90572	0.90858	0.90952	0.91257	0.91585	0.91943
688	0.89958	0.90583	0.90869	0.90963	0.91267	0.91594	0.91952
689	0.89970	0.90594	0.90880	0.90974	0.91277	0.91604	0.91961
690	0.89983	0.90605	0.90891	0.90984	0.91287	0.91614	0.91970
691	0.89994	0.90616	0.90901	0.90995	0.91298	0.91624	0.91980
692	0.90006	0.90627	0.90912	0.91005	0.91308	0.91633	0.91989
693	0.90017	0.90639	0.90922	0.91016	0.91318	0.91643	0.91998
694	0.90030	0.90649	0.90933	0.91026	0.91328	0.91653	0.92007
695	0.90042	0.90660	0.90944	0.91037	0.91338	0.91662	0.92017
696	0.90054	0.90671	0.90955	0.91047	0.91348	0.91672	0.92026
697	0.90066	0.90682	0.90965	0.91057	0.91358	0.91681	0.92035
698	0.90078	0.90692	0.90976	0.91068	0.91369	0.91691	0.92044
699	0.90089	0.90703	0.90986	0.91079	0.91378	0.91701	0.92053

N	0.1%	0.5%	1%	1.25%	2.5%	5%	10%
700	0.90101	0.90714	0.90996	0.91089	0.91388	0.91710	0.92062
701	0.90112	0.90724	0.91006	0.91099	0.91398	0.91719	0.92071
702	0.90124	0.90735	0.91017	0.91109	0.91408	0.91729	0.92080
703	0.90137	0.90746	0.91028	0.91120	0.91418	0.91738	0.92089
704	0.90148	0.90757	0.91038	0.91130	0.91427	0.91748	0.92098
705	0.90160	0.90768	0.91049	0.91140	0.91437	0.91757	0.92107
706	0.90171	0.90779	0.91059	0.91151	0.91447	0.91767	0.92116
707	0.90182	0.90789	0.91069	0.91161	0.91457	0.91776	0.92124
708	0.90194	0.90800	0.91079	0.91171	0.91467	0.91785	0.92133
709	0.90205	0.90810	0.91089	0.91181	0.91476	0.91795	0.92142
710	0.90217	0.90821	0.91099	0.91191	0.91486	0.91804	0.92151
711	0.90227	0.90831	0.91109	0.91201	0.91495	0.91813	0.92160
712	0.90240	0.90842	0.91119	0.91211	0.91505	0.91822	0.92169
713	0.90250	0.90853	0.91129	0.91221	0.91515	0.91831	0.92177
714	0.90261	0.90863	0.91139	0.91230	0.91524	0.91840	0.92186
715	0.90272	0.90873	0.91149	0.91240	0.91534	0.91850	0.92195
716	0.90284	0.90883	0.91159	0.91250	0.91544	0.91859	0.92204
717	0.90296	0.90893	0.91170	0.91260	0.91553	0.91868	0.92212
718	0.90307	0.90903	0.91180	0.91270	0.91563	0.91877	0.92221
719	0.90318	0.90914	0.91189	0.91280	0.91572	0.91886	0.92230
720	0.90329	0.90924	0.91200	0.91290	0.91582	0.91895	0.92238
721	0.90340	0.90935	0.91210	0.91300	0.91591	0.91904	0.92247
722	0.90351	0.90944	0.91220	0.91309	0.91601	0.91913	0.92255
723	0.90362	0.90954	0.91230	0.91319	0.91610	0.91922	0.92264
724	0.90372	0.90965	0.91239	0.91329	0.91619	0.91931	0.92273
725	0.90383	0.90975	0.91249	0.91339	0.91629	0.91940	0.92281
726	0.90394	0.90985	0.91259	0.91348	0.91638	0.91949	0.92289
727	0.90403	0.90995	0.91268	0.91358	0.91647	0.91958	0.92298
728	0.90414	0.91005	0.91278	0.91367	0.91656	0.91967	0.92306
729	0.90426	0.91016	0.91288	0.91377	0.91665	0.91976	0.92315
730	0.90437	0.91026	0.91297	0.91387	0.91675	0.91985	0.92323
731	0.90448	0.91036	0.91307	0.91396	0.91684	0.91993	0.92332
732	0.90460	0.91046	0.91317	0.91405	0.91693	0.92002	0.92340
733	0.90470	0.91055	0.91326	0.91415	0.91703	0.92011	0.92348
734	0.90481	0.91065	0.91336	0.91425	0.91712	0.92020	0.92357
735	0.90492	0.91076	0.91346	0.91434	0.91721	0.92028	0.92365
736	0.90502	0.91086	0.91356	0.91444	0.91730	0.92037	0.92373
737	0.90513	0.91096	0.91365	0.91453	0.91739	0.92045	0.92382
738	0.90524	0.91105	0.91374	0.91462	0.91748	0.92054	0.92390
739	0.90534	0.91115	0.91384	0.91472	0.91757	0.92063	0.92398
740	0.90544	0.91125	0.91394	0.91482	0.91766	0.92071	0.92406
741	0.90555	0.91134	0.91403	0.91491	0.91775	0.92080	0.92415
742	0.90565	0.91144	0.91413	0.91500	0.91784	0.92089	0.92423
743	0.90575	0.91154	0.91422	0.91509	0.91793	0.92097	0.92431
744	0.90585	0.91163	0.91431	0.91519	0.91802	0.92106	0.92439
745	0.90595	0.91173	0.91440	0.91528	0.91810	0.92114	0.92447
746	0.90605	0.91183	0.91450	0.91537	0.91819	0.92123	0.92455
747	0.90617	0.91192	0.91459	0.91546	0.91828	0.92131	0.92463
748	0.90628	0.91203	0.91468	0.91556	0.91837	0.92140	0.92471
749	0.90638	0.91212	0.91478	0.91565	0.91846	0.92148	0.92479
750	0.90648	0.91222	0.91486	0.91574	0.91854	0.92157	0.92487
751	0.90659	0.91231	0.91496	0.91583	0.91863	0.92165	0.92495
752	0.90668	0.91241	0.91505	0.91592	0.91872	0.92173	0.92503
753	0.90679	0.91250	0.91514	0.91601	0.91881	0.92182	0.92511
754	0.90689	0.91259	0.91523	0.91610	0.91890	0.92190	0.92519
755	0.90700	0.91269	0.91532	0.91619	0.91898	0.92199	0.92527
756	0.90710	0.91278	0.91541	0.91628	0.91907	0.92207	0.92535
757	0.90719	0.91288	0.91550	0.91637	0.91916	0.92215	0.92543
758	0.90730	0.91297	0.91559	0.91646	0.91924	0.92223	0.92551
759	0.90739	0.91306	0.91568	0.91655	0.91933	0.92232	0.92558
760	0.90748	0.91315	0.91578	0.91664	0.91941	0.92240	0.92566
761	0.90759	0.91324	0.91587	0.91673	0.91950	0.92248	0.92574
762	0.90768	0.91334	0.91595	0.91682	0.91958	0.92256	0.92582
763	0.90777	0.91343	0.91605	0.91690	0.91967	0.92265	0.92590
764	0.90788	0.91352	0.91613	0.91699	0.91975	0.92273	0.92598
765	0.90797	0.91361	0.91622	0.91708	0.91984	0.92281	0.92605
766	0.90808	0.91371	0.91631	0.91717	0.91992	0.92289	0.92613
767	0.90817	0.91380	0.91640	0.91725	0.92001	0.92297	0.92621
768	0.90828	0.91389	0.91649	0.91734	0.92009	0.92305	0.92628
769	0.90837	0.91398	0.91658	0.91743	0.92018	0.92313	0.92636
770	0.90846	0.91407	0.91666	0.91752	0.92026	0.92321	0.92644
771	0.90856	0.91417	0.91675	0.91761	0.92034	0.92329	0.92651
772	0.90866	0.91426	0.91684	0.91769	0.92043	0.92337	0.92659
773	0.90875	0.91435	0.91692	0.91778	0.92051	0.92345	0.92667

N	0.1%	0.5%	1%	1.25%	2.5%	5%	10%
774	0.90886	0.91444	0.91701	0.91787	0.92059	0.92353	0.92674
775	0.90896	0.91453	0.91710	0.91795	0.92068	0.92361	0.92682
776	0.90907	0.91462	0.91718	0.91803	0.92076	0.92369	0.92689
777	0.90916	0.91471	0.91727	0.91812	0.92084	0.92377	0.92697
778	0.90926	0.91480	0.91736	0.91821	0.92092	0.92385	0.92704
779	0.90934	0.91489	0.91744	0.91829	0.92101	0.92392	0.92712
780	0.90944	0.91498	0.91752	0.91838	0.92109	0.92400	0.92719
781	0.90954	0.91507	0.91761	0.91846	0.92117	0.92408	0.92727
782	0.90963	0.91516	0.91769	0.91854	0.92125	0.92416	0.92734
783	0.90973	0.91525	0.91778	0.91863	0.92133	0.92424	0.92742
784	0.90982	0.91534	0.91787	0.91871	0.92142	0.92432	0.92749
785	0.90991	0.91543	0.91795	0.91879	0.92149	0.92439	0.92756
786	0.91000	0.91552	0.91804	0.91888	0.92157	0.92447	0.92764
787	0.91010	0.91561	0.91812	0.91896	0.92166	0.92455	0.92771
788	0.91020	0.91569	0.91820	0.91904	0.92174	0.92462	0.92779
789	0.91029	0.91579	0.91829	0.91913	0.92182	0.92470	0.92786
790	0.91039	0.91587	0.91838	0.91921	0.92190	0.92478	0.92793
791	0.91049	0.91596	0.91846	0.91929	0.92198	0.92485	0.92801
792	0.91059	0.91605	0.91854	0.91938	0.92206	0.92493	0.92808
793	0.91069	0.91613	0.91863	0.91946	0.92214	0.92501	0.92815
794	0.91078	0.91622	0.91871	0.91954	0.92222	0.92508	0.92822
795	0.91087	0.91631	0.91879	0.91962	0.92230	0.92516	0.92830
796	0.91096	0.91639	0.91888	0.91971	0.92238	0.92524	0.92837
797	0.91104	0.91648	0.91896	0.91979	0.92246	0.92531	0.92844
798	0.91114	0.91656	0.91904	0.91987	0.92254	0.92539	0.92851
799	0.91123	0.91665	0.91913	0.91996	0.92261	0.92546	0.92858
800	0.91132	0.91673	0.91921	0.92003	0.92269	0.92554	0.92865
801	0.91140	0.91681	0.91929	0.92011	0.92277	0.92561	0.92873
802	0.91149	0.91690	0.91937	0.92020	0.92285	0.92569	0.92880
803	0.91158	0.91698	0.91945	0.92028	0.92293	0.92576	0.92887
804	0.91167	0.91707	0.91953	0.92036	0.92301	0.92584	0.92894
805	0.91176	0.91715	0.91962	0.92044	0.92308	0.92591	0.92901
806	0.91186	0.91724	0.91970	0.92052	0.92316	0.92598	0.92908
807	0.91194	0.91732	0.91978	0.92060	0.92324	0.92606	0.92915
808	0.91203	0.91741	0.91986	0.92068	0.92331	0.92613	0.92922
809	0.91212	0.91749	0.91994	0.92076	0.92339	0.92621	0.92929
810	0.91221	0.91757	0.92003	0.92084	0.92347	0.92628	0.92936
811	0.91231	0.91766	0.92011	0.92092	0.92354	0.92635	0.92943
812	0.91239	0.91774	0.92019	0.92100	0.92362	0.92643	0.92950
813	0.91248	0.91782	0.92027	0.92108	0.92370	0.92650	0.92957
814	0.91256	0.91790	0.92035	0.92116	0.92377	0.92657	0.92964
815	0.91266	0.91799	0.92043	0.92124	0.92385	0.92664	0.92971
816	0.91275	0.91807	0.92051	0.92132	0.92393	0.92672	0.92978
817	0.91284	0.91815	0.92059	0.92140	0.92400	0.92679	0.92985
818	0.91291	0.91824	0.92067	0.92148	0.92408	0.92686	0.92991
819	0.91300	0.91832	0.92075	0.92155	0.92415	0.92693	0.92998
820	0.91308	0.91840	0.92083	0.92163	0.92423	0.92700	0.93005
821	0.91317	0.91848	0.92091	0.92171	0.92430	0.92708	0.93012
822	0.91326	0.91856	0.92099	0.92179	0.92437	0.92715	0.93019
823	0.91335	0.91864	0.92106	0.92186	0.92445	0.92722	0.93026
824	0.91343	0.91872	0.92114	0.92194	0.92453	0.92729	0.93032
825	0.91353	0.91880	0.92122	0.92202	0.92460	0.92736	0.93039
826	0.91361	0.91888	0.92130	0.92210	0.92468	0.92743	0.93046
827	0.91370	0.91897	0.92138	0.92217	0.92475	0.92750	0.93053
828	0.91380	0.91905	0.92145	0.92225	0.92482	0.92758	0.93059
829	0.91388	0.91912	0.92153	0.92233	0.92490	0.92764	0.93066
830	0.91395	0.91920	0.92161	0.92240	0.92497	0.92772	0.93073
831	0.91404	0.91928	0.92168	0.92248	0.92504	0.92779	0.93080
832	0.91413	0.91936	0.92176	0.92256	0.92512	0.92786	0.93086
833	0.91422	0.91944	0.92183	0.92263	0.92519	0.92793	0.93093
834	0.91430	0.91952	0.92191	0.92270	0.92526	0.92800	0.93100
835	0.91439	0.91960	0.92199	0.92278	0.92533	0.92807	0.93106
836	0.91447	0.91968	0.92206	0.92286	0.92541	0.92814	0.93113
837	0.91456	0.91976	0.92214	0.92293	0.92548	0.92821	0.93119
838	0.91464	0.91984	0.92222	0.92301	0.92555	0.92828	0.93126
839	0.91473	0.91992	0.92229	0.92308	0.92562	0.92834	0.93133
840	0.91482	0.92000	0.92237	0.92316	0.92569	0.92841	0.93139
841	0.91490	0.92007	0.92244	0.92323	0.92577	0.92848	0.93146
842	0.91499	0.92015	0.92252	0.92331	0.92584	0.92855	0.93152
843	0.91507	0.92023	0.92260	0.92338	0.92591	0.92862	0.93159
844	0.91515	0.92030	0.92267	0.92345	0.92598	0.92869	0.93165
845	0.91523	0.92038	0.92274	0.92353	0.92605	0.92876	0.93172
846	0.91531	0.92045	0.92282	0.92360	0.92612	0.92882	0.93178
847	0.91540	0.92053	0.92289	0.92368	0.92620	0.92889	0.93185

N	0.1%	0.5%	1%	1.25%	2.5%	5%	10%
848	0.91548	0.92060	0.92297	0.92375	0.92627	0.92896	0.93191
849	0.91555	0.92068	0.92304	0.92382	0.92634	0.92903	0.93198
850	0.91563	0.92075	0.92311	0.92390	0.92641	0.92909	0.93204
851	0.91571	0.92083	0.92319	0.92397	0.92648	0.92916	0.93211
852	0.91580	0.92091	0.92326	0.92405	0.92655	0.92923	0.93217
853	0.91588	0.92098	0.92334	0.92411	0.92662	0.92930	0.93223
854	0.91596	0.92106	0.92341	0.92419	0.92669	0.92936	0.93230
855	0.91604	0.92113	0.92348	0.92426	0.92676	0.92943	0.93236
856	0.91613	0.92121	0.92355	0.92434	0.92683	0.92950	0.93243
857	0.91620	0.92129	0.92363	0.92441	0.92690	0.92956	0.93249
858	0.91630	0.92136	0.92370	0.92448	0.92697	0.92963	0.93255
859	0.91638	0.92144	0.92378	0.92455	0.92704	0.92970	0.93262
860	0.91644	0.92151	0.92385	0.92462	0.92711	0.92976	0.93268
861	0.91653	0.92159	0.92392	0.92469	0.92718	0.92983	0.93274
862	0.91660	0.92166	0.92399	0.92477	0.92724	0.92989	0.93281
863	0.91668	0.92174	0.92407	0.92484	0.92731	0.92996	0.93287
864	0.91676	0.92181	0.92414	0.92491	0.92738	0.93003	0.93293
865	0.91684	0.92189	0.92421	0.92498	0.92745	0.93009	0.93299
866	0.91692	0.92197	0.92428	0.92505	0.92752	0.93016	0.93306
867	0.91701	0.92204	0.92436	0.92512	0.92759	0.93023	0.93312
868	0.91707	0.92211	0.92443	0.92520	0.92766	0.93029	0.93318
869	0.91715	0.92219	0.92450	0.92527	0.92772	0.93036	0.93324
870	0.91723	0.92226	0.92457	0.92534	0.92779	0.93042	0.93330
871	0.91731	0.92233	0.92464	0.92541	0.92786	0.93049	0.93337
872	0.91739	0.92240	0.92471	0.92547	0.92793	0.93055	0.93343
873	0.91747	0.92248	0.92478	0.92554	0.92799	0.93061	0.93349
874	0.91755	0.92255	0.92485	0.92561	0.92806	0.93068	0.93355
875	0.91764	0.92262	0.92492	0.92568	0.92813	0.93074	0.93361
876	0.91772	0.92269	0.92499	0.92575	0.92819	0.93081	0.93367
877	0.91779	0.92277	0.92506	0.92582	0.92826	0.93087	0.93373
878	0.91786	0.92284	0.92513	0.92589	0.92833	0.93093	0.93380
879	0.91795	0.92291	0.92520	0.92596	0.92839	0.93100	0.93386
880	0.91802	0.92298	0.92527	0.92603	0.92846	0.93106	0.93392
881	0.91810	0.92305	0.92534	0.92610	0.92853	0.93113	0.93398
882	0.91818	0.92312	0.92541	0.92617	0.92859	0.93119	0.93404
883	0.91825	0.92320	0.92548	0.92623	0.92866	0.93125	0.93410
884	0.91833	0.92327	0.92554	0.92630	0.92872	0.93132	0.93416
885	0.91841	0.92334	0.92561	0.92637	0.92879	0.93138	0.93422
886	0.91848	0.92341	0.92568	0.92644	0.92885	0.93144	0.93428
887	0.91856	0.92349	0.92575	0.92651	0.92892	0.93151	0.93434
888	0.91863	0.92356	0.92582	0.92657	0.92898	0.93157	0.93440
889	0.91870	0.92363	0.92588	0.92664	0.92905	0.93163	0.93446
890	0.91878	0.92370	0.92595	0.92671	0.92911	0.93169	0.93452
891	0.91885	0.92377	0.92602	0.92678	0.92918	0.93176	0.93458
892	0.91892	0.92384	0.92609	0.92684	0.92924	0.93182	0.93464
893	0.91900	0.92391	0.92616	0.92691	0.92931	0.93188	0.93470
894	0.91908	0.92398	0.92623	0.92698	0.92937	0.93194	0.93475
895	0.91916	0.92405	0.92630	0.92704	0.92944	0.93200	0.93481
896	0.91924	0.92412	0.92636	0.92711	0.92950	0.93207	0.93487
897	0.91931	0.92419	0.92643	0.92717	0.92957	0.93213	0.93493
898	0.91939	0.92425	0.92650	0.92724	0.92963	0.93219	0.93499
899	0.91946	0.92432	0.92657	0.92731	0.92969	0.93225	0.93505
900	0.91954	0.92439	0.92664	0.92738	0.92976	0.93231	0.93511
901	0.91961	0.92446	0.92670	0.92744	0.92982	0.93237	0.93516
902	0.91968	0.92453	0.92677	0.92751	0.92988	0.93244	0.93522
903	0.91975	0.92460	0.92684	0.92758	0.92995	0.93250	0.93528
904	0.91983	0.92467	0.92690	0.92764	0.93001	0.93256	0.93534
905	0.91990	0.92474	0.92697	0.92771	0.93008	0.93262	0.93539
906	0.91998	0.92481	0.92703	0.92777	0.93014	0.93268	0.93545
907	0.92004	0.92488	0.92710	0.92784	0.93020	0.93274	0.93551
908	0.92012	0.92495	0.92716	0.92790	0.93026	0.93280	0.93557
909	0.92019	0.92502	0.92723	0.92797	0.93033	0.93286	0.93562
910	0.92027	0.92508	0.92729	0.92803	0.93039	0.93291	0.93568
911	0.92034	0.92515	0.92736	0.92809	0.93045	0.93298	0.93574
912	0.92041	0.92521	0.92743	0.92816	0.93052	0.93303	0.93579
913	0.92049	0.92528	0.92749	0.92822	0.93058	0.93309	0.93585
914	0.92057	0.92536	0.92755	0.92829	0.93064	0.93315	0.93591
915	0.92063	0.92542	0.92761	0.92835	0.93070	0.93321	0.93596
916	0.92071	0.92549	0.92768	0.92842	0.93076	0.93327	0.93602
917	0.92078	0.92555	0.92775	0.92848	0.93083	0.93333	0.93608
918	0.92085	0.92562	0.92781	0.92855	0.93089	0.93339	0.93613
919	0.92093	0.92568	0.92788	0.92861	0.93095	0.93345	0.93619
920	0.92100	0.92575	0.92794	0.92867	0.93101	0.93351	0.93625
921	0.92107	0.92582	0.92801	0.92873	0.93107	0.93357	0.93630

N	0.1%	0.5%	1%	1.25%	2.5%	5%	10%
922	0.92115	0.92589	0.92807	0.92880	0.93113	0.93363	0.93636
923	0.92122	0.92595	0.92813	0.92886	0.93119	0.93368	0.93641
924	0.92129	0.92602	0.92820	0.92893	0.93125	0.93374	0.93647
925	0.92136	0.92608	0.92826	0.92899	0.93131	0.93380	0.93652
926	0.92142	0.92615	0.92832	0.92905	0.93137	0.93386	0.93658
927	0.92149	0.92621	0.92838	0.92911	0.93143	0.93392	0.93663
928	0.92156	0.92628	0.92845	0.92918	0.93149	0.93397	0.93669
929	0.92163	0.92635	0.92851	0.92924	0.93156	0.93403	0.93674
930	0.92170	0.92641	0.92857	0.92930	0.93161	0.93409	0.93680
931	0.92177	0.92648	0.92863	0.92936	0.93167	0.93415	0.93685
932	0.92184	0.92654	0.92870	0.92942	0.93173	0.93420	0.93691
933	0.92192	0.92661	0.92876	0.92949	0.93179	0.93426	0.93696
934	0.92198	0.92668	0.92882	0.92955	0.93186	0.93432	0.93702
935	0.92206	0.92674	0.92888	0.92961	0.93191	0.93437	0.93707
936	0.92214	0.92681	0.92895	0.92967	0.93197	0.93443	0.93713
937	0.92221	0.92687	0.92901	0.92973	0.93203	0.93449	0.93718
938	0.92228	0.92694	0.92907	0.92979	0.93209	0.93454	0.93723
939	0.92234	0.92700	0.92913	0.92985	0.93215	0.93460	0.93729
940	0.92241	0.92707	0.92919	0.92992	0.93221	0.93466	0.93734
941	0.92248	0.92713	0.92926	0.92998	0.93227	0.93471	0.93739
942	0.92255	0.92719	0.92932	0.93004	0.93233	0.93477	0.93745
943	0.92263	0.92726	0.92938	0.93010	0.93239	0.93483	0.93750
944	0.92269	0.92732	0.92944	0.93016	0.93244	0.93488	0.93756
945	0.92276	0.92739	0.92951	0.93022	0.93250	0.93494	0.93761
946	0.92282	0.92745	0.92957	0.93028	0.93256	0.93500	0.93766
947	0.92288	0.92751	0.92963	0.93034	0.93262	0.93505	0.93772
948	0.92294	0.92757	0.92969	0.93040	0.93268	0.93511	0.93777
949	0.92301	0.92764	0.92975	0.93046	0.93274	0.93516	0.93782
950	0.92307	0.92770	0.92981	0.93052	0.93280	0.93522	0.93788
951	0.92313	0.92776	0.92987	0.93058	0.93285	0.93527	0.93793
952	0.92319	0.92782	0.92993	0.93064	0.93291	0.93533	0.93798
953	0.92326	0.92788	0.92999	0.93070	0.93297	0.93538	0.93803
954	0.92332	0.92794	0.93005	0.93076	0.93302	0.93544	0.93809
955	0.92338	0.92801	0.93011	0.93082	0.93308	0.93550	0.93814
956	0.92345	0.92807	0.93017	0.93088	0.93314	0.93555	0.93819
957	0.92352	0.92813	0.93023	0.93094	0.93320	0.93560	0.93824
958	0.92358	0.92819	0.93030	0.93100	0.93325	0.93566	0.93830
959	0.92364	0.92826	0.93035	0.93106	0.93331	0.93571	0.93835
960	0.92372	0.92832	0.93041	0.93112	0.93337	0.93577	0.93840
961	0.92379	0.92838	0.93047	0.93118	0.93342	0.93582	0.93845
962	0.92385	0.92844	0.93053	0.93124	0.93348	0.93588	0.93850
963	0.92392	0.92851	0.93060	0.93129	0.93354	0.93593	0.93856
964	0.92398	0.92857	0.93065	0.93135	0.93359	0.93599	0.93861
965	0.92404	0.92863	0.93071	0.93141	0.93365	0.93604	0.93866
966	0.92411	0.92868	0.93077	0.93147	0.93370	0.93609	0.93871
967	0.92418	0.92874	0.93083	0.93153	0.93376	0.93615	0.93876
968	0.92424	0.92880	0.93089	0.93159	0.93381	0.93620	0.93881
969	0.92431	0.92887	0.93095	0.93164	0.93387	0.93625	0.93886
970	0.92437	0.92893	0.93101	0.93170	0.93393	0.93631	0.93891
971	0.92445	0.92899	0.93107	0.93176	0.93398	0.93636	0.93896
972	0.92450	0.92905	0.93112	0.93182	0.93404	0.93641	0.93902
973	0.92457	0.92911	0.93118	0.93187	0.93409	0.93647	0.93907
974	0.92465	0.92917	0.93124	0.93193	0.93415	0.93652	0.93912
975	0.92471	0.92923	0.93130	0.93199	0.93420	0.93657	0.93917
976	0.92478	0.92929	0.93135	0.93205	0.93426	0.93662	0.93922
977	0.92485	0.92935	0.93141	0.93210	0.93431	0.93668	0.93927
978	0.92491	0.92941	0.93147	0.93216	0.93437	0.93673	0.93932
979	0.92498	0.92947	0.93153	0.93222	0.93442	0.93678	0.93937
980	0.92504	0.92953	0.93158	0.93227	0.93448	0.93684	0.93942
981	0.92511	0.92959	0.93164	0.93233	0.93453	0.93689	0.93947
982	0.92517	0.92964	0.93170	0.93238	0.93459	0.93694	0.93952
983	0.92523	0.92971	0.93176	0.93244	0.93464	0.93700	0.93957
984	0.92530	0.92977	0.93181	0.93250	0.93470	0.93705	0.93962
985	0.92537	0.92983	0.93187	0.93255	0.93475	0.93710	0.93967
986	0.92543	0.92989	0.93193	0.93261	0.93480	0.93715	0.93972
987	0.92550	0.92995	0.93198	0.93266	0.93486	0.93720	0.93977
988	0.92556	0.93000	0.93204	0.93272	0.93491	0.93726	0.93982
989	0.92563	0.93006	0.93210	0.93278	0.93497	0.93731	0.93987
990	0.92569	0.93012	0.93215	0.93283	0.93502	0.93736	0.93992
991	0.92575	0.93018	0.93221	0.93289	0.93508	0.93741	0.93997
992	0.92581	0.93024	0.93227	0.93294	0.93513	0.93746	0.94002
993	0.92588	0.93029	0.93232	0.93300	0.93518	0.93751	0.94006
994	0.92594	0.93036	0.93238	0.93306	0.93524	0.93756	0.94011
995	0.92600	0.93042	0.93243	0.93311	0.93529	0.93761	0.94016

N	0.1%	0.5%	1%	1.25%	2.5%	5%	10%
996	0.92607	0.93047	0.93249	0.93317	0.93534	0.93767	0.94021
997	0.92614	0.93053	0.93255	0.93322	0.93540	0.93772	0.94026
998	0.92620	0.93059	0.93260	0.93328	0.93545	0.93777	0.94031
999	0.92626	0.93064	0.93265	0.93333	0.93550	0.93782	0.94036
1000	0.92632	0.93070	0.93271	0.93339	0.93556	0.93787	0.94040

Table 3 – *GM6(4H2L or 2H4L)*. Critical values of upper significance levels for the Grubbs multiple-6 outlier test, applicable in situations where the four highest and two lowest or two highest and four lowest observations are simultaneously identified as outliers, denoted as *GM6(4H2L)* and *GM6(2H4L)*, respectively.

N	0.1%	0.5%	1%	1.25%	2.5%	5%	10%
8	0.00000	0.00000	0.00000	0.00000	0.00001	0.00003	0.00013
9	0.00002	0.00011	0.00022	0.00028	0.00058	0.00122	0.00264
10	0.00033	0.00100	0.00164	0.00193	0.00320	0.00539	0.00929
11	0.00145	0.00345	0.00504	0.00571	0.00844	0.01270	0.01949
12	0.00383	0.00772	0.01054	0.01167	0.01613	0.02259	0.03220
13	0.00757	0.01375	0.01793	0.01956	0.02581	0.03440	0.04660
14	0.01266	0.02128	0.02689	0.02901	0.03694	0.04749	0.06204
15	0.01900	0.03009	0.03700	0.03961	0.04915	0.06149	0.07801
16	0.02629	0.03993	0.04809	0.05113	0.06207	0.07603	0.09428
17	0.03458	0.05055	0.05988	0.06330	0.07553	0.09083	0.11055
18	0.04363	0.06172	0.07210	0.07587	0.08917	0.10573	0.12672
19	0.05306	0.07328	0.08455	0.08860	0.10298	0.12050	0.14257
20	0.06298	0.08492	0.09711	0.10151	0.11675	0.13520	0.15812
21	0.07342	0.09675	0.10973	0.11432	0.13042	0.14961	0.17328
22	0.08372	0.10873	0.12237	0.12721	0.14390	0.16379	0.18805
23	0.09435	0.12072	0.13485	0.13984	0.15721	0.17757	0.20242
24	0.10507	0.13261	0.14723	0.15241	0.17024	0.19109	0.21629
25	0.11565	0.14432	0.15951	0.16488	0.18300	0.20429	0.22977
26	0.12626	0.15594	0.17149	0.17698	0.19551	0.21705	0.24283
27	0.13685	0.16725	0.18322	0.18879	0.20765	0.22947	0.25546
28	0.14746	0.17853	0.19470	0.20036	0.21946	0.24153	0.26769
29	0.15784	0.18952	0.20597	0.21171	0.23098	0.25327	0.27953
30	0.16813	0.20034	0.21695	0.22278	0.24224	0.26466	0.29100
31	0.17800	0.21094	0.22777	0.23357	0.25319	0.27574	0.30208
32	0.18781	0.22129	0.23835	0.24422	0.26385	0.28646	0.31278
33	0.19765	0.23148	0.24862	0.25448	0.27429	0.29689	0.32321
34	0.20721	0.24156	0.25864	0.26455	0.28435	0.30700	0.33327
35	0.21669	0.25116	0.26842	0.27428	0.29426	0.31683	0.34303
36	0.22588	0.26062	0.27795	0.28386	0.30384	0.32638	0.35247
37	0.23500	0.26981	0.28723	0.29319	0.31317	0.33565	0.36161
38	0.24369	0.27879	0.29625	0.30223	0.32222	0.34461	0.37047
39	0.25226	0.28760	0.30508	0.31112	0.33103	0.35335	0.37913
40	0.26083	0.29620	0.31371	0.31973	0.33958	0.36183	0.38749
41	0.26922	0.30462	0.32214	0.32815	0.34797	0.37014	0.39559
42	0.27750	0.31278	0.33032	0.33626	0.35611	0.37820	0.40351
43	0.28552	0.32091	0.33831	0.34427	0.36398	0.38598	0.41121
44	0.29337	0.32878	0.34612	0.35200	0.37170	0.39362	0.41866
45	0.30113	0.33650	0.35372	0.35962	0.37923	0.40103	0.42589
46	0.30870	0.34391	0.36107	0.36700	0.38655	0.40823	0.43292
47	0.31602	0.35122	0.36838	0.37428	0.39369	0.41526	0.43979
48	0.32336	0.35848	0.37547	0.38132	0.40067	0.42210	0.44646
49	0.33052	0.36541	0.38247	0.38827	0.40745	0.42878	0.45293
50	0.33743	0.37224	0.38928	0.39501	0.41409	0.43526	0.45927
51	0.34438	0.37882	0.39585	0.40160	0.42054	0.44160	0.46546
52	0.35108	0.38540	0.40227	0.40803	0.42688	0.44777	0.47146
53	0.35760	0.39187	0.40858	0.41425	0.43303	0.45382	0.47734
54	0.36391	0.39804	0.41473	0.42040	0.43911	0.45975	0.48308
55	0.37006	0.40413	0.42077	0.42645	0.44503	0.46552	0.48867
56	0.37605	0.41017	0.42668	0.43231	0.45075	0.47112	0.49412
57	0.38213	0.41606	0.43248	0.43807	0.45639	0.47665	0.49945
58	0.38782	0.42177	0.43815	0.44369	0.46192	0.48201	0.50464
59	0.39383	0.42746	0.44368	0.44916	0.46727	0.48727	0.50973
60	0.39931	0.43285	0.44906	0.45451	0.47254	0.49238	0.51469
61	0.40491	0.43827	0.45437	0.45979	0.47771	0.49739	0.51954
62	0.41018	0.44357	0.45950	0.46496	0.48278	0.50229	0.52426
63	0.41549	0.44880	0.46459	0.47001	0.48772	0.50712	0.52889
64	0.42055	0.45377	0.46959	0.47492	0.49255	0.51184	0.53343
65	0.42573	0.45874	0.47439	0.47970	0.49731	0.51643	0.53787
66	0.43074	0.46354	0.47923	0.48452	0.50192	0.52092	0.54222
67	0.43578	0.46832	0.48393	0.48918	0.50645	0.52536	0.54646

N	0.1%	0.5%	1%	1.25%	2.5%	5%	10%
68	0.44074	0.47307	0.48850	0.49378	0.51091	0.52965	0.55063
69	0.44546	0.47763	0.49306	0.49826	0.51526	0.53389	0.55473
70	0.45020	0.48228	0.49754	0.50267	0.51955	0.53803	0.55872
71	0.45485	0.48677	0.50192	0.50702	0.52375	0.54210	0.56263
72	0.45923	0.49110	0.50615	0.51119	0.52786	0.54611	0.56647
73	0.46360	0.49530	0.51028	0.51538	0.53189	0.55001	0.57025
74	0.46811	0.49949	0.51437	0.51940	0.53584	0.55387	0.57393
75	0.47219	0.50360	0.51845	0.52345	0.53975	0.55762	0.57756
76	0.47644	0.50768	0.52239	0.52735	0.54355	0.56131	0.58110
77	0.48047	0.51161	0.52624	0.53120	0.54728	0.56494	0.58460
78	0.48488	0.51543	0.53006	0.53501	0.55096	0.56850	0.58802
79	0.48887	0.51932	0.53381	0.53871	0.55461	0.57202	0.59138
80	0.49266	0.52307	0.53751	0.54235	0.55815	0.57543	0.59469
81	0.49656	0.52675	0.54109	0.54594	0.56160	0.57879	0.59792
82	0.50044	0.53041	0.54467	0.54946	0.56504	0.58210	0.60110
83	0.50416	0.53403	0.54812	0.55287	0.56842	0.58539	0.60424
84	0.50765	0.53754	0.55157	0.55627	0.57174	0.58858	0.60731
85	0.51140	0.54101	0.55493	0.55964	0.57500	0.59172	0.61031
86	0.51488	0.54438	0.55829	0.56298	0.57824	0.59480	0.61327
87	0.51835	0.54769	0.56161	0.56625	0.58137	0.59784	0.61618
88	0.52191	0.55104	0.56485	0.56943	0.58449	0.60083	0.61903
89	0.52530	0.55431	0.56802	0.57255	0.58752	0.60376	0.62185
90	0.52861	0.55750	0.57111	0.57566	0.59052	0.60664	0.62462
91	0.53196	0.56064	0.57416	0.57869	0.59348	0.60950	0.62734
92	0.53526	0.56376	0.57719	0.58167	0.59636	0.61231	0.63004
93	0.53851	0.56678	0.58012	0.58461	0.59922	0.61507	0.63267
94	0.54160	0.56976	0.58307	0.58753	0.60201	0.61777	0.63527
95	0.54450	0.57265	0.58594	0.59036	0.60479	0.62043	0.63782
96	0.54776	0.57562	0.58880	0.59320	0.60754	0.62306	0.64035
97	0.55093	0.57854	0.59161	0.59600	0.61021	0.62565	0.64282
98	0.55387	0.58138	0.59432	0.59868	0.61286	0.62821	0.64526
99	0.55680	0.58416	0.59704	0.60139	0.61546	0.63070	0.64765
100	0.55975	0.58689	0.59972	0.60403	0.61803	0.63318	0.65002
101	0.56256	0.58965	0.60234	0.60666	0.62055	0.63563	0.65234
102	0.56533	0.59227	0.60493	0.60921	0.62303	0.63802	0.65463
103	0.56807	0.59496	0.60749	0.61174	0.62547	0.64041	0.65690
104	0.57078	0.59756	0.61002	0.61425	0.62790	0.64273	0.65912
105	0.57334	0.60010	0.61249	0.61671	0.63031	0.64502	0.66131
106	0.57616	0.60257	0.61497	0.61916	0.63267	0.64730	0.66346
107	0.57885	0.60503	0.61741	0.62156	0.63500	0.64954	0.66561
108	0.58141	0.60753	0.61983	0.62398	0.63731	0.65175	0.66770
109	0.58401	0.60997	0.62218	0.62625	0.63955	0.65391	0.66977
110	0.58652	0.61233	0.62450	0.62856	0.64177	0.65604	0.67183
111	0.58907	0.61466	0.62678	0.63083	0.64396	0.65815	0.67385
112	0.59147	0.61702	0.62906	0.63307	0.64612	0.66025	0.67583
113	0.59405	0.61932	0.63130	0.63529	0.64824	0.66229	0.67780
114	0.59644	0.62154	0.63348	0.63744	0.65034	0.66431	0.67973
115	0.59882	0.62373	0.63563	0.63958	0.65240	0.66632	0.68164
116	0.60100	0.62593	0.63778	0.64173	0.65445	0.66827	0.68353
117	0.60349	0.62810	0.63990	0.64385	0.65647	0.67021	0.68538
118	0.60576	0.63027	0.64200	0.64588	0.65848	0.67213	0.68721
119	0.60793	0.63241	0.64403	0.64792	0.66045	0.67403	0.68902
120	0.61011	0.63452	0.64607	0.64994	0.66241	0.67590	0.69082
121	0.61226	0.63662	0.64805	0.65193	0.66433	0.67774	0.69257
122	0.61435	0.63868	0.65004	0.65392	0.66623	0.67956	0.69432
123	0.61658	0.64074	0.65201	0.65586	0.66809	0.68135	0.69605
124	0.61881	0.64274	0.65396	0.65778	0.66994	0.68315	0.69774
125	0.62093	0.64466	0.65586	0.65962	0.67178	0.68492	0.69942
126	0.62296	0.64660	0.65773	0.66150	0.67360	0.68662	0.70108
127	0.62495	0.64848	0.65960	0.66333	0.67537	0.68836	0.70271
128	0.62708	0.65042	0.66145	0.66516	0.67715	0.69005	0.70433
129	0.62905	0.65228	0.66327	0.66695	0.67889	0.69174	0.70593
130	0.63097	0.65416	0.66508	0.66874	0.68059	0.69338	0.70750
131	0.63287	0.65600	0.66686	0.67051	0.68231	0.69502	0.70906
132	0.63479	0.65778	0.66861	0.67226	0.68398	0.69663	0.71060
133	0.63665	0.65961	0.67035	0.67397	0.68563	0.69823	0.71213
134	0.63857	0.66139	0.67208	0.67566	0.68728	0.69979	0.71363
135	0.64039	0.66313	0.67380	0.67737	0.68889	0.70135	0.71511
136	0.64227	0.66487	0.67546	0.67900	0.69049	0.70290	0.71658
137	0.64409	0.66662	0.67714	0.68068	0.69206	0.70441	0.71803
138	0.64592	0.66832	0.67877	0.68228	0.69362	0.70590	0.71946
139	0.64771	0.66996	0.68037	0.68387	0.69517	0.70739	0.72088
140	0.64948	0.67160	0.68198	0.68547	0.69671	0.70888	0.72229
141	0.65128	0.67325	0.68360	0.68706	0.69822	0.71031	0.72367

N	0.1%	0.5%	1%	1.25%	2.5%	5%	10%
142	0.65293	0.67486	0.68517	0.68861	0.69973	0.71176	0.72504
143	0.65465	0.67647	0.68671	0.69013	0.70122	0.71319	0.72640
144	0.65639	0.67805	0.68825	0.69164	0.70269	0.71460	0.72774
145	0.65798	0.67965	0.68977	0.69316	0.70415	0.71600	0.72906
146	0.65964	0.68118	0.69129	0.69464	0.70558	0.71737	0.73037
147	0.66121	0.68273	0.69274	0.69614	0.70699	0.71874	0.73167
148	0.66285	0.68423	0.69420	0.69758	0.70841	0.72008	0.73295
149	0.66447	0.68571	0.69566	0.69901	0.70980	0.72142	0.73422
150	0.66607	0.68722	0.69712	0.70045	0.71118	0.72274	0.73547
151	0.66760	0.68867	0.69854	0.70186	0.71255	0.72403	0.73672
152	0.66922	0.69014	0.69993	0.70324	0.71388	0.72533	0.73795
153	0.67073	0.69158	0.70135	0.70461	0.71523	0.72661	0.73917
154	0.67222	0.69302	0.70271	0.70597	0.71654	0.72789	0.74037
155	0.67375	0.69446	0.70409	0.70735	0.71786	0.72915	0.74157
156	0.67531	0.69582	0.70544	0.70869	0.71915	0.73037	0.74275
157	0.67669	0.69721	0.70678	0.71001	0.72042	0.73161	0.74392
158	0.67820	0.69859	0.70811	0.71133	0.72168	0.73282	0.74508
159	0.67958	0.69994	0.70942	0.71260	0.72294	0.73402	0.74622
160	0.68107	0.70132	0.71073	0.71389	0.72418	0.73522	0.74736
161	0.68255	0.70262	0.71199	0.71515	0.72541	0.73640	0.74848
162	0.68387	0.70395	0.71324	0.71642	0.72662	0.73756	0.74958
163	0.68532	0.70526	0.71453	0.71767	0.72781	0.73871	0.75070
164	0.68665	0.70657	0.71582	0.71892	0.72900	0.73986	0.75178
165	0.68799	0.70785	0.71705	0.72014	0.73019	0.74099	0.75287
166	0.68924	0.70908	0.71827	0.72135	0.73137	0.74211	0.75394
167	0.69060	0.71032	0.71947	0.72253	0.73252	0.74323	0.75500
168	0.69198	0.71159	0.72069	0.72372	0.73368	0.74433	0.75604
169	0.69336	0.71284	0.72188	0.72491	0.73482	0.74542	0.75708
170	0.69468	0.71407	0.72305	0.72607	0.73594	0.74649	0.75810
171	0.69588	0.71527	0.72425	0.72726	0.73706	0.74756	0.75912
172	0.69722	0.71648	0.72541	0.72841	0.73817	0.74863	0.76013
173	0.69847	0.71767	0.72658	0.72955	0.73926	0.74968	0.76113
174	0.69964	0.71883	0.72772	0.73067	0.74034	0.75072	0.76212
175	0.70094	0.72003	0.72884	0.73179	0.74143	0.75175	0.76310
176	0.70215	0.72118	0.72996	0.73288	0.74250	0.75277	0.76407
177	0.70335	0.72234	0.73108	0.73398	0.74355	0.75379	0.76504
178	0.70462	0.72346	0.73216	0.73506	0.74459	0.75480	0.76600
179	0.70579	0.72458	0.73324	0.73614	0.74563	0.75579	0.76694
180	0.70696	0.72569	0.73432	0.73721	0.74666	0.75678	0.76789
181	0.70813	0.72680	0.73539	0.73827	0.74767	0.75775	0.76881
182	0.70931	0.72788	0.73644	0.73932	0.74868	0.75872	0.76973
183	0.71050	0.72896	0.73752	0.74037	0.74968	0.75968	0.77064
184	0.71163	0.73005	0.73855	0.74140	0.75067	0.76063	0.77154
185	0.71273	0.73112	0.73959	0.74243	0.75165	0.76156	0.77244
186	0.71381	0.73218	0.74062	0.74344	0.75263	0.76249	0.77334
187	0.71503	0.73324	0.74162	0.74444	0.75359	0.76342	0.77422
188	0.71610	0.73427	0.74263	0.74543	0.75454	0.76434	0.77510
189	0.71727	0.73529	0.74364	0.74642	0.75548	0.76526	0.77596
190	0.71829	0.73633	0.74462	0.74740	0.75643	0.76616	0.77682
191	0.71939	0.73731	0.74561	0.74838	0.75737	0.76705	0.77766
192	0.72038	0.73831	0.74660	0.74934	0.75830	0.76793	0.77851
193	0.72139	0.73931	0.74756	0.75029	0.75922	0.76882	0.77935
194	0.72247	0.74027	0.74850	0.75124	0.76012	0.76969	0.78018
195	0.72349	0.74127	0.74946	0.75218	0.76102	0.77055	0.78101
196	0.72451	0.74226	0.75040	0.75311	0.76191	0.77141	0.78182
197	0.72562	0.74318	0.75132	0.75403	0.76282	0.77226	0.78263
198	0.72661	0.74414	0.75225	0.75493	0.76369	0.77311	0.78343
199	0.72765	0.74512	0.75316	0.75583	0.76457	0.77394	0.78422
200	0.72866	0.74606	0.75406	0.75673	0.76543	0.77477	0.78501
201	0.72960	0.74699	0.75498	0.75762	0.76630	0.77559	0.78579
202	0.73059	0.74793	0.75587	0.75851	0.76715	0.77641	0.78657
203	0.73159	0.74883	0.75676	0.75939	0.76799	0.77722	0.78734
204	0.73257	0.74974	0.75765	0.76028	0.76883	0.77802	0.78811
205	0.73360	0.75063	0.75851	0.76114	0.76965	0.77882	0.78887
206	0.73452	0.75154	0.75936	0.76200	0.77048	0.77962	0.78963
207	0.73547	0.75245	0.76024	0.76284	0.77130	0.78040	0.79037
208	0.73643	0.75333	0.76111	0.76370	0.77211	0.78118	0.79111
209	0.73733	0.75423	0.76194	0.76455	0.77292	0.78196	0.79185
210	0.73831	0.75506	0.76279	0.76538	0.77372	0.78271	0.79258
211	0.73924	0.75592	0.76364	0.76620	0.77452	0.78347	0.79330
212	0.74018	0.75680	0.76446	0.76704	0.77531	0.78422	0.79403
213	0.74110	0.75763	0.76527	0.76783	0.77610	0.78497	0.79474
214	0.74202	0.75847	0.76609	0.76864	0.77687	0.78572	0.79544
215	0.74287	0.75931	0.76690	0.76945	0.77763	0.78645	0.79614

N	0.1%	0.5%	1%	1.25%	2.5%	5%	10%
216	0.74380	0.76014	0.76771	0.77025	0.77840	0.78718	0.79683
217	0.74466	0.76096	0.76850	0.77103	0.77916	0.78791	0.79752
218	0.74554	0.76179	0.76931	0.77181	0.77992	0.78864	0.79821
219	0.74642	0.76256	0.77010	0.77259	0.78066	0.78934	0.79889
220	0.74733	0.76338	0.77088	0.77335	0.78141	0.79005	0.79956
221	0.74815	0.76418	0.77165	0.77412	0.78214	0.79076	0.80023
222	0.74899	0.76497	0.77242	0.77488	0.78286	0.79147	0.80089
223	0.74987	0.76576	0.77318	0.77563	0.78360	0.79216	0.80155
224	0.75071	0.76653	0.77392	0.77637	0.78431	0.79285	0.80221
225	0.75154	0.76732	0.77467	0.77713	0.78502	0.79353	0.80286
226	0.75239	0.76809	0.77543	0.77785	0.78573	0.79422	0.80351
227	0.75317	0.76885	0.77617	0.77859	0.78643	0.79489	0.80415
228	0.75401	0.76964	0.77689	0.77930	0.78713	0.79555	0.80479
229	0.75479	0.77039	0.77762	0.78003	0.78783	0.79621	0.80542
230	0.75558	0.77114	0.77835	0.78074	0.78851	0.79687	0.80605
231	0.75634	0.77187	0.77906	0.78148	0.78919	0.79753	0.80668
232	0.75713	0.77260	0.77978	0.78218	0.78988	0.79818	0.80729
233	0.75789	0.77331	0.78049	0.78287	0.79055	0.79883	0.80791
234	0.75867	0.77403	0.78119	0.78355	0.79121	0.79946	0.80852
235	0.75946	0.77476	0.78190	0.78424	0.79188	0.80010	0.80912
236	0.76024	0.77549	0.78259	0.78493	0.79255	0.80073	0.80972
237	0.76098	0.77618	0.78326	0.78560	0.79320	0.80136	0.81032
238	0.76174	0.77688	0.78395	0.78628	0.79384	0.80198	0.81091
239	0.76248	0.77759	0.78464	0.78697	0.79449	0.80260	0.81150
240	0.76322	0.77829	0.78531	0.78763	0.79514	0.80321	0.81209
241	0.76399	0.77899	0.78600	0.78830	0.79577	0.80382	0.81267
242	0.76473	0.77967	0.78666	0.78896	0.79641	0.80443	0.81324
243	0.76541	0.78039	0.78731	0.78962	0.79704	0.80502	0.81382
244	0.76619	0.78106	0.78797	0.79026	0.79766	0.80563	0.81438
245	0.76690	0.78174	0.78861	0.79090	0.79828	0.80622	0.81495
246	0.76764	0.78241	0.78927	0.79153	0.79890	0.80682	0.81551
247	0.76834	0.78309	0.78991	0.79216	0.79951	0.80740	0.81607
248	0.76908	0.78373	0.79054	0.79280	0.80012	0.80798	0.81663
249	0.76976	0.78437	0.79119	0.79342	0.80072	0.80856	0.81718
250	0.77045	0.78503	0.79180	0.79405	0.80132	0.80914	0.81773
251	0.77112	0.78567	0.79242	0.79469	0.80192	0.80971	0.81826
252	0.77184	0.78632	0.79303	0.79529	0.80252	0.81028	0.81881
253	0.77249	0.78697	0.79364	0.79590	0.80310	0.81084	0.81934
254	0.77320	0.78759	0.79426	0.79650	0.80368	0.81140	0.81988
255	0.77384	0.78824	0.79487	0.79710	0.80427	0.81195	0.82040
256	0.77454	0.78887	0.79548	0.79770	0.80485	0.81249	0.82094
257	0.77516	0.78948	0.79608	0.79831	0.80542	0.81305	0.82146
258	0.77586	0.79011	0.79670	0.79888	0.80598	0.81360	0.82197
259	0.77655	0.79072	0.79728	0.79946	0.80655	0.81414	0.82248
260	0.77721	0.79132	0.79787	0.80005	0.80712	0.81468	0.82300
261	0.77788	0.79192	0.79846	0.80063	0.80767	0.81521	0.82351
262	0.77853	0.79252	0.79903	0.80120	0.80822	0.81574	0.82401
263	0.77916	0.79313	0.79961	0.80178	0.80877	0.81626	0.82451
264	0.77981	0.79373	0.80019	0.80236	0.80932	0.81679	0.82501
265	0.78048	0.79431	0.80076	0.80291	0.80987	0.81732	0.82550
266	0.78112	0.79490	0.80134	0.80347	0.81041	0.81784	0.82600
267	0.78171	0.79548	0.80191	0.80404	0.81095	0.81835	0.82649
268	0.78236	0.79606	0.80247	0.80459	0.81148	0.81887	0.82698
269	0.78297	0.79664	0.80303	0.80515	0.81201	0.81938	0.82746
270	0.78355	0.79721	0.80359	0.80569	0.81253	0.81988	0.82794
271	0.78419	0.79777	0.80413	0.80624	0.81306	0.82038	0.82842
272	0.78480	0.79834	0.80470	0.80678	0.81358	0.82088	0.82889
273	0.78542	0.79889	0.80523	0.80732	0.81410	0.82138	0.82937
274	0.78599	0.79946	0.80578	0.80786	0.81461	0.82187	0.82984
275	0.78661	0.80002	0.80632	0.80838	0.81513	0.82236	0.83030
276	0.78719	0.80057	0.80684	0.80891	0.81564	0.82284	0.83076
277	0.78774	0.80113	0.80738	0.80943	0.81615	0.82333	0.83123
278	0.78836	0.80167	0.80791	0.80995	0.81665	0.82382	0.83169
279	0.78899	0.80221	0.80844	0.81047	0.81716	0.82430	0.83214
280	0.78953	0.80276	0.80895	0.81098	0.81765	0.82478	0.83260
281	0.79007	0.80333	0.80948	0.81150	0.81813	0.82525	0.83305
282	0.79067	0.80388	0.80999	0.81201	0.81864	0.82571	0.83349
283	0.79121	0.80441	0.81050	0.81253	0.81913	0.82618	0.83394
284	0.79181	0.80493	0.81100	0.81304	0.81961	0.82664	0.83438
285	0.79236	0.80546	0.81152	0.81353	0.82009	0.82710	0.83482
286	0.79287	0.80599	0.81203	0.81403	0.82057	0.82756	0.83526
287	0.79345	0.80649	0.81254	0.81453	0.82104	0.82802	0.83569
288	0.79400	0.80702	0.81302	0.81503	0.82152	0.82847	0.83612
289	0.79455	0.80755	0.81352	0.81551	0.82199	0.82893	0.83654

N	0.1%	0.5%	1%	1.25%	2.5%	5%	10%
290	0.79507	0.80804	0.81401	0.81600	0.82246	0.82937	0.83697
291	0.79564	0.80855	0.81449	0.81648	0.82293	0.82982	0.83740
292	0.79617	0.80905	0.81499	0.81696	0.82339	0.83026	0.83782
293	0.79668	0.80955	0.81546	0.81744	0.82386	0.83070	0.83824
294	0.79721	0.81004	0.81593	0.81792	0.82431	0.83114	0.83866
295	0.79775	0.81054	0.81642	0.81839	0.82477	0.83158	0.83907
296	0.79827	0.81103	0.81690	0.81886	0.82522	0.83202	0.83948
297	0.79878	0.81152	0.81737	0.81934	0.82567	0.83245	0.83989
298	0.79929	0.81200	0.81783	0.81980	0.82612	0.83288	0.84030
299	0.79982	0.81249	0.81830	0.82027	0.82657	0.83329	0.84070
300	0.80033	0.81297	0.81877	0.82072	0.82701	0.83372	0.84110
301	0.80087	0.81345	0.81923	0.82118	0.82745	0.83415	0.84150
302	0.80139	0.81392	0.81968	0.82164	0.82788	0.83456	0.84190
303	0.80192	0.81440	0.82015	0.82209	0.82831	0.83497	0.84230
304	0.80237	0.81485	0.82060	0.82254	0.82874	0.83539	0.84269
305	0.80288	0.81532	0.82105	0.82298	0.82917	0.83581	0.84308
306	0.80336	0.81579	0.82150	0.82342	0.82961	0.83622	0.84347
307	0.80388	0.81626	0.82195	0.82386	0.83003	0.83663	0.84386
308	0.80435	0.81672	0.82240	0.82430	0.83046	0.83703	0.84425
309	0.80489	0.81718	0.82285	0.82475	0.83088	0.83743	0.84463
310	0.80534	0.81764	0.82329	0.82518	0.83130	0.83783	0.84501
311	0.80583	0.81808	0.82373	0.82562	0.83171	0.83823	0.84539
312	0.80632	0.81852	0.82416	0.82604	0.83213	0.83863	0.84577
313	0.80679	0.81897	0.82460	0.82647	0.83254	0.83902	0.84614
314	0.80726	0.81942	0.82503	0.82690	0.83295	0.83942	0.84651
315	0.80774	0.81987	0.82546	0.82732	0.83335	0.83980	0.84689
316	0.80819	0.82031	0.82589	0.82774	0.83376	0.84019	0.84725
317	0.80868	0.82075	0.82632	0.82817	0.83417	0.84058	0.84763
318	0.80916	0.82118	0.82673	0.82859	0.83458	0.84096	0.84799
319	0.80957	0.82160	0.82715	0.82900	0.83497	0.84134	0.84835
320	0.81006	0.82204	0.82757	0.82941	0.83536	0.84172	0.84871
321	0.81051	0.82246	0.82800	0.82983	0.83577	0.84211	0.84907
322	0.81095	0.82288	0.82842	0.83024	0.83615	0.84248	0.84944
323	0.81139	0.82330	0.82884	0.83065	0.83654	0.84285	0.84979
324	0.81186	0.82373	0.82924	0.83106	0.83693	0.84322	0.85015
325	0.81233	0.82416	0.82965	0.83146	0.83732	0.84360	0.85050
326	0.81279	0.82459	0.83004	0.83186	0.83770	0.84396	0.85085
327	0.81324	0.82502	0.83045	0.83225	0.83808	0.84433	0.85119
328	0.81366	0.82541	0.83085	0.83264	0.83846	0.84469	0.85154
329	0.81412	0.82582	0.83125	0.83305	0.83883	0.84505	0.85188
330	0.81456	0.82623	0.83164	0.83343	0.83921	0.84541	0.85223
331	0.81498	0.82663	0.83203	0.83383	0.83958	0.84577	0.85256
332	0.81542	0.82704	0.83243	0.83421	0.83995	0.84613	0.85290
333	0.81580	0.82744	0.83282	0.83459	0.84032	0.84649	0.85324
334	0.81623	0.82783	0.83320	0.83497	0.84070	0.84684	0.85358
335	0.81665	0.82823	0.83359	0.83536	0.84106	0.84719	0.85391
336	0.81708	0.82864	0.83397	0.83575	0.84143	0.84755	0.85425
337	0.81755	0.82903	0.83435	0.83612	0.84179	0.84790	0.85458
338	0.81799	0.82945	0.83474	0.83649	0.84215	0.84824	0.85491
339	0.81843	0.82983	0.83511	0.83686	0.84251	0.84859	0.85524
340	0.81880	0.83022	0.83549	0.83723	0.84287	0.84893	0.85556
341	0.81923	0.83059	0.83586	0.83760	0.84323	0.84926	0.85589
342	0.81964	0.83098	0.83623	0.83798	0.84358	0.84960	0.85621
343	0.82007	0.83136	0.83660	0.83834	0.84394	0.84994	0.85653
344	0.82049	0.83175	0.83697	0.83870	0.84429	0.85028	0.85685
345	0.82091	0.83212	0.83734	0.83907	0.84464	0.85061	0.85717
346	0.82129	0.83251	0.83770	0.83944	0.84499	0.85094	0.85749
347	0.82171	0.83289	0.83807	0.83980	0.84534	0.85128	0.85780
348	0.82210	0.83327	0.83843	0.84015	0.84568	0.85161	0.85812
349	0.82250	0.83365	0.83879	0.84051	0.84602	0.85194	0.85843
350	0.82290	0.83401	0.83915	0.84086	0.84636	0.85226	0.85873
351	0.82329	0.83437	0.83951	0.84121	0.84671	0.85259	0.85905
352	0.82368	0.83475	0.83986	0.84156	0.84704	0.85291	0.85936
353	0.82407	0.83511	0.84022	0.84190	0.84737	0.85323	0.85967
354	0.82448	0.83547	0.84057	0.84226	0.84771	0.85355	0.85997
355	0.82485	0.83583	0.84092	0.84260	0.84805	0.85387	0.86027
356	0.82522	0.83620	0.84126	0.84294	0.84838	0.85419	0.86057
357	0.82558	0.83655	0.84162	0.84329	0.84871	0.85450	0.86087
358	0.82600	0.83691	0.84196	0.84363	0.84904	0.85481	0.86117
359	0.82639	0.83726	0.84230	0.84397	0.84936	0.85513	0.86147
360	0.82676	0.83762	0.84265	0.84430	0.84969	0.85544	0.86177
361	0.82713	0.83797	0.84299	0.84465	0.85001	0.85575	0.86206
362	0.82751	0.83833	0.84332	0.84499	0.85034	0.85606	0.86235
363	0.82788	0.83868	0.84366	0.84532	0.85066	0.85636	0.86264

N	0.1%	0.5%	1%	1.25%	2.5%	5%	10%
364	0.82824	0.83902	0.84400	0.84565	0.85098	0.85667	0.86293
365	0.82860	0.83936	0.84433	0.84597	0.85129	0.85697	0.86322
366	0.82899	0.83972	0.84466	0.84631	0.85161	0.85728	0.86351
367	0.82934	0.84006	0.84500	0.84663	0.85192	0.85758	0.86380
368	0.82970	0.84041	0.84532	0.84695	0.85224	0.85788	0.86409
369	0.83003	0.84075	0.84565	0.84727	0.85255	0.85818	0.86437
370	0.83041	0.84107	0.84598	0.84759	0.85286	0.85848	0.86465
371	0.83075	0.84141	0.84631	0.84792	0.85316	0.85877	0.86493
372	0.83109	0.84174	0.84662	0.84824	0.85347	0.85906	0.86521
373	0.83146	0.84207	0.84694	0.84856	0.85378	0.85935	0.86549
374	0.83182	0.84241	0.84727	0.84888	0.85408	0.85965	0.86577
375	0.83219	0.84274	0.84758	0.84919	0.85438	0.85994	0.86605
376	0.83253	0.84308	0.84791	0.84951	0.85468	0.86023	0.86632
377	0.83289	0.84339	0.84823	0.84982	0.85498	0.86052	0.86659
378	0.83325	0.84372	0.84854	0.85013	0.85527	0.86081	0.86687
379	0.83357	0.84404	0.84885	0.85044	0.85557	0.86109	0.86714
380	0.83391	0.84435	0.84916	0.85075	0.85588	0.86137	0.86741
381	0.83428	0.84468	0.84947	0.85106	0.85616	0.86166	0.86768
382	0.83462	0.84500	0.84978	0.85137	0.85646	0.86194	0.86795
383	0.83493	0.84531	0.85009	0.85167	0.85676	0.86222	0.86822
384	0.83529	0.84563	0.85040	0.85196	0.85705	0.86250	0.86848
385	0.83565	0.84594	0.85070	0.85226	0.85734	0.86278	0.86875
386	0.83599	0.84625	0.85102	0.85257	0.85762	0.86305	0.86901
387	0.83633	0.84656	0.85131	0.85287	0.85792	0.86333	0.86927
388	0.83667	0.84687	0.85161	0.85316	0.85820	0.86360	0.86953
389	0.83698	0.84718	0.85190	0.85345	0.85849	0.86387	0.86980
390	0.83731	0.84749	0.85220	0.85375	0.85877	0.86415	0.87005
391	0.83765	0.84781	0.85250	0.85404	0.85904	0.86442	0.87031
392	0.83798	0.84811	0.85279	0.85434	0.85933	0.86468	0.87056
393	0.83831	0.84841	0.85308	0.85463	0.85961	0.86495	0.87082
394	0.83865	0.84872	0.85337	0.85492	0.85988	0.86522	0.87107
395	0.83897	0.84901	0.85366	0.85520	0.86016	0.86549	0.87132
396	0.83928	0.84932	0.85395	0.85550	0.86044	0.86575	0.87157
397	0.83961	0.84962	0.85424	0.85578	0.86071	0.86601	0.87182
398	0.83992	0.84992	0.85453	0.85605	0.86099	0.86628	0.87208
399	0.84024	0.85021	0.85482	0.85634	0.86126	0.86654	0.87232
400	0.84056	0.85051	0.85510	0.85661	0.86153	0.86680	0.87257
401	0.84087	0.85080	0.85538	0.85690	0.86180	0.86706	0.87282
402	0.84123	0.85109	0.85567	0.85719	0.86207	0.86732	0.87306
403	0.84153	0.85138	0.85595	0.85746	0.86234	0.86757	0.87331
404	0.84183	0.85168	0.85623	0.85774	0.86261	0.86783	0.87355
405	0.84212	0.85196	0.85651	0.85802	0.86288	0.86809	0.87379
406	0.84243	0.85225	0.85679	0.85829	0.86315	0.86834	0.87404
407	0.84272	0.85255	0.85707	0.85857	0.86341	0.86859	0.87427
408	0.84302	0.85284	0.85736	0.85885	0.86367	0.86883	0.87452
409	0.84332	0.85313	0.85762	0.85911	0.86393	0.86909	0.87475
410	0.84361	0.85341	0.85788	0.85938	0.86419	0.86934	0.87499
411	0.84393	0.85370	0.85816	0.85964	0.86445	0.86959	0.87523
412	0.84423	0.85398	0.85843	0.85992	0.86471	0.86983	0.87546
413	0.84453	0.85426	0.85870	0.86018	0.86496	0.87008	0.87570
414	0.84480	0.85453	0.85897	0.86045	0.86522	0.87032	0.87593
415	0.84510	0.85480	0.85924	0.86071	0.86547	0.87057	0.87616
416	0.84540	0.85508	0.85950	0.86097	0.86572	0.87081	0.87639
417	0.84571	0.85535	0.85977	0.86123	0.86598	0.87105	0.87662
418	0.84600	0.85562	0.86004	0.86149	0.86623	0.87129	0.87685
419	0.84631	0.85588	0.86030	0.86175	0.86648	0.87153	0.87708
420	0.84660	0.85616	0.86056	0.86202	0.86673	0.87177	0.87730
421	0.84688	0.85643	0.86082	0.86227	0.86698	0.87201	0.87753
422	0.84718	0.85670	0.86108	0.86253	0.86723	0.87225	0.87776
423	0.84748	0.85697	0.86134	0.86278	0.86747	0.87248	0.87798
424	0.84776	0.85724	0.86159	0.86304	0.86772	0.87271	0.87821
425	0.84804	0.85751	0.86184	0.86329	0.86797	0.87295	0.87843
426	0.84836	0.85778	0.86210	0.86355	0.86821	0.87318	0.87865
427	0.84862	0.85804	0.86236	0.86380	0.86845	0.87342	0.87888
428	0.84890	0.85830	0.86261	0.86405	0.86870	0.87365	0.87910
429	0.84921	0.85856	0.86286	0.86429	0.86894	0.87388	0.87932
430	0.84949	0.85882	0.86312	0.86454	0.86917	0.87410	0.87954
431	0.84976	0.85909	0.86337	0.86480	0.86941	0.87433	0.87976
432	0.85004	0.85935	0.86362	0.86505	0.86965	0.87456	0.87997
433	0.85029	0.85961	0.86388	0.86530	0.86988	0.87479	0.88019
434	0.85055	0.85987	0.86413	0.86554	0.87012	0.87502	0.88040
435	0.85085	0.86012	0.86437	0.86579	0.87035	0.87524	0.88061
436	0.85111	0.86038	0.86462	0.86603	0.87059	0.87547	0.88082
437	0.85139	0.86063	0.86487	0.86628	0.87082	0.87569	0.88104

N	0.1%	0.5%	1%	1.25%	2.5%	5%	10%
438	0.85166	0.86088	0.86511	0.86651	0.87105	0.87591	0.88125
439	0.85193	0.86113	0.86535	0.86676	0.87128	0.87614	0.88146
440	0.85221	0.86138	0.86558	0.86699	0.87152	0.87635	0.88167
441	0.85246	0.86164	0.86582	0.86723	0.87174	0.87657	0.88188
442	0.85274	0.86188	0.86606	0.86746	0.87197	0.87680	0.88209
443	0.85301	0.86213	0.86631	0.86770	0.87220	0.87701	0.88230
444	0.85326	0.86237	0.86655	0.86794	0.87243	0.87723	0.88250
445	0.85354	0.86261	0.86679	0.86817	0.87265	0.87745	0.88271
446	0.85380	0.86286	0.86702	0.86841	0.87288	0.87766	0.88291
447	0.85405	0.86311	0.86726	0.86865	0.87311	0.87788	0.88312
448	0.85433	0.86334	0.86750	0.86888	0.87333	0.87809	0.88332
449	0.85461	0.86359	0.86773	0.86911	0.87355	0.87830	0.88352
450	0.85487	0.86383	0.86797	0.86935	0.87377	0.87851	0.88372
451	0.85513	0.86407	0.86820	0.86958	0.87399	0.87872	0.88393
452	0.85539	0.86431	0.86843	0.86980	0.87421	0.87893	0.88413
453	0.85563	0.86455	0.86866	0.87002	0.87443	0.87915	0.88433
454	0.85589	0.86479	0.86889	0.87025	0.87465	0.87935	0.88453
455	0.85615	0.86502	0.86912	0.87048	0.87487	0.87956	0.88472
456	0.85641	0.86525	0.86935	0.87070	0.87509	0.87977	0.88492
457	0.85667	0.86549	0.86957	0.87093	0.87531	0.87998	0.88512
458	0.85691	0.86572	0.86979	0.87115	0.87552	0.88018	0.88531
459	0.85714	0.86596	0.87001	0.87137	0.87573	0.88038	0.88551
460	0.85741	0.86619	0.87024	0.87159	0.87594	0.88059	0.88570
461	0.85767	0.86643	0.87047	0.87181	0.87616	0.88079	0.88590
462	0.85790	0.86665	0.87069	0.87204	0.87637	0.88099	0.88609
463	0.85815	0.86688	0.87092	0.87226	0.87658	0.88120	0.88628
464	0.85839	0.86712	0.87114	0.87247	0.87679	0.88140	0.88647
465	0.85862	0.86734	0.87136	0.87269	0.87700	0.88160	0.88667
466	0.85886	0.86758	0.87157	0.87291	0.87721	0.88180	0.88686
467	0.85910	0.86780	0.87179	0.87312	0.87742	0.88200	0.88705
468	0.85934	0.86801	0.87200	0.87334	0.87762	0.88220	0.88724
469	0.85958	0.86823	0.87222	0.87356	0.87783	0.88240	0.88742
470	0.85980	0.86845	0.87244	0.87376	0.87803	0.88259	0.88761
471	0.86004	0.86868	0.87266	0.87398	0.87824	0.88279	0.88780
472	0.86028	0.86891	0.87286	0.87418	0.87844	0.88299	0.88798
473	0.86052	0.86913	0.87308	0.87439	0.87864	0.88318	0.88817
474	0.86076	0.86934	0.87329	0.87460	0.87885	0.88338	0.88835
475	0.86099	0.86956	0.87351	0.87482	0.87904	0.88357	0.88853
476	0.86124	0.86978	0.87372	0.87503	0.87925	0.88376	0.88872
477	0.86144	0.87000	0.87393	0.87523	0.87945	0.88395	0.88890
478	0.86168	0.87021	0.87414	0.87544	0.87965	0.88415	0.88908
479	0.86193	0.87042	0.87435	0.87565	0.87985	0.88434	0.88927
480	0.86217	0.87064	0.87457	0.87586	0.88005	0.88453	0.88945
481	0.86241	0.87086	0.87477	0.87606	0.88025	0.88472	0.88963
482	0.86265	0.87108	0.87498	0.87626	0.88045	0.88491	0.88981
483	0.86286	0.87129	0.87518	0.87647	0.88064	0.88510	0.88999
484	0.86309	0.87149	0.87538	0.87667	0.88084	0.88528	0.89016
485	0.86331	0.87171	0.87559	0.87687	0.88103	0.88547	0.89034
486	0.86353	0.87192	0.87580	0.87707	0.88122	0.88566	0.89052
487	0.86376	0.87212	0.87600	0.87727	0.88142	0.88584	0.89070
488	0.86399	0.87234	0.87620	0.87747	0.88161	0.88603	0.89087
489	0.86420	0.87255	0.87640	0.87767	0.88180	0.88621	0.89105
490	0.86443	0.87276	0.87660	0.87787	0.88199	0.88639	0.89122
491	0.86465	0.87297	0.87680	0.87806	0.88218	0.88658	0.89139
492	0.86487	0.87317	0.87700	0.87826	0.88237	0.88676	0.89157
493	0.86511	0.87338	0.87720	0.87846	0.88255	0.88694	0.89174
494	0.86531	0.87358	0.87740	0.87866	0.88275	0.88712	0.89191
495	0.86556	0.87379	0.87759	0.87886	0.88294	0.88730	0.89209
496	0.86575	0.87399	0.87779	0.87905	0.88312	0.88748	0.89226
497	0.86599	0.87419	0.87799	0.87925	0.88331	0.88766	0.89243
498	0.86620	0.87440	0.87819	0.87944	0.88350	0.88784	0.89260
499	0.86642	0.87460	0.87838	0.87963	0.88369	0.88802	0.89277
500	0.86664	0.87480	0.87858	0.87983	0.88387	0.88820	0.89293
501	0.86684	0.87501	0.87877	0.88002	0.88405	0.88838	0.89310
502	0.86704	0.87521	0.87897	0.88021	0.88424	0.88855	0.89327
503	0.86725	0.87541	0.87916	0.88040	0.88442	0.88873	0.89344
504	0.86747	0.87560	0.87935	0.88059	0.88460	0.88890	0.89360
505	0.86766	0.87579	0.87954	0.88078	0.88478	0.88908	0.89377
506	0.86788	0.87598	0.87973	0.88097	0.88496	0.88925	0.89394
507	0.86810	0.87617	0.87993	0.88116	0.88514	0.88942	0.89410
508	0.86833	0.87638	0.88012	0.88134	0.88532	0.88960	0.89427
509	0.86853	0.87657	0.88030	0.88152	0.88550	0.88977	0.89443
510	0.86874	0.87677	0.88048	0.88171	0.88568	0.88994	0.89459
511	0.86893	0.87696	0.88068	0.88189	0.88586	0.89011	0.89475

N	0.1%	0.5%	1%	1.25%	2.5%	5%	10%
512	0.86912	0.87715	0.88087	0.88208	0.88603	0.89027	0.89492
513	0.86932	0.87734	0.88105	0.88226	0.88621	0.89044	0.89508
514	0.86953	0.87753	0.88123	0.88244	0.88639	0.89061	0.89524
515	0.86973	0.87772	0.88142	0.88263	0.88656	0.89078	0.89540
516	0.86994	0.87791	0.88160	0.88281	0.88673	0.89095	0.89556
517	0.87015	0.87810	0.88178	0.88299	0.88691	0.89112	0.89571
518	0.87034	0.87829	0.88196	0.88318	0.88708	0.89128	0.89588
519	0.87054	0.87848	0.88214	0.88335	0.88726	0.89145	0.89603
520	0.87074	0.87867	0.88232	0.88353	0.88743	0.89162	0.89619
521	0.87096	0.87886	0.88250	0.88371	0.88760	0.89178	0.89635
522	0.87115	0.87905	0.88269	0.88389	0.88777	0.89195	0.89651
523	0.87135	0.87924	0.88287	0.88407	0.88794	0.89211	0.89666
524	0.87156	0.87942	0.88305	0.88425	0.88811	0.89227	0.89682
525	0.87176	0.87961	0.88323	0.88443	0.88829	0.89244	0.89697
526	0.87197	0.87979	0.88341	0.88461	0.88846	0.89260	0.89713
527	0.87216	0.87998	0.88359	0.88478	0.88863	0.89276	0.89728
528	0.87235	0.88016	0.88377	0.88495	0.88880	0.89292	0.89743
529	0.87254	0.88035	0.88394	0.88513	0.88896	0.89308	0.89759
530	0.87273	0.88053	0.88412	0.88530	0.88913	0.89324	0.89774
531	0.87292	0.88071	0.88429	0.88547	0.88930	0.89339	0.89789
532	0.87310	0.88089	0.88447	0.88564	0.88946	0.89355	0.89804
533	0.87332	0.88107	0.88464	0.88581	0.88963	0.89371	0.89819
534	0.87351	0.88125	0.88481	0.88599	0.88979	0.89387	0.89834
535	0.87370	0.88144	0.88498	0.88616	0.88996	0.89403	0.89849
536	0.87391	0.88162	0.88515	0.88633	0.89012	0.89418	0.89864
537	0.87408	0.88180	0.88532	0.88650	0.89028	0.89434	0.89879
538	0.87428	0.88197	0.88550	0.88667	0.89044	0.89450	0.89894
539	0.87445	0.88215	0.88567	0.88684	0.89061	0.89465	0.89909
540	0.87464	0.88234	0.88584	0.88701	0.89077	0.89481	0.89923
541	0.87483	0.88251	0.88601	0.88717	0.89093	0.89496	0.89938
542	0.87500	0.88268	0.88617	0.88734	0.89109	0.89512	0.89953
543	0.87519	0.88285	0.88634	0.88751	0.89125	0.89527	0.89967
544	0.87539	0.88302	0.88652	0.88767	0.89141	0.89543	0.89982
545	0.87557	0.88319	0.88668	0.88784	0.89157	0.89558	0.89996
546	0.87574	0.88335	0.88685	0.88800	0.89173	0.89573	0.90011
547	0.87594	0.88352	0.88702	0.88816	0.89189	0.89588	0.90026
548	0.87611	0.88369	0.88718	0.88832	0.89205	0.89604	0.90040
549	0.87630	0.88386	0.88735	0.88849	0.89220	0.89619	0.90054
550	0.87649	0.88403	0.88751	0.88865	0.89236	0.89634	0.90069
551	0.87667	0.88420	0.88767	0.88882	0.89252	0.89649	0.90083
552	0.87686	0.88438	0.88784	0.88898	0.89267	0.89663	0.90097
553	0.87704	0.88455	0.88800	0.88914	0.89283	0.89678	0.90111
554	0.87722	0.88471	0.88816	0.88930	0.89298	0.89693	0.90126
555	0.87741	0.88488	0.88832	0.88946	0.89314	0.89708	0.90140
556	0.87758	0.88505	0.88848	0.88962	0.89329	0.89722	0.90153
557	0.87776	0.88521	0.88865	0.88978	0.89344	0.89737	0.90168
558	0.87795	0.88538	0.88880	0.88994	0.89360	0.89752	0.90181
559	0.87812	0.88554	0.88897	0.89010	0.89375	0.89766	0.90195
560	0.87829	0.88571	0.88913	0.89026	0.89390	0.89781	0.90209
561	0.87848	0.88587	0.88930	0.89042	0.89405	0.89796	0.90223
562	0.87864	0.88603	0.88945	0.89057	0.89420	0.89810	0.90237
563	0.87882	0.88621	0.88961	0.89073	0.89435	0.89824	0.90251
564	0.87897	0.88636	0.88976	0.89089	0.89451	0.89839	0.90264
565	0.87913	0.88653	0.88992	0.89104	0.89465	0.89853	0.90278
566	0.87930	0.88670	0.89008	0.89120	0.89480	0.89867	0.90292
567	0.87947	0.88687	0.89023	0.89135	0.89495	0.89881	0.90305
568	0.87967	0.88702	0.89039	0.89151	0.89510	0.89896	0.90319
569	0.87983	0.88719	0.89054	0.89166	0.89524	0.89910	0.90332
570	0.87999	0.88734	0.89070	0.89181	0.89539	0.89924	0.90346
571	0.88015	0.88750	0.89085	0.89196	0.89554	0.89938	0.90359
572	0.88031	0.88766	0.89100	0.89212	0.89569	0.89952	0.90373
573	0.88047	0.88781	0.89116	0.89226	0.89583	0.89966	0.90386
574	0.88065	0.88797	0.89131	0.89241	0.89598	0.89980	0.90399
575	0.88082	0.88812	0.89146	0.89257	0.89612	0.89994	0.90412
576	0.88099	0.88827	0.89162	0.89271	0.89627	0.90008	0.90425
577	0.88115	0.88843	0.89177	0.89286	0.89641	0.90021	0.90438
578	0.88133	0.88859	0.89192	0.89301	0.89656	0.90035	0.90451
579	0.88149	0.88874	0.89207	0.89316	0.89670	0.90049	0.90465
580	0.88168	0.88890	0.89221	0.89330	0.89684	0.90063	0.90478
581	0.88185	0.88905	0.89237	0.89345	0.89698	0.90077	0.90491
582	0.88203	0.88920	0.89251	0.89360	0.89713	0.90090	0.90503
583	0.88217	0.88936	0.89266	0.89374	0.89727	0.90104	0.90516
584	0.88233	0.88951	0.89280	0.89389	0.89741	0.90117	0.90529
585	0.88251	0.88967	0.89295	0.89404	0.89755	0.90131	0.90542

N	0.1%	0.5%	1%	1.25%	2.5%	5%	10%
586	0.88268	0.88982	0.89310	0.89418	0.89769	0.90144	0.90555
587	0.88282	0.88996	0.89324	0.89433	0.89783	0.90157	0.90568
588	0.88299	0.89012	0.89339	0.89448	0.89797	0.90170	0.90580
589	0.88315	0.89027	0.89354	0.89462	0.89811	0.90184	0.90593
590	0.88331	0.89041	0.89368	0.89477	0.89825	0.90197	0.90606
591	0.88347	0.89057	0.89382	0.89491	0.89839	0.90210	0.90619
592	0.88362	0.89072	0.89397	0.89505	0.89853	0.90223	0.90631
593	0.88378	0.89087	0.89411	0.89519	0.89866	0.90237	0.90644
594	0.88397	0.89102	0.89426	0.89534	0.89880	0.90250	0.90656
595	0.88414	0.89117	0.89441	0.89548	0.89893	0.90263	0.90669
596	0.88428	0.89131	0.89455	0.89562	0.89907	0.90276	0.90681
597	0.88445	0.89146	0.89469	0.89575	0.89921	0.90289	0.90694
598	0.88460	0.89161	0.89483	0.89589	0.89934	0.90302	0.90706
599	0.88475	0.89175	0.89497	0.89604	0.89948	0.90315	0.90719
600	0.88490	0.89190	0.89512	0.89618	0.89961	0.90327	0.90731
601	0.88504	0.89205	0.89525	0.89631	0.89974	0.90340	0.90743
602	0.88518	0.89219	0.89539	0.89645	0.89988	0.90353	0.90755
603	0.88535	0.89233	0.89553	0.89659	0.90001	0.90366	0.90767
604	0.88552	0.89248	0.89567	0.89673	0.90014	0.90379	0.90780
605	0.88565	0.89262	0.89581	0.89687	0.90027	0.90392	0.90792
606	0.88580	0.89276	0.89594	0.89700	0.90040	0.90404	0.90804
607	0.88596	0.89290	0.89608	0.89715	0.90053	0.90417	0.90816
608	0.88610	0.89304	0.89622	0.89728	0.90067	0.90430	0.90828
609	0.88626	0.89319	0.89636	0.89741	0.90080	0.90442	0.90840
610	0.88640	0.89333	0.89649	0.89754	0.90093	0.90455	0.90852
611	0.88654	0.89347	0.89663	0.89768	0.90106	0.90467	0.90864
612	0.88670	0.89361	0.89677	0.89781	0.90119	0.90480	0.90876
613	0.88685	0.89375	0.89690	0.89795	0.90132	0.90492	0.90888
614	0.88701	0.89388	0.89704	0.89808	0.90145	0.90505	0.90899
615	0.88716	0.89403	0.89717	0.89822	0.90158	0.90517	0.90911
616	0.88730	0.89417	0.89731	0.89835	0.90171	0.90529	0.90923
617	0.88745	0.89431	0.89744	0.89848	0.90184	0.90541	0.90934
618	0.88761	0.89445	0.89758	0.89862	0.90196	0.90553	0.90946
619	0.88776	0.89458	0.89771	0.89875	0.90209	0.90566	0.90958
620	0.88791	0.89472	0.89785	0.89888	0.90222	0.90578	0.90970
621	0.88805	0.89485	0.89798	0.89901	0.90235	0.90590	0.90981
622	0.88821	0.89499	0.89811	0.89915	0.90247	0.90602	0.90993
623	0.88833	0.89514	0.89825	0.89927	0.90260	0.90614	0.91005
624	0.88848	0.89527	0.89838	0.89941	0.90272	0.90626	0.91016
625	0.88862	0.89541	0.89850	0.89954	0.90285	0.90638	0.91027
626	0.88875	0.89554	0.89864	0.89967	0.90297	0.90650	0.91039
627	0.88891	0.89568	0.89876	0.89980	0.90310	0.90662	0.91050
628	0.88904	0.89582	0.89890	0.89993	0.90323	0.90674	0.91061
629	0.88919	0.89595	0.89903	0.90006	0.90335	0.90686	0.91073
630	0.88934	0.89608	0.89915	0.90019	0.90347	0.90698	0.91084
631	0.88948	0.89621	0.89928	0.90031	0.90359	0.90710	0.91095
632	0.88963	0.89634	0.89941	0.90043	0.90371	0.90722	0.91107
633	0.88977	0.89648	0.89954	0.90056	0.90384	0.90734	0.91118
634	0.88991	0.89660	0.89967	0.90069	0.90396	0.90745	0.91129
635	0.89007	0.89673	0.89980	0.90081	0.90408	0.90757	0.91140
636	0.89021	0.89687	0.89993	0.90094	0.90420	0.90768	0.91151
637	0.89035	0.89700	0.90005	0.90107	0.90432	0.90780	0.91162
638	0.89050	0.89713	0.90018	0.90119	0.90445	0.90792	0.91173
639	0.89063	0.89726	0.90030	0.90131	0.90457	0.90803	0.91184
640	0.89077	0.89738	0.90043	0.90144	0.90469	0.90815	0.91195
641	0.89091	0.89751	0.90055	0.90156	0.90480	0.90826	0.91206
642	0.89105	0.89764	0.90068	0.90169	0.90492	0.90838	0.91217
643	0.89119	0.89777	0.90080	0.90181	0.90504	0.90849	0.91228
644	0.89132	0.89790	0.90092	0.90193	0.90516	0.90860	0.91239
645	0.89146	0.89803	0.90104	0.90205	0.90528	0.90872	0.91250
646	0.89160	0.89816	0.90117	0.90218	0.90539	0.90883	0.91260
647	0.89177	0.89828	0.90129	0.90230	0.90551	0.90894	0.91271
648	0.89189	0.89841	0.90141	0.90242	0.90563	0.90906	0.91282
649	0.89203	0.89854	0.90154	0.90255	0.90575	0.90917	0.91293
650	0.89217	0.89867	0.90166	0.90267	0.90587	0.90928	0.91303
651	0.89230	0.89879	0.90178	0.90279	0.90599	0.90939	0.91314
652	0.89243	0.89892	0.90190	0.90291	0.90610	0.90951	0.91325
653	0.89257	0.89905	0.90202	0.90303	0.90622	0.90962	0.91335
654	0.89271	0.89918	0.90215	0.90315	0.90633	0.90973	0.91346
655	0.89284	0.89930	0.90227	0.90327	0.90645	0.90984	0.91357
656	0.89297	0.89943	0.90239	0.90339	0.90656	0.90995	0.91367
657	0.89311	0.89955	0.90251	0.90350	0.90668	0.91006	0.91378
658	0.89325	0.89967	0.90263	0.90362	0.90679	0.91017	0.91388
659	0.89337	0.89980	0.90275	0.90374	0.90691	0.91028	0.91399

N	0.1%	0.5%	1%	1.25%	2.5%	5%	10%
660	0.89351	0.89992	0.90287	0.90386	0.90702	0.91039	0.91409
661	0.89364	0.90005	0.90299	0.90398	0.90713	0.91050	0.91419
662	0.89377	0.90016	0.90311	0.90409	0.90725	0.91061	0.91430
663	0.89391	0.90029	0.90322	0.90421	0.90736	0.91071	0.91440
664	0.89403	0.90041	0.90334	0.90433	0.90747	0.91082	0.91450
665	0.89416	0.90053	0.90346	0.90445	0.90758	0.91093	0.91461
666	0.89430	0.90065	0.90358	0.90456	0.90770	0.91104	0.91471
667	0.89443	0.90077	0.90370	0.90468	0.90781	0.91114	0.91481
668	0.89456	0.90089	0.90381	0.90479	0.90792	0.91125	0.91491
669	0.89468	0.90102	0.90392	0.90491	0.90803	0.91136	0.91502
670	0.89480	0.90114	0.90404	0.90502	0.90814	0.91146	0.91512
671	0.89493	0.90126	0.90416	0.90514	0.90825	0.91157	0.91522
672	0.89507	0.90138	0.90428	0.90525	0.90837	0.91168	0.91532
673	0.89520	0.90149	0.90440	0.90537	0.90848	0.91178	0.91542
674	0.89531	0.90161	0.90450	0.90547	0.90858	0.91189	0.91552
675	0.89544	0.90173	0.90462	0.90559	0.90869	0.91199	0.91562
676	0.89558	0.90184	0.90474	0.90570	0.90880	0.91210	0.91572
677	0.89570	0.90197	0.90485	0.90582	0.90891	0.91220	0.91581
678	0.89583	0.90209	0.90496	0.90593	0.90902	0.91230	0.91592
679	0.89595	0.90221	0.90508	0.90604	0.90913	0.91241	0.91601
680	0.89608	0.90232	0.90519	0.90615	0.90923	0.91251	0.91611
681	0.89622	0.90245	0.90531	0.90626	0.90934	0.91262	0.91621
682	0.89634	0.90256	0.90542	0.90637	0.90945	0.91272	0.91631
683	0.89646	0.90267	0.90554	0.90648	0.90956	0.91282	0.91641
684	0.89658	0.90279	0.90564	0.90660	0.90966	0.91292	0.91650
685	0.89672	0.90291	0.90576	0.90671	0.90977	0.91302	0.91660
686	0.89683	0.90303	0.90587	0.90682	0.90988	0.91313	0.91670
687	0.89696	0.90314	0.90598	0.90693	0.90998	0.91323	0.91679
688	0.89708	0.90325	0.90610	0.90704	0.91009	0.91333	0.91689
689	0.89720	0.90336	0.90621	0.90715	0.91019	0.91343	0.91699
690	0.89732	0.90348	0.90632	0.90726	0.91030	0.91353	0.91708
691	0.89743	0.90359	0.90643	0.90736	0.91040	0.91363	0.91718
692	0.89756	0.90371	0.90654	0.90747	0.91050	0.91374	0.91728
693	0.89768	0.90382	0.90664	0.90758	0.91061	0.91384	0.91737
694	0.89781	0.90393	0.90675	0.90769	0.91072	0.91394	0.91747
695	0.89792	0.90405	0.90686	0.90780	0.91082	0.91403	0.91756
696	0.89805	0.90416	0.90698	0.90791	0.91092	0.91413	0.91766
697	0.89817	0.90427	0.90708	0.90801	0.91103	0.91423	0.91775
698	0.89827	0.90438	0.90719	0.90812	0.91113	0.91433	0.91785
699	0.89839	0.90450	0.90730	0.90823	0.91123	0.91443	0.91794
700	0.89852	0.90461	0.90741	0.90833	0.91133	0.91453	0.91804
701	0.89862	0.90472	0.90751	0.90844	0.91143	0.91463	0.91813
702	0.89874	0.90483	0.90762	0.90855	0.91154	0.91472	0.91822
703	0.89886	0.90495	0.90773	0.90865	0.91164	0.91482	0.91831
704	0.89899	0.90505	0.90783	0.90876	0.91174	0.91492	0.91841
705	0.89910	0.90517	0.90794	0.90886	0.91184	0.91502	0.91850
706	0.89922	0.90528	0.90805	0.90897	0.91194	0.91511	0.91859
707	0.89933	0.90538	0.90815	0.90907	0.91204	0.91521	0.91869
708	0.89946	0.90549	0.90826	0.90918	0.91215	0.91530	0.91878
709	0.89957	0.90560	0.90837	0.90929	0.91225	0.91540	0.91887
710	0.89969	0.90571	0.90847	0.90939	0.91234	0.91549	0.91896
711	0.89982	0.90582	0.90858	0.90949	0.91244	0.91559	0.91905
712	0.89993	0.90593	0.90868	0.90959	0.91254	0.91569	0.91914
713	0.90005	0.90603	0.90879	0.90970	0.91265	0.91578	0.91923
714	0.90016	0.90614	0.90889	0.90980	0.91274	0.91588	0.91932
715	0.90027	0.90624	0.90899	0.90990	0.91284	0.91598	0.91941
716	0.90038	0.90635	0.90910	0.91000	0.91294	0.91607	0.91950
717	0.90049	0.90646	0.90920	0.91010	0.91304	0.91616	0.91959
718	0.90062	0.90656	0.90930	0.91020	0.91314	0.91626	0.91968
719	0.90073	0.90667	0.90941	0.91030	0.91324	0.91635	0.91977
720	0.90084	0.90678	0.90951	0.91041	0.91333	0.91645	0.91986
721	0.90095	0.90688	0.90962	0.91051	0.91343	0.91654	0.91995
722	0.90107	0.90698	0.90972	0.91061	0.91353	0.91663	0.92004
723	0.90118	0.90709	0.90982	0.91072	0.91362	0.91672	0.92013
724	0.90129	0.90720	0.90992	0.91082	0.91372	0.91682	0.92022
725	0.90140	0.90730	0.91002	0.91092	0.91382	0.91691	0.92031
726	0.90151	0.90740	0.91012	0.91102	0.91391	0.91700	0.92039
727	0.90162	0.90751	0.91022	0.91112	0.91401	0.91709	0.92048
728	0.90172	0.90762	0.91032	0.91122	0.91411	0.91719	0.92057
729	0.90184	0.90772	0.91042	0.91132	0.91420	0.91728	0.92066
730	0.90196	0.90783	0.91052	0.91142	0.91430	0.91737	0.92074
731	0.90207	0.90793	0.91062	0.91152	0.91439	0.91746	0.92083
732	0.90219	0.90804	0.91072	0.91162	0.91449	0.91755	0.92092
733	0.90228	0.90814	0.91082	0.91171	0.91458	0.91764	0.92100

N	0.1%	0.5%	1%	1.25%	2.5%	5%	10%
734	0.90239	0.90824	0.91092	0.91181	0.91467	0.91773	0.92109
735	0.90250	0.90834	0.91102	0.91191	0.91477	0.91782	0.92118
736	0.90260	0.90844	0.91112	0.91200	0.91486	0.91791	0.92126
737	0.90271	0.90854	0.91122	0.91210	0.91496	0.91800	0.92135
738	0.90282	0.90864	0.91132	0.91220	0.91505	0.91809	0.92143
739	0.90294	0.90875	0.91141	0.91229	0.91515	0.91818	0.92152
740	0.90304	0.90885	0.91151	0.91239	0.91524	0.91827	0.92160
741	0.90317	0.90895	0.91161	0.91248	0.91533	0.91836	0.92169
742	0.90328	0.90905	0.91171	0.91258	0.91542	0.91845	0.92177
743	0.90339	0.90915	0.91180	0.91268	0.91551	0.91853	0.92186
744	0.90349	0.90925	0.91190	0.91277	0.91560	0.91862	0.92194
745	0.90359	0.90935	0.91199	0.91287	0.91570	0.91871	0.92203
746	0.90370	0.90945	0.91209	0.91297	0.91579	0.91880	0.92211
747	0.90380	0.90954	0.91219	0.91306	0.91588	0.91889	0.92219
748	0.90391	0.90964	0.91229	0.91316	0.91597	0.91897	0.92227
749	0.90401	0.90974	0.91238	0.91326	0.91606	0.91906	0.92236
750	0.90412	0.90984	0.91248	0.91335	0.91616	0.91915	0.92244
751	0.90423	0.90994	0.91257	0.91344	0.91625	0.91924	0.92253
752	0.90434	0.91005	0.91267	0.91354	0.91634	0.91932	0.92261
753	0.90444	0.91014	0.91276	0.91363	0.91643	0.91941	0.92269
754	0.90455	0.91024	0.91285	0.91372	0.91652	0.91950	0.92277
755	0.90466	0.91034	0.91295	0.91382	0.91661	0.91958	0.92286
756	0.90476	0.91043	0.91305	0.91391	0.91670	0.91967	0.92294
757	0.90486	0.91053	0.91314	0.91400	0.91679	0.91975	0.92302
758	0.90496	0.91062	0.91323	0.91410	0.91688	0.91984	0.92310
759	0.90506	0.91072	0.91333	0.91419	0.91697	0.91992	0.92318
760	0.90516	0.91081	0.91342	0.91429	0.91705	0.92001	0.92326
761	0.90527	0.91091	0.91351	0.91438	0.91714	0.92010	0.92334
762	0.90537	0.91100	0.91361	0.91447	0.91723	0.92018	0.92343
763	0.90548	0.91109	0.91370	0.91456	0.91732	0.92026	0.92351
764	0.90558	0.91119	0.91379	0.91465	0.91741	0.92035	0.92359
765	0.90568	0.91128	0.91388	0.91474	0.91749	0.92043	0.92367
766	0.90577	0.91138	0.91397	0.91483	0.91758	0.92052	0.92375
767	0.90587	0.91148	0.91407	0.91492	0.91767	0.92060	0.92383
768	0.90598	0.91157	0.91416	0.91502	0.91775	0.92068	0.92391
769	0.90608	0.91167	0.91425	0.91511	0.91784	0.92077	0.92399
770	0.90618	0.91176	0.91434	0.91520	0.91792	0.92085	0.92407
771	0.90628	0.91185	0.91443	0.91529	0.91801	0.92093	0.92415
772	0.90638	0.91194	0.91452	0.91538	0.91810	0.92101	0.92422
773	0.90647	0.91203	0.91461	0.91547	0.91818	0.92110	0.92430
774	0.90658	0.91213	0.91470	0.91555	0.91827	0.92118	0.92438
775	0.90668	0.91222	0.91479	0.91564	0.91835	0.92126	0.92446
776	0.90677	0.91231	0.91487	0.91573	0.91844	0.92134	0.92454
777	0.90687	0.91240	0.91497	0.91582	0.91852	0.92143	0.92462
778	0.90697	0.91249	0.91505	0.91590	0.91861	0.92151	0.92469
779	0.90708	0.91258	0.91514	0.91599	0.91869	0.92159	0.92477
780	0.90717	0.91268	0.91523	0.91608	0.91878	0.92167	0.92485
781	0.90727	0.91277	0.91532	0.91616	0.91886	0.92175	0.92493
782	0.90738	0.91287	0.91541	0.91625	0.91895	0.92184	0.92500
783	0.90747	0.91296	0.91550	0.91634	0.91903	0.92192	0.92508
784	0.90757	0.91305	0.91559	0.91643	0.91912	0.92200	0.92516
785	0.90766	0.91314	0.91568	0.91652	0.91920	0.92208	0.92524
786	0.90776	0.91323	0.91576	0.91660	0.91928	0.92216	0.92531
787	0.90786	0.91333	0.91585	0.91669	0.91937	0.92224	0.92539
788	0.90794	0.91341	0.91594	0.91677	0.91945	0.92232	0.92547
789	0.90805	0.91351	0.91603	0.91686	0.91953	0.92240	0.92554
790	0.90815	0.91360	0.91611	0.91694	0.91962	0.92248	0.92562
791	0.90824	0.91369	0.91620	0.91703	0.91970	0.92256	0.92569
792	0.90833	0.91378	0.91629	0.91712	0.91978	0.92264	0.92577
793	0.90843	0.91387	0.91637	0.91720	0.91986	0.92272	0.92585
794	0.90853	0.91396	0.91646	0.91729	0.91995	0.92279	0.92592
795	0.90863	0.91405	0.91654	0.91737	0.92003	0.92287	0.92600
796	0.90872	0.91414	0.91663	0.91745	0.92011	0.92295	0.92607
797	0.90882	0.91422	0.91671	0.91754	0.92019	0.92303	0.92615
798	0.90890	0.91432	0.91680	0.91762	0.92027	0.92311	0.92622
799	0.90900	0.91441	0.91688	0.91771	0.92035	0.92319	0.92630
800	0.90910	0.91449	0.91697	0.91779	0.92043	0.92326	0.92637
801	0.90919	0.91457	0.91705	0.91788	0.92052	0.92334	0.92644
802	0.90929	0.91466	0.91714	0.91796	0.92060	0.92342	0.92652
803	0.90938	0.91474	0.91722	0.91804	0.92068	0.92350	0.92659
804	0.90947	0.91483	0.91731	0.91813	0.92075	0.92358	0.92666
805	0.90956	0.91492	0.91739	0.91821	0.92084	0.92365	0.92674
806	0.90966	0.91501	0.91748	0.91829	0.92091	0.92373	0.92681
807	0.90975	0.91509	0.91756	0.91837	0.92100	0.92380	0.92688

N	0.1%	0.5%	1%	1.25%	2.5%	5%	10%
808	0.90984	0.91518	0.91764	0.91846	0.92108	0.92388	0.92696
809	0.90994	0.91526	0.91773	0.91854	0.92116	0.92396	0.92703
810	0.91002	0.91535	0.91781	0.91862	0.92123	0.92404	0.92710
811	0.91012	0.91544	0.91789	0.91871	0.92131	0.92411	0.92717
812	0.91021	0.91552	0.91797	0.91879	0.92139	0.92419	0.92725
813	0.91030	0.91561	0.91806	0.91887	0.92147	0.92426	0.92732
814	0.91039	0.91569	0.91814	0.91895	0.92155	0.92434	0.92739
815	0.91048	0.91578	0.91822	0.91903	0.92163	0.92441	0.92746
816	0.91058	0.91586	0.91830	0.91911	0.92171	0.92449	0.92753
817	0.91067	0.91594	0.91839	0.91920	0.92178	0.92456	0.92760
818	0.91076	0.91603	0.91847	0.91928	0.92186	0.92464	0.92767
819	0.91084	0.91612	0.91855	0.91936	0.92194	0.92471	0.92775
820	0.91094	0.91620	0.91863	0.91944	0.92202	0.92479	0.92782
821	0.91103	0.91628	0.91871	0.91952	0.92210	0.92486	0.92789
822	0.91113	0.91637	0.91879	0.91960	0.92217	0.92493	0.92796
823	0.91122	0.91645	0.91888	0.91968	0.92225	0.92501	0.92803
824	0.91130	0.91653	0.91896	0.91976	0.92232	0.92508	0.92810
825	0.91139	0.91662	0.91904	0.91983	0.92240	0.92515	0.92817
826	0.91147	0.91670	0.91912	0.91992	0.92248	0.92523	0.92824
827	0.91156	0.91679	0.91920	0.91999	0.92255	0.92530	0.92831
828	0.91165	0.91687	0.91928	0.92007	0.92263	0.92538	0.92838
829	0.91174	0.91695	0.91936	0.92015	0.92271	0.92545	0.92845
830	0.91183	0.91703	0.91944	0.92023	0.92278	0.92552	0.92852
831	0.91192	0.91711	0.91951	0.92031	0.92286	0.92560	0.92859
832	0.91200	0.91719	0.91959	0.92038	0.92293	0.92567	0.92866
833	0.91209	0.91728	0.91967	0.92046	0.92301	0.92574	0.92873
834	0.91218	0.91736	0.91975	0.92054	0.92308	0.92581	0.92880
835	0.91228	0.91744	0.91983	0.92062	0.92316	0.92588	0.92887
836	0.91236	0.91752	0.91991	0.92070	0.92324	0.92596	0.92893
837	0.91245	0.91760	0.91998	0.92077	0.92331	0.92603	0.92900
838	0.91254	0.91768	0.92006	0.92085	0.92339	0.92610	0.92907
839	0.91262	0.91777	0.92014	0.92093	0.92346	0.92617	0.92914
840	0.91271	0.91785	0.92021	0.92100	0.92354	0.92625	0.92921
841	0.91281	0.91792	0.92029	0.92108	0.92361	0.92632	0.92928
842	0.91290	0.91801	0.92037	0.92116	0.92368	0.92639	0.92934
843	0.91298	0.91808	0.92045	0.92123	0.92376	0.92646	0.92941
844	0.91306	0.91816	0.92053	0.92131	0.92383	0.92653	0.92948
845	0.91315	0.91824	0.92060	0.92139	0.92390	0.92660	0.92955
846	0.91324	0.91832	0.92068	0.92146	0.92398	0.92667	0.92961
847	0.91332	0.91841	0.92076	0.92154	0.92405	0.92674	0.92968
848	0.91341	0.91848	0.92083	0.92161	0.92412	0.92681	0.92975
849	0.91349	0.91856	0.92091	0.92169	0.92419	0.92688	0.92981
850	0.91357	0.91864	0.92098	0.92177	0.92427	0.92695	0.92988
851	0.91364	0.91872	0.92106	0.92184	0.92434	0.92702	0.92995
852	0.91374	0.91880	0.92114	0.92192	0.92441	0.92709	0.93001
853	0.91382	0.91888	0.92121	0.92199	0.92448	0.92716	0.93008
854	0.91390	0.91895	0.92129	0.92206	0.92456	0.92723	0.93015
855	0.91398	0.91903	0.92136	0.92214	0.92463	0.92730	0.93021
856	0.91407	0.91912	0.92144	0.92221	0.92470	0.92737	0.93028
857	0.91415	0.91919	0.92151	0.92229	0.92477	0.92743	0.93034
858	0.91424	0.91927	0.92159	0.92236	0.92484	0.92750	0.93041
859	0.91432	0.91935	0.92167	0.92244	0.92491	0.92757	0.93048
860	0.91440	0.91943	0.92174	0.92251	0.92499	0.92764	0.93054
861	0.91448	0.91950	0.92181	0.92259	0.92506	0.92771	0.93061
862	0.91457	0.91958	0.92189	0.92266	0.92513	0.92778	0.93067
863	0.91465	0.91966	0.92196	0.92273	0.92520	0.92785	0.93074
864	0.91472	0.91973	0.92203	0.92280	0.92527	0.92792	0.93080
865	0.91480	0.91981	0.92211	0.92288	0.92534	0.92798	0.93087
866	0.91488	0.91989	0.92218	0.92295	0.92541	0.92805	0.93093
867	0.91497	0.91997	0.92226	0.92302	0.92548	0.92812	0.93100
868	0.91506	0.92004	0.92233	0.92310	0.92555	0.92818	0.93106
869	0.91513	0.92012	0.92240	0.92317	0.92562	0.92825	0.93112
870	0.91521	0.92019	0.92248	0.92324	0.92569	0.92832	0.93119
871	0.91529	0.92027	0.92255	0.92332	0.92577	0.92839	0.93125
872	0.91538	0.92035	0.92262	0.92339	0.92583	0.92845	0.93132
873	0.91544	0.92042	0.92269	0.92346	0.92590	0.92852	0.93138
874	0.91552	0.92049	0.92277	0.92353	0.92597	0.92859	0.93144
875	0.91560	0.92057	0.92284	0.92360	0.92604	0.92865	0.93151
876	0.91568	0.92064	0.92291	0.92367	0.92611	0.92872	0.93157
877	0.91576	0.92072	0.92299	0.92375	0.92618	0.92878	0.93163
878	0.91584	0.92080	0.92306	0.92381	0.92625	0.92885	0.93170
879	0.91592	0.92087	0.92313	0.92389	0.92632	0.92892	0.93176
880	0.91599	0.92094	0.92320	0.92396	0.92639	0.92898	0.93182
881	0.91608	0.92102	0.92327	0.92403	0.92645	0.92905	0.93189

N	0.1%	0.5%	1%	1.25%	2.5%	5%	10%
882	0.91615	0.92109	0.92334	0.92410	0.92652	0.92911	0.93195
883	0.91622	0.92117	0.92341	0.92417	0.92659	0.92918	0.93201
884	0.91631	0.92124	0.92348	0.92424	0.92666	0.92924	0.93207
885	0.91639	0.92131	0.92355	0.92431	0.92672	0.92931	0.93214
886	0.91648	0.92138	0.92362	0.92438	0.92679	0.92937	0.93220
887	0.91656	0.92146	0.92369	0.92445	0.92686	0.92944	0.93226
888	0.91664	0.92153	0.92376	0.92452	0.92693	0.92950	0.93232
889	0.91671	0.92160	0.92383	0.92459	0.92699	0.92957	0.93238
890	0.91679	0.92168	0.92391	0.92466	0.92706	0.92963	0.93245
891	0.91687	0.92175	0.92398	0.92473	0.92713	0.92970	0.93251
892	0.91695	0.92183	0.92405	0.92480	0.92720	0.92976	0.93257
893	0.91702	0.92190	0.92411	0.92486	0.92726	0.92982	0.93263
894	0.91711	0.92197	0.92418	0.92493	0.92733	0.92989	0.93269
895	0.91717	0.92204	0.92425	0.92500	0.92739	0.92995	0.93275
896	0.91725	0.92211	0.92432	0.92507	0.92746	0.93001	0.93281
897	0.91732	0.92218	0.92439	0.92514	0.92753	0.93008	0.93287
898	0.91740	0.92226	0.92446	0.92521	0.92759	0.93014	0.93293
899	0.91748	0.92233	0.92453	0.92527	0.92766	0.93021	0.93300
900	0.91755	0.92240	0.92460	0.92534	0.92773	0.93027	0.93306
901	0.91762	0.92247	0.92467	0.92541	0.92779	0.93033	0.93312
902	0.91769	0.92254	0.92474	0.92548	0.92786	0.93039	0.93318
903	0.91778	0.92261	0.92481	0.92555	0.92792	0.93046	0.93324
904	0.91785	0.92268	0.92488	0.92562	0.92799	0.93052	0.93330
905	0.91792	0.92275	0.92495	0.92568	0.92805	0.93058	0.93336
906	0.91800	0.92282	0.92502	0.92575	0.92812	0.93065	0.93342
907	0.91808	0.92289	0.92508	0.92582	0.92818	0.93071	0.93347
908	0.91816	0.92296	0.92515	0.92589	0.92825	0.93077	0.93353
909	0.91823	0.92303	0.92522	0.92596	0.92831	0.93083	0.93359
910	0.91831	0.92310	0.92529	0.92602	0.92838	0.93090	0.93365
911	0.91839	0.92317	0.92536	0.92609	0.92844	0.93096	0.93371
912	0.91846	0.92325	0.92542	0.92615	0.92850	0.93102	0.93377
913	0.91853	0.92332	0.92549	0.92622	0.92857	0.93108	0.93383
914	0.91861	0.92338	0.92556	0.92629	0.92863	0.93114	0.93389
915	0.91868	0.92345	0.92562	0.92635	0.92870	0.93120	0.93395
916	0.91875	0.92352	0.92569	0.92642	0.92876	0.93127	0.93400
917	0.91884	0.92359	0.92576	0.92649	0.92882	0.93133	0.93406
918	0.91891	0.92366	0.92582	0.92655	0.92889	0.93139	0.93412
919	0.91899	0.92373	0.92589	0.92662	0.92895	0.93145	0.93418
920	0.91906	0.92379	0.92596	0.92668	0.92901	0.93151	0.93424
921	0.91914	0.92387	0.92602	0.92675	0.92908	0.93157	0.93429
922	0.91921	0.92394	0.92609	0.92681	0.92914	0.93163	0.93435
923	0.91929	0.92401	0.92616	0.92688	0.92920	0.93169	0.93441
924	0.91935	0.92407	0.92622	0.92694	0.92927	0.93175	0.93447
925	0.91942	0.92414	0.92629	0.92701	0.92933	0.93181	0.93452
926	0.91950	0.92421	0.92635	0.92707	0.92939	0.93187	0.93458
927	0.91957	0.92428	0.92642	0.92714	0.92945	0.93193	0.93464
928	0.91964	0.92434	0.92648	0.92720	0.92952	0.93199	0.93470
929	0.91971	0.92441	0.92655	0.92726	0.92958	0.93205	0.93475
930	0.91978	0.92448	0.92661	0.92733	0.92964	0.93211	0.93481
931	0.91986	0.92454	0.92668	0.92739	0.92970	0.93217	0.93487
932	0.91993	0.92461	0.92675	0.92746	0.92976	0.93223	0.93493
933	0.92000	0.92467	0.92681	0.92752	0.92982	0.93229	0.93498
934	0.92008	0.92474	0.92688	0.92759	0.92989	0.93235	0.93504
935	0.92015	0.92481	0.92694	0.92765	0.92995	0.93240	0.93510
936	0.92021	0.92487	0.92701	0.92771	0.93001	0.93246	0.93515
937	0.92029	0.92494	0.92707	0.92778	0.93007	0.93252	0.93521
938	0.92035	0.92501	0.92714	0.92784	0.93013	0.93258	0.93526
939	0.92043	0.92507	0.92720	0.92791	0.93019	0.93264	0.93532
940	0.92050	0.92513	0.92726	0.92797	0.93025	0.93270	0.93537
941	0.92057	0.92520	0.92733	0.92803	0.93031	0.93276	0.93543
942	0.92064	0.92527	0.92739	0.92810	0.93037	0.93282	0.93549
943	0.92072	0.92534	0.92746	0.92816	0.93043	0.93287	0.93554
944	0.92079	0.92540	0.92752	0.92822	0.93050	0.93293	0.93560
945	0.92086	0.92546	0.92758	0.92828	0.93056	0.93299	0.93565
946	0.92093	0.92553	0.92765	0.92835	0.93062	0.93305	0.93571
947	0.92100	0.92559	0.92771	0.92841	0.93068	0.93311	0.93576
948	0.92107	0.92566	0.92777	0.92847	0.93074	0.93316	0.93582
949	0.92114	0.92572	0.92783	0.92853	0.93080	0.93322	0.93587
950	0.92121	0.92579	0.92790	0.92859	0.93086	0.93328	0.93593
951	0.92127	0.92585	0.92796	0.92866	0.93092	0.93334	0.93598
952	0.92134	0.92592	0.92802	0.92872	0.93098	0.93339	0.93604
953	0.92141	0.92599	0.92808	0.92878	0.93104	0.93345	0.93609
954	0.92148	0.92605	0.92815	0.92884	0.93110	0.93351	0.93615
955	0.92155	0.92611	0.92821	0.92890	0.93116	0.93357	0.93620

N	0.1%	0.5%	1%	1.25%	2.5%	5%	10%
956	0.92160	0.92617	0.92828	0.92897	0.93121	0.93362	0.93625
957	0.92167	0.92624	0.92834	0.92903	0.93127	0.93368	0.93631
958	0.92174	0.92630	0.92839	0.92909	0.93133	0.93374	0.93636
959	0.92181	0.92637	0.92846	0.92915	0.93139	0.93379	0.93642
960	0.92187	0.92643	0.92852	0.92921	0.93145	0.93385	0.93647
961	0.92195	0.92650	0.92858	0.92927	0.93151	0.93390	0.93652
962	0.92202	0.92656	0.92864	0.92933	0.93157	0.93396	0.93658
963	0.92209	0.92662	0.92871	0.92939	0.93163	0.93402	0.93663
964	0.92216	0.92669	0.92877	0.92945	0.93169	0.93407	0.93668
965	0.92222	0.92675	0.92883	0.92951	0.93174	0.93413	0.93674
966	0.92229	0.92682	0.92889	0.92957	0.93180	0.93418	0.93679
967	0.92235	0.92688	0.92895	0.92963	0.93186	0.93424	0.93684
968	0.92241	0.92694	0.92901	0.92970	0.93191	0.93429	0.93690
969	0.92248	0.92701	0.92907	0.92975	0.93197	0.93435	0.93695
970	0.92255	0.92707	0.92913	0.92981	0.93203	0.93441	0.93700
971	0.92263	0.92713	0.92919	0.92987	0.93209	0.93446	0.93705
972	0.92269	0.92719	0.92925	0.92993	0.93214	0.93451	0.93711
973	0.92275	0.92726	0.92931	0.92999	0.93220	0.93457	0.93716
974	0.92282	0.92732	0.92937	0.93005	0.93226	0.93463	0.93721
975	0.92289	0.92738	0.92943	0.93011	0.93232	0.93468	0.93727
976	0.92296	0.92744	0.92949	0.93017	0.93237	0.93474	0.93732
977	0.92302	0.92751	0.92955	0.93023	0.93243	0.93479	0.93737
978	0.92309	0.92757	0.92961	0.93029	0.93249	0.93485	0.93742
979	0.92315	0.92763	0.92967	0.93035	0.93254	0.93490	0.93747
980	0.92322	0.92769	0.92973	0.93040	0.93260	0.93495	0.93752
981	0.92328	0.92775	0.92979	0.93046	0.93266	0.93501	0.93758
982	0.92334	0.92781	0.92985	0.93052	0.93271	0.93506	0.93763
983	0.92341	0.92787	0.92990	0.93058	0.93277	0.93512	0.93768
984	0.92348	0.92793	0.92996	0.93064	0.93283	0.93517	0.93773
985	0.92355	0.92800	0.93002	0.93070	0.93288	0.93522	0.93779
986	0.92361	0.92806	0.93008	0.93076	0.93294	0.93528	0.93784
987	0.92368	0.92811	0.93014	0.93081	0.93300	0.93533	0.93789
988	0.92374	0.92817	0.93020	0.93087	0.93305	0.93539	0.93794
989	0.92380	0.92823	0.93026	0.93093	0.93311	0.93544	0.93799
990	0.92387	0.92829	0.93032	0.93099	0.93316	0.93549	0.93804
991	0.92393	0.92835	0.93038	0.93104	0.93322	0.93555	0.93809
992	0.92399	0.92841	0.93044	0.93110	0.93327	0.93560	0.93814
993	0.92406	0.92847	0.93049	0.93116	0.93333	0.93565	0.93819
994	0.92412	0.92853	0.93055	0.93122	0.93338	0.93570	0.93824
995	0.92418	0.92859	0.93061	0.93128	0.93344	0.93576	0.93829
996	0.92424	0.92865	0.93066	0.93133	0.93349	0.93581	0.93835
997	0.92430	0.92871	0.93072	0.93139	0.93355	0.93586	0.93840
998	0.92437	0.92877	0.93078	0.93145	0.93360	0.93592	0.93844
999	0.92443	0.92883	0.93083	0.93150	0.93366	0.93597	0.93849
1000	0.92450	0.92889	0.93089	0.93156	0.93371	0.93602	0.93855

Table 4 – *GM6(3H3L)*. Critical values of upper significance levels for the Grubbs multiple-6 outlier test, applicable in situations where the three highest and three lowest observations are simultaneously identified as outliers, denoted as *GM6(3H3L)*.

N	0.1%	0.5%	1%	1.25%	2.5%	5%	10%
8	0.00000	0.00000	0.00000	0.00000	0.00001	0.00003	0.00012
9	0.00002	0.00010	0.00020	0.00026	0.00053	0.00112	0.00242
10	0.00030	0.00093	0.00152	0.00179	0.00297	0.00500	0.00864
11	0.00137	0.00322	0.00471	0.00534	0.00792	0.01189	0.01825
12	0.00360	0.00727	0.00992	0.01099	0.01521	0.02129	0.03039
13	0.00720	0.01304	0.01699	0.01853	0.02442	0.03259	0.04422
14	0.01209	0.02030	0.02561	0.02762	0.03516	0.04524	0.05914
15	0.01822	0.02879	0.03540	0.03787	0.04698	0.05879	0.07466
16	0.02534	0.03831	0.04611	0.04904	0.05953	0.07293	0.09049
17	0.03342	0.04857	0.05758	0.06086	0.07264	0.08738	0.10642
18	0.04207	0.05952	0.06948	0.07312	0.08604	0.10195	0.12226
19	0.05127	0.07071	0.08168	0.08561	0.09950	0.11648	0.13783
20	0.06107	0.08217	0.09404	0.09828	0.11304	0.13091	0.15316
21	0.07117	0.09396	0.10649	0.11098	0.12648	0.14512	0.16813
22	0.08146	0.10573	0.11890	0.12358	0.13980	0.15910	0.18273
23	0.09169	0.11738	0.13125	0.13612	0.15293	0.17277	0.19697
24	0.10235	0.12914	0.14345	0.14851	0.16580	0.18613	0.21073
25	0.11282	0.14071	0.15552	0.16072	0.17846	0.19921	0.22412
26	0.12329	0.15215	0.16744	0.17271	0.19079	0.21189	0.23709
27	0.13371	0.16354	0.17905	0.18447	0.20291	0.22425	0.24970
28	0.14428	0.17463	0.19045	0.19596	0.21468	0.23627	0.26187
29	0.15445	0.18560	0.20165	0.20726	0.22615	0.24792	0.27371

N	0.1%	0.5%	1%	1.25%	2.5%	5%	10%
30	0.16438	0.19631	0.21250	0.21825	0.23733	0.25927	0.28510
31	0.17445	0.20673	0.22326	0.22902	0.24827	0.27029	0.29618
32	0.18427	0.21710	0.23373	0.23952	0.25887	0.28099	0.30688
33	0.19408	0.22723	0.24400	0.24979	0.26924	0.29140	0.31727
34	0.20355	0.23703	0.25397	0.25981	0.27933	0.30150	0.32731
35	0.21306	0.24671	0.26370	0.26960	0.28915	0.31133	0.33709
36	0.22219	0.25618	0.27322	0.27906	0.29871	0.32085	0.34656
37	0.23130	0.26545	0.28248	0.28835	0.30801	0.33014	0.35573
38	0.24006	0.27435	0.29151	0.29741	0.31701	0.33915	0.36465
39	0.24871	0.28315	0.30034	0.30625	0.32584	0.34792	0.37328
40	0.25711	0.29177	0.30894	0.31493	0.33443	0.35642	0.38169
41	0.26545	0.30016	0.31739	0.32329	0.34278	0.36473	0.38980
42	0.27353	0.30842	0.32561	0.33147	0.35093	0.37282	0.39775
43	0.28158	0.31647	0.33363	0.33947	0.35886	0.38064	0.40544
44	0.28944	0.32432	0.34139	0.34724	0.36662	0.38826	0.41290
45	0.29726	0.33193	0.34905	0.35483	0.37418	0.39570	0.42017
46	0.30467	0.33940	0.35647	0.36225	0.38153	0.40291	0.42726
47	0.31216	0.34684	0.36372	0.36949	0.38866	0.40997	0.43417
48	0.31942	0.35394	0.37087	0.37658	0.39564	0.41682	0.44090
49	0.32641	0.36095	0.37775	0.38349	0.40251	0.42353	0.44744
50	0.33334	0.36788	0.38450	0.39021	0.40914	0.43007	0.45380
51	0.34002	0.37453	0.39116	0.39687	0.41558	0.43643	0.46001
52	0.34680	0.38106	0.39773	0.40335	0.42192	0.44264	0.46606
53	0.35327	0.38743	0.40399	0.40959	0.42813	0.44872	0.47199
54	0.35965	0.39367	0.41018	0.41578	0.43421	0.45463	0.47774
55	0.36583	0.39979	0.41621	0.42175	0.44013	0.46045	0.48335
56	0.37188	0.40586	0.42214	0.42776	0.44593	0.46611	0.48885
57	0.37800	0.41166	0.42797	0.43349	0.45157	0.47161	0.49423
58	0.38393	0.41736	0.43355	0.43902	0.45716	0.47705	0.49946
59	0.38965	0.42292	0.43912	0.44454	0.46256	0.48232	0.50458
60	0.39522	0.42845	0.44453	0.44994	0.46785	0.48752	0.50957
61	0.40083	0.43387	0.44987	0.45529	0.47305	0.49253	0.51445
62	0.40635	0.43923	0.45514	0.46050	0.47814	0.49747	0.51925
63	0.41151	0.44445	0.46026	0.46555	0.48311	0.50233	0.52390
64	0.41683	0.44954	0.46521	0.47047	0.48796	0.50706	0.52848
65	0.42201	0.45453	0.47007	0.47538	0.49276	0.51171	0.53293
66	0.42696	0.45944	0.47490	0.48014	0.49743	0.51621	0.53731
67	0.43192	0.46426	0.47965	0.48488	0.50197	0.52066	0.54160
68	0.43676	0.46896	0.48431	0.48943	0.50644	0.52501	0.54581
69	0.44150	0.47359	0.48883	0.49397	0.51081	0.52925	0.54993
70	0.44624	0.47817	0.49332	0.49840	0.51511	0.53343	0.55396
71	0.45070	0.48266	0.49767	0.50277	0.51938	0.53754	0.55792
72	0.45534	0.48695	0.50198	0.50702	0.52349	0.54156	0.56181
73	0.45985	0.49124	0.50619	0.51124	0.52756	0.54550	0.56559
74	0.46411	0.49544	0.51028	0.51530	0.53154	0.54937	0.56932
75	0.46842	0.49963	0.51438	0.51931	0.53548	0.55317	0.57296
76	0.47266	0.50368	0.51837	0.52327	0.53930	0.55690	0.57654
77	0.47684	0.50764	0.52223	0.52710	0.54307	0.56055	0.58007
78	0.48099	0.51159	0.52607	0.53093	0.54677	0.56413	0.58351
79	0.48500	0.51546	0.52983	0.53463	0.55042	0.56765	0.58691
80	0.48901	0.51926	0.53354	0.53830	0.55401	0.57112	0.59025
81	0.49286	0.52302	0.53717	0.54192	0.55748	0.57452	0.59351
82	0.49672	0.52662	0.54075	0.54549	0.56092	0.57785	0.59672
83	0.50036	0.53018	0.54421	0.54892	0.56431	0.58113	0.59988
84	0.50416	0.53372	0.54769	0.55236	0.56765	0.58435	0.60297
85	0.50782	0.53724	0.55109	0.55575	0.57096	0.58753	0.60602
86	0.51128	0.54066	0.55443	0.55908	0.57420	0.59066	0.60902
87	0.51480	0.54400	0.55770	0.56235	0.57735	0.59371	0.61196
88	0.51834	0.54737	0.56101	0.56560	0.58052	0.59674	0.61485
89	0.52185	0.55059	0.56418	0.56873	0.58356	0.59970	0.61769
90	0.52517	0.55384	0.56731	0.57185	0.58658	0.60261	0.62047
91	0.52852	0.55702	0.57041	0.57489	0.58955	0.60550	0.62323
92	0.53182	0.56018	0.57344	0.57793	0.59250	0.60832	0.62594
93	0.53500	0.56323	0.57642	0.58087	0.59534	0.61110	0.62860
94	0.53816	0.56626	0.57935	0.58378	0.59820	0.61383	0.63124
95	0.54124	0.56922	0.58226	0.58664	0.60096	0.61651	0.63383
96	0.54430	0.57213	0.58517	0.58949	0.60372	0.61917	0.63636
97	0.54739	0.57508	0.58799	0.59229	0.60643	0.62177	0.63886
98	0.55050	0.57789	0.59072	0.59504	0.60909	0.62437	0.64132
99	0.55347	0.58074	0.59345	0.59772	0.61172	0.62691	0.64376
100	0.55646	0.58343	0.59614	0.60042	0.61432	0.62941	0.64615
101	0.55916	0.58613	0.59879	0.60302	0.61688	0.63188	0.64851
102	0.56200	0.58880	0.60142	0.60563	0.61938	0.63430	0.65081
103	0.56471	0.59149	0.60402	0.60821	0.62186	0.63669	0.65310

N	0.1%	0.5%	1%	1.25%	2.5%	5%	10%
104	0.56754	0.59414	0.60658	0.61071	0.62429	0.63903	0.65534
105	0.57017	0.59669	0.60908	0.61320	0.62672	0.64137	0.65756
106	0.57284	0.59921	0.61154	0.61568	0.62910	0.64364	0.65975
107	0.57559	0.60174	0.61397	0.61813	0.63145	0.64590	0.66190
108	0.57807	0.60424	0.61640	0.62049	0.63377	0.64812	0.66403
109	0.58074	0.60669	0.61879	0.62285	0.63603	0.65032	0.66613
110	0.58320	0.60910	0.62112	0.62514	0.63826	0.65248	0.66821
111	0.58575	0.61147	0.62345	0.62744	0.64048	0.65461	0.67025
112	0.58820	0.61380	0.62576	0.62970	0.64266	0.65671	0.67224
113	0.59072	0.61613	0.62800	0.63193	0.64481	0.65878	0.67422
114	0.59314	0.61839	0.63024	0.63412	0.64694	0.66083	0.67619
115	0.59542	0.62066	0.63239	0.63629	0.64903	0.66284	0.67811
116	0.59772	0.62287	0.63452	0.63842	0.65111	0.66484	0.68001
117	0.60011	0.62504	0.63667	0.64053	0.65316	0.66680	0.68188
118	0.60247	0.62725	0.63878	0.64262	0.65517	0.66873	0.68373
119	0.60469	0.62935	0.64085	0.64470	0.65716	0.67065	0.68556
120	0.60690	0.63151	0.64292	0.64674	0.65913	0.67254	0.68737
121	0.60912	0.63360	0.64494	0.64875	0.66105	0.67440	0.68916
122	0.61128	0.63570	0.64692	0.65075	0.66296	0.67625	0.69090
123	0.61346	0.63770	0.64894	0.65271	0.66485	0.67806	0.69265
124	0.61567	0.63975	0.65091	0.65466	0.66672	0.67985	0.69437
125	0.61776	0.64174	0.65284	0.65656	0.66859	0.68163	0.69607
126	0.61991	0.64370	0.65473	0.65844	0.67041	0.68338	0.69775
127	0.62197	0.64562	0.65664	0.66032	0.67219	0.68512	0.69940
128	0.62403	0.64754	0.65847	0.66217	0.67396	0.68682	0.70103
129	0.62597	0.64943	0.66031	0.66399	0.67572	0.68853	0.70265
130	0.62788	0.65126	0.66217	0.66581	0.67746	0.69018	0.70424
131	0.62977	0.65307	0.66395	0.66758	0.67919	0.69184	0.70581
132	0.63178	0.65491	0.66574	0.66933	0.68087	0.69348	0.70739
133	0.63369	0.65668	0.66747	0.67102	0.68254	0.69510	0.70892
134	0.63563	0.65846	0.66919	0.67272	0.68418	0.69668	0.71044
135	0.63751	0.66024	0.67090	0.67442	0.68583	0.69825	0.71195
136	0.63941	0.66198	0.67255	0.67609	0.68743	0.69981	0.71344
137	0.64120	0.66373	0.67424	0.67776	0.68904	0.70134	0.71490
138	0.64304	0.66541	0.67590	0.67936	0.69063	0.70286	0.71636
139	0.64482	0.66709	0.67752	0.68097	0.69219	0.70437	0.71779
140	0.64654	0.66879	0.67912	0.68258	0.69372	0.70586	0.71921
141	0.64829	0.67042	0.68074	0.68417	0.69526	0.70733	0.72061
142	0.65004	0.67208	0.68231	0.68571	0.69679	0.70878	0.72200
143	0.65175	0.67373	0.68389	0.68725	0.69830	0.71021	0.72338
144	0.65351	0.67533	0.68542	0.68880	0.69980	0.71165	0.72472
145	0.65517	0.67689	0.68695	0.69033	0.70125	0.71306	0.72607
146	0.65686	0.67846	0.68849	0.69182	0.70269	0.71445	0.72740
147	0.65850	0.67999	0.68999	0.69333	0.70414	0.71582	0.72872
148	0.66012	0.68152	0.69146	0.69480	0.70554	0.71717	0.73001
149	0.66176	0.68299	0.69295	0.69624	0.70695	0.71853	0.73129
150	0.66335	0.68450	0.69441	0.69770	0.70834	0.71987	0.73256
151	0.66490	0.68602	0.69583	0.69913	0.70971	0.72118	0.73381
152	0.66654	0.68747	0.69724	0.70051	0.71107	0.72249	0.73505
153	0.66805	0.68897	0.69869	0.70190	0.71241	0.72378	0.73629
154	0.66958	0.69044	0.70007	0.70327	0.71374	0.72506	0.73751
155	0.67117	0.69182	0.70147	0.70466	0.71506	0.72633	0.73872
156	0.67274	0.69323	0.70284	0.70600	0.71636	0.72757	0.73992
157	0.67412	0.69460	0.70417	0.70734	0.71765	0.72882	0.74111
158	0.67559	0.69602	0.70550	0.70866	0.71893	0.73005	0.74227
159	0.67702	0.69738	0.70683	0.70998	0.72019	0.73126	0.74343
160	0.67849	0.69876	0.70816	0.71129	0.72144	0.73246	0.74458
161	0.67990	0.70008	0.70948	0.71256	0.72267	0.73365	0.74570
162	0.68124	0.70141	0.71073	0.71384	0.72391	0.73483	0.74682
163	0.68264	0.70275	0.71202	0.71510	0.72512	0.73600	0.74793
164	0.68406	0.70404	0.71327	0.71635	0.72632	0.73715	0.74903
165	0.68534	0.70532	0.71451	0.71761	0.72753	0.73829	0.75013
166	0.68667	0.70657	0.71578	0.71884	0.72872	0.73941	0.75121
167	0.68804	0.70780	0.71700	0.72004	0.72988	0.74054	0.75228
168	0.68943	0.70905	0.71820	0.72124	0.73104	0.74166	0.75334
169	0.69078	0.71030	0.71942	0.72244	0.73219	0.74276	0.75439
170	0.69213	0.71155	0.72060	0.72363	0.73333	0.74386	0.75543
171	0.69342	0.71280	0.72178	0.72477	0.73446	0.74494	0.75646
172	0.69471	0.71404	0.72295	0.72591	0.73559	0.74601	0.75748
173	0.69596	0.71523	0.72411	0.72708	0.73669	0.74707	0.75850
174	0.69717	0.71641	0.72528	0.72822	0.73781	0.74813	0.75950
175	0.69840	0.71759	0.72643	0.72935	0.73889	0.74916	0.76049
176	0.69962	0.71875	0.72754	0.73047	0.73996	0.75019	0.76148
177	0.70083	0.71990	0.72866	0.73159	0.74102	0.75122	0.76245

N	0.1%	0.5%	1%	1.25%	2.5%	5%	10%
178	0.70205	0.72106	0.72976	0.73270	0.74208	0.75223	0.76341
179	0.70323	0.72219	0.73087	0.73377	0.74314	0.75324	0.76437
180	0.70446	0.72333	0.73194	0.73485	0.74419	0.75424	0.76532
181	0.70565	0.72443	0.73302	0.73591	0.74520	0.75522	0.76626
182	0.70686	0.72550	0.73408	0.73697	0.74623	0.75620	0.76719
183	0.70805	0.72661	0.73514	0.73800	0.74723	0.75717	0.76811
184	0.70920	0.72771	0.73620	0.73904	0.74825	0.75813	0.76902
185	0.71036	0.72878	0.73724	0.74008	0.74924	0.75909	0.76992
186	0.71152	0.72982	0.73826	0.74108	0.75021	0.76003	0.77082
187	0.71267	0.73086	0.73931	0.74210	0.75119	0.76097	0.77172
188	0.71383	0.73192	0.74030	0.74310	0.75215	0.76190	0.77260
189	0.71495	0.73295	0.74131	0.74410	0.75311	0.76281	0.77348
190	0.71600	0.73398	0.74232	0.74510	0.75407	0.76373	0.77435
191	0.71709	0.73500	0.74332	0.74607	0.75503	0.76464	0.77521
192	0.71815	0.73598	0.74428	0.74704	0.75596	0.76554	0.77607
193	0.71921	0.73702	0.74524	0.74799	0.75688	0.76643	0.77692
194	0.72028	0.73800	0.74620	0.74894	0.75780	0.76731	0.77777
195	0.72132	0.73901	0.74716	0.74991	0.75872	0.76818	0.77860
196	0.72243	0.73999	0.74812	0.75083	0.75962	0.76906	0.77942
197	0.72346	0.74096	0.74905	0.75177	0.76052	0.76992	0.78024
198	0.72444	0.74193	0.74999	0.75268	0.76140	0.77076	0.78105
199	0.72549	0.74290	0.75092	0.75361	0.76229	0.77161	0.78186
200	0.72648	0.74385	0.75180	0.75450	0.76315	0.77244	0.78265
201	0.72743	0.74477	0.75273	0.75542	0.76402	0.77327	0.78344
202	0.72846	0.74570	0.75365	0.75632	0.76490	0.77410	0.78423
203	0.72942	0.74664	0.75456	0.75722	0.76575	0.77492	0.78501
204	0.73045	0.74755	0.75545	0.75810	0.76660	0.77573	0.78579
205	0.73145	0.74847	0.75635	0.75898	0.76744	0.77654	0.78656
206	0.73248	0.74939	0.75723	0.75983	0.76827	0.77734	0.78732
207	0.73339	0.75029	0.75809	0.76070	0.76910	0.77814	0.78807
208	0.73434	0.75116	0.75897	0.76156	0.76990	0.77892	0.78883
209	0.73527	0.75204	0.75982	0.76242	0.77072	0.77970	0.78956
210	0.73621	0.75291	0.76067	0.76324	0.77153	0.78047	0.79031
211	0.73715	0.75379	0.76152	0.76408	0.77232	0.78124	0.79103
212	0.73803	0.75467	0.76237	0.76491	0.77312	0.78200	0.79176
213	0.73892	0.75555	0.76320	0.76573	0.77392	0.78276	0.79248
214	0.73986	0.75637	0.76402	0.76656	0.77471	0.78351	0.79320
215	0.74074	0.75722	0.76483	0.76736	0.77547	0.78426	0.79391
216	0.74164	0.75809	0.76565	0.76816	0.77625	0.78500	0.79462
217	0.74255	0.75888	0.76644	0.76896	0.77702	0.78574	0.79532
218	0.74345	0.75971	0.76726	0.76975	0.77779	0.78647	0.79601
219	0.74435	0.76051	0.76805	0.77052	0.77855	0.78719	0.79670
220	0.74525	0.76133	0.76884	0.77128	0.77930	0.78791	0.79738
221	0.74610	0.76213	0.76961	0.77206	0.78004	0.78862	0.79806
222	0.74697	0.76293	0.77038	0.77282	0.78078	0.78933	0.79873
223	0.74784	0.76373	0.77114	0.77357	0.78152	0.79002	0.79940
224	0.74866	0.76451	0.77190	0.77433	0.78224	0.79071	0.80006
225	0.74945	0.76529	0.77268	0.77508	0.78297	0.79141	0.80073
226	0.75030	0.76607	0.77341	0.77582	0.78368	0.79210	0.80138
227	0.75110	0.76684	0.77415	0.77656	0.78439	0.79278	0.80203
228	0.75192	0.76761	0.77488	0.77728	0.78509	0.79346	0.80268
229	0.75273	0.76838	0.77561	0.77801	0.78580	0.79413	0.80332
230	0.75356	0.76915	0.77635	0.77874	0.78648	0.79479	0.80395
231	0.75432	0.76989	0.77707	0.77946	0.78718	0.79546	0.80459
232	0.75513	0.77062	0.77778	0.78017	0.78787	0.79611	0.80521
233	0.75586	0.77136	0.77851	0.78089	0.78855	0.79677	0.80583
234	0.75669	0.77211	0.77923	0.78158	0.78923	0.79742	0.80645
235	0.75745	0.77281	0.77993	0.78229	0.78990	0.79807	0.80706
236	0.75819	0.77354	0.78062	0.78297	0.79056	0.79870	0.80767
237	0.75901	0.77426	0.78130	0.78365	0.79122	0.79933	0.80827
238	0.75979	0.77497	0.78202	0.78434	0.79188	0.79996	0.80887
239	0.76053	0.77568	0.78270	0.78502	0.79254	0.80059	0.80947
240	0.76126	0.77639	0.78338	0.78569	0.79318	0.80121	0.81006
241	0.76203	0.77708	0.78405	0.78636	0.79383	0.80182	0.81064
242	0.76277	0.77777	0.78474	0.78702	0.79445	0.80244	0.81122
243	0.76352	0.77847	0.78540	0.78768	0.79509	0.80305	0.81181
244	0.76427	0.77917	0.78606	0.78834	0.79571	0.80366	0.81238
245	0.76503	0.77984	0.78674	0.78900	0.79635	0.80426	0.81296
246	0.76573	0.78051	0.78739	0.78963	0.79697	0.80485	0.81352
247	0.76640	0.78117	0.78804	0.79028	0.79757	0.80544	0.81409
248	0.76711	0.78182	0.78867	0.79091	0.79819	0.80603	0.81465
249	0.76784	0.78250	0.78929	0.79154	0.79880	0.80661	0.81521
250	0.76851	0.78316	0.78991	0.79218	0.79941	0.80720	0.81576
251	0.76928	0.78379	0.79055	0.79280	0.80002	0.80777	0.81631

N	0.1%	0.5%	1%	1.25%	2.5%	5%	10%
252	0.76995	0.78446	0.79117	0.79342	0.80061	0.80835	0.81686
253	0.77065	0.78511	0.79180	0.79404	0.80121	0.80891	0.81740
254	0.77134	0.78575	0.79242	0.79465	0.80181	0.80948	0.81794
255	0.77202	0.78637	0.79304	0.79525	0.80238	0.81004	0.81848
256	0.77268	0.78702	0.79365	0.79585	0.80296	0.81060	0.81901
257	0.77336	0.78765	0.79426	0.79646	0.80354	0.81115	0.81953
258	0.77404	0.78828	0.79486	0.79705	0.80410	0.81170	0.82006
259	0.77467	0.78891	0.79545	0.79764	0.80469	0.81226	0.82058
260	0.77534	0.78952	0.79603	0.79822	0.80525	0.81281	0.82110
261	0.77602	0.79014	0.79664	0.79880	0.80582	0.81334	0.82161
262	0.77667	0.79074	0.79722	0.79938	0.80638	0.81388	0.82212
263	0.77729	0.79134	0.79782	0.79997	0.80694	0.81441	0.82263
264	0.77794	0.79194	0.79840	0.80053	0.80750	0.81494	0.82314
265	0.77855	0.79252	0.79897	0.80110	0.80805	0.81547	0.82364
266	0.77920	0.79312	0.79955	0.80168	0.80860	0.81599	0.82414
267	0.77986	0.79372	0.80013	0.80223	0.80914	0.81651	0.82463
268	0.78046	0.79430	0.80069	0.80279	0.80968	0.81703	0.82513
269	0.78110	0.79490	0.80126	0.80334	0.81022	0.81754	0.82562
270	0.78170	0.79548	0.80180	0.80389	0.81074	0.81806	0.82610
271	0.78230	0.79606	0.80237	0.80445	0.81127	0.81856	0.82659
272	0.78291	0.79661	0.80292	0.80501	0.81181	0.81907	0.82706
273	0.78357	0.79719	0.80346	0.80555	0.81233	0.81957	0.82754
274	0.78414	0.79777	0.80401	0.80610	0.81284	0.82007	0.82802
275	0.78476	0.79832	0.80456	0.80664	0.81336	0.82057	0.82849
276	0.78535	0.79889	0.80511	0.80717	0.81388	0.82106	0.82896
277	0.78597	0.79945	0.80566	0.80770	0.81439	0.82156	0.82943
278	0.78655	0.80000	0.80618	0.80824	0.81489	0.82204	0.82990
279	0.78718	0.80054	0.80670	0.80876	0.81540	0.82252	0.83036
280	0.78777	0.80107	0.80723	0.80928	0.81590	0.82300	0.83082
281	0.78834	0.80162	0.80776	0.80979	0.81639	0.82349	0.83127
282	0.78894	0.80217	0.80829	0.81031	0.81689	0.82397	0.83173
283	0.78946	0.80271	0.80883	0.81082	0.81739	0.82444	0.83218
284	0.79004	0.80326	0.80933	0.81133	0.81788	0.82490	0.83262
285	0.79061	0.80378	0.80983	0.81184	0.81837	0.82537	0.83307
286	0.79120	0.80430	0.81035	0.81235	0.81885	0.82584	0.83351
287	0.79171	0.80482	0.81084	0.81284	0.81933	0.82629	0.83395
288	0.79227	0.80534	0.81134	0.81335	0.81981	0.82675	0.83439
289	0.79285	0.80588	0.81184	0.81383	0.82028	0.82721	0.83482
290	0.79339	0.80638	0.81234	0.81432	0.82075	0.82766	0.83525
291	0.79391	0.80689	0.81284	0.81481	0.82123	0.82811	0.83568
292	0.79444	0.80740	0.81334	0.81530	0.82170	0.82856	0.83611
293	0.79499	0.80789	0.81382	0.81578	0.82217	0.82901	0.83653
294	0.79553	0.80840	0.81431	0.81627	0.82262	0.82945	0.83696
295	0.79605	0.80890	0.81478	0.81675	0.82308	0.82989	0.83737
296	0.79653	0.80938	0.81526	0.81723	0.82355	0.83034	0.83779
297	0.79705	0.80988	0.81574	0.81771	0.82402	0.83077	0.83820
298	0.79761	0.81036	0.81622	0.81817	0.82447	0.83120	0.83862
299	0.79814	0.81085	0.81670	0.81864	0.82491	0.83163	0.83903
300	0.79867	0.81135	0.81716	0.81909	0.82536	0.83206	0.83943
301	0.79921	0.81184	0.81763	0.81956	0.82580	0.83249	0.83984
302	0.79971	0.81232	0.81808	0.82002	0.82624	0.83291	0.84024
303	0.80022	0.81279	0.81855	0.82048	0.82668	0.83333	0.84064
304	0.80074	0.81325	0.81901	0.82093	0.82713	0.83375	0.84104
305	0.80121	0.81372	0.81946	0.82138	0.82755	0.83417	0.84144
306	0.80174	0.81419	0.81992	0.82182	0.82799	0.83458	0.84183
307	0.80225	0.81463	0.82037	0.82227	0.82842	0.83499	0.84222
308	0.80274	0.81510	0.82082	0.82272	0.82884	0.83540	0.84262
309	0.80324	0.81556	0.82128	0.82316	0.82926	0.83581	0.84300
310	0.80376	0.81604	0.82171	0.82360	0.82968	0.83621	0.84339
311	0.80424	0.81650	0.82215	0.82403	0.83011	0.83661	0.84377
312	0.80474	0.81696	0.82259	0.82448	0.83053	0.83701	0.84415
313	0.80519	0.81742	0.82303	0.82490	0.83095	0.83741	0.84453
314	0.80565	0.81786	0.82346	0.82533	0.83137	0.83781	0.84491
315	0.80613	0.81831	0.82391	0.82577	0.83178	0.83820	0.84529
316	0.80661	0.81874	0.82434	0.82620	0.83219	0.83859	0.84566
317	0.80705	0.81918	0.82476	0.82662	0.83259	0.83899	0.84603
318	0.80753	0.81962	0.82519	0.82703	0.83299	0.83937	0.84640
319	0.80801	0.82006	0.82562	0.82746	0.83340	0.83976	0.84676
320	0.80849	0.82050	0.82604	0.82787	0.83381	0.84015	0.84713
321	0.80895	0.82094	0.82645	0.82830	0.83420	0.84053	0.84749
322	0.80942	0.82137	0.82688	0.82869	0.83460	0.84091	0.84785
323	0.80987	0.82179	0.82730	0.82910	0.83499	0.84128	0.84821
324	0.81031	0.82222	0.82770	0.82952	0.83538	0.84167	0.84857
325	0.81074	0.82266	0.82812	0.82993	0.83578	0.84204	0.84893

N	0.1%	0.5%	1%	1.25%	2.5%	5%	10%
326	0.81120	0.82309	0.82853	0.83033	0.83616	0.84241	0.84928
327	0.81165	0.82351	0.82893	0.83073	0.83654	0.84278	0.84964
328	0.81212	0.82392	0.82934	0.83114	0.83693	0.84315	0.84998
329	0.81256	0.82434	0.82974	0.83154	0.83731	0.84352	0.85033
330	0.81300	0.82475	0.83013	0.83192	0.83769	0.84388	0.85068
331	0.81345	0.82516	0.83053	0.83231	0.83807	0.84424	0.85102
332	0.81387	0.82557	0.83093	0.83270	0.83844	0.84460	0.85137
333	0.81428	0.82597	0.83132	0.83310	0.83882	0.84496	0.85171
334	0.81472	0.82638	0.83171	0.83349	0.83919	0.84532	0.85205
335	0.81514	0.82679	0.83211	0.83387	0.83956	0.84567	0.85239
336	0.81557	0.82718	0.83250	0.83425	0.83993	0.84603	0.85273
337	0.81599	0.82759	0.83288	0.83463	0.84030	0.84638	0.85306
338	0.81643	0.82799	0.83326	0.83501	0.84066	0.84674	0.85340
339	0.81686	0.82839	0.83364	0.83539	0.84103	0.84708	0.85373
340	0.81728	0.82876	0.83402	0.83577	0.84139	0.84742	0.85406
341	0.81768	0.82916	0.83441	0.83615	0.84175	0.84777	0.85439
342	0.81809	0.82954	0.83478	0.83652	0.84211	0.84811	0.85472
343	0.81852	0.82991	0.83515	0.83689	0.84247	0.84845	0.85504
344	0.81892	0.83032	0.83552	0.83726	0.84282	0.84879	0.85537
345	0.81932	0.83070	0.83588	0.83763	0.84317	0.84913	0.85569
346	0.81975	0.83107	0.83626	0.83800	0.84352	0.84947	0.85601
347	0.82016	0.83145	0.83663	0.83835	0.84387	0.84981	0.85633
348	0.82058	0.83183	0.83699	0.83871	0.84422	0.85015	0.85665
349	0.82100	0.83220	0.83736	0.83907	0.84456	0.85047	0.85696
350	0.82138	0.83257	0.83773	0.83943	0.84491	0.85080	0.85727
351	0.82180	0.83296	0.83810	0.83979	0.84525	0.85113	0.85759
352	0.82218	0.83332	0.83846	0.84014	0.84559	0.85146	0.85790
353	0.82256	0.83370	0.83881	0.84049	0.84593	0.85178	0.85821
354	0.82296	0.83407	0.83917	0.84085	0.84627	0.85211	0.85852
355	0.82337	0.83443	0.83952	0.84120	0.84660	0.85243	0.85883
356	0.82374	0.83477	0.83987	0.84156	0.84694	0.85275	0.85913
357	0.82411	0.83514	0.84023	0.84189	0.84727	0.85307	0.85944
358	0.82451	0.83550	0.84058	0.84224	0.84761	0.85339	0.85974
359	0.82488	0.83586	0.84092	0.84258	0.84794	0.85371	0.86004
360	0.82527	0.83621	0.84126	0.84293	0.84827	0.85402	0.86034
361	0.82567	0.83656	0.84160	0.84327	0.84860	0.85433	0.86064
362	0.82605	0.83693	0.84193	0.84360	0.84892	0.85465	0.86094
363	0.82641	0.83728	0.84228	0.84394	0.84925	0.85496	0.86123
364	0.82679	0.83763	0.84262	0.84428	0.84957	0.85526	0.86152
365	0.82716	0.83797	0.84295	0.84460	0.84989	0.85557	0.86182
366	0.82755	0.83832	0.84330	0.84494	0.85020	0.85588	0.86211
367	0.82795	0.83867	0.84363	0.84527	0.85052	0.85618	0.86240
368	0.82830	0.83901	0.84397	0.84559	0.85084	0.85648	0.86269
369	0.82865	0.83935	0.84429	0.84592	0.85115	0.85678	0.86297
370	0.82903	0.83969	0.84462	0.84625	0.85146	0.85709	0.86326
371	0.82938	0.84003	0.84494	0.84657	0.85178	0.85738	0.86355
372	0.82974	0.84036	0.84527	0.84690	0.85209	0.85768	0.86383
373	0.83008	0.84070	0.84560	0.84722	0.85240	0.85798	0.86411
374	0.83045	0.84103	0.84593	0.84754	0.85271	0.85827	0.86439
375	0.83082	0.84136	0.84625	0.84785	0.85301	0.85857	0.86468
376	0.83114	0.84170	0.84658	0.84817	0.85332	0.85886	0.86495
377	0.83150	0.84204	0.84689	0.84848	0.85362	0.85915	0.86523
378	0.83187	0.84236	0.84721	0.84879	0.85393	0.85944	0.86550
379	0.83221	0.84269	0.84752	0.84911	0.85423	0.85973	0.86578
380	0.83257	0.84301	0.84784	0.84943	0.85452	0.86001	0.86605
381	0.83294	0.84333	0.84816	0.84973	0.85483	0.86030	0.86632
382	0.83326	0.84366	0.84846	0.85004	0.85512	0.86058	0.86660
383	0.83360	0.84397	0.84877	0.85034	0.85541	0.86087	0.86686
384	0.83394	0.84428	0.84908	0.85064	0.85570	0.86115	0.86713
385	0.83430	0.84461	0.84940	0.85095	0.85600	0.86143	0.86740
386	0.83465	0.84493	0.84969	0.85125	0.85629	0.86171	0.86767
387	0.83501	0.84525	0.85000	0.85156	0.85658	0.86199	0.86793
388	0.83534	0.84555	0.85030	0.85186	0.85687	0.86226	0.86819
389	0.83569	0.84587	0.85060	0.85216	0.85716	0.86254	0.86846
390	0.83603	0.84618	0.85091	0.85245	0.85744	0.86282	0.86872
391	0.83636	0.84649	0.85120	0.85274	0.85773	0.86309	0.86898
392	0.83671	0.84680	0.85149	0.85304	0.85801	0.86336	0.86924
393	0.83702	0.84710	0.85179	0.85333	0.85829	0.86363	0.86950
394	0.83738	0.84741	0.85208	0.85363	0.85858	0.86390	0.86975
395	0.83770	0.84770	0.85238	0.85392	0.85886	0.86417	0.87001
396	0.83802	0.84801	0.85268	0.85422	0.85914	0.86444	0.87027
397	0.83835	0.84831	0.85296	0.85450	0.85941	0.86470	0.87052
398	0.83866	0.84862	0.85326	0.85479	0.85970	0.86496	0.87077
399	0.83897	0.84892	0.85354	0.85506	0.85997	0.86523	0.87102

N	0.1%	0.5%	1%	1.25%	2.5%	5%	10%
400	0.83930	0.84921	0.85383	0.85535	0.86025	0.86549	0.87127
401	0.83961	0.84950	0.85411	0.85563	0.86052	0.86576	0.87152
402	0.83992	0.84980	0.85441	0.85593	0.86079	0.86602	0.87177
403	0.84026	0.85009	0.85469	0.85621	0.86107	0.86628	0.87202
404	0.84054	0.85039	0.85497	0.85649	0.86134	0.86654	0.87226
405	0.84086	0.85068	0.85526	0.85676	0.86160	0.86680	0.87251
406	0.84119	0.85096	0.85554	0.85704	0.86188	0.86705	0.87275
407	0.84150	0.85125	0.85582	0.85731	0.86214	0.86731	0.87299
408	0.84182	0.85154	0.85609	0.85759	0.86240	0.86756	0.87323
409	0.84213	0.85183	0.85637	0.85786	0.86267	0.86781	0.87347
410	0.84243	0.85213	0.85664	0.85813	0.86293	0.86806	0.87371
411	0.84274	0.85240	0.85692	0.85840	0.86319	0.86831	0.87395
412	0.84305	0.85268	0.85719	0.85867	0.86346	0.86857	0.87419
413	0.84334	0.85297	0.85746	0.85894	0.86371	0.86881	0.87443
414	0.84365	0.85325	0.85773	0.85921	0.86397	0.86906	0.87466
415	0.84395	0.85353	0.85800	0.85948	0.86423	0.86931	0.87489
416	0.84425	0.85381	0.85826	0.85974	0.86448	0.86955	0.87512
417	0.84455	0.85409	0.85854	0.86001	0.86474	0.86980	0.87536
418	0.84485	0.85436	0.85881	0.86027	0.86499	0.87005	0.87559
419	0.84515	0.85464	0.85907	0.86053	0.86524	0.87029	0.87582
420	0.84543	0.85492	0.85933	0.86079	0.86549	0.87053	0.87605
421	0.84572	0.85519	0.85959	0.86105	0.86575	0.87077	0.87628
422	0.84599	0.85546	0.85985	0.86131	0.86600	0.87101	0.87651
423	0.84631	0.85573	0.86012	0.86157	0.86624	0.87124	0.87674
424	0.84658	0.85600	0.86037	0.86182	0.86649	0.87148	0.87696
425	0.84685	0.85627	0.86063	0.86208	0.86674	0.87172	0.87719
426	0.84714	0.85653	0.86089	0.86234	0.86699	0.87195	0.87741
427	0.84744	0.85680	0.86116	0.86259	0.86723	0.87219	0.87764
428	0.84772	0.85706	0.86141	0.86284	0.86748	0.87242	0.87786
429	0.84803	0.85731	0.86166	0.86310	0.86772	0.87265	0.87809
430	0.84831	0.85758	0.86192	0.86334	0.86796	0.87289	0.87831
431	0.84860	0.85784	0.86217	0.86360	0.86821	0.87312	0.87853
432	0.84888	0.85810	0.86242	0.86384	0.86844	0.87334	0.87874
433	0.84916	0.85836	0.86267	0.86409	0.86868	0.87357	0.87897
434	0.84943	0.85862	0.86293	0.86435	0.86892	0.87380	0.87918
435	0.84972	0.85888	0.86317	0.86460	0.86916	0.87403	0.87940
436	0.84999	0.85915	0.86343	0.86485	0.86939	0.87426	0.87961
437	0.85025	0.85941	0.86368	0.86509	0.86963	0.87449	0.87983
438	0.85054	0.85965	0.86393	0.86533	0.86986	0.87471	0.88004
439	0.85080	0.85991	0.86418	0.86557	0.87010	0.87493	0.88026
440	0.85108	0.86016	0.86442	0.86581	0.87033	0.87515	0.88047
441	0.85133	0.86042	0.86466	0.86606	0.87056	0.87538	0.88068
442	0.85162	0.86068	0.86490	0.86629	0.87079	0.87560	0.88089
443	0.85188	0.86092	0.86514	0.86654	0.87102	0.87582	0.88110
444	0.85214	0.86117	0.86538	0.86678	0.87125	0.87604	0.88131
445	0.85241	0.86142	0.86562	0.86702	0.87148	0.87626	0.88152
446	0.85267	0.86166	0.86587	0.86726	0.87171	0.87648	0.88173
447	0.85293	0.86191	0.86611	0.86749	0.87193	0.87670	0.88193
448	0.85318	0.86216	0.86634	0.86772	0.87216	0.87692	0.88214
449	0.85343	0.86240	0.86658	0.86796	0.87239	0.87713	0.88234
450	0.85369	0.86265	0.86681	0.86820	0.87261	0.87735	0.88255
451	0.85395	0.86290	0.86706	0.86843	0.87283	0.87756	0.88275
452	0.85420	0.86315	0.86728	0.86866	0.87305	0.87777	0.88296
453	0.85448	0.86338	0.86751	0.86889	0.87328	0.87798	0.88316
454	0.85474	0.86362	0.86774	0.86911	0.87350	0.87820	0.88336
455	0.85499	0.86387	0.86798	0.86934	0.87372	0.87841	0.88356
456	0.85526	0.86412	0.86820	0.86957	0.87394	0.87862	0.88376
457	0.85551	0.86436	0.86843	0.86979	0.87416	0.87883	0.88395
458	0.85578	0.86459	0.86866	0.87002	0.87437	0.87904	0.88415
459	0.85602	0.86483	0.86889	0.87025	0.87459	0.87924	0.88435
460	0.85628	0.86506	0.86911	0.87047	0.87480	0.87945	0.88454
461	0.85653	0.86530	0.86934	0.87069	0.87502	0.87965	0.88474
462	0.85678	0.86553	0.86956	0.87092	0.87523	0.87986	0.88494
463	0.85702	0.86576	0.86978	0.87113	0.87544	0.88006	0.88513
464	0.85728	0.86600	0.87000	0.87135	0.87565	0.88027	0.88532
465	0.85749	0.86622	0.87022	0.87157	0.87587	0.88047	0.88552
466	0.85772	0.86646	0.87045	0.87180	0.87608	0.88067	0.88571
467	0.85797	0.86668	0.87067	0.87201	0.87629	0.88087	0.88590
468	0.85821	0.86690	0.87088	0.87222	0.87649	0.88107	0.88609
469	0.85844	0.86713	0.87110	0.87244	0.87670	0.88127	0.88628
470	0.85867	0.86736	0.87132	0.87266	0.87691	0.88147	0.88647
471	0.85893	0.86758	0.87153	0.87287	0.87711	0.88167	0.88666
472	0.85919	0.86780	0.87176	0.87309	0.87731	0.88187	0.88685
473	0.85943	0.86802	0.87198	0.87330	0.87752	0.88206	0.88704

N	0.1%	0.5%	1%	1.25%	2.5%	5%	10%
474	0.85968	0.86825	0.87219	0.87351	0.87773	0.88226	0.88722
475	0.85990	0.86847	0.87240	0.87373	0.87793	0.88246	0.88741
476	0.86013	0.86869	0.87261	0.87393	0.87813	0.88266	0.88759
477	0.86038	0.86890	0.87283	0.87414	0.87834	0.88285	0.88778
478	0.86062	0.86912	0.87304	0.87435	0.87854	0.88304	0.88796
479	0.86086	0.86934	0.87325	0.87457	0.87874	0.88324	0.88815
480	0.86109	0.86955	0.87347	0.87478	0.87894	0.88343	0.88833
481	0.86132	0.86977	0.87367	0.87498	0.87914	0.88362	0.88851
482	0.86154	0.86998	0.87388	0.87519	0.87934	0.88381	0.88870
483	0.86177	0.87020	0.87409	0.87539	0.87954	0.88399	0.88888
484	0.86201	0.87042	0.87430	0.87559	0.87973	0.88418	0.88906
485	0.86224	0.87063	0.87451	0.87580	0.87993	0.88437	0.88924
486	0.86245	0.87084	0.87471	0.87600	0.88013	0.88456	0.88942
487	0.86268	0.87105	0.87491	0.87620	0.88033	0.88475	0.88959
488	0.86290	0.87126	0.87512	0.87640	0.88052	0.88494	0.88977
489	0.86314	0.87146	0.87532	0.87660	0.88071	0.88512	0.88995
490	0.86335	0.87168	0.87553	0.87680	0.88091	0.88531	0.89012
491	0.86355	0.87188	0.87572	0.87700	0.88110	0.88549	0.89030
492	0.86377	0.87209	0.87593	0.87720	0.88129	0.88568	0.89048
493	0.86401	0.87230	0.87613	0.87740	0.88148	0.88586	0.89065
494	0.86422	0.87250	0.87633	0.87759	0.88167	0.88605	0.89083
495	0.86445	0.87272	0.87652	0.87779	0.88187	0.88623	0.89100
496	0.86469	0.87293	0.87672	0.87798	0.88206	0.88641	0.89117
497	0.86492	0.87312	0.87692	0.87818	0.88225	0.88659	0.89134
498	0.86515	0.87333	0.87712	0.87837	0.88243	0.88677	0.89152
499	0.86537	0.87353	0.87732	0.87857	0.88262	0.88695	0.89169
500	0.86558	0.87373	0.87752	0.87877	0.88281	0.88713	0.89186
501	0.86579	0.87394	0.87771	0.87896	0.88299	0.88731	0.89203
502	0.86598	0.87415	0.87791	0.87916	0.88318	0.88748	0.89220
503	0.86620	0.87436	0.87810	0.87935	0.88336	0.88766	0.89236
504	0.86641	0.87455	0.87829	0.87954	0.88355	0.88784	0.89253
505	0.86663	0.87475	0.87849	0.87973	0.88373	0.88801	0.89270
506	0.86686	0.87494	0.87867	0.87992	0.88392	0.88819	0.89286
507	0.86707	0.87514	0.87887	0.88012	0.88410	0.88837	0.89303
508	0.86728	0.87533	0.87906	0.88031	0.88428	0.88854	0.89320
509	0.86751	0.87553	0.87925	0.88050	0.88446	0.88871	0.89337
510	0.86772	0.87574	0.87944	0.88069	0.88464	0.88889	0.89353
511	0.86792	0.87594	0.87963	0.88087	0.88482	0.88906	0.89370
512	0.86813	0.87613	0.87982	0.88106	0.88500	0.88923	0.89386
513	0.86832	0.87633	0.88001	0.88124	0.88518	0.88940	0.89402
514	0.86854	0.87651	0.88020	0.88143	0.88535	0.88957	0.89419
515	0.86874	0.87670	0.88039	0.88161	0.88553	0.88974	0.89435
516	0.86894	0.87690	0.88057	0.88179	0.88570	0.88991	0.89451
517	0.86914	0.87709	0.88076	0.88197	0.88588	0.89008	0.89467
518	0.86934	0.87728	0.88094	0.88216	0.88606	0.89025	0.89483
519	0.86955	0.87747	0.88113	0.88234	0.88623	0.89042	0.89499
520	0.86975	0.87766	0.88132	0.88252	0.88640	0.89058	0.89515
521	0.86997	0.87785	0.88150	0.88270	0.88658	0.89075	0.89531
522	0.87016	0.87804	0.88168	0.88288	0.88675	0.89092	0.89547
523	0.87035	0.87824	0.88185	0.88306	0.88692	0.89108	0.89563
524	0.87054	0.87842	0.88204	0.88324	0.88710	0.89125	0.89578
525	0.87076	0.87860	0.88222	0.88341	0.88727	0.89141	0.89594
526	0.87095	0.87879	0.88240	0.88359	0.88744	0.89158	0.89610
527	0.87115	0.87897	0.88258	0.88377	0.88761	0.89174	0.89625
528	0.87135	0.87916	0.88275	0.88394	0.88778	0.89190	0.89641
529	0.87153	0.87934	0.88293	0.88412	0.88795	0.89207	0.89656
530	0.87170	0.87952	0.88311	0.88429	0.88812	0.89223	0.89672
531	0.87191	0.87970	0.88328	0.88447	0.88828	0.89239	0.89687
532	0.87210	0.87988	0.88345	0.88464	0.88845	0.89255	0.89702
533	0.87231	0.88007	0.88362	0.88482	0.88862	0.89271	0.89718
534	0.87248	0.88025	0.88380	0.88499	0.88879	0.89287	0.89733
535	0.87266	0.88042	0.88398	0.88516	0.88895	0.89302	0.89748
536	0.87288	0.88061	0.88416	0.88533	0.88912	0.89318	0.89763
537	0.87307	0.88079	0.88433	0.88550	0.88929	0.89334	0.89778
538	0.87324	0.88096	0.88450	0.88567	0.88945	0.89350	0.89793
539	0.87344	0.88113	0.88467	0.88584	0.88961	0.89366	0.89808
540	0.87362	0.88131	0.88484	0.88601	0.88978	0.89381	0.89823
541	0.87381	0.88149	0.88501	0.88619	0.88994	0.89397	0.89838
542	0.87400	0.88167	0.88518	0.88635	0.89010	0.89412	0.89853
543	0.87418	0.88184	0.88535	0.88652	0.89026	0.89428	0.89868
544	0.87437	0.88202	0.88552	0.88668	0.89042	0.89444	0.89882
545	0.87458	0.88220	0.88570	0.88685	0.89058	0.89459	0.89897
546	0.87477	0.88237	0.88587	0.88701	0.89074	0.89474	0.89912
547	0.87496	0.88254	0.88603	0.88718	0.89090	0.89490	0.89926

N	0.1%	0.5%	1%	1.25%	2.5%	5%	10%
548	0.87514	0.88272	0.88619	0.88735	0.89106	0.89505	0.89941
549	0.87532	0.88290	0.88636	0.88751	0.89122	0.89521	0.89955
550	0.87552	0.88307	0.88653	0.88768	0.89138	0.89536	0.89970
551	0.87570	0.88324	0.88669	0.88784	0.89154	0.89551	0.89984
552	0.87587	0.88341	0.88686	0.88800	0.89170	0.89566	0.89999
553	0.87605	0.88358	0.88702	0.88817	0.89185	0.89581	0.90013
554	0.87624	0.88375	0.88719	0.88833	0.89201	0.89596	0.90027
555	0.87641	0.88392	0.88735	0.88849	0.89217	0.89611	0.90041
556	0.87660	0.88408	0.88751	0.88865	0.89232	0.89626	0.90056
557	0.87680	0.88424	0.88767	0.88882	0.89248	0.89641	0.90070
558	0.87699	0.88441	0.88784	0.88898	0.89263	0.89656	0.90084
559	0.87717	0.88458	0.88801	0.88913	0.89279	0.89670	0.90098
560	0.87734	0.88475	0.88817	0.88929	0.89294	0.89685	0.90112
561	0.87751	0.88491	0.88833	0.88945	0.89309	0.89699	0.90126
562	0.87769	0.88508	0.88849	0.88961	0.89324	0.89714	0.90140
563	0.87786	0.88525	0.88864	0.88977	0.89340	0.89729	0.90154
564	0.87804	0.88541	0.88880	0.88992	0.89355	0.89743	0.90168
565	0.87821	0.88558	0.88896	0.89008	0.89370	0.89758	0.90181
566	0.87839	0.88575	0.88913	0.89025	0.89385	0.89772	0.90195
567	0.87858	0.88591	0.88929	0.89040	0.89400	0.89786	0.90209
568	0.87875	0.88607	0.88944	0.89056	0.89415	0.89801	0.90223
569	0.87892	0.88623	0.88960	0.89071	0.89430	0.89815	0.90236
570	0.87908	0.88638	0.88975	0.89087	0.89445	0.89830	0.90250
571	0.87924	0.88655	0.88991	0.89102	0.89460	0.89844	0.90263
572	0.87943	0.88671	0.89006	0.89117	0.89475	0.89858	0.90277
573	0.87959	0.88686	0.89022	0.89132	0.89489	0.89872	0.90290
574	0.87977	0.88703	0.89037	0.89147	0.89504	0.89886	0.90304
575	0.87994	0.88718	0.89052	0.89162	0.89519	0.89900	0.90317
576	0.88009	0.88733	0.89067	0.89177	0.89533	0.89914	0.90331
577	0.88027	0.88749	0.89083	0.89192	0.89548	0.89928	0.90344
578	0.88042	0.88765	0.89098	0.89207	0.89562	0.89942	0.90357
579	0.88060	0.88781	0.89113	0.89222	0.89577	0.89956	0.90370
580	0.88077	0.88796	0.89128	0.89237	0.89591	0.89969	0.90384
581	0.88094	0.88812	0.89144	0.89252	0.89606	0.89983	0.90397
582	0.88110	0.88828	0.89158	0.89267	0.89620	0.89997	0.90410
583	0.88127	0.88844	0.89174	0.89281	0.89634	0.90011	0.90423
584	0.88145	0.88859	0.89188	0.89296	0.89648	0.90024	0.90436
585	0.88162	0.88874	0.89203	0.89311	0.89662	0.90038	0.90449
586	0.88176	0.88890	0.89218	0.89326	0.89676	0.90051	0.90462
587	0.88192	0.88906	0.89233	0.89340	0.89691	0.90065	0.90475
588	0.88211	0.88921	0.89248	0.89355	0.89705	0.90078	0.90488
589	0.88226	0.88936	0.89263	0.89369	0.89719	0.90092	0.90501
590	0.88243	0.88951	0.89278	0.89384	0.89733	0.90105	0.90513
591	0.88259	0.88967	0.89292	0.89398	0.89747	0.90118	0.90526
592	0.88274	0.88982	0.89307	0.89413	0.89761	0.90132	0.90539
593	0.88291	0.88997	0.89321	0.89428	0.89775	0.90145	0.90552
594	0.88307	0.89012	0.89336	0.89442	0.89789	0.90159	0.90564
595	0.88323	0.89027	0.89350	0.89456	0.89802	0.90172	0.90577
596	0.88339	0.89042	0.89364	0.89471	0.89816	0.90185	0.90589
597	0.88355	0.89057	0.89378	0.89485	0.89830	0.90198	0.90602
598	0.88371	0.89072	0.89393	0.89499	0.89844	0.90211	0.90615
599	0.88387	0.89086	0.89407	0.89513	0.89857	0.90224	0.90627
600	0.88401	0.89101	0.89422	0.89527	0.89871	0.90237	0.90639
601	0.88416	0.89116	0.89436	0.89542	0.89885	0.90250	0.90652
602	0.88432	0.89130	0.89450	0.89555	0.89898	0.90263	0.90664
603	0.88449	0.89145	0.89464	0.89569	0.89912	0.90276	0.90676
604	0.88464	0.89159	0.89478	0.89583	0.89925	0.90288	0.90688
605	0.88480	0.89173	0.89492	0.89597	0.89938	0.90301	0.90701
606	0.88494	0.89188	0.89506	0.89611	0.89951	0.90314	0.90713
607	0.88511	0.89202	0.89520	0.89625	0.89965	0.90327	0.90725
608	0.88526	0.89216	0.89534	0.89638	0.89978	0.90340	0.90737
609	0.88542	0.89230	0.89548	0.89652	0.89991	0.90352	0.90749
610	0.88558	0.89245	0.89562	0.89666	0.90004	0.90365	0.90761
611	0.88573	0.89258	0.89576	0.89679	0.90017	0.90378	0.90773
612	0.88589	0.89272	0.89590	0.89693	0.90030	0.90390	0.90786
613	0.88602	0.89287	0.89603	0.89707	0.90043	0.90403	0.90798
614	0.88618	0.89301	0.89617	0.89720	0.90056	0.90416	0.90809
615	0.88633	0.89315	0.89630	0.89734	0.90069	0.90428	0.90821
616	0.88648	0.89330	0.89644	0.89747	0.90082	0.90441	0.90833
617	0.88664	0.89344	0.89658	0.89761	0.90096	0.90453	0.90845
618	0.88677	0.89358	0.89671	0.89774	0.90108	0.90466	0.90857
619	0.88692	0.89371	0.89684	0.89788	0.90121	0.90478	0.90869
620	0.88706	0.89385	0.89698	0.89801	0.90134	0.90490	0.90880
621	0.88720	0.89399	0.89712	0.89814	0.90147	0.90503	0.90892

N	0.1%	0.5%	1%	1.25%	2.5%	5%	10%
622	0.88735	0.89413	0.89725	0.89827	0.90160	0.90515	0.90904
623	0.88750	0.89427	0.89738	0.89840	0.90172	0.90527	0.90915
624	0.88765	0.89441	0.89751	0.89853	0.90185	0.90539	0.90927
625	0.88779	0.89454	0.89765	0.89866	0.90198	0.90551	0.90939
626	0.88795	0.89467	0.89778	0.89879	0.90211	0.90563	0.90950
627	0.88810	0.89481	0.89791	0.89892	0.90223	0.90575	0.90962
628	0.88826	0.89494	0.89804	0.89905	0.90236	0.90587	0.90973
629	0.88839	0.89508	0.89818	0.89918	0.90248	0.90600	0.90985
630	0.88854	0.89521	0.89830	0.89932	0.90261	0.90612	0.90996
631	0.88868	0.89535	0.89844	0.89944	0.90273	0.90623	0.91007
632	0.88882	0.89548	0.89857	0.89957	0.90285	0.90636	0.91019
633	0.88895	0.89561	0.89870	0.89970	0.90298	0.90647	0.91030
634	0.88910	0.89575	0.89883	0.89983	0.90310	0.90659	0.91041
635	0.88925	0.89588	0.89896	0.89995	0.90322	0.90671	0.91053
636	0.88939	0.89602	0.89908	0.90008	0.90335	0.90683	0.91064
637	0.88955	0.89615	0.89921	0.90021	0.90347	0.90694	0.91075
638	0.88971	0.89628	0.89934	0.90034	0.90359	0.90706	0.91086
639	0.88985	0.89641	0.89947	0.90046	0.90371	0.90718	0.91098
640	0.88998	0.89654	0.89959	0.90058	0.90383	0.90729	0.91108
641	0.89012	0.89667	0.89972	0.90071	0.90396	0.90741	0.91120
642	0.89027	0.89680	0.89985	0.90084	0.90407	0.90753	0.91130
643	0.89040	0.89692	0.89997	0.90096	0.90419	0.90764	0.91142
644	0.89054	0.89705	0.90010	0.90109	0.90431	0.90776	0.91152
645	0.89068	0.89719	0.90023	0.90121	0.90443	0.90787	0.91163
646	0.89080	0.89731	0.90035	0.90134	0.90455	0.90799	0.91175
647	0.89094	0.89744	0.90048	0.90146	0.90467	0.90810	0.91185
648	0.89109	0.89757	0.90060	0.90158	0.90479	0.90821	0.91196
649	0.89122	0.89770	0.90072	0.90171	0.90491	0.90833	0.91207
650	0.89136	0.89783	0.90084	0.90183	0.90502	0.90844	0.91218
651	0.89150	0.89796	0.90096	0.90195	0.90514	0.90855	0.91229
652	0.89163	0.89809	0.90108	0.90207	0.90526	0.90867	0.91239
653	0.89177	0.89822	0.90121	0.90219	0.90537	0.90878	0.91250
654	0.89191	0.89834	0.90133	0.90232	0.90549	0.90889	0.91261
655	0.89204	0.89847	0.90145	0.90243	0.90561	0.90901	0.91272
656	0.89217	0.89859	0.90157	0.90256	0.90572	0.90912	0.91282
657	0.89231	0.89872	0.90169	0.90268	0.90584	0.90923	0.91293
658	0.89244	0.89885	0.90181	0.90280	0.90595	0.90934	0.91303
659	0.89258	0.89897	0.90193	0.90292	0.90607	0.90945	0.91314
660	0.89270	0.89910	0.90205	0.90304	0.90618	0.90956	0.91324
661	0.89284	0.89922	0.90218	0.90316	0.90630	0.90967	0.91335
662	0.89297	0.89934	0.90229	0.90327	0.90641	0.90978	0.91345
663	0.89311	0.89946	0.90241	0.90339	0.90652	0.90989	0.91356
664	0.89324	0.89959	0.90253	0.90351	0.90664	0.91000	0.91366
665	0.89337	0.89972	0.90265	0.90362	0.90675	0.91011	0.91377
666	0.89350	0.89984	0.90277	0.90374	0.90687	0.91022	0.91387
667	0.89363	0.89996	0.90289	0.90386	0.90698	0.91033	0.91397
668	0.89376	0.90007	0.90300	0.90397	0.90709	0.91043	0.91408
669	0.89389	0.90019	0.90312	0.90409	0.90720	0.91054	0.91418
670	0.89402	0.90032	0.90324	0.90421	0.90731	0.91065	0.91428
671	0.89415	0.90044	0.90336	0.90432	0.90743	0.91075	0.91438
672	0.89428	0.90056	0.90348	0.90443	0.90754	0.91086	0.91449
673	0.89442	0.90069	0.90359	0.90455	0.90765	0.91097	0.91459
674	0.89454	0.90081	0.90370	0.90467	0.90776	0.91107	0.91469
675	0.89467	0.90092	0.90382	0.90478	0.90787	0.91118	0.91479
676	0.89479	0.90104	0.90393	0.90490	0.90798	0.91129	0.91489
677	0.89492	0.90116	0.90405	0.90501	0.90809	0.91139	0.91499
678	0.89505	0.90128	0.90416	0.90512	0.90820	0.91150	0.91509
679	0.89518	0.90140	0.90428	0.90524	0.90831	0.91160	0.91519
680	0.89531	0.90152	0.90440	0.90535	0.90842	0.91171	0.91530
681	0.89543	0.90164	0.90451	0.90546	0.90853	0.91181	0.91539
682	0.89556	0.90175	0.90463	0.90558	0.90864	0.91191	0.91549
683	0.89569	0.90188	0.90474	0.90569	0.90874	0.91202	0.91559
684	0.89582	0.90199	0.90485	0.90580	0.90885	0.91212	0.91569
685	0.89594	0.90211	0.90497	0.90592	0.90896	0.91223	0.91579
686	0.89606	0.90222	0.90508	0.90603	0.90907	0.91233	0.91589
687	0.89620	0.90234	0.90519	0.90614	0.90917	0.91243	0.91598
688	0.89632	0.90245	0.90530	0.90625	0.90928	0.91253	0.91608
689	0.89643	0.90257	0.90541	0.90636	0.90938	0.91263	0.91618
690	0.89656	0.90268	0.90552	0.90647	0.90949	0.91274	0.91628
691	0.89669	0.90280	0.90564	0.90658	0.90959	0.91284	0.91637
692	0.89681	0.90292	0.90575	0.90668	0.90970	0.91294	0.91647
693	0.89692	0.90303	0.90586	0.90679	0.90981	0.91304	0.91657
694	0.89706	0.90315	0.90597	0.90690	0.90991	0.91314	0.91666
695	0.89718	0.90326	0.90608	0.90701	0.91002	0.91324	0.91676

N	0.1%	0.5%	1%	1.25%	2.5%	5%	10%
696	0.89730	0.90338	0.90619	0.90712	0.91012	0.91334	0.91686
697	0.89742	0.90349	0.90630	0.90723	0.91022	0.91344	0.91695
698	0.89754	0.90361	0.90641	0.90733	0.91033	0.91354	0.91705
699	0.89766	0.90372	0.90652	0.90744	0.91043	0.91364	0.91714
700	0.89777	0.90383	0.90662	0.90755	0.91053	0.91374	0.91724
701	0.89789	0.90394	0.90673	0.90766	0.91064	0.91384	0.91733
702	0.89801	0.90405	0.90684	0.90777	0.91074	0.91394	0.91742
703	0.89814	0.90416	0.90694	0.90787	0.91084	0.91404	0.91752
704	0.89825	0.90427	0.90706	0.90798	0.91094	0.91414	0.91761
705	0.89836	0.90438	0.90716	0.90809	0.91105	0.91424	0.91771
706	0.89848	0.90449	0.90727	0.90819	0.91115	0.91434	0.91780
707	0.89860	0.90461	0.90738	0.90830	0.91125	0.91443	0.91789
708	0.89871	0.90472	0.90748	0.90841	0.91135	0.91453	0.91799
709	0.89884	0.90483	0.90759	0.90851	0.91146	0.91463	0.91808
710	0.89896	0.90494	0.90770	0.90862	0.91156	0.91472	0.91817
711	0.89907	0.90504	0.90780	0.90872	0.91166	0.91482	0.91827
712	0.89918	0.90515	0.90791	0.90883	0.91176	0.91492	0.91836
713	0.89929	0.90527	0.90801	0.90893	0.91186	0.91501	0.91845
714	0.89941	0.90538	0.90812	0.90903	0.91196	0.91511	0.91854
715	0.89953	0.90548	0.90822	0.90914	0.91206	0.91520	0.91863
716	0.89964	0.90559	0.90832	0.90924	0.91216	0.91530	0.91872
717	0.89976	0.90570	0.90843	0.90934	0.91226	0.91540	0.91882
718	0.89988	0.90581	0.90854	0.90944	0.91236	0.91549	0.91891
719	0.89999	0.90592	0.90864	0.90955	0.91246	0.91559	0.91900
720	0.90011	0.90603	0.90874	0.90965	0.91256	0.91568	0.91909
721	0.90023	0.90613	0.90884	0.90975	0.91266	0.91577	0.91918
722	0.90034	0.90623	0.90894	0.90985	0.91275	0.91587	0.91927
723	0.90046	0.90634	0.90904	0.90995	0.91285	0.91596	0.91936
724	0.90057	0.90645	0.90915	0.91005	0.91295	0.91605	0.91945
725	0.90068	0.90656	0.90925	0.91016	0.91305	0.91615	0.91953
726	0.90079	0.90666	0.90935	0.91025	0.91314	0.91624	0.91962
727	0.90091	0.90676	0.90945	0.91035	0.91324	0.91633	0.91971
728	0.90101	0.90687	0.90955	0.91045	0.91334	0.91643	0.91980
729	0.90112	0.90697	0.90965	0.91055	0.91343	0.91652	0.91989
730	0.90124	0.90707	0.90976	0.91066	0.91353	0.91661	0.91997
731	0.90135	0.90718	0.90986	0.91076	0.91363	0.91670	0.92006
732	0.90148	0.90729	0.90996	0.91086	0.91372	0.91679	0.92015
733	0.90159	0.90739	0.91006	0.91096	0.91382	0.91689	0.92024
734	0.90169	0.90750	0.91016	0.91106	0.91391	0.91698	0.92033
735	0.90180	0.90760	0.91026	0.91116	0.91401	0.91707	0.92041
736	0.90191	0.90770	0.91036	0.91125	0.91410	0.91716	0.92050
737	0.90203	0.90780	0.91046	0.91135	0.91420	0.91725	0.92059
738	0.90213	0.90790	0.91055	0.91145	0.91429	0.91734	0.92067
739	0.90224	0.90800	0.91066	0.91155	0.91439	0.91743	0.92076
740	0.90234	0.90810	0.91076	0.91165	0.91448	0.91752	0.92085
741	0.90245	0.90820	0.91085	0.91175	0.91457	0.91761	0.92093
742	0.90257	0.90830	0.91095	0.91184	0.91467	0.91770	0.92102
743	0.90268	0.90841	0.91105	0.91194	0.91476	0.91779	0.92110
744	0.90278	0.90851	0.91115	0.91203	0.91485	0.91788	0.92119
745	0.90289	0.90861	0.91124	0.91213	0.91495	0.91797	0.92127
746	0.90300	0.90871	0.91134	0.91222	0.91504	0.91806	0.92136
747	0.90311	0.90882	0.91144	0.91232	0.91513	0.91815	0.92144
748	0.90323	0.90891	0.91154	0.91242	0.91522	0.91824	0.92153
749	0.90334	0.90901	0.91163	0.91251	0.91532	0.91832	0.92161
750	0.90344	0.90911	0.91173	0.91261	0.91541	0.91841	0.92170
751	0.90354	0.90921	0.91182	0.91271	0.91550	0.91850	0.92178
752	0.90365	0.90931	0.91192	0.91280	0.91559	0.91859	0.92186
753	0.90374	0.90940	0.91202	0.91290	0.91568	0.91868	0.92195
754	0.90384	0.90950	0.91211	0.91299	0.91577	0.91876	0.92203
755	0.90396	0.90960	0.91221	0.91308	0.91586	0.91885	0.92211
756	0.90407	0.90971	0.91230	0.91318	0.91595	0.91894	0.92220
757	0.90416	0.90980	0.91240	0.91327	0.91605	0.91902	0.92228
758	0.90427	0.90990	0.91249	0.91337	0.91614	0.91911	0.92236
759	0.90437	0.91000	0.91259	0.91346	0.91623	0.91920	0.92244
760	0.90448	0.91010	0.91268	0.91355	0.91632	0.91928	0.92253
761	0.90458	0.91020	0.91277	0.91364	0.91640	0.91937	0.92261
762	0.90468	0.91029	0.91287	0.91373	0.91650	0.91946	0.92269
763	0.90478	0.91039	0.91296	0.91383	0.91658	0.91954	0.92277
764	0.90488	0.91048	0.91306	0.91392	0.91667	0.91962	0.92285
765	0.90498	0.91058	0.91315	0.91401	0.91676	0.91971	0.92294
766	0.90509	0.91067	0.91324	0.91410	0.91685	0.91980	0.92302
767	0.90519	0.91078	0.91334	0.91420	0.91693	0.91988	0.92310
768	0.90529	0.91087	0.91343	0.91429	0.91702	0.91996	0.92318
769	0.90540	0.91096	0.91352	0.91438	0.91711	0.92005	0.92326

N	0.1%	0.5%	1%	1.25%	2.5%	5%	10%
770	0.90549	0.91106	0.91362	0.91447	0.91720	0.92013	0.92334
771	0.90558	0.91116	0.91370	0.91456	0.91729	0.92022	0.92342
772	0.90569	0.91125	0.91379	0.91465	0.91737	0.92030	0.92350
773	0.90580	0.91135	0.91388	0.91474	0.91746	0.92038	0.92358
774	0.90590	0.91144	0.91397	0.91483	0.91755	0.92047	0.92366
775	0.90601	0.91153	0.91406	0.91492	0.91763	0.92055	0.92374
776	0.90611	0.91163	0.91415	0.91501	0.91772	0.92063	0.92381
777	0.90622	0.91172	0.91424	0.91510	0.91781	0.92071	0.92389
778	0.90631	0.91181	0.91433	0.91519	0.91789	0.92079	0.92397
779	0.90641	0.91190	0.91442	0.91527	0.91798	0.92088	0.92405
780	0.90652	0.91200	0.91451	0.91536	0.91806	0.92096	0.92413
781	0.90661	0.91209	0.91460	0.91545	0.91815	0.92104	0.92421
782	0.90671	0.91219	0.91469	0.91554	0.91824	0.92112	0.92429
783	0.90682	0.91228	0.91478	0.91563	0.91832	0.92120	0.92436
784	0.90691	0.91237	0.91487	0.91571	0.91841	0.92129	0.92444
785	0.90701	0.91246	0.91496	0.91580	0.91849	0.92137	0.92452
786	0.90710	0.91255	0.91505	0.91589	0.91857	0.92145	0.92460
787	0.90720	0.91264	0.91514	0.91597	0.91866	0.92153	0.92467
788	0.90728	0.91273	0.91522	0.91606	0.91874	0.92161	0.92475
789	0.90739	0.91282	0.91531	0.91615	0.91882	0.92169	0.92483
790	0.90748	0.91292	0.91540	0.91623	0.91891	0.92177	0.92491
791	0.90759	0.91301	0.91549	0.91632	0.91899	0.92185	0.92498
792	0.90769	0.91310	0.91558	0.91640	0.91907	0.92193	0.92506
793	0.90778	0.91319	0.91566	0.91649	0.91916	0.92202	0.92514
794	0.90788	0.91328	0.91575	0.91658	0.91924	0.92209	0.92521
795	0.90798	0.91337	0.91584	0.91666	0.91932	0.92218	0.92529
796	0.90807	0.91346	0.91593	0.91675	0.91941	0.92225	0.92536
797	0.90816	0.91355	0.91601	0.91684	0.91949	0.92233	0.92544
798	0.90826	0.91364	0.91610	0.91692	0.91957	0.92241	0.92552
799	0.90835	0.91373	0.91619	0.91701	0.91965	0.92249	0.92559
800	0.90845	0.91382	0.91627	0.91709	0.91973	0.92257	0.92567
801	0.90855	0.91390	0.91635	0.91717	0.91981	0.92265	0.92574
802	0.90864	0.91399	0.91644	0.91726	0.91990	0.92272	0.92582
803	0.90873	0.91408	0.91652	0.91734	0.91998	0.92280	0.92589
804	0.90884	0.91417	0.91661	0.91743	0.92006	0.92288	0.92596
805	0.90892	0.91426	0.91669	0.91751	0.92014	0.92296	0.92604
806	0.90902	0.91435	0.91678	0.91760	0.92022	0.92304	0.92611
807	0.90912	0.91443	0.91687	0.91768	0.92030	0.92311	0.92619
808	0.90921	0.91452	0.91695	0.91776	0.92038	0.92319	0.92626
809	0.90931	0.91460	0.91704	0.91785	0.92046	0.92327	0.92633
810	0.90941	0.91469	0.91712	0.91793	0.92054	0.92335	0.92641
811	0.90950	0.91478	0.91721	0.91801	0.92062	0.92342	0.92648
812	0.90959	0.91486	0.91729	0.91809	0.92070	0.92350	0.92655
813	0.90968	0.91495	0.91737	0.91817	0.92078	0.92357	0.92663
814	0.90978	0.91503	0.91745	0.91826	0.92086	0.92365	0.92670
815	0.90987	0.91512	0.91754	0.91834	0.92094	0.92373	0.92677
816	0.90996	0.91521	0.91762	0.91842	0.92102	0.92380	0.92684
817	0.91005	0.91530	0.91770	0.91850	0.92110	0.92388	0.92692
818	0.91014	0.91538	0.91779	0.91858	0.92117	0.92395	0.92699
819	0.91023	0.91547	0.91787	0.91867	0.92125	0.92403	0.92706
820	0.91032	0.91555	0.91795	0.91875	0.92133	0.92410	0.92713
821	0.91041	0.91563	0.91803	0.91883	0.92141	0.92418	0.92720
822	0.91051	0.91572	0.91811	0.91891	0.92149	0.92425	0.92728
823	0.91061	0.91580	0.91819	0.91899	0.92157	0.92433	0.92735
824	0.91070	0.91588	0.91827	0.91907	0.92165	0.92440	0.92742
825	0.91079	0.91597	0.91836	0.91915	0.92172	0.92448	0.92749
826	0.91087	0.91605	0.91844	0.91923	0.92180	0.92455	0.92756
827	0.91096	0.91613	0.91852	0.91931	0.92188	0.92463	0.92763
828	0.91106	0.91622	0.91860	0.91940	0.92196	0.92470	0.92770
829	0.91114	0.91630	0.91869	0.91947	0.92203	0.92477	0.92777
830	0.91123	0.91638	0.91876	0.91955	0.92211	0.92485	0.92784
831	0.91132	0.91647	0.91884	0.91963	0.92218	0.92492	0.92791
832	0.91140	0.91656	0.91892	0.91971	0.92226	0.92499	0.92798
833	0.91149	0.91664	0.91901	0.91979	0.92234	0.92507	0.92805
834	0.91157	0.91671	0.91909	0.91987	0.92241	0.92514	0.92812
835	0.91166	0.91680	0.91917	0.91995	0.92249	0.92521	0.92819
836	0.91175	0.91688	0.91925	0.92003	0.92257	0.92528	0.92826
837	0.91183	0.91697	0.91933	0.92011	0.92264	0.92536	0.92833
838	0.91193	0.91705	0.91940	0.92018	0.92272	0.92543	0.92840
839	0.91201	0.91713	0.91948	0.92026	0.92279	0.92550	0.92847
840	0.91209	0.91721	0.91956	0.92034	0.92287	0.92558	0.92854
841	0.91219	0.91729	0.91964	0.92042	0.92294	0.92565	0.92861
842	0.91227	0.91738	0.91972	0.92050	0.92301	0.92572	0.92868
843	0.91236	0.91746	0.91980	0.92057	0.92309	0.92579	0.92874

N	0.1%	0.5%	1%	1.25%	2.5%	5%	10%
844	0.91244	0.91754	0.91987	0.92065	0.92316	0.92586	0.92881
845	0.91253	0.91762	0.91995	0.92073	0.92324	0.92593	0.92888
846	0.91261	0.91770	0.92003	0.92081	0.92331	0.92601	0.92895
847	0.91269	0.91778	0.92011	0.92088	0.92339	0.92608	0.92902
848	0.91278	0.91786	0.92018	0.92096	0.92346	0.92615	0.92908
849	0.91286	0.91794	0.92026	0.92103	0.92353	0.92622	0.92915
850	0.91294	0.91802	0.92033	0.92111	0.92361	0.92629	0.92922
851	0.91302	0.91810	0.92041	0.92118	0.92368	0.92636	0.92929
852	0.91311	0.91817	0.92049	0.92126	0.92375	0.92643	0.92935
853	0.91319	0.91826	0.92056	0.92134	0.92382	0.92650	0.92942
854	0.91328	0.91833	0.92064	0.92141	0.92390	0.92657	0.92949
855	0.91336	0.91841	0.92071	0.92149	0.92397	0.92664	0.92955
856	0.91344	0.91849	0.92079	0.92156	0.92404	0.92671	0.92962
857	0.91353	0.91857	0.92087	0.92163	0.92411	0.92678	0.92969
858	0.91364	0.91865	0.92095	0.92171	0.92419	0.92685	0.92975
859	0.91372	0.91874	0.92102	0.92179	0.92426	0.92692	0.92982
860	0.91380	0.91881	0.92110	0.92186	0.92433	0.92699	0.92989
861	0.91388	0.91889	0.92118	0.92194	0.92440	0.92706	0.92995
862	0.91396	0.91896	0.92125	0.92201	0.92448	0.92713	0.93002
863	0.91404	0.91904	0.92132	0.92208	0.92455	0.92719	0.93008
864	0.91413	0.91911	0.92140	0.92216	0.92462	0.92726	0.93015
865	0.91421	0.91919	0.92147	0.92223	0.92469	0.92733	0.93022
866	0.91430	0.91927	0.92154	0.92230	0.92476	0.92740	0.93028
867	0.91438	0.91934	0.92162	0.92238	0.92483	0.92747	0.93035
868	0.91445	0.91942	0.92170	0.92245	0.92490	0.92753	0.93041
869	0.91453	0.91949	0.92177	0.92253	0.92497	0.92760	0.93047
870	0.91461	0.91958	0.92184	0.92260	0.92505	0.92767	0.93054
871	0.91469	0.91965	0.92192	0.92267	0.92512	0.92774	0.93060
872	0.91477	0.91973	0.92199	0.92274	0.92519	0.92780	0.93067
873	0.91485	0.91981	0.92206	0.92281	0.92526	0.92787	0.93073
874	0.91493	0.91988	0.92213	0.92289	0.92533	0.92794	0.93080
875	0.91502	0.91996	0.92221	0.92296	0.92540	0.92800	0.93086
876	0.91509	0.92003	0.92228	0.92303	0.92547	0.92807	0.93092
877	0.91517	0.92011	0.92235	0.92311	0.92554	0.92814	0.93099
878	0.91525	0.92018	0.92243	0.92318	0.92561	0.92821	0.93105
879	0.91533	0.92026	0.92250	0.92325	0.92567	0.92827	0.93112
880	0.91542	0.92033	0.92257	0.92332	0.92574	0.92834	0.93118
881	0.91550	0.92040	0.92264	0.92339	0.92581	0.92841	0.93124
882	0.91557	0.92047	0.92272	0.92346	0.92588	0.92847	0.93131
883	0.91566	0.92055	0.92279	0.92353	0.92595	0.92854	0.93137
884	0.91573	0.92062	0.92286	0.92360	0.92602	0.92860	0.93143
885	0.91580	0.92069	0.92293	0.92367	0.92609	0.92867	0.93149
886	0.91588	0.92077	0.92300	0.92374	0.92616	0.92873	0.93156
887	0.91596	0.92084	0.92307	0.92382	0.92623	0.92880	0.93162
888	0.91604	0.92091	0.92315	0.92389	0.92630	0.92887	0.93168
889	0.91611	0.92098	0.92322	0.92396	0.92636	0.92893	0.93175
890	0.91619	0.92105	0.92329	0.92403	0.92643	0.92900	0.93181
891	0.91627	0.92113	0.92336	0.92410	0.92650	0.92906	0.93187
892	0.91635	0.92120	0.92343	0.92417	0.92657	0.92913	0.93193
893	0.91642	0.92127	0.92350	0.92424	0.92663	0.92919	0.93199
894	0.91651	0.92135	0.92357	0.92431	0.92670	0.92925	0.93205
895	0.91659	0.92142	0.92364	0.92438	0.92677	0.92932	0.93212
896	0.91666	0.92149	0.92371	0.92445	0.92684	0.92938	0.93218
897	0.91674	0.92157	0.92378	0.92452	0.92690	0.92945	0.93224
898	0.91681	0.92164	0.92385	0.92459	0.92697	0.92951	0.93230
899	0.91689	0.92171	0.92392	0.92466	0.92704	0.92958	0.93236
900	0.91697	0.92178	0.92399	0.92473	0.92710	0.92964	0.93242
901	0.91704	0.92186	0.92407	0.92480	0.92717	0.92970	0.93248
902	0.91712	0.92193	0.92414	0.92486	0.92723	0.92977	0.93255
903	0.91720	0.92200	0.92420	0.92493	0.92730	0.92983	0.93261
904	0.91727	0.92207	0.92427	0.92500	0.92737	0.92989	0.93267
905	0.91734	0.92214	0.92434	0.92507	0.92743	0.92996	0.93273
906	0.91742	0.92221	0.92441	0.92514	0.92750	0.93002	0.93279
907	0.91749	0.92229	0.92448	0.92521	0.92756	0.93008	0.93285
908	0.91757	0.92235	0.92455	0.92528	0.92763	0.93015	0.93291
909	0.91764	0.92242	0.92462	0.92534	0.92769	0.93021	0.93297
910	0.91771	0.92249	0.92468	0.92541	0.92776	0.93027	0.93303
911	0.91779	0.92256	0.92475	0.92548	0.92782	0.93033	0.93309
912	0.91786	0.92263	0.92482	0.92555	0.92789	0.93039	0.93315
913	0.91794	0.92270	0.92489	0.92561	0.92795	0.93046	0.93320
914	0.91802	0.92277	0.92496	0.92568	0.92801	0.93052	0.93326
915	0.91810	0.92284	0.92503	0.92575	0.92808	0.93058	0.93332
916	0.91818	0.92291	0.92509	0.92581	0.92814	0.93064	0.93338
917	0.91825	0.92298	0.92516	0.92588	0.92821	0.93070	0.93344

N	0.1%	0.5%	1%	1.25%	2.5%	5%	10%
918	0.91832	0.92305	0.92523	0.92595	0.92827	0.93077	0.93350
919	0.91840	0.92312	0.92529	0.92601	0.92833	0.93083	0.93356
920	0.91847	0.92319	0.92536	0.92608	0.92840	0.93089	0.93362
921	0.91855	0.92326	0.92543	0.92614	0.92846	0.93095	0.93368
922	0.91862	0.92333	0.92550	0.92621	0.92853	0.93101	0.93373
923	0.91871	0.92340	0.92556	0.92628	0.92859	0.93107	0.93379
924	0.91877	0.92347	0.92563	0.92634	0.92866	0.93113	0.93385
925	0.91884	0.92353	0.92569	0.92641	0.92872	0.93119	0.93391
926	0.91892	0.92360	0.92576	0.92647	0.92878	0.93125	0.93397
927	0.91899	0.92367	0.92582	0.92654	0.92885	0.93131	0.93402
928	0.91907	0.92374	0.92589	0.92660	0.92891	0.93137	0.93408
929	0.91915	0.92381	0.92595	0.92667	0.92897	0.93144	0.93414
930	0.91921	0.92387	0.92602	0.92673	0.92903	0.93150	0.93420
931	0.91929	0.92394	0.92609	0.92680	0.92910	0.93156	0.93426
932	0.91936	0.92401	0.92615	0.92686	0.92916	0.93162	0.93431
933	0.91943	0.92408	0.92621	0.92693	0.92922	0.93167	0.93437
934	0.91951	0.92414	0.92628	0.92699	0.92928	0.93173	0.93443
935	0.91958	0.92421	0.92634	0.92706	0.92934	0.93179	0.93448
936	0.91965	0.92428	0.92641	0.92712	0.92941	0.93186	0.93454
937	0.91973	0.92435	0.92647	0.92718	0.92947	0.93192	0.93460
938	0.91980	0.92441	0.92654	0.92725	0.92953	0.93198	0.93465
939	0.91988	0.92448	0.92661	0.92731	0.92959	0.93203	0.93471
940	0.91994	0.92454	0.92667	0.92738	0.92965	0.93209	0.93477
941	0.92002	0.92460	0.92673	0.92744	0.92971	0.93215	0.93483
942	0.92009	0.92467	0.92680	0.92750	0.92977	0.93221	0.93488
943	0.92015	0.92474	0.92686	0.92757	0.92984	0.93227	0.93494
944	0.92022	0.92480	0.92693	0.92763	0.92990	0.93233	0.93499
945	0.92028	0.92487	0.92699	0.92769	0.92996	0.93239	0.93505
946	0.92036	0.92493	0.92705	0.92776	0.93002	0.93245	0.93511
947	0.92042	0.92500	0.92712	0.92782	0.93008	0.93251	0.93516
948	0.92049	0.92506	0.92718	0.92788	0.93014	0.93256	0.93522
949	0.92056	0.92513	0.92724	0.92795	0.93020	0.93262	0.93527
950	0.92063	0.92520	0.92731	0.92801	0.93026	0.93268	0.93533
951	0.92069	0.92526	0.92737	0.92807	0.93032	0.93274	0.93538
952	0.92076	0.92533	0.92743	0.92813	0.93038	0.93280	0.93544
953	0.92083	0.92540	0.92749	0.92820	0.93044	0.93285	0.93549
954	0.92090	0.92546	0.92756	0.92826	0.93050	0.93291	0.93555
955	0.92096	0.92553	0.92762	0.92832	0.93056	0.93297	0.93560
956	0.92104	0.92559	0.92769	0.92838	0.93062	0.93303	0.93566
957	0.92110	0.92565	0.92775	0.92845	0.93068	0.93308	0.93571
958	0.92117	0.92572	0.92781	0.92851	0.93074	0.93314	0.93577
959	0.92124	0.92579	0.92787	0.92857	0.93080	0.93320	0.93582
960	0.92131	0.92585	0.92793	0.92863	0.93086	0.93326	0.93588
961	0.92138	0.92592	0.92800	0.92869	0.93092	0.93331	0.93593
962	0.92145	0.92598	0.92806	0.92875	0.93098	0.93337	0.93599
963	0.92151	0.92604	0.92812	0.92881	0.93104	0.93343	0.93604
964	0.92159	0.92611	0.92818	0.92887	0.93110	0.93348	0.93609
965	0.92166	0.92617	0.92825	0.92893	0.93116	0.93354	0.93615
966	0.92172	0.92623	0.92831	0.92899	0.93121	0.93359	0.93620
967	0.92178	0.92630	0.92837	0.92905	0.93127	0.93365	0.93625
968	0.92185	0.92636	0.92843	0.92912	0.93133	0.93371	0.93631
969	0.92192	0.92643	0.92849	0.92917	0.93139	0.93376	0.93636
970	0.92199	0.92649	0.92855	0.92924	0.93145	0.93382	0.93642
971	0.92205	0.92655	0.92861	0.92930	0.93151	0.93387	0.93647
972	0.92212	0.92661	0.92867	0.92936	0.93156	0.93393	0.93652
973	0.92219	0.92667	0.92873	0.92942	0.93162	0.93398	0.93657
974	0.92226	0.92674	0.92880	0.92948	0.93168	0.93404	0.93663
975	0.92232	0.92680	0.92886	0.92954	0.93174	0.93410	0.93668
976	0.92238	0.92686	0.92892	0.92960	0.93179	0.93415	0.93673
977	0.92245	0.92692	0.92898	0.92966	0.93185	0.93421	0.93679
978	0.92252	0.92698	0.92904	0.92971	0.93191	0.93426	0.93684
979	0.92258	0.92705	0.92910	0.92977	0.93197	0.93432	0.93689
980	0.92265	0.92711	0.92916	0.92983	0.93202	0.93437	0.93694
981	0.92271	0.92717	0.92922	0.92989	0.93208	0.93443	0.93700
982	0.92278	0.92723	0.92928	0.92995	0.93214	0.93448	0.93705
983	0.92284	0.92729	0.92934	0.93001	0.93220	0.93454	0.93710
984	0.92291	0.92735	0.92940	0.93007	0.93225	0.93459	0.93715
985	0.92298	0.92742	0.92946	0.93012	0.93231	0.93465	0.93721
986	0.92305	0.92748	0.92952	0.93018	0.93236	0.93470	0.93726
987	0.92311	0.92754	0.92957	0.93024	0.93242	0.93476	0.93731
988	0.92317	0.92760	0.92963	0.93030	0.93248	0.93481	0.93736
989	0.92323	0.92766	0.92969	0.93036	0.93254	0.93486	0.93741
990	0.92331	0.92772	0.92975	0.93042	0.93259	0.93492	0.93746
991	0.92337	0.92778	0.92981	0.93048	0.93265	0.93497	0.93752

N	0.1%	0.5%	1%	1.25%	2.5%	5%	10%
992	0.92344	0.92784	0.92986	0.93053	0.93271	0.93503	0.93757
993	0.92350	0.92790	0.92992	0.93059	0.93276	0.93508	0.93762
994	0.92357	0.92795	0.92998	0.93065	0.93282	0.93513	0.93767
995	0.92363	0.92802	0.93004	0.93071	0.93287	0.93519	0.93772
996	0.92370	0.92808	0.93009	0.93077	0.93293	0.93524	0.93777
997	0.92376	0.92814	0.93015	0.93082	0.93298	0.93529	0.93782
998	0.92382	0.92820	0.93021	0.93088	0.93304	0.93535	0.93787
999	0.92388	0.92826	0.93027	0.93094	0.93309	0.93540	0.93792
1000	0.92395	0.92832	0.93033	0.93099	0.93315	0.93545	0.93797
