# Supplementary material for: A generalized outlier method with an automated test protocol applied to proficiency testing schemes: utilizing Monte Carlo simulation for generating critical values for the extended Grubbs statistic
Source: J AOAC Int. 2026 Apr 29;109(6):974–84. doi: 10.1093/jaoacint/qsag036 (PMC13732683; doi:10.1093/jaoacint/qsag036)
Supplement: Supplementary file 1 [file qsag036_supplementary_data.zip › aoac-25-0307-File019.pdf]

Extended Grubbs Statistics Tables for Multiple-7 Outliers

Table 1 – *GM7(7H0L or 0H7L)*. Critical values of upper significance levels for the Grubbs multiple-7 outlier test, applicable in situations where the seven highest or seven lowest observations are simultaneously identified as outliers, denoted as *GM7(7H0L)* and *GM7(0H7L)*, respectively.

N	0.1%	0.5%	1%	1.25%	2.5%	5%	10%
9	0.00000	0.00000	0.00000	0.00001	0.00002	0.00009	0.00037
10	0.00005	0.00024	0.00049	0.00062	0.00127	0.00267	0.00572
11	0.00061	0.00186	0.00304	0.00357	0.00590	0.00992	0.01706
12	0.00240	0.00569	0.00832	0.00942	0.01396	0.02095	0.03217
13	0.00580	0.01173	0.01606	0.01779	0.02460	0.03452	0.04942
14	0.01078	0.01970	0.02573	0.02810	0.03712	0.04966	0.06765
15	0.01728	0.02911	0.03685	0.03978	0.05082	0.06572	0.08636
16	0.02496	0.03977	0.04905	0.05251	0.06532	0.08220	0.10505
17	0.03358	0.05125	0.06193	0.06590	0.08029	0.09880	0.12344
18	0.04307	0.06327	0.07522	0.07959	0.09533	0.11531	0.14144
19	0.05311	0.07578	0.08881	0.09356	0.11042	0.13158	0.15892
20	0.06358	0.08836	0.10236	0.10741	0.12531	0.14755	0.17586
21	0.07459	0.10109	0.11598	0.12127	0.14006	0.16310	0.19222
22	0.08577	0.11385	0.12937	0.13498	0.15451	0.17834	0.20797
23	0.09682	0.12647	0.14257	0.14840	0.16867	0.19303	0.22315
24	0.10796	0.13894	0.15567	0.16157	0.18246	0.20723	0.23774
25	0.11905	0.15110	0.16847	0.17459	0.19585	0.22100	0.25178
26	0.13019	0.16328	0.18095	0.18726	0.20884	0.23428	0.26525
27	0.14115	0.17516	0.19320	0.19961	0.22147	0.24711	0.27822
28	0.15191	0.18677	0.20516	0.21163	0.23370	0.25955	0.29069
29	0.16237	0.19808	0.21677	0.22331	0.24560	0.27159	0.30267
30	0.17301	0.20908	0.22809	0.23473	0.25712	0.28317	0.31428
31	0.18340	0.22006	0.23918	0.24579	0.26830	0.29439	0.32542
32	0.19333	0.23073	0.24985	0.25651	0.27909	0.30522	0.33620
33	0.20334	0.24114	0.26028	0.26702	0.28963	0.31569	0.34652
34	0.21288	0.25117	0.27046	0.27712	0.29980	0.32587	0.35654
35	0.22257	0.26106	0.28036	0.28709	0.30967	0.33565	0.36617
36	0.23220	0.27080	0.28999	0.29674	0.31929	0.34516	0.37552
37	0.24133	0.27988	0.29937	0.30601	0.32861	0.35437	0.38456
38	0.25040	0.28897	0.30852	0.31520	0.33768	0.36333	0.39329
39	0.25927	0.29787	0.31733	0.32399	0.34644	0.37202	0.40172
40	0.26795	0.30649	0.32596	0.33264	0.35497	0.38044	0.40991
41	0.27624	0.31503	0.33438	0.34103	0.36326	0.38858	0.41787
42	0.28457	0.32309	0.34258	0.34917	0.37138	0.39647	0.42554
43	0.29266	0.33118	0.35052	0.35718	0.37926	0.40412	0.43296
44	0.30039	0.33895	0.35823	0.36483	0.38685	0.41162	0.44020
45	0.30819	0.34664	0.36581	0.37239	0.39428	0.41884	0.44724
46	0.31567	0.35409	0.37316	0.37973	0.40142	0.42590	0.45406
47	0.32276	0.36142	0.38041	0.38687	0.40855	0.43274	0.46070
48	0.33005	0.36850	0.38739	0.39389	0.41535	0.43939	0.46711
49	0.33694	0.37533	0.39420	0.40058	0.42202	0.44591	0.47338
50	0.34404	0.38216	0.40084	0.40727	0.42851	0.45227	0.47946
51	0.35067	0.38875	0.40743	0.41374	0.43486	0.45842	0.48539
52	0.35743	0.39523	0.41373	0.42008	0.44104	0.46444	0.49119
53	0.36387	0.40155	0.41986	0.42625	0.44709	0.47027	0.49682
54	0.37020	0.40767	0.42600	0.43222	0.45295	0.47601	0.50232
55	0.37637	0.41371	0.43188	0.43805	0.45872	0.48159	0.50764
56	0.38231	0.41956	0.43770	0.44387	0.46433	0.48700	0.51286
57	0.38835	0.42536	0.44337	0.44951	0.46983	0.49231	0.51796
58	0.39415	0.43099	0.44895	0.45506	0.47516	0.49750	0.52292
59	0.39984	0.43654	0.45430	0.46039	0.48047	0.50253	0.52778
60	0.40520	0.44195	0.45964	0.46570	0.48554	0.50751	0.53251
61	0.41083	0.44722	0.46489	0.47086	0.49055	0.51238	0.53715
62	0.41629	0.45240	0.46994	0.47589	0.49542	0.51713	0.54168
63	0.42152	0.45747	0.47492	0.48083	0.50025	0.52174	0.54611
64	0.42671	0.46237	0.47978	0.48567	0.50489	0.52624	0.55041
65	0.43161	0.46728	0.48454	0.49037	0.50952	0.53066	0.55462
66	0.43665	0.47205	0.48917	0.49501	0.51398	0.53496	0.55875
67	0.44160	0.47676	0.49375	0.49951	0.51838	0.53921	0.56280
68	0.44653	0.48130	0.49828	0.50400	0.52266	0.54336	0.56676
69	0.45110	0.48586	0.50270	0.50836	0.52690	0.54743	0.57065
70	0.45559	0.49028	0.50702	0.51265	0.53108	0.55141	0.57445
71	0.46021	0.49463	0.51131	0.51686	0.53514	0.55532	0.57818
72	0.46461	0.49885	0.51543	0.52095	0.53912	0.55913	0.58181
73	0.46895	0.50309	0.51950	0.52499	0.54303	0.56290	0.58540
74	0.47327	0.50723	0.52346	0.52892	0.54686	0.56656	0.58890

N	0.1%	0.5%	1%	1.25%	2.5%	5%	10%
75	0.47754	0.51126	0.52735	0.53282	0.55061	0.57019	0.59234
76	0.48159	0.51517	0.53122	0.53657	0.55428	0.57373	0.59569
77	0.48572	0.51906	0.53499	0.54032	0.55791	0.57718	0.59899
78	0.48968	0.52292	0.53870	0.54402	0.56146	0.58062	0.60224
79	0.49349	0.52660	0.54237	0.54760	0.56495	0.58396	0.60543
80	0.49746	0.53036	0.54593	0.55117	0.56836	0.58723	0.60854
81	0.50116	0.53394	0.54944	0.55464	0.57171	0.59047	0.61160
82	0.50498	0.53743	0.55289	0.55808	0.57501	0.59364	0.61462
83	0.50860	0.54096	0.55630	0.56147	0.57828	0.59677	0.61759
84	0.51221	0.54442	0.55963	0.56473	0.58146	0.59981	0.62048
85	0.51587	0.54777	0.56291	0.56797	0.58458	0.60282	0.62333
86	0.51937	0.55108	0.56615	0.57119	0.58767	0.60580	0.62616
87	0.52269	0.55433	0.56930	0.57434	0.59068	0.60870	0.62890
88	0.52618	0.55752	0.57240	0.57736	0.59370	0.61156	0.63160
89	0.52954	0.56072	0.57546	0.58039	0.59659	0.61433	0.63427
90	0.53277	0.56376	0.57844	0.58337	0.59946	0.61711	0.63689
91	0.53613	0.56688	0.58144	0.58631	0.60231	0.61983	0.63948
92	0.53929	0.56988	0.58435	0.58921	0.60510	0.62250	0.64199
93	0.54243	0.57283	0.58726	0.59207	0.60785	0.62516	0.64448
94	0.54547	0.57577	0.59008	0.59491	0.61058	0.62774	0.64693
95	0.54842	0.57868	0.59286	0.59767	0.61323	0.63027	0.64933
96	0.55138	0.58151	0.59562	0.60038	0.61583	0.63279	0.65171
97	0.55440	0.58428	0.59837	0.60306	0.61842	0.63525	0.65405
98	0.55728	0.58706	0.60103	0.60568	0.62093	0.63769	0.65637
99	0.56015	0.58974	0.60366	0.60830	0.62343	0.64009	0.65862
100	0.56301	0.59241	0.60623	0.61084	0.62589	0.64245	0.66087
101	0.56597	0.59506	0.60880	0.61338	0.62834	0.64477	0.66307
102	0.56871	0.59769	0.61132	0.61588	0.63074	0.64705	0.66522
103	0.57134	0.60025	0.61379	0.61830	0.63312	0.64931	0.66737
104	0.57415	0.60273	0.61620	0.62074	0.63545	0.65151	0.66948
105	0.57679	0.60528	0.61864	0.62310	0.63774	0.65371	0.67155
106	0.57926	0.60775	0.62100	0.62542	0.64001	0.65587	0.67359
107	0.58186	0.61013	0.62334	0.62774	0.64224	0.65800	0.67561
108	0.58453	0.61252	0.62568	0.63004	0.64442	0.66010	0.67759
109	0.58692	0.61484	0.62797	0.63231	0.64658	0.66219	0.67956
110	0.58946	0.61721	0.63021	0.63454	0.64869	0.66423	0.68149
111	0.59181	0.61949	0.63236	0.63671	0.65079	0.66623	0.68339
112	0.59423	0.62172	0.63452	0.63881	0.65289	0.66822	0.68527
113	0.59666	0.62397	0.63668	0.64099	0.65496	0.67020	0.68712
114	0.59890	0.62614	0.63880	0.64308	0.65698	0.67213	0.68895
115	0.60126	0.62828	0.64090	0.64518	0.65896	0.67402	0.69075
116	0.60361	0.63038	0.64299	0.64720	0.66094	0.67590	0.69255
117	0.60588	0.63253	0.64504	0.64923	0.66288	0.67775	0.69429
118	0.60807	0.63459	0.64702	0.65122	0.66479	0.67958	0.69603
119	0.61023	0.63664	0.64901	0.65319	0.66669	0.68138	0.69773
120	0.61252	0.63869	0.65100	0.65511	0.66854	0.68316	0.69941
121	0.61455	0.64074	0.65296	0.65701	0.67039	0.68493	0.70109
122	0.61677	0.64273	0.65484	0.65891	0.67219	0.68666	0.70275
123	0.61884	0.64470	0.65673	0.66081	0.67398	0.68836	0.70437
124	0.62086	0.64666	0.65861	0.66265	0.67575	0.69005	0.70598
125	0.62288	0.64851	0.66045	0.66446	0.67752	0.69175	0.70757
126	0.62500	0.65042	0.66229	0.66627	0.67925	0.69341	0.70915
127	0.62690	0.65228	0.66410	0.66806	0.68096	0.69506	0.71069
128	0.62897	0.65411	0.66588	0.66985	0.68266	0.69665	0.71221
129	0.63082	0.65593	0.66765	0.67158	0.68434	0.69825	0.71372
130	0.63284	0.65774	0.66938	0.67327	0.68597	0.69983	0.71521
131	0.63474	0.65953	0.67111	0.67499	0.68762	0.70138	0.71670
132	0.63668	0.66130	0.67279	0.67666	0.68922	0.70292	0.71815
133	0.63848	0.66301	0.67447	0.67831	0.69082	0.70446	0.71958
134	0.64041	0.66474	0.67614	0.67997	0.69240	0.70596	0.72101
135	0.64222	0.66643	0.67778	0.68158	0.69396	0.70746	0.72243
136	0.64400	0.66813	0.67938	0.68321	0.69552	0.70893	0.72381
137	0.64573	0.66984	0.68098	0.68478	0.69705	0.71039	0.72519
138	0.64749	0.67142	0.68258	0.68634	0.69855	0.71182	0.72654
139	0.64927	0.67305	0.68418	0.68792	0.70003	0.71324	0.72788
140	0.65106	0.67467	0.68573	0.68943	0.70152	0.71465	0.72921
141	0.65266	0.67622	0.68727	0.69098	0.70298	0.71604	0.73052
142	0.65446	0.67786	0.68881	0.69249	0.70442	0.71741	0.73182
143	0.65609	0.67940	0.69031	0.69398	0.70585	0.71879	0.73312
144	0.65772	0.68097	0.69179	0.69543	0.70724	0.72013	0.73438
145	0.65939	0.68247	0.69330	0.69690	0.70863	0.72147	0.73565
146	0.66105	0.68399	0.69474	0.69835	0.71000	0.72279	0.73690
147	0.66270	0.68548	0.69620	0.69977	0.71136	0.72407	0.73813
148	0.66429	0.68694	0.69761	0.70118	0.71272	0.72535	0.73934

N	0.1%	0.5%	1%	1.25%	2.5%	5%	10%
149	0.66587	0.68840	0.69901	0.70256	0.71405	0.72662	0.74054
150	0.66742	0.68986	0.70042	0.70393	0.71538	0.72789	0.74173
151	0.66890	0.69130	0.70178	0.70529	0.71669	0.72912	0.74291
152	0.67049	0.69274	0.70317	0.70666	0.71800	0.73036	0.74407
153	0.67200	0.69418	0.70455	0.70800	0.71927	0.73158	0.74523
154	0.67353	0.69554	0.70583	0.70930	0.72055	0.73280	0.74637
155	0.67495	0.69693	0.70718	0.71061	0.72183	0.73401	0.74750
156	0.67644	0.69823	0.70849	0.71193	0.72307	0.73518	0.74863
157	0.67788	0.69960	0.70978	0.71318	0.72432	0.73636	0.74973
158	0.67934	0.70094	0.71107	0.71448	0.72554	0.73752	0.75083
159	0.68078	0.70224	0.71233	0.71573	0.72674	0.73866	0.75192
160	0.68220	0.70356	0.71360	0.71697	0.72792	0.73981	0.75299
161	0.68354	0.70484	0.71483	0.71821	0.72911	0.74094	0.75405
162	0.68488	0.70609	0.71607	0.71942	0.73030	0.74205	0.75511
163	0.68631	0.70731	0.71731	0.72063	0.73144	0.74316	0.75616
164	0.68751	0.70858	0.71852	0.72184	0.73260	0.74426	0.75719
165	0.68894	0.70985	0.71972	0.72301	0.73374	0.74535	0.75822
166	0.69030	0.71113	0.72091	0.72418	0.73487	0.74644	0.75924
167	0.69154	0.71234	0.72208	0.72536	0.73599	0.74750	0.76025
168	0.69281	0.71353	0.72323	0.72649	0.73712	0.74855	0.76125
169	0.69410	0.71476	0.72441	0.72763	0.73820	0.74960	0.76224
170	0.69534	0.71597	0.72555	0.72876	0.73929	0.75063	0.76321
171	0.69662	0.71713	0.72666	0.72989	0.74034	0.75166	0.76418
172	0.69790	0.71828	0.72781	0.73100	0.74142	0.75267	0.76514
173	0.69910	0.71946	0.72891	0.73210	0.74248	0.75369	0.76609
174	0.70036	0.72063	0.73003	0.73321	0.74351	0.75468	0.76703
175	0.70157	0.72174	0.73110	0.73427	0.74454	0.75567	0.76796
176	0.70283	0.72285	0.73217	0.73534	0.74557	0.75664	0.76888
177	0.70394	0.72398	0.73324	0.73640	0.74659	0.75761	0.76980
178	0.70518	0.72508	0.73433	0.73748	0.74760	0.75857	0.77072
179	0.70638	0.72619	0.73539	0.73855	0.74860	0.75952	0.77162
180	0.70750	0.72730	0.73643	0.73957	0.74959	0.76046	0.77251
181	0.70864	0.72837	0.73747	0.74058	0.75059	0.76141	0.77338
182	0.70977	0.72943	0.73851	0.74159	0.75155	0.76232	0.77426
183	0.71085	0.73049	0.73952	0.74258	0.75253	0.76326	0.77513
184	0.71195	0.73155	0.74054	0.74359	0.75349	0.76417	0.77599
185	0.71311	0.73255	0.74153	0.74457	0.75446	0.76507	0.77684
186	0.71412	0.73359	0.74255	0.74556	0.75540	0.76596	0.77768
187	0.71522	0.73460	0.74353	0.74651	0.75633	0.76686	0.77853
188	0.71629	0.73561	0.74451	0.74749	0.75725	0.76774	0.77936
189	0.71732	0.73660	0.74546	0.74846	0.75818	0.76861	0.78019
190	0.71840	0.73755	0.74644	0.74941	0.75908	0.76947	0.78100
191	0.71952	0.73855	0.74737	0.75036	0.75998	0.77032	0.78181
192	0.72058	0.73952	0.74831	0.75129	0.76087	0.77117	0.78261
193	0.72162	0.74049	0.74927	0.75222	0.76174	0.77201	0.78341
194	0.72265	0.74146	0.75019	0.75313	0.76261	0.77285	0.78420
195	0.72366	0.74243	0.75111	0.75405	0.76349	0.77369	0.78499
196	0.72472	0.74339	0.75202	0.75493	0.76435	0.77450	0.78577
197	0.72573	0.74433	0.75291	0.75584	0.76520	0.77532	0.78653
198	0.72672	0.74527	0.75382	0.75673	0.76606	0.77612	0.78730
199	0.72773	0.74616	0.75471	0.75760	0.76689	0.77694	0.78806
200	0.72866	0.74712	0.75561	0.75846	0.76773	0.77774	0.78880
201	0.72965	0.74802	0.75649	0.75932	0.76856	0.77853	0.78955
202	0.73064	0.74893	0.75736	0.76018	0.76939	0.77931	0.79029
203	0.73160	0.74981	0.75822	0.76103	0.77020	0.78010	0.79102
204	0.73258	0.75070	0.75907	0.76186	0.77100	0.78086	0.79175
205	0.73352	0.75157	0.75991	0.76270	0.77180	0.78163	0.79248
206	0.73445	0.75240	0.76078	0.76355	0.77260	0.78239	0.79320
207	0.73538	0.75328	0.76159	0.76438	0.77338	0.78314	0.79392
208	0.73636	0.75414	0.76244	0.76519	0.77417	0.78389	0.79462
209	0.73729	0.75499	0.76326	0.76599	0.77496	0.78464	0.79532
210	0.73824	0.75584	0.76407	0.76679	0.77573	0.78538	0.79602
211	0.73910	0.75671	0.76487	0.76761	0.77650	0.78611	0.79670
212	0.73997	0.75754	0.76568	0.76840	0.77725	0.78683	0.79739
213	0.74084	0.75833	0.76649	0.76919	0.77799	0.78755	0.79807
214	0.74171	0.75916	0.76730	0.76997	0.77875	0.78826	0.79874
215	0.74261	0.75998	0.76808	0.77075	0.77949	0.78897	0.79941
216	0.74345	0.76078	0.76887	0.77154	0.78024	0.78968	0.80008
217	0.74432	0.76158	0.76963	0.77230	0.78097	0.79038	0.80074
218	0.74518	0.76238	0.77041	0.77306	0.78170	0.79108	0.80140
219	0.74609	0.76319	0.77117	0.77383	0.78243	0.79176	0.80204
220	0.74694	0.76395	0.77192	0.77457	0.78315	0.79244	0.80269
221	0.74778	0.76475	0.77265	0.77531	0.78384	0.79313	0.80333
222	0.74860	0.76552	0.77340	0.77605	0.78456	0.79381	0.80397

N	0.1%	0.5%	1%	1.25%	2.5%	5%	10%
223	0.74938	0.76627	0.77415	0.77677	0.78527	0.79447	0.80460
224	0.75024	0.76703	0.77488	0.77749	0.78596	0.79514	0.80522
225	0.75105	0.76775	0.77562	0.77822	0.78665	0.79579	0.80584
226	0.75186	0.76852	0.77634	0.77895	0.78735	0.79645	0.80646
227	0.75262	0.76924	0.77704	0.77965	0.78803	0.79709	0.80707
228	0.75340	0.76996	0.77775	0.78036	0.78869	0.79773	0.80768
229	0.75420	0.77073	0.77845	0.78105	0.78936	0.79838	0.80829
230	0.75500	0.77147	0.77918	0.78174	0.79004	0.79901	0.80889
231	0.75575	0.77221	0.77987	0.78243	0.79070	0.79964	0.80949
232	0.75655	0.77294	0.78056	0.78310	0.79137	0.80027	0.81008
233	0.75728	0.77365	0.78126	0.78380	0.79202	0.80089	0.81067
234	0.75805	0.77434	0.78195	0.78447	0.79266	0.80151	0.81125
235	0.75881	0.77506	0.78261	0.78514	0.79331	0.80212	0.81182
236	0.75956	0.77577	0.78328	0.78581	0.79394	0.80274	0.81239
237	0.76031	0.77648	0.78396	0.78647	0.79457	0.80333	0.81297
238	0.76105	0.77719	0.78465	0.78713	0.79521	0.80393	0.81353
239	0.76177	0.77788	0.78531	0.78779	0.79583	0.80454	0.81410
240	0.76249	0.77853	0.78596	0.78843	0.79646	0.80512	0.81467
241	0.76324	0.77923	0.78660	0.78908	0.79708	0.80571	0.81523
242	0.76397	0.77989	0.78725	0.78971	0.79769	0.80630	0.81577
243	0.76469	0.78056	0.78790	0.79036	0.79830	0.80688	0.81632
244	0.76537	0.78121	0.78855	0.79100	0.79891	0.80746	0.81686
245	0.76605	0.78188	0.78920	0.79161	0.79950	0.80803	0.81741
246	0.76680	0.78254	0.78982	0.79224	0.80009	0.80860	0.81794
247	0.76754	0.78318	0.79045	0.79286	0.80068	0.80916	0.81848
248	0.76825	0.78381	0.79106	0.79347	0.80128	0.80973	0.81901
249	0.76895	0.78448	0.79170	0.79409	0.80186	0.81028	0.81954
250	0.76965	0.78510	0.79230	0.79470	0.80244	0.81083	0.82006
251	0.77031	0.78573	0.79289	0.79529	0.80302	0.81139	0.82058
252	0.77098	0.78634	0.79350	0.79588	0.80360	0.81193	0.82110
253	0.77164	0.78696	0.79411	0.79648	0.80416	0.81248	0.82161
254	0.77229	0.78759	0.79470	0.79707	0.80473	0.81302	0.82212
255	0.77297	0.78820	0.79531	0.79766	0.80530	0.81356	0.82263
256	0.77366	0.78882	0.79590	0.79826	0.80586	0.81409	0.82313
257	0.77433	0.78941	0.79648	0.79884	0.80641	0.81462	0.82363
258	0.77493	0.79002	0.79705	0.79942	0.80697	0.81514	0.82413
259	0.77557	0.79062	0.79764	0.79997	0.80752	0.81566	0.82463
260	0.77621	0.79123	0.79821	0.80054	0.80805	0.81618	0.82512
261	0.77684	0.79184	0.79879	0.80111	0.80860	0.81670	0.82561
262	0.77742	0.79243	0.79935	0.80167	0.80913	0.81721	0.82609
263	0.77810	0.79301	0.79991	0.80222	0.80967	0.81771	0.82657
264	0.77873	0.79360	0.80046	0.80278	0.81020	0.81822	0.82705
265	0.77938	0.79415	0.80102	0.80332	0.81073	0.81873	0.82752
266	0.78001	0.79474	0.80157	0.80387	0.81125	0.81922	0.82799
267	0.78065	0.79531	0.80212	0.80441	0.81178	0.81973	0.82847
268	0.78126	0.79587	0.80267	0.80495	0.81230	0.82022	0.82894
269	0.78189	0.79643	0.80322	0.80549	0.81281	0.82071	0.82940
270	0.78247	0.79702	0.80377	0.80602	0.81333	0.82120	0.82986
271	0.78306	0.79757	0.80429	0.80654	0.81383	0.82168	0.83032
272	0.78370	0.79813	0.80483	0.80707	0.81435	0.82217	0.83077
273	0.78427	0.79868	0.80535	0.80760	0.81485	0.82264	0.83123
274	0.78487	0.79923	0.80589	0.80812	0.81535	0.82312	0.83167
275	0.78547	0.79978	0.80641	0.80864	0.81584	0.82359	0.83212
276	0.78607	0.80033	0.80694	0.80916	0.81634	0.82406	0.83257
277	0.78661	0.80089	0.80746	0.80966	0.81683	0.82453	0.83301
278	0.78725	0.80142	0.80795	0.81017	0.81732	0.82499	0.83344
279	0.78785	0.80195	0.80848	0.81067	0.81781	0.82546	0.83388
280	0.78841	0.80248	0.80899	0.81117	0.81829	0.82592	0.83432
281	0.78893	0.80301	0.80951	0.81167	0.81876	0.82637	0.83475
282	0.78947	0.80354	0.81000	0.81217	0.81923	0.82682	0.83518
283	0.79003	0.80406	0.81051	0.81267	0.81970	0.82727	0.83560
284	0.79058	0.80457	0.81100	0.81316	0.82017	0.82772	0.83603
285	0.79117	0.80507	0.81150	0.81365	0.82064	0.82817	0.83645
286	0.79174	0.80559	0.81201	0.81412	0.82111	0.82861	0.83687
287	0.79227	0.80611	0.81251	0.81461	0.82158	0.82906	0.83728
288	0.79283	0.80661	0.81298	0.81510	0.82203	0.82949	0.83770
289	0.79338	0.80712	0.81347	0.81558	0.82250	0.82993	0.83811
290	0.79391	0.80762	0.81395	0.81606	0.82295	0.83037	0.83852
291	0.79444	0.80812	0.81442	0.81653	0.82340	0.83080	0.83893
292	0.79495	0.80861	0.81491	0.81699	0.82385	0.83123	0.83933
293	0.79551	0.80909	0.81538	0.81747	0.82431	0.83166	0.83974
294	0.79597	0.80957	0.81585	0.81792	0.82475	0.83208	0.84014
295	0.79652	0.81005	0.81632	0.81840	0.82520	0.83250	0.84054
296	0.79704	0.81055	0.81678	0.81886	0.82564	0.83292	0.84094

N	0.1%	0.5%	1%	1.25%	2.5%	5%	10%
297	0.79757	0.81102	0.81724	0.81930	0.82607	0.83334	0.84133
298	0.79812	0.81148	0.81769	0.81976	0.82650	0.83375	0.84172
299	0.79862	0.81196	0.81815	0.82022	0.82693	0.83416	0.84212
300	0.79916	0.81243	0.81860	0.82066	0.82736	0.83457	0.84250
301	0.79961	0.81290	0.81905	0.82109	0.82778	0.83498	0.84288
302	0.80009	0.81337	0.81951	0.82154	0.82821	0.83538	0.84327
303	0.80062	0.81383	0.81995	0.82199	0.82863	0.83578	0.84365
304	0.80111	0.81429	0.82040	0.82243	0.82905	0.83618	0.84402
305	0.80163	0.81477	0.82084	0.82287	0.82947	0.83658	0.84440
306	0.80207	0.81522	0.82129	0.82330	0.82989	0.83697	0.84477
307	0.80260	0.81568	0.82172	0.82373	0.83030	0.83736	0.84515
308	0.80307	0.81613	0.82215	0.82415	0.83072	0.83776	0.84552
309	0.80356	0.81657	0.82258	0.82458	0.83113	0.83815	0.84589
310	0.80405	0.81701	0.82302	0.82501	0.83153	0.83854	0.84626
311	0.80452	0.81745	0.82345	0.82544	0.83193	0.83893	0.84663
312	0.80500	0.81790	0.82387	0.82586	0.83233	0.83932	0.84699
313	0.80550	0.81833	0.82428	0.82628	0.83272	0.83970	0.84735
314	0.80597	0.81876	0.82470	0.82670	0.83313	0.84008	0.84771
315	0.80643	0.81922	0.82513	0.82711	0.83353	0.84046	0.84807
316	0.80688	0.81965	0.82555	0.82751	0.83393	0.84084	0.84843
317	0.80730	0.82007	0.82596	0.82792	0.83431	0.84121	0.84878
318	0.80777	0.82049	0.82637	0.82832	0.83471	0.84158	0.84913
319	0.80824	0.82091	0.82678	0.82873	0.83510	0.84195	0.84948
320	0.80872	0.82134	0.82718	0.82913	0.83548	0.84232	0.84983
321	0.80917	0.82177	0.82759	0.82953	0.83587	0.84269	0.85018
322	0.80964	0.82220	0.82798	0.82994	0.83625	0.84305	0.85052
323	0.81009	0.82261	0.82839	0.83034	0.83663	0.84342	0.85087
324	0.81054	0.82302	0.82878	0.83073	0.83701	0.84377	0.85121
325	0.81096	0.82343	0.82919	0.83112	0.83740	0.84414	0.85154
326	0.81144	0.82383	0.82957	0.83151	0.83776	0.84450	0.85188
327	0.81187	0.82426	0.82997	0.83189	0.83813	0.84485	0.85222
328	0.81232	0.82466	0.83037	0.83229	0.83850	0.84520	0.85255
329	0.81276	0.82507	0.83076	0.83268	0.83887	0.84556	0.85288
330	0.81318	0.82546	0.83114	0.83305	0.83924	0.84590	0.85322
331	0.81364	0.82584	0.83153	0.83343	0.83960	0.84625	0.85355
332	0.81407	0.82623	0.83191	0.83381	0.83996	0.84659	0.85388
333	0.81447	0.82663	0.83230	0.83419	0.84032	0.84694	0.85420
334	0.81491	0.82703	0.83267	0.83457	0.84068	0.84728	0.85452
335	0.81532	0.82741	0.83305	0.83494	0.84104	0.84762	0.85485
336	0.81570	0.82781	0.83342	0.83531	0.84139	0.84795	0.85517
337	0.81613	0.82820	0.83381	0.83568	0.84175	0.84829	0.85548
338	0.81655	0.82858	0.83417	0.83605	0.84211	0.84863	0.85580
339	0.81695	0.82898	0.83455	0.83641	0.84246	0.84896	0.85612
340	0.81739	0.82937	0.83492	0.83678	0.84281	0.84930	0.85643
341	0.81775	0.82975	0.83528	0.83714	0.84316	0.84963	0.85675
342	0.81818	0.83013	0.83565	0.83750	0.84351	0.84996	0.85706
343	0.81855	0.83050	0.83601	0.83787	0.84385	0.85028	0.85737
344	0.81895	0.83087	0.83637	0.83823	0.84418	0.85060	0.85767
345	0.81939	0.83126	0.83674	0.83858	0.84453	0.85093	0.85798
346	0.81980	0.83163	0.83710	0.83894	0.84487	0.85126	0.85829
347	0.82019	0.83200	0.83745	0.83929	0.84521	0.85158	0.85859
348	0.82058	0.83238	0.83781	0.83963	0.84554	0.85189	0.85889
349	0.82095	0.83274	0.83815	0.83999	0.84587	0.85221	0.85919
350	0.82135	0.83312	0.83851	0.84034	0.84620	0.85253	0.85949
351	0.82175	0.83349	0.83886	0.84069	0.84654	0.85284	0.85979
352	0.82212	0.83385	0.83920	0.84103	0.84687	0.85316	0.86009
353	0.82250	0.83420	0.83955	0.84137	0.84721	0.85347	0.86039
354	0.82290	0.83456	0.83990	0.84171	0.84753	0.85378	0.86068
355	0.82326	0.83490	0.84025	0.84205	0.84785	0.85408	0.86098
356	0.82365	0.83524	0.84059	0.84239	0.84818	0.85440	0.86127
357	0.82404	0.83559	0.84093	0.84272	0.84850	0.85470	0.86156
358	0.82443	0.83593	0.84127	0.84304	0.84882	0.85501	0.86184
359	0.82481	0.83628	0.84160	0.84338	0.84914	0.85532	0.86213
360	0.82516	0.83663	0.84194	0.84371	0.84945	0.85562	0.86242
361	0.82555	0.83697	0.84227	0.84404	0.84977	0.85592	0.86270
362	0.82594	0.83732	0.84260	0.84438	0.85008	0.85622	0.86299
363	0.82631	0.83766	0.84292	0.84469	0.85039	0.85652	0.86327
364	0.82666	0.83800	0.84325	0.84501	0.85070	0.85681	0.86355
365	0.82702	0.83832	0.84358	0.84533	0.85101	0.85710	0.86383
366	0.82738	0.83866	0.84391	0.84566	0.85132	0.85740	0.86411
367	0.82777	0.83899	0.84423	0.84598	0.85163	0.85769	0.86438
368	0.82814	0.83932	0.84455	0.84630	0.85193	0.85799	0.86466
369	0.82849	0.83966	0.84487	0.84662	0.85224	0.85828	0.86493
370	0.82882	0.83998	0.84519	0.84694	0.85254	0.85856	0.86521

N	0.1%	0.5%	1%	1.25%	2.5%	5%	10%
371	0.82916	0.84031	0.84550	0.84725	0.85284	0.85885	0.86548
372	0.82953	0.84065	0.84583	0.84756	0.85315	0.85914	0.86575
373	0.82988	0.84100	0.84614	0.84788	0.85344	0.85943	0.86602
374	0.83022	0.84133	0.84646	0.84819	0.85374	0.85971	0.86629
375	0.83057	0.84164	0.84678	0.84850	0.85403	0.86000	0.86656
376	0.83093	0.84196	0.84709	0.84881	0.85433	0.86028	0.86682
377	0.83127	0.84228	0.84741	0.84912	0.85462	0.86056	0.86709
378	0.83159	0.84261	0.84771	0.84942	0.85491	0.86083	0.86735
379	0.83192	0.84293	0.84802	0.84971	0.85520	0.86111	0.86761
380	0.83225	0.84324	0.84832	0.85002	0.85549	0.86139	0.86787
381	0.83259	0.84356	0.84863	0.85033	0.85578	0.86166	0.86813
382	0.83295	0.84388	0.84894	0.85062	0.85606	0.86193	0.86839
383	0.83326	0.84419	0.84924	0.85092	0.85635	0.86221	0.86865
384	0.83359	0.84450	0.84954	0.85122	0.85663	0.86248	0.86890
385	0.83392	0.84481	0.84984	0.85150	0.85692	0.86275	0.86916
386	0.83428	0.84513	0.85014	0.85180	0.85720	0.86302	0.86942
387	0.83461	0.84543	0.85044	0.85210	0.85748	0.86328	0.86967
388	0.83493	0.84573	0.85073	0.85239	0.85776	0.86354	0.86992
389	0.83529	0.84604	0.85102	0.85267	0.85803	0.86381	0.87017
390	0.83561	0.84635	0.85130	0.85296	0.85831	0.86408	0.87042
391	0.83596	0.84664	0.85160	0.85324	0.85859	0.86434	0.87067
392	0.83630	0.84695	0.85189	0.85353	0.85886	0.86460	0.87092
393	0.83661	0.84725	0.85218	0.85381	0.85913	0.86487	0.87117
394	0.83691	0.84755	0.85247	0.85409	0.85940	0.86512	0.87142
395	0.83727	0.84784	0.85275	0.85437	0.85967	0.86538	0.87166
396	0.83760	0.84814	0.85304	0.85465	0.85995	0.86564	0.87190
397	0.83790	0.84844	0.85332	0.85493	0.86021	0.86590	0.87215
398	0.83821	0.84874	0.85360	0.85521	0.86048	0.86616	0.87239
399	0.83851	0.84903	0.85388	0.85549	0.86074	0.86641	0.87263
400	0.83883	0.84932	0.85416	0.85577	0.86101	0.86667	0.87287
401	0.83913	0.84961	0.85444	0.85605	0.86127	0.86691	0.87311
402	0.83943	0.84990	0.85472	0.85632	0.86154	0.86716	0.87334
403	0.83974	0.85018	0.85500	0.85660	0.86180	0.86741	0.87358
404	0.84004	0.85047	0.85528	0.85688	0.86207	0.86766	0.87382
405	0.84038	0.85075	0.85555	0.85715	0.86233	0.86791	0.87405
406	0.84068	0.85103	0.85583	0.85742	0.86259	0.86816	0.87429
407	0.84100	0.85131	0.85611	0.85769	0.86285	0.86841	0.87452
408	0.84130	0.85158	0.85638	0.85795	0.86311	0.86865	0.87475
409	0.84159	0.85186	0.85665	0.85822	0.86336	0.86889	0.87498
410	0.84189	0.85215	0.85691	0.85849	0.86361	0.86914	0.87521
411	0.84219	0.85242	0.85717	0.85875	0.86387	0.86938	0.87544
412	0.84246	0.85270	0.85745	0.85902	0.86411	0.86962	0.87567
413	0.84277	0.85298	0.85771	0.85928	0.86437	0.86986	0.87589
414	0.84306	0.85325	0.85798	0.85954	0.86462	0.87010	0.87612
415	0.84335	0.85352	0.85824	0.85981	0.86487	0.87034	0.87635
416	0.84364	0.85380	0.85850	0.86006	0.86511	0.87057	0.87657
417	0.84391	0.85406	0.85876	0.86032	0.86536	0.87081	0.87680
418	0.84420	0.85433	0.85902	0.86058	0.86561	0.87105	0.87702
419	0.84448	0.85461	0.85928	0.86083	0.86586	0.87128	0.87724
420	0.84477	0.85488	0.85954	0.86108	0.86610	0.87151	0.87746
421	0.84506	0.85514	0.85981	0.86134	0.86634	0.87174	0.87768
422	0.84533	0.85541	0.86005	0.86159	0.86659	0.87198	0.87790
423	0.84562	0.85567	0.86030	0.86183	0.86683	0.87221	0.87812
424	0.84589	0.85593	0.86056	0.86209	0.86706	0.87244	0.87834
425	0.84618	0.85619	0.86081	0.86233	0.86731	0.87266	0.87856
426	0.84649	0.85646	0.86107	0.86258	0.86755	0.87289	0.87877
427	0.84674	0.85671	0.86131	0.86283	0.86778	0.87312	0.87898
428	0.84702	0.85697	0.86156	0.86308	0.86802	0.87335	0.87920
429	0.84730	0.85723	0.86181	0.86332	0.86826	0.87357	0.87941
430	0.84757	0.85749	0.86206	0.86357	0.86849	0.87379	0.87962
431	0.84785	0.85773	0.86231	0.86381	0.86872	0.87402	0.87983
432	0.84813	0.85800	0.86255	0.86406	0.86896	0.87423	0.88004
433	0.84840	0.85826	0.86281	0.86430	0.86919	0.87445	0.88025
434	0.84867	0.85851	0.86304	0.86454	0.86942	0.87467	0.88046
435	0.84893	0.85876	0.86329	0.86479	0.86965	0.87489	0.88067
436	0.84919	0.85900	0.86352	0.86503	0.86988	0.87511	0.88088
437	0.84948	0.85925	0.86376	0.86527	0.87010	0.87533	0.88108
438	0.84973	0.85951	0.86399	0.86550	0.87033	0.87555	0.88129
439	0.84999	0.85974	0.86424	0.86574	0.87056	0.87576	0.88149
440	0.85027	0.85999	0.86447	0.86597	0.87079	0.87598	0.88170
441	0.85053	0.86022	0.86471	0.86620	0.87101	0.87619	0.88190
442	0.85079	0.86047	0.86495	0.86643	0.87124	0.87641	0.88210
443	0.85105	0.86071	0.86519	0.86667	0.87146	0.87662	0.88230
444	0.85134	0.86097	0.86542	0.86689	0.87168	0.87683	0.88250

N	0.1%	0.5%	1%	1.25%	2.5%	5%	10%
445	0.85161	0.86120	0.86566	0.86712	0.87190	0.87704	0.88271
446	0.85186	0.86144	0.86589	0.86736	0.87213	0.87725	0.88291
447	0.85213	0.86169	0.86612	0.86758	0.87235	0.87747	0.88311
448	0.85239	0.86193	0.86635	0.86781	0.87256	0.87767	0.88330
449	0.85264	0.86217	0.86658	0.86803	0.87278	0.87788	0.88350
450	0.85291	0.86241	0.86680	0.86826	0.87300	0.87808	0.88369
451	0.85317	0.86265	0.86702	0.86849	0.87321	0.87829	0.88389
452	0.85341	0.86288	0.86726	0.86872	0.87343	0.87850	0.88409
453	0.85369	0.86311	0.86748	0.86894	0.87365	0.87870	0.88428
454	0.85393	0.86335	0.86770	0.86916	0.87386	0.87891	0.88447
455	0.85422	0.86357	0.86794	0.86938	0.87407	0.87911	0.88466
456	0.85443	0.86382	0.86816	0.86960	0.87428	0.87932	0.88486
457	0.85468	0.86405	0.86838	0.86983	0.87450	0.87952	0.88505
458	0.85491	0.86429	0.86861	0.87005	0.87471	0.87972	0.88524
459	0.85517	0.86452	0.86883	0.87027	0.87491	0.87992	0.88543
460	0.85541	0.86475	0.86904	0.87049	0.87512	0.88012	0.88562
461	0.85565	0.86498	0.86926	0.87070	0.87533	0.88032	0.88581
462	0.85591	0.86520	0.86948	0.87092	0.87554	0.88052	0.88599
463	0.85616	0.86543	0.86970	0.87114	0.87574	0.88071	0.88618
464	0.85639	0.86565	0.86991	0.87136	0.87595	0.88091	0.88637
465	0.85663	0.86588	0.87014	0.87157	0.87616	0.88111	0.88656
466	0.85686	0.86610	0.87034	0.87177	0.87636	0.88130	0.88674
467	0.85709	0.86632	0.87056	0.87199	0.87657	0.88149	0.88693
468	0.85733	0.86655	0.87078	0.87220	0.87677	0.88169	0.88711
469	0.85759	0.86676	0.87100	0.87242	0.87698	0.88188	0.88730
470	0.85781	0.86698	0.87121	0.87263	0.87718	0.88207	0.88748
471	0.85806	0.86720	0.87142	0.87285	0.87738	0.88226	0.88766
472	0.85831	0.86742	0.87164	0.87305	0.87758	0.88245	0.88784
473	0.85853	0.86765	0.87185	0.87325	0.87778	0.88265	0.88802
474	0.85877	0.86786	0.87206	0.87346	0.87798	0.88284	0.88820
475	0.85898	0.86808	0.87227	0.87367	0.87817	0.88303	0.88838
476	0.85923	0.86829	0.87248	0.87388	0.87838	0.88321	0.88856
477	0.85946	0.86851	0.87269	0.87408	0.87857	0.88340	0.88874
478	0.85969	0.86873	0.87289	0.87429	0.87877	0.88359	0.88892
479	0.85991	0.86894	0.87310	0.87449	0.87896	0.88378	0.88909
480	0.86013	0.86916	0.87330	0.87469	0.87916	0.88396	0.88927
481	0.86034	0.86937	0.87351	0.87490	0.87935	0.88415	0.88945
482	0.86058	0.86958	0.87372	0.87510	0.87954	0.88433	0.88962
483	0.86080	0.86979	0.87392	0.87531	0.87973	0.88452	0.88979
484	0.86101	0.86999	0.87412	0.87550	0.87992	0.88470	0.88997
485	0.86124	0.87021	0.87433	0.87570	0.88012	0.88488	0.89014
486	0.86149	0.87041	0.87453	0.87590	0.88030	0.88506	0.89031
487	0.86172	0.87062	0.87472	0.87609	0.88049	0.88525	0.89048
488	0.86196	0.87082	0.87492	0.87629	0.88069	0.88543	0.89065
489	0.86217	0.87103	0.87511	0.87648	0.88088	0.88561	0.89082
490	0.86239	0.87124	0.87531	0.87667	0.88106	0.88579	0.89099
491	0.86258	0.87143	0.87551	0.87687	0.88125	0.88597	0.89116
492	0.86282	0.87163	0.87570	0.87706	0.88144	0.88615	0.89133
493	0.86306	0.87184	0.87590	0.87725	0.88163	0.88632	0.89150
494	0.86328	0.87204	0.87610	0.87744	0.88181	0.88650	0.89167
495	0.86348	0.87225	0.87629	0.87764	0.88200	0.88667	0.89183
496	0.86370	0.87246	0.87648	0.87783	0.88219	0.88685	0.89200
497	0.86391	0.87266	0.87668	0.87802	0.88237	0.88703	0.89217
498	0.86412	0.87286	0.87687	0.87821	0.88255	0.88720	0.89233
499	0.86435	0.87306	0.87707	0.87839	0.88273	0.88737	0.89249
500	0.86454	0.87326	0.87726	0.87859	0.88292	0.88755	0.89266
501	0.86477	0.87346	0.87745	0.87877	0.88310	0.88772	0.89282
502	0.86496	0.87365	0.87764	0.87896	0.88328	0.88790	0.89299
503	0.86518	0.87385	0.87783	0.87915	0.88346	0.88807	0.89315
504	0.86540	0.87405	0.87801	0.87934	0.88364	0.88824	0.89331
505	0.86562	0.87424	0.87821	0.87953	0.88382	0.88840	0.89347
506	0.86582	0.87445	0.87840	0.87971	0.88400	0.88857	0.89363
507	0.86605	0.87464	0.87859	0.87990	0.88417	0.88875	0.89379
508	0.86626	0.87483	0.87878	0.88008	0.88435	0.88892	0.89395
509	0.86647	0.87501	0.87896	0.88027	0.88453	0.88908	0.89411
510	0.86667	0.87521	0.87914	0.88045	0.88470	0.88925	0.89427
511	0.86686	0.87540	0.87933	0.88063	0.88488	0.88942	0.89443
512	0.86708	0.87560	0.87952	0.88082	0.88506	0.88958	0.89459
513	0.86730	0.87579	0.87970	0.88100	0.88523	0.88975	0.89474
514	0.86750	0.87597	0.87988	0.88118	0.88540	0.88991	0.89490
515	0.86769	0.87616	0.88006	0.88136	0.88557	0.89008	0.89506
516	0.86790	0.87635	0.88024	0.88154	0.88575	0.89024	0.89521
517	0.86809	0.87654	0.88042	0.88173	0.88591	0.89041	0.89537
518	0.86831	0.87673	0.88060	0.88190	0.88609	0.89057	0.89552

N	0.1%	0.5%	1%	1.25%	2.5%	5%	10%
519	0.86849	0.87692	0.88078	0.88208	0.88625	0.89073	0.89568
520	0.86870	0.87710	0.88096	0.88226	0.88643	0.89090	0.89583
521	0.86890	0.87729	0.88114	0.88243	0.88659	0.89106	0.89598
522	0.86911	0.87747	0.88132	0.88261	0.88676	0.89122	0.89614
523	0.86932	0.87766	0.88150	0.88279	0.88693	0.89138	0.89629
524	0.86952	0.87785	0.88168	0.88296	0.88709	0.89154	0.89644
525	0.86973	0.87803	0.88185	0.88314	0.88726	0.89170	0.89659
526	0.86993	0.87821	0.88202	0.88331	0.88743	0.89186	0.89675
527	0.87011	0.87839	0.88219	0.88348	0.88759	0.89202	0.89690
528	0.87030	0.87857	0.88236	0.88365	0.88776	0.89218	0.89705
529	0.87049	0.87875	0.88254	0.88381	0.88792	0.89234	0.89719
530	0.87070	0.87892	0.88271	0.88398	0.88809	0.89249	0.89734
531	0.87088	0.87910	0.88289	0.88416	0.88826	0.89265	0.89749
532	0.87108	0.87928	0.88307	0.88433	0.88842	0.89280	0.89764
533	0.87127	0.87946	0.88323	0.88450	0.88858	0.89296	0.89779
534	0.87147	0.87963	0.88341	0.88467	0.88874	0.89312	0.89793
535	0.87166	0.87981	0.88358	0.88483	0.88890	0.89327	0.89808
536	0.87184	0.87998	0.88374	0.88501	0.88907	0.89343	0.89823
537	0.87205	0.88016	0.88392	0.88517	0.88923	0.89358	0.89837
538	0.87222	0.88034	0.88409	0.88534	0.88939	0.89373	0.89852
539	0.87242	0.88051	0.88426	0.88550	0.88955	0.89388	0.89866
540	0.87260	0.88068	0.88442	0.88566	0.88971	0.89404	0.89881
541	0.87278	0.88084	0.88460	0.88583	0.88987	0.89419	0.89895
542	0.87295	0.88103	0.88476	0.88600	0.89003	0.89434	0.89909
543	0.87314	0.88120	0.88492	0.88616	0.89019	0.89449	0.89923
544	0.87332	0.88137	0.88509	0.88633	0.89034	0.89464	0.89938
545	0.87350	0.88155	0.88525	0.88649	0.89050	0.89479	0.89952
546	0.87369	0.88172	0.88542	0.88665	0.89065	0.89494	0.89966
547	0.87387	0.88189	0.88558	0.88681	0.89081	0.89509	0.89980
548	0.87406	0.88206	0.88574	0.88697	0.89096	0.89524	0.89994
549	0.87424	0.88222	0.88590	0.88713	0.89112	0.89538	0.90008
550	0.87441	0.88239	0.88607	0.88729	0.89127	0.89553	0.90022
551	0.87460	0.88256	0.88623	0.88745	0.89142	0.89568	0.90036
552	0.87478	0.88273	0.88639	0.88761	0.89158	0.89582	0.90050
553	0.87497	0.88290	0.88655	0.88778	0.89173	0.89597	0.90064
554	0.87515	0.88307	0.88671	0.88794	0.89188	0.89611	0.90078
555	0.87534	0.88324	0.88687	0.88810	0.89203	0.89626	0.90091
556	0.87550	0.88341	0.88703	0.88825	0.89218	0.89640	0.90105
557	0.87569	0.88357	0.88719	0.88841	0.89233	0.89655	0.90119
558	0.87588	0.88373	0.88735	0.88857	0.89248	0.89669	0.90132
559	0.87608	0.88389	0.88751	0.88872	0.89263	0.89684	0.90146
560	0.87626	0.88405	0.88767	0.88888	0.89278	0.89698	0.90160
561	0.87645	0.88422	0.88783	0.88903	0.89293	0.89712	0.90173
562	0.87661	0.88438	0.88799	0.88919	0.89308	0.89726	0.90186
563	0.87678	0.88455	0.88814	0.88934	0.89323	0.89740	0.90200
564	0.87694	0.88471	0.88830	0.88950	0.89338	0.89754	0.90213
565	0.87712	0.88487	0.88846	0.88964	0.89352	0.89769	0.90227
566	0.87729	0.88504	0.88861	0.88981	0.89367	0.89783	0.90240
567	0.87748	0.88521	0.88877	0.88996	0.89381	0.89797	0.90253
568	0.87765	0.88537	0.88892	0.89011	0.89396	0.89811	0.90266
569	0.87780	0.88552	0.88907	0.89026	0.89410	0.89824	0.90280
570	0.87797	0.88569	0.88922	0.89041	0.89425	0.89838	0.90293
571	0.87813	0.88585	0.88938	0.89056	0.89439	0.89852	0.90306
572	0.87830	0.88600	0.88953	0.89072	0.89454	0.89866	0.90319
573	0.87845	0.88615	0.88968	0.89087	0.89468	0.89880	0.90332
574	0.87862	0.88631	0.88983	0.89101	0.89482	0.89893	0.90345
575	0.87879	0.88647	0.88998	0.89116	0.89497	0.89907	0.90358
576	0.87896	0.88662	0.89013	0.89131	0.89511	0.89921	0.90370
577	0.87912	0.88677	0.89028	0.89145	0.89526	0.89934	0.90383
578	0.87929	0.88693	0.89043	0.89160	0.89540	0.89948	0.90396
579	0.87946	0.88707	0.89057	0.89175	0.89554	0.89961	0.90409
580	0.87964	0.88722	0.89072	0.89189	0.89568	0.89975	0.90422
581	0.87980	0.88738	0.89087	0.89204	0.89582	0.89988	0.90434
582	0.87998	0.88753	0.89102	0.89218	0.89596	0.90002	0.90447
583	0.88014	0.88768	0.89117	0.89233	0.89610	0.90015	0.90460
584	0.88032	0.88784	0.89132	0.89247	0.89623	0.90028	0.90472
585	0.88047	0.88798	0.89146	0.89262	0.89637	0.90041	0.90485
586	0.88062	0.88814	0.89161	0.89276	0.89651	0.90055	0.90497
587	0.88078	0.88829	0.89175	0.89290	0.89665	0.90068	0.90510
588	0.88095	0.88843	0.89190	0.89305	0.89678	0.90081	0.90522
589	0.88113	0.88858	0.89205	0.89318	0.89692	0.90094	0.90535
590	0.88130	0.88874	0.89219	0.89333	0.89706	0.90107	0.90547
591	0.88146	0.88889	0.89233	0.89347	0.89720	0.90120	0.90560
592	0.88161	0.88904	0.89248	0.89362	0.89733	0.90133	0.90572

N	0.1%	0.5%	1%	1.25%	2.5%	5%	10%
593	0.88178	0.88920	0.89262	0.89376	0.89747	0.90146	0.90584
594	0.88193	0.88934	0.89276	0.89390	0.89760	0.90159	0.90596
595	0.88209	0.88949	0.89291	0.89404	0.89774	0.90172	0.90609
596	0.88223	0.88964	0.89305	0.89418	0.89787	0.90185	0.90621
597	0.88238	0.88978	0.89320	0.89432	0.89801	0.90198	0.90633
598	0.88255	0.88993	0.89334	0.89446	0.89814	0.90210	0.90645
599	0.88269	0.89007	0.89347	0.89460	0.89828	0.90223	0.90657
600	0.88285	0.89022	0.89361	0.89473	0.89841	0.90236	0.90669
601	0.88301	0.89036	0.89375	0.89487	0.89854	0.90248	0.90681
602	0.88316	0.89051	0.89389	0.89501	0.89868	0.90261	0.90693
603	0.88330	0.89065	0.89403	0.89515	0.89880	0.90274	0.90705
604	0.88345	0.89079	0.89417	0.89529	0.89894	0.90286	0.90717
605	0.88361	0.89093	0.89431	0.89543	0.89907	0.90299	0.90729
606	0.88374	0.89107	0.89445	0.89556	0.89920	0.90311	0.90740
607	0.88389	0.89122	0.89459	0.89570	0.89933	0.90323	0.90752
608	0.88405	0.89135	0.89473	0.89583	0.89946	0.90336	0.90764
609	0.88419	0.89149	0.89486	0.89597	0.89959	0.90348	0.90776
610	0.88436	0.89163	0.89500	0.89611	0.89971	0.90360	0.90788
611	0.88451	0.89178	0.89513	0.89624	0.89984	0.90373	0.90799
612	0.88468	0.89192	0.89527	0.89637	0.89997	0.90385	0.90811
613	0.88483	0.89206	0.89540	0.89650	0.90010	0.90397	0.90823
614	0.88497	0.89220	0.89554	0.89663	0.90023	0.90409	0.90834
615	0.88511	0.89234	0.89567	0.89677	0.90036	0.90422	0.90846
616	0.88526	0.89248	0.89580	0.89690	0.90048	0.90434	0.90857
617	0.88542	0.89261	0.89594	0.89703	0.90061	0.90446	0.90869
618	0.88558	0.89276	0.89607	0.89716	0.90074	0.90458	0.90880
619	0.88573	0.89289	0.89620	0.89729	0.90086	0.90470	0.90892
620	0.88586	0.89303	0.89634	0.89743	0.90099	0.90482	0.90903
621	0.88602	0.89316	0.89647	0.89756	0.90112	0.90494	0.90914
622	0.88616	0.89329	0.89660	0.89769	0.90125	0.90506	0.90925
623	0.88631	0.89343	0.89673	0.89782	0.90137	0.90518	0.90937
624	0.88645	0.89357	0.89686	0.89795	0.90149	0.90530	0.90948
625	0.88661	0.89370	0.89699	0.89808	0.90162	0.90542	0.90959
626	0.88673	0.89383	0.89712	0.89821	0.90174	0.90554	0.90970
627	0.88689	0.89397	0.89725	0.89834	0.90186	0.90565	0.90981
628	0.88704	0.89410	0.89738	0.89847	0.90199	0.90577	0.90993
629	0.88718	0.89423	0.89750	0.89859	0.90211	0.90588	0.91004
630	0.88733	0.89437	0.89763	0.89872	0.90223	0.90600	0.91015
631	0.88746	0.89450	0.89776	0.89884	0.90235	0.90612	0.91026
632	0.88762	0.89463	0.89788	0.89897	0.90247	0.90623	0.91037
633	0.88777	0.89476	0.89801	0.89910	0.90259	0.90635	0.91048
634	0.88788	0.89490	0.89814	0.89922	0.90271	0.90646	0.91059
635	0.88803	0.89503	0.89826	0.89935	0.90283	0.90658	0.91070
636	0.88816	0.89516	0.89839	0.89947	0.90295	0.90670	0.91081
637	0.88830	0.89529	0.89852	0.89960	0.90307	0.90681	0.91091
638	0.88845	0.89542	0.89865	0.89972	0.90319	0.90692	0.91102
639	0.88858	0.89555	0.89877	0.89985	0.90331	0.90703	0.91113
640	0.88871	0.89568	0.89890	0.89998	0.90343	0.90715	0.91124
641	0.88885	0.89581	0.89903	0.90009	0.90355	0.90726	0.91134
642	0.88897	0.89594	0.89915	0.90022	0.90367	0.90737	0.91145
643	0.88912	0.89607	0.89927	0.90034	0.90378	0.90749	0.91156
644	0.88925	0.89620	0.89940	0.90046	0.90390	0.90760	0.91167
645	0.88940	0.89632	0.89952	0.90058	0.90402	0.90771	0.91177
646	0.88953	0.89645	0.89964	0.90070	0.90413	0.90782	0.91188
647	0.88966	0.89658	0.89976	0.90083	0.90425	0.90793	0.91198
648	0.88979	0.89671	0.89989	0.90095	0.90437	0.90804	0.91209
649	0.88993	0.89683	0.90001	0.90107	0.90448	0.90815	0.91219
650	0.89006	0.89695	0.90013	0.90119	0.90460	0.90826	0.91230
651	0.89021	0.89708	0.90026	0.90132	0.90472	0.90837	0.91240
652	0.89035	0.89720	0.90038	0.90144	0.90483	0.90849	0.91251
653	0.89047	0.89733	0.90050	0.90155	0.90494	0.90860	0.91261
654	0.89061	0.89745	0.90062	0.90167	0.90506	0.90870	0.91272
655	0.89075	0.89758	0.90074	0.90179	0.90517	0.90881	0.91282
656	0.89090	0.89771	0.90086	0.90191	0.90529	0.90892	0.91292
657	0.89104	0.89783	0.90098	0.90203	0.90540	0.90903	0.91303
658	0.89117	0.89796	0.90110	0.90214	0.90552	0.90914	0.91313
659	0.89131	0.89808	0.90122	0.90226	0.90563	0.90925	0.91323
660	0.89145	0.89820	0.90134	0.90237	0.90574	0.90935	0.91333
661	0.89158	0.89833	0.90145	0.90249	0.90586	0.90946	0.91344
662	0.89171	0.89845	0.90158	0.90261	0.90597	0.90957	0.91354
663	0.89183	0.89857	0.90170	0.90272	0.90608	0.90967	0.91364
664	0.89196	0.89869	0.90181	0.90284	0.90619	0.90978	0.91374
665	0.89209	0.89881	0.90193	0.90296	0.90630	0.90989	0.91384
666	0.89221	0.89893	0.90204	0.90307	0.90641	0.90999	0.91394

N	0.1%	0.5%	1%	1.25%	2.5%	5%	10%
667	0.89233	0.89905	0.90216	0.90319	0.90652	0.91010	0.91404
668	0.89245	0.89917	0.90227	0.90330	0.90663	0.91020	0.91414
669	0.89258	0.89929	0.90239	0.90341	0.90673	0.91031	0.91424
670	0.89271	0.89942	0.90250	0.90353	0.90684	0.91041	0.91434
671	0.89283	0.89953	0.90262	0.90364	0.90695	0.91052	0.91444
672	0.89296	0.89965	0.90273	0.90376	0.90706	0.91062	0.91454
673	0.89308	0.89977	0.90285	0.90387	0.90717	0.91073	0.91463
674	0.89321	0.89988	0.90297	0.90399	0.90728	0.91083	0.91473
675	0.89335	0.90000	0.90308	0.90410	0.90739	0.91094	0.91483
676	0.89349	0.90012	0.90319	0.90421	0.90750	0.91104	0.91493
677	0.89361	0.90023	0.90331	0.90433	0.90761	0.91114	0.91503
678	0.89374	0.90035	0.90342	0.90444	0.90771	0.91125	0.91513
679	0.89387	0.90047	0.90353	0.90455	0.90782	0.91135	0.91522
680	0.89398	0.90059	0.90364	0.90466	0.90793	0.91145	0.91532
681	0.89410	0.90071	0.90376	0.90477	0.90804	0.91155	0.91542
682	0.89423	0.90082	0.90387	0.90488	0.90814	0.91165	0.91551
683	0.89435	0.90093	0.90398	0.90499	0.90825	0.91175	0.91561
684	0.89448	0.90105	0.90409	0.90510	0.90836	0.91186	0.91571
685	0.89461	0.90116	0.90421	0.90521	0.90846	0.91196	0.91580
686	0.89474	0.90128	0.90431	0.90532	0.90856	0.91206	0.91590
687	0.89487	0.90139	0.90443	0.90543	0.90867	0.91216	0.91599
688	0.89498	0.90151	0.90453	0.90554	0.90877	0.91226	0.91609
689	0.89510	0.90163	0.90464	0.90565	0.90888	0.91236	0.91618
690	0.89523	0.90174	0.90475	0.90576	0.90898	0.91246	0.91628
691	0.89535	0.90186	0.90486	0.90586	0.90909	0.91256	0.91637
692	0.89546	0.90197	0.90497	0.90597	0.90919	0.91266	0.91647
693	0.89559	0.90208	0.90508	0.90608	0.90930	0.91276	0.91656
694	0.89571	0.90219	0.90519	0.90619	0.90940	0.91286	0.91665
695	0.89584	0.90231	0.90530	0.90630	0.90950	0.91295	0.91675
696	0.89595	0.90242	0.90541	0.90640	0.90960	0.91305	0.91684
697	0.89607	0.90253	0.90552	0.90651	0.90970	0.91315	0.91693
698	0.89619	0.90265	0.90562	0.90661	0.90980	0.91325	0.91703
699	0.89631	0.90276	0.90573	0.90672	0.90991	0.91335	0.91712
700	0.89642	0.90286	0.90583	0.90683	0.91001	0.91344	0.91721
701	0.89654	0.90297	0.90594	0.90693	0.91011	0.91354	0.91730
702	0.89667	0.90309	0.90605	0.90704	0.91021	0.91364	0.91740
703	0.89677	0.90320	0.90616	0.90714	0.91031	0.91373	0.91749
704	0.89688	0.90331	0.90626	0.90725	0.91041	0.91383	0.91758
705	0.89701	0.90342	0.90637	0.90736	0.91052	0.91392	0.91767
706	0.89713	0.90354	0.90647	0.90746	0.91061	0.91402	0.91776
707	0.89725	0.90365	0.90658	0.90756	0.91072	0.91412	0.91785
708	0.89736	0.90375	0.90669	0.90766	0.91082	0.91421	0.91794
709	0.89748	0.90386	0.90679	0.90777	0.91092	0.91430	0.91803
710	0.89760	0.90397	0.90689	0.90787	0.91102	0.91440	0.91812
711	0.89771	0.90408	0.90700	0.90797	0.91111	0.91449	0.91821
712	0.89783	0.90418	0.90711	0.90808	0.91121	0.91459	0.91830
713	0.89796	0.90429	0.90721	0.90818	0.91131	0.91468	0.91839
714	0.89808	0.90440	0.90731	0.90828	0.91141	0.91478	0.91848
715	0.89819	0.90451	0.90741	0.90838	0.91151	0.91487	0.91857
716	0.89831	0.90462	0.90752	0.90849	0.91161	0.91497	0.91866
717	0.89843	0.90473	0.90762	0.90859	0.91170	0.91506	0.91875
718	0.89854	0.90484	0.90772	0.90869	0.91180	0.91515	0.91884
719	0.89866	0.90494	0.90783	0.90879	0.91190	0.91524	0.91892
720	0.89877	0.90504	0.90793	0.90889	0.91200	0.91534	0.91901
721	0.89887	0.90516	0.90803	0.90899	0.91209	0.91543	0.91910
722	0.89900	0.90525	0.90813	0.90909	0.91219	0.91552	0.91919
723	0.89910	0.90536	0.90823	0.90919	0.91228	0.91561	0.91927
724	0.89920	0.90547	0.90833	0.90928	0.91238	0.91570	0.91936
725	0.89931	0.90557	0.90843	0.90938	0.91248	0.91580	0.91945
726	0.89943	0.90568	0.90853	0.90948	0.91257	0.91589	0.91953
727	0.89953	0.90579	0.90863	0.90958	0.91267	0.91598	0.91962
728	0.89964	0.90590	0.90873	0.90968	0.91276	0.91607	0.91971
729	0.89976	0.90600	0.90883	0.90977	0.91285	0.91616	0.91979
730	0.89987	0.90611	0.90892	0.90987	0.91295	0.91625	0.91988
731	0.89998	0.90621	0.90902	0.90997	0.91304	0.91635	0.91997
732	0.90010	0.90631	0.90912	0.91007	0.91313	0.91644	0.92005
733	0.90021	0.90641	0.90922	0.91017	0.91323	0.91652	0.92014
734	0.90031	0.90651	0.90932	0.91026	0.91332	0.91661	0.92022
735	0.90042	0.90661	0.90942	0.91036	0.91342	0.91670	0.92030
736	0.90053	0.90671	0.90952	0.91046	0.91351	0.91679	0.92039
737	0.90065	0.90681	0.90962	0.91055	0.91360	0.91688	0.92047
738	0.90075	0.90692	0.90972	0.91065	0.91369	0.91697	0.92056
739	0.90086	0.90701	0.90981	0.91075	0.91378	0.91706	0.92064
740	0.90096	0.90711	0.90991	0.91085	0.91388	0.91715	0.92073

N	0.1%	0.5%	1%	1.25%	2.5%	5%	10%
741	0.90106	0.90721	0.91001	0.91094	0.91397	0.91723	0.92081
742	0.90117	0.90732	0.91011	0.91104	0.91406	0.91732	0.92089
743	0.90127	0.90741	0.91020	0.91113	0.91415	0.91741	0.92097
744	0.90137	0.90751	0.91030	0.91123	0.91424	0.91749	0.92106
745	0.90149	0.90760	0.91040	0.91132	0.91433	0.91758	0.92114
746	0.90159	0.90771	0.91049	0.91142	0.91443	0.91767	0.92122
747	0.90170	0.90781	0.91059	0.91151	0.91452	0.91775	0.92131
748	0.90180	0.90791	0.91068	0.91161	0.91461	0.91784	0.92139
749	0.90190	0.90801	0.91078	0.91170	0.91470	0.91793	0.92147
750	0.90201	0.90810	0.91087	0.91180	0.91479	0.91801	0.92155
751	0.90213	0.90820	0.91097	0.91188	0.91488	0.91810	0.92164
752	0.90222	0.90830	0.91106	0.91198	0.91497	0.91818	0.92172
753	0.90233	0.90840	0.91115	0.91207	0.91506	0.91827	0.92180
754	0.90243	0.90850	0.91125	0.91216	0.91515	0.91836	0.92188
755	0.90255	0.90860	0.91134	0.91226	0.91524	0.91844	0.92196
756	0.90265	0.90870	0.91144	0.91235	0.91532	0.91853	0.92204
757	0.90275	0.90879	0.91153	0.91244	0.91541	0.91861	0.92212
758	0.90286	0.90888	0.91162	0.91254	0.91550	0.91870	0.92220
759	0.90296	0.90898	0.91172	0.91263	0.91559	0.91878	0.92228
760	0.90305	0.90907	0.91181	0.91272	0.91568	0.91887	0.92236
761	0.90316	0.90917	0.91191	0.91281	0.91577	0.91895	0.92244
762	0.90326	0.90926	0.91200	0.91290	0.91586	0.91904	0.92252
763	0.90336	0.90936	0.91209	0.91299	0.91595	0.91912	0.92260
764	0.90345	0.90946	0.91218	0.91309	0.91603	0.91921	0.92268
765	0.90356	0.90955	0.91227	0.91318	0.91612	0.91929	0.92276
766	0.90366	0.90964	0.91236	0.91327	0.91621	0.91937	0.92284
767	0.90375	0.90973	0.91245	0.91336	0.91629	0.91945	0.92292
768	0.90385	0.90983	0.91254	0.91345	0.91638	0.91954	0.92300
769	0.90396	0.90992	0.91263	0.91354	0.91647	0.91962	0.92308
770	0.90407	0.91001	0.91272	0.91363	0.91655	0.91970	0.92316
771	0.90418	0.91010	0.91281	0.91371	0.91664	0.91978	0.92323
772	0.90428	0.91020	0.91290	0.91380	0.91672	0.91986	0.92331
773	0.90437	0.91030	0.91300	0.91390	0.91681	0.91995	0.92339
774	0.90448	0.91039	0.91308	0.91398	0.91689	0.92003	0.92347
775	0.90457	0.91048	0.91317	0.91407	0.91698	0.92011	0.92354
776	0.90467	0.91057	0.91326	0.91416	0.91706	0.92019	0.92362
777	0.90477	0.91066	0.91335	0.91425	0.91715	0.92027	0.92370
778	0.90488	0.91076	0.91344	0.91434	0.91723	0.92035	0.92378
779	0.90498	0.91085	0.91353	0.91442	0.91731	0.92043	0.92385
780	0.90508	0.91094	0.91362	0.91451	0.91740	0.92051	0.92393
781	0.90518	0.91103	0.91371	0.91460	0.91748	0.92060	0.92400
782	0.90528	0.91112	0.91379	0.91468	0.91756	0.92068	0.92408
783	0.90539	0.91121	0.91388	0.91476	0.91765	0.92076	0.92416
784	0.90548	0.91130	0.91397	0.91485	0.91774	0.92084	0.92423
785	0.90559	0.91139	0.91405	0.91494	0.91782	0.92092	0.92431
786	0.90568	0.91148	0.91414	0.91503	0.91790	0.92100	0.92439
787	0.90578	0.91157	0.91423	0.91511	0.91798	0.92108	0.92446
788	0.90587	0.91167	0.91432	0.91520	0.91806	0.92116	0.92454
789	0.90598	0.91175	0.91440	0.91528	0.91815	0.92123	0.92461
790	0.90607	0.91185	0.91449	0.91537	0.91823	0.92131	0.92469
791	0.90617	0.91194	0.91457	0.91545	0.91831	0.92139	0.92476
792	0.90627	0.91203	0.91467	0.91554	0.91839	0.92147	0.92484
793	0.90635	0.91212	0.91475	0.91562	0.91847	0.92154	0.92491
794	0.90646	0.91220	0.91484	0.91571	0.91856	0.92162	0.92499
795	0.90655	0.91229	0.91492	0.91579	0.91864	0.92170	0.92506
796	0.90664	0.91238	0.91501	0.91587	0.91872	0.92178	0.92513
797	0.90673	0.91247	0.91509	0.91596	0.91880	0.92186	0.92521
798	0.90683	0.91256	0.91518	0.91605	0.91888	0.92193	0.92528
799	0.90692	0.91264	0.91526	0.91613	0.91896	0.92201	0.92535
800	0.90700	0.91273	0.91535	0.91621	0.91904	0.92209	0.92543
801	0.90708	0.91282	0.91544	0.91630	0.91912	0.92216	0.92550
802	0.90718	0.91291	0.91552	0.91638	0.91920	0.92224	0.92557
803	0.90728	0.91300	0.91560	0.91646	0.91928	0.92232	0.92565
804	0.90736	0.91308	0.91568	0.91655	0.91936	0.92239	0.92572
805	0.90745	0.91317	0.91577	0.91663	0.91944	0.92247	0.92579
806	0.90755	0.91326	0.91586	0.91672	0.91952	0.92255	0.92586
807	0.90764	0.91335	0.91594	0.91680	0.91960	0.92262	0.92594
808	0.90775	0.91343	0.91602	0.91688	0.91967	0.92270	0.92601
809	0.90784	0.91352	0.91611	0.91696	0.91976	0.92277	0.92608
810	0.90793	0.91361	0.91618	0.91705	0.91984	0.92285	0.92615
811	0.90802	0.91369	0.91627	0.91713	0.91992	0.92292	0.92623
812	0.90811	0.91378	0.91635	0.91721	0.92000	0.92300	0.92630
813	0.90820	0.91386	0.91643	0.91729	0.92008	0.92307	0.92637
814	0.90830	0.91395	0.91652	0.91737	0.92015	0.92314	0.92644

N	0.1%	0.5%	1%	1.25%	2.5%	5%	10%
815	0.90839	0.91403	0.91660	0.91745	0.92023	0.92322	0.92651
816	0.90849	0.91412	0.91668	0.91753	0.92031	0.92329	0.92658
817	0.90858	0.91420	0.91676	0.91761	0.92038	0.92337	0.92665
818	0.90867	0.91429	0.91684	0.91769	0.92046	0.92344	0.92672
819	0.90875	0.91437	0.91692	0.91777	0.92054	0.92351	0.92679
820	0.90886	0.91445	0.91700	0.91785	0.92062	0.92359	0.92686
821	0.90895	0.91454	0.91709	0.91793	0.92069	0.92366	0.92693
822	0.90902	0.91462	0.91717	0.91801	0.92077	0.92373	0.92700
823	0.90911	0.91470	0.91725	0.91809	0.92084	0.92381	0.92707
824	0.90920	0.91479	0.91733	0.91817	0.92092	0.92388	0.92714
825	0.90929	0.91487	0.91741	0.91825	0.92100	0.92396	0.92721
826	0.90938	0.91495	0.91749	0.91833	0.92108	0.92403	0.92728
827	0.90946	0.91503	0.91757	0.91841	0.92115	0.92410	0.92735
828	0.90955	0.91511	0.91764	0.91849	0.92123	0.92417	0.92742
829	0.90964	0.91519	0.91773	0.91857	0.92130	0.92425	0.92749
830	0.90974	0.91528	0.91781	0.91864	0.92138	0.92432	0.92756
831	0.90982	0.91536	0.91788	0.91872	0.92145	0.92439	0.92762
832	0.90991	0.91544	0.91796	0.91880	0.92152	0.92446	0.92769
833	0.91000	0.91552	0.91804	0.91888	0.92160	0.92454	0.92776
834	0.91009	0.91561	0.91812	0.91896	0.92167	0.92461	0.92783
835	0.91017	0.91569	0.91820	0.91904	0.92175	0.92468	0.92790
836	0.91026	0.91577	0.91828	0.91912	0.92182	0.92475	0.92796
837	0.91035	0.91585	0.91836	0.91919	0.92190	0.92482	0.92803
838	0.91044	0.91593	0.91844	0.91927	0.92197	0.92489	0.92810
839	0.91053	0.91601	0.91851	0.91935	0.92205	0.92497	0.92817
840	0.91063	0.91609	0.91859	0.91942	0.92212	0.92504	0.92823
841	0.91071	0.91617	0.91867	0.91950	0.92219	0.92511	0.92830
842	0.91079	0.91625	0.91874	0.91958	0.92227	0.92518	0.92837
843	0.91088	0.91633	0.91882	0.91965	0.92235	0.92525	0.92843
844	0.91097	0.91641	0.91890	0.91973	0.92242	0.92532	0.92850
845	0.91106	0.91649	0.91898	0.91981	0.92249	0.92539	0.92857
846	0.91114	0.91657	0.91905	0.91988	0.92256	0.92546	0.92863
847	0.91122	0.91664	0.91913	0.91996	0.92264	0.92553	0.92870
848	0.91131	0.91673	0.91921	0.92003	0.92271	0.92560	0.92877
849	0.91139	0.91680	0.91928	0.92011	0.92278	0.92566	0.92883
850	0.91147	0.91687	0.91936	0.92018	0.92286	0.92573	0.92890
851	0.91156	0.91696	0.91944	0.92026	0.92293	0.92580	0.92897
852	0.91164	0.91704	0.91951	0.92034	0.92300	0.92587	0.92903
853	0.91172	0.91712	0.91959	0.92041	0.92307	0.92594	0.92910
854	0.91180	0.91719	0.91966	0.92049	0.92314	0.92601	0.92916
855	0.91189	0.91728	0.91974	0.92056	0.92322	0.92608	0.92923
856	0.91199	0.91736	0.91981	0.92063	0.92329	0.92615	0.92929
857	0.91206	0.91743	0.91989	0.92071	0.92336	0.92622	0.92936
858	0.91215	0.91751	0.91996	0.92079	0.92343	0.92629	0.92942
859	0.91222	0.91759	0.92004	0.92086	0.92350	0.92635	0.92949
860	0.91230	0.91767	0.92011	0.92093	0.92357	0.92642	0.92955
861	0.91238	0.91775	0.92019	0.92100	0.92364	0.92649	0.92962
862	0.91247	0.91782	0.92026	0.92108	0.92371	0.92656	0.92968
863	0.91255	0.91789	0.92034	0.92115	0.92378	0.92663	0.92975
864	0.91263	0.91797	0.92041	0.92122	0.92385	0.92669	0.92981
865	0.91272	0.91805	0.92049	0.92130	0.92392	0.92676	0.92988
866	0.91280	0.91813	0.92056	0.92138	0.92400	0.92683	0.92994
867	0.91287	0.91820	0.92063	0.92145	0.92406	0.92690	0.93000
868	0.91296	0.91828	0.92071	0.92152	0.92414	0.92696	0.93007
869	0.91305	0.91836	0.92078	0.92159	0.92421	0.92703	0.93013
870	0.91313	0.91843	0.92086	0.92166	0.92428	0.92710	0.93019
871	0.91320	0.91851	0.92093	0.92174	0.92435	0.92717	0.93026
872	0.91329	0.91859	0.92100	0.92181	0.92441	0.92723	0.93032
873	0.91337	0.91866	0.92107	0.92188	0.92448	0.92730	0.93038
874	0.91344	0.91873	0.92115	0.92195	0.92455	0.92736	0.93045
875	0.91353	0.91881	0.92122	0.92203	0.92462	0.92743	0.93051
876	0.91361	0.91888	0.92129	0.92210	0.92469	0.92750	0.93057
877	0.91369	0.91896	0.92136	0.92217	0.92476	0.92756	0.93064
878	0.91376	0.91903	0.92143	0.92224	0.92483	0.92763	0.93070
879	0.91384	0.91911	0.92151	0.92231	0.92490	0.92769	0.93076
880	0.91392	0.91918	0.92158	0.92239	0.92497	0.92776	0.93082
881	0.91400	0.91926	0.92165	0.92246	0.92504	0.92782	0.93088
882	0.91407	0.91933	0.92172	0.92253	0.92510	0.92789	0.93095
883	0.91415	0.91940	0.92179	0.92260	0.92517	0.92795	0.93101
884	0.91424	0.91948	0.92186	0.92267	0.92524	0.92802	0.93107
885	0.91431	0.91955	0.92194	0.92274	0.92531	0.92808	0.93113
886	0.91439	0.91963	0.92200	0.92280	0.92537	0.92814	0.93119
887	0.91448	0.91970	0.92208	0.92288	0.92544	0.92821	0.93125
888	0.91455	0.91977	0.92215	0.92295	0.92551	0.92827	0.93132

N	0.1%	0.5%	1%	1.25%	2.5%	5%	10%
889	0.91462	0.91985	0.92222	0.92301	0.92558	0.92834	0.93138
890	0.91470	0.91992	0.92229	0.92309	0.92564	0.92840	0.93144
891	0.91479	0.92000	0.92236	0.92316	0.92571	0.92847	0.93150
892	0.91487	0.92007	0.92243	0.92322	0.92578	0.92853	0.93156
893	0.91494	0.92013	0.92250	0.92329	0.92584	0.92859	0.93162
894	0.91501	0.92021	0.92257	0.92336	0.92591	0.92866	0.93168
895	0.91508	0.92028	0.92264	0.92343	0.92597	0.92872	0.93174
896	0.91516	0.92035	0.92271	0.92350	0.92604	0.92879	0.93180
897	0.91524	0.92043	0.92278	0.92357	0.92611	0.92885	0.93186
898	0.91531	0.92050	0.92285	0.92364	0.92617	0.92891	0.93192
899	0.91539	0.92057	0.92292	0.92370	0.92624	0.92898	0.93198
900	0.91546	0.92064	0.92299	0.92377	0.92631	0.92904	0.93204
901	0.91554	0.92071	0.92306	0.92384	0.92637	0.92910	0.93210
902	0.91561	0.92078	0.92313	0.92391	0.92644	0.92916	0.93216
903	0.91569	0.92085	0.92319	0.92398	0.92650	0.92923	0.93222
904	0.91577	0.92093	0.92326	0.92405	0.92657	0.92929	0.93228
905	0.91585	0.92100	0.92333	0.92411	0.92664	0.92935	0.93234
906	0.91592	0.92107	0.92340	0.92418	0.92670	0.92941	0.93240
907	0.91600	0.92113	0.92347	0.92425	0.92676	0.92947	0.93246
908	0.91608	0.92121	0.92353	0.92432	0.92683	0.92954	0.93252
909	0.91615	0.92128	0.92360	0.92438	0.92689	0.92960	0.93257
910	0.91624	0.92135	0.92367	0.92445	0.92696	0.92966	0.93263
911	0.91631	0.92142	0.92373	0.92452	0.92702	0.92972	0.93269
912	0.91638	0.92149	0.92380	0.92458	0.92708	0.92978	0.93275
913	0.91645	0.92156	0.92387	0.92465	0.92715	0.92984	0.93281
914	0.91653	0.92163	0.92394	0.92471	0.92721	0.92990	0.93287
915	0.91661	0.92170	0.92401	0.92478	0.92728	0.92996	0.93292
916	0.91667	0.92176	0.92407	0.92485	0.92734	0.93002	0.93298
917	0.91675	0.92183	0.92414	0.92491	0.92740	0.93009	0.93304
918	0.91682	0.92190	0.92421	0.92498	0.92747	0.93015	0.93310
919	0.91689	0.92197	0.92427	0.92504	0.92753	0.93021	0.93315
920	0.91696	0.92204	0.92434	0.92511	0.92759	0.93027	0.93321
921	0.91704	0.92211	0.92441	0.92518	0.92766	0.93033	0.93327
922	0.91712	0.92218	0.92447	0.92524	0.92772	0.93039	0.93332
923	0.91720	0.92225	0.92454	0.92530	0.92778	0.93045	0.93338
924	0.91727	0.92232	0.92461	0.92537	0.92785	0.93051	0.93344
925	0.91735	0.92238	0.92467	0.92544	0.92791	0.93057	0.93350
926	0.91742	0.92245	0.92474	0.92551	0.92797	0.93063	0.93355
927	0.91749	0.92252	0.92481	0.92557	0.92803	0.93069	0.93361
928	0.91756	0.92259	0.92487	0.92563	0.92809	0.93075	0.93367
929	0.91764	0.92266	0.92494	0.92570	0.92815	0.93081	0.93373
930	0.91770	0.92272	0.92500	0.92576	0.92822	0.93086	0.93378
931	0.91778	0.92279	0.92507	0.92583	0.92828	0.93092	0.93384
932	0.91785	0.92286	0.92513	0.92589	0.92834	0.93098	0.93389
933	0.91793	0.92292	0.92520	0.92596	0.92840	0.93104	0.93395
934	0.91801	0.92298	0.92526	0.92602	0.92846	0.93110	0.93401
935	0.91807	0.92305	0.92532	0.92608	0.92852	0.93116	0.93406
936	0.91814	0.92312	0.92539	0.92615	0.92859	0.93122	0.93412
937	0.91822	0.92319	0.92545	0.92621	0.92865	0.93127	0.93417
938	0.91829	0.92326	0.92551	0.92627	0.92871	0.93133	0.93423
939	0.91836	0.92332	0.92558	0.92634	0.92877	0.93139	0.93428
940	0.91843	0.92339	0.92564	0.92640	0.92883	0.93145	0.93434
941	0.91851	0.92345	0.92570	0.92646	0.92889	0.93151	0.93439
942	0.91858	0.92352	0.92577	0.92653	0.92895	0.93157	0.93445
943	0.91865	0.92358	0.92583	0.92659	0.92901	0.93163	0.93450
944	0.91872	0.92365	0.92589	0.92665	0.92907	0.93168	0.93456
945	0.91879	0.92371	0.92596	0.92672	0.92913	0.93174	0.93461
946	0.91887	0.92378	0.92602	0.92678	0.92919	0.93180	0.93467
947	0.91893	0.92385	0.92608	0.92684	0.92925	0.93185	0.93472
948	0.91900	0.92391	0.92615	0.92690	0.92931	0.93191	0.93478
949	0.91907	0.92398	0.92621	0.92696	0.92937	0.93197	0.93483
950	0.91914	0.92404	0.92628	0.92702	0.92943	0.93202	0.93489
951	0.91921	0.92410	0.92634	0.92709	0.92949	0.93208	0.93494
952	0.91928	0.92417	0.92640	0.92715	0.92955	0.93214	0.93499
953	0.91935	0.92424	0.92646	0.92721	0.92961	0.93220	0.93505
954	0.91941	0.92430	0.92652	0.92727	0.92967	0.93225	0.93510
955	0.91948	0.92436	0.92658	0.92733	0.92972	0.93231	0.93516
956	0.91955	0.92443	0.92664	0.92739	0.92978	0.93237	0.93521
957	0.91962	0.92449	0.92671	0.92745	0.92984	0.93242	0.93526
958	0.91969	0.92455	0.92677	0.92751	0.92990	0.93248	0.93532
959	0.91975	0.92462	0.92683	0.92757	0.92996	0.93254	0.93537
960	0.91982	0.92468	0.92689	0.92764	0.93002	0.93259	0.93542
961	0.91989	0.92474	0.92696	0.92769	0.93008	0.93265	0.93548
962	0.91996	0.92481	0.92702	0.92776	0.93014	0.93271	0.93553

N	0.1%	0.5%	1%	1.25%	2.5%	5%	10%
963	0.92002	0.92488	0.92708	0.92782	0.93020	0.93276	0.93558
964	0.92009	0.92493	0.92714	0.92788	0.93025	0.93282	0.93564
965	0.92016	0.92500	0.92720	0.92794	0.93031	0.93287	0.93569
966	0.92022	0.92506	0.92726	0.92800	0.93037	0.93293	0.93574
967	0.92029	0.92512	0.92732	0.92806	0.93043	0.93298	0.93580
968	0.92037	0.92518	0.92738	0.92812	0.93048	0.93304	0.93585
969	0.92042	0.92524	0.92744	0.92818	0.93054	0.93310	0.93590
970	0.92050	0.92531	0.92750	0.92824	0.93060	0.93315	0.93595
971	0.92057	0.92537	0.92756	0.92830	0.93066	0.93321	0.93601
972	0.92063	0.92543	0.92762	0.92836	0.93071	0.93326	0.93606
973	0.92069	0.92550	0.92768	0.92842	0.93077	0.93331	0.93611
974	0.92076	0.92556	0.92774	0.92847	0.93083	0.93337	0.93616
975	0.92084	0.92562	0.92780	0.92853	0.93088	0.93342	0.93621
976	0.92091	0.92568	0.92786	0.92859	0.93094	0.93348	0.93626
977	0.92098	0.92575	0.92792	0.92865	0.93100	0.93353	0.93632
978	0.92104	0.92580	0.92798	0.92871	0.93106	0.93358	0.93637
979	0.92111	0.92586	0.92804	0.92877	0.93111	0.93364	0.93642
980	0.92118	0.92592	0.92810	0.92883	0.93117	0.93369	0.93647
981	0.92124	0.92599	0.92816	0.92888	0.93123	0.93374	0.93652
982	0.92130	0.92605	0.92822	0.92894	0.93128	0.93380	0.93657
983	0.92137	0.92611	0.92828	0.92900	0.93134	0.93385	0.93663
984	0.92144	0.92617	0.92834	0.92906	0.93140	0.93391	0.93668
985	0.92151	0.92624	0.92840	0.92912	0.93145	0.93396	0.93673
986	0.92157	0.92630	0.92846	0.92918	0.93151	0.93401	0.93678
987	0.92164	0.92636	0.92852	0.92924	0.93156	0.93407	0.93683
988	0.92171	0.92642	0.92858	0.92929	0.93162	0.93412	0.93688
989	0.92177	0.92648	0.92864	0.92935	0.93168	0.93418	0.93693
990	0.92183	0.92654	0.92869	0.92941	0.93173	0.93423	0.93698
991	0.92190	0.92660	0.92875	0.92947	0.93179	0.93428	0.93703
992	0.92197	0.92666	0.92881	0.92952	0.93184	0.93434	0.93708
993	0.92203	0.92672	0.92887	0.92958	0.93190	0.93439	0.93713
994	0.92209	0.92678	0.92892	0.92964	0.93195	0.93444	0.93718
995	0.92215	0.92684	0.92898	0.92969	0.93201	0.93449	0.93723
996	0.92222	0.92690	0.92904	0.92975	0.93206	0.93455	0.93728
997	0.92228	0.92696	0.92910	0.92981	0.93212	0.93460	0.93733
998	0.92234	0.92702	0.92915	0.92986	0.93217	0.93465	0.93738
999	0.92240	0.92708	0.92921	0.92992	0.93223	0.93470	0.93743
1000	0.92246	0.92714	0.92927	0.92998	0.93228	0.93476	0.93748

Table 2 – *GM7(6H1L or 1H6L)*. Critical values of upper significance levels for the Grubbs multiple-7 outlier test, applicable in situations where the six highest and one lowest or one highest and six lowest observations are simultaneously identified as outliers, denoted as *GM7(6H1L)* and *GM7(1H6L)*, respectively.

N	0.1%	0.5%	1%	1.25%	2.5%	5%	10%
9	0.00000	0.00000	0.00000	0.00000	0.00001	0.00003	0.00014
10	0.00002	0.00011	0.00023	0.00029	0.00060	0.00127	0.00274
11	0.00033	0.00101	0.00165	0.00194	0.00321	0.00541	0.00936
12	0.00142	0.00337	0.00494	0.00559	0.00829	0.01251	0.01927
13	0.00366	0.00743	0.01017	0.01127	0.01566	0.02195	0.03146
14	0.00719	0.01314	0.01715	0.01874	0.02478	0.03318	0.04519
15	0.01195	0.02021	0.02557	0.02761	0.03533	0.04561	0.05989
16	0.01793	0.02849	0.03511	0.03761	0.04680	0.05887	0.07509
17	0.02475	0.03761	0.04547	0.04837	0.05900	0.07258	0.09057
18	0.03238	0.04755	0.05656	0.05984	0.07169	0.08662	0.10607
19	0.04086	0.05806	0.06802	0.07169	0.08459	0.10073	0.12143
20	0.04976	0.06892	0.07981	0.08374	0.09768	0.11482	0.13659
21	0.05901	0.08000	0.09172	0.09593	0.11074	0.12878	0.15144
22	0.06866	0.09118	0.10362	0.10810	0.12371	0.14250	0.16594
23	0.07846	0.10242	0.11561	0.12029	0.13647	0.15597	0.18009
24	0.08844	0.11362	0.12745	0.13234	0.14917	0.16918	0.19383
25	0.09842	0.12499	0.13921	0.14427	0.16161	0.18212	0.20720
26	0.10846	0.13615	0.15085	0.15606	0.17381	0.19476	0.22011
27	0.11871	0.14719	0.16228	0.16754	0.18566	0.20700	0.23270
28	0.12876	0.15820	0.17345	0.17885	0.19737	0.21895	0.24489
29	0.13893	0.16877	0.18445	0.18993	0.20875	0.23056	0.25669
30	0.14866	0.17932	0.19517	0.20078	0.21984	0.24183	0.26812
31	0.15821	0.18958	0.20576	0.21143	0.23060	0.25286	0.27923
32	0.16792	0.19974	0.21614	0.22183	0.24119	0.26355	0.28994
33	0.17741	0.20978	0.22622	0.23200	0.25152	0.27397	0.30038
34	0.18670	0.21941	0.23615	0.24199	0.26157	0.28410	0.31047
35	0.19615	0.22900	0.24589	0.25170	0.27143	0.29389	0.32028

N	0.1%	0.5%	1%	1.25%	2.5%	5%	10%
36	0.20504	0.23848	0.25543	0.26130	0.28096	0.30344	0.32977
37	0.21352	0.24764	0.26468	0.27052	0.29020	0.31273	0.33898
38	0.22232	0.25663	0.27371	0.27959	0.29925	0.32176	0.34791
39	0.23102	0.26533	0.28240	0.28825	0.30806	0.33055	0.35658
40	0.23922	0.27385	0.29098	0.29695	0.31664	0.33907	0.36503
41	0.24759	0.28221	0.29942	0.30531	0.32506	0.34739	0.37318
42	0.25551	0.29044	0.30757	0.31350	0.33320	0.35545	0.38115
43	0.26363	0.29844	0.31557	0.32152	0.34119	0.36335	0.38890
44	0.27125	0.30614	0.32337	0.32930	0.34893	0.37099	0.39641
45	0.27901	0.31384	0.33107	0.33700	0.35645	0.37840	0.40376
46	0.28657	0.32130	0.33846	0.34431	0.36381	0.38568	0.41087
47	0.29385	0.32859	0.34572	0.35157	0.37101	0.39280	0.41781
48	0.30093	0.33574	0.35282	0.35862	0.37803	0.39969	0.42457
49	0.30812	0.34274	0.35971	0.36549	0.38484	0.40645	0.43116
50	0.31490	0.34956	0.36651	0.37231	0.39150	0.41304	0.43757
51	0.32188	0.35629	0.37326	0.37900	0.39808	0.41944	0.44384
52	0.32853	0.36287	0.37971	0.38544	0.40450	0.42569	0.44993
53	0.33504	0.36926	0.38604	0.39173	0.41071	0.43185	0.45586
54	0.34141	0.37555	0.39224	0.39792	0.41678	0.43781	0.46167
55	0.34763	0.38178	0.39835	0.40398	0.42272	0.44363	0.46736
56	0.35362	0.38783	0.40430	0.40988	0.42855	0.44933	0.47288
57	0.35960	0.39376	0.41011	0.41571	0.43427	0.45489	0.47828
58	0.36536	0.39953	0.41584	0.42143	0.43987	0.46033	0.48355
59	0.37148	0.40519	0.42149	0.42700	0.44526	0.46567	0.48873
60	0.37713	0.41074	0.42694	0.43245	0.45060	0.47088	0.49378
61	0.38252	0.41628	0.43233	0.43782	0.45584	0.47598	0.49871
62	0.38812	0.42154	0.43760	0.44303	0.46097	0.48098	0.50353
63	0.39341	0.42668	0.44274	0.44813	0.46602	0.48586	0.50826
64	0.39877	0.43171	0.44777	0.45311	0.47091	0.49064	0.51286
65	0.40388	0.43674	0.45268	0.45803	0.47574	0.49528	0.51739
66	0.40895	0.44167	0.45751	0.46284	0.48047	0.49987	0.52180
67	0.41395	0.44662	0.46227	0.46758	0.48507	0.50437	0.52612
68	0.41888	0.45133	0.46699	0.47227	0.48957	0.50876	0.53038
69	0.42374	0.45593	0.47156	0.47674	0.49401	0.51310	0.53453
70	0.42832	0.46052	0.47607	0.48127	0.49841	0.51731	0.53861
71	0.43301	0.46507	0.48045	0.48563	0.50266	0.52144	0.54263
72	0.43742	0.46954	0.48472	0.48993	0.50686	0.52550	0.54652
73	0.44187	0.47386	0.48898	0.49416	0.51100	0.52949	0.55037
74	0.44619	0.47814	0.49321	0.49834	0.51501	0.53341	0.55413
75	0.45047	0.48230	0.49728	0.50239	0.51899	0.53724	0.55781
76	0.45462	0.48635	0.50132	0.50633	0.52287	0.54102	0.56144
77	0.45893	0.49041	0.50525	0.51024	0.52670	0.54474	0.56500
78	0.46331	0.49440	0.50911	0.51408	0.53045	0.54838	0.56851
79	0.46724	0.49832	0.51293	0.51786	0.53413	0.55195	0.57194
80	0.47116	0.50208	0.51665	0.52161	0.53774	0.55546	0.57530
81	0.47524	0.50583	0.52033	0.52526	0.54129	0.55889	0.57862
82	0.47909	0.50953	0.52393	0.52886	0.54480	0.56227	0.58189
83	0.48281	0.51317	0.52752	0.53240	0.54824	0.56560	0.58507
84	0.48652	0.51673	0.53104	0.53587	0.55164	0.56888	0.58820
85	0.49029	0.52025	0.53449	0.53928	0.55497	0.57211	0.59130
86	0.49377	0.52371	0.53789	0.54266	0.55821	0.57527	0.59433
87	0.49736	0.52710	0.54125	0.54597	0.56145	0.57837	0.59732
88	0.50098	0.53056	0.54450	0.54920	0.56461	0.58145	0.60026
89	0.50443	0.53387	0.54774	0.55240	0.56771	0.58444	0.60314
90	0.50786	0.53711	0.55091	0.55557	0.57079	0.58741	0.60599
91	0.51123	0.54035	0.55410	0.55870	0.57379	0.59034	0.60878
92	0.51452	0.54349	0.55719	0.56176	0.57677	0.59320	0.61152
93	0.51784	0.54664	0.56021	0.56475	0.57970	0.59603	0.61423
94	0.52100	0.54967	0.56319	0.56771	0.58257	0.59881	0.61687
95	0.52423	0.55274	0.56614	0.57064	0.58540	0.60152	0.61951
96	0.52740	0.55572	0.56907	0.57353	0.58819	0.60422	0.62208
97	0.53050	0.55861	0.57187	0.57635	0.59095	0.60689	0.62464
98	0.53337	0.56152	0.57469	0.57916	0.59367	0.60950	0.62714
99	0.53639	0.56438	0.57747	0.58190	0.59631	0.61205	0.62959
100	0.53936	0.56717	0.58022	0.58462	0.59894	0.61459	0.63204
101	0.54228	0.56994	0.58294	0.58728	0.60154	0.61709	0.63443
102	0.54517	0.57269	0.58562	0.58994	0.60408	0.61955	0.63679
103	0.54800	0.57535	0.58821	0.59253	0.60657	0.62197	0.63910
104	0.55078	0.57795	0.59081	0.59511	0.60908	0.62437	0.64138
105	0.55340	0.58058	0.59338	0.59765	0.61154	0.62673	0.64364
106	0.55625	0.58319	0.59590	0.60014	0.61395	0.62904	0.64587
107	0.55899	0.58574	0.59839	0.60260	0.61634	0.63134	0.64807
108	0.56151	0.58829	0.60080	0.60505	0.61869	0.63361	0.65023
109	0.56418	0.59079	0.60321	0.60741	0.62100	0.63584	0.65236

N	0.1%	0.5%	1%	1.25%	2.5%	5%	10%
110	0.56678	0.59326	0.60559	0.60977	0.62327	0.63802	0.65446
111	0.56939	0.59556	0.60796	0.61209	0.62552	0.64018	0.65653
112	0.57188	0.59801	0.61028	0.61434	0.62773	0.64233	0.65858
113	0.57434	0.60036	0.61256	0.61665	0.62993	0.64446	0.66060
114	0.57688	0.60266	0.61483	0.61887	0.63208	0.64654	0.66259
115	0.57937	0.60492	0.61702	0.62106	0.63423	0.64860	0.66454
116	0.58171	0.60716	0.61920	0.62325	0.63635	0.65061	0.66648
117	0.58403	0.60939	0.62139	0.62540	0.63843	0.65260	0.66839
118	0.58640	0.61161	0.62354	0.62750	0.64049	0.65458	0.67029
119	0.58881	0.61380	0.62566	0.62959	0.64252	0.65653	0.67216
120	0.59102	0.61594	0.62774	0.63168	0.64454	0.65846	0.67400
121	0.59323	0.61806	0.62981	0.63372	0.64651	0.66036	0.67581
122	0.59543	0.62017	0.63186	0.63576	0.64846	0.66223	0.67760
123	0.59763	0.62224	0.63388	0.63775	0.65038	0.66409	0.67936
124	0.59984	0.62431	0.63585	0.63969	0.65230	0.66594	0.68111
125	0.60200	0.62633	0.63777	0.64162	0.65419	0.66774	0.68284
126	0.60408	0.62838	0.63970	0.64352	0.65604	0.66952	0.68457
127	0.60617	0.63032	0.64162	0.64546	0.65788	0.67130	0.68626
128	0.60823	0.63226	0.64352	0.64734	0.65972	0.67304	0.68793
129	0.61026	0.63419	0.64539	0.64919	0.66150	0.67476	0.68959
130	0.61221	0.63612	0.64722	0.65100	0.66326	0.67648	0.69120
131	0.61422	0.63802	0.64907	0.65282	0.66502	0.67818	0.69281
132	0.61631	0.63991	0.65088	0.65459	0.66676	0.67984	0.69439
133	0.61830	0.64172	0.65267	0.65636	0.66848	0.68149	0.69598
134	0.62022	0.64353	0.65442	0.65810	0.67015	0.68311	0.69753
135	0.62210	0.64535	0.65617	0.65981	0.67184	0.68473	0.69907
136	0.62401	0.64713	0.65789	0.66155	0.67348	0.68632	0.70058
137	0.62587	0.64892	0.65962	0.66325	0.67512	0.68790	0.70207
138	0.62779	0.65067	0.66129	0.66493	0.67671	0.68945	0.70354
139	0.62957	0.65235	0.66301	0.66658	0.67832	0.69099	0.70501
140	0.63146	0.65407	0.66466	0.66822	0.67990	0.69250	0.70646
141	0.63324	0.65573	0.66628	0.66986	0.68147	0.69401	0.70789
142	0.63500	0.65735	0.66792	0.67149	0.68303	0.69549	0.70932
143	0.63678	0.65900	0.66955	0.67308	0.68456	0.69698	0.71072
144	0.63853	0.66064	0.67116	0.67466	0.68608	0.69843	0.71210
145	0.64028	0.66231	0.67271	0.67623	0.68759	0.69987	0.71347
146	0.64190	0.66391	0.67431	0.67776	0.68905	0.70130	0.71484
147	0.64352	0.66552	0.67585	0.67928	0.69051	0.70272	0.71618
148	0.64522	0.66707	0.67736	0.68079	0.69196	0.70410	0.71750
149	0.64691	0.66859	0.67886	0.68229	0.69340	0.70548	0.71881
150	0.64854	0.67019	0.68037	0.68375	0.69483	0.70684	0.72012
151	0.65019	0.67169	0.68182	0.68523	0.69622	0.70819	0.72140
152	0.65184	0.67320	0.68330	0.68667	0.69762	0.70954	0.72268
153	0.65333	0.67469	0.68473	0.68812	0.69901	0.71086	0.72394
154	0.65490	0.67615	0.68619	0.68952	0.70037	0.71216	0.72519
155	0.65647	0.67763	0.68761	0.69093	0.70173	0.71348	0.72642
156	0.65805	0.67906	0.68899	0.69231	0.70308	0.71476	0.72765
157	0.65951	0.68048	0.69038	0.69366	0.70440	0.71604	0.72886
158	0.66106	0.68192	0.69172	0.69500	0.70571	0.71728	0.73007
159	0.66253	0.68331	0.69310	0.69635	0.70701	0.71852	0.73126
160	0.66401	0.68471	0.69446	0.69771	0.70831	0.71975	0.73243
161	0.66554	0.68607	0.69578	0.69903	0.70957	0.72097	0.73359
162	0.66692	0.68742	0.69709	0.70033	0.71086	0.72218	0.73474
163	0.66829	0.68879	0.69839	0.70161	0.71210	0.72338	0.73589
164	0.66980	0.69009	0.69970	0.70289	0.71333	0.72458	0.73702
165	0.67120	0.69141	0.70099	0.70419	0.71455	0.72576	0.73813
166	0.67264	0.69272	0.70227	0.70545	0.71577	0.72691	0.73925
167	0.67399	0.69402	0.70350	0.70669	0.71696	0.72806	0.74035
168	0.67539	0.69533	0.70479	0.70791	0.71815	0.72921	0.74144
169	0.67679	0.69662	0.70601	0.70916	0.71934	0.73034	0.74252
170	0.67814	0.69790	0.70721	0.71034	0.72049	0.73147	0.74358
171	0.67945	0.69916	0.70842	0.71155	0.72165	0.73257	0.74464
172	0.68078	0.70043	0.70965	0.71273	0.72279	0.73367	0.74569
173	0.68198	0.70167	0.71085	0.71391	0.72393	0.73476	0.74673
174	0.68327	0.70289	0.71201	0.71507	0.72506	0.73585	0.74775
175	0.68460	0.70409	0.71322	0.71623	0.72617	0.73692	0.74877
176	0.68585	0.70525	0.71435	0.71737	0.72726	0.73798	0.74978
177	0.68709	0.70645	0.71550	0.71852	0.72836	0.73903	0.75078
178	0.68833	0.70764	0.71663	0.71966	0.72946	0.74008	0.75178
179	0.68954	0.70880	0.71776	0.72077	0.73053	0.74111	0.75277
180	0.69079	0.70998	0.71890	0.72188	0.73160	0.74213	0.75374
181	0.69204	0.71111	0.71999	0.72298	0.73266	0.74314	0.75470
182	0.69326	0.71225	0.72109	0.72406	0.73371	0.74415	0.75566
183	0.69451	0.71336	0.72219	0.72515	0.73474	0.74514	0.75661

N	0.1%	0.5%	1%	1.25%	2.5%	5%	10%
184	0.69572	0.71447	0.72327	0.72621	0.73579	0.74613	0.75755
185	0.69683	0.71556	0.72434	0.72728	0.73681	0.74711	0.75849
186	0.69797	0.71667	0.72541	0.72832	0.73782	0.74808	0.75941
187	0.69910	0.71772	0.72646	0.72936	0.73882	0.74904	0.76032
188	0.70024	0.71881	0.72750	0.73039	0.73980	0.75000	0.76123
189	0.70134	0.71990	0.72854	0.73143	0.74079	0.75095	0.76213
190	0.70246	0.72097	0.72956	0.73243	0.74178	0.75187	0.76302
191	0.70358	0.72200	0.73059	0.73344	0.74276	0.75281	0.76391
192	0.70462	0.72302	0.73161	0.73445	0.74372	0.75374	0.76478
193	0.70572	0.72403	0.73260	0.73543	0.74466	0.75465	0.76566
194	0.70689	0.72505	0.73358	0.73641	0.74562	0.75556	0.76652
195	0.70795	0.72608	0.73455	0.73738	0.74656	0.75645	0.76738
196	0.70903	0.72708	0.73554	0.73836	0.74748	0.75735	0.76823
197	0.71009	0.72808	0.73651	0.73932	0.74841	0.75823	0.76907
198	0.71118	0.72909	0.73748	0.74026	0.74932	0.75911	0.76991
199	0.71223	0.73008	0.73841	0.74120	0.75023	0.75998	0.77074
200	0.71328	0.73104	0.73935	0.74213	0.75113	0.76085	0.77156
201	0.71430	0.73200	0.74029	0.74307	0.75202	0.76170	0.77238
202	0.71539	0.73296	0.74122	0.74400	0.75291	0.76255	0.77319
203	0.71636	0.73391	0.74216	0.74490	0.75379	0.76340	0.77399
204	0.71736	0.73489	0.74307	0.74579	0.75466	0.76424	0.77479
205	0.71839	0.73580	0.74396	0.74669	0.75551	0.76507	0.77557
206	0.71935	0.73677	0.74486	0.74757	0.75637	0.76589	0.77636
207	0.72027	0.73769	0.74578	0.74847	0.75724	0.76671	0.77713
208	0.72123	0.73859	0.74666	0.74934	0.75809	0.76751	0.77791
209	0.72221	0.73951	0.74754	0.75022	0.75892	0.76832	0.77868
210	0.72317	0.74043	0.74841	0.75108	0.75975	0.76911	0.77944
211	0.72412	0.74131	0.74928	0.75196	0.76058	0.76992	0.78019
212	0.72510	0.74221	0.75014	0.75281	0.76141	0.77070	0.78094
213	0.72604	0.74309	0.75099	0.75363	0.76222	0.77148	0.78168
214	0.72701	0.74397	0.75185	0.75447	0.76302	0.77225	0.78242
215	0.72797	0.74482	0.75269	0.75531	0.76383	0.77303	0.78316
216	0.72887	0.74568	0.75352	0.75612	0.76462	0.77379	0.78388
217	0.72975	0.74654	0.75437	0.75695	0.76541	0.77454	0.78460
218	0.73066	0.74737	0.75520	0.75777	0.76620	0.77530	0.78532
219	0.73158	0.74821	0.75602	0.75857	0.76697	0.77604	0.78603
220	0.73240	0.74905	0.75682	0.75938	0.76775	0.77678	0.78674
221	0.73332	0.74992	0.75762	0.76016	0.76851	0.77753	0.78743
222	0.73421	0.75078	0.75843	0.76095	0.76927	0.77826	0.78813
223	0.73511	0.75161	0.75921	0.76174	0.77003	0.77898	0.78881
224	0.73597	0.75241	0.75999	0.76251	0.77078	0.77970	0.78950
225	0.73683	0.75320	0.76077	0.76330	0.77152	0.78041	0.79018
226	0.73771	0.75399	0.76155	0.76405	0.77226	0.78112	0.79086
227	0.73852	0.75477	0.76232	0.76482	0.77299	0.78183	0.79152
228	0.73930	0.75558	0.76309	0.76558	0.77372	0.78252	0.79219
229	0.74018	0.75637	0.76385	0.76632	0.77443	0.78321	0.79286
230	0.74095	0.75715	0.76461	0.76708	0.77516	0.78390	0.79351
231	0.74174	0.75790	0.76538	0.76783	0.77587	0.78458	0.79416
232	0.74258	0.75866	0.76611	0.76858	0.77659	0.78526	0.79481
233	0.74339	0.75942	0.76682	0.76930	0.77728	0.78595	0.79545
234	0.74425	0.76017	0.76755	0.77002	0.77799	0.78661	0.79609
235	0.74500	0.76089	0.76829	0.77075	0.77868	0.78727	0.79672
236	0.74583	0.76167	0.76902	0.77146	0.77936	0.78793	0.79735
237	0.74665	0.76239	0.76974	0.77218	0.78004	0.78858	0.79797
238	0.74742	0.76315	0.77046	0.77287	0.78073	0.78923	0.79859
239	0.74824	0.76387	0.77116	0.77358	0.78140	0.78987	0.79921
240	0.74900	0.76458	0.77186	0.77428	0.78207	0.79052	0.79982
241	0.74974	0.76530	0.77256	0.77496	0.78275	0.79116	0.80042
242	0.75050	0.76604	0.77325	0.77564	0.78342	0.79179	0.80102
243	0.75128	0.76677	0.77396	0.77632	0.78406	0.79242	0.80162
244	0.75204	0.76750	0.77464	0.77701	0.78472	0.79305	0.80222
245	0.75284	0.76819	0.77532	0.77767	0.78536	0.79366	0.80281
246	0.75356	0.76890	0.77602	0.77836	0.78601	0.79428	0.80340
247	0.75429	0.76959	0.77668	0.77902	0.78665	0.79489	0.80397
248	0.75505	0.77029	0.77734	0.77968	0.78729	0.79550	0.80456
249	0.75576	0.77097	0.77800	0.78032	0.78792	0.79611	0.80514
250	0.75648	0.77165	0.77864	0.78097	0.78855	0.79671	0.80570
251	0.75720	0.77231	0.77929	0.78163	0.78918	0.79731	0.80627
252	0.75791	0.77300	0.77994	0.78226	0.78979	0.79790	0.80683
253	0.75860	0.77367	0.78060	0.78289	0.79040	0.79849	0.80739
254	0.75934	0.77432	0.78124	0.78354	0.79100	0.79908	0.80795
255	0.76009	0.77496	0.78187	0.78415	0.79163	0.79965	0.80850
256	0.76083	0.77562	0.78252	0.78479	0.79222	0.80023	0.80905
257	0.76152	0.77625	0.78316	0.78542	0.79280	0.80080	0.80960

N	0.1%	0.5%	1%	1.25%	2.5%	5%	10%
258	0.76219	0.77691	0.78377	0.78604	0.79340	0.80137	0.81014
259	0.76290	0.77753	0.78438	0.78666	0.79399	0.80194	0.81068
260	0.76360	0.77817	0.78500	0.78725	0.79458	0.80251	0.81122
261	0.76423	0.77878	0.78562	0.78786	0.79516	0.80307	0.81175
262	0.76492	0.77945	0.78621	0.78846	0.79574	0.80362	0.81228
263	0.76561	0.78006	0.78681	0.78906	0.79631	0.80417	0.81280
264	0.76628	0.78069	0.78738	0.78966	0.79688	0.80472	0.81332
265	0.76698	0.78132	0.78798	0.79024	0.79744	0.80527	0.81385
266	0.76759	0.78195	0.78858	0.79084	0.79801	0.80580	0.81436
267	0.76825	0.78255	0.78919	0.79141	0.79857	0.80635	0.81488
268	0.76888	0.78316	0.78977	0.79200	0.79914	0.80688	0.81539
269	0.76955	0.78375	0.79035	0.79257	0.79970	0.80742	0.81589
270	0.77023	0.78436	0.79093	0.79314	0.80025	0.80794	0.81640
271	0.77085	0.78495	0.79150	0.79372	0.80080	0.80847	0.81690
272	0.77148	0.78555	0.79207	0.79428	0.80135	0.80899	0.81739
273	0.77211	0.78615	0.79264	0.79483	0.80189	0.80951	0.81789
274	0.77271	0.78672	0.79320	0.79540	0.80243	0.81003	0.81838
275	0.77333	0.78730	0.79378	0.79595	0.80296	0.81053	0.81887
276	0.77397	0.78788	0.79434	0.79651	0.80349	0.81104	0.81935
277	0.77456	0.78845	0.79491	0.79706	0.80403	0.81156	0.81984
278	0.77513	0.78901	0.79545	0.79761	0.80456	0.81205	0.82032
279	0.77577	0.78959	0.79600	0.79815	0.80508	0.81256	0.82079
280	0.77637	0.79015	0.79656	0.79869	0.80560	0.81305	0.82126
281	0.77700	0.79071	0.79711	0.79923	0.80611	0.81355	0.82174
282	0.77761	0.79127	0.79765	0.79976	0.80663	0.81403	0.82221
283	0.77817	0.79181	0.79820	0.80030	0.80713	0.81453	0.82268
284	0.77876	0.79237	0.79873	0.80083	0.80764	0.81501	0.82313
285	0.77933	0.79293	0.79926	0.80134	0.80815	0.81549	0.82360
286	0.77994	0.79348	0.79977	0.80188	0.80865	0.81598	0.82406
287	0.78050	0.79401	0.80031	0.80240	0.80915	0.81645	0.82451
288	0.78106	0.79456	0.80083	0.80291	0.80965	0.81693	0.82496
289	0.78164	0.79510	0.80135	0.80342	0.81014	0.81740	0.82541
290	0.78222	0.79563	0.80188	0.80394	0.81063	0.81788	0.82586
291	0.78276	0.79616	0.80239	0.80445	0.81111	0.81834	0.82630
292	0.78334	0.79670	0.80290	0.80495	0.81160	0.81881	0.82674
293	0.78389	0.79720	0.80340	0.80545	0.81208	0.81927	0.82718
294	0.78443	0.79772	0.80391	0.80595	0.81256	0.81973	0.82762
295	0.78501	0.79825	0.80441	0.80645	0.81304	0.82019	0.82805
296	0.78553	0.79877	0.80490	0.80695	0.81351	0.82065	0.82849
297	0.78607	0.79928	0.80539	0.80744	0.81398	0.82109	0.82892
298	0.78663	0.79978	0.80589	0.80792	0.81446	0.82155	0.82935
299	0.78716	0.80029	0.80637	0.80840	0.81492	0.82200	0.82978
300	0.78771	0.80080	0.80686	0.80888	0.81538	0.82244	0.83020
301	0.78824	0.80130	0.80734	0.80937	0.81585	0.82288	0.83062
302	0.78880	0.80179	0.80782	0.80983	0.81631	0.82331	0.83104
303	0.78930	0.80227	0.80829	0.81029	0.81676	0.82375	0.83145
304	0.78988	0.80277	0.80877	0.81076	0.81721	0.82418	0.83186
305	0.79037	0.80326	0.80925	0.81124	0.81766	0.82461	0.83228
306	0.79087	0.80374	0.80972	0.81170	0.81812	0.82504	0.83269
307	0.79142	0.80424	0.81020	0.81217	0.81856	0.82546	0.83309
308	0.79189	0.80471	0.81064	0.81262	0.81900	0.82589	0.83349
309	0.79240	0.80520	0.81111	0.81308	0.81945	0.82632	0.83390
310	0.79291	0.80566	0.81157	0.81353	0.81988	0.82674	0.83430
311	0.79344	0.80616	0.81202	0.81398	0.82033	0.82716	0.83470
312	0.79395	0.80662	0.81248	0.81444	0.82077	0.82757	0.83510
313	0.79443	0.80709	0.81291	0.81488	0.82120	0.82799	0.83549
314	0.79494	0.80756	0.81337	0.81531	0.82163	0.82839	0.83588
315	0.79543	0.80804	0.81382	0.81576	0.82206	0.82881	0.83627
316	0.79595	0.80848	0.81426	0.81619	0.82248	0.82921	0.83666
317	0.79647	0.80894	0.81470	0.81664	0.82291	0.82962	0.83705
318	0.79694	0.80940	0.81515	0.81706	0.82333	0.83003	0.83743
319	0.79742	0.80987	0.81559	0.81750	0.82374	0.83043	0.83781
320	0.79790	0.81031	0.81602	0.81794	0.82415	0.83083	0.83819
321	0.79838	0.81077	0.81645	0.81837	0.82457	0.83123	0.83857
322	0.79887	0.81120	0.81688	0.81880	0.82498	0.83163	0.83894
323	0.79934	0.81166	0.81732	0.81923	0.82540	0.83202	0.83932
324	0.79983	0.81208	0.81775	0.81965	0.82580	0.83241	0.83968
325	0.80033	0.81252	0.81818	0.82007	0.82621	0.83279	0.84006
326	0.80081	0.81297	0.81860	0.82049	0.82661	0.83318	0.84042
327	0.80128	0.81340	0.81903	0.82090	0.82701	0.83357	0.84078
328	0.80174	0.81383	0.81945	0.82132	0.82741	0.83394	0.84115
329	0.80221	0.81425	0.81987	0.82174	0.82780	0.83433	0.84151
330	0.80264	0.81467	0.82028	0.82215	0.82820	0.83470	0.84187
331	0.80311	0.81509	0.82069	0.82256	0.82860	0.83508	0.84223

N	0.1%	0.5%	1%	1.25%	2.5%	5%	10%
332	0.80356	0.81551	0.82110	0.82296	0.82899	0.83546	0.84258
333	0.80402	0.81593	0.82151	0.82336	0.82938	0.83584	0.84294
334	0.80445	0.81636	0.82193	0.82377	0.82976	0.83621	0.84329
335	0.80492	0.81678	0.82233	0.82417	0.83015	0.83658	0.84364
336	0.80538	0.81720	0.82273	0.82457	0.83054	0.83694	0.84400
337	0.80580	0.81762	0.82313	0.82497	0.83092	0.83731	0.84434
338	0.80623	0.81802	0.82353	0.82537	0.83129	0.83767	0.84469
339	0.80668	0.81843	0.82393	0.82576	0.83167	0.83803	0.84503
340	0.80709	0.81885	0.82432	0.82615	0.83204	0.83839	0.84537
341	0.80752	0.81926	0.82472	0.82655	0.83242	0.83875	0.84571
342	0.80794	0.81965	0.82512	0.82693	0.83279	0.83910	0.84606
343	0.80840	0.82006	0.82551	0.82732	0.83317	0.83946	0.84639
344	0.80884	0.82048	0.82589	0.82770	0.83353	0.83981	0.84673
345	0.80928	0.82088	0.82628	0.82808	0.83390	0.84016	0.84706
346	0.80969	0.82128	0.82666	0.82846	0.83426	0.84052	0.84739
347	0.81009	0.82166	0.82704	0.82883	0.83462	0.84086	0.84772
348	0.81054	0.82205	0.82741	0.82921	0.83499	0.84121	0.84805
349	0.81094	0.82244	0.82779	0.82959	0.83535	0.84156	0.84838
350	0.81134	0.82284	0.82817	0.82995	0.83571	0.84190	0.84871
351	0.81177	0.82324	0.82854	0.83032	0.83606	0.84224	0.84904
352	0.81218	0.82363	0.82891	0.83069	0.83642	0.84258	0.84936
353	0.81259	0.82400	0.82928	0.83105	0.83677	0.84292	0.84969
354	0.81298	0.82440	0.82965	0.83142	0.83712	0.84326	0.85000
355	0.81338	0.82477	0.83003	0.83178	0.83747	0.84359	0.85032
356	0.81379	0.82516	0.83039	0.83213	0.83783	0.84392	0.85064
357	0.81419	0.82553	0.83076	0.83250	0.83817	0.84426	0.85096
358	0.81457	0.82591	0.83113	0.83286	0.83852	0.84459	0.85127
359	0.81498	0.82626	0.83149	0.83321	0.83886	0.84492	0.85158
360	0.81538	0.82663	0.83184	0.83356	0.83920	0.84525	0.85189
361	0.81576	0.82700	0.83219	0.83392	0.83954	0.84558	0.85220
362	0.81613	0.82737	0.83255	0.83428	0.83988	0.84590	0.85251
363	0.81654	0.82775	0.83291	0.83463	0.84022	0.84622	0.85282
364	0.81692	0.82810	0.83326	0.83498	0.84056	0.84654	0.85312
365	0.81730	0.82845	0.83360	0.83532	0.84089	0.84686	0.85343
366	0.81768	0.82883	0.83395	0.83566	0.84122	0.84718	0.85373
367	0.81806	0.82919	0.83430	0.83602	0.84155	0.84750	0.85403
368	0.81844	0.82955	0.83464	0.83636	0.84188	0.84781	0.85433
369	0.81881	0.82990	0.83498	0.83669	0.84220	0.84813	0.85463
370	0.81920	0.83026	0.83533	0.83703	0.84253	0.84844	0.85493
371	0.81954	0.83061	0.83567	0.83737	0.84285	0.84875	0.85523
372	0.81992	0.83096	0.83601	0.83771	0.84318	0.84906	0.85553
373	0.82031	0.83131	0.83635	0.83804	0.84350	0.84937	0.85582
374	0.82070	0.83165	0.83669	0.83838	0.84382	0.84968	0.85611
375	0.82106	0.83200	0.83703	0.83871	0.84413	0.84999	0.85640
376	0.82143	0.83235	0.83736	0.83904	0.84445	0.85029	0.85669
377	0.82182	0.83270	0.83770	0.83937	0.84477	0.85060	0.85698
378	0.82217	0.83304	0.83802	0.83970	0.84508	0.85090	0.85727
379	0.82253	0.83337	0.83835	0.84002	0.84539	0.85120	0.85756
380	0.82288	0.83371	0.83868	0.84034	0.84571	0.85149	0.85785
381	0.82324	0.83405	0.83902	0.84066	0.84602	0.85179	0.85813
382	0.82361	0.83439	0.83934	0.84099	0.84633	0.85208	0.85841
383	0.82395	0.83474	0.83967	0.84131	0.84664	0.85238	0.85869
384	0.82428	0.83507	0.83998	0.84162	0.84694	0.85267	0.85897
385	0.82463	0.83539	0.84030	0.84195	0.84725	0.85296	0.85925
386	0.82501	0.83572	0.84062	0.84226	0.84755	0.85326	0.85953
387	0.82537	0.83605	0.84094	0.84257	0.84785	0.85354	0.85980
388	0.82570	0.83638	0.84125	0.84289	0.84815	0.85383	0.86008
389	0.82605	0.83670	0.84157	0.84319	0.84845	0.85412	0.86035
390	0.82638	0.83702	0.84189	0.84350	0.84875	0.85440	0.86062
391	0.82674	0.83734	0.84221	0.84381	0.84905	0.85469	0.86089
392	0.82710	0.83767	0.84251	0.84412	0.84934	0.85497	0.86115
393	0.82743	0.83800	0.84282	0.84443	0.84963	0.85526	0.86142
394	0.82777	0.83831	0.84313	0.84473	0.84992	0.85554	0.86169
395	0.82810	0.83862	0.84345	0.84504	0.85021	0.85582	0.86196
396	0.82846	0.83894	0.84375	0.84534	0.85051	0.85609	0.86222
397	0.82879	0.83925	0.84405	0.84564	0.85080	0.85637	0.86249
398	0.82911	0.83956	0.84434	0.84594	0.85108	0.85665	0.86276
399	0.82945	0.83987	0.84465	0.84624	0.85137	0.85692	0.86302
400	0.82982	0.84017	0.84496	0.84654	0.85166	0.85720	0.86328
401	0.83014	0.84049	0.84525	0.84683	0.85195	0.85747	0.86354
402	0.83046	0.84081	0.84554	0.84713	0.85223	0.85774	0.86380
403	0.83080	0.84111	0.84584	0.84742	0.85251	0.85802	0.86406
404	0.83112	0.84141	0.84614	0.84772	0.85280	0.85829	0.86431
405	0.83144	0.84172	0.84644	0.84802	0.85308	0.85855	0.86457

N	0.1%	0.5%	1%	1.25%	2.5%	5%	10%
406	0.83175	0.84204	0.84673	0.84830	0.85336	0.85882	0.86483
407	0.83206	0.84233	0.84702	0.84859	0.85364	0.85909	0.86508
408	0.83237	0.84264	0.84732	0.84888	0.85392	0.85935	0.86533
409	0.83273	0.84293	0.84761	0.84916	0.85419	0.85962	0.86558
410	0.83301	0.84322	0.84789	0.84944	0.85446	0.85988	0.86583
411	0.83335	0.84351	0.84817	0.84972	0.85473	0.86014	0.86608
412	0.83369	0.84380	0.84846	0.85000	0.85501	0.86040	0.86633
413	0.83400	0.84410	0.84874	0.85029	0.85528	0.86066	0.86658
414	0.83433	0.84440	0.84902	0.85057	0.85555	0.86092	0.86682
415	0.83465	0.84469	0.84931	0.85084	0.85582	0.86117	0.86707
416	0.83496	0.84497	0.84958	0.85112	0.85608	0.86143	0.86731
417	0.83525	0.84527	0.84986	0.85140	0.85635	0.86169	0.86755
418	0.83555	0.84555	0.85015	0.85169	0.85661	0.86194	0.86780
419	0.83588	0.84584	0.85042	0.85196	0.85689	0.86219	0.86804
420	0.83618	0.84614	0.85071	0.85224	0.85715	0.86245	0.86828
421	0.83648	0.84641	0.85097	0.85252	0.85741	0.86270	0.86852
422	0.83678	0.84669	0.85125	0.85278	0.85767	0.86295	0.86876
423	0.83710	0.84698	0.85152	0.85305	0.85793	0.86320	0.86900
424	0.83741	0.84726	0.85179	0.85331	0.85820	0.86344	0.86923
425	0.83769	0.84752	0.85207	0.85358	0.85845	0.86369	0.86947
426	0.83800	0.84781	0.85234	0.85385	0.85871	0.86393	0.86970
427	0.83831	0.84808	0.85261	0.85411	0.85896	0.86418	0.86994
428	0.83859	0.84836	0.85287	0.85438	0.85921	0.86442	0.87017
429	0.83890	0.84864	0.85314	0.85464	0.85947	0.86466	0.87040
430	0.83921	0.84891	0.85339	0.85490	0.85972	0.86491	0.87063
431	0.83950	0.84919	0.85366	0.85517	0.85997	0.86515	0.87086
432	0.83979	0.84947	0.85393	0.85543	0.86022	0.86539	0.87109
433	0.84007	0.84974	0.85420	0.85569	0.86047	0.86563	0.87132
434	0.84036	0.85000	0.85445	0.85594	0.86072	0.86587	0.87154
435	0.84066	0.85026	0.85471	0.85620	0.86097	0.86611	0.87177
436	0.84093	0.85054	0.85497	0.85646	0.86121	0.86635	0.87199
437	0.84124	0.85080	0.85524	0.85672	0.86146	0.86658	0.87222
438	0.84156	0.85106	0.85550	0.85696	0.86170	0.86681	0.87244
439	0.84184	0.85132	0.85575	0.85722	0.86195	0.86705	0.87267
440	0.84209	0.85158	0.85600	0.85748	0.86219	0.86728	0.87289
441	0.84236	0.85185	0.85626	0.85772	0.86243	0.86751	0.87311
442	0.84263	0.85210	0.85651	0.85797	0.86267	0.86774	0.87333
443	0.84291	0.85237	0.85677	0.85822	0.86291	0.86798	0.87355
444	0.84319	0.85263	0.85702	0.85847	0.86315	0.86820	0.87377
445	0.84346	0.85288	0.85727	0.85872	0.86339	0.86843	0.87398
446	0.84373	0.85315	0.85752	0.85897	0.86363	0.86866	0.87420
447	0.84401	0.85340	0.85778	0.85921	0.86387	0.86888	0.87441
448	0.84429	0.85365	0.85802	0.85946	0.86411	0.86911	0.87463
449	0.84456	0.85390	0.85826	0.85970	0.86434	0.86933	0.87484
450	0.84482	0.85416	0.85851	0.85994	0.86458	0.86956	0.87506
451	0.84509	0.85441	0.85876	0.86018	0.86481	0.86978	0.87527
452	0.84535	0.85466	0.85900	0.86043	0.86504	0.87000	0.87548
453	0.84562	0.85491	0.85924	0.86067	0.86527	0.87023	0.87569
454	0.84590	0.85517	0.85948	0.86091	0.86550	0.87045	0.87590
455	0.84617	0.85541	0.85971	0.86114	0.86573	0.87067	0.87611
456	0.84644	0.85565	0.85995	0.86138	0.86596	0.87089	0.87632
457	0.84671	0.85590	0.86019	0.86162	0.86619	0.87110	0.87653
458	0.84698	0.85615	0.86043	0.86185	0.86641	0.87132	0.87674
459	0.84724	0.85639	0.86066	0.86208	0.86664	0.87154	0.87694
460	0.84751	0.85662	0.86090	0.86232	0.86686	0.87175	0.87715
461	0.84779	0.85687	0.86114	0.86255	0.86709	0.87197	0.87735
462	0.84805	0.85711	0.86138	0.86278	0.86731	0.87218	0.87756
463	0.84830	0.85736	0.86161	0.86302	0.86753	0.87240	0.87776
464	0.84855	0.85759	0.86184	0.86325	0.86776	0.87261	0.87796
465	0.84882	0.85784	0.86207	0.86348	0.86797	0.87282	0.87817
466	0.84909	0.85808	0.86230	0.86371	0.86820	0.87303	0.87837
467	0.84933	0.85832	0.86253	0.86393	0.86841	0.87324	0.87857
468	0.84957	0.85855	0.86277	0.86416	0.86863	0.87346	0.87876
469	0.84983	0.85879	0.86300	0.86439	0.86885	0.87366	0.87897
470	0.85009	0.85902	0.86323	0.86461	0.86907	0.87387	0.87917
471	0.85034	0.85927	0.86345	0.86484	0.86929	0.87407	0.87937
472	0.85059	0.85950	0.86367	0.86507	0.86950	0.87428	0.87956
473	0.85082	0.85973	0.86389	0.86528	0.86972	0.87449	0.87976
474	0.85106	0.85996	0.86412	0.86551	0.86993	0.87470	0.87996
475	0.85130	0.86019	0.86433	0.86573	0.87015	0.87490	0.88015
476	0.85155	0.86042	0.86456	0.86595	0.87036	0.87510	0.88034
477	0.85182	0.86064	0.86478	0.86616	0.87058	0.87530	0.88054
478	0.85208	0.86087	0.86500	0.86638	0.87078	0.87551	0.88073
479	0.85231	0.86112	0.86522	0.86661	0.87099	0.87571	0.88092

N	0.1%	0.5%	1%	1.25%	2.5%	5%	10%
480	0.85256	0.86135	0.86544	0.86683	0.87121	0.87591	0.88112
481	0.85280	0.86157	0.86566	0.86705	0.87142	0.87611	0.88131
482	0.85303	0.86180	0.86588	0.86726	0.87162	0.87631	0.88149
483	0.85329	0.86203	0.86610	0.86747	0.87183	0.87651	0.88168
484	0.85350	0.86225	0.86632	0.86768	0.87204	0.87671	0.88187
485	0.85376	0.86247	0.86653	0.86789	0.87224	0.87690	0.88206
486	0.85398	0.86269	0.86675	0.86811	0.87245	0.87710	0.88225
487	0.85424	0.86291	0.86696	0.86832	0.87266	0.87730	0.88243
488	0.85446	0.86314	0.86717	0.86853	0.87286	0.87750	0.88262
489	0.85469	0.86336	0.86739	0.86874	0.87306	0.87769	0.88280
490	0.85494	0.86357	0.86760	0.86895	0.87326	0.87788	0.88299
491	0.85517	0.86379	0.86782	0.86916	0.87346	0.87808	0.88317
492	0.85539	0.86401	0.86802	0.86936	0.87366	0.87827	0.88335
493	0.85562	0.86422	0.86822	0.86957	0.87386	0.87846	0.88354
494	0.85585	0.86444	0.86844	0.86977	0.87406	0.87865	0.88372
495	0.85608	0.86467	0.86864	0.86997	0.87426	0.87885	0.88390
496	0.85633	0.86489	0.86885	0.87018	0.87446	0.87903	0.88408
497	0.85654	0.86510	0.86906	0.87039	0.87466	0.87923	0.88426
498	0.85678	0.86532	0.86927	0.87060	0.87485	0.87941	0.88444
499	0.85701	0.86553	0.86948	0.87080	0.87505	0.87960	0.88462
500	0.85725	0.86575	0.86969	0.87101	0.87525	0.87979	0.88480
501	0.85748	0.86595	0.86989	0.87121	0.87544	0.87998	0.88498
502	0.85771	0.86615	0.87010	0.87141	0.87564	0.88016	0.88516
503	0.85795	0.86635	0.87030	0.87161	0.87583	0.88035	0.88533
504	0.85817	0.86657	0.87050	0.87181	0.87603	0.88053	0.88551
505	0.85837	0.86678	0.87070	0.87202	0.87622	0.88072	0.88568
506	0.85859	0.86698	0.87090	0.87222	0.87641	0.88090	0.88586
507	0.85883	0.86720	0.87111	0.87241	0.87660	0.88109	0.88603
508	0.85907	0.86740	0.87131	0.87261	0.87679	0.88127	0.88621
509	0.85927	0.86761	0.87151	0.87280	0.87698	0.88145	0.88638
510	0.85949	0.86781	0.87170	0.87300	0.87717	0.88163	0.88655
511	0.85970	0.86801	0.87189	0.87319	0.87736	0.88181	0.88673
512	0.85993	0.86822	0.87210	0.87338	0.87754	0.88199	0.88690
513	0.86016	0.86843	0.87230	0.87358	0.87773	0.88217	0.88707
514	0.86036	0.86863	0.87250	0.87377	0.87792	0.88234	0.88724
515	0.86058	0.86882	0.87268	0.87397	0.87810	0.88252	0.88741
516	0.86080	0.86902	0.87287	0.87415	0.87829	0.88270	0.88757
517	0.86100	0.86923	0.87308	0.87435	0.87848	0.88288	0.88774
518	0.86120	0.86943	0.87327	0.87454	0.87866	0.88306	0.88791
519	0.86141	0.86963	0.87346	0.87473	0.87884	0.88323	0.88808
520	0.86161	0.86983	0.87365	0.87492	0.87902	0.88340	0.88825
521	0.86183	0.87003	0.87384	0.87512	0.87920	0.88358	0.88841
522	0.86204	0.87023	0.87403	0.87531	0.87938	0.88376	0.88858
523	0.86226	0.87042	0.87422	0.87549	0.87956	0.88393	0.88875
524	0.86247	0.87062	0.87442	0.87568	0.87974	0.88410	0.88891
525	0.86267	0.87081	0.87461	0.87586	0.87992	0.88428	0.88907
526	0.86287	0.87100	0.87480	0.87605	0.88010	0.88445	0.88924
527	0.86310	0.87119	0.87497	0.87623	0.88028	0.88462	0.88940
528	0.86329	0.87139	0.87516	0.87641	0.88046	0.88479	0.88957
529	0.86350	0.87158	0.87534	0.87659	0.88063	0.88496	0.88973
530	0.86370	0.87178	0.87553	0.87677	0.88081	0.88513	0.88989
531	0.86391	0.87197	0.87572	0.87695	0.88098	0.88530	0.89005
532	0.86412	0.87217	0.87590	0.87714	0.88116	0.88547	0.89021
533	0.86432	0.87236	0.87609	0.87732	0.88134	0.88563	0.89037
534	0.86454	0.87255	0.87626	0.87749	0.88151	0.88580	0.89053
535	0.86473	0.87274	0.87645	0.87768	0.88169	0.88597	0.89069
536	0.86492	0.87293	0.87663	0.87786	0.88186	0.88613	0.89085
537	0.86512	0.87311	0.87681	0.87804	0.88204	0.88630	0.89100
538	0.86532	0.87330	0.87699	0.87822	0.88220	0.88646	0.89116
539	0.86549	0.87350	0.87717	0.87840	0.88238	0.88663	0.89132
540	0.86571	0.87368	0.87736	0.87858	0.88255	0.88679	0.89147
541	0.86590	0.87386	0.87754	0.87875	0.88272	0.88696	0.89163
542	0.86610	0.87405	0.87772	0.87893	0.88289	0.88712	0.89178
543	0.86629	0.87423	0.87789	0.87910	0.88306	0.88728	0.89194
544	0.86649	0.87441	0.87807	0.87927	0.88323	0.88744	0.89210
545	0.86668	0.87460	0.87824	0.87945	0.88340	0.88761	0.89225
546	0.86686	0.87478	0.87842	0.87962	0.88356	0.88777	0.89240
547	0.86706	0.87496	0.87859	0.87979	0.88373	0.88793	0.89256
548	0.86725	0.87514	0.87877	0.87996	0.88389	0.88809	0.89271
549	0.86744	0.87531	0.87895	0.88014	0.88406	0.88825	0.89286
550	0.86763	0.87549	0.87913	0.88031	0.88422	0.88841	0.89301
551	0.86783	0.87568	0.87929	0.88048	0.88439	0.88857	0.89317
552	0.86802	0.87585	0.87947	0.88065	0.88456	0.88873	0.89332
553	0.86821	0.87603	0.87964	0.88083	0.88472	0.88888	0.89347

N	0.1%	0.5%	1%	1.25%	2.5%	5%	10%
554	0.86840	0.87620	0.87982	0.88100	0.88489	0.88904	0.89361
555	0.86858	0.87638	0.87998	0.88117	0.88505	0.88920	0.89377
556	0.86875	0.87656	0.88015	0.88134	0.88521	0.88935	0.89392
557	0.86895	0.87673	0.88032	0.88151	0.88537	0.88951	0.89406
558	0.86914	0.87691	0.88050	0.88168	0.88553	0.88966	0.89421
559	0.86932	0.87709	0.88066	0.88184	0.88569	0.88982	0.89436
560	0.86951	0.87726	0.88083	0.88201	0.88585	0.88997	0.89451
561	0.86971	0.87744	0.88100	0.88218	0.88601	0.89013	0.89465
562	0.86989	0.87761	0.88117	0.88234	0.88617	0.89028	0.89480
563	0.87009	0.87779	0.88134	0.88251	0.88633	0.89043	0.89494
564	0.87026	0.87796	0.88150	0.88268	0.88649	0.89058	0.89509
565	0.87044	0.87814	0.88167	0.88284	0.88665	0.89074	0.89523
566	0.87061	0.87831	0.88184	0.88301	0.88681	0.89089	0.89538
567	0.87080	0.87849	0.88200	0.88317	0.88696	0.89104	0.89552
568	0.87100	0.87865	0.88217	0.88333	0.88712	0.89119	0.89567
569	0.87118	0.87882	0.88233	0.88349	0.88728	0.89134	0.89581
570	0.87135	0.87899	0.88250	0.88365	0.88743	0.89149	0.89595
571	0.87152	0.87916	0.88266	0.88382	0.88759	0.89164	0.89609
572	0.87171	0.87934	0.88282	0.88398	0.88774	0.89179	0.89623
573	0.87188	0.87950	0.88299	0.88414	0.88790	0.89194	0.89637
574	0.87207	0.87967	0.88315	0.88430	0.88805	0.89208	0.89651
575	0.87224	0.87983	0.88331	0.88446	0.88821	0.89223	0.89665
576	0.87241	0.87999	0.88347	0.88461	0.88836	0.89238	0.89680
577	0.87257	0.88016	0.88363	0.88478	0.88851	0.89252	0.89694
578	0.87277	0.88033	0.88379	0.88493	0.88866	0.89267	0.89707
579	0.87292	0.88050	0.88395	0.88509	0.88881	0.89282	0.89721
580	0.87311	0.88066	0.88411	0.88525	0.88896	0.89296	0.89735
581	0.87330	0.88083	0.88427	0.88541	0.88912	0.89311	0.89749
582	0.87348	0.88098	0.88442	0.88556	0.88927	0.89325	0.89763
583	0.87365	0.88115	0.88458	0.88572	0.88942	0.89339	0.89777
584	0.87382	0.88131	0.88474	0.88587	0.88957	0.89354	0.89790
585	0.87399	0.88147	0.88490	0.88602	0.88971	0.89368	0.89804
586	0.87414	0.88163	0.88505	0.88618	0.88986	0.89382	0.89818
587	0.87431	0.88179	0.88521	0.88634	0.89001	0.89397	0.89831
588	0.87449	0.88195	0.88537	0.88649	0.89016	0.89411	0.89845
589	0.87467	0.88211	0.88552	0.88665	0.89030	0.89424	0.89858
590	0.87483	0.88227	0.88567	0.88680	0.89045	0.89439	0.89871
591	0.87502	0.88243	0.88583	0.88696	0.89060	0.89453	0.89885
592	0.87519	0.88259	0.88599	0.88711	0.89074	0.89467	0.89898
593	0.87535	0.88275	0.88615	0.88725	0.89089	0.89481	0.89912
594	0.87552	0.88290	0.88629	0.88741	0.89104	0.89494	0.89925
595	0.87568	0.88306	0.88644	0.88756	0.89118	0.89508	0.89938
596	0.87584	0.88322	0.88660	0.88772	0.89132	0.89522	0.89951
597	0.87603	0.88338	0.88675	0.88786	0.89147	0.89536	0.89964
598	0.87619	0.88353	0.88690	0.88801	0.89161	0.89549	0.89977
599	0.87636	0.88369	0.88705	0.88816	0.89175	0.89563	0.89991
600	0.87653	0.88384	0.88720	0.88830	0.89189	0.89577	0.90004
601	0.87668	0.88400	0.88735	0.88844	0.89203	0.89591	0.90017
602	0.87685	0.88415	0.88750	0.88860	0.89218	0.89604	0.90030
603	0.87700	0.88430	0.88764	0.88874	0.89232	0.89618	0.90043
604	0.87715	0.88446	0.88779	0.88888	0.89246	0.89632	0.90055
605	0.87730	0.88461	0.88794	0.88903	0.89260	0.89645	0.90068
606	0.87746	0.88476	0.88808	0.88918	0.89274	0.89658	0.90081
607	0.87762	0.88490	0.88823	0.88932	0.89288	0.89672	0.90094
608	0.87779	0.88506	0.88837	0.88947	0.89302	0.89685	0.90107
609	0.87795	0.88521	0.88851	0.88961	0.89316	0.89698	0.90119
610	0.87811	0.88536	0.88866	0.88976	0.89329	0.89712	0.90132
611	0.87827	0.88551	0.88880	0.88989	0.89343	0.89725	0.90145
612	0.87843	0.88566	0.88896	0.89004	0.89357	0.89738	0.90157
613	0.87858	0.88581	0.88910	0.89018	0.89371	0.89751	0.90170
614	0.87874	0.88596	0.88924	0.89032	0.89385	0.89765	0.90182
615	0.87890	0.88610	0.88939	0.89047	0.89399	0.89778	0.90195
616	0.87907	0.88625	0.88953	0.89061	0.89412	0.89791	0.90208
617	0.87923	0.88641	0.88967	0.89075	0.89426	0.89804	0.90220
618	0.87940	0.88655	0.88981	0.89089	0.89440	0.89817	0.90232
619	0.87956	0.88669	0.88995	0.89104	0.89453	0.89830	0.90245
620	0.87972	0.88683	0.89010	0.89117	0.89467	0.89843	0.90257
621	0.87987	0.88698	0.89024	0.89132	0.89480	0.89856	0.90269
622	0.88004	0.88712	0.89038	0.89145	0.89494	0.89869	0.90282
623	0.88018	0.88727	0.89052	0.89159	0.89507	0.89882	0.90294
624	0.88034	0.88741	0.89066	0.89173	0.89521	0.89895	0.90306
625	0.88049	0.88756	0.89080	0.89187	0.89534	0.89908	0.90318
626	0.88064	0.88770	0.89093	0.89200	0.89547	0.89920	0.90330
627	0.88080	0.88785	0.89107	0.89214	0.89561	0.89933	0.90343

N	0.1%	0.5%	1%	1.25%	2.5%	5%	10%
628	0.88096	0.88799	0.89121	0.89228	0.89573	0.89946	0.90355
629	0.88112	0.88813	0.89135	0.89241	0.89587	0.89958	0.90367
630	0.88127	0.88827	0.89149	0.89255	0.89600	0.89971	0.90379
631	0.88143	0.88841	0.89162	0.89268	0.89613	0.89983	0.90391
632	0.88158	0.88856	0.89176	0.89282	0.89626	0.89996	0.90402
633	0.88173	0.88869	0.89189	0.89295	0.89639	0.90008	0.90414
634	0.88187	0.88883	0.89203	0.89308	0.89652	0.90021	0.90426
635	0.88202	0.88897	0.89216	0.89321	0.89665	0.90033	0.90438
636	0.88218	0.88912	0.89230	0.89335	0.89678	0.90046	0.90450
637	0.88231	0.88925	0.89243	0.89348	0.89690	0.90058	0.90462
638	0.88246	0.88939	0.89257	0.89361	0.89703	0.90070	0.90474
639	0.88260	0.88954	0.89270	0.89375	0.89716	0.90082	0.90485
640	0.88275	0.88967	0.89284	0.89388	0.89729	0.90095	0.90497
641	0.88290	0.88980	0.89297	0.89401	0.89741	0.90107	0.90509
642	0.88303	0.88994	0.89310	0.89415	0.89754	0.90119	0.90520
643	0.88317	0.89008	0.89323	0.89428	0.89767	0.90131	0.90532
644	0.88332	0.89023	0.89336	0.89441	0.89779	0.90143	0.90543
645	0.88346	0.89036	0.89349	0.89454	0.89792	0.90155	0.90555
646	0.88362	0.89049	0.89363	0.89467	0.89804	0.90167	0.90566
647	0.88376	0.89063	0.89376	0.89479	0.89817	0.90179	0.90578
648	0.88389	0.89076	0.89389	0.89492	0.89829	0.90191	0.90589
649	0.88404	0.89090	0.89402	0.89505	0.89841	0.90203	0.90600
650	0.88420	0.89104	0.89415	0.89518	0.89854	0.90215	0.90612
651	0.88433	0.89117	0.89428	0.89532	0.89866	0.90227	0.90623
652	0.88449	0.89130	0.89441	0.89545	0.89879	0.90239	0.90634
653	0.88464	0.89144	0.89454	0.89557	0.89891	0.90251	0.90646
654	0.88479	0.89156	0.89466	0.89570	0.89903	0.90263	0.90657
655	0.88493	0.89170	0.89479	0.89582	0.89916	0.90275	0.90668
656	0.88508	0.89183	0.89492	0.89595	0.89927	0.90286	0.90679
657	0.88522	0.89196	0.89505	0.89608	0.89940	0.90298	0.90691
658	0.88535	0.89210	0.89518	0.89621	0.89952	0.90310	0.90702
659	0.88550	0.89222	0.89531	0.89633	0.89964	0.90321	0.90713
660	0.88564	0.89236	0.89543	0.89646	0.89976	0.90333	0.90724
661	0.88576	0.89248	0.89556	0.89658	0.89988	0.90344	0.90735
662	0.88591	0.89261	0.89569	0.89671	0.90000	0.90356	0.90746
663	0.88604	0.89274	0.89581	0.89683	0.90012	0.90367	0.90757
664	0.88618	0.89287	0.89594	0.89695	0.90024	0.90378	0.90768
665	0.88632	0.89300	0.89606	0.89707	0.90036	0.90390	0.90779
666	0.88646	0.89313	0.89618	0.89720	0.90048	0.90401	0.90790
667	0.88660	0.89326	0.89631	0.89732	0.90060	0.90413	0.90800
668	0.88673	0.89339	0.89643	0.89744	0.90072	0.90424	0.90811
669	0.88687	0.89351	0.89655	0.89756	0.90083	0.90435	0.90822
670	0.88701	0.89365	0.89668	0.89769	0.90095	0.90446	0.90833
671	0.88715	0.89377	0.89680	0.89781	0.90107	0.90458	0.90844
672	0.88729	0.89390	0.89693	0.89793	0.90118	0.90469	0.90854
673	0.88743	0.89402	0.89705	0.89805	0.90130	0.90480	0.90865
674	0.88756	0.89415	0.89717	0.89817	0.90141	0.90492	0.90876
675	0.88769	0.89428	0.89729	0.89830	0.90153	0.90503	0.90886
676	0.88781	0.89440	0.89741	0.89842	0.90164	0.90514	0.90897
677	0.88794	0.89453	0.89753	0.89854	0.90176	0.90525	0.90908
678	0.88808	0.89465	0.89766	0.89866	0.90187	0.90536	0.90918
679	0.88821	0.89478	0.89778	0.89878	0.90199	0.90547	0.90929
680	0.88833	0.89490	0.89790	0.89889	0.90210	0.90558	0.90939
681	0.88847	0.89503	0.89802	0.89902	0.90221	0.90569	0.90950
682	0.88861	0.89515	0.89814	0.89913	0.90233	0.90580	0.90960
683	0.88874	0.89527	0.89826	0.89925	0.90245	0.90591	0.90971
684	0.88887	0.89540	0.89837	0.89937	0.90256	0.90602	0.90981
685	0.88901	0.89552	0.89849	0.89948	0.90268	0.90613	0.90992
686	0.88915	0.89564	0.89861	0.89960	0.90279	0.90623	0.91002
687	0.88928	0.89576	0.89873	0.89972	0.90290	0.90634	0.91012
688	0.88940	0.89588	0.89884	0.89984	0.90302	0.90645	0.91022
689	0.88953	0.89600	0.89896	0.89995	0.90313	0.90656	0.91033
690	0.88967	0.89612	0.89908	0.90007	0.90324	0.90666	0.91043
691	0.88980	0.89624	0.89920	0.90018	0.90335	0.90677	0.91053
692	0.88992	0.89636	0.89932	0.90030	0.90346	0.90688	0.91063
693	0.89006	0.89648	0.89943	0.90041	0.90357	0.90699	0.91073
694	0.89018	0.89660	0.89955	0.90053	0.90369	0.90709	0.91084
695	0.89032	0.89672	0.89967	0.90064	0.90380	0.90720	0.91094
696	0.89045	0.89683	0.89978	0.90076	0.90391	0.90730	0.91104
697	0.89058	0.89695	0.89990	0.90087	0.90402	0.90741	0.91114
698	0.89071	0.89707	0.90001	0.90099	0.90413	0.90751	0.91124
699	0.89085	0.89719	0.90013	0.90110	0.90423	0.90762	0.91134
700	0.89097	0.89730	0.90024	0.90121	0.90434	0.90772	0.91144
701	0.89110	0.89742	0.90035	0.90133	0.90445	0.90783	0.91154

N	0.1%	0.5%	1%	1.25%	2.5%	5%	10%
702	0.89123	0.89754	0.90047	0.90144	0.90456	0.90793	0.91164
703	0.89135	0.89766	0.90058	0.90155	0.90467	0.90804	0.91174
704	0.89148	0.89778	0.90069	0.90167	0.90478	0.90814	0.91184
705	0.89160	0.89790	0.90081	0.90178	0.90489	0.90824	0.91194
706	0.89173	0.89802	0.90092	0.90189	0.90499	0.90835	0.91203
707	0.89186	0.89813	0.90104	0.90200	0.90510	0.90845	0.91213
708	0.89198	0.89825	0.90115	0.90211	0.90521	0.90856	0.91223
709	0.89210	0.89836	0.90127	0.90222	0.90532	0.90866	0.91233
710	0.89221	0.89847	0.90138	0.90234	0.90542	0.90876	0.91242
711	0.89235	0.89859	0.90148	0.90244	0.90553	0.90886	0.91252
712	0.89247	0.89870	0.90159	0.90255	0.90564	0.90896	0.91262
713	0.89258	0.89882	0.90170	0.90266	0.90574	0.90906	0.91271
714	0.89270	0.89893	0.90181	0.90277	0.90584	0.90916	0.91281
715	0.89282	0.89904	0.90192	0.90288	0.90595	0.90927	0.91291
716	0.89294	0.89916	0.90203	0.90298	0.90606	0.90937	0.91300
717	0.89307	0.89927	0.90214	0.90309	0.90616	0.90947	0.91310
718	0.89319	0.89938	0.90225	0.90320	0.90626	0.90956	0.91320
719	0.89332	0.89949	0.90236	0.90331	0.90637	0.90966	0.91329
720	0.89344	0.89960	0.90247	0.90342	0.90647	0.90976	0.91339
721	0.89356	0.89972	0.90258	0.90352	0.90658	0.90987	0.91348
722	0.89368	0.89983	0.90269	0.90363	0.90668	0.90996	0.91358
723	0.89380	0.89994	0.90279	0.90374	0.90678	0.91006	0.91367
724	0.89391	0.90005	0.90290	0.90385	0.90688	0.91016	0.91376
725	0.89403	0.90017	0.90301	0.90395	0.90699	0.91026	0.91386
726	0.89414	0.90028	0.90311	0.90406	0.90709	0.91036	0.91395
727	0.89426	0.90039	0.90322	0.90416	0.90719	0.91046	0.91404
728	0.89438	0.90050	0.90333	0.90427	0.90729	0.91055	0.91414
729	0.89450	0.90061	0.90344	0.90437	0.90739	0.91065	0.91423
730	0.89462	0.90072	0.90355	0.90448	0.90750	0.91075	0.91432
731	0.89473	0.90084	0.90365	0.90459	0.90760	0.91085	0.91442
732	0.89484	0.90095	0.90376	0.90469	0.90770	0.91095	0.91451
733	0.89496	0.90106	0.90386	0.90479	0.90780	0.91104	0.91460
734	0.89509	0.90117	0.90397	0.90490	0.90790	0.91114	0.91469
735	0.89520	0.90127	0.90407	0.90500	0.90800	0.91124	0.91479
736	0.89531	0.90138	0.90418	0.90510	0.90810	0.91133	0.91488
737	0.89543	0.90149	0.90429	0.90521	0.90820	0.91142	0.91497
738	0.89554	0.90160	0.90439	0.90531	0.90830	0.91152	0.91506
739	0.89566	0.90171	0.90449	0.90542	0.90840	0.91162	0.91515
740	0.89576	0.90182	0.90459	0.90552	0.90850	0.91171	0.91524
741	0.89588	0.90192	0.90470	0.90562	0.90860	0.91180	0.91533
742	0.89600	0.90202	0.90480	0.90572	0.90869	0.91190	0.91542
743	0.89612	0.90213	0.90491	0.90582	0.90879	0.91199	0.91551
744	0.89623	0.90223	0.90501	0.90593	0.90889	0.91208	0.91560
745	0.89635	0.90234	0.90511	0.90603	0.90899	0.91218	0.91569
746	0.89645	0.90245	0.90521	0.90613	0.90909	0.91227	0.91578
747	0.89658	0.90256	0.90532	0.90623	0.90919	0.91237	0.91587
748	0.89670	0.90267	0.90542	0.90634	0.90928	0.91246	0.91596
749	0.89682	0.90277	0.90552	0.90644	0.90938	0.91255	0.91605
750	0.89692	0.90288	0.90562	0.90653	0.90948	0.91265	0.91614
751	0.89702	0.90298	0.90573	0.90663	0.90957	0.91274	0.91623
752	0.89714	0.90309	0.90582	0.90674	0.90967	0.91283	0.91631
753	0.89724	0.90319	0.90592	0.90683	0.90977	0.91293	0.91640
754	0.89735	0.90329	0.90602	0.90693	0.90986	0.91302	0.91649
755	0.89746	0.90340	0.90612	0.90703	0.90996	0.91311	0.91658
756	0.89757	0.90350	0.90622	0.90713	0.91005	0.91320	0.91667
757	0.89768	0.90360	0.90632	0.90723	0.91015	0.91329	0.91675
758	0.89779	0.90370	0.90642	0.90733	0.91024	0.91338	0.91684
759	0.89790	0.90381	0.90652	0.90743	0.91034	0.91348	0.91693
760	0.89801	0.90391	0.90662	0.90752	0.91043	0.91357	0.91701
761	0.89812	0.90401	0.90672	0.90762	0.91052	0.91366	0.91710
762	0.89823	0.90411	0.90682	0.90772	0.91062	0.91375	0.91719
763	0.89833	0.90421	0.90692	0.90781	0.91071	0.91384	0.91727
764	0.89845	0.90431	0.90701	0.90791	0.91081	0.91393	0.91736
765	0.89856	0.90441	0.90711	0.90800	0.91090	0.91402	0.91744
766	0.89866	0.90451	0.90721	0.90810	0.91099	0.91411	0.91753
767	0.89877	0.90460	0.90731	0.90820	0.91108	0.91419	0.91761
768	0.89887	0.90471	0.90740	0.90829	0.91118	0.91428	0.91770
769	0.89897	0.90481	0.90750	0.90839	0.91127	0.91437	0.91778
770	0.89908	0.90490	0.90760	0.90849	0.91136	0.91446	0.91787
771	0.89919	0.90500	0.90769	0.90858	0.91145	0.91455	0.91795
772	0.89930	0.90510	0.90778	0.90868	0.91155	0.91464	0.91804
773	0.89940	0.90520	0.90788	0.90877	0.91164	0.91473	0.91812
774	0.89952	0.90530	0.90798	0.90887	0.91173	0.91481	0.91821
775	0.89961	0.90541	0.90807	0.90896	0.91182	0.91490	0.91829

N	0.1%	0.5%	1%	1.25%	2.5%	5%	10%
776	0.89972	0.90550	0.90817	0.90906	0.91191	0.91499	0.91837
777	0.89984	0.90560	0.90826	0.90915	0.91200	0.91508	0.91846
778	0.89994	0.90570	0.90836	0.90924	0.91209	0.91516	0.91854
779	0.90005	0.90580	0.90845	0.90934	0.91218	0.91525	0.91862
780	0.90015	0.90589	0.90854	0.90943	0.91227	0.91534	0.91870
781	0.90026	0.90599	0.90864	0.90952	0.91236	0.91542	0.91879
782	0.90036	0.90609	0.90874	0.90962	0.91245	0.91551	0.91887
783	0.90045	0.90619	0.90883	0.90971	0.91254	0.91560	0.91895
784	0.90056	0.90629	0.90892	0.90980	0.91263	0.91568	0.91903
785	0.90066	0.90639	0.90902	0.90990	0.91272	0.91577	0.91912
786	0.90075	0.90648	0.90911	0.90999	0.91281	0.91585	0.91920
787	0.90086	0.90658	0.90920	0.91008	0.91290	0.91594	0.91928
788	0.90096	0.90667	0.90929	0.91017	0.91298	0.91602	0.91936
789	0.90106	0.90678	0.90939	0.91027	0.91307	0.91611	0.91944
790	0.90116	0.90687	0.90948	0.91036	0.91316	0.91619	0.91952
791	0.90127	0.90697	0.90957	0.91045	0.91325	0.91628	0.91960
792	0.90137	0.90706	0.90966	0.91054	0.91334	0.91636	0.91968
793	0.90148	0.90716	0.90976	0.91063	0.91343	0.91645	0.91976
794	0.90157	0.90725	0.90985	0.91072	0.91352	0.91653	0.91984
795	0.90167	0.90734	0.90994	0.91081	0.91361	0.91661	0.91992
796	0.90177	0.90744	0.91003	0.91090	0.91369	0.91670	0.92000
797	0.90185	0.90754	0.91012	0.91099	0.91378	0.91678	0.92008
798	0.90196	0.90763	0.91021	0.91108	0.91386	0.91686	0.92016
799	0.90205	0.90772	0.91030	0.91117	0.91395	0.91695	0.92024
800	0.90214	0.90781	0.91039	0.91126	0.91403	0.91703	0.92032
801	0.90225	0.90791	0.91048	0.91134	0.91412	0.91711	0.92040
802	0.90235	0.90800	0.91058	0.91143	0.91421	0.91720	0.92048
803	0.90245	0.90810	0.91066	0.91152	0.91429	0.91728	0.92056
804	0.90255	0.90818	0.91076	0.91161	0.91438	0.91736	0.92064
805	0.90265	0.90828	0.91085	0.91170	0.91447	0.91744	0.92071
806	0.90276	0.90837	0.91093	0.91179	0.91455	0.91752	0.92079
807	0.90286	0.90846	0.91102	0.91187	0.91464	0.91761	0.92087
808	0.90295	0.90855	0.91111	0.91196	0.91472	0.91769	0.92095
809	0.90304	0.90864	0.91120	0.91205	0.91480	0.91777	0.92102
810	0.90314	0.90873	0.91129	0.91214	0.91489	0.91785	0.92110
811	0.90324	0.90883	0.91138	0.91222	0.91497	0.91793	0.92118
812	0.90333	0.90892	0.91147	0.91231	0.91506	0.91801	0.92126
813	0.90343	0.90901	0.91156	0.91240	0.91514	0.91809	0.92133
814	0.90353	0.90910	0.91165	0.91249	0.91523	0.91817	0.92141
815	0.90363	0.90919	0.91174	0.91258	0.91531	0.91825	0.92149
816	0.90373	0.90928	0.91182	0.91267	0.91539	0.91833	0.92156
817	0.90383	0.90937	0.91191	0.91275	0.91548	0.91841	0.92164
818	0.90392	0.90945	0.91199	0.91283	0.91556	0.91849	0.92172
819	0.90403	0.90954	0.91208	0.91292	0.91564	0.91857	0.92179
820	0.90412	0.90963	0.91217	0.91301	0.91573	0.91865	0.92187
821	0.90422	0.90972	0.91225	0.91309	0.91581	0.91873	0.92194
822	0.90432	0.90981	0.91234	0.91318	0.91589	0.91881	0.92202
823	0.90441	0.90990	0.91243	0.91326	0.91597	0.91889	0.92209
824	0.90450	0.90999	0.91251	0.91335	0.91605	0.91897	0.92217
825	0.90460	0.91008	0.91260	0.91343	0.91614	0.91905	0.92225
826	0.90469	0.91017	0.91269	0.91352	0.91622	0.91912	0.92232
827	0.90479	0.91025	0.91277	0.91360	0.91630	0.91920	0.92240
828	0.90488	0.91034	0.91286	0.91368	0.91638	0.91928	0.92247
829	0.90497	0.91043	0.91294	0.91377	0.91646	0.91936	0.92255
830	0.90506	0.91052	0.91302	0.91385	0.91654	0.91943	0.92262
831	0.90516	0.91060	0.91311	0.91393	0.91662	0.91951	0.92270
832	0.90525	0.91069	0.91319	0.91401	0.91670	0.91959	0.92277
833	0.90535	0.91077	0.91328	0.91410	0.91678	0.91967	0.92284
834	0.90544	0.91086	0.91336	0.91419	0.91686	0.91974	0.92292
835	0.90553	0.91095	0.91344	0.91427	0.91694	0.91982	0.92299
836	0.90562	0.91104	0.91353	0.91435	0.91702	0.91990	0.92306
837	0.90572	0.91112	0.91361	0.91444	0.91711	0.91997	0.92314
838	0.90581	0.91121	0.91369	0.91452	0.91719	0.92005	0.92321
839	0.90590	0.91130	0.91378	0.91460	0.91726	0.92013	0.92328
840	0.90598	0.91138	0.91386	0.91468	0.91734	0.92020	0.92335
841	0.90608	0.91146	0.91395	0.91476	0.91743	0.92028	0.92343
842	0.90617	0.91155	0.91403	0.91485	0.91750	0.92036	0.92350
843	0.90626	0.91164	0.91411	0.91492	0.91758	0.92043	0.92357
844	0.90636	0.91172	0.91419	0.91501	0.91766	0.92051	0.92364
845	0.90645	0.91181	0.91428	0.91509	0.91774	0.92058	0.92371
846	0.90653	0.91189	0.91436	0.91517	0.91782	0.92066	0.92379
847	0.90663	0.91198	0.91444	0.91525	0.91790	0.92073	0.92386
848	0.90673	0.91206	0.91452	0.91533	0.91798	0.92081	0.92393
849	0.90682	0.91215	0.91460	0.91541	0.91805	0.92088	0.92400

N	0.1%	0.5%	1%	1.25%	2.5%	5%	10%
850	0.90690	0.91223	0.91468	0.91549	0.91813	0.92096	0.92407
851	0.90697	0.91231	0.91476	0.91557	0.91821	0.92103	0.92414
852	0.90707	0.91240	0.91484	0.91565	0.91828	0.92111	0.92422
853	0.90715	0.91248	0.91493	0.91574	0.91836	0.92118	0.92429
854	0.90723	0.91256	0.91501	0.91582	0.91844	0.92125	0.92436
855	0.90732	0.91264	0.91509	0.91589	0.91852	0.92133	0.92443
856	0.90742	0.91272	0.91517	0.91597	0.91859	0.92140	0.92450
857	0.90751	0.91281	0.91525	0.91605	0.91867	0.92148	0.92457
858	0.90760	0.91289	0.91533	0.91613	0.91875	0.92155	0.92464
859	0.90767	0.91297	0.91541	0.91621	0.91882	0.92162	0.92471
860	0.90776	0.91306	0.91549	0.91629	0.91890	0.92169	0.92478
861	0.90785	0.91314	0.91557	0.91637	0.91898	0.92177	0.92485
862	0.90794	0.91322	0.91565	0.91645	0.91905	0.92184	0.92492
863	0.90803	0.91330	0.91572	0.91653	0.91913	0.92192	0.92499
864	0.90811	0.91338	0.91580	0.91661	0.91920	0.92199	0.92506
865	0.90819	0.91346	0.91588	0.91669	0.91928	0.92206	0.92513
866	0.90829	0.91354	0.91596	0.91676	0.91935	0.92213	0.92519
867	0.90837	0.91363	0.91604	0.91684	0.91943	0.92221	0.92526
868	0.90846	0.91371	0.91612	0.91692	0.91950	0.92228	0.92533
869	0.90854	0.91379	0.91620	0.91700	0.91958	0.92235	0.92540
870	0.90863	0.91387	0.91628	0.91707	0.91965	0.92242	0.92547
871	0.90871	0.91395	0.91636	0.91715	0.91972	0.92249	0.92554
872	0.90881	0.91403	0.91643	0.91723	0.91980	0.92256	0.92560
873	0.90889	0.91411	0.91651	0.91730	0.91987	0.92263	0.92567
874	0.90897	0.91419	0.91658	0.91738	0.91995	0.92271	0.92574
875	0.90905	0.91427	0.91666	0.91746	0.92002	0.92278	0.92581
876	0.90914	0.91435	0.91674	0.91754	0.92009	0.92285	0.92588
877	0.90922	0.91443	0.91682	0.91761	0.92017	0.92292	0.92594
878	0.90931	0.91451	0.91689	0.91768	0.92024	0.92299	0.92601
879	0.90940	0.91459	0.91697	0.91776	0.92032	0.92306	0.92608
880	0.90948	0.91467	0.91705	0.91784	0.92039	0.92313	0.92615
881	0.90957	0.91475	0.91713	0.91791	0.92046	0.92320	0.92621
882	0.90965	0.91483	0.91720	0.91799	0.92053	0.92327	0.92628
883	0.90973	0.91490	0.91727	0.91806	0.92061	0.92334	0.92635
884	0.90982	0.91498	0.91735	0.91814	0.92068	0.92341	0.92642
885	0.90991	0.91506	0.91743	0.91822	0.92075	0.92348	0.92648
886	0.90999	0.91514	0.91750	0.91829	0.92082	0.92355	0.92655
887	0.91006	0.91521	0.91758	0.91837	0.92089	0.92362	0.92661
888	0.91015	0.91529	0.91765	0.91844	0.92097	0.92369	0.92668
889	0.91024	0.91537	0.91773	0.91852	0.92104	0.92376	0.92675
890	0.91031	0.91545	0.91780	0.91859	0.92111	0.92383	0.92681
891	0.91039	0.91553	0.91788	0.91866	0.92118	0.92390	0.92688
892	0.91047	0.91561	0.91795	0.91874	0.92125	0.92396	0.92694
893	0.91057	0.91568	0.91803	0.91881	0.92132	0.92403	0.92701
894	0.91065	0.91576	0.91810	0.91889	0.92140	0.92410	0.92707
895	0.91073	0.91584	0.91818	0.91896	0.92147	0.92417	0.92714
896	0.91081	0.91591	0.91825	0.91903	0.92154	0.92424	0.92720
897	0.91089	0.91599	0.91832	0.91910	0.92161	0.92431	0.92727
898	0.91098	0.91607	0.91840	0.91918	0.92168	0.92437	0.92733
899	0.91106	0.91614	0.91847	0.91925	0.92175	0.92444	0.92740
900	0.91114	0.91622	0.91855	0.91932	0.92182	0.92451	0.92746
901	0.91122	0.91630	0.91862	0.91940	0.92189	0.92458	0.92753
902	0.91130	0.91637	0.91870	0.91947	0.92196	0.92464	0.92759
903	0.91138	0.91644	0.91877	0.91955	0.92203	0.92471	0.92766
904	0.91146	0.91652	0.91884	0.91962	0.92210	0.92478	0.92772
905	0.91154	0.91660	0.91891	0.91969	0.92217	0.92485	0.92778
906	0.91163	0.91667	0.91899	0.91976	0.92224	0.92491	0.92785
907	0.91170	0.91674	0.91906	0.91983	0.92231	0.92498	0.92791
908	0.91178	0.91682	0.91913	0.91991	0.92238	0.92504	0.92797
909	0.91187	0.91689	0.91920	0.91998	0.92245	0.92511	0.92804
910	0.91194	0.91697	0.91928	0.92005	0.92252	0.92518	0.92810
911	0.91202	0.91704	0.91935	0.92012	0.92258	0.92524	0.92816
912	0.91211	0.91711	0.91942	0.92019	0.92265	0.92531	0.92823
913	0.91219	0.91719	0.91949	0.92026	0.92273	0.92537	0.92829
914	0.91227	0.91726	0.91956	0.92033	0.92279	0.92544	0.92835
915	0.91234	0.91734	0.91964	0.92040	0.92286	0.92550	0.92842
916	0.91242	0.91741	0.91970	0.92047	0.92293	0.92557	0.92848
917	0.91250	0.91748	0.91978	0.92054	0.92300	0.92563	0.92854
918	0.91257	0.91755	0.91985	0.92061	0.92306	0.92570	0.92860
919	0.91265	0.91762	0.91992	0.92068	0.92313	0.92576	0.92866
920	0.91273	0.91770	0.91999	0.92075	0.92320	0.92583	0.92873
921	0.91282	0.91777	0.92006	0.92082	0.92326	0.92589	0.92879
922	0.91291	0.91784	0.92013	0.92089	0.92333	0.92596	0.92885
923	0.91299	0.91792	0.92020	0.92096	0.92340	0.92602	0.92891

N	0.1%	0.5%	1%	1.25%	2.5%	5%	10%
924	0.91305	0.91799	0.92027	0.92103	0.92347	0.92609	0.92897
925	0.91313	0.91807	0.92034	0.92110	0.92353	0.92615	0.92904
926	0.91321	0.91814	0.92041	0.92117	0.92360	0.92622	0.92910
927	0.91329	0.91821	0.92048	0.92124	0.92367	0.92628	0.92916
928	0.91337	0.91828	0.92055	0.92131	0.92373	0.92634	0.92922
929	0.91345	0.91836	0.92061	0.92137	0.92380	0.92641	0.92928
930	0.91351	0.91843	0.92068	0.92144	0.92387	0.92647	0.92934
931	0.91359	0.91851	0.92075	0.92151	0.92394	0.92653	0.92940
932	0.91367	0.91857	0.92082	0.92157	0.92400	0.92660	0.92946
933	0.91375	0.91864	0.92089	0.92165	0.92407	0.92666	0.92952
934	0.91383	0.91871	0.92096	0.92171	0.92413	0.92673	0.92958
935	0.91391	0.91878	0.92103	0.92178	0.92420	0.92679	0.92964
936	0.91399	0.91886	0.92110	0.92185	0.92426	0.92685	0.92970
937	0.91406	0.91892	0.92117	0.92192	0.92433	0.92692	0.92976
938	0.91413	0.91900	0.92124	0.92199	0.92439	0.92698	0.92982
939	0.91420	0.91907	0.92131	0.92205	0.92446	0.92704	0.92988
940	0.91428	0.91914	0.92138	0.92212	0.92452	0.92710	0.92994
941	0.91436	0.91921	0.92144	0.92219	0.92459	0.92717	0.93000
942	0.91443	0.91928	0.92151	0.92226	0.92465	0.92723	0.93006
943	0.91451	0.91936	0.92158	0.92232	0.92472	0.92729	0.93012
944	0.91458	0.91942	0.92165	0.92239	0.92478	0.92735	0.93018
945	0.91465	0.91949	0.92171	0.92246	0.92485	0.92741	0.93024
946	0.91473	0.91956	0.92178	0.92253	0.92491	0.92748	0.93030
947	0.91479	0.91963	0.92185	0.92259	0.92498	0.92754	0.93035
948	0.91488	0.91970	0.92192	0.92266	0.92504	0.92760	0.93041
949	0.91494	0.91977	0.92198	0.92272	0.92510	0.92766	0.93047
950	0.91502	0.91983	0.92205	0.92279	0.92516	0.92772	0.93053
951	0.91509	0.91991	0.92211	0.92285	0.92523	0.92778	0.93059
952	0.91516	0.91997	0.92218	0.92292	0.92529	0.92784	0.93065
953	0.91523	0.92004	0.92224	0.92298	0.92536	0.92790	0.93071
954	0.91530	0.92011	0.92231	0.92305	0.92542	0.92796	0.93076
955	0.91538	0.92018	0.92238	0.92312	0.92548	0.92802	0.93082
956	0.91544	0.92025	0.92244	0.92318	0.92555	0.92809	0.93088
957	0.91552	0.92031	0.92251	0.92325	0.92561	0.92815	0.93094
958	0.91559	0.92038	0.92258	0.92331	0.92567	0.92821	0.93100
959	0.91566	0.92045	0.92264	0.92337	0.92574	0.92827	0.93106
960	0.91574	0.92052	0.92271	0.92344	0.92580	0.92833	0.93111
961	0.91581	0.92058	0.92277	0.92350	0.92586	0.92839	0.93117
962	0.91588	0.92065	0.92284	0.92357	0.92593	0.92845	0.93123
963	0.91595	0.92072	0.92291	0.92364	0.92599	0.92851	0.93129
964	0.91603	0.92078	0.92297	0.92370	0.92605	0.92857	0.93134
965	0.91610	0.92085	0.92304	0.92376	0.92611	0.92863	0.93140
966	0.91617	0.92092	0.92310	0.92383	0.92617	0.92869	0.93146
967	0.91624	0.92099	0.92316	0.92389	0.92624	0.92875	0.93151
968	0.91631	0.92105	0.92323	0.92396	0.92630	0.92881	0.93157
969	0.91639	0.92112	0.92329	0.92402	0.92636	0.92887	0.93163
970	0.91645	0.92118	0.92336	0.92408	0.92642	0.92892	0.93168
971	0.91652	0.92125	0.92342	0.92415	0.92648	0.92898	0.93174
972	0.91659	0.92131	0.92349	0.92421	0.92654	0.92904	0.93180
973	0.91667	0.92138	0.92355	0.92427	0.92660	0.92910	0.93185
974	0.91674	0.92145	0.92362	0.92434	0.92666	0.92916	0.93191
975	0.91682	0.92151	0.92368	0.92440	0.92672	0.92922	0.93196
976	0.91688	0.92158	0.92375	0.92446	0.92679	0.92928	0.93202
977	0.91696	0.92165	0.92381	0.92452	0.92684	0.92934	0.93208
978	0.91703	0.92171	0.92387	0.92459	0.92691	0.92939	0.93213
979	0.91710	0.92177	0.92393	0.92465	0.92697	0.92945	0.93219
980	0.91716	0.92184	0.92400	0.92471	0.92702	0.92951	0.93224
981	0.91723	0.92190	0.92406	0.92478	0.92708	0.92957	0.93230
982	0.91729	0.92196	0.92412	0.92484	0.92715	0.92963	0.93235
983	0.91737	0.92203	0.92419	0.92490	0.92721	0.92968	0.93241
984	0.91744	0.92210	0.92425	0.92496	0.92727	0.92974	0.93246
985	0.91752	0.92216	0.92431	0.92502	0.92733	0.92980	0.93252
986	0.91759	0.92222	0.92437	0.92508	0.92739	0.92986	0.93257
987	0.91766	0.92229	0.92444	0.92515	0.92745	0.92992	0.93263
988	0.91773	0.92236	0.92450	0.92521	0.92751	0.92997	0.93268
989	0.91779	0.92242	0.92456	0.92527	0.92757	0.93003	0.93274
990	0.91786	0.92248	0.92463	0.92533	0.92763	0.93009	0.93279
991	0.91793	0.92255	0.92469	0.92540	0.92769	0.93014	0.93285
992	0.91801	0.92262	0.92475	0.92546	0.92775	0.93020	0.93290
993	0.91808	0.92268	0.92481	0.92552	0.92781	0.93026	0.93296
994	0.91814	0.92274	0.92488	0.92558	0.92787	0.93031	0.93301
995	0.91821	0.92280	0.92494	0.92564	0.92793	0.93037	0.93306
996	0.91828	0.92287	0.92500	0.92570	0.92799	0.93043	0.93312
997	0.91835	0.92293	0.92506	0.92576	0.92805	0.93048	0.93317

N	0.1%	0.5%	1%	1.25%	2.5%	5%	10%
998	0.91842	0.92299	0.92512	0.92582	0.92810	0.93054	0.93323
999	0.91849	0.92306	0.92518	0.92589	0.92816	0.93060	0.93328
1000	0.91855	0.92312	0.92524	0.92595	0.92822	0.93065	0.93333

Table 3 – *GM7(5H2L or 2H5L)*. Critical values of upper significance levels for the Grubbs multiple-7 outlier test, applicable in situations where the five highest and two lowest or two highest and five lowest observations are simultaneously identified as outliers, denoted as *GM7(5H2L)* and *GM7(2H5L)*, respectively.

N	0.1%	0.5%	1%	1.25%	2.5%	5%	10%
9	0.00000	0.00000	0.00000	0.00000	0.00001	0.00002	0.00009
10	0.00002	0.00008	0.00016	0.00021	0.00043	0.00090	0.00194
11	0.00024	0.00074	0.00122	0.00143	0.00239	0.00403	0.00699
12	0.00110	0.00260	0.00381	0.00432	0.00642	0.00969	0.01497
13	0.00293	0.00592	0.00812	0.00899	0.01246	0.01753	0.02515
14	0.00582	0.01065	0.01398	0.01526	0.02021	0.02707	0.03694
15	0.00990	0.01677	0.02120	0.02292	0.02933	0.03792	0.04980
16	0.01499	0.02397	0.02960	0.03169	0.03946	0.04965	0.06338
17	0.02110	0.03217	0.03886	0.04133	0.05040	0.06202	0.07739
18	0.02798	0.04110	0.04879	0.05165	0.06193	0.07482	0.09160
19	0.03553	0.05055	0.05927	0.06246	0.07374	0.08779	0.10583
20	0.04360	0.06047	0.07008	0.07354	0.08579	0.10088	0.12001
21	0.05215	0.07070	0.08105	0.08478	0.09797	0.11394	0.13400
22	0.06105	0.08107	0.09223	0.09623	0.11016	0.12695	0.14780
23	0.07029	0.09165	0.10347	0.10767	0.12224	0.13975	0.16129
24	0.07964	0.10237	0.11469	0.11911	0.13433	0.15239	0.17452
25	0.08900	0.11302	0.12591	0.13054	0.14626	0.16478	0.18742
26	0.09856	0.12364	0.13707	0.14184	0.15793	0.17700	0.20002
27	0.10815	0.13419	0.14805	0.15289	0.16942	0.18885	0.21231
28	0.11777	0.14471	0.15887	0.16375	0.18071	0.20045	0.22423
29	0.12733	0.15499	0.16936	0.17448	0.19174	0.21182	0.23581
30	0.13664	0.16514	0.17980	0.18500	0.20256	0.22287	0.24708
31	0.14607	0.17528	0.19015	0.19539	0.21313	0.23365	0.25804
32	0.15530	0.18512	0.20034	0.20559	0.22353	0.24422	0.26871
33	0.16461	0.19482	0.21022	0.21555	0.23371	0.25453	0.27902
34	0.17377	0.20436	0.21996	0.22540	0.24358	0.26448	0.28910
35	0.18283	0.21388	0.22947	0.23490	0.25328	0.27427	0.29887
36	0.19168	0.22306	0.23886	0.24433	0.26273	0.28376	0.30839
37	0.20018	0.23196	0.24795	0.25351	0.27195	0.29302	0.31759
38	0.20859	0.24073	0.25688	0.26238	0.28095	0.30197	0.32655
39	0.21699	0.24945	0.26560	0.27118	0.28964	0.31074	0.33530
40	0.22518	0.25782	0.27409	0.27968	0.29827	0.31930	0.34376
41	0.23344	0.26606	0.28242	0.28799	0.30666	0.32765	0.35200
42	0.24136	0.27412	0.29057	0.29614	0.31480	0.33580	0.36007
43	0.24924	0.28215	0.29851	0.30408	0.32276	0.34372	0.36792
44	0.25706	0.28995	0.30625	0.31193	0.33054	0.35141	0.37553
45	0.26473	0.29752	0.31393	0.31955	0.33816	0.35897	0.38297
46	0.27211	0.30494	0.32132	0.32693	0.34549	0.36629	0.39017
47	0.27940	0.31233	0.32861	0.33427	0.35274	0.37345	0.39721
48	0.28646	0.31956	0.33578	0.34133	0.35982	0.38042	0.40407
49	0.29362	0.32651	0.34275	0.34830	0.36668	0.38720	0.41077
50	0.30056	0.33333	0.34960	0.35509	0.37344	0.39389	0.41730
51	0.30731	0.34012	0.35632	0.36182	0.38004	0.40036	0.42367
52	0.31390	0.34666	0.36289	0.36830	0.38651	0.40674	0.42989
53	0.32053	0.35318	0.36920	0.37464	0.39280	0.41293	0.43596
54	0.32688	0.35952	0.37548	0.38092	0.39896	0.41902	0.44191
55	0.33299	0.36565	0.38163	0.38703	0.40502	0.42500	0.44769
56	0.33907	0.37173	0.38761	0.39301	0.41091	0.43080	0.45337
57	0.34484	0.37775	0.39350	0.39888	0.41671	0.43647	0.45891
58	0.35100	0.38365	0.39932	0.40463	0.42240	0.44198	0.46432
59	0.35677	0.38935	0.40498	0.41026	0.42791	0.44744	0.46961
60	0.36235	0.39494	0.41047	0.41577	0.43333	0.45276	0.47478
61	0.36789	0.40046	0.41587	0.42119	0.43866	0.45795	0.47985
62	0.37356	0.40581	0.42121	0.42649	0.44387	0.46306	0.48479
63	0.37881	0.41109	0.42645	0.43167	0.44899	0.46806	0.48961
64	0.38401	0.41615	0.43155	0.43673	0.45395	0.47294	0.49435
65	0.38936	0.42122	0.43655	0.44172	0.45888	0.47772	0.49900
66	0.39445	0.42628	0.44143	0.44664	0.46366	0.48242	0.50357
67	0.39954	0.43119	0.44628	0.45144	0.46837	0.48699	0.50803
68	0.40465	0.43603	0.45101	0.45616	0.47302	0.49149	0.51237
69	0.40942	0.44084	0.45564	0.46076	0.47756	0.49594	0.51668
70	0.41419	0.44547	0.46027	0.46533	0.48199	0.50027	0.52088

N	0.1%	0.5%	1%	1.25%	2.5%	5%	10%
71	0.41897	0.45008	0.46481	0.46985	0.48635	0.50454	0.52499
72	0.42341	0.45452	0.46924	0.47421	0.49066	0.50868	0.52903
73	0.42796	0.45885	0.47354	0.47853	0.49484	0.51279	0.53301
74	0.43239	0.46323	0.47775	0.48276	0.49896	0.51683	0.53690
75	0.43677	0.46742	0.48199	0.48692	0.50303	0.52076	0.54072
76	0.44100	0.47159	0.48609	0.49099	0.50698	0.52463	0.54445
77	0.44540	0.47567	0.49014	0.49503	0.51089	0.52844	0.54813
78	0.44984	0.47970	0.49406	0.49894	0.51476	0.53215	0.55174
79	0.45383	0.48365	0.49800	0.50280	0.51855	0.53583	0.55526
80	0.45782	0.48749	0.50178	0.50660	0.52227	0.53946	0.55877
81	0.46181	0.49133	0.50551	0.51028	0.52589	0.54299	0.56220
82	0.46577	0.49512	0.50920	0.51394	0.52949	0.54647	0.56558
83	0.46956	0.49879	0.51283	0.51756	0.53302	0.54990	0.56886
84	0.47333	0.50252	0.51641	0.52112	0.53650	0.55327	0.57210
85	0.47703	0.50612	0.51995	0.52466	0.53993	0.55659	0.57530
86	0.48085	0.50965	0.52345	0.52805	0.54331	0.55986	0.57844
87	0.48430	0.51310	0.52687	0.53148	0.54658	0.56307	0.58152
88	0.48789	0.51657	0.53022	0.53489	0.54982	0.56620	0.58457
89	0.49146	0.51997	0.53354	0.53814	0.55303	0.56932	0.58756
90	0.49481	0.52332	0.53681	0.54135	0.55619	0.57237	0.59050
91	0.49841	0.52665	0.54005	0.54456	0.55928	0.57538	0.59339
92	0.50176	0.52988	0.54319	0.54769	0.56231	0.57835	0.59621
93	0.50503	0.53303	0.54631	0.55079	0.56534	0.58126	0.59901
94	0.50830	0.53613	0.54938	0.55382	0.56831	0.58411	0.60177
95	0.51148	0.53918	0.55236	0.55677	0.57122	0.58692	0.60450
96	0.51479	0.54228	0.55534	0.55973	0.57411	0.58972	0.60717
97	0.51796	0.54535	0.55829	0.56262	0.57692	0.59246	0.60981
98	0.52110	0.54827	0.56113	0.56552	0.57973	0.59518	0.61240
99	0.52406	0.55121	0.56402	0.56837	0.58243	0.59782	0.61496
100	0.52706	0.55408	0.56684	0.57114	0.58516	0.60043	0.61749
101	0.53002	0.55692	0.56959	0.57388	0.58782	0.60303	0.61996
102	0.53294	0.55975	0.57234	0.57657	0.59044	0.60557	0.62241
103	0.53576	0.56246	0.57503	0.57927	0.59303	0.60808	0.62483
104	0.53870	0.56523	0.57769	0.58193	0.59563	0.61055	0.62720
105	0.54150	0.56788	0.58031	0.58453	0.59816	0.61298	0.62953
106	0.54434	0.57052	0.58292	0.58710	0.60065	0.61538	0.63183
107	0.54709	0.57314	0.58547	0.58963	0.60312	0.61777	0.63412
108	0.54984	0.57572	0.58801	0.59214	0.60558	0.62012	0.63636
109	0.55256	0.57825	0.59051	0.59457	0.60793	0.62243	0.63858
110	0.55515	0.58078	0.59291	0.59701	0.61028	0.62469	0.64076
111	0.55769	0.58325	0.59535	0.59940	0.61259	0.62694	0.64292
112	0.56026	0.58567	0.59771	0.60176	0.61488	0.62915	0.64505
113	0.56274	0.58806	0.60006	0.60408	0.61716	0.63134	0.64715
114	0.56527	0.59046	0.60236	0.60636	0.61937	0.63349	0.64922
115	0.56775	0.59280	0.60464	0.60860	0.62157	0.63561	0.65127
116	0.57016	0.59514	0.60690	0.61089	0.62373	0.63771	0.65328
117	0.57259	0.59741	0.60915	0.61312	0.62589	0.63978	0.65525
118	0.57504	0.59970	0.61138	0.61531	0.62802	0.64183	0.65722
119	0.57737	0.60192	0.61352	0.61744	0.63012	0.64385	0.65915
120	0.57964	0.60407	0.61568	0.61957	0.63220	0.64584	0.66107
121	0.58195	0.60631	0.61780	0.62167	0.63423	0.64781	0.66298
122	0.58417	0.60846	0.61992	0.62375	0.63625	0.64977	0.66484
123	0.58647	0.61063	0.62197	0.62579	0.63824	0.65169	0.66667
124	0.58875	0.61278	0.62403	0.62784	0.64022	0.65359	0.66849
125	0.59093	0.61481	0.62602	0.62982	0.64218	0.65548	0.67029
126	0.59304	0.61687	0.62801	0.63178	0.64411	0.65732	0.67208
127	0.59522	0.61888	0.63000	0.63376	0.64600	0.65915	0.67384
128	0.59736	0.62089	0.63197	0.63569	0.64787	0.66097	0.67558
129	0.59942	0.62288	0.63390	0.63759	0.64973	0.66277	0.67730
130	0.60150	0.62485	0.63581	0.63949	0.65156	0.66454	0.67898
131	0.60346	0.62681	0.63773	0.64135	0.65336	0.66629	0.68065
132	0.60561	0.62865	0.63959	0.64321	0.65514	0.66801	0.68231
133	0.60758	0.63057	0.64142	0.64504	0.65690	0.66971	0.68395
134	0.60966	0.63245	0.64326	0.64685	0.65866	0.67141	0.68557
135	0.61154	0.63430	0.64506	0.64865	0.66038	0.67308	0.68717
136	0.61350	0.63611	0.64683	0.65043	0.66209	0.67472	0.68874
137	0.61548	0.63792	0.64860	0.65219	0.66378	0.67634	0.69029
138	0.61744	0.63972	0.65034	0.65391	0.66545	0.67794	0.69184
139	0.61929	0.64148	0.65208	0.65561	0.66710	0.67954	0.69337
140	0.62116	0.64329	0.65377	0.65730	0.66873	0.68112	0.69487
141	0.62294	0.64501	0.65546	0.65898	0.67036	0.68268	0.69635
142	0.62468	0.64669	0.65713	0.66064	0.67195	0.68423	0.69783
143	0.62654	0.64844	0.65878	0.66225	0.67356	0.68577	0.69929
144	0.62833	0.65010	0.66042	0.66389	0.67512	0.68730	0.70073

N	0.1%	0.5%	1%	1.25%	2.5%	5%	10%
145	0.63009	0.65180	0.66204	0.66551	0.67667	0.68878	0.70217
146	0.63188	0.65343	0.66367	0.66710	0.67821	0.69026	0.70359
147	0.63354	0.65505	0.66528	0.66868	0.67975	0.69171	0.70499
148	0.63518	0.65662	0.66684	0.67022	0.68124	0.69316	0.70636
149	0.63691	0.65822	0.66838	0.67177	0.68273	0.69460	0.70774
150	0.63851	0.65981	0.66991	0.67330	0.68421	0.69601	0.70909
151	0.64021	0.66138	0.67144	0.67479	0.68564	0.69742	0.71042
152	0.64187	0.66294	0.67295	0.67628	0.68709	0.69880	0.71175
153	0.64354	0.66447	0.67442	0.67775	0.68852	0.70018	0.71306
154	0.64513	0.66599	0.67591	0.67921	0.68994	0.70155	0.71436
155	0.64676	0.66754	0.67738	0.68067	0.69133	0.70290	0.71565
156	0.64831	0.66901	0.67882	0.68209	0.69273	0.70422	0.71693
157	0.64976	0.67047	0.68023	0.68349	0.69408	0.70554	0.71819
158	0.65138	0.67196	0.68166	0.68491	0.69544	0.70685	0.71944
159	0.65287	0.67340	0.68306	0.68629	0.69678	0.70814	0.72068
160	0.65444	0.67486	0.68445	0.68767	0.69811	0.70942	0.72190
161	0.65594	0.67627	0.68580	0.68903	0.69943	0.71068	0.72311
162	0.65737	0.67767	0.68713	0.69035	0.70074	0.71193	0.72431
163	0.65890	0.67902	0.68851	0.69170	0.70202	0.71319	0.72550
164	0.66035	0.68043	0.68985	0.69302	0.70331	0.71441	0.72668
165	0.66182	0.68178	0.69118	0.69433	0.70457	0.71563	0.72786
166	0.66322	0.68308	0.69248	0.69561	0.70583	0.71685	0.72901
167	0.66462	0.68447	0.69379	0.69691	0.70708	0.71805	0.73015
168	0.66600	0.68583	0.69507	0.69817	0.70831	0.71924	0.73129
169	0.66743	0.68714	0.69636	0.69944	0.70951	0.72042	0.73241
170	0.66883	0.68846	0.69763	0.70069	0.71072	0.72158	0.73352
171	0.67014	0.68974	0.69887	0.70194	0.71193	0.72273	0.73462
172	0.67153	0.69102	0.70010	0.70318	0.71312	0.72387	0.73571
173	0.67287	0.69228	0.70133	0.70439	0.71430	0.72501	0.73679
174	0.67424	0.69357	0.70255	0.70560	0.71547	0.72612	0.73787
175	0.67558	0.69480	0.70375	0.70679	0.71662	0.72723	0.73892
176	0.67680	0.69603	0.70496	0.70798	0.71775	0.72833	0.73998
177	0.67804	0.69726	0.70616	0.70915	0.71889	0.72943	0.74102
178	0.67936	0.69845	0.70732	0.71032	0.72001	0.73052	0.74205
179	0.68060	0.69964	0.70849	0.71147	0.72113	0.73159	0.74308
180	0.68189	0.70084	0.70965	0.71261	0.72224	0.73265	0.74410
181	0.68310	0.70202	0.71079	0.71375	0.72333	0.73370	0.74511
182	0.68437	0.70321	0.71192	0.71487	0.72442	0.73474	0.74611
183	0.68562	0.70436	0.71306	0.71599	0.72549	0.73577	0.74709
184	0.68682	0.70552	0.71418	0.71708	0.72657	0.73680	0.74807
185	0.68801	0.70665	0.71529	0.71819	0.72761	0.73781	0.74905
186	0.68924	0.70778	0.71639	0.71926	0.72865	0.73881	0.75001
187	0.69047	0.70893	0.71748	0.72035	0.72970	0.73982	0.75096
188	0.69161	0.71004	0.71857	0.72140	0.73073	0.74080	0.75192
189	0.69275	0.71113	0.71963	0.72244	0.73174	0.74178	0.75285
190	0.69390	0.71220	0.72068	0.72351	0.73276	0.74276	0.75378
191	0.69503	0.71324	0.72175	0.72457	0.73377	0.74372	0.75470
192	0.69612	0.71433	0.72280	0.72559	0.73476	0.74469	0.75561
193	0.69723	0.71544	0.72384	0.72662	0.73576	0.74564	0.75652
194	0.69837	0.71649	0.72484	0.72762	0.73673	0.74659	0.75743
195	0.69949	0.71754	0.72586	0.72864	0.73771	0.74752	0.75832
196	0.70056	0.71858	0.72686	0.72965	0.73867	0.74845	0.75921
197	0.70167	0.71958	0.72788	0.73064	0.73963	0.74937	0.76008
198	0.70273	0.72061	0.72888	0.73161	0.74059	0.75028	0.76095
199	0.70377	0.72164	0.72984	0.73257	0.74153	0.75118	0.76182
200	0.70486	0.72263	0.73083	0.73355	0.74247	0.75208	0.76267
201	0.70597	0.72365	0.73179	0.73452	0.74338	0.75297	0.76352
202	0.70708	0.72465	0.73275	0.73547	0.74432	0.75385	0.76436
203	0.70818	0.72564	0.73370	0.73641	0.74523	0.75472	0.76521
204	0.70916	0.72662	0.73465	0.73735	0.74613	0.75560	0.76603
205	0.71016	0.72754	0.73560	0.73829	0.74704	0.75646	0.76685
206	0.71123	0.72850	0.73654	0.73921	0.74793	0.75732	0.76768
207	0.71225	0.72946	0.73745	0.74013	0.74880	0.75817	0.76849
208	0.71326	0.73039	0.73839	0.74103	0.74968	0.75901	0.76929
209	0.71427	0.73135	0.73931	0.74192	0.75055	0.75985	0.77009
210	0.71523	0.73227	0.74020	0.74282	0.75141	0.76068	0.77088
211	0.71625	0.73318	0.74109	0.74372	0.75228	0.76150	0.77167
212	0.71721	0.73413	0.74198	0.74461	0.75312	0.76231	0.77245
213	0.71823	0.73503	0.74287	0.74547	0.75396	0.76312	0.77323
214	0.71919	0.73594	0.74374	0.74632	0.75480	0.76392	0.77399
215	0.72012	0.73682	0.74461	0.74721	0.75563	0.76473	0.77476
216	0.72112	0.73774	0.74548	0.74806	0.75646	0.76554	0.77551
217	0.72201	0.73861	0.74631	0.74890	0.75728	0.76632	0.77626
218	0.72294	0.73951	0.74717	0.74975	0.75809	0.76710	0.77700

N	0.1%	0.5%	1%	1.25%	2.5%	5%	10%
219	0.72381	0.74037	0.74802	0.75058	0.75890	0.76787	0.77774
220	0.72479	0.74121	0.74886	0.75142	0.75970	0.76863	0.77848
221	0.72571	0.74208	0.74969	0.75225	0.76049	0.76940	0.77921
222	0.72665	0.74294	0.75053	0.75306	0.76128	0.77017	0.77993
223	0.72751	0.74379	0.75134	0.75387	0.76207	0.77092	0.78064
224	0.72843	0.74463	0.75215	0.75466	0.76284	0.77167	0.78136
225	0.72932	0.74546	0.75298	0.75546	0.76361	0.77241	0.78207
226	0.73022	0.74627	0.75376	0.75627	0.76438	0.77313	0.78278
227	0.73106	0.74710	0.75455	0.75705	0.76513	0.77386	0.78347
228	0.73188	0.74795	0.75535	0.75784	0.76588	0.77458	0.78416
229	0.73273	0.74875	0.75615	0.75861	0.76663	0.77531	0.78485
230	0.73361	0.74954	0.75693	0.75937	0.76737	0.77603	0.78554
231	0.73442	0.75034	0.75770	0.76015	0.76812	0.77674	0.78622
232	0.73530	0.75113	0.75845	0.76090	0.76885	0.77745	0.78689
233	0.73613	0.75189	0.75922	0.76165	0.76958	0.77815	0.78757
234	0.73693	0.75269	0.75999	0.76240	0.77031	0.77884	0.78823
235	0.73777	0.75348	0.76073	0.76314	0.77103	0.77953	0.78889
236	0.73863	0.75426	0.76147	0.76389	0.77174	0.78022	0.78954
237	0.73946	0.75502	0.76220	0.76461	0.77245	0.78089	0.79019
238	0.74023	0.75578	0.76294	0.76536	0.77315	0.78156	0.79084
239	0.74105	0.75652	0.76368	0.76609	0.77385	0.78223	0.79148
240	0.74188	0.75726	0.76442	0.76680	0.77454	0.78290	0.79212
241	0.74266	0.75801	0.76516	0.76752	0.77523	0.78356	0.79275
242	0.74342	0.75876	0.76587	0.76822	0.77591	0.78423	0.79338
243	0.74418	0.75949	0.76658	0.76894	0.77658	0.78488	0.79400
244	0.74506	0.76021	0.76728	0.76965	0.77726	0.78553	0.79462
245	0.74581	0.76094	0.76799	0.77034	0.77795	0.78618	0.79524
246	0.74656	0.76166	0.76870	0.77103	0.77861	0.78682	0.79585
247	0.74730	0.76240	0.76938	0.77172	0.77927	0.78745	0.79646
248	0.74807	0.76311	0.77007	0.77241	0.77994	0.78808	0.79706
249	0.74880	0.76381	0.77076	0.77309	0.78059	0.78870	0.79766
250	0.74957	0.76451	0.77145	0.77376	0.78124	0.78933	0.79826
251	0.75032	0.76522	0.77211	0.77442	0.78189	0.78995	0.79885
252	0.75107	0.76590	0.77277	0.77508	0.78254	0.79057	0.79943
253	0.75182	0.76657	0.77344	0.77574	0.78317	0.79118	0.80002
254	0.75258	0.76727	0.77410	0.77640	0.78381	0.79179	0.80060
255	0.75325	0.76795	0.77476	0.77704	0.78443	0.79239	0.80118
256	0.75398	0.76863	0.77542	0.77769	0.78505	0.79300	0.80175
257	0.75469	0.76929	0.77608	0.77834	0.78567	0.79359	0.80232
258	0.75538	0.76994	0.77672	0.77896	0.78630	0.79419	0.80289
259	0.75615	0.77060	0.77735	0.77960	0.78691	0.79478	0.80345
260	0.75685	0.77126	0.77798	0.78024	0.78752	0.79536	0.80401
261	0.75756	0.77191	0.77862	0.78086	0.78812	0.79594	0.80456
262	0.75827	0.77256	0.77926	0.78148	0.78872	0.79651	0.80511
263	0.75897	0.77317	0.77989	0.78210	0.78932	0.79709	0.80566
264	0.75965	0.77383	0.78051	0.78272	0.78991	0.79766	0.80620
265	0.76037	0.77446	0.78113	0.78331	0.79050	0.79822	0.80674
266	0.76105	0.77511	0.78175	0.78393	0.79109	0.79879	0.80728
267	0.76166	0.77572	0.78236	0.78455	0.79167	0.79935	0.80782
268	0.76233	0.77636	0.78295	0.78514	0.79225	0.79991	0.80835
269	0.76300	0.77698	0.78356	0.78574	0.79282	0.80046	0.80887
270	0.76369	0.77758	0.78415	0.78634	0.79340	0.80101	0.80940
271	0.76433	0.77820	0.78474	0.78693	0.79396	0.80156	0.80992
272	0.76491	0.77882	0.78533	0.78752	0.79453	0.80210	0.81045
273	0.76558	0.77942	0.78592	0.78809	0.79510	0.80264	0.81096
274	0.76618	0.78004	0.78652	0.78868	0.79566	0.80318	0.81147
275	0.76684	0.78065	0.78709	0.78926	0.79622	0.80371	0.81198
276	0.76752	0.78124	0.78768	0.78982	0.79678	0.80424	0.81249
277	0.76814	0.78184	0.78826	0.79040	0.79733	0.80478	0.81299
278	0.76877	0.78243	0.78883	0.79096	0.79788	0.80530	0.81349
279	0.76944	0.78301	0.78940	0.79152	0.79842	0.80582	0.81399
280	0.77002	0.78362	0.78996	0.79208	0.79895	0.80634	0.81449
281	0.77065	0.78422	0.79053	0.79264	0.79949	0.80685	0.81498
282	0.77127	0.78479	0.79108	0.79320	0.80002	0.80736	0.81547
283	0.77186	0.78536	0.79163	0.79376	0.80056	0.80786	0.81595
284	0.77246	0.78592	0.79220	0.79430	0.80109	0.80837	0.81644
285	0.77306	0.78650	0.79275	0.79485	0.80161	0.80887	0.81692
286	0.77364	0.78707	0.79332	0.79538	0.80212	0.80937	0.81740
287	0.77423	0.78762	0.79386	0.79592	0.80264	0.80986	0.81787
288	0.77487	0.78817	0.79440	0.79645	0.80316	0.81036	0.81834
289	0.77542	0.78873	0.79493	0.79699	0.80367	0.81086	0.81881
290	0.77600	0.78928	0.79546	0.79751	0.80417	0.81135	0.81928
291	0.77659	0.78983	0.79600	0.79803	0.80468	0.81183	0.81975
292	0.77719	0.79038	0.79654	0.79856	0.80519	0.81231	0.82020

N	0.1%	0.5%	1%	1.25%	2.5%	5%	10%
293	0.77773	0.79090	0.79705	0.79906	0.80569	0.81279	0.82066
294	0.77834	0.79143	0.79756	0.79958	0.80619	0.81327	0.82111
295	0.77891	0.79197	0.79807	0.80010	0.80669	0.81375	0.82157
296	0.77948	0.79250	0.79859	0.80061	0.80718	0.81422	0.82202
297	0.78003	0.79303	0.79910	0.80113	0.80767	0.81469	0.82247
298	0.78065	0.79356	0.79962	0.80163	0.80815	0.81516	0.82291
299	0.78116	0.79409	0.80013	0.80213	0.80864	0.81562	0.82335
300	0.78172	0.79460	0.80063	0.80263	0.80911	0.81608	0.82379
301	0.78230	0.79512	0.80113	0.80313	0.80959	0.81654	0.82423
302	0.78285	0.79563	0.80163	0.80361	0.81007	0.81700	0.82466
303	0.78340	0.79614	0.80212	0.80411	0.81054	0.81745	0.82510
304	0.78393	0.79664	0.80262	0.80460	0.81100	0.81791	0.82553
305	0.78445	0.79715	0.80310	0.80508	0.81147	0.81836	0.82595
306	0.78496	0.79765	0.80360	0.80556	0.81193	0.81880	0.82638
307	0.78547	0.79816	0.80407	0.80603	0.81240	0.81925	0.82680
308	0.78601	0.79865	0.80455	0.80652	0.81286	0.81969	0.82722
309	0.78651	0.79914	0.80503	0.80700	0.81332	0.82013	0.82764
310	0.78702	0.79964	0.80550	0.80746	0.81377	0.82057	0.82806
311	0.78754	0.80013	0.80599	0.80793	0.81423	0.82100	0.82848
312	0.78809	0.80063	0.80646	0.80839	0.81468	0.82144	0.82889
313	0.78859	0.80111	0.80691	0.80885	0.81513	0.82187	0.82930
314	0.78910	0.80159	0.80739	0.80931	0.81557	0.82230	0.82971
315	0.78961	0.80207	0.80786	0.80977	0.81602	0.82272	0.83011
316	0.79014	0.80255	0.80832	0.81024	0.81646	0.82315	0.83052
317	0.79063	0.80302	0.80878	0.81069	0.81690	0.82356	0.83092
318	0.79111	0.80349	0.80923	0.81114	0.81734	0.82398	0.83132
319	0.79161	0.80396	0.80969	0.81161	0.81776	0.82440	0.83172
320	0.79212	0.80443	0.81014	0.81205	0.81820	0.82482	0.83211
321	0.79263	0.80488	0.81060	0.81250	0.81863	0.82523	0.83251
322	0.79314	0.80536	0.81105	0.81294	0.81906	0.82564	0.83290
323	0.79362	0.80582	0.81150	0.81338	0.81948	0.82605	0.83329
324	0.79412	0.80628	0.81194	0.81382	0.81991	0.82645	0.83367
325	0.79461	0.80674	0.81238	0.81425	0.82031	0.82686	0.83406
326	0.79508	0.80719	0.81282	0.81469	0.82074	0.82726	0.83445
327	0.79557	0.80765	0.81326	0.81512	0.82115	0.82766	0.83483
328	0.79606	0.80810	0.81369	0.81555	0.82157	0.82805	0.83521
329	0.79657	0.80854	0.81411	0.81597	0.82198	0.82844	0.83558
330	0.79701	0.80898	0.81452	0.81640	0.82239	0.82884	0.83596
331	0.79749	0.80942	0.81496	0.81682	0.82279	0.82923	0.83634
332	0.79796	0.80985	0.81538	0.81724	0.82320	0.82962	0.83671
333	0.79841	0.81030	0.81580	0.81767	0.82360	0.83001	0.83708
334	0.79885	0.81073	0.81622	0.81808	0.82401	0.83040	0.83744
335	0.79930	0.81115	0.81664	0.81850	0.82441	0.83078	0.83781
336	0.79976	0.81158	0.81706	0.81890	0.82480	0.83117	0.83818
337	0.80023	0.81200	0.81747	0.81931	0.82520	0.83154	0.83854
338	0.80067	0.81243	0.81789	0.81971	0.82560	0.83192	0.83890
339	0.80112	0.81286	0.81831	0.82013	0.82599	0.83229	0.83926
340	0.80156	0.81328	0.81871	0.82053	0.82638	0.83267	0.83961
341	0.80200	0.81370	0.81912	0.82094	0.82677	0.83304	0.83997
342	0.80248	0.81412	0.81953	0.82133	0.82716	0.83342	0.84033
343	0.80291	0.81453	0.81993	0.82173	0.82755	0.83378	0.84068
344	0.80338	0.81495	0.82033	0.82212	0.82794	0.83416	0.84103
345	0.80381	0.81536	0.82073	0.82252	0.82832	0.83452	0.84138
346	0.80421	0.81578	0.82113	0.82292	0.82870	0.83488	0.84172
347	0.80465	0.81618	0.82153	0.82331	0.82907	0.83525	0.84207
348	0.80508	0.81659	0.82193	0.82370	0.82945	0.83561	0.84241
349	0.80552	0.81700	0.82232	0.82409	0.82982	0.83597	0.84275
350	0.80596	0.81740	0.82271	0.82447	0.83019	0.83633	0.84309
351	0.80641	0.81780	0.82309	0.82485	0.83056	0.83668	0.84343
352	0.80683	0.81821	0.82347	0.82524	0.83093	0.83703	0.84377
353	0.80725	0.81860	0.82386	0.82561	0.83130	0.83738	0.84411
354	0.80766	0.81899	0.82426	0.82600	0.83166	0.83773	0.84444
355	0.80806	0.81938	0.82463	0.82637	0.83203	0.83808	0.84477
356	0.80847	0.81977	0.82501	0.82674	0.83239	0.83843	0.84510
357	0.80889	0.82016	0.82539	0.82712	0.83275	0.83878	0.84544
358	0.80928	0.82055	0.82576	0.82749	0.83311	0.83912	0.84576
359	0.80969	0.82093	0.82613	0.82786	0.83346	0.83945	0.84609
360	0.81011	0.82132	0.82650	0.82823	0.83381	0.83980	0.84641
361	0.81053	0.82171	0.82687	0.82859	0.83417	0.84014	0.84673
362	0.81094	0.82208	0.82724	0.82895	0.83452	0.84048	0.84705
363	0.81136	0.82246	0.82760	0.82931	0.83487	0.84081	0.84738
364	0.81170	0.82283	0.82797	0.82968	0.83522	0.84114	0.84770
365	0.81212	0.82320	0.82833	0.83005	0.83557	0.84148	0.84801
366	0.81251	0.82360	0.82869	0.83040	0.83591	0.84180	0.84834

N	0.1%	0.5%	1%	1.25%	2.5%	5%	10%
367	0.81289	0.82396	0.82905	0.83076	0.83625	0.84214	0.84865
368	0.81329	0.82434	0.82940	0.83110	0.83659	0.84246	0.84896
369	0.81366	0.82470	0.82976	0.83146	0.83692	0.84279	0.84927
370	0.81405	0.82507	0.83012	0.83182	0.83726	0.84311	0.84959
371	0.81444	0.82541	0.83047	0.83217	0.83760	0.84344	0.84990
372	0.81482	0.82578	0.83083	0.83251	0.83794	0.84377	0.85020
373	0.81521	0.82613	0.83118	0.83285	0.83827	0.84408	0.85051
374	0.81558	0.82650	0.83153	0.83319	0.83860	0.84441	0.85081
375	0.81598	0.82686	0.83187	0.83354	0.83893	0.84472	0.85111
376	0.81637	0.82723	0.83222	0.83388	0.83926	0.84504	0.85141
377	0.81676	0.82759	0.83256	0.83422	0.83959	0.84535	0.85171
378	0.81716	0.82793	0.83291	0.83456	0.83992	0.84567	0.85201
379	0.81751	0.82828	0.83325	0.83489	0.84024	0.84598	0.85231
380	0.81787	0.82861	0.83359	0.83523	0.84057	0.84629	0.85261
381	0.81824	0.82897	0.83392	0.83557	0.84089	0.84661	0.85291
382	0.81861	0.82932	0.83426	0.83591	0.84121	0.84691	0.85320
383	0.81898	0.82967	0.83459	0.83624	0.84152	0.84722	0.85349
384	0.81934	0.83001	0.83492	0.83657	0.84184	0.84752	0.85379
385	0.81971	0.83036	0.83526	0.83689	0.84216	0.84782	0.85408
386	0.82008	0.83070	0.83559	0.83722	0.84248	0.84813	0.85436
387	0.82046	0.83104	0.83592	0.83753	0.84279	0.84843	0.85465
388	0.82081	0.83138	0.83624	0.83786	0.84310	0.84873	0.85494
389	0.82119	0.83172	0.83657	0.83818	0.84341	0.84903	0.85522
390	0.82153	0.83206	0.83689	0.83850	0.84371	0.84933	0.85551
391	0.82190	0.83239	0.83721	0.83882	0.84402	0.84962	0.85579
392	0.82225	0.83272	0.83753	0.83914	0.84433	0.84992	0.85607
393	0.82260	0.83304	0.83784	0.83944	0.84463	0.85021	0.85635
394	0.82297	0.83338	0.83817	0.83977	0.84494	0.85050	0.85663
395	0.82330	0.83370	0.83848	0.84008	0.84524	0.85079	0.85690
396	0.82363	0.83403	0.83880	0.84039	0.84554	0.85108	0.85718
397	0.82397	0.83436	0.83911	0.84070	0.84584	0.85137	0.85746
398	0.82430	0.83468	0.83943	0.84101	0.84614	0.85166	0.85773
399	0.82467	0.83500	0.83975	0.84132	0.84644	0.85194	0.85800
400	0.82503	0.83531	0.84006	0.84163	0.84673	0.85223	0.85827
401	0.82537	0.83563	0.84036	0.84193	0.84703	0.85251	0.85854
402	0.82573	0.83594	0.84068	0.84225	0.84732	0.85280	0.85881
403	0.82609	0.83626	0.84098	0.84255	0.84762	0.85308	0.85908
404	0.82643	0.83658	0.84129	0.84285	0.84791	0.85336	0.85935
405	0.82675	0.83689	0.84158	0.84315	0.84820	0.85364	0.85962
406	0.82706	0.83721	0.84189	0.84346	0.84849	0.85391	0.85988
407	0.82739	0.83753	0.84220	0.84375	0.84878	0.85419	0.86014
408	0.82773	0.83783	0.84251	0.84405	0.84907	0.85447	0.86041
409	0.82803	0.83815	0.84280	0.84434	0.84935	0.85474	0.86067
410	0.82836	0.83847	0.84310	0.84464	0.84964	0.85501	0.86093
411	0.82867	0.83877	0.84339	0.84493	0.84992	0.85528	0.86119
412	0.82900	0.83907	0.84370	0.84522	0.85020	0.85555	0.86145
413	0.82933	0.83937	0.84399	0.84551	0.85049	0.85582	0.86171
414	0.82963	0.83967	0.84428	0.84580	0.85076	0.85609	0.86197
415	0.82993	0.83998	0.84456	0.84610	0.85104	0.85636	0.86222
416	0.83026	0.84027	0.84485	0.84638	0.85132	0.85662	0.86248
417	0.83058	0.84056	0.84514	0.84667	0.85160	0.85689	0.86273
418	0.83090	0.84086	0.84544	0.84694	0.85187	0.85716	0.86298
419	0.83122	0.84115	0.84573	0.84723	0.85214	0.85742	0.86323
420	0.83153	0.84144	0.84601	0.84752	0.85241	0.85768	0.86348
421	0.83186	0.84174	0.84630	0.84781	0.85269	0.85794	0.86373
422	0.83218	0.84203	0.84658	0.84808	0.85296	0.85820	0.86398
423	0.83251	0.84233	0.84686	0.84836	0.85323	0.85846	0.86423
424	0.83282	0.84261	0.84714	0.84864	0.85350	0.85872	0.86447
425	0.83314	0.84290	0.84741	0.84891	0.85377	0.85897	0.86472
426	0.83345	0.84318	0.84770	0.84919	0.85403	0.85923	0.86496
427	0.83375	0.84347	0.84797	0.84946	0.85430	0.85949	0.86521
428	0.83404	0.84375	0.84825	0.84974	0.85456	0.85974	0.86545
429	0.83436	0.84404	0.84852	0.85001	0.85482	0.85999	0.86569
430	0.83468	0.84432	0.84879	0.85028	0.85509	0.86024	0.86593
431	0.83497	0.84461	0.84906	0.85056	0.85534	0.86050	0.86617
432	0.83528	0.84489	0.84934	0.85083	0.85560	0.86075	0.86641
433	0.83559	0.84518	0.84962	0.85110	0.85586	0.86100	0.86664
434	0.83589	0.84546	0.84989	0.85137	0.85612	0.86125	0.86688
435	0.83620	0.84573	0.85015	0.85163	0.85638	0.86149	0.86711
436	0.83649	0.84601	0.85043	0.85190	0.85663	0.86174	0.86735
437	0.83678	0.84629	0.85070	0.85217	0.85689	0.86198	0.86758
438	0.83710	0.84657	0.85096	0.85243	0.85714	0.86223	0.86782
439	0.83738	0.84684	0.85123	0.85269	0.85740	0.86247	0.86805
440	0.83765	0.84711	0.85149	0.85295	0.85765	0.86272	0.86828

N	0.1%	0.5%	1%	1.25%	2.5%	5%	10%
441	0.83795	0.84738	0.85176	0.85321	0.85790	0.86295	0.86851
442	0.83824	0.84764	0.85201	0.85346	0.85815	0.86319	0.86874
443	0.83855	0.84791	0.85227	0.85372	0.85840	0.86344	0.86897
444	0.83883	0.84817	0.85254	0.85399	0.85865	0.86367	0.86920
445	0.83913	0.84843	0.85280	0.85423	0.85890	0.86391	0.86942
446	0.83941	0.84871	0.85305	0.85449	0.85914	0.86414	0.86965
447	0.83969	0.84897	0.85331	0.85475	0.85939	0.86438	0.86987
448	0.84001	0.84923	0.85357	0.85501	0.85963	0.86461	0.87010
449	0.84029	0.84949	0.85383	0.85526	0.85988	0.86485	0.87032
450	0.84057	0.84976	0.85408	0.85551	0.86012	0.86508	0.87054
451	0.84083	0.85003	0.85434	0.85577	0.86036	0.86531	0.87076
452	0.84109	0.85028	0.85458	0.85602	0.86060	0.86554	0.87098
453	0.84136	0.85054	0.85483	0.85626	0.86083	0.86577	0.87120
454	0.84165	0.85081	0.85508	0.85651	0.86108	0.86600	0.87142
455	0.84191	0.85106	0.85533	0.85675	0.86132	0.86623	0.87164
456	0.84218	0.85132	0.85558	0.85699	0.86155	0.86646	0.87186
457	0.84245	0.85159	0.85583	0.85724	0.86179	0.86668	0.87207
458	0.84274	0.85184	0.85607	0.85748	0.86203	0.86691	0.87229
459	0.84301	0.85210	0.85631	0.85773	0.86226	0.86713	0.87250
460	0.84327	0.85236	0.85655	0.85797	0.86250	0.86735	0.87272
461	0.84357	0.85261	0.85680	0.85821	0.86273	0.86758	0.87293
462	0.84383	0.85286	0.85704	0.85846	0.86296	0.86780	0.87314
463	0.84410	0.85310	0.85729	0.85869	0.86319	0.86802	0.87336
464	0.84438	0.85334	0.85753	0.85892	0.86342	0.86825	0.87357
465	0.84463	0.85360	0.85777	0.85916	0.86365	0.86847	0.87378
466	0.84487	0.85385	0.85801	0.85940	0.86388	0.86869	0.87399
467	0.84515	0.85409	0.85824	0.85963	0.86411	0.86891	0.87420
468	0.84541	0.85433	0.85848	0.85987	0.86433	0.86913	0.87440
469	0.84568	0.85457	0.85872	0.86010	0.86456	0.86934	0.87461
470	0.84593	0.85481	0.85896	0.86033	0.86479	0.86955	0.87482
471	0.84620	0.85505	0.85919	0.86056	0.86501	0.86977	0.87502
472	0.84644	0.85530	0.85942	0.86078	0.86523	0.86999	0.87523
473	0.84672	0.85553	0.85965	0.86101	0.86546	0.87020	0.87543
474	0.84696	0.85577	0.85989	0.86125	0.86568	0.87041	0.87563
475	0.84719	0.85602	0.86012	0.86148	0.86589	0.87063	0.87584
476	0.84744	0.85626	0.86035	0.86171	0.86611	0.87084	0.87604
477	0.84770	0.85649	0.86058	0.86193	0.86633	0.87105	0.87624
478	0.84796	0.85673	0.86081	0.86216	0.86655	0.87126	0.87645
479	0.84820	0.85696	0.86105	0.86239	0.86677	0.87147	0.87664
480	0.84846	0.85720	0.86127	0.86261	0.86699	0.87168	0.87684
481	0.84873	0.85744	0.86150	0.86284	0.86721	0.87189	0.87704
482	0.84897	0.85768	0.86172	0.86307	0.86743	0.87210	0.87724
483	0.84922	0.85791	0.86194	0.86329	0.86764	0.87230	0.87744
484	0.84947	0.85814	0.86217	0.86351	0.86785	0.87251	0.87763
485	0.84971	0.85837	0.86239	0.86373	0.86806	0.87271	0.87783
486	0.84995	0.85860	0.86262	0.86395	0.86828	0.87292	0.87802
487	0.85019	0.85883	0.86284	0.86417	0.86849	0.87312	0.87822
488	0.85044	0.85906	0.86306	0.86438	0.86870	0.87332	0.87841
489	0.85068	0.85928	0.86327	0.86460	0.86891	0.87352	0.87860
490	0.85092	0.85952	0.86349	0.86481	0.86912	0.87372	0.87880
491	0.85116	0.85975	0.86371	0.86503	0.86933	0.87393	0.87899
492	0.85139	0.85997	0.86393	0.86524	0.86953	0.87413	0.87918
493	0.85165	0.86019	0.86414	0.86546	0.86974	0.87432	0.87937
494	0.85188	0.86042	0.86436	0.86567	0.86995	0.87452	0.87956
495	0.85211	0.86064	0.86458	0.86589	0.87016	0.87473	0.87975
496	0.85236	0.86086	0.86480	0.86610	0.87036	0.87492	0.87994
497	0.85260	0.86108	0.86501	0.86632	0.87057	0.87512	0.88013
498	0.85281	0.86131	0.86523	0.86652	0.87077	0.87531	0.88032
499	0.85304	0.86153	0.86543	0.86674	0.87098	0.87551	0.88050
500	0.85328	0.86175	0.86565	0.86695	0.87118	0.87570	0.88069
501	0.85351	0.86196	0.86586	0.86716	0.87138	0.87590	0.88088
502	0.85374	0.86218	0.86608	0.86737	0.87159	0.87609	0.88106
503	0.85397	0.86239	0.86629	0.86758	0.87178	0.87628	0.88124
504	0.85419	0.86260	0.86650	0.86779	0.87199	0.87647	0.88143
505	0.85443	0.86282	0.86671	0.86800	0.87218	0.87667	0.88161
506	0.85466	0.86304	0.86692	0.86821	0.87238	0.87686	0.88179
507	0.85489	0.86325	0.86712	0.86841	0.87258	0.87705	0.88197
508	0.85514	0.86347	0.86733	0.86862	0.87278	0.87724	0.88215
509	0.85536	0.86369	0.86754	0.86882	0.87297	0.87743	0.88234
510	0.85557	0.86389	0.86775	0.86902	0.87317	0.87761	0.88251
511	0.85579	0.86411	0.86794	0.86922	0.87336	0.87780	0.88269
512	0.85600	0.86432	0.86815	0.86943	0.87356	0.87799	0.88287
513	0.85623	0.86452	0.86835	0.86963	0.87375	0.87817	0.88305
514	0.85644	0.86473	0.86856	0.86983	0.87394	0.87836	0.88322

N	0.1%	0.5%	1%	1.25%	2.5%	5%	10%
515	0.85667	0.86493	0.86876	0.87003	0.87414	0.87855	0.88340
516	0.85689	0.86514	0.86896	0.87023	0.87432	0.87873	0.88358
517	0.85712	0.86535	0.86916	0.87043	0.87452	0.87892	0.88375
518	0.85733	0.86555	0.86936	0.87062	0.87471	0.87910	0.88393
519	0.85756	0.86576	0.86956	0.87082	0.87490	0.87928	0.88410
520	0.85778	0.86598	0.86976	0.87102	0.87509	0.87946	0.88428
521	0.85801	0.86618	0.86996	0.87121	0.87528	0.87965	0.88445
522	0.85823	0.86638	0.87016	0.87141	0.87546	0.87983	0.88462
523	0.85843	0.86659	0.87035	0.87160	0.87565	0.88001	0.88479
524	0.85865	0.86679	0.87055	0.87180	0.87584	0.88019	0.88496
525	0.85886	0.86699	0.87074	0.87199	0.87603	0.88037	0.88514
526	0.85909	0.86720	0.87094	0.87218	0.87621	0.88055	0.88531
527	0.85930	0.86741	0.87114	0.87238	0.87640	0.88072	0.88548
528	0.85951	0.86760	0.87132	0.87257	0.87658	0.88090	0.88565
529	0.85970	0.86779	0.87152	0.87276	0.87676	0.88108	0.88582
530	0.85992	0.86799	0.87171	0.87295	0.87695	0.88125	0.88598
531	0.86014	0.86819	0.87190	0.87313	0.87713	0.88143	0.88615
532	0.86035	0.86839	0.87210	0.87332	0.87731	0.88160	0.88632
533	0.86056	0.86860	0.87229	0.87351	0.87749	0.88177	0.88648
534	0.86079	0.86879	0.87247	0.87370	0.87768	0.88195	0.88665
535	0.86099	0.86898	0.87266	0.87389	0.87786	0.88212	0.88682
536	0.86120	0.86917	0.87284	0.87407	0.87804	0.88230	0.88698
537	0.86141	0.86936	0.87303	0.87426	0.87822	0.88247	0.88714
538	0.86161	0.86956	0.87322	0.87444	0.87839	0.88264	0.88731
539	0.86182	0.86975	0.87341	0.87463	0.87857	0.88281	0.88747
540	0.86203	0.86995	0.87359	0.87481	0.87875	0.88298	0.88763
541	0.86224	0.87013	0.87378	0.87499	0.87893	0.88315	0.88779
542	0.86244	0.87033	0.87396	0.87517	0.87910	0.88332	0.88796
543	0.86262	0.87051	0.87414	0.87536	0.87928	0.88349	0.88812
544	0.86281	0.87070	0.87433	0.87554	0.87945	0.88366	0.88828
545	0.86302	0.87088	0.87451	0.87571	0.87963	0.88383	0.88844
546	0.86322	0.87106	0.87469	0.87590	0.87980	0.88400	0.88860
547	0.86342	0.87124	0.87488	0.87608	0.87997	0.88416	0.88876
548	0.86362	0.87142	0.87505	0.87625	0.88015	0.88433	0.88892
549	0.86381	0.87161	0.87524	0.87643	0.88032	0.88449	0.88908
550	0.86401	0.87180	0.87542	0.87661	0.88049	0.88466	0.88924
551	0.86421	0.87198	0.87560	0.87679	0.88066	0.88482	0.88940
552	0.86441	0.87217	0.87577	0.87696	0.88083	0.88499	0.88955
553	0.86460	0.87235	0.87596	0.87714	0.88100	0.88515	0.88971
554	0.86480	0.87254	0.87613	0.87732	0.88118	0.88532	0.88987
555	0.86499	0.87273	0.87631	0.87750	0.88135	0.88548	0.89002
556	0.86520	0.87290	0.87648	0.87767	0.88151	0.88564	0.89017
557	0.86540	0.87309	0.87666	0.87784	0.88168	0.88580	0.89033
558	0.86560	0.87328	0.87683	0.87801	0.88185	0.88597	0.89048
559	0.86578	0.87346	0.87701	0.87818	0.88202	0.88613	0.89064
560	0.86597	0.87364	0.87719	0.87836	0.88218	0.88629	0.89079
561	0.86617	0.87382	0.87736	0.87853	0.88235	0.88645	0.89094
562	0.86635	0.87399	0.87754	0.87871	0.88252	0.88660	0.89109
563	0.86655	0.87418	0.87771	0.87888	0.88268	0.88676	0.89124
564	0.86675	0.87436	0.87788	0.87906	0.88285	0.88692	0.89140
565	0.86693	0.87454	0.87806	0.87923	0.88301	0.88708	0.89155
566	0.86711	0.87472	0.87823	0.87940	0.88318	0.88723	0.89170
567	0.86730	0.87490	0.87840	0.87956	0.88333	0.88739	0.89185
568	0.86749	0.87507	0.87857	0.87973	0.88350	0.88755	0.89200
569	0.86768	0.87524	0.87874	0.87989	0.88366	0.88770	0.89214
570	0.86785	0.87541	0.87891	0.88006	0.88382	0.88786	0.89229
571	0.86803	0.87559	0.87908	0.88024	0.88398	0.88801	0.89244
572	0.86822	0.87576	0.87925	0.88040	0.88414	0.88816	0.89259
573	0.86839	0.87594	0.87941	0.88057	0.88430	0.88832	0.89273
574	0.86859	0.87610	0.87958	0.88073	0.88446	0.88847	0.89288
575	0.86876	0.87628	0.87975	0.88089	0.88462	0.88862	0.89303
576	0.86896	0.87644	0.87991	0.88106	0.88478	0.88878	0.89317
577	0.86913	0.87661	0.88008	0.88122	0.88494	0.88893	0.89332
578	0.86929	0.87678	0.88024	0.88139	0.88509	0.88908	0.89346
579	0.86947	0.87695	0.88040	0.88155	0.88525	0.88923	0.89360
580	0.86967	0.87712	0.88057	0.88171	0.88541	0.88938	0.89375
581	0.86985	0.87729	0.88074	0.88188	0.88556	0.88953	0.89389
582	0.87005	0.87746	0.88090	0.88203	0.88572	0.88968	0.89404
583	0.87022	0.87763	0.88106	0.88220	0.88587	0.88983	0.89418
584	0.87041	0.87780	0.88122	0.88236	0.88603	0.88998	0.89432
585	0.87060	0.87797	0.88138	0.88252	0.88619	0.89013	0.89446
586	0.87078	0.87812	0.88153	0.88267	0.88634	0.89028	0.89461
587	0.87096	0.87829	0.88170	0.88283	0.88650	0.89042	0.89474
588	0.87112	0.87846	0.88186	0.88299	0.88665	0.89057	0.89489

N	0.1%	0.5%	1%	1.25%	2.5%	5%	10%
589	0.87129	0.87863	0.88202	0.88315	0.88680	0.89072	0.89503
590	0.87146	0.87878	0.88218	0.88331	0.88695	0.89086	0.89517
591	0.87164	0.87894	0.88234	0.88347	0.88711	0.89101	0.89531
592	0.87181	0.87911	0.88250	0.88362	0.88725	0.89115	0.89544
593	0.87196	0.87928	0.88266	0.88378	0.88741	0.89130	0.89558
594	0.87216	0.87944	0.88281	0.88393	0.88756	0.89144	0.89572
595	0.87231	0.87960	0.88297	0.88409	0.88771	0.89159	0.89586
596	0.87250	0.87977	0.88313	0.88425	0.88786	0.89173	0.89600
597	0.87267	0.87992	0.88329	0.88440	0.88801	0.89187	0.89613
598	0.87285	0.88008	0.88345	0.88455	0.88816	0.89202	0.89627
599	0.87301	0.88025	0.88360	0.88471	0.88830	0.89216	0.89641
600	0.87317	0.88040	0.88376	0.88486	0.88845	0.89230	0.89654
601	0.87333	0.88056	0.88391	0.88501	0.88860	0.89244	0.89668
602	0.87351	0.88072	0.88406	0.88516	0.88874	0.89259	0.89681
603	0.87369	0.88088	0.88421	0.88531	0.88889	0.89273	0.89695
604	0.87385	0.88103	0.88436	0.88546	0.88904	0.89287	0.89708
605	0.87400	0.88120	0.88452	0.88562	0.88918	0.89301	0.89722
606	0.87417	0.88135	0.88467	0.88577	0.88932	0.89315	0.89735
607	0.87435	0.88151	0.88483	0.88592	0.88947	0.89329	0.89748
608	0.87452	0.88167	0.88498	0.88607	0.88961	0.89342	0.89761
609	0.87468	0.88182	0.88512	0.88622	0.88976	0.89356	0.89775
610	0.87483	0.88198	0.88528	0.88637	0.88990	0.89370	0.89788
611	0.87498	0.88213	0.88542	0.88652	0.89005	0.89384	0.89801
612	0.87515	0.88228	0.88557	0.88666	0.89019	0.89397	0.89814
613	0.87530	0.88243	0.88572	0.88681	0.89034	0.89411	0.89828
614	0.87548	0.88258	0.88587	0.88696	0.89048	0.89425	0.89841
615	0.87565	0.88274	0.88602	0.88710	0.89062	0.89438	0.89854
616	0.87582	0.88289	0.88617	0.88725	0.89076	0.89452	0.89867
617	0.87599	0.88303	0.88632	0.88740	0.89090	0.89465	0.89880
618	0.87616	0.88319	0.88646	0.88754	0.89105	0.89479	0.89893
619	0.87632	0.88335	0.88661	0.88769	0.89119	0.89492	0.89906
620	0.87648	0.88351	0.88676	0.88784	0.89132	0.89506	0.89918
621	0.87662	0.88365	0.88690	0.88798	0.89147	0.89519	0.89931
622	0.87679	0.88380	0.88705	0.88812	0.89160	0.89532	0.89944
623	0.87695	0.88395	0.88720	0.88827	0.89174	0.89546	0.89957
624	0.87710	0.88410	0.88734	0.88841	0.89188	0.89559	0.89970
625	0.87726	0.88425	0.88748	0.88855	0.89202	0.89572	0.89982
626	0.87743	0.88439	0.88762	0.88869	0.89216	0.89585	0.89995
627	0.87760	0.88454	0.88776	0.88883	0.89229	0.89598	0.90007
628	0.87777	0.88469	0.88791	0.88898	0.89243	0.89612	0.90020
629	0.87792	0.88483	0.88805	0.88912	0.89256	0.89625	0.90033
630	0.87807	0.88498	0.88819	0.88926	0.89270	0.89638	0.90045
631	0.87824	0.88513	0.88833	0.88939	0.89284	0.89651	0.90057
632	0.87839	0.88527	0.88847	0.88953	0.89297	0.89664	0.90070
633	0.87853	0.88542	0.88861	0.88968	0.89311	0.89677	0.90082
634	0.87868	0.88556	0.88875	0.88981	0.89324	0.89690	0.90095
635	0.87883	0.88570	0.88889	0.88995	0.89337	0.89703	0.90107
636	0.87898	0.88585	0.88904	0.89009	0.89351	0.89716	0.90119
637	0.87914	0.88598	0.88918	0.89023	0.89364	0.89729	0.90131
638	0.87931	0.88613	0.88932	0.89037	0.89377	0.89741	0.90144
639	0.87946	0.88628	0.88945	0.89050	0.89390	0.89754	0.90156
640	0.87959	0.88642	0.88959	0.89064	0.89404	0.89767	0.90168
641	0.87974	0.88656	0.88972	0.89077	0.89417	0.89780	0.90180
642	0.87991	0.88670	0.88986	0.89091	0.89430	0.89792	0.90192
643	0.88006	0.88685	0.89000	0.89105	0.89443	0.89805	0.90204
644	0.88019	0.88699	0.89014	0.89118	0.89456	0.89817	0.90216
645	0.88036	0.88712	0.89027	0.89132	0.89469	0.89830	0.90228
646	0.88050	0.88726	0.89040	0.89145	0.89482	0.89842	0.90240
647	0.88065	0.88740	0.89054	0.89158	0.89495	0.89855	0.90252
648	0.88080	0.88755	0.89068	0.89171	0.89508	0.89867	0.90263
649	0.88095	0.88769	0.89081	0.89185	0.89521	0.89879	0.90275
650	0.88109	0.88783	0.89094	0.89198	0.89534	0.89892	0.90287
651	0.88125	0.88797	0.89108	0.89211	0.89546	0.89904	0.90299
652	0.88140	0.88810	0.89121	0.89224	0.89559	0.89917	0.90311
653	0.88155	0.88824	0.89135	0.89238	0.89572	0.89929	0.90323
654	0.88169	0.88838	0.89148	0.89251	0.89585	0.89941	0.90334
655	0.88184	0.88852	0.89161	0.89264	0.89597	0.89953	0.90346
656	0.88198	0.88865	0.89174	0.89277	0.89610	0.89965	0.90358
657	0.88212	0.88879	0.89188	0.89290	0.89623	0.89978	0.90369
658	0.88226	0.88892	0.89201	0.89303	0.89635	0.89990	0.90381
659	0.88239	0.88906	0.89214	0.89316	0.89648	0.90002	0.90392
660	0.88254	0.88920	0.89227	0.89329	0.89660	0.90014	0.90404
661	0.88269	0.88934	0.89240	0.89342	0.89673	0.90026	0.90415
662	0.88283	0.88947	0.89253	0.89355	0.89685	0.90038	0.90427

N	0.1%	0.5%	1%	1.25%	2.5%	5%	10%
663	0.88300	0.88960	0.89266	0.89368	0.89697	0.90050	0.90438
664	0.88312	0.88974	0.89279	0.89381	0.89710	0.90062	0.90450
665	0.88325	0.88986	0.89291	0.89394	0.89722	0.90073	0.90461
666	0.88340	0.89000	0.89304	0.89406	0.89735	0.90085	0.90472
667	0.88354	0.89013	0.89317	0.89419	0.89747	0.90097	0.90484
668	0.88368	0.89026	0.89330	0.89432	0.89759	0.90109	0.90495
669	0.88384	0.89040	0.89343	0.89444	0.89771	0.90120	0.90506
670	0.88397	0.89053	0.89355	0.89457	0.89783	0.90132	0.90517
671	0.88412	0.89066	0.89368	0.89470	0.89796	0.90144	0.90528
672	0.88425	0.89079	0.89380	0.89482	0.89808	0.90156	0.90540
673	0.88439	0.89092	0.89393	0.89495	0.89820	0.90167	0.90551
674	0.88452	0.89105	0.89406	0.89507	0.89832	0.90179	0.90562
675	0.88466	0.89118	0.89418	0.89520	0.89844	0.90190	0.90573
676	0.88479	0.89131	0.89431	0.89532	0.89856	0.90202	0.90584
677	0.88493	0.89144	0.89444	0.89545	0.89868	0.90213	0.90595
678	0.88507	0.89157	0.89457	0.89557	0.89879	0.90225	0.90606
679	0.88521	0.89170	0.89470	0.89570	0.89891	0.90236	0.90617
680	0.88535	0.89183	0.89482	0.89582	0.89903	0.90248	0.90628
681	0.88551	0.89196	0.89494	0.89594	0.89915	0.90259	0.90639
682	0.88563	0.89209	0.89506	0.89607	0.89927	0.90271	0.90650
683	0.88577	0.89221	0.89519	0.89619	0.89939	0.90282	0.90661
684	0.88590	0.89234	0.89530	0.89631	0.89951	0.90293	0.90671
685	0.88604	0.89247	0.89543	0.89643	0.89963	0.90305	0.90682
686	0.88617	0.89260	0.89555	0.89655	0.89974	0.90316	0.90693
687	0.88630	0.89272	0.89567	0.89667	0.89986	0.90327	0.90704
688	0.88644	0.89285	0.89580	0.89679	0.89998	0.90338	0.90714
689	0.88657	0.89297	0.89592	0.89691	0.90009	0.90350	0.90725
690	0.88670	0.89310	0.89604	0.89703	0.90021	0.90361	0.90735
691	0.88684	0.89322	0.89616	0.89715	0.90032	0.90372	0.90746
692	0.88697	0.89335	0.89628	0.89727	0.90043	0.90383	0.90757
693	0.88710	0.89347	0.89640	0.89739	0.90055	0.90394	0.90767
694	0.88723	0.89359	0.89653	0.89751	0.90067	0.90405	0.90778
695	0.88735	0.89372	0.89665	0.89763	0.90078	0.90416	0.90788
696	0.88748	0.89385	0.89677	0.89775	0.90090	0.90427	0.90799
697	0.88763	0.89397	0.89689	0.89786	0.90101	0.90438	0.90809
698	0.88776	0.89408	0.89701	0.89798	0.90112	0.90449	0.90820
699	0.88787	0.89421	0.89713	0.89810	0.90124	0.90460	0.90830
700	0.88801	0.89434	0.89725	0.89822	0.90135	0.90470	0.90840
701	0.88813	0.89446	0.89736	0.89833	0.90146	0.90481	0.90851
702	0.88827	0.89458	0.89747	0.89845	0.90157	0.90492	0.90861
703	0.88839	0.89469	0.89759	0.89857	0.90168	0.90503	0.90872
704	0.88853	0.89481	0.89771	0.89868	0.90179	0.90513	0.90882
705	0.88866	0.89493	0.89783	0.89880	0.90191	0.90524	0.90892
706	0.88878	0.89505	0.89795	0.89891	0.90202	0.90535	0.90902
707	0.88890	0.89516	0.89806	0.89903	0.90213	0.90546	0.90912
708	0.88902	0.89529	0.89818	0.89914	0.90224	0.90556	0.90923
709	0.88915	0.89541	0.89829	0.89925	0.90235	0.90567	0.90933
710	0.88928	0.89554	0.89841	0.89937	0.90246	0.90578	0.90943
711	0.88940	0.89565	0.89852	0.89948	0.90257	0.90588	0.90953
712	0.88953	0.89578	0.89863	0.89959	0.90268	0.90599	0.90963
713	0.88965	0.89589	0.89875	0.89971	0.90279	0.90609	0.90973
714	0.88978	0.89601	0.89887	0.89982	0.90290	0.90620	0.90983
715	0.88989	0.89612	0.89898	0.89993	0.90301	0.90630	0.90993
716	0.89004	0.89623	0.89909	0.90005	0.90312	0.90641	0.91003
717	0.89016	0.89635	0.89920	0.90016	0.90323	0.90651	0.91013
718	0.89028	0.89647	0.89931	0.90027	0.90334	0.90662	0.91023
719	0.89041	0.89659	0.89943	0.90038	0.90344	0.90672	0.91033
720	0.89053	0.89670	0.89955	0.90050	0.90355	0.90682	0.91043
721	0.89067	0.89682	0.89966	0.90061	0.90366	0.90693	0.91053
722	0.89079	0.89694	0.89978	0.90071	0.90377	0.90703	0.91063
723	0.89090	0.89705	0.89988	0.90083	0.90387	0.90713	0.91073
724	0.89102	0.89717	0.90000	0.90094	0.90398	0.90723	0.91082
725	0.89114	0.89729	0.90011	0.90105	0.90408	0.90733	0.91092
726	0.89126	0.89740	0.90022	0.90116	0.90419	0.90743	0.91102
727	0.89138	0.89752	0.90033	0.90127	0.90430	0.90754	0.91112
728	0.89150	0.89763	0.90044	0.90138	0.90440	0.90764	0.91121
729	0.89163	0.89774	0.90055	0.90148	0.90451	0.90774	0.91131
730	0.89174	0.89786	0.90066	0.90159	0.90461	0.90784	0.91140
731	0.89186	0.89797	0.90078	0.90170	0.90472	0.90794	0.91150
732	0.89198	0.89808	0.90088	0.90181	0.90482	0.90804	0.91160
733	0.89210	0.89820	0.90099	0.90192	0.90493	0.90814	0.91169
734	0.89223	0.89832	0.90110	0.90203	0.90503	0.90824	0.91179
735	0.89235	0.89843	0.90121	0.90214	0.90513	0.90834	0.91188
736	0.89246	0.89854	0.90132	0.90225	0.90524	0.90844	0.91198

N	0.1%	0.5%	1%	1.25%	2.5%	5%	10%
737	0.89258	0.89865	0.90143	0.90235	0.90534	0.90854	0.91207
738	0.89271	0.89876	0.90154	0.90246	0.90545	0.90864	0.91217
739	0.89282	0.89887	0.90164	0.90257	0.90555	0.90874	0.91226
740	0.89293	0.89898	0.90175	0.90267	0.90565	0.90884	0.91236
741	0.89306	0.89909	0.90186	0.90278	0.90575	0.90894	0.91245
742	0.89318	0.89920	0.90197	0.90289	0.90585	0.90904	0.91255
743	0.89330	0.89931	0.90207	0.90299	0.90595	0.90913	0.91264
744	0.89342	0.89942	0.90218	0.90309	0.90606	0.90923	0.91273
745	0.89353	0.89953	0.90228	0.90320	0.90616	0.90933	0.91283
746	0.89365	0.89963	0.90239	0.90331	0.90626	0.90942	0.91292
747	0.89377	0.89974	0.90249	0.90341	0.90636	0.90952	0.91301
748	0.89387	0.89985	0.90260	0.90352	0.90646	0.90962	0.91310
749	0.89399	0.89996	0.90270	0.90362	0.90656	0.90971	0.91320
750	0.89410	0.90007	0.90281	0.90372	0.90666	0.90981	0.91329
751	0.89423	0.90017	0.90291	0.90383	0.90676	0.90991	0.91338
752	0.89436	0.90029	0.90302	0.90393	0.90686	0.91000	0.91347
753	0.89448	0.90039	0.90312	0.90404	0.90696	0.91010	0.91356
754	0.89459	0.90049	0.90322	0.90414	0.90706	0.91019	0.91365
755	0.89470	0.90060	0.90333	0.90424	0.90716	0.91029	0.91374
756	0.89482	0.90072	0.90343	0.90434	0.90726	0.91038	0.91384
757	0.89493	0.90082	0.90354	0.90445	0.90736	0.91048	0.91393
758	0.89505	0.90093	0.90364	0.90455	0.90746	0.91057	0.91402
759	0.89516	0.90103	0.90375	0.90465	0.90756	0.91067	0.91411
760	0.89527	0.90114	0.90385	0.90475	0.90765	0.91076	0.91420
761	0.89538	0.90123	0.90396	0.90485	0.90775	0.91086	0.91429
762	0.89549	0.90134	0.90406	0.90495	0.90785	0.91095	0.91438
763	0.89559	0.90145	0.90416	0.90505	0.90795	0.91104	0.91447
764	0.89570	0.90155	0.90426	0.90515	0.90804	0.91114	0.91456
765	0.89581	0.90165	0.90436	0.90525	0.90814	0.91123	0.91464
766	0.89593	0.90175	0.90446	0.90535	0.90823	0.91132	0.91473
767	0.89604	0.90186	0.90455	0.90545	0.90833	0.91142	0.91482
768	0.89615	0.90197	0.90466	0.90555	0.90843	0.91151	0.91491
769	0.89625	0.90207	0.90475	0.90565	0.90852	0.91160	0.91500
770	0.89637	0.90217	0.90485	0.90575	0.90862	0.91169	0.91509
771	0.89647	0.90227	0.90495	0.90585	0.90871	0.91178	0.91518
772	0.89658	0.90237	0.90505	0.90594	0.90881	0.91187	0.91526
773	0.89669	0.90248	0.90515	0.90604	0.90890	0.91196	0.91535
774	0.89680	0.90258	0.90525	0.90614	0.90900	0.91206	0.91544
775	0.89691	0.90268	0.90535	0.90624	0.90909	0.91215	0.91552
776	0.89703	0.90278	0.90545	0.90633	0.90918	0.91224	0.91561
777	0.89713	0.90289	0.90555	0.90643	0.90928	0.91233	0.91570
778	0.89725	0.90299	0.90565	0.90653	0.90937	0.91242	0.91578
779	0.89735	0.90308	0.90575	0.90662	0.90947	0.91251	0.91587
780	0.89746	0.90319	0.90585	0.90672	0.90956	0.91260	0.91595
781	0.89757	0.90329	0.90594	0.90682	0.90965	0.91269	0.91604
782	0.89767	0.90339	0.90605	0.90691	0.90974	0.91278	0.91613
783	0.89778	0.90349	0.90614	0.90701	0.90984	0.91287	0.91621
784	0.89789	0.90359	0.90624	0.90711	0.90993	0.91296	0.91630
785	0.89798	0.90369	0.90633	0.90720	0.91002	0.91304	0.91638
786	0.89807	0.90379	0.90643	0.90730	0.91011	0.91313	0.91647
787	0.89818	0.90389	0.90653	0.90740	0.91021	0.91322	0.91655
788	0.89830	0.90399	0.90663	0.90749	0.91030	0.91331	0.91664
789	0.89840	0.90409	0.90672	0.90758	0.91039	0.91340	0.91672
790	0.89851	0.90419	0.90681	0.90768	0.91048	0.91349	0.91680
791	0.89861	0.90428	0.90691	0.90777	0.91057	0.91358	0.91689
792	0.89871	0.90438	0.90701	0.90787	0.91066	0.91366	0.91697
793	0.89882	0.90448	0.90710	0.90796	0.91075	0.91375	0.91706
794	0.89893	0.90458	0.90719	0.90806	0.91084	0.91384	0.91714
795	0.89902	0.90468	0.90729	0.90815	0.91093	0.91392	0.91722
796	0.89913	0.90478	0.90738	0.90824	0.91102	0.91401	0.91731
797	0.89924	0.90488	0.90747	0.90833	0.91111	0.91410	0.91739
798	0.89934	0.90497	0.90757	0.90843	0.91120	0.91418	0.91747
799	0.89944	0.90506	0.90766	0.90852	0.91129	0.91427	0.91755
800	0.89955	0.90516	0.90775	0.90861	0.91138	0.91436	0.91764
801	0.89965	0.90526	0.90785	0.90871	0.91147	0.91444	0.91772
802	0.89976	0.90535	0.90794	0.90880	0.91156	0.91453	0.91780
803	0.89985	0.90544	0.90803	0.90889	0.91165	0.91461	0.91788
804	0.89995	0.90554	0.90813	0.90898	0.91174	0.91470	0.91796
805	0.90005	0.90563	0.90822	0.90907	0.91183	0.91478	0.91804
806	0.90015	0.90573	0.90831	0.90916	0.91192	0.91487	0.91813
807	0.90025	0.90582	0.90840	0.90926	0.91201	0.91495	0.91821
808	0.90035	0.90592	0.90849	0.90935	0.91209	0.91504	0.91829
809	0.90045	0.90601	0.90859	0.90944	0.91218	0.91512	0.91837
810	0.90055	0.90611	0.90868	0.90953	0.91227	0.91521	0.91845

N	0.1%	0.5%	1%	1.25%	2.5%	5%	10%
811	0.90067	0.90620	0.90877	0.90962	0.91236	0.91529	0.91853
812	0.90076	0.90630	0.90886	0.90971	0.91244	0.91538	0.91861
813	0.90085	0.90639	0.90895	0.90980	0.91253	0.91546	0.91869
814	0.90095	0.90649	0.90904	0.90989	0.91262	0.91554	0.91877
815	0.90105	0.90658	0.90913	0.90998	0.91271	0.91563	0.91885
816	0.90114	0.90668	0.90922	0.91007	0.91279	0.91571	0.91893
817	0.90124	0.90677	0.90931	0.91015	0.91288	0.91579	0.91901
818	0.90134	0.90686	0.90940	0.91024	0.91297	0.91587	0.91909
819	0.90144	0.90696	0.90949	0.91033	0.91305	0.91596	0.91916
820	0.90155	0.90705	0.90958	0.91042	0.91314	0.91604	0.91924
821	0.90165	0.90714	0.90967	0.91051	0.91322	0.91612	0.91932
822	0.90176	0.90723	0.90976	0.91060	0.91331	0.91620	0.91940
823	0.90184	0.90733	0.90985	0.91069	0.91339	0.91628	0.91948
824	0.90194	0.90742	0.90993	0.91077	0.91348	0.91637	0.91956
825	0.90204	0.90751	0.91002	0.91086	0.91356	0.91645	0.91963
826	0.90213	0.90760	0.91011	0.91095	0.91364	0.91653	0.91971
827	0.90223	0.90769	0.91020	0.91104	0.91373	0.91661	0.91979
828	0.90233	0.90778	0.91029	0.91112	0.91381	0.91669	0.91987
829	0.90243	0.90787	0.91037	0.91121	0.91390	0.91677	0.91995
830	0.90252	0.90796	0.91045	0.91129	0.91398	0.91685	0.92002
831	0.90262	0.90804	0.91054	0.91138	0.91406	0.91693	0.92010
832	0.90271	0.90813	0.91063	0.91146	0.91415	0.91701	0.92018
833	0.90281	0.90823	0.91072	0.91155	0.91423	0.91709	0.92025
834	0.90290	0.90832	0.91080	0.91163	0.91431	0.91717	0.92033
835	0.90300	0.90842	0.91089	0.91172	0.91439	0.91725	0.92040
836	0.90309	0.90851	0.91098	0.91180	0.91448	0.91733	0.92048
837	0.90318	0.90860	0.91107	0.91189	0.91456	0.91741	0.92056
838	0.90328	0.90869	0.91115	0.91198	0.91464	0.91749	0.92063
839	0.90337	0.90878	0.91124	0.91206	0.91473	0.91757	0.92071
840	0.90346	0.90887	0.91132	0.91214	0.91481	0.91765	0.92079
841	0.90356	0.90895	0.91141	0.91223	0.91489	0.91773	0.92086
842	0.90367	0.90904	0.91150	0.91231	0.91497	0.91781	0.92093
843	0.90376	0.90913	0.91158	0.91240	0.91505	0.91789	0.92101
844	0.90385	0.90921	0.91166	0.91248	0.91513	0.91796	0.92108
845	0.90395	0.90930	0.91175	0.91257	0.91521	0.91804	0.92116
846	0.90404	0.90939	0.91183	0.91265	0.91529	0.91812	0.92123
847	0.90413	0.90947	0.91192	0.91274	0.91538	0.91820	0.92131
848	0.90424	0.90956	0.91200	0.91282	0.91546	0.91827	0.92138
849	0.90432	0.90964	0.91209	0.91290	0.91554	0.91835	0.92146
850	0.90441	0.90973	0.91217	0.91298	0.91562	0.91843	0.92153
851	0.90450	0.90981	0.91225	0.91307	0.91569	0.91851	0.92161
852	0.90460	0.90990	0.91234	0.91315	0.91578	0.91858	0.92168
853	0.90470	0.90999	0.91242	0.91323	0.91586	0.91866	0.92175
854	0.90479	0.91007	0.91250	0.91331	0.91594	0.91874	0.92183
855	0.90489	0.91016	0.91259	0.91340	0.91602	0.91881	0.92190
856	0.90498	0.91025	0.91267	0.91348	0.91610	0.91889	0.92197
857	0.90507	0.91033	0.91275	0.91356	0.91618	0.91897	0.92204
858	0.90517	0.91042	0.91284	0.91364	0.91626	0.91904	0.92212
859	0.90525	0.91051	0.91292	0.91373	0.91634	0.91912	0.92219
860	0.90535	0.91059	0.91300	0.91381	0.91641	0.91920	0.92226
861	0.90544	0.91067	0.91309	0.91388	0.91649	0.91927	0.92233
862	0.90553	0.91076	0.91317	0.91397	0.91657	0.91935	0.92241
863	0.90562	0.91084	0.91325	0.91405	0.91665	0.91942	0.92248
864	0.90571	0.91093	0.91333	0.91413	0.91673	0.91950	0.92255
865	0.90579	0.91101	0.91341	0.91421	0.91680	0.91957	0.92262
866	0.90588	0.91109	0.91349	0.91429	0.91688	0.91965	0.92269
867	0.90596	0.91118	0.91357	0.91437	0.91696	0.91972	0.92277
868	0.90605	0.91126	0.91365	0.91445	0.91703	0.91980	0.92284
869	0.90614	0.91135	0.91374	0.91453	0.91711	0.91987	0.92291
870	0.90624	0.91143	0.91382	0.91461	0.91719	0.91995	0.92298
871	0.90633	0.91152	0.91390	0.91469	0.91727	0.92002	0.92305
872	0.90641	0.91160	0.91397	0.91477	0.91734	0.92009	0.92312
873	0.90651	0.91168	0.91406	0.91485	0.91742	0.92017	0.92319
874	0.90659	0.91176	0.91414	0.91493	0.91750	0.92024	0.92326
875	0.90668	0.91184	0.91422	0.91501	0.91757	0.92031	0.92333
876	0.90675	0.91193	0.91429	0.91508	0.91765	0.92039	0.92340
877	0.90684	0.91201	0.91438	0.91516	0.91773	0.92046	0.92347
878	0.90693	0.91209	0.91446	0.91524	0.91780	0.92053	0.92354
879	0.90702	0.91217	0.91453	0.91532	0.91787	0.92061	0.92361
880	0.90710	0.91225	0.91461	0.91539	0.91795	0.92068	0.92369
881	0.90719	0.91233	0.91469	0.91547	0.91802	0.92075	0.92376
882	0.90727	0.91241	0.91477	0.91555	0.91810	0.92082	0.92383
883	0.90735	0.91249	0.91484	0.91563	0.91817	0.92090	0.92389
884	0.90744	0.91257	0.91492	0.91570	0.91825	0.92097	0.92396

N	0.1%	0.5%	1%	1.25%	2.5%	5%	10%
885	0.90753	0.91265	0.91500	0.91578	0.91832	0.92104	0.92403
886	0.90762	0.91273	0.91508	0.91586	0.91840	0.92111	0.92410
887	0.90771	0.91281	0.91516	0.91594	0.91847	0.92119	0.92417
888	0.90780	0.91290	0.91523	0.91601	0.91855	0.92126	0.92424
889	0.90787	0.91297	0.91531	0.91609	0.91862	0.92133	0.92431
890	0.90796	0.91306	0.91539	0.91617	0.91870	0.92140	0.92437
891	0.90805	0.91314	0.91547	0.91625	0.91877	0.92147	0.92444
892	0.90813	0.91322	0.91554	0.91632	0.91884	0.92154	0.92451
893	0.90822	0.91330	0.91562	0.91640	0.91892	0.92161	0.92458
894	0.90830	0.91338	0.91569	0.91647	0.91899	0.92169	0.92464
895	0.90839	0.91345	0.91577	0.91654	0.91906	0.92176	0.92471
896	0.90847	0.91353	0.91585	0.91662	0.91914	0.92183	0.92478
897	0.90855	0.91361	0.91593	0.91670	0.91921	0.92190	0.92485
898	0.90864	0.91369	0.91600	0.91677	0.91928	0.92197	0.92492
899	0.90873	0.91377	0.91608	0.91685	0.91936	0.92204	0.92498
900	0.90881	0.91385	0.91616	0.91692	0.91943	0.92211	0.92505
901	0.90890	0.91393	0.91623	0.91700	0.91950	0.92218	0.92512
902	0.90897	0.91401	0.91631	0.91708	0.91957	0.92225	0.92518
903	0.90905	0.91409	0.91638	0.91715	0.91964	0.92232	0.92525
904	0.90913	0.91416	0.91646	0.91723	0.91972	0.92239	0.92532
905	0.90922	0.91424	0.91654	0.91730	0.91979	0.92246	0.92538
906	0.90930	0.91432	0.91662	0.91738	0.91986	0.92252	0.92545
907	0.90937	0.91439	0.91669	0.91745	0.91993	0.92259	0.92552
908	0.90946	0.91447	0.91677	0.91753	0.92000	0.92266	0.92558
909	0.90954	0.91455	0.91684	0.91760	0.92008	0.92273	0.92565
910	0.90962	0.91463	0.91692	0.91767	0.92015	0.92280	0.92571
911	0.90971	0.91470	0.91699	0.91775	0.92022	0.92287	0.92578
912	0.90978	0.91478	0.91707	0.91782	0.92029	0.92294	0.92584
913	0.90986	0.91486	0.91714	0.91790	0.92036	0.92300	0.92591
914	0.90994	0.91493	0.91721	0.91797	0.92043	0.92307	0.92597
915	0.91002	0.91501	0.91729	0.91804	0.92051	0.92314	0.92604
916	0.91011	0.91508	0.91736	0.91811	0.92057	0.92321	0.92610
917	0.91019	0.91515	0.91743	0.91818	0.92064	0.92328	0.92617
918	0.91027	0.91523	0.91750	0.91826	0.92071	0.92335	0.92623
919	0.91036	0.91530	0.91758	0.91833	0.92078	0.92341	0.92630
920	0.91043	0.91538	0.91765	0.91840	0.92085	0.92348	0.92636
921	0.91052	0.91545	0.91772	0.91847	0.92093	0.92355	0.92643
922	0.91060	0.91553	0.91780	0.91855	0.92099	0.92361	0.92649
923	0.91067	0.91560	0.91787	0.91862	0.92106	0.92368	0.92655
924	0.91075	0.91568	0.91794	0.91869	0.92113	0.92375	0.92662
925	0.91084	0.91576	0.91801	0.91877	0.92120	0.92381	0.92668
926	0.91092	0.91583	0.91808	0.91884	0.92127	0.92388	0.92675
927	0.91100	0.91590	0.91816	0.91891	0.92134	0.92395	0.92681
928	0.91108	0.91597	0.91823	0.91898	0.92141	0.92401	0.92687
929	0.91116	0.91605	0.91830	0.91905	0.92148	0.92408	0.92694
930	0.91123	0.91612	0.91837	0.91912	0.92154	0.92414	0.92700
931	0.91132	0.91619	0.91844	0.91919	0.92161	0.92421	0.92706
932	0.91140	0.91627	0.91851	0.91926	0.92168	0.92428	0.92713
933	0.91149	0.91635	0.91858	0.91934	0.92175	0.92434	0.92719
934	0.91156	0.91642	0.91866	0.91940	0.92182	0.92441	0.92725
935	0.91163	0.91649	0.91873	0.91947	0.92189	0.92447	0.92731
936	0.91172	0.91657	0.91880	0.91954	0.92195	0.92454	0.92738
937	0.91179	0.91664	0.91887	0.91962	0.92202	0.92460	0.92744
938	0.91186	0.91671	0.91894	0.91969	0.92209	0.92467	0.92750
939	0.91194	0.91679	0.91901	0.91976	0.92216	0.92473	0.92756
940	0.91202	0.91686	0.91908	0.91983	0.92222	0.92480	0.92762
941	0.91210	0.91693	0.91916	0.91990	0.92229	0.92486	0.92769
942	0.91219	0.91701	0.91923	0.91997	0.92236	0.92493	0.92775
943	0.91226	0.91708	0.91930	0.92004	0.92243	0.92499	0.92781
944	0.91234	0.91715	0.91937	0.92011	0.92249	0.92506	0.92787
945	0.91241	0.91722	0.91944	0.92018	0.92256	0.92512	0.92793
946	0.91249	0.91729	0.91951	0.92025	0.92263	0.92519	0.92799
947	0.91256	0.91736	0.91957	0.92031	0.92269	0.92525	0.92805
948	0.91264	0.91744	0.91964	0.92038	0.92276	0.92531	0.92812
949	0.91272	0.91751	0.91971	0.92046	0.92282	0.92538	0.92818
950	0.91280	0.91758	0.91978	0.92052	0.92289	0.92544	0.92824
951	0.91287	0.91765	0.91985	0.92059	0.92295	0.92550	0.92830
952	0.91295	0.91772	0.91992	0.92066	0.92302	0.92557	0.92836
953	0.91302	0.91779	0.91999	0.92072	0.92309	0.92563	0.92842
954	0.91310	0.91786	0.92006	0.92079	0.92315	0.92569	0.92848
955	0.91316	0.91793	0.92012	0.92086	0.92322	0.92576	0.92854
956	0.91323	0.91800	0.92019	0.92093	0.92328	0.92582	0.92860
957	0.91331	0.91807	0.92026	0.92099	0.92335	0.92588	0.92866
958	0.91338	0.91814	0.92033	0.92106	0.92341	0.92595	0.92872

N	0.1%	0.5%	1%	1.25%	2.5%	5%	10%
959	0.91346	0.91822	0.92040	0.92113	0.92348	0.92601	0.92878
960	0.91352	0.91829	0.92047	0.92120	0.92354	0.92607	0.92884
961	0.91360	0.91836	0.92054	0.92126	0.92361	0.92613	0.92890
962	0.91367	0.91843	0.92060	0.92133	0.92367	0.92620	0.92896
963	0.91375	0.91850	0.92067	0.92139	0.92374	0.92626	0.92902
964	0.91382	0.91857	0.92074	0.92146	0.92380	0.92632	0.92908
965	0.91390	0.91863	0.92080	0.92153	0.92387	0.92638	0.92914
966	0.91397	0.91870	0.92087	0.92159	0.92393	0.92644	0.92920
967	0.91406	0.91877	0.92094	0.92166	0.92400	0.92650	0.92925
968	0.91413	0.91884	0.92101	0.92173	0.92406	0.92657	0.92931
969	0.91419	0.91891	0.92107	0.92179	0.92413	0.92663	0.92937
970	0.91427	0.91898	0.92114	0.92186	0.92419	0.92669	0.92943
971	0.91434	0.91905	0.92120	0.92192	0.92425	0.92675	0.92949
972	0.91440	0.91912	0.92127	0.92199	0.92432	0.92681	0.92955
973	0.91448	0.91919	0.92134	0.92206	0.92438	0.92687	0.92960
974	0.91456	0.91926	0.92141	0.92212	0.92444	0.92693	0.92966
975	0.91463	0.91932	0.92147	0.92219	0.92450	0.92699	0.92972
976	0.91470	0.91939	0.92154	0.92225	0.92456	0.92705	0.92978
977	0.91478	0.91946	0.92160	0.92232	0.92463	0.92712	0.92984
978	0.91484	0.91952	0.92167	0.92238	0.92469	0.92718	0.92990
979	0.91492	0.91959	0.92173	0.92245	0.92475	0.92724	0.92995
980	0.91499	0.91966	0.92179	0.92251	0.92482	0.92730	0.93001
981	0.91507	0.91973	0.92186	0.92257	0.92488	0.92736	0.93007
982	0.91513	0.91980	0.92193	0.92264	0.92494	0.92742	0.93013
983	0.91520	0.91986	0.92199	0.92270	0.92500	0.92748	0.93019
984	0.91528	0.91993	0.92206	0.92277	0.92506	0.92754	0.93024
985	0.91535	0.92000	0.92212	0.92283	0.92513	0.92760	0.93030
986	0.91543	0.92007	0.92218	0.92290	0.92519	0.92766	0.93036
987	0.91550	0.92013	0.92225	0.92296	0.92525	0.92772	0.93041
988	0.91556	0.92020	0.92231	0.92303	0.92531	0.92778	0.93047
989	0.91563	0.92026	0.92238	0.92309	0.92537	0.92784	0.93053
990	0.91570	0.92033	0.92244	0.92315	0.92544	0.92789	0.93058
991	0.91578	0.92040	0.92251	0.92321	0.92550	0.92795	0.93064
992	0.91585	0.92046	0.92257	0.92328	0.92556	0.92801	0.93070
993	0.91592	0.92053	0.92263	0.92334	0.92562	0.92807	0.93075
994	0.91599	0.92059	0.92270	0.92340	0.92568	0.92813	0.93081
995	0.91606	0.92066	0.92276	0.92347	0.92574	0.92819	0.93087
996	0.91613	0.92072	0.92283	0.92353	0.92581	0.92825	0.93092
997	0.91619	0.92078	0.92289	0.92359	0.92587	0.92831	0.93098
998	0.91627	0.92085	0.92295	0.92366	0.92593	0.92836	0.93103
999	0.91634	0.92092	0.92302	0.92372	0.92599	0.92842	0.93109
1000	0.91641	0.92098	0.92308	0.92378	0.92605	0.92848	0.93115

Table 4 – *GM7(4H3L or 3H4L)*. Critical values of upper significance levels for the Grubbs multiple-7 outlier test, applicable in situations where the four highest and three lowest or three highest and four lowest observations are simultaneously identified as outliers, denoted as *GM7(4H3L)* and *GM7(3H4L)*, respectively.

N	0.1%	0.5%	1%	1.25%	2.5%	5%	10%
9	0.00000	0.00000	0.00000	0.00000	0.00000	0.00002	0.00008
10	0.00001	0.00007	0.00014	0.00018	0.00037	0.00077	0.00168
11	0.00021	0.00065	0.00107	0.00126	0.00210	0.00356	0.00618
12	0.00098	0.00233	0.00341	0.00386	0.00575	0.00867	0.01339
13	0.00264	0.00534	0.00733	0.00812	0.01127	0.01585	0.02278
14	0.00534	0.00976	0.01276	0.01394	0.01845	0.02473	0.03377
15	0.00916	0.01544	0.01952	0.02109	0.02699	0.03490	0.04590
16	0.01397	0.02227	0.02743	0.02938	0.03657	0.04602	0.05880
17	0.01965	0.02997	0.03624	0.03856	0.04701	0.05782	0.07216
18	0.02615	0.03851	0.04572	0.04839	0.05795	0.07007	0.08582
19	0.03338	0.04753	0.05569	0.05869	0.06931	0.08253	0.09957
20	0.04113	0.05699	0.06604	0.06933	0.08095	0.09521	0.11330
21	0.04944	0.06689	0.07671	0.08023	0.09270	0.10788	0.12689
22	0.05797	0.07706	0.08753	0.09133	0.10455	0.12047	0.14034
23	0.06679	0.08726	0.09845	0.10246	0.11637	0.13301	0.15357
24	0.07582	0.09763	0.10945	0.11365	0.12809	0.14536	0.16654
25	0.08504	0.10802	0.12041	0.12472	0.13975	0.15757	0.17923
26	0.09439	0.11842	0.13125	0.13574	0.15126	0.16952	0.19163
27	0.10373	0.12879	0.14195	0.14655	0.16256	0.18121	0.20373
28	0.11311	0.13902	0.15260	0.15735	0.17366	0.19267	0.21554
29	0.12248	0.14913	0.16308	0.16804	0.18458	0.20385	0.22703
30	0.13172	0.15916	0.17340	0.17840	0.19527	0.21484	0.23820
31	0.14103	0.16905	0.18359	0.18860	0.20573	0.22559	0.24909

N	0.1%	0.5%	1%	1.25%	2.5%	5%	10%
32	0.15010	0.17879	0.19355	0.19868	0.21607	0.23603	0.25967
33	0.15932	0.18847	0.20340	0.20862	0.22607	0.24621	0.26998
34	0.16827	0.19788	0.21301	0.21829	0.23592	0.25617	0.28000
35	0.17715	0.20714	0.22249	0.22778	0.24550	0.26586	0.28975
36	0.18571	0.21627	0.23172	0.23701	0.25494	0.27532	0.29923
37	0.19452	0.22518	0.24072	0.24611	0.26408	0.28458	0.30843
38	0.20300	0.23382	0.24956	0.25493	0.27301	0.29354	0.31741
39	0.21131	0.24240	0.25818	0.26365	0.28171	0.30230	0.32616
40	0.21934	0.25090	0.26670	0.27213	0.29029	0.31082	0.33465
41	0.22725	0.25915	0.27494	0.28040	0.29863	0.31915	0.34292
42	0.23521	0.26718	0.28308	0.28851	0.30681	0.32732	0.35101
43	0.24307	0.27510	0.29110	0.29650	0.31471	0.33525	0.35885
44	0.25075	0.28296	0.29883	0.30434	0.32250	0.34296	0.36649
45	0.25830	0.29048	0.30647	0.31191	0.33010	0.35052	0.37395
46	0.26550	0.29794	0.31388	0.31934	0.33752	0.35785	0.38119
47	0.27300	0.30537	0.32122	0.32662	0.34476	0.36506	0.38827
48	0.27991	0.31254	0.32835	0.33379	0.35183	0.37206	0.39519
49	0.28709	0.31951	0.33535	0.34080	0.35875	0.37894	0.40193
50	0.29419	0.32638	0.34222	0.34760	0.36550	0.38558	0.40854
51	0.30094	0.33315	0.34890	0.35429	0.37215	0.39210	0.41494
52	0.30756	0.33972	0.35546	0.36082	0.37864	0.39850	0.42119
53	0.31407	0.34612	0.36185	0.36722	0.38495	0.40473	0.42732
54	0.32045	0.35242	0.36815	0.37347	0.39117	0.41084	0.43330
55	0.32660	0.35867	0.37427	0.37965	0.39726	0.41685	0.43914
56	0.33261	0.36479	0.38038	0.38570	0.40315	0.42269	0.44485
57	0.33850	0.37067	0.38629	0.39155	0.40903	0.42840	0.45045
58	0.34461	0.37653	0.39202	0.39734	0.41472	0.43401	0.45591
59	0.35049	0.38228	0.39777	0.40299	0.42027	0.43949	0.46124
60	0.35606	0.38782	0.40327	0.40851	0.42572	0.44486	0.46647
61	0.36155	0.39335	0.40875	0.41395	0.43111	0.45007	0.47158
62	0.36713	0.39885	0.41406	0.41923	0.43637	0.45524	0.47660
63	0.37250	0.40411	0.41934	0.42445	0.44152	0.46026	0.48149
64	0.37792	0.40935	0.42443	0.42953	0.44651	0.46520	0.48627
65	0.38308	0.41442	0.42944	0.43456	0.45145	0.47001	0.49095
66	0.38812	0.41944	0.43442	0.43950	0.45630	0.47476	0.49554
67	0.39321	0.42434	0.43928	0.44436	0.46105	0.47937	0.50006
68	0.39809	0.42923	0.44407	0.44910	0.46570	0.48391	0.50447
69	0.40301	0.43403	0.44875	0.45376	0.47023	0.48840	0.50881
70	0.40779	0.43869	0.45339	0.45838	0.47474	0.49274	0.51308
71	0.41260	0.44332	0.45795	0.46290	0.47917	0.49703	0.51726
72	0.41723	0.44783	0.46236	0.46727	0.48347	0.50124	0.52135
73	0.42183	0.45223	0.46674	0.47166	0.48771	0.50539	0.52536
74	0.42628	0.45658	0.47099	0.47586	0.49185	0.50947	0.52930
75	0.43070	0.46083	0.47520	0.48008	0.49597	0.51348	0.53314
76	0.43501	0.46508	0.47934	0.48420	0.49998	0.51738	0.53693
77	0.43934	0.46911	0.48338	0.48818	0.50395	0.52120	0.54068
78	0.44367	0.47319	0.48735	0.49218	0.50782	0.52498	0.54431
79	0.44774	0.47730	0.49133	0.49610	0.51167	0.52870	0.54792
80	0.45165	0.48125	0.49519	0.49992	0.51542	0.53237	0.55144
81	0.45571	0.48507	0.49898	0.50366	0.51906	0.53593	0.55491
82	0.45970	0.48883	0.50272	0.50736	0.52268	0.53949	0.55834
83	0.46371	0.49255	0.50636	0.51095	0.52624	0.54295	0.56168
84	0.46738	0.49623	0.50997	0.51456	0.52973	0.54637	0.56498
85	0.47119	0.49982	0.51355	0.51814	0.53323	0.54974	0.56823
86	0.47485	0.50347	0.51707	0.52163	0.53662	0.55302	0.57143
87	0.47841	0.50698	0.52053	0.52508	0.53995	0.55626	0.57454
88	0.48190	0.51048	0.52392	0.52846	0.54326	0.55946	0.57761
89	0.48552	0.51392	0.52724	0.53181	0.54649	0.56259	0.58064
90	0.48893	0.51727	0.53057	0.53504	0.54970	0.56569	0.58363
91	0.49240	0.52061	0.53385	0.53830	0.55283	0.56875	0.58656
92	0.49596	0.52386	0.53698	0.54145	0.55593	0.57174	0.58944
93	0.49921	0.52703	0.54010	0.54454	0.55895	0.57468	0.59228
94	0.50262	0.53015	0.54324	0.54765	0.56193	0.57757	0.59510
95	0.50585	0.53325	0.54628	0.55062	0.56488	0.58044	0.59785
96	0.50902	0.53635	0.54932	0.55366	0.56777	0.58328	0.60058
97	0.51225	0.53940	0.55230	0.55663	0.57062	0.58605	0.60325
98	0.51530	0.54242	0.55519	0.55946	0.57347	0.58879	0.60588
99	0.51837	0.54540	0.55803	0.56231	0.57627	0.59148	0.60847
100	0.52149	0.54828	0.56087	0.56512	0.57900	0.59416	0.61103
101	0.52444	0.55113	0.56369	0.56796	0.58169	0.59679	0.61356
102	0.52731	0.55391	0.56644	0.57064	0.58436	0.59938	0.61603
103	0.53015	0.55675	0.56919	0.57333	0.58697	0.60191	0.61849
104	0.53310	0.55949	0.57187	0.57600	0.58957	0.60440	0.62090
105	0.53591	0.56216	0.57451	0.57864	0.59215	0.60687	0.62327

N	0.1%	0.5%	1%	1.25%	2.5%	5%	10%
106	0.53874	0.56484	0.57712	0.58125	0.59467	0.60931	0.62562
107	0.54149	0.56750	0.57969	0.58381	0.59718	0.61170	0.62794
108	0.54422	0.57009	0.58225	0.58637	0.59966	0.61411	0.63022
109	0.54687	0.57266	0.58477	0.58885	0.60207	0.61645	0.63247
110	0.54955	0.57526	0.58722	0.59129	0.60441	0.61875	0.63469
111	0.55228	0.57774	0.58967	0.59371	0.60678	0.62103	0.63688
112	0.55481	0.58026	0.59209	0.59611	0.60910	0.62328	0.63904
113	0.55738	0.58272	0.59447	0.59846	0.61141	0.62550	0.64117
114	0.55992	0.58509	0.59682	0.60079	0.61366	0.62770	0.64327
115	0.56235	0.58743	0.59915	0.60310	0.61590	0.62984	0.64535
116	0.56478	0.58979	0.60146	0.60534	0.61810	0.63198	0.64739
117	0.56739	0.59209	0.60372	0.60759	0.62029	0.63408	0.64941
118	0.56982	0.59438	0.60593	0.60984	0.62244	0.63615	0.65139
119	0.57221	0.59664	0.60815	0.61201	0.62456	0.63821	0.65336
120	0.57449	0.59889	0.61035	0.61421	0.62665	0.64023	0.65532
121	0.57683	0.60112	0.61248	0.61635	0.62873	0.64223	0.65725
122	0.57909	0.60334	0.61462	0.61843	0.63079	0.64420	0.65914
123	0.58149	0.60547	0.61672	0.62051	0.63281	0.64614	0.66102
124	0.58371	0.60761	0.61882	0.62258	0.63482	0.64808	0.66287
125	0.58593	0.60969	0.62087	0.62462	0.63680	0.64999	0.66471
126	0.58813	0.61172	0.62287	0.62661	0.63874	0.65187	0.66652
127	0.59030	0.61377	0.62486	0.62860	0.64066	0.65374	0.66831
128	0.59246	0.61582	0.62686	0.63057	0.64257	0.65556	0.67006
129	0.59450	0.61786	0.62884	0.63249	0.64445	0.65738	0.67181
130	0.59662	0.61984	0.63076	0.63442	0.64631	0.65917	0.67352
131	0.59860	0.62181	0.63263	0.63631	0.64814	0.66094	0.67522
132	0.60073	0.62374	0.63456	0.63818	0.64995	0.66271	0.67691
133	0.60267	0.62562	0.63643	0.64006	0.65172	0.66446	0.67856
134	0.60478	0.62755	0.63829	0.64188	0.65350	0.66615	0.68021
135	0.60677	0.62942	0.64012	0.64369	0.65526	0.66785	0.68183
136	0.60872	0.63128	0.64191	0.64549	0.65699	0.66953	0.68344
137	0.61067	0.63307	0.64372	0.64727	0.65870	0.67117	0.68503
138	0.61255	0.63488	0.64549	0.64904	0.66039	0.67282	0.68661
139	0.61442	0.63668	0.64723	0.65072	0.66206	0.67444	0.68815
140	0.61629	0.63848	0.64896	0.65243	0.66371	0.67604	0.68969
141	0.61813	0.64026	0.65067	0.65415	0.66537	0.67763	0.69121
142	0.61996	0.64200	0.65233	0.65581	0.66701	0.67919	0.69271
143	0.62180	0.64370	0.65401	0.65746	0.66863	0.68074	0.69420
144	0.62363	0.64543	0.65567	0.65909	0.67021	0.68228	0.69568
145	0.62536	0.64708	0.65731	0.66072	0.67180	0.68380	0.69712
146	0.62720	0.64879	0.65890	0.66235	0.67334	0.68530	0.69856
147	0.62891	0.65040	0.66052	0.66393	0.67490	0.68678	0.69999
148	0.63067	0.65205	0.66210	0.66548	0.67642	0.68824	0.70139
149	0.63231	0.65364	0.66369	0.66705	0.67792	0.68970	0.70278
150	0.63405	0.65525	0.66527	0.66862	0.67940	0.69115	0.70415
151	0.63574	0.65683	0.66678	0.67012	0.68089	0.69258	0.70551
152	0.63745	0.65839	0.66830	0.67162	0.68235	0.69398	0.70687
153	0.63913	0.65998	0.66984	0.67310	0.68380	0.69538	0.70821
154	0.64075	0.66155	0.67130	0.67459	0.68524	0.69676	0.70953
155	0.64235	0.66307	0.67280	0.67608	0.68665	0.69813	0.71084
156	0.64393	0.66455	0.67427	0.67753	0.68806	0.69948	0.71213
157	0.64543	0.66603	0.67571	0.67894	0.68946	0.70083	0.71342
158	0.64701	0.66754	0.67715	0.68037	0.69082	0.70216	0.71468
159	0.64850	0.66898	0.67857	0.68177	0.69219	0.70347	0.71594
160	0.65004	0.67044	0.68000	0.68316	0.69354	0.70478	0.71719
161	0.65159	0.67187	0.68138	0.68453	0.69489	0.70606	0.71842
162	0.65297	0.67327	0.68275	0.68588	0.69620	0.70733	0.71964
163	0.65458	0.67468	0.68412	0.68725	0.69751	0.70859	0.72084
164	0.65610	0.67610	0.68547	0.68860	0.69881	0.70984	0.72204
165	0.65744	0.67750	0.68683	0.68992	0.70009	0.71109	0.72323
166	0.65884	0.67887	0.68814	0.69123	0.70136	0.71232	0.72441
167	0.66029	0.68020	0.68945	0.69254	0.70263	0.71354	0.72558
168	0.66169	0.68156	0.69076	0.69385	0.70387	0.71474	0.72673
169	0.66312	0.68289	0.69206	0.69512	0.70510	0.71594	0.72787
170	0.66459	0.68422	0.69333	0.69639	0.70634	0.71711	0.72899
171	0.66600	0.68554	0.69460	0.69766	0.70755	0.71828	0.73012
172	0.66734	0.68685	0.69586	0.69891	0.70877	0.71945	0.73123
173	0.66865	0.68814	0.69713	0.70014	0.70997	0.72061	0.73232
174	0.67001	0.68942	0.69834	0.70137	0.71114	0.72175	0.73342
175	0.67136	0.69069	0.69959	0.70259	0.71231	0.72287	0.73450
176	0.67268	0.69194	0.70079	0.70377	0.71347	0.72397	0.73557
177	0.67401	0.69319	0.70201	0.70498	0.71463	0.72510	0.73664
178	0.67524	0.69440	0.70322	0.70615	0.71578	0.72620	0.73769
179	0.67649	0.69559	0.70437	0.70733	0.71691	0.72730	0.73874

N	0.1%	0.5%	1%	1.25%	2.5%	5%	10%
180	0.67778	0.69683	0.70556	0.70848	0.71805	0.72839	0.73977
181	0.67905	0.69800	0.70671	0.70963	0.71915	0.72944	0.74079
182	0.68031	0.69917	0.70784	0.71076	0.72026	0.73050	0.74181
183	0.68158	0.70036	0.70900	0.71189	0.72135	0.73155	0.74281
184	0.68282	0.70151	0.71014	0.71302	0.72243	0.73259	0.74381
185	0.68406	0.70264	0.71125	0.71415	0.72350	0.73362	0.74480
186	0.68528	0.70379	0.71239	0.71523	0.72457	0.73464	0.74578
187	0.68645	0.70492	0.71348	0.71635	0.72562	0.73566	0.74675
188	0.68765	0.70607	0.71457	0.71742	0.72666	0.73667	0.74772
189	0.68888	0.70718	0.71566	0.71849	0.72770	0.73768	0.74867
190	0.69004	0.70829	0.71673	0.71956	0.72872	0.73867	0.74962
191	0.69124	0.70937	0.71781	0.72061	0.72976	0.73966	0.75056
192	0.69234	0.71044	0.71886	0.72166	0.73077	0.74063	0.75149
193	0.69353	0.71156	0.71990	0.72270	0.73178	0.74160	0.75242
194	0.69472	0.71259	0.72092	0.72372	0.73278	0.74255	0.75334
195	0.69581	0.71366	0.72196	0.72475	0.73376	0.74350	0.75424
196	0.69696	0.71475	0.72298	0.72575	0.73475	0.74444	0.75515
197	0.69808	0.71578	0.72398	0.72674	0.73573	0.74537	0.75604
198	0.69911	0.71682	0.72498	0.72774	0.73669	0.74630	0.75692
199	0.70020	0.71782	0.72599	0.72872	0.73764	0.74722	0.75781
200	0.70128	0.71886	0.72698	0.72968	0.73859	0.74813	0.75868
201	0.70231	0.71988	0.72796	0.73067	0.73953	0.74903	0.75954
202	0.70340	0.72089	0.72894	0.73164	0.74047	0.74994	0.76040
203	0.70447	0.72187	0.72991	0.73261	0.74140	0.75082	0.76125
204	0.70551	0.72287	0.73086	0.73356	0.74232	0.75171	0.76210
205	0.70657	0.72384	0.73181	0.73451	0.74323	0.75259	0.76294
206	0.70761	0.72483	0.73276	0.73545	0.74413	0.75346	0.76377
207	0.70864	0.72579	0.73371	0.73637	0.74503	0.75433	0.76459
208	0.70965	0.72677	0.73465	0.73730	0.74591	0.75518	0.76542
209	0.71064	0.72772	0.73559	0.73822	0.74679	0.75602	0.76624
210	0.71164	0.72865	0.73649	0.73912	0.74767	0.75686	0.76704
211	0.71258	0.72958	0.73741	0.74004	0.74853	0.75770	0.76784
212	0.71362	0.73053	0.73833	0.74094	0.74939	0.75853	0.76864
213	0.71462	0.73144	0.73924	0.74183	0.75025	0.75936	0.76942
214	0.71564	0.73234	0.74012	0.74272	0.75110	0.76017	0.77021
215	0.71661	0.73324	0.74100	0.74358	0.75194	0.76099	0.77098
216	0.71756	0.73419	0.74187	0.74445	0.75279	0.76179	0.77175
217	0.71852	0.73504	0.74274	0.74531	0.75362	0.76259	0.77251
218	0.71951	0.73593	0.74361	0.74618	0.75445	0.76339	0.77328
219	0.72046	0.73681	0.74446	0.74702	0.75527	0.76418	0.77403
220	0.72137	0.73768	0.74529	0.74787	0.75609	0.76497	0.77478
221	0.72229	0.73854	0.74614	0.74870	0.75690	0.76574	0.77552
222	0.72326	0.73941	0.74698	0.74953	0.75770	0.76651	0.77626
223	0.72414	0.74024	0.74784	0.75034	0.75850	0.76727	0.77698
224	0.72505	0.74110	0.74864	0.75115	0.75928	0.76803	0.77771
225	0.72593	0.74198	0.74948	0.75196	0.76007	0.76879	0.77843
226	0.72683	0.74280	0.75031	0.75278	0.76084	0.76954	0.77915
227	0.72768	0.74364	0.75110	0.75358	0.76161	0.77029	0.77986
228	0.72857	0.74445	0.75190	0.75436	0.76238	0.77102	0.78056
229	0.72942	0.74526	0.75269	0.75514	0.76314	0.77175	0.78126
230	0.73027	0.74608	0.75348	0.75593	0.76390	0.77248	0.78196
231	0.73112	0.74689	0.75428	0.75670	0.76464	0.77320	0.78265
232	0.73194	0.74767	0.75503	0.75747	0.76538	0.77392	0.78334
233	0.73275	0.74848	0.75582	0.75825	0.76612	0.77462	0.78402
234	0.73361	0.74929	0.75658	0.75901	0.76686	0.77533	0.78470
235	0.73445	0.75008	0.75734	0.75976	0.76760	0.77603	0.78537
236	0.73528	0.75086	0.75810	0.76051	0.76832	0.77673	0.78603
237	0.73610	0.75162	0.75884	0.76126	0.76904	0.77741	0.78669
238	0.73692	0.75239	0.75960	0.76200	0.76976	0.77810	0.78735
239	0.73776	0.75319	0.76035	0.76274	0.77047	0.77879	0.78800
240	0.73853	0.75394	0.76109	0.76346	0.77117	0.77946	0.78865
241	0.73933	0.75470	0.76183	0.76420	0.77186	0.78014	0.78929
242	0.74015	0.75547	0.76256	0.76492	0.77256	0.78081	0.78993
243	0.74091	0.75623	0.76327	0.76564	0.77325	0.78148	0.79057
244	0.74172	0.75698	0.76401	0.76634	0.77394	0.78214	0.79120
245	0.74249	0.75770	0.76473	0.76704	0.77462	0.78280	0.79182
246	0.74331	0.75843	0.76544	0.76775	0.77529	0.78345	0.79244
247	0.74410	0.75918	0.76613	0.76844	0.77597	0.78410	0.79307
248	0.74491	0.75990	0.76681	0.76913	0.77664	0.78474	0.79369
249	0.74564	0.76061	0.76749	0.76981	0.77731	0.78537	0.79430
250	0.74637	0.76133	0.76818	0.77049	0.77797	0.78601	0.79490
251	0.74714	0.76202	0.76886	0.77116	0.77863	0.78664	0.79550
252	0.74788	0.76272	0.76954	0.77183	0.77927	0.78728	0.79611
253	0.74861	0.76340	0.77023	0.77250	0.77993	0.78790	0.79670

N	0.1%	0.5%	1%	1.25%	2.5%	5%	10%
254	0.74939	0.76409	0.77089	0.77317	0.78057	0.78851	0.79729
255	0.75014	0.76477	0.77156	0.77383	0.78121	0.78912	0.79787
256	0.75090	0.76546	0.77223	0.77449	0.78184	0.78973	0.79846
257	0.75160	0.76612	0.77289	0.77514	0.78246	0.79034	0.79904
258	0.75228	0.76681	0.77354	0.77580	0.78310	0.79094	0.79961
259	0.75300	0.76748	0.77420	0.77645	0.78373	0.79154	0.80019
260	0.75377	0.76816	0.77484	0.77708	0.78435	0.79214	0.80075
261	0.75446	0.76880	0.77549	0.77771	0.78496	0.79272	0.80131
262	0.75513	0.76946	0.77613	0.77834	0.78557	0.79331	0.80188
263	0.75586	0.77011	0.77676	0.77896	0.78617	0.79389	0.80243
264	0.75655	0.77079	0.77740	0.77959	0.78678	0.79447	0.80299
265	0.75726	0.77142	0.77803	0.78020	0.78738	0.79505	0.80354
266	0.75797	0.77205	0.77865	0.78083	0.78797	0.79562	0.80409
267	0.75862	0.77269	0.77928	0.78146	0.78856	0.79620	0.80464
268	0.75929	0.77334	0.77989	0.78206	0.78915	0.79676	0.80518
269	0.75993	0.77396	0.78051	0.78267	0.78974	0.79732	0.80572
270	0.76059	0.77459	0.78113	0.78327	0.79031	0.79789	0.80626
271	0.76125	0.77521	0.78173	0.78388	0.79088	0.79844	0.80679
272	0.76189	0.77583	0.78233	0.78449	0.79147	0.79900	0.80731
273	0.76254	0.77644	0.78292	0.78507	0.79204	0.79954	0.80784
274	0.76318	0.77705	0.78351	0.78567	0.79260	0.80009	0.80836
275	0.76381	0.77769	0.78412	0.78624	0.79317	0.80063	0.80888
276	0.76449	0.77828	0.78471	0.78683	0.79372	0.80117	0.80940
277	0.76513	0.77889	0.78528	0.78739	0.79429	0.80171	0.80991
278	0.76579	0.77949	0.78586	0.78797	0.79485	0.80224	0.81042
279	0.76645	0.78007	0.78643	0.78854	0.79541	0.80278	0.81093
280	0.76704	0.78068	0.78700	0.78911	0.79595	0.80330	0.81143
281	0.76767	0.78128	0.78757	0.78968	0.79649	0.80383	0.81194
282	0.76831	0.78185	0.78815	0.79023	0.79704	0.80435	0.81243
283	0.76896	0.78243	0.78872	0.79080	0.79757	0.80487	0.81293
284	0.76955	0.78301	0.78927	0.79135	0.79811	0.80538	0.81342
285	0.77019	0.78359	0.78983	0.79190	0.79864	0.80589	0.81391
286	0.77080	0.78417	0.79038	0.79243	0.79916	0.80640	0.81440
287	0.77137	0.78475	0.79093	0.79298	0.79969	0.80690	0.81487
288	0.77196	0.78531	0.79148	0.79352	0.80021	0.80740	0.81535
289	0.77252	0.78589	0.79203	0.79407	0.80073	0.80791	0.81583
290	0.77312	0.78646	0.79256	0.79460	0.80125	0.80841	0.81630
291	0.77374	0.78699	0.79311	0.79515	0.80177	0.80891	0.81678
292	0.77429	0.78754	0.79364	0.79567	0.80228	0.80939	0.81725
293	0.77488	0.78811	0.79417	0.79620	0.80279	0.80989	0.81771
294	0.77546	0.78864	0.79469	0.79671	0.80330	0.81037	0.81817
295	0.77604	0.78918	0.79523	0.79724	0.80380	0.81086	0.81863
296	0.77660	0.78972	0.79575	0.79775	0.80430	0.81134	0.81909
297	0.77718	0.79026	0.79628	0.79828	0.80479	0.81182	0.81955
298	0.77779	0.79078	0.79679	0.79879	0.80529	0.81229	0.82000
299	0.77835	0.79132	0.79730	0.79930	0.80578	0.81276	0.82046
300	0.77893	0.79187	0.79781	0.79981	0.80627	0.81323	0.82090
301	0.77951	0.79240	0.79831	0.80030	0.80675	0.81370	0.82135
302	0.78008	0.79289	0.79882	0.80081	0.80724	0.81416	0.82180
303	0.78062	0.79341	0.79932	0.80130	0.80772	0.81462	0.82224
304	0.78115	0.79392	0.79981	0.80179	0.80819	0.81508	0.82268
305	0.78170	0.79444	0.80031	0.80228	0.80867	0.81554	0.82311
306	0.78223	0.79495	0.80081	0.80278	0.80915	0.81600	0.82355
307	0.78278	0.79545	0.80130	0.80327	0.80962	0.81645	0.82397
308	0.78328	0.79596	0.80179	0.80375	0.81009	0.81689	0.82440
309	0.78385	0.79645	0.80229	0.80424	0.81055	0.81734	0.82484
310	0.78435	0.79694	0.80277	0.80471	0.81102	0.81778	0.82526
311	0.78485	0.79744	0.80325	0.80519	0.81147	0.81822	0.82568
312	0.78536	0.79794	0.80372	0.80565	0.81193	0.81866	0.82610
313	0.78587	0.79843	0.80419	0.80613	0.81239	0.81910	0.82652
314	0.78642	0.79893	0.80467	0.80659	0.81285	0.81954	0.82694
315	0.78693	0.79942	0.80515	0.80706	0.81329	0.81998	0.82735
316	0.78742	0.79989	0.80562	0.80752	0.81374	0.82041	0.82776
317	0.78792	0.80039	0.80609	0.80799	0.81419	0.82084	0.82817
318	0.78847	0.80085	0.80655	0.80845	0.81463	0.82126	0.82858
319	0.78896	0.80132	0.80702	0.80891	0.81508	0.82169	0.82898
320	0.78949	0.80179	0.80749	0.80936	0.81552	0.82211	0.82938
321	0.79000	0.80227	0.80795	0.80982	0.81595	0.82253	0.82979
322	0.79047	0.80272	0.80839	0.81026	0.81639	0.82295	0.83019
323	0.79096	0.80321	0.80885	0.81072	0.81681	0.82336	0.83058
324	0.79146	0.80368	0.80930	0.81117	0.81723	0.82377	0.83097
325	0.79195	0.80414	0.80974	0.81161	0.81766	0.82418	0.83137
326	0.79242	0.80460	0.81018	0.81205	0.81808	0.82459	0.83176
327	0.79296	0.80506	0.81063	0.81249	0.81851	0.82499	0.83215

N	0.1%	0.5%	1%	1.25%	2.5%	5%	10%
328	0.79345	0.80551	0.81108	0.81293	0.81893	0.82540	0.83253
329	0.79394	0.80597	0.81151	0.81337	0.81935	0.82580	0.83292
330	0.79441	0.80641	0.81195	0.81379	0.81977	0.82620	0.83330
331	0.79488	0.80685	0.81238	0.81422	0.82019	0.82660	0.83368
332	0.79539	0.80729	0.81281	0.81465	0.82059	0.82699	0.83406
333	0.79585	0.80773	0.81324	0.81508	0.82101	0.82738	0.83444
334	0.79631	0.80817	0.81368	0.81551	0.82142	0.82778	0.83481
335	0.79677	0.80861	0.81409	0.81593	0.82183	0.82817	0.83519
336	0.79724	0.80904	0.81452	0.81636	0.82223	0.82856	0.83555
337	0.79770	0.80948	0.81495	0.81677	0.82262	0.82894	0.83593
338	0.79816	0.80992	0.81537	0.81718	0.82303	0.82933	0.83629
339	0.79862	0.81035	0.81579	0.81759	0.82342	0.82971	0.83666
340	0.79907	0.81077	0.81620	0.81800	0.82382	0.83009	0.83703
341	0.79953	0.81120	0.81662	0.81842	0.82421	0.83047	0.83739
342	0.79998	0.81163	0.81703	0.81882	0.82460	0.83085	0.83775
343	0.80043	0.81203	0.81744	0.81923	0.82500	0.83123	0.83811
344	0.80084	0.81244	0.81784	0.81963	0.82539	0.83160	0.83846
345	0.80126	0.81285	0.81826	0.82004	0.82578	0.83197	0.83882
346	0.80170	0.81327	0.81867	0.82043	0.82616	0.83234	0.83917
347	0.80215	0.81369	0.81906	0.82082	0.82655	0.83271	0.83953
348	0.80262	0.81410	0.81946	0.82121	0.82693	0.83308	0.83988
349	0.80306	0.81451	0.81985	0.82161	0.82731	0.83344	0.84023
350	0.80348	0.81491	0.82024	0.82200	0.82769	0.83381	0.84057
351	0.80392	0.81533	0.82064	0.82239	0.82807	0.83417	0.84092
352	0.80435	0.81573	0.82104	0.82278	0.82844	0.83453	0.84126
353	0.80473	0.81613	0.82143	0.82317	0.82882	0.83489	0.84160
354	0.80514	0.81653	0.82181	0.82355	0.82919	0.83525	0.84194
355	0.80558	0.81695	0.82220	0.82393	0.82955	0.83560	0.84228
356	0.80601	0.81733	0.82258	0.82431	0.82992	0.83595	0.84262
357	0.80642	0.81772	0.82297	0.82468	0.83029	0.83630	0.84295
358	0.80684	0.81812	0.82335	0.82507	0.83066	0.83665	0.84329
359	0.80726	0.81851	0.82373	0.82545	0.83101	0.83700	0.84362
360	0.80765	0.81891	0.82411	0.82582	0.83137	0.83735	0.84395
361	0.80807	0.81927	0.82449	0.82619	0.83173	0.83769	0.84428
362	0.80849	0.81965	0.82485	0.82656	0.83209	0.83803	0.84461
363	0.80887	0.82003	0.82522	0.82693	0.83245	0.83838	0.84493
364	0.80932	0.82042	0.82559	0.82730	0.83280	0.83872	0.84526
365	0.80972	0.82081	0.82596	0.82766	0.83315	0.83906	0.84558
366	0.81012	0.82119	0.82633	0.82803	0.83349	0.83939	0.84590
367	0.81051	0.82158	0.82669	0.82839	0.83384	0.83973	0.84622
368	0.81093	0.82195	0.82705	0.82874	0.83419	0.84006	0.84654
369	0.81129	0.82233	0.82742	0.82910	0.83453	0.84039	0.84686
370	0.81169	0.82269	0.82778	0.82946	0.83487	0.84072	0.84717
371	0.81209	0.82306	0.82814	0.82981	0.83522	0.84105	0.84748
372	0.81247	0.82343	0.82850	0.83017	0.83555	0.84138	0.84780
373	0.81285	0.82380	0.82885	0.83052	0.83590	0.84171	0.84811
374	0.81324	0.82417	0.82920	0.83087	0.83624	0.84203	0.84842
375	0.81364	0.82453	0.82956	0.83121	0.83657	0.84236	0.84873
376	0.81403	0.82489	0.82991	0.83157	0.83691	0.84268	0.84904
377	0.81441	0.82524	0.83026	0.83191	0.83724	0.84300	0.84934
378	0.81481	0.82560	0.83061	0.83225	0.83757	0.84331	0.84965
379	0.81520	0.82596	0.83095	0.83260	0.83790	0.84363	0.84995
380	0.81555	0.82631	0.83130	0.83294	0.83823	0.84395	0.85025
381	0.81595	0.82666	0.83164	0.83328	0.83855	0.84427	0.85056
382	0.81633	0.82701	0.83198	0.83362	0.83888	0.84458	0.85085
383	0.81672	0.82736	0.83231	0.83395	0.83920	0.84488	0.85115
384	0.81706	0.82770	0.83265	0.83428	0.83952	0.84520	0.85145
385	0.81745	0.82805	0.83298	0.83461	0.83985	0.84551	0.85174
386	0.81785	0.82841	0.83331	0.83494	0.84017	0.84581	0.85203
387	0.81822	0.82876	0.83366	0.83527	0.84048	0.84612	0.85233
388	0.81856	0.82909	0.83399	0.83560	0.84080	0.84643	0.85262
389	0.81892	0.82944	0.83432	0.83592	0.84112	0.84673	0.85291
390	0.81927	0.82978	0.83464	0.83625	0.84143	0.84703	0.85320
391	0.81963	0.83011	0.83498	0.83657	0.84174	0.84734	0.85349
392	0.82003	0.83044	0.83529	0.83690	0.84206	0.84763	0.85377
393	0.82038	0.83078	0.83561	0.83722	0.84236	0.84793	0.85406
394	0.82074	0.83111	0.83594	0.83754	0.84267	0.84822	0.85435
395	0.82110	0.83145	0.83627	0.83786	0.84298	0.84852	0.85463
396	0.82144	0.83178	0.83658	0.83818	0.84329	0.84882	0.85491
397	0.82179	0.83211	0.83690	0.83849	0.84359	0.84911	0.85519
398	0.82214	0.83244	0.83722	0.83881	0.84390	0.84940	0.85547
399	0.82250	0.83277	0.83753	0.83912	0.84421	0.84969	0.85575
400	0.82283	0.83309	0.83784	0.83943	0.84450	0.84998	0.85602
401	0.82318	0.83341	0.83816	0.83975	0.84481	0.85027	0.85630

N	0.1%	0.5%	1%	1.25%	2.5%	5%	10%
402	0.82353	0.83373	0.83848	0.84005	0.84511	0.85056	0.85657
403	0.82387	0.83404	0.83879	0.84036	0.84541	0.85085	0.85684
404	0.82421	0.83437	0.83911	0.84067	0.84571	0.85114	0.85712
405	0.82455	0.83469	0.83942	0.84098	0.84600	0.85142	0.85739
406	0.82488	0.83501	0.83972	0.84129	0.84630	0.85170	0.85766
407	0.82524	0.83533	0.84003	0.84159	0.84660	0.85198	0.85793
408	0.82559	0.83565	0.84034	0.84190	0.84688	0.85226	0.85819
409	0.82591	0.83596	0.84064	0.84219	0.84717	0.85254	0.85846
410	0.82622	0.83628	0.84094	0.84249	0.84746	0.85281	0.85872
411	0.82655	0.83659	0.84123	0.84278	0.84774	0.85309	0.85899
412	0.82687	0.83690	0.84154	0.84308	0.84803	0.85337	0.85925
413	0.82719	0.83721	0.84183	0.84337	0.84831	0.85365	0.85952
414	0.82755	0.83751	0.84212	0.84366	0.84860	0.85392	0.85978
415	0.82788	0.83781	0.84242	0.84396	0.84888	0.85419	0.86004
416	0.82820	0.83812	0.84271	0.84424	0.84917	0.85446	0.86029
417	0.82853	0.83844	0.84301	0.84454	0.84945	0.85473	0.86055
418	0.82887	0.83873	0.84330	0.84483	0.84972	0.85500	0.86081
419	0.82919	0.83903	0.84359	0.84512	0.85000	0.85526	0.86107
420	0.82954	0.83934	0.84389	0.84540	0.85028	0.85553	0.86132
421	0.82984	0.83963	0.84418	0.84569	0.85055	0.85580	0.86157
422	0.83015	0.83994	0.84446	0.84598	0.85083	0.85606	0.86183
423	0.83045	0.84023	0.84475	0.84626	0.85110	0.85632	0.86208
424	0.83078	0.84052	0.84503	0.84654	0.85138	0.85658	0.86233
425	0.83110	0.84082	0.84531	0.84681	0.85165	0.85685	0.86258
426	0.83142	0.84111	0.84560	0.84710	0.85192	0.85711	0.86283
427	0.83173	0.84138	0.84588	0.84738	0.85219	0.85737	0.86308
428	0.83202	0.84167	0.84615	0.84766	0.85246	0.85762	0.86332
429	0.83231	0.84196	0.84644	0.84793	0.85272	0.85788	0.86357
430	0.83265	0.84226	0.84672	0.84821	0.85299	0.85814	0.86381
431	0.83295	0.84254	0.84700	0.84849	0.85325	0.85839	0.86405
432	0.83325	0.84283	0.84727	0.84876	0.85352	0.85864	0.86430
433	0.83355	0.84311	0.84755	0.84902	0.85379	0.85890	0.86454
434	0.83386	0.84339	0.84783	0.84930	0.85404	0.85915	0.86478
435	0.83418	0.84367	0.84810	0.84957	0.85431	0.85940	0.86502
436	0.83448	0.84397	0.84837	0.84983	0.85457	0.85965	0.86526
437	0.83477	0.84425	0.84864	0.85010	0.85483	0.85990	0.86550
438	0.83506	0.84452	0.84891	0.85037	0.85509	0.86015	0.86573
439	0.83535	0.84479	0.84917	0.85064	0.85534	0.86039	0.86597
440	0.83564	0.84507	0.84944	0.85090	0.85560	0.86064	0.86621
441	0.83594	0.84534	0.84971	0.85116	0.85586	0.86088	0.86644
442	0.83624	0.84562	0.84998	0.85142	0.85611	0.86113	0.86668
443	0.83651	0.84589	0.85024	0.85169	0.85636	0.86138	0.86691
444	0.83679	0.84617	0.85051	0.85195	0.85662	0.86162	0.86714
445	0.83708	0.84644	0.85077	0.85221	0.85687	0.86186	0.86737
446	0.83736	0.84670	0.85104	0.85248	0.85712	0.86210	0.86760
447	0.83768	0.84697	0.85130	0.85274	0.85737	0.86234	0.86783
448	0.83795	0.84724	0.85156	0.85299	0.85762	0.86258	0.86806
449	0.83825	0.84750	0.85182	0.85325	0.85787	0.86282	0.86829
450	0.83855	0.84778	0.85208	0.85350	0.85811	0.86305	0.86851
451	0.83881	0.84805	0.85234	0.85376	0.85836	0.86329	0.86874
452	0.83909	0.84831	0.85259	0.85401	0.85860	0.86352	0.86896
453	0.83937	0.84857	0.85284	0.85426	0.85885	0.86376	0.86919
454	0.83965	0.84883	0.85309	0.85451	0.85909	0.86399	0.86941
455	0.83992	0.84910	0.85334	0.85476	0.85933	0.86422	0.86963
456	0.84020	0.84935	0.85359	0.85501	0.85957	0.86445	0.86985
457	0.84049	0.84963	0.85383	0.85526	0.85981	0.86468	0.87007
458	0.84076	0.84989	0.85410	0.85550	0.86005	0.86491	0.87029
459	0.84103	0.85015	0.85434	0.85576	0.86029	0.86514	0.87050
460	0.84133	0.85041	0.85459	0.85600	0.86053	0.86536	0.87072
461	0.84162	0.85066	0.85485	0.85624	0.86076	0.86559	0.87094
462	0.84186	0.85091	0.85509	0.85649	0.86100	0.86582	0.87116
463	0.84214	0.85116	0.85534	0.85673	0.86124	0.86605	0.87137
464	0.84243	0.85141	0.85559	0.85697	0.86147	0.86627	0.87159
465	0.84269	0.85166	0.85584	0.85722	0.86170	0.86649	0.87180
466	0.84294	0.85191	0.85608	0.85746	0.86193	0.86672	0.87202
467	0.84320	0.85216	0.85633	0.85770	0.86216	0.86694	0.87223
468	0.84346	0.85240	0.85656	0.85793	0.86238	0.86716	0.87244
469	0.84371	0.85265	0.85680	0.85817	0.86261	0.86739	0.87265
470	0.84396	0.85289	0.85704	0.85840	0.86284	0.86761	0.87286
471	0.84420	0.85314	0.85727	0.85864	0.86307	0.86783	0.87307
472	0.84448	0.85339	0.85751	0.85887	0.86329	0.86804	0.87328
473	0.84477	0.85363	0.85775	0.85911	0.86352	0.86826	0.87349
474	0.84502	0.85388	0.85799	0.85934	0.86375	0.86848	0.87369
475	0.84527	0.85412	0.85822	0.85958	0.86397	0.86870	0.87390

N	0.1%	0.5%	1%	1.25%	2.5%	5%	10%
476	0.84553	0.85436	0.85846	0.85981	0.86420	0.86891	0.87411
477	0.84578	0.85461	0.85869	0.86004	0.86442	0.86913	0.87431
478	0.84605	0.85484	0.85892	0.86027	0.86465	0.86934	0.87451
479	0.84631	0.85508	0.85916	0.86049	0.86487	0.86956	0.87471
480	0.84656	0.85532	0.85939	0.86072	0.86509	0.86977	0.87492
481	0.84681	0.85556	0.85961	0.86095	0.86531	0.86998	0.87512
482	0.84708	0.85580	0.85984	0.86118	0.86553	0.87019	0.87532
483	0.84733	0.85604	0.86007	0.86140	0.86575	0.87040	0.87552
484	0.84757	0.85627	0.86029	0.86163	0.86596	0.87062	0.87572
485	0.84784	0.85651	0.86052	0.86185	0.86618	0.87082	0.87592
486	0.84810	0.85674	0.86075	0.86207	0.86640	0.87103	0.87612
487	0.84833	0.85697	0.86097	0.86229	0.86661	0.87124	0.87632
488	0.84857	0.85719	0.86119	0.86251	0.86683	0.87144	0.87651
489	0.84883	0.85743	0.86141	0.86274	0.86703	0.87165	0.87671
490	0.84908	0.85767	0.86164	0.86296	0.86725	0.87185	0.87690
491	0.84931	0.85789	0.86186	0.86319	0.86746	0.87206	0.87710
492	0.84956	0.85812	0.86208	0.86340	0.86767	0.87226	0.87730
493	0.84979	0.85834	0.86230	0.86362	0.86788	0.87246	0.87749
494	0.85003	0.85857	0.86252	0.86384	0.86809	0.87267	0.87768
495	0.85029	0.85879	0.86274	0.86405	0.86830	0.87287	0.87787
496	0.85052	0.85902	0.86296	0.86426	0.86851	0.87307	0.87807
497	0.85077	0.85924	0.86318	0.86448	0.86872	0.87327	0.87825
498	0.85100	0.85946	0.86340	0.86470	0.86892	0.87347	0.87844
499	0.85125	0.85969	0.86361	0.86490	0.86913	0.87367	0.87864
500	0.85147	0.85991	0.86383	0.86512	0.86934	0.87386	0.87883
501	0.85171	0.86014	0.86405	0.86533	0.86954	0.87406	0.87901
502	0.85194	0.86036	0.86426	0.86555	0.86974	0.87425	0.87920
503	0.85218	0.86058	0.86448	0.86576	0.86995	0.87445	0.87939
504	0.85242	0.86079	0.86469	0.86597	0.87016	0.87465	0.87957
505	0.85264	0.86101	0.86490	0.86618	0.87036	0.87484	0.87976
506	0.85288	0.86123	0.86511	0.86639	0.87056	0.87504	0.87995
507	0.85311	0.86145	0.86532	0.86660	0.87076	0.87523	0.88013
508	0.85334	0.86167	0.86554	0.86681	0.87096	0.87542	0.88032
509	0.85356	0.86188	0.86574	0.86702	0.87116	0.87561	0.88050
510	0.85379	0.86210	0.86595	0.86722	0.87136	0.87581	0.88068
511	0.85401	0.86230	0.86616	0.86743	0.87156	0.87600	0.88087
512	0.85425	0.86251	0.86636	0.86763	0.87175	0.87618	0.88105
513	0.85447	0.86273	0.86657	0.86784	0.87195	0.87638	0.88123
514	0.85470	0.86296	0.86678	0.86804	0.87214	0.87656	0.88141
515	0.85493	0.86317	0.86698	0.86824	0.87233	0.87675	0.88159
516	0.85514	0.86338	0.86719	0.86845	0.87253	0.87694	0.88177
517	0.85536	0.86359	0.86738	0.86865	0.87272	0.87713	0.88194
518	0.85557	0.86380	0.86759	0.86885	0.87292	0.87731	0.88212
519	0.85579	0.86400	0.86780	0.86906	0.87311	0.87750	0.88230
520	0.85603	0.86420	0.86800	0.86925	0.87330	0.87768	0.88248
521	0.85624	0.86441	0.86821	0.86945	0.87350	0.87786	0.88266
522	0.85648	0.86462	0.86841	0.86965	0.87369	0.87805	0.88283
523	0.85671	0.86483	0.86861	0.86985	0.87388	0.87823	0.88301
524	0.85691	0.86503	0.86880	0.87004	0.87407	0.87841	0.88318
525	0.85713	0.86523	0.86900	0.87024	0.87426	0.87860	0.88335
526	0.85734	0.86543	0.86921	0.87043	0.87445	0.87878	0.88353
527	0.85754	0.86564	0.86940	0.87063	0.87464	0.87896	0.88370
528	0.85777	0.86585	0.86960	0.87082	0.87483	0.87914	0.88387
529	0.85797	0.86605	0.86979	0.87102	0.87501	0.87932	0.88405
530	0.85818	0.86625	0.86999	0.87121	0.87519	0.87950	0.88422
531	0.85838	0.86644	0.87018	0.87141	0.87538	0.87968	0.88439
532	0.85860	0.86664	0.87037	0.87159	0.87556	0.87985	0.88456
533	0.85883	0.86684	0.87056	0.87178	0.87575	0.88003	0.88473
534	0.85904	0.86704	0.87076	0.87197	0.87594	0.88021	0.88489
535	0.85925	0.86725	0.87095	0.87217	0.87612	0.88038	0.88506
536	0.85946	0.86744	0.87114	0.87236	0.87630	0.88056	0.88523
537	0.85967	0.86764	0.87133	0.87254	0.87648	0.88073	0.88540
538	0.85989	0.86783	0.87151	0.87272	0.87667	0.88091	0.88556
539	0.86009	0.86803	0.87170	0.87291	0.87684	0.88108	0.88573
540	0.86031	0.86822	0.87190	0.87310	0.87703	0.88125	0.88590
541	0.86053	0.86843	0.87208	0.87329	0.87721	0.88142	0.88606
542	0.86072	0.86861	0.87227	0.87347	0.87739	0.88160	0.88623
543	0.86092	0.86880	0.87246	0.87365	0.87756	0.88177	0.88639
544	0.86112	0.86900	0.87264	0.87384	0.87774	0.88194	0.88655
545	0.86134	0.86919	0.87283	0.87403	0.87792	0.88211	0.88672
546	0.86156	0.86939	0.87301	0.87421	0.87809	0.88228	0.88688
547	0.86176	0.86957	0.87320	0.87439	0.87827	0.88245	0.88704
548	0.86196	0.86976	0.87338	0.87457	0.87845	0.88262	0.88721
549	0.86215	0.86995	0.87356	0.87475	0.87862	0.88279	0.88737

N	0.1%	0.5%	1%	1.25%	2.5%	5%	10%
550	0.86235	0.87014	0.87374	0.87493	0.87880	0.88296	0.88753
551	0.86256	0.87033	0.87393	0.87512	0.87897	0.88313	0.88769
552	0.86274	0.87052	0.87411	0.87530	0.87915	0.88330	0.88785
553	0.86294	0.87070	0.87429	0.87548	0.87933	0.88346	0.88801
554	0.86315	0.87089	0.87447	0.87565	0.87950	0.88363	0.88817
555	0.86336	0.87107	0.87464	0.87583	0.87967	0.88379	0.88833
556	0.86358	0.87126	0.87482	0.87600	0.87984	0.88396	0.88848
557	0.86377	0.87144	0.87501	0.87618	0.88001	0.88412	0.88864
558	0.86396	0.87163	0.87519	0.87636	0.88018	0.88429	0.88880
559	0.86416	0.87181	0.87537	0.87654	0.88035	0.88445	0.88895
560	0.86435	0.87199	0.87554	0.87672	0.88052	0.88461	0.88911
561	0.86453	0.87217	0.87572	0.87689	0.88069	0.88478	0.88926
562	0.86472	0.87235	0.87589	0.87707	0.88086	0.88494	0.88942
563	0.86490	0.87253	0.87607	0.87724	0.88103	0.88510	0.88957
564	0.86512	0.87272	0.87624	0.87742	0.88119	0.88526	0.88973
565	0.86529	0.87290	0.87642	0.87759	0.88136	0.88542	0.88988
566	0.86547	0.87308	0.87660	0.87776	0.88153	0.88557	0.89003
567	0.86566	0.87326	0.87677	0.87794	0.88169	0.88573	0.89019
568	0.86587	0.87343	0.87694	0.87811	0.88186	0.88589	0.89034
569	0.86605	0.87361	0.87711	0.87828	0.88202	0.88605	0.89049
570	0.86623	0.87378	0.87729	0.87844	0.88219	0.88621	0.89064
571	0.86642	0.87396	0.87745	0.87860	0.88235	0.88636	0.89079
572	0.86661	0.87413	0.87763	0.87877	0.88251	0.88652	0.89094
573	0.86680	0.87431	0.87780	0.87894	0.88267	0.88667	0.89109
574	0.86699	0.87448	0.87796	0.87911	0.88284	0.88683	0.89124
575	0.86718	0.87466	0.87813	0.87928	0.88300	0.88699	0.89139
576	0.86736	0.87484	0.87830	0.87944	0.88316	0.88714	0.89154
577	0.86754	0.87501	0.87847	0.87961	0.88332	0.88730	0.89168
578	0.86773	0.87518	0.87864	0.87977	0.88348	0.88745	0.89183
579	0.86790	0.87535	0.87880	0.87994	0.88364	0.88761	0.89198
580	0.86808	0.87552	0.87896	0.88010	0.88380	0.88776	0.89213
581	0.86828	0.87569	0.87913	0.88027	0.88396	0.88791	0.89227
582	0.86844	0.87586	0.87930	0.88044	0.88412	0.88806	0.89242
583	0.86862	0.87603	0.87947	0.88060	0.88427	0.88822	0.89256
584	0.86880	0.87621	0.87963	0.88077	0.88443	0.88837	0.89271
585	0.86897	0.87637	0.87980	0.88093	0.88459	0.88852	0.89285
586	0.86914	0.87654	0.87996	0.88109	0.88474	0.88867	0.89300
587	0.86932	0.87671	0.88012	0.88125	0.88490	0.88882	0.89314
588	0.86951	0.87688	0.88029	0.88141	0.88506	0.88897	0.89328
589	0.86968	0.87705	0.88045	0.88158	0.88521	0.88911	0.89342
590	0.86986	0.87722	0.88061	0.88173	0.88536	0.88926	0.89357
591	0.87005	0.87738	0.88077	0.88189	0.88552	0.88942	0.89371
592	0.87022	0.87755	0.88093	0.88206	0.88567	0.88956	0.89385
593	0.87038	0.87772	0.88109	0.88221	0.88583	0.88971	0.89399
594	0.87058	0.87788	0.88126	0.88237	0.88598	0.88986	0.89413
595	0.87075	0.87805	0.88142	0.88253	0.88613	0.89001	0.89427
596	0.87094	0.87821	0.88158	0.88269	0.88629	0.89015	0.89441
597	0.87110	0.87838	0.88173	0.88285	0.88643	0.89029	0.89455
598	0.87127	0.87854	0.88189	0.88300	0.88659	0.89044	0.89469
599	0.87143	0.87870	0.88204	0.88316	0.88674	0.89058	0.89483
600	0.87159	0.87886	0.88220	0.88331	0.88689	0.89073	0.89497
601	0.87178	0.87902	0.88235	0.88347	0.88704	0.89087	0.89510
602	0.87195	0.87918	0.88252	0.88362	0.88719	0.89101	0.89524
603	0.87211	0.87934	0.88267	0.88377	0.88734	0.89116	0.89538
604	0.87228	0.87950	0.88283	0.88393	0.88748	0.89130	0.89551
605	0.87244	0.87966	0.88298	0.88408	0.88763	0.89144	0.89565
606	0.87261	0.87982	0.88313	0.88423	0.88777	0.89159	0.89578
607	0.87278	0.87999	0.88329	0.88439	0.88792	0.89173	0.89592
608	0.87296	0.88014	0.88344	0.88454	0.88807	0.89187	0.89605
609	0.87313	0.88030	0.88360	0.88469	0.88822	0.89201	0.89619
610	0.87329	0.88045	0.88375	0.88484	0.88836	0.89215	0.89632
611	0.87347	0.88061	0.88390	0.88498	0.88850	0.89229	0.89646
612	0.87363	0.88076	0.88405	0.88514	0.88865	0.89243	0.89659
613	0.87379	0.88091	0.88420	0.88529	0.88879	0.89257	0.89672
614	0.87396	0.88106	0.88435	0.88544	0.88894	0.89271	0.89686
615	0.87414	0.88123	0.88450	0.88559	0.88908	0.89285	0.89699
616	0.87429	0.88138	0.88465	0.88573	0.88922	0.89299	0.89712
617	0.87445	0.88153	0.88480	0.88588	0.88937	0.89312	0.89725
618	0.87461	0.88168	0.88495	0.88603	0.88951	0.89326	0.89739
619	0.87479	0.88184	0.88510	0.88618	0.88966	0.89340	0.89752
620	0.87496	0.88199	0.88525	0.88633	0.88980	0.89354	0.89765
621	0.87513	0.88214	0.88540	0.88647	0.88994	0.89367	0.89778
622	0.87530	0.88229	0.88555	0.88662	0.89008	0.89381	0.89791
623	0.87547	0.88245	0.88569	0.88676	0.89022	0.89394	0.89804

N	0.1%	0.5%	1%	1.25%	2.5%	5%	10%
624	0.87563	0.88260	0.88584	0.88691	0.89036	0.89408	0.89817
625	0.87578	0.88275	0.88598	0.88705	0.89050	0.89421	0.89830
626	0.87594	0.88290	0.88613	0.88720	0.89064	0.89434	0.89843
627	0.87611	0.88304	0.88628	0.88734	0.89078	0.89448	0.89855
628	0.87626	0.88320	0.88642	0.88749	0.89091	0.89461	0.89868
629	0.87641	0.88335	0.88657	0.88763	0.89105	0.89475	0.89881
630	0.87658	0.88350	0.88671	0.88777	0.89119	0.89488	0.89893
631	0.87672	0.88364	0.88686	0.88791	0.89133	0.89501	0.89906
632	0.87689	0.88379	0.88700	0.88806	0.89146	0.89515	0.89919
633	0.87704	0.88394	0.88714	0.88820	0.89160	0.89528	0.89931
634	0.87720	0.88408	0.88728	0.88834	0.89174	0.89541	0.89944
635	0.87735	0.88423	0.88743	0.88848	0.89188	0.89554	0.89956
636	0.87751	0.88437	0.88757	0.88862	0.89201	0.89567	0.89969
637	0.87767	0.88451	0.88771	0.88876	0.89215	0.89580	0.89981
638	0.87783	0.88466	0.88785	0.88889	0.89228	0.89593	0.89994
639	0.87798	0.88481	0.88799	0.88903	0.89242	0.89606	0.90006
640	0.87814	0.88495	0.88813	0.88917	0.89255	0.89619	0.90018
641	0.87830	0.88509	0.88827	0.88931	0.89268	0.89632	0.90031
642	0.87844	0.88523	0.88841	0.88945	0.89281	0.89645	0.90043
643	0.87858	0.88538	0.88855	0.88958	0.89295	0.89658	0.90055
644	0.87875	0.88552	0.88869	0.88972	0.89308	0.89670	0.90067
645	0.87890	0.88566	0.88883	0.88986	0.89321	0.89683	0.90080
646	0.87904	0.88581	0.88896	0.88999	0.89334	0.89696	0.90092
647	0.87918	0.88594	0.88910	0.89012	0.89347	0.89708	0.90104
648	0.87932	0.88608	0.88923	0.89026	0.89360	0.89721	0.90116
649	0.87947	0.88623	0.88938	0.89039	0.89374	0.89734	0.90128
650	0.87962	0.88637	0.88950	0.89053	0.89387	0.89746	0.90140
651	0.87977	0.88652	0.88964	0.89067	0.89400	0.89758	0.90152
652	0.87992	0.88666	0.88978	0.89080	0.89413	0.89771	0.90164
653	0.88007	0.88681	0.88991	0.89093	0.89426	0.89784	0.90176
654	0.88023	0.88694	0.89005	0.89107	0.89439	0.89796	0.90188
655	0.88038	0.88708	0.89017	0.89120	0.89452	0.89808	0.90200
656	0.88051	0.88722	0.89031	0.89133	0.89465	0.89821	0.90211
657	0.88066	0.88736	0.89044	0.89147	0.89477	0.89833	0.90223
658	0.88081	0.88751	0.89057	0.89160	0.89490	0.89846	0.90235
659	0.88095	0.88763	0.89071	0.89173	0.89503	0.89858	0.90247
660	0.88111	0.88777	0.89084	0.89186	0.89516	0.89870	0.90259
661	0.88125	0.88791	0.89097	0.89200	0.89528	0.89882	0.90270
662	0.88141	0.88804	0.89111	0.89213	0.89541	0.89895	0.90282
663	0.88156	0.88818	0.89124	0.89226	0.89553	0.89907	0.90294
664	0.88170	0.88831	0.89137	0.89239	0.89566	0.89919	0.90305
665	0.88184	0.88845	0.89150	0.89252	0.89579	0.89931	0.90317
666	0.88199	0.88859	0.89163	0.89265	0.89591	0.89943	0.90328
667	0.88214	0.88872	0.89176	0.89278	0.89604	0.89955	0.90340
668	0.88228	0.88885	0.89189	0.89290	0.89616	0.89967	0.90351
669	0.88243	0.88898	0.89202	0.89304	0.89629	0.89979	0.90363
670	0.88259	0.88912	0.89214	0.89316	0.89641	0.89991	0.90374
671	0.88271	0.88925	0.89227	0.89329	0.89653	0.90003	0.90385
672	0.88286	0.88938	0.89240	0.89341	0.89666	0.90015	0.90397
673	0.88300	0.88952	0.89253	0.89354	0.89678	0.90026	0.90408
674	0.88313	0.88965	0.89266	0.89367	0.89690	0.90038	0.90419
675	0.88327	0.88978	0.89279	0.89379	0.89702	0.90050	0.90430
676	0.88340	0.88991	0.89291	0.89391	0.89715	0.90062	0.90442
677	0.88355	0.89004	0.89304	0.89404	0.89727	0.90073	0.90453
678	0.88369	0.89018	0.89317	0.89416	0.89739	0.90085	0.90464
679	0.88384	0.89031	0.89330	0.89429	0.89751	0.90097	0.90475
680	0.88397	0.89044	0.89343	0.89442	0.89763	0.90108	0.90486
681	0.88411	0.89057	0.89355	0.89454	0.89775	0.90120	0.90497
682	0.88425	0.89069	0.89368	0.89467	0.89787	0.90132	0.90508
683	0.88438	0.89083	0.89381	0.89480	0.89799	0.90143	0.90519
684	0.88452	0.89095	0.89393	0.89492	0.89811	0.90155	0.90530
685	0.88466	0.89109	0.89406	0.89504	0.89823	0.90166	0.90541
686	0.88480	0.89121	0.89418	0.89517	0.89835	0.90177	0.90552
687	0.88495	0.89134	0.89430	0.89529	0.89847	0.90189	0.90563
688	0.88509	0.89147	0.89443	0.89542	0.89859	0.90200	0.90574
689	0.88522	0.89159	0.89455	0.89553	0.89870	0.90211	0.90585
690	0.88536	0.89172	0.89468	0.89565	0.89882	0.90222	0.90596
691	0.88548	0.89185	0.89480	0.89578	0.89894	0.90234	0.90606
692	0.88562	0.89198	0.89492	0.89590	0.89905	0.90245	0.90617
693	0.88576	0.89210	0.89504	0.89602	0.89917	0.90257	0.90628
694	0.88589	0.89223	0.89516	0.89614	0.89929	0.90268	0.90639
695	0.88601	0.89236	0.89528	0.89626	0.89940	0.90279	0.90650
696	0.88614	0.89248	0.89541	0.89638	0.89952	0.90290	0.90660
697	0.88628	0.89261	0.89553	0.89650	0.89963	0.90301	0.90671

N	0.1%	0.5%	1%	1.25%	2.5%	5%	10%
698	0.88641	0.89273	0.89565	0.89662	0.89975	0.90313	0.90682
699	0.88654	0.89286	0.89577	0.89674	0.89986	0.90324	0.90692
700	0.88668	0.89298	0.89589	0.89685	0.89997	0.90334	0.90703
701	0.88681	0.89310	0.89601	0.89697	0.90009	0.90345	0.90713
702	0.88694	0.89322	0.89613	0.89709	0.90020	0.90356	0.90724
703	0.88707	0.89335	0.89625	0.89721	0.90032	0.90367	0.90734
704	0.88721	0.89347	0.89637	0.89733	0.90043	0.90378	0.90745
705	0.88733	0.89359	0.89649	0.89745	0.90054	0.90389	0.90755
706	0.88747	0.89371	0.89661	0.89756	0.90066	0.90400	0.90765
707	0.88760	0.89383	0.89672	0.89768	0.90077	0.90410	0.90776
708	0.88772	0.89395	0.89684	0.89780	0.90089	0.90421	0.90786
709	0.88785	0.89407	0.89696	0.89791	0.90100	0.90432	0.90796
710	0.88798	0.89419	0.89708	0.89803	0.90111	0.90443	0.90807
711	0.88810	0.89431	0.89719	0.89815	0.90122	0.90454	0.90817
712	0.88823	0.89443	0.89731	0.89826	0.90134	0.90464	0.90827
713	0.88835	0.89456	0.89742	0.89838	0.90145	0.90475	0.90838
714	0.88847	0.89467	0.89754	0.89849	0.90156	0.90486	0.90848
715	0.88860	0.89479	0.89765	0.89860	0.90167	0.90496	0.90858
716	0.88871	0.89491	0.89777	0.89872	0.90178	0.90507	0.90868
717	0.88884	0.89503	0.89788	0.89883	0.90189	0.90518	0.90878
718	0.88896	0.89515	0.89800	0.89895	0.90200	0.90528	0.90889
719	0.88909	0.89528	0.89811	0.89906	0.90210	0.90539	0.90899
720	0.88921	0.89539	0.89823	0.89917	0.90222	0.90549	0.90909
721	0.88934	0.89550	0.89834	0.89928	0.90232	0.90560	0.90919
722	0.88946	0.89562	0.89846	0.89940	0.90243	0.90570	0.90929
723	0.88959	0.89574	0.89857	0.89951	0.90254	0.90581	0.90939
724	0.88972	0.89587	0.89868	0.89962	0.90265	0.90591	0.90949
725	0.88982	0.89598	0.89879	0.89973	0.90276	0.90601	0.90958
726	0.88996	0.89610	0.89891	0.89984	0.90286	0.90612	0.90968
727	0.89008	0.89621	0.89902	0.89996	0.90297	0.90622	0.90978
728	0.89021	0.89633	0.89913	0.90006	0.90308	0.90632	0.90988
729	0.89032	0.89644	0.89924	0.90017	0.90318	0.90642	0.90998
730	0.89045	0.89655	0.89936	0.90029	0.90329	0.90653	0.91007
731	0.89058	0.89667	0.89947	0.90040	0.90340	0.90663	0.91017
732	0.89070	0.89679	0.89958	0.90051	0.90350	0.90673	0.91027
733	0.89082	0.89690	0.89969	0.90062	0.90361	0.90683	0.91037
734	0.89095	0.89701	0.89980	0.90073	0.90372	0.90693	0.91047
735	0.89107	0.89713	0.89991	0.90084	0.90382	0.90703	0.91056
736	0.89120	0.89724	0.90002	0.90094	0.90393	0.90714	0.91066
737	0.89131	0.89735	0.90013	0.90105	0.90403	0.90724	0.91076
738	0.89144	0.89747	0.90024	0.90116	0.90414	0.90734	0.91085
739	0.89155	0.89758	0.90035	0.90127	0.90424	0.90744	0.91095
740	0.89167	0.89769	0.90046	0.90138	0.90434	0.90754	0.91105
741	0.89179	0.89780	0.90057	0.90149	0.90445	0.90764	0.91114
742	0.89189	0.89791	0.90068	0.90159	0.90455	0.90774	0.91124
743	0.89201	0.89802	0.90078	0.90170	0.90466	0.90784	0.91133
744	0.89213	0.89814	0.90090	0.90180	0.90476	0.90794	0.91143
745	0.89224	0.89825	0.90101	0.90191	0.90486	0.90804	0.91152
746	0.89237	0.89837	0.90111	0.90202	0.90497	0.90813	0.91162
747	0.89250	0.89848	0.90122	0.90213	0.90507	0.90823	0.91171
748	0.89262	0.89858	0.90133	0.90223	0.90517	0.90833	0.91181
749	0.89274	0.89869	0.90144	0.90234	0.90527	0.90843	0.91190
750	0.89286	0.89880	0.90154	0.90244	0.90537	0.90853	0.91199
751	0.89299	0.89891	0.90164	0.90255	0.90547	0.90863	0.91209
752	0.89310	0.89902	0.90175	0.90265	0.90558	0.90872	0.91218
753	0.89321	0.89912	0.90186	0.90276	0.90568	0.90882	0.91227
754	0.89334	0.89923	0.90196	0.90286	0.90578	0.90892	0.91237
755	0.89345	0.89934	0.90206	0.90297	0.90588	0.90901	0.91246
756	0.89357	0.89945	0.90217	0.90308	0.90598	0.90911	0.91255
757	0.89368	0.89956	0.90228	0.90318	0.90608	0.90921	0.91264
758	0.89378	0.89967	0.90238	0.90328	0.90618	0.90930	0.91273
759	0.89390	0.89978	0.90249	0.90338	0.90628	0.90940	0.91283
760	0.89402	0.89989	0.90259	0.90348	0.90638	0.90950	0.91292
761	0.89413	0.89999	0.90270	0.90359	0.90648	0.90959	0.91301
762	0.89425	0.90010	0.90280	0.90369	0.90658	0.90969	0.91310
763	0.89436	0.90020	0.90290	0.90379	0.90667	0.90978	0.91319
764	0.89448	0.90031	0.90300	0.90390	0.90677	0.90988	0.91328
765	0.89459	0.90041	0.90311	0.90400	0.90687	0.90997	0.91337
766	0.89469	0.90052	0.90321	0.90410	0.90697	0.91007	0.91346
767	0.89481	0.90063	0.90331	0.90419	0.90707	0.91016	0.91355
768	0.89492	0.90074	0.90341	0.90430	0.90717	0.91025	0.91364
769	0.89504	0.90084	0.90352	0.90440	0.90726	0.91035	0.91373
770	0.89515	0.90095	0.90362	0.90450	0.90736	0.91044	0.91382
771	0.89525	0.90105	0.90371	0.90460	0.90746	0.91053	0.91391

N	0.1%	0.5%	1%	1.25%	2.5%	5%	10%
772	0.89537	0.90115	0.90381	0.90470	0.90755	0.91063	0.91400
773	0.89549	0.90125	0.90391	0.90480	0.90765	0.91072	0.91409
774	0.89560	0.90135	0.90401	0.90490	0.90774	0.91081	0.91418
775	0.89570	0.90146	0.90411	0.90500	0.90784	0.91090	0.91427
776	0.89581	0.90157	0.90421	0.90510	0.90794	0.91099	0.91435
777	0.89591	0.90167	0.90431	0.90520	0.90803	0.91108	0.91444
778	0.89603	0.90177	0.90441	0.90529	0.90813	0.91118	0.91453
779	0.89614	0.90187	0.90451	0.90539	0.90822	0.91127	0.91462
780	0.89625	0.90197	0.90461	0.90549	0.90832	0.91136	0.91470
781	0.89636	0.90208	0.90471	0.90559	0.90841	0.91145	0.91479
782	0.89648	0.90218	0.90481	0.90568	0.90851	0.91154	0.91488
783	0.89658	0.90228	0.90491	0.90578	0.90860	0.91163	0.91497
784	0.89668	0.90239	0.90501	0.90588	0.90869	0.91172	0.91505
785	0.89678	0.90248	0.90510	0.90598	0.90879	0.91181	0.91514
786	0.89689	0.90259	0.90520	0.90607	0.90888	0.91190	0.91522
787	0.89701	0.90268	0.90530	0.90617	0.90898	0.91199	0.91531
788	0.89710	0.90278	0.90540	0.90626	0.90907	0.91208	0.91540
789	0.89722	0.90289	0.90550	0.90636	0.90917	0.91217	0.91548
790	0.89733	0.90298	0.90559	0.90646	0.90926	0.91226	0.91557
791	0.89743	0.90308	0.90568	0.90655	0.90935	0.91235	0.91565
792	0.89754	0.90319	0.90578	0.90665	0.90944	0.91244	0.91574
793	0.89765	0.90329	0.90587	0.90674	0.90953	0.91253	0.91583
794	0.89775	0.90339	0.90597	0.90684	0.90963	0.91262	0.91591
795	0.89786	0.90348	0.90607	0.90693	0.90972	0.91270	0.91600
796	0.89795	0.90358	0.90616	0.90702	0.90981	0.91279	0.91608
797	0.89805	0.90368	0.90626	0.90712	0.90990	0.91288	0.91616
798	0.89816	0.90378	0.90635	0.90721	0.90999	0.91297	0.91625
799	0.89827	0.90388	0.90645	0.90731	0.91008	0.91306	0.91633
800	0.89837	0.90397	0.90655	0.90740	0.91017	0.91314	0.91642
801	0.89846	0.90407	0.90664	0.90749	0.91026	0.91323	0.91650
802	0.89857	0.90416	0.90673	0.90759	0.91035	0.91332	0.91658
803	0.89868	0.90426	0.90683	0.90768	0.91044	0.91341	0.91667
804	0.89878	0.90435	0.90692	0.90778	0.91053	0.91349	0.91675
805	0.89888	0.90445	0.90702	0.90787	0.91062	0.91358	0.91683
806	0.89899	0.90455	0.90711	0.90796	0.91071	0.91366	0.91691
807	0.89910	0.90464	0.90721	0.90805	0.91080	0.91375	0.91700
808	0.89920	0.90474	0.90730	0.90814	0.91089	0.91384	0.91708
809	0.89930	0.90484	0.90739	0.90824	0.91098	0.91392	0.91716
810	0.89941	0.90493	0.90749	0.90833	0.91107	0.91401	0.91724
811	0.89952	0.90503	0.90758	0.90842	0.91115	0.91409	0.91733
812	0.89962	0.90512	0.90767	0.90851	0.91124	0.91418	0.91741
813	0.89971	0.90521	0.90776	0.90860	0.91133	0.91426	0.91749
814	0.89982	0.90531	0.90785	0.90869	0.91142	0.91435	0.91757
815	0.89992	0.90540	0.90795	0.90879	0.91151	0.91443	0.91765
816	0.90002	0.90550	0.90804	0.90888	0.91160	0.91452	0.91773
817	0.90011	0.90560	0.90813	0.90896	0.91168	0.91460	0.91781
818	0.90022	0.90569	0.90822	0.90905	0.91177	0.91469	0.91789
819	0.90031	0.90579	0.90832	0.90914	0.91186	0.91477	0.91797
820	0.90041	0.90588	0.90840	0.90923	0.91195	0.91485	0.91805
821	0.90051	0.90596	0.90849	0.90932	0.91203	0.91494	0.91813
822	0.90060	0.90606	0.90858	0.90941	0.91212	0.91502	0.91821
823	0.90070	0.90616	0.90868	0.90951	0.91220	0.91510	0.91829
824	0.90080	0.90625	0.90877	0.90959	0.91229	0.91519	0.91837
825	0.90090	0.90634	0.90886	0.90968	0.91238	0.91527	0.91845
826	0.90100	0.90643	0.90895	0.90977	0.91246	0.91535	0.91853
827	0.90110	0.90652	0.90904	0.90986	0.91255	0.91544	0.91861
828	0.90120	0.90662	0.90913	0.90995	0.91263	0.91552	0.91869
829	0.90130	0.90671	0.90922	0.91004	0.91272	0.91560	0.91877
830	0.90140	0.90680	0.90930	0.91012	0.91280	0.91568	0.91884
831	0.90149	0.90690	0.90939	0.91021	0.91288	0.91576	0.91892
832	0.90158	0.90699	0.90948	0.91030	0.91297	0.91584	0.91900
833	0.90168	0.90708	0.90957	0.91039	0.91305	0.91592	0.91908
834	0.90179	0.90717	0.90966	0.91048	0.91314	0.91601	0.91916
835	0.90190	0.90726	0.90975	0.91057	0.91322	0.91609	0.91923
836	0.90198	0.90735	0.90983	0.91065	0.91331	0.91617	0.91931
837	0.90207	0.90744	0.90992	0.91074	0.91339	0.91625	0.91939
838	0.90218	0.90753	0.91001	0.91082	0.91348	0.91633	0.91947
839	0.90227	0.90762	0.91010	0.91091	0.91356	0.91641	0.91954
840	0.90237	0.90771	0.91019	0.91100	0.91364	0.91649	0.91962
841	0.90247	0.90780	0.91027	0.91108	0.91373	0.91657	0.91970
842	0.90256	0.90790	0.91036	0.91117	0.91381	0.91665	0.91977
843	0.90266	0.90798	0.91045	0.91126	0.91389	0.91673	0.91985
844	0.90276	0.90807	0.91053	0.91134	0.91397	0.91681	0.91993
845	0.90286	0.90817	0.91062	0.91142	0.91405	0.91689	0.92000

N	0.1%	0.5%	1%	1.25%	2.5%	5%	10%
846	0.90294	0.90825	0.91071	0.91151	0.91414	0.91697	0.92008
847	0.90304	0.90834	0.91079	0.91159	0.91422	0.91704	0.92016
848	0.90313	0.90843	0.91088	0.91167	0.91430	0.91712	0.92023
849	0.90322	0.90852	0.91096	0.91176	0.91438	0.91720	0.92031
850	0.90332	0.90860	0.91105	0.91184	0.91446	0.91728	0.92038
851	0.90341	0.90869	0.91113	0.91193	0.91454	0.91736	0.92046
852	0.90350	0.90878	0.91122	0.91201	0.91462	0.91744	0.92053
853	0.90359	0.90887	0.91130	0.91210	0.91471	0.91751	0.92061
854	0.90368	0.90895	0.91139	0.91218	0.91479	0.91759	0.92068
855	0.90378	0.90904	0.91147	0.91227	0.91487	0.91767	0.92075
856	0.90387	0.90914	0.91156	0.91235	0.91495	0.91775	0.92083
857	0.90396	0.90922	0.91164	0.91243	0.91503	0.91783	0.92090
858	0.90407	0.90931	0.91172	0.91251	0.91510	0.91790	0.92098
859	0.90416	0.90940	0.91180	0.91260	0.91519	0.91798	0.92105
860	0.90425	0.90948	0.91189	0.91268	0.91527	0.91806	0.92113
861	0.90434	0.90957	0.91197	0.91276	0.91535	0.91814	0.92120
862	0.90443	0.90965	0.91205	0.91285	0.91543	0.91821	0.92127
863	0.90452	0.90974	0.91213	0.91293	0.91551	0.91829	0.92135
864	0.90460	0.90982	0.91222	0.91301	0.91559	0.91837	0.92142
865	0.90469	0.90991	0.91230	0.91309	0.91567	0.91844	0.92149
866	0.90478	0.90999	0.91238	0.91317	0.91575	0.91852	0.92156
867	0.90488	0.91008	0.91247	0.91325	0.91583	0.91860	0.92164
868	0.90496	0.91016	0.91255	0.91334	0.91590	0.91867	0.92171
869	0.90505	0.91025	0.91263	0.91342	0.91598	0.91875	0.92178
870	0.90515	0.91033	0.91271	0.91350	0.91606	0.91882	0.92185
871	0.90524	0.91041	0.91279	0.91358	0.91614	0.91890	0.92193
872	0.90533	0.91050	0.91287	0.91366	0.91622	0.91897	0.92200
873	0.90541	0.91058	0.91295	0.91374	0.91629	0.91905	0.92207
874	0.90551	0.91066	0.91303	0.91382	0.91637	0.91912	0.92214
875	0.90560	0.91074	0.91311	0.91390	0.91645	0.91919	0.92221
876	0.90569	0.91082	0.91319	0.91398	0.91653	0.91927	0.92229
877	0.90577	0.91091	0.91327	0.91406	0.91660	0.91934	0.92236
878	0.90586	0.91099	0.91335	0.91414	0.91668	0.91942	0.92243
879	0.90595	0.91108	0.91344	0.91422	0.91676	0.91949	0.92250
880	0.90603	0.91116	0.91352	0.91429	0.91683	0.91957	0.92257
881	0.90612	0.91124	0.91360	0.91437	0.91691	0.91964	0.92264
882	0.90621	0.91133	0.91368	0.91445	0.91699	0.91971	0.92271
883	0.90628	0.91141	0.91376	0.91453	0.91706	0.91979	0.92278
884	0.90637	0.91149	0.91383	0.91461	0.91714	0.91986	0.92285
885	0.90646	0.91157	0.91392	0.91469	0.91721	0.91993	0.92292
886	0.90656	0.91165	0.91399	0.91476	0.91729	0.92001	0.92299
887	0.90664	0.91173	0.91407	0.91484	0.91737	0.92008	0.92306
888	0.90673	0.91182	0.91415	0.91492	0.91744	0.92015	0.92313
889	0.90681	0.91190	0.91423	0.91500	0.91752	0.92022	0.92320
890	0.90692	0.91198	0.91431	0.91508	0.91759	0.92030	0.92327
891	0.90699	0.91206	0.91439	0.91516	0.91767	0.92037	0.92334
892	0.90708	0.91214	0.91447	0.91524	0.91775	0.92044	0.92341
893	0.90716	0.91222	0.91455	0.91531	0.91782	0.92051	0.92348
894	0.90725	0.91230	0.91462	0.91539	0.91789	0.92059	0.92355
895	0.90734	0.91238	0.91470	0.91547	0.91797	0.92066	0.92362
896	0.90742	0.91246	0.91478	0.91555	0.91804	0.92073	0.92368
897	0.90751	0.91254	0.91486	0.91563	0.91812	0.92080	0.92375
898	0.90759	0.91262	0.91493	0.91570	0.91819	0.92087	0.92382
899	0.90768	0.91270	0.91501	0.91578	0.91827	0.92095	0.92389
900	0.90776	0.91277	0.91509	0.91585	0.91834	0.92102	0.92396
901	0.90784	0.91285	0.91517	0.91593	0.91841	0.92109	0.92403
902	0.90792	0.91294	0.91524	0.91601	0.91848	0.92116	0.92409
903	0.90800	0.91301	0.91532	0.91608	0.91856	0.92123	0.92416
904	0.90809	0.91309	0.91540	0.91616	0.91863	0.92130	0.92423
905	0.90817	0.91317	0.91547	0.91623	0.91870	0.92137	0.92430
906	0.90825	0.91325	0.91555	0.91631	0.91878	0.92144	0.92436
907	0.90832	0.91333	0.91562	0.91638	0.91885	0.92151	0.92443
908	0.90841	0.91341	0.91570	0.91646	0.91892	0.92158	0.92450
909	0.90850	0.91349	0.91578	0.91653	0.91900	0.92165	0.92456
910	0.90858	0.91356	0.91585	0.91661	0.91907	0.92172	0.92463
911	0.90866	0.91364	0.91593	0.91669	0.91914	0.92179	0.92470
912	0.90874	0.91372	0.91600	0.91676	0.91921	0.92186	0.92476
913	0.90882	0.91380	0.91608	0.91684	0.91929	0.92193	0.92483
914	0.90891	0.91387	0.91615	0.91691	0.91936	0.92199	0.92489
915	0.90899	0.91395	0.91622	0.91698	0.91943	0.92206	0.92496
916	0.90908	0.91403	0.91630	0.91705	0.91950	0.92213	0.92503
917	0.90916	0.91410	0.91637	0.91713	0.91957	0.92220	0.92509
918	0.90925	0.91418	0.91645	0.91720	0.91964	0.92227	0.92516
919	0.90934	0.91426	0.91652	0.91728	0.91971	0.92234	0.92522

N	0.1%	0.5%	1%	1.25%	2.5%	5%	10%
920	0.90941	0.91434	0.91660	0.91735	0.91978	0.92241	0.92529
921	0.90949	0.91442	0.91667	0.91742	0.91985	0.92247	0.92536
922	0.90958	0.91449	0.91675	0.91750	0.91993	0.92254	0.92542
923	0.90966	0.91457	0.91682	0.91757	0.92000	0.92261	0.92549
924	0.90974	0.91464	0.91689	0.91764	0.92007	0.92268	0.92555
925	0.90982	0.91472	0.91697	0.91771	0.92014	0.92275	0.92562
926	0.90989	0.91479	0.91704	0.91779	0.92021	0.92281	0.92568
927	0.90998	0.91487	0.91711	0.91786	0.92028	0.92288	0.92575
928	0.91005	0.91495	0.91719	0.91793	0.92035	0.92295	0.92581
929	0.91013	0.91502	0.91726	0.91800	0.92042	0.92301	0.92587
930	0.91021	0.91509	0.91733	0.91807	0.92049	0.92308	0.92594
931	0.91030	0.91517	0.91740	0.91815	0.92056	0.92315	0.92600
932	0.91038	0.91524	0.91747	0.91822	0.92062	0.92321	0.92607
933	0.91046	0.91532	0.91754	0.91829	0.92070	0.92328	0.92613
934	0.91055	0.91539	0.91762	0.91836	0.92077	0.92335	0.92620
935	0.91063	0.91547	0.91769	0.91843	0.92083	0.92341	0.92626
936	0.91071	0.91554	0.91776	0.91850	0.92091	0.92348	0.92632
937	0.91078	0.91562	0.91783	0.91857	0.92097	0.92355	0.92639
938	0.91086	0.91569	0.91791	0.91864	0.92104	0.92361	0.92645
939	0.91094	0.91576	0.91798	0.91872	0.92111	0.92368	0.92651
940	0.91102	0.91584	0.91805	0.91879	0.92118	0.92374	0.92658
941	0.91110	0.91591	0.91813	0.91886	0.92125	0.92381	0.92664
942	0.91118	0.91599	0.91819	0.91893	0.92132	0.92388	0.92670
943	0.91126	0.91606	0.91827	0.91900	0.92139	0.92394	0.92676
944	0.91133	0.91613	0.91834	0.91906	0.92145	0.92401	0.92683
945	0.91140	0.91620	0.91841	0.91914	0.92152	0.92407	0.92689
946	0.91148	0.91627	0.91848	0.91921	0.92159	0.92414	0.92695
947	0.91156	0.91634	0.91855	0.91928	0.92165	0.92420	0.92702
948	0.91164	0.91642	0.91862	0.91935	0.92172	0.92427	0.92708
949	0.91171	0.91649	0.91869	0.91942	0.92179	0.92433	0.92714
950	0.91178	0.91656	0.91876	0.91949	0.92186	0.92440	0.92720
951	0.91186	0.91663	0.91882	0.91956	0.92192	0.92446	0.92726
952	0.91193	0.91671	0.91890	0.91963	0.92199	0.92453	0.92732
953	0.91201	0.91678	0.91897	0.91970	0.92206	0.92459	0.92739
954	0.91208	0.91685	0.91904	0.91976	0.92212	0.92466	0.92745
955	0.91215	0.91692	0.91911	0.91983	0.92219	0.92472	0.92751
956	0.91222	0.91699	0.91918	0.91990	0.92226	0.92479	0.92757
957	0.91230	0.91706	0.91925	0.91997	0.92232	0.92485	0.92763
958	0.91238	0.91713	0.91932	0.92004	0.92239	0.92491	0.92769
959	0.91246	0.91721	0.91938	0.92011	0.92246	0.92498	0.92775
960	0.91253	0.91728	0.91945	0.92018	0.92252	0.92504	0.92781
961	0.91261	0.91735	0.91952	0.92025	0.92259	0.92510	0.92787
962	0.91269	0.91742	0.91959	0.92032	0.92265	0.92517	0.92793
963	0.91277	0.91749	0.91966	0.92039	0.92272	0.92523	0.92799
964	0.91284	0.91756	0.91973	0.92045	0.92278	0.92529	0.92805
965	0.91291	0.91763	0.91980	0.92052	0.92285	0.92535	0.92811
966	0.91300	0.91770	0.91986	0.92059	0.92291	0.92542	0.92817
967	0.91307	0.91778	0.91993	0.92066	0.92298	0.92548	0.92823
968	0.91315	0.91785	0.92000	0.92072	0.92304	0.92554	0.92829
969	0.91322	0.91792	0.92007	0.92079	0.92311	0.92561	0.92835
970	0.91330	0.91799	0.92014	0.92086	0.92317	0.92567	0.92841
971	0.91336	0.91806	0.92020	0.92093	0.92324	0.92573	0.92847
972	0.91343	0.91812	0.92027	0.92099	0.92330	0.92579	0.92853
973	0.91351	0.91819	0.92034	0.92106	0.92337	0.92585	0.92859
974	0.91359	0.91826	0.92040	0.92113	0.92343	0.92592	0.92865
975	0.91366	0.91833	0.92047	0.92119	0.92350	0.92598	0.92871
976	0.91374	0.91840	0.92054	0.92126	0.92356	0.92604	0.92877
977	0.91381	0.91847	0.92061	0.92132	0.92362	0.92610	0.92883
978	0.91388	0.91854	0.92067	0.92139	0.92369	0.92616	0.92889
979	0.91395	0.91861	0.92074	0.92145	0.92375	0.92622	0.92895
980	0.91403	0.91868	0.92080	0.92152	0.92381	0.92629	0.92901
981	0.91409	0.91874	0.92087	0.92158	0.92388	0.92635	0.92906
982	0.91417	0.91881	0.92094	0.92165	0.92394	0.92641	0.92912
983	0.91424	0.91888	0.92101	0.92172	0.92400	0.92647	0.92918
984	0.91432	0.91895	0.92107	0.92178	0.92406	0.92653	0.92924
985	0.91439	0.91902	0.92114	0.92184	0.92413	0.92659	0.92930
986	0.91447	0.91909	0.92120	0.92191	0.92419	0.92665	0.92935
987	0.91454	0.91915	0.92127	0.92197	0.92425	0.92671	0.92941
988	0.91460	0.91922	0.92133	0.92204	0.92432	0.92677	0.92947
989	0.91468	0.91929	0.92140	0.92210	0.92438	0.92683	0.92953
990	0.91475	0.91936	0.92146	0.92217	0.92444	0.92689	0.92959
991	0.91482	0.91942	0.92153	0.92223	0.92451	0.92695	0.92964
992	0.91489	0.91949	0.92160	0.92229	0.92457	0.92701	0.92970
993	0.91496	0.91955	0.92166	0.92236	0.92463	0.92707	0.92976

N	0.1%	0.5%	1%	1.25%	2.5%	5%	10%
994	0.91504	0.91962	0.92173	0.92242	0.92469	0.92713	0.92981
995	0.91511	0.91969	0.92179	0.92249	0.92475	0.92719	0.92987
996	0.91518	0.91975	0.92185	0.92255	0.92482	0.92725	0.92993
997	0.91526	0.91982	0.92191	0.92261	0.92488	0.92731	0.92999
998	0.91533	0.91988	0.92198	0.92268	0.92494	0.92737	0.93004
999	0.91540	0.91995	0.92204	0.92274	0.92500	0.92743	0.93010
1000	0.91547	0.92002	0.92210	0.92280	0.92506	0.92749	0.93016
