# Supplementary material for: A generalized outlier method with an automated test protocol applied to proficiency testing schemes: utilizing Monte Carlo simulation for generating critical values for the extended Grubbs statistic
Source: J AOAC Int. 2026 Apr 29;109(6):974–84. doi: 10.1093/jaoacint/qsag036 (PMC13732683; doi:10.1093/jaoacint/qsag036)
Supplement: Supplementary file 1 [file qsag036_supplementary_data.zip › aoac-25-0307-File020.pdf]

Extended Grubbs Statistics Tables for Multiple-8 Outliers

Table 1 – *GM8(8H0L or 0H8L)*. Critical values of upper significance levels for the Grubbs multiple-8 outlier test, applicable in situations where the eight highest or eight lowest observations are simultaneously identified as outliers, denoted as *GM8(8H0L)* and *GM8(0H8L)*, respectively.

N	0.1%	0.5%	1%	1.25%	2.5%	5%	10%
10	0.00000	0.00000	0.00000	0.00000	0.00002	0.00007	0.00030
11	0.00004	0.00020	0.00040	0.00050	0.00103	0.00217	0.00468
12	0.00050	0.00152	0.00249	0.00292	0.00484	0.00816	0.01409
13	0.00199	0.00468	0.00685	0.00777	0.01156	0.01741	0.02687
14	0.00482	0.00976	0.01337	0.01483	0.02057	0.02896	0.04167
15	0.00899	0.01649	0.02160	0.02357	0.03125	0.04201	0.05758
16	0.01450	0.02458	0.03117	0.03368	0.04318	0.05603	0.07404
17	0.02099	0.03378	0.04177	0.04478	0.05591	0.07060	0.09072
18	0.02866	0.04381	0.05305	0.05653	0.06909	0.08539	0.10729
19	0.03679	0.05445	0.06490	0.06878	0.08258	0.10029	0.12363
20	0.04571	0.06548	0.07692	0.08113	0.09617	0.11504	0.13969
21	0.05509	0.07684	0.08921	0.09371	0.10974	0.12965	0.15530
22	0.06470	0.08825	0.10150	0.10629	0.12314	0.14404	0.17050
23	0.07477	0.09989	0.11378	0.11881	0.13645	0.15806	0.18520
24	0.08482	0.11137	0.12593	0.13117	0.14958	0.17174	0.19944
25	0.09499	0.12272	0.13792	0.14340	0.16232	0.18509	0.21326
26	0.10499	0.13412	0.14987	0.15544	0.17485	0.19799	0.22652
27	0.11520	0.14542	0.16154	0.16728	0.18705	0.21053	0.23934
28	0.12531	0.15644	0.17301	0.17885	0.19892	0.22276	0.25176
29	0.13522	0.16726	0.18418	0.19010	0.21058	0.23460	0.26376
30	0.14519	0.17791	0.19511	0.20118	0.22187	0.24603	0.27536
31	0.15508	0.18839	0.20581	0.21194	0.23287	0.25726	0.28655
32	0.16457	0.19869	0.21639	0.22250	0.24353	0.26802	0.29739
33	0.17425	0.20871	0.22659	0.23279	0.25392	0.27847	0.30780
34	0.18353	0.21867	0.23646	0.24276	0.26403	0.28866	0.31793
35	0.19262	0.22826	0.24636	0.25261	0.27383	0.29850	0.32770
36	0.20176	0.23771	0.25576	0.26209	0.28343	0.30806	0.33717
37	0.21083	0.24694	0.26505	0.27142	0.29275	0.31732	0.34637
38	0.21973	0.25583	0.27415	0.28052	0.30180	0.32634	0.35527
39	0.22839	0.26467	0.28293	0.28926	0.31063	0.33512	0.36387
40	0.23694	0.27320	0.29161	0.29792	0.31918	0.34363	0.37221
41	0.24527	0.28155	0.29997	0.30631	0.32757	0.35193	0.38036
42	0.25332	0.28973	0.30816	0.31452	0.33574	0.35992	0.38823
43	0.26119	0.29770	0.31611	0.32243	0.34367	0.36779	0.39586
44	0.26893	0.30550	0.32388	0.33018	0.35141	0.37538	0.40326
45	0.27677	0.31314	0.33149	0.33783	0.35894	0.38276	0.41051
46	0.28405	0.32075	0.33891	0.34527	0.36623	0.38995	0.41751
47	0.29124	0.32803	0.34619	0.35249	0.37342	0.39696	0.42435
48	0.29835	0.33511	0.35326	0.35960	0.38034	0.40380	0.43098
49	0.30541	0.34204	0.36019	0.36642	0.38713	0.41046	0.43745
50	0.31227	0.34885	0.36693	0.37311	0.39374	0.41698	0.44372
51	0.31895	0.35555	0.37355	0.37967	0.40020	0.42332	0.44986
52	0.32571	0.36214	0.37994	0.38609	0.40655	0.42950	0.45587
53	0.33217	0.36855	0.38625	0.39239	0.41271	0.43548	0.46171
54	0.33863	0.37465	0.39244	0.39848	0.41873	0.44141	0.46738
55	0.34470	0.38073	0.39844	0.40450	0.42464	0.44714	0.47291
56	0.35080	0.38674	0.40434	0.41036	0.43040	0.45275	0.47833
57	0.35661	0.39258	0.41015	0.41616	0.43609	0.45821	0.48363
58	0.36245	0.39838	0.41580	0.42184	0.44155	0.46356	0.48879
59	0.36824	0.40396	0.42135	0.42730	0.44694	0.46880	0.49385
60	0.37385	0.40950	0.42681	0.43272	0.45220	0.47394	0.49880
61	0.37944	0.41499	0.43214	0.43803	0.45739	0.47899	0.50364
62	0.38486	0.42025	0.43728	0.44314	0.46246	0.48387	0.50836
63	0.39018	0.42542	0.44245	0.44824	0.46739	0.48866	0.51295
64	0.39548	0.43041	0.44743	0.45316	0.47221	0.49335	0.51743
65	0.40053	0.43541	0.45227	0.45804	0.47694	0.49794	0.52185
66	0.40578	0.44029	0.45712	0.46285	0.48154	0.50242	0.52617
67	0.41075	0.44507	0.46181	0.46748	0.48611	0.50682	0.53040
68	0.41560	0.44977	0.46643	0.47211	0.49057	0.51113	0.53454
69	0.42039	0.45444	0.47098	0.47657	0.49496	0.51536	0.53862
70	0.42500	0.45889	0.47546	0.48099	0.49927	0.51952	0.54258
71	0.42958	0.46337	0.47984	0.48534	0.50349	0.52359	0.54647
72	0.43418	0.46783	0.48407	0.48962	0.50762	0.52758	0.55030
73	0.43856	0.47213	0.48833	0.49375	0.51168	0.53151	0.55402
74	0.44299	0.47632	0.49244	0.49785	0.51566	0.53534	0.55771
75	0.44730	0.48053	0.49645	0.50181	0.51958	0.53911	0.56131

N	0.1%	0.5%	1%	1.25%	2.5%	5%	10%
76	0.45148	0.48460	0.50042	0.50576	0.52337	0.54280	0.56484
77	0.45562	0.48866	0.50433	0.50963	0.52714	0.54642	0.56829
78	0.45967	0.49262	0.50818	0.51345	0.53084	0.54998	0.57172
79	0.46386	0.49639	0.51200	0.51723	0.53447	0.55347	0.57507
80	0.46776	0.50020	0.51571	0.52090	0.53797	0.55692	0.57836
81	0.47164	0.50386	0.51930	0.52452	0.54148	0.56029	0.58156
82	0.47557	0.50754	0.52289	0.52802	0.54493	0.56360	0.58475
83	0.47924	0.51115	0.52642	0.53155	0.54832	0.56688	0.58786
84	0.48296	0.51474	0.52989	0.53499	0.55165	0.57007	0.59091
85	0.48668	0.51828	0.53331	0.53834	0.55491	0.57324	0.59393
86	0.49043	0.52165	0.53664	0.54167	0.55813	0.57637	0.59689
87	0.49386	0.52503	0.53992	0.54495	0.56132	0.57940	0.59978
88	0.49745	0.52834	0.54315	0.54812	0.56445	0.58239	0.60264
89	0.50084	0.53163	0.54635	0.55129	0.56747	0.58531	0.60544
90	0.50421	0.53486	0.54943	0.55436	0.57048	0.58821	0.60819
91	0.50755	0.53802	0.55258	0.55746	0.57345	0.59108	0.61093
92	0.51085	0.54123	0.55561	0.56045	0.57635	0.59388	0.61359
93	0.51418	0.54428	0.55862	0.56345	0.57926	0.59662	0.61622
94	0.51727	0.54731	0.56158	0.56642	0.58210	0.59935	0.61879
95	0.52032	0.55028	0.56450	0.56926	0.58488	0.60205	0.62134
96	0.52335	0.55328	0.56731	0.57211	0.58762	0.60468	0.62385
97	0.52647	0.55616	0.57014	0.57487	0.59031	0.60727	0.62633
98	0.52941	0.55905	0.57295	0.57762	0.59297	0.60985	0.62877
99	0.53241	0.56185	0.57570	0.58033	0.59562	0.61236	0.63115
100	0.53540	0.56460	0.57840	0.58302	0.59819	0.61486	0.63352
101	0.53831	0.56741	0.58108	0.58568	0.60074	0.61730	0.63585
102	0.54118	0.57013	0.58372	0.58827	0.60327	0.61971	0.63814
103	0.54400	0.57275	0.58631	0.59083	0.60575	0.62210	0.64041
104	0.54687	0.57538	0.58884	0.59336	0.60819	0.62443	0.64264
105	0.54947	0.57801	0.59136	0.59582	0.61060	0.62675	0.64483
106	0.55229	0.58062	0.59383	0.59826	0.61300	0.62901	0.64701
107	0.55499	0.58307	0.59630	0.60071	0.61533	0.63128	0.64915
108	0.55760	0.58555	0.59874	0.60313	0.61765	0.63349	0.65124
109	0.56011	0.58800	0.60114	0.60549	0.61990	0.63568	0.65334
110	0.56259	0.59041	0.60349	0.60783	0.62215	0.63785	0.65538
111	0.56520	0.59281	0.60578	0.61011	0.62434	0.63995	0.65739
112	0.56767	0.59515	0.60801	0.61233	0.62651	0.64205	0.65938
113	0.57024	0.59746	0.61027	0.61461	0.62870	0.64414	0.66135
114	0.57258	0.59982	0.61248	0.61680	0.63083	0.64617	0.66330
115	0.57505	0.60205	0.61470	0.61896	0.63292	0.64818	0.66519
116	0.57749	0.60425	0.61687	0.62109	0.63501	0.65018	0.66708
117	0.57982	0.60642	0.61901	0.62327	0.63704	0.65212	0.66895
118	0.58204	0.60860	0.62113	0.62532	0.63907	0.65405	0.67081
119	0.58434	0.61074	0.62320	0.62739	0.64107	0.65596	0.67263
120	0.58669	0.61288	0.62527	0.62944	0.64304	0.65785	0.67441
121	0.58889	0.61500	0.62734	0.63148	0.64500	0.65971	0.67618
122	0.59109	0.61710	0.62933	0.63343	0.64690	0.66153	0.67794
123	0.59327	0.61921	0.63132	0.63542	0.64877	0.66334	0.67967
124	0.59534	0.62123	0.63328	0.63737	0.65066	0.66514	0.68138
125	0.59746	0.62325	0.63524	0.63929	0.65250	0.66692	0.68307
126	0.59957	0.62519	0.63716	0.64118	0.65432	0.66870	0.68474
127	0.60168	0.62711	0.63908	0.64307	0.65616	0.67044	0.68639
128	0.60371	0.62903	0.64094	0.64492	0.65794	0.67213	0.68800
129	0.60576	0.63097	0.64276	0.64676	0.65971	0.67383	0.68962
130	0.60782	0.63287	0.64461	0.64853	0.66144	0.67549	0.69119
131	0.60981	0.63472	0.64642	0.65036	0.66318	0.67716	0.69277
132	0.61189	0.63659	0.64819	0.65213	0.66488	0.67880	0.69432
133	0.61379	0.63839	0.64999	0.65390	0.66656	0.68043	0.69585
134	0.61573	0.64022	0.65176	0.65560	0.66824	0.68201	0.69738
135	0.61757	0.64201	0.65346	0.65732	0.66989	0.68359	0.69888
136	0.61942	0.64379	0.65515	0.65900	0.67152	0.68516	0.70036
137	0.62135	0.64558	0.65685	0.66065	0.67315	0.68668	0.70183
138	0.62317	0.64727	0.65852	0.66232	0.67473	0.68822	0.70327
139	0.62505	0.64898	0.66021	0.66400	0.67630	0.68974	0.70469
140	0.62682	0.65070	0.66184	0.66563	0.67786	0.69124	0.70612
141	0.62862	0.65232	0.66345	0.66722	0.67942	0.69270	0.70752
142	0.63046	0.65399	0.66506	0.66882	0.68094	0.69417	0.70891
143	0.63224	0.65563	0.66667	0.67038	0.68245	0.69562	0.71028
144	0.63384	0.65724	0.66825	0.67195	0.68393	0.69705	0.71163
145	0.63561	0.65886	0.66980	0.67348	0.68541	0.69847	0.71298
146	0.63732	0.66044	0.67135	0.67500	0.68689	0.69985	0.71431
147	0.63901	0.66203	0.67287	0.67649	0.68833	0.70124	0.71562
148	0.64066	0.66357	0.67435	0.67797	0.68973	0.70261	0.71691
149	0.64234	0.66508	0.67586	0.67943	0.69116	0.70395	0.71820

N	0.1%	0.5%	1%	1.25%	2.5%	5%	10%
150	0.64399	0.66663	0.67733	0.68092	0.69257	0.70529	0.71947
151	0.64561	0.66813	0.67877	0.68235	0.69396	0.70663	0.72073
152	0.64724	0.66962	0.68023	0.68378	0.69532	0.70794	0.72198
153	0.64880	0.67112	0.68168	0.68520	0.69668	0.70924	0.72321
154	0.65041	0.67256	0.68308	0.68658	0.69804	0.71054	0.72443
155	0.65196	0.67401	0.68448	0.68798	0.69940	0.71182	0.72564
156	0.65353	0.67545	0.68589	0.68937	0.70071	0.71307	0.72684
157	0.65504	0.67688	0.68724	0.69072	0.70201	0.71432	0.72803
158	0.65651	0.67828	0.68860	0.69205	0.70331	0.71556	0.72919
159	0.65801	0.67964	0.68994	0.69336	0.70458	0.71678	0.73036
160	0.65948	0.68105	0.69127	0.69472	0.70584	0.71800	0.73151
161	0.66092	0.68241	0.69258	0.69601	0.70710	0.71920	0.73264
162	0.66231	0.68375	0.69388	0.69731	0.70837	0.72039	0.73377
163	0.66377	0.68506	0.69518	0.69858	0.70960	0.72157	0.73489
164	0.66515	0.68641	0.69647	0.69987	0.71082	0.72274	0.73598
165	0.66657	0.68774	0.69774	0.70110	0.71203	0.72390	0.73709
166	0.66797	0.68906	0.69901	0.70234	0.71325	0.72506	0.73818
167	0.66931	0.69031	0.70025	0.70360	0.71442	0.72618	0.73926
168	0.67066	0.69158	0.70146	0.70482	0.71559	0.72731	0.74034
169	0.67204	0.69288	0.70269	0.70601	0.71675	0.72843	0.74140
170	0.67338	0.69417	0.70393	0.70722	0.71791	0.72952	0.74245
171	0.67469	0.69540	0.70511	0.70839	0.71903	0.73062	0.74348
172	0.67599	0.69664	0.70632	0.70960	0.72018	0.73171	0.74450
173	0.67729	0.69789	0.70750	0.71076	0.72131	0.73279	0.74551
174	0.67870	0.69909	0.70867	0.71192	0.72243	0.73384	0.74652
175	0.67996	0.70029	0.70980	0.71306	0.72351	0.73490	0.74752
176	0.68131	0.70146	0.71097	0.71419	0.72460	0.73593	0.74852
177	0.68249	0.70265	0.71208	0.71529	0.72568	0.73696	0.74950
178	0.68373	0.70381	0.71321	0.71641	0.72677	0.73799	0.75047
179	0.68496	0.70497	0.71434	0.71754	0.72782	0.73901	0.75143
180	0.68619	0.70614	0.71547	0.71864	0.72889	0.74002	0.75239
181	0.68747	0.70728	0.71656	0.71972	0.72995	0.74102	0.75333
182	0.68861	0.70840	0.71767	0.72079	0.73098	0.74200	0.75427
183	0.68978	0.70952	0.71874	0.72187	0.73200	0.74300	0.75520
184	0.69093	0.71065	0.71980	0.72295	0.73303	0.74398	0.75613
185	0.69206	0.71173	0.72086	0.72397	0.73405	0.74494	0.75704
186	0.69315	0.71281	0.72194	0.72501	0.73504	0.74589	0.75795
187	0.69433	0.71392	0.72298	0.72604	0.73604	0.74684	0.75885
188	0.69541	0.71497	0.72402	0.72706	0.73702	0.74779	0.75974
189	0.69657	0.71604	0.72505	0.72808	0.73801	0.74873	0.76061
190	0.69770	0.71706	0.72605	0.72909	0.73897	0.74965	0.76150
191	0.69881	0.71812	0.72706	0.73010	0.73993	0.75056	0.76237
192	0.70001	0.71916	0.72808	0.73107	0.74088	0.75146	0.76323
193	0.70105	0.72019	0.72909	0.73207	0.74182	0.75236	0.76409
194	0.70212	0.72119	0.73009	0.73304	0.74275	0.75326	0.76493
195	0.70323	0.72219	0.73106	0.73401	0.74368	0.75414	0.76578
196	0.70428	0.72322	0.73202	0.73496	0.74460	0.75501	0.76661
197	0.70534	0.72421	0.73298	0.73591	0.74551	0.75588	0.76744
198	0.70641	0.72519	0.73394	0.73685	0.74642	0.75675	0.76825
199	0.70742	0.72616	0.73489	0.73779	0.74731	0.75762	0.76907
200	0.70840	0.72715	0.73582	0.73872	0.74821	0.75847	0.76988
201	0.70942	0.72809	0.73675	0.73965	0.74908	0.75932	0.77068
202	0.71051	0.72904	0.73768	0.74057	0.74997	0.76016	0.77147
203	0.71155	0.72998	0.73859	0.74146	0.75084	0.76100	0.77226
204	0.71257	0.73095	0.73950	0.74235	0.75170	0.76182	0.77304
205	0.71348	0.73188	0.74042	0.74323	0.75256	0.76264	0.77382
206	0.71454	0.73278	0.74130	0.74413	0.75341	0.76346	0.77459
207	0.71557	0.73370	0.74219	0.74501	0.75426	0.76426	0.77535
208	0.71657	0.73462	0.74307	0.74586	0.75509	0.76507	0.77611
209	0.71756	0.73552	0.74393	0.74675	0.75592	0.76588	0.77687
210	0.71853	0.73640	0.74480	0.74761	0.75675	0.76666	0.77762
211	0.71952	0.73733	0.74568	0.74846	0.75757	0.76744	0.77836
212	0.72039	0.73823	0.74653	0.74931	0.75838	0.76822	0.77909
213	0.72137	0.73909	0.74740	0.75013	0.75916	0.76898	0.77982
214	0.72227	0.73996	0.74825	0.75098	0.75996	0.76975	0.78054
215	0.72321	0.74082	0.74908	0.75181	0.76077	0.77051	0.78126
216	0.72408	0.74167	0.74992	0.75264	0.76155	0.77126	0.78198
217	0.72499	0.74253	0.75074	0.75344	0.76235	0.77201	0.78269
218	0.72592	0.74338	0.75155	0.75425	0.76313	0.77275	0.78340
219	0.72691	0.74422	0.75236	0.75506	0.76390	0.77349	0.78409
220	0.72781	0.74503	0.75315	0.75586	0.76468	0.77422	0.78479
221	0.72864	0.74586	0.75393	0.75664	0.76543	0.77495	0.78548
222	0.72960	0.74670	0.75473	0.75742	0.76617	0.77568	0.78616
223	0.73044	0.74752	0.75552	0.75821	0.76694	0.77639	0.78684

N	0.1%	0.5%	1%	1.25%	2.5%	5%	10%
224	0.73124	0.74832	0.75632	0.75898	0.76768	0.77710	0.78750
225	0.73213	0.74909	0.75709	0.75976	0.76841	0.77780	0.78818
226	0.73297	0.74989	0.75785	0.76050	0.76916	0.77851	0.78884
227	0.73378	0.75068	0.75858	0.76125	0.76989	0.77920	0.78949
228	0.73460	0.75148	0.75935	0.76201	0.77060	0.77989	0.79015
229	0.73546	0.75226	0.76010	0.76276	0.77132	0.78058	0.79080
230	0.73627	0.75306	0.76087	0.76351	0.77204	0.78126	0.79145
231	0.73712	0.75384	0.76163	0.76423	0.77274	0.78193	0.79209
232	0.73792	0.75463	0.76237	0.76497	0.77345	0.78260	0.79274
233	0.73870	0.75536	0.76311	0.76570	0.77415	0.78328	0.79337
234	0.73954	0.75612	0.76384	0.76643	0.77484	0.78394	0.79399
235	0.74033	0.75688	0.76457	0.76713	0.77553	0.78459	0.79462
236	0.74109	0.75764	0.76529	0.76784	0.77621	0.78524	0.79523
237	0.74191	0.75840	0.76601	0.76855	0.77689	0.78590	0.79585
238	0.74268	0.75912	0.76670	0.76927	0.77757	0.78654	0.79646
239	0.74344	0.75985	0.76743	0.76996	0.77824	0.78718	0.79707
240	0.74420	0.76055	0.76814	0.77066	0.77890	0.78782	0.79767
241	0.74499	0.76126	0.76884	0.77135	0.77956	0.78845	0.79827
242	0.74576	0.76199	0.76951	0.77203	0.78020	0.78908	0.79887
243	0.74653	0.76270	0.77020	0.77271	0.78086	0.78970	0.79946
244	0.74731	0.76341	0.77089	0.77339	0.78152	0.79032	0.80004
245	0.74802	0.76411	0.77156	0.77406	0.78217	0.79094	0.80062
246	0.74878	0.76481	0.77223	0.77472	0.78279	0.79155	0.80120
247	0.74956	0.76550	0.77289	0.77538	0.78343	0.79215	0.80177
248	0.75031	0.76618	0.77356	0.77603	0.78407	0.79276	0.80235
249	0.75106	0.76685	0.77422	0.77670	0.78467	0.79335	0.80292
250	0.75179	0.76751	0.77489	0.77735	0.78530	0.79394	0.80348
251	0.75251	0.76817	0.77552	0.77798	0.78592	0.79454	0.80405
252	0.75325	0.76886	0.77616	0.77860	0.78653	0.79512	0.80460
253	0.75395	0.76952	0.77682	0.77925	0.78715	0.79571	0.80516
254	0.75461	0.77018	0.77747	0.77988	0.78775	0.79629	0.80571
255	0.75538	0.77085	0.77809	0.78052	0.78836	0.79687	0.80625
256	0.75606	0.77151	0.77874	0.78116	0.78897	0.79744	0.80680
257	0.75674	0.77215	0.77935	0.78177	0.78956	0.79801	0.80733
258	0.75746	0.77278	0.77998	0.78239	0.79016	0.79857	0.80787
259	0.75813	0.77344	0.78059	0.78298	0.79075	0.79913	0.80840
260	0.75882	0.77406	0.78121	0.78359	0.79132	0.79969	0.80893
261	0.75951	0.77469	0.78181	0.78418	0.79191	0.80025	0.80946
262	0.76015	0.77531	0.78242	0.78479	0.79249	0.80080	0.80998
263	0.76079	0.77595	0.78302	0.78538	0.79307	0.80134	0.81050
264	0.76151	0.77657	0.78362	0.78598	0.79364	0.80189	0.81102
265	0.76212	0.77719	0.78420	0.78655	0.79419	0.80242	0.81153
266	0.76279	0.77781	0.78480	0.78714	0.79475	0.80296	0.81204
267	0.76339	0.77843	0.78539	0.78772	0.79530	0.80350	0.81255
268	0.76406	0.77904	0.78598	0.78830	0.79586	0.80403	0.81305
269	0.76472	0.77964	0.78656	0.78887	0.79641	0.80456	0.81355
270	0.76540	0.78025	0.78714	0.78944	0.79696	0.80509	0.81404
271	0.76603	0.78085	0.78770	0.79001	0.79751	0.80561	0.81453
272	0.76670	0.78144	0.78827	0.79057	0.79806	0.80613	0.81503
273	0.76734	0.78201	0.78884	0.79113	0.79860	0.80665	0.81551
274	0.76801	0.78259	0.78941	0.79168	0.79914	0.80717	0.81600
275	0.76860	0.78318	0.78996	0.79224	0.79967	0.80767	0.81649
276	0.76922	0.78378	0.79053	0.79280	0.80019	0.80818	0.81696
277	0.76982	0.78437	0.79108	0.79336	0.80071	0.80868	0.81744
278	0.77044	0.78493	0.79163	0.79389	0.80124	0.80918	0.81792
279	0.77107	0.78550	0.79218	0.79444	0.80176	0.80967	0.81839
280	0.77171	0.78608	0.79273	0.79497	0.80227	0.81016	0.81886
281	0.77230	0.78664	0.79327	0.79550	0.80278	0.81066	0.81932
282	0.77289	0.78720	0.79382	0.79604	0.80329	0.81115	0.81978
283	0.77345	0.78776	0.79435	0.79656	0.80380	0.81163	0.82025
284	0.77403	0.78830	0.79488	0.79710	0.80430	0.81211	0.82070
285	0.77463	0.78885	0.79542	0.79761	0.80480	0.81260	0.82115
286	0.77521	0.78941	0.79594	0.79814	0.80531	0.81308	0.82160
287	0.77581	0.78994	0.79646	0.79866	0.80581	0.81355	0.82206
288	0.77639	0.79048	0.79700	0.79917	0.80631	0.81402	0.82251
289	0.77701	0.79100	0.79751	0.79969	0.80680	0.81449	0.82295
290	0.77755	0.79154	0.79803	0.80020	0.80729	0.81495	0.82339
291	0.77811	0.79210	0.79854	0.80070	0.80778	0.81541	0.82383
292	0.77868	0.79263	0.79904	0.80121	0.80826	0.81587	0.82427
293	0.77922	0.79315	0.79955	0.80170	0.80874	0.81632	0.82471
294	0.77976	0.79367	0.80004	0.80220	0.80923	0.81678	0.82514
295	0.78032	0.79416	0.80055	0.80269	0.80970	0.81724	0.82557
296	0.78089	0.79468	0.80104	0.80319	0.81019	0.81769	0.82600
297	0.78145	0.79519	0.80156	0.80367	0.81065	0.81814	0.82643

N	0.1%	0.5%	1%	1.25%	2.5%	5%	10%
298	0.78200	0.79571	0.80204	0.80415	0.81110	0.81859	0.82685
299	0.78260	0.79620	0.80252	0.80464	0.81157	0.81904	0.82727
300	0.78308	0.79670	0.80301	0.80512	0.81203	0.81947	0.82769
301	0.78362	0.79721	0.80349	0.80559	0.81249	0.81991	0.82810
302	0.78414	0.79774	0.80398	0.80608	0.81294	0.82035	0.82852
303	0.78467	0.79822	0.80444	0.80655	0.81339	0.82078	0.82893
304	0.78519	0.79870	0.80493	0.80702	0.81385	0.82121	0.82934
305	0.78574	0.79918	0.80540	0.80748	0.81430	0.82164	0.82975
306	0.78623	0.79968	0.80588	0.80795	0.81475	0.82206	0.83015
307	0.78677	0.80015	0.80635	0.80841	0.81519	0.82249	0.83056
308	0.78727	0.80062	0.80681	0.80887	0.81562	0.82290	0.83096
309	0.78778	0.80109	0.80728	0.80933	0.81606	0.82333	0.83136
310	0.78832	0.80158	0.80773	0.80979	0.81649	0.82375	0.83175
311	0.78882	0.80205	0.80819	0.81025	0.81694	0.82417	0.83215
312	0.78932	0.80253	0.80865	0.81070	0.81737	0.82458	0.83254
313	0.78983	0.80299	0.80910	0.81115	0.81780	0.82500	0.83293
314	0.79035	0.80347	0.80954	0.81159	0.81824	0.82541	0.83332
315	0.79083	0.80394	0.80999	0.81204	0.81867	0.82582	0.83371
316	0.79131	0.80440	0.81043	0.81247	0.81909	0.82622	0.83410
317	0.79181	0.80485	0.81089	0.81291	0.81951	0.82662	0.83448
318	0.79228	0.80531	0.81133	0.81333	0.81992	0.82702	0.83486
319	0.79276	0.80577	0.81176	0.81376	0.82034	0.82742	0.83524
320	0.79324	0.80621	0.81221	0.81420	0.82076	0.82783	0.83561
321	0.79376	0.80668	0.81263	0.81463	0.82118	0.82822	0.83599
322	0.79425	0.80712	0.81306	0.81506	0.82159	0.82861	0.83636
323	0.79471	0.80756	0.81350	0.81548	0.82200	0.82900	0.83673
324	0.79522	0.80800	0.81393	0.81592	0.82240	0.82939	0.83710
325	0.79571	0.80844	0.81436	0.81633	0.82281	0.82978	0.83747
326	0.79615	0.80888	0.81478	0.81674	0.82321	0.83016	0.83784
327	0.79663	0.80932	0.81519	0.81717	0.82361	0.83055	0.83820
328	0.79711	0.80976	0.81561	0.81759	0.82401	0.83093	0.83856
329	0.79759	0.81019	0.81603	0.81800	0.82440	0.83131	0.83892
330	0.79802	0.81061	0.81644	0.81842	0.82481	0.83168	0.83928
331	0.79851	0.81102	0.81685	0.81882	0.82520	0.83205	0.83963
332	0.79899	0.81144	0.81726	0.81923	0.82559	0.83243	0.83998
333	0.79943	0.81187	0.81768	0.81963	0.82597	0.83280	0.84034
334	0.79987	0.81229	0.81808	0.82004	0.82636	0.83317	0.84069
335	0.80032	0.81270	0.81848	0.82044	0.82675	0.83354	0.84104
336	0.80076	0.81312	0.81889	0.82083	0.82713	0.83390	0.84138
337	0.80119	0.81354	0.81930	0.82123	0.82750	0.83427	0.84173
338	0.80164	0.81395	0.81970	0.82163	0.82789	0.83463	0.84207
339	0.80208	0.81437	0.82009	0.82203	0.82826	0.83499	0.84241
340	0.80250	0.81478	0.82049	0.82241	0.82863	0.83535	0.84276
341	0.80295	0.81519	0.82089	0.82279	0.82901	0.83570	0.84309
342	0.80336	0.81560	0.82128	0.82320	0.82938	0.83606	0.84343
343	0.80377	0.81601	0.82166	0.82357	0.82975	0.83641	0.84377
344	0.80420	0.81641	0.82206	0.82396	0.83012	0.83676	0.84410
345	0.80466	0.81680	0.82244	0.82434	0.83048	0.83711	0.84443
346	0.80506	0.81720	0.82283	0.82472	0.83085	0.83746	0.84476
347	0.80550	0.81760	0.82321	0.82511	0.83122	0.83781	0.84509
348	0.80592	0.81801	0.82359	0.82549	0.83157	0.83815	0.84542
349	0.80634	0.81841	0.82397	0.82585	0.83193	0.83850	0.84574
350	0.80677	0.81881	0.82435	0.82624	0.83230	0.83884	0.84607
351	0.80717	0.81921	0.82474	0.82661	0.83266	0.83918	0.84639
352	0.80757	0.81958	0.82511	0.82697	0.83301	0.83951	0.84672
353	0.80799	0.81997	0.82548	0.82734	0.83337	0.83986	0.84704
354	0.80842	0.82035	0.82585	0.82771	0.83373	0.84019	0.84735
355	0.80883	0.82074	0.82623	0.82808	0.83407	0.84052	0.84767
356	0.80926	0.82110	0.82659	0.82844	0.83442	0.84085	0.84798
357	0.80969	0.82149	0.82695	0.82880	0.83476	0.84119	0.84830
358	0.81007	0.82184	0.82731	0.82916	0.83511	0.84152	0.84861
359	0.81050	0.82222	0.82767	0.82952	0.83545	0.84184	0.84893
360	0.81090	0.82259	0.82803	0.82986	0.83580	0.84217	0.84924
361	0.81132	0.82296	0.82839	0.83022	0.83614	0.84250	0.84955
362	0.81168	0.82333	0.82876	0.83058	0.83647	0.84282	0.84985
363	0.81207	0.82368	0.82910	0.83092	0.83681	0.84315	0.85016
364	0.81247	0.82404	0.82946	0.83126	0.83715	0.84346	0.85046
365	0.81285	0.82440	0.82981	0.83160	0.83747	0.84378	0.85076
366	0.81323	0.82476	0.83017	0.83195	0.83781	0.84410	0.85106
367	0.81362	0.82512	0.83051	0.83229	0.83814	0.84442	0.85137
368	0.81403	0.82548	0.83086	0.83264	0.83847	0.84473	0.85166
369	0.81437	0.82584	0.83120	0.83299	0.83879	0.84504	0.85196
370	0.81474	0.82618	0.83154	0.83333	0.83912	0.84535	0.85226
371	0.81514	0.82653	0.83189	0.83367	0.83944	0.84567	0.85255

N	0.1%	0.5%	1%	1.25%	2.5%	5%	10%
372	0.81551	0.82690	0.83223	0.83400	0.83977	0.84598	0.85284
373	0.81585	0.82724	0.83257	0.83433	0.84008	0.84629	0.85314
374	0.81623	0.82761	0.83292	0.83468	0.84040	0.84659	0.85343
375	0.81661	0.82796	0.83326	0.83502	0.84072	0.84690	0.85372
376	0.81698	0.82830	0.83358	0.83535	0.84104	0.84721	0.85401
377	0.81737	0.82864	0.83392	0.83568	0.84135	0.84751	0.85430
378	0.81775	0.82900	0.83424	0.83600	0.84167	0.84781	0.85459
379	0.81811	0.82933	0.83457	0.83634	0.84198	0.84811	0.85487
380	0.81846	0.82967	0.83491	0.83665	0.84230	0.84841	0.85515
381	0.81879	0.83001	0.83523	0.83698	0.84261	0.84871	0.85543
382	0.81919	0.83035	0.83555	0.83730	0.84291	0.84900	0.85571
383	0.81952	0.83069	0.83588	0.83762	0.84321	0.84930	0.85599
384	0.81986	0.83102	0.83620	0.83793	0.84353	0.84959	0.85627
385	0.82020	0.83135	0.83652	0.83825	0.84383	0.84988	0.85655
386	0.82058	0.83168	0.83685	0.83856	0.84414	0.85017	0.85682
387	0.82091	0.83201	0.83717	0.83888	0.84444	0.85046	0.85709
388	0.82126	0.83234	0.83748	0.83919	0.84474	0.85075	0.85737
389	0.82159	0.83267	0.83779	0.83950	0.84505	0.85104	0.85764
390	0.82194	0.83300	0.83810	0.83981	0.84535	0.85132	0.85791
391	0.82230	0.83331	0.83842	0.84012	0.84565	0.85161	0.85818
392	0.82266	0.83364	0.83873	0.84043	0.84594	0.85189	0.85845
393	0.82300	0.83396	0.83904	0.84074	0.84624	0.85217	0.85872
394	0.82335	0.83429	0.83935	0.84103	0.84653	0.85245	0.85898
395	0.82368	0.83461	0.83966	0.84134	0.84682	0.85273	0.85925
396	0.82403	0.83493	0.83997	0.84164	0.84711	0.85301	0.85951
397	0.82435	0.83524	0.84028	0.84194	0.84740	0.85328	0.85978
398	0.82467	0.83555	0.84058	0.84224	0.84769	0.85356	0.86004
399	0.82499	0.83588	0.84088	0.84253	0.84797	0.85384	0.86031
400	0.82533	0.83617	0.84118	0.84283	0.84826	0.85411	0.86056
401	0.82569	0.83648	0.84148	0.84314	0.84855	0.85438	0.86082
402	0.82600	0.83680	0.84179	0.84343	0.84884	0.85466	0.86108
403	0.82633	0.83711	0.84209	0.84373	0.84912	0.85493	0.86134
404	0.82668	0.83742	0.84239	0.84402	0.84940	0.85520	0.86159
405	0.82701	0.83772	0.84268	0.84432	0.84968	0.85547	0.86185
406	0.82733	0.83802	0.84298	0.84461	0.84996	0.85573	0.86210
407	0.82767	0.83832	0.84327	0.84490	0.85024	0.85600	0.86235
408	0.82802	0.83863	0.84356	0.84519	0.85052	0.85627	0.86261
409	0.82834	0.83892	0.84385	0.84547	0.85079	0.85653	0.86286
410	0.82864	0.83922	0.84414	0.84576	0.85107	0.85679	0.86311
411	0.82895	0.83952	0.84442	0.84605	0.85134	0.85705	0.86336
412	0.82928	0.83981	0.84472	0.84633	0.85161	0.85731	0.86360
413	0.82960	0.84010	0.84500	0.84662	0.85189	0.85757	0.86385
414	0.82990	0.84041	0.84529	0.84690	0.85215	0.85783	0.86409
415	0.83022	0.84070	0.84557	0.84718	0.85243	0.85809	0.86434
416	0.83051	0.84099	0.84584	0.84745	0.85270	0.85834	0.86458
417	0.83083	0.84128	0.84612	0.84773	0.85297	0.85859	0.86482
418	0.83112	0.84157	0.84641	0.84802	0.85323	0.85885	0.86507
419	0.83142	0.84186	0.84668	0.84829	0.85349	0.85910	0.86531
420	0.83175	0.84215	0.84696	0.84856	0.85375	0.85936	0.86555
421	0.83206	0.84244	0.84724	0.84883	0.85401	0.85961	0.86579
422	0.83235	0.84272	0.84752	0.84910	0.85428	0.85986	0.86602
423	0.83265	0.84300	0.84779	0.84938	0.85453	0.86011	0.86626
424	0.83295	0.84328	0.84806	0.84964	0.85478	0.86035	0.86650
425	0.83327	0.84356	0.84834	0.84991	0.85505	0.86060	0.86673
426	0.83359	0.84386	0.84861	0.85019	0.85530	0.86085	0.86697
427	0.83388	0.84413	0.84888	0.85045	0.85556	0.86109	0.86720
428	0.83417	0.84442	0.84915	0.85071	0.85582	0.86134	0.86743
429	0.83448	0.84470	0.84942	0.85098	0.85607	0.86158	0.86766
430	0.83476	0.84497	0.84968	0.85125	0.85632	0.86182	0.86789
431	0.83507	0.84524	0.84995	0.85151	0.85658	0.86207	0.86812
432	0.83540	0.84552	0.85021	0.85177	0.85683	0.86231	0.86835
433	0.83569	0.84580	0.85048	0.85203	0.85708	0.86255	0.86858
434	0.83596	0.84607	0.85074	0.85229	0.85733	0.86279	0.86881
435	0.83624	0.84635	0.85100	0.85255	0.85758	0.86302	0.86903
436	0.83654	0.84660	0.85126	0.85280	0.85782	0.86326	0.86926
437	0.83683	0.84687	0.85151	0.85306	0.85807	0.86349	0.86948
438	0.83713	0.84714	0.85177	0.85331	0.85831	0.86373	0.86971
439	0.83739	0.84739	0.85203	0.85356	0.85856	0.86396	0.86993
440	0.83768	0.84765	0.85228	0.85382	0.85880	0.86419	0.87015
441	0.83796	0.84791	0.85254	0.85407	0.85905	0.86442	0.87037
442	0.83825	0.84819	0.85279	0.85432	0.85928	0.86465	0.87059
443	0.83854	0.84845	0.85305	0.85457	0.85953	0.86488	0.87081
444	0.83882	0.84871	0.85330	0.85483	0.85977	0.86512	0.87103
445	0.83911	0.84898	0.85355	0.85507	0.86001	0.86534	0.87124

N	0.1%	0.5%	1%	1.25%	2.5%	5%	10%
446	0.83937	0.84924	0.85379	0.85532	0.86025	0.86557	0.87146
447	0.83965	0.84949	0.85405	0.85557	0.86049	0.86580	0.87168
448	0.83993	0.84975	0.85429	0.85581	0.86072	0.86603	0.87189
449	0.84020	0.85000	0.85453	0.85605	0.86096	0.86625	0.87210
450	0.84048	0.85026	0.85478	0.85630	0.86119	0.86648	0.87232
451	0.84075	0.85051	0.85503	0.85654	0.86142	0.86671	0.87253
452	0.84103	0.85075	0.85526	0.85678	0.86166	0.86693	0.87274
453	0.84130	0.85101	0.85551	0.85702	0.86190	0.86715	0.87296
454	0.84157	0.85127	0.85576	0.85726	0.86212	0.86737	0.87316
455	0.84184	0.85151	0.85600	0.85751	0.86236	0.86759	0.87337
456	0.84210	0.85177	0.85624	0.85775	0.86259	0.86781	0.87358
457	0.84238	0.85202	0.85649	0.85799	0.86282	0.86802	0.87379
458	0.84263	0.85227	0.85673	0.85822	0.86305	0.86824	0.87399
459	0.84289	0.85251	0.85697	0.85845	0.86327	0.86846	0.87420
460	0.84315	0.85276	0.85722	0.85869	0.86350	0.86868	0.87441
461	0.84343	0.85301	0.85745	0.85893	0.86372	0.86890	0.87461
462	0.84373	0.85325	0.85769	0.85916	0.86395	0.86911	0.87482
463	0.84397	0.85350	0.85793	0.85939	0.86417	0.86932	0.87502
464	0.84421	0.85375	0.85816	0.85962	0.86440	0.86953	0.87523
465	0.84448	0.85401	0.85839	0.85985	0.86462	0.86975	0.87543
466	0.84472	0.85424	0.85862	0.86008	0.86484	0.86996	0.87563
467	0.84497	0.85447	0.85887	0.86032	0.86506	0.87017	0.87583
468	0.84523	0.85471	0.85910	0.86055	0.86528	0.87038	0.87603
469	0.84550	0.85495	0.85932	0.86078	0.86550	0.87059	0.87623
470	0.84575	0.85518	0.85956	0.86101	0.86572	0.87080	0.87643
471	0.84600	0.85542	0.85978	0.86124	0.86593	0.87101	0.87663
472	0.84625	0.85565	0.86000	0.86146	0.86615	0.87121	0.87682
473	0.84649	0.85588	0.86024	0.86168	0.86637	0.87142	0.87702
474	0.84674	0.85611	0.86046	0.86191	0.86658	0.87163	0.87721
475	0.84698	0.85635	0.86068	0.86213	0.86679	0.87183	0.87741
476	0.84723	0.85659	0.86091	0.86235	0.86700	0.87204	0.87760
477	0.84747	0.85682	0.86114	0.86258	0.86722	0.87224	0.87780
478	0.84773	0.85705	0.86136	0.86280	0.86743	0.87244	0.87799
479	0.84798	0.85728	0.86158	0.86302	0.86765	0.87265	0.87818
480	0.84823	0.85752	0.86181	0.86324	0.86785	0.87285	0.87837
481	0.84848	0.85774	0.86203	0.86346	0.86806	0.87305	0.87856
482	0.84872	0.85797	0.86225	0.86368	0.86827	0.87325	0.87875
483	0.84895	0.85820	0.86247	0.86389	0.86848	0.87345	0.87894
484	0.84918	0.85843	0.86268	0.86411	0.86869	0.87364	0.87913
485	0.84943	0.85866	0.86289	0.86432	0.86889	0.87384	0.87932
486	0.84968	0.85888	0.86311	0.86453	0.86910	0.87404	0.87951
487	0.84992	0.85910	0.86333	0.86474	0.86931	0.87424	0.87969
488	0.85019	0.85932	0.86353	0.86495	0.86952	0.87443	0.87988
489	0.85040	0.85954	0.86375	0.86516	0.86972	0.87463	0.88006
490	0.85063	0.85977	0.86397	0.86538	0.86992	0.87482	0.88025
491	0.85088	0.85998	0.86419	0.86558	0.87013	0.87502	0.88043
492	0.85113	0.86020	0.86440	0.86579	0.87033	0.87521	0.88062
493	0.85138	0.86042	0.86460	0.86599	0.87053	0.87540	0.88080
494	0.85158	0.86063	0.86481	0.86620	0.87073	0.87559	0.88098
495	0.85180	0.86085	0.86502	0.86641	0.87093	0.87578	0.88116
496	0.85204	0.86107	0.86522	0.86662	0.87113	0.87598	0.88134
497	0.85227	0.86129	0.86544	0.86682	0.87133	0.87616	0.88152
498	0.85253	0.86150	0.86565	0.86703	0.87153	0.87635	0.88170
499	0.85274	0.86172	0.86586	0.86723	0.87172	0.87654	0.88188
500	0.85297	0.86194	0.86607	0.86744	0.87191	0.87673	0.88206
501	0.85322	0.86216	0.86628	0.86765	0.87211	0.87692	0.88224
502	0.85343	0.86236	0.86647	0.86785	0.87230	0.87711	0.88241
503	0.85366	0.86258	0.86668	0.86804	0.87250	0.87729	0.88259
504	0.85389	0.86278	0.86689	0.86825	0.87270	0.87748	0.88277
505	0.85412	0.86299	0.86710	0.86845	0.87289	0.87766	0.88294
506	0.85434	0.86321	0.86730	0.86866	0.87309	0.87784	0.88311
507	0.85458	0.86342	0.86750	0.86886	0.87328	0.87803	0.88329
508	0.85480	0.86362	0.86769	0.86906	0.87347	0.87822	0.88346
509	0.85502	0.86384	0.86789	0.86926	0.87366	0.87840	0.88364
510	0.85524	0.86404	0.86809	0.86946	0.87385	0.87858	0.88381
511	0.85546	0.86423	0.86829	0.86965	0.87404	0.87876	0.88398
512	0.85569	0.86444	0.86849	0.86985	0.87423	0.87894	0.88416
513	0.85589	0.86465	0.86869	0.87004	0.87442	0.87912	0.88433
514	0.85613	0.86486	0.86889	0.87024	0.87461	0.87930	0.88450
515	0.85636	0.86507	0.86908	0.87044	0.87480	0.87948	0.88467
516	0.85657	0.86527	0.86928	0.87064	0.87498	0.87966	0.88484
517	0.85679	0.86547	0.86947	0.87083	0.87517	0.87984	0.88501
518	0.85699	0.86567	0.86967	0.87102	0.87535	0.88001	0.88518
519	0.85718	0.86587	0.86987	0.87121	0.87554	0.88019	0.88534

N	0.1%	0.5%	1%	1.25%	2.5%	5%	10%
520	0.85741	0.86607	0.87006	0.87140	0.87572	0.88037	0.88551
521	0.85762	0.86627	0.87025	0.87160	0.87591	0.88054	0.88568
522	0.85783	0.86647	0.87045	0.87179	0.87609	0.88072	0.88584
523	0.85806	0.86666	0.87064	0.87198	0.87627	0.88089	0.88601
524	0.85829	0.86687	0.87083	0.87216	0.87646	0.88106	0.88617
525	0.85849	0.86705	0.87101	0.87235	0.87664	0.88124	0.88634
526	0.85871	0.86724	0.87120	0.87253	0.87681	0.88141	0.88650
527	0.85889	0.86744	0.87139	0.87272	0.87699	0.88158	0.88667
528	0.85912	0.86763	0.87157	0.87291	0.87717	0.88175	0.88683
529	0.85931	0.86783	0.87176	0.87309	0.87735	0.88192	0.88699
530	0.85951	0.86803	0.87195	0.87328	0.87752	0.88210	0.88716
531	0.85973	0.86822	0.87214	0.87346	0.87771	0.88227	0.88732
532	0.85995	0.86842	0.87232	0.87364	0.87788	0.88243	0.88748
533	0.86017	0.86861	0.87251	0.87382	0.87806	0.88260	0.88764
534	0.86038	0.86880	0.87270	0.87401	0.87823	0.88277	0.88780
535	0.86056	0.86899	0.87287	0.87419	0.87841	0.88294	0.88796
536	0.86077	0.86917	0.87306	0.87437	0.87858	0.88311	0.88812
537	0.86099	0.86936	0.87325	0.87455	0.87876	0.88328	0.88828
538	0.86119	0.86955	0.87342	0.87473	0.87893	0.88344	0.88843
539	0.86140	0.86974	0.87361	0.87491	0.87911	0.88361	0.88859
540	0.86161	0.86992	0.87379	0.87509	0.87928	0.88378	0.88875
541	0.86179	0.87010	0.87397	0.87526	0.87945	0.88394	0.88890
542	0.86199	0.87029	0.87415	0.87544	0.87962	0.88411	0.88906
543	0.86219	0.87047	0.87433	0.87562	0.87979	0.88427	0.88921
544	0.86237	0.87066	0.87450	0.87580	0.87996	0.88443	0.88937
545	0.86259	0.87086	0.87469	0.87597	0.88013	0.88459	0.88952
546	0.86278	0.87104	0.87486	0.87615	0.88029	0.88475	0.88968
547	0.86296	0.87122	0.87504	0.87632	0.88046	0.88492	0.88983
548	0.86315	0.87140	0.87521	0.87650	0.88063	0.88508	0.88998
549	0.86334	0.87158	0.87538	0.87667	0.88080	0.88524	0.89014
550	0.86355	0.87176	0.87556	0.87685	0.88096	0.88540	0.89029
551	0.86371	0.87195	0.87573	0.87702	0.88112	0.88556	0.89044
552	0.86393	0.87214	0.87591	0.87719	0.88129	0.88572	0.89059
553	0.86413	0.87233	0.87609	0.87736	0.88146	0.88587	0.89074
554	0.86432	0.87251	0.87626	0.87753	0.88162	0.88603	0.89089
555	0.86451	0.87269	0.87643	0.87771	0.88178	0.88619	0.89104
556	0.86470	0.87287	0.87661	0.87788	0.88194	0.88635	0.89119
557	0.86491	0.87304	0.87678	0.87804	0.88211	0.88650	0.89134
558	0.86511	0.87322	0.87695	0.87822	0.88227	0.88666	0.89149
559	0.86530	0.87339	0.87712	0.87838	0.88243	0.88681	0.89164
560	0.86549	0.87357	0.87729	0.87856	0.88260	0.88697	0.89179
561	0.86567	0.87373	0.87746	0.87872	0.88276	0.88713	0.89193
562	0.86586	0.87391	0.87763	0.87889	0.88292	0.88728	0.89208
563	0.86605	0.87409	0.87780	0.87906	0.88308	0.88744	0.89223
564	0.86623	0.87427	0.87797	0.87922	0.88324	0.88759	0.89237
565	0.86643	0.87444	0.87814	0.87938	0.88340	0.88774	0.89252
566	0.86663	0.87462	0.87831	0.87955	0.88356	0.88789	0.89266
567	0.86682	0.87480	0.87848	0.87972	0.88372	0.88804	0.89281
568	0.86699	0.87498	0.87865	0.87988	0.88388	0.88819	0.89295
569	0.86717	0.87514	0.87881	0.88004	0.88403	0.88835	0.89309
570	0.86734	0.87531	0.87898	0.88021	0.88419	0.88849	0.89324
571	0.86750	0.87548	0.87914	0.88038	0.88435	0.88865	0.89338
572	0.86770	0.87566	0.87931	0.88054	0.88450	0.88880	0.89352
573	0.86788	0.87583	0.87947	0.88070	0.88466	0.88894	0.89366
574	0.86805	0.87599	0.87963	0.88086	0.88481	0.88909	0.89380
575	0.86823	0.87615	0.87979	0.88102	0.88497	0.88924	0.89394
576	0.86841	0.87632	0.87995	0.88118	0.88512	0.88939	0.89408
577	0.86860	0.87648	0.88011	0.88134	0.88528	0.88954	0.89422
578	0.86876	0.87665	0.88027	0.88150	0.88543	0.88968	0.89436
579	0.86894	0.87682	0.88044	0.88166	0.88558	0.88983	0.89450
580	0.86913	0.87698	0.88060	0.88181	0.88574	0.88998	0.89464
581	0.86932	0.87715	0.88076	0.88197	0.88589	0.89013	0.89478
582	0.86949	0.87730	0.88091	0.88212	0.88604	0.89027	0.89492
583	0.86968	0.87747	0.88107	0.88228	0.88619	0.89042	0.89506
584	0.86985	0.87764	0.88124	0.88244	0.88634	0.89056	0.89520
585	0.87002	0.87780	0.88140	0.88260	0.88649	0.89070	0.89533
586	0.87020	0.87796	0.88155	0.88275	0.88664	0.89085	0.89547
587	0.87039	0.87813	0.88172	0.88290	0.88679	0.89099	0.89560
588	0.87056	0.87829	0.88187	0.88306	0.88694	0.89113	0.89574
589	0.87074	0.87846	0.88203	0.88321	0.88709	0.89127	0.89587
590	0.87092	0.87862	0.88219	0.88337	0.88724	0.89142	0.89601
591	0.87109	0.87878	0.88234	0.88352	0.88739	0.89156	0.89614
592	0.87125	0.87894	0.88249	0.88368	0.88753	0.89170	0.89628
593	0.87141	0.87911	0.88265	0.88383	0.88768	0.89184	0.89641

N	0.1%	0.5%	1%	1.25%	2.5%	5%	10%
594	0.87160	0.87927	0.88280	0.88398	0.88783	0.89198	0.89654
595	0.87177	0.87942	0.88296	0.88414	0.88797	0.89212	0.89668
596	0.87195	0.87959	0.88311	0.88429	0.88812	0.89226	0.89681
597	0.87212	0.87974	0.88327	0.88444	0.88827	0.89240	0.89694
598	0.87228	0.87991	0.88342	0.88459	0.88841	0.89253	0.89707
599	0.87245	0.88006	0.88357	0.88474	0.88856	0.89267	0.89721
600	0.87260	0.88022	0.88372	0.88489	0.88870	0.89281	0.89734
601	0.87276	0.88038	0.88388	0.88504	0.88884	0.89295	0.89747
602	0.87293	0.88053	0.88403	0.88519	0.88899	0.89309	0.89760
603	0.87308	0.88068	0.88418	0.88534	0.88913	0.89322	0.89773
604	0.87324	0.88083	0.88433	0.88549	0.88927	0.89336	0.89786
605	0.87341	0.88098	0.88448	0.88564	0.88942	0.89350	0.89798
606	0.87355	0.88114	0.88463	0.88578	0.88955	0.89363	0.89811
607	0.87371	0.88129	0.88477	0.88593	0.88969	0.89377	0.89824
608	0.87387	0.88145	0.88492	0.88608	0.88983	0.89390	0.89837
609	0.87405	0.88159	0.88507	0.88623	0.88997	0.89404	0.89850
610	0.87420	0.88174	0.88522	0.88638	0.89011	0.89417	0.89863
611	0.87435	0.88190	0.88536	0.88652	0.89025	0.89431	0.89875
612	0.87453	0.88205	0.88551	0.88667	0.89039	0.89444	0.89888
613	0.87469	0.88220	0.88565	0.88681	0.89053	0.89457	0.89901
614	0.87486	0.88235	0.88580	0.88695	0.89067	0.89471	0.89913
615	0.87500	0.88250	0.88594	0.88709	0.89080	0.89484	0.89926
616	0.87518	0.88264	0.88609	0.88723	0.89094	0.89497	0.89939
617	0.87534	0.88280	0.88624	0.88737	0.89108	0.89510	0.89951
618	0.87551	0.88295	0.88638	0.88751	0.89122	0.89523	0.89964
619	0.87567	0.88310	0.88653	0.88766	0.89136	0.89536	0.89976
620	0.87582	0.88325	0.88667	0.88780	0.89149	0.89549	0.89989
621	0.87600	0.88340	0.88681	0.88795	0.89163	0.89562	0.90001
622	0.87616	0.88354	0.88696	0.88808	0.89177	0.89575	0.90013
623	0.87631	0.88369	0.88710	0.88822	0.89190	0.89588	0.90026
624	0.87646	0.88383	0.88723	0.88837	0.89204	0.89601	0.90038
625	0.87663	0.88397	0.88738	0.88851	0.89217	0.89614	0.90050
626	0.87677	0.88412	0.88752	0.88864	0.89231	0.89627	0.90062
627	0.87693	0.88427	0.88766	0.88879	0.89244	0.89640	0.90074
628	0.87710	0.88441	0.88780	0.88892	0.89257	0.89652	0.90086
629	0.87726	0.88455	0.88794	0.88906	0.89270	0.89665	0.90098
630	0.87741	0.88470	0.88807	0.88920	0.89284	0.89677	0.90111
631	0.87756	0.88484	0.88821	0.88933	0.89297	0.89690	0.90123
632	0.87772	0.88498	0.88835	0.88947	0.89310	0.89703	0.90135
633	0.87788	0.88512	0.88849	0.88961	0.89323	0.89715	0.90147
634	0.87801	0.88526	0.88862	0.88975	0.89337	0.89728	0.90158
635	0.87817	0.88541	0.88876	0.88988	0.89350	0.89740	0.90170
636	0.87831	0.88556	0.88889	0.89001	0.89363	0.89753	0.90182
637	0.87847	0.88570	0.88903	0.89015	0.89376	0.89765	0.90194
638	0.87861	0.88584	0.88916	0.89028	0.89389	0.89778	0.90206
639	0.87877	0.88597	0.88930	0.89042	0.89401	0.89790	0.90218
640	0.87892	0.88611	0.88944	0.89055	0.89414	0.89802	0.90229
641	0.87905	0.88625	0.88957	0.89069	0.89427	0.89815	0.90241
642	0.87919	0.88640	0.88971	0.89082	0.89440	0.89827	0.90253
643	0.87934	0.88654	0.88985	0.89096	0.89453	0.89839	0.90264
644	0.87950	0.88668	0.88998	0.89109	0.89465	0.89851	0.90276
645	0.87964	0.88681	0.89011	0.89121	0.89478	0.89864	0.90287
646	0.87977	0.88694	0.89024	0.89135	0.89491	0.89876	0.90299
647	0.87992	0.88708	0.89037	0.89148	0.89503	0.89888	0.90311
648	0.88007	0.88722	0.89050	0.89161	0.89516	0.89900	0.90322
649	0.88023	0.88736	0.89064	0.89174	0.89529	0.89912	0.90334
650	0.88036	0.88750	0.89078	0.89187	0.89542	0.89924	0.90345
651	0.88052	0.88763	0.89090	0.89201	0.89554	0.89936	0.90356
652	0.88067	0.88777	0.89103	0.89214	0.89567	0.89948	0.90368
653	0.88082	0.88790	0.89116	0.89226	0.89579	0.89959	0.90379
654	0.88097	0.88804	0.89129	0.89239	0.89592	0.89971	0.90391
655	0.88110	0.88817	0.89142	0.89252	0.89604	0.89983	0.90402
656	0.88126	0.88831	0.89155	0.89265	0.89616	0.89995	0.90413
657	0.88142	0.88844	0.89168	0.89277	0.89629	0.90007	0.90424
658	0.88155	0.88857	0.89181	0.89290	0.89641	0.90019	0.90435
659	0.88170	0.88871	0.89194	0.89303	0.89653	0.90031	0.90447
660	0.88184	0.88884	0.89207	0.89315	0.89665	0.90042	0.90458
661	0.88199	0.88897	0.89219	0.89328	0.89678	0.90054	0.90469
662	0.88213	0.88911	0.89232	0.89340	0.89690	0.90066	0.90480
663	0.88226	0.88924	0.89246	0.89353	0.89702	0.90077	0.90491
664	0.88241	0.88937	0.89258	0.89365	0.89714	0.90089	0.90502
665	0.88254	0.88951	0.89271	0.89378	0.89726	0.90100	0.90513
666	0.88267	0.88964	0.89283	0.89391	0.89738	0.90112	0.90524
667	0.88282	0.88977	0.89296	0.89404	0.89750	0.90123	0.90535

N	0.1%	0.5%	1%	1.25%	2.5%	5%	10%
668	0.88296	0.88989	0.89308	0.89416	0.89762	0.90135	0.90546
669	0.88308	0.89002	0.89321	0.89428	0.89774	0.90146	0.90557
670	0.88323	0.89016	0.89333	0.89440	0.89786	0.90157	0.90568
671	0.88335	0.89028	0.89346	0.89453	0.89798	0.90169	0.90578
672	0.88349	0.89042	0.89359	0.89465	0.89810	0.90180	0.90589
673	0.88363	0.89055	0.89371	0.89478	0.89821	0.90191	0.90600
674	0.88378	0.89067	0.89384	0.89490	0.89833	0.90203	0.90611
675	0.88393	0.89080	0.89396	0.89502	0.89845	0.90214	0.90621
676	0.88407	0.89092	0.89408	0.89514	0.89857	0.90225	0.90632
677	0.88421	0.89105	0.89421	0.89527	0.89868	0.90237	0.90643
678	0.88434	0.89118	0.89433	0.89539	0.89880	0.90248	0.90654
679	0.88446	0.89131	0.89446	0.89551	0.89892	0.90259	0.90664
680	0.88459	0.89142	0.89458	0.89563	0.89903	0.90270	0.90675
681	0.88473	0.89155	0.89470	0.89575	0.89915	0.90281	0.90686
682	0.88485	0.89167	0.89482	0.89587	0.89926	0.90292	0.90696
683	0.88500	0.89180	0.89494	0.89599	0.89938	0.90303	0.90707
684	0.88512	0.89192	0.89506	0.89611	0.89949	0.90314	0.90717
685	0.88527	0.89205	0.89518	0.89623	0.89961	0.90325	0.90727
686	0.88540	0.89216	0.89529	0.89635	0.89972	0.90336	0.90738
687	0.88553	0.89229	0.89542	0.89647	0.89984	0.90346	0.90748
688	0.88567	0.89242	0.89554	0.89658	0.89995	0.90357	0.90759
689	0.88580	0.89254	0.89565	0.89670	0.90006	0.90368	0.90769
690	0.88593	0.89266	0.89577	0.89682	0.90018	0.90379	0.90779
691	0.88605	0.89278	0.89589	0.89693	0.90029	0.90390	0.90790
692	0.88618	0.89291	0.89601	0.89705	0.90040	0.90401	0.90800
693	0.88632	0.89304	0.89613	0.89717	0.90052	0.90412	0.90810
694	0.88644	0.89316	0.89625	0.89729	0.90063	0.90423	0.90820
695	0.88658	0.89328	0.89637	0.89741	0.90074	0.90434	0.90831
696	0.88670	0.89340	0.89649	0.89752	0.90085	0.90444	0.90841
697	0.88682	0.89352	0.89661	0.89764	0.90096	0.90455	0.90851
698	0.88693	0.89364	0.89672	0.89775	0.90107	0.90466	0.90861
699	0.88707	0.89377	0.89684	0.89787	0.90118	0.90476	0.90871
700	0.88721	0.89388	0.89695	0.89798	0.90130	0.90487	0.90881
701	0.88733	0.89399	0.89707	0.89810	0.90140	0.90497	0.90891
702	0.88746	0.89412	0.89719	0.89821	0.90152	0.90508	0.90901
703	0.88759	0.89423	0.89730	0.89833	0.90163	0.90518	0.90911
704	0.88772	0.89435	0.89742	0.89844	0.90174	0.90529	0.90921
705	0.88786	0.89448	0.89753	0.89856	0.90185	0.90539	0.90931
706	0.88798	0.89460	0.89765	0.89867	0.90195	0.90550	0.90941
707	0.88810	0.89471	0.89776	0.89878	0.90206	0.90560	0.90951
708	0.88823	0.89483	0.89788	0.89889	0.90217	0.90570	0.90961
709	0.88836	0.89495	0.89799	0.89901	0.90228	0.90581	0.90971
710	0.88847	0.89507	0.89811	0.89912	0.90238	0.90591	0.90981
711	0.88861	0.89519	0.89822	0.89923	0.90249	0.90601	0.90990
712	0.88873	0.89530	0.89833	0.89934	0.90260	0.90612	0.91000
713	0.88886	0.89541	0.89844	0.89945	0.90271	0.90622	0.91010
714	0.88898	0.89553	0.89856	0.89956	0.90281	0.90632	0.91020
715	0.88911	0.89566	0.89867	0.89967	0.90292	0.90642	0.91029
716	0.88924	0.89578	0.89878	0.89978	0.90303	0.90653	0.91039
717	0.88934	0.89590	0.89889	0.89990	0.90313	0.90663	0.91049
718	0.88947	0.89601	0.89900	0.90000	0.90324	0.90673	0.91059
719	0.88960	0.89612	0.89911	0.90011	0.90334	0.90683	0.91068
720	0.88971	0.89623	0.89922	0.90022	0.90345	0.90693	0.91078
721	0.88984	0.89635	0.89933	0.90033	0.90355	0.90703	0.91087
722	0.88996	0.89647	0.89944	0.90044	0.90366	0.90714	0.91097
723	0.89009	0.89658	0.89955	0.90054	0.90376	0.90724	0.91106
724	0.89021	0.89670	0.89966	0.90065	0.90386	0.90734	0.91116
725	0.89034	0.89681	0.89977	0.90076	0.90397	0.90744	0.91125
726	0.89045	0.89692	0.89988	0.90087	0.90407	0.90754	0.91135
727	0.89056	0.89703	0.89999	0.90098	0.90418	0.90764	0.91144
728	0.89069	0.89714	0.90010	0.90108	0.90428	0.90773	0.91154
729	0.89081	0.89726	0.90020	0.90119	0.90438	0.90783	0.91163
730	0.89093	0.89737	0.90031	0.90130	0.90448	0.90793	0.91173
731	0.89105	0.89748	0.90042	0.90140	0.90459	0.90803	0.91182
732	0.89118	0.89759	0.90053	0.90151	0.90469	0.90813	0.91191
733	0.89129	0.89770	0.90063	0.90162	0.90479	0.90823	0.91201
734	0.89141	0.89782	0.90074	0.90173	0.90489	0.90832	0.91210
735	0.89152	0.89793	0.90085	0.90183	0.90500	0.90842	0.91219
736	0.89164	0.89804	0.90096	0.90193	0.90510	0.90852	0.91228
737	0.89175	0.89815	0.90106	0.90204	0.90520	0.90861	0.91238
738	0.89187	0.89826	0.90117	0.90214	0.90530	0.90871	0.91247
739	0.89198	0.89837	0.90127	0.90225	0.90540	0.90881	0.91256
740	0.89210	0.89848	0.90138	0.90235	0.90550	0.90890	0.91265
741	0.89222	0.89859	0.90148	0.90246	0.90560	0.90900	0.91274

N	0.1%	0.5%	1%	1.25%	2.5%	5%	10%
742	0.89233	0.89869	0.90159	0.90256	0.90570	0.90909	0.91283
743	0.89245	0.89881	0.90169	0.90266	0.90580	0.90919	0.91292
744	0.89255	0.89891	0.90180	0.90277	0.90590	0.90928	0.91301
745	0.89266	0.89902	0.90190	0.90287	0.90599	0.90938	0.91310
746	0.89278	0.89913	0.90200	0.90297	0.90609	0.90947	0.91319
747	0.89290	0.89924	0.90211	0.90308	0.90619	0.90957	0.91328
748	0.89301	0.89935	0.90221	0.90318	0.90629	0.90966	0.91337
749	0.89311	0.89945	0.90231	0.90328	0.90639	0.90976	0.91346
750	0.89323	0.89956	0.90242	0.90337	0.90649	0.90985	0.91355
751	0.89335	0.89966	0.90252	0.90348	0.90658	0.90994	0.91364
752	0.89348	0.89977	0.90262	0.90358	0.90668	0.91004	0.91373
753	0.89359	0.89987	0.90272	0.90368	0.90678	0.91013	0.91382
754	0.89370	0.89998	0.90282	0.90378	0.90687	0.91022	0.91391
755	0.89382	0.90008	0.90293	0.90388	0.90697	0.91032	0.91400
756	0.89393	0.90019	0.90303	0.90398	0.90707	0.91041	0.91409
757	0.89405	0.90029	0.90313	0.90409	0.90716	0.91050	0.91418
758	0.89415	0.90039	0.90323	0.90418	0.90726	0.91060	0.91426
759	0.89427	0.90050	0.90334	0.90429	0.90736	0.91069	0.91435
760	0.89438	0.90060	0.90343	0.90439	0.90745	0.91078	0.91444
761	0.89449	0.90070	0.90354	0.90449	0.90755	0.91087	0.91453
762	0.89461	0.90080	0.90364	0.90458	0.90765	0.91096	0.91461
763	0.89471	0.90091	0.90374	0.90468	0.90774	0.91105	0.91470
764	0.89482	0.90100	0.90384	0.90478	0.90784	0.91115	0.91479
765	0.89492	0.90110	0.90393	0.90488	0.90793	0.91124	0.91487
766	0.89505	0.90121	0.90403	0.90497	0.90802	0.91133	0.91496
767	0.89516	0.90131	0.90413	0.90507	0.90812	0.91142	0.91505
768	0.89526	0.90141	0.90422	0.90517	0.90821	0.91151	0.91513
769	0.89538	0.90151	0.90432	0.90527	0.90830	0.91160	0.91522
770	0.89549	0.90161	0.90442	0.90536	0.90840	0.91169	0.91531
771	0.89560	0.90171	0.90452	0.90546	0.90849	0.91178	0.91539
772	0.89570	0.90181	0.90462	0.90556	0.90859	0.91187	0.91548
773	0.89581	0.90191	0.90472	0.90566	0.90868	0.91196	0.91556
774	0.89591	0.90202	0.90481	0.90575	0.90877	0.91205	0.91565
775	0.89601	0.90211	0.90491	0.90585	0.90887	0.91213	0.91573
776	0.89613	0.90222	0.90500	0.90594	0.90896	0.91222	0.91581
777	0.89623	0.90232	0.90510	0.90604	0.90905	0.91231	0.91590
778	0.89634	0.90242	0.90520	0.90613	0.90914	0.91240	0.91598
779	0.89645	0.90252	0.90530	0.90623	0.90923	0.91248	0.91607
780	0.89657	0.90262	0.90539	0.90632	0.90933	0.91257	0.91615
781	0.89667	0.90272	0.90549	0.90642	0.90942	0.91266	0.91623
782	0.89677	0.90282	0.90558	0.90651	0.90951	0.91275	0.91632
783	0.89689	0.90291	0.90568	0.90660	0.90960	0.91284	0.91640
784	0.89698	0.90301	0.90578	0.90670	0.90969	0.91292	0.91648
785	0.89710	0.90311	0.90587	0.90679	0.90978	0.91301	0.91657
786	0.89720	0.90320	0.90596	0.90688	0.90987	0.91310	0.91665
787	0.89731	0.90331	0.90606	0.90698	0.90996	0.91318	0.91673
788	0.89741	0.90340	0.90615	0.90707	0.91005	0.91327	0.91681
789	0.89751	0.90350	0.90624	0.90716	0.91014	0.91336	0.91690
790	0.89762	0.90360	0.90634	0.90726	0.91023	0.91344	0.91698
791	0.89772	0.90370	0.90644	0.90735	0.91032	0.91353	0.91706
792	0.89784	0.90380	0.90653	0.90744	0.91041	0.91361	0.91714
793	0.89792	0.90389	0.90662	0.90754	0.91050	0.91370	0.91722
794	0.89803	0.90399	0.90671	0.90763	0.91059	0.91378	0.91731
795	0.89813	0.90408	0.90681	0.90772	0.91068	0.91387	0.91739
796	0.89823	0.90418	0.90690	0.90781	0.91077	0.91395	0.91747
797	0.89834	0.90428	0.90700	0.90791	0.91085	0.91404	0.91755
798	0.89843	0.90437	0.90709	0.90800	0.91094	0.91412	0.91763
799	0.89854	0.90447	0.90718	0.90809	0.91103	0.91421	0.91771
800	0.89864	0.90456	0.90728	0.90818	0.91112	0.91429	0.91779
801	0.89873	0.90466	0.90737	0.90827	0.91121	0.91437	0.91787
802	0.89883	0.90475	0.90746	0.90836	0.91129	0.91446	0.91795
803	0.89893	0.90485	0.90755	0.90845	0.91138	0.91454	0.91803
804	0.89903	0.90494	0.90764	0.90854	0.91147	0.91463	0.91811
805	0.89914	0.90504	0.90774	0.90863	0.91155	0.91471	0.91819
806	0.89923	0.90513	0.90783	0.90872	0.91164	0.91479	0.91827
807	0.89933	0.90522	0.90792	0.90881	0.91173	0.91487	0.91834
808	0.89943	0.90531	0.90801	0.90890	0.91181	0.91496	0.91842
809	0.89953	0.90541	0.90810	0.90899	0.91190	0.91504	0.91850
810	0.89964	0.90550	0.90819	0.90908	0.91198	0.91512	0.91858
811	0.89974	0.90559	0.90828	0.90917	0.91207	0.91520	0.91866
812	0.89984	0.90569	0.90837	0.90926	0.91216	0.91528	0.91874
813	0.89993	0.90578	0.90845	0.90934	0.91224	0.91536	0.91881
814	0.90004	0.90587	0.90854	0.90943	0.91233	0.91545	0.91889
815	0.90014	0.90596	0.90863	0.90952	0.91241	0.91553	0.91897

N	0.1%	0.5%	1%	1.25%	2.5%	5%	10%
816	0.90024	0.90605	0.90872	0.90961	0.91249	0.91561	0.91905
817	0.90033	0.90614	0.90881	0.90969	0.91258	0.91569	0.91912
818	0.90044	0.90624	0.90889	0.90978	0.91266	0.91577	0.91920
819	0.90054	0.90633	0.90898	0.90987	0.91275	0.91585	0.91928
820	0.90065	0.90642	0.90907	0.90995	0.91283	0.91593	0.91936
821	0.90074	0.90651	0.90916	0.91004	0.91291	0.91601	0.91943
822	0.90084	0.90660	0.90925	0.91012	0.91300	0.91609	0.91951
823	0.90093	0.90669	0.90933	0.91021	0.91308	0.91617	0.91959
824	0.90103	0.90678	0.90942	0.91030	0.91316	0.91625	0.91966
825	0.90113	0.90687	0.90951	0.91039	0.91325	0.91633	0.91974
826	0.90121	0.90695	0.90959	0.91047	0.91333	0.91641	0.91981
827	0.90132	0.90705	0.90968	0.91056	0.91341	0.91649	0.91989
828	0.90140	0.90713	0.90977	0.91065	0.91349	0.91657	0.91996
829	0.90150	0.90722	0.90986	0.91073	0.91358	0.91665	0.92004
830	0.90160	0.90731	0.90995	0.91082	0.91366	0.91673	0.92011
831	0.90170	0.90740	0.91003	0.91090	0.91374	0.91680	0.92019
832	0.90179	0.90749	0.91012	0.91099	0.91382	0.91688	0.92026
833	0.90188	0.90758	0.91020	0.91107	0.91390	0.91696	0.92034
834	0.90199	0.90767	0.91029	0.91116	0.91398	0.91704	0.92041
835	0.90208	0.90776	0.91037	0.91124	0.91407	0.91712	0.92049
836	0.90217	0.90785	0.91046	0.91133	0.91415	0.91720	0.92056
837	0.90226	0.90793	0.91054	0.91141	0.91423	0.91727	0.92063
838	0.90236	0.90802	0.91063	0.91149	0.91431	0.91735	0.92071
839	0.90246	0.90811	0.91071	0.91158	0.91439	0.91743	0.92078
840	0.90255	0.90819	0.91080	0.91166	0.91447	0.91751	0.92085
841	0.90264	0.90828	0.91089	0.91174	0.91455	0.91758	0.92093
842	0.90273	0.90837	0.91097	0.91183	0.91463	0.91766	0.92100
843	0.90282	0.90846	0.91105	0.91191	0.91471	0.91774	0.92108
844	0.90293	0.90854	0.91113	0.91199	0.91479	0.91781	0.92115
845	0.90301	0.90863	0.91121	0.91208	0.91487	0.91789	0.92122
846	0.90310	0.90872	0.91130	0.91216	0.91495	0.91797	0.92130
847	0.90319	0.90880	0.91138	0.91224	0.91503	0.91804	0.92137
848	0.90329	0.90889	0.91147	0.91233	0.91511	0.91812	0.92144
849	0.90338	0.90897	0.91155	0.91241	0.91519	0.91819	0.92151
850	0.90347	0.90906	0.91163	0.91249	0.91526	0.91827	0.92158
851	0.90356	0.90914	0.91171	0.91257	0.91534	0.91834	0.92166
852	0.90366	0.90923	0.91179	0.91265	0.91542	0.91842	0.92173
853	0.90375	0.90931	0.91188	0.91273	0.91550	0.91850	0.92180
854	0.90384	0.90940	0.91196	0.91281	0.91558	0.91857	0.92187
855	0.90393	0.90948	0.91204	0.91289	0.91566	0.91864	0.92194
856	0.90402	0.90957	0.91212	0.91298	0.91574	0.91872	0.92201
857	0.90411	0.90966	0.91220	0.91306	0.91581	0.91880	0.92209
858	0.90419	0.90974	0.91228	0.91314	0.91589	0.91887	0.92216
859	0.90428	0.90982	0.91237	0.91322	0.91597	0.91894	0.92223
860	0.90437	0.90991	0.91245	0.91330	0.91604	0.91902	0.92230
861	0.90445	0.90999	0.91253	0.91338	0.91612	0.91909	0.92237
862	0.90454	0.91007	0.91261	0.91346	0.91620	0.91917	0.92244
863	0.90464	0.91015	0.91269	0.91354	0.91628	0.91924	0.92251
864	0.90472	0.91024	0.91277	0.91362	0.91635	0.91932	0.92258
865	0.90480	0.91032	0.91285	0.91370	0.91643	0.91939	0.92265
866	0.90489	0.91041	0.91294	0.91378	0.91651	0.91946	0.92272
867	0.90499	0.91049	0.91301	0.91386	0.91658	0.91954	0.92279
868	0.90507	0.91058	0.91309	0.91394	0.91666	0.91961	0.92286
869	0.90516	0.91066	0.91317	0.91402	0.91674	0.91968	0.92293
870	0.90524	0.91074	0.91326	0.91409	0.91681	0.91976	0.92300
871	0.90534	0.91082	0.91333	0.91417	0.91689	0.91983	0.92307
872	0.90543	0.91090	0.91341	0.91425	0.91696	0.91990	0.92314
873	0.90551	0.91098	0.91349	0.91433	0.91704	0.91997	0.92321
874	0.90560	0.91106	0.91357	0.91441	0.91711	0.92005	0.92328
875	0.90568	0.91114	0.91365	0.91449	0.91719	0.92012	0.92335
876	0.90577	0.91122	0.91372	0.91457	0.91727	0.92019	0.92341
877	0.90587	0.91130	0.91381	0.91465	0.91734	0.92026	0.92348
878	0.90595	0.91138	0.91388	0.91472	0.91742	0.92033	0.92355
879	0.90603	0.91146	0.91396	0.91480	0.91749	0.92041	0.92362
880	0.90611	0.91154	0.91404	0.91488	0.91756	0.92048	0.92369
881	0.90620	0.91162	0.91412	0.91496	0.91764	0.92055	0.92376
882	0.90628	0.91171	0.91419	0.91504	0.91771	0.92062	0.92382
883	0.90636	0.91179	0.91427	0.91511	0.91779	0.92069	0.92389
884	0.90645	0.91187	0.91435	0.91519	0.91786	0.92076	0.92396
885	0.90653	0.91195	0.91443	0.91526	0.91794	0.92083	0.92403
886	0.90662	0.91203	0.91451	0.91534	0.91801	0.92090	0.92409
887	0.90670	0.91211	0.91458	0.91542	0.91808	0.92097	0.92416
888	0.90679	0.91219	0.91466	0.91549	0.91815	0.92104	0.92423
889	0.90688	0.91227	0.91474	0.91557	0.91823	0.92111	0.92429

N	0.1%	0.5%	1%	1.25%	2.5%	5%	10%
890	0.90696	0.91235	0.91481	0.91564	0.91830	0.92118	0.92436
891	0.90706	0.91242	0.91489	0.91572	0.91837	0.92126	0.92443
892	0.90713	0.91250	0.91497	0.91579	0.91844	0.92133	0.92449
893	0.90721	0.91258	0.91504	0.91587	0.91852	0.92139	0.92456
894	0.90729	0.91266	0.91512	0.91594	0.91859	0.92146	0.92462
895	0.90737	0.91274	0.91519	0.91602	0.91866	0.92153	0.92469
896	0.90745	0.91282	0.91527	0.91609	0.91873	0.92160	0.92476
897	0.90753	0.91290	0.91534	0.91617	0.91881	0.92167	0.92482
898	0.90762	0.91297	0.91542	0.91624	0.91888	0.92174	0.92489
899	0.90770	0.91305	0.91549	0.91631	0.91895	0.92181	0.92495
900	0.90779	0.91313	0.91557	0.91639	0.91902	0.92187	0.92502
901	0.90788	0.91320	0.91564	0.91646	0.91910	0.92194	0.92508
902	0.90795	0.91328	0.91572	0.91653	0.91917	0.92201	0.92515
903	0.90803	0.91336	0.91579	0.91661	0.91924	0.92208	0.92521
904	0.90813	0.91344	0.91587	0.91668	0.91931	0.92215	0.92528
905	0.90821	0.91352	0.91594	0.91675	0.91938	0.92222	0.92535
906	0.90829	0.91359	0.91602	0.91683	0.91945	0.92228	0.92541
907	0.90837	0.91367	0.91609	0.91690	0.91952	0.92235	0.92547
908	0.90845	0.91374	0.91616	0.91697	0.91959	0.92242	0.92554
909	0.90853	0.91382	0.91623	0.91704	0.91966	0.92249	0.92560
910	0.90861	0.91390	0.91631	0.91712	0.91973	0.92255	0.92567
911	0.90869	0.91397	0.91638	0.91719	0.91980	0.92262	0.92573
912	0.90877	0.91405	0.91645	0.91727	0.91987	0.92269	0.92579
913	0.90885	0.91412	0.91653	0.91734	0.91994	0.92275	0.92586
914	0.90893	0.91419	0.91660	0.91741	0.92001	0.92282	0.92592
915	0.90902	0.91427	0.91668	0.91748	0.92008	0.92288	0.92599
916	0.90909	0.91435	0.91675	0.91755	0.92015	0.92295	0.92605
917	0.90917	0.91442	0.91682	0.91762	0.92022	0.92302	0.92611
918	0.90925	0.91449	0.91689	0.91770	0.92029	0.92308	0.92617
919	0.90932	0.91457	0.91696	0.91777	0.92036	0.92315	0.92624
920	0.90941	0.91464	0.91704	0.91784	0.92042	0.92322	0.92630
921	0.90949	0.91471	0.91711	0.91791	0.92049	0.92328	0.92636
922	0.90958	0.91479	0.91718	0.91798	0.92056	0.92335	0.92643
923	0.90965	0.91487	0.91725	0.91805	0.92063	0.92341	0.92649
924	0.90972	0.91494	0.91732	0.91812	0.92070	0.92348	0.92655
925	0.90981	0.91501	0.91740	0.91819	0.92077	0.92354	0.92661
926	0.90989	0.91509	0.91747	0.91827	0.92083	0.92361	0.92668
927	0.90996	0.91517	0.91754	0.91834	0.92090	0.92367	0.92674
928	0.91003	0.91524	0.91762	0.91841	0.92097	0.92374	0.92680
929	0.91011	0.91531	0.91769	0.91848	0.92103	0.92381	0.92686
930	0.91019	0.91539	0.91775	0.91855	0.92110	0.92387	0.92692
931	0.91027	0.91546	0.91783	0.91862	0.92117	0.92393	0.92698
932	0.91034	0.91553	0.91790	0.91868	0.92124	0.92400	0.92705
933	0.91043	0.91560	0.91797	0.91876	0.92130	0.92406	0.92711
934	0.91051	0.91568	0.91804	0.91882	0.92137	0.92413	0.92717
935	0.91059	0.91575	0.91811	0.91889	0.92144	0.92419	0.92723
936	0.91066	0.91582	0.91818	0.91896	0.92151	0.92426	0.92729
937	0.91074	0.91590	0.91825	0.91903	0.92157	0.92432	0.92735
938	0.91082	0.91597	0.91832	0.91910	0.92164	0.92438	0.92741
939	0.91090	0.91604	0.91838	0.91917	0.92170	0.92445	0.92747
940	0.91098	0.91611	0.91845	0.91924	0.92177	0.92451	0.92753
941	0.91106	0.91619	0.91853	0.91931	0.92183	0.92457	0.92759
942	0.91113	0.91626	0.91860	0.91938	0.92190	0.92464	0.92765
943	0.91120	0.91633	0.91866	0.91945	0.92197	0.92470	0.92771
944	0.91128	0.91640	0.91873	0.91952	0.92203	0.92476	0.92778
945	0.91135	0.91647	0.91880	0.91958	0.92210	0.92483	0.92784
946	0.91143	0.91655	0.91887	0.91965	0.92216	0.92489	0.92790
947	0.91150	0.91661	0.91894	0.91972	0.92223	0.92495	0.92796
948	0.91158	0.91668	0.91900	0.91979	0.92229	0.92502	0.92802
949	0.91165	0.91676	0.91907	0.91985	0.92236	0.92508	0.92808
950	0.91173	0.91683	0.91914	0.91992	0.92243	0.92514	0.92813
951	0.91180	0.91690	0.91921	0.91998	0.92249	0.92520	0.92819
952	0.91188	0.91697	0.91928	0.92005	0.92256	0.92526	0.92825
953	0.91195	0.91703	0.91935	0.92012	0.92262	0.92533	0.92831
954	0.91204	0.91711	0.91941	0.92019	0.92268	0.92539	0.92837
955	0.91211	0.91718	0.91948	0.92025	0.92275	0.92545	0.92843
956	0.91219	0.91725	0.91955	0.92032	0.92281	0.92551	0.92849
957	0.91226	0.91732	0.91962	0.92039	0.92287	0.92557	0.92855
958	0.91233	0.91738	0.91968	0.92045	0.92294	0.92564	0.92861
959	0.91240	0.91745	0.91975	0.92052	0.92301	0.92570	0.92866
960	0.91247	0.91752	0.91982	0.92059	0.92307	0.92576	0.92872
961	0.91256	0.91759	0.91989	0.92065	0.92313	0.92582	0.92878
962	0.91263	0.91766	0.91995	0.92072	0.92320	0.92588	0.92884
963	0.91271	0.91773	0.92002	0.92079	0.92326	0.92594	0.92890

N	0.1%	0.5%	1%	1.25%	2.5%	5%	10%
964	0.91278	0.91780	0.92008	0.92085	0.92333	0.92600	0.92896
965	0.91286	0.91787	0.92015	0.92092	0.92339	0.92606	0.92901
966	0.91293	0.91793	0.92022	0.92098	0.92345	0.92613	0.92907
967	0.91301	0.91800	0.92028	0.92105	0.92352	0.92619	0.92913
968	0.91308	0.91807	0.92035	0.92111	0.92358	0.92624	0.92919
969	0.91315	0.91813	0.92041	0.92117	0.92364	0.92631	0.92924
970	0.91323	0.91820	0.92048	0.92124	0.92370	0.92637	0.92930
971	0.91330	0.91827	0.92054	0.92131	0.92377	0.92642	0.92936
972	0.91338	0.91833	0.92061	0.92137	0.92383	0.92648	0.92942
973	0.91345	0.91840	0.92067	0.92143	0.92389	0.92654	0.92947
974	0.91352	0.91847	0.92074	0.92150	0.92395	0.92660	0.92953
975	0.91359	0.91853	0.92080	0.92156	0.92401	0.92666	0.92959
976	0.91366	0.91860	0.92087	0.92163	0.92408	0.92672	0.92964
977	0.91373	0.91867	0.92093	0.92169	0.92414	0.92678	0.92970
978	0.91379	0.91874	0.92100	0.92175	0.92420	0.92684	0.92976
979	0.91386	0.91880	0.92107	0.92182	0.92426	0.92690	0.92981
980	0.91394	0.91887	0.92113	0.92188	0.92432	0.92696	0.92987
981	0.91402	0.91893	0.92119	0.92194	0.92439	0.92702	0.92993
982	0.91408	0.91900	0.92126	0.92201	0.92445	0.92708	0.92998
983	0.91416	0.91907	0.92132	0.92207	0.92451	0.92714	0.93004
984	0.91422	0.91913	0.92139	0.92214	0.92457	0.92719	0.93009
985	0.91431	0.91920	0.92145	0.92220	0.92463	0.92725	0.93015
986	0.91438	0.91927	0.92152	0.92226	0.92469	0.92731	0.93021
987	0.91446	0.91933	0.92158	0.92233	0.92475	0.92737	0.93026
988	0.91453	0.91940	0.92164	0.92239	0.92482	0.92743	0.93032
989	0.91460	0.91947	0.92170	0.92245	0.92488	0.92749	0.93037
990	0.91467	0.91953	0.92177	0.92251	0.92494	0.92755	0.93043
991	0.91473	0.91960	0.92183	0.92258	0.92500	0.92760	0.93048
992	0.91480	0.91966	0.92189	0.92264	0.92506	0.92766	0.93054
993	0.91487	0.91973	0.92196	0.92270	0.92512	0.92772	0.93059
994	0.91493	0.91980	0.92202	0.92276	0.92518	0.92778	0.93065
995	0.91501	0.91986	0.92208	0.92283	0.92524	0.92784	0.93071
996	0.91508	0.91992	0.92215	0.92289	0.92530	0.92789	0.93076
997	0.91515	0.91998	0.92221	0.92295	0.92536	0.92795	0.93081
998	0.91522	0.92005	0.92227	0.92301	0.92542	0.92801	0.93087
999	0.91528	0.92011	0.92234	0.92307	0.92548	0.92806	0.93092
1000	0.91535	0.92018	0.92240	0.92314	0.92554	0.92812	0.93098

Table 2 – *GM8(7H1L or 1H7L)*. Critical values of upper significance levels for the Grubbs multiple-8 outlier test, applicable in situations where the seven highest and one lowest or one highest and seven lowest observations are simultaneously identified as outliers, denoted as *GM8(7H1L)* and *GM8(1H7L)*, respectively.

N	0.1%	0.5%	1%	1.25%	2.5%	5%	10%
10	0.00000	0.00000	0.00000	0.00000	0.00001	0.00003	0.00011
11	0.00002	0.00009	0.00018	0.00023	0.00048	0.00101	0.00219
12	0.00026	0.00080	0.00132	0.00155	0.00257	0.00436	0.00757
13	0.00115	0.00272	0.00400	0.00453	0.00674	0.01018	0.01574
14	0.00297	0.00605	0.00831	0.00922	0.01282	0.01807	0.02602
15	0.00587	0.01078	0.01413	0.01545	0.02051	0.02756	0.03775
16	0.00988	0.01674	0.02122	0.02297	0.02947	0.03824	0.05046
17	0.01486	0.02378	0.02940	0.03152	0.03938	0.04975	0.06379
18	0.02075	0.03167	0.03840	0.04090	0.05004	0.06181	0.07746
19	0.02738	0.04032	0.04805	0.05093	0.06123	0.07422	0.09132
20	0.03467	0.04950	0.05815	0.06133	0.07265	0.08683	0.10520
21	0.04238	0.05918	0.06864	0.07212	0.08431	0.09953	0.11896
22	0.05059	0.06899	0.07932	0.08303	0.09612	0.11218	0.13255
23	0.05921	0.07900	0.09006	0.09400	0.10786	0.12476	0.14591
24	0.06798	0.08915	0.10088	0.10506	0.11958	0.13716	0.15903
25	0.07678	0.09943	0.11164	0.11603	0.13122	0.14939	0.17185
26	0.08599	0.10971	0.12244	0.12698	0.14270	0.16145	0.18432
27	0.09522	0.12000	0.13317	0.13786	0.15398	0.17319	0.19656
28	0.10453	0.13015	0.14377	0.14855	0.16513	0.18469	0.20842
29	0.11365	0.14010	0.15417	0.15911	0.17608	0.19594	0.22002
30	0.12296	0.15005	0.16442	0.16946	0.18675	0.20694	0.23124
31	0.13180	0.15990	0.17454	0.17964	0.19720	0.21769	0.24220
32	0.14076	0.16960	0.18447	0.18968	0.20751	0.22817	0.25283
33	0.14978	0.17913	0.19425	0.19957	0.21753	0.23837	0.26316
34	0.15872	0.18853	0.20387	0.20923	0.22742	0.24838	0.27325
35	0.16759	0.19775	0.21333	0.21877	0.23706	0.25810	0.28304
36	0.17635	0.20687	0.22261	0.22810	0.24647	0.26757	0.29253
37	0.18478	0.21584	0.23172	0.23719	0.25560	0.27682	0.30177

N	0.1%	0.5%	1%	1.25%	2.5%	5%	10%
38	0.19321	0.22456	0.24049	0.24601	0.26457	0.28583	0.31078
39	0.20144	0.23308	0.24914	0.25465	0.27329	0.29461	0.31952
40	0.20962	0.24149	0.25758	0.26316	0.28185	0.30313	0.32803
41	0.21755	0.24979	0.26590	0.27148	0.29020	0.31148	0.33627
42	0.22544	0.25777	0.27404	0.27962	0.29834	0.31960	0.34435
43	0.23332	0.26584	0.28198	0.28760	0.30631	0.32753	0.35223
44	0.24077	0.27352	0.28969	0.29535	0.31410	0.33524	0.35984
45	0.24838	0.28107	0.29736	0.30293	0.32169	0.34278	0.36729
46	0.25585	0.28843	0.30475	0.31036	0.32904	0.35016	0.37455
47	0.26284	0.29577	0.31208	0.31766	0.33629	0.35734	0.38163
48	0.26993	0.30296	0.31918	0.32474	0.34337	0.36433	0.38853
49	0.27691	0.30991	0.32607	0.33167	0.35025	0.37118	0.39529
50	0.28379	0.31673	0.33290	0.33852	0.35705	0.37787	0.40186
51	0.29056	0.32349	0.33971	0.34527	0.36367	0.38438	0.40826
52	0.29720	0.33001	0.34620	0.35168	0.37015	0.39079	0.41451
53	0.30382	0.33649	0.35258	0.35815	0.37646	0.39702	0.42059
54	0.31019	0.34270	0.35889	0.36434	0.38268	0.40316	0.42658
55	0.31633	0.34899	0.36500	0.37049	0.38873	0.40911	0.43240
56	0.32231	0.35507	0.37107	0.37651	0.39463	0.41494	0.43811
57	0.32814	0.36107	0.37695	0.38239	0.40048	0.42065	0.44368
58	0.33408	0.36692	0.38283	0.38819	0.40614	0.42622	0.44914
59	0.33999	0.37269	0.38847	0.39382	0.41172	0.43167	0.45445
60	0.34578	0.37829	0.39403	0.39940	0.41718	0.43706	0.45969
61	0.35138	0.38384	0.39951	0.40485	0.42255	0.44228	0.46478
62	0.35675	0.38925	0.40486	0.41017	0.42778	0.44743	0.46977
63	0.36218	0.39454	0.41009	0.41544	0.43295	0.45247	0.47465
64	0.36749	0.39965	0.41520	0.42052	0.43796	0.45739	0.47942
65	0.37283	0.40473	0.42027	0.42551	0.44290	0.46217	0.48412
66	0.37796	0.40973	0.42519	0.43047	0.44777	0.46690	0.48870
67	0.38308	0.41469	0.43008	0.43529	0.45251	0.47156	0.49318
68	0.38792	0.41957	0.43487	0.44006	0.45713	0.47611	0.49760
69	0.39281	0.42427	0.43955	0.44471	0.46169	0.48055	0.50194
70	0.39761	0.42900	0.44421	0.44935	0.46620	0.48493	0.50616
71	0.40215	0.43363	0.44871	0.45382	0.47063	0.48925	0.51032
72	0.40678	0.43811	0.45315	0.45821	0.47494	0.49345	0.51440
73	0.41130	0.44257	0.45746	0.46254	0.47919	0.49759	0.51839
74	0.41563	0.44698	0.46178	0.46682	0.48336	0.50163	0.52232
75	0.42021	0.45125	0.46598	0.47098	0.48745	0.50562	0.52617
76	0.42446	0.45544	0.47011	0.47509	0.49146	0.50954	0.52997
77	0.42876	0.45953	0.47418	0.47910	0.49542	0.51339	0.53366
78	0.43312	0.46366	0.47819	0.48309	0.49932	0.51716	0.53732
79	0.43720	0.46765	0.48209	0.48700	0.50309	0.52089	0.54089
80	0.44128	0.47159	0.48597	0.49083	0.50685	0.52452	0.54443
81	0.44532	0.47542	0.48974	0.49463	0.51051	0.52810	0.54789
82	0.44928	0.47924	0.49348	0.49834	0.51419	0.53162	0.55129
83	0.45302	0.48299	0.49716	0.50201	0.51775	0.53511	0.55463
84	0.45681	0.48668	0.50080	0.50559	0.52124	0.53852	0.55792
85	0.46077	0.49029	0.50439	0.50912	0.52469	0.54187	0.56113
86	0.46442	0.49387	0.50791	0.51264	0.52809	0.54517	0.56432
87	0.46803	0.49736	0.51139	0.51607	0.53142	0.54841	0.56744
88	0.47176	0.50085	0.51475	0.51940	0.53474	0.55159	0.57052
89	0.47535	0.50430	0.51808	0.52271	0.53796	0.55473	0.57354
90	0.47886	0.50774	0.52139	0.52600	0.54115	0.55783	0.57655
91	0.48228	0.51105	0.52464	0.52924	0.54429	0.56088	0.57947
92	0.48561	0.51430	0.52782	0.53242	0.54738	0.56388	0.58236
93	0.48907	0.51750	0.53096	0.53555	0.55045	0.56685	0.58518
94	0.49232	0.52071	0.53409	0.53866	0.55346	0.56973	0.58797
95	0.49555	0.52381	0.53716	0.54166	0.55641	0.57256	0.59073
96	0.49874	0.52691	0.54017	0.54465	0.55931	0.57539	0.59345
97	0.50182	0.52990	0.54314	0.54760	0.56217	0.57817	0.59612
98	0.50491	0.53287	0.54610	0.55050	0.56502	0.58090	0.59877
99	0.50799	0.53584	0.54899	0.55340	0.56778	0.58361	0.60136
100	0.51108	0.53879	0.55181	0.55619	0.57052	0.58627	0.60391
101	0.51407	0.54163	0.55464	0.55901	0.57325	0.58888	0.60643
102	0.51705	0.54447	0.55742	0.56172	0.57589	0.59147	0.60889
103	0.52002	0.54732	0.56014	0.56445	0.57851	0.59401	0.61135
104	0.52301	0.55005	0.56280	0.56714	0.58113	0.59653	0.61376
105	0.52593	0.55278	0.56547	0.56977	0.58368	0.59899	0.61615
106	0.52871	0.55548	0.56810	0.57236	0.58623	0.60142	0.61848
107	0.53143	0.55813	0.57069	0.57493	0.58870	0.60384	0.62080
108	0.53410	0.56077	0.57323	0.57747	0.59117	0.60621	0.62308
109	0.53677	0.56333	0.57576	0.57997	0.59359	0.60856	0.62531
110	0.53949	0.56590	0.57826	0.58242	0.59595	0.61088	0.62752
111	0.54211	0.56836	0.58071	0.58484	0.59832	0.61316	0.62972

N	0.1%	0.5%	1%	1.25%	2.5%	5%	10%
112	0.54470	0.57084	0.58312	0.58726	0.60062	0.61541	0.63188
113	0.54725	0.57324	0.58555	0.58965	0.60295	0.61763	0.63402
114	0.54981	0.57567	0.58789	0.59195	0.60521	0.61982	0.63611
115	0.55236	0.57806	0.59022	0.59424	0.60745	0.62199	0.63818
116	0.55494	0.58042	0.59247	0.59650	0.60970	0.62412	0.64023
117	0.55732	0.58274	0.59475	0.59877	0.61184	0.62621	0.64224
118	0.55976	0.58503	0.59701	0.60098	0.61401	0.62829	0.64424
119	0.56217	0.58729	0.59919	0.60315	0.61613	0.63034	0.64621
120	0.56455	0.58951	0.60141	0.60534	0.61824	0.63237	0.64815
121	0.56685	0.59175	0.60358	0.60751	0.62033	0.63437	0.65008
122	0.56911	0.59395	0.60570	0.60963	0.62239	0.63633	0.65198
123	0.57143	0.59609	0.60779	0.61171	0.62441	0.63830	0.65384
124	0.57379	0.59823	0.60989	0.61375	0.62643	0.64024	0.65570
125	0.57602	0.60036	0.61190	0.61577	0.62841	0.64215	0.65754
126	0.57810	0.60247	0.61392	0.61778	0.63037	0.64404	0.65936
127	0.58032	0.60452	0.61595	0.61980	0.63230	0.64590	0.66115
128	0.58245	0.60656	0.61796	0.62178	0.63422	0.64775	0.66291
129	0.58455	0.60861	0.61992	0.62371	0.63611	0.64957	0.66466
130	0.58663	0.61056	0.62183	0.62560	0.63795	0.65137	0.66638
131	0.58874	0.61255	0.62377	0.62753	0.63981	0.65316	0.66809
132	0.59095	0.61449	0.62563	0.62937	0.64162	0.65491	0.66978
133	0.59294	0.61643	0.62755	0.63123	0.64342	0.65665	0.67145
134	0.59498	0.61833	0.62936	0.63307	0.64520	0.65837	0.67309
135	0.59698	0.62022	0.63122	0.63490	0.64697	0.66009	0.67472
136	0.59889	0.62208	0.63303	0.63670	0.64871	0.66176	0.67632
137	0.60086	0.62396	0.63484	0.63850	0.65043	0.66342	0.67791
138	0.60280	0.62580	0.63657	0.64023	0.65213	0.66508	0.67948
139	0.60468	0.62759	0.63838	0.64199	0.65382	0.66670	0.68103
140	0.60661	0.62941	0.64013	0.64370	0.65550	0.66832	0.68257
141	0.60848	0.63115	0.64183	0.64542	0.65716	0.66992	0.68407
142	0.61032	0.63288	0.64354	0.64712	0.65878	0.67148	0.68558
143	0.61217	0.63460	0.64524	0.64880	0.66040	0.67304	0.68708
144	0.61393	0.63631	0.64691	0.65045	0.66199	0.67458	0.68855
145	0.61578	0.63807	0.64857	0.65209	0.66358	0.67611	0.69001
146	0.61758	0.63975	0.65020	0.65370	0.66514	0.67762	0.69145
147	0.61929	0.64141	0.65184	0.65532	0.66669	0.67911	0.69287
148	0.62108	0.64304	0.65343	0.65691	0.66821	0.68058	0.69428
149	0.62284	0.64467	0.65502	0.65846	0.66973	0.68203	0.69567
150	0.62450	0.64629	0.65657	0.66001	0.67123	0.68348	0.69706
151	0.62624	0.64789	0.65813	0.66158	0.67272	0.68492	0.69843
152	0.62784	0.64946	0.65967	0.66311	0.67421	0.68633	0.69979
153	0.62946	0.65103	0.66121	0.66461	0.67567	0.68774	0.70114
154	0.63113	0.65256	0.66274	0.66611	0.67711	0.68913	0.70247
155	0.63281	0.65409	0.66422	0.66760	0.67854	0.69052	0.70378
156	0.63445	0.65565	0.66572	0.66904	0.67994	0.69186	0.70508
157	0.63602	0.65715	0.66717	0.67048	0.68136	0.69322	0.70636
158	0.63762	0.65867	0.66859	0.67188	0.68275	0.69454	0.70764
159	0.63919	0.66015	0.67002	0.67331	0.68412	0.69586	0.70891
160	0.64074	0.66163	0.67144	0.67473	0.68549	0.69716	0.71016
161	0.64229	0.66303	0.67285	0.67613	0.68684	0.69847	0.71139
162	0.64372	0.66445	0.67422	0.67749	0.68818	0.69977	0.71263
163	0.64527	0.66590	0.67558	0.67886	0.68949	0.70103	0.71384
164	0.64679	0.66728	0.67696	0.68023	0.69080	0.70229	0.71505
165	0.64827	0.66869	0.67830	0.68159	0.69210	0.70355	0.71623
166	0.64972	0.67006	0.67968	0.68291	0.69340	0.70477	0.71742
167	0.65121	0.67141	0.68099	0.68422	0.69467	0.70601	0.71860
168	0.65261	0.67276	0.68229	0.68552	0.69592	0.70722	0.71976
169	0.65409	0.67413	0.68361	0.68681	0.69718	0.70842	0.72091
170	0.65547	0.67547	0.68493	0.68810	0.69842	0.70962	0.72204
171	0.65685	0.67678	0.68621	0.68935	0.69963	0.71079	0.72317
172	0.65825	0.67814	0.68751	0.69062	0.70084	0.71196	0.72428
173	0.65963	0.67944	0.68876	0.69186	0.70203	0.71312	0.72539
174	0.66100	0.68074	0.69000	0.69308	0.70323	0.71427	0.72649
175	0.66235	0.68198	0.69123	0.69432	0.70442	0.71541	0.72758
176	0.66357	0.68323	0.69244	0.69551	0.70558	0.71653	0.72866
177	0.66488	0.68450	0.69366	0.69673	0.70675	0.71765	0.72972
178	0.66621	0.68570	0.69485	0.69791	0.70789	0.71876	0.73078
179	0.66750	0.68697	0.69606	0.69912	0.70903	0.71987	0.73184
180	0.66877	0.68819	0.69724	0.70028	0.71017	0.72096	0.73287
181	0.67004	0.68940	0.69842	0.70143	0.71129	0.72203	0.73390
182	0.67138	0.69058	0.69958	0.70260	0.71241	0.72309	0.73493
183	0.67264	0.69179	0.70073	0.70373	0.71352	0.72415	0.73594
184	0.67388	0.69296	0.70187	0.70487	0.71462	0.72519	0.73695
185	0.67516	0.69411	0.70300	0.70599	0.71570	0.72624	0.73795

N	0.1%	0.5%	1%	1.25%	2.5%	5%	10%
186	0.67637	0.69526	0.70412	0.70711	0.71678	0.72727	0.73893
187	0.67758	0.69638	0.70524	0.70821	0.71785	0.72831	0.73991
188	0.67874	0.69753	0.70633	0.70930	0.71890	0.72931	0.74088
189	0.67990	0.69868	0.70744	0.71040	0.71996	0.73032	0.74183
190	0.68111	0.69980	0.70852	0.71147	0.72100	0.73132	0.74278
191	0.68230	0.70090	0.70961	0.71252	0.72205	0.73231	0.74374
192	0.68346	0.70198	0.71069	0.71360	0.72306	0.73330	0.74467
193	0.68454	0.70307	0.71175	0.71463	0.72407	0.73427	0.74560
194	0.68573	0.70415	0.71277	0.71566	0.72508	0.73524	0.74653
195	0.68691	0.70521	0.71382	0.71669	0.72606	0.73620	0.74744
196	0.68801	0.70627	0.71486	0.71774	0.72705	0.73714	0.74835
197	0.68916	0.70731	0.71590	0.71874	0.72803	0.73809	0.74925
198	0.69029	0.70835	0.71691	0.71974	0.72901	0.73901	0.75014
199	0.69134	0.70939	0.71791	0.72074	0.72997	0.73995	0.75104
200	0.69250	0.71043	0.71894	0.72172	0.73093	0.74087	0.75191
201	0.69356	0.71145	0.71992	0.72272	0.73189	0.74178	0.75279
202	0.69473	0.71248	0.72090	0.72369	0.73284	0.74270	0.75365
203	0.69575	0.71347	0.72189	0.72466	0.73377	0.74360	0.75451
204	0.69680	0.71445	0.72285	0.72563	0.73469	0.74449	0.75537
205	0.69782	0.71546	0.72381	0.72658	0.73559	0.74538	0.75621
206	0.69883	0.71644	0.72477	0.72752	0.73651	0.74626	0.75705
207	0.69983	0.71743	0.72571	0.72847	0.73742	0.74713	0.75788
208	0.70085	0.71838	0.72667	0.72941	0.73833	0.74799	0.75870
209	0.70189	0.71938	0.72758	0.73034	0.73923	0.74885	0.75952
210	0.70293	0.72035	0.72852	0.73124	0.74010	0.74970	0.76033
211	0.70392	0.72132	0.72944	0.73217	0.74098	0.75056	0.76114
212	0.70492	0.72222	0.73036	0.73307	0.74186	0.75139	0.76194
213	0.70592	0.72318	0.73127	0.73396	0.74271	0.75222	0.76273
214	0.70693	0.72410	0.73215	0.73486	0.74358	0.75304	0.76352
215	0.70792	0.72501	0.73306	0.73575	0.74443	0.75387	0.76430
216	0.70886	0.72595	0.73394	0.73662	0.74528	0.75469	0.76508
217	0.70979	0.72686	0.73482	0.73746	0.74613	0.75549	0.76587
218	0.71078	0.72775	0.73570	0.73835	0.74696	0.75629	0.76663
219	0.71173	0.72864	0.73658	0.73919	0.74780	0.75709	0.76738
220	0.71269	0.72953	0.73744	0.74005	0.74862	0.75788	0.76814
221	0.71357	0.73044	0.73828	0.74091	0.74944	0.75867	0.76889
222	0.71452	0.73133	0.73914	0.74176	0.75024	0.75945	0.76964
223	0.71545	0.73218	0.73999	0.74258	0.75104	0.76023	0.77037
224	0.71635	0.73302	0.74082	0.74341	0.75183	0.76099	0.77111
225	0.71730	0.73387	0.74164	0.74423	0.75263	0.76175	0.77184
226	0.71824	0.73471	0.74246	0.74504	0.75343	0.76251	0.77256
227	0.71909	0.73557	0.74327	0.74583	0.75421	0.76327	0.77327
228	0.71993	0.73641	0.74408	0.74664	0.75498	0.76401	0.77399
229	0.72086	0.73726	0.74489	0.74743	0.75575	0.76474	0.77469
230	0.72173	0.73809	0.74570	0.74824	0.75651	0.76549	0.77540
231	0.72257	0.73889	0.74651	0.74903	0.75727	0.76622	0.77610
232	0.72338	0.73970	0.74730	0.74981	0.75803	0.76695	0.77679
233	0.72429	0.74054	0.74807	0.75058	0.75879	0.76767	0.77748
234	0.72514	0.74133	0.74884	0.75136	0.75953	0.76838	0.77817
235	0.72605	0.74212	0.74961	0.75213	0.76027	0.76909	0.77884
236	0.72693	0.74291	0.75038	0.75289	0.76100	0.76980	0.77952
237	0.72772	0.74368	0.75114	0.75365	0.76173	0.77049	0.78019
238	0.72855	0.74448	0.75192	0.75440	0.76247	0.77119	0.78085
239	0.72937	0.74524	0.75267	0.75514	0.76319	0.77189	0.78151
240	0.73019	0.74601	0.75341	0.75588	0.76390	0.77257	0.78216
241	0.73105	0.74677	0.75414	0.75663	0.76461	0.77325	0.78281
242	0.73183	0.74752	0.75489	0.75736	0.76532	0.77392	0.78346
243	0.73261	0.74829	0.75564	0.75809	0.76601	0.77460	0.78410
244	0.73338	0.74905	0.75635	0.75882	0.76671	0.77527	0.78474
245	0.73420	0.74980	0.75708	0.75954	0.76740	0.77594	0.78537
246	0.73499	0.75056	0.75781	0.76025	0.76809	0.77660	0.78601
247	0.73576	0.75127	0.75854	0.76093	0.76877	0.77725	0.78663
248	0.73655	0.75199	0.75926	0.76165	0.76945	0.77790	0.78725
249	0.73731	0.75270	0.75996	0.76235	0.77012	0.77855	0.78786
250	0.73803	0.75344	0.76063	0.76304	0.77079	0.77919	0.78848
251	0.73881	0.75414	0.76135	0.76372	0.77145	0.77983	0.78909
252	0.73954	0.75487	0.76203	0.76440	0.77212	0.78047	0.78970
253	0.74031	0.75559	0.76272	0.76508	0.77277	0.78110	0.79030
254	0.74108	0.75628	0.76340	0.76575	0.77343	0.78173	0.79090
255	0.74185	0.75698	0.76407	0.76642	0.77407	0.78235	0.79149
256	0.74261	0.75769	0.76474	0.76709	0.77472	0.78297	0.79208
257	0.74335	0.75838	0.76542	0.76777	0.77535	0.78358	0.79267
258	0.74407	0.75907	0.76608	0.76842	0.77599	0.78419	0.79325
259	0.74480	0.75973	0.76673	0.76906	0.77662	0.78480	0.79384

N	0.1%	0.5%	1%	1.25%	2.5%	5%	10%
260	0.74554	0.76041	0.76739	0.76971	0.77724	0.78541	0.79441
261	0.74626	0.76109	0.76806	0.77036	0.77786	0.78600	0.79498
262	0.74700	0.76176	0.76870	0.77101	0.77847	0.78659	0.79555
263	0.74769	0.76242	0.76933	0.77165	0.77909	0.78718	0.79611
264	0.74842	0.76310	0.76998	0.77228	0.77970	0.78777	0.79667
265	0.74914	0.76374	0.77061	0.77290	0.78031	0.78835	0.79723
266	0.74983	0.76441	0.77124	0.77352	0.78092	0.78894	0.79778
267	0.75053	0.76505	0.77187	0.77413	0.78153	0.78951	0.79834
268	0.75120	0.76569	0.77250	0.77475	0.78213	0.79009	0.79888
269	0.75186	0.76633	0.77312	0.77536	0.78272	0.79065	0.79943
270	0.75259	0.76697	0.77375	0.77596	0.78331	0.79123	0.79997
271	0.75324	0.76762	0.77434	0.77657	0.78389	0.79178	0.80050
272	0.75390	0.76826	0.77495	0.77717	0.78448	0.79234	0.80104
273	0.75456	0.76888	0.77555	0.77776	0.78507	0.79290	0.80158
274	0.75520	0.76950	0.77615	0.77837	0.78564	0.79346	0.80210
275	0.75583	0.77010	0.77675	0.77896	0.78621	0.79400	0.80263
276	0.75648	0.77072	0.77735	0.77956	0.78677	0.79455	0.80315
277	0.75716	0.77135	0.77795	0.78017	0.78734	0.79509	0.80367
278	0.75784	0.77197	0.77854	0.78074	0.78790	0.79563	0.80418
279	0.75844	0.77258	0.77914	0.78134	0.78846	0.79617	0.80469
280	0.75914	0.77317	0.77972	0.78192	0.78902	0.79671	0.80520
281	0.75981	0.77377	0.78030	0.78249	0.78957	0.79724	0.80571
282	0.76047	0.77436	0.78089	0.78305	0.79012	0.79777	0.80622
283	0.76108	0.77495	0.78147	0.78363	0.79066	0.79829	0.80672
284	0.76167	0.77554	0.78204	0.78419	0.79121	0.79881	0.80721
285	0.76228	0.77613	0.78262	0.78475	0.79175	0.79932	0.80771
286	0.76294	0.77671	0.78317	0.78531	0.79229	0.79984	0.80821
287	0.76351	0.77729	0.78373	0.78586	0.79283	0.80035	0.80869
288	0.76410	0.77786	0.78427	0.78642	0.79335	0.80086	0.80918
289	0.76472	0.77843	0.78483	0.78697	0.79388	0.80137	0.80966
290	0.76534	0.77902	0.78539	0.78751	0.79441	0.80188	0.81014
291	0.76591	0.77958	0.78593	0.78805	0.79493	0.80238	0.81063
292	0.76650	0.78014	0.78647	0.78859	0.79545	0.80288	0.81110
293	0.76710	0.78069	0.78702	0.78913	0.79596	0.80338	0.81157
294	0.76773	0.78123	0.78755	0.78966	0.79647	0.80387	0.81204
295	0.76832	0.78180	0.78809	0.79020	0.79699	0.80436	0.81251
296	0.76890	0.78236	0.78864	0.79072	0.79749	0.80486	0.81297
297	0.76950	0.78292	0.78916	0.79124	0.79800	0.80534	0.81343
298	0.77005	0.78343	0.78968	0.79176	0.79851	0.80582	0.81389
299	0.77061	0.78397	0.79020	0.79228	0.79900	0.80630	0.81436
300	0.77113	0.78451	0.79072	0.79280	0.79950	0.80677	0.81481
301	0.77177	0.78504	0.79122	0.79330	0.79999	0.80724	0.81526
302	0.77229	0.78555	0.79174	0.79380	0.80048	0.80772	0.81571
303	0.77285	0.78609	0.79225	0.79430	0.80097	0.80818	0.81616
304	0.77342	0.78661	0.79275	0.79480	0.80145	0.80865	0.81660
305	0.77399	0.78713	0.79327	0.79531	0.80193	0.80910	0.81705
306	0.77449	0.78765	0.79377	0.79581	0.80241	0.80957	0.81750
307	0.77506	0.78818	0.79427	0.79631	0.80290	0.81003	0.81793
308	0.77560	0.78869	0.79476	0.79680	0.80337	0.81049	0.81836
309	0.77615	0.78921	0.79526	0.79730	0.80385	0.81095	0.81880
310	0.77667	0.78972	0.79575	0.79779	0.80432	0.81139	0.81923
311	0.77726	0.79021	0.79623	0.79825	0.80478	0.81184	0.81966
312	0.77777	0.79071	0.79673	0.79874	0.80525	0.81229	0.82009
313	0.77833	0.79121	0.79721	0.79921	0.80572	0.81273	0.82051
314	0.77887	0.79170	0.79768	0.79970	0.80618	0.81317	0.82093
315	0.77941	0.79221	0.79816	0.80017	0.80664	0.81361	0.82135
316	0.77992	0.79270	0.79863	0.80063	0.80710	0.81405	0.82177
317	0.78042	0.79318	0.79910	0.80110	0.80754	0.81449	0.82219
318	0.78096	0.79367	0.79957	0.80156	0.80799	0.81493	0.82260
319	0.78149	0.79416	0.80004	0.80203	0.80844	0.81536	0.82301
320	0.78200	0.79464	0.80051	0.80250	0.80889	0.81580	0.82341
321	0.78252	0.79513	0.80099	0.80296	0.80933	0.81622	0.82383
322	0.78303	0.79561	0.80144	0.80342	0.80978	0.81665	0.82423
323	0.78354	0.79608	0.80190	0.80388	0.81021	0.81707	0.82463
324	0.78406	0.79654	0.80236	0.80432	0.81065	0.81749	0.82503
325	0.78456	0.79702	0.80283	0.80477	0.81109	0.81791	0.82543
326	0.78509	0.79749	0.80328	0.80521	0.81151	0.81832	0.82583
327	0.78555	0.79795	0.80375	0.80567	0.81195	0.81874	0.82622
328	0.78605	0.79841	0.80419	0.80612	0.81238	0.81914	0.82661
329	0.78653	0.79887	0.80463	0.80656	0.81280	0.81955	0.82700
330	0.78703	0.79932	0.80507	0.80701	0.81324	0.81995	0.82739
331	0.78756	0.79976	0.80551	0.80744	0.81365	0.82036	0.82777
332	0.78802	0.80021	0.80595	0.80787	0.81407	0.82077	0.82816
333	0.78850	0.80065	0.80639	0.80831	0.81449	0.82117	0.82854

N	0.1%	0.5%	1%	1.25%	2.5%	5%	10%
334	0.78895	0.80111	0.80684	0.80874	0.81491	0.82157	0.82892
335	0.78941	0.80156	0.80727	0.80916	0.81532	0.82196	0.82930
336	0.78990	0.80201	0.80770	0.80959	0.81573	0.82236	0.82968
337	0.79039	0.80245	0.80813	0.81001	0.81614	0.82275	0.83006
338	0.79083	0.80289	0.80855	0.81043	0.81655	0.82314	0.83043
339	0.79129	0.80333	0.80899	0.81086	0.81695	0.82353	0.83080
340	0.79175	0.80377	0.80941	0.81127	0.81735	0.82392	0.83117
341	0.79219	0.80420	0.80982	0.81169	0.81775	0.82431	0.83154
342	0.79270	0.80465	0.81025	0.81211	0.81815	0.82469	0.83191
343	0.79314	0.80508	0.81067	0.81252	0.81856	0.82507	0.83227
344	0.79359	0.80551	0.81107	0.81294	0.81895	0.82545	0.83263
345	0.79407	0.80594	0.81150	0.81334	0.81934	0.82583	0.83299
346	0.79452	0.80636	0.81191	0.81375	0.81973	0.82621	0.83335
347	0.79496	0.80679	0.81231	0.81415	0.82013	0.82658	0.83371
348	0.79540	0.80721	0.81273	0.81455	0.82052	0.82696	0.83406
349	0.79585	0.80762	0.81312	0.81496	0.82091	0.82733	0.83441
350	0.79628	0.80804	0.81353	0.81535	0.82129	0.82770	0.83477
351	0.79674	0.80845	0.81394	0.81575	0.82168	0.82807	0.83512
352	0.79716	0.80886	0.81434	0.81615	0.82205	0.82843	0.83547
353	0.79760	0.80928	0.81474	0.81656	0.82243	0.82880	0.83582
354	0.79803	0.80969	0.81514	0.81695	0.82281	0.82916	0.83616
355	0.79847	0.81011	0.81553	0.81733	0.82319	0.82952	0.83651
356	0.79889	0.81051	0.81592	0.81772	0.82356	0.82988	0.83685
357	0.79930	0.81091	0.81630	0.81810	0.82393	0.83024	0.83719
358	0.79973	0.81131	0.81669	0.81849	0.82430	0.83059	0.83753
359	0.80016	0.81170	0.81708	0.81887	0.82467	0.83095	0.83787
360	0.80058	0.81211	0.81747	0.81926	0.82503	0.83130	0.83820
361	0.80095	0.81250	0.81785	0.81964	0.82540	0.83165	0.83854
362	0.80137	0.81290	0.81824	0.82002	0.82577	0.83200	0.83887
363	0.80179	0.81328	0.81861	0.82038	0.82613	0.83235	0.83920
364	0.80216	0.81366	0.81899	0.82075	0.82649	0.83270	0.83953
365	0.80262	0.81404	0.81935	0.82112	0.82685	0.83304	0.83986
366	0.80304	0.81443	0.81973	0.82149	0.82720	0.83338	0.84019
367	0.80344	0.81482	0.82010	0.82186	0.82755	0.83372	0.84051
368	0.80387	0.81521	0.82046	0.82223	0.82791	0.83406	0.84084
369	0.80429	0.81558	0.82083	0.82259	0.82826	0.83440	0.84116
370	0.80469	0.81596	0.82120	0.82295	0.82861	0.83473	0.84148
371	0.80510	0.81633	0.82157	0.82331	0.82896	0.83507	0.84181
372	0.80548	0.81671	0.82194	0.82367	0.82931	0.83540	0.84212
373	0.80587	0.81710	0.82230	0.82403	0.82965	0.83573	0.84244
374	0.80627	0.81746	0.82266	0.82439	0.83000	0.83607	0.84276
375	0.80667	0.81782	0.82302	0.82475	0.83034	0.83640	0.84308
376	0.80708	0.81819	0.82338	0.82510	0.83068	0.83673	0.84339
377	0.80746	0.81855	0.82374	0.82545	0.83102	0.83705	0.84370
378	0.80787	0.81892	0.82409	0.82580	0.83136	0.83738	0.84401
379	0.80824	0.81928	0.82444	0.82615	0.83170	0.83770	0.84432
380	0.80860	0.81966	0.82480	0.82649	0.83204	0.83802	0.84463
381	0.80898	0.82003	0.82516	0.82683	0.83238	0.83835	0.84494
382	0.80939	0.82039	0.82550	0.82718	0.83271	0.83866	0.84524
383	0.80976	0.82075	0.82584	0.82754	0.83304	0.83898	0.84554
384	0.81012	0.82111	0.82619	0.82787	0.83337	0.83929	0.84584
385	0.81047	0.82145	0.82653	0.82821	0.83369	0.83961	0.84614
386	0.81085	0.82180	0.82688	0.82855	0.83403	0.83992	0.84644
387	0.81125	0.82216	0.82721	0.82889	0.83434	0.84024	0.84674
388	0.81163	0.82250	0.82754	0.82922	0.83466	0.84055	0.84704
389	0.81200	0.82284	0.82789	0.82955	0.83498	0.84086	0.84733
390	0.81235	0.82318	0.82823	0.82989	0.83531	0.84116	0.84763
391	0.81271	0.82353	0.82857	0.83022	0.83563	0.84147	0.84792
392	0.81309	0.82389	0.82890	0.83055	0.83595	0.84178	0.84821
393	0.81344	0.82423	0.82922	0.83088	0.83627	0.84208	0.84850
394	0.81382	0.82457	0.82956	0.83121	0.83658	0.84238	0.84879
395	0.81419	0.82492	0.82990	0.83153	0.83689	0.84268	0.84908
396	0.81453	0.82525	0.83023	0.83186	0.83720	0.84298	0.84936
397	0.81488	0.82560	0.83055	0.83219	0.83751	0.84328	0.84965
398	0.81523	0.82593	0.83087	0.83251	0.83782	0.84358	0.84993
399	0.81560	0.82626	0.83120	0.83283	0.83813	0.84388	0.85022
400	0.81596	0.82660	0.83152	0.83315	0.83844	0.84418	0.85050
401	0.81630	0.82693	0.83183	0.83346	0.83875	0.84447	0.85078
402	0.81667	0.82725	0.83215	0.83378	0.83906	0.84476	0.85106
403	0.81701	0.82757	0.83248	0.83410	0.83936	0.84505	0.85134
404	0.81737	0.82790	0.83279	0.83442	0.83967	0.84534	0.85162
405	0.81771	0.82824	0.83311	0.83473	0.83997	0.84563	0.85189
406	0.81805	0.82857	0.83343	0.83504	0.84027	0.84593	0.85217
407	0.81841	0.82890	0.83374	0.83535	0.84057	0.84621	0.85244

N	0.1%	0.5%	1%	1.25%	2.5%	5%	10%
408	0.81872	0.82922	0.83405	0.83566	0.84087	0.84650	0.85272
409	0.81908	0.82954	0.83436	0.83596	0.84116	0.84678	0.85299
410	0.81941	0.82984	0.83466	0.83627	0.84146	0.84706	0.85326
411	0.81977	0.83016	0.83497	0.83657	0.84175	0.84735	0.85353
412	0.82008	0.83047	0.83527	0.83688	0.84205	0.84762	0.85380
413	0.82043	0.83079	0.83558	0.83718	0.84234	0.84790	0.85406
414	0.82075	0.83110	0.83589	0.83748	0.84263	0.84818	0.85433
415	0.82109	0.83142	0.83619	0.83777	0.84292	0.84846	0.85460
416	0.82140	0.83172	0.83649	0.83806	0.84321	0.84874	0.85486
417	0.82177	0.83204	0.83679	0.83837	0.84349	0.84902	0.85512
418	0.82210	0.83234	0.83709	0.83867	0.84378	0.84929	0.85539
419	0.82243	0.83265	0.83739	0.83896	0.84407	0.84957	0.85565
420	0.82275	0.83295	0.83769	0.83926	0.84435	0.84984	0.85591
421	0.82309	0.83326	0.83799	0.83956	0.84463	0.85010	0.85616
422	0.82343	0.83357	0.83828	0.83984	0.84491	0.85037	0.85642
423	0.82372	0.83387	0.83857	0.84013	0.84519	0.85064	0.85668
424	0.82404	0.83416	0.83886	0.84042	0.84547	0.85091	0.85693
425	0.82434	0.83447	0.83916	0.84071	0.84575	0.85118	0.85719
426	0.82466	0.83477	0.83945	0.84100	0.84603	0.85144	0.85744
427	0.82501	0.83506	0.83973	0.84128	0.84630	0.85171	0.85770
428	0.82532	0.83536	0.84002	0.84156	0.84657	0.85197	0.85795
429	0.82563	0.83566	0.84031	0.84185	0.84684	0.85224	0.85820
430	0.82595	0.83594	0.84059	0.84213	0.84711	0.85250	0.85845
431	0.82626	0.83623	0.84088	0.84241	0.84739	0.85276	0.85870
432	0.82658	0.83653	0.84116	0.84269	0.84765	0.85302	0.85894
433	0.82688	0.83680	0.84145	0.84297	0.84793	0.85327	0.85919
434	0.82718	0.83710	0.84172	0.84325	0.84819	0.85354	0.85944
435	0.82749	0.83739	0.84199	0.84353	0.84846	0.85379	0.85968
436	0.82779	0.83767	0.84228	0.84380	0.84872	0.85405	0.85992
437	0.82810	0.83797	0.84256	0.84408	0.84899	0.85430	0.86017
438	0.82843	0.83825	0.84284	0.84436	0.84925	0.85455	0.86041
439	0.82872	0.83853	0.84311	0.84463	0.84951	0.85481	0.86065
440	0.82903	0.83880	0.84339	0.84489	0.84977	0.85506	0.86089
441	0.82932	0.83908	0.84365	0.84517	0.85003	0.85531	0.86113
442	0.82959	0.83936	0.84393	0.84543	0.85029	0.85556	0.86137
443	0.82990	0.83963	0.84420	0.84570	0.85055	0.85581	0.86161
444	0.83020	0.83992	0.84446	0.84597	0.85081	0.85606	0.86184
445	0.83048	0.84019	0.84473	0.84623	0.85107	0.85630	0.86207
446	0.83079	0.84047	0.84501	0.84650	0.85133	0.85655	0.86231
447	0.83108	0.84074	0.84528	0.84677	0.85158	0.85680	0.86254
448	0.83138	0.84102	0.84554	0.84703	0.85184	0.85704	0.86278
449	0.83169	0.84129	0.84580	0.84729	0.85209	0.85728	0.86301
450	0.83196	0.84156	0.84606	0.84755	0.85234	0.85752	0.86324
451	0.83227	0.84183	0.84633	0.84782	0.85259	0.85776	0.86347
452	0.83255	0.84209	0.84658	0.84808	0.85284	0.85800	0.86370
453	0.83286	0.84236	0.84684	0.84833	0.85309	0.85824	0.86393
454	0.83316	0.84264	0.84710	0.84858	0.85334	0.85848	0.86416
455	0.83342	0.84290	0.84736	0.84883	0.85359	0.85872	0.86438
456	0.83373	0.84316	0.84762	0.84909	0.85384	0.85895	0.86461
457	0.83402	0.84344	0.84788	0.84935	0.85409	0.85919	0.86484
458	0.83428	0.84370	0.84814	0.84961	0.85433	0.85942	0.86506
459	0.83457	0.84395	0.84839	0.84986	0.85457	0.85966	0.86528
460	0.83484	0.84421	0.84864	0.85011	0.85481	0.85989	0.86550
461	0.83512	0.84448	0.84889	0.85036	0.85505	0.86013	0.86573
462	0.83541	0.84474	0.84915	0.85061	0.85529	0.86035	0.86595
463	0.83569	0.84501	0.84941	0.85086	0.85554	0.86059	0.86617
464	0.83596	0.84527	0.84965	0.85110	0.85577	0.86082	0.86639
465	0.83624	0.84553	0.84990	0.85136	0.85602	0.86105	0.86661
466	0.83651	0.84579	0.85015	0.85161	0.85625	0.86127	0.86683
467	0.83678	0.84605	0.85040	0.85186	0.85649	0.86150	0.86704
468	0.83705	0.84630	0.85065	0.85210	0.85672	0.86173	0.86726
469	0.83733	0.84656	0.85089	0.85234	0.85696	0.86195	0.86747
470	0.83759	0.84680	0.85114	0.85259	0.85720	0.86218	0.86769
471	0.83787	0.84706	0.85138	0.85282	0.85743	0.86241	0.86791
472	0.83812	0.84731	0.85163	0.85306	0.85766	0.86263	0.86812
473	0.83839	0.84755	0.85187	0.85329	0.85789	0.86285	0.86833
474	0.83864	0.84780	0.85211	0.85353	0.85812	0.86307	0.86854
475	0.83890	0.84805	0.85235	0.85377	0.85835	0.86329	0.86876
476	0.83915	0.84830	0.85259	0.85400	0.85858	0.86351	0.86897
477	0.83943	0.84854	0.85283	0.85424	0.85881	0.86373	0.86918
478	0.83972	0.84879	0.85306	0.85448	0.85904	0.86395	0.86939
479	0.83998	0.84904	0.85330	0.85471	0.85927	0.86417	0.86959
480	0.84024	0.84929	0.85354	0.85494	0.85949	0.86438	0.86980
481	0.84049	0.84953	0.85377	0.85518	0.85972	0.86460	0.87001

N	0.1%	0.5%	1%	1.25%	2.5%	5%	10%
482	0.84076	0.84976	0.85401	0.85542	0.85994	0.86482	0.87021
483	0.84101	0.84999	0.85424	0.85564	0.86016	0.86503	0.87042
484	0.84127	0.85024	0.85447	0.85587	0.86039	0.86525	0.87062
485	0.84152	0.85048	0.85470	0.85610	0.86061	0.86546	0.87083
486	0.84178	0.85072	0.85494	0.85632	0.86083	0.86567	0.87103
487	0.84201	0.85095	0.85516	0.85656	0.86105	0.86588	0.87123
488	0.84230	0.85119	0.85540	0.85678	0.86127	0.86609	0.87143
489	0.84253	0.85143	0.85563	0.85701	0.86149	0.86630	0.87163
490	0.84277	0.85167	0.85586	0.85724	0.86171	0.86651	0.87183
491	0.84303	0.85191	0.85608	0.85747	0.86192	0.86672	0.87203
492	0.84328	0.85214	0.85630	0.85769	0.86214	0.86693	0.87223
493	0.84351	0.85237	0.85653	0.85791	0.86236	0.86714	0.87243
494	0.84378	0.85261	0.85676	0.85813	0.86258	0.86735	0.87263
495	0.84403	0.85284	0.85698	0.85835	0.86280	0.86755	0.87282
496	0.84429	0.85307	0.85720	0.85857	0.86301	0.86776	0.87302
497	0.84453	0.85331	0.85743	0.85879	0.86322	0.86797	0.87321
498	0.84479	0.85353	0.85765	0.85902	0.86343	0.86817	0.87341
499	0.84503	0.85378	0.85787	0.85924	0.86364	0.86837	0.87360
500	0.84528	0.85400	0.85810	0.85945	0.86385	0.86857	0.87380
501	0.84553	0.85423	0.85831	0.85967	0.86407	0.86877	0.87399
502	0.84578	0.85444	0.85854	0.85989	0.86428	0.86897	0.87418
503	0.84601	0.85467	0.85876	0.86011	0.86449	0.86917	0.87437
504	0.84627	0.85490	0.85898	0.86033	0.86470	0.86938	0.87456
505	0.84650	0.85512	0.85919	0.86054	0.86490	0.86958	0.87476
506	0.84674	0.85535	0.85941	0.86076	0.86511	0.86978	0.87495
507	0.84699	0.85557	0.85963	0.86097	0.86531	0.86998	0.87514
508	0.84724	0.85580	0.85984	0.86118	0.86551	0.87017	0.87532
509	0.84747	0.85601	0.86006	0.86139	0.86572	0.87037	0.87551
510	0.84772	0.85624	0.86027	0.86161	0.86592	0.87056	0.87570
511	0.84794	0.85646	0.86048	0.86182	0.86613	0.87076	0.87589
512	0.84818	0.85668	0.86069	0.86202	0.86633	0.87095	0.87607
513	0.84841	0.85690	0.86091	0.86223	0.86653	0.87115	0.87626
514	0.84863	0.85712	0.86112	0.86244	0.86673	0.87134	0.87644
515	0.84885	0.85733	0.86133	0.86265	0.86693	0.87153	0.87662
516	0.84909	0.85754	0.86153	0.86286	0.86713	0.87173	0.87681
517	0.84931	0.85776	0.86175	0.86307	0.86733	0.87192	0.87699
518	0.84952	0.85797	0.86195	0.86327	0.86753	0.87211	0.87717
519	0.84974	0.85819	0.86215	0.86347	0.86773	0.87230	0.87736
520	0.84996	0.85839	0.86236	0.86368	0.86793	0.87249	0.87754
521	0.85018	0.85862	0.86257	0.86388	0.86812	0.87268	0.87772
522	0.85040	0.85883	0.86278	0.86409	0.86832	0.87287	0.87790
523	0.85064	0.85904	0.86298	0.86429	0.86851	0.87306	0.87808
524	0.85088	0.85925	0.86318	0.86449	0.86871	0.87324	0.87826
525	0.85111	0.85946	0.86338	0.86469	0.86890	0.87343	0.87843
526	0.85132	0.85967	0.86359	0.86489	0.86909	0.87361	0.87861
527	0.85154	0.85988	0.86379	0.86509	0.86928	0.87380	0.87879
528	0.85177	0.86009	0.86398	0.86528	0.86947	0.87398	0.87896
529	0.85199	0.86030	0.86418	0.86547	0.86967	0.87416	0.87914
530	0.85221	0.86051	0.86438	0.86567	0.86986	0.87435	0.87932
531	0.85243	0.86071	0.86458	0.86586	0.87005	0.87453	0.87949
532	0.85262	0.86092	0.86478	0.86606	0.87024	0.87471	0.87966
533	0.85285	0.86113	0.86498	0.86626	0.87043	0.87490	0.87984
534	0.85308	0.86132	0.86517	0.86646	0.87062	0.87507	0.88001
535	0.85329	0.86153	0.86537	0.86665	0.87081	0.87525	0.88018
536	0.85349	0.86175	0.86556	0.86684	0.87100	0.87544	0.88035
537	0.85371	0.86195	0.86576	0.86704	0.87119	0.87562	0.88052
538	0.85390	0.86216	0.86596	0.86724	0.87137	0.87579	0.88070
539	0.85413	0.86236	0.86615	0.86742	0.87155	0.87597	0.88086
540	0.85434	0.86256	0.86634	0.86762	0.87174	0.87615	0.88104
541	0.85456	0.86276	0.86653	0.86780	0.87192	0.87633	0.88121
542	0.85475	0.86296	0.86672	0.86799	0.87211	0.87650	0.88138
543	0.85496	0.86315	0.86691	0.86818	0.87229	0.87668	0.88155
544	0.85517	0.86334	0.86711	0.86837	0.87247	0.87686	0.88171
545	0.85537	0.86354	0.86730	0.86856	0.87265	0.87704	0.88188
546	0.85559	0.86373	0.86749	0.86875	0.87283	0.87721	0.88205
547	0.85579	0.86393	0.86767	0.86893	0.87301	0.87738	0.88221
548	0.85600	0.86412	0.86787	0.86912	0.87319	0.87755	0.88238
549	0.85620	0.86432	0.86805	0.86931	0.87337	0.87773	0.88254
550	0.85643	0.86451	0.86824	0.86949	0.87355	0.87790	0.88271
551	0.85664	0.86471	0.86842	0.86967	0.87373	0.87807	0.88287
552	0.85683	0.86490	0.86861	0.86987	0.87390	0.87824	0.88303
553	0.85703	0.86510	0.86880	0.87005	0.87408	0.87841	0.88320
554	0.85724	0.86528	0.86899	0.87023	0.87426	0.87858	0.88336
555	0.85742	0.86548	0.86917	0.87042	0.87444	0.87876	0.88353

N	0.1%	0.5%	1%	1.25%	2.5%	5%	10%
556	0.85762	0.86567	0.86936	0.87060	0.87461	0.87893	0.88369
557	0.85783	0.86586	0.86955	0.87077	0.87478	0.87909	0.88385
558	0.85804	0.86605	0.86974	0.87095	0.87496	0.87926	0.88401
559	0.85825	0.86625	0.86991	0.87113	0.87513	0.87943	0.88417
560	0.85844	0.86643	0.87010	0.87131	0.87531	0.87960	0.88433
561	0.85864	0.86662	0.87028	0.87150	0.87548	0.87976	0.88449
562	0.85884	0.86681	0.87046	0.87167	0.87565	0.87993	0.88465
563	0.85906	0.86700	0.87064	0.87185	0.87583	0.88010	0.88481
564	0.85924	0.86718	0.87082	0.87204	0.87600	0.88026	0.88496
565	0.85943	0.86737	0.87100	0.87221	0.87617	0.88042	0.88512
566	0.85963	0.86755	0.87117	0.87239	0.87634	0.88059	0.88528
567	0.85982	0.86774	0.87135	0.87257	0.87651	0.88075	0.88543
568	0.86001	0.86793	0.87152	0.87274	0.87668	0.88092	0.88559
569	0.86022	0.86810	0.87170	0.87291	0.87685	0.88108	0.88574
570	0.86042	0.86828	0.87188	0.87309	0.87702	0.88124	0.88590
571	0.86061	0.86846	0.87205	0.87326	0.87718	0.88140	0.88605
572	0.86080	0.86865	0.87224	0.87343	0.87735	0.88157	0.88621
573	0.86099	0.86883	0.87241	0.87361	0.87752	0.88173	0.88636
574	0.86118	0.86901	0.87258	0.87378	0.87769	0.88188	0.88651
575	0.86135	0.86918	0.87276	0.87395	0.87785	0.88204	0.88666
576	0.86152	0.86936	0.87293	0.87412	0.87802	0.88220	0.88682
577	0.86171	0.86953	0.87310	0.87429	0.87818	0.88236	0.88697
578	0.86192	0.86972	0.87328	0.87447	0.87835	0.88252	0.88712
579	0.86209	0.86989	0.87345	0.87464	0.87851	0.88268	0.88727
580	0.86227	0.87007	0.87362	0.87481	0.87867	0.88283	0.88742
581	0.86248	0.87025	0.87379	0.87498	0.87884	0.88299	0.88757
582	0.86267	0.87042	0.87396	0.87514	0.87900	0.88315	0.88772
583	0.86284	0.87059	0.87412	0.87531	0.87915	0.88330	0.88787
584	0.86301	0.87077	0.87430	0.87548	0.87931	0.88346	0.88802
585	0.86319	0.87094	0.87446	0.87564	0.87948	0.88361	0.88817
586	0.86338	0.87111	0.87463	0.87582	0.87964	0.88377	0.88831
587	0.86358	0.87128	0.87480	0.87598	0.87980	0.88392	0.88846
588	0.86376	0.87146	0.87497	0.87615	0.87996	0.88407	0.88861
589	0.86397	0.87163	0.87514	0.87632	0.88012	0.88423	0.88875
590	0.86415	0.87180	0.87530	0.87648	0.88027	0.88438	0.88890
591	0.86432	0.87197	0.87547	0.87664	0.88044	0.88453	0.88904
592	0.86449	0.87214	0.87564	0.87681	0.88059	0.88468	0.88919
593	0.86468	0.87231	0.87581	0.87698	0.88075	0.88483	0.88934
594	0.86485	0.87248	0.87597	0.87714	0.88091	0.88498	0.88948
595	0.86503	0.87266	0.87614	0.87729	0.88107	0.88513	0.88962
596	0.86521	0.87282	0.87630	0.87746	0.88122	0.88528	0.88977
597	0.86538	0.87299	0.87647	0.87762	0.88138	0.88543	0.88991
598	0.86557	0.87315	0.87664	0.87778	0.88153	0.88558	0.89005
599	0.86575	0.87331	0.87680	0.87794	0.88169	0.88573	0.89020
600	0.86592	0.87348	0.87696	0.87810	0.88184	0.88588	0.89034
601	0.86608	0.87365	0.87712	0.87826	0.88199	0.88603	0.89048
602	0.86626	0.87381	0.87728	0.87842	0.88215	0.88618	0.89062
603	0.86644	0.87398	0.87744	0.87858	0.88230	0.88632	0.89076
604	0.86661	0.87414	0.87760	0.87874	0.88245	0.88647	0.89090
605	0.86677	0.87432	0.87775	0.87890	0.88261	0.88661	0.89104
606	0.86695	0.87448	0.87792	0.87905	0.88276	0.88676	0.89118
607	0.86712	0.87464	0.87807	0.87921	0.88291	0.88690	0.89132
608	0.86728	0.87481	0.87823	0.87937	0.88306	0.88705	0.89146
609	0.86746	0.87496	0.87839	0.87952	0.88321	0.88719	0.89160
610	0.86763	0.87513	0.87854	0.87968	0.88336	0.88734	0.89173
611	0.86780	0.87529	0.87870	0.87983	0.88351	0.88748	0.89187
612	0.86797	0.87545	0.87885	0.87999	0.88365	0.88763	0.89200
613	0.86814	0.87561	0.87901	0.88014	0.88381	0.88777	0.89214
614	0.86831	0.87577	0.87916	0.88029	0.88396	0.88791	0.89228
615	0.86849	0.87593	0.87932	0.88045	0.88411	0.88805	0.89241
616	0.86867	0.87608	0.87947	0.88060	0.88425	0.88820	0.89255
617	0.86885	0.87624	0.87963	0.88076	0.88440	0.88834	0.89268
618	0.86900	0.87640	0.87978	0.88091	0.88455	0.88848	0.89282
619	0.86918	0.87656	0.87993	0.88106	0.88469	0.88862	0.89295
620	0.86935	0.87673	0.88009	0.88121	0.88484	0.88876	0.89309
621	0.86953	0.87688	0.88024	0.88136	0.88498	0.88890	0.89322
622	0.86970	0.87704	0.88039	0.88151	0.88513	0.88904	0.89335
623	0.86988	0.87719	0.88054	0.88167	0.88527	0.88918	0.89349
624	0.87004	0.87734	0.88069	0.88181	0.88542	0.88932	0.89362
625	0.87020	0.87749	0.88085	0.88196	0.88556	0.88946	0.89375
626	0.87036	0.87766	0.88100	0.88211	0.88571	0.88960	0.89389
627	0.87053	0.87781	0.88114	0.88226	0.88585	0.88973	0.89402
628	0.87069	0.87796	0.88130	0.88241	0.88599	0.88987	0.89415
629	0.87088	0.87812	0.88145	0.88255	0.88613	0.89001	0.89428

N	0.1%	0.5%	1%	1.25%	2.5%	5%	10%
630	0.87103	0.87827	0.88159	0.88269	0.88627	0.89014	0.89441
631	0.87120	0.87842	0.88173	0.88284	0.88642	0.89028	0.89454
632	0.87137	0.87857	0.88188	0.88299	0.88656	0.89042	0.89467
633	0.87153	0.87872	0.88203	0.88313	0.88670	0.89055	0.89480
634	0.87169	0.87887	0.88217	0.88327	0.88684	0.89069	0.89493
635	0.87185	0.87902	0.88232	0.88342	0.88698	0.89082	0.89506
636	0.87201	0.87917	0.88247	0.88357	0.88712	0.89096	0.89518
637	0.87216	0.87932	0.88262	0.88370	0.88726	0.89109	0.89531
638	0.87233	0.87947	0.88277	0.88385	0.88740	0.89122	0.89544
639	0.87247	0.87962	0.88291	0.88399	0.88754	0.89136	0.89557
640	0.87264	0.87978	0.88306	0.88413	0.88768	0.89149	0.89569
641	0.87279	0.87993	0.88320	0.88427	0.88781	0.89162	0.89582
642	0.87294	0.88006	0.88335	0.88442	0.88795	0.89175	0.89595
643	0.87308	0.88022	0.88348	0.88456	0.88809	0.89188	0.89607
644	0.87325	0.88037	0.88363	0.88470	0.88822	0.89201	0.89620
645	0.87339	0.88051	0.88377	0.88484	0.88836	0.89214	0.89632
646	0.87355	0.88065	0.88391	0.88498	0.88850	0.89227	0.89645
647	0.87372	0.88081	0.88405	0.88513	0.88863	0.89240	0.89657
648	0.87389	0.88095	0.88419	0.88527	0.88876	0.89253	0.89670
649	0.87403	0.88110	0.88433	0.88541	0.88890	0.89266	0.89682
650	0.87419	0.88124	0.88448	0.88555	0.88903	0.89279	0.89694
651	0.87435	0.88139	0.88462	0.88569	0.88916	0.89292	0.89707
652	0.87450	0.88153	0.88475	0.88583	0.88930	0.89305	0.89719
653	0.87464	0.88167	0.88490	0.88596	0.88944	0.89318	0.89731
654	0.87480	0.88181	0.88503	0.88610	0.88957	0.89331	0.89743
655	0.87496	0.88195	0.88517	0.88624	0.88970	0.89344	0.89756
656	0.87512	0.88209	0.88531	0.88637	0.88983	0.89357	0.89768
657	0.87528	0.88224	0.88545	0.88651	0.88996	0.89369	0.89780
658	0.87541	0.88238	0.88559	0.88664	0.89010	0.89382	0.89792
659	0.87559	0.88252	0.88573	0.88678	0.89022	0.89395	0.89804
660	0.87574	0.88266	0.88586	0.88692	0.89035	0.89407	0.89816
661	0.87589	0.88280	0.88600	0.88706	0.89049	0.89420	0.89828
662	0.87604	0.88294	0.88613	0.88719	0.89062	0.89432	0.89840
663	0.87618	0.88308	0.88627	0.88732	0.89075	0.89445	0.89852
664	0.87633	0.88323	0.88641	0.88746	0.89088	0.89458	0.89864
665	0.87648	0.88336	0.88654	0.88759	0.89101	0.89470	0.89876
666	0.87662	0.88350	0.88667	0.88772	0.89114	0.89482	0.89888
667	0.87678	0.88364	0.88681	0.88786	0.89127	0.89494	0.89900
668	0.87692	0.88377	0.88694	0.88799	0.89139	0.89507	0.89912
669	0.87707	0.88391	0.88708	0.88812	0.89151	0.89519	0.89923
670	0.87721	0.88404	0.88721	0.88825	0.89164	0.89531	0.89935
671	0.87736	0.88419	0.88734	0.88838	0.89177	0.89543	0.89947
672	0.87749	0.88433	0.88748	0.88852	0.89190	0.89556	0.89958
673	0.87764	0.88446	0.88761	0.88865	0.89202	0.89568	0.89970
674	0.87779	0.88460	0.88774	0.88878	0.89215	0.89580	0.89981
675	0.87793	0.88473	0.88787	0.88891	0.89227	0.89592	0.89993
676	0.87807	0.88486	0.88800	0.88904	0.89240	0.89604	0.90005
677	0.87823	0.88500	0.88813	0.88917	0.89252	0.89616	0.90016
678	0.87836	0.88515	0.88827	0.88930	0.89265	0.89628	0.90028
679	0.87852	0.88529	0.88840	0.88943	0.89277	0.89640	0.90039
680	0.87865	0.88542	0.88853	0.88956	0.89290	0.89652	0.90051
681	0.87880	0.88555	0.88866	0.88968	0.89302	0.89664	0.90062
682	0.87895	0.88569	0.88879	0.88981	0.89314	0.89676	0.90073
683	0.87907	0.88582	0.88891	0.88994	0.89327	0.89688	0.90085
684	0.87921	0.88595	0.88904	0.89006	0.89339	0.89699	0.90096
685	0.87935	0.88608	0.88917	0.89019	0.89351	0.89711	0.90107
686	0.87950	0.88621	0.88929	0.89032	0.89364	0.89723	0.90119
687	0.87965	0.88635	0.88942	0.89044	0.89376	0.89734	0.90130
688	0.87978	0.88647	0.88955	0.89057	0.89388	0.89746	0.90141
689	0.87993	0.88661	0.88967	0.89069	0.89401	0.89758	0.90152
690	0.88007	0.88673	0.88980	0.89082	0.89413	0.89770	0.90163
691	0.88020	0.88687	0.88993	0.89094	0.89425	0.89781	0.90174
692	0.88035	0.88700	0.89005	0.89107	0.89437	0.89793	0.90185
693	0.88050	0.88713	0.89018	0.89120	0.89449	0.89805	0.90196
694	0.88064	0.88725	0.89031	0.89132	0.89461	0.89816	0.90207
695	0.88078	0.88738	0.89043	0.89145	0.89473	0.89827	0.90219
696	0.88092	0.88751	0.89056	0.89157	0.89484	0.89839	0.90230
697	0.88105	0.88763	0.89068	0.89169	0.89497	0.89851	0.90241
698	0.88118	0.88777	0.89081	0.89182	0.89508	0.89862	0.90252
699	0.88134	0.88789	0.89093	0.89194	0.89520	0.89873	0.90263
700	0.88146	0.88802	0.89105	0.89206	0.89532	0.89885	0.90273
701	0.88160	0.88815	0.89118	0.89218	0.89544	0.89896	0.90284
702	0.88174	0.88827	0.89130	0.89231	0.89556	0.89907	0.90295
703	0.88187	0.88840	0.89142	0.89243	0.89567	0.89918	0.90306

N	0.1%	0.5%	1%	1.25%	2.5%	5%	10%
704	0.88201	0.88853	0.89154	0.89255	0.89579	0.89930	0.90317
705	0.88215	0.88866	0.89167	0.89267	0.89591	0.89941	0.90327
706	0.88228	0.88878	0.89179	0.89280	0.89602	0.89952	0.90338
707	0.88241	0.88891	0.89192	0.89292	0.89614	0.89964	0.90349
708	0.88255	0.88903	0.89203	0.89304	0.89626	0.89974	0.90359
709	0.88268	0.88916	0.89216	0.89316	0.89637	0.89985	0.90370
710	0.88281	0.88928	0.89227	0.89328	0.89649	0.89997	0.90380
711	0.88293	0.88940	0.89239	0.89339	0.89660	0.90008	0.90391
712	0.88306	0.88953	0.89251	0.89351	0.89672	0.90019	0.90401
713	0.88320	0.88966	0.89263	0.89362	0.89683	0.90030	0.90412
714	0.88331	0.88977	0.89275	0.89374	0.89694	0.90041	0.90422
715	0.88345	0.88990	0.89287	0.89386	0.89706	0.90052	0.90433
716	0.88357	0.89002	0.89298	0.89398	0.89717	0.90063	0.90443
717	0.88371	0.89014	0.89310	0.89410	0.89729	0.90074	0.90454
718	0.88384	0.89026	0.89322	0.89421	0.89740	0.90085	0.90464
719	0.88398	0.89039	0.89333	0.89433	0.89751	0.90096	0.90475
720	0.88410	0.89051	0.89345	0.89445	0.89763	0.90107	0.90485
721	0.88423	0.89063	0.89357	0.89457	0.89774	0.90117	0.90496
722	0.88436	0.89075	0.89369	0.89468	0.89785	0.90128	0.90506
723	0.88450	0.89087	0.89381	0.89480	0.89796	0.90139	0.90516
724	0.88462	0.89099	0.89392	0.89491	0.89807	0.90149	0.90526
725	0.88474	0.89111	0.89404	0.89503	0.89819	0.90160	0.90537
726	0.88487	0.89123	0.89416	0.89514	0.89830	0.90171	0.90547
727	0.88500	0.89135	0.89427	0.89525	0.89841	0.90181	0.90557
728	0.88513	0.89147	0.89439	0.89536	0.89851	0.90192	0.90567
729	0.88527	0.89159	0.89451	0.89548	0.89863	0.90203	0.90577
730	0.88540	0.89171	0.89462	0.89559	0.89874	0.90213	0.90587
731	0.88552	0.89183	0.89474	0.89571	0.89885	0.90224	0.90597
732	0.88564	0.89195	0.89486	0.89582	0.89896	0.90234	0.90607
733	0.88578	0.89206	0.89497	0.89593	0.89907	0.90245	0.90618
734	0.88591	0.89218	0.89508	0.89605	0.89917	0.90255	0.90628
735	0.88603	0.89230	0.89520	0.89616	0.89928	0.90266	0.90638
736	0.88615	0.89242	0.89531	0.89627	0.89939	0.90276	0.90648
737	0.88628	0.89254	0.89542	0.89639	0.89950	0.90286	0.90658
738	0.88639	0.89265	0.89554	0.89650	0.89961	0.90297	0.90668
739	0.88651	0.89277	0.89565	0.89661	0.89971	0.90307	0.90677
740	0.88663	0.89289	0.89576	0.89672	0.89982	0.90317	0.90687
741	0.88676	0.89300	0.89587	0.89683	0.89993	0.90328	0.90697
742	0.88687	0.89311	0.89598	0.89694	0.90004	0.90338	0.90707
743	0.88701	0.89323	0.89610	0.89705	0.90015	0.90348	0.90717
744	0.88713	0.89334	0.89621	0.89716	0.90025	0.90359	0.90727
745	0.88725	0.89346	0.89632	0.89727	0.90035	0.90369	0.90736
746	0.88737	0.89357	0.89643	0.89738	0.90046	0.90379	0.90746
747	0.88750	0.89369	0.89654	0.89749	0.90057	0.90389	0.90756
748	0.88763	0.89380	0.89666	0.89760	0.90067	0.90399	0.90765
749	0.88775	0.89392	0.89676	0.89771	0.90077	0.90409	0.90775
750	0.88786	0.89403	0.89687	0.89782	0.90088	0.90419	0.90785
751	0.88798	0.89415	0.89698	0.89793	0.90098	0.90429	0.90794
752	0.88809	0.89426	0.89709	0.89804	0.90109	0.90439	0.90804
753	0.88823	0.89437	0.89720	0.89815	0.90120	0.90449	0.90814
754	0.88835	0.89447	0.89730	0.89825	0.90130	0.90460	0.90823
755	0.88846	0.89458	0.89741	0.89836	0.90141	0.90469	0.90833
756	0.88858	0.89469	0.89753	0.89847	0.90151	0.90479	0.90842
757	0.88870	0.89481	0.89763	0.89857	0.90161	0.90490	0.90852
758	0.88882	0.89492	0.89774	0.89868	0.90171	0.90499	0.90861
759	0.88892	0.89503	0.89784	0.89879	0.90182	0.90509	0.90871
760	0.88905	0.89514	0.89795	0.89890	0.90192	0.90519	0.90880
761	0.88917	0.89526	0.89806	0.89900	0.90202	0.90529	0.90890
762	0.88929	0.89536	0.89817	0.89911	0.90212	0.90539	0.90899
763	0.88940	0.89547	0.89827	0.89921	0.90222	0.90549	0.90908
764	0.88952	0.89558	0.89838	0.89932	0.90232	0.90558	0.90918
765	0.88963	0.89568	0.89848	0.89942	0.90242	0.90568	0.90927
766	0.88975	0.89579	0.89859	0.89952	0.90253	0.90578	0.90936
767	0.88985	0.89590	0.89869	0.89962	0.90263	0.90588	0.90946
768	0.88998	0.89601	0.89880	0.89973	0.90273	0.90597	0.90955
769	0.89010	0.89613	0.89890	0.89983	0.90283	0.90607	0.90964
770	0.89021	0.89623	0.89901	0.89994	0.90293	0.90617	0.90974
771	0.89033	0.89634	0.89911	0.90003	0.90303	0.90626	0.90983
772	0.89044	0.89644	0.89922	0.90014	0.90313	0.90636	0.90992
773	0.89055	0.89655	0.89932	0.90024	0.90323	0.90645	0.91001
774	0.89067	0.89665	0.89943	0.90035	0.90333	0.90654	0.91010
775	0.89077	0.89676	0.89953	0.90045	0.90343	0.90664	0.91019
776	0.89089	0.89687	0.89963	0.90055	0.90352	0.90674	0.91028
777	0.89101	0.89698	0.89973	0.90065	0.90362	0.90683	0.91038

N	0.1%	0.5%	1%	1.25%	2.5%	5%	10%
778	0.89112	0.89709	0.89984	0.90076	0.90372	0.90693	0.91047
779	0.89124	0.89719	0.89994	0.90086	0.90382	0.90702	0.91056
780	0.89135	0.89729	0.90004	0.90096	0.90392	0.90711	0.91065
781	0.89146	0.89741	0.90014	0.90106	0.90401	0.90721	0.91074
782	0.89157	0.89751	0.90025	0.90116	0.90411	0.90730	0.91082
783	0.89167	0.89761	0.90035	0.90126	0.90421	0.90740	0.91091
784	0.89179	0.89772	0.90045	0.90136	0.90431	0.90749	0.91100
785	0.89189	0.89782	0.90055	0.90147	0.90441	0.90758	0.91109
786	0.89199	0.89792	0.90065	0.90157	0.90450	0.90768	0.91118
787	0.89211	0.89803	0.90075	0.90167	0.90460	0.90777	0.91127
788	0.89223	0.89813	0.90085	0.90177	0.90469	0.90786	0.91136
789	0.89233	0.89823	0.90095	0.90186	0.90479	0.90795	0.91145
790	0.89243	0.89833	0.90105	0.90196	0.90488	0.90805	0.91154
791	0.89255	0.89843	0.90115	0.90206	0.90498	0.90814	0.91162
792	0.89265	0.89853	0.90125	0.90216	0.90507	0.90823	0.91171
793	0.89276	0.89865	0.90135	0.90226	0.90517	0.90832	0.91180
794	0.89286	0.89875	0.90145	0.90236	0.90526	0.90841	0.91189
795	0.89298	0.89885	0.90155	0.90246	0.90536	0.90851	0.91197
796	0.89308	0.89895	0.90165	0.90255	0.90545	0.90860	0.91206
797	0.89318	0.89906	0.90175	0.90265	0.90555	0.90869	0.91215
798	0.89330	0.89916	0.90184	0.90275	0.90564	0.90878	0.91224
799	0.89340	0.89925	0.90194	0.90285	0.90574	0.90887	0.91232
800	0.89350	0.89935	0.90204	0.90294	0.90583	0.90896	0.91241
801	0.89362	0.89946	0.90214	0.90304	0.90592	0.90905	0.91249
802	0.89372	0.89956	0.90224	0.90313	0.90601	0.90914	0.91258
803	0.89383	0.89966	0.90233	0.90323	0.90611	0.90922	0.91267
804	0.89394	0.89976	0.90243	0.90333	0.90620	0.90931	0.91275
805	0.89404	0.89986	0.90253	0.90342	0.90630	0.90940	0.91284
806	0.89416	0.89996	0.90263	0.90352	0.90639	0.90949	0.91292
807	0.89426	0.90006	0.90273	0.90361	0.90648	0.90958	0.91301
808	0.89436	0.90016	0.90282	0.90371	0.90657	0.90967	0.91309
809	0.89447	0.90026	0.90292	0.90381	0.90666	0.90976	0.91317
810	0.89458	0.90036	0.90301	0.90390	0.90676	0.90985	0.91326
811	0.89469	0.90046	0.90311	0.90400	0.90685	0.90994	0.91334
812	0.89479	0.90056	0.90320	0.90409	0.90694	0.91003	0.91343
813	0.89489	0.90066	0.90330	0.90418	0.90703	0.91011	0.91351
814	0.89499	0.90075	0.90340	0.90427	0.90712	0.91020	0.91359
815	0.89509	0.90085	0.90349	0.90437	0.90721	0.91029	0.91368
816	0.89520	0.90094	0.90359	0.90446	0.90730	0.91037	0.91376
817	0.89531	0.90104	0.90368	0.90456	0.90739	0.91046	0.91384
818	0.89541	0.90114	0.90378	0.90465	0.90748	0.91055	0.91393
819	0.89551	0.90123	0.90387	0.90475	0.90758	0.91063	0.91401
820	0.89562	0.90133	0.90396	0.90484	0.90766	0.91072	0.91409
821	0.89572	0.90142	0.90406	0.90493	0.90775	0.91080	0.91418
822	0.89583	0.90151	0.90415	0.90502	0.90784	0.91089	0.91426
823	0.89593	0.90161	0.90425	0.90511	0.90793	0.91098	0.91434
824	0.89603	0.90171	0.90434	0.90521	0.90802	0.91106	0.91442
825	0.89612	0.90180	0.90443	0.90530	0.90811	0.91115	0.91450
826	0.89622	0.90190	0.90452	0.90539	0.90820	0.91123	0.91458
827	0.89633	0.90200	0.90461	0.90548	0.90829	0.91132	0.91467
828	0.89643	0.90210	0.90470	0.90557	0.90837	0.91140	0.91475
829	0.89653	0.90219	0.90479	0.90566	0.90846	0.91149	0.91483
830	0.89663	0.90229	0.90489	0.90575	0.90855	0.91157	0.91491
831	0.89674	0.90238	0.90498	0.90584	0.90864	0.91166	0.91499
832	0.89684	0.90247	0.90507	0.90593	0.90872	0.91174	0.91507
833	0.89694	0.90256	0.90516	0.90603	0.90881	0.91183	0.91515
834	0.89704	0.90266	0.90525	0.90612	0.90890	0.91191	0.91523
835	0.89715	0.90275	0.90534	0.90621	0.90898	0.91199	0.91531
836	0.89725	0.90285	0.90544	0.90630	0.90907	0.91208	0.91539
837	0.89735	0.90294	0.90553	0.90638	0.90916	0.91216	0.91547
838	0.89745	0.90303	0.90561	0.90647	0.90924	0.91225	0.91555
839	0.89754	0.90313	0.90570	0.90657	0.90933	0.91233	0.91563
840	0.89764	0.90322	0.90579	0.90665	0.90942	0.91241	0.91571
841	0.89774	0.90331	0.90589	0.90674	0.90950	0.91249	0.91579
842	0.89784	0.90341	0.90597	0.90683	0.90959	0.91258	0.91587
843	0.89793	0.90350	0.90607	0.90692	0.90967	0.91266	0.91595
844	0.89805	0.90359	0.90615	0.90701	0.90976	0.91274	0.91603
845	0.89813	0.90369	0.90624	0.90710	0.90985	0.91282	0.91611
846	0.89823	0.90378	0.90633	0.90719	0.90993	0.91291	0.91618
847	0.89832	0.90387	0.90642	0.90727	0.91002	0.91299	0.91626
848	0.89842	0.90396	0.90651	0.90736	0.91010	0.91307	0.91634
849	0.89852	0.90405	0.90660	0.90745	0.91019	0.91315	0.91642
850	0.89862	0.90414	0.90669	0.90753	0.91027	0.91323	0.91649
851	0.89871	0.90423	0.90677	0.90762	0.91035	0.91331	0.91657

N	0.1%	0.5%	1%	1.25%	2.5%	5%	10%
852	0.89881	0.90431	0.90686	0.90771	0.91044	0.91339	0.91665
853	0.89890	0.90440	0.90695	0.90780	0.91052	0.91347	0.91673
854	0.89900	0.90450	0.90704	0.90788	0.91061	0.91355	0.91680
855	0.89910	0.90459	0.90713	0.90797	0.91069	0.91364	0.91688
856	0.89918	0.90468	0.90721	0.90806	0.91078	0.91372	0.91696
857	0.89928	0.90476	0.90730	0.90814	0.91086	0.91380	0.91703
858	0.89938	0.90486	0.90739	0.90823	0.91094	0.91388	0.91711
859	0.89947	0.90495	0.90748	0.90831	0.91102	0.91395	0.91719
860	0.89955	0.90504	0.90756	0.90840	0.91111	0.91404	0.91726
861	0.89965	0.90513	0.90765	0.90849	0.91119	0.91411	0.91734
862	0.89975	0.90522	0.90774	0.90857	0.91127	0.91419	0.91741
863	0.89985	0.90531	0.90782	0.90866	0.91136	0.91427	0.91749
864	0.89994	0.90540	0.90790	0.90874	0.91144	0.91435	0.91757
865	0.90003	0.90548	0.90799	0.90883	0.91152	0.91443	0.91764
866	0.90012	0.90557	0.90808	0.90891	0.91161	0.91451	0.91772
867	0.90022	0.90565	0.90816	0.90900	0.91169	0.91459	0.91779
868	0.90032	0.90574	0.90824	0.90908	0.91177	0.91467	0.91787
869	0.90040	0.90583	0.90833	0.90916	0.91185	0.91475	0.91794
870	0.90050	0.90592	0.90842	0.90925	0.91193	0.91482	0.91802
871	0.90058	0.90601	0.90850	0.90933	0.91201	0.91490	0.91809
872	0.90068	0.90609	0.90858	0.90942	0.91209	0.91498	0.91817
873	0.90077	0.90617	0.90867	0.90950	0.91217	0.91505	0.91824
874	0.90086	0.90626	0.90875	0.90958	0.91225	0.91513	0.91832
875	0.90095	0.90635	0.90883	0.90966	0.91233	0.91521	0.91839
876	0.90106	0.90644	0.90892	0.90975	0.91241	0.91529	0.91846
877	0.90114	0.90652	0.90900	0.90983	0.91250	0.91536	0.91854
878	0.90124	0.90661	0.90908	0.90991	0.91257	0.91544	0.91861
879	0.90133	0.90669	0.90917	0.90999	0.91265	0.91552	0.91868
880	0.90142	0.90677	0.90925	0.91008	0.91273	0.91560	0.91876
881	0.90151	0.90686	0.90933	0.91016	0.91281	0.91567	0.91883
882	0.90160	0.90694	0.90941	0.91024	0.91289	0.91575	0.91890
883	0.90169	0.90703	0.90950	0.91032	0.91297	0.91582	0.91898
884	0.90178	0.90712	0.90958	0.91040	0.91305	0.91590	0.91905
885	0.90186	0.90721	0.90967	0.91048	0.91312	0.91597	0.91912
886	0.90195	0.90729	0.90975	0.91056	0.91320	0.91605	0.91919
887	0.90204	0.90737	0.90983	0.91065	0.91328	0.91613	0.91927
888	0.90213	0.90746	0.90991	0.91073	0.91336	0.91620	0.91934
889	0.90222	0.90754	0.90999	0.91081	0.91344	0.91628	0.91941
890	0.90231	0.90762	0.91008	0.91089	0.91352	0.91635	0.91948
891	0.90239	0.90771	0.91015	0.91097	0.91359	0.91643	0.91955
892	0.90249	0.90779	0.91024	0.91105	0.91367	0.91650	0.91963
893	0.90257	0.90788	0.91031	0.91113	0.91375	0.91658	0.91970
894	0.90266	0.90796	0.91039	0.91121	0.91382	0.91665	0.91977
895	0.90274	0.90805	0.91048	0.91129	0.91390	0.91672	0.91984
896	0.90283	0.90813	0.91056	0.91137	0.91398	0.91680	0.91991
897	0.90291	0.90821	0.91064	0.91145	0.91406	0.91687	0.91998
898	0.90301	0.90829	0.91072	0.91153	0.91413	0.91695	0.92005
899	0.90310	0.90837	0.91080	0.91161	0.91421	0.91702	0.92012
900	0.90319	0.90845	0.91088	0.91169	0.91428	0.91709	0.92019
901	0.90328	0.90854	0.91096	0.91177	0.91436	0.91717	0.92026
902	0.90337	0.90862	0.91104	0.91185	0.91444	0.91724	0.92033
903	0.90345	0.90870	0.91112	0.91193	0.91451	0.91731	0.92040
904	0.90354	0.90878	0.91120	0.91201	0.91459	0.91739	0.92047
905	0.90362	0.90886	0.91128	0.91208	0.91467	0.91746	0.92054
906	0.90371	0.90894	0.91136	0.91216	0.91474	0.91753	0.92061
907	0.90380	0.90902	0.91143	0.91224	0.91482	0.91760	0.92068
908	0.90389	0.90910	0.91152	0.91232	0.91489	0.91768	0.92075
909	0.90397	0.90918	0.91159	0.91239	0.91497	0.91775	0.92082
910	0.90405	0.90926	0.91167	0.91247	0.91504	0.91782	0.92089
911	0.90413	0.90934	0.91175	0.91255	0.91512	0.91789	0.92096
912	0.90423	0.90942	0.91183	0.91263	0.91519	0.91797	0.92103
913	0.90432	0.90950	0.91191	0.91270	0.91527	0.91804	0.92110
914	0.90441	0.90958	0.91198	0.91278	0.91534	0.91811	0.92116
915	0.90449	0.90966	0.91206	0.91285	0.91541	0.91818	0.92123
916	0.90458	0.90975	0.91214	0.91293	0.91549	0.91825	0.92130
917	0.90465	0.90982	0.91221	0.91301	0.91556	0.91832	0.92137
918	0.90473	0.90989	0.91229	0.91308	0.91563	0.91839	0.92144
919	0.90481	0.90998	0.91236	0.91316	0.91571	0.91846	0.92151
920	0.90490	0.91006	0.91244	0.91324	0.91578	0.91853	0.92157
921	0.90499	0.91014	0.91252	0.91331	0.91585	0.91860	0.92164
922	0.90508	0.91022	0.91260	0.91339	0.91593	0.91867	0.92171
923	0.90517	0.91030	0.91267	0.91346	0.91600	0.91874	0.92177
924	0.90525	0.91038	0.91275	0.91354	0.91607	0.91881	0.92184
925	0.90533	0.91046	0.91282	0.91362	0.91615	0.91888	0.92191

N	0.1%	0.5%	1%	1.25%	2.5%	5%	10%
926	0.90542	0.91054	0.91290	0.91369	0.91622	0.91895	0.92198
927	0.90551	0.91061	0.91298	0.91376	0.91630	0.91902	0.92204
928	0.90559	0.91069	0.91305	0.91384	0.91637	0.91909	0.92211
929	0.90566	0.91078	0.91313	0.91391	0.91644	0.91916	0.92218
930	0.90575	0.91085	0.91320	0.91398	0.91651	0.91923	0.92224
931	0.90583	0.91093	0.91328	0.91406	0.91658	0.91930	0.92231
932	0.90591	0.91101	0.91335	0.91413	0.91666	0.91937	0.92237
933	0.90600	0.91108	0.91342	0.91421	0.91673	0.91944	0.92244
934	0.90609	0.91116	0.91350	0.91428	0.91680	0.91951	0.92251
935	0.90617	0.91123	0.91357	0.91435	0.91687	0.91958	0.92257
936	0.90626	0.91131	0.91365	0.91443	0.91694	0.91965	0.92264
937	0.90633	0.91139	0.91372	0.91450	0.91701	0.91972	0.92271
938	0.90640	0.91147	0.91380	0.91458	0.91708	0.91978	0.92277
939	0.90648	0.91155	0.91387	0.91465	0.91715	0.91985	0.92284
940	0.90656	0.91162	0.91395	0.91473	0.91722	0.91992	0.92290
941	0.90664	0.91170	0.91402	0.91480	0.91729	0.91999	0.92296
942	0.90673	0.91177	0.91410	0.91487	0.91736	0.92005	0.92303
943	0.90681	0.91185	0.91417	0.91495	0.91744	0.92012	0.92309
944	0.90689	0.91192	0.91424	0.91502	0.91751	0.92019	0.92316
945	0.90697	0.91200	0.91431	0.91509	0.91758	0.92026	0.92322
946	0.90705	0.91207	0.91439	0.91516	0.91765	0.92032	0.92329
947	0.90712	0.91214	0.91446	0.91524	0.91771	0.92039	0.92335
948	0.90721	0.91222	0.91454	0.91531	0.91778	0.92046	0.92342
949	0.90729	0.91230	0.91461	0.91538	0.91785	0.92053	0.92348
950	0.90736	0.91237	0.91468	0.91545	0.91792	0.92059	0.92354
951	0.90745	0.91245	0.91475	0.91552	0.91799	0.92066	0.92361
952	0.90752	0.91252	0.91482	0.91559	0.91806	0.92073	0.92367
953	0.90761	0.91260	0.91490	0.91566	0.91813	0.92079	0.92374
954	0.90769	0.91267	0.91497	0.91573	0.91820	0.92086	0.92380
955	0.90777	0.91275	0.91504	0.91580	0.91827	0.92093	0.92386
956	0.90784	0.91282	0.91511	0.91587	0.91834	0.92099	0.92393
957	0.90791	0.91289	0.91518	0.91594	0.91841	0.92106	0.92399
958	0.90799	0.91296	0.91526	0.91601	0.91847	0.92112	0.92405
959	0.90807	0.91304	0.91532	0.91608	0.91854	0.92119	0.92412
960	0.90815	0.91311	0.91540	0.91615	0.91861	0.92126	0.92418
961	0.90822	0.91318	0.91547	0.91622	0.91868	0.92132	0.92424
962	0.90830	0.91326	0.91554	0.91629	0.91875	0.92139	0.92430
963	0.90838	0.91333	0.91561	0.91637	0.91882	0.92145	0.92437
964	0.90846	0.91340	0.91568	0.91644	0.91888	0.92152	0.92443
965	0.90854	0.91348	0.91575	0.91651	0.91895	0.92158	0.92449
966	0.90862	0.91355	0.91582	0.91657	0.91902	0.92165	0.92455
967	0.90870	0.91362	0.91589	0.91665	0.91908	0.92171	0.92461
968	0.90878	0.91369	0.91596	0.91672	0.91915	0.92178	0.92468
969	0.90885	0.91376	0.91603	0.91678	0.91922	0.92184	0.92474
970	0.90893	0.91384	0.91610	0.91685	0.91928	0.92191	0.92480
971	0.90902	0.91391	0.91617	0.91692	0.91935	0.92197	0.92486
972	0.90909	0.91398	0.91624	0.91699	0.91942	0.92203	0.92492
973	0.90917	0.91405	0.91631	0.91706	0.91948	0.92210	0.92498
974	0.90925	0.91412	0.91638	0.91713	0.91955	0.92216	0.92505
975	0.90932	0.91419	0.91645	0.91720	0.91962	0.92223	0.92511
976	0.90940	0.91426	0.91652	0.91727	0.91968	0.92229	0.92517
977	0.90948	0.91433	0.91659	0.91733	0.91975	0.92235	0.92523
978	0.90955	0.91440	0.91666	0.91740	0.91981	0.92242	0.92529
979	0.90962	0.91447	0.91672	0.91747	0.91988	0.92248	0.92535
980	0.90969	0.91454	0.91679	0.91754	0.91994	0.92254	0.92541
981	0.90976	0.91462	0.91686	0.91761	0.92001	0.92261	0.92547
982	0.90984	0.91469	0.91693	0.91767	0.92008	0.92267	0.92553
983	0.90993	0.91476	0.91700	0.91774	0.92014	0.92274	0.92559
984	0.91000	0.91483	0.91706	0.91781	0.92021	0.92280	0.92565
985	0.91008	0.91490	0.91713	0.91787	0.92027	0.92286	0.92571
986	0.91016	0.91497	0.91720	0.91794	0.92034	0.92292	0.92577
987	0.91023	0.91504	0.91727	0.91801	0.92040	0.92298	0.92583
988	0.91031	0.91511	0.91734	0.91808	0.92047	0.92305	0.92589
989	0.91037	0.91517	0.91741	0.91815	0.92053	0.92311	0.92595
990	0.91045	0.91525	0.91747	0.91821	0.92060	0.92317	0.92601
991	0.91053	0.91532	0.91754	0.91828	0.92066	0.92323	0.92607
992	0.91059	0.91539	0.91761	0.91835	0.92073	0.92329	0.92613
993	0.91067	0.91545	0.91768	0.91841	0.92079	0.92336	0.92619
994	0.91074	0.91552	0.91774	0.91848	0.92086	0.92342	0.92625
995	0.91082	0.91559	0.91781	0.91855	0.92092	0.92348	0.92631
996	0.91090	0.91565	0.91787	0.91861	0.92099	0.92354	0.92637
997	0.91098	0.91572	0.91794	0.91868	0.92105	0.92360	0.92643
998	0.91104	0.91579	0.91801	0.91874	0.92111	0.92366	0.92648
999	0.91112	0.91586	0.91808	0.91881	0.92118	0.92373	0.92654

N	0.1%	0.5%	1%	1.25%	2.5%	5%	10%
1000	0.91119	0.91593	0.91814	0.91888	0.92124	0.92379	0.92660

Table 3 – *GM8(6H2L or 2H6L)*. Critical values of upper significance levels for the Grubbs multiple-8 outlier test, applicable in situations where the six highest and two lowest or two highest and six lowest observations are simultaneously identified as outliers, denoted as *GM8(6H2L)* and *GM8(2H6L)*, respectively.

N	0.1%	0.5%	1%	1.25%	2.5%	5%	10%
10	0.00000	0.00000	0.00000	0.00000	0.00000	0.00002	0.00007
11	0.00001	0.00006	0.00012	0.00016	0.00033	0.00069	0.00150
12	0.00019	0.00058	0.00095	0.00112	0.00186	0.00315	0.00550
13	0.00086	0.00204	0.00300	0.00341	0.00508	0.00769	0.01193
14	0.00231	0.00470	0.00646	0.00717	0.00997	0.01408	0.02031
15	0.00467	0.00859	0.01126	0.01232	0.01635	0.02203	0.03017
16	0.00802	0.01363	0.01727	0.01868	0.02397	0.03112	0.04112
17	0.01234	0.01970	0.02434	0.02609	0.03258	0.04116	0.05284
18	0.01739	0.02663	0.03223	0.03434	0.04198	0.05188	0.06506
19	0.02321	0.03422	0.04078	0.04320	0.05193	0.06302	0.07757
20	0.02970	0.04243	0.04986	0.05260	0.06231	0.07449	0.09027
21	0.03654	0.05108	0.05935	0.06235	0.07297	0.08617	0.10296
22	0.04404	0.06010	0.06907	0.07236	0.08385	0.09789	0.11565
23	0.05196	0.06931	0.07898	0.08248	0.09473	0.10960	0.12819
24	0.06011	0.07874	0.08908	0.09277	0.10573	0.12128	0.14060
25	0.06831	0.08834	0.09921	0.10310	0.11668	0.13284	0.15281
26	0.07683	0.09798	0.10948	0.11351	0.12754	0.14431	0.16482
27	0.08542	0.10767	0.11955	0.12376	0.13832	0.15553	0.17656
28	0.09401	0.11726	0.12967	0.13396	0.14892	0.16663	0.18807
29	0.10292	0.12687	0.13963	0.14405	0.15943	0.17749	0.19928
30	0.11165	0.13632	0.14946	0.15404	0.16977	0.18815	0.21022
31	0.12030	0.14579	0.15922	0.16388	0.17989	0.19857	0.22097
32	0.12899	0.15524	0.16879	0.17362	0.18989	0.20880	0.23143
33	0.13753	0.16440	0.17826	0.18320	0.19972	0.21884	0.24158
34	0.14631	0.17348	0.18773	0.19267	0.20931	0.22864	0.25156
35	0.15470	0.18258	0.19694	0.20190	0.21874	0.23821	0.26124
36	0.16316	0.19141	0.20597	0.21102	0.22799	0.24757	0.27068
37	0.17138	0.20007	0.21481	0.21989	0.23706	0.25669	0.27987
38	0.17954	0.20870	0.22347	0.22867	0.24590	0.26559	0.28881
39	0.18756	0.21702	0.23204	0.23717	0.25448	0.27434	0.29756
40	0.19527	0.22522	0.24039	0.24558	0.26295	0.28283	0.30606
41	0.20316	0.23334	0.24855	0.25379	0.27125	0.29115	0.31436
42	0.21103	0.24129	0.25660	0.26183	0.27934	0.29928	0.32249
43	0.21870	0.24911	0.26446	0.26968	0.28730	0.30722	0.33037
44	0.22622	0.25682	0.27214	0.27744	0.29509	0.31494	0.33808
45	0.23366	0.26427	0.27971	0.28503	0.30268	0.32249	0.34560
46	0.24085	0.27161	0.28706	0.29238	0.31005	0.32987	0.35290
47	0.24792	0.27884	0.29434	0.29964	0.31730	0.33711	0.36006
48	0.25488	0.28604	0.30152	0.30679	0.32442	0.34416	0.36703
49	0.26179	0.29297	0.30839	0.31368	0.33132	0.35108	0.37385
50	0.26875	0.29990	0.31525	0.32052	0.33811	0.35781	0.38053
51	0.27543	0.30666	0.32203	0.32726	0.34480	0.36438	0.38702
52	0.28216	0.31320	0.32855	0.33377	0.35132	0.37083	0.39337
53	0.28846	0.31964	0.33496	0.34020	0.35764	0.37712	0.39959
54	0.29467	0.32602	0.34128	0.34654	0.36387	0.38336	0.40567
55	0.30094	0.33217	0.34746	0.35270	0.37002	0.38944	0.41161
56	0.30705	0.33826	0.35354	0.35869	0.37601	0.39535	0.41743
57	0.31289	0.34429	0.35946	0.36461	0.38190	0.40113	0.42313
58	0.31887	0.35023	0.36529	0.37046	0.38767	0.40680	0.42868
59	0.32474	0.35604	0.37100	0.37618	0.39329	0.41236	0.43413
60	0.33054	0.36165	0.37667	0.38179	0.39882	0.41781	0.43945
61	0.33598	0.36721	0.38221	0.38731	0.40426	0.42312	0.44468
62	0.34156	0.37262	0.38763	0.39269	0.40958	0.42834	0.44978
63	0.34693	0.37794	0.39289	0.39798	0.41483	0.43350	0.45476
64	0.35224	0.38315	0.39809	0.40311	0.41991	0.43853	0.45969
65	0.35754	0.38829	0.40315	0.40820	0.42494	0.44344	0.46449
66	0.36282	0.39337	0.40813	0.41323	0.42988	0.44827	0.46919
67	0.36784	0.39838	0.41311	0.41809	0.43472	0.45300	0.47380
68	0.37290	0.40328	0.41794	0.42293	0.43944	0.45765	0.47833
69	0.37773	0.40815	0.42264	0.42768	0.44409	0.46224	0.48277
70	0.38259	0.41287	0.42740	0.43235	0.44867	0.46672	0.48714
71	0.38728	0.41754	0.43205	0.43700	0.45316	0.47110	0.49143
72	0.39191	0.42216	0.43653	0.44145	0.45757	0.47541	0.49562
73	0.39649	0.42662	0.44101	0.44586	0.46193	0.47964	0.49974

N	0.1%	0.5%	1%	1.25%	2.5%	5%	10%
74	0.40095	0.43104	0.44534	0.45020	0.46617	0.48381	0.50378
75	0.40532	0.43531	0.44966	0.45449	0.47036	0.48790	0.50775
76	0.40960	0.43955	0.45389	0.45869	0.47445	0.49192	0.51163
77	0.41415	0.44382	0.45804	0.46281	0.47850	0.49584	0.51547
78	0.41855	0.44793	0.46207	0.46683	0.48246	0.49972	0.51925
79	0.42280	0.45206	0.46609	0.47081	0.48638	0.50353	0.52292
80	0.42694	0.45600	0.47001	0.47472	0.49021	0.50728	0.52659
81	0.43080	0.45993	0.47381	0.47852	0.49399	0.51096	0.53015
82	0.43485	0.46379	0.47762	0.48235	0.49771	0.51459	0.53366
83	0.43860	0.46761	0.48139	0.48606	0.50137	0.51818	0.53710
84	0.44249	0.47144	0.48510	0.48974	0.50498	0.52168	0.54050
85	0.44634	0.47502	0.48872	0.49336	0.50851	0.52514	0.54385
86	0.45006	0.47872	0.49229	0.49690	0.51202	0.52855	0.54713
87	0.45385	0.48232	0.49582	0.50043	0.51541	0.53187	0.55034
88	0.45762	0.48586	0.49938	0.50393	0.51879	0.53515	0.55354
89	0.46129	0.48933	0.50279	0.50732	0.52214	0.53838	0.55664
90	0.46474	0.49283	0.50615	0.51069	0.52541	0.54157	0.55973
91	0.46836	0.49623	0.50949	0.51399	0.52865	0.54470	0.56277
92	0.47177	0.49954	0.51276	0.51721	0.53180	0.54780	0.56575
93	0.47519	0.50284	0.51600	0.52042	0.53488	0.55084	0.56869
94	0.47850	0.50603	0.51918	0.52357	0.53796	0.55384	0.57157
95	0.48186	0.50920	0.52222	0.52667	0.54103	0.55679	0.57443
96	0.48513	0.51240	0.52538	0.52974	0.54405	0.55969	0.57724
97	0.48836	0.51556	0.52840	0.53276	0.54699	0.56255	0.58000
98	0.49155	0.51868	0.53138	0.53572	0.54990	0.56540	0.58275
99	0.49473	0.52167	0.53438	0.53872	0.55276	0.56815	0.58543
100	0.49782	0.52460	0.53726	0.54156	0.55557	0.57089	0.58810
101	0.50089	0.52755	0.54019	0.54442	0.55835	0.57361	0.59071
102	0.50393	0.53047	0.54305	0.54728	0.56111	0.57629	0.59328
103	0.50695	0.53331	0.54584	0.55005	0.56383	0.57890	0.59581
104	0.50982	0.53617	0.54862	0.55278	0.56650	0.58147	0.59831
105	0.51268	0.53891	0.55136	0.55551	0.56913	0.58404	0.60077
106	0.51566	0.54163	0.55402	0.55819	0.57176	0.58656	0.60320
107	0.51844	0.54441	0.55670	0.56079	0.57432	0.58905	0.60559
108	0.52124	0.54710	0.55930	0.56344	0.57686	0.59152	0.60796
109	0.52402	0.54977	0.56190	0.56601	0.57936	0.59395	0.61030
110	0.52676	0.55238	0.56447	0.56853	0.58181	0.59631	0.61259
111	0.52937	0.55491	0.56698	0.57100	0.58426	0.59869	0.61486
112	0.53203	0.55748	0.56946	0.57346	0.58663	0.60101	0.61711
113	0.53475	0.55997	0.57191	0.57591	0.58901	0.60331	0.61934
114	0.53729	0.56243	0.57432	0.57831	0.59134	0.60557	0.62152
115	0.53989	0.56490	0.57669	0.58066	0.59366	0.60780	0.62367
116	0.54244	0.56729	0.57907	0.58303	0.59594	0.61001	0.62580
117	0.54489	0.56971	0.58142	0.58535	0.59819	0.61220	0.62789
118	0.54729	0.57212	0.58370	0.58765	0.60042	0.61435	0.62996
119	0.54969	0.57441	0.58598	0.58990	0.60262	0.61646	0.63200
120	0.55211	0.57670	0.58826	0.59213	0.60479	0.61858	0.63402
121	0.55452	0.57897	0.59049	0.59433	0.60693	0.62065	0.63603
122	0.55683	0.58124	0.59268	0.59651	0.60904	0.62272	0.63801
123	0.55921	0.58347	0.59484	0.59865	0.61115	0.62473	0.63995
124	0.56155	0.58569	0.59695	0.60078	0.61324	0.62674	0.64188
125	0.56386	0.58782	0.59905	0.60286	0.61529	0.62872	0.64378
126	0.56614	0.58996	0.60114	0.60492	0.61731	0.63067	0.64566
127	0.56830	0.59208	0.60323	0.60698	0.61930	0.63262	0.64753
128	0.57055	0.59421	0.60526	0.60902	0.62127	0.63452	0.64937
129	0.57270	0.59623	0.60730	0.61104	0.62323	0.63642	0.65118
130	0.57486	0.59827	0.60928	0.61301	0.62514	0.63828	0.65298
131	0.57685	0.60036	0.61129	0.61501	0.62704	0.64014	0.65475
132	0.57910	0.60232	0.61324	0.61691	0.62892	0.64196	0.65649
133	0.58115	0.60430	0.61517	0.61883	0.63078	0.64377	0.65823
134	0.58331	0.60628	0.61710	0.62074	0.63262	0.64555	0.65995
135	0.58535	0.60818	0.61899	0.62263	0.63445	0.64732	0.66165
136	0.58738	0.61011	0.62086	0.62450	0.63625	0.64907	0.66331
137	0.58947	0.61197	0.62272	0.62634	0.63803	0.65078	0.66496
138	0.59145	0.61388	0.62454	0.62813	0.63977	0.65247	0.66661
139	0.59346	0.61570	0.62634	0.62989	0.64154	0.65415	0.66823
140	0.59536	0.61757	0.62816	0.63168	0.64326	0.65583	0.66982
141	0.59725	0.61935	0.62992	0.63345	0.64499	0.65748	0.67140
142	0.59912	0.62116	0.63168	0.63519	0.64668	0.65911	0.67296
143	0.60099	0.62301	0.63343	0.63694	0.64834	0.66074	0.67452
144	0.60287	0.62477	0.63514	0.63864	0.65000	0.66234	0.67605
145	0.60469	0.62653	0.63685	0.64034	0.65165	0.66393	0.67758
146	0.60645	0.62823	0.63855	0.64201	0.65327	0.66550	0.67909
147	0.60823	0.62995	0.64021	0.64367	0.65489	0.66702	0.68058

N	0.1%	0.5%	1%	1.25%	2.5%	5%	10%
148	0.61012	0.63160	0.64186	0.64529	0.65646	0.66856	0.68204
149	0.61185	0.63326	0.64347	0.64691	0.65802	0.67008	0.68349
150	0.61366	0.63496	0.64511	0.64854	0.65959	0.67159	0.68493
151	0.61534	0.63664	0.64670	0.65011	0.66113	0.67307	0.68635
152	0.61702	0.63827	0.64829	0.65169	0.66263	0.67454	0.68776
153	0.61874	0.63987	0.64987	0.65323	0.66416	0.67601	0.68916
154	0.62045	0.64147	0.65145	0.65479	0.66564	0.67745	0.69054
155	0.62209	0.64306	0.65300	0.65630	0.66713	0.67888	0.69191
156	0.62377	0.64462	0.65452	0.65779	0.66859	0.68028	0.69326
157	0.62531	0.64616	0.65604	0.65930	0.67002	0.68168	0.69461
158	0.62695	0.64769	0.65754	0.66078	0.67148	0.68306	0.69594
159	0.62851	0.64921	0.65902	0.66224	0.67289	0.68444	0.69726
160	0.63015	0.65074	0.66047	0.66369	0.67430	0.68579	0.69856
161	0.63173	0.65221	0.66191	0.66513	0.67571	0.68715	0.69985
162	0.63326	0.65368	0.66333	0.66656	0.67708	0.68850	0.70112
163	0.63481	0.65516	0.66475	0.66796	0.67844	0.68981	0.70240
164	0.63633	0.65659	0.66617	0.66936	0.67979	0.69111	0.70365
165	0.63788	0.65806	0.66754	0.67075	0.68114	0.69241	0.70490
166	0.63938	0.65945	0.66893	0.67210	0.68247	0.69369	0.70613
167	0.64091	0.66087	0.67031	0.67346	0.68379	0.69496	0.70735
168	0.64246	0.66226	0.67166	0.67481	0.68510	0.69623	0.70856
169	0.64396	0.66369	0.67302	0.67617	0.68637	0.69749	0.70975
170	0.64537	0.66508	0.67436	0.67749	0.68766	0.69872	0.71094
171	0.64668	0.66644	0.67569	0.67880	0.68894	0.69993	0.71211
172	0.64812	0.66777	0.67701	0.68011	0.69020	0.70115	0.71327
173	0.64952	0.66912	0.67831	0.68140	0.69145	0.70236	0.71443
174	0.65089	0.67045	0.67959	0.68266	0.69269	0.70355	0.71557
175	0.65234	0.67180	0.68087	0.68392	0.69392	0.70474	0.71670
176	0.65363	0.67307	0.68211	0.68517	0.69512	0.70591	0.71783
177	0.65496	0.67439	0.68337	0.68641	0.69633	0.70707	0.71894
178	0.65633	0.67562	0.68462	0.68764	0.69751	0.70823	0.72005
179	0.65762	0.67689	0.68586	0.68886	0.69869	0.70936	0.72114
180	0.65900	0.67816	0.68709	0.69007	0.69987	0.71049	0.72223
181	0.66029	0.67940	0.68829	0.69128	0.70102	0.71161	0.72330
182	0.66162	0.68066	0.68949	0.69248	0.70219	0.71272	0.72437
183	0.66297	0.68190	0.69070	0.69366	0.70333	0.71381	0.72543
184	0.66425	0.68311	0.69187	0.69481	0.70446	0.71491	0.72647
185	0.66548	0.68427	0.69303	0.69598	0.70558	0.71599	0.72751
186	0.66675	0.68548	0.69418	0.69712	0.70668	0.71707	0.72853
187	0.66800	0.68666	0.69534	0.69826	0.70779	0.71813	0.72956
188	0.66925	0.68785	0.69651	0.69939	0.70888	0.71920	0.73057
189	0.67051	0.68900	0.69764	0.70051	0.70997	0.72024	0.73157
190	0.67166	0.69014	0.69876	0.70161	0.71103	0.72128	0.73257
191	0.67289	0.69126	0.69988	0.70273	0.71210	0.72230	0.73356
192	0.67408	0.69242	0.70100	0.70384	0.71317	0.72333	0.73454
193	0.67525	0.69354	0.70207	0.70493	0.71423	0.72434	0.73551
194	0.67642	0.69466	0.70316	0.70598	0.71527	0.72534	0.73647
195	0.67759	0.69577	0.70423	0.70707	0.71630	0.72633	0.73742
196	0.67873	0.69687	0.70530	0.70814	0.71732	0.72733	0.73837
197	0.67983	0.69796	0.70636	0.70918	0.71834	0.72831	0.73931
198	0.68100	0.69904	0.70740	0.71021	0.71936	0.72928	0.74024
199	0.68215	0.70010	0.70845	0.71125	0.72036	0.73025	0.74116
200	0.68333	0.70113	0.70951	0.71225	0.72135	0.73121	0.74207
201	0.68446	0.70219	0.71052	0.71328	0.72234	0.73216	0.74298
202	0.68555	0.70326	0.71155	0.71431	0.72331	0.73310	0.74388
203	0.68665	0.70432	0.71257	0.71530	0.72429	0.73402	0.74478
204	0.68771	0.70536	0.71356	0.71630	0.72524	0.73495	0.74568
205	0.68879	0.70635	0.71456	0.71729	0.72621	0.73588	0.74656
206	0.68983	0.70738	0.71553	0.71826	0.72715	0.73679	0.74744
207	0.69093	0.70841	0.71654	0.71924	0.72809	0.73769	0.74830
208	0.69203	0.70939	0.71752	0.72020	0.72903	0.73861	0.74916
209	0.69302	0.71041	0.71850	0.72117	0.72995	0.73951	0.75002
210	0.69408	0.71138	0.71944	0.72210	0.73088	0.74038	0.75086
211	0.69512	0.71234	0.72038	0.72307	0.73179	0.74125	0.75170
212	0.69617	0.71333	0.72131	0.72400	0.73270	0.74213	0.75254
213	0.69716	0.71430	0.72225	0.72492	0.73359	0.74299	0.75337
214	0.69819	0.71526	0.72319	0.72584	0.73449	0.74385	0.75419
215	0.69922	0.71621	0.72410	0.72678	0.73536	0.74470	0.75501
216	0.70026	0.71716	0.72503	0.72767	0.73626	0.74557	0.75582
217	0.70126	0.71808	0.72595	0.72857	0.73712	0.74640	0.75662
218	0.70224	0.71903	0.72685	0.72946	0.73799	0.74724	0.75742
219	0.70321	0.71994	0.72774	0.73036	0.73885	0.74806	0.75822
220	0.70414	0.72085	0.72865	0.73125	0.73970	0.74887	0.75901
221	0.70510	0.72178	0.72954	0.73213	0.74055	0.74970	0.75979

N	0.1%	0.5%	1%	1.25%	2.5%	5%	10%
222	0.70609	0.72268	0.73042	0.73298	0.74140	0.75051	0.76057
223	0.70702	0.72358	0.73128	0.73383	0.74223	0.75130	0.76134
224	0.70793	0.72448	0.73214	0.73470	0.74306	0.75210	0.76210
225	0.70892	0.72536	0.73300	0.73554	0.74387	0.75289	0.76286
226	0.70985	0.72622	0.73385	0.73638	0.74470	0.75368	0.76361
227	0.71074	0.72710	0.73470	0.73721	0.74550	0.75446	0.76437
228	0.71161	0.72796	0.73553	0.73805	0.74630	0.75524	0.76510
229	0.71252	0.72882	0.73634	0.73889	0.74709	0.75601	0.76584
230	0.71343	0.72969	0.73719	0.73971	0.74789	0.75678	0.76657
231	0.71435	0.73056	0.73802	0.74052	0.74867	0.75754	0.76731
232	0.71519	0.73140	0.73883	0.74133	0.74946	0.75829	0.76803
233	0.71611	0.73222	0.73964	0.74213	0.75024	0.75904	0.76875
234	0.71697	0.73304	0.74044	0.74291	0.75103	0.75977	0.76946
235	0.71786	0.73385	0.74123	0.74370	0.75179	0.76052	0.77017
236	0.71876	0.73468	0.74203	0.74450	0.75254	0.76126	0.77087
237	0.71968	0.73550	0.74284	0.74528	0.75331	0.76198	0.77157
238	0.72052	0.73629	0.74363	0.74606	0.75405	0.76270	0.77226
239	0.72140	0.73710	0.74441	0.74684	0.75481	0.76342	0.77296
240	0.72223	0.73790	0.74519	0.74760	0.75555	0.76414	0.77364
241	0.72303	0.73869	0.74597	0.74837	0.75627	0.76485	0.77432
242	0.72386	0.73948	0.74672	0.74912	0.75700	0.76555	0.77499
243	0.72468	0.74027	0.74748	0.74988	0.75773	0.76625	0.77566
244	0.72556	0.74106	0.74823	0.75063	0.75845	0.76695	0.77633
245	0.72637	0.74182	0.74897	0.75136	0.75917	0.76764	0.77699
246	0.72716	0.74259	0.74972	0.75211	0.75988	0.76832	0.77764
247	0.72800	0.74335	0.75046	0.75284	0.76060	0.76901	0.77829
248	0.72874	0.74409	0.75120	0.75357	0.76130	0.76968	0.77894
249	0.72956	0.74483	0.75193	0.75428	0.76200	0.77036	0.77959
250	0.73037	0.74558	0.75264	0.75500	0.76270	0.77102	0.78023
251	0.73119	0.74633	0.75336	0.75572	0.76339	0.77170	0.78086
252	0.73197	0.74703	0.75408	0.75643	0.76408	0.77235	0.78150
253	0.73277	0.74775	0.75479	0.75713	0.76476	0.77301	0.78212
254	0.73358	0.74849	0.75549	0.75782	0.76542	0.77366	0.78274
255	0.73433	0.74921	0.75619	0.75852	0.76609	0.77431	0.78336
256	0.73512	0.74995	0.75691	0.75920	0.76676	0.77496	0.78398
257	0.73583	0.75064	0.75759	0.75988	0.76743	0.77559	0.78459
258	0.73656	0.75135	0.75828	0.76057	0.76809	0.77622	0.78519
259	0.73734	0.75204	0.75896	0.76125	0.76874	0.77686	0.78581
260	0.73810	0.75275	0.75963	0.76193	0.76939	0.77749	0.78641
261	0.73890	0.75345	0.76031	0.76260	0.77003	0.77811	0.78700
262	0.73962	0.75414	0.76098	0.76326	0.77068	0.77873	0.78760
263	0.74033	0.75481	0.76165	0.76393	0.77132	0.77934	0.78818
264	0.74106	0.75549	0.76231	0.76458	0.77196	0.77995	0.78877
265	0.74175	0.75616	0.76295	0.76523	0.77259	0.78055	0.78935
266	0.74250	0.75683	0.76362	0.76588	0.77321	0.78116	0.78993
267	0.74315	0.75750	0.76428	0.76654	0.77383	0.78177	0.79051
268	0.74389	0.75816	0.76492	0.76717	0.77446	0.78236	0.79108
269	0.74461	0.75883	0.76556	0.76780	0.77508	0.78295	0.79165
270	0.74529	0.75949	0.76620	0.76844	0.77568	0.78353	0.79221
271	0.74597	0.76014	0.76685	0.76906	0.77629	0.78412	0.79277
272	0.74664	0.76081	0.76748	0.76970	0.77690	0.78471	0.79333
273	0.74732	0.76144	0.76810	0.77032	0.77751	0.78529	0.79389
274	0.74795	0.76210	0.76872	0.77094	0.77811	0.78586	0.79444
275	0.74863	0.76273	0.76935	0.77154	0.77872	0.78644	0.79498
276	0.74930	0.76338	0.76997	0.77215	0.77930	0.78701	0.79553
277	0.74996	0.76404	0.77059	0.77277	0.77989	0.78758	0.79607
278	0.75064	0.76466	0.77119	0.77337	0.78047	0.78814	0.79660
279	0.75134	0.76529	0.77179	0.77399	0.78105	0.78869	0.79714
280	0.75202	0.76591	0.77240	0.77459	0.78163	0.78924	0.79767
281	0.75269	0.76654	0.77299	0.77518	0.78221	0.78979	0.79821
282	0.75341	0.76716	0.77360	0.77577	0.78278	0.79034	0.79873
283	0.75396	0.76777	0.77421	0.77636	0.78334	0.79089	0.79925
284	0.75461	0.76838	0.77479	0.77695	0.78391	0.79143	0.79978
285	0.75529	0.76899	0.77538	0.77753	0.78447	0.79197	0.80030
286	0.75588	0.76960	0.77598	0.77811	0.78503	0.79251	0.80081
287	0.75655	0.77018	0.77657	0.77869	0.78558	0.79304	0.80132
288	0.75716	0.77077	0.77713	0.77925	0.78613	0.79357	0.80183
289	0.75778	0.77135	0.77770	0.77982	0.78667	0.79411	0.80233
290	0.75841	0.77196	0.77827	0.78037	0.78721	0.79462	0.80284
291	0.75905	0.77251	0.77885	0.78093	0.78776	0.79515	0.80333
292	0.75961	0.77310	0.77942	0.78150	0.78830	0.79568	0.80383
293	0.76020	0.77367	0.77996	0.78205	0.78884	0.79619	0.80432
294	0.76082	0.77424	0.78052	0.78260	0.78937	0.79670	0.80481
295	0.76145	0.77481	0.78108	0.78315	0.78991	0.79721	0.80530

N	0.1%	0.5%	1%	1.25%	2.5%	5%	10%
296	0.76203	0.77537	0.78163	0.78370	0.79043	0.79771	0.80579
297	0.76260	0.77598	0.78217	0.78424	0.79096	0.79822	0.80627
298	0.76320	0.77652	0.78272	0.78478	0.79149	0.79872	0.80675
299	0.76384	0.77708	0.78326	0.78531	0.79201	0.79922	0.80723
300	0.76442	0.77764	0.78381	0.78585	0.79251	0.79971	0.80770
301	0.76505	0.77819	0.78433	0.78638	0.79302	0.80021	0.80817
302	0.76563	0.77874	0.78486	0.78691	0.79353	0.80070	0.80864
303	0.76621	0.77928	0.78538	0.78742	0.79403	0.80119	0.80911
304	0.76680	0.77982	0.78592	0.78794	0.79454	0.80168	0.80957
305	0.76733	0.78037	0.78644	0.78846	0.79504	0.80216	0.81003
306	0.76789	0.78090	0.78697	0.78900	0.79554	0.80263	0.81049
307	0.76844	0.78144	0.78748	0.78951	0.79604	0.80311	0.81094
308	0.76901	0.78195	0.78799	0.79001	0.79654	0.80359	0.81140
309	0.76958	0.78250	0.78851	0.79053	0.79703	0.80406	0.81185
310	0.77016	0.78302	0.78902	0.79103	0.79752	0.80453	0.81230
311	0.77070	0.78355	0.78952	0.79153	0.79801	0.80500	0.81275
312	0.77124	0.78406	0.79002	0.79203	0.79849	0.80546	0.81319
313	0.77179	0.78457	0.79051	0.79252	0.79897	0.80592	0.81364
314	0.77231	0.78508	0.79102	0.79301	0.79945	0.80639	0.81408
315	0.77289	0.78561	0.79152	0.79350	0.79993	0.80685	0.81452
316	0.77341	0.78610	0.79203	0.79399	0.80040	0.80731	0.81495
317	0.77395	0.78662	0.79251	0.79448	0.80087	0.80775	0.81539
318	0.77446	0.78711	0.79299	0.79497	0.80133	0.80821	0.81582
319	0.77500	0.78762	0.79349	0.79544	0.80180	0.80866	0.81625
320	0.77555	0.78813	0.79397	0.79592	0.80226	0.80910	0.81667
321	0.77607	0.78862	0.79447	0.79640	0.80272	0.80955	0.81709
322	0.77663	0.78911	0.79495	0.79688	0.80318	0.80999	0.81751
323	0.77712	0.78960	0.79542	0.79735	0.80365	0.81042	0.81793
324	0.77766	0.79009	0.79589	0.79782	0.80409	0.81086	0.81835
325	0.77818	0.79058	0.79636	0.79828	0.80454	0.81130	0.81877
326	0.77873	0.79107	0.79683	0.79875	0.80499	0.81173	0.81918
327	0.77924	0.79155	0.79730	0.79921	0.80544	0.81216	0.81960
328	0.77970	0.79202	0.79777	0.79966	0.80588	0.81259	0.82000
329	0.78025	0.79250	0.79823	0.80013	0.80633	0.81301	0.82041
330	0.78074	0.79298	0.79867	0.80058	0.80677	0.81343	0.82081
331	0.78125	0.79345	0.79913	0.80104	0.80720	0.81385	0.82121
332	0.78175	0.79393	0.79960	0.80148	0.80764	0.81427	0.82161
333	0.78222	0.79440	0.80005	0.80193	0.80807	0.81470	0.82202
334	0.78271	0.79486	0.80049	0.80238	0.80850	0.81512	0.82241
335	0.78316	0.79530	0.80094	0.80281	0.80893	0.81553	0.82281
336	0.78368	0.79577	0.80138	0.80327	0.80935	0.81594	0.82320
337	0.78417	0.79623	0.80182	0.80371	0.80978	0.81634	0.82359
338	0.78468	0.79668	0.80227	0.80415	0.81020	0.81675	0.82398
339	0.78519	0.79713	0.80272	0.80459	0.81062	0.81716	0.82437
340	0.78562	0.79757	0.80315	0.80503	0.81105	0.81756	0.82476
341	0.78606	0.79802	0.80358	0.80546	0.81147	0.81796	0.82514
342	0.78654	0.79847	0.80402	0.80589	0.81188	0.81836	0.82552
343	0.78701	0.79893	0.80445	0.80631	0.81229	0.81876	0.82590
344	0.78750	0.79937	0.80488	0.80674	0.81271	0.81915	0.82628
345	0.78799	0.79982	0.80530	0.80716	0.81312	0.81955	0.82665
346	0.78843	0.80026	0.80574	0.80758	0.81352	0.81994	0.82703
347	0.78889	0.80069	0.80617	0.80800	0.81393	0.82033	0.82740
348	0.78934	0.80113	0.80659	0.80842	0.81434	0.82073	0.82778
349	0.78984	0.80157	0.80702	0.80883	0.81473	0.82111	0.82815
350	0.79029	0.80201	0.80743	0.80925	0.81513	0.82149	0.82851
351	0.79077	0.80243	0.80785	0.80966	0.81553	0.82187	0.82888
352	0.79120	0.80286	0.80825	0.81007	0.81593	0.82225	0.82924
353	0.79168	0.80329	0.80867	0.81048	0.81632	0.82263	0.82960
354	0.79210	0.80371	0.80909	0.81088	0.81671	0.82301	0.82996
355	0.79253	0.80413	0.80949	0.81128	0.81710	0.82339	0.83032
356	0.79297	0.80454	0.80990	0.81169	0.81749	0.82376	0.83068
357	0.79341	0.80496	0.81030	0.81209	0.81787	0.82413	0.83104
358	0.79385	0.80536	0.81071	0.81249	0.81826	0.82450	0.83139
359	0.79430	0.80577	0.81111	0.81289	0.81865	0.82488	0.83174
360	0.79472	0.80618	0.81151	0.81327	0.81902	0.82524	0.83209
361	0.79514	0.80659	0.81190	0.81366	0.81941	0.82560	0.83244
362	0.79558	0.80700	0.81230	0.81406	0.81978	0.82596	0.83279
363	0.79602	0.80740	0.81269	0.81445	0.82016	0.82632	0.83314
364	0.79640	0.80781	0.81308	0.81484	0.82053	0.82669	0.83348
365	0.79682	0.80821	0.81347	0.81522	0.82090	0.82704	0.83383
366	0.79725	0.80862	0.81387	0.81561	0.82127	0.82740	0.83417
367	0.79769	0.80902	0.81424	0.81598	0.82164	0.82775	0.83451
368	0.79810	0.80940	0.81461	0.81636	0.82200	0.82811	0.83485
369	0.79853	0.80979	0.81500	0.81673	0.82237	0.82846	0.83519

N	0.1%	0.5%	1%	1.25%	2.5%	5%	10%
370	0.79894	0.81018	0.81538	0.81712	0.82274	0.82881	0.83552
371	0.79931	0.81056	0.81577	0.81749	0.82310	0.82916	0.83585
372	0.79972	0.81095	0.81614	0.81787	0.82347	0.82950	0.83619
373	0.80013	0.81134	0.81652	0.81823	0.82382	0.82985	0.83652
374	0.80056	0.81173	0.81689	0.81861	0.82418	0.83020	0.83685
375	0.80098	0.81213	0.81726	0.81898	0.82454	0.83054	0.83718
376	0.80137	0.81251	0.81764	0.81935	0.82489	0.83088	0.83750
377	0.80176	0.81288	0.81800	0.81971	0.82525	0.83123	0.83783
378	0.80217	0.81326	0.81836	0.82006	0.82560	0.83156	0.83815
379	0.80258	0.81364	0.81874	0.82043	0.82595	0.83189	0.83847
380	0.80297	0.81400	0.81910	0.82079	0.82630	0.83223	0.83879
381	0.80339	0.81439	0.81946	0.82114	0.82665	0.83257	0.83911
382	0.80377	0.81476	0.81982	0.82151	0.82699	0.83290	0.83942
383	0.80414	0.81512	0.82019	0.82187	0.82733	0.83323	0.83974
384	0.80453	0.81549	0.82055	0.82223	0.82767	0.83356	0.84006
385	0.80495	0.81586	0.82090	0.82257	0.82801	0.83388	0.84037
386	0.80537	0.81622	0.82125	0.82292	0.82835	0.83421	0.84068
387	0.80575	0.81658	0.82159	0.82326	0.82869	0.83454	0.84100
388	0.80611	0.81695	0.82194	0.82360	0.82902	0.83486	0.84131
389	0.80648	0.81732	0.82229	0.82395	0.82935	0.83518	0.84161
390	0.80688	0.81768	0.82265	0.82429	0.82969	0.83550	0.84192
391	0.80728	0.81806	0.82299	0.82464	0.83002	0.83582	0.84223
392	0.80763	0.81840	0.82334	0.82498	0.83035	0.83614	0.84253
393	0.80803	0.81875	0.82366	0.82532	0.83068	0.83645	0.84283
394	0.80840	0.81910	0.82402	0.82567	0.83100	0.83677	0.84313
395	0.80879	0.81945	0.82436	0.82600	0.83133	0.83708	0.84344
396	0.80913	0.81981	0.82471	0.82633	0.83166	0.83740	0.84373
397	0.80950	0.82014	0.82504	0.82666	0.83198	0.83771	0.84403
398	0.80986	0.82049	0.82538	0.82700	0.83230	0.83802	0.84433
399	0.81024	0.82084	0.82572	0.82734	0.83262	0.83833	0.84462
400	0.81061	0.82117	0.82605	0.82766	0.83294	0.83864	0.84492
401	0.81097	0.82152	0.82638	0.82799	0.83325	0.83895	0.84521
402	0.81133	0.82187	0.82671	0.82833	0.83358	0.83925	0.84550
403	0.81170	0.82221	0.82704	0.82865	0.83389	0.83956	0.84579
404	0.81204	0.82255	0.82737	0.82898	0.83421	0.83986	0.84608
405	0.81241	0.82289	0.82769	0.82931	0.83452	0.84016	0.84637
406	0.81274	0.82322	0.82802	0.82963	0.83484	0.84046	0.84666
407	0.81309	0.82356	0.82836	0.82996	0.83515	0.84076	0.84695
408	0.81348	0.82389	0.82868	0.83026	0.83546	0.84106	0.84723
409	0.81383	0.82423	0.82901	0.83058	0.83576	0.84135	0.84752
410	0.81417	0.82455	0.82932	0.83089	0.83607	0.84164	0.84780
411	0.81450	0.82487	0.82964	0.83121	0.83638	0.84194	0.84808
412	0.81486	0.82521	0.82996	0.83153	0.83668	0.84223	0.84836
413	0.81520	0.82552	0.83027	0.83184	0.83698	0.84252	0.84864
414	0.81552	0.82584	0.83058	0.83214	0.83728	0.84281	0.84891
415	0.81585	0.82618	0.83089	0.83246	0.83758	0.84309	0.84919
416	0.81618	0.82649	0.83120	0.83277	0.83788	0.84338	0.84947
417	0.81651	0.82681	0.83151	0.83307	0.83818	0.84367	0.84974
418	0.81687	0.82713	0.83183	0.83338	0.83847	0.84395	0.85001
419	0.81719	0.82744	0.83214	0.83370	0.83877	0.84424	0.85029
420	0.81754	0.82774	0.83244	0.83400	0.83907	0.84452	0.85055
421	0.81789	0.82805	0.83275	0.83431	0.83936	0.84481	0.85082
422	0.81822	0.82837	0.83306	0.83461	0.83964	0.84509	0.85110
423	0.81855	0.82867	0.83335	0.83491	0.83994	0.84537	0.85136
424	0.81889	0.82899	0.83365	0.83520	0.84023	0.84564	0.85163
425	0.81924	0.82931	0.83395	0.83550	0.84052	0.84592	0.85189
426	0.81956	0.82960	0.83425	0.83580	0.84081	0.84619	0.85216
427	0.81989	0.82991	0.83455	0.83610	0.84109	0.84647	0.85242
428	0.82021	0.83021	0.83485	0.83639	0.84137	0.84674	0.85269
429	0.82055	0.83053	0.83515	0.83668	0.84165	0.84702	0.85295
430	0.82089	0.83084	0.83544	0.83697	0.84193	0.84729	0.85321
431	0.82121	0.83113	0.83574	0.83727	0.84221	0.84756	0.85347
432	0.82154	0.83144	0.83603	0.83755	0.84249	0.84783	0.85373
433	0.82185	0.83175	0.83632	0.83785	0.84277	0.84810	0.85398
434	0.82219	0.83204	0.83662	0.83814	0.84305	0.84837	0.85424
435	0.82252	0.83234	0.83690	0.83843	0.84333	0.84864	0.85449
436	0.82282	0.83264	0.83720	0.83871	0.84361	0.84890	0.85475
437	0.82315	0.83294	0.83748	0.83900	0.84389	0.84916	0.85500
438	0.82346	0.83324	0.83777	0.83928	0.84416	0.84943	0.85525
439	0.82378	0.83353	0.83805	0.83956	0.84444	0.84969	0.85550
440	0.82413	0.83382	0.83833	0.83984	0.84470	0.84995	0.85576
441	0.82441	0.83411	0.83861	0.84012	0.84497	0.85021	0.85600
442	0.82469	0.83439	0.83889	0.84040	0.84524	0.85048	0.85625
443	0.82500	0.83468	0.83917	0.84068	0.84551	0.85073	0.85650

N	0.1%	0.5%	1%	1.25%	2.5%	5%	10%
444	0.82532	0.83497	0.83946	0.84096	0.84578	0.85099	0.85674
445	0.82563	0.83525	0.83973	0.84123	0.84604	0.85124	0.85699
446	0.82594	0.83554	0.84002	0.84151	0.84631	0.85150	0.85723
447	0.82621	0.83583	0.84029	0.84179	0.84657	0.85176	0.85748
448	0.82654	0.83611	0.84057	0.84206	0.84683	0.85201	0.85772
449	0.82684	0.83638	0.84084	0.84234	0.84710	0.85226	0.85796
450	0.82716	0.83667	0.84112	0.84260	0.84736	0.85251	0.85820
451	0.82745	0.83695	0.84139	0.84288	0.84762	0.85276	0.85844
452	0.82775	0.83723	0.84166	0.84315	0.84788	0.85301	0.85868
453	0.82802	0.83750	0.84192	0.84341	0.84814	0.85326	0.85892
454	0.82831	0.83779	0.84219	0.84367	0.84840	0.85351	0.85916
455	0.82860	0.83806	0.84246	0.84393	0.84866	0.85376	0.85939
456	0.82890	0.83834	0.84273	0.84419	0.84891	0.85400	0.85963
457	0.82919	0.83863	0.84300	0.84445	0.84916	0.85424	0.85986
458	0.82948	0.83890	0.84327	0.84472	0.84942	0.85449	0.86010
459	0.82977	0.83917	0.84353	0.84498	0.84967	0.85473	0.86033
460	0.83007	0.83944	0.84380	0.84524	0.84993	0.85498	0.86056
461	0.83037	0.83971	0.84406	0.84550	0.85018	0.85522	0.86079
462	0.83064	0.83998	0.84432	0.84577	0.85043	0.85546	0.86102
463	0.83092	0.84024	0.84458	0.84603	0.85067	0.85570	0.86125
464	0.83123	0.84052	0.84483	0.84628	0.85092	0.85594	0.86149
465	0.83151	0.84078	0.84510	0.84654	0.85118	0.85618	0.86172
466	0.83179	0.84105	0.84535	0.84679	0.85142	0.85641	0.86194
467	0.83205	0.84131	0.84560	0.84704	0.85167	0.85665	0.86217
468	0.83232	0.84157	0.84586	0.84730	0.85191	0.85689	0.86239
469	0.83261	0.84183	0.84612	0.84755	0.85216	0.85712	0.86262
470	0.83288	0.84210	0.84637	0.84780	0.85240	0.85735	0.86284
471	0.83317	0.84235	0.84662	0.84805	0.85264	0.85759	0.86307
472	0.83344	0.84261	0.84687	0.84829	0.85289	0.85783	0.86329
473	0.83373	0.84286	0.84713	0.84853	0.85312	0.85806	0.86351
474	0.83401	0.84312	0.84738	0.84879	0.85336	0.85829	0.86373
475	0.83426	0.84338	0.84762	0.84904	0.85360	0.85851	0.86395
476	0.83454	0.84363	0.84787	0.84928	0.85384	0.85874	0.86417
477	0.83480	0.84389	0.84812	0.84953	0.85408	0.85897	0.86439
478	0.83510	0.84414	0.84836	0.84977	0.85431	0.85920	0.86460
479	0.83537	0.84441	0.84862	0.85001	0.85455	0.85943	0.86482
480	0.83564	0.84465	0.84886	0.85026	0.85479	0.85965	0.86504
481	0.83589	0.84491	0.84910	0.85050	0.85503	0.85988	0.86525
482	0.83615	0.84517	0.84935	0.85074	0.85526	0.86010	0.86547
483	0.83642	0.84541	0.84959	0.85098	0.85549	0.86033	0.86568
484	0.83670	0.84566	0.84983	0.85121	0.85571	0.86055	0.86590
485	0.83697	0.84590	0.85007	0.85145	0.85594	0.86077	0.86611
486	0.83721	0.84616	0.85031	0.85169	0.85617	0.86099	0.86632
487	0.83747	0.84640	0.85055	0.85193	0.85640	0.86122	0.86653
488	0.83774	0.84665	0.85078	0.85216	0.85663	0.86144	0.86674
489	0.83798	0.84688	0.85101	0.85240	0.85685	0.86165	0.86695
490	0.83825	0.84714	0.85125	0.85262	0.85708	0.86187	0.86715
491	0.83851	0.84740	0.85148	0.85286	0.85731	0.86209	0.86737
492	0.83877	0.84764	0.85172	0.85309	0.85753	0.86230	0.86757
493	0.83902	0.84787	0.85195	0.85332	0.85775	0.86252	0.86778
494	0.83927	0.84811	0.85219	0.85356	0.85798	0.86274	0.86798
495	0.83955	0.84834	0.85242	0.85378	0.85820	0.86295	0.86819
496	0.83982	0.84859	0.85266	0.85401	0.85842	0.86316	0.86839
497	0.84007	0.84883	0.85289	0.85424	0.85864	0.86337	0.86860
498	0.84033	0.84907	0.85312	0.85447	0.85887	0.86359	0.86880
499	0.84057	0.84931	0.85335	0.85470	0.85908	0.86380	0.86900
500	0.84084	0.84955	0.85358	0.85492	0.85930	0.86401	0.86920
501	0.84108	0.84978	0.85381	0.85515	0.85952	0.86422	0.86941
502	0.84132	0.85001	0.85405	0.85537	0.85974	0.86443	0.86961
503	0.84158	0.85024	0.85426	0.85560	0.85996	0.86464	0.86981
504	0.84181	0.85046	0.85449	0.85582	0.86018	0.86484	0.87001
505	0.84206	0.85070	0.85472	0.85605	0.86039	0.86505	0.87021
506	0.84230	0.85092	0.85494	0.85627	0.86061	0.86526	0.87040
507	0.84255	0.85115	0.85516	0.85649	0.86082	0.86547	0.87060
508	0.84280	0.85138	0.85539	0.85672	0.86103	0.86567	0.87080
509	0.84306	0.85161	0.85561	0.85694	0.86124	0.86588	0.87099
510	0.84330	0.85183	0.85583	0.85716	0.86145	0.86608	0.87118
511	0.84353	0.85206	0.85605	0.85737	0.86166	0.86628	0.87138
512	0.84376	0.85229	0.85628	0.85759	0.86187	0.86649	0.87158
513	0.84400	0.85252	0.85650	0.85781	0.86208	0.86669	0.87177
514	0.84423	0.85275	0.85672	0.85803	0.86229	0.86689	0.87196
515	0.84448	0.85296	0.85694	0.85825	0.86250	0.86709	0.87215
516	0.84473	0.85319	0.85715	0.85846	0.86270	0.86729	0.87234
517	0.84495	0.85341	0.85736	0.85868	0.86291	0.86749	0.87253

N	0.1%	0.5%	1%	1.25%	2.5%	5%	10%
518	0.84518	0.85363	0.85758	0.85889	0.86311	0.86769	0.87272
519	0.84544	0.85386	0.85779	0.85909	0.86332	0.86788	0.87291
520	0.84567	0.85407	0.85800	0.85930	0.86352	0.86808	0.87310
521	0.84591	0.85429	0.85821	0.85952	0.86373	0.86828	0.87329
522	0.84615	0.85452	0.85843	0.85973	0.86393	0.86847	0.87348
523	0.84639	0.85474	0.85864	0.85994	0.86413	0.86867	0.87366
524	0.84662	0.85496	0.85886	0.86014	0.86434	0.86886	0.87385
525	0.84686	0.85518	0.85906	0.86035	0.86454	0.86905	0.87403
526	0.84707	0.85539	0.85928	0.86056	0.86474	0.86925	0.87422
527	0.84729	0.85561	0.85948	0.86077	0.86494	0.86944	0.87440
528	0.84751	0.85582	0.85968	0.86097	0.86514	0.86963	0.87459
529	0.84771	0.85603	0.85989	0.86118	0.86533	0.86982	0.87477
530	0.84795	0.85625	0.86010	0.86138	0.86554	0.87001	0.87496
531	0.84819	0.85646	0.86031	0.86158	0.86573	0.87020	0.87514
532	0.84842	0.85668	0.86051	0.86179	0.86593	0.87039	0.87532
533	0.84866	0.85689	0.86072	0.86199	0.86613	0.87058	0.87550
534	0.84888	0.85711	0.86092	0.86219	0.86632	0.87077	0.87567
535	0.84911	0.85732	0.86113	0.86240	0.86652	0.87096	0.87586
536	0.84934	0.85753	0.86133	0.86259	0.86671	0.87114	0.87603
537	0.84954	0.85773	0.86153	0.86280	0.86691	0.87133	0.87621
538	0.84976	0.85794	0.86173	0.86300	0.86710	0.87152	0.87639
539	0.84997	0.85816	0.86194	0.86320	0.86729	0.87170	0.87657
540	0.85021	0.85836	0.86214	0.86340	0.86748	0.87189	0.87674
541	0.85044	0.85857	0.86234	0.86359	0.86768	0.87207	0.87692
542	0.85064	0.85877	0.86254	0.86379	0.86786	0.87225	0.87710
543	0.85086	0.85897	0.86273	0.86398	0.86805	0.87244	0.87727
544	0.85109	0.85917	0.86294	0.86418	0.86824	0.87262	0.87745
545	0.85130	0.85937	0.86313	0.86438	0.86843	0.87280	0.87762
546	0.85151	0.85957	0.86333	0.86458	0.86862	0.87298	0.87779
547	0.85173	0.85977	0.86352	0.86478	0.86881	0.87316	0.87797
548	0.85193	0.85997	0.86371	0.86496	0.86900	0.87334	0.87814
549	0.85212	0.86017	0.86391	0.86516	0.86918	0.87352	0.87831
550	0.85233	0.86037	0.86410	0.86535	0.86937	0.87370	0.87849
551	0.85255	0.86057	0.86430	0.86554	0.86955	0.87388	0.87866
552	0.85278	0.86077	0.86449	0.86573	0.86974	0.87406	0.87883
553	0.85298	0.86097	0.86468	0.86592	0.86993	0.87424	0.87900
554	0.85318	0.86117	0.86487	0.86611	0.87011	0.87442	0.87917
555	0.85339	0.86137	0.86507	0.86631	0.87029	0.87459	0.87934
556	0.85361	0.86156	0.86526	0.86649	0.87047	0.87477	0.87950
557	0.85383	0.86176	0.86545	0.86668	0.87065	0.87494	0.87967
558	0.85404	0.86196	0.86564	0.86687	0.87083	0.87512	0.87984
559	0.85424	0.86215	0.86583	0.86705	0.87101	0.87529	0.88001
560	0.85445	0.86235	0.86602	0.86724	0.87120	0.87547	0.88017
561	0.85464	0.86254	0.86621	0.86743	0.87138	0.87564	0.88034
562	0.85485	0.86272	0.86639	0.86761	0.87156	0.87581	0.88050
563	0.85504	0.86292	0.86658	0.86780	0.87173	0.87599	0.88067
564	0.85525	0.86311	0.86677	0.86798	0.87191	0.87616	0.88083
565	0.85544	0.86331	0.86696	0.86817	0.87209	0.87633	0.88099
566	0.85565	0.86351	0.86714	0.86836	0.87227	0.87650	0.88116
567	0.85587	0.86370	0.86732	0.86854	0.87244	0.87667	0.88132
568	0.85606	0.86389	0.86751	0.86872	0.87262	0.87684	0.88148
569	0.85626	0.86408	0.86769	0.86889	0.87279	0.87701	0.88164
570	0.85645	0.86426	0.86788	0.86907	0.87297	0.87718	0.88181
571	0.85664	0.86446	0.86806	0.86925	0.87314	0.87734	0.88197
572	0.85685	0.86463	0.86825	0.86944	0.87331	0.87751	0.88213
573	0.85706	0.86483	0.86842	0.86962	0.87349	0.87768	0.88229
574	0.85725	0.86500	0.86860	0.86980	0.87366	0.87784	0.88245
575	0.85744	0.86519	0.86878	0.86997	0.87383	0.87801	0.88260
576	0.85766	0.86537	0.86896	0.87015	0.87400	0.87817	0.88276
577	0.85784	0.86556	0.86914	0.87032	0.87418	0.87833	0.88292
578	0.85803	0.86574	0.86932	0.87050	0.87435	0.87850	0.88308
579	0.85823	0.86592	0.86949	0.87068	0.87452	0.87866	0.88324
580	0.85843	0.86611	0.86966	0.87086	0.87469	0.87882	0.88339
581	0.85863	0.86629	0.86984	0.87103	0.87486	0.87899	0.88355
582	0.85883	0.86647	0.87002	0.87120	0.87503	0.87915	0.88370
583	0.85900	0.86665	0.87019	0.87137	0.87519	0.87931	0.88386
584	0.85920	0.86683	0.87037	0.87154	0.87536	0.87947	0.88401
585	0.85938	0.86701	0.87054	0.87172	0.87553	0.87964	0.88417
586	0.85955	0.86719	0.87071	0.87189	0.87570	0.87980	0.88432
587	0.85975	0.86737	0.87089	0.87206	0.87587	0.87995	0.88447
588	0.85995	0.86755	0.87106	0.87223	0.87603	0.88011	0.88463
589	0.86015	0.86773	0.87123	0.87240	0.87620	0.88027	0.88478
590	0.86033	0.86790	0.87140	0.87257	0.87636	0.88043	0.88493
591	0.86052	0.86807	0.87158	0.87274	0.87653	0.88059	0.88508

N	0.1%	0.5%	1%	1.25%	2.5%	5%	10%
592	0.86070	0.86825	0.87175	0.87292	0.87669	0.88074	0.88523
593	0.86089	0.86842	0.87193	0.87309	0.87685	0.88090	0.88538
594	0.86107	0.86860	0.87209	0.87325	0.87701	0.88106	0.88554
595	0.86126	0.86877	0.87226	0.87342	0.87717	0.88122	0.88568
596	0.86144	0.86895	0.87243	0.87359	0.87734	0.88137	0.88584
597	0.86163	0.86912	0.87260	0.87376	0.87750	0.88152	0.88598
598	0.86181	0.86929	0.87277	0.87393	0.87766	0.88168	0.88613
599	0.86201	0.86946	0.87294	0.87409	0.87782	0.88183	0.88628
600	0.86217	0.86964	0.87310	0.87426	0.87798	0.88199	0.88643
601	0.86235	0.86980	0.87327	0.87442	0.87814	0.88214	0.88657
602	0.86252	0.86997	0.87343	0.87458	0.87830	0.88229	0.88672
603	0.86272	0.87014	0.87360	0.87475	0.87845	0.88245	0.88687
604	0.86289	0.87032	0.87377	0.87491	0.87861	0.88260	0.88701
605	0.86306	0.87048	0.87393	0.87508	0.87877	0.88275	0.88716
606	0.86324	0.87065	0.87409	0.87524	0.87892	0.88290	0.88730
607	0.86342	0.87082	0.87426	0.87540	0.87908	0.88305	0.88745
608	0.86360	0.87099	0.87443	0.87556	0.87924	0.88320	0.88759
609	0.86376	0.87115	0.87459	0.87572	0.87939	0.88335	0.88773
610	0.86393	0.87132	0.87474	0.87589	0.87955	0.88351	0.88788
611	0.86410	0.87149	0.87491	0.87604	0.87971	0.88365	0.88802
612	0.86429	0.87166	0.87507	0.87621	0.87986	0.88380	0.88816
613	0.86448	0.87182	0.87523	0.87637	0.88002	0.88395	0.88831
614	0.86465	0.87198	0.87539	0.87652	0.88017	0.88410	0.88845
615	0.86483	0.87215	0.87555	0.87668	0.88032	0.88425	0.88859
616	0.86500	0.87232	0.87570	0.87684	0.88048	0.88440	0.88873
617	0.86518	0.87248	0.87586	0.87699	0.88063	0.88455	0.88887
618	0.86536	0.87266	0.87603	0.87715	0.88079	0.88469	0.88901
619	0.86554	0.87281	0.87619	0.87731	0.88094	0.88484	0.88915
620	0.86572	0.87298	0.87635	0.87747	0.88109	0.88499	0.88929
621	0.86590	0.87315	0.87650	0.87763	0.88124	0.88513	0.88943
622	0.86605	0.87331	0.87665	0.87778	0.88139	0.88528	0.88957
623	0.86623	0.87347	0.87681	0.87794	0.88154	0.88542	0.88971
624	0.86641	0.87363	0.87697	0.87809	0.88169	0.88556	0.88985
625	0.86657	0.87379	0.87712	0.87824	0.88184	0.88571	0.88999
626	0.86674	0.87395	0.87727	0.87839	0.88199	0.88585	0.89012
627	0.86691	0.87411	0.87743	0.87854	0.88214	0.88600	0.89026
628	0.86708	0.87427	0.87759	0.87870	0.88229	0.88614	0.89040
629	0.86725	0.87442	0.87775	0.87885	0.88243	0.88628	0.89053
630	0.86741	0.87458	0.87790	0.87900	0.88258	0.88642	0.89067
631	0.86758	0.87474	0.87805	0.87915	0.88273	0.88657	0.89081
632	0.86775	0.87490	0.87820	0.87930	0.88287	0.88671	0.89094
633	0.86793	0.87505	0.87835	0.87945	0.88302	0.88685	0.89108
634	0.86809	0.87521	0.87850	0.87960	0.88316	0.88699	0.89121
635	0.86825	0.87537	0.87865	0.87975	0.88331	0.88713	0.89134
636	0.86843	0.87553	0.87881	0.87990	0.88346	0.88727	0.89148
637	0.86860	0.87568	0.87895	0.88005	0.88360	0.88741	0.89161
638	0.86877	0.87584	0.87910	0.88020	0.88374	0.88755	0.89175
639	0.86893	0.87599	0.87925	0.88035	0.88389	0.88768	0.89188
640	0.86909	0.87614	0.87940	0.88050	0.88403	0.88782	0.89201
641	0.86925	0.87630	0.87954	0.88065	0.88417	0.88796	0.89214
642	0.86941	0.87645	0.87969	0.88080	0.88431	0.88809	0.89227
643	0.86958	0.87660	0.87984	0.88094	0.88445	0.88823	0.89240
644	0.86973	0.87675	0.87999	0.88109	0.88459	0.88837	0.89253
645	0.86989	0.87689	0.88014	0.88123	0.88473	0.88851	0.89266
646	0.87005	0.87705	0.88028	0.88137	0.88487	0.88864	0.89279
647	0.87021	0.87720	0.88043	0.88151	0.88501	0.88877	0.89292
648	0.87037	0.87735	0.88058	0.88166	0.88515	0.88891	0.89305
649	0.87053	0.87751	0.88072	0.88180	0.88529	0.88904	0.89318
650	0.87069	0.87766	0.88086	0.88195	0.88543	0.88918	0.89331
651	0.87084	0.87781	0.88101	0.88209	0.88557	0.88931	0.89344
652	0.87099	0.87795	0.88116	0.88223	0.88571	0.88945	0.89356
653	0.87117	0.87810	0.88131	0.88238	0.88585	0.88958	0.89369
654	0.87134	0.87825	0.88145	0.88252	0.88599	0.88971	0.89382
655	0.87149	0.87840	0.88159	0.88267	0.88612	0.88985	0.89395
656	0.87165	0.87855	0.88174	0.88281	0.88626	0.88998	0.89408
657	0.87179	0.87869	0.88188	0.88295	0.88640	0.89011	0.89420
658	0.87195	0.87884	0.88202	0.88309	0.88654	0.89024	0.89433
659	0.87210	0.87899	0.88216	0.88323	0.88667	0.89037	0.89445
660	0.87225	0.87913	0.88231	0.88337	0.88681	0.89051	0.89458
661	0.87240	0.87928	0.88245	0.88351	0.88694	0.89064	0.89470
662	0.87257	0.87942	0.88259	0.88365	0.88708	0.89077	0.89483
663	0.87272	0.87957	0.88273	0.88379	0.88721	0.89090	0.89495
664	0.87286	0.87972	0.88287	0.88393	0.88734	0.89102	0.89508
665	0.87301	0.87985	0.88301	0.88407	0.88748	0.89115	0.89520

N	0.1%	0.5%	1%	1.25%	2.5%	5%	10%
666	0.87315	0.88000	0.88315	0.88421	0.88761	0.89128	0.89532
667	0.87332	0.88015	0.88329	0.88435	0.88775	0.89141	0.89544
668	0.87347	0.88029	0.88343	0.88449	0.88788	0.89154	0.89557
669	0.87362	0.88043	0.88357	0.88463	0.88801	0.89166	0.89569
670	0.87378	0.88057	0.88370	0.88476	0.88814	0.89179	0.89581
671	0.87391	0.88072	0.88384	0.88489	0.88828	0.89192	0.89593
672	0.87406	0.88085	0.88398	0.88502	0.88841	0.89204	0.89605
673	0.87422	0.88100	0.88412	0.88516	0.88854	0.89217	0.89617
674	0.87436	0.88114	0.88425	0.88530	0.88867	0.89229	0.89629
675	0.87452	0.88128	0.88439	0.88543	0.88881	0.89242	0.89642
676	0.87467	0.88142	0.88453	0.88557	0.88894	0.89255	0.89654
677	0.87482	0.88156	0.88467	0.88571	0.88907	0.89267	0.89666
678	0.87497	0.88170	0.88480	0.88584	0.88919	0.89280	0.89678
679	0.87513	0.88184	0.88494	0.88597	0.88932	0.89292	0.89690
680	0.87528	0.88198	0.88507	0.88610	0.88945	0.89304	0.89702
681	0.87542	0.88212	0.88521	0.88624	0.88958	0.89317	0.89713
682	0.87556	0.88226	0.88534	0.88638	0.88971	0.89329	0.89725
683	0.87571	0.88240	0.88548	0.88651	0.88984	0.89342	0.89737
684	0.87586	0.88253	0.88561	0.88664	0.88997	0.89354	0.89749
685	0.87600	0.88266	0.88574	0.88677	0.89010	0.89367	0.89761
686	0.87616	0.88280	0.88587	0.88690	0.89022	0.89379	0.89772
687	0.87630	0.88294	0.88601	0.88703	0.89035	0.89391	0.89784
688	0.87645	0.88308	0.88614	0.88716	0.89047	0.89403	0.89796
689	0.87659	0.88321	0.88627	0.88729	0.89060	0.89415	0.89807
690	0.87672	0.88334	0.88640	0.88742	0.89072	0.89427	0.89819
691	0.87687	0.88347	0.88653	0.88755	0.89085	0.89439	0.89830
692	0.87701	0.88360	0.88666	0.88768	0.89097	0.89451	0.89842
693	0.87717	0.88374	0.88679	0.88780	0.89110	0.89463	0.89853
694	0.87732	0.88387	0.88692	0.88793	0.89122	0.89475	0.89865
695	0.87745	0.88401	0.88705	0.88806	0.89135	0.89487	0.89876
696	0.87760	0.88414	0.88718	0.88819	0.89147	0.89499	0.89888
697	0.87774	0.88428	0.88731	0.88832	0.89159	0.89511	0.89899
698	0.87789	0.88440	0.88743	0.88845	0.89172	0.89523	0.89911
699	0.87803	0.88454	0.88756	0.88858	0.89184	0.89534	0.89922
700	0.87816	0.88467	0.88769	0.88870	0.89196	0.89546	0.89933
701	0.87830	0.88481	0.88782	0.88883	0.89209	0.89558	0.89944
702	0.87842	0.88494	0.88795	0.88895	0.89221	0.89570	0.89956
703	0.87856	0.88507	0.88808	0.88908	0.89233	0.89582	0.89967
704	0.87870	0.88520	0.88820	0.88921	0.89245	0.89593	0.89978
705	0.87883	0.88533	0.88832	0.88933	0.89257	0.89605	0.89989
706	0.87896	0.88546	0.88845	0.88946	0.89269	0.89617	0.90001
707	0.87908	0.88559	0.88857	0.88958	0.89282	0.89628	0.90012
708	0.87922	0.88572	0.88870	0.88970	0.89294	0.89640	0.90023
709	0.87936	0.88585	0.88883	0.88983	0.89306	0.89651	0.90034
710	0.87950	0.88597	0.88895	0.88995	0.89318	0.89663	0.90045
711	0.87967	0.88610	0.88908	0.89007	0.89329	0.89674	0.90056
712	0.87980	0.88623	0.88920	0.89019	0.89341	0.89686	0.90067
713	0.87992	0.88635	0.88932	0.89032	0.89353	0.89697	0.90078
714	0.88005	0.88648	0.88944	0.89044	0.89365	0.89709	0.90089
715	0.88018	0.88660	0.88956	0.89056	0.89377	0.89720	0.90100
716	0.88030	0.88673	0.88969	0.89068	0.89389	0.89732	0.90111
717	0.88044	0.88686	0.88981	0.89080	0.89400	0.89743	0.90121
718	0.88059	0.88699	0.88994	0.89093	0.89412	0.89754	0.90132
719	0.88072	0.88711	0.89006	0.89105	0.89424	0.89765	0.90143
720	0.88087	0.88724	0.89018	0.89117	0.89435	0.89777	0.90154
721	0.88100	0.88737	0.89030	0.89129	0.89447	0.89788	0.90165
722	0.88112	0.88749	0.89042	0.89141	0.89458	0.89799	0.90175
723	0.88125	0.88762	0.89055	0.89153	0.89470	0.89810	0.90186
724	0.88139	0.88774	0.89067	0.89165	0.89481	0.89821	0.90197
725	0.88152	0.88786	0.89079	0.89177	0.89493	0.89832	0.90207
726	0.88165	0.88799	0.89091	0.89189	0.89504	0.89844	0.90218
727	0.88179	0.88811	0.89103	0.89201	0.89516	0.89855	0.90228
728	0.88192	0.88824	0.89115	0.89212	0.89528	0.89866	0.90239
729	0.88204	0.88836	0.89127	0.89224	0.89539	0.89876	0.90249
730	0.88216	0.88849	0.89139	0.89236	0.89550	0.89887	0.90260
731	0.88231	0.88861	0.89151	0.89247	0.89562	0.89898	0.90270
732	0.88243	0.88873	0.89163	0.89259	0.89573	0.89909	0.90281
733	0.88256	0.88885	0.89175	0.89271	0.89584	0.89920	0.90291
734	0.88269	0.88898	0.89186	0.89283	0.89596	0.89931	0.90302
735	0.88284	0.88910	0.89198	0.89294	0.89607	0.89942	0.90312
736	0.88296	0.88922	0.89210	0.89306	0.89618	0.89953	0.90323
737	0.88309	0.88933	0.89222	0.89318	0.89629	0.89964	0.90333
738	0.88322	0.88945	0.89234	0.89330	0.89640	0.89974	0.90343
739	0.88334	0.88958	0.89245	0.89341	0.89652	0.89985	0.90354

N	0.1%	0.5%	1%	1.25%	2.5%	5%	10%
740	0.88347	0.88970	0.89257	0.89353	0.89663	0.89996	0.90364
741	0.88360	0.88981	0.89269	0.89364	0.89674	0.90006	0.90374
742	0.88372	0.88993	0.89281	0.89376	0.89685	0.90017	0.90384
743	0.88385	0.89005	0.89292	0.89387	0.89696	0.90028	0.90395
744	0.88396	0.89017	0.89303	0.89398	0.89707	0.90039	0.90405
745	0.88409	0.89029	0.89315	0.89409	0.89718	0.90049	0.90415
746	0.88422	0.89041	0.89326	0.89421	0.89729	0.90060	0.90425
747	0.88436	0.89053	0.89337	0.89433	0.89740	0.90070	0.90435
748	0.88448	0.89064	0.89349	0.89444	0.89751	0.90080	0.90445
749	0.88460	0.89076	0.89361	0.89455	0.89761	0.90091	0.90455
750	0.88473	0.89088	0.89371	0.89466	0.89772	0.90101	0.90465
751	0.88486	0.89100	0.89383	0.89478	0.89783	0.90112	0.90475
752	0.88497	0.89111	0.89395	0.89489	0.89794	0.90122	0.90485
753	0.88510	0.89122	0.89406	0.89500	0.89805	0.90133	0.90495
754	0.88522	0.89133	0.89417	0.89511	0.89816	0.90143	0.90505
755	0.88534	0.89145	0.89428	0.89522	0.89827	0.90153	0.90515
756	0.88547	0.89157	0.89439	0.89533	0.89838	0.90164	0.90525
757	0.88559	0.89168	0.89451	0.89545	0.89848	0.90174	0.90535
758	0.88572	0.89180	0.89462	0.89556	0.89859	0.90185	0.90545
759	0.88584	0.89191	0.89473	0.89567	0.89870	0.90195	0.90555
760	0.88595	0.89203	0.89484	0.89578	0.89880	0.90205	0.90565
761	0.88607	0.89214	0.89496	0.89589	0.89891	0.90215	0.90574
762	0.88620	0.89226	0.89506	0.89600	0.89902	0.90226	0.90584
763	0.88630	0.89237	0.89517	0.89611	0.89912	0.90236	0.90594
764	0.88643	0.89249	0.89528	0.89622	0.89922	0.90246	0.90604
765	0.88655	0.89259	0.89539	0.89632	0.89933	0.90256	0.90613
766	0.88666	0.89271	0.89550	0.89643	0.89944	0.90266	0.90623
767	0.88678	0.89282	0.89561	0.89654	0.89954	0.90276	0.90633
768	0.88691	0.89294	0.89572	0.89664	0.89964	0.90286	0.90642
769	0.88703	0.89305	0.89583	0.89675	0.89974	0.90296	0.90652
770	0.88716	0.89316	0.89594	0.89686	0.89985	0.90306	0.90661
771	0.88727	0.89327	0.89605	0.89697	0.89995	0.90316	0.90671
772	0.88740	0.89338	0.89615	0.89707	0.90006	0.90326	0.90680
773	0.88751	0.89349	0.89626	0.89718	0.90016	0.90336	0.90690
774	0.88764	0.89359	0.89637	0.89729	0.90026	0.90346	0.90699
775	0.88775	0.89371	0.89647	0.89739	0.90036	0.90355	0.90709
776	0.88788	0.89382	0.89658	0.89750	0.90047	0.90365	0.90718
777	0.88800	0.89393	0.89669	0.89761	0.90057	0.90375	0.90728
778	0.88812	0.89404	0.89679	0.89771	0.90067	0.90385	0.90737
779	0.88823	0.89414	0.89690	0.89781	0.90077	0.90395	0.90746
780	0.88834	0.89425	0.89701	0.89792	0.90087	0.90405	0.90756
781	0.88845	0.89437	0.89711	0.89802	0.90097	0.90414	0.90765
782	0.88857	0.89447	0.89721	0.89813	0.90107	0.90424	0.90775
783	0.88869	0.89458	0.89732	0.89824	0.90118	0.90434	0.90784
784	0.88880	0.89469	0.89743	0.89834	0.90128	0.90443	0.90793
785	0.88892	0.89480	0.89753	0.89845	0.90138	0.90453	0.90802
786	0.88901	0.89491	0.89763	0.89855	0.90148	0.90463	0.90812
787	0.88913	0.89502	0.89774	0.89865	0.90158	0.90472	0.90821
788	0.88924	0.89512	0.89784	0.89875	0.90168	0.90482	0.90830
789	0.88935	0.89523	0.89795	0.89886	0.90178	0.90492	0.90839
790	0.88946	0.89534	0.89805	0.89896	0.90188	0.90501	0.90848
791	0.88957	0.89544	0.89815	0.89906	0.90197	0.90511	0.90858
792	0.88970	0.89555	0.89826	0.89917	0.90207	0.90520	0.90867
793	0.88981	0.89566	0.89836	0.89927	0.90217	0.90530	0.90876
794	0.88992	0.89577	0.89846	0.89937	0.90227	0.90539	0.90885
795	0.89003	0.89587	0.89857	0.89947	0.90237	0.90549	0.90894
796	0.89015	0.89597	0.89867	0.89957	0.90247	0.90558	0.90903
797	0.89025	0.89608	0.89877	0.89967	0.90257	0.90568	0.90912
798	0.89036	0.89619	0.89887	0.89977	0.90266	0.90577	0.90921
799	0.89047	0.89629	0.89898	0.89987	0.90276	0.90587	0.90930
800	0.89058	0.89639	0.89908	0.89997	0.90285	0.90596	0.90939
801	0.89070	0.89649	0.89918	0.90007	0.90295	0.90605	0.90948
802	0.89081	0.89660	0.89927	0.90017	0.90305	0.90615	0.90957
803	0.89091	0.89670	0.89938	0.90027	0.90315	0.90624	0.90966
804	0.89102	0.89680	0.89948	0.90037	0.90324	0.90633	0.90975
805	0.89113	0.89691	0.89957	0.90047	0.90334	0.90642	0.90984
806	0.89124	0.89701	0.89967	0.90057	0.90343	0.90652	0.90993
807	0.89135	0.89711	0.89977	0.90067	0.90353	0.90661	0.91002
808	0.89146	0.89721	0.89988	0.90077	0.90362	0.90670	0.91011
809	0.89155	0.89732	0.89998	0.90087	0.90372	0.90679	0.91019
810	0.89166	0.89742	0.90008	0.90097	0.90381	0.90688	0.91028
811	0.89178	0.89752	0.90017	0.90106	0.90391	0.90697	0.91037
812	0.89188	0.89763	0.90027	0.90116	0.90401	0.90707	0.91046
813	0.89199	0.89773	0.90037	0.90126	0.90410	0.90716	0.91054

N	0.1%	0.5%	1%	1.25%	2.5%	5%	10%
814	0.89210	0.89783	0.90047	0.90135	0.90419	0.90725	0.91063
815	0.89220	0.89793	0.90057	0.90145	0.90429	0.90734	0.91071
816	0.89232	0.89804	0.90067	0.90155	0.90438	0.90743	0.91080
817	0.89242	0.89814	0.90076	0.90164	0.90448	0.90752	0.91089
818	0.89253	0.89824	0.90086	0.90173	0.90457	0.90761	0.91097
819	0.89263	0.89834	0.90095	0.90183	0.90466	0.90770	0.91106
820	0.89274	0.89844	0.90105	0.90193	0.90476	0.90779	0.91115
821	0.89284	0.89854	0.90115	0.90203	0.90485	0.90788	0.91123
822	0.89296	0.89864	0.90125	0.90212	0.90494	0.90797	0.91132
823	0.89307	0.89874	0.90134	0.90222	0.90504	0.90806	0.91140
824	0.89318	0.89884	0.90144	0.90231	0.90513	0.90814	0.91149
825	0.89329	0.89893	0.90153	0.90240	0.90522	0.90823	0.91157
826	0.89339	0.89903	0.90163	0.90250	0.90531	0.90832	0.91166
827	0.89349	0.89913	0.90172	0.90260	0.90540	0.90841	0.91174
828	0.89361	0.89923	0.90181	0.90269	0.90549	0.90850	0.91183
829	0.89372	0.89932	0.90191	0.90278	0.90559	0.90859	0.91191
830	0.89381	0.89942	0.90200	0.90288	0.90568	0.90867	0.91200
831	0.89393	0.89952	0.90210	0.90297	0.90577	0.90876	0.91208
832	0.89402	0.89961	0.90219	0.90306	0.90586	0.90885	0.91216
833	0.89412	0.89971	0.90228	0.90315	0.90595	0.90894	0.91225
834	0.89422	0.89981	0.90238	0.90325	0.90604	0.90902	0.91233
835	0.89434	0.89991	0.90247	0.90334	0.90613	0.90911	0.91241
836	0.89443	0.90001	0.90257	0.90343	0.90622	0.90920	0.91250
837	0.89454	0.90010	0.90266	0.90352	0.90631	0.90928	0.91258
838	0.89463	0.90020	0.90276	0.90362	0.90640	0.90937	0.91266
839	0.89474	0.90029	0.90285	0.90371	0.90649	0.90946	0.91274
840	0.89484	0.90039	0.90294	0.90380	0.90658	0.90954	0.91283
841	0.89494	0.90049	0.90304	0.90390	0.90667	0.90963	0.91291
842	0.89503	0.90058	0.90313	0.90399	0.90676	0.90971	0.91299
843	0.89514	0.90068	0.90323	0.90408	0.90685	0.90980	0.91307
844	0.89525	0.90077	0.90332	0.90417	0.90693	0.90988	0.91315
845	0.89535	0.90087	0.90341	0.90426	0.90702	0.90997	0.91324
846	0.89545	0.90096	0.90350	0.90435	0.90711	0.91006	0.91332
847	0.89556	0.90105	0.90359	0.90444	0.90720	0.91014	0.91340
848	0.89565	0.90114	0.90368	0.90453	0.90728	0.91022	0.91348
849	0.89576	0.90123	0.90377	0.90462	0.90737	0.91031	0.91356
850	0.89585	0.90133	0.90386	0.90471	0.90746	0.91039	0.91364
851	0.89594	0.90142	0.90395	0.90480	0.90755	0.91048	0.91372
852	0.89605	0.90152	0.90405	0.90489	0.90763	0.91056	0.91381
853	0.89614	0.90161	0.90414	0.90499	0.90772	0.91065	0.91388
854	0.89624	0.90170	0.90423	0.90507	0.90780	0.91073	0.91396
855	0.89634	0.90180	0.90432	0.90516	0.90789	0.91081	0.91405
856	0.89644	0.90189	0.90441	0.90525	0.90798	0.91090	0.91412
857	0.89655	0.90199	0.90450	0.90534	0.90807	0.91098	0.91421
858	0.89665	0.90208	0.90459	0.90543	0.90815	0.91106	0.91428
859	0.89675	0.90218	0.90468	0.90552	0.90824	0.91114	0.91436
860	0.89685	0.90227	0.90477	0.90561	0.90832	0.91123	0.91444
861	0.89693	0.90236	0.90486	0.90570	0.90841	0.91131	0.91452
862	0.89703	0.90245	0.90495	0.90578	0.90849	0.91139	0.91460
863	0.89712	0.90254	0.90504	0.90587	0.90858	0.91147	0.91468
864	0.89721	0.90263	0.90513	0.90596	0.90866	0.91156	0.91476
865	0.89731	0.90272	0.90521	0.90605	0.90874	0.91164	0.91484
866	0.89740	0.90281	0.90530	0.90614	0.90883	0.91172	0.91491
867	0.89751	0.90290	0.90539	0.90622	0.90891	0.91180	0.91499
868	0.89761	0.90299	0.90548	0.90631	0.90900	0.91188	0.91507
869	0.89769	0.90308	0.90557	0.90640	0.90908	0.91197	0.91515
870	0.89779	0.90318	0.90566	0.90648	0.90917	0.91205	0.91523
871	0.89789	0.90326	0.90574	0.90657	0.90925	0.91213	0.91530
872	0.89799	0.90335	0.90582	0.90665	0.90934	0.91221	0.91538
873	0.89808	0.90345	0.90591	0.90674	0.90942	0.91229	0.91546
874	0.89817	0.90354	0.90600	0.90683	0.90950	0.91237	0.91554
875	0.89826	0.90363	0.90609	0.90691	0.90959	0.91245	0.91561
876	0.89836	0.90371	0.90617	0.90700	0.90967	0.91253	0.91569
877	0.89845	0.90380	0.90626	0.90708	0.90975	0.91261	0.91577
878	0.89854	0.90389	0.90635	0.90717	0.90983	0.91269	0.91584
879	0.89865	0.90398	0.90643	0.90725	0.90992	0.91277	0.91592
880	0.89874	0.90407	0.90651	0.90734	0.91000	0.91285	0.91599
881	0.89885	0.90415	0.90660	0.90742	0.91008	0.91293	0.91607
882	0.89894	0.90423	0.90668	0.90751	0.91016	0.91301	0.91615
883	0.89904	0.90432	0.90677	0.90759	0.91024	0.91309	0.91622
884	0.89912	0.90441	0.90685	0.90767	0.91033	0.91316	0.91630
885	0.89921	0.90450	0.90694	0.90776	0.91041	0.91324	0.91637
886	0.89929	0.90458	0.90702	0.90784	0.91049	0.91332	0.91645
887	0.89938	0.90467	0.90711	0.90792	0.91057	0.91340	0.91653

N	0.1%	0.5%	1%	1.25%	2.5%	5%	10%
888	0.89947	0.90475	0.90719	0.90801	0.91065	0.91348	0.91660
889	0.89957	0.90484	0.90728	0.90809	0.91073	0.91355	0.91667
890	0.89966	0.90493	0.90737	0.90817	0.91081	0.91363	0.91675
891	0.89975	0.90501	0.90745	0.90826	0.91089	0.91371	0.91682
892	0.89984	0.90510	0.90754	0.90834	0.91097	0.91379	0.91690
893	0.89994	0.90518	0.90762	0.90842	0.91105	0.91386	0.91697
894	0.90003	0.90527	0.90770	0.90850	0.91113	0.91394	0.91704
895	0.90011	0.90536	0.90779	0.90859	0.91121	0.91402	0.91712
896	0.90020	0.90545	0.90787	0.90867	0.91129	0.91409	0.91719
897	0.90029	0.90553	0.90795	0.90875	0.91137	0.91417	0.91726
898	0.90039	0.90562	0.90803	0.90883	0.91145	0.91425	0.91734
899	0.90047	0.90570	0.90812	0.90891	0.91153	0.91433	0.91741
900	0.90057	0.90579	0.90820	0.90900	0.91161	0.91440	0.91748
901	0.90065	0.90588	0.90828	0.90908	0.91169	0.91448	0.91756
902	0.90074	0.90596	0.90836	0.90916	0.91177	0.91455	0.91763
903	0.90083	0.90605	0.90845	0.90924	0.91184	0.91463	0.91770
904	0.90092	0.90613	0.90853	0.90932	0.91192	0.91471	0.91778
905	0.90101	0.90621	0.90861	0.90941	0.91200	0.91478	0.91785
906	0.90109	0.90630	0.90870	0.90949	0.91208	0.91485	0.91792
907	0.90118	0.90639	0.90877	0.90957	0.91216	0.91493	0.91799
908	0.90127	0.90647	0.90886	0.90965	0.91223	0.91500	0.91806
909	0.90135	0.90655	0.90894	0.90973	0.91231	0.91508	0.91814
910	0.90144	0.90663	0.90902	0.90981	0.91239	0.91515	0.91821
911	0.90154	0.90671	0.90910	0.90989	0.91247	0.91523	0.91828
912	0.90162	0.90680	0.90919	0.90997	0.91254	0.91530	0.91835
913	0.90171	0.90688	0.90927	0.91005	0.91262	0.91538	0.91842
914	0.90179	0.90696	0.90935	0.91013	0.91270	0.91545	0.91849
915	0.90189	0.90705	0.90942	0.91021	0.91277	0.91553	0.91856
916	0.90197	0.90713	0.90951	0.91029	0.91285	0.91560	0.91864
917	0.90205	0.90721	0.90958	0.91037	0.91293	0.91567	0.91871
918	0.90214	0.90729	0.90966	0.91045	0.91300	0.91575	0.91878
919	0.90223	0.90737	0.90974	0.91053	0.91308	0.91582	0.91885
920	0.90232	0.90746	0.90982	0.91060	0.91315	0.91590	0.91892
921	0.90241	0.90754	0.90990	0.91068	0.91323	0.91597	0.91899
922	0.90250	0.90762	0.90998	0.91076	0.91330	0.91604	0.91906
923	0.90259	0.90770	0.91006	0.91084	0.91338	0.91611	0.91913
924	0.90268	0.90778	0.91014	0.91092	0.91346	0.91619	0.91920
925	0.90277	0.90786	0.91022	0.91100	0.91353	0.91626	0.91927
926	0.90285	0.90794	0.91029	0.91107	0.91361	0.91633	0.91934
927	0.90294	0.90802	0.91037	0.91115	0.91368	0.91640	0.91941
928	0.90304	0.90810	0.91045	0.91123	0.91375	0.91648	0.91948
929	0.90311	0.90818	0.91053	0.91130	0.91383	0.91655	0.91955
930	0.90319	0.90827	0.91061	0.91138	0.91390	0.91662	0.91961
931	0.90328	0.90835	0.91068	0.91146	0.91398	0.91669	0.91968
932	0.90337	0.90842	0.91076	0.91153	0.91405	0.91677	0.91975
933	0.90345	0.90851	0.91084	0.91161	0.91413	0.91684	0.91982
934	0.90354	0.90859	0.91092	0.91169	0.91420	0.91691	0.91989
935	0.90363	0.90867	0.91100	0.91176	0.91427	0.91698	0.91996
936	0.90372	0.90875	0.91108	0.91184	0.91435	0.91705	0.92003
937	0.90381	0.90882	0.91115	0.91192	0.91442	0.91712	0.92009
938	0.90389	0.90890	0.91123	0.91199	0.91450	0.91719	0.92016
939	0.90397	0.90898	0.91131	0.91207	0.91457	0.91726	0.92023
940	0.90404	0.90906	0.91139	0.91214	0.91464	0.91733	0.92030
941	0.90413	0.90914	0.91146	0.91222	0.91472	0.91741	0.92036
942	0.90421	0.90922	0.91154	0.91230	0.91479	0.91747	0.92043
943	0.90429	0.90930	0.91162	0.91237	0.91486	0.91755	0.92050
944	0.90438	0.90937	0.91169	0.91245	0.91494	0.91762	0.92057
945	0.90446	0.90945	0.91176	0.91252	0.91501	0.91769	0.92063
946	0.90454	0.90953	0.91184	0.91260	0.91508	0.91776	0.92070
947	0.90462	0.90960	0.91191	0.91267	0.91515	0.91783	0.92077
948	0.90470	0.90969	0.91199	0.91275	0.91523	0.91790	0.92083
949	0.90479	0.90976	0.91206	0.91282	0.91530	0.91796	0.92090
950	0.90487	0.90984	0.91214	0.91289	0.91537	0.91803	0.92097
951	0.90495	0.90991	0.91221	0.91297	0.91544	0.91810	0.92103
952	0.90503	0.90999	0.91229	0.91304	0.91551	0.91817	0.92110
953	0.90511	0.91007	0.91236	0.91311	0.91559	0.91824	0.92117
954	0.90518	0.91014	0.91244	0.91319	0.91566	0.91831	0.92123
955	0.90527	0.91022	0.91251	0.91326	0.91573	0.91838	0.92130
956	0.90535	0.91029	0.91258	0.91333	0.91580	0.91845	0.92136
957	0.90543	0.91037	0.91266	0.91341	0.91587	0.91852	0.92143
958	0.90551	0.91045	0.91273	0.91348	0.91594	0.91859	0.92149
959	0.90559	0.91053	0.91281	0.91356	0.91601	0.91866	0.92156
960	0.90567	0.91060	0.91288	0.91363	0.91608	0.91872	0.92163
961	0.90575	0.91068	0.91295	0.91370	0.91615	0.91879	0.92169

N	0.1%	0.5%	1%	1.25%	2.5%	5%	10%
962	0.90584	0.91075	0.91303	0.91378	0.91622	0.91886	0.92176
963	0.90592	0.91083	0.91310	0.91385	0.91629	0.91893	0.92182
964	0.90600	0.91090	0.91317	0.91392	0.91636	0.91899	0.92189
965	0.90608	0.91098	0.91325	0.91399	0.91643	0.91906	0.92195
966	0.90616	0.91105	0.91332	0.91407	0.91650	0.91913	0.92201
967	0.90624	0.91112	0.91339	0.91414	0.91657	0.91920	0.92208
968	0.90631	0.91120	0.91346	0.91421	0.91664	0.91926	0.92214
969	0.90640	0.91128	0.91354	0.91428	0.91671	0.91933	0.92221
970	0.90647	0.91136	0.91361	0.91435	0.91678	0.91939	0.92227
971	0.90655	0.91143	0.91368	0.91442	0.91685	0.91946	0.92234
972	0.90663	0.91150	0.91375	0.91450	0.91692	0.91953	0.92240
973	0.90672	0.91158	0.91383	0.91457	0.91699	0.91960	0.92246
974	0.90680	0.91165	0.91390	0.91464	0.91706	0.91966	0.92253
975	0.90687	0.91173	0.91397	0.91471	0.91712	0.91973	0.92259
976	0.90695	0.91180	0.91404	0.91478	0.91719	0.91979	0.92266
977	0.90702	0.91187	0.91411	0.91485	0.91726	0.91986	0.92272
978	0.90711	0.91194	0.91418	0.91492	0.91733	0.91993	0.92278
979	0.90718	0.91202	0.91425	0.91499	0.91740	0.91999	0.92284
980	0.90726	0.91209	0.91432	0.91506	0.91747	0.92006	0.92291
981	0.90733	0.91216	0.91439	0.91513	0.91753	0.92013	0.92297
982	0.90741	0.91224	0.91446	0.91520	0.91760	0.92019	0.92303
983	0.90749	0.91231	0.91453	0.91527	0.91767	0.92026	0.92310
984	0.90757	0.91238	0.91460	0.91534	0.91773	0.92032	0.92316
985	0.90765	0.91246	0.91467	0.91541	0.91780	0.92039	0.92322
986	0.90772	0.91253	0.91474	0.91548	0.91787	0.92045	0.92328
987	0.90781	0.91260	0.91481	0.91556	0.91794	0.92051	0.92335
988	0.90788	0.91267	0.91488	0.91562	0.91801	0.92058	0.92341
989	0.90796	0.91275	0.91495	0.91569	0.91807	0.92064	0.92347
990	0.90803	0.91282	0.91503	0.91576	0.91814	0.92071	0.92353
991	0.90811	0.91289	0.91509	0.91583	0.91821	0.92077	0.92359
992	0.90818	0.91296	0.91517	0.91590	0.91828	0.92084	0.92366
993	0.90827	0.91303	0.91523	0.91597	0.91834	0.92090	0.92372
994	0.90833	0.91310	0.91530	0.91604	0.91841	0.92097	0.92378
995	0.90841	0.91317	0.91537	0.91610	0.91848	0.92103	0.92384
996	0.90849	0.91324	0.91544	0.91617	0.91854	0.92109	0.92390
997	0.90857	0.91331	0.91551	0.91624	0.91861	0.92116	0.92396
998	0.90864	0.91339	0.91558	0.91631	0.91867	0.92122	0.92402
999	0.90872	0.91346	0.91565	0.91638	0.91874	0.92128	0.92408
1000	0.90879	0.91353	0.91571	0.91645	0.91880	0.92135	0.92415

Table 4 – *GM8(5H3L or 3H5L)*. Critical values of upper significance levels for the Grubbs multiple-8 outlier test, applicable in situations where the five highest and three lowest or three highest and five lowest observations are simultaneously identified as outliers, denoted as *GM8(5H3L)* and *GM8(3H5L)*, respectively.

N	0.1%	0.5%	1%	1.25%	2.5%	5%	10%
10	0.00000	0.00000	0.00000	0.00000	0.00000	0.00001	0.00006
11	0.00001	0.00005	0.00010	0.00013	0.00027	0.00057	0.00125
12	0.00016	0.00049	0.00080	0.00095	0.00158	0.00269	0.00468
13	0.00074	0.00177	0.00260	0.00295	0.00439	0.00665	0.01033
14	0.00202	0.00413	0.00567	0.00629	0.00876	0.01237	0.01786
15	0.00416	0.00764	0.01001	0.01095	0.01454	0.01956	0.02685
16	0.00720	0.01225	0.01553	0.01680	0.02152	0.02796	0.03695
17	0.01109	0.01780	0.02201	0.02361	0.02950	0.03727	0.04787
18	0.01585	0.02426	0.02938	0.03129	0.03829	0.04730	0.05936
19	0.02125	0.03142	0.03742	0.03962	0.04762	0.05781	0.07119
20	0.02733	0.03908	0.04593	0.04846	0.05747	0.06870	0.08328
21	0.03403	0.04722	0.05489	0.05767	0.06757	0.07982	0.09543
22	0.04100	0.05587	0.06422	0.06726	0.07794	0.09107	0.10763
23	0.04852	0.06472	0.07376	0.07702	0.08842	0.10234	0.11975
24	0.05616	0.07381	0.08349	0.08690	0.09901	0.11362	0.13177
25	0.06410	0.08300	0.09330	0.09696	0.10962	0.12486	0.14363
26	0.07228	0.09227	0.10310	0.10692	0.12018	0.13597	0.15535
27	0.08061	0.10169	0.11291	0.11691	0.13066	0.14697	0.16683
28	0.08907	0.11107	0.12272	0.12682	0.14101	0.15777	0.17809
29	0.09742	0.12031	0.13245	0.13668	0.15128	0.16840	0.18914
30	0.10599	0.12965	0.14204	0.14639	0.16141	0.17889	0.19994
31	0.11448	0.13889	0.15159	0.15608	0.17128	0.18916	0.21047
32	0.12300	0.14810	0.16103	0.16560	0.18117	0.19925	0.22082
33	0.13137	0.15710	0.17043	0.17506	0.19084	0.20914	0.23090
34	0.13987	0.16607	0.17957	0.18432	0.20030	0.21879	0.24076
35	0.14819	0.17486	0.18867	0.19345	0.20960	0.22824	0.25036

N	0.1%	0.5%	1%	1.25%	2.5%	5%	10%
36	0.15644	0.18366	0.19756	0.20238	0.21875	0.23755	0.25973
37	0.16441	0.19220	0.20632	0.21121	0.22772	0.24659	0.26889
38	0.17240	0.20059	0.21489	0.21988	0.23645	0.25543	0.27778
39	0.18046	0.20882	0.22332	0.22830	0.24498	0.26405	0.28650
40	0.18828	0.21699	0.23162	0.23660	0.25341	0.27256	0.29501
41	0.19602	0.22511	0.23972	0.24473	0.26165	0.28088	0.30330
42	0.20366	0.23293	0.24766	0.25268	0.26973	0.28897	0.31142
43	0.21119	0.24072	0.25549	0.26054	0.27762	0.29688	0.31935
44	0.21879	0.24835	0.26315	0.26827	0.28534	0.30462	0.32703
45	0.22597	0.25581	0.27069	0.27579	0.29291	0.31218	0.33455
46	0.23316	0.26308	0.27802	0.28318	0.30031	0.31959	0.34188
47	0.24019	0.27039	0.28529	0.29039	0.30757	0.32681	0.34909
48	0.24714	0.27743	0.29243	0.29752	0.31463	0.33388	0.35611
49	0.25416	0.28441	0.29939	0.30453	0.32157	0.34079	0.36294
50	0.26098	0.29130	0.30626	0.31138	0.32834	0.34755	0.36966
51	0.26768	0.29788	0.31296	0.31805	0.33505	0.35416	0.37620
52	0.27433	0.30445	0.31952	0.32463	0.34161	0.36066	0.38257
53	0.28092	0.31092	0.32595	0.33106	0.34796	0.36698	0.38884
54	0.28701	0.31724	0.33227	0.33733	0.35423	0.37320	0.39497
55	0.29305	0.32348	0.33846	0.34353	0.36042	0.37932	0.40094
56	0.29900	0.32960	0.34452	0.34966	0.36645	0.38529	0.40682
57	0.30496	0.33557	0.35054	0.35560	0.37232	0.39111	0.41257
58	0.31091	0.34157	0.35635	0.36139	0.37815	0.39681	0.41817
59	0.31680	0.34735	0.36211	0.36709	0.38382	0.40240	0.42369
60	0.32252	0.35290	0.36772	0.37276	0.38939	0.40789	0.42906
61	0.32816	0.35854	0.37324	0.37827	0.39484	0.41326	0.43434
62	0.33355	0.36405	0.37863	0.38366	0.40021	0.41856	0.43950
63	0.33902	0.36938	0.38398	0.38897	0.40546	0.42372	0.44456
64	0.34438	0.37461	0.38917	0.39415	0.41060	0.42881	0.44949
65	0.34965	0.37975	0.39431	0.39926	0.41565	0.43375	0.45437
66	0.35482	0.38492	0.39933	0.40426	0.42060	0.43863	0.45911
67	0.35987	0.38991	0.40430	0.40922	0.42545	0.44342	0.46379
68	0.36491	0.39484	0.40917	0.41404	0.43026	0.44808	0.46837
69	0.36984	0.39962	0.41395	0.41879	0.43492	0.45271	0.47287
70	0.37465	0.40444	0.41870	0.42354	0.43954	0.45724	0.47728
71	0.37943	0.40914	0.42337	0.42816	0.44409	0.46167	0.48162
72	0.38423	0.41376	0.42788	0.43268	0.44854	0.46602	0.48587
73	0.38879	0.41821	0.43235	0.43712	0.45292	0.47032	0.49005
74	0.39334	0.42268	0.43674	0.44149	0.45722	0.47453	0.49414
75	0.39781	0.42704	0.44108	0.44582	0.46143	0.47870	0.49817
76	0.40207	0.43137	0.44531	0.45004	0.46559	0.48275	0.50213
77	0.40664	0.43552	0.44946	0.45418	0.46968	0.48672	0.50601
78	0.41125	0.43970	0.45359	0.45828	0.47368	0.49062	0.50982
79	0.41523	0.44381	0.45768	0.46231	0.47767	0.49450	0.51359
80	0.41935	0.44790	0.46161	0.46630	0.48153	0.49830	0.51729
81	0.42333	0.45186	0.46552	0.47015	0.48533	0.50203	0.52091
82	0.42736	0.45578	0.46939	0.47398	0.48908	0.50569	0.52447
83	0.43142	0.45961	0.47313	0.47774	0.49276	0.50933	0.52798
84	0.43518	0.46334	0.47691	0.48142	0.49640	0.51287	0.53143
85	0.43897	0.46706	0.48059	0.48513	0.49999	0.51639	0.53481
86	0.44282	0.47076	0.48420	0.48872	0.50353	0.51981	0.53815
87	0.44649	0.47438	0.48779	0.49225	0.50702	0.52318	0.54143
88	0.45014	0.47798	0.49130	0.49576	0.51044	0.52652	0.54467
89	0.45381	0.48152	0.49477	0.49922	0.51378	0.52979	0.54785
90	0.45737	0.48502	0.49819	0.50261	0.51712	0.53302	0.55097
91	0.46092	0.48845	0.50157	0.50598	0.52037	0.53621	0.55404
92	0.46452	0.49186	0.50490	0.50927	0.52358	0.53936	0.55709
93	0.46793	0.49515	0.50810	0.51249	0.52674	0.54243	0.56007
94	0.47124	0.49839	0.51131	0.51566	0.52986	0.54546	0.56302
95	0.47451	0.50156	0.51447	0.51882	0.53292	0.54846	0.56592
96	0.47783	0.50477	0.51761	0.52193	0.53599	0.55142	0.56878
97	0.48132	0.50795	0.52070	0.52498	0.53898	0.55433	0.57159
98	0.48442	0.51105	0.52374	0.52800	0.54192	0.55720	0.57437
99	0.48764	0.51415	0.52673	0.53096	0.54482	0.56001	0.57710
100	0.49069	0.51713	0.52970	0.53387	0.54769	0.56281	0.57979
101	0.49379	0.52011	0.53258	0.53678	0.55053	0.56555	0.58245
102	0.49677	0.52309	0.53546	0.53963	0.55330	0.56828	0.58508
103	0.49977	0.52597	0.53832	0.54246	0.55604	0.57096	0.58768
104	0.50276	0.52882	0.54112	0.54524	0.55876	0.57356	0.59023
105	0.50563	0.53169	0.54390	0.54802	0.56147	0.57617	0.59272
106	0.50850	0.53444	0.54664	0.55071	0.56410	0.57873	0.59519
107	0.51136	0.53710	0.54931	0.55338	0.56672	0.58129	0.59763
108	0.51419	0.53984	0.55200	0.55604	0.56930	0.58379	0.60005
109	0.51699	0.54248	0.55464	0.55866	0.57185	0.58626	0.60242

N	0.1%	0.5%	1%	1.25%	2.5%	5%	10%
110	0.51979	0.54517	0.55717	0.56121	0.57432	0.58868	0.60477
111	0.52260	0.54780	0.55972	0.56372	0.57679	0.59109	0.60709
112	0.52522	0.55042	0.56223	0.56619	0.57923	0.59345	0.60936
113	0.52791	0.55295	0.56472	0.56866	0.58164	0.59580	0.61162
114	0.53045	0.55542	0.56716	0.57110	0.58400	0.59810	0.61385
115	0.53299	0.55793	0.56958	0.57352	0.58633	0.60037	0.61603
116	0.53557	0.56034	0.57198	0.57593	0.58865	0.60261	0.61820
117	0.53810	0.56276	0.57437	0.57829	0.59098	0.60483	0.62034
118	0.54066	0.56519	0.57672	0.58061	0.59325	0.60702	0.62245
119	0.54315	0.56753	0.57903	0.58288	0.59546	0.60919	0.62453
120	0.54562	0.56989	0.58133	0.58515	0.59769	0.61133	0.62660
121	0.54799	0.57218	0.58358	0.58738	0.59985	0.61343	0.62864
122	0.55047	0.57446	0.58580	0.58960	0.60202	0.61552	0.63066
123	0.55288	0.57673	0.58799	0.59176	0.60415	0.61758	0.63265
124	0.55517	0.57896	0.59017	0.59395	0.60626	0.61962	0.63462
125	0.55751	0.58115	0.59230	0.59607	0.60834	0.62163	0.63655
126	0.55967	0.58333	0.59442	0.59819	0.61038	0.62362	0.63848
127	0.56196	0.58546	0.59651	0.60027	0.61242	0.62559	0.64038
128	0.56423	0.58763	0.59861	0.60233	0.61442	0.62753	0.64226
129	0.56641	0.58970	0.60069	0.60438	0.61641	0.62946	0.64411
130	0.56857	0.59175	0.60271	0.60641	0.61835	0.63135	0.64593
131	0.57080	0.59383	0.60473	0.60838	0.62029	0.63324	0.64773
132	0.57290	0.59586	0.60671	0.61037	0.62219	0.63511	0.64952
133	0.57496	0.59786	0.60870	0.61231	0.62409	0.63693	0.65129
134	0.57708	0.59987	0.61062	0.61424	0.62597	0.63875	0.65303
135	0.57913	0.60188	0.61256	0.61615	0.62784	0.64054	0.65476
136	0.58122	0.60378	0.61444	0.61804	0.62965	0.64233	0.65647
137	0.58330	0.60571	0.61633	0.61992	0.63146	0.64407	0.65816
138	0.58531	0.60760	0.61818	0.62176	0.63324	0.64581	0.65983
139	0.58722	0.60945	0.62000	0.62357	0.63501	0.64753	0.66148
140	0.58921	0.61131	0.62185	0.62536	0.63678	0.64922	0.66311
141	0.59122	0.61318	0.62364	0.62715	0.63853	0.65090	0.66473
142	0.59305	0.61499	0.62542	0.62895	0.64025	0.65257	0.66633
143	0.59505	0.61681	0.62717	0.63069	0.64195	0.65423	0.66792
144	0.59692	0.61865	0.62894	0.63240	0.64366	0.65585	0.66948
145	0.59873	0.62043	0.63066	0.63412	0.64534	0.65748	0.67103
146	0.60061	0.62218	0.63236	0.63581	0.64697	0.65907	0.67257
147	0.60237	0.62389	0.63408	0.63749	0.64862	0.66063	0.67408
148	0.60418	0.62560	0.63574	0.63913	0.65021	0.66219	0.67557
149	0.60602	0.62732	0.63742	0.64080	0.65179	0.66373	0.67705
150	0.60774	0.62899	0.63905	0.64244	0.65339	0.66528	0.67852
151	0.60956	0.63068	0.64068	0.64405	0.65495	0.66679	0.67997
152	0.61127	0.63232	0.64230	0.64564	0.65650	0.66829	0.68142
153	0.61304	0.63398	0.64388	0.64722	0.65804	0.66978	0.68284
154	0.61469	0.63558	0.64546	0.64880	0.65956	0.67125	0.68425
155	0.61639	0.63718	0.64704	0.65037	0.66108	0.67270	0.68563
156	0.61806	0.63878	0.64857	0.65189	0.66257	0.67414	0.68702
157	0.61975	0.64031	0.65012	0.65340	0.66403	0.67557	0.68840
158	0.62135	0.64190	0.65164	0.65490	0.66548	0.67699	0.68975
159	0.62294	0.64341	0.65314	0.65637	0.66693	0.67838	0.69109
160	0.62468	0.64500	0.65462	0.65785	0.66838	0.67977	0.69243
161	0.62633	0.64654	0.65610	0.65930	0.66979	0.68114	0.69375
162	0.62786	0.64801	0.65752	0.66074	0.67120	0.68249	0.69506
163	0.62942	0.64948	0.65899	0.66218	0.67257	0.68384	0.69634
164	0.63095	0.65099	0.66044	0.66360	0.67395	0.68516	0.69762
165	0.63249	0.65244	0.66186	0.66503	0.67532	0.68649	0.69889
166	0.63399	0.65388	0.66325	0.66639	0.67667	0.68780	0.70015
167	0.63547	0.65533	0.66464	0.66778	0.67801	0.68910	0.70139
168	0.63699	0.65676	0.66604	0.66916	0.67935	0.69039	0.70262
169	0.63841	0.65818	0.66742	0.67052	0.68066	0.69165	0.70385
170	0.63986	0.65953	0.66878	0.67187	0.68196	0.69291	0.70506
171	0.64133	0.66091	0.67012	0.67319	0.68325	0.69415	0.70626
172	0.64272	0.66227	0.67145	0.67452	0.68453	0.69540	0.70744
173	0.64413	0.66365	0.67277	0.67584	0.68580	0.69663	0.70861
174	0.64563	0.66504	0.67407	0.67713	0.68707	0.69785	0.70978
175	0.64704	0.66635	0.67538	0.67840	0.68831	0.69906	0.71094
176	0.64838	0.66765	0.67665	0.67967	0.68955	0.70024	0.71209
177	0.64975	0.66898	0.67794	0.68093	0.69078	0.70142	0.71323
178	0.65107	0.67027	0.67919	0.68218	0.69199	0.70260	0.71436
179	0.65241	0.67157	0.68043	0.68339	0.69319	0.70377	0.71548
180	0.65380	0.67284	0.68169	0.68464	0.69439	0.70493	0.71659
181	0.65513	0.67410	0.68291	0.68585	0.69557	0.70606	0.71767
182	0.65645	0.67536	0.68413	0.68707	0.69675	0.70719	0.71877
183	0.65777	0.67661	0.68535	0.68827	0.69791	0.70831	0.71984

N	0.1%	0.5%	1%	1.25%	2.5%	5%	10%
184	0.65905	0.67786	0.68655	0.68949	0.69907	0.70942	0.72090
185	0.66033	0.67905	0.68774	0.69069	0.70020	0.71053	0.72197
186	0.66162	0.68026	0.68892	0.69183	0.70132	0.71161	0.72302
187	0.66284	0.68146	0.69011	0.69299	0.70246	0.71271	0.72407
188	0.66413	0.68266	0.69129	0.69414	0.70356	0.71378	0.72511
189	0.66540	0.68383	0.69243	0.69528	0.70467	0.71485	0.72613
190	0.66663	0.68498	0.69357	0.69642	0.70577	0.71592	0.72714
191	0.66786	0.68613	0.69469	0.69754	0.70686	0.71696	0.72815
192	0.66903	0.68729	0.69583	0.69866	0.70796	0.71800	0.72915
193	0.67024	0.68844	0.69694	0.69976	0.70904	0.71904	0.73013
194	0.67146	0.68958	0.69802	0.70084	0.71008	0.72006	0.73112
195	0.67269	0.69069	0.69914	0.70194	0.71113	0.72108	0.73210
196	0.67387	0.69180	0.70022	0.70301	0.71217	0.72207	0.73306
197	0.67504	0.69288	0.70131	0.70409	0.71322	0.72309	0.73402
198	0.67612	0.69400	0.70236	0.70516	0.71424	0.72407	0.73498
199	0.67735	0.69505	0.70343	0.70621	0.71526	0.72506	0.73592
200	0.67850	0.69616	0.70448	0.70725	0.71628	0.72604	0.73686
201	0.67960	0.69726	0.70552	0.70827	0.71728	0.72702	0.73779
202	0.68074	0.69832	0.70653	0.70928	0.71828	0.72797	0.73870
203	0.68188	0.69939	0.70756	0.71031	0.71927	0.72892	0.73962
204	0.68297	0.70045	0.70860	0.71133	0.72025	0.72987	0.74053
205	0.68407	0.70148	0.70961	0.71233	0.72122	0.73082	0.74143
206	0.68514	0.70253	0.71061	0.71333	0.72217	0.73175	0.74232
207	0.68620	0.70356	0.71161	0.71433	0.72314	0.73267	0.74321
208	0.68728	0.70458	0.71262	0.71531	0.72408	0.73359	0.74409
209	0.68831	0.70559	0.71361	0.71629	0.72503	0.73450	0.74497
210	0.68937	0.70657	0.71458	0.71725	0.72597	0.73539	0.74583
211	0.69041	0.70756	0.71556	0.71821	0.72689	0.73628	0.74669
212	0.69150	0.70855	0.71652	0.71917	0.72782	0.73717	0.74755
213	0.69257	0.70951	0.71746	0.72011	0.72875	0.73805	0.74839
214	0.69363	0.71052	0.71842	0.72106	0.72966	0.73892	0.74923
215	0.69462	0.71149	0.71936	0.72200	0.73056	0.73980	0.75006
216	0.69565	0.71246	0.72028	0.72293	0.73145	0.74068	0.75090
217	0.69662	0.71338	0.72119	0.72386	0.73233	0.74153	0.75172
218	0.69764	0.71433	0.72213	0.72476	0.73322	0.74238	0.75254
219	0.69865	0.71527	0.72306	0.72567	0.73409	0.74323	0.75334
220	0.69958	0.71618	0.72396	0.72655	0.73497	0.74406	0.75415
221	0.70057	0.71711	0.72486	0.72745	0.73583	0.74490	0.75495
222	0.70157	0.71804	0.72575	0.72834	0.73669	0.74572	0.75574
223	0.70252	0.71897	0.72665	0.72921	0.73754	0.74654	0.75652
224	0.70350	0.71987	0.72752	0.73007	0.73838	0.74735	0.75731
225	0.70446	0.72077	0.72840	0.73095	0.73921	0.74817	0.75808
226	0.70542	0.72166	0.72927	0.73180	0.74004	0.74898	0.75885
227	0.70637	0.72253	0.73011	0.73266	0.74088	0.74977	0.75962
228	0.70730	0.72341	0.73096	0.73350	0.74168	0.75056	0.76037
229	0.70823	0.72429	0.73183	0.73434	0.74251	0.75134	0.76113
230	0.70914	0.72515	0.73267	0.73518	0.74331	0.75213	0.76188
231	0.71001	0.72601	0.73351	0.73602	0.74412	0.75290	0.76263
232	0.71087	0.72687	0.73433	0.73682	0.74491	0.75367	0.76336
233	0.71174	0.72770	0.73516	0.73766	0.74571	0.75444	0.76410
234	0.71263	0.72856	0.73599	0.73847	0.74650	0.75519	0.76483
235	0.71347	0.72939	0.73680	0.73926	0.74728	0.75594	0.76555
236	0.71438	0.73026	0.73761	0.74007	0.74804	0.75669	0.76626
237	0.71526	0.73109	0.73842	0.74085	0.74881	0.75743	0.76697
238	0.71619	0.73189	0.73922	0.74163	0.74958	0.75817	0.76768
239	0.71703	0.73269	0.74000	0.74243	0.75034	0.75889	0.76839
240	0.71790	0.73351	0.74079	0.74321	0.75109	0.75963	0.76909
241	0.71876	0.73433	0.74158	0.74399	0.75184	0.76035	0.76979
242	0.71951	0.73514	0.74234	0.74478	0.75259	0.76107	0.77047
243	0.72040	0.73592	0.74311	0.74554	0.75333	0.76179	0.77115
244	0.72126	0.73671	0.74390	0.74630	0.75408	0.76250	0.77184
245	0.72209	0.73750	0.74467	0.74704	0.75480	0.76320	0.77251
246	0.72295	0.73827	0.74542	0.74780	0.75553	0.76390	0.77318
247	0.72379	0.73904	0.74617	0.74854	0.75624	0.76459	0.77385
248	0.72459	0.73982	0.74690	0.74928	0.75697	0.76529	0.77452
249	0.72539	0.74058	0.74764	0.75000	0.75769	0.76597	0.77517
250	0.72620	0.74135	0.74837	0.75074	0.75839	0.76665	0.77583
251	0.72699	0.74211	0.74911	0.75146	0.75910	0.76733	0.77648
252	0.72778	0.74284	0.74984	0.75217	0.75981	0.76801	0.77712
253	0.72863	0.74359	0.75056	0.75289	0.76050	0.76867	0.77776
254	0.72937	0.74434	0.75127	0.75360	0.76118	0.76934	0.77840
255	0.73011	0.74506	0.75199	0.75431	0.76186	0.77000	0.77903
256	0.73095	0.74580	0.75270	0.75502	0.76253	0.77066	0.77966
257	0.73171	0.74651	0.75341	0.75571	0.76321	0.77131	0.78028

N	0.1%	0.5%	1%	1.25%	2.5%	5%	10%
258	0.73245	0.74721	0.75411	0.75641	0.76387	0.77196	0.78090
259	0.73320	0.74794	0.75481	0.75709	0.76454	0.77261	0.78152
260	0.73396	0.74865	0.75550	0.75778	0.76521	0.77324	0.78214
261	0.73474	0.74934	0.75617	0.75846	0.76587	0.77388	0.78275
262	0.73550	0.75004	0.75686	0.75913	0.76652	0.77451	0.78335
263	0.73624	0.75076	0.75754	0.75980	0.76718	0.77514	0.78395
264	0.73699	0.75144	0.75822	0.76048	0.76784	0.77576	0.78455
265	0.73775	0.75213	0.75889	0.76114	0.76847	0.77638	0.78514
266	0.73846	0.75283	0.75957	0.76180	0.76912	0.77700	0.78573
267	0.73919	0.75350	0.76023	0.76247	0.76975	0.77762	0.78632
268	0.73991	0.75418	0.76089	0.76313	0.77038	0.77822	0.78691
269	0.74061	0.75486	0.76156	0.76377	0.77101	0.77882	0.78749
270	0.74130	0.75551	0.76221	0.76443	0.77163	0.77943	0.78806
271	0.74199	0.75619	0.76286	0.76506	0.77225	0.78002	0.78864
272	0.74269	0.75685	0.76351	0.76571	0.77287	0.78062	0.78921
273	0.74336	0.75749	0.76415	0.76634	0.77348	0.78121	0.78978
274	0.74403	0.75816	0.76479	0.76696	0.77409	0.78179	0.79034
275	0.74475	0.75882	0.76542	0.76759	0.77470	0.78237	0.79089
276	0.74544	0.75948	0.76605	0.76822	0.77531	0.78295	0.79145
277	0.74614	0.76013	0.76667	0.76883	0.77591	0.78353	0.79201
278	0.74689	0.76076	0.76731	0.76945	0.77651	0.78411	0.79256
279	0.74754	0.76139	0.76793	0.77007	0.77711	0.78468	0.79311
280	0.74818	0.76205	0.76853	0.77067	0.77770	0.78525	0.79365
281	0.74884	0.76268	0.76913	0.77127	0.77828	0.78582	0.79420
282	0.74954	0.76331	0.76975	0.77187	0.77886	0.78638	0.79473
283	0.75015	0.76394	0.77036	0.77248	0.77944	0.78693	0.79527
284	0.75084	0.76455	0.77095	0.77306	0.78001	0.78748	0.79580
285	0.75145	0.76517	0.77156	0.77365	0.78058	0.78804	0.79633
286	0.75211	0.76578	0.77214	0.77425	0.78115	0.78858	0.79685
287	0.75277	0.76640	0.77273	0.77483	0.78170	0.78913	0.79737
288	0.75341	0.76699	0.77331	0.77541	0.78227	0.78967	0.79790
289	0.75401	0.76760	0.77389	0.77600	0.78283	0.79021	0.79841
290	0.75464	0.76820	0.77447	0.77656	0.78338	0.79075	0.79892
291	0.75530	0.76880	0.77505	0.77714	0.78394	0.79129	0.79943
292	0.75588	0.76941	0.77563	0.77771	0.78449	0.79182	0.79994
293	0.75653	0.77000	0.77618	0.77827	0.78504	0.79234	0.80044
294	0.75714	0.77058	0.77676	0.77882	0.78559	0.79287	0.80094
295	0.75780	0.77114	0.77732	0.77939	0.78613	0.79340	0.80144
296	0.75840	0.77171	0.77787	0.77994	0.78667	0.79391	0.80194
297	0.75898	0.77230	0.77844	0.78049	0.78720	0.79442	0.80243
298	0.75961	0.77286	0.77899	0.78105	0.78773	0.79494	0.80291
299	0.76021	0.77341	0.77954	0.78160	0.78826	0.79544	0.80340
300	0.76084	0.77397	0.78010	0.78214	0.78879	0.79595	0.80388
301	0.76145	0.77454	0.78064	0.78268	0.78930	0.79645	0.80436
302	0.76204	0.77509	0.78119	0.78321	0.78982	0.79695	0.80484
303	0.76268	0.77565	0.78172	0.78374	0.79034	0.79745	0.80532
304	0.76324	0.77617	0.78225	0.78427	0.79085	0.79794	0.80579
305	0.76381	0.77674	0.78278	0.78481	0.79137	0.79844	0.80627
306	0.76438	0.77728	0.78332	0.78533	0.79188	0.79893	0.80674
307	0.76491	0.77782	0.78386	0.78586	0.79239	0.79941	0.80720
308	0.76546	0.77836	0.78438	0.78637	0.79289	0.79990	0.80767
309	0.76607	0.77890	0.78491	0.78689	0.79339	0.80038	0.80813
310	0.76661	0.77941	0.78542	0.78740	0.79389	0.80086	0.80859
311	0.76717	0.77993	0.78594	0.78792	0.79439	0.80133	0.80905
312	0.76774	0.78047	0.78645	0.78843	0.79487	0.80182	0.80950
313	0.76827	0.78100	0.78696	0.78893	0.79536	0.80228	0.80995
314	0.76884	0.78152	0.78746	0.78945	0.79586	0.80276	0.81041
315	0.76938	0.78207	0.78798	0.78994	0.79634	0.80322	0.81086
316	0.76996	0.78257	0.78848	0.79044	0.79682	0.80369	0.81130
317	0.77048	0.78308	0.78898	0.79094	0.79730	0.80415	0.81174
318	0.77100	0.78361	0.78948	0.79143	0.79777	0.80461	0.81219
319	0.77157	0.78411	0.78998	0.79191	0.79826	0.80507	0.81262
320	0.77212	0.78461	0.79047	0.79240	0.79873	0.80552	0.81306
321	0.77265	0.78512	0.79096	0.79290	0.79920	0.80598	0.81349
322	0.77316	0.78562	0.79145	0.79338	0.79966	0.80643	0.81393
323	0.77368	0.78613	0.79193	0.79386	0.80013	0.80688	0.81435
324	0.77422	0.78662	0.79242	0.79434	0.80059	0.80732	0.81478
325	0.77474	0.78712	0.79289	0.79482	0.80104	0.80776	0.81520
326	0.77526	0.78762	0.79337	0.79528	0.80150	0.80820	0.81562
327	0.77579	0.78811	0.79385	0.79576	0.80196	0.80864	0.81604
328	0.77631	0.78858	0.79432	0.79622	0.80242	0.80907	0.81646
329	0.77685	0.78909	0.79477	0.79669	0.80287	0.80951	0.81688
330	0.77734	0.78955	0.79524	0.79715	0.80331	0.80995	0.81729
331	0.77785	0.79003	0.79571	0.79762	0.80376	0.81038	0.81771

N	0.1%	0.5%	1%	1.25%	2.5%	5%	10%
332	0.77835	0.79051	0.79618	0.79807	0.80420	0.81080	0.81811
333	0.77882	0.79098	0.79664	0.79854	0.80465	0.81123	0.81852
334	0.77931	0.79146	0.79710	0.79898	0.80509	0.81166	0.81893
335	0.77983	0.79193	0.79755	0.79943	0.80553	0.81208	0.81934
336	0.78032	0.79239	0.79802	0.79989	0.80597	0.81250	0.81974
337	0.78082	0.79285	0.79846	0.80034	0.80640	0.81292	0.82014
338	0.78130	0.79333	0.79892	0.80078	0.80684	0.81333	0.82054
339	0.78181	0.79379	0.79937	0.80123	0.80726	0.81374	0.82094
340	0.78226	0.79424	0.79982	0.80167	0.80769	0.81416	0.82133
341	0.78277	0.79470	0.80025	0.80211	0.80811	0.81457	0.82172
342	0.78323	0.79515	0.80070	0.80254	0.80854	0.81497	0.82211
343	0.78369	0.79558	0.80113	0.80298	0.80895	0.81538	0.82250
344	0.78418	0.79604	0.80157	0.80341	0.80938	0.81579	0.82288
345	0.78466	0.79649	0.80201	0.80385	0.80979	0.81619	0.82327
346	0.78512	0.79694	0.80245	0.80428	0.81021	0.81659	0.82365
347	0.78558	0.79738	0.80288	0.80471	0.81062	0.81699	0.82403
348	0.78609	0.79783	0.80331	0.80513	0.81103	0.81739	0.82441
349	0.78655	0.79827	0.80373	0.80555	0.81144	0.81778	0.82479
350	0.78699	0.79871	0.80416	0.80597	0.81185	0.81817	0.82516
351	0.78743	0.79914	0.80459	0.80639	0.81225	0.81856	0.82554
352	0.78788	0.79957	0.80501	0.80681	0.81266	0.81895	0.82591
353	0.78831	0.80000	0.80542	0.80723	0.81307	0.81933	0.82629
354	0.78877	0.80044	0.80584	0.80764	0.81346	0.81972	0.82666
355	0.78923	0.80088	0.80624	0.80805	0.81386	0.82010	0.82702
356	0.78970	0.80131	0.80667	0.80847	0.81425	0.82048	0.82739
357	0.79017	0.80172	0.80708	0.80887	0.81465	0.82086	0.82776
358	0.79059	0.80214	0.80750	0.80927	0.81504	0.82124	0.82812
359	0.79106	0.80257	0.80789	0.80968	0.81542	0.82161	0.82847
360	0.79147	0.80299	0.80829	0.81008	0.81581	0.82198	0.82883
361	0.79192	0.80340	0.80870	0.81048	0.81621	0.82236	0.82918
362	0.79238	0.80382	0.80911	0.81088	0.81659	0.82273	0.82954
363	0.79282	0.80423	0.80951	0.81128	0.81697	0.82310	0.82989
364	0.79322	0.80465	0.80991	0.81167	0.81735	0.82347	0.83025
365	0.79366	0.80506	0.81031	0.81207	0.81773	0.82383	0.83060
366	0.79409	0.80547	0.81070	0.81246	0.81810	0.82420	0.83095
367	0.79450	0.80587	0.81110	0.81285	0.81847	0.82456	0.83129
368	0.79493	0.80626	0.81149	0.81322	0.81885	0.82492	0.83164
369	0.79534	0.80667	0.81188	0.81362	0.81923	0.82528	0.83198
370	0.79578	0.80708	0.81226	0.81400	0.81959	0.82564	0.83233
371	0.79619	0.80747	0.81265	0.81438	0.81997	0.82599	0.83267
372	0.79659	0.80786	0.81304	0.81477	0.82034	0.82635	0.83301
373	0.79699	0.80824	0.81342	0.81515	0.82070	0.82670	0.83335
374	0.79743	0.80864	0.81380	0.81552	0.82106	0.82705	0.83369
375	0.79785	0.80903	0.81418	0.81590	0.82142	0.82741	0.83402
376	0.79825	0.80941	0.81456	0.81628	0.82179	0.82775	0.83435
377	0.79866	0.80979	0.81494	0.81665	0.82214	0.82810	0.83469
378	0.79907	0.81017	0.81531	0.81701	0.82250	0.82844	0.83502
379	0.79950	0.81057	0.81568	0.81738	0.82286	0.82878	0.83534
380	0.79988	0.81094	0.81605	0.81775	0.82321	0.82913	0.83567
381	0.80030	0.81133	0.81643	0.81812	0.82356	0.82948	0.83600
382	0.80070	0.81171	0.81680	0.81848	0.82392	0.82981	0.83632
383	0.80113	0.81208	0.81715	0.81884	0.82427	0.83015	0.83664
384	0.80151	0.81244	0.81752	0.81921	0.82462	0.83049	0.83697
385	0.80191	0.81282	0.81788	0.81956	0.82497	0.83082	0.83729
386	0.80231	0.81319	0.81825	0.81992	0.82531	0.83115	0.83761
387	0.80272	0.81355	0.81860	0.82028	0.82566	0.83149	0.83792
388	0.80312	0.81392	0.81895	0.82063	0.82600	0.83181	0.83824
389	0.80349	0.81429	0.81931	0.82097	0.82634	0.83214	0.83856
390	0.80387	0.81467	0.81967	0.82132	0.82668	0.83247	0.83887
391	0.80429	0.81505	0.82000	0.82167	0.82702	0.83279	0.83918
392	0.80465	0.81541	0.82036	0.82202	0.82735	0.83312	0.83950
393	0.80502	0.81577	0.82071	0.82237	0.82768	0.83344	0.83980
394	0.80541	0.81612	0.82106	0.82272	0.82802	0.83376	0.84011
395	0.80580	0.81648	0.82142	0.82306	0.82835	0.83408	0.84042
396	0.80620	0.81682	0.82175	0.82341	0.82869	0.83441	0.84072
397	0.80655	0.81718	0.82210	0.82374	0.82901	0.83473	0.84103
398	0.80692	0.81753	0.82245	0.82409	0.82934	0.83504	0.84133
399	0.80730	0.81788	0.82279	0.82442	0.82966	0.83536	0.84163
400	0.80765	0.81822	0.82313	0.82475	0.82999	0.83567	0.84194
401	0.80803	0.81857	0.82346	0.82508	0.83031	0.83598	0.84224
402	0.80843	0.81892	0.82381	0.82542	0.83063	0.83629	0.84253
403	0.80879	0.81926	0.82415	0.82576	0.83096	0.83660	0.84283
404	0.80916	0.81962	0.82448	0.82609	0.83128	0.83691	0.84313
405	0.80951	0.81996	0.82482	0.82641	0.83160	0.83722	0.84342

N	0.1%	0.5%	1%	1.25%	2.5%	5%	10%
406	0.80989	0.82029	0.82515	0.82674	0.83192	0.83753	0.84371
407	0.81023	0.82065	0.82548	0.82707	0.83224	0.83784	0.84400
408	0.81059	0.82099	0.82581	0.82739	0.83256	0.83814	0.84429
409	0.81096	0.82133	0.82613	0.82772	0.83287	0.83844	0.84458
410	0.81132	0.82166	0.82645	0.82804	0.83318	0.83873	0.84487
411	0.81167	0.82199	0.82677	0.82836	0.83349	0.83903	0.84516
412	0.81201	0.82232	0.82711	0.82867	0.83381	0.83933	0.84545
413	0.81237	0.82265	0.82742	0.82899	0.83411	0.83962	0.84573
414	0.81273	0.82298	0.82774	0.82932	0.83442	0.83992	0.84602
415	0.81307	0.82331	0.82806	0.82963	0.83472	0.84021	0.84630
416	0.81342	0.82365	0.82838	0.82994	0.83503	0.84051	0.84658
417	0.81376	0.82398	0.82870	0.83025	0.83533	0.84080	0.84686
418	0.81411	0.82430	0.82902	0.83057	0.83563	0.84109	0.84714
419	0.81446	0.82463	0.82934	0.83088	0.83593	0.84138	0.84742
420	0.81482	0.82494	0.82965	0.83119	0.83624	0.84167	0.84769
421	0.81518	0.82525	0.82996	0.83149	0.83653	0.84196	0.84797
422	0.81552	0.82557	0.83026	0.83180	0.83683	0.84225	0.84825
423	0.81586	0.82589	0.83057	0.83211	0.83712	0.84253	0.84852
424	0.81617	0.82619	0.83088	0.83242	0.83742	0.84281	0.84879
425	0.81650	0.82651	0.83119	0.83272	0.83771	0.84310	0.84906
426	0.81682	0.82683	0.83149	0.83303	0.83800	0.84338	0.84933
427	0.81716	0.82713	0.83179	0.83333	0.83830	0.84366	0.84960
428	0.81753	0.82744	0.83209	0.83363	0.83859	0.84394	0.84987
429	0.81785	0.82776	0.83240	0.83393	0.83887	0.84422	0.85013
430	0.81819	0.82807	0.83269	0.83423	0.83916	0.84450	0.85040
431	0.81851	0.82838	0.83299	0.83452	0.83945	0.84477	0.85067
432	0.81887	0.82869	0.83329	0.83481	0.83973	0.84505	0.85093
433	0.81917	0.82900	0.83359	0.83510	0.84001	0.84533	0.85119
434	0.81952	0.82932	0.83389	0.83540	0.84030	0.84559	0.85145
435	0.81984	0.82961	0.83418	0.83569	0.84058	0.84587	0.85171
436	0.82015	0.82991	0.83448	0.83599	0.84087	0.84614	0.85197
437	0.82048	0.83021	0.83477	0.83627	0.84114	0.84641	0.85223
438	0.82080	0.83051	0.83506	0.83656	0.84142	0.84668	0.85249
439	0.82112	0.83082	0.83534	0.83684	0.84170	0.84695	0.85275
440	0.82144	0.83112	0.83564	0.83713	0.84199	0.84721	0.85300
441	0.82176	0.83141	0.83593	0.83741	0.84226	0.84748	0.85326
442	0.82206	0.83170	0.83621	0.83770	0.84253	0.84775	0.85351
443	0.82238	0.83199	0.83649	0.83797	0.84281	0.84801	0.85376
444	0.82268	0.83229	0.83678	0.83826	0.84308	0.84828	0.85402
445	0.82301	0.83259	0.83707	0.83854	0.84335	0.84854	0.85427
446	0.82330	0.83287	0.83734	0.83883	0.84363	0.84880	0.85452
447	0.82362	0.83317	0.83762	0.83911	0.84390	0.84906	0.85477
448	0.82394	0.83346	0.83790	0.83938	0.84417	0.84932	0.85502
449	0.82425	0.83375	0.83818	0.83966	0.84444	0.84957	0.85526
450	0.82455	0.83404	0.83846	0.83994	0.84470	0.84983	0.85551
451	0.82487	0.83433	0.83875	0.84022	0.84497	0.85008	0.85575
452	0.82515	0.83461	0.83902	0.84048	0.84523	0.85034	0.85600
453	0.82543	0.83489	0.83929	0.84075	0.84550	0.85059	0.85624
454	0.82575	0.83518	0.83957	0.84103	0.84577	0.85084	0.85648
455	0.82605	0.83545	0.83984	0.84130	0.84602	0.85109	0.85672
456	0.82636	0.83574	0.84011	0.84157	0.84628	0.85135	0.85697
457	0.82665	0.83602	0.84038	0.84183	0.84654	0.85160	0.85721
458	0.82695	0.83630	0.84065	0.84209	0.84680	0.85185	0.85744
459	0.82723	0.83657	0.84092	0.84236	0.84706	0.85210	0.85768
460	0.82753	0.83685	0.84119	0.84262	0.84731	0.85234	0.85792
461	0.82783	0.83713	0.84146	0.84289	0.84757	0.85259	0.85815
462	0.82812	0.83740	0.84173	0.84316	0.84783	0.85283	0.85839
463	0.82842	0.83768	0.84199	0.84342	0.84808	0.85308	0.85863
464	0.82872	0.83795	0.84226	0.84369	0.84833	0.85333	0.85886
465	0.82901	0.83821	0.84252	0.84395	0.84858	0.85357	0.85909
466	0.82929	0.83849	0.84278	0.84421	0.84884	0.85381	0.85933
467	0.82956	0.83876	0.84305	0.84447	0.84909	0.85405	0.85956
468	0.82985	0.83902	0.84330	0.84472	0.84934	0.85429	0.85979
469	0.83012	0.83928	0.84355	0.84498	0.84958	0.85453	0.86001
470	0.83040	0.83954	0.84381	0.84523	0.84983	0.85477	0.86024
471	0.83068	0.83981	0.84407	0.84549	0.85008	0.85501	0.86047
472	0.83097	0.84006	0.84431	0.84574	0.85032	0.85525	0.86070
473	0.83126	0.84034	0.84458	0.84600	0.85056	0.85549	0.86093
474	0.83152	0.84062	0.84484	0.84625	0.85081	0.85572	0.86115
475	0.83180	0.84088	0.84509	0.84650	0.85105	0.85596	0.86137
476	0.83209	0.84113	0.84535	0.84675	0.85129	0.85619	0.86160
477	0.83234	0.84138	0.84561	0.84700	0.85154	0.85643	0.86182
478	0.83264	0.84165	0.84586	0.84725	0.85178	0.85666	0.86204
479	0.83294	0.84190	0.84610	0.84749	0.85202	0.85689	0.86226

N	0.1%	0.5%	1%	1.25%	2.5%	5%	10%
480	0.83320	0.84216	0.84635	0.84774	0.85226	0.85712	0.86249
481	0.83349	0.84242	0.84659	0.84799	0.85249	0.85735	0.86271
482	0.83379	0.84268	0.84684	0.84824	0.85273	0.85758	0.86293
483	0.83404	0.84293	0.84709	0.84848	0.85297	0.85781	0.86314
484	0.83428	0.84318	0.84735	0.84872	0.85320	0.85804	0.86336
485	0.83455	0.84344	0.84758	0.84896	0.85344	0.85826	0.86358
486	0.83481	0.84369	0.84783	0.84920	0.85367	0.85848	0.86380
487	0.83508	0.84394	0.84807	0.84944	0.85391	0.85871	0.86401
488	0.83535	0.84418	0.84832	0.84968	0.85414	0.85893	0.86422
489	0.83563	0.84444	0.84856	0.84992	0.85437	0.85916	0.86444
490	0.83589	0.84468	0.84880	0.85016	0.85459	0.85938	0.86465
491	0.83614	0.84493	0.84904	0.85040	0.85482	0.85960	0.86486
492	0.83642	0.84517	0.84927	0.85063	0.85505	0.85982	0.86507
493	0.83667	0.84542	0.84951	0.85087	0.85527	0.86004	0.86528
494	0.83691	0.84566	0.84975	0.85111	0.85550	0.86026	0.86550
495	0.83719	0.84591	0.84998	0.85134	0.85573	0.86048	0.86570
496	0.83746	0.84615	0.85022	0.85157	0.85596	0.86069	0.86591
497	0.83771	0.84640	0.85045	0.85181	0.85619	0.86092	0.86612
498	0.83795	0.84664	0.85068	0.85204	0.85641	0.86113	0.86633
499	0.83823	0.84688	0.85092	0.85227	0.85663	0.86134	0.86654
500	0.83847	0.84712	0.85115	0.85250	0.85686	0.86156	0.86675
501	0.83873	0.84736	0.85138	0.85274	0.85708	0.86177	0.86695
502	0.83898	0.84758	0.85162	0.85296	0.85730	0.86199	0.86716
503	0.83921	0.84783	0.85185	0.85320	0.85752	0.86220	0.86736
504	0.83946	0.84806	0.85208	0.85342	0.85774	0.86242	0.86756
505	0.83972	0.84830	0.85231	0.85365	0.85797	0.86263	0.86777
506	0.83997	0.84855	0.85254	0.85388	0.85819	0.86284	0.86797
507	0.84022	0.84878	0.85277	0.85410	0.85841	0.86304	0.86817
508	0.84048	0.84902	0.85300	0.85433	0.85862	0.86326	0.86837
509	0.84072	0.84925	0.85323	0.85455	0.85884	0.86347	0.86857
510	0.84095	0.84948	0.85345	0.85477	0.85905	0.86367	0.86877
511	0.84118	0.84970	0.85367	0.85500	0.85927	0.86388	0.86896
512	0.84141	0.84993	0.85390	0.85522	0.85948	0.86408	0.86916
513	0.84167	0.85016	0.85412	0.85544	0.85970	0.86429	0.86936
514	0.84191	0.85039	0.85435	0.85566	0.85991	0.86450	0.86955
515	0.84216	0.85062	0.85457	0.85588	0.86012	0.86470	0.86975
516	0.84241	0.85084	0.85478	0.85610	0.86033	0.86490	0.86995
517	0.84264	0.85107	0.85500	0.85632	0.86054	0.86511	0.87014
518	0.84286	0.85130	0.85522	0.85653	0.86075	0.86531	0.87033
519	0.84310	0.85152	0.85545	0.85675	0.86095	0.86551	0.87053
520	0.84335	0.85175	0.85566	0.85696	0.86117	0.86571	0.87072
521	0.84359	0.85197	0.85587	0.85718	0.86137	0.86591	0.87091
522	0.84383	0.85219	0.85609	0.85739	0.86158	0.86611	0.87110
523	0.84410	0.85242	0.85631	0.85760	0.86179	0.86631	0.87130
524	0.84434	0.85264	0.85652	0.85782	0.86199	0.86651	0.87149
525	0.84456	0.85285	0.85674	0.85803	0.86220	0.86670	0.87168
526	0.84480	0.85308	0.85695	0.85825	0.86241	0.86690	0.87186
527	0.84503	0.85330	0.85717	0.85845	0.86261	0.86710	0.87205
528	0.84524	0.85351	0.85738	0.85866	0.86281	0.86729	0.87224
529	0.84546	0.85373	0.85759	0.85887	0.86301	0.86749	0.87243
530	0.84570	0.85395	0.85780	0.85908	0.86321	0.86768	0.87261
531	0.84594	0.85416	0.85801	0.85929	0.86341	0.86788	0.87280
532	0.84614	0.85438	0.85822	0.85949	0.86361	0.86807	0.87298
533	0.84639	0.85460	0.85842	0.85970	0.86381	0.86827	0.87317
534	0.84660	0.85482	0.85864	0.85991	0.86401	0.86846	0.87335
535	0.84683	0.85504	0.85885	0.86011	0.86421	0.86865	0.87353
536	0.84706	0.85525	0.85906	0.86031	0.86441	0.86883	0.87371
537	0.84727	0.85545	0.85927	0.86052	0.86461	0.86903	0.87390
538	0.84749	0.85566	0.85947	0.86072	0.86480	0.86922	0.87408
539	0.84770	0.85587	0.85967	0.86093	0.86500	0.86941	0.87426
540	0.84793	0.85608	0.85988	0.86113	0.86520	0.86959	0.87444
541	0.84814	0.85629	0.86009	0.86133	0.86539	0.86978	0.87462
542	0.84839	0.85650	0.86029	0.86152	0.86559	0.86997	0.87480
543	0.84861	0.85671	0.86049	0.86173	0.86578	0.87016	0.87498
544	0.84883	0.85691	0.86069	0.86192	0.86597	0.87035	0.87516
545	0.84907	0.85713	0.86089	0.86212	0.86616	0.87053	0.87534
546	0.84927	0.85733	0.86108	0.86233	0.86636	0.87072	0.87552
547	0.84948	0.85753	0.86128	0.86252	0.86655	0.87090	0.87569
548	0.84970	0.85773	0.86149	0.86272	0.86674	0.87108	0.87587
549	0.84993	0.85793	0.86168	0.86292	0.86693	0.87127	0.87605
550	0.85014	0.85814	0.86187	0.86311	0.86712	0.87145	0.87622
551	0.85038	0.85835	0.86207	0.86331	0.86731	0.87163	0.87640
552	0.85058	0.85856	0.86227	0.86350	0.86750	0.87181	0.87657
553	0.85080	0.85876	0.86246	0.86370	0.86769	0.87199	0.87674

N	0.1%	0.5%	1%	1.25%	2.5%	5%	10%
554	0.85101	0.85896	0.86266	0.86389	0.86788	0.87218	0.87692
555	0.85122	0.85916	0.86285	0.86408	0.86806	0.87235	0.87709
556	0.85144	0.85936	0.86305	0.86427	0.86825	0.87253	0.87726
557	0.85165	0.85956	0.86324	0.86447	0.86843	0.87271	0.87743
558	0.85188	0.85976	0.86343	0.86466	0.86862	0.87289	0.87761
559	0.85208	0.85996	0.86362	0.86485	0.86880	0.87307	0.87778
560	0.85228	0.86015	0.86382	0.86504	0.86899	0.87325	0.87794
561	0.85250	0.86035	0.86401	0.86523	0.86917	0.87343	0.87811
562	0.85269	0.86056	0.86419	0.86542	0.86935	0.87360	0.87828
563	0.85290	0.86075	0.86439	0.86561	0.86953	0.87378	0.87845
564	0.85310	0.86095	0.86458	0.86579	0.86972	0.87395	0.87862
565	0.85331	0.86114	0.86477	0.86599	0.86990	0.87413	0.87878
566	0.85352	0.86134	0.86496	0.86618	0.87008	0.87430	0.87895
567	0.85372	0.86152	0.86515	0.86636	0.87026	0.87447	0.87912
568	0.85393	0.86172	0.86534	0.86654	0.87044	0.87464	0.87928
569	0.85412	0.86191	0.86552	0.86673	0.87062	0.87482	0.87945
570	0.85434	0.86210	0.86570	0.86691	0.87079	0.87499	0.87961
571	0.85453	0.86230	0.86589	0.86709	0.87097	0.87516	0.87978
572	0.85473	0.86249	0.86608	0.86728	0.87115	0.87533	0.87994
573	0.85491	0.86268	0.86627	0.86746	0.87133	0.87550	0.88010
574	0.85513	0.86287	0.86645	0.86764	0.87150	0.87567	0.88026
575	0.85532	0.86305	0.86662	0.86782	0.87168	0.87584	0.88043
576	0.85553	0.86323	0.86680	0.86800	0.87186	0.87601	0.88059
577	0.85573	0.86342	0.86699	0.86819	0.87203	0.87617	0.88075
578	0.85592	0.86361	0.86717	0.86836	0.87220	0.87634	0.88091
579	0.85612	0.86380	0.86735	0.86855	0.87237	0.87651	0.88107
580	0.85635	0.86397	0.86753	0.86871	0.87255	0.87668	0.88123
581	0.85654	0.86416	0.86771	0.86890	0.87272	0.87684	0.88139
582	0.85673	0.86435	0.86789	0.86907	0.87289	0.87701	0.88155
583	0.85691	0.86453	0.86807	0.86925	0.87306	0.87718	0.88171
584	0.85711	0.86472	0.86825	0.86943	0.87323	0.87734	0.88186
585	0.85732	0.86489	0.86842	0.86960	0.87340	0.87750	0.88202
586	0.85751	0.86507	0.86860	0.86977	0.87357	0.87767	0.88218
587	0.85769	0.86526	0.86878	0.86995	0.87374	0.87783	0.88233
588	0.85787	0.86544	0.86897	0.87013	0.87392	0.87799	0.88249
589	0.85806	0.86562	0.86914	0.87030	0.87409	0.87815	0.88265
590	0.85824	0.86580	0.86932	0.87047	0.87425	0.87831	0.88280
591	0.85843	0.86598	0.86949	0.87065	0.87442	0.87848	0.88296
592	0.85861	0.86616	0.86967	0.87082	0.87458	0.87864	0.88311
593	0.85880	0.86634	0.86984	0.87099	0.87476	0.87880	0.88326
594	0.85900	0.86652	0.87001	0.87117	0.87492	0.87896	0.88342
595	0.85919	0.86669	0.87019	0.87134	0.87508	0.87912	0.88357
596	0.85939	0.86686	0.87036	0.87152	0.87525	0.87927	0.88372
597	0.85957	0.86704	0.87053	0.87168	0.87541	0.87943	0.88388
598	0.85975	0.86721	0.87070	0.87186	0.87557	0.87959	0.88403
599	0.85993	0.86740	0.87087	0.87202	0.87574	0.87975	0.88418
600	0.86011	0.86757	0.87104	0.87219	0.87591	0.87990	0.88433
601	0.86030	0.86774	0.87121	0.87236	0.87607	0.88006	0.88448
602	0.86050	0.86792	0.87138	0.87253	0.87623	0.88022	0.88463
603	0.86068	0.86809	0.87154	0.87270	0.87639	0.88037	0.88478
604	0.86085	0.86826	0.87171	0.87286	0.87655	0.88053	0.88493
605	0.86103	0.86844	0.87188	0.87303	0.87671	0.88068	0.88508
606	0.86121	0.86860	0.87205	0.87319	0.87687	0.88084	0.88522
607	0.86140	0.86878	0.87222	0.87336	0.87703	0.88099	0.88537
608	0.86159	0.86895	0.87239	0.87352	0.87719	0.88114	0.88552
609	0.86177	0.86912	0.87255	0.87368	0.87734	0.88130	0.88566
610	0.86195	0.86930	0.87271	0.87385	0.87750	0.88145	0.88581
611	0.86213	0.86946	0.87287	0.87401	0.87766	0.88160	0.88596
612	0.86232	0.86963	0.87305	0.87418	0.87782	0.88176	0.88610
613	0.86249	0.86980	0.87321	0.87434	0.87798	0.88191	0.88625
614	0.86267	0.86997	0.87337	0.87450	0.87813	0.88206	0.88640
615	0.86284	0.87014	0.87353	0.87466	0.87829	0.88221	0.88654
616	0.86302	0.87031	0.87369	0.87482	0.87845	0.88236	0.88669
617	0.86321	0.87047	0.87385	0.87498	0.87861	0.88251	0.88683
618	0.86338	0.87064	0.87402	0.87515	0.87876	0.88266	0.88697
619	0.86356	0.87081	0.87418	0.87531	0.87891	0.88281	0.88712
620	0.86373	0.87097	0.87435	0.87547	0.87907	0.88296	0.88726
621	0.86391	0.87113	0.87450	0.87563	0.87923	0.88311	0.88740
622	0.86409	0.87130	0.87466	0.87578	0.87938	0.88326	0.88754
623	0.86427	0.87147	0.87482	0.87594	0.87953	0.88340	0.88768
624	0.86444	0.87163	0.87499	0.87609	0.87968	0.88355	0.88783
625	0.86462	0.87179	0.87515	0.87625	0.87983	0.88370	0.88797
626	0.86479	0.87195	0.87530	0.87641	0.87998	0.88384	0.88811
627	0.86496	0.87211	0.87546	0.87656	0.88013	0.88399	0.88825

N	0.1%	0.5%	1%	1.25%	2.5%	5%	10%
628	0.86513	0.87228	0.87562	0.87672	0.88029	0.88414	0.88838
629	0.86530	0.87244	0.87577	0.87688	0.88043	0.88428	0.88852
630	0.86548	0.87260	0.87593	0.87703	0.88058	0.88443	0.88866
631	0.86565	0.87276	0.87608	0.87719	0.88073	0.88457	0.88880
632	0.86582	0.87292	0.87623	0.87734	0.88088	0.88472	0.88894
633	0.86598	0.87308	0.87639	0.87749	0.88103	0.88486	0.88907
634	0.86615	0.87323	0.87654	0.87764	0.88118	0.88500	0.88921
635	0.86633	0.87339	0.87670	0.87780	0.88133	0.88514	0.88935
636	0.86649	0.87355	0.87686	0.87796	0.88147	0.88529	0.88948
637	0.86667	0.87371	0.87701	0.87810	0.88162	0.88543	0.88962
638	0.86685	0.87387	0.87716	0.87826	0.88177	0.88557	0.88976
639	0.86701	0.87403	0.87732	0.87841	0.88191	0.88571	0.88989
640	0.86717	0.87419	0.87746	0.87856	0.88206	0.88585	0.89003
641	0.86734	0.87433	0.87762	0.87870	0.88220	0.88599	0.89016
642	0.86749	0.87450	0.87776	0.87885	0.88234	0.88613	0.89030
643	0.86765	0.87464	0.87791	0.87900	0.88249	0.88627	0.89043
644	0.86782	0.87480	0.87806	0.87915	0.88264	0.88641	0.89056
645	0.86798	0.87495	0.87821	0.87930	0.88278	0.88655	0.89069
646	0.86815	0.87511	0.87836	0.87945	0.88292	0.88669	0.89083
647	0.86830	0.87526	0.87851	0.87959	0.88307	0.88682	0.89096
648	0.86846	0.87542	0.87866	0.87974	0.88321	0.88696	0.89109
649	0.86863	0.87557	0.87881	0.87988	0.88336	0.88710	0.89122
650	0.86879	0.87572	0.87896	0.88003	0.88350	0.88723	0.89136
651	0.86895	0.87587	0.87911	0.88018	0.88364	0.88737	0.89149
652	0.86911	0.87603	0.87925	0.88032	0.88378	0.88751	0.89162
653	0.86928	0.87618	0.87939	0.88047	0.88392	0.88764	0.89175
654	0.86945	0.87633	0.87954	0.88061	0.88406	0.88778	0.89188
655	0.86959	0.87648	0.87968	0.88075	0.88420	0.88792	0.89201
656	0.86975	0.87664	0.87983	0.88090	0.88435	0.88805	0.89214
657	0.86990	0.87679	0.87998	0.88104	0.88448	0.88819	0.89227
658	0.87005	0.87693	0.88013	0.88119	0.88462	0.88832	0.89240
659	0.87022	0.87709	0.88027	0.88133	0.88476	0.88845	0.89252
660	0.87037	0.87723	0.88041	0.88147	0.88489	0.88859	0.89265
661	0.87053	0.87738	0.88056	0.88161	0.88503	0.88872	0.89278
662	0.87068	0.87753	0.88070	0.88175	0.88517	0.88885	0.89291
663	0.87083	0.87767	0.88084	0.88190	0.88531	0.88898	0.89303
664	0.87098	0.87782	0.88099	0.88204	0.88544	0.88912	0.89316
665	0.87113	0.87797	0.88113	0.88219	0.88558	0.88925	0.89329
666	0.87131	0.87811	0.88127	0.88232	0.88572	0.88938	0.89341
667	0.87146	0.87826	0.88141	0.88246	0.88585	0.88951	0.89354
668	0.87161	0.87840	0.88155	0.88260	0.88599	0.88964	0.89366
669	0.87177	0.87855	0.88169	0.88274	0.88612	0.88977	0.89379
670	0.87193	0.87869	0.88183	0.88287	0.88626	0.88990	0.89391
671	0.87208	0.87883	0.88198	0.88301	0.88639	0.89003	0.89404
672	0.87223	0.87898	0.88211	0.88315	0.88653	0.89016	0.89416
673	0.87238	0.87913	0.88225	0.88329	0.88666	0.89029	0.89429
674	0.87254	0.87926	0.88239	0.88343	0.88680	0.89042	0.89441
675	0.87269	0.87941	0.88253	0.88357	0.88693	0.89054	0.89453
676	0.87283	0.87955	0.88267	0.88370	0.88706	0.89067	0.89465
677	0.87299	0.87970	0.88281	0.88384	0.88719	0.89080	0.89477
678	0.87314	0.87984	0.88295	0.88398	0.88733	0.89093	0.89490
679	0.87330	0.87998	0.88309	0.88412	0.88746	0.89106	0.89502
680	0.87346	0.88012	0.88322	0.88425	0.88759	0.89118	0.89514
681	0.87361	0.88025	0.88336	0.88439	0.88772	0.89131	0.89526
682	0.87375	0.88039	0.88349	0.88452	0.88785	0.89143	0.89538
683	0.87389	0.88054	0.88363	0.88466	0.88798	0.89156	0.89550
684	0.87405	0.88068	0.88376	0.88480	0.88811	0.89168	0.89562
685	0.87418	0.88083	0.88390	0.88493	0.88824	0.89181	0.89574
686	0.87433	0.88097	0.88403	0.88506	0.88836	0.89193	0.89586
687	0.87449	0.88110	0.88417	0.88519	0.88849	0.89205	0.89598
688	0.87463	0.88124	0.88431	0.88533	0.88862	0.89218	0.89610
689	0.87477	0.88138	0.88444	0.88546	0.88875	0.89230	0.89622
690	0.87492	0.88152	0.88457	0.88559	0.88888	0.89243	0.89634
691	0.87506	0.88166	0.88471	0.88572	0.88900	0.89255	0.89645
692	0.87521	0.88180	0.88483	0.88585	0.88913	0.89267	0.89657
693	0.87534	0.88193	0.88497	0.88598	0.88926	0.89280	0.89669
694	0.87550	0.88206	0.88510	0.88611	0.88939	0.89292	0.89681
695	0.87564	0.88220	0.88524	0.88624	0.88951	0.89304	0.89692
696	0.87578	0.88234	0.88537	0.88638	0.88964	0.89316	0.89704
697	0.87593	0.88247	0.88550	0.88651	0.88977	0.89329	0.89716
698	0.87608	0.88261	0.88563	0.88663	0.88989	0.89341	0.89727
699	0.87623	0.88275	0.88576	0.88676	0.89001	0.89353	0.89739
700	0.87636	0.88288	0.88589	0.88689	0.89014	0.89365	0.89750
701	0.87648	0.88301	0.88602	0.88702	0.89026	0.89377	0.89762

N	0.1%	0.5%	1%	1.25%	2.5%	5%	10%
702	0.87662	0.88314	0.88615	0.88715	0.89038	0.89389	0.89773
703	0.87677	0.88328	0.88627	0.88728	0.89051	0.89401	0.89785
704	0.87690	0.88341	0.88640	0.88740	0.89063	0.89413	0.89796
705	0.87704	0.88355	0.88653	0.88753	0.89075	0.89425	0.89808
706	0.87717	0.88368	0.88666	0.88766	0.89087	0.89436	0.89819
707	0.87732	0.88381	0.88679	0.88779	0.89100	0.89448	0.89831
708	0.87746	0.88394	0.88691	0.88791	0.89112	0.89460	0.89842
709	0.87760	0.88408	0.88704	0.88804	0.89125	0.89472	0.89853
710	0.87774	0.88421	0.88717	0.88816	0.89137	0.89484	0.89864
711	0.87787	0.88434	0.88730	0.88829	0.89149	0.89495	0.89875
712	0.87802	0.88447	0.88743	0.88842	0.89161	0.89507	0.89887
713	0.87815	0.88460	0.88755	0.88854	0.89173	0.89519	0.89898
714	0.87828	0.88473	0.88767	0.88866	0.89185	0.89531	0.89909
715	0.87841	0.88485	0.88780	0.88879	0.89197	0.89542	0.89920
716	0.87856	0.88498	0.88793	0.88891	0.89209	0.89554	0.89932
717	0.87869	0.88512	0.88805	0.88903	0.89221	0.89565	0.89943
718	0.87885	0.88525	0.88817	0.88916	0.89234	0.89577	0.89954
719	0.87898	0.88537	0.88830	0.88929	0.89246	0.89588	0.89965
720	0.87910	0.88550	0.88842	0.88941	0.89258	0.89599	0.89976
721	0.87924	0.88563	0.88855	0.88953	0.89270	0.89611	0.89987
722	0.87938	0.88576	0.88867	0.88965	0.89281	0.89622	0.89998
723	0.87951	0.88589	0.88880	0.88977	0.89293	0.89634	0.90008
724	0.87964	0.88602	0.88891	0.88990	0.89305	0.89645	0.90019
725	0.87977	0.88615	0.88904	0.89001	0.89316	0.89656	0.90030
726	0.87990	0.88627	0.88917	0.89014	0.89328	0.89667	0.90041
727	0.88004	0.88640	0.88929	0.89026	0.89340	0.89679	0.90052
728	0.88016	0.88652	0.88941	0.89038	0.89351	0.89690	0.90062
729	0.88030	0.88665	0.88954	0.89050	0.89363	0.89701	0.90073
730	0.88043	0.88677	0.88966	0.89062	0.89375	0.89712	0.90084
731	0.88057	0.88690	0.88978	0.89074	0.89387	0.89723	0.90095
732	0.88071	0.88702	0.88990	0.89086	0.89398	0.89734	0.90105
733	0.88084	0.88714	0.89002	0.89098	0.89410	0.89746	0.90116
734	0.88097	0.88727	0.89014	0.89110	0.89421	0.89757	0.90127
735	0.88110	0.88739	0.89026	0.89121	0.89432	0.89768	0.90137
736	0.88124	0.88751	0.89038	0.89133	0.89444	0.89779	0.90148
737	0.88137	0.88763	0.89050	0.89145	0.89456	0.89790	0.90158
738	0.88150	0.88776	0.89061	0.89157	0.89467	0.89801	0.90169
739	0.88163	0.88788	0.89073	0.89169	0.89479	0.89812	0.90179
740	0.88176	0.88800	0.89085	0.89181	0.89490	0.89823	0.90190
741	0.88189	0.88812	0.89096	0.89192	0.89501	0.89834	0.90200
742	0.88202	0.88824	0.89108	0.89204	0.89513	0.89845	0.90211
743	0.88216	0.88836	0.89120	0.89216	0.89524	0.89855	0.90221
744	0.88228	0.88848	0.89132	0.89228	0.89535	0.89866	0.90231
745	0.88240	0.88860	0.89143	0.89239	0.89546	0.89877	0.90242
746	0.88252	0.88872	0.89155	0.89251	0.89558	0.89887	0.90252
747	0.88265	0.88884	0.89167	0.89262	0.89569	0.89898	0.90262
748	0.88278	0.88896	0.89179	0.89274	0.89580	0.89909	0.90273
749	0.88291	0.88908	0.89190	0.89285	0.89591	0.89920	0.90283
750	0.88304	0.88920	0.89202	0.89296	0.89602	0.89930	0.90293
751	0.88316	0.88932	0.89213	0.89308	0.89613	0.89941	0.90304
752	0.88328	0.88944	0.89225	0.89319	0.89624	0.89952	0.90314
753	0.88341	0.88956	0.89236	0.89330	0.89635	0.89962	0.90324
754	0.88355	0.88967	0.89248	0.89342	0.89646	0.89973	0.90334
755	0.88367	0.88979	0.89259	0.89353	0.89657	0.89983	0.90344
756	0.88379	0.88991	0.89271	0.89365	0.89668	0.89994	0.90354
757	0.88392	0.89003	0.89282	0.89376	0.89679	0.90005	0.90364
758	0.88405	0.89014	0.89294	0.89387	0.89690	0.90015	0.90374
759	0.88417	0.89025	0.89305	0.89398	0.89701	0.90025	0.90384
760	0.88429	0.89037	0.89316	0.89409	0.89712	0.90036	0.90394
761	0.88441	0.89049	0.89327	0.89421	0.89723	0.90046	0.90405
762	0.88452	0.89060	0.89339	0.89432	0.89734	0.90057	0.90414
763	0.88464	0.89072	0.89350	0.89443	0.89744	0.90067	0.90424
764	0.88476	0.89083	0.89361	0.89454	0.89755	0.90077	0.90434
765	0.88489	0.89095	0.89373	0.89465	0.89766	0.90088	0.90444
766	0.88501	0.89106	0.89383	0.89476	0.89776	0.90098	0.90454
767	0.88512	0.89117	0.89395	0.89487	0.89787	0.90108	0.90464
768	0.88525	0.89129	0.89406	0.89498	0.89797	0.90118	0.90474
769	0.88538	0.89140	0.89417	0.89509	0.89808	0.90129	0.90484
770	0.88550	0.89151	0.89428	0.89520	0.89818	0.90139	0.90493
771	0.88562	0.89163	0.89439	0.89531	0.89829	0.90149	0.90503
772	0.88575	0.89174	0.89450	0.89542	0.89839	0.90159	0.90513
773	0.88587	0.89184	0.89461	0.89553	0.89850	0.90169	0.90523
774	0.88598	0.89196	0.89472	0.89563	0.89860	0.90179	0.90533
775	0.88610	0.89207	0.89483	0.89574	0.89871	0.90189	0.90542

N	0.1%	0.5%	1%	1.25%	2.5%	5%	10%
776	0.88622	0.89218	0.89494	0.89584	0.89881	0.90199	0.90552
777	0.88632	0.89229	0.89505	0.89595	0.89892	0.90209	0.90561
778	0.88645	0.89240	0.89516	0.89606	0.89902	0.90220	0.90571
779	0.88657	0.89252	0.89527	0.89617	0.89912	0.90230	0.90581
780	0.88670	0.89263	0.89537	0.89628	0.89923	0.90239	0.90590
781	0.88682	0.89274	0.89548	0.89638	0.89933	0.90249	0.90600
782	0.88693	0.89285	0.89559	0.89649	0.89943	0.90259	0.90609
783	0.88705	0.89296	0.89569	0.89660	0.89953	0.90269	0.90619
784	0.88717	0.89308	0.89580	0.89670	0.89964	0.90279	0.90628
785	0.88729	0.89318	0.89590	0.89681	0.89974	0.90289	0.90638
786	0.88741	0.89330	0.89601	0.89691	0.89984	0.90299	0.90647
787	0.88753	0.89341	0.89612	0.89702	0.89994	0.90309	0.90657
788	0.88763	0.89352	0.89622	0.89712	0.90004	0.90318	0.90666
789	0.88775	0.89363	0.89633	0.89723	0.90014	0.90328	0.90675
790	0.88786	0.89374	0.89643	0.89733	0.90025	0.90338	0.90685
791	0.88798	0.89384	0.89654	0.89743	0.90035	0.90348	0.90694
792	0.88810	0.89395	0.89665	0.89754	0.90045	0.90357	0.90703
793	0.88821	0.89406	0.89675	0.89764	0.90055	0.90367	0.90713
794	0.88833	0.89416	0.89686	0.89774	0.90065	0.90377	0.90722
795	0.88845	0.89428	0.89696	0.89785	0.90075	0.90387	0.90731
796	0.88856	0.89438	0.89706	0.89795	0.90085	0.90396	0.90740
797	0.88867	0.89449	0.89717	0.89806	0.90095	0.90406	0.90750
798	0.88878	0.89459	0.89727	0.89816	0.90105	0.90415	0.90759
799	0.88890	0.89470	0.89737	0.89826	0.90115	0.90425	0.90768
800	0.88901	0.89480	0.89747	0.89837	0.90125	0.90435	0.90777
801	0.88913	0.89491	0.89758	0.89847	0.90135	0.90444	0.90786
802	0.88924	0.89502	0.89768	0.89857	0.90145	0.90453	0.90796
803	0.88935	0.89512	0.89778	0.89867	0.90155	0.90463	0.90805
804	0.88946	0.89522	0.89788	0.89877	0.90164	0.90473	0.90814
805	0.88957	0.89532	0.89799	0.89887	0.90174	0.90482	0.90823
806	0.88970	0.89543	0.89809	0.89897	0.90184	0.90491	0.90832
807	0.88981	0.89553	0.89819	0.89907	0.90193	0.90501	0.90841
808	0.88992	0.89564	0.89829	0.89917	0.90203	0.90510	0.90850
809	0.89003	0.89574	0.89839	0.89927	0.90213	0.90520	0.90859
810	0.89015	0.89585	0.89849	0.89938	0.90222	0.90529	0.90868
811	0.89026	0.89596	0.89860	0.89948	0.90232	0.90538	0.90877
812	0.89036	0.89605	0.89869	0.89957	0.90241	0.90548	0.90886
813	0.89046	0.89615	0.89879	0.89967	0.90251	0.90557	0.90895
814	0.89056	0.89626	0.89889	0.89977	0.90261	0.90566	0.90903
815	0.89069	0.89636	0.89899	0.89987	0.90271	0.90575	0.90912
816	0.89078	0.89647	0.89910	0.89997	0.90280	0.90585	0.90921
817	0.89089	0.89657	0.89919	0.90007	0.90290	0.90594	0.90930
818	0.89100	0.89667	0.89929	0.90016	0.90299	0.90603	0.90939
819	0.89111	0.89678	0.89940	0.90026	0.90309	0.90612	0.90948
820	0.89122	0.89688	0.89950	0.90036	0.90318	0.90621	0.90956
821	0.89132	0.89698	0.89959	0.90046	0.90328	0.90630	0.90965
822	0.89144	0.89708	0.89969	0.90056	0.90337	0.90640	0.90974
823	0.89154	0.89719	0.89979	0.90065	0.90346	0.90649	0.90982
824	0.89166	0.89728	0.89989	0.90075	0.90356	0.90658	0.90991
825	0.89177	0.89738	0.89999	0.90085	0.90365	0.90667	0.91000
826	0.89187	0.89748	0.90008	0.90094	0.90374	0.90676	0.91009
827	0.89196	0.89758	0.90018	0.90104	0.90383	0.90685	0.91017
828	0.89208	0.89768	0.90028	0.90113	0.90393	0.90694	0.91026
829	0.89218	0.89778	0.90037	0.90123	0.90402	0.90703	0.91035
830	0.89229	0.89788	0.90047	0.90133	0.90411	0.90712	0.91043
831	0.89239	0.89798	0.90057	0.90142	0.90421	0.90721	0.91052
832	0.89250	0.89808	0.90067	0.90152	0.90430	0.90729	0.91060
833	0.89260	0.89818	0.90076	0.90162	0.90439	0.90738	0.91069
834	0.89271	0.89828	0.90086	0.90171	0.90448	0.90747	0.91077
835	0.89282	0.89838	0.90096	0.90181	0.90457	0.90756	0.91086
836	0.89292	0.89847	0.90105	0.90190	0.90467	0.90765	0.91094
837	0.89303	0.89857	0.90115	0.90200	0.90476	0.90774	0.91103
838	0.89314	0.89867	0.90125	0.90209	0.90485	0.90782	0.91111
839	0.89324	0.89877	0.90134	0.90218	0.90494	0.90791	0.91120
840	0.89335	0.89887	0.90143	0.90228	0.90503	0.90800	0.91128
841	0.89345	0.89896	0.90153	0.90237	0.90512	0.90809	0.91136
842	0.89355	0.89906	0.90163	0.90247	0.90521	0.90818	0.91145
843	0.89365	0.89916	0.90172	0.90256	0.90530	0.90826	0.91153
844	0.89376	0.89926	0.90181	0.90265	0.90539	0.90835	0.91161
845	0.89385	0.89935	0.90190	0.90274	0.90548	0.90844	0.91170
846	0.89395	0.89945	0.90200	0.90284	0.90557	0.90852	0.91178
847	0.89404	0.89954	0.90209	0.90293	0.90566	0.90861	0.91186
848	0.89415	0.89964	0.90218	0.90302	0.90575	0.90870	0.91195
849	0.89426	0.89973	0.90228	0.90311	0.90584	0.90878	0.91203

N	0.1%	0.5%	1%	1.25%	2.5%	5%	10%
850	0.89436	0.89983	0.90237	0.90320	0.90592	0.90887	0.91211
851	0.89446	0.89992	0.90246	0.90329	0.90601	0.90895	0.91219
852	0.89457	0.90002	0.90255	0.90339	0.90610	0.90904	0.91228
853	0.89466	0.90011	0.90264	0.90348	0.90619	0.90913	0.91236
854	0.89476	0.90020	0.90274	0.90357	0.90628	0.90921	0.91244
855	0.89486	0.90030	0.90283	0.90366	0.90637	0.90929	0.91252
856	0.89497	0.90040	0.90292	0.90375	0.90645	0.90938	0.91260
857	0.89506	0.90049	0.90301	0.90384	0.90654	0.90946	0.91268
858	0.89517	0.90059	0.90310	0.90393	0.90663	0.90955	0.91276
859	0.89527	0.90068	0.90319	0.90402	0.90672	0.90963	0.91285
860	0.89537	0.90078	0.90328	0.90411	0.90681	0.90972	0.91293
861	0.89546	0.90087	0.90337	0.90420	0.90690	0.90980	0.91301
862	0.89555	0.90096	0.90346	0.90429	0.90698	0.90989	0.91309
863	0.89565	0.90105	0.90355	0.90438	0.90707	0.90997	0.91317
864	0.89575	0.90115	0.90364	0.90447	0.90715	0.91005	0.91325
865	0.89584	0.90124	0.90373	0.90456	0.90724	0.91014	0.91333
866	0.89595	0.90133	0.90382	0.90465	0.90733	0.91022	0.91341
867	0.89604	0.90142	0.90391	0.90474	0.90741	0.91030	0.91349
868	0.89614	0.90152	0.90400	0.90482	0.90750	0.91039	0.91357
869	0.89623	0.90161	0.90409	0.90491	0.90759	0.91047	0.91365
870	0.89634	0.90170	0.90418	0.90500	0.90767	0.91055	0.91373
871	0.89645	0.90179	0.90427	0.90508	0.90776	0.91064	0.91380
872	0.89653	0.90188	0.90435	0.90517	0.90784	0.91072	0.91388
873	0.89662	0.90197	0.90444	0.90526	0.90792	0.91080	0.91396
874	0.89673	0.90206	0.90453	0.90535	0.90801	0.91088	0.91404
875	0.89682	0.90215	0.90462	0.90543	0.90810	0.91096	0.91412
876	0.89692	0.90224	0.90471	0.90552	0.90818	0.91104	0.91420
877	0.89701	0.90233	0.90480	0.90561	0.90826	0.91112	0.91428
878	0.89710	0.90243	0.90489	0.90570	0.90834	0.91120	0.91435
879	0.89720	0.90252	0.90497	0.90578	0.90843	0.91129	0.91443
880	0.89729	0.90260	0.90506	0.90587	0.90851	0.91137	0.91451
881	0.89738	0.90269	0.90514	0.90596	0.90860	0.91145	0.91459
882	0.89748	0.90278	0.90523	0.90604	0.90868	0.91153	0.91466
883	0.89757	0.90287	0.90532	0.90613	0.90876	0.91161	0.91474
884	0.89767	0.90296	0.90540	0.90621	0.90885	0.91169	0.91482
885	0.89775	0.90305	0.90549	0.90630	0.90893	0.91177	0.91490
886	0.89784	0.90314	0.90557	0.90638	0.90901	0.91185	0.91497
887	0.89794	0.90323	0.90566	0.90647	0.90909	0.91193	0.91505
888	0.89803	0.90332	0.90574	0.90655	0.90918	0.91201	0.91513
889	0.89812	0.90340	0.90583	0.90664	0.90926	0.91209	0.91520
890	0.89822	0.90349	0.90592	0.90672	0.90934	0.91217	0.91528
891	0.89832	0.90358	0.90601	0.90681	0.90942	0.91224	0.91535
892	0.89842	0.90367	0.90609	0.90689	0.90950	0.91232	0.91543
893	0.89850	0.90375	0.90618	0.90697	0.90959	0.91240	0.91551
894	0.89860	0.90385	0.90626	0.90706	0.90967	0.91248	0.91558
895	0.89868	0.90394	0.90634	0.90714	0.90975	0.91256	0.91566
896	0.89877	0.90402	0.90643	0.90723	0.90983	0.91264	0.91573
897	0.89886	0.90411	0.90651	0.90731	0.90991	0.91272	0.91581
898	0.89896	0.90420	0.90660	0.90740	0.90999	0.91279	0.91588
899	0.89906	0.90428	0.90668	0.90748	0.91007	0.91287	0.91596
900	0.89915	0.90437	0.90677	0.90756	0.91016	0.91295	0.91603
901	0.89924	0.90446	0.90685	0.90765	0.91024	0.91303	0.91611
902	0.89934	0.90454	0.90693	0.90773	0.91032	0.91310	0.91618
903	0.89942	0.90462	0.90701	0.90781	0.91039	0.91318	0.91626
904	0.89951	0.90471	0.90710	0.90790	0.91048	0.91326	0.91633
905	0.89960	0.90479	0.90718	0.90798	0.91056	0.91333	0.91640
906	0.89969	0.90488	0.90727	0.90806	0.91063	0.91341	0.91648
907	0.89978	0.90497	0.90735	0.90814	0.91071	0.91349	0.91655
908	0.89986	0.90505	0.90743	0.90822	0.91079	0.91357	0.91662
909	0.89996	0.90514	0.90752	0.90830	0.91087	0.91364	0.91670
910	0.90006	0.90522	0.90760	0.90839	0.91095	0.91372	0.91677
911	0.90014	0.90531	0.90768	0.90847	0.91103	0.91380	0.91684
912	0.90023	0.90539	0.90776	0.90855	0.91111	0.91387	0.91692
913	0.90032	0.90548	0.90785	0.90863	0.91119	0.91395	0.91699
914	0.90041	0.90556	0.90793	0.90872	0.91127	0.91402	0.91706
915	0.90049	0.90564	0.90801	0.90879	0.91135	0.91410	0.91713
916	0.90058	0.90573	0.90809	0.90887	0.91142	0.91417	0.91721
917	0.90068	0.90581	0.90817	0.90895	0.91150	0.91425	0.91728
918	0.90076	0.90590	0.90825	0.90903	0.91158	0.91432	0.91735
919	0.90086	0.90598	0.90833	0.90911	0.91166	0.91440	0.91742
920	0.90095	0.90607	0.90841	0.90919	0.91173	0.91447	0.91749
921	0.90104	0.90615	0.90849	0.90927	0.91181	0.91455	0.91756
922	0.90113	0.90624	0.90858	0.90935	0.91189	0.91462	0.91764
923	0.90122	0.90632	0.90866	0.90943	0.91197	0.91469	0.91771

N	0.1%	0.5%	1%	1.25%	2.5%	5%	10%
924	0.90130	0.90640	0.90874	0.90951	0.91204	0.91477	0.91778
925	0.90138	0.90648	0.90882	0.90959	0.91212	0.91484	0.91785
926	0.90147	0.90656	0.90890	0.90967	0.91220	0.91492	0.91792
927	0.90156	0.90664	0.90898	0.90975	0.91227	0.91499	0.91799
928	0.90165	0.90672	0.90906	0.90983	0.91235	0.91506	0.91806
929	0.90173	0.90680	0.90913	0.90991	0.91242	0.91514	0.91813
930	0.90182	0.90688	0.90921	0.90998	0.91250	0.91521	0.91820
931	0.90191	0.90696	0.90929	0.91006	0.91257	0.91528	0.91827
932	0.90200	0.90705	0.90937	0.91014	0.91265	0.91536	0.91834
933	0.90209	0.90713	0.90945	0.91021	0.91272	0.91543	0.91841
934	0.90218	0.90721	0.90953	0.91029	0.91280	0.91550	0.91848
935	0.90226	0.90729	0.90961	0.91037	0.91287	0.91557	0.91855
936	0.90234	0.90737	0.90969	0.91045	0.91295	0.91565	0.91862
937	0.90242	0.90745	0.90977	0.91053	0.91302	0.91572	0.91869
938	0.90251	0.90753	0.90984	0.91061	0.91310	0.91579	0.91876
939	0.90260	0.90761	0.90992	0.91068	0.91317	0.91586	0.91883
940	0.90269	0.90769	0.90999	0.91076	0.91325	0.91594	0.91890
941	0.90277	0.90777	0.91007	0.91084	0.91332	0.91601	0.91897
942	0.90286	0.90785	0.91015	0.91091	0.91340	0.91608	0.91904
943	0.90294	0.90793	0.91023	0.91099	0.91347	0.91615	0.91911
944	0.90303	0.90801	0.91031	0.91107	0.91355	0.91622	0.91918
945	0.90311	0.90809	0.91039	0.91115	0.91362	0.91630	0.91925
946	0.90319	0.90817	0.91046	0.91122	0.91369	0.91637	0.91931
947	0.90327	0.90824	0.91054	0.91130	0.91377	0.91644	0.91938
948	0.90335	0.90833	0.91062	0.91137	0.91384	0.91651	0.91945
949	0.90344	0.90840	0.91069	0.91145	0.91392	0.91658	0.91952
950	0.90352	0.90848	0.91077	0.91153	0.91399	0.91665	0.91958
951	0.90360	0.90856	0.91084	0.91160	0.91406	0.91672	0.91965
952	0.90368	0.90864	0.91092	0.91168	0.91413	0.91679	0.91972
953	0.90377	0.90872	0.91100	0.91175	0.91421	0.91686	0.91979
954	0.90384	0.90880	0.91107	0.91183	0.91428	0.91693	0.91986
955	0.90393	0.90887	0.91115	0.91190	0.91435	0.91700	0.91992
956	0.90401	0.90895	0.91122	0.91198	0.91442	0.91707	0.91999
957	0.90409	0.90903	0.91130	0.91205	0.91450	0.91714	0.92006
958	0.90417	0.90911	0.91137	0.91213	0.91457	0.91721	0.92012
959	0.90425	0.90919	0.91145	0.91220	0.91464	0.91728	0.92019
960	0.90434	0.90926	0.91153	0.91228	0.91471	0.91735	0.92026
961	0.90442	0.90934	0.91160	0.91235	0.91479	0.91742	0.92032
962	0.90450	0.90941	0.91168	0.91242	0.91486	0.91749	0.92039
963	0.90458	0.90949	0.91175	0.91250	0.91493	0.91756	0.92046
964	0.90465	0.90957	0.91183	0.91257	0.91500	0.91763	0.92052
965	0.90473	0.90964	0.91190	0.91264	0.91507	0.91770	0.92059
966	0.90481	0.90972	0.91198	0.91272	0.91514	0.91776	0.92065
967	0.90491	0.90980	0.91204	0.91279	0.91521	0.91783	0.92072
968	0.90499	0.90988	0.91212	0.91286	0.91528	0.91790	0.92079
969	0.90506	0.90995	0.91219	0.91293	0.91536	0.91797	0.92085
970	0.90516	0.91002	0.91227	0.91301	0.91543	0.91804	0.92092
971	0.90523	0.91011	0.91234	0.91308	0.91550	0.91811	0.92098
972	0.90530	0.91018	0.91241	0.91316	0.91557	0.91817	0.92105
973	0.90538	0.91025	0.91249	0.91323	0.91564	0.91824	0.92111
974	0.90546	0.91033	0.91256	0.91330	0.91571	0.91831	0.92118
975	0.90555	0.91040	0.91263	0.91338	0.91578	0.91838	0.92124
976	0.90563	0.91048	0.91270	0.91344	0.91585	0.91844	0.92131
977	0.90571	0.91055	0.91277	0.91352	0.91592	0.91851	0.92137
978	0.90578	0.91063	0.91285	0.91359	0.91598	0.91858	0.92144
979	0.90585	0.91071	0.91292	0.91366	0.91605	0.91865	0.92150
980	0.90594	0.91078	0.91299	0.91373	0.91612	0.91871	0.92156
981	0.90602	0.91085	0.91306	0.91380	0.91619	0.91878	0.92163
982	0.90609	0.91093	0.91314	0.91387	0.91626	0.91885	0.92169
983	0.90617	0.91100	0.91321	0.91394	0.91633	0.91891	0.92176
984	0.90626	0.91108	0.91328	0.91401	0.91640	0.91898	0.92182
985	0.90634	0.91115	0.91335	0.91409	0.91647	0.91905	0.92188
986	0.90641	0.91122	0.91342	0.91416	0.91654	0.91911	0.92195
987	0.90649	0.91129	0.91349	0.91423	0.91660	0.91918	0.92201
988	0.90657	0.91137	0.91357	0.91430	0.91667	0.91925	0.92207
989	0.90664	0.91144	0.91364	0.91437	0.91674	0.91931	0.92214
990	0.90673	0.91151	0.91371	0.91444	0.91681	0.91938	0.92220
991	0.90680	0.91158	0.91378	0.91451	0.91688	0.91944	0.92226
992	0.90688	0.91165	0.91385	0.91458	0.91695	0.91951	0.92233
993	0.90696	0.91173	0.91392	0.91465	0.91702	0.91958	0.92239
994	0.90704	0.91180	0.91399	0.91472	0.91709	0.91964	0.92245
995	0.90712	0.91187	0.91406	0.91479	0.91715	0.91971	0.92251
996	0.90720	0.91194	0.91413	0.91486	0.91722	0.91977	0.92258
997	0.90727	0.91202	0.91420	0.91493	0.91729	0.91984	0.92264

N	0.1%	0.5%	1%	1.25%	2.5%	5%	10%
998	0.90735	0.91209	0.91427	0.91500	0.91735	0.91990	0.92270
999	0.90742	0.91216	0.91434	0.91507	0.91742	0.91997	0.92276
1000	0.90749	0.91223	0.91441	0.91513	0.91749	0.92003	0.92283

Table 5 – *GM8(4H4L)*. Critical values of upper significance levels for the Grubbs multiple-8 outlier test, applicable in situations where the four highest and four lowest observations are simultaneously identified as outliers, denoted as *GM8(4H4L)*.

N	0.1%	0.5%	1%	1.25%	2.5%	5%	10%
10	0.00000	0.00000	0.00000	0.00000	0.00000	0.00001	0.00005
11	0.00001	0.00005	0.00010	0.00012	0.00026	0.00054	0.00118
12	0.00015	0.00047	0.00077	0.00090	0.00150	0.00255	0.00445
13	0.00071	0.00169	0.00249	0.00282	0.00421	0.00637	0.00989
14	0.00196	0.00397	0.00545	0.00604	0.00841	0.01189	0.01717
15	0.00404	0.00737	0.00966	0.01056	0.01401	0.01887	0.02592
16	0.00696	0.01185	0.01501	0.01622	0.02080	0.02704	0.03575
17	0.01079	0.01727	0.02135	0.02290	0.02859	0.03614	0.04641
18	0.01539	0.02354	0.02853	0.03041	0.03718	0.04597	0.05767
19	0.02069	0.03050	0.03636	0.03854	0.04635	0.05628	0.06933
20	0.02671	0.03810	0.04477	0.04721	0.05597	0.06698	0.08120
21	0.03320	0.04614	0.05363	0.05636	0.06597	0.07791	0.09317
22	0.04012	0.05462	0.06282	0.06575	0.07615	0.08900	0.10523
23	0.04742	0.06337	0.07216	0.07534	0.08654	0.10015	0.11720
24	0.05504	0.07227	0.08174	0.08515	0.09697	0.11131	0.12911
25	0.06296	0.08132	0.09140	0.09503	0.10747	0.12242	0.14087
26	0.07101	0.09052	0.10113	0.10492	0.11791	0.13342	0.15246
27	0.07921	0.09979	0.11085	0.11475	0.12825	0.14429	0.16387
28	0.08751	0.10909	0.12057	0.12462	0.13856	0.15502	0.17505
29	0.09583	0.11829	0.13020	0.13439	0.14874	0.16561	0.18604
30	0.10419	0.12748	0.13983	0.14407	0.15878	0.17602	0.19678
31	0.11268	0.13661	0.14929	0.15369	0.16874	0.18626	0.20732
32	0.12105	0.14577	0.15867	0.16318	0.17849	0.19628	0.21758
33	0.12944	0.15484	0.16794	0.17250	0.18807	0.20612	0.22764
34	0.13781	0.16374	0.17707	0.18173	0.19755	0.21578	0.23742
35	0.14609	0.17251	0.18610	0.19084	0.20678	0.22519	0.24702
36	0.15427	0.18121	0.19499	0.19975	0.21588	0.23442	0.25638
37	0.16251	0.18975	0.20374	0.20853	0.22479	0.24348	0.26549
38	0.17041	0.19813	0.21223	0.21707	0.23348	0.25231	0.27442
39	0.17846	0.20635	0.22058	0.22554	0.24207	0.26096	0.28311
40	0.18611	0.21446	0.22878	0.23375	0.25047	0.26946	0.29159
41	0.19381	0.22243	0.23693	0.24189	0.25864	0.27771	0.29987
42	0.20156	0.23035	0.24486	0.24987	0.26672	0.28580	0.30799
43	0.20902	0.23809	0.25270	0.25773	0.27460	0.29371	0.31589
44	0.21641	0.24570	0.26039	0.26545	0.28231	0.30141	0.32357
45	0.22380	0.25312	0.26786	0.27297	0.28987	0.30897	0.33111
46	0.23097	0.26044	0.27524	0.28029	0.29729	0.31635	0.33846
47	0.23797	0.26763	0.28251	0.28761	0.30453	0.32359	0.34566
48	0.24475	0.27478	0.28960	0.29469	0.31161	0.33067	0.35270
49	0.25166	0.28169	0.29657	0.30164	0.31857	0.33760	0.35957
50	0.25855	0.28856	0.30340	0.30843	0.32537	0.34434	0.36625
51	0.26513	0.29527	0.31009	0.31513	0.33202	0.35098	0.37281
52	0.27191	0.30188	0.31665	0.32172	0.33855	0.35747	0.37925
53	0.27827	0.30834	0.32311	0.32818	0.34497	0.36382	0.38550
54	0.28458	0.31458	0.32940	0.33451	0.35123	0.37004	0.39165
55	0.29059	0.32083	0.33559	0.34062	0.35743	0.37615	0.39764
56	0.29657	0.32697	0.34172	0.34674	0.36345	0.38211	0.40351
57	0.30263	0.33292	0.34767	0.35271	0.36935	0.38795	0.40926
58	0.30855	0.33878	0.35350	0.35854	0.37518	0.39370	0.41488
59	0.31459	0.34461	0.35928	0.36425	0.38084	0.39932	0.42043
60	0.32019	0.35024	0.36488	0.36992	0.38639	0.40482	0.42581
61	0.32577	0.35584	0.37043	0.37540	0.39188	0.41018	0.43110
62	0.33129	0.36128	0.37588	0.38086	0.39724	0.41551	0.43628
63	0.33661	0.36660	0.38122	0.38616	0.40250	0.42070	0.44136
64	0.34185	0.37193	0.38642	0.39135	0.40765	0.42577	0.44633
65	0.34708	0.37710	0.39157	0.39648	0.41276	0.43074	0.45120
66	0.35234	0.38218	0.39662	0.40151	0.41772	0.43562	0.45599
67	0.35743	0.38723	0.40161	0.40649	0.42259	0.44041	0.46066
68	0.36249	0.39214	0.40652	0.41140	0.42737	0.44511	0.46526
69	0.36728	0.39700	0.41129	0.41612	0.43206	0.44973	0.46979
70	0.37222	0.40179	0.41604	0.42088	0.43667	0.45427	0.47420
71	0.37700	0.40652	0.42067	0.42549	0.44125	0.45871	0.47857
72	0.38167	0.41114	0.42522	0.42999	0.44574	0.46311	0.48283
73	0.38635	0.41568	0.42967	0.43444	0.45010	0.46740	0.48700

N	0.1%	0.5%	1%	1.25%	2.5%	5%	10%
74	0.39085	0.42008	0.43407	0.43882	0.45439	0.47164	0.49112
75	0.39536	0.42453	0.43845	0.44317	0.45865	0.47579	0.49519
76	0.39976	0.42880	0.44273	0.44738	0.46280	0.47986	0.49915
77	0.40430	0.43302	0.44684	0.45151	0.46687	0.48385	0.50305
78	0.40856	0.43722	0.45094	0.45564	0.47091	0.48778	0.50689
79	0.41291	0.44133	0.45502	0.45967	0.47491	0.49166	0.51066
80	0.41678	0.44541	0.45901	0.46359	0.47877	0.49549	0.51439
81	0.42093	0.44940	0.46290	0.46750	0.48261	0.49925	0.51801
82	0.42503	0.45324	0.46676	0.47135	0.48637	0.50293	0.52160
83	0.42908	0.45708	0.47055	0.47511	0.49010	0.50657	0.52511
84	0.43293	0.46089	0.47430	0.47881	0.49372	0.51011	0.52857
85	0.43678	0.46465	0.47799	0.48250	0.49733	0.51365	0.53198
86	0.44062	0.46833	0.48161	0.48611	0.50088	0.51711	0.53535
87	0.44431	0.47195	0.48519	0.48968	0.50435	0.52047	0.53865
88	0.44798	0.47560	0.48874	0.49323	0.50782	0.52380	0.54188
89	0.45167	0.47907	0.49219	0.49665	0.51119	0.52710	0.54508
90	0.45513	0.48258	0.49563	0.50001	0.51453	0.53037	0.54822
91	0.45864	0.48604	0.49898	0.50338	0.51782	0.53357	0.55131
92	0.46215	0.48941	0.50226	0.50672	0.52104	0.53673	0.55437
93	0.46560	0.49271	0.50556	0.50993	0.52421	0.53982	0.55738
94	0.46913	0.49598	0.50883	0.51317	0.52734	0.54286	0.56034
95	0.47242	0.49924	0.51200	0.51629	0.53042	0.54586	0.56325
96	0.47573	0.50243	0.51511	0.51943	0.53348	0.54884	0.56611
97	0.47899	0.50558	0.51824	0.52251	0.53646	0.55178	0.56895
98	0.48237	0.50872	0.52129	0.52554	0.53945	0.55467	0.57172
99	0.48541	0.51181	0.52431	0.52849	0.54236	0.55749	0.57448
100	0.48853	0.51484	0.52726	0.53144	0.54525	0.56031	0.57720
101	0.49167	0.51782	0.53018	0.53437	0.54809	0.56307	0.57988
102	0.49472	0.52072	0.53303	0.53722	0.55090	0.56581	0.58250
103	0.49766	0.52365	0.53593	0.54006	0.55364	0.56848	0.58510
104	0.50068	0.52646	0.53878	0.54287	0.55635	0.57111	0.58766
105	0.50358	0.52930	0.54152	0.54565	0.55906	0.57373	0.59020
106	0.50647	0.53211	0.54425	0.54837	0.56171	0.57629	0.59268
107	0.50932	0.53485	0.54693	0.55102	0.56435	0.57884	0.59514
108	0.51211	0.53762	0.54961	0.55371	0.56694	0.58137	0.59757
109	0.51495	0.54028	0.55227	0.55632	0.56948	0.58385	0.59994
110	0.51775	0.54292	0.55485	0.55890	0.57196	0.58629	0.60231
111	0.52045	0.54558	0.55743	0.56146	0.57445	0.58871	0.60464
112	0.52319	0.54819	0.55998	0.56398	0.57689	0.59109	0.60692
113	0.52583	0.55078	0.56249	0.56647	0.57931	0.59344	0.60919
114	0.52837	0.55325	0.56495	0.56892	0.58170	0.59575	0.61142
115	0.53095	0.55575	0.56742	0.57135	0.58406	0.59803	0.61363
116	0.53345	0.55814	0.56980	0.57371	0.58637	0.60032	0.61580
117	0.53614	0.56059	0.57219	0.57607	0.58868	0.60254	0.61796
118	0.53861	0.56302	0.57455	0.57842	0.59096	0.60472	0.62008
119	0.54118	0.56540	0.57687	0.58072	0.59320	0.60691	0.62218
120	0.54366	0.56775	0.57917	0.58297	0.59542	0.60905	0.62425
121	0.54604	0.57010	0.58140	0.58522	0.59760	0.61117	0.62629
122	0.54840	0.57238	0.58362	0.58747	0.59977	0.61326	0.62832
123	0.55078	0.57465	0.58585	0.58964	0.60193	0.61531	0.63032
124	0.55311	0.57689	0.58806	0.59184	0.60407	0.61738	0.63229
125	0.55547	0.57906	0.59022	0.59398	0.60616	0.61940	0.63426
126	0.55779	0.58125	0.59236	0.59608	0.60824	0.62140	0.63618
127	0.56007	0.58341	0.59447	0.59817	0.61025	0.62338	0.63810
128	0.56237	0.58553	0.59655	0.60024	0.61226	0.62533	0.63998
129	0.56446	0.58766	0.59863	0.60229	0.61424	0.62726	0.64185
130	0.56670	0.58974	0.60066	0.60432	0.61621	0.62916	0.64368
131	0.56884	0.59185	0.60268	0.60633	0.61816	0.63107	0.64550
132	0.57097	0.59385	0.60469	0.60830	0.62009	0.63294	0.64731
133	0.57299	0.59588	0.60667	0.61028	0.62199	0.63480	0.64908
134	0.57521	0.59791	0.60860	0.61221	0.62386	0.63662	0.65084
135	0.57723	0.59988	0.61052	0.61412	0.62574	0.63842	0.65258
136	0.57931	0.60180	0.61245	0.61601	0.62757	0.64019	0.65430
137	0.58135	0.60373	0.61435	0.61785	0.62941	0.64196	0.65600
138	0.58335	0.60562	0.61619	0.61970	0.63120	0.64372	0.65768
139	0.58533	0.60758	0.61799	0.62149	0.63296	0.64545	0.65934
140	0.58724	0.60946	0.61983	0.62332	0.63472	0.64715	0.66099
141	0.58925	0.61134	0.62164	0.62514	0.63647	0.64884	0.66262
142	0.59122	0.61314	0.62344	0.62692	0.63819	0.65051	0.66424
143	0.59305	0.61498	0.62522	0.62866	0.63993	0.65217	0.66581
144	0.59501	0.61675	0.62698	0.63040	0.64163	0.65380	0.66740
145	0.59682	0.61856	0.62870	0.63213	0.64332	0.65544	0.66896
146	0.59869	0.62031	0.63040	0.63385	0.64496	0.65703	0.67050
147	0.60056	0.62201	0.63212	0.63552	0.64659	0.65861	0.67202

N	0.1%	0.5%	1%	1.25%	2.5%	5%	10%
148	0.60240	0.62373	0.63378	0.63717	0.64821	0.66017	0.67353
149	0.60417	0.62544	0.63547	0.63885	0.64982	0.66173	0.67502
150	0.60592	0.62713	0.63716	0.64052	0.65140	0.66326	0.67650
151	0.60774	0.62882	0.63878	0.64214	0.65299	0.66478	0.67795
152	0.60954	0.63049	0.64041	0.64373	0.65453	0.66630	0.67940
153	0.61134	0.63213	0.64201	0.64532	0.65608	0.66780	0.68084
154	0.61305	0.63376	0.64360	0.64690	0.65761	0.66928	0.68227
155	0.61469	0.63536	0.64519	0.64845	0.65913	0.67076	0.68367
156	0.61634	0.63696	0.64672	0.64998	0.66062	0.67220	0.68506
157	0.61801	0.63851	0.64825	0.65148	0.66210	0.67364	0.68644
158	0.61961	0.64016	0.64977	0.65300	0.66358	0.67506	0.68780
159	0.62122	0.64168	0.65127	0.65452	0.66504	0.67646	0.68915
160	0.62278	0.64318	0.65278	0.65600	0.66648	0.67787	0.69049
161	0.62433	0.64474	0.65425	0.65747	0.66790	0.67924	0.69182
162	0.62593	0.64621	0.65571	0.65891	0.66930	0.68059	0.69313
163	0.62754	0.64769	0.65719	0.66036	0.67069	0.68195	0.69443
164	0.62917	0.64920	0.65866	0.66181	0.67209	0.68328	0.69571
165	0.63061	0.65065	0.66007	0.66323	0.67348	0.68461	0.69699
166	0.63208	0.65211	0.66148	0.66463	0.67482	0.68592	0.69826
167	0.63357	0.65355	0.66286	0.66599	0.67617	0.68722	0.69951
168	0.63507	0.65496	0.66425	0.66738	0.67751	0.68852	0.70075
169	0.63654	0.65638	0.66563	0.66875	0.67884	0.68981	0.70197
170	0.63803	0.65779	0.66699	0.67009	0.68015	0.69107	0.70319
171	0.63948	0.65922	0.66837	0.67142	0.68145	0.69233	0.70439
172	0.64091	0.66060	0.66969	0.67274	0.68273	0.69357	0.70560
173	0.64234	0.66194	0.67101	0.67409	0.68401	0.69480	0.70678
174	0.64373	0.66330	0.67236	0.67540	0.68527	0.69603	0.70795
175	0.64522	0.66464	0.67366	0.67671	0.68653	0.69723	0.70911
176	0.64664	0.66597	0.67493	0.67798	0.68777	0.69843	0.71026
177	0.64805	0.66728	0.67623	0.67926	0.68900	0.69962	0.71141
178	0.64941	0.66860	0.67752	0.68051	0.69022	0.70081	0.71255
179	0.65069	0.66988	0.67879	0.68175	0.69144	0.70198	0.71368
180	0.65205	0.67116	0.68002	0.68297	0.69264	0.70314	0.71479
181	0.65338	0.67246	0.68124	0.68420	0.69382	0.70428	0.71589
182	0.65470	0.67371	0.68247	0.68541	0.69501	0.70543	0.71699
183	0.65607	0.67496	0.68372	0.68663	0.69618	0.70655	0.71808
184	0.65737	0.67621	0.68492	0.68783	0.69733	0.70768	0.71914
185	0.65865	0.67741	0.68611	0.68902	0.69848	0.70880	0.72021
186	0.65999	0.67861	0.68727	0.69020	0.69962	0.70990	0.72127
187	0.66126	0.67981	0.68846	0.69136	0.70076	0.71098	0.72233
188	0.66257	0.68102	0.68960	0.69250	0.70188	0.71207	0.72337
189	0.66385	0.68218	0.69076	0.69364	0.70300	0.71313	0.72440
190	0.66512	0.68339	0.69192	0.69479	0.70410	0.71419	0.72542
191	0.66632	0.68455	0.69304	0.69590	0.70519	0.71526	0.72644
192	0.66752	0.68573	0.69415	0.69700	0.70627	0.71630	0.72743
193	0.66867	0.68686	0.69526	0.69810	0.70736	0.71734	0.72844
194	0.66990	0.68799	0.69637	0.69921	0.70842	0.71837	0.72943
195	0.67110	0.68913	0.69750	0.70031	0.70948	0.71939	0.73041
196	0.67227	0.69026	0.69859	0.70138	0.71054	0.72040	0.73139
197	0.67352	0.69136	0.69968	0.70244	0.71159	0.72142	0.73235
198	0.67469	0.69245	0.70074	0.70352	0.71262	0.72242	0.73331
199	0.67579	0.69356	0.70181	0.70457	0.71364	0.72340	0.73426
200	0.67690	0.69463	0.70287	0.70562	0.71466	0.72438	0.73520
201	0.67806	0.69572	0.70394	0.70669	0.71565	0.72535	0.73614
202	0.67917	0.69679	0.70497	0.70772	0.71667	0.72633	0.73706
203	0.68029	0.69784	0.70601	0.70876	0.71766	0.72729	0.73799
204	0.68140	0.69891	0.70705	0.70978	0.71865	0.72823	0.73891
205	0.68253	0.69995	0.70807	0.71079	0.71964	0.72920	0.73981
206	0.68365	0.70101	0.70909	0.71180	0.72062	0.73013	0.74071
207	0.68475	0.70202	0.71011	0.71279	0.72157	0.73106	0.74160
208	0.68578	0.70304	0.71110	0.71380	0.72252	0.73198	0.74249
209	0.68682	0.70408	0.71209	0.71476	0.72347	0.73291	0.74337
210	0.68789	0.70508	0.71307	0.71573	0.72441	0.73381	0.74424
211	0.68890	0.70607	0.71404	0.71668	0.72534	0.73470	0.74511
212	0.68995	0.70708	0.71500	0.71766	0.72627	0.73560	0.74597
213	0.69097	0.70806	0.71595	0.71861	0.72720	0.73648	0.74681
214	0.69206	0.70904	0.71691	0.71954	0.72811	0.73736	0.74766
215	0.69302	0.71001	0.71787	0.72049	0.72902	0.73824	0.74850
216	0.69406	0.71098	0.71881	0.72142	0.72993	0.73911	0.74933
217	0.69506	0.71191	0.71973	0.72233	0.73083	0.73999	0.75016
218	0.69610	0.71288	0.72066	0.72326	0.73171	0.74085	0.75098
219	0.69712	0.71382	0.72158	0.72418	0.73258	0.74170	0.75179
220	0.69814	0.71477	0.72249	0.72508	0.73345	0.74254	0.75261
221	0.69914	0.71568	0.72339	0.72599	0.73432	0.74337	0.75341

N	0.1%	0.5%	1%	1.25%	2.5%	5%	10%
222	0.70012	0.71659	0.72428	0.72686	0.73519	0.74422	0.75421
223	0.70108	0.71751	0.72519	0.72773	0.73604	0.74504	0.75500
224	0.70202	0.71844	0.72606	0.72862	0.73688	0.74586	0.75579
225	0.70301	0.71935	0.72694	0.72948	0.73773	0.74668	0.75657
226	0.70397	0.72025	0.72782	0.73035	0.73857	0.74748	0.75735
227	0.70487	0.72113	0.72869	0.73120	0.73940	0.74828	0.75812
228	0.70578	0.72201	0.72952	0.73206	0.74022	0.74907	0.75889
229	0.70669	0.72290	0.73038	0.73290	0.74105	0.74985	0.75964
230	0.70764	0.72377	0.73124	0.73373	0.74186	0.75064	0.76040
231	0.70858	0.72463	0.73208	0.73456	0.74266	0.75142	0.76114
232	0.70943	0.72546	0.73292	0.73539	0.74347	0.75220	0.76189
233	0.71033	0.72634	0.73375	0.73623	0.74427	0.75297	0.76262
234	0.71127	0.72718	0.73460	0.73706	0.74505	0.75373	0.76335
235	0.71211	0.72801	0.73542	0.73788	0.74583	0.75449	0.76408
236	0.71303	0.72886	0.73622	0.73868	0.74661	0.75524	0.76480
237	0.71395	0.72969	0.73703	0.73947	0.74738	0.75598	0.76552
238	0.71485	0.73052	0.73782	0.74027	0.74816	0.75673	0.76624
239	0.71568	0.73133	0.73862	0.74107	0.74893	0.75746	0.76695
240	0.71652	0.73215	0.73942	0.74185	0.74968	0.75820	0.76764
241	0.71743	0.73296	0.74020	0.74263	0.75044	0.75892	0.76834
242	0.71824	0.73378	0.74098	0.74340	0.75120	0.75965	0.76903
243	0.71908	0.73458	0.74177	0.74417	0.75193	0.76037	0.76972
244	0.71993	0.73537	0.74254	0.74494	0.75267	0.76108	0.77040
245	0.72079	0.73616	0.74331	0.74571	0.75341	0.76179	0.77108
246	0.72160	0.73694	0.74408	0.74647	0.75414	0.76250	0.77176
247	0.72249	0.73771	0.74483	0.74721	0.75486	0.76320	0.77243
248	0.72332	0.73847	0.74558	0.74795	0.75560	0.76390	0.77310
249	0.72415	0.73925	0.74632	0.74868	0.75631	0.76458	0.77376
250	0.72490	0.74002	0.74706	0.74941	0.75702	0.76528	0.77442
251	0.72570	0.74077	0.74778	0.75013	0.75772	0.76596	0.77508
252	0.72650	0.74151	0.74852	0.75084	0.75843	0.76663	0.77573
253	0.72727	0.74225	0.74924	0.75158	0.75914	0.76731	0.77637
254	0.72813	0.74297	0.74997	0.75228	0.75983	0.76797	0.77701
255	0.72890	0.74372	0.75067	0.75300	0.76053	0.76864	0.77765
256	0.72968	0.74447	0.75140	0.75371	0.76119	0.76931	0.77829
257	0.73046	0.74518	0.75211	0.75441	0.76187	0.76996	0.77891
258	0.73118	0.74589	0.75282	0.75510	0.76256	0.77061	0.77954
259	0.73192	0.74661	0.75350	0.75580	0.76323	0.77126	0.78016
260	0.73269	0.74732	0.75422	0.75648	0.76390	0.77190	0.78078
261	0.73344	0.74802	0.75489	0.75717	0.76455	0.77254	0.78139
262	0.73416	0.74876	0.75558	0.75785	0.76522	0.77318	0.78200
263	0.73493	0.74947	0.75627	0.75853	0.76588	0.77380	0.78260
264	0.73566	0.75017	0.75695	0.75920	0.76652	0.77443	0.78321
265	0.73641	0.75086	0.75761	0.75987	0.76717	0.77505	0.78381
266	0.73712	0.75156	0.75827	0.76054	0.76781	0.77568	0.78440
267	0.73788	0.75224	0.75896	0.76120	0.76844	0.77630	0.78499
268	0.73858	0.75293	0.75962	0.76185	0.76908	0.77691	0.78558
269	0.73928	0.75362	0.76026	0.76250	0.76973	0.77752	0.78617
270	0.73997	0.75428	0.76091	0.76315	0.77034	0.77813	0.78675
271	0.74068	0.75497	0.76156	0.76379	0.77096	0.77873	0.78733
272	0.74141	0.75564	0.76221	0.76444	0.77159	0.77933	0.78790
273	0.74211	0.75630	0.76286	0.76509	0.77220	0.77992	0.78847
274	0.74281	0.75696	0.76351	0.76572	0.77281	0.78052	0.78904
275	0.74350	0.75763	0.76413	0.76634	0.77342	0.78110	0.78960
276	0.74419	0.75828	0.76477	0.76697	0.77403	0.78169	0.79016
277	0.74490	0.75892	0.76540	0.76760	0.77464	0.78227	0.79072
278	0.74557	0.75956	0.76603	0.76823	0.77524	0.78285	0.79128
279	0.74627	0.76020	0.76664	0.76882	0.77584	0.78343	0.79183
280	0.74693	0.76082	0.76725	0.76942	0.77643	0.78401	0.79238
281	0.74761	0.76147	0.76787	0.77005	0.77702	0.78457	0.79292
282	0.74823	0.76210	0.76848	0.77066	0.77760	0.78514	0.79347
283	0.74889	0.76273	0.76910	0.77127	0.77819	0.78569	0.79400
284	0.74954	0.76334	0.76971	0.77185	0.77876	0.78625	0.79454
285	0.75020	0.76396	0.77032	0.77244	0.77934	0.78680	0.79507
286	0.75087	0.76457	0.77091	0.77304	0.77992	0.78736	0.79560
287	0.75150	0.76520	0.77151	0.77361	0.78048	0.78790	0.79612
288	0.75213	0.76579	0.77210	0.77421	0.78104	0.78844	0.79664
289	0.75278	0.76639	0.77270	0.77479	0.78161	0.78898	0.79716
290	0.75337	0.76701	0.77326	0.77536	0.78217	0.78953	0.79768
291	0.75403	0.76762	0.77386	0.77595	0.78273	0.79006	0.79820
292	0.75467	0.76823	0.77443	0.77652	0.78328	0.79060	0.79871
293	0.75527	0.76883	0.77500	0.77708	0.78383	0.79113	0.79921
294	0.75589	0.76940	0.77558	0.77764	0.78438	0.79166	0.79972
295	0.75650	0.76997	0.77614	0.77822	0.78492	0.79218	0.80022

N	0.1%	0.5%	1%	1.25%	2.5%	5%	10%
296	0.75712	0.77054	0.77671	0.77877	0.78546	0.79271	0.80073
297	0.75774	0.77113	0.77727	0.77934	0.78600	0.79323	0.80122
298	0.75835	0.77169	0.77782	0.77987	0.78653	0.79374	0.80171
299	0.75896	0.77224	0.77838	0.78042	0.78707	0.79425	0.80220
300	0.75959	0.77283	0.77893	0.78097	0.78759	0.79475	0.80269
301	0.76016	0.77339	0.77948	0.78150	0.78812	0.79526	0.80318
302	0.76076	0.77396	0.78003	0.78204	0.78864	0.79576	0.80366
303	0.76133	0.77451	0.78056	0.78258	0.78916	0.79626	0.80414
304	0.76195	0.77506	0.78110	0.78311	0.78967	0.79677	0.80462
305	0.76257	0.77560	0.78164	0.78363	0.79020	0.79726	0.80509
306	0.76315	0.77616	0.78217	0.78417	0.79071	0.79775	0.80557
307	0.76376	0.77670	0.78269	0.78470	0.79122	0.79824	0.80603
308	0.76432	0.77725	0.78324	0.78522	0.79173	0.79873	0.80650
309	0.76491	0.77777	0.78376	0.78575	0.79223	0.79922	0.80697
310	0.76546	0.77830	0.78429	0.78627	0.79273	0.79969	0.80743
311	0.76601	0.77883	0.78480	0.78680	0.79323	0.80017	0.80789
312	0.76654	0.77936	0.78533	0.78729	0.79373	0.80065	0.80835
313	0.76707	0.77989	0.78583	0.78780	0.79421	0.80112	0.80881
314	0.76763	0.78041	0.78634	0.78831	0.79470	0.80160	0.80926
315	0.76820	0.78094	0.78685	0.78883	0.79519	0.80208	0.80972
316	0.76871	0.78147	0.78734	0.78932	0.79568	0.80254	0.81016
317	0.76926	0.78199	0.78786	0.78982	0.79617	0.80301	0.81061
318	0.76980	0.78252	0.78835	0.79031	0.79664	0.80348	0.81105
319	0.77037	0.78302	0.78885	0.79082	0.79712	0.80394	0.81149
320	0.77095	0.78354	0.78935	0.79131	0.79760	0.80440	0.81193
321	0.77149	0.78405	0.78984	0.79179	0.79808	0.80485	0.81236
322	0.77200	0.78453	0.79033	0.79227	0.79854	0.80531	0.81280
323	0.77254	0.78504	0.79082	0.79276	0.79901	0.80576	0.81323
324	0.77308	0.78554	0.79131	0.79324	0.79948	0.80620	0.81366
325	0.77361	0.78602	0.79180	0.79372	0.79993	0.80665	0.81409
326	0.77410	0.78652	0.79229	0.79420	0.80039	0.80710	0.81451
327	0.77465	0.78700	0.79277	0.79468	0.80085	0.80754	0.81493
328	0.77519	0.78749	0.79324	0.79515	0.80131	0.80798	0.81535
329	0.77571	0.78798	0.79372	0.79562	0.80176	0.80842	0.81577
330	0.77622	0.78848	0.79418	0.79607	0.80220	0.80885	0.81619
331	0.77673	0.78895	0.79465	0.79654	0.80265	0.80928	0.81660
332	0.77724	0.78943	0.79512	0.79700	0.80310	0.80971	0.81702
333	0.77775	0.78991	0.79558	0.79746	0.80355	0.81015	0.81743
334	0.77828	0.79038	0.79604	0.79791	0.80400	0.81057	0.81784
335	0.77875	0.79087	0.79650	0.79838	0.80443	0.81100	0.81824
336	0.77925	0.79135	0.79695	0.79883	0.80487	0.81142	0.81865
337	0.77978	0.79182	0.79741	0.79929	0.80530	0.81184	0.81905
338	0.78029	0.79228	0.79786	0.79973	0.80574	0.81227	0.81945
339	0.78077	0.79274	0.79830	0.80018	0.80617	0.81268	0.81985
340	0.78123	0.79319	0.79876	0.80063	0.80661	0.81310	0.82024
341	0.78174	0.79364	0.79921	0.80107	0.80704	0.81350	0.82064
342	0.78221	0.79410	0.79965	0.80151	0.80746	0.81392	0.82104
343	0.78271	0.79455	0.80010	0.80195	0.80788	0.81433	0.82143
344	0.78316	0.79501	0.80054	0.80239	0.80830	0.81473	0.82182
345	0.78362	0.79545	0.80098	0.80282	0.80872	0.81513	0.82221
346	0.78411	0.79590	0.80142	0.80326	0.80914	0.81553	0.82260
347	0.78458	0.79634	0.80185	0.80368	0.80956	0.81594	0.82298
348	0.78505	0.79679	0.80228	0.80411	0.80997	0.81634	0.82336
349	0.78553	0.79723	0.80271	0.80453	0.81039	0.81673	0.82374
350	0.78597	0.79766	0.80313	0.80495	0.81079	0.81713	0.82412
351	0.78644	0.79811	0.80356	0.80538	0.81120	0.81752	0.82450
352	0.78689	0.79855	0.80398	0.80580	0.81161	0.81791	0.82487
353	0.78734	0.79898	0.80440	0.80621	0.81202	0.81830	0.82525
354	0.78777	0.79941	0.80482	0.80663	0.81242	0.81869	0.82562
355	0.78823	0.79984	0.80524	0.80704	0.81282	0.81908	0.82599
356	0.78871	0.80027	0.80564	0.80745	0.81322	0.81946	0.82636
357	0.78913	0.80069	0.80607	0.80785	0.81361	0.81984	0.82672
358	0.78960	0.80113	0.80648	0.80826	0.81401	0.82022	0.82708
359	0.79003	0.80153	0.80688	0.80867	0.81441	0.82060	0.82744
360	0.79046	0.80195	0.80730	0.80907	0.81480	0.82097	0.82781
361	0.79091	0.80236	0.80771	0.80947	0.81518	0.82135	0.82817
362	0.79133	0.80277	0.80811	0.80987	0.81557	0.82172	0.82852
363	0.79181	0.80320	0.80851	0.81026	0.81596	0.82209	0.82888
364	0.79222	0.80362	0.80891	0.81067	0.81634	0.82246	0.82923
365	0.79268	0.80404	0.80930	0.81106	0.81672	0.82283	0.82959
366	0.79311	0.80445	0.80970	0.81145	0.81710	0.82320	0.82994
367	0.79355	0.80486	0.81010	0.81185	0.81747	0.82356	0.83029
368	0.79396	0.80525	0.81049	0.81223	0.81785	0.82393	0.83063
369	0.79438	0.80566	0.81088	0.81262	0.81822	0.82429	0.83098

N	0.1%	0.5%	1%	1.25%	2.5%	5%	10%
370	0.79483	0.80606	0.81128	0.81299	0.81860	0.82464	0.83133
371	0.79527	0.80647	0.81166	0.81338	0.81898	0.82500	0.83167
372	0.79568	0.80686	0.81204	0.81377	0.81935	0.82536	0.83201
373	0.79607	0.80725	0.81243	0.81415	0.81971	0.82571	0.83235
374	0.79649	0.80765	0.81281	0.81453	0.82008	0.82606	0.83269
375	0.79691	0.80805	0.81320	0.81491	0.82045	0.82642	0.83303
376	0.79731	0.80843	0.81358	0.81528	0.82081	0.82677	0.83337
377	0.79771	0.80882	0.81396	0.81565	0.82118	0.82712	0.83370
378	0.79815	0.80920	0.81433	0.81602	0.82153	0.82746	0.83403
379	0.79854	0.80958	0.81470	0.81639	0.82189	0.82780	0.83436
380	0.79896	0.80998	0.81508	0.81677	0.82225	0.82815	0.83469
381	0.79937	0.81036	0.81546	0.81713	0.82260	0.82850	0.83502
382	0.79977	0.81074	0.81583	0.81749	0.82296	0.82884	0.83534
383	0.80016	0.81112	0.81618	0.81785	0.82331	0.82918	0.83567
384	0.80058	0.81150	0.81654	0.81822	0.82366	0.82952	0.83600
385	0.80095	0.81187	0.81691	0.81857	0.82400	0.82985	0.83632
386	0.80138	0.81225	0.81727	0.81894	0.82435	0.83019	0.83664
387	0.80177	0.81261	0.81763	0.81929	0.82470	0.83052	0.83696
388	0.80214	0.81298	0.81798	0.81965	0.82505	0.83085	0.83728
389	0.80256	0.81335	0.81835	0.82000	0.82539	0.83118	0.83760
390	0.80297	0.81372	0.81872	0.82036	0.82573	0.83151	0.83792
391	0.80338	0.81408	0.81907	0.82070	0.82607	0.83184	0.83823
392	0.80375	0.81446	0.81942	0.82106	0.82641	0.83217	0.83854
393	0.80413	0.81481	0.81976	0.82140	0.82674	0.83249	0.83886
394	0.80451	0.81518	0.82011	0.82175	0.82708	0.83281	0.83917
395	0.80489	0.81554	0.82046	0.82210	0.82742	0.83313	0.83947
396	0.80528	0.81589	0.82081	0.82245	0.82775	0.83346	0.83978
397	0.80562	0.81624	0.82116	0.82279	0.82808	0.83377	0.84009
398	0.80600	0.81660	0.82150	0.82313	0.82841	0.83409	0.84039
399	0.80640	0.81695	0.82186	0.82347	0.82874	0.83441	0.84070
400	0.80679	0.81731	0.82219	0.82381	0.82907	0.83472	0.84100
401	0.80717	0.81765	0.82253	0.82415	0.82940	0.83504	0.84130
402	0.80754	0.81800	0.82288	0.82449	0.82972	0.83535	0.84160
403	0.80793	0.81836	0.82321	0.82484	0.83004	0.83567	0.84190
404	0.80828	0.81870	0.82355	0.82516	0.83037	0.83598	0.84219
405	0.80865	0.81904	0.82388	0.82549	0.83069	0.83629	0.84249
406	0.80904	0.81938	0.82422	0.82582	0.83101	0.83660	0.84278
407	0.80940	0.81973	0.82455	0.82616	0.83133	0.83690	0.84307
408	0.80977	0.82007	0.82488	0.82649	0.83165	0.83721	0.84336
409	0.81013	0.82040	0.82521	0.82681	0.83197	0.83751	0.84366
410	0.81049	0.82074	0.82554	0.82713	0.83228	0.83781	0.84395
411	0.81085	0.82107	0.82587	0.82745	0.83259	0.83811	0.84424
412	0.81119	0.82141	0.82619	0.82777	0.83290	0.83841	0.84453
413	0.81153	0.82174	0.82652	0.82809	0.83321	0.83871	0.84481
414	0.81187	0.82208	0.82683	0.82840	0.83351	0.83901	0.84510
415	0.81226	0.82241	0.82716	0.82873	0.83382	0.83930	0.84538
416	0.81259	0.82272	0.82747	0.82905	0.83413	0.83960	0.84566
417	0.81292	0.82306	0.82780	0.82936	0.83443	0.83990	0.84595
418	0.81329	0.82338	0.82811	0.82967	0.83473	0.84019	0.84623
419	0.81364	0.82371	0.82843	0.82998	0.83504	0.84048	0.84651
420	0.81400	0.82404	0.82874	0.83029	0.83534	0.84077	0.84678
421	0.81432	0.82436	0.82905	0.83060	0.83564	0.84106	0.84707
422	0.81467	0.82467	0.82936	0.83091	0.83594	0.84135	0.84734
423	0.81497	0.82501	0.82967	0.83121	0.83624	0.84163	0.84762
424	0.81530	0.82533	0.82998	0.83153	0.83653	0.84192	0.84789
425	0.81562	0.82565	0.83029	0.83183	0.83683	0.84220	0.84816
426	0.81597	0.82596	0.83059	0.83213	0.83712	0.84249	0.84843
427	0.81630	0.82629	0.83089	0.83243	0.83741	0.84278	0.84871
428	0.81665	0.82659	0.83120	0.83273	0.83770	0.84306	0.84898
429	0.81697	0.82691	0.83151	0.83303	0.83800	0.84334	0.84924
430	0.81731	0.82720	0.83181	0.83333	0.83829	0.84362	0.84951
431	0.81764	0.82752	0.83212	0.83364	0.83857	0.84389	0.84978
432	0.81798	0.82783	0.83241	0.83393	0.83886	0.84417	0.85004
433	0.81831	0.82812	0.83271	0.83423	0.83914	0.84444	0.85031
434	0.81867	0.82845	0.83302	0.83452	0.83943	0.84472	0.85057
435	0.81900	0.82874	0.83331	0.83482	0.83972	0.84499	0.85083
436	0.81933	0.82905	0.83361	0.83511	0.84000	0.84527	0.85109
437	0.81966	0.82936	0.83390	0.83540	0.84028	0.84554	0.85135
438	0.81996	0.82966	0.83420	0.83568	0.84056	0.84581	0.85161
439	0.82028	0.82995	0.83448	0.83597	0.84084	0.84608	0.85187
440	0.82060	0.83025	0.83478	0.83626	0.84112	0.84635	0.85213
441	0.82090	0.83054	0.83506	0.83655	0.84140	0.84662	0.85239
442	0.82120	0.83084	0.83535	0.83684	0.84168	0.84689	0.85264
443	0.82150	0.83113	0.83564	0.83712	0.84195	0.84715	0.85290

N	0.1%	0.5%	1%	1.25%	2.5%	5%	10%
444	0.82178	0.83142	0.83592	0.83741	0.84223	0.84741	0.85315
445	0.82212	0.83173	0.83622	0.83769	0.84250	0.84767	0.85340
446	0.82241	0.83200	0.83650	0.83798	0.84277	0.84794	0.85365
447	0.82274	0.83231	0.83678	0.83826	0.84304	0.84820	0.85391
448	0.82308	0.83260	0.83707	0.83854	0.84331	0.84846	0.85415
449	0.82337	0.83289	0.83735	0.83882	0.84358	0.84872	0.85440
450	0.82370	0.83319	0.83763	0.83909	0.84385	0.84898	0.85465
451	0.82400	0.83348	0.83792	0.83938	0.84412	0.84924	0.85489
452	0.82429	0.83376	0.83818	0.83965	0.84438	0.84950	0.85514
453	0.82458	0.83404	0.83845	0.83991	0.84465	0.84975	0.85538
454	0.82489	0.83434	0.83873	0.84019	0.84492	0.85001	0.85563
455	0.82519	0.83462	0.83901	0.84046	0.84518	0.85026	0.85587
456	0.82550	0.83490	0.83928	0.84072	0.84545	0.85051	0.85611
457	0.82580	0.83518	0.83955	0.84099	0.84571	0.85077	0.85635
458	0.82609	0.83546	0.83982	0.84126	0.84597	0.85102	0.85659
459	0.82637	0.83575	0.84009	0.84153	0.84622	0.85127	0.85683
460	0.82670	0.83602	0.84036	0.84180	0.84648	0.85152	0.85708
461	0.82697	0.83630	0.84063	0.84206	0.84674	0.85177	0.85731
462	0.82729	0.83658	0.84091	0.84232	0.84699	0.85201	0.85755
463	0.82758	0.83685	0.84117	0.84259	0.84725	0.85226	0.85778
464	0.82785	0.83711	0.84143	0.84285	0.84750	0.85250	0.85802
465	0.82811	0.83738	0.84170	0.84312	0.84776	0.85275	0.85825
466	0.82841	0.83766	0.84196	0.84339	0.84801	0.85299	0.85849
467	0.82868	0.83794	0.84222	0.84365	0.84826	0.85323	0.85872
468	0.82897	0.83821	0.84248	0.84391	0.84851	0.85348	0.85895
469	0.82925	0.83848	0.84274	0.84416	0.84876	0.85372	0.85918
470	0.82955	0.83874	0.84300	0.84442	0.84901	0.85396	0.85941
471	0.82985	0.83901	0.84326	0.84468	0.84926	0.85420	0.85964
472	0.83009	0.83928	0.84352	0.84493	0.84951	0.85444	0.85987
473	0.83037	0.83955	0.84378	0.84519	0.84976	0.85467	0.86010
474	0.83066	0.83982	0.84404	0.84544	0.85000	0.85491	0.86032
475	0.83095	0.84007	0.84429	0.84570	0.85024	0.85514	0.86055
476	0.83123	0.84034	0.84455	0.84595	0.85048	0.85538	0.86078
477	0.83152	0.84059	0.84481	0.84620	0.85072	0.85561	0.86100
478	0.83179	0.84085	0.84506	0.84645	0.85097	0.85585	0.86123
479	0.83207	0.84112	0.84531	0.84670	0.85121	0.85608	0.86145
480	0.83234	0.84137	0.84556	0.84695	0.85146	0.85631	0.86167
481	0.83260	0.84163	0.84581	0.84720	0.85170	0.85655	0.86189
482	0.83290	0.84188	0.84605	0.84744	0.85193	0.85678	0.86211
483	0.83318	0.84214	0.84630	0.84769	0.85217	0.85700	0.86233
484	0.83347	0.84239	0.84655	0.84793	0.85240	0.85723	0.86255
485	0.83375	0.84264	0.84680	0.84818	0.85264	0.85746	0.86277
486	0.83404	0.84290	0.84704	0.84842	0.85288	0.85769	0.86299
487	0.83431	0.84315	0.84728	0.84867	0.85311	0.85791	0.86321
488	0.83457	0.84340	0.84752	0.84890	0.85334	0.85814	0.86342
489	0.83482	0.84364	0.84776	0.84915	0.85357	0.85837	0.86364
490	0.83509	0.84388	0.84801	0.84939	0.85381	0.85859	0.86385
491	0.83534	0.84414	0.84825	0.84963	0.85404	0.85881	0.86407
492	0.83562	0.84439	0.84849	0.84987	0.85427	0.85903	0.86428
493	0.83588	0.84463	0.84873	0.85010	0.85450	0.85925	0.86449
494	0.83613	0.84488	0.84897	0.85034	0.85473	0.85947	0.86471
495	0.83639	0.84512	0.84920	0.85057	0.85495	0.85969	0.86491
496	0.83666	0.84537	0.84945	0.85081	0.85518	0.85991	0.86512
497	0.83693	0.84562	0.84969	0.85105	0.85541	0.86013	0.86533
498	0.83719	0.84586	0.84992	0.85127	0.85564	0.86035	0.86554
499	0.83744	0.84610	0.85015	0.85150	0.85586	0.86057	0.86575
500	0.83770	0.84634	0.85039	0.85174	0.85609	0.86078	0.86596
501	0.83793	0.84658	0.85062	0.85197	0.85631	0.86100	0.86617
502	0.83816	0.84682	0.85086	0.85220	0.85653	0.86121	0.86637
503	0.83842	0.84705	0.85109	0.85244	0.85675	0.86143	0.86657
504	0.83867	0.84730	0.85133	0.85267	0.85698	0.86164	0.86678
505	0.83892	0.84753	0.85156	0.85290	0.85720	0.86186	0.86698
506	0.83918	0.84778	0.85179	0.85312	0.85742	0.86207	0.86719
507	0.83943	0.84801	0.85202	0.85335	0.85764	0.86228	0.86739
508	0.83969	0.84824	0.85224	0.85358	0.85786	0.86249	0.86759
509	0.83995	0.84847	0.85247	0.85380	0.85808	0.86270	0.86779
510	0.84019	0.84870	0.85270	0.85403	0.85829	0.86291	0.86799
511	0.84043	0.84893	0.85291	0.85425	0.85851	0.86312	0.86819
512	0.84067	0.84917	0.85314	0.85447	0.85872	0.86332	0.86839
513	0.84091	0.84940	0.85336	0.85470	0.85894	0.86353	0.86859
514	0.84116	0.84963	0.85358	0.85491	0.85915	0.86373	0.86879
515	0.84140	0.84986	0.85381	0.85514	0.85936	0.86394	0.86898
516	0.84164	0.85009	0.85403	0.85535	0.85957	0.86414	0.86918
517	0.84189	0.85031	0.85426	0.85557	0.85979	0.86435	0.86937

N	0.1%	0.5%	1%	1.25%	2.5%	5%	10%
518	0.84213	0.85054	0.85448	0.85579	0.86000	0.86455	0.86957
519	0.84235	0.85077	0.85470	0.85601	0.86021	0.86476	0.86976
520	0.84260	0.85099	0.85492	0.85622	0.86042	0.86496	0.86996
521	0.84286	0.85122	0.85514	0.85644	0.86063	0.86516	0.87015
522	0.84311	0.85145	0.85535	0.85666	0.86084	0.86536	0.87035
523	0.84336	0.85168	0.85557	0.85688	0.86105	0.86556	0.87054
524	0.84357	0.85190	0.85578	0.85709	0.86126	0.86576	0.87073
525	0.84381	0.85210	0.85601	0.85730	0.86147	0.86596	0.87092
526	0.84404	0.85233	0.85622	0.85751	0.86167	0.86615	0.87111
527	0.84428	0.85256	0.85643	0.85772	0.86188	0.86635	0.87130
528	0.84450	0.85278	0.85664	0.85793	0.86208	0.86655	0.87149
529	0.84473	0.85299	0.85685	0.85814	0.86228	0.86675	0.87168
530	0.84498	0.85321	0.85707	0.85834	0.86248	0.86694	0.87186
531	0.84520	0.85343	0.85727	0.85855	0.86268	0.86714	0.87205
532	0.84543	0.85365	0.85748	0.85876	0.86288	0.86733	0.87224
533	0.84566	0.85386	0.85770	0.85897	0.86309	0.86753	0.87242
534	0.84590	0.85409	0.85790	0.85917	0.86328	0.86772	0.87261
535	0.84612	0.85429	0.85811	0.85938	0.86349	0.86792	0.87279
536	0.84633	0.85451	0.85832	0.85959	0.86369	0.86811	0.87298
537	0.84656	0.85472	0.85852	0.85979	0.86388	0.86830	0.87316
538	0.84678	0.85494	0.85873	0.85999	0.86408	0.86849	0.87334
539	0.84699	0.85515	0.85894	0.86020	0.86428	0.86868	0.87353
540	0.84722	0.85536	0.85914	0.86040	0.86448	0.86887	0.87371
541	0.84745	0.85558	0.85934	0.86061	0.86468	0.86906	0.87389
542	0.84766	0.85579	0.85955	0.86081	0.86487	0.86925	0.87407
543	0.84787	0.85600	0.85975	0.86101	0.86507	0.86944	0.87425
544	0.84810	0.85621	0.85995	0.86121	0.86526	0.86962	0.87443
545	0.84831	0.85642	0.86015	0.86140	0.86546	0.86981	0.87461
546	0.84853	0.85662	0.86035	0.86160	0.86565	0.87000	0.87479
547	0.84874	0.85683	0.86055	0.86180	0.86584	0.87018	0.87497
548	0.84897	0.85703	0.86075	0.86200	0.86604	0.87037	0.87515
549	0.84920	0.85724	0.86095	0.86219	0.86623	0.87055	0.87532
550	0.84942	0.85745	0.86116	0.86239	0.86642	0.87073	0.87550
551	0.84963	0.85765	0.86136	0.86259	0.86660	0.87092	0.87567
552	0.84986	0.85786	0.86156	0.86279	0.86679	0.87110	0.87585
553	0.85011	0.85806	0.86176	0.86299	0.86699	0.87128	0.87602
554	0.85031	0.85826	0.86196	0.86318	0.86718	0.87146	0.87620
555	0.85053	0.85846	0.86215	0.86337	0.86737	0.87164	0.87637
556	0.85074	0.85866	0.86234	0.86356	0.86755	0.87183	0.87654
557	0.85097	0.85886	0.86254	0.86376	0.86774	0.87200	0.87671
558	0.85116	0.85906	0.86273	0.86395	0.86793	0.87218	0.87689
559	0.85138	0.85926	0.86293	0.86414	0.86811	0.87236	0.87706
560	0.85159	0.85947	0.86312	0.86434	0.86830	0.87254	0.87723
561	0.85179	0.85966	0.86331	0.86453	0.86848	0.87272	0.87740
562	0.85199	0.85986	0.86350	0.86472	0.86867	0.87290	0.87757
563	0.85219	0.86006	0.86370	0.86491	0.86885	0.87308	0.87774
564	0.85240	0.86026	0.86389	0.86509	0.86903	0.87325	0.87791
565	0.85260	0.86045	0.86408	0.86528	0.86921	0.87343	0.87807
566	0.85282	0.86065	0.86427	0.86548	0.86940	0.87360	0.87824
567	0.85303	0.86084	0.86446	0.86567	0.86957	0.87378	0.87841
568	0.85324	0.86104	0.86465	0.86585	0.86975	0.87395	0.87857
569	0.85344	0.86123	0.86484	0.86603	0.86993	0.87412	0.87874
570	0.85365	0.86142	0.86502	0.86621	0.87012	0.87429	0.87891
571	0.85385	0.86161	0.86521	0.86639	0.87030	0.87447	0.87907
572	0.85405	0.86180	0.86539	0.86658	0.87047	0.87464	0.87924
573	0.85423	0.86199	0.86558	0.86677	0.87065	0.87481	0.87940
574	0.85442	0.86218	0.86576	0.86695	0.87083	0.87498	0.87956
575	0.85463	0.86237	0.86594	0.86713	0.87100	0.87515	0.87973
576	0.85482	0.86257	0.86612	0.86731	0.87118	0.87532	0.87989
577	0.85503	0.86276	0.86631	0.86749	0.87135	0.87549	0.88005
578	0.85522	0.86295	0.86649	0.86767	0.87153	0.87566	0.88022
579	0.85541	0.86313	0.86668	0.86785	0.87170	0.87583	0.88038
580	0.85562	0.86332	0.86685	0.86803	0.87188	0.87599	0.88054
581	0.85583	0.86351	0.86704	0.86822	0.87205	0.87616	0.88070
582	0.85601	0.86369	0.86721	0.86839	0.87222	0.87633	0.88086
583	0.85622	0.86387	0.86739	0.86857	0.87239	0.87649	0.88102
584	0.85641	0.86406	0.86758	0.86875	0.87257	0.87666	0.88118
585	0.85660	0.86425	0.86775	0.86893	0.87274	0.87682	0.88133
586	0.85677	0.86442	0.86793	0.86911	0.87291	0.87699	0.88149
587	0.85697	0.86461	0.86810	0.86929	0.87308	0.87715	0.88165
588	0.85718	0.86479	0.86828	0.86946	0.87325	0.87731	0.88180
589	0.85737	0.86497	0.86847	0.86964	0.87342	0.87748	0.88196
590	0.85757	0.86515	0.86864	0.86982	0.87359	0.87764	0.88212
591	0.85775	0.86532	0.86882	0.86999	0.87376	0.87780	0.88227

N	0.1%	0.5%	1%	1.25%	2.5%	5%	10%
592	0.85794	0.86552	0.86900	0.87016	0.87393	0.87797	0.88243
593	0.85814	0.86569	0.86918	0.87034	0.87410	0.87813	0.88258
594	0.85834	0.86587	0.86935	0.87051	0.87426	0.87829	0.88274
595	0.85853	0.86606	0.86952	0.87069	0.87443	0.87845	0.88289
596	0.85872	0.86624	0.86970	0.87086	0.87459	0.87861	0.88305
597	0.85892	0.86641	0.86987	0.87103	0.87476	0.87877	0.88320
598	0.85910	0.86659	0.87005	0.87120	0.87492	0.87892	0.88335
599	0.85930	0.86676	0.87022	0.87137	0.87508	0.87908	0.88350
600	0.85948	0.86694	0.87039	0.87154	0.87525	0.87924	0.88365
601	0.85965	0.86711	0.87055	0.87170	0.87541	0.87940	0.88381
602	0.85985	0.86728	0.87073	0.87188	0.87558	0.87955	0.88396
603	0.86003	0.86746	0.87089	0.87204	0.87573	0.87971	0.88411
604	0.86022	0.86764	0.87107	0.87221	0.87590	0.87987	0.88426
605	0.86039	0.86781	0.87124	0.87238	0.87606	0.88002	0.88441
606	0.86057	0.86798	0.87140	0.87255	0.87622	0.88017	0.88456
607	0.86075	0.86816	0.87157	0.87271	0.87638	0.88033	0.88471
608	0.86093	0.86833	0.87174	0.87288	0.87654	0.88049	0.88485
609	0.86113	0.86850	0.87190	0.87304	0.87670	0.88064	0.88500
610	0.86129	0.86867	0.87207	0.87320	0.87686	0.88080	0.88515
611	0.86147	0.86884	0.87223	0.87336	0.87702	0.88095	0.88530
612	0.86166	0.86901	0.87240	0.87353	0.87717	0.88111	0.88545
613	0.86184	0.86918	0.87257	0.87369	0.87733	0.88126	0.88559
614	0.86203	0.86935	0.87273	0.87386	0.87749	0.88141	0.88574
615	0.86221	0.86953	0.87289	0.87402	0.87765	0.88156	0.88588
616	0.86239	0.86970	0.87305	0.87418	0.87780	0.88171	0.88603
617	0.86257	0.86986	0.87322	0.87434	0.87796	0.88187	0.88617
618	0.86275	0.87003	0.87338	0.87450	0.87812	0.88202	0.88632
619	0.86293	0.87019	0.87355	0.87467	0.87827	0.88217	0.88646
620	0.86309	0.87035	0.87371	0.87483	0.87843	0.88232	0.88661
621	0.86328	0.87052	0.87386	0.87499	0.87859	0.88247	0.88675
622	0.86344	0.87068	0.87402	0.87514	0.87874	0.88261	0.88689
623	0.86362	0.87084	0.87418	0.87530	0.87889	0.88276	0.88704
624	0.86380	0.87101	0.87434	0.87546	0.87905	0.88291	0.88718
625	0.86398	0.87118	0.87450	0.87562	0.87920	0.88305	0.88732
626	0.86417	0.87134	0.87466	0.87577	0.87935	0.88320	0.88746
627	0.86436	0.87150	0.87481	0.87593	0.87950	0.88335	0.88760
628	0.86451	0.87166	0.87497	0.87608	0.87965	0.88350	0.88774
629	0.86469	0.87183	0.87513	0.87624	0.87980	0.88365	0.88788
630	0.86486	0.87199	0.87529	0.87639	0.87995	0.88379	0.88802
631	0.86502	0.87215	0.87545	0.87655	0.88010	0.88394	0.88816
632	0.86519	0.87231	0.87561	0.87670	0.88026	0.88408	0.88830
633	0.86536	0.87246	0.87577	0.87686	0.88041	0.88423	0.88844
634	0.86555	0.87263	0.87592	0.87701	0.88056	0.88437	0.88858
635	0.86570	0.87278	0.87608	0.87717	0.88070	0.88451	0.88871
636	0.86588	0.87294	0.87624	0.87732	0.88085	0.88465	0.88885
637	0.86606	0.87311	0.87639	0.87747	0.88100	0.88480	0.88899
638	0.86623	0.87326	0.87654	0.87762	0.88115	0.88494	0.88912
639	0.86640	0.87343	0.87670	0.87778	0.88129	0.88508	0.88926
640	0.86656	0.87358	0.87684	0.87793	0.88144	0.88522	0.88939
641	0.86673	0.87374	0.87700	0.87808	0.88158	0.88536	0.88953
642	0.86688	0.87389	0.87715	0.87823	0.88173	0.88550	0.88966
643	0.86705	0.87405	0.87730	0.87838	0.88187	0.88565	0.88980
644	0.86722	0.87420	0.87745	0.87853	0.88202	0.88579	0.88993
645	0.86739	0.87435	0.87760	0.87868	0.88216	0.88592	0.89007
646	0.86754	0.87450	0.87775	0.87883	0.88230	0.88606	0.89020
647	0.86769	0.87466	0.87790	0.87898	0.88245	0.88620	0.89033
648	0.86784	0.87481	0.87805	0.87912	0.88259	0.88634	0.89047
649	0.86801	0.87496	0.87820	0.87927	0.88273	0.88648	0.89060
650	0.86818	0.87512	0.87835	0.87942	0.88288	0.88662	0.89073
651	0.86834	0.87528	0.87850	0.87957	0.88302	0.88676	0.89086
652	0.86849	0.87543	0.87865	0.87971	0.88316	0.88689	0.89099
653	0.86866	0.87558	0.87879	0.87986	0.88331	0.88703	0.89112
654	0.86882	0.87573	0.87894	0.88001	0.88345	0.88717	0.89125
655	0.86898	0.87589	0.87908	0.88015	0.88359	0.88730	0.89138
656	0.86914	0.87604	0.87923	0.88030	0.88373	0.88744	0.89152
657	0.86930	0.87620	0.87938	0.88044	0.88387	0.88757	0.89165
658	0.86946	0.87636	0.87953	0.88059	0.88401	0.88771	0.89178
659	0.86963	0.87650	0.87967	0.88073	0.88415	0.88784	0.89190
660	0.86978	0.87665	0.87981	0.88087	0.88429	0.88797	0.89203
661	0.86994	0.87679	0.87995	0.88101	0.88443	0.88811	0.89216
662	0.87010	0.87694	0.88010	0.88116	0.88457	0.88824	0.89229
663	0.87025	0.87708	0.88024	0.88130	0.88470	0.88838	0.89242
664	0.87041	0.87724	0.88038	0.88144	0.88484	0.88851	0.89255
665	0.87056	0.87739	0.88053	0.88159	0.88498	0.88864	0.89268

N	0.1%	0.5%	1%	1.25%	2.5%	5%	10%
666	0.87072	0.87753	0.88068	0.88173	0.88511	0.88877	0.89280
667	0.87088	0.87768	0.88082	0.88187	0.88525	0.88891	0.89293
668	0.87104	0.87782	0.88096	0.88201	0.88538	0.88904	0.89305
669	0.87119	0.87796	0.88110	0.88214	0.88552	0.88917	0.89318
670	0.87135	0.87811	0.88124	0.88228	0.88566	0.88930	0.89330
671	0.87150	0.87826	0.88138	0.88242	0.88579	0.88943	0.89343
672	0.87165	0.87840	0.88152	0.88256	0.88593	0.88956	0.89355
673	0.87179	0.87855	0.88166	0.88270	0.88606	0.88969	0.89368
674	0.87195	0.87869	0.88180	0.88284	0.88619	0.88982	0.89380
675	0.87210	0.87884	0.88194	0.88298	0.88633	0.88995	0.89393
676	0.87225	0.87898	0.88207	0.88312	0.88646	0.89007	0.89405
677	0.87240	0.87913	0.88221	0.88325	0.88660	0.89020	0.89417
678	0.87257	0.87927	0.88235	0.88339	0.88673	0.89033	0.89430
679	0.87273	0.87941	0.88249	0.88352	0.88686	0.89046	0.89442
680	0.87289	0.87955	0.88263	0.88366	0.88699	0.89059	0.89454
681	0.87303	0.87969	0.88277	0.88379	0.88712	0.89071	0.89466
682	0.87316	0.87983	0.88290	0.88393	0.88725	0.89084	0.89478
683	0.87331	0.87997	0.88304	0.88407	0.88739	0.89097	0.89491
684	0.87346	0.88012	0.88318	0.88420	0.88752	0.89110	0.89503
685	0.87361	0.88026	0.88332	0.88434	0.88765	0.89122	0.89515
686	0.87375	0.88039	0.88345	0.88447	0.88778	0.89135	0.89527
687	0.87391	0.88053	0.88359	0.88461	0.88791	0.89147	0.89539
688	0.87406	0.88067	0.88373	0.88474	0.88804	0.89160	0.89551
689	0.87421	0.88081	0.88386	0.88488	0.88817	0.89172	0.89563
690	0.87435	0.88095	0.88399	0.88501	0.88829	0.89184	0.89575
691	0.87449	0.88108	0.88413	0.88515	0.88842	0.89197	0.89586
692	0.87464	0.88122	0.88426	0.88527	0.88855	0.89209	0.89598
693	0.87479	0.88135	0.88439	0.88540	0.88868	0.89221	0.89610
694	0.87494	0.88149	0.88452	0.88553	0.88881	0.89234	0.89622
695	0.87507	0.88163	0.88466	0.88567	0.88893	0.89246	0.89634
696	0.87522	0.88177	0.88479	0.88580	0.88906	0.89258	0.89645
697	0.87537	0.88190	0.88492	0.88593	0.88918	0.89271	0.89657
698	0.87551	0.88203	0.88505	0.88606	0.88931	0.89283	0.89669
699	0.87565	0.88217	0.88519	0.88619	0.88944	0.89295	0.89680
700	0.87580	0.88231	0.88532	0.88632	0.88956	0.89307	0.89692
701	0.87593	0.88244	0.88545	0.88645	0.88969	0.89319	0.89703
702	0.87607	0.88258	0.88558	0.88658	0.88981	0.89331	0.89715
703	0.87622	0.88271	0.88571	0.88671	0.88994	0.89343	0.89727
704	0.87637	0.88285	0.88584	0.88684	0.89006	0.89355	0.89738
705	0.87650	0.88298	0.88597	0.88697	0.89018	0.89367	0.89750
706	0.87664	0.88312	0.88610	0.88709	0.89031	0.89379	0.89761
707	0.87677	0.88324	0.88623	0.88723	0.89043	0.89391	0.89772
708	0.87691	0.88338	0.88636	0.88735	0.89056	0.89402	0.89784
709	0.87706	0.88351	0.88648	0.88748	0.89068	0.89414	0.89795
710	0.87718	0.88365	0.88662	0.88761	0.89080	0.89426	0.89807
711	0.87733	0.88377	0.88674	0.88773	0.89092	0.89438	0.89818
712	0.87748	0.88390	0.88687	0.88786	0.89105	0.89450	0.89829
713	0.87763	0.88403	0.88699	0.88798	0.89117	0.89461	0.89841
714	0.87775	0.88415	0.88712	0.88811	0.89129	0.89473	0.89852
715	0.87789	0.88428	0.88725	0.88823	0.89141	0.89485	0.89863
716	0.87802	0.88442	0.88737	0.88836	0.89153	0.89496	0.89874
717	0.87816	0.88455	0.88750	0.88848	0.89165	0.89508	0.89885
718	0.87831	0.88467	0.88762	0.88860	0.89177	0.89519	0.89897
719	0.87845	0.88480	0.88775	0.88872	0.89189	0.89531	0.89908
720	0.87858	0.88493	0.88788	0.88885	0.89201	0.89543	0.89919
721	0.87871	0.88505	0.88800	0.88898	0.89213	0.89554	0.89930
722	0.87885	0.88518	0.88812	0.88910	0.89225	0.89566	0.89941
723	0.87898	0.88531	0.88824	0.88922	0.89237	0.89577	0.89952
724	0.87911	0.88544	0.88837	0.88934	0.89248	0.89588	0.89963
725	0.87924	0.88557	0.88849	0.88946	0.89260	0.89600	0.89974
726	0.87937	0.88570	0.88862	0.88958	0.89272	0.89611	0.89985
727	0.87951	0.88582	0.88874	0.88971	0.89283	0.89623	0.89996
728	0.87965	0.88595	0.88886	0.88983	0.89295	0.89634	0.90006
729	0.87977	0.88608	0.88899	0.88995	0.89307	0.89645	0.90017
730	0.87990	0.88620	0.88911	0.89007	0.89318	0.89657	0.90028
731	0.88003	0.88633	0.88923	0.89019	0.89331	0.89668	0.90039
732	0.88017	0.88645	0.88935	0.89031	0.89342	0.89679	0.90050
733	0.88031	0.88658	0.88948	0.89043	0.89354	0.89690	0.90060
734	0.88046	0.88670	0.88960	0.89055	0.89366	0.89701	0.90071
735	0.88058	0.88682	0.88972	0.89067	0.89377	0.89713	0.90082
736	0.88072	0.88695	0.88984	0.89079	0.89389	0.89724	0.90092
737	0.88085	0.88707	0.88996	0.89091	0.89400	0.89735	0.90103
738	0.88098	0.88720	0.89008	0.89103	0.89412	0.89746	0.90114
739	0.88112	0.88732	0.89020	0.89115	0.89424	0.89757	0.90124

N	0.1%	0.5%	1%	1.25%	2.5%	5%	10%
740	0.88125	0.88744	0.89032	0.89127	0.89435	0.89768	0.90135
741	0.88137	0.88756	0.89044	0.89139	0.89446	0.89779	0.90146
742	0.88149	0.88769	0.89055	0.89150	0.89458	0.89790	0.90156
743	0.88161	0.88780	0.89067	0.89162	0.89469	0.89801	0.90166
744	0.88174	0.88792	0.89078	0.89173	0.89480	0.89812	0.90177
745	0.88187	0.88804	0.89090	0.89185	0.89491	0.89822	0.90187
746	0.88200	0.88817	0.89102	0.89197	0.89503	0.89833	0.90197
747	0.88213	0.88829	0.89114	0.89208	0.89514	0.89844	0.90208
748	0.88226	0.88841	0.89126	0.89220	0.89525	0.89855	0.90218
749	0.88238	0.88853	0.89137	0.89232	0.89536	0.89866	0.90229
750	0.88252	0.88865	0.89149	0.89244	0.89548	0.89877	0.90239
751	0.88264	0.88877	0.89160	0.89255	0.89559	0.89887	0.90249
752	0.88278	0.88889	0.89172	0.89267	0.89570	0.89898	0.90259
753	0.88290	0.88900	0.89183	0.89278	0.89581	0.89909	0.90270
754	0.88302	0.88912	0.89195	0.89289	0.89592	0.89919	0.90280
755	0.88316	0.88925	0.89207	0.89301	0.89603	0.89930	0.90290
756	0.88329	0.88936	0.89218	0.89312	0.89614	0.89940	0.90300
757	0.88341	0.88948	0.89230	0.89324	0.89625	0.89951	0.90310
758	0.88353	0.88960	0.89241	0.89335	0.89636	0.89961	0.90320
759	0.88365	0.88971	0.89253	0.89346	0.89647	0.89972	0.90330
760	0.88378	0.88983	0.89264	0.89358	0.89657	0.89982	0.90341
761	0.88390	0.88995	0.89275	0.89369	0.89669	0.89993	0.90351
762	0.88403	0.89006	0.89286	0.89380	0.89680	0.90003	0.90361
763	0.88415	0.89018	0.89298	0.89391	0.89690	0.90014	0.90371
764	0.88426	0.89030	0.89309	0.89403	0.89701	0.90024	0.90381
765	0.88438	0.89041	0.89320	0.89414	0.89712	0.90034	0.90391
766	0.88451	0.89052	0.89331	0.89425	0.89722	0.90045	0.90401
767	0.88464	0.89064	0.89342	0.89436	0.89733	0.90055	0.90411
768	0.88476	0.89077	0.89353	0.89447	0.89744	0.90066	0.90421
769	0.88489	0.89088	0.89364	0.89458	0.89754	0.90076	0.90430
770	0.88500	0.89099	0.89375	0.89468	0.89765	0.90086	0.90440
771	0.88512	0.89110	0.89386	0.89479	0.89776	0.90096	0.90450
772	0.88524	0.89122	0.89397	0.89490	0.89786	0.90107	0.90460
773	0.88537	0.89133	0.89408	0.89501	0.89797	0.90117	0.90470
774	0.88549	0.89144	0.89419	0.89512	0.89807	0.90127	0.90480
775	0.88560	0.89155	0.89430	0.89522	0.89817	0.90137	0.90489
776	0.88571	0.89166	0.89441	0.89533	0.89828	0.90147	0.90499
777	0.88583	0.89178	0.89452	0.89544	0.89838	0.90157	0.90509
778	0.88595	0.89189	0.89463	0.89555	0.89849	0.90167	0.90518
779	0.88607	0.89200	0.89473	0.89565	0.89860	0.90177	0.90528
780	0.88619	0.89211	0.89484	0.89576	0.89870	0.90187	0.90537
781	0.88631	0.89223	0.89495	0.89587	0.89880	0.90197	0.90547
782	0.88644	0.89234	0.89506	0.89597	0.89890	0.90207	0.90557
783	0.88658	0.89245	0.89517	0.89608	0.89901	0.90217	0.90566
784	0.88669	0.89257	0.89528	0.89619	0.89911	0.90227	0.90576
785	0.88681	0.89268	0.89539	0.89629	0.89921	0.90237	0.90585
786	0.88693	0.89279	0.89549	0.89640	0.89932	0.90247	0.90595
787	0.88704	0.89290	0.89560	0.89651	0.89942	0.90256	0.90604
788	0.88715	0.89301	0.89571	0.89661	0.89952	0.90266	0.90614
789	0.88726	0.89311	0.89581	0.89672	0.89962	0.90276	0.90623
790	0.88738	0.89322	0.89592	0.89682	0.89973	0.90286	0.90633
791	0.88749	0.89333	0.89602	0.89693	0.89983	0.90296	0.90642
792	0.88761	0.89344	0.89613	0.89703	0.89993	0.90306	0.90651
793	0.88773	0.89355	0.89623	0.89713	0.90003	0.90315	0.90661
794	0.88786	0.89366	0.89633	0.89723	0.90013	0.90325	0.90670
795	0.88796	0.89376	0.89644	0.89734	0.90024	0.90335	0.90680
796	0.88808	0.89387	0.89654	0.89744	0.90034	0.90345	0.90689
797	0.88820	0.89398	0.89665	0.89755	0.90043	0.90354	0.90698
798	0.88831	0.89409	0.89676	0.89765	0.90053	0.90364	0.90707
799	0.88842	0.89419	0.89686	0.89775	0.90063	0.90374	0.90717
800	0.88852	0.89430	0.89696	0.89785	0.90073	0.90383	0.90726
801	0.88863	0.89441	0.89706	0.89795	0.90083	0.90393	0.90735
802	0.88876	0.89451	0.89717	0.89806	0.90093	0.90402	0.90744
803	0.88887	0.89461	0.89727	0.89816	0.90103	0.90412	0.90754
804	0.88898	0.89472	0.89738	0.89826	0.90113	0.90421	0.90763
805	0.88908	0.89483	0.89748	0.89836	0.90123	0.90431	0.90772
806	0.88920	0.89493	0.89758	0.89846	0.90133	0.90440	0.90781
807	0.88932	0.89504	0.89768	0.89856	0.90142	0.90450	0.90790
808	0.88942	0.89515	0.89778	0.89867	0.90152	0.90459	0.90799
809	0.88953	0.89524	0.89788	0.89877	0.90162	0.90469	0.90808
810	0.88965	0.89535	0.89798	0.89887	0.90172	0.90478	0.90817
811	0.88976	0.89546	0.89808	0.89897	0.90181	0.90487	0.90826
812	0.88986	0.89556	0.89818	0.89907	0.90191	0.90497	0.90835
813	0.88999	0.89567	0.89829	0.89917	0.90201	0.90506	0.90844

N	0.1%	0.5%	1%	1.25%	2.5%	5%	10%
814	0.89010	0.89577	0.89839	0.89927	0.90210	0.90515	0.90853
815	0.89020	0.89588	0.89849	0.89936	0.90220	0.90525	0.90862
816	0.89032	0.89598	0.89859	0.89946	0.90229	0.90534	0.90871
817	0.89042	0.89609	0.89869	0.89956	0.90239	0.90543	0.90880
818	0.89054	0.89619	0.89879	0.89966	0.90249	0.90553	0.90888
819	0.89065	0.89630	0.89889	0.89976	0.90258	0.90562	0.90897
820	0.89076	0.89640	0.89899	0.89985	0.90268	0.90571	0.90906
821	0.89088	0.89650	0.89909	0.89995	0.90277	0.90580	0.90915
822	0.89098	0.89660	0.89919	0.90005	0.90287	0.90589	0.90924
823	0.89109	0.89670	0.89929	0.90015	0.90296	0.90598	0.90933
824	0.89119	0.89680	0.89938	0.90025	0.90306	0.90608	0.90941
825	0.89129	0.89690	0.89949	0.90035	0.90315	0.90617	0.90950
826	0.89140	0.89700	0.89958	0.90045	0.90325	0.90626	0.90959
827	0.89150	0.89710	0.89968	0.90055	0.90334	0.90635	0.90967
828	0.89160	0.89721	0.89977	0.90064	0.90343	0.90644	0.90976
829	0.89172	0.89731	0.89987	0.90074	0.90352	0.90653	0.90985
830	0.89182	0.89741	0.89997	0.90083	0.90362	0.90662	0.90994
831	0.89193	0.89751	0.90006	0.90093	0.90371	0.90671	0.91002
832	0.89203	0.89761	0.90016	0.90102	0.90380	0.90680	0.91011
833	0.89213	0.89771	0.90026	0.90112	0.90390	0.90689	0.91019
834	0.89224	0.89781	0.90036	0.90121	0.90399	0.90698	0.91028
835	0.89235	0.89790	0.90046	0.90131	0.90408	0.90707	0.91036
836	0.89245	0.89799	0.90055	0.90141	0.90418	0.90716	0.91045
837	0.89255	0.89810	0.90065	0.90150	0.90427	0.90725	0.91053
838	0.89266	0.89820	0.90074	0.90160	0.90436	0.90733	0.91062
839	0.89276	0.89830	0.90084	0.90169	0.90445	0.90742	0.91070
840	0.89287	0.89839	0.90094	0.90179	0.90454	0.90751	0.91079
841	0.89298	0.89849	0.90103	0.90188	0.90463	0.90760	0.91087
842	0.89308	0.89859	0.90113	0.90197	0.90472	0.90768	0.91096
843	0.89318	0.89869	0.90122	0.90207	0.90481	0.90777	0.91104
844	0.89328	0.89879	0.90132	0.90217	0.90490	0.90786	0.91113
845	0.89338	0.89889	0.90141	0.90226	0.90499	0.90795	0.91121
846	0.89349	0.89898	0.90150	0.90235	0.90508	0.90803	0.91129
847	0.89359	0.89908	0.90160	0.90244	0.90517	0.90812	0.91138
848	0.89369	0.89918	0.90169	0.90254	0.90526	0.90821	0.91146
849	0.89379	0.89927	0.90179	0.90263	0.90535	0.90830	0.91154
850	0.89389	0.89936	0.90188	0.90272	0.90544	0.90838	0.91163
851	0.89399	0.89946	0.90197	0.90281	0.90553	0.90847	0.91171
852	0.89409	0.89956	0.90206	0.90290	0.90562	0.90855	0.91179
853	0.89419	0.89965	0.90216	0.90300	0.90571	0.90864	0.91188
854	0.89429	0.89975	0.90225	0.90308	0.90579	0.90873	0.91196
855	0.89439	0.89985	0.90234	0.90318	0.90588	0.90881	0.91204
856	0.89449	0.89994	0.90243	0.90327	0.90597	0.90890	0.91212
857	0.89460	0.90004	0.90253	0.90335	0.90606	0.90898	0.91220
858	0.89470	0.90013	0.90262	0.90344	0.90615	0.90906	0.91228
859	0.89480	0.90023	0.90271	0.90354	0.90624	0.90915	0.91237
860	0.89492	0.90032	0.90280	0.90363	0.90632	0.90923	0.91245
861	0.89501	0.90041	0.90289	0.90372	0.90641	0.90932	0.91253
862	0.89512	0.90050	0.90298	0.90381	0.90650	0.90940	0.91261
863	0.89521	0.90059	0.90307	0.90390	0.90659	0.90949	0.91269
864	0.89530	0.90069	0.90316	0.90399	0.90668	0.90957	0.91277
865	0.89541	0.90078	0.90325	0.90408	0.90676	0.90965	0.91285
866	0.89550	0.90088	0.90334	0.90417	0.90685	0.90974	0.91293
867	0.89559	0.90096	0.90343	0.90426	0.90693	0.90982	0.91301
868	0.89569	0.90106	0.90352	0.90434	0.90702	0.90991	0.91309
869	0.89579	0.90115	0.90361	0.90443	0.90711	0.90999	0.91317
870	0.89590	0.90125	0.90370	0.90452	0.90719	0.91007	0.91325
871	0.89599	0.90134	0.90379	0.90461	0.90728	0.91015	0.91333
872	0.89609	0.90143	0.90388	0.90470	0.90736	0.91024	0.91341
873	0.89618	0.90151	0.90397	0.90478	0.90745	0.91032	0.91349
874	0.89628	0.90161	0.90406	0.90487	0.90753	0.91040	0.91357
875	0.89638	0.90170	0.90415	0.90496	0.90762	0.91048	0.91365
876	0.89648	0.90179	0.90424	0.90505	0.90770	0.91057	0.91373
877	0.89658	0.90188	0.90433	0.90513	0.90779	0.91065	0.91380
878	0.89667	0.90197	0.90441	0.90522	0.90787	0.91073	0.91388
879	0.89677	0.90206	0.90450	0.90531	0.90796	0.91081	0.91396
880	0.89686	0.90215	0.90459	0.90540	0.90804	0.91089	0.91404
881	0.89696	0.90224	0.90467	0.90549	0.90813	0.91097	0.91412
882	0.89705	0.90233	0.90476	0.90557	0.90821	0.91106	0.91419
883	0.89714	0.90242	0.90485	0.90566	0.90829	0.91114	0.91427
884	0.89723	0.90251	0.90493	0.90574	0.90838	0.91122	0.91435
885	0.89732	0.90260	0.90502	0.90583	0.90846	0.91130	0.91443
886	0.89742	0.90269	0.90511	0.90592	0.90854	0.91138	0.91450
887	0.89752	0.90277	0.90520	0.90600	0.90863	0.91146	0.91458

N	0.1%	0.5%	1%	1.25%	2.5%	5%	10%
888	0.89762	0.90286	0.90528	0.90608	0.90871	0.91154	0.91466
889	0.89770	0.90295	0.90537	0.90617	0.90880	0.91162	0.91473
890	0.89780	0.90304	0.90545	0.90626	0.90888	0.91170	0.91481
891	0.89789	0.90313	0.90554	0.90634	0.90896	0.91178	0.91489
892	0.89798	0.90321	0.90563	0.90643	0.90904	0.91186	0.91496
893	0.89808	0.90330	0.90572	0.90652	0.90912	0.91194	0.91504
894	0.89817	0.90339	0.90580	0.90660	0.90920	0.91201	0.91512
895	0.89827	0.90347	0.90589	0.90668	0.90929	0.91209	0.91519
896	0.89835	0.90356	0.90597	0.90677	0.90937	0.91217	0.91527
897	0.89845	0.90365	0.90606	0.90685	0.90945	0.91225	0.91534
898	0.89854	0.90374	0.90614	0.90694	0.90953	0.91233	0.91542
899	0.89863	0.90382	0.90622	0.90702	0.90961	0.91241	0.91549
900	0.89872	0.90391	0.90631	0.90711	0.90970	0.91249	0.91557
901	0.89881	0.90399	0.90639	0.90719	0.90978	0.91256	0.91564
902	0.89890	0.90408	0.90647	0.90727	0.90986	0.91264	0.91572
903	0.89899	0.90417	0.90656	0.90735	0.90994	0.91272	0.91579
904	0.89908	0.90425	0.90664	0.90744	0.91002	0.91280	0.91587
905	0.89917	0.90434	0.90672	0.90752	0.91010	0.91288	0.91594
906	0.89926	0.90442	0.90681	0.90760	0.91018	0.91295	0.91602
907	0.89933	0.90451	0.90689	0.90768	0.91025	0.91303	0.91609
908	0.89943	0.90460	0.90698	0.90777	0.91034	0.91311	0.91616
909	0.89952	0.90468	0.90706	0.90785	0.91041	0.91318	0.91624
910	0.89961	0.90477	0.90714	0.90793	0.91050	0.91326	0.91631
911	0.89969	0.90485	0.90723	0.90801	0.91057	0.91334	0.91638
912	0.89979	0.90494	0.90731	0.90809	0.91066	0.91341	0.91646
913	0.89987	0.90502	0.90739	0.90818	0.91073	0.91349	0.91653
914	0.89996	0.90510	0.90747	0.90826	0.91081	0.91356	0.91660
915	0.90005	0.90519	0.90756	0.90834	0.91089	0.91364	0.91668
916	0.90016	0.90527	0.90764	0.90842	0.91097	0.91371	0.91675
917	0.90025	0.90535	0.90772	0.90850	0.91105	0.91379	0.91682
918	0.90033	0.90543	0.90780	0.90858	0.91113	0.91386	0.91689
919	0.90042	0.90552	0.90788	0.90867	0.91120	0.91394	0.91697
920	0.90051	0.90560	0.90796	0.90875	0.91128	0.91401	0.91704
921	0.90060	0.90569	0.90804	0.90883	0.91136	0.91409	0.91711
922	0.90069	0.90578	0.90812	0.90891	0.91144	0.91417	0.91718
923	0.90077	0.90586	0.90820	0.90899	0.91151	0.91424	0.91725
924	0.90086	0.90595	0.90828	0.90907	0.91159	0.91431	0.91733
925	0.90095	0.90603	0.90836	0.90915	0.91167	0.91439	0.91740
926	0.90105	0.90611	0.90844	0.90923	0.91175	0.91446	0.91747
927	0.90113	0.90620	0.90852	0.90931	0.91182	0.91454	0.91754
928	0.90121	0.90628	0.90860	0.90939	0.91190	0.91461	0.91761
929	0.90129	0.90636	0.90869	0.90946	0.91198	0.91469	0.91768
930	0.90138	0.90644	0.90877	0.90954	0.91205	0.91476	0.91775
931	0.90148	0.90652	0.90884	0.90962	0.91213	0.91483	0.91783
932	0.90157	0.90660	0.90892	0.90970	0.91220	0.91491	0.91790
933	0.90166	0.90669	0.90900	0.90978	0.91228	0.91498	0.91797
934	0.90175	0.90677	0.90908	0.90985	0.91236	0.91505	0.91804
935	0.90184	0.90685	0.90916	0.90993	0.91243	0.91512	0.91811
936	0.90193	0.90693	0.90924	0.91001	0.91251	0.91520	0.91818
937	0.90202	0.90701	0.90931	0.91009	0.91258	0.91527	0.91825
938	0.90210	0.90709	0.90939	0.91017	0.91266	0.91534	0.91832
939	0.90219	0.90717	0.90947	0.91025	0.91273	0.91542	0.91839
940	0.90228	0.90724	0.90955	0.91032	0.91281	0.91549	0.91846
941	0.90237	0.90732	0.90963	0.91040	0.91288	0.91556	0.91853
942	0.90245	0.90741	0.90971	0.91048	0.91296	0.91563	0.91860
943	0.90253	0.90749	0.90978	0.91055	0.91304	0.91571	0.91867
944	0.90260	0.90757	0.90986	0.91063	0.91311	0.91578	0.91874
945	0.90269	0.90764	0.90994	0.91071	0.91318	0.91585	0.91881
946	0.90277	0.90772	0.91002	0.91079	0.91326	0.91592	0.91887
947	0.90285	0.90780	0.91009	0.91086	0.91333	0.91600	0.91894
948	0.90294	0.90788	0.91017	0.91094	0.91341	0.91607	0.91901
949	0.90302	0.90796	0.91025	0.91102	0.91348	0.91614	0.91908
950	0.90311	0.90804	0.91033	0.91109	0.91355	0.91621	0.91915
951	0.90318	0.90812	0.91040	0.91116	0.91363	0.91628	0.91922
952	0.90327	0.90820	0.91048	0.91124	0.91370	0.91635	0.91928
953	0.90335	0.90828	0.91056	0.91132	0.91377	0.91642	0.91935
954	0.90343	0.90835	0.91063	0.91139	0.91385	0.91649	0.91942
955	0.90351	0.90843	0.91071	0.91147	0.91392	0.91656	0.91949
956	0.90359	0.90851	0.91079	0.91154	0.91400	0.91663	0.91955
957	0.90367	0.90859	0.91086	0.91162	0.91407	0.91670	0.91962
958	0.90375	0.90867	0.91094	0.91169	0.91414	0.91677	0.91969
959	0.90384	0.90875	0.91102	0.91177	0.91421	0.91684	0.91976
960	0.90393	0.90883	0.91109	0.91184	0.91429	0.91691	0.91982
961	0.90402	0.90891	0.91117	0.91192	0.91436	0.91698	0.91989

N	0.1%	0.5%	1%	1.25%	2.5%	5%	10%
962	0.90410	0.90898	0.91124	0.91199	0.91443	0.91705	0.91996
963	0.90418	0.90906	0.91132	0.91207	0.91450	0.91712	0.92002
964	0.90427	0.90914	0.91139	0.91214	0.91457	0.91719	0.92009
965	0.90435	0.90922	0.91147	0.91222	0.91464	0.91726	0.92015
966	0.90444	0.90929	0.91154	0.91229	0.91472	0.91733	0.92022
967	0.90451	0.90937	0.91161	0.91237	0.91479	0.91740	0.92029
968	0.90460	0.90944	0.91168	0.91244	0.91486	0.91746	0.92035
969	0.90467	0.90952	0.91176	0.91251	0.91493	0.91753	0.92042
970	0.90476	0.90960	0.91183	0.91259	0.91500	0.91760	0.92048
971	0.90484	0.90967	0.91191	0.91266	0.91507	0.91767	0.92055
972	0.90493	0.90974	0.91198	0.91273	0.91514	0.91774	0.92061
973	0.90501	0.90982	0.91206	0.91281	0.91521	0.91781	0.92068
974	0.90508	0.90989	0.91213	0.91288	0.91528	0.91787	0.92075
975	0.90516	0.90997	0.91220	0.91295	0.91535	0.91794	0.92081
976	0.90524	0.91004	0.91228	0.91302	0.91542	0.91801	0.92088
977	0.90532	0.91012	0.91235	0.91310	0.91549	0.91808	0.92094
978	0.90539	0.91019	0.91243	0.91317	0.91556	0.91815	0.92101
979	0.90547	0.91026	0.91250	0.91324	0.91563	0.91821	0.92107
980	0.90554	0.91034	0.91257	0.91331	0.91570	0.91828	0.92114
981	0.90561	0.91041	0.91264	0.91338	0.91577	0.91835	0.92120
982	0.90569	0.91049	0.91272	0.91346	0.91584	0.91841	0.92127
983	0.90578	0.91056	0.91279	0.91353	0.91591	0.91848	0.92133
984	0.90585	0.91063	0.91286	0.91360	0.91598	0.91855	0.92139
985	0.90593	0.91071	0.91293	0.91367	0.91605	0.91862	0.92146
986	0.90601	0.91078	0.91301	0.91374	0.91612	0.91868	0.92152
987	0.90609	0.91086	0.91308	0.91381	0.91619	0.91875	0.92159
988	0.90615	0.91094	0.91315	0.91388	0.91626	0.91882	0.92165
989	0.90623	0.91101	0.91322	0.91395	0.91633	0.91888	0.92171
990	0.90632	0.91108	0.91329	0.91403	0.91640	0.91895	0.92178
991	0.90639	0.91115	0.91336	0.91410	0.91647	0.91901	0.92184
992	0.90647	0.91123	0.91343	0.91417	0.91653	0.91908	0.92190
993	0.90655	0.91130	0.91350	0.91424	0.91660	0.91915	0.92197
994	0.90662	0.91137	0.91358	0.91431	0.91667	0.91921	0.92203
995	0.90670	0.91145	0.91365	0.91438	0.91674	0.91928	0.92209
996	0.90677	0.91152	0.91372	0.91445	0.91680	0.91934	0.92216
997	0.90686	0.91159	0.91379	0.91452	0.91688	0.91941	0.92222
998	0.90693	0.91167	0.91386	0.91459	0.91694	0.91947	0.92228
999	0.90701	0.91174	0.91393	0.91465	0.91701	0.91954	0.92235
1000	0.90709	0.91181	0.91400	0.91472	0.91708	0.91960	0.92241
