# Supplementary material for: A generalized outlier method with an automated test protocol applied to proficiency testing schemes: utilizing Monte Carlo simulation for generating critical values for the extended Grubbs statistic
Source: J AOAC Int. 2026 Apr 29;109(6):974–84. doi: 10.1093/jaoacint/qsag036 (PMC13732683; doi:10.1093/jaoacint/qsag036)
Supplement: Supplementary file 1 [file qsag036_supplementary_data.zip › aoac-25-0307-File021.pdf]

Extended Grubbs Statistics Tables for Multiple-9 Outliers

Table 1 – *GM9(9H0L or 0H9L)*. Critical values of upper significance levels for the Grubbs multiple-9 outlier test, applicable in situations where the nine highest or nine lowest observations are simultaneously identified as outliers, denoted as *GM9(9H0L)* and *GM9(0H9L)*, respectively.

N	0.1%	0.5%	1%	1.25%	2.5%	5%	10%
11	0.00000	0.00000	0.00000	0.00000	0.00001	0.00006	0.00025
12	0.00003	0.00016	0.00033	0.00042	0.00086	0.00182	0.00391
13	0.00042	0.00128	0.00209	0.00245	0.00407	0.00687	0.01190
14	0.00168	0.00395	0.00578	0.00656	0.00977	0.01477	0.02290
15	0.00408	0.00827	0.01134	0.01258	0.01751	0.02474	0.03576
16	0.00765	0.01407	0.01844	0.02017	0.02682	0.03616	0.04979
17	0.01237	0.02108	0.02680	0.02899	0.03728	0.04855	0.06444
18	0.01815	0.02916	0.03612	0.03878	0.04856	0.06153	0.07940
19	0.02468	0.03799	0.04615	0.04918	0.06036	0.07482	0.09442
20	0.03198	0.04741	0.05665	0.06009	0.07247	0.08831	0.10938
21	0.03994	0.05732	0.06754	0.07127	0.08477	0.10182	0.12411
22	0.04823	0.06759	0.07868	0.08274	0.09715	0.11524	0.13859
23	0.05693	0.07802	0.08989	0.09418	0.10949	0.12848	0.15272
24	0.06588	0.08852	0.10120	0.10570	0.12175	0.14149	0.16648
25	0.07513	0.09907	0.11236	0.11716	0.13389	0.15426	0.17987
26	0.08441	0.10968	0.12351	0.12849	0.14577	0.16673	0.19284
27	0.09370	0.12031	0.13457	0.13970	0.15752	0.17889	0.20548
28	0.10306	0.13071	0.14552	0.15077	0.16894	0.19076	0.21769
29	0.11214	0.14092	0.15616	0.16158	0.18019	0.20234	0.22954
30	0.12156	0.15106	0.16670	0.17218	0.19117	0.21360	0.24105
31	0.13102	0.16107	0.17702	0.18268	0.20196	0.22463	0.25220
32	0.14006	0.17102	0.18719	0.19292	0.21239	0.23529	0.26296
33	0.14910	0.18071	0.19714	0.20290	0.22262	0.24562	0.27341
34	0.15802	0.19030	0.20686	0.21269	0.23255	0.25578	0.28358
35	0.16708	0.19964	0.21646	0.22234	0.24226	0.26554	0.29339
36	0.17572	0.20888	0.22576	0.23167	0.25176	0.27508	0.30292
37	0.18426	0.21784	0.23490	0.24079	0.26102	0.28439	0.31215
38	0.19281	0.22673	0.24382	0.24977	0.27003	0.29339	0.32116
39	0.20130	0.23524	0.25253	0.25855	0.27878	0.30221	0.32988
40	0.20967	0.24365	0.26110	0.26706	0.28733	0.31073	0.33835
41	0.21785	0.25201	0.26943	0.27546	0.29573	0.31906	0.34661
42	0.22566	0.26008	0.27760	0.28369	0.30389	0.32716	0.35459
43	0.23360	0.26803	0.28548	0.29152	0.31184	0.33507	0.36234
44	0.24121	0.27574	0.29324	0.29934	0.31957	0.34279	0.36990
45	0.24861	0.28336	0.30091	0.30696	0.32723	0.35025	0.37730
46	0.25604	0.29086	0.30835	0.31436	0.33456	0.35757	0.38445
47	0.26301	0.29816	0.31563	0.32171	0.34181	0.36470	0.39143
48	0.27011	0.30524	0.32274	0.32877	0.34887	0.37163	0.39820
49	0.27704	0.31221	0.32970	0.33567	0.35568	0.37842	0.40483
50	0.28395	0.31903	0.33643	0.34247	0.36239	0.38501	0.41127
51	0.29075	0.32575	0.34313	0.34903	0.36896	0.39148	0.41756
52	0.29739	0.33234	0.34950	0.35549	0.37539	0.39780	0.42371
53	0.30387	0.33877	0.35595	0.36189	0.38164	0.40392	0.42968
54	0.31024	0.34493	0.36214	0.36809	0.38777	0.40997	0.43553
55	0.31630	0.35113	0.36828	0.37411	0.39376	0.41585	0.44123
56	0.32252	0.35714	0.37420	0.38014	0.39965	0.42157	0.44684
57	0.32850	0.36309	0.38012	0.38593	0.40540	0.42717	0.45227
58	0.33433	0.36885	0.38589	0.39170	0.41101	0.43265	0.45758
59	0.34004	0.37458	0.39146	0.39728	0.41653	0.43805	0.46280
60	0.34565	0.38017	0.39702	0.40277	0.42190	0.44333	0.46791
61	0.35130	0.38575	0.40242	0.40819	0.42719	0.44850	0.47292
62	0.35679	0.39098	0.40768	0.41344	0.43241	0.45352	0.47779
63	0.36205	0.39628	0.41295	0.41864	0.43742	0.45846	0.48255
64	0.36728	0.40140	0.41805	0.42366	0.44234	0.46327	0.48721
65	0.37245	0.40650	0.42296	0.42858	0.44720	0.46800	0.49175
66	0.37773	0.41144	0.42790	0.43348	0.45194	0.47263	0.49623
67	0.38276	0.41633	0.43271	0.43825	0.45664	0.47715	0.50062
68	0.38780	0.42111	0.43743	0.44296	0.46122	0.48160	0.50492
69	0.39245	0.42584	0.44206	0.44758	0.46574	0.48600	0.50912
70	0.39732	0.43047	0.44665	0.45210	0.47020	0.49026	0.51325
71	0.40184	0.43502	0.45108	0.45658	0.47452	0.49448	0.51729
72	0.40637	0.43949	0.45547	0.46092	0.47875	0.49861	0.52126
73	0.41098	0.44392	0.45983	0.46515	0.48295	0.50268	0.52513
74	0.41548	0.44821	0.46407	0.46936	0.48705	0.50662	0.52896
75	0.41983	0.45245	0.46819	0.47350	0.49108	0.51054	0.53270
76	0.42408	0.45663	0.47226	0.47755	0.49501	0.51434	0.53639

N	0.1%	0.5%	1%	1.25%	2.5%	5%	10%
77	0.42832	0.46073	0.47630	0.48154	0.49889	0.51810	0.53998
78	0.43262	0.46476	0.48024	0.48548	0.50269	0.52179	0.54353
79	0.43671	0.46871	0.48414	0.48935	0.50646	0.52541	0.54704
80	0.44072	0.47257	0.48799	0.49314	0.51012	0.52898	0.55046
81	0.44457	0.47637	0.49169	0.49683	0.51370	0.53247	0.55381
82	0.44854	0.48013	0.49535	0.50049	0.51726	0.53593	0.55710
83	0.45238	0.48388	0.49901	0.50408	0.52080	0.53932	0.56035
84	0.45614	0.48758	0.50257	0.50761	0.52424	0.54266	0.56355
85	0.46000	0.49115	0.50610	0.51108	0.52759	0.54594	0.56670
86	0.46373	0.49462	0.50953	0.51450	0.53091	0.54919	0.56977
87	0.46732	0.49812	0.51295	0.51791	0.53423	0.55232	0.57281
88	0.47085	0.50150	0.51626	0.52125	0.53746	0.55545	0.57579
89	0.47441	0.50491	0.51959	0.52449	0.54062	0.55849	0.57872
90	0.47796	0.50824	0.52275	0.52767	0.54375	0.56150	0.58160
91	0.48133	0.51152	0.52595	0.53086	0.54686	0.56448	0.58446
92	0.48474	0.51472	0.52911	0.53397	0.54984	0.56740	0.58725
93	0.48816	0.51791	0.53227	0.53708	0.55285	0.57028	0.59000
94	0.49132	0.52106	0.53534	0.54011	0.55579	0.57310	0.59269
95	0.49440	0.52417	0.53829	0.54308	0.55870	0.57589	0.59536
96	0.49756	0.52720	0.54125	0.54599	0.56155	0.57864	0.59799
97	0.50072	0.53028	0.54415	0.54888	0.56435	0.58136	0.60058
98	0.50372	0.53317	0.54702	0.55170	0.56712	0.58404	0.60314
99	0.50691	0.53604	0.54988	0.55455	0.56985	0.58668	0.60563
100	0.50987	0.53893	0.55268	0.55736	0.57253	0.58928	0.60812
101	0.51272	0.54182	0.55549	0.56009	0.57519	0.59183	0.61056
102	0.51589	0.54466	0.55824	0.56278	0.57783	0.59435	0.61297
103	0.51868	0.54738	0.56092	0.56543	0.58039	0.59682	0.61534
104	0.52167	0.55002	0.56355	0.56804	0.58294	0.59926	0.61767
105	0.52452	0.55277	0.56612	0.57062	0.58545	0.60170	0.61998
106	0.52730	0.55546	0.56868	0.57316	0.58797	0.60409	0.62226
107	0.53019	0.55804	0.57126	0.57572	0.59038	0.60644	0.62450
108	0.53280	0.56065	0.57380	0.57826	0.59279	0.60875	0.62671
109	0.53545	0.56316	0.57632	0.58070	0.59515	0.61103	0.62889
110	0.53802	0.56567	0.57873	0.58308	0.59748	0.61329	0.63104
111	0.54063	0.56816	0.58111	0.58549	0.59980	0.61552	0.63316
112	0.54318	0.57059	0.58348	0.58780	0.60206	0.61771	0.63526
113	0.54581	0.57302	0.58579	0.59015	0.60434	0.61988	0.63732
114	0.54827	0.57540	0.58808	0.59243	0.60654	0.62202	0.63937
115	0.55072	0.57775	0.59040	0.59469	0.60875	0.62413	0.64138
116	0.55321	0.58005	0.59264	0.59693	0.61089	0.62622	0.64337
117	0.55569	0.58232	0.59489	0.59916	0.61305	0.62826	0.64533
118	0.55809	0.58455	0.59711	0.60131	0.61514	0.63027	0.64727
119	0.56042	0.58677	0.59928	0.60347	0.61723	0.63227	0.64919
120	0.56292	0.58895	0.60142	0.60562	0.61927	0.63425	0.65108
121	0.56508	0.59116	0.60356	0.60773	0.62133	0.63623	0.65294
122	0.56726	0.59338	0.60567	0.60979	0.62333	0.63814	0.65478
123	0.56958	0.59554	0.60776	0.61184	0.62530	0.64004	0.65661
124	0.57178	0.59764	0.60977	0.61386	0.62726	0.64192	0.65840
125	0.57397	0.59971	0.61180	0.61589	0.62920	0.64381	0.66019
126	0.57620	0.60179	0.61379	0.61786	0.63113	0.64566	0.66195
127	0.57835	0.60379	0.61578	0.61980	0.63303	0.64749	0.66368
128	0.58046	0.60583	0.61773	0.62176	0.63490	0.64927	0.66538
129	0.58257	0.60780	0.61966	0.62367	0.63674	0.65105	0.66708
130	0.58461	0.60981	0.62158	0.62555	0.63859	0.65280	0.66875
131	0.58668	0.61175	0.62348	0.62744	0.64040	0.65456	0.67040
132	0.58884	0.61363	0.62536	0.62930	0.64217	0.65628	0.67203
133	0.59081	0.61551	0.62724	0.63116	0.64394	0.65797	0.67366
134	0.59285	0.61739	0.62904	0.63295	0.64570	0.65966	0.67527
135	0.59473	0.61927	0.63083	0.63471	0.64743	0.66130	0.67685
136	0.59664	0.62115	0.63262	0.63647	0.64915	0.66295	0.67841
137	0.59870	0.62300	0.63438	0.63820	0.65083	0.66456	0.67995
138	0.60062	0.62478	0.63610	0.63997	0.65250	0.66618	0.68148
139	0.60257	0.62654	0.63786	0.64169	0.65416	0.66778	0.68298
140	0.60453	0.62830	0.63958	0.64341	0.65581	0.66934	0.68448
141	0.60624	0.63004	0.64127	0.64507	0.65742	0.67089	0.68596
142	0.60812	0.63176	0.64298	0.64675	0.65902	0.67243	0.68742
143	0.61001	0.63352	0.64465	0.64840	0.66060	0.67396	0.68888
144	0.61168	0.63519	0.64630	0.65005	0.66216	0.67547	0.69031
145	0.61352	0.63685	0.64794	0.65164	0.66370	0.67696	0.69173
146	0.61529	0.63853	0.64956	0.65322	0.66524	0.67843	0.69314
147	0.61710	0.64017	0.65115	0.65479	0.66677	0.67988	0.69452
148	0.61876	0.64178	0.65269	0.65633	0.66825	0.68132	0.69588
149	0.62045	0.64337	0.65426	0.65788	0.66977	0.68275	0.69725
150	0.62217	0.64498	0.65579	0.65940	0.67124	0.68416	0.69860

N	0.1%	0.5%	1%	1.25%	2.5%	5%	10%
151	0.62380	0.64656	0.65731	0.66091	0.67268	0.68555	0.69993
152	0.62558	0.64813	0.65883	0.66244	0.67414	0.68695	0.70123
153	0.62724	0.64965	0.66034	0.66394	0.67558	0.68832	0.70255
154	0.62885	0.65118	0.66182	0.66537	0.67698	0.68969	0.70385
155	0.63043	0.65267	0.66331	0.66682	0.67840	0.69104	0.70511
156	0.63212	0.65423	0.66475	0.66829	0.67979	0.69237	0.70638
157	0.63364	0.65567	0.66620	0.66971	0.68115	0.69368	0.70764
158	0.63517	0.65713	0.66761	0.67112	0.68251	0.69498	0.70889
159	0.63681	0.65858	0.66903	0.67252	0.68388	0.69627	0.71012
160	0.63835	0.66005	0.67044	0.67391	0.68519	0.69755	0.71133
161	0.63981	0.66149	0.67180	0.67530	0.68652	0.69883	0.71253
162	0.64132	0.66288	0.67318	0.67663	0.68784	0.70008	0.71373
163	0.64278	0.66430	0.67452	0.67797	0.68913	0.70133	0.71491
164	0.64419	0.66568	0.67587	0.67928	0.69042	0.70256	0.71608
165	0.64564	0.66706	0.67720	0.68061	0.69169	0.70380	0.71725
166	0.64715	0.66845	0.67853	0.68192	0.69296	0.70500	0.71840
167	0.64858	0.66978	0.67985	0.68322	0.69420	0.70619	0.71955
168	0.65002	0.67111	0.68115	0.68450	0.69543	0.70738	0.72068
169	0.65151	0.67246	0.68242	0.68579	0.69665	0.70856	0.72182
170	0.65292	0.67380	0.68372	0.68707	0.69789	0.70973	0.72292
171	0.65425	0.67511	0.68496	0.68830	0.69908	0.71087	0.72402
172	0.65566	0.67639	0.68623	0.68952	0.70029	0.71202	0.72511
173	0.65705	0.67770	0.68745	0.69074	0.70148	0.71317	0.72618
174	0.65843	0.67897	0.68869	0.69197	0.70266	0.71428	0.72725
175	0.65977	0.68021	0.68989	0.69316	0.70381	0.71540	0.72830
176	0.66112	0.68145	0.69111	0.69438	0.70496	0.71650	0.72936
177	0.66240	0.68269	0.69228	0.69553	0.70608	0.71759	0.73040
178	0.66368	0.68392	0.69348	0.69670	0.70723	0.71867	0.73143
179	0.66503	0.68514	0.69464	0.69787	0.70835	0.71974	0.73245
180	0.66623	0.68635	0.69581	0.69901	0.70947	0.72082	0.73346
181	0.66753	0.68757	0.69698	0.70017	0.71059	0.72187	0.73446
182	0.66889	0.68877	0.69814	0.70129	0.71169	0.72292	0.73546
183	0.67015	0.68994	0.69928	0.70245	0.71277	0.72395	0.73645
184	0.67124	0.69110	0.70038	0.70356	0.71383	0.72500	0.73742
185	0.67241	0.69224	0.70150	0.70463	0.71491	0.72602	0.73838
186	0.67362	0.69340	0.70263	0.70573	0.71596	0.72704	0.73936
187	0.67480	0.69452	0.70372	0.70683	0.71701	0.72804	0.74032
188	0.67597	0.69565	0.70481	0.70791	0.71805	0.72904	0.74126
189	0.67717	0.69676	0.70588	0.70898	0.71909	0.73003	0.74220
190	0.67835	0.69787	0.70695	0.71003	0.72010	0.73100	0.74312
191	0.67952	0.69896	0.70802	0.71109	0.72112	0.73198	0.74404
192	0.68074	0.70003	0.70909	0.71213	0.72211	0.73292	0.74497
193	0.68187	0.70111	0.71014	0.71318	0.72310	0.73388	0.74587
194	0.68298	0.70216	0.71116	0.71420	0.72409	0.73483	0.74677
195	0.68410	0.70324	0.71221	0.71521	0.72507	0.73577	0.74767
196	0.68526	0.70431	0.71324	0.71624	0.72604	0.73670	0.74856
197	0.68638	0.70537	0.71427	0.71721	0.72699	0.73762	0.74943
198	0.68743	0.70640	0.71528	0.71822	0.72797	0.73854	0.75031
199	0.68848	0.70742	0.71628	0.71920	0.72892	0.73945	0.75117
200	0.68954	0.70845	0.71726	0.72019	0.72987	0.74035	0.75203
201	0.69058	0.70946	0.71826	0.72119	0.73079	0.74126	0.75288
202	0.69166	0.71044	0.71922	0.72216	0.73173	0.74215	0.75372
203	0.69270	0.71141	0.72021	0.72311	0.73265	0.74303	0.75456
204	0.69382	0.71243	0.72116	0.72404	0.73356	0.74390	0.75539
205	0.69480	0.71342	0.72209	0.72498	0.73445	0.74476	0.75622
206	0.69592	0.71436	0.72302	0.72592	0.73534	0.74562	0.75704
207	0.69698	0.71533	0.72396	0.72684	0.73624	0.74649	0.75785
208	0.69801	0.71630	0.72487	0.72775	0.73712	0.74734	0.75866
209	0.69903	0.71726	0.72581	0.72867	0.73800	0.74820	0.75946
210	0.70007	0.71819	0.72671	0.72956	0.73887	0.74902	0.76026
211	0.70106	0.71915	0.72764	0.73048	0.73974	0.74986	0.76105
212	0.70206	0.72010	0.72853	0.73139	0.74059	0.75068	0.76183
213	0.70312	0.72100	0.72943	0.73227	0.74143	0.75149	0.76261
214	0.70407	0.72192	0.73033	0.73314	0.74229	0.75230	0.76337
215	0.70503	0.72283	0.73123	0.73402	0.74314	0.75311	0.76414
216	0.70596	0.72373	0.73209	0.73488	0.74398	0.75391	0.76490
217	0.70694	0.72462	0.73297	0.73575	0.74482	0.75470	0.76566
218	0.70786	0.72552	0.73383	0.73661	0.74563	0.75549	0.76641
219	0.70885	0.72640	0.73469	0.73743	0.74646	0.75628	0.76715
220	0.70977	0.72728	0.73553	0.73828	0.74727	0.75705	0.76789
221	0.71066	0.72814	0.73637	0.73911	0.74807	0.75782	0.76862
222	0.71161	0.72901	0.73721	0.73994	0.74887	0.75858	0.76935
223	0.71254	0.72985	0.73804	0.74077	0.74966	0.75934	0.77007
224	0.71345	0.73071	0.73885	0.74157	0.75046	0.76010	0.77077

N	0.1%	0.5%	1%	1.25%	2.5%	5%	10%
225	0.71428	0.73157	0.73967	0.74238	0.75123	0.76085	0.77150
226	0.71515	0.73241	0.74047	0.74319	0.75203	0.76159	0.77220
227	0.71605	0.73323	0.74128	0.74398	0.75279	0.76232	0.77290
228	0.71692	0.73407	0.74209	0.74478	0.75357	0.76306	0.77360
229	0.71781	0.73490	0.74289	0.74557	0.75433	0.76379	0.77430
230	0.71864	0.73570	0.74370	0.74636	0.75509	0.76451	0.77498
231	0.71954	0.73654	0.74448	0.74714	0.75583	0.76523	0.77566
232	0.72034	0.73733	0.74525	0.74791	0.75658	0.76594	0.77635
233	0.72119	0.73814	0.74605	0.74869	0.75732	0.76665	0.77702
234	0.72205	0.73895	0.74681	0.74946	0.75805	0.76735	0.77769
235	0.72291	0.73974	0.74758	0.75020	0.75876	0.76805	0.77835
236	0.72374	0.74053	0.74835	0.75096	0.75948	0.76874	0.77900
237	0.72455	0.74129	0.74911	0.75172	0.76020	0.76943	0.77967
238	0.72543	0.74210	0.74986	0.75246	0.76092	0.77012	0.78031
239	0.72624	0.74285	0.75061	0.75322	0.76163	0.77080	0.78096
240	0.72705	0.74360	0.75134	0.75395	0.76233	0.77148	0.78161
241	0.72789	0.74437	0.75209	0.75468	0.76303	0.77214	0.78225
242	0.72871	0.74511	0.75282	0.75540	0.76374	0.77281	0.78288
243	0.72946	0.74588	0.75355	0.75611	0.76443	0.77348	0.78350
244	0.73031	0.74662	0.75427	0.75682	0.76512	0.77412	0.78413
245	0.73107	0.74735	0.75499	0.75753	0.76579	0.77479	0.78475
246	0.73186	0.74811	0.75569	0.75822	0.76646	0.77544	0.78537
247	0.73264	0.74883	0.75640	0.75890	0.76714	0.77607	0.78599
248	0.73345	0.74957	0.75711	0.75961	0.76782	0.77672	0.78659
249	0.73425	0.75027	0.75781	0.76032	0.76847	0.77736	0.78720
250	0.73498	0.75096	0.75851	0.76100	0.76912	0.77799	0.78780
251	0.73572	0.75166	0.75918	0.76167	0.76978	0.77862	0.78840
252	0.73649	0.75238	0.75987	0.76235	0.77045	0.77924	0.78899
253	0.73721	0.75308	0.76054	0.76303	0.77109	0.77986	0.78959
254	0.73798	0.75377	0.76122	0.76371	0.77174	0.78049	0.79018
255	0.73879	0.75448	0.76189	0.76435	0.77238	0.78110	0.79076
256	0.73953	0.75519	0.76256	0.76503	0.77302	0.78171	0.79133
257	0.74027	0.75588	0.76322	0.76568	0.77365	0.78231	0.79191
258	0.74101	0.75653	0.76387	0.76633	0.77428	0.78292	0.79248
259	0.74171	0.75722	0.76452	0.76697	0.77490	0.78351	0.79305
260	0.74239	0.75788	0.76516	0.76763	0.77552	0.78411	0.79361
261	0.74316	0.75854	0.76581	0.76827	0.77614	0.78470	0.79417
262	0.74381	0.75921	0.76645	0.76890	0.77675	0.78528	0.79473
263	0.74452	0.75988	0.76706	0.76952	0.77736	0.78586	0.79528
264	0.74523	0.76054	0.76771	0.77014	0.77797	0.78643	0.79583
265	0.74591	0.76118	0.76835	0.77075	0.77856	0.78701	0.79638
266	0.74664	0.76184	0.76898	0.77138	0.77917	0.78758	0.79693
267	0.74728	0.76248	0.76959	0.77199	0.77975	0.78816	0.79747
268	0.74804	0.76313	0.77023	0.77261	0.78034	0.78872	0.79800
269	0.74867	0.76377	0.77085	0.77321	0.78093	0.78928	0.79854
270	0.74933	0.76442	0.77146	0.77381	0.78152	0.78984	0.79907
271	0.75000	0.76506	0.77207	0.77442	0.78210	0.79041	0.79959
272	0.75066	0.76569	0.77267	0.77501	0.78269	0.79096	0.80012
273	0.75133	0.76631	0.77328	0.77560	0.78325	0.79151	0.80064
274	0.75203	0.76692	0.77386	0.77619	0.78382	0.79205	0.80115
275	0.75265	0.76754	0.77446	0.77679	0.78437	0.79259	0.80167
276	0.75329	0.76815	0.77505	0.77736	0.78493	0.79313	0.80219
277	0.75393	0.76877	0.77563	0.77795	0.78550	0.79366	0.80270
278	0.75462	0.76939	0.77621	0.77853	0.78605	0.79419	0.80320
279	0.75525	0.76999	0.77679	0.77910	0.78660	0.79472	0.80371
280	0.75593	0.77058	0.77738	0.77967	0.78714	0.79525	0.80421
281	0.75657	0.77118	0.77796	0.78023	0.78769	0.79578	0.80471
282	0.75718	0.77177	0.77853	0.78080	0.78823	0.79630	0.80520
283	0.75779	0.77238	0.77911	0.78137	0.78876	0.79682	0.80570
284	0.75843	0.77298	0.77967	0.78194	0.78931	0.79734	0.80618
285	0.75905	0.77355	0.78023	0.78247	0.78984	0.79785	0.80667
286	0.75967	0.77413	0.78078	0.78305	0.79037	0.79836	0.80715
287	0.76030	0.77470	0.78134	0.78359	0.79090	0.79886	0.80763
288	0.76088	0.77526	0.78190	0.78415	0.79143	0.79936	0.80811
289	0.76152	0.77581	0.78245	0.78468	0.79196	0.79986	0.80859
290	0.76209	0.77638	0.78300	0.78522	0.79248	0.80036	0.80906
291	0.76270	0.77694	0.78354	0.78576	0.79299	0.80085	0.80953
292	0.76330	0.77751	0.78408	0.78629	0.79351	0.80133	0.81000
293	0.76390	0.77807	0.78461	0.78682	0.79403	0.80182	0.81046
294	0.76444	0.77862	0.78514	0.78734	0.79454	0.80230	0.81092
295	0.76505	0.77917	0.78567	0.78788	0.79505	0.80279	0.81138
296	0.76565	0.77970	0.78621	0.78840	0.79556	0.80327	0.81184
297	0.76626	0.78024	0.78673	0.78891	0.79605	0.80375	0.81230
298	0.76683	0.78078	0.78724	0.78943	0.79654	0.80423	0.81275

N	0.1%	0.5%	1%	1.25%	2.5%	5%	10%
299	0.76746	0.78131	0.78776	0.78994	0.79703	0.80470	0.81319
300	0.76799	0.78184	0.78827	0.79046	0.79753	0.80518	0.81364
301	0.76858	0.78237	0.78878	0.79096	0.79801	0.80565	0.81409
302	0.76913	0.78291	0.78929	0.79146	0.79849	0.80611	0.81453
303	0.76966	0.78343	0.78980	0.79195	0.79898	0.80657	0.81496
304	0.77024	0.78394	0.79030	0.79246	0.79945	0.80703	0.81540
305	0.77078	0.78446	0.79081	0.79295	0.79994	0.80749	0.81584
306	0.77129	0.78499	0.79131	0.79345	0.80042	0.80794	0.81628
307	0.77189	0.78550	0.79181	0.79394	0.80088	0.80839	0.81670
308	0.77243	0.78599	0.79230	0.79443	0.80135	0.80884	0.81714
309	0.77301	0.78649	0.79279	0.79491	0.80182	0.80929	0.81757
310	0.77354	0.78701	0.79328	0.79540	0.80229	0.80974	0.81799
311	0.77411	0.78752	0.79378	0.79588	0.80275	0.81019	0.81841
312	0.77459	0.78802	0.79425	0.79636	0.80322	0.81062	0.81883
313	0.77513	0.78852	0.79473	0.79684	0.80368	0.81106	0.81925
314	0.77565	0.78902	0.79521	0.79730	0.80414	0.81150	0.81966
315	0.77616	0.78953	0.79569	0.79779	0.80459	0.81193	0.82008
316	0.77669	0.79001	0.79616	0.79824	0.80504	0.81237	0.82049
317	0.77723	0.79049	0.79663	0.79870	0.80549	0.81279	0.82090
318	0.77775	0.79096	0.79710	0.79917	0.80593	0.81322	0.82131
319	0.77822	0.79144	0.79758	0.79963	0.80638	0.81365	0.82171
320	0.77875	0.79193	0.79803	0.80008	0.80682	0.81407	0.82212
321	0.77930	0.79240	0.79848	0.80053	0.80726	0.81450	0.82252
322	0.77978	0.79287	0.79895	0.80100	0.80770	0.81492	0.82292
323	0.78028	0.79334	0.79941	0.80145	0.80814	0.81534	0.82332
324	0.78077	0.79380	0.79988	0.80191	0.80857	0.81575	0.82371
325	0.78130	0.79427	0.80032	0.80236	0.80901	0.81616	0.82410
326	0.78180	0.79473	0.80076	0.80281	0.80944	0.81657	0.82449
327	0.78224	0.79519	0.80121	0.80325	0.80987	0.81698	0.82488
328	0.78274	0.79566	0.80167	0.80369	0.81029	0.81739	0.82526
329	0.78325	0.79613	0.80212	0.80413	0.81071	0.81780	0.82565
330	0.78376	0.79659	0.80254	0.80456	0.81113	0.81819	0.82603
331	0.78423	0.79702	0.80299	0.80500	0.81155	0.81859	0.82641
332	0.78476	0.79748	0.80342	0.80543	0.81196	0.81899	0.82679
333	0.78524	0.79793	0.80386	0.80586	0.81237	0.81939	0.82717
334	0.78572	0.79838	0.80429	0.80628	0.81279	0.81978	0.82754
335	0.78621	0.79883	0.80472	0.80671	0.81319	0.82018	0.82792
336	0.78668	0.79927	0.80516	0.80713	0.81359	0.82057	0.82829
337	0.78715	0.79973	0.80558	0.80755	0.81400	0.82096	0.82866
338	0.78756	0.80014	0.80600	0.80798	0.81440	0.82135	0.82903
339	0.78802	0.80058	0.80642	0.80840	0.81481	0.82173	0.82940
340	0.78849	0.80101	0.80685	0.80880	0.81521	0.82211	0.82976
341	0.78894	0.80144	0.80727	0.80922	0.81561	0.82250	0.83012
342	0.78941	0.80186	0.80769	0.80963	0.81600	0.82287	0.83048
343	0.78987	0.80229	0.80809	0.81005	0.81639	0.82325	0.83084
344	0.79030	0.80271	0.80850	0.81046	0.81679	0.82363	0.83120
345	0.79074	0.80314	0.80893	0.81086	0.81719	0.82400	0.83155
346	0.79120	0.80357	0.80934	0.81128	0.81757	0.82437	0.83190
347	0.79165	0.80400	0.80975	0.81169	0.81796	0.82474	0.83226
348	0.79211	0.80443	0.81016	0.81208	0.81835	0.82511	0.83261
349	0.79256	0.80485	0.81056	0.81248	0.81872	0.82548	0.83296
350	0.79301	0.80526	0.81096	0.81289	0.81911	0.82585	0.83331
351	0.79345	0.80569	0.81136	0.81329	0.81949	0.82621	0.83366
352	0.79388	0.80608	0.81176	0.81367	0.81987	0.82657	0.83400
353	0.79431	0.80648	0.81215	0.81407	0.82025	0.82693	0.83434
354	0.79477	0.80689	0.81255	0.81446	0.82063	0.82729	0.83468
355	0.79523	0.80730	0.81295	0.81485	0.82100	0.82765	0.83502
356	0.79564	0.80770	0.81334	0.81524	0.82137	0.82800	0.83536
357	0.79607	0.80811	0.81373	0.81561	0.82174	0.82835	0.83570
358	0.79651	0.80849	0.81410	0.81599	0.82211	0.82871	0.83604
359	0.79695	0.80890	0.81449	0.81636	0.82248	0.82905	0.83637
360	0.79734	0.80930	0.81487	0.81673	0.82284	0.82941	0.83670
361	0.79775	0.80969	0.81525	0.81712	0.82320	0.82975	0.83704
362	0.79817	0.81008	0.81564	0.81749	0.82356	0.83010	0.83736
363	0.79857	0.81047	0.81600	0.81786	0.82392	0.83045	0.83769
364	0.79896	0.81084	0.81638	0.81824	0.82428	0.83079	0.83802
365	0.79937	0.81122	0.81676	0.81860	0.82464	0.83113	0.83834
366	0.79978	0.81160	0.81714	0.81897	0.82499	0.83148	0.83866
367	0.80020	0.81199	0.81750	0.81935	0.82534	0.83181	0.83898
368	0.80062	0.81236	0.81787	0.81971	0.82569	0.83214	0.83930
369	0.80104	0.81274	0.81823	0.82007	0.82603	0.83248	0.83962
370	0.80144	0.81311	0.81860	0.82043	0.82638	0.83282	0.83994
371	0.80184	0.81349	0.81897	0.82079	0.82673	0.83315	0.84026
372	0.80224	0.81386	0.81934	0.82116	0.82707	0.83348	0.84057

N	0.1%	0.5%	1%	1.25%	2.5%	5%	10%
373	0.80260	0.81424	0.81969	0.82151	0.82741	0.83382	0.84089
374	0.80303	0.81461	0.82006	0.82187	0.82775	0.83414	0.84119
375	0.80341	0.81496	0.82043	0.82223	0.82809	0.83447	0.84151
376	0.80381	0.81534	0.82079	0.82258	0.82844	0.83480	0.84182
377	0.80421	0.81572	0.82113	0.82293	0.82877	0.83512	0.84212
378	0.80459	0.81608	0.82148	0.82328	0.82911	0.83544	0.84243
379	0.80495	0.81644	0.82183	0.82363	0.82944	0.83576	0.84273
380	0.80532	0.81681	0.82218	0.82396	0.82978	0.83609	0.84304
381	0.80571	0.81716	0.82252	0.82431	0.83011	0.83640	0.84334
382	0.80611	0.81753	0.82287	0.82464	0.83044	0.83672	0.84364
383	0.80646	0.81789	0.82322	0.82499	0.83076	0.83703	0.84394
384	0.80680	0.81823	0.82356	0.82533	0.83109	0.83734	0.84424
385	0.80716	0.81860	0.82390	0.82567	0.83142	0.83765	0.84454
386	0.80754	0.81895	0.82423	0.82601	0.83174	0.83796	0.84483
387	0.80793	0.81930	0.82458	0.82634	0.83206	0.83827	0.84513
388	0.80830	0.81966	0.82492	0.82668	0.83238	0.83858	0.84542
389	0.80867	0.82000	0.82525	0.82701	0.83271	0.83889	0.84571
390	0.80907	0.82034	0.82558	0.82733	0.83303	0.83919	0.84600
391	0.80945	0.82069	0.82591	0.82766	0.83335	0.83950	0.84629
392	0.80980	0.82104	0.82625	0.82799	0.83366	0.83980	0.84658
393	0.81015	0.82138	0.82659	0.82831	0.83397	0.84010	0.84686
394	0.81049	0.82172	0.82692	0.82864	0.83428	0.84040	0.84715
395	0.81082	0.82205	0.82725	0.82896	0.83460	0.84070	0.84743
396	0.81120	0.82240	0.82757	0.82928	0.83491	0.84100	0.84772
397	0.81156	0.82273	0.82790	0.82961	0.83522	0.84130	0.84800
398	0.81193	0.82307	0.82821	0.82993	0.83553	0.84159	0.84828
399	0.81227	0.82341	0.82854	0.83024	0.83584	0.84189	0.84857
400	0.81264	0.82373	0.82886	0.83056	0.83614	0.84218	0.84885
401	0.81299	0.82406	0.82918	0.83089	0.83645	0.84247	0.84912
402	0.81335	0.82439	0.82950	0.83120	0.83676	0.84276	0.84940
403	0.81368	0.82472	0.82982	0.83152	0.83706	0.84305	0.84967
404	0.81405	0.82506	0.83013	0.83183	0.83736	0.84334	0.84995
405	0.81439	0.82536	0.83045	0.83214	0.83766	0.84363	0.85022
406	0.81473	0.82570	0.83076	0.83245	0.83796	0.84391	0.85050
407	0.81509	0.82603	0.83108	0.83275	0.83826	0.84420	0.85077
408	0.81544	0.82635	0.83139	0.83306	0.83855	0.84449	0.85104
409	0.81577	0.82666	0.83169	0.83337	0.83884	0.84477	0.85131
410	0.81612	0.82699	0.83201	0.83367	0.83913	0.84505	0.85158
411	0.81645	0.82730	0.83231	0.83398	0.83943	0.84533	0.85184
412	0.81679	0.82761	0.83261	0.83428	0.83972	0.84560	0.85211
413	0.81715	0.82792	0.83292	0.83458	0.84001	0.84588	0.85237
414	0.81747	0.82824	0.83323	0.83488	0.84030	0.84616	0.85264
415	0.81777	0.82854	0.83353	0.83518	0.84059	0.84643	0.85290
416	0.81810	0.82885	0.83383	0.83547	0.84087	0.84670	0.85316
417	0.81844	0.82917	0.83412	0.83577	0.84116	0.84698	0.85342
418	0.81874	0.82947	0.83442	0.83607	0.84144	0.84725	0.85368
419	0.81906	0.82978	0.83471	0.83637	0.84172	0.84752	0.85394
420	0.81941	0.83008	0.83502	0.83665	0.84201	0.84779	0.85420
421	0.81974	0.83039	0.83531	0.83694	0.84228	0.84806	0.85445
422	0.82008	0.83068	0.83561	0.83723	0.84256	0.84833	0.85471
423	0.82038	0.83099	0.83590	0.83753	0.84283	0.84860	0.85496
424	0.82071	0.83129	0.83620	0.83781	0.84311	0.84886	0.85521
425	0.82104	0.83160	0.83648	0.83810	0.84339	0.84912	0.85547
426	0.82138	0.83190	0.83677	0.83840	0.84367	0.84939	0.85572
427	0.82168	0.83219	0.83706	0.83868	0.84394	0.84966	0.85597
428	0.82201	0.83249	0.83734	0.83897	0.84421	0.84991	0.85621
429	0.82232	0.83279	0.83763	0.83925	0.84449	0.85017	0.85646
430	0.82263	0.83307	0.83792	0.83953	0.84476	0.85043	0.85671
431	0.82296	0.83336	0.83821	0.83981	0.84503	0.85070	0.85696
432	0.82331	0.83368	0.83848	0.84009	0.84530	0.85095	0.85720
433	0.82362	0.83396	0.83877	0.84036	0.84557	0.85121	0.85745
434	0.82393	0.83424	0.83905	0.84064	0.84584	0.85146	0.85769
435	0.82424	0.83454	0.83933	0.84092	0.84610	0.85172	0.85794
436	0.82454	0.83482	0.83960	0.84119	0.84636	0.85197	0.85818
437	0.82484	0.83511	0.83988	0.84146	0.84662	0.85222	0.85842
438	0.82514	0.83540	0.84015	0.84174	0.84689	0.85247	0.85866
439	0.82546	0.83568	0.84042	0.84200	0.84715	0.85273	0.85890
440	0.82576	0.83597	0.84070	0.84228	0.84741	0.85297	0.85914
441	0.82604	0.83624	0.84097	0.84254	0.84768	0.85322	0.85937
442	0.82634	0.83652	0.84123	0.84280	0.84793	0.85347	0.85961
443	0.82662	0.83679	0.84150	0.84307	0.84819	0.85372	0.85984
444	0.82691	0.83706	0.84178	0.84334	0.84845	0.85396	0.86008
445	0.82721	0.83734	0.84205	0.84361	0.84869	0.85421	0.86031
446	0.82750	0.83762	0.84231	0.84387	0.84895	0.85445	0.86054

N	0.1%	0.5%	1%	1.25%	2.5%	5%	10%
447	0.82781	0.83790	0.84257	0.84414	0.84920	0.85470	0.86078
448	0.82811	0.83817	0.84285	0.84440	0.84945	0.85494	0.86100
449	0.82838	0.83843	0.84311	0.84466	0.84970	0.85518	0.86123
450	0.82868	0.83871	0.84337	0.84492	0.84995	0.85542	0.86146
451	0.82897	0.83898	0.84363	0.84518	0.85021	0.85566	0.86169
452	0.82926	0.83925	0.84390	0.84544	0.85046	0.85590	0.86192
453	0.82956	0.83951	0.84416	0.84570	0.85071	0.85614	0.86215
454	0.82985	0.83978	0.84442	0.84595	0.85095	0.85638	0.86237
455	0.83013	0.84006	0.84469	0.84621	0.85120	0.85661	0.86260
456	0.83042	0.84032	0.84495	0.84646	0.85145	0.85685	0.86282
457	0.83072	0.84059	0.84521	0.84672	0.85169	0.85708	0.86305
458	0.83096	0.84084	0.84546	0.84697	0.85194	0.85732	0.86327
459	0.83124	0.84111	0.84572	0.84723	0.85218	0.85755	0.86349
460	0.83155	0.84137	0.84597	0.84748	0.85242	0.85778	0.86371
461	0.83182	0.84163	0.84623	0.84773	0.85267	0.85801	0.86393
462	0.83211	0.84190	0.84648	0.84798	0.85291	0.85824	0.86415
463	0.83239	0.84216	0.84673	0.84822	0.85315	0.85847	0.86437
464	0.83267	0.84242	0.84698	0.84847	0.85339	0.85870	0.86459
465	0.83293	0.84269	0.84722	0.84872	0.85363	0.85893	0.86481
466	0.83320	0.84295	0.84747	0.84897	0.85386	0.85916	0.86502
467	0.83344	0.84319	0.84773	0.84922	0.85410	0.85939	0.86524
468	0.83371	0.84345	0.84797	0.84947	0.85434	0.85961	0.86546
469	0.83399	0.84370	0.84822	0.84971	0.85457	0.85984	0.86567
470	0.83425	0.84396	0.84847	0.84995	0.85480	0.86006	0.86588
471	0.83452	0.84420	0.84871	0.85020	0.85503	0.86028	0.86609
472	0.83480	0.84446	0.84894	0.85043	0.85527	0.86050	0.86630
473	0.83505	0.84471	0.84919	0.85068	0.85549	0.86072	0.86651
474	0.83533	0.84496	0.84944	0.85091	0.85572	0.86095	0.86673
475	0.83559	0.84521	0.84968	0.85115	0.85595	0.86116	0.86693
476	0.83585	0.84545	0.84992	0.85139	0.85618	0.86139	0.86714
477	0.83610	0.84571	0.85016	0.85163	0.85641	0.86160	0.86735
478	0.83637	0.84596	0.85040	0.85187	0.85664	0.86182	0.86756
479	0.83665	0.84621	0.85063	0.85210	0.85687	0.86204	0.86777
480	0.83688	0.84645	0.85087	0.85234	0.85710	0.86225	0.86797
481	0.83715	0.84670	0.85110	0.85257	0.85732	0.86246	0.86818
482	0.83745	0.84694	0.85134	0.85281	0.85754	0.86268	0.86838
483	0.83769	0.84718	0.85157	0.85304	0.85776	0.86289	0.86858
484	0.83797	0.84742	0.85180	0.85328	0.85799	0.86310	0.86879
485	0.83822	0.84766	0.85203	0.85350	0.85821	0.86332	0.86899
486	0.83847	0.84790	0.85226	0.85372	0.85843	0.86353	0.86919
487	0.83873	0.84813	0.85249	0.85395	0.85865	0.86375	0.86939
488	0.83900	0.84838	0.85271	0.85417	0.85887	0.86396	0.86959
489	0.83924	0.84861	0.85294	0.85440	0.85909	0.86416	0.86979
490	0.83949	0.84884	0.85317	0.85462	0.85930	0.86437	0.86999
491	0.83976	0.84907	0.85340	0.85485	0.85953	0.86458	0.87019
492	0.84001	0.84930	0.85362	0.85507	0.85974	0.86479	0.87038
493	0.84026	0.84954	0.85385	0.85529	0.85995	0.86499	0.87058
494	0.84050	0.84978	0.85408	0.85551	0.86017	0.86520	0.87077
495	0.84073	0.85002	0.85430	0.85574	0.86038	0.86541	0.87097
496	0.84099	0.85024	0.85452	0.85596	0.86059	0.86561	0.87117
497	0.84124	0.85047	0.85475	0.85618	0.86080	0.86582	0.87136
498	0.84147	0.85070	0.85497	0.85639	0.86101	0.86602	0.87155
499	0.84174	0.85094	0.85519	0.85662	0.86122	0.86622	0.87175
500	0.84197	0.85117	0.85542	0.85684	0.86143	0.86642	0.87194
501	0.84221	0.85140	0.85564	0.85706	0.86164	0.86662	0.87213
502	0.84245	0.85163	0.85585	0.85728	0.86186	0.86682	0.87232
503	0.84271	0.85185	0.85607	0.85749	0.86207	0.86703	0.87251
504	0.84295	0.85207	0.85628	0.85770	0.86228	0.86722	0.87270
505	0.84318	0.85231	0.85651	0.85792	0.86248	0.86742	0.87289
506	0.84340	0.85253	0.85672	0.85813	0.86269	0.86762	0.87308
507	0.84366	0.85274	0.85693	0.85835	0.86290	0.86782	0.87327
508	0.84389	0.85297	0.85715	0.85856	0.86311	0.86801	0.87345
509	0.84413	0.85319	0.85736	0.85877	0.86331	0.86821	0.87364
510	0.84437	0.85341	0.85758	0.85898	0.86351	0.86840	0.87382
511	0.84459	0.85363	0.85779	0.85919	0.86372	0.86860	0.87401
512	0.84483	0.85384	0.85800	0.85940	0.86392	0.86879	0.87420
513	0.84508	0.85406	0.85822	0.85960	0.86412	0.86898	0.87438
514	0.84531	0.85429	0.85843	0.85981	0.86432	0.86918	0.87456
515	0.84556	0.85451	0.85863	0.86002	0.86452	0.86937	0.87475
516	0.84579	0.85472	0.85885	0.86023	0.86472	0.86956	0.87493
517	0.84602	0.85494	0.85906	0.86044	0.86492	0.86975	0.87511
518	0.84624	0.85514	0.85926	0.86064	0.86512	0.86994	0.87529
519	0.84645	0.85535	0.85947	0.86085	0.86532	0.87013	0.87547
520	0.84669	0.85556	0.85967	0.86105	0.86552	0.87032	0.87565

N	0.1%	0.5%	1%	1.25%	2.5%	5%	10%
521	0.84690	0.85577	0.85988	0.86126	0.86572	0.87050	0.87583
522	0.84715	0.85598	0.86009	0.86147	0.86591	0.87069	0.87601
523	0.84735	0.85620	0.86029	0.86166	0.86611	0.87088	0.87619
524	0.84759	0.85641	0.86050	0.86186	0.86630	0.87107	0.87637
525	0.84781	0.85661	0.86069	0.86206	0.86649	0.87125	0.87654
526	0.84806	0.85683	0.86090	0.86226	0.86668	0.87144	0.87672
527	0.84829	0.85703	0.86109	0.86246	0.86688	0.87163	0.87690
528	0.84851	0.85723	0.86129	0.86266	0.86707	0.87181	0.87708
529	0.84871	0.85744	0.86149	0.86286	0.86726	0.87199	0.87725
530	0.84893	0.85765	0.86169	0.86305	0.86745	0.87217	0.87742
531	0.84916	0.85786	0.86189	0.86325	0.86764	0.87235	0.87760
532	0.84940	0.85807	0.86209	0.86345	0.86783	0.87253	0.87777
533	0.84961	0.85828	0.86229	0.86365	0.86802	0.87272	0.87795
534	0.84985	0.85848	0.86249	0.86384	0.86821	0.87290	0.87812
535	0.85007	0.85869	0.86269	0.86403	0.86839	0.87308	0.87829
536	0.85030	0.85889	0.86288	0.86423	0.86858	0.87326	0.87846
537	0.85051	0.85909	0.86308	0.86442	0.86877	0.87344	0.87863
538	0.85072	0.85929	0.86328	0.86461	0.86895	0.87362	0.87880
539	0.85093	0.85950	0.86346	0.86481	0.86914	0.87380	0.87897
540	0.85114	0.85969	0.86366	0.86500	0.86932	0.87398	0.87914
541	0.85134	0.85989	0.86385	0.86519	0.86951	0.87416	0.87931
542	0.85155	0.86009	0.86405	0.86538	0.86969	0.87433	0.87947
543	0.85175	0.86028	0.86424	0.86557	0.86987	0.87451	0.87964
544	0.85197	0.86047	0.86443	0.86576	0.87005	0.87468	0.87981
545	0.85219	0.86068	0.86462	0.86595	0.87023	0.87486	0.87997
546	0.85241	0.86087	0.86481	0.86614	0.87041	0.87503	0.88014
547	0.85261	0.86107	0.86500	0.86632	0.87059	0.87521	0.88030
548	0.85282	0.86125	0.86519	0.86651	0.87077	0.87538	0.88047
549	0.85302	0.86145	0.86537	0.86669	0.87095	0.87555	0.88063
550	0.85322	0.86165	0.86556	0.86688	0.87113	0.87572	0.88080
551	0.85342	0.86184	0.86575	0.86706	0.87131	0.87589	0.88096
552	0.85363	0.86204	0.86594	0.86724	0.87149	0.87606	0.88112
553	0.85384	0.86224	0.86612	0.86743	0.87167	0.87624	0.88128
554	0.85405	0.86244	0.86631	0.86762	0.87184	0.87641	0.88144
555	0.85424	0.86263	0.86649	0.86780	0.87201	0.87657	0.88160
556	0.85444	0.86282	0.86667	0.86798	0.87218	0.87674	0.88177
557	0.85466	0.86301	0.86686	0.86816	0.87236	0.87691	0.88192
558	0.85486	0.86320	0.86705	0.86834	0.87254	0.87708	0.88209
559	0.85508	0.86340	0.86723	0.86852	0.87271	0.87725	0.88224
560	0.85528	0.86358	0.86742	0.86870	0.87288	0.87741	0.88241
561	0.85550	0.86376	0.86760	0.86888	0.87306	0.87758	0.88256
562	0.85571	0.86396	0.86778	0.86906	0.87323	0.87774	0.88272
563	0.85590	0.86415	0.86796	0.86924	0.87340	0.87791	0.88288
564	0.85609	0.86433	0.86814	0.86942	0.87358	0.87808	0.88304
565	0.85629	0.86452	0.86832	0.86959	0.87375	0.87824	0.88319
566	0.85650	0.86471	0.86850	0.86977	0.87391	0.87841	0.88334
567	0.85669	0.86489	0.86868	0.86995	0.87408	0.87857	0.88350
568	0.85688	0.86507	0.86886	0.87013	0.87425	0.87873	0.88366
569	0.85709	0.86525	0.86903	0.87030	0.87442	0.87889	0.88381
570	0.85727	0.86543	0.86921	0.87048	0.87458	0.87905	0.88397
571	0.85745	0.86561	0.86939	0.87065	0.87475	0.87921	0.88412
572	0.85767	0.86580	0.86956	0.87083	0.87492	0.87937	0.88427
573	0.85786	0.86598	0.86974	0.87101	0.87509	0.87953	0.88443
574	0.85804	0.86616	0.86991	0.87118	0.87526	0.87969	0.88458
575	0.85823	0.86634	0.87009	0.87135	0.87542	0.87985	0.88473
576	0.85843	0.86651	0.87026	0.87152	0.87559	0.88001	0.88488
577	0.85860	0.86668	0.87043	0.87169	0.87576	0.88017	0.88503
578	0.85880	0.86686	0.87061	0.87186	0.87592	0.88033	0.88518
579	0.85898	0.86705	0.87079	0.87203	0.87608	0.88049	0.88533
580	0.85920	0.86723	0.87095	0.87220	0.87625	0.88065	0.88548
581	0.85937	0.86740	0.87112	0.87237	0.87641	0.88081	0.88563
582	0.85957	0.86758	0.87129	0.87253	0.87657	0.88096	0.88578
583	0.85976	0.86775	0.87146	0.87270	0.87673	0.88112	0.88593
584	0.85994	0.86794	0.87163	0.87287	0.87690	0.88127	0.88608
585	0.86013	0.86810	0.87180	0.87304	0.87706	0.88143	0.88623
586	0.86028	0.86828	0.87197	0.87320	0.87722	0.88158	0.88637
587	0.86048	0.86846	0.87214	0.87337	0.87738	0.88173	0.88652
588	0.86069	0.86863	0.87231	0.87354	0.87753	0.88189	0.88667
589	0.86088	0.86881	0.87248	0.87370	0.87770	0.88204	0.88681
590	0.86106	0.86898	0.87264	0.87387	0.87785	0.88219	0.88696
591	0.86124	0.86914	0.87281	0.87404	0.87802	0.88234	0.88710
592	0.86141	0.86932	0.87298	0.87420	0.87817	0.88249	0.88724
593	0.86160	0.86949	0.87315	0.87436	0.87833	0.88264	0.88739
594	0.86177	0.86966	0.87330	0.87452	0.87849	0.88280	0.88753

N	0.1%	0.5%	1%	1.25%	2.5%	5%	10%
595	0.86196	0.86984	0.87347	0.87469	0.87865	0.88295	0.88768
596	0.86215	0.87001	0.87364	0.87485	0.87881	0.88309	0.88782
597	0.86232	0.87018	0.87380	0.87501	0.87896	0.88325	0.88796
598	0.86251	0.87035	0.87397	0.87517	0.87912	0.88340	0.88810
599	0.86269	0.87051	0.87413	0.87533	0.87928	0.88354	0.88825
600	0.86287	0.87068	0.87429	0.87550	0.87943	0.88369	0.88839
601	0.86304	0.87086	0.87445	0.87566	0.87958	0.88384	0.88853
602	0.86323	0.87101	0.87461	0.87582	0.87974	0.88399	0.88867
603	0.86339	0.87117	0.87478	0.87598	0.87989	0.88413	0.88881
604	0.86355	0.87134	0.87494	0.87614	0.88004	0.88428	0.88895
605	0.86372	0.87150	0.87510	0.87630	0.88020	0.88443	0.88909
606	0.86388	0.87166	0.87526	0.87646	0.88035	0.88457	0.88923
607	0.86406	0.87183	0.87542	0.87661	0.88050	0.88472	0.88936
608	0.86423	0.87199	0.87558	0.87677	0.88065	0.88486	0.88950
609	0.86439	0.87216	0.87574	0.87692	0.88080	0.88501	0.88964
610	0.86458	0.87231	0.87590	0.87708	0.88095	0.88515	0.88978
611	0.86474	0.87248	0.87605	0.87724	0.88110	0.88529	0.88992
612	0.86492	0.87264	0.87621	0.87739	0.88125	0.88544	0.89005
613	0.86510	0.87281	0.87636	0.87755	0.88140	0.88558	0.89019
614	0.86526	0.87296	0.87651	0.87770	0.88155	0.88573	0.89032
615	0.86545	0.87313	0.87667	0.87785	0.88169	0.88587	0.89046
616	0.86561	0.87328	0.87682	0.87801	0.88184	0.88601	0.89060
617	0.86578	0.87345	0.87697	0.87816	0.88198	0.88615	0.89073
618	0.86598	0.87361	0.87713	0.87832	0.88213	0.88629	0.89087
619	0.86614	0.87377	0.87729	0.87847	0.88228	0.88643	0.89100
620	0.86631	0.87394	0.87744	0.87862	0.88243	0.88657	0.89113
621	0.86648	0.87410	0.87759	0.87877	0.88258	0.88671	0.89127
622	0.86664	0.87425	0.87774	0.87892	0.88272	0.88685	0.89140
623	0.86681	0.87441	0.87790	0.87907	0.88287	0.88699	0.89153
624	0.86698	0.87456	0.87804	0.87921	0.88301	0.88713	0.89167
625	0.86714	0.87471	0.87820	0.87937	0.88316	0.88727	0.89180
626	0.86732	0.87487	0.87835	0.87952	0.88330	0.88741	0.89193
627	0.86748	0.87502	0.87850	0.87967	0.88344	0.88755	0.89206
628	0.86766	0.87518	0.87865	0.87982	0.88359	0.88768	0.89219
629	0.86783	0.87532	0.87880	0.87996	0.88373	0.88782	0.89232
630	0.86798	0.87549	0.87894	0.88011	0.88387	0.88796	0.89245
631	0.86814	0.87564	0.87910	0.88025	0.88401	0.88809	0.89258
632	0.86831	0.87579	0.87925	0.88041	0.88416	0.88823	0.89271
633	0.86848	0.87594	0.87940	0.88055	0.88430	0.88836	0.89284
634	0.86864	0.87609	0.87953	0.88070	0.88444	0.88850	0.89297
635	0.86882	0.87624	0.87968	0.88084	0.88458	0.88863	0.89310
636	0.86900	0.87639	0.87983	0.88099	0.88472	0.88877	0.89322
637	0.86914	0.87654	0.87997	0.88113	0.88486	0.88890	0.89335
638	0.86930	0.87669	0.88012	0.88128	0.88499	0.88903	0.89348
639	0.86945	0.87685	0.88027	0.88142	0.88513	0.88916	0.89361
640	0.86960	0.87700	0.88041	0.88157	0.88527	0.88930	0.89373
641	0.86974	0.87715	0.88056	0.88171	0.88541	0.88943	0.89386
642	0.86990	0.87730	0.88070	0.88185	0.88555	0.88956	0.89399
643	0.87005	0.87745	0.88084	0.88200	0.88569	0.88969	0.89411
644	0.87021	0.87759	0.88098	0.88214	0.88582	0.88982	0.89424
645	0.87036	0.87774	0.88112	0.88227	0.88596	0.88995	0.89436
646	0.87050	0.87788	0.88126	0.88241	0.88609	0.89008	0.89449
647	0.87067	0.87802	0.88141	0.88255	0.88623	0.89021	0.89461
648	0.87083	0.87817	0.88155	0.88269	0.88637	0.89034	0.89474
649	0.87099	0.87832	0.88169	0.88283	0.88651	0.89047	0.89486
650	0.87114	0.87846	0.88184	0.88297	0.88664	0.89060	0.89498
651	0.87130	0.87861	0.88198	0.88312	0.88678	0.89073	0.89510
652	0.87147	0.87875	0.88212	0.88325	0.88691	0.89086	0.89523
653	0.87161	0.87890	0.88226	0.88339	0.88705	0.89099	0.89535
654	0.87177	0.87904	0.88239	0.88353	0.88718	0.89112	0.89547
655	0.87192	0.87919	0.88253	0.88367	0.88731	0.89124	0.89559
656	0.87208	0.87933	0.88267	0.88381	0.88744	0.89137	0.89571
657	0.87223	0.87948	0.88280	0.88394	0.88758	0.89150	0.89583
658	0.87238	0.87962	0.88295	0.88408	0.88771	0.89162	0.89595
659	0.87252	0.87978	0.88309	0.88422	0.88784	0.89175	0.89608
660	0.87269	0.87991	0.88322	0.88435	0.88797	0.89188	0.89619
661	0.87284	0.88005	0.88336	0.88449	0.88810	0.89200	0.89632
662	0.87298	0.88019	0.88350	0.88462	0.88823	0.89213	0.89644
663	0.87313	0.88033	0.88364	0.88476	0.88836	0.89225	0.89656
664	0.87329	0.88048	0.88377	0.88489	0.88849	0.89238	0.89668
665	0.87345	0.88062	0.88391	0.88502	0.88862	0.89250	0.89679
666	0.87360	0.88075	0.88405	0.88516	0.88875	0.89263	0.89691
667	0.87375	0.88090	0.88418	0.88529	0.88888	0.89275	0.89703
668	0.87390	0.88103	0.88432	0.88542	0.88901	0.89288	0.89715

N	0.1%	0.5%	1%	1.25%	2.5%	5%	10%
669	0.87404	0.88117	0.88445	0.88556	0.88914	0.89300	0.89727
670	0.87419	0.88131	0.88458	0.88569	0.88926	0.89312	0.89738
671	0.87433	0.88145	0.88472	0.88582	0.88939	0.89324	0.89750
672	0.87448	0.88159	0.88486	0.88596	0.88952	0.89336	0.89762
673	0.87462	0.88173	0.88499	0.88609	0.88964	0.89349	0.89773
674	0.87477	0.88187	0.88513	0.88622	0.88977	0.89361	0.89785
675	0.87493	0.88200	0.88526	0.88636	0.88990	0.89373	0.89796
676	0.87507	0.88214	0.88539	0.88649	0.89002	0.89385	0.89808
677	0.87522	0.88227	0.88552	0.88662	0.89015	0.89397	0.89819
678	0.87537	0.88241	0.88566	0.88675	0.89028	0.89409	0.89831
679	0.87552	0.88254	0.88579	0.88688	0.89040	0.89421	0.89843
680	0.87565	0.88268	0.88591	0.88700	0.89052	0.89433	0.89854
681	0.87579	0.88281	0.88605	0.88714	0.89065	0.89445	0.89865
682	0.87592	0.88294	0.88618	0.88727	0.89077	0.89457	0.89877
683	0.87606	0.88308	0.88631	0.88739	0.89090	0.89469	0.89888
684	0.87619	0.88322	0.88643	0.88752	0.89102	0.89480	0.89899
685	0.87635	0.88335	0.88657	0.88765	0.89115	0.89492	0.89911
686	0.87649	0.88348	0.88670	0.88778	0.89127	0.89504	0.89922
687	0.87665	0.88361	0.88682	0.88790	0.89139	0.89516	0.89933
688	0.87678	0.88374	0.88696	0.88803	0.89151	0.89527	0.89944
689	0.87693	0.88387	0.88708	0.88815	0.89164	0.89539	0.89955
690	0.87707	0.88401	0.88720	0.88828	0.89175	0.89551	0.89967
691	0.87721	0.88413	0.88733	0.88841	0.89188	0.89563	0.89978
692	0.87735	0.88426	0.88746	0.88853	0.89200	0.89574	0.89989
693	0.87749	0.88440	0.88759	0.88866	0.89212	0.89586	0.90000
694	0.87764	0.88452	0.88772	0.88879	0.89224	0.89598	0.90011
695	0.87776	0.88465	0.88784	0.88891	0.89236	0.89609	0.90022
696	0.87789	0.88478	0.88797	0.88904	0.89248	0.89621	0.90033
697	0.87804	0.88492	0.88809	0.88916	0.89260	0.89632	0.90044
698	0.87818	0.88505	0.88822	0.88928	0.89272	0.89644	0.90055
699	0.87832	0.88518	0.88834	0.88940	0.89284	0.89655	0.90066
700	0.87845	0.88531	0.88847	0.88953	0.89296	0.89667	0.90076
701	0.87858	0.88544	0.88859	0.88965	0.89308	0.89678	0.90087
702	0.87872	0.88557	0.88872	0.88977	0.89319	0.89689	0.90098
703	0.87885	0.88570	0.88885	0.88989	0.89331	0.89700	0.90109
704	0.87898	0.88583	0.88897	0.89002	0.89343	0.89712	0.90120
705	0.87912	0.88596	0.88910	0.89014	0.89355	0.89723	0.90130
706	0.87926	0.88608	0.88922	0.89026	0.89367	0.89734	0.90141
707	0.87939	0.88621	0.88934	0.89039	0.89378	0.89745	0.90152
708	0.87953	0.88634	0.88946	0.89050	0.89390	0.89757	0.90163
709	0.87968	0.88646	0.88958	0.89063	0.89401	0.89768	0.90173
710	0.87981	0.88658	0.88971	0.89075	0.89413	0.89779	0.90184
711	0.87994	0.88670	0.88982	0.89087	0.89425	0.89790	0.90194
712	0.88007	0.88683	0.88995	0.89099	0.89436	0.89801	0.90205
713	0.88020	0.88696	0.89007	0.89111	0.89447	0.89812	0.90215
714	0.88033	0.88708	0.89019	0.89122	0.89459	0.89823	0.90226
715	0.88047	0.88721	0.89032	0.89135	0.89470	0.89834	0.90236
716	0.88060	0.88733	0.89043	0.89147	0.89482	0.89845	0.90247
717	0.88072	0.88746	0.89055	0.89159	0.89493	0.89856	0.90257
718	0.88086	0.88758	0.89067	0.89170	0.89504	0.89867	0.90268
719	0.88100	0.88771	0.89079	0.89182	0.89516	0.89878	0.90278
720	0.88112	0.88783	0.89090	0.89194	0.89527	0.89889	0.90289
721	0.88124	0.88795	0.89102	0.89205	0.89539	0.89900	0.90299
722	0.88137	0.88807	0.89114	0.89217	0.89550	0.89910	0.90309
723	0.88152	0.88819	0.89126	0.89228	0.89561	0.89921	0.90320
724	0.88165	0.88832	0.89137	0.89240	0.89572	0.89932	0.90330
725	0.88177	0.88843	0.89149	0.89251	0.89583	0.89943	0.90340
726	0.88190	0.88857	0.89161	0.89263	0.89595	0.89954	0.90351
727	0.88203	0.88868	0.89172	0.89275	0.89606	0.89964	0.90361
728	0.88216	0.88880	0.89184	0.89286	0.89617	0.89975	0.90371
729	0.88229	0.88892	0.89195	0.89298	0.89628	0.89985	0.90381
730	0.88242	0.88903	0.89207	0.89309	0.89639	0.89996	0.90391
731	0.88254	0.88915	0.89219	0.89321	0.89650	0.90007	0.90401
732	0.88267	0.88928	0.89231	0.89332	0.89661	0.90017	0.90412
733	0.88280	0.88939	0.89243	0.89344	0.89672	0.90028	0.90422
734	0.88293	0.88951	0.89254	0.89355	0.89683	0.90038	0.90432
735	0.88305	0.88964	0.89265	0.89366	0.89694	0.90049	0.90442
736	0.88316	0.88975	0.89276	0.89378	0.89705	0.90059	0.90451
737	0.88329	0.88987	0.89288	0.89389	0.89716	0.90070	0.90461
738	0.88341	0.88999	0.89299	0.89400	0.89727	0.90080	0.90471
739	0.88353	0.89010	0.89310	0.89411	0.89737	0.90090	0.90481
740	0.88365	0.89023	0.89322	0.89422	0.89748	0.90101	0.90491
741	0.88378	0.89034	0.89333	0.89433	0.89759	0.90111	0.90501
742	0.88390	0.89046	0.89344	0.89445	0.89769	0.90121	0.90511

N	0.1%	0.5%	1%	1.25%	2.5%	5%	10%
743	0.88402	0.89056	0.89355	0.89456	0.89780	0.90132	0.90521
744	0.88415	0.89068	0.89367	0.89467	0.89791	0.90142	0.90530
745	0.88426	0.89080	0.89378	0.89478	0.89801	0.90152	0.90540
746	0.88439	0.89092	0.89389	0.89489	0.89812	0.90163	0.90550
747	0.88451	0.89103	0.89400	0.89500	0.89822	0.90173	0.90559
748	0.88464	0.89114	0.89411	0.89511	0.89833	0.90183	0.90569
749	0.88475	0.89126	0.89422	0.89522	0.89843	0.90193	0.90579
750	0.88487	0.89138	0.89433	0.89532	0.89854	0.90203	0.90589
751	0.88499	0.89150	0.89445	0.89544	0.89865	0.90213	0.90598
752	0.88512	0.89161	0.89456	0.89554	0.89875	0.90223	0.90608
753	0.88524	0.89173	0.89466	0.89565	0.89885	0.90233	0.90617
754	0.88536	0.89184	0.89477	0.89576	0.89896	0.90243	0.90627
755	0.88547	0.89196	0.89488	0.89587	0.89906	0.90253	0.90637
756	0.88561	0.89206	0.89499	0.89598	0.89917	0.90263	0.90646
757	0.88574	0.89217	0.89510	0.89609	0.89927	0.90273	0.90656
758	0.88585	0.89228	0.89521	0.89619	0.89938	0.90284	0.90665
759	0.88597	0.89239	0.89532	0.89630	0.89948	0.90294	0.90675
760	0.88609	0.89250	0.89542	0.89641	0.89958	0.90303	0.90684
761	0.88621	0.89261	0.89553	0.89651	0.89969	0.90313	0.90694
762	0.88634	0.89272	0.89564	0.89662	0.89979	0.90323	0.90703
763	0.88646	0.89283	0.89575	0.89672	0.89989	0.90333	0.90712
764	0.88659	0.89294	0.89586	0.89683	0.89999	0.90343	0.90722
765	0.88669	0.89304	0.89596	0.89694	0.90010	0.90353	0.90731
766	0.88681	0.89315	0.89607	0.89704	0.90020	0.90363	0.90741
767	0.88691	0.89326	0.89617	0.89714	0.90030	0.90372	0.90750
768	0.88703	0.89336	0.89628	0.89725	0.90040	0.90382	0.90759
769	0.88715	0.89347	0.89638	0.89736	0.90050	0.90392	0.90769
770	0.88727	0.89357	0.89648	0.89746	0.90060	0.90401	0.90778
771	0.88739	0.89368	0.89659	0.89756	0.90070	0.90411	0.90787
772	0.88752	0.89379	0.89670	0.89767	0.90080	0.90420	0.90796
773	0.88763	0.89390	0.89680	0.89778	0.90090	0.90430	0.90805
774	0.88773	0.89401	0.89691	0.89788	0.90100	0.90440	0.90815
775	0.88785	0.89412	0.89701	0.89798	0.90110	0.90449	0.90824
776	0.88797	0.89423	0.89712	0.89808	0.90120	0.90459	0.90833
777	0.88808	0.89434	0.89722	0.89819	0.90130	0.90468	0.90842
778	0.88820	0.89445	0.89733	0.89829	0.90139	0.90478	0.90851
779	0.88832	0.89455	0.89743	0.89839	0.90149	0.90487	0.90860
780	0.88844	0.89466	0.89753	0.89849	0.90159	0.90497	0.90869
781	0.88855	0.89476	0.89764	0.89859	0.90169	0.90506	0.90878
782	0.88867	0.89487	0.89774	0.89869	0.90179	0.90516	0.90887
783	0.88879	0.89497	0.89784	0.89880	0.90189	0.90525	0.90896
784	0.88890	0.89509	0.89794	0.89890	0.90199	0.90535	0.90905
785	0.88902	0.89519	0.89804	0.89899	0.90209	0.90544	0.90914
786	0.88912	0.89529	0.89814	0.89909	0.90219	0.90553	0.90923
787	0.88924	0.89540	0.89825	0.89920	0.90229	0.90563	0.90932
788	0.88935	0.89550	0.89835	0.89930	0.90238	0.90572	0.90941
789	0.88946	0.89560	0.89845	0.89940	0.90248	0.90581	0.90950
790	0.88956	0.89571	0.89855	0.89950	0.90257	0.90591	0.90958
791	0.88967	0.89581	0.89865	0.89960	0.90267	0.90600	0.90967
792	0.88977	0.89592	0.89875	0.89970	0.90276	0.90609	0.90976
793	0.88990	0.89602	0.89885	0.89980	0.90286	0.90618	0.90985
794	0.89000	0.89612	0.89896	0.89990	0.90296	0.90627	0.90994
795	0.89011	0.89623	0.89905	0.89999	0.90305	0.90636	0.91002
796	0.89022	0.89633	0.89915	0.90009	0.90315	0.90646	0.91011
797	0.89033	0.89643	0.89925	0.90019	0.90324	0.90655	0.91020
798	0.89042	0.89653	0.89935	0.90029	0.90334	0.90664	0.91028
799	0.89053	0.89663	0.89945	0.90039	0.90343	0.90673	0.91037
800	0.89064	0.89674	0.89955	0.90048	0.90352	0.90682	0.91046
801	0.89076	0.89684	0.89965	0.90058	0.90362	0.90691	0.91054
802	0.89086	0.89694	0.89975	0.90068	0.90372	0.90700	0.91063
803	0.89097	0.89704	0.89985	0.90078	0.90381	0.90709	0.91071
804	0.89107	0.89714	0.89995	0.90087	0.90390	0.90718	0.91080
805	0.89118	0.89724	0.90005	0.90097	0.90399	0.90727	0.91089
806	0.89129	0.89735	0.90015	0.90107	0.90409	0.90736	0.91097
807	0.89139	0.89745	0.90025	0.90116	0.90418	0.90745	0.91106
808	0.89150	0.89754	0.90034	0.90126	0.90427	0.90754	0.91114
809	0.89161	0.89765	0.90044	0.90136	0.90436	0.90763	0.91123
810	0.89171	0.89774	0.90054	0.90145	0.90446	0.90772	0.91131
811	0.89182	0.89784	0.90063	0.90155	0.90455	0.90780	0.91140
812	0.89193	0.89794	0.90073	0.90164	0.90465	0.90789	0.91148
813	0.89204	0.89804	0.90082	0.90174	0.90474	0.90798	0.91157
814	0.89216	0.89814	0.90092	0.90183	0.90483	0.90807	0.91165
815	0.89227	0.89824	0.90101	0.90192	0.90492	0.90816	0.91173
816	0.89238	0.89833	0.90110	0.90202	0.90501	0.90824	0.91182

N	0.1%	0.5%	1%	1.25%	2.5%	5%	10%
817	0.89248	0.89843	0.90120	0.90211	0.90510	0.90833	0.91190
818	0.89259	0.89853	0.90129	0.90221	0.90519	0.90841	0.91199
819	0.89270	0.89863	0.90139	0.90230	0.90528	0.90850	0.91207
820	0.89279	0.89873	0.90148	0.90239	0.90537	0.90859	0.91215
821	0.89290	0.89883	0.90157	0.90249	0.90546	0.90868	0.91223
822	0.89300	0.89893	0.90167	0.90258	0.90555	0.90876	0.91232
823	0.89309	0.89902	0.90176	0.90267	0.90564	0.90885	0.91240
824	0.89320	0.89912	0.90186	0.90277	0.90573	0.90894	0.91248
825	0.89330	0.89921	0.90195	0.90286	0.90582	0.90902	0.91256
826	0.89340	0.89931	0.90204	0.90295	0.90591	0.90911	0.91265
827	0.89350	0.89941	0.90214	0.90304	0.90600	0.90919	0.91273
828	0.89360	0.89950	0.90223	0.90314	0.90609	0.90928	0.91281
829	0.89370	0.89960	0.90233	0.90323	0.90618	0.90936	0.91289
830	0.89381	0.89969	0.90242	0.90332	0.90626	0.90945	0.91297
831	0.89393	0.89979	0.90251	0.90341	0.90635	0.90953	0.91305
832	0.89402	0.89988	0.90260	0.90350	0.90644	0.90962	0.91313
833	0.89411	0.89998	0.90269	0.90359	0.90653	0.90970	0.91321
834	0.89422	0.90007	0.90279	0.90368	0.90661	0.90978	0.91329
835	0.89432	0.90017	0.90288	0.90377	0.90670	0.90987	0.91338
836	0.89442	0.90026	0.90297	0.90387	0.90679	0.90996	0.91346
837	0.89453	0.90036	0.90306	0.90396	0.90687	0.91004	0.91353
838	0.89463	0.90045	0.90316	0.90405	0.90696	0.91012	0.91362
839	0.89473	0.90054	0.90325	0.90414	0.90705	0.91021	0.91370
840	0.89483	0.90064	0.90334	0.90423	0.90714	0.91029	0.91377
841	0.89492	0.90073	0.90343	0.90432	0.90722	0.91037	0.91385
842	0.89502	0.90082	0.90352	0.90441	0.90731	0.91045	0.91393
843	0.89514	0.90091	0.90361	0.90450	0.90739	0.91054	0.91401
844	0.89525	0.90101	0.90370	0.90459	0.90748	0.91062	0.91409
845	0.89534	0.90111	0.90379	0.90468	0.90756	0.91070	0.91417
846	0.89544	0.90119	0.90387	0.90477	0.90765	0.91078	0.91425
847	0.89554	0.90128	0.90396	0.90486	0.90773	0.91087	0.91433
848	0.89564	0.90138	0.90405	0.90494	0.90782	0.91095	0.91441
849	0.89573	0.90147	0.90414	0.90503	0.90791	0.91103	0.91448
850	0.89583	0.90156	0.90423	0.90512	0.90799	0.91111	0.91456
851	0.89593	0.90165	0.90432	0.90521	0.90808	0.91119	0.91464
852	0.89602	0.90174	0.90440	0.90530	0.90817	0.91127	0.91472
853	0.89612	0.90184	0.90449	0.90538	0.90825	0.91136	0.91480
854	0.89621	0.90193	0.90458	0.90547	0.90833	0.91144	0.91487
855	0.89632	0.90202	0.90466	0.90556	0.90842	0.91152	0.91495
856	0.89642	0.90211	0.90476	0.90564	0.90850	0.91160	0.91503
857	0.89651	0.90221	0.90484	0.90573	0.90858	0.91168	0.91510
858	0.89661	0.90230	0.90493	0.90581	0.90867	0.91176	0.91518
859	0.89670	0.90239	0.90502	0.90590	0.90875	0.91184	0.91526
860	0.89678	0.90248	0.90511	0.90599	0.90883	0.91192	0.91533
861	0.89688	0.90257	0.90520	0.90607	0.90892	0.91200	0.91541
862	0.89698	0.90266	0.90528	0.90616	0.90900	0.91208	0.91549
863	0.89707	0.90274	0.90537	0.90625	0.90908	0.91216	0.91556
864	0.89717	0.90283	0.90546	0.90633	0.90917	0.91224	0.91564
865	0.89726	0.90292	0.90554	0.90642	0.90925	0.91232	0.91571
866	0.89736	0.90302	0.90563	0.90650	0.90934	0.91240	0.91579
867	0.89745	0.90310	0.90572	0.90659	0.90942	0.91248	0.91586
868	0.89755	0.90320	0.90580	0.90668	0.90950	0.91256	0.91594
869	0.89764	0.90329	0.90589	0.90676	0.90958	0.91264	0.91602
870	0.89773	0.90337	0.90598	0.90684	0.90966	0.91272	0.91609
871	0.89782	0.90345	0.90606	0.90693	0.90974	0.91280	0.91617
872	0.89792	0.90355	0.90614	0.90701	0.90982	0.91287	0.91624
873	0.89801	0.90363	0.90623	0.90710	0.90991	0.91295	0.91631
874	0.89810	0.90372	0.90631	0.90718	0.90998	0.91303	0.91639
875	0.89820	0.90381	0.90640	0.90726	0.91007	0.91311	0.91646
876	0.89829	0.90389	0.90648	0.90735	0.91015	0.91319	0.91654
877	0.89839	0.90398	0.90656	0.90743	0.91023	0.91326	0.91661
878	0.89848	0.90407	0.90665	0.90751	0.91031	0.91334	0.91669
879	0.89857	0.90416	0.90673	0.90760	0.91039	0.91342	0.91676
880	0.89866	0.90424	0.90682	0.90768	0.91047	0.91350	0.91683
881	0.89875	0.90433	0.90690	0.90777	0.91055	0.91357	0.91691
882	0.89884	0.90441	0.90698	0.90785	0.91063	0.91365	0.91698
883	0.89893	0.90450	0.90707	0.90793	0.91071	0.91373	0.91705
884	0.89902	0.90459	0.90715	0.90801	0.91079	0.91380	0.91713
885	0.89913	0.90467	0.90724	0.90810	0.91087	0.91388	0.91720
886	0.89921	0.90476	0.90732	0.90818	0.91095	0.91395	0.91727
887	0.89931	0.90484	0.90740	0.90826	0.91103	0.91403	0.91734
888	0.89940	0.90493	0.90749	0.90835	0.91111	0.91411	0.91742
889	0.89949	0.90502	0.90757	0.90843	0.91119	0.91418	0.91749
890	0.89958	0.90511	0.90765	0.90851	0.91126	0.91426	0.91756

N	0.1%	0.5%	1%	1.25%	2.5%	5%	10%
891	0.89968	0.90519	0.90773	0.90859	0.91134	0.91433	0.91763
892	0.89976	0.90527	0.90781	0.90867	0.91142	0.91441	0.91770
893	0.89985	0.90536	0.90789	0.90875	0.91150	0.91448	0.91778
894	0.89992	0.90544	0.90797	0.90883	0.91158	0.91456	0.91785
895	0.90002	0.90552	0.90806	0.90891	0.91165	0.91463	0.91792
896	0.90011	0.90561	0.90814	0.90899	0.91173	0.91471	0.91799
897	0.90020	0.90569	0.90822	0.90907	0.91181	0.91478	0.91806
898	0.90029	0.90577	0.90830	0.90915	0.91189	0.91485	0.91813
899	0.90038	0.90586	0.90838	0.90923	0.91196	0.91493	0.91821
900	0.90047	0.90593	0.90846	0.90931	0.91204	0.91500	0.91828
901	0.90055	0.90602	0.90854	0.90939	0.91212	0.91508	0.91835
902	0.90064	0.90610	0.90862	0.90947	0.91220	0.91515	0.91842
903	0.90074	0.90618	0.90870	0.90955	0.91227	0.91522	0.91849
904	0.90083	0.90627	0.90878	0.90963	0.91235	0.91530	0.91856
905	0.90092	0.90635	0.90886	0.90971	0.91243	0.91537	0.91863
906	0.90101	0.90643	0.90894	0.90978	0.91250	0.91544	0.91870
907	0.90109	0.90651	0.90902	0.90986	0.91257	0.91552	0.91877
908	0.90117	0.90660	0.90910	0.90994	0.91265	0.91559	0.91884
909	0.90125	0.90668	0.90917	0.91002	0.91273	0.91566	0.91891
910	0.90133	0.90676	0.90925	0.91010	0.91280	0.91574	0.91898
911	0.90141	0.90685	0.90933	0.91018	0.91288	0.91581	0.91905
912	0.90150	0.90693	0.90941	0.91025	0.91295	0.91588	0.91911
913	0.90159	0.90701	0.90949	0.91033	0.91303	0.91595	0.91918
914	0.90167	0.90709	0.90957	0.91041	0.91311	0.91602	0.91925
915	0.90176	0.90717	0.90965	0.91049	0.91318	0.91609	0.91932
916	0.90184	0.90725	0.90973	0.91056	0.91326	0.91616	0.91939
917	0.90193	0.90733	0.90980	0.91064	0.91333	0.91624	0.91946
918	0.90201	0.90741	0.90988	0.91072	0.91340	0.91631	0.91952
919	0.90209	0.90748	0.90996	0.91079	0.91348	0.91638	0.91959
920	0.90218	0.90757	0.91004	0.91087	0.91355	0.91645	0.91966
921	0.90227	0.90765	0.91012	0.91095	0.91363	0.91652	0.91973
922	0.90236	0.90773	0.91019	0.91103	0.91370	0.91659	0.91980
923	0.90244	0.90781	0.91027	0.91110	0.91377	0.91666	0.91986
924	0.90252	0.90789	0.91035	0.91118	0.91384	0.91673	0.91993
925	0.90261	0.90796	0.91043	0.91125	0.91392	0.91681	0.92000
926	0.90270	0.90804	0.91050	0.91133	0.91399	0.91688	0.92007
927	0.90278	0.90812	0.91058	0.91140	0.91407	0.91695	0.92013
928	0.90286	0.90821	0.91066	0.91148	0.91414	0.91702	0.92020
929	0.90294	0.90828	0.91074	0.91156	0.91421	0.91709	0.92027
930	0.90302	0.90836	0.91082	0.91163	0.91428	0.91716	0.92033
931	0.90312	0.90845	0.91089	0.91171	0.91436	0.91723	0.92040
932	0.90320	0.90852	0.91097	0.91178	0.91443	0.91730	0.92047
933	0.90328	0.90860	0.91105	0.91185	0.91450	0.91737	0.92053
934	0.90336	0.90868	0.91112	0.91193	0.91457	0.91744	0.92060
935	0.90345	0.90876	0.91119	0.91200	0.91465	0.91751	0.92067
936	0.90353	0.90884	0.91127	0.91208	0.91472	0.91757	0.92073
937	0.90360	0.90891	0.91134	0.91215	0.91479	0.91764	0.92080
938	0.90368	0.90900	0.91142	0.91223	0.91486	0.91771	0.92087
939	0.90378	0.90907	0.91149	0.91230	0.91493	0.91778	0.92093
940	0.90386	0.90915	0.91157	0.91238	0.91500	0.91785	0.92100
941	0.90395	0.90923	0.91164	0.91245	0.91507	0.91792	0.92106
942	0.90402	0.90930	0.91172	0.91252	0.91515	0.91799	0.92113
943	0.90410	0.90938	0.91179	0.91260	0.91522	0.91805	0.92119
944	0.90418	0.90946	0.91186	0.91267	0.91529	0.91812	0.92126
945	0.90426	0.90953	0.91194	0.91275	0.91536	0.91819	0.92132
946	0.90434	0.90960	0.91201	0.91282	0.91543	0.91826	0.92139
947	0.90441	0.90968	0.91208	0.91289	0.91550	0.91833	0.92145
948	0.90450	0.90976	0.91215	0.91297	0.91557	0.91840	0.92152
949	0.90457	0.90983	0.91223	0.91304	0.91564	0.91846	0.92158
950	0.90465	0.90991	0.91230	0.91311	0.91571	0.91853	0.92165
951	0.90474	0.90999	0.91238	0.91318	0.91578	0.91860	0.92171
952	0.90482	0.91006	0.91245	0.91325	0.91585	0.91866	0.92178
953	0.90491	0.91013	0.91252	0.91332	0.91592	0.91873	0.92184
954	0.90499	0.91021	0.91260	0.91340	0.91599	0.91880	0.92190
955	0.90506	0.91029	0.91267	0.91347	0.91606	0.91886	0.92197
956	0.90514	0.91036	0.91274	0.91354	0.91613	0.91893	0.92203
957	0.90523	0.91043	0.91282	0.91361	0.91620	0.91900	0.92210
958	0.90531	0.91051	0.91289	0.91368	0.91627	0.91906	0.92216
959	0.90539	0.91058	0.91296	0.91376	0.91634	0.91913	0.92222
960	0.90546	0.91065	0.91304	0.91383	0.91641	0.91920	0.92229
961	0.90555	0.91073	0.91311	0.91390	0.91648	0.91926	0.92235
962	0.90563	0.91080	0.91318	0.91397	0.91655	0.91933	0.92241
963	0.90571	0.91087	0.91325	0.91404	0.91661	0.91939	0.92247
964	0.90579	0.91094	0.91332	0.91411	0.91668	0.91946	0.92254

N	0.1%	0.5%	1%	1.25%	2.5%	5%	10%
965	0.90587	0.91101	0.91339	0.91418	0.91675	0.91953	0.92260
966	0.90593	0.91109	0.91347	0.91425	0.91682	0.91959	0.92266
967	0.90602	0.91116	0.91354	0.91432	0.91688	0.91966	0.92272
968	0.90610	0.91124	0.91361	0.91439	0.91695	0.91972	0.92279
969	0.90618	0.91131	0.91368	0.91446	0.91702	0.91979	0.92285
970	0.90626	0.91138	0.91375	0.91453	0.91709	0.91985	0.92291
971	0.90636	0.91146	0.91382	0.91460	0.91716	0.91992	0.92297
972	0.90643	0.91153	0.91389	0.91467	0.91722	0.91998	0.92304
973	0.90649	0.91160	0.91396	0.91474	0.91729	0.92005	0.92310
974	0.90658	0.91168	0.91403	0.91481	0.91736	0.92011	0.92316
975	0.90666	0.91175	0.91410	0.91488	0.91742	0.92017	0.92322
976	0.90673	0.91182	0.91417	0.91494	0.91749	0.92024	0.92328
977	0.90680	0.91189	0.91424	0.91501	0.91755	0.92030	0.92334
978	0.90688	0.91196	0.91431	0.91508	0.91762	0.92036	0.92340
979	0.90695	0.91203	0.91438	0.91515	0.91769	0.92043	0.92346
980	0.90703	0.91211	0.91445	0.91522	0.91776	0.92049	0.92353
981	0.90711	0.91218	0.91452	0.91529	0.91782	0.92056	0.92359
982	0.90719	0.91225	0.91459	0.91536	0.91789	0.92062	0.92365
983	0.90727	0.91232	0.91466	0.91543	0.91796	0.92069	0.92371
984	0.90735	0.91239	0.91473	0.91550	0.91802	0.92075	0.92377
985	0.90743	0.91246	0.91479	0.91556	0.91809	0.92081	0.92383
986	0.90752	0.91253	0.91486	0.91563	0.91815	0.92088	0.92389
987	0.90758	0.91260	0.91493	0.91570	0.91822	0.92094	0.92395
988	0.90765	0.91267	0.91500	0.91577	0.91828	0.92100	0.92401
989	0.90772	0.91275	0.91507	0.91584	0.91835	0.92106	0.92407
990	0.90780	0.91282	0.91514	0.91590	0.91842	0.92113	0.92413
991	0.90788	0.91289	0.91520	0.91597	0.91848	0.92119	0.92419
992	0.90795	0.91296	0.91527	0.91604	0.91855	0.92125	0.92425
993	0.90802	0.91303	0.91534	0.91611	0.91861	0.92131	0.92431
994	0.90810	0.91310	0.91541	0.91618	0.91868	0.92138	0.92437
995	0.90817	0.91317	0.91548	0.91624	0.91874	0.92144	0.92443
996	0.90824	0.91324	0.91555	0.91631	0.91880	0.92150	0.92449
997	0.90831	0.91331	0.91561	0.91638	0.91887	0.92156	0.92455
998	0.90838	0.91338	0.91568	0.91644	0.91893	0.92163	0.92461
999	0.90846	0.91345	0.91575	0.91651	0.91900	0.92169	0.92466
1000	0.90854	0.91352	0.91582	0.91657	0.91906	0.92175	0.92472

Table 2 – *GM9(8H1L or 1H8L).* Critical values of upper significance levels for the Grubbs multiple-9 outlier test, applicable in situations where the eight highest and one lowest or one highest and eight lowest observations are simultaneously identified as outliers, denoted as *GM9(8H1L)* and *GM9(1H8L)*, respectively.

N	0.1%	0.5%	1%	1.25%	2.5%	5%	10%
11	0.00000	0.00000	0.00000	0.00000	0.00001	0.00002	0.00009
12	0.00001	0.00007	0.00015	0.00019	0.00039	0.00083	0.00180
13	0.00022	0.00066	0.00108	0.00127	0.00212	0.00360	0.00627
14	0.00095	0.00225	0.00331	0.00376	0.00560	0.00849	0.01318
15	0.00247	0.00505	0.00695	0.00772	0.01075	0.01520	0.02196
16	0.00491	0.00906	0.01191	0.01303	0.01733	0.02336	0.03214
17	0.00831	0.01418	0.01802	0.01949	0.02508	0.03267	0.04328
18	0.01263	0.02026	0.02509	0.02693	0.03375	0.04276	0.05506
19	0.01776	0.02718	0.03301	0.03517	0.04315	0.05346	0.06729
20	0.02349	0.03476	0.04149	0.04398	0.05302	0.06456	0.07977
21	0.02984	0.04288	0.05050	0.05330	0.06331	0.07590	0.09234
22	0.03669	0.05144	0.05985	0.06293	0.07382	0.08740	0.10490
23	0.04404	0.06022	0.06942	0.07277	0.08451	0.09895	0.11739
24	0.05169	0.06935	0.07921	0.08279	0.09520	0.11050	0.12976
25	0.05957	0.07864	0.08908	0.09282	0.10596	0.12198	0.14193
26	0.06768	0.08794	0.09897	0.10292	0.11670	0.13332	0.15387
27	0.07599	0.09737	0.10891	0.11305	0.12732	0.14447	0.16559
28	0.08440	0.10683	0.11879	0.12310	0.13789	0.15549	0.17707
29	0.09288	0.11612	0.12867	0.13306	0.14830	0.16632	0.18833
30	0.10140	0.12542	0.13832	0.14287	0.15852	0.17689	0.19928
31	0.10977	0.13477	0.14796	0.15263	0.16855	0.18732	0.21004
32	0.11823	0.14389	0.15745	0.16221	0.17849	0.19753	0.22046
33	0.12656	0.15304	0.16682	0.17171	0.18823	0.20750	0.23067
34	0.13493	0.16208	0.17612	0.18105	0.19779	0.21726	0.24062
35	0.14324	0.17086	0.18522	0.19025	0.20718	0.22684	0.25031
36	0.15163	0.17968	0.19423	0.19928	0.21637	0.23618	0.25975
37	0.15968	0.18827	0.20300	0.20812	0.22535	0.24529	0.26897
38	0.16797	0.19678	0.21164	0.21679	0.23418	0.25423	0.27791
39	0.17590	0.20508	0.22010	0.22530	0.24277	0.26293	0.28664

N	0.1%	0.5%	1%	1.25%	2.5%	5%	10%
40	0.18380	0.21333	0.22839	0.23363	0.25124	0.27144	0.29517
41	0.19158	0.22136	0.23659	0.24183	0.25951	0.27975	0.30347
42	0.19911	0.22932	0.24454	0.24986	0.26764	0.28785	0.31157
43	0.20681	0.23717	0.25241	0.25771	0.27554	0.29578	0.31950
44	0.21425	0.24479	0.26011	0.26541	0.28324	0.30351	0.32720
45	0.22153	0.25231	0.26763	0.27298	0.29080	0.31107	0.33472
46	0.22867	0.25952	0.27501	0.28037	0.29821	0.31848	0.34205
47	0.23574	0.26678	0.28222	0.28757	0.30547	0.32569	0.34922
48	0.24260	0.27389	0.28928	0.29464	0.31255	0.33274	0.35621
49	0.24958	0.28072	0.29628	0.30159	0.31946	0.33964	0.36306
50	0.25635	0.28756	0.30309	0.30843	0.32624	0.34641	0.36973
51	0.26303	0.29422	0.30988	0.31517	0.33290	0.35297	0.37623
52	0.26972	0.30081	0.31638	0.32168	0.33943	0.35945	0.38259
53	0.27594	0.30728	0.32276	0.32809	0.34583	0.36578	0.38880
54	0.28233	0.31358	0.32908	0.33434	0.35206	0.37196	0.39490
55	0.28848	0.31978	0.33525	0.34055	0.35818	0.37802	0.40084
56	0.29443	0.32590	0.34136	0.34665	0.36415	0.38397	0.40667
57	0.30038	0.33182	0.34731	0.35255	0.37014	0.38976	0.41234
58	0.30612	0.33783	0.35313	0.35842	0.37584	0.39546	0.41793
59	0.31200	0.34364	0.35887	0.36411	0.38149	0.40100	0.42341
60	0.31764	0.34919	0.36453	0.36973	0.38704	0.40645	0.42874
61	0.32322	0.35476	0.37003	0.37523	0.39248	0.41183	0.43399
62	0.32867	0.36019	0.37543	0.38063	0.39783	0.41707	0.43910
63	0.33417	0.36560	0.38079	0.38591	0.40308	0.42222	0.44413
64	0.33947	0.37080	0.38597	0.39107	0.40814	0.42725	0.44904
65	0.34489	0.37584	0.39107	0.39620	0.41317	0.43217	0.45385
66	0.34998	0.38096	0.39610	0.40123	0.41814	0.43699	0.45857
67	0.35524	0.38600	0.40105	0.40610	0.42298	0.44174	0.46319
68	0.36015	0.39096	0.40587	0.41096	0.42771	0.44640	0.46771
69	0.36516	0.39574	0.41066	0.41570	0.43238	0.45100	0.47217
70	0.37006	0.40053	0.41536	0.42042	0.43700	0.45550	0.47655
71	0.37471	0.40520	0.41997	0.42498	0.44151	0.45991	0.48085
72	0.37930	0.40974	0.42449	0.42946	0.44592	0.46426	0.48504
73	0.38385	0.41423	0.42894	0.43387	0.45030	0.46849	0.48920
74	0.38835	0.41868	0.43333	0.43826	0.45455	0.47265	0.49325
75	0.39281	0.42311	0.43760	0.44252	0.45878	0.47675	0.49723
76	0.39716	0.42741	0.44174	0.44664	0.46287	0.48079	0.50113
77	0.40137	0.43161	0.44594	0.45083	0.46693	0.48476	0.50499
78	0.40582	0.43579	0.45005	0.45492	0.47094	0.48866	0.50876
79	0.40985	0.43987	0.45407	0.45890	0.47484	0.49248	0.51246
80	0.41412	0.44382	0.45801	0.46283	0.47870	0.49622	0.51612
81	0.41815	0.44778	0.46190	0.46669	0.48248	0.49993	0.51970
82	0.42214	0.45165	0.46573	0.47047	0.48622	0.50358	0.52322
83	0.42612	0.45549	0.46955	0.47429	0.48990	0.50717	0.52668
84	0.43006	0.45928	0.47326	0.47798	0.49350	0.51070	0.53009
85	0.43401	0.46298	0.47687	0.48159	0.49708	0.51416	0.53345
86	0.43780	0.46663	0.48050	0.48521	0.50056	0.51757	0.53676
87	0.44146	0.47021	0.48403	0.48870	0.50400	0.52092	0.53999
88	0.44508	0.47379	0.48752	0.49214	0.50742	0.52425	0.54319
89	0.44884	0.47736	0.49096	0.49556	0.51075	0.52745	0.54634
90	0.45234	0.48080	0.49435	0.49895	0.51401	0.53068	0.54945
91	0.45591	0.48419	0.49768	0.50227	0.51726	0.53384	0.55249
92	0.45938	0.48747	0.50098	0.50551	0.52048	0.53694	0.55550
93	0.46283	0.49089	0.50424	0.50875	0.52366	0.54000	0.55845
94	0.46610	0.49414	0.50745	0.51195	0.52675	0.54300	0.56135
95	0.46940	0.49737	0.51061	0.51508	0.52980	0.54597	0.56422
96	0.47262	0.50054	0.51375	0.51820	0.53281	0.54889	0.56705
97	0.47594	0.50360	0.51680	0.52121	0.53577	0.55177	0.56983
98	0.47892	0.50668	0.51983	0.52421	0.53869	0.55461	0.57259
99	0.48211	0.50971	0.52283	0.52716	0.54155	0.55744	0.57527
100	0.48521	0.51274	0.52573	0.53010	0.54439	0.56019	0.57794
101	0.48836	0.51569	0.52864	0.53298	0.54722	0.56291	0.58056
102	0.49145	0.51867	0.53148	0.53584	0.54999	0.56559	0.58315
103	0.49438	0.52157	0.53430	0.53865	0.55271	0.56824	0.58570
104	0.49747	0.52436	0.53713	0.54140	0.55539	0.57084	0.58822
105	0.50037	0.52716	0.53988	0.54415	0.55805	0.57342	0.59072
106	0.50331	0.52996	0.54253	0.54682	0.56070	0.57596	0.59316
107	0.50613	0.53269	0.54525	0.54946	0.56329	0.57847	0.59558
108	0.50890	0.53541	0.54787	0.55210	0.56583	0.58095	0.59796
109	0.51172	0.53806	0.55046	0.55470	0.56832	0.58339	0.60029
110	0.51445	0.54066	0.55306	0.55727	0.57077	0.58580	0.60261
111	0.51725	0.54325	0.55559	0.55976	0.57324	0.58818	0.60490
112	0.51992	0.54582	0.55814	0.56223	0.57565	0.59053	0.60717
113	0.52248	0.54835	0.56062	0.56472	0.57806	0.59285	0.60940

N	0.1%	0.5%	1%	1.25%	2.5%	5%	10%
114	0.52510	0.55087	0.56300	0.56712	0.58045	0.59511	0.61160
115	0.52773	0.55333	0.56542	0.56948	0.58277	0.59737	0.61376
116	0.53024	0.55574	0.56777	0.57184	0.58508	0.59960	0.61590
117	0.53277	0.55814	0.57012	0.57419	0.58736	0.60180	0.61802
118	0.53529	0.56049	0.57248	0.57650	0.58958	0.60395	0.62011
119	0.53777	0.56284	0.57479	0.57874	0.59178	0.60611	0.62215
120	0.54020	0.56516	0.57707	0.58101	0.59399	0.60823	0.62420
121	0.54266	0.56744	0.57932	0.58327	0.59618	0.61031	0.62621
122	0.54496	0.56973	0.58155	0.58550	0.59831	0.61238	0.62821
123	0.54730	0.57202	0.58372	0.58764	0.60040	0.61442	0.63017
124	0.54967	0.57425	0.58589	0.58978	0.60250	0.61643	0.63211
125	0.55209	0.57645	0.58801	0.59190	0.60456	0.61844	0.63404
126	0.55421	0.57861	0.59011	0.59400	0.60660	0.62040	0.63595
127	0.55647	0.58073	0.59222	0.59608	0.60861	0.62236	0.63783
128	0.55881	0.58284	0.59429	0.59812	0.61060	0.62430	0.63967
129	0.56090	0.58494	0.59632	0.60015	0.61258	0.62622	0.64150
130	0.56300	0.58698	0.59831	0.60214	0.61453	0.62810	0.64331
131	0.56523	0.58905	0.60032	0.60411	0.61645	0.62995	0.64510
132	0.56745	0.59106	0.60229	0.60607	0.61834	0.63180	0.64687
133	0.56951	0.59311	0.60426	0.60801	0.62023	0.63361	0.64861
134	0.57157	0.59506	0.60617	0.60993	0.62209	0.63542	0.65034
135	0.57371	0.59698	0.60810	0.61180	0.62394	0.63721	0.65206
136	0.57573	0.59893	0.61003	0.61368	0.62575	0.63898	0.65374
137	0.57782	0.60088	0.61191	0.61555	0.62753	0.64070	0.65542
138	0.57975	0.60280	0.61370	0.61739	0.62934	0.64245	0.65707
139	0.58181	0.60465	0.61555	0.61921	0.63109	0.64415	0.65871
140	0.58378	0.60657	0.61735	0.62100	0.63285	0.64583	0.66031
141	0.58564	0.60836	0.61913	0.62279	0.63457	0.64751	0.66191
142	0.58751	0.61018	0.62091	0.62456	0.63629	0.64915	0.66349
143	0.58947	0.61200	0.62268	0.62631	0.63800	0.65079	0.66506
144	0.59131	0.61377	0.62444	0.62805	0.63966	0.65241	0.66661
145	0.59321	0.61557	0.62617	0.62973	0.64132	0.65400	0.66815
146	0.59512	0.61732	0.62787	0.63140	0.64295	0.65559	0.66967
147	0.59698	0.61904	0.62956	0.63312	0.64457	0.65716	0.67116
148	0.59875	0.62079	0.63124	0.63475	0.64617	0.65870	0.67265
149	0.60046	0.62247	0.63288	0.63638	0.64777	0.66023	0.67412
150	0.60230	0.62417	0.63453	0.63801	0.64933	0.66175	0.67558
151	0.60411	0.62583	0.63616	0.63962	0.65090	0.66326	0.67702
152	0.60580	0.62747	0.63778	0.64122	0.65245	0.66474	0.67846
153	0.60748	0.62915	0.63937	0.64279	0.65396	0.66623	0.67988
154	0.60910	0.63070	0.64092	0.64436	0.65547	0.66768	0.68128
155	0.61088	0.63231	0.64251	0.64590	0.65699	0.66912	0.68265
156	0.61262	0.63392	0.64405	0.64744	0.65845	0.67055	0.68402
157	0.61422	0.63547	0.64557	0.64896	0.65993	0.67196	0.68538
158	0.61585	0.63704	0.64705	0.65042	0.66138	0.67338	0.68674
159	0.61751	0.63860	0.64855	0.65189	0.66283	0.67477	0.68806
160	0.61915	0.64017	0.65004	0.65338	0.66426	0.67614	0.68938
161	0.62071	0.64165	0.65151	0.65484	0.66567	0.67751	0.69068
162	0.62223	0.64311	0.65296	0.65627	0.66708	0.67886	0.69197
163	0.62383	0.64463	0.65439	0.65769	0.66848	0.68019	0.69326
164	0.62536	0.64610	0.65581	0.65912	0.66985	0.68152	0.69453
165	0.62698	0.64753	0.65726	0.66054	0.67121	0.68284	0.69578
166	0.62854	0.64899	0.65866	0.66192	0.67257	0.68413	0.69704
167	0.62997	0.65041	0.66005	0.66330	0.67390	0.68543	0.69827
168	0.63149	0.65183	0.66143	0.66467	0.67523	0.68670	0.69949
169	0.63299	0.65323	0.66281	0.66601	0.67654	0.68797	0.70071
170	0.63443	0.65464	0.66417	0.66735	0.67785	0.68922	0.70191
171	0.63586	0.65601	0.66551	0.66870	0.67911	0.69047	0.70310
172	0.63732	0.65741	0.66685	0.67001	0.68038	0.69169	0.70428
173	0.63872	0.65876	0.66819	0.67131	0.68164	0.69292	0.70545
174	0.64016	0.66012	0.66946	0.67261	0.68289	0.69414	0.70662
175	0.64155	0.66143	0.67076	0.67390	0.68413	0.69533	0.70776
176	0.64291	0.66273	0.67205	0.67518	0.68536	0.69650	0.70889
177	0.64429	0.66407	0.67332	0.67645	0.68658	0.69768	0.71002
178	0.64563	0.66535	0.67458	0.67768	0.68780	0.69885	0.71115
179	0.64694	0.66667	0.67585	0.67893	0.68899	0.70003	0.71226
180	0.64835	0.66794	0.67708	0.68016	0.69021	0.70117	0.71336
181	0.64968	0.66916	0.67830	0.68138	0.69138	0.70230	0.71445
182	0.65102	0.67042	0.67953	0.68261	0.69255	0.70343	0.71553
183	0.65231	0.67168	0.68073	0.68380	0.69372	0.70453	0.71660
184	0.65363	0.67289	0.68194	0.68497	0.69487	0.70563	0.71766
185	0.65493	0.67411	0.68312	0.68615	0.69600	0.70674	0.71872
186	0.65618	0.67533	0.68430	0.68733	0.69715	0.70782	0.71976
187	0.65746	0.67649	0.68547	0.68848	0.69827	0.70891	0.72079

N	0.1%	0.5%	1%	1.25%	2.5%	5%	10%
188	0.65871	0.67766	0.68662	0.68962	0.69938	0.70998	0.72182
189	0.65995	0.67886	0.68779	0.69077	0.70051	0.71104	0.72283
190	0.66123	0.68004	0.68892	0.69190	0.70159	0.71209	0.72384
191	0.66243	0.68123	0.69005	0.69301	0.70268	0.71314	0.72484
192	0.66361	0.68235	0.69118	0.69413	0.70375	0.71418	0.72583
193	0.66483	0.68350	0.69228	0.69523	0.70480	0.71522	0.72681
194	0.66603	0.68461	0.69338	0.69632	0.70587	0.71623	0.72779
195	0.66729	0.68573	0.69446	0.69741	0.70691	0.71724	0.72876
196	0.66843	0.68686	0.69556	0.69851	0.70796	0.71824	0.72972
197	0.66959	0.68794	0.69663	0.69957	0.70899	0.71923	0.73068
198	0.67076	0.68906	0.69770	0.70060	0.71000	0.72022	0.73162
199	0.67196	0.69014	0.69877	0.70167	0.71102	0.72121	0.73257
200	0.67313	0.69124	0.69983	0.70269	0.71205	0.72219	0.73349
201	0.67424	0.69230	0.70089	0.70374	0.71306	0.72315	0.73442
202	0.67540	0.69336	0.70191	0.70476	0.71406	0.72410	0.73533
203	0.67652	0.69442	0.70294	0.70578	0.71503	0.72506	0.73624
204	0.67755	0.69546	0.70396	0.70679	0.71600	0.72601	0.73714
205	0.67862	0.69650	0.70498	0.70778	0.71696	0.72695	0.73803
206	0.67973	0.69753	0.70598	0.70876	0.71793	0.72787	0.73893
207	0.68077	0.69857	0.70697	0.70976	0.71891	0.72879	0.73980
208	0.68184	0.69960	0.70797	0.71075	0.71984	0.72971	0.74068
209	0.68294	0.70064	0.70896	0.71173	0.72077	0.73062	0.74155
210	0.68399	0.70163	0.70993	0.71267	0.72170	0.73152	0.74241
211	0.68504	0.70263	0.71091	0.71363	0.72263	0.73242	0.74327
212	0.68608	0.70361	0.71187	0.71460	0.72355	0.73331	0.74412
213	0.68709	0.70458	0.71281	0.71554	0.72446	0.73418	0.74496
214	0.68815	0.70558	0.71376	0.71648	0.72536	0.73505	0.74579
215	0.68921	0.70654	0.71469	0.71741	0.72627	0.73591	0.74662
216	0.69020	0.70750	0.71562	0.71831	0.72717	0.73678	0.74745
217	0.69122	0.70844	0.71654	0.71924	0.72806	0.73763	0.74827
218	0.69223	0.70939	0.71747	0.72015	0.72895	0.73847	0.74908
219	0.69324	0.71034	0.71836	0.72106	0.72983	0.73932	0.74988
220	0.69423	0.71125	0.71927	0.72197	0.73070	0.74016	0.75068
221	0.69511	0.71220	0.72019	0.72284	0.73156	0.74100	0.75148
222	0.69607	0.71313	0.72109	0.72374	0.73241	0.74181	0.75227
223	0.69704	0.71403	0.72198	0.72463	0.73326	0.74263	0.75305
224	0.69804	0.71494	0.72284	0.72549	0.73410	0.74344	0.75382
225	0.69903	0.71584	0.72371	0.72636	0.73494	0.74424	0.75460
226	0.69999	0.71672	0.72457	0.72722	0.73579	0.74505	0.75537
227	0.70088	0.71760	0.72543	0.72805	0.73660	0.74585	0.75613
228	0.70177	0.71848	0.72629	0.72890	0.73741	0.74664	0.75689
229	0.70279	0.71937	0.72715	0.72973	0.73822	0.74743	0.75763
230	0.70370	0.72023	0.72799	0.73058	0.73904	0.74821	0.75838
231	0.70465	0.72111	0.72885	0.73141	0.73984	0.74899	0.75912
232	0.70553	0.72196	0.72968	0.73223	0.74064	0.74975	0.75986
233	0.70644	0.72282	0.73049	0.73304	0.74144	0.75052	0.76059
234	0.70730	0.72365	0.73132	0.73386	0.74222	0.75127	0.76131
235	0.70818	0.72449	0.73211	0.73468	0.74300	0.75202	0.76203
236	0.70913	0.72531	0.73295	0.73547	0.74378	0.75277	0.76275
237	0.71001	0.72613	0.73374	0.73627	0.74456	0.75351	0.76346
238	0.71089	0.72695	0.73454	0.73706	0.74533	0.75425	0.76416
239	0.71172	0.72778	0.73535	0.73785	0.74610	0.75498	0.76487
240	0.71259	0.72861	0.73613	0.73863	0.74684	0.75571	0.76556
241	0.71346	0.72940	0.73691	0.73943	0.74759	0.75643	0.76626
242	0.71437	0.73018	0.73769	0.74021	0.74834	0.75715	0.76694
243	0.71517	0.73101	0.73847	0.74097	0.74906	0.75786	0.76762
244	0.71600	0.73179	0.73925	0.74172	0.74980	0.75858	0.76830
245	0.71682	0.73257	0.74002	0.74249	0.75053	0.75928	0.76897
246	0.71765	0.73335	0.74077	0.74324	0.75126	0.75998	0.76964
247	0.71848	0.73413	0.74153	0.74399	0.75199	0.76068	0.77031
248	0.71924	0.73490	0.74227	0.74473	0.75271	0.76137	0.77097
249	0.72002	0.73564	0.74303	0.74547	0.75342	0.76205	0.77162
250	0.72085	0.73643	0.74376	0.74619	0.75413	0.76272	0.77227
251	0.72164	0.73716	0.74449	0.74692	0.75483	0.76341	0.77293
252	0.72244	0.73793	0.74522	0.74765	0.75553	0.76408	0.77357
253	0.72324	0.73867	0.74595	0.74836	0.75622	0.76474	0.77421
254	0.72403	0.73940	0.74667	0.74907	0.75690	0.76541	0.77485
255	0.72479	0.74014	0.74738	0.74977	0.75759	0.76607	0.77547
256	0.72558	0.74091	0.74810	0.75048	0.75827	0.76673	0.77610
257	0.72637	0.74162	0.74881	0.75119	0.75895	0.76737	0.77673
258	0.72714	0.74234	0.74949	0.75187	0.75963	0.76802	0.77735
259	0.72795	0.74305	0.75019	0.75256	0.76029	0.76867	0.77796
260	0.72869	0.74377	0.75088	0.75324	0.76095	0.76931	0.77857
261	0.72946	0.74447	0.75156	0.75392	0.76161	0.76995	0.77918

N	0.1%	0.5%	1%	1.25%	2.5%	5%	10%
262	0.73025	0.74519	0.75225	0.75460	0.76226	0.77058	0.77978
263	0.73097	0.74589	0.75293	0.75527	0.76292	0.77120	0.78038
264	0.73167	0.74660	0.75361	0.75596	0.76357	0.77182	0.78097
265	0.73245	0.74726	0.75426	0.75661	0.76421	0.77245	0.78157
266	0.73321	0.74796	0.75493	0.75727	0.76484	0.77306	0.78216
267	0.73389	0.74865	0.75558	0.75792	0.76548	0.77367	0.78275
268	0.73462	0.74934	0.75627	0.75858	0.76611	0.77428	0.78333
269	0.73530	0.75001	0.75691	0.75921	0.76675	0.77489	0.78391
270	0.73607	0.75069	0.75757	0.75987	0.76738	0.77549	0.78449
271	0.73671	0.75137	0.75821	0.76050	0.76800	0.77609	0.78506
272	0.73745	0.75203	0.75884	0.76114	0.76861	0.77669	0.78563
273	0.73813	0.75269	0.75949	0.76177	0.76923	0.77727	0.78620
274	0.73886	0.75334	0.76013	0.76240	0.76984	0.77786	0.78676
275	0.73954	0.75400	0.76077	0.76303	0.77044	0.77845	0.78732
276	0.74021	0.75464	0.76140	0.76365	0.77104	0.77902	0.78787
277	0.74090	0.75529	0.76204	0.76429	0.77164	0.77960	0.78843
278	0.74160	0.75594	0.76266	0.76490	0.77223	0.78017	0.78897
279	0.74226	0.75658	0.76328	0.76552	0.77283	0.78074	0.78952
280	0.74296	0.75721	0.76390	0.76614	0.77343	0.78131	0.79006
281	0.74366	0.75787	0.76452	0.76674	0.77401	0.78187	0.79060
282	0.74435	0.75849	0.76513	0.76736	0.77459	0.78243	0.79114
283	0.74497	0.75910	0.76574	0.76795	0.77517	0.78299	0.79167
284	0.74561	0.75975	0.76634	0.76856	0.77575	0.78355	0.79220
285	0.74628	0.76034	0.76694	0.76915	0.77632	0.78410	0.79273
286	0.74693	0.76100	0.76754	0.76973	0.77689	0.78464	0.79325
287	0.74761	0.76160	0.76812	0.77032	0.77746	0.78519	0.79377
288	0.74823	0.76221	0.76871	0.77090	0.77802	0.78573	0.79429
289	0.74888	0.76280	0.76930	0.77148	0.77858	0.78627	0.79481
290	0.74954	0.76340	0.76988	0.77205	0.77915	0.78681	0.79532
291	0.75017	0.76398	0.77046	0.77264	0.77969	0.78734	0.79583
292	0.75079	0.76458	0.77104	0.77322	0.78024	0.78786	0.79633
293	0.75144	0.76516	0.77161	0.77379	0.78079	0.78840	0.79684
294	0.75206	0.76575	0.77218	0.77435	0.78133	0.78892	0.79734
295	0.75269	0.76633	0.77275	0.77492	0.78187	0.78944	0.79784
296	0.75327	0.76693	0.77332	0.77547	0.78241	0.78997	0.79833
297	0.75386	0.76750	0.77388	0.77600	0.78294	0.79048	0.79882
298	0.75446	0.76806	0.77444	0.77656	0.78348	0.79099	0.79931
299	0.75508	0.76863	0.77499	0.77711	0.78400	0.79150	0.79980
300	0.75570	0.76921	0.77554	0.77765	0.78454	0.79200	0.80029
301	0.75631	0.76977	0.77609	0.77820	0.78506	0.79251	0.80077
302	0.75691	0.77032	0.77662	0.77873	0.78558	0.79300	0.80125
303	0.75746	0.77087	0.77715	0.77926	0.78609	0.79350	0.80173
304	0.75808	0.77142	0.77768	0.77978	0.78661	0.79400	0.80220
305	0.75862	0.77197	0.77823	0.78031	0.78712	0.79450	0.80267
306	0.75916	0.77252	0.77877	0.78086	0.78763	0.79499	0.80315
307	0.75979	0.77307	0.77930	0.78137	0.78814	0.79547	0.80361
308	0.76035	0.77360	0.77982	0.78190	0.78865	0.79595	0.80407
309	0.76091	0.77416	0.78034	0.78242	0.78915	0.79644	0.80453
310	0.76145	0.77470	0.78085	0.78294	0.78964	0.79692	0.80500
311	0.76202	0.77523	0.78136	0.78345	0.79014	0.79739	0.80545
312	0.76258	0.77578	0.78189	0.78396	0.79064	0.79787	0.80591
313	0.76318	0.77629	0.78240	0.78445	0.79113	0.79835	0.80635
314	0.76375	0.77683	0.78290	0.78496	0.79162	0.79882	0.80681
315	0.76431	0.77736	0.78343	0.78546	0.79211	0.79929	0.80726
316	0.76485	0.77787	0.78392	0.78596	0.79259	0.79976	0.80770
317	0.76539	0.77840	0.78441	0.78646	0.79307	0.80022	0.80815
318	0.76593	0.77891	0.78492	0.78696	0.79354	0.80068	0.80859
319	0.76652	0.77941	0.78542	0.78745	0.79402	0.80114	0.80903
320	0.76705	0.77993	0.78592	0.78794	0.79450	0.80160	0.80946
321	0.76761	0.78042	0.78641	0.78843	0.79497	0.80206	0.80990
322	0.76814	0.78092	0.78690	0.78892	0.79544	0.80251	0.81033
323	0.76868	0.78143	0.78740	0.78940	0.79591	0.80296	0.81076
324	0.76921	0.78194	0.78788	0.78987	0.79638	0.80340	0.81119
325	0.76975	0.78244	0.78835	0.79035	0.79684	0.80385	0.81161
326	0.77028	0.78293	0.78883	0.79083	0.79729	0.80429	0.81203
327	0.77080	0.78343	0.78931	0.79130	0.79774	0.80473	0.81245
328	0.77133	0.78391	0.78979	0.79178	0.79820	0.80516	0.81287
329	0.77182	0.78440	0.79026	0.79225	0.79865	0.80560	0.81328
330	0.77233	0.78487	0.79073	0.79271	0.79911	0.80603	0.81370
331	0.77287	0.78534	0.79119	0.79318	0.79956	0.80647	0.81411
332	0.77332	0.78583	0.79166	0.79364	0.80000	0.80690	0.81452
333	0.77382	0.78631	0.79213	0.79410	0.80046	0.80733	0.81493
334	0.77433	0.78678	0.79261	0.79456	0.80090	0.80775	0.81534
335	0.77485	0.78726	0.79307	0.79501	0.80133	0.80817	0.81574

N	0.1%	0.5%	1%	1.25%	2.5%	5%	10%
336	0.77537	0.78773	0.79352	0.79547	0.80177	0.80860	0.81614
337	0.77589	0.78820	0.79398	0.79591	0.80220	0.80901	0.81655
338	0.77636	0.78866	0.79443	0.79637	0.80263	0.80944	0.81695
339	0.77682	0.78912	0.79488	0.79681	0.80306	0.80985	0.81734
340	0.77735	0.78958	0.79533	0.79726	0.80349	0.81026	0.81773
341	0.77778	0.79005	0.79578	0.79770	0.80392	0.81067	0.81813
342	0.77827	0.79051	0.79622	0.79814	0.80434	0.81108	0.81852
343	0.77873	0.79097	0.79667	0.79857	0.80476	0.81148	0.81891
344	0.77920	0.79142	0.79711	0.79901	0.80518	0.81189	0.81930
345	0.77969	0.79188	0.79755	0.79944	0.80560	0.81230	0.81968
346	0.78018	0.79233	0.79800	0.79988	0.80601	0.81269	0.82006
347	0.78068	0.79278	0.79842	0.80030	0.80644	0.81309	0.82044
348	0.78115	0.79322	0.79886	0.80073	0.80684	0.81349	0.82082
349	0.78163	0.79367	0.79929	0.80116	0.80726	0.81388	0.82120
350	0.78211	0.79411	0.79972	0.80159	0.80767	0.81427	0.82157
351	0.78258	0.79455	0.80015	0.80201	0.80808	0.81467	0.82195
352	0.78302	0.79497	0.80058	0.80244	0.80849	0.81506	0.82232
353	0.78345	0.79540	0.80099	0.80285	0.80890	0.81545	0.82269
354	0.78392	0.79585	0.80142	0.80328	0.80929	0.81584	0.82306
355	0.78442	0.79626	0.80185	0.80368	0.80969	0.81622	0.82343
356	0.78485	0.79669	0.80226	0.80410	0.81009	0.81660	0.82380
357	0.78530	0.79712	0.80268	0.80450	0.81049	0.81698	0.82417
358	0.78572	0.79755	0.80309	0.80492	0.81088	0.81736	0.82453
359	0.78622	0.79797	0.80349	0.80532	0.81126	0.81774	0.82489
360	0.78665	0.79841	0.80390	0.80573	0.81165	0.81812	0.82525
361	0.78706	0.79882	0.80432	0.80614	0.81204	0.81849	0.82560
362	0.78753	0.79925	0.80472	0.80654	0.81243	0.81886	0.82596
363	0.78794	0.79964	0.80512	0.80693	0.81282	0.81923	0.82631
364	0.78837	0.80005	0.80552	0.80733	0.81320	0.81960	0.82667
365	0.78881	0.80046	0.80590	0.80772	0.81358	0.81996	0.82702
366	0.78925	0.80087	0.80630	0.80812	0.81396	0.82033	0.82737
367	0.78970	0.80127	0.80670	0.80850	0.81434	0.82069	0.82772
368	0.79013	0.80167	0.80709	0.80889	0.81471	0.82106	0.82806
369	0.79055	0.80207	0.80749	0.80928	0.81509	0.82141	0.82841
370	0.79097	0.80247	0.80788	0.80966	0.81547	0.82177	0.82875
371	0.79142	0.80286	0.80827	0.81005	0.81584	0.82213	0.82910
372	0.79184	0.80327	0.80865	0.81044	0.81620	0.82249	0.82944
373	0.79224	0.80366	0.80903	0.81081	0.81657	0.82284	0.82978
374	0.79266	0.80406	0.80941	0.81119	0.81694	0.82320	0.83012
375	0.79311	0.80445	0.80979	0.81157	0.81731	0.82355	0.83045
376	0.79353	0.80486	0.81018	0.81194	0.81767	0.82390	0.83079
377	0.79393	0.80524	0.81056	0.81232	0.81804	0.82424	0.83112
378	0.79434	0.80562	0.81094	0.81269	0.81840	0.82458	0.83145
379	0.79470	0.80600	0.81130	0.81305	0.81876	0.82494	0.83178
380	0.79510	0.80638	0.81169	0.81342	0.81912	0.82528	0.83211
381	0.79554	0.80678	0.81206	0.81379	0.81947	0.82562	0.83244
382	0.79598	0.80717	0.81243	0.81415	0.81982	0.82596	0.83276
383	0.79632	0.80754	0.81279	0.81452	0.82017	0.82630	0.83309
384	0.79674	0.80792	0.81315	0.81489	0.82053	0.82664	0.83341
385	0.79710	0.80830	0.81352	0.81525	0.82087	0.82698	0.83373
386	0.79753	0.80867	0.81388	0.81561	0.82122	0.82731	0.83405
387	0.79792	0.80905	0.81424	0.81596	0.82156	0.82764	0.83437
388	0.79831	0.80941	0.81460	0.81631	0.82191	0.82797	0.83468
389	0.79869	0.80977	0.81496	0.81668	0.82225	0.82830	0.83500
390	0.79906	0.81016	0.81532	0.81704	0.82260	0.82863	0.83532
391	0.79945	0.81053	0.81568	0.81740	0.82294	0.82896	0.83563
392	0.79985	0.81090	0.81603	0.81774	0.82328	0.82929	0.83594
393	0.80024	0.81126	0.81638	0.81808	0.82361	0.82961	0.83625
394	0.80063	0.81161	0.81674	0.81844	0.82394	0.82993	0.83656
395	0.80100	0.81196	0.81709	0.81878	0.82428	0.83025	0.83687
396	0.80142	0.81234	0.81744	0.81913	0.82461	0.83057	0.83718
397	0.80177	0.81269	0.81779	0.81947	0.82494	0.83089	0.83748
398	0.80212	0.81305	0.81813	0.81981	0.82527	0.83121	0.83778
399	0.80250	0.81340	0.81847	0.82015	0.82560	0.83153	0.83809
400	0.80288	0.81375	0.81881	0.82049	0.82593	0.83184	0.83839
401	0.80325	0.81411	0.81915	0.82082	0.82626	0.83216	0.83869
402	0.80360	0.81446	0.81950	0.82117	0.82659	0.83247	0.83899
403	0.80399	0.81480	0.81984	0.82151	0.82691	0.83278	0.83928
404	0.80435	0.81515	0.82017	0.82184	0.82724	0.83309	0.83958
405	0.80471	0.81550	0.82051	0.82217	0.82756	0.83340	0.83988
406	0.80510	0.81585	0.82084	0.82250	0.82788	0.83371	0.84017
407	0.80546	0.81619	0.82118	0.82283	0.82820	0.83401	0.84046
408	0.80584	0.81654	0.82151	0.82316	0.82852	0.83432	0.84076
409	0.80618	0.81687	0.82184	0.82348	0.82884	0.83462	0.84105

N	0.1%	0.5%	1%	1.25%	2.5%	5%	10%
410	0.80652	0.81722	0.82217	0.82380	0.82915	0.83492	0.84134
411	0.80693	0.81754	0.82249	0.82412	0.82946	0.83522	0.84163
412	0.80728	0.81787	0.82282	0.82444	0.82978	0.83553	0.84191
413	0.80763	0.81819	0.82315	0.82476	0.83009	0.83583	0.84220
414	0.80798	0.81852	0.82347	0.82509	0.83040	0.83613	0.84249
415	0.80834	0.81887	0.82379	0.82540	0.83071	0.83643	0.84277
416	0.80869	0.81920	0.82411	0.82572	0.83102	0.83672	0.84306
417	0.80902	0.81952	0.82443	0.82604	0.83132	0.83701	0.84334
418	0.80937	0.81985	0.82476	0.82636	0.83162	0.83731	0.84362
419	0.80974	0.82018	0.82507	0.82667	0.83193	0.83760	0.84389
420	0.81007	0.82051	0.82538	0.82699	0.83223	0.83789	0.84418
421	0.81043	0.82083	0.82570	0.82730	0.83252	0.83818	0.84445
422	0.81078	0.82115	0.82601	0.82761	0.83282	0.83846	0.84473
423	0.81112	0.82148	0.82632	0.82792	0.83312	0.83875	0.84500
424	0.81146	0.82180	0.82663	0.82824	0.83342	0.83904	0.84527
425	0.81181	0.82211	0.82694	0.82854	0.83372	0.83933	0.84555
426	0.81215	0.82242	0.82725	0.82885	0.83401	0.83961	0.84582
427	0.81248	0.82274	0.82755	0.82915	0.83431	0.83989	0.84609
428	0.81281	0.82305	0.82786	0.82945	0.83459	0.84018	0.84636
429	0.81317	0.82336	0.82816	0.82976	0.83488	0.84046	0.84663
430	0.81347	0.82369	0.82847	0.83005	0.83518	0.84074	0.84690
431	0.81382	0.82399	0.82877	0.83035	0.83546	0.84102	0.84716
432	0.81417	0.82430	0.82907	0.83064	0.83575	0.84129	0.84742
433	0.81448	0.82461	0.82937	0.83095	0.83604	0.84156	0.84769
434	0.81480	0.82492	0.82966	0.83124	0.83632	0.84184	0.84795
435	0.81513	0.82522	0.82997	0.83153	0.83661	0.84212	0.84821
436	0.81544	0.82552	0.83025	0.83182	0.83689	0.84239	0.84847
437	0.81579	0.82582	0.83055	0.83212	0.83717	0.84267	0.84873
438	0.81611	0.82612	0.83085	0.83240	0.83745	0.84294	0.84899
439	0.81641	0.82643	0.83114	0.83269	0.83774	0.84320	0.84925
440	0.81675	0.82673	0.83143	0.83298	0.83802	0.84348	0.84951
441	0.81704	0.82702	0.83172	0.83327	0.83829	0.84374	0.84976
442	0.81734	0.82732	0.83201	0.83355	0.83857	0.84401	0.85002
443	0.81768	0.82762	0.83230	0.83383	0.83885	0.84427	0.85027
444	0.81799	0.82792	0.83258	0.83412	0.83912	0.84454	0.85052
445	0.81831	0.82822	0.83286	0.83439	0.83940	0.84480	0.85077
446	0.81862	0.82852	0.83315	0.83468	0.83967	0.84507	0.85103
447	0.81892	0.82881	0.83345	0.83497	0.83994	0.84533	0.85128
448	0.81927	0.82912	0.83372	0.83525	0.84021	0.84559	0.85153
449	0.81958	0.82940	0.83400	0.83552	0.84048	0.84585	0.85178
450	0.81987	0.82969	0.83428	0.83580	0.84075	0.84610	0.85202
451	0.82019	0.82996	0.83456	0.83608	0.84102	0.84636	0.85227
452	0.82047	0.83026	0.83484	0.83636	0.84129	0.84662	0.85252
453	0.82078	0.83054	0.83513	0.83663	0.84155	0.84688	0.85276
454	0.82107	0.83082	0.83539	0.83690	0.84182	0.84713	0.85300
455	0.82138	0.83110	0.83568	0.83718	0.84209	0.84738	0.85325
456	0.82169	0.83140	0.83595	0.83745	0.84234	0.84763	0.85349
457	0.82200	0.83168	0.83622	0.83772	0.84261	0.84789	0.85373
458	0.82230	0.83196	0.83650	0.83800	0.84287	0.84814	0.85397
459	0.82258	0.83223	0.83676	0.83827	0.84313	0.84839	0.85421
460	0.82289	0.83252	0.83703	0.83854	0.84339	0.84864	0.85445
461	0.82319	0.83278	0.83731	0.83881	0.84365	0.84889	0.85469
462	0.82349	0.83307	0.83758	0.83908	0.84391	0.84913	0.85493
463	0.82376	0.83334	0.83785	0.83935	0.84416	0.84938	0.85516
464	0.82406	0.83361	0.83812	0.83961	0.84442	0.84963	0.85540
465	0.82435	0.83389	0.83838	0.83988	0.84468	0.84988	0.85563
466	0.82465	0.83416	0.83864	0.84013	0.84493	0.85012	0.85587
467	0.82493	0.83444	0.83890	0.84040	0.84519	0.85036	0.85610
468	0.82521	0.83471	0.83917	0.84066	0.84544	0.85061	0.85633
469	0.82549	0.83498	0.83943	0.84092	0.84569	0.85085	0.85657
470	0.82577	0.83524	0.83969	0.84118	0.84593	0.85109	0.85679
471	0.82606	0.83552	0.83996	0.84143	0.84618	0.85133	0.85702
472	0.82636	0.83577	0.84021	0.84168	0.84643	0.85156	0.85725
473	0.82663	0.83604	0.84047	0.84194	0.84668	0.85180	0.85748
474	0.82693	0.83630	0.84072	0.84219	0.84692	0.85204	0.85771
475	0.82716	0.83657	0.84097	0.84245	0.84717	0.85228	0.85794
476	0.82746	0.83683	0.84123	0.84270	0.84741	0.85251	0.85816
477	0.82775	0.83708	0.84148	0.84295	0.84766	0.85275	0.85839
478	0.82805	0.83735	0.84174	0.84320	0.84790	0.85298	0.85861
479	0.82833	0.83762	0.84200	0.84346	0.84814	0.85322	0.85884
480	0.82860	0.83788	0.84225	0.84370	0.84838	0.85345	0.85906
481	0.82886	0.83814	0.84250	0.84394	0.84862	0.85368	0.85928
482	0.82917	0.83840	0.84276	0.84420	0.84887	0.85391	0.85950
483	0.82944	0.83865	0.84301	0.84445	0.84910	0.85414	0.85972

N	0.1%	0.5%	1%	1.25%	2.5%	5%	10%
484	0.82969	0.83891	0.84325	0.84469	0.84934	0.85437	0.85994
485	0.82996	0.83916	0.84350	0.84494	0.84958	0.85460	0.86015
486	0.83023	0.83941	0.84375	0.84519	0.84982	0.85482	0.86037
487	0.83051	0.83966	0.84399	0.84543	0.85005	0.85505	0.86059
488	0.83078	0.83992	0.84424	0.84566	0.85029	0.85527	0.86080
489	0.83105	0.84017	0.84448	0.84591	0.85052	0.85550	0.86102
490	0.83131	0.84042	0.84472	0.84615	0.85075	0.85572	0.86123
491	0.83156	0.84068	0.84497	0.84639	0.85098	0.85595	0.86145
492	0.83183	0.84094	0.84520	0.84662	0.85122	0.85617	0.86166
493	0.83209	0.84118	0.84544	0.84685	0.85144	0.85639	0.86188
494	0.83235	0.84143	0.84569	0.84710	0.85168	0.85661	0.86209
495	0.83262	0.84167	0.84593	0.84733	0.85191	0.85684	0.86230
496	0.83290	0.84191	0.84617	0.84758	0.85214	0.85706	0.86251
497	0.83315	0.84216	0.84640	0.84781	0.85237	0.85728	0.86271
498	0.83342	0.84240	0.84663	0.84805	0.85260	0.85749	0.86292
499	0.83369	0.84266	0.84688	0.84828	0.85282	0.85771	0.86313
500	0.83393	0.84290	0.84710	0.84851	0.85304	0.85793	0.86334
501	0.83418	0.84315	0.84734	0.84874	0.85327	0.85814	0.86354
502	0.83445	0.84337	0.84758	0.84898	0.85350	0.85836	0.86375
503	0.83470	0.84362	0.84781	0.84920	0.85372	0.85857	0.86396
504	0.83496	0.84387	0.84805	0.84943	0.85394	0.85878	0.86416
505	0.83522	0.84409	0.84828	0.84967	0.85416	0.85900	0.86437
506	0.83547	0.84433	0.84851	0.84989	0.85438	0.85921	0.86457
507	0.83573	0.84457	0.84874	0.85012	0.85460	0.85942	0.86477
508	0.83596	0.84480	0.84896	0.85034	0.85482	0.85964	0.86497
509	0.83622	0.84504	0.84919	0.85058	0.85504	0.85985	0.86518
510	0.83647	0.84528	0.84942	0.85080	0.85526	0.86006	0.86538
511	0.83673	0.84551	0.84964	0.85103	0.85547	0.86027	0.86558
512	0.83699	0.84574	0.84987	0.85126	0.85569	0.86048	0.86578
513	0.83724	0.84598	0.85010	0.85147	0.85591	0.86068	0.86597
514	0.83748	0.84621	0.85033	0.85170	0.85612	0.86089	0.86617
515	0.83773	0.84643	0.85055	0.85192	0.85634	0.86110	0.86637
516	0.83798	0.84666	0.85077	0.85214	0.85655	0.86130	0.86657
517	0.83820	0.84689	0.85100	0.85236	0.85676	0.86151	0.86677
518	0.83843	0.84711	0.85122	0.85257	0.85698	0.86171	0.86696
519	0.83867	0.84734	0.85144	0.85279	0.85719	0.86192	0.86716
520	0.83892	0.84756	0.85166	0.85301	0.85740	0.86212	0.86735
521	0.83915	0.84779	0.85188	0.85322	0.85761	0.86232	0.86754
522	0.83942	0.84803	0.85210	0.85344	0.85782	0.86252	0.86774
523	0.83967	0.84825	0.85232	0.85365	0.85803	0.86273	0.86793
524	0.83988	0.84848	0.85254	0.85387	0.85823	0.86292	0.86812
525	0.84015	0.84871	0.85275	0.85409	0.85844	0.86312	0.86831
526	0.84037	0.84894	0.85296	0.85430	0.85865	0.86332	0.86850
527	0.84059	0.84915	0.85317	0.85452	0.85885	0.86352	0.86870
528	0.84083	0.84937	0.85338	0.85472	0.85905	0.86372	0.86888
529	0.84107	0.84959	0.85359	0.85493	0.85926	0.86392	0.86907
530	0.84132	0.84982	0.85380	0.85514	0.85947	0.86411	0.86926
531	0.84154	0.85004	0.85402	0.85535	0.85967	0.86431	0.86945
532	0.84177	0.85025	0.85423	0.85556	0.85987	0.86450	0.86963
533	0.84201	0.85047	0.85445	0.85577	0.86007	0.86469	0.86982
534	0.84223	0.85070	0.85466	0.85598	0.86028	0.86489	0.87000
535	0.84245	0.85091	0.85486	0.85618	0.86047	0.86508	0.87019
536	0.84268	0.85113	0.85508	0.85639	0.86067	0.86527	0.87037
537	0.84290	0.85135	0.85529	0.85660	0.86087	0.86547	0.87056
538	0.84311	0.85156	0.85550	0.85680	0.86107	0.86566	0.87074
539	0.84333	0.85178	0.85570	0.85701	0.86128	0.86585	0.87092
540	0.84356	0.85199	0.85590	0.85722	0.86148	0.86604	0.87111
541	0.84376	0.85221	0.85610	0.85742	0.86167	0.86623	0.87129
542	0.84399	0.85242	0.85631	0.85763	0.86187	0.86642	0.87147
543	0.84421	0.85263	0.85651	0.85783	0.86206	0.86661	0.87165
544	0.84442	0.85284	0.85672	0.85803	0.86226	0.86679	0.87183
545	0.84467	0.85305	0.85692	0.85823	0.86245	0.86698	0.87201
546	0.84488	0.85326	0.85712	0.85843	0.86264	0.86717	0.87219
547	0.84510	0.85348	0.85732	0.85863	0.86284	0.86736	0.87237
548	0.84535	0.85368	0.85751	0.85883	0.86303	0.86754	0.87255
549	0.84556	0.85388	0.85772	0.85902	0.86322	0.86772	0.87273
550	0.84578	0.85409	0.85793	0.85922	0.86341	0.86791	0.87290
551	0.84598	0.85430	0.85813	0.85941	0.86360	0.86809	0.87308
552	0.84619	0.85450	0.85833	0.85962	0.86379	0.86828	0.87325
553	0.84641	0.85471	0.85853	0.85981	0.86398	0.86846	0.87343
554	0.84664	0.85491	0.85873	0.86001	0.86417	0.86864	0.87361
555	0.84685	0.85511	0.85893	0.86020	0.86436	0.86882	0.87378
556	0.84707	0.85531	0.85912	0.86040	0.86454	0.86900	0.87395
557	0.84729	0.85552	0.85932	0.86060	0.86473	0.86918	0.87413

N	0.1%	0.5%	1%	1.25%	2.5%	5%	10%
558	0.84751	0.85572	0.85952	0.86079	0.86492	0.86936	0.87430
559	0.84773	0.85593	0.85970	0.86098	0.86510	0.86955	0.87447
560	0.84795	0.85613	0.85990	0.86116	0.86529	0.86973	0.87464
561	0.84815	0.85633	0.86009	0.86135	0.86548	0.86991	0.87481
562	0.84837	0.85654	0.86029	0.86155	0.86566	0.87008	0.87498
563	0.84859	0.85673	0.86048	0.86174	0.86584	0.87026	0.87516
564	0.84878	0.85693	0.86067	0.86193	0.86602	0.87044	0.87532
565	0.84899	0.85714	0.86086	0.86213	0.86621	0.87062	0.87549
566	0.84921	0.85733	0.86106	0.86231	0.86639	0.87079	0.87566
567	0.84943	0.85752	0.86125	0.86250	0.86657	0.87097	0.87583
568	0.84963	0.85772	0.86144	0.86268	0.86675	0.87114	0.87599
569	0.84983	0.85791	0.86163	0.86287	0.86694	0.87132	0.87616
570	0.85004	0.85811	0.86181	0.86306	0.86711	0.87149	0.87633
571	0.85023	0.85830	0.86200	0.86324	0.86730	0.87166	0.87649
572	0.85042	0.85850	0.86219	0.86343	0.86748	0.87184	0.87666
573	0.85062	0.85868	0.86237	0.86362	0.86766	0.87201	0.87682
574	0.85082	0.85887	0.86256	0.86380	0.86783	0.87218	0.87698
575	0.85102	0.85906	0.86275	0.86398	0.86801	0.87235	0.87715
576	0.85121	0.85925	0.86294	0.86417	0.86819	0.87252	0.87731
577	0.85142	0.85944	0.86312	0.86435	0.86837	0.87269	0.87747
578	0.85163	0.85963	0.86331	0.86453	0.86854	0.87286	0.87764
579	0.85185	0.85981	0.86350	0.86472	0.86871	0.87303	0.87780
580	0.85205	0.86000	0.86368	0.86489	0.86889	0.87320	0.87796
581	0.85223	0.86020	0.86386	0.86507	0.86906	0.87337	0.87812
582	0.85244	0.86039	0.86404	0.86525	0.86924	0.87353	0.87828
583	0.85263	0.86057	0.86422	0.86543	0.86941	0.87370	0.87844
584	0.85283	0.86075	0.86440	0.86561	0.86958	0.87387	0.87860
585	0.85303	0.86094	0.86457	0.86579	0.86975	0.87403	0.87876
586	0.85323	0.86111	0.86476	0.86596	0.86992	0.87420	0.87892
587	0.85342	0.86130	0.86493	0.86614	0.87010	0.87436	0.87908
588	0.85362	0.86149	0.86511	0.86632	0.87027	0.87453	0.87923
589	0.85382	0.86168	0.86529	0.86650	0.87044	0.87469	0.87939
590	0.85400	0.86186	0.86547	0.86668	0.87061	0.87485	0.87955
591	0.85418	0.86204	0.86564	0.86686	0.87078	0.87502	0.87971
592	0.85438	0.86222	0.86582	0.86703	0.87095	0.87518	0.87986
593	0.85459	0.86240	0.86600	0.86720	0.87112	0.87534	0.88002
594	0.85478	0.86259	0.86617	0.86738	0.87129	0.87551	0.88017
595	0.85496	0.86276	0.86635	0.86755	0.87146	0.87567	0.88033
596	0.85515	0.86294	0.86653	0.86773	0.87162	0.87583	0.88048
597	0.85534	0.86313	0.86671	0.86790	0.87179	0.87599	0.88063
598	0.85553	0.86331	0.86688	0.86807	0.87195	0.87615	0.88079
599	0.85572	0.86348	0.86705	0.86825	0.87212	0.87631	0.88094
600	0.85591	0.86366	0.86723	0.86842	0.87228	0.87647	0.88109
601	0.85609	0.86384	0.86740	0.86859	0.87245	0.87662	0.88124
602	0.85628	0.86402	0.86757	0.86876	0.87261	0.87678	0.88139
603	0.85646	0.86419	0.86774	0.86893	0.87278	0.87694	0.88155
604	0.85666	0.86437	0.86792	0.86910	0.87294	0.87710	0.88170
605	0.85682	0.86454	0.86808	0.86927	0.87311	0.87725	0.88185
606	0.85700	0.86472	0.86825	0.86944	0.87327	0.87741	0.88200
607	0.85719	0.86489	0.86842	0.86960	0.87343	0.87756	0.88215
608	0.85737	0.86506	0.86859	0.86977	0.87359	0.87772	0.88230
609	0.85755	0.86524	0.86876	0.86994	0.87375	0.87787	0.88245
610	0.85774	0.86541	0.86893	0.87010	0.87391	0.87803	0.88259
611	0.85792	0.86558	0.86909	0.87027	0.87407	0.87818	0.88274
612	0.85810	0.86575	0.86926	0.87044	0.87423	0.87834	0.88289
613	0.85829	0.86593	0.86943	0.87060	0.87439	0.87849	0.88303
614	0.85847	0.86610	0.86959	0.87077	0.87455	0.87865	0.88318
615	0.85864	0.86626	0.86976	0.87093	0.87470	0.87880	0.88333
616	0.85883	0.86643	0.86992	0.87109	0.87487	0.87895	0.88347
617	0.85901	0.86660	0.87008	0.87125	0.87503	0.87910	0.88362
618	0.85921	0.86677	0.87025	0.87142	0.87519	0.87926	0.88376
619	0.85939	0.86695	0.87041	0.87159	0.87534	0.87941	0.88391
620	0.85957	0.86712	0.87058	0.87175	0.87549	0.87956	0.88405
621	0.85976	0.86728	0.87074	0.87191	0.87565	0.87971	0.88419
622	0.85993	0.86745	0.87090	0.87207	0.87580	0.87986	0.88434
623	0.86012	0.86762	0.87106	0.87223	0.87595	0.88001	0.88448
624	0.86029	0.86778	0.87122	0.87239	0.87611	0.88015	0.88462
625	0.86046	0.86794	0.87139	0.87255	0.87626	0.88030	0.88476
626	0.86062	0.86811	0.87155	0.87271	0.87641	0.88045	0.88491
627	0.86081	0.86827	0.87171	0.87287	0.87657	0.88060	0.88505
628	0.86098	0.86844	0.87187	0.87302	0.87672	0.88075	0.88519
629	0.86116	0.86861	0.87203	0.87318	0.87688	0.88090	0.88533
630	0.86133	0.86877	0.87218	0.87334	0.87703	0.88104	0.88547
631	0.86152	0.86894	0.87234	0.87349	0.87718	0.88119	0.88561

N	0.1%	0.5%	1%	1.25%	2.5%	5%	10%
632	0.86170	0.86909	0.87250	0.87365	0.87733	0.88133	0.88575
633	0.86186	0.86925	0.87265	0.87380	0.87748	0.88148	0.88589
634	0.86202	0.86941	0.87281	0.87395	0.87763	0.88162	0.88602
635	0.86220	0.86957	0.87297	0.87411	0.87778	0.88176	0.88616
636	0.86237	0.86973	0.87312	0.87426	0.87794	0.88191	0.88630
637	0.86254	0.86989	0.87328	0.87441	0.87808	0.88205	0.88644
638	0.86272	0.87005	0.87343	0.87457	0.87823	0.88220	0.88658
639	0.86288	0.87022	0.87359	0.87472	0.87838	0.88234	0.88671
640	0.86304	0.87038	0.87374	0.87488	0.87853	0.88248	0.88685
641	0.86321	0.87054	0.87389	0.87503	0.87868	0.88262	0.88699
642	0.86337	0.87069	0.87404	0.87518	0.87883	0.88277	0.88712
643	0.86353	0.87084	0.87420	0.87533	0.87897	0.88290	0.88726
644	0.86369	0.87100	0.87436	0.87548	0.87912	0.88304	0.88739
645	0.86384	0.87116	0.87450	0.87563	0.87926	0.88319	0.88752
646	0.86400	0.87131	0.87466	0.87578	0.87941	0.88333	0.88766
647	0.86418	0.87147	0.87481	0.87593	0.87955	0.88347	0.88779
648	0.86435	0.87162	0.87496	0.87608	0.87970	0.88360	0.88793
649	0.86452	0.87178	0.87511	0.87623	0.87984	0.88374	0.88806
650	0.86469	0.87193	0.87526	0.87638	0.87999	0.88388	0.88819
651	0.86485	0.87209	0.87542	0.87653	0.88013	0.88402	0.88833
652	0.86502	0.87224	0.87557	0.87667	0.88028	0.88416	0.88846
653	0.86520	0.87239	0.87572	0.87682	0.88042	0.88430	0.88859
654	0.86535	0.87254	0.87587	0.87697	0.88056	0.88444	0.88873
655	0.86553	0.87269	0.87601	0.87711	0.88070	0.88457	0.88886
656	0.86569	0.87285	0.87616	0.87726	0.88084	0.88471	0.88899
657	0.86585	0.87300	0.87631	0.87741	0.88098	0.88485	0.88912
658	0.86600	0.87316	0.87646	0.87755	0.88112	0.88498	0.88925
659	0.86616	0.87331	0.87661	0.87770	0.88126	0.88512	0.88938
660	0.86632	0.87346	0.87675	0.87784	0.88140	0.88525	0.88951
661	0.86648	0.87361	0.87690	0.87799	0.88154	0.88539	0.88963
662	0.86665	0.87375	0.87704	0.87813	0.88168	0.88553	0.88976
663	0.86680	0.87390	0.87719	0.87827	0.88182	0.88566	0.88989
664	0.86696	0.87405	0.87733	0.87842	0.88196	0.88579	0.89002
665	0.86711	0.87421	0.87747	0.87857	0.88210	0.88593	0.89015
666	0.86726	0.87435	0.87762	0.87871	0.88224	0.88606	0.89028
667	0.86743	0.87450	0.87776	0.87885	0.88238	0.88619	0.89040
668	0.86758	0.87464	0.87790	0.87899	0.88251	0.88632	0.89053
669	0.86775	0.87479	0.87805	0.87914	0.88265	0.88646	0.89066
670	0.86791	0.87493	0.87819	0.87928	0.88278	0.88659	0.89078
671	0.86806	0.87508	0.87833	0.87942	0.88292	0.88672	0.89091
672	0.86820	0.87523	0.87848	0.87956	0.88305	0.88684	0.89104
673	0.86834	0.87537	0.87862	0.87970	0.88319	0.88698	0.89116
674	0.86850	0.87552	0.87877	0.87984	0.88332	0.88711	0.89128
675	0.86866	0.87566	0.87890	0.87998	0.88346	0.88723	0.89141
676	0.86882	0.87581	0.87904	0.88012	0.88359	0.88737	0.89153
677	0.86897	0.87596	0.87918	0.88026	0.88373	0.88750	0.89166
678	0.86914	0.87610	0.87932	0.88040	0.88386	0.88763	0.89178
679	0.86928	0.87626	0.87946	0.88054	0.88400	0.88775	0.89190
680	0.86942	0.87639	0.87960	0.88067	0.88413	0.88788	0.89203
681	0.86958	0.87653	0.87974	0.88081	0.88426	0.88801	0.89215
682	0.86973	0.87668	0.87987	0.88095	0.88439	0.88814	0.89227
683	0.86990	0.87682	0.88001	0.88108	0.88453	0.88827	0.89239
684	0.87005	0.87696	0.88015	0.88122	0.88466	0.88839	0.89251
685	0.87019	0.87711	0.88029	0.88135	0.88479	0.88852	0.89264
686	0.87034	0.87724	0.88042	0.88149	0.88492	0.88864	0.89276
687	0.87050	0.87739	0.88056	0.88162	0.88505	0.88877	0.89288
688	0.87066	0.87752	0.88070	0.88176	0.88518	0.88890	0.89300
689	0.87080	0.87767	0.88083	0.88189	0.88531	0.88902	0.89312
690	0.87095	0.87780	0.88096	0.88202	0.88544	0.88915	0.89324
691	0.87111	0.87794	0.88110	0.88216	0.88557	0.88927	0.89336
692	0.87125	0.87808	0.88124	0.88229	0.88570	0.88940	0.89348
693	0.87141	0.87822	0.88137	0.88243	0.88583	0.88952	0.89360
694	0.87155	0.87836	0.88150	0.88256	0.88596	0.88965	0.89372
695	0.87171	0.87850	0.88164	0.88269	0.88609	0.88977	0.89384
696	0.87186	0.87863	0.88177	0.88282	0.88622	0.88989	0.89395
697	0.87200	0.87877	0.88191	0.88296	0.88635	0.89002	0.89407
698	0.87215	0.87890	0.88204	0.88309	0.88647	0.89014	0.89419
699	0.87230	0.87904	0.88217	0.88322	0.88659	0.89026	0.89431
700	0.87245	0.87918	0.88231	0.88335	0.88672	0.89038	0.89443
701	0.87259	0.87932	0.88244	0.88348	0.88685	0.89050	0.89454
702	0.87273	0.87945	0.88257	0.88361	0.88697	0.89062	0.89466
703	0.87287	0.87959	0.88270	0.88374	0.88710	0.89075	0.89477
704	0.87302	0.87973	0.88283	0.88388	0.88723	0.89087	0.89489
705	0.87317	0.87986	0.88296	0.88401	0.88735	0.89099	0.89500

N	0.1%	0.5%	1%	1.25%	2.5%	5%	10%
706	0.87331	0.87999	0.88309	0.88414	0.88748	0.89111	0.89512
707	0.87345	0.88013	0.88322	0.88427	0.88760	0.89123	0.89523
708	0.87360	0.88026	0.88335	0.88439	0.88773	0.89135	0.89535
709	0.87373	0.88040	0.88348	0.88453	0.88786	0.89147	0.89546
710	0.87387	0.88053	0.88361	0.88465	0.88798	0.89159	0.89557
711	0.87401	0.88067	0.88374	0.88477	0.88810	0.89171	0.89569
712	0.87415	0.88080	0.88386	0.88490	0.88822	0.89183	0.89580
713	0.87428	0.88093	0.88399	0.88503	0.88834	0.89194	0.89592
714	0.87442	0.88106	0.88411	0.88515	0.88847	0.89206	0.89603
715	0.87456	0.88119	0.88424	0.88528	0.88859	0.89218	0.89614
716	0.87469	0.88132	0.88437	0.88541	0.88871	0.89230	0.89626
717	0.87485	0.88145	0.88449	0.88553	0.88883	0.89242	0.89637
718	0.87498	0.88159	0.88462	0.88565	0.88896	0.89253	0.89648
719	0.87511	0.88171	0.88475	0.88577	0.88908	0.89265	0.89659
720	0.87525	0.88184	0.88488	0.88590	0.88919	0.89277	0.89670
721	0.87540	0.88198	0.88501	0.88603	0.88932	0.89288	0.89682
722	0.87553	0.88210	0.88513	0.88615	0.88944	0.89300	0.89693
723	0.87564	0.88223	0.88526	0.88627	0.88955	0.89311	0.89704
724	0.87578	0.88236	0.88539	0.88640	0.88968	0.89323	0.89715
725	0.87591	0.88249	0.88551	0.88652	0.88980	0.89334	0.89726
726	0.87605	0.88262	0.88563	0.88664	0.88992	0.89346	0.89737
727	0.87618	0.88275	0.88576	0.88676	0.89004	0.89357	0.89748
728	0.87634	0.88288	0.88588	0.88689	0.89015	0.89369	0.89759
729	0.87647	0.88301	0.88601	0.88701	0.89027	0.89380	0.89769
730	0.87661	0.88313	0.88613	0.88713	0.89039	0.89391	0.89780
731	0.87676	0.88326	0.88625	0.88726	0.89051	0.89403	0.89791
732	0.87688	0.88339	0.88638	0.88738	0.89063	0.89414	0.89802
733	0.87702	0.88352	0.88650	0.88750	0.89074	0.89425	0.89813
734	0.87714	0.88364	0.88662	0.88762	0.89086	0.89436	0.89824
735	0.87727	0.88377	0.88675	0.88774	0.89098	0.89448	0.89835
736	0.87741	0.88389	0.88687	0.88786	0.89109	0.89459	0.89845
737	0.87753	0.88402	0.88699	0.88799	0.89121	0.89470	0.89856
738	0.87765	0.88414	0.88711	0.88810	0.89133	0.89481	0.89867
739	0.87778	0.88426	0.88723	0.88822	0.89144	0.89492	0.89878
740	0.87791	0.88439	0.88735	0.88834	0.89155	0.89503	0.89888
741	0.87806	0.88451	0.88748	0.88845	0.89167	0.89514	0.89899
742	0.87819	0.88464	0.88759	0.88857	0.89179	0.89525	0.89909
743	0.87832	0.88476	0.88771	0.88870	0.89190	0.89536	0.89920
744	0.87845	0.88488	0.88783	0.88881	0.89201	0.89548	0.89930
745	0.87858	0.88500	0.88796	0.88893	0.89213	0.89559	0.89941
746	0.87870	0.88513	0.88808	0.88905	0.89224	0.89570	0.89951
747	0.87883	0.88525	0.88820	0.88917	0.89235	0.89580	0.89962
748	0.87896	0.88537	0.88832	0.88929	0.89247	0.89591	0.89972
749	0.87910	0.88549	0.88843	0.88940	0.89258	0.89602	0.89983
750	0.87923	0.88560	0.88854	0.88953	0.89269	0.89613	0.89993
751	0.87935	0.88573	0.88866	0.88964	0.89281	0.89624	0.90004
752	0.87949	0.88585	0.88878	0.88976	0.89292	0.89634	0.90014
753	0.87962	0.88597	0.88890	0.88987	0.89303	0.89645	0.90024
754	0.87974	0.88609	0.88901	0.88998	0.89314	0.89656	0.90034
755	0.87986	0.88621	0.88913	0.89010	0.89325	0.89667	0.90045
756	0.88000	0.88632	0.88924	0.89022	0.89336	0.89677	0.90055
757	0.88014	0.88644	0.88936	0.89033	0.89347	0.89688	0.90065
758	0.88026	0.88656	0.88947	0.89045	0.89358	0.89699	0.90076
759	0.88038	0.88668	0.88959	0.89056	0.89369	0.89710	0.90086
760	0.88052	0.88679	0.88970	0.89067	0.89380	0.89720	0.90096
761	0.88065	0.88691	0.88982	0.89078	0.89391	0.89731	0.90106
762	0.88077	0.88703	0.88994	0.89090	0.89402	0.89742	0.90116
763	0.88089	0.88714	0.89005	0.89101	0.89413	0.89752	0.90126
764	0.88101	0.88726	0.89016	0.89112	0.89424	0.89763	0.90136
765	0.88113	0.88737	0.89027	0.89123	0.89435	0.89773	0.90146
766	0.88126	0.88749	0.89038	0.89134	0.89446	0.89783	0.90156
767	0.88138	0.88761	0.89049	0.89146	0.89457	0.89794	0.90166
768	0.88151	0.88773	0.89061	0.89157	0.89468	0.89804	0.90176
769	0.88164	0.88784	0.89072	0.89168	0.89479	0.89815	0.90186
770	0.88175	0.88796	0.89083	0.89179	0.89489	0.89825	0.90196
771	0.88187	0.88807	0.89094	0.89190	0.89500	0.89835	0.90206
772	0.88199	0.88819	0.89105	0.89201	0.89511	0.89846	0.90216
773	0.88212	0.88831	0.89116	0.89212	0.89521	0.89856	0.90226
774	0.88225	0.88842	0.89127	0.89223	0.89532	0.89866	0.90236
775	0.88238	0.88854	0.89139	0.89234	0.89543	0.89876	0.90246
776	0.88248	0.88865	0.89150	0.89245	0.89554	0.89886	0.90255
777	0.88261	0.88877	0.89161	0.89256	0.89564	0.89896	0.90265
778	0.88273	0.88888	0.89172	0.89267	0.89574	0.89907	0.90275
779	0.88286	0.88899	0.89183	0.89278	0.89585	0.89917	0.90285

N	0.1%	0.5%	1%	1.25%	2.5%	5%	10%
780	0.88298	0.88910	0.89194	0.89289	0.89595	0.89927	0.90294
781	0.88309	0.88922	0.89205	0.89300	0.89606	0.89937	0.90304
782	0.88322	0.88932	0.89216	0.89311	0.89616	0.89947	0.90314
783	0.88334	0.88944	0.89227	0.89321	0.89627	0.89957	0.90324
784	0.88345	0.88955	0.89237	0.89332	0.89637	0.89967	0.90333
785	0.88357	0.88966	0.89248	0.89343	0.89647	0.89977	0.90343
786	0.88367	0.88977	0.89259	0.89354	0.89658	0.89987	0.90352
787	0.88380	0.88988	0.89270	0.89364	0.89668	0.89997	0.90362
788	0.88391	0.88999	0.89280	0.89375	0.89678	0.90007	0.90371
789	0.88403	0.89011	0.89291	0.89386	0.89689	0.90017	0.90381
790	0.88415	0.89022	0.89302	0.89396	0.89699	0.90027	0.90390
791	0.88426	0.89032	0.89313	0.89407	0.89709	0.90037	0.90400
792	0.88437	0.89044	0.89323	0.89417	0.89719	0.90047	0.90409
793	0.88449	0.89055	0.89334	0.89428	0.89730	0.90057	0.90419
794	0.88460	0.89066	0.89344	0.89439	0.89740	0.90067	0.90428
795	0.88472	0.89077	0.89355	0.89449	0.89750	0.90077	0.90438
796	0.88484	0.89087	0.89366	0.89460	0.89760	0.90086	0.90447
797	0.88496	0.89098	0.89376	0.89470	0.89771	0.90096	0.90456
798	0.88506	0.89109	0.89387	0.89480	0.89781	0.90106	0.90466
799	0.88517	0.89119	0.89398	0.89491	0.89790	0.90115	0.90475
800	0.88528	0.89130	0.89408	0.89501	0.89800	0.90125	0.90484
801	0.88539	0.89141	0.89419	0.89511	0.89810	0.90135	0.90494
802	0.88550	0.89152	0.89429	0.89522	0.89820	0.90145	0.90503
803	0.88562	0.89162	0.89439	0.89532	0.89831	0.90154	0.90512
804	0.88573	0.89174	0.89450	0.89542	0.89841	0.90164	0.90521
805	0.88586	0.89184	0.89460	0.89553	0.89851	0.90173	0.90530
806	0.88597	0.89194	0.89470	0.89563	0.89860	0.90183	0.90540
807	0.88608	0.89205	0.89480	0.89573	0.89870	0.90192	0.90549
808	0.88621	0.89215	0.89491	0.89583	0.89880	0.90202	0.90558
809	0.88632	0.89226	0.89501	0.89594	0.89890	0.90212	0.90567
810	0.88645	0.89237	0.89512	0.89604	0.89900	0.90221	0.90576
811	0.88654	0.89248	0.89522	0.89614	0.89910	0.90231	0.90585
812	0.88665	0.89258	0.89532	0.89624	0.89920	0.90240	0.90594
813	0.88676	0.89269	0.89542	0.89634	0.89929	0.90249	0.90603
814	0.88687	0.89279	0.89553	0.89644	0.89939	0.90259	0.90612
815	0.88699	0.89289	0.89563	0.89654	0.89949	0.90268	0.90621
816	0.88709	0.89300	0.89573	0.89664	0.89959	0.90277	0.90630
817	0.88720	0.89310	0.89583	0.89674	0.89968	0.90287	0.90639
818	0.88732	0.89321	0.89593	0.89684	0.89978	0.90296	0.90648
819	0.88743	0.89331	0.89604	0.89694	0.89988	0.90305	0.90657
820	0.88754	0.89341	0.89614	0.89704	0.89997	0.90314	0.90666
821	0.88766	0.89352	0.89624	0.89714	0.90007	0.90323	0.90675
822	0.88776	0.89362	0.89634	0.89724	0.90016	0.90333	0.90684
823	0.88787	0.89372	0.89644	0.89734	0.90026	0.90342	0.90693
824	0.88799	0.89382	0.89654	0.89744	0.90036	0.90351	0.90702
825	0.88810	0.89394	0.89664	0.89754	0.90045	0.90361	0.90710
826	0.88820	0.89403	0.89674	0.89764	0.90055	0.90370	0.90719
827	0.88830	0.89414	0.89684	0.89774	0.90064	0.90379	0.90728
828	0.88840	0.89424	0.89694	0.89783	0.90073	0.90388	0.90737
829	0.88851	0.89435	0.89703	0.89793	0.90083	0.90397	0.90746
830	0.88863	0.89445	0.89714	0.89803	0.90092	0.90406	0.90754
831	0.88874	0.89454	0.89723	0.89813	0.90102	0.90415	0.90763
832	0.88884	0.89464	0.89733	0.89823	0.90111	0.90424	0.90772
833	0.88895	0.89474	0.89742	0.89832	0.90121	0.90434	0.90780
834	0.88904	0.89484	0.89752	0.89842	0.90130	0.90443	0.90789
835	0.88915	0.89495	0.89762	0.89851	0.90139	0.90452	0.90798
836	0.88927	0.89505	0.89771	0.89861	0.90149	0.90461	0.90806
837	0.88938	0.89515	0.89781	0.89871	0.90158	0.90470	0.90815
838	0.88949	0.89525	0.89791	0.89881	0.90167	0.90479	0.90823
839	0.88960	0.89535	0.89801	0.89890	0.90177	0.90488	0.90832
840	0.88971	0.89545	0.89810	0.89900	0.90186	0.90497	0.90840
841	0.88981	0.89554	0.89820	0.89909	0.90195	0.90506	0.90849
842	0.88990	0.89564	0.89830	0.89919	0.90204	0.90515	0.90857
843	0.89002	0.89574	0.89839	0.89928	0.90214	0.90524	0.90866
844	0.89013	0.89584	0.89849	0.89937	0.90223	0.90532	0.90874
845	0.89023	0.89594	0.89858	0.89947	0.90232	0.90541	0.90883
846	0.89033	0.89604	0.89868	0.89956	0.90241	0.90550	0.90891
847	0.89042	0.89613	0.89877	0.89966	0.90250	0.90559	0.90900
848	0.89052	0.89623	0.89886	0.89975	0.90260	0.90567	0.90908
849	0.89062	0.89633	0.89896	0.89984	0.90269	0.90576	0.90917
850	0.89071	0.89642	0.89905	0.89994	0.90278	0.90585	0.90925
851	0.89082	0.89652	0.89915	0.90003	0.90287	0.90594	0.90933
852	0.89092	0.89662	0.89925	0.90012	0.90296	0.90602	0.90942
853	0.89102	0.89671	0.89934	0.90022	0.90305	0.90611	0.90950

N	0.1%	0.5%	1%	1.25%	2.5%	5%	10%
854	0.89112	0.89681	0.89944	0.90031	0.90314	0.90620	0.90958
855	0.89123	0.89691	0.89953	0.90041	0.90323	0.90629	0.90967
856	0.89133	0.89700	0.89962	0.90050	0.90331	0.90637	0.90975
857	0.89145	0.89710	0.89971	0.90059	0.90341	0.90646	0.90983
858	0.89155	0.89720	0.89981	0.90068	0.90349	0.90654	0.90991
859	0.89164	0.89730	0.89990	0.90078	0.90359	0.90663	0.91000
860	0.89174	0.89739	0.90000	0.90086	0.90367	0.90672	0.91008
861	0.89184	0.89749	0.90009	0.90096	0.90376	0.90680	0.91016
862	0.89194	0.89758	0.90018	0.90105	0.90385	0.90689	0.91024
863	0.89204	0.89768	0.90027	0.90114	0.90394	0.90697	0.91032
864	0.89214	0.89778	0.90036	0.90123	0.90403	0.90706	0.91041
865	0.89225	0.89787	0.90046	0.90132	0.90412	0.90714	0.91049
866	0.89235	0.89796	0.90055	0.90141	0.90420	0.90723	0.91057
867	0.89244	0.89805	0.90064	0.90150	0.90429	0.90731	0.91065
868	0.89254	0.89815	0.90074	0.90160	0.90438	0.90740	0.91073
869	0.89263	0.89824	0.90083	0.90169	0.90447	0.90748	0.91081
870	0.89274	0.89833	0.90092	0.90178	0.90456	0.90756	0.91089
871	0.89283	0.89843	0.90101	0.90187	0.90464	0.90765	0.91097
872	0.89293	0.89852	0.90110	0.90196	0.90473	0.90773	0.91105
873	0.89304	0.89862	0.90119	0.90204	0.90482	0.90781	0.91113
874	0.89313	0.89871	0.90128	0.90213	0.90490	0.90790	0.91121
875	0.89324	0.89880	0.90136	0.90222	0.90499	0.90798	0.91129
876	0.89333	0.89889	0.90146	0.90231	0.90507	0.90806	0.91137
877	0.89343	0.89898	0.90155	0.90240	0.90516	0.90815	0.91145
878	0.89353	0.89907	0.90163	0.90249	0.90525	0.90823	0.91153
879	0.89362	0.89917	0.90172	0.90258	0.90533	0.90831	0.91161
880	0.89372	0.89926	0.90181	0.90267	0.90542	0.90839	0.91169
881	0.89383	0.89934	0.90190	0.90276	0.90550	0.90848	0.91177
882	0.89391	0.89944	0.90198	0.90284	0.90559	0.90856	0.91185
883	0.89402	0.89953	0.90207	0.90293	0.90567	0.90864	0.91193
884	0.89411	0.89962	0.90216	0.90302	0.90576	0.90872	0.91201
885	0.89421	0.89971	0.90225	0.90311	0.90584	0.90880	0.91208
886	0.89431	0.89980	0.90234	0.90319	0.90593	0.90889	0.91216
887	0.89439	0.89989	0.90243	0.90328	0.90601	0.90897	0.91224
888	0.89449	0.89998	0.90252	0.90336	0.90609	0.90905	0.91232
889	0.89458	0.90007	0.90260	0.90345	0.90618	0.90913	0.91239
890	0.89468	0.90016	0.90269	0.90354	0.90626	0.90921	0.91247
891	0.89477	0.90025	0.90278	0.90362	0.90635	0.90929	0.91255
892	0.89486	0.90034	0.90287	0.90371	0.90643	0.90937	0.91263
893	0.89495	0.90043	0.90295	0.90380	0.90651	0.90945	0.91270
894	0.89504	0.90052	0.90304	0.90388	0.90660	0.90953	0.91278
895	0.89514	0.90061	0.90312	0.90397	0.90668	0.90961	0.91286
896	0.89524	0.90070	0.90321	0.90406	0.90676	0.90969	0.91293
897	0.89534	0.90079	0.90329	0.90414	0.90684	0.90977	0.91301
898	0.89544	0.90088	0.90338	0.90423	0.90693	0.90985	0.91309
899	0.89553	0.90096	0.90347	0.90431	0.90701	0.90993	0.91316
900	0.89562	0.90105	0.90356	0.90440	0.90709	0.91001	0.91324
901	0.89573	0.90114	0.90365	0.90448	0.90717	0.91009	0.91332
902	0.89582	0.90122	0.90373	0.90456	0.90725	0.91017	0.91339
903	0.89590	0.90131	0.90382	0.90465	0.90734	0.91025	0.91347
904	0.89600	0.90140	0.90390	0.90473	0.90742	0.91033	0.91354
905	0.89610	0.90149	0.90398	0.90482	0.90750	0.91040	0.91362
906	0.89618	0.90158	0.90407	0.90490	0.90758	0.91048	0.91369
907	0.89626	0.90166	0.90415	0.90499	0.90766	0.91056	0.91377
908	0.89635	0.90175	0.90424	0.90507	0.90774	0.91064	0.91384
909	0.89645	0.90183	0.90432	0.90515	0.90782	0.91071	0.91391
910	0.89653	0.90192	0.90441	0.90524	0.90790	0.91079	0.91399
911	0.89662	0.90200	0.90449	0.90532	0.90798	0.91087	0.91406
912	0.89672	0.90209	0.90458	0.90541	0.90806	0.91094	0.91414
913	0.89682	0.90218	0.90466	0.90549	0.90814	0.91102	0.91421
914	0.89691	0.90226	0.90474	0.90557	0.90822	0.91110	0.91428
915	0.89700	0.90234	0.90482	0.90565	0.90830	0.91118	0.91436
916	0.89709	0.90243	0.90491	0.90574	0.90838	0.91125	0.91443
917	0.89718	0.90251	0.90499	0.90582	0.90846	0.91133	0.91451
918	0.89726	0.90260	0.90507	0.90589	0.90854	0.91141	0.91458
919	0.89734	0.90268	0.90515	0.90597	0.90862	0.91148	0.91465
920	0.89744	0.90277	0.90523	0.90606	0.90870	0.91156	0.91473
921	0.89754	0.90286	0.90531	0.90614	0.90878	0.91164	0.91480
922	0.89764	0.90295	0.90539	0.90622	0.90886	0.91171	0.91487
923	0.89773	0.90303	0.90548	0.90630	0.90894	0.91179	0.91494
924	0.89782	0.90311	0.90556	0.90638	0.90901	0.91186	0.91501
925	0.89791	0.90320	0.90564	0.90646	0.90909	0.91194	0.91509
926	0.89800	0.90328	0.90573	0.90655	0.90917	0.91201	0.91516
927	0.89809	0.90336	0.90581	0.90662	0.90925	0.91209	0.91523

N	0.1%	0.5%	1%	1.25%	2.5%	5%	10%
928	0.89817	0.90345	0.90589	0.90670	0.90933	0.91216	0.91530
929	0.89826	0.90354	0.90597	0.90678	0.90941	0.91224	0.91538
930	0.89834	0.90362	0.90605	0.90686	0.90948	0.91231	0.91545
931	0.89843	0.90370	0.90613	0.90695	0.90956	0.91239	0.91552
932	0.89853	0.90378	0.90621	0.90702	0.90964	0.91246	0.91559
933	0.89862	0.90386	0.90629	0.90710	0.90972	0.91254	0.91566
934	0.89871	0.90395	0.90637	0.90718	0.90979	0.91261	0.91573
935	0.89880	0.90402	0.90645	0.90726	0.90987	0.91268	0.91580
936	0.89889	0.90411	0.90653	0.90735	0.90995	0.91276	0.91588
937	0.89897	0.90419	0.90661	0.90742	0.91002	0.91283	0.91595
938	0.89907	0.90428	0.90669	0.90750	0.91010	0.91290	0.91602
939	0.89915	0.90436	0.90677	0.90758	0.91017	0.91298	0.91609
940	0.89924	0.90444	0.90685	0.90766	0.91025	0.91305	0.91616
941	0.89932	0.90453	0.90693	0.90774	0.91033	0.91312	0.91623
942	0.89941	0.90461	0.90701	0.90782	0.91040	0.91320	0.91630
943	0.89950	0.90469	0.90709	0.90790	0.91048	0.91327	0.91637
944	0.89958	0.90477	0.90717	0.90797	0.91055	0.91334	0.91644
945	0.89968	0.90485	0.90725	0.90805	0.91063	0.91342	0.91651
946	0.89975	0.90493	0.90733	0.90813	0.91070	0.91349	0.91658
947	0.89982	0.90501	0.90740	0.90820	0.91078	0.91356	0.91665
948	0.89991	0.90509	0.90748	0.90828	0.91085	0.91363	0.91671
949	0.89999	0.90517	0.90756	0.90836	0.91093	0.91371	0.91678
950	0.90008	0.90525	0.90764	0.90843	0.91100	0.91378	0.91685
951	0.90017	0.90533	0.90772	0.90851	0.91108	0.91385	0.91692
952	0.90026	0.90541	0.90779	0.90858	0.91115	0.91392	0.91699
953	0.90034	0.90549	0.90787	0.90866	0.91123	0.91399	0.91706
954	0.90042	0.90556	0.90795	0.90874	0.91130	0.91407	0.91713
955	0.90051	0.90565	0.90803	0.90881	0.91137	0.91414	0.91720
956	0.90060	0.90573	0.90810	0.90889	0.91145	0.91421	0.91726
957	0.90068	0.90580	0.90818	0.90897	0.91152	0.91428	0.91733
958	0.90076	0.90588	0.90825	0.90904	0.91159	0.91435	0.91740
959	0.90084	0.90596	0.90833	0.90912	0.91167	0.91442	0.91747
960	0.90092	0.90604	0.90841	0.90920	0.91174	0.91449	0.91754
961	0.90100	0.90612	0.90848	0.90927	0.91182	0.91456	0.91761
962	0.90108	0.90620	0.90856	0.90935	0.91189	0.91463	0.91767
963	0.90116	0.90627	0.90864	0.90942	0.91196	0.91470	0.91774
964	0.90125	0.90635	0.90872	0.90949	0.91204	0.91477	0.91781
965	0.90133	0.90643	0.90879	0.90957	0.91211	0.91484	0.91787
966	0.90141	0.90651	0.90886	0.90964	0.91218	0.91491	0.91794
967	0.90150	0.90658	0.90894	0.90972	0.91225	0.91498	0.91801
968	0.90158	0.90666	0.90901	0.90979	0.91233	0.91505	0.91807
969	0.90166	0.90674	0.90909	0.90987	0.91240	0.91512	0.91814
970	0.90174	0.90681	0.90916	0.90994	0.91247	0.91519	0.91821
971	0.90183	0.90689	0.90924	0.91002	0.91254	0.91526	0.91827
972	0.90191	0.90697	0.90931	0.91009	0.91261	0.91533	0.91834
973	0.90199	0.90705	0.90939	0.91017	0.91268	0.91540	0.91841
974	0.90207	0.90712	0.90946	0.91024	0.91275	0.91547	0.91847
975	0.90215	0.90720	0.90954	0.91031	0.91283	0.91554	0.91854
976	0.90224	0.90727	0.90961	0.91039	0.91290	0.91560	0.91860
977	0.90232	0.90735	0.90968	0.91046	0.91297	0.91567	0.91867
978	0.90240	0.90743	0.90976	0.91053	0.91304	0.91574	0.91873
979	0.90248	0.90750	0.90983	0.91060	0.91311	0.91581	0.91880
980	0.90256	0.90758	0.90991	0.91067	0.91318	0.91588	0.91887
981	0.90263	0.90766	0.90998	0.91075	0.91325	0.91595	0.91893
982	0.90271	0.90773	0.91005	0.91082	0.91332	0.91602	0.91900
983	0.90279	0.90781	0.91013	0.91089	0.91339	0.91608	0.91906
984	0.90287	0.90788	0.91020	0.91097	0.91346	0.91615	0.91913
985	0.90296	0.90796	0.91027	0.91104	0.91353	0.91622	0.91919
986	0.90305	0.90804	0.91034	0.91111	0.91360	0.91629	0.91926
987	0.90313	0.90811	0.91042	0.91119	0.91367	0.91635	0.91932
988	0.90320	0.90818	0.91049	0.91126	0.91374	0.91642	0.91939
989	0.90328	0.90826	0.91057	0.91133	0.91381	0.91649	0.91945
990	0.90336	0.90834	0.91064	0.91140	0.91388	0.91655	0.91952
991	0.90345	0.90841	0.91071	0.91147	0.91395	0.91662	0.91958
992	0.90354	0.90848	0.91078	0.91154	0.91402	0.91669	0.91965
993	0.90361	0.90856	0.91085	0.91162	0.91409	0.91676	0.91971
994	0.90369	0.90863	0.91093	0.91169	0.91416	0.91682	0.91977
995	0.90377	0.90870	0.91100	0.91176	0.91423	0.91689	0.91984
996	0.90384	0.90878	0.91107	0.91183	0.91430	0.91695	0.91990
997	0.90391	0.90885	0.91114	0.91190	0.91436	0.91702	0.91996
998	0.90400	0.90893	0.91121	0.91197	0.91443	0.91709	0.92003
999	0.90407	0.90900	0.91128	0.91204	0.91450	0.91715	0.92009
1000	0.90415	0.90907	0.91136	0.91211	0.91457	0.91722	0.92015

Table 3 – *GM9(7H2L or 2H7L).* Critical values of upper significance levels for the Grubbs multiple-9 outlier test, applicable in situations where the seven highest and two lowest or two highest and seven lowest observations are simultaneously identified as outliers, denoted as *GM9(7H2L)* and *GM9(2H7L)*, respectively.

N	0.1%	0.5%	1%	1.25%	2.5%	5%	10%
11	0.00000	0.00000	0.00000	0.00000	0.00000	0.00001	0.00006
12	0.00001	0.00005	0.00010	0.00013	0.00026	0.00055	0.00121
13	0.00015	0.00046	0.00077	0.00090	0.00150	0.00255	0.00446
14	0.00070	0.00166	0.00244	0.00277	0.00414	0.00628	0.00978
15	0.00188	0.00386	0.00530	0.00588	0.00820	0.01163	0.01685
16	0.00386	0.00710	0.00932	0.01019	0.01357	0.01834	0.02526
17	0.00666	0.01136	0.01442	0.01561	0.02007	0.02616	0.03472
18	0.01031	0.01650	0.02046	0.02197	0.02750	0.03486	0.04495
19	0.01461	0.02246	0.02725	0.02904	0.03566	0.04421	0.05569
20	0.01966	0.02907	0.03473	0.03683	0.04441	0.05406	0.06683
21	0.02516	0.03629	0.04276	0.04512	0.05360	0.06429	0.07820
22	0.03129	0.04394	0.05113	0.05376	0.06311	0.07473	0.08971
23	0.03784	0.05190	0.05982	0.06268	0.07280	0.08533	0.10122
24	0.04492	0.06018	0.06873	0.07182	0.08271	0.09598	0.11274
25	0.05206	0.06869	0.07783	0.08114	0.09268	0.10668	0.12414
26	0.05955	0.07739	0.08709	0.09057	0.10267	0.11734	0.13545
27	0.06721	0.08608	0.09636	0.10001	0.11268	0.12784	0.14661
28	0.07495	0.09490	0.10562	0.10942	0.12259	0.13833	0.15758
29	0.08296	0.10378	0.11490	0.11883	0.13247	0.14863	0.16834
30	0.09091	0.11255	0.12414	0.12823	0.14221	0.15881	0.17892
31	0.09892	0.12131	0.13334	0.13755	0.15189	0.16881	0.18930
32	0.10697	0.13021	0.14240	0.14671	0.16143	0.17869	0.19946
33	0.11496	0.13885	0.15141	0.15587	0.17085	0.18836	0.20940
34	0.12318	0.14755	0.16034	0.16479	0.18011	0.19789	0.21916
35	0.13099	0.15615	0.16915	0.17373	0.18927	0.20722	0.22870
36	0.13909	0.16457	0.17786	0.18256	0.19824	0.21635	0.23799
37	0.14692	0.17292	0.18643	0.19114	0.20706	0.22531	0.24709
38	0.15475	0.18115	0.19483	0.19958	0.21564	0.23408	0.25592
39	0.16254	0.18922	0.20309	0.20793	0.22411	0.24266	0.26462
40	0.16991	0.19719	0.21126	0.21607	0.23241	0.25108	0.27308
41	0.17742	0.20518	0.21925	0.22415	0.24051	0.25932	0.28136
42	0.18481	0.21292	0.22716	0.23207	0.24853	0.26737	0.28946
43	0.19241	0.22058	0.23491	0.23984	0.25635	0.27525	0.29734
44	0.19962	0.22809	0.24246	0.24744	0.26407	0.28296	0.30505
45	0.20690	0.23549	0.24992	0.25491	0.27161	0.29047	0.31262
46	0.21386	0.24264	0.25719	0.26221	0.27897	0.29789	0.31998
47	0.22073	0.24980	0.26434	0.26936	0.28618	0.30511	0.32720
48	0.22749	0.25682	0.27145	0.27641	0.29325	0.31216	0.33421
49	0.23442	0.26377	0.27831	0.28334	0.30016	0.31912	0.34110
50	0.24119	0.27062	0.28506	0.29012	0.30693	0.32589	0.34785
51	0.24767	0.27726	0.29186	0.29688	0.31362	0.33251	0.35441
52	0.25420	0.28374	0.29829	0.30335	0.32016	0.33902	0.36086
53	0.26051	0.29010	0.30474	0.30975	0.32653	0.34541	0.36715
54	0.26666	0.29645	0.31102	0.31607	0.33277	0.35167	0.37333
55	0.27283	0.30261	0.31725	0.32224	0.33898	0.35782	0.37938
56	0.27882	0.30866	0.32334	0.32830	0.34502	0.36378	0.38532
57	0.28472	0.31472	0.32933	0.33429	0.35097	0.36964	0.39110
58	0.29066	0.32057	0.33515	0.34011	0.35679	0.37540	0.39677
59	0.29636	0.32646	0.34089	0.34587	0.36252	0.38103	0.40234
60	0.30203	0.33209	0.34657	0.35154	0.36807	0.38656	0.40778
61	0.30763	0.33763	0.35217	0.35713	0.37356	0.39198	0.41311
62	0.31325	0.34311	0.35762	0.36256	0.37897	0.39731	0.41833
63	0.31869	0.34850	0.36295	0.36789	0.38428	0.40254	0.42343
64	0.32400	0.35372	0.36820	0.37309	0.38946	0.40767	0.42846
65	0.32936	0.35894	0.37332	0.37828	0.39457	0.41268	0.43339
66	0.33452	0.36410	0.37841	0.38329	0.39957	0.41763	0.43822
67	0.33971	0.36915	0.38342	0.38829	0.40447	0.42245	0.44296
68	0.34469	0.37411	0.38833	0.39321	0.40933	0.42718	0.44763
69	0.34960	0.37897	0.39315	0.39801	0.41406	0.43187	0.45221
70	0.35438	0.38366	0.39798	0.40280	0.41874	0.43647	0.45668
71	0.35929	0.38847	0.40269	0.40744	0.42335	0.44099	0.46108
72	0.36375	0.39311	0.40722	0.41201	0.42785	0.44541	0.46540
73	0.36827	0.39769	0.41174	0.41650	0.43229	0.44974	0.46963
74	0.37280	0.40220	0.41621	0.42093	0.43660	0.45402	0.47379
75	0.37714	0.40662	0.42057	0.42527	0.44092	0.45822	0.47790
76	0.38168	0.41089	0.42486	0.42955	0.44510	0.46234	0.48191
77	0.38615	0.41513	0.42908	0.43375	0.44923	0.46639	0.48586
78	0.39060	0.41941	0.43322	0.43792	0.45331	0.47039	0.48976

N	0.1%	0.5%	1%	1.25%	2.5%	5%	10%
79	0.39488	0.42356	0.43732	0.44196	0.45735	0.47432	0.49358
80	0.39902	0.42763	0.44137	0.44596	0.46126	0.47818	0.49735
81	0.40305	0.43161	0.44524	0.44987	0.46512	0.48195	0.50103
82	0.40723	0.43557	0.44917	0.45374	0.46893	0.48568	0.50467
83	0.41102	0.43946	0.45300	0.45760	0.47269	0.48938	0.50824
84	0.41495	0.44334	0.45678	0.46139	0.47639	0.49301	0.51175
85	0.41887	0.44706	0.46050	0.46507	0.48006	0.49656	0.51521
86	0.42275	0.45074	0.46416	0.46871	0.48365	0.50008	0.51861
87	0.42647	0.45446	0.46779	0.47232	0.48718	0.50353	0.52197
88	0.43031	0.45809	0.47138	0.47589	0.49064	0.50691	0.52526
89	0.43409	0.46164	0.47492	0.47937	0.49405	0.51024	0.52849
90	0.43758	0.46517	0.47837	0.48282	0.49743	0.51354	0.53169
91	0.44126	0.46867	0.48178	0.48623	0.50078	0.51678	0.53484
92	0.44485	0.47207	0.48512	0.48955	0.50405	0.51997	0.53795
93	0.44819	0.47542	0.48846	0.49289	0.50725	0.52314	0.54101
94	0.45156	0.47878	0.49174	0.49612	0.51043	0.52624	0.54400
95	0.45508	0.48207	0.49494	0.49927	0.51357	0.52928	0.54697
96	0.45841	0.48530	0.49813	0.50247	0.51670	0.53228	0.54988
97	0.46166	0.48852	0.50126	0.50556	0.51971	0.53528	0.55277
98	0.46486	0.49167	0.50436	0.50865	0.52273	0.53820	0.55563
99	0.46812	0.49479	0.50740	0.51169	0.52570	0.54107	0.55843
100	0.47127	0.49788	0.51038	0.51467	0.52862	0.54392	0.56119
101	0.47458	0.50090	0.51337	0.51763	0.53149	0.54675	0.56391
102	0.47766	0.50388	0.51634	0.52054	0.53432	0.54951	0.56658
103	0.48083	0.50681	0.51922	0.52342	0.53714	0.55225	0.56922
104	0.48373	0.50974	0.52210	0.52626	0.53994	0.55493	0.57182
105	0.48662	0.51258	0.52492	0.52906	0.54268	0.55759	0.57440
106	0.48952	0.51544	0.52769	0.53185	0.54536	0.56021	0.57692
107	0.49241	0.51825	0.53041	0.53454	0.54803	0.56282	0.57944
108	0.49526	0.52103	0.53316	0.53725	0.55065	0.56538	0.58192
109	0.49814	0.52376	0.53583	0.53991	0.55324	0.56791	0.58435
110	0.50087	0.52647	0.53849	0.54252	0.55579	0.57038	0.58674
111	0.50367	0.52912	0.54108	0.54510	0.55832	0.57284	0.58912
112	0.50644	0.53173	0.54367	0.54766	0.56081	0.57526	0.59147
113	0.50913	0.53432	0.54622	0.55017	0.56325	0.57766	0.59379
114	0.51174	0.53690	0.54873	0.55265	0.56567	0.58002	0.59607
115	0.51444	0.53941	0.55118	0.55511	0.56808	0.58233	0.59832
116	0.51708	0.54189	0.55362	0.55758	0.57047	0.58463	0.60054
117	0.51959	0.54438	0.55607	0.55996	0.57279	0.58690	0.60273
118	0.52209	0.54687	0.55846	0.56232	0.57512	0.58914	0.60490
119	0.52469	0.54926	0.56082	0.56466	0.57740	0.59135	0.60704
120	0.52720	0.55162	0.56316	0.56699	0.57969	0.59354	0.60916
121	0.52965	0.55396	0.56547	0.56929	0.58190	0.59574	0.61125
122	0.53197	0.55634	0.56775	0.57156	0.58410	0.59787	0.61332
123	0.53443	0.55862	0.56999	0.57379	0.58628	0.59999	0.61536
124	0.53674	0.56088	0.57222	0.57602	0.58845	0.60207	0.61738
125	0.53909	0.56310	0.57438	0.57817	0.59058	0.60415	0.61937
126	0.54144	0.56531	0.57656	0.58033	0.59269	0.60618	0.62134
127	0.54373	0.56748	0.57870	0.58246	0.59478	0.60821	0.62329
128	0.54604	0.56970	0.58083	0.58454	0.59684	0.61022	0.62522
129	0.54821	0.57184	0.58291	0.58664	0.59887	0.61219	0.62712
130	0.55048	0.57395	0.58502	0.58869	0.60085	0.61415	0.62900
131	0.55267	0.57610	0.58706	0.59072	0.60286	0.61607	0.63086
132	0.55499	0.57814	0.58908	0.59273	0.60481	0.61798	0.63271
133	0.55708	0.58018	0.59109	0.59475	0.60676	0.61987	0.63452
134	0.55927	0.58222	0.59308	0.59672	0.60867	0.62174	0.63633
135	0.56132	0.58423	0.59507	0.59870	0.61057	0.62359	0.63811
136	0.56349	0.58623	0.59701	0.60063	0.61247	0.62542	0.63986
137	0.56559	0.58821	0.59894	0.60257	0.61432	0.62722	0.64160
138	0.56768	0.59016	0.60084	0.60445	0.61615	0.62899	0.64332
139	0.56968	0.59209	0.60272	0.60631	0.61798	0.63075	0.64502
140	0.57173	0.59402	0.60462	0.60817	0.61981	0.63250	0.64670
141	0.57365	0.59589	0.60645	0.61002	0.62159	0.63424	0.64836
142	0.57566	0.59774	0.60826	0.61181	0.62337	0.63595	0.65001
143	0.57760	0.59960	0.61011	0.61361	0.62513	0.63764	0.65165
144	0.57947	0.60149	0.61190	0.61540	0.62686	0.63932	0.65326
145	0.58154	0.60331	0.61366	0.61716	0.62857	0.64099	0.65485
146	0.58331	0.60508	0.61545	0.61888	0.63027	0.64263	0.65644
147	0.58513	0.60686	0.61717	0.62062	0.63195	0.64424	0.65799
148	0.58703	0.60865	0.61885	0.62231	0.63360	0.64584	0.65953
149	0.58890	0.61036	0.62058	0.62401	0.63522	0.64744	0.66106
150	0.59073	0.61209	0.62228	0.62570	0.63686	0.64902	0.66257
151	0.59245	0.61377	0.62395	0.62736	0.63847	0.65060	0.66407
152	0.59424	0.61552	0.62560	0.62902	0.64007	0.65213	0.66556

N	0.1%	0.5%	1%	1.25%	2.5%	5%	10%
153	0.59606	0.61721	0.62724	0.63062	0.64166	0.65367	0.66704
154	0.59777	0.61886	0.62888	0.63224	0.64322	0.65518	0.66849
155	0.59955	0.62051	0.63048	0.63385	0.64477	0.65667	0.66994
156	0.60124	0.62214	0.63207	0.63542	0.64629	0.65814	0.67136
157	0.60293	0.62375	0.63368	0.63700	0.64783	0.65962	0.67278
158	0.60463	0.62534	0.63524	0.63855	0.64931	0.66106	0.67419
159	0.60617	0.62691	0.63678	0.64005	0.65082	0.66252	0.67557
160	0.60780	0.62850	0.63831	0.64158	0.65230	0.66394	0.67695
161	0.60947	0.63009	0.63982	0.64308	0.65376	0.66536	0.67831
162	0.61111	0.63160	0.64131	0.64457	0.65520	0.66677	0.67965
163	0.61270	0.63313	0.64281	0.64606	0.65664	0.66816	0.68099
164	0.61427	0.63466	0.64427	0.64750	0.65806	0.66953	0.68231
165	0.61588	0.63616	0.64574	0.64896	0.65947	0.67089	0.68362
166	0.61748	0.63764	0.64719	0.65039	0.66087	0.67224	0.68493
167	0.61911	0.63912	0.64862	0.65180	0.66226	0.67358	0.68622
168	0.62061	0.64056	0.65005	0.65322	0.66363	0.67490	0.68749
169	0.62214	0.64203	0.65146	0.65461	0.66497	0.67623	0.68875
170	0.62361	0.64348	0.65288	0.65603	0.66631	0.67753	0.69000
171	0.62504	0.64488	0.65424	0.65741	0.66765	0.67882	0.69122
172	0.62653	0.64631	0.65562	0.65879	0.66898	0.68009	0.69245
173	0.62794	0.64770	0.65700	0.66013	0.67029	0.68136	0.69368
174	0.62944	0.64911	0.65834	0.66147	0.67159	0.68261	0.69488
175	0.63091	0.65049	0.65968	0.66278	0.67287	0.68386	0.69607
176	0.63227	0.65181	0.66101	0.66407	0.67415	0.68509	0.69727
177	0.63361	0.65318	0.66232	0.66540	0.67541	0.68632	0.69845
178	0.63498	0.65450	0.66362	0.66669	0.67667	0.68753	0.69960
179	0.63646	0.65584	0.66490	0.66797	0.67791	0.68875	0.70076
180	0.63781	0.65716	0.66619	0.66925	0.67915	0.68994	0.70191
181	0.63925	0.65845	0.66744	0.67051	0.68037	0.69111	0.70303
182	0.64055	0.65974	0.66870	0.67174	0.68158	0.69227	0.70417
183	0.64198	0.66104	0.66998	0.67298	0.68276	0.69344	0.70528
184	0.64333	0.66229	0.67120	0.67419	0.68397	0.69459	0.70639
185	0.64460	0.66356	0.67244	0.67540	0.68516	0.69573	0.70749
186	0.64590	0.66482	0.67364	0.67661	0.68631	0.69687	0.70858
187	0.64725	0.66604	0.67485	0.67780	0.68748	0.69799	0.70965
188	0.64843	0.66731	0.67606	0.67901	0.68862	0.69909	0.71073
189	0.64976	0.66851	0.67724	0.68019	0.68977	0.70020	0.71178
190	0.65105	0.66971	0.67843	0.68135	0.69090	0.70130	0.71283
191	0.65239	0.67091	0.67960	0.68252	0.69203	0.70239	0.71388
192	0.65358	0.67208	0.68076	0.68368	0.69314	0.70346	0.71492
193	0.65476	0.67327	0.68191	0.68482	0.69425	0.70453	0.71594
194	0.65597	0.67442	0.68304	0.68594	0.69535	0.70558	0.71696
195	0.65722	0.67559	0.68418	0.68705	0.69643	0.70663	0.71797
196	0.65841	0.67674	0.68532	0.68817	0.69750	0.70769	0.71898
197	0.65969	0.67788	0.68643	0.68929	0.69857	0.70872	0.71997
198	0.66089	0.67901	0.68751	0.69037	0.69964	0.70974	0.72096
199	0.66203	0.68013	0.68862	0.69144	0.70070	0.71077	0.72194
200	0.66323	0.68124	0.68970	0.69253	0.70175	0.71178	0.72290
201	0.66440	0.68235	0.69078	0.69359	0.70280	0.71278	0.72386
202	0.66553	0.68345	0.69187	0.69465	0.70382	0.71378	0.72482
203	0.66672	0.68458	0.69293	0.69571	0.70483	0.71477	0.72577
204	0.66780	0.68566	0.69398	0.69675	0.70585	0.71574	0.72671
205	0.66897	0.68672	0.69502	0.69780	0.70685	0.71672	0.72764
206	0.67014	0.68780	0.69604	0.69883	0.70786	0.71769	0.72857
207	0.67113	0.68889	0.69709	0.69986	0.70886	0.71865	0.72948
208	0.67227	0.68995	0.69812	0.70088	0.70983	0.71959	0.73040
209	0.67336	0.69098	0.69914	0.70189	0.71081	0.72054	0.73130
210	0.67446	0.69200	0.70014	0.70289	0.71179	0.72147	0.73220
211	0.67554	0.69304	0.70116	0.70388	0.71273	0.72240	0.73309
212	0.67662	0.69408	0.70215	0.70486	0.71370	0.72331	0.73398
213	0.67769	0.69509	0.70314	0.70582	0.71464	0.72423	0.73486
214	0.67873	0.69609	0.70413	0.70681	0.71558	0.72514	0.73572
215	0.67980	0.69709	0.70507	0.70777	0.71653	0.72604	0.73659
216	0.68093	0.69808	0.70606	0.70871	0.71746	0.72695	0.73745
217	0.68191	0.69906	0.70701	0.70967	0.71837	0.72783	0.73831
218	0.68298	0.70004	0.70796	0.71061	0.71929	0.72871	0.73916
219	0.68400	0.70100	0.70890	0.71155	0.72019	0.72958	0.74000
220	0.68504	0.70196	0.70986	0.71251	0.72110	0.73045	0.74083
221	0.68603	0.70290	0.71079	0.71342	0.72200	0.73131	0.74167
222	0.68703	0.70387	0.71171	0.71432	0.72288	0.73218	0.74249
223	0.68804	0.70481	0.71263	0.71523	0.72376	0.73303	0.74331
224	0.68897	0.70573	0.71353	0.71612	0.72464	0.73388	0.74412
225	0.68995	0.70666	0.71443	0.71702	0.72551	0.73471	0.74492
226	0.69095	0.70756	0.71531	0.71791	0.72638	0.73555	0.74572

N	0.1%	0.5%	1%	1.25%	2.5%	5%	10%
227	0.69187	0.70849	0.71621	0.71879	0.72723	0.73637	0.74652
228	0.69282	0.70941	0.71709	0.71967	0.72806	0.73719	0.74730
229	0.69377	0.71030	0.71798	0.72056	0.72891	0.73801	0.74809
230	0.69473	0.71120	0.71885	0.72141	0.72976	0.73881	0.74887
231	0.69568	0.71210	0.71972	0.72226	0.73058	0.73962	0.74965
232	0.69666	0.71299	0.72058	0.72311	0.73141	0.74042	0.75041
233	0.69759	0.71387	0.72144	0.72396	0.73224	0.74121	0.75117
234	0.69849	0.71471	0.72228	0.72479	0.73304	0.74200	0.75193
235	0.69934	0.71560	0.72312	0.72563	0.73385	0.74278	0.75268
236	0.70032	0.71646	0.72396	0.72647	0.73465	0.74356	0.75343
237	0.70119	0.71733	0.72479	0.72729	0.73545	0.74433	0.75416
238	0.70214	0.71817	0.72560	0.72811	0.73624	0.74509	0.75490
239	0.70302	0.71901	0.72644	0.72892	0.73705	0.74586	0.75563
240	0.70392	0.71982	0.72725	0.72974	0.73782	0.74661	0.75636
241	0.70480	0.72066	0.72806	0.73054	0.73860	0.74736	0.75708
242	0.70564	0.72148	0.72886	0.73134	0.73937	0.74811	0.75780
243	0.70653	0.72230	0.72968	0.73215	0.74014	0.74885	0.75851
244	0.70738	0.72313	0.73047	0.73293	0.74090	0.74959	0.75922
245	0.70825	0.72397	0.73125	0.73369	0.74166	0.75033	0.75992
246	0.70912	0.72476	0.73205	0.73448	0.74241	0.75105	0.76062
247	0.71000	0.72557	0.73281	0.73526	0.74316	0.75178	0.76131
248	0.71085	0.72636	0.73360	0.73603	0.74391	0.75249	0.76200
249	0.71169	0.72715	0.73436	0.73678	0.74465	0.75321	0.76268
250	0.71254	0.72795	0.73513	0.73755	0.74538	0.75392	0.76336
251	0.71334	0.72872	0.73589	0.73831	0.74611	0.75463	0.76404
252	0.71420	0.72949	0.73665	0.73906	0.74684	0.75532	0.76471
253	0.71500	0.73025	0.73740	0.73979	0.74756	0.75602	0.76538
254	0.71580	0.73101	0.73814	0.74053	0.74828	0.75672	0.76604
255	0.71662	0.73178	0.73889	0.74125	0.74899	0.75740	0.76670
256	0.71747	0.73254	0.73962	0.74199	0.74970	0.75809	0.76735
257	0.71825	0.73329	0.74036	0.74271	0.75039	0.75876	0.76800
258	0.71902	0.73402	0.74108	0.74342	0.75110	0.75944	0.76864
259	0.71981	0.73478	0.74179	0.74415	0.75179	0.76011	0.76929
260	0.72061	0.73552	0.74251	0.74485	0.75248	0.76077	0.76993
261	0.72139	0.73623	0.74323	0.74556	0.75316	0.76143	0.77056
262	0.72216	0.73695	0.74393	0.74627	0.75385	0.76209	0.77119
263	0.72294	0.73769	0.74463	0.74697	0.75452	0.76274	0.77182
264	0.72372	0.73839	0.74534	0.74767	0.75519	0.76338	0.77245
265	0.72445	0.73910	0.74602	0.74834	0.75585	0.76403	0.77307
266	0.72525	0.73984	0.74672	0.74903	0.75651	0.76467	0.77368
267	0.72596	0.74055	0.74740	0.74973	0.75717	0.76530	0.77429
268	0.72673	0.74124	0.74809	0.75039	0.75784	0.76594	0.77490
269	0.72749	0.74196	0.74877	0.75107	0.75850	0.76656	0.77551
270	0.72819	0.74265	0.74944	0.75174	0.75915	0.76720	0.77611
271	0.72888	0.74335	0.75012	0.75240	0.75979	0.76782	0.77671
272	0.72960	0.74404	0.75079	0.75307	0.76043	0.76843	0.77731
273	0.73032	0.74472	0.75145	0.75371	0.76107	0.76905	0.77789
274	0.73107	0.74538	0.75211	0.75437	0.76170	0.76966	0.77848
275	0.73173	0.74605	0.75276	0.75502	0.76233	0.77027	0.77906
276	0.73244	0.74672	0.75342	0.75566	0.76296	0.77087	0.77963
277	0.73309	0.74740	0.75407	0.75632	0.76359	0.77148	0.78021
278	0.73383	0.74808	0.75470	0.75696	0.76420	0.77207	0.78079
279	0.73455	0.74872	0.75536	0.75759	0.76482	0.77267	0.78135
280	0.73527	0.74939	0.75600	0.75823	0.76543	0.77325	0.78192
281	0.73598	0.75006	0.75664	0.75886	0.76603	0.77384	0.78248
282	0.73664	0.75069	0.75727	0.75948	0.76665	0.77442	0.78304
283	0.73730	0.75135	0.75788	0.76012	0.76724	0.77501	0.78360
284	0.73803	0.75199	0.75851	0.76073	0.76784	0.77558	0.78416
285	0.73868	0.75264	0.75914	0.76135	0.76843	0.77615	0.78470
286	0.73939	0.75327	0.75977	0.76195	0.76903	0.77673	0.78526
287	0.74006	0.75391	0.76039	0.76257	0.76962	0.77729	0.78580
288	0.74067	0.75453	0.76100	0.76317	0.77020	0.77785	0.78634
289	0.74132	0.75514	0.76161	0.76377	0.77078	0.77842	0.78687
290	0.74196	0.75575	0.76220	0.76437	0.77135	0.77897	0.78741
291	0.74263	0.75635	0.76280	0.76495	0.77193	0.77953	0.78794
292	0.74328	0.75698	0.76340	0.76555	0.77250	0.78008	0.78847
293	0.74393	0.75758	0.76399	0.76613	0.77308	0.78063	0.78899
294	0.74452	0.75819	0.76459	0.76671	0.77364	0.78118	0.78952
295	0.74516	0.75882	0.76518	0.76730	0.77420	0.78172	0.79003
296	0.74581	0.75942	0.76577	0.76788	0.77476	0.78226	0.79055
297	0.74643	0.76003	0.76634	0.76846	0.77532	0.78280	0.79107
298	0.74708	0.76060	0.76691	0.76902	0.77587	0.78333	0.79158
299	0.74768	0.76119	0.76748	0.76960	0.77642	0.78386	0.79209
300	0.74833	0.76178	0.76807	0.77017	0.77697	0.78438	0.79259

N	0.1%	0.5%	1%	1.25%	2.5%	5%	10%
301	0.74898	0.76235	0.76862	0.77072	0.77751	0.78490	0.79309
302	0.74962	0.76292	0.76918	0.77128	0.77805	0.78542	0.79359
303	0.75020	0.76349	0.76974	0.77182	0.77858	0.78594	0.79409
304	0.75081	0.76407	0.77029	0.77237	0.77911	0.78646	0.79459
305	0.75141	0.76465	0.77084	0.77292	0.77965	0.78697	0.79508
306	0.75198	0.76522	0.77140	0.77348	0.78019	0.78748	0.79557
307	0.75261	0.76579	0.77196	0.77402	0.78072	0.78798	0.79605
308	0.75317	0.76633	0.77250	0.77456	0.78124	0.78849	0.79653
309	0.75375	0.76692	0.77304	0.77509	0.78176	0.78899	0.79702
310	0.75433	0.76747	0.77358	0.77563	0.78227	0.78950	0.79750
311	0.75495	0.76802	0.77412	0.77616	0.78279	0.78999	0.79798
312	0.75554	0.76857	0.77466	0.77669	0.78331	0.79049	0.79845
313	0.75609	0.76911	0.77518	0.77721	0.78382	0.79098	0.79892
314	0.75668	0.76965	0.77571	0.77774	0.78432	0.79147	0.79940
315	0.75720	0.77021	0.77623	0.77825	0.78483	0.79196	0.79986
316	0.75782	0.77072	0.77676	0.77878	0.78533	0.79244	0.80033
317	0.75837	0.77127	0.77727	0.77929	0.78584	0.79293	0.80079
318	0.75895	0.77180	0.77780	0.77981	0.78633	0.79341	0.80125
319	0.75951	0.77234	0.77833	0.78031	0.78683	0.79389	0.80170
320	0.76010	0.77287	0.77884	0.78083	0.78732	0.79436	0.80216
321	0.76061	0.77337	0.77934	0.78133	0.78781	0.79483	0.80261
322	0.76119	0.77388	0.77986	0.78184	0.78829	0.79530	0.80306
323	0.76173	0.77442	0.78036	0.78235	0.78878	0.79577	0.80351
324	0.76230	0.77493	0.78085	0.78285	0.78927	0.79624	0.80396
325	0.76285	0.77544	0.78136	0.78334	0.78974	0.79670	0.80440
326	0.76343	0.77596	0.78186	0.78384	0.79022	0.79716	0.80484
327	0.76393	0.77647	0.78235	0.78433	0.79070	0.79762	0.80528
328	0.76446	0.77698	0.78283	0.78481	0.79117	0.79807	0.80571
329	0.76500	0.77748	0.78333	0.78529	0.79163	0.79852	0.80615
330	0.76552	0.77797	0.78382	0.78579	0.79210	0.79897	0.80658
331	0.76604	0.77847	0.78431	0.78626	0.79257	0.79942	0.80701
332	0.76655	0.77897	0.78479	0.78673	0.79303	0.79987	0.80744
333	0.76707	0.77949	0.78527	0.78721	0.79349	0.80031	0.80786
334	0.76756	0.77998	0.78574	0.78769	0.79394	0.80075	0.80828
335	0.76804	0.78046	0.78622	0.78815	0.79439	0.80119	0.80871
336	0.76858	0.78093	0.78669	0.78862	0.79485	0.80164	0.80913
337	0.76913	0.78143	0.78715	0.78909	0.79530	0.80207	0.80955
338	0.76965	0.78191	0.78764	0.78955	0.79575	0.80250	0.80996
339	0.77015	0.78238	0.78812	0.79002	0.79620	0.80293	0.81037
340	0.77069	0.78286	0.78858	0.79048	0.79665	0.80337	0.81078
341	0.77118	0.78335	0.78905	0.79094	0.79709	0.80379	0.81120
342	0.77166	0.78381	0.78951	0.79140	0.79754	0.80421	0.81161
343	0.77217	0.78429	0.78996	0.79185	0.79798	0.80464	0.81201
344	0.77267	0.78475	0.79041	0.79232	0.79841	0.80506	0.81241
345	0.77318	0.78524	0.79087	0.79275	0.79885	0.80548	0.81281
346	0.77364	0.78568	0.79133	0.79320	0.79927	0.80590	0.81321
347	0.77412	0.78615	0.79178	0.79365	0.79971	0.80632	0.81361
348	0.77461	0.78660	0.79223	0.79409	0.80015	0.80673	0.81401
349	0.77510	0.78706	0.79267	0.79453	0.80056	0.80714	0.81440
350	0.77559	0.78753	0.79311	0.79497	0.80099	0.80756	0.81479
351	0.77607	0.78800	0.79355	0.79542	0.80142	0.80796	0.81519
352	0.77655	0.78845	0.79399	0.79585	0.80184	0.80837	0.81558
353	0.77702	0.78889	0.79443	0.79629	0.80226	0.80877	0.81597
354	0.77748	0.78935	0.79486	0.79672	0.80269	0.80917	0.81635
355	0.77795	0.78979	0.79528	0.79715	0.80310	0.80957	0.81673
356	0.77842	0.79025	0.79573	0.79757	0.80351	0.80997	0.81712
357	0.77889	0.79068	0.79616	0.79800	0.80392	0.81037	0.81749
358	0.77932	0.79112	0.79657	0.79842	0.80433	0.81076	0.81787
359	0.77977	0.79155	0.79700	0.79884	0.80474	0.81116	0.81825
360	0.78026	0.79199	0.79744	0.79926	0.80514	0.81155	0.81862
361	0.78071	0.79243	0.79787	0.79968	0.80555	0.81194	0.81900
362	0.78118	0.79286	0.79829	0.80010	0.80595	0.81233	0.81937
363	0.78164	0.79328	0.79870	0.80051	0.80635	0.81271	0.81974
364	0.78209	0.79371	0.79910	0.80092	0.80675	0.81309	0.82011
365	0.78253	0.79412	0.79952	0.80132	0.80714	0.81347	0.82047
366	0.78297	0.79455	0.79994	0.80172	0.80754	0.81385	0.82084
367	0.78345	0.79498	0.80035	0.80213	0.80794	0.81423	0.82121
368	0.78389	0.79540	0.80075	0.80253	0.80832	0.81460	0.82156
369	0.78432	0.79582	0.80115	0.80293	0.80871	0.81498	0.82193
370	0.78471	0.79622	0.80156	0.80334	0.80910	0.81535	0.82228
371	0.78512	0.79664	0.80197	0.80373	0.80948	0.81573	0.82264
372	0.78559	0.79706	0.80236	0.80414	0.80987	0.81610	0.82300
373	0.78602	0.79748	0.80276	0.80452	0.81026	0.81646	0.82335
374	0.78647	0.79788	0.80316	0.80492	0.81063	0.81684	0.82370

N	0.1%	0.5%	1%	1.25%	2.5%	5%	10%
375	0.78691	0.79828	0.80355	0.80530	0.81101	0.81720	0.82405
376	0.78730	0.79869	0.80395	0.80570	0.81139	0.81756	0.82440
377	0.78776	0.79908	0.80435	0.80608	0.81177	0.81792	0.82475
378	0.78820	0.79946	0.80473	0.80647	0.81214	0.81828	0.82509
379	0.78862	0.79987	0.80512	0.80686	0.81252	0.81864	0.82544
380	0.78902	0.80026	0.80551	0.80724	0.81289	0.81900	0.82578
381	0.78947	0.80066	0.80590	0.80762	0.81325	0.81936	0.82612
382	0.78989	0.80105	0.80629	0.80801	0.81363	0.81971	0.82646
383	0.79026	0.80144	0.80667	0.80838	0.81399	0.82006	0.82680
384	0.79069	0.80182	0.80705	0.80876	0.81435	0.82042	0.82713
385	0.79110	0.80221	0.80742	0.80913	0.81471	0.82076	0.82747
386	0.79153	0.80260	0.80780	0.80951	0.81507	0.82111	0.82780
387	0.79193	0.80299	0.80816	0.80987	0.81543	0.82146	0.82813
388	0.79233	0.80338	0.80853	0.81024	0.81579	0.82180	0.82846
389	0.79273	0.80377	0.80890	0.81060	0.81615	0.82215	0.82880
390	0.79313	0.80415	0.80928	0.81097	0.81650	0.82249	0.82912
391	0.79357	0.80454	0.80964	0.81133	0.81685	0.82283	0.82945
392	0.79395	0.80492	0.81001	0.81169	0.81721	0.82317	0.82977
393	0.79436	0.80528	0.81037	0.81204	0.81756	0.82351	0.83009
394	0.79475	0.80566	0.81073	0.81241	0.81791	0.82385	0.83042
395	0.79518	0.80603	0.81109	0.81277	0.81825	0.82418	0.83074
396	0.79556	0.80641	0.81145	0.81313	0.81860	0.82451	0.83106
397	0.79597	0.80677	0.81181	0.81349	0.81894	0.82485	0.83138
398	0.79632	0.80713	0.81217	0.81384	0.81929	0.82518	0.83170
399	0.79672	0.80750	0.81253	0.81420	0.81963	0.82551	0.83201
400	0.79710	0.80786	0.81287	0.81455	0.81996	0.82584	0.83233
401	0.79747	0.80822	0.81322	0.81490	0.82030	0.82617	0.83264
402	0.79784	0.80859	0.81358	0.81524	0.82065	0.82649	0.83295
403	0.79823	0.80896	0.81393	0.81560	0.82099	0.82682	0.83326
404	0.79859	0.80933	0.81429	0.81594	0.82133	0.82714	0.83357
405	0.79898	0.80968	0.81464	0.81628	0.82166	0.82746	0.83388
406	0.79935	0.81005	0.81499	0.81663	0.82199	0.82778	0.83419
407	0.79972	0.81039	0.81533	0.81697	0.82233	0.82810	0.83450
408	0.80013	0.81074	0.81567	0.81732	0.82266	0.82842	0.83480
409	0.80049	0.81110	0.81601	0.81765	0.82298	0.82873	0.83511
410	0.80084	0.81145	0.81635	0.81799	0.82331	0.82904	0.83541
411	0.80123	0.81180	0.81669	0.81832	0.82364	0.82936	0.83571
412	0.80158	0.81214	0.81703	0.81866	0.82395	0.82967	0.83601
413	0.80196	0.81249	0.81736	0.81899	0.82427	0.82998	0.83631
414	0.80230	0.81284	0.81770	0.81932	0.82459	0.83029	0.83660
415	0.80264	0.81318	0.81803	0.81964	0.82491	0.83060	0.83690
416	0.80300	0.81351	0.81835	0.81997	0.82523	0.83090	0.83719
417	0.80336	0.81385	0.81869	0.82031	0.82555	0.83121	0.83749
418	0.80373	0.81419	0.81902	0.82063	0.82587	0.83151	0.83778
419	0.80411	0.81452	0.81935	0.82096	0.82618	0.83182	0.83807
420	0.80444	0.81485	0.81968	0.82128	0.82650	0.83212	0.83836
421	0.80481	0.81519	0.81999	0.82161	0.82681	0.83242	0.83865
422	0.80515	0.81553	0.82033	0.82193	0.82712	0.83272	0.83894
423	0.80550	0.81586	0.82065	0.82225	0.82743	0.83302	0.83922
424	0.80587	0.81618	0.82097	0.82257	0.82774	0.83332	0.83951
425	0.80621	0.81652	0.82129	0.82288	0.82804	0.83361	0.83979
426	0.80657	0.81685	0.82161	0.82321	0.82834	0.83391	0.84008
427	0.80692	0.81716	0.82192	0.82352	0.82865	0.83420	0.84036
428	0.80726	0.81749	0.82224	0.82383	0.82894	0.83449	0.84064
429	0.80759	0.81781	0.82256	0.82414	0.82925	0.83479	0.84092
430	0.80797	0.81814	0.82287	0.82445	0.82955	0.83508	0.84120
431	0.80833	0.81846	0.82318	0.82476	0.82985	0.83537	0.84148
432	0.80868	0.81879	0.82350	0.82507	0.83015	0.83565	0.84175
433	0.80899	0.81910	0.82380	0.82538	0.83046	0.83594	0.84203
434	0.80934	0.81941	0.82411	0.82568	0.83076	0.83623	0.84230
435	0.80967	0.81973	0.82442	0.82599	0.83105	0.83651	0.84258
436	0.81000	0.82004	0.82473	0.82629	0.83134	0.83680	0.84285
437	0.81034	0.82037	0.82504	0.82660	0.83163	0.83708	0.84312
438	0.81067	0.82067	0.82535	0.82691	0.83193	0.83736	0.84339
439	0.81102	0.82100	0.82565	0.82720	0.83222	0.83764	0.84366
440	0.81132	0.82130	0.82596	0.82750	0.83251	0.83792	0.84393
441	0.81164	0.82160	0.82626	0.82780	0.83280	0.83820	0.84419
442	0.81197	0.82192	0.82655	0.82809	0.83308	0.83848	0.84446
443	0.81229	0.82222	0.82685	0.82839	0.83336	0.83876	0.84473
444	0.81264	0.82252	0.82715	0.82869	0.83365	0.83903	0.84499
445	0.81296	0.82283	0.82745	0.82898	0.83393	0.83931	0.84525
446	0.81326	0.82314	0.82774	0.82927	0.83422	0.83958	0.84551
447	0.81357	0.82345	0.82804	0.82957	0.83451	0.83985	0.84577
448	0.81390	0.82374	0.82833	0.82986	0.83479	0.84012	0.84603

N	0.1%	0.5%	1%	1.25%	2.5%	5%	10%
449	0.81427	0.82403	0.82862	0.83015	0.83507	0.84039	0.84629
450	0.81459	0.82434	0.82891	0.83045	0.83535	0.84066	0.84655
451	0.81490	0.82464	0.82921	0.83074	0.83562	0.84093	0.84681
452	0.81520	0.82494	0.82950	0.83102	0.83590	0.84120	0.84706
453	0.81548	0.82525	0.82978	0.83130	0.83618	0.84146	0.84732
454	0.81580	0.82554	0.83007	0.83157	0.83645	0.84173	0.84757
455	0.81610	0.82583	0.83034	0.83186	0.83673	0.84199	0.84783
456	0.81643	0.82613	0.83064	0.83214	0.83700	0.84225	0.84808
457	0.81673	0.82642	0.83092	0.83243	0.83728	0.84252	0.84833
458	0.81704	0.82671	0.83120	0.83270	0.83755	0.84277	0.84858
459	0.81735	0.82700	0.83149	0.83298	0.83782	0.84304	0.84883
460	0.81765	0.82728	0.83178	0.83327	0.83809	0.84329	0.84908
461	0.81796	0.82757	0.83205	0.83355	0.83835	0.84355	0.84933
462	0.81824	0.82787	0.83233	0.83382	0.83862	0.84381	0.84958
463	0.81855	0.82815	0.83261	0.83410	0.83889	0.84406	0.84982
464	0.81885	0.82843	0.83289	0.83437	0.83916	0.84432	0.85007
465	0.81915	0.82871	0.83317	0.83465	0.83942	0.84457	0.85031
466	0.81945	0.82900	0.83344	0.83492	0.83969	0.84483	0.85056
467	0.81975	0.82928	0.83371	0.83518	0.83995	0.84509	0.85080
468	0.82005	0.82956	0.83398	0.83545	0.84021	0.84534	0.85104
469	0.82035	0.82983	0.83425	0.83572	0.84047	0.84559	0.85128
470	0.82064	0.83011	0.83452	0.83599	0.84073	0.84584	0.85152
471	0.82096	0.83039	0.83478	0.83625	0.84099	0.84609	0.85176
472	0.82123	0.83066	0.83505	0.83652	0.84125	0.84634	0.85200
473	0.82154	0.83094	0.83532	0.83678	0.84150	0.84659	0.85224
474	0.82184	0.83122	0.83559	0.83704	0.84176	0.84684	0.85248
475	0.82213	0.83149	0.83585	0.83731	0.84202	0.84708	0.85271
476	0.82241	0.83175	0.83611	0.83757	0.84227	0.84733	0.85294
477	0.82269	0.83203	0.83637	0.83783	0.84252	0.84757	0.85318
478	0.82299	0.83231	0.83664	0.83809	0.84277	0.84781	0.85341
479	0.82327	0.83258	0.83690	0.83835	0.84303	0.84805	0.85365
480	0.82359	0.83285	0.83717	0.83861	0.84328	0.84830	0.85388
481	0.82386	0.83312	0.83743	0.83886	0.84353	0.84854	0.85411
482	0.82414	0.83339	0.83769	0.83913	0.84378	0.84878	0.85434
483	0.82441	0.83365	0.83795	0.83938	0.84403	0.84902	0.85457
484	0.82469	0.83390	0.83820	0.83963	0.84428	0.84926	0.85480
485	0.82496	0.83417	0.83846	0.83989	0.84453	0.84949	0.85503
486	0.82525	0.83444	0.83872	0.84014	0.84477	0.84973	0.85525
487	0.82553	0.83470	0.83898	0.84039	0.84501	0.84997	0.85548
488	0.82582	0.83496	0.83923	0.84064	0.84525	0.85020	0.85571
489	0.82608	0.83523	0.83948	0.84089	0.84549	0.85043	0.85593
490	0.82636	0.83550	0.83973	0.84114	0.84573	0.85066	0.85615
491	0.82663	0.83576	0.83999	0.84138	0.84597	0.85090	0.85637
492	0.82691	0.83602	0.84023	0.84164	0.84621	0.85113	0.85659
493	0.82718	0.83626	0.84048	0.84189	0.84645	0.85136	0.85682
494	0.82745	0.83651	0.84074	0.84213	0.84669	0.85159	0.85704
495	0.82771	0.83677	0.84097	0.84237	0.84693	0.85182	0.85726
496	0.82801	0.83702	0.84122	0.84262	0.84717	0.85205	0.85748
497	0.82828	0.83729	0.84147	0.84286	0.84740	0.85228	0.85770
498	0.82856	0.83754	0.84172	0.84310	0.84763	0.85251	0.85791
499	0.82884	0.83779	0.84196	0.84335	0.84787	0.85274	0.85813
500	0.82911	0.83806	0.84221	0.84359	0.84810	0.85296	0.85835
501	0.82938	0.83831	0.84245	0.84383	0.84833	0.85318	0.85856
502	0.82960	0.83855	0.84269	0.84407	0.84857	0.85341	0.85878
503	0.82986	0.83879	0.84293	0.84431	0.84880	0.85363	0.85899
504	0.83013	0.83904	0.84317	0.84455	0.84903	0.85386	0.85921
505	0.83039	0.83928	0.84342	0.84480	0.84926	0.85408	0.85942
506	0.83065	0.83953	0.84365	0.84502	0.84949	0.85430	0.85963
507	0.83093	0.83978	0.84389	0.84526	0.84972	0.85452	0.85984
508	0.83120	0.84002	0.84413	0.84550	0.84995	0.85474	0.86005
509	0.83147	0.84027	0.84436	0.84574	0.85018	0.85496	0.86026
510	0.83171	0.84051	0.84460	0.84597	0.85040	0.85518	0.86047
511	0.83197	0.84075	0.84483	0.84619	0.85062	0.85539	0.86068
512	0.83222	0.84099	0.84507	0.84643	0.85085	0.85561	0.86089
513	0.83246	0.84122	0.84531	0.84666	0.85107	0.85582	0.86109
514	0.83271	0.84146	0.84555	0.84690	0.85129	0.85604	0.86130
515	0.83300	0.84170	0.84578	0.84712	0.85151	0.85626	0.86151
516	0.83326	0.84194	0.84601	0.84735	0.85173	0.85647	0.86171
517	0.83350	0.84218	0.84623	0.84758	0.85196	0.85668	0.86191
518	0.83376	0.84241	0.84646	0.84781	0.85217	0.85690	0.86212
519	0.83401	0.84265	0.84669	0.84803	0.85239	0.85711	0.86232
520	0.83426	0.84287	0.84691	0.84826	0.85261	0.85732	0.86252
521	0.83450	0.84312	0.84713	0.84848	0.85283	0.85753	0.86272
522	0.83476	0.84335	0.84737	0.84872	0.85305	0.85774	0.86293

N	0.1%	0.5%	1%	1.25%	2.5%	5%	10%
523	0.83498	0.84359	0.84759	0.84893	0.85327	0.85795	0.86313
524	0.83522	0.84383	0.84782	0.84916	0.85348	0.85816	0.86333
525	0.83545	0.84406	0.84804	0.84938	0.85369	0.85836	0.86353
526	0.83571	0.84428	0.84827	0.84960	0.85391	0.85857	0.86373
527	0.83595	0.84451	0.84849	0.84982	0.85413	0.85878	0.86392
528	0.83619	0.84474	0.84870	0.85003	0.85434	0.85898	0.86412
529	0.83642	0.84496	0.84893	0.85025	0.85455	0.85918	0.86432
530	0.83668	0.84519	0.84915	0.85047	0.85477	0.85939	0.86451
531	0.83691	0.84542	0.84936	0.85069	0.85497	0.85959	0.86471
532	0.83716	0.84566	0.84959	0.85091	0.85518	0.85979	0.86490
533	0.83741	0.84588	0.84980	0.85112	0.85539	0.86000	0.86510
534	0.83765	0.84610	0.85002	0.85134	0.85560	0.86020	0.86529
535	0.83787	0.84632	0.85024	0.85156	0.85581	0.86040	0.86548
536	0.83810	0.84654	0.85046	0.85177	0.85602	0.86060	0.86568
537	0.83835	0.84676	0.85068	0.85199	0.85622	0.86080	0.86587
538	0.83859	0.84698	0.85090	0.85220	0.85643	0.86100	0.86606
539	0.83881	0.84721	0.85111	0.85241	0.85663	0.86120	0.86625
540	0.83903	0.84743	0.85132	0.85262	0.85684	0.86140	0.86644
541	0.83926	0.84765	0.85154	0.85283	0.85705	0.86159	0.86663
542	0.83949	0.84787	0.85176	0.85304	0.85725	0.86179	0.86682
543	0.83971	0.84808	0.85196	0.85325	0.85745	0.86199	0.86701
544	0.83996	0.84830	0.85218	0.85345	0.85766	0.86218	0.86719
545	0.84019	0.84852	0.85238	0.85367	0.85786	0.86238	0.86738
546	0.84040	0.84873	0.85260	0.85388	0.85806	0.86257	0.86757
547	0.84064	0.84895	0.85280	0.85408	0.85826	0.86277	0.86775
548	0.84086	0.84916	0.85301	0.85428	0.85846	0.86296	0.86794
549	0.84109	0.84938	0.85322	0.85449	0.85866	0.86315	0.86813
550	0.84130	0.84960	0.85342	0.85470	0.85886	0.86335	0.86831
551	0.84153	0.84981	0.85363	0.85490	0.85906	0.86353	0.86849
552	0.84178	0.85002	0.85384	0.85511	0.85926	0.86372	0.86868
553	0.84199	0.85023	0.85405	0.85531	0.85945	0.86391	0.86886
554	0.84222	0.85043	0.85426	0.85552	0.85965	0.86410	0.86904
555	0.84245	0.85065	0.85447	0.85573	0.85984	0.86430	0.86922
556	0.84267	0.85086	0.85466	0.85592	0.86004	0.86448	0.86940
557	0.84290	0.85107	0.85487	0.85613	0.86023	0.86467	0.86958
558	0.84311	0.85127	0.85507	0.85633	0.86042	0.86486	0.86976
559	0.84336	0.85149	0.85527	0.85653	0.86062	0.86505	0.86994
560	0.84356	0.85169	0.85547	0.85673	0.86081	0.86524	0.87012
561	0.84377	0.85188	0.85567	0.85692	0.86101	0.86542	0.87030
562	0.84401	0.85209	0.85588	0.85712	0.86120	0.86561	0.87048
563	0.84423	0.85230	0.85608	0.85732	0.86139	0.86579	0.87065
564	0.84443	0.85251	0.85628	0.85752	0.86158	0.86597	0.87083
565	0.84465	0.85272	0.85648	0.85772	0.86177	0.86616	0.87100
566	0.84487	0.85292	0.85667	0.85791	0.86196	0.86634	0.87118
567	0.84508	0.85314	0.85687	0.85811	0.86214	0.86652	0.87136
568	0.84530	0.85333	0.85706	0.85830	0.86233	0.86670	0.87153
569	0.84552	0.85353	0.85725	0.85850	0.86252	0.86689	0.87170
570	0.84573	0.85372	0.85745	0.85869	0.86271	0.86707	0.87187
571	0.84593	0.85393	0.85764	0.85888	0.86289	0.86724	0.87205
572	0.84614	0.85413	0.85784	0.85907	0.86308	0.86742	0.87222
573	0.84634	0.85432	0.85803	0.85927	0.86326	0.86760	0.87239
574	0.84655	0.85452	0.85822	0.85946	0.86345	0.86778	0.87256
575	0.84677	0.85471	0.85841	0.85965	0.86363	0.86796	0.87273
576	0.84698	0.85491	0.85861	0.85983	0.86382	0.86813	0.87290
577	0.84718	0.85511	0.85880	0.86003	0.86400	0.86831	0.87307
578	0.84738	0.85531	0.85899	0.86022	0.86418	0.86849	0.87324
579	0.84760	0.85550	0.85918	0.86041	0.86437	0.86866	0.87341
580	0.84782	0.85570	0.85937	0.86060	0.86455	0.86884	0.87358
581	0.84803	0.85590	0.85955	0.86078	0.86473	0.86901	0.87375
582	0.84823	0.85609	0.85974	0.86096	0.86491	0.86919	0.87391
583	0.84843	0.85629	0.85992	0.86114	0.86510	0.86936	0.87408
584	0.84864	0.85648	0.86011	0.86133	0.86527	0.86953	0.87424
585	0.84883	0.85667	0.86030	0.86151	0.86545	0.86971	0.87441
586	0.84902	0.85686	0.86048	0.86170	0.86563	0.86988	0.87457
587	0.84922	0.85704	0.86066	0.86188	0.86581	0.87004	0.87474
588	0.84941	0.85724	0.86085	0.86206	0.86599	0.87022	0.87490
589	0.84962	0.85743	0.86103	0.86225	0.86616	0.87039	0.87507
590	0.84981	0.85762	0.86122	0.86243	0.86634	0.87056	0.87523
591	0.85001	0.85780	0.86140	0.86261	0.86652	0.87073	0.87539
592	0.85023	0.85799	0.86159	0.86279	0.86669	0.87090	0.87556
593	0.85045	0.85818	0.86177	0.86297	0.86687	0.87106	0.87572
594	0.85063	0.85836	0.86196	0.86316	0.86705	0.87123	0.87588
595	0.85082	0.85855	0.86214	0.86334	0.86722	0.87140	0.87604
596	0.85102	0.85873	0.86232	0.86352	0.86739	0.87157	0.87620

N	0.1%	0.5%	1%	1.25%	2.5%	5%	10%
597	0.85122	0.85892	0.86250	0.86370	0.86756	0.87173	0.87636
598	0.85142	0.85910	0.86268	0.86388	0.86774	0.87190	0.87652
599	0.85163	0.85928	0.86286	0.86405	0.86791	0.87206	0.87668
600	0.85181	0.85947	0.86304	0.86423	0.86808	0.87223	0.87684
601	0.85201	0.85965	0.86321	0.86441	0.86825	0.87239	0.87699
602	0.85220	0.85983	0.86339	0.86458	0.86842	0.87256	0.87715
603	0.85239	0.86002	0.86356	0.86476	0.86859	0.87272	0.87731
604	0.85258	0.86020	0.86374	0.86493	0.86876	0.87289	0.87747
605	0.85275	0.86038	0.86392	0.86511	0.86893	0.87305	0.87762
606	0.85295	0.86056	0.86409	0.86528	0.86910	0.87321	0.87778
607	0.85315	0.86074	0.86427	0.86545	0.86927	0.87337	0.87793
608	0.85333	0.86092	0.86444	0.86562	0.86943	0.87353	0.87809
609	0.85352	0.86110	0.86462	0.86579	0.86960	0.87369	0.87825
610	0.85370	0.86128	0.86478	0.86596	0.86976	0.87386	0.87840
611	0.85389	0.86145	0.86496	0.86614	0.86993	0.87402	0.87855
612	0.85408	0.86163	0.86513	0.86631	0.87010	0.87417	0.87871
613	0.85428	0.86180	0.86530	0.86648	0.87026	0.87434	0.87886
614	0.85446	0.86198	0.86547	0.86665	0.87043	0.87449	0.87901
615	0.85464	0.86215	0.86565	0.86682	0.87059	0.87465	0.87916
616	0.85482	0.86233	0.86582	0.86698	0.87076	0.87481	0.87931
617	0.85501	0.86251	0.86599	0.86715	0.87092	0.87497	0.87946
618	0.85519	0.86268	0.86617	0.86732	0.87108	0.87513	0.87962
619	0.85538	0.86287	0.86633	0.86749	0.87124	0.87529	0.87977
620	0.85556	0.86303	0.86650	0.86766	0.87141	0.87544	0.87992
621	0.85574	0.86321	0.86667	0.86783	0.87157	0.87560	0.88007
622	0.85592	0.86338	0.86684	0.86799	0.87173	0.87576	0.88021
623	0.85610	0.86356	0.86700	0.86816	0.87189	0.87591	0.88036
624	0.85628	0.86373	0.86717	0.86833	0.87205	0.87606	0.88051
625	0.85646	0.86390	0.86734	0.86849	0.87222	0.87622	0.88066
626	0.85665	0.86407	0.86750	0.86865	0.87237	0.87637	0.88081
627	0.85684	0.86425	0.86767	0.86882	0.87253	0.87653	0.88096
628	0.85702	0.86442	0.86783	0.86899	0.87269	0.87668	0.88110
629	0.85721	0.86459	0.86800	0.86915	0.87285	0.87683	0.88125
630	0.85738	0.86475	0.86816	0.86931	0.87300	0.87699	0.88140
631	0.85755	0.86492	0.86832	0.86946	0.87316	0.87714	0.88154
632	0.85773	0.86509	0.86849	0.86963	0.87332	0.87729	0.88169
633	0.85792	0.86526	0.86865	0.86979	0.87348	0.87744	0.88183
634	0.85810	0.86542	0.86881	0.86995	0.87363	0.87759	0.88198
635	0.85828	0.86559	0.86898	0.87011	0.87379	0.87774	0.88212
636	0.85845	0.86575	0.86913	0.87027	0.87394	0.87789	0.88227
637	0.85862	0.86592	0.86930	0.87043	0.87410	0.87804	0.88241
638	0.85881	0.86607	0.86946	0.87059	0.87425	0.87819	0.88255
639	0.85898	0.86624	0.86962	0.87075	0.87440	0.87834	0.88269
640	0.85914	0.86641	0.86978	0.87091	0.87456	0.87849	0.88284
641	0.85933	0.86657	0.86994	0.87106	0.87471	0.87864	0.88298
642	0.85949	0.86673	0.87010	0.87122	0.87486	0.87878	0.88312
643	0.85966	0.86690	0.87026	0.87138	0.87501	0.87893	0.88326
644	0.85983	0.86706	0.87041	0.87154	0.87516	0.87908	0.88340
645	0.85999	0.86723	0.87056	0.87169	0.87531	0.87922	0.88354
646	0.86017	0.86738	0.87072	0.87185	0.87546	0.87937	0.88368
647	0.86033	0.86754	0.87088	0.87200	0.87561	0.87951	0.88382
648	0.86051	0.86771	0.87104	0.87216	0.87576	0.87966	0.88396
649	0.86068	0.86787	0.87120	0.87231	0.87592	0.87980	0.88410
650	0.86087	0.86803	0.87135	0.87246	0.87606	0.87995	0.88423
651	0.86102	0.86819	0.87150	0.87262	0.87621	0.88009	0.88437
652	0.86118	0.86834	0.87166	0.87277	0.87636	0.88023	0.88451
653	0.86135	0.86851	0.87181	0.87292	0.87651	0.88038	0.88465
654	0.86153	0.86867	0.87197	0.87308	0.87666	0.88052	0.88479
655	0.86168	0.86883	0.87212	0.87323	0.87681	0.88066	0.88492
656	0.86186	0.86898	0.87227	0.87338	0.87695	0.88080	0.88506
657	0.86204	0.86914	0.87243	0.87353	0.87710	0.88095	0.88519
658	0.86220	0.86929	0.87258	0.87369	0.87725	0.88109	0.88533
659	0.86236	0.86945	0.87273	0.87384	0.87739	0.88123	0.88546
660	0.86253	0.86961	0.87288	0.87399	0.87754	0.88137	0.88560
661	0.86267	0.86976	0.87303	0.87414	0.87768	0.88151	0.88573
662	0.86284	0.86990	0.87318	0.87429	0.87783	0.88165	0.88587
663	0.86300	0.87006	0.87333	0.87443	0.87797	0.88179	0.88600
664	0.86317	0.87022	0.87348	0.87458	0.87812	0.88193	0.88613
665	0.86332	0.87037	0.87364	0.87473	0.87826	0.88206	0.88627
666	0.86348	0.87053	0.87379	0.87488	0.87840	0.88220	0.88640
667	0.86363	0.87068	0.87394	0.87503	0.87855	0.88234	0.88653
668	0.86380	0.87083	0.87409	0.87518	0.87869	0.88247	0.88666
669	0.86397	0.87099	0.87424	0.87533	0.87883	0.88261	0.88679
670	0.86413	0.87114	0.87438	0.87547	0.87897	0.88275	0.88693

N	0.1%	0.5%	1%	1.25%	2.5%	5%	10%
671	0.86430	0.87129	0.87453	0.87562	0.87911	0.88289	0.88706
672	0.86446	0.87145	0.87467	0.87577	0.87926	0.88302	0.88719
673	0.86461	0.87160	0.87482	0.87591	0.87940	0.88316	0.88732
674	0.86478	0.87175	0.87497	0.87605	0.87954	0.88329	0.88745
675	0.86493	0.87189	0.87512	0.87620	0.87968	0.88343	0.88758
676	0.86511	0.87204	0.87527	0.87634	0.87981	0.88357	0.88771
677	0.86525	0.87220	0.87541	0.87648	0.87995	0.88370	0.88784
678	0.86543	0.87236	0.87556	0.87663	0.88009	0.88383	0.88797
679	0.86558	0.87250	0.87570	0.87677	0.88023	0.88397	0.88809
680	0.86574	0.87265	0.87585	0.87691	0.88037	0.88410	0.88822
681	0.86589	0.87280	0.87599	0.87705	0.88051	0.88423	0.88835
682	0.86605	0.87295	0.87613	0.87720	0.88064	0.88436	0.88847
683	0.86619	0.87309	0.87627	0.87734	0.88078	0.88450	0.88860
684	0.86636	0.87324	0.87642	0.87748	0.88092	0.88463	0.88873
685	0.86651	0.87338	0.87657	0.87762	0.88105	0.88476	0.88886
686	0.86666	0.87353	0.87670	0.87776	0.88119	0.88489	0.88899
687	0.86684	0.87368	0.87685	0.87790	0.88132	0.88502	0.88911
688	0.86698	0.87383	0.87699	0.87804	0.88146	0.88515	0.88924
689	0.86714	0.87397	0.87713	0.87818	0.88160	0.88528	0.88936
690	0.86729	0.87410	0.87727	0.87832	0.88173	0.88541	0.88949
691	0.86744	0.87425	0.87741	0.87845	0.88186	0.88554	0.88961
692	0.86760	0.87439	0.87755	0.87859	0.88200	0.88567	0.88973
693	0.86776	0.87454	0.87769	0.87873	0.88213	0.88580	0.88986
694	0.86791	0.87468	0.87782	0.87887	0.88227	0.88593	0.88998
695	0.86806	0.87482	0.87796	0.87901	0.88240	0.88606	0.89011
696	0.86822	0.87497	0.87810	0.87915	0.88254	0.88619	0.89023
697	0.86837	0.87511	0.87824	0.87929	0.88267	0.88631	0.89035
698	0.86851	0.87526	0.87838	0.87942	0.88280	0.88644	0.89047
699	0.86868	0.87539	0.87851	0.87956	0.88293	0.88657	0.89059
700	0.86881	0.87553	0.87865	0.87969	0.88306	0.88669	0.89072
701	0.86896	0.87567	0.87878	0.87982	0.88319	0.88682	0.89084
702	0.86911	0.87582	0.87892	0.87996	0.88333	0.88695	0.89096
703	0.86926	0.87595	0.87906	0.88009	0.88345	0.88707	0.89108
704	0.86941	0.87609	0.87919	0.88023	0.88358	0.88720	0.89120
705	0.86954	0.87624	0.87932	0.88036	0.88372	0.88732	0.89132
706	0.86968	0.87637	0.87946	0.88050	0.88385	0.88745	0.89144
707	0.86983	0.87651	0.87960	0.88063	0.88398	0.88758	0.89156
708	0.86997	0.87664	0.87973	0.88076	0.88410	0.88770	0.89168
709	0.87011	0.87679	0.87987	0.88089	0.88423	0.88782	0.89180
710	0.87026	0.87692	0.88000	0.88102	0.88436	0.88795	0.89192
711	0.87041	0.87706	0.88013	0.88115	0.88449	0.88807	0.89204
712	0.87056	0.87720	0.88027	0.88129	0.88462	0.88819	0.89215
713	0.87070	0.87733	0.88039	0.88142	0.88474	0.88832	0.89227
714	0.87083	0.87747	0.88052	0.88155	0.88487	0.88844	0.89239
715	0.87098	0.87760	0.88066	0.88168	0.88500	0.88856	0.89251
716	0.87113	0.87773	0.88080	0.88181	0.88512	0.88868	0.89263
717	0.87126	0.87786	0.88093	0.88193	0.88525	0.88881	0.89274
718	0.87142	0.87800	0.88105	0.88207	0.88537	0.88893	0.89286
719	0.87157	0.87814	0.88119	0.88219	0.88550	0.88905	0.89298
720	0.87171	0.87827	0.88132	0.88233	0.88563	0.88917	0.89309
721	0.87185	0.87841	0.88145	0.88246	0.88575	0.88929	0.89321
722	0.87198	0.87854	0.88158	0.88258	0.88587	0.88941	0.89332
723	0.87213	0.87867	0.88171	0.88271	0.88600	0.88953	0.89344
724	0.87227	0.87881	0.88184	0.88284	0.88612	0.88965	0.89355
725	0.87241	0.87894	0.88197	0.88297	0.88625	0.88977	0.89367
726	0.87255	0.87908	0.88210	0.88310	0.88637	0.88989	0.89378
727	0.87269	0.87921	0.88223	0.88323	0.88650	0.89001	0.89389
728	0.87283	0.87934	0.88236	0.88335	0.88662	0.89012	0.89401
729	0.87299	0.87948	0.88248	0.88347	0.88674	0.89024	0.89412
730	0.87313	0.87961	0.88261	0.88360	0.88686	0.89036	0.89423
731	0.87327	0.87974	0.88274	0.88373	0.88698	0.89048	0.89435
732	0.87343	0.87987	0.88287	0.88386	0.88711	0.89060	0.89446
733	0.87355	0.88001	0.88299	0.88399	0.88723	0.89072	0.89458
734	0.87368	0.88013	0.88312	0.88411	0.88735	0.89083	0.89469
735	0.87382	0.88026	0.88325	0.88423	0.88747	0.89095	0.89480
736	0.87395	0.88039	0.88337	0.88436	0.88759	0.89106	0.89491
737	0.87410	0.88052	0.88350	0.88449	0.88771	0.89118	0.89502
738	0.87422	0.88065	0.88363	0.88461	0.88783	0.89130	0.89513
739	0.87436	0.88078	0.88375	0.88473	0.88795	0.89141	0.89524
740	0.87450	0.88091	0.88387	0.88486	0.88807	0.89153	0.89536
741	0.87462	0.88103	0.88400	0.88498	0.88819	0.89164	0.89547
742	0.87476	0.88116	0.88412	0.88510	0.88831	0.89175	0.89557
743	0.87489	0.88129	0.88425	0.88522	0.88843	0.89187	0.89568
744	0.87502	0.88141	0.88437	0.88534	0.88854	0.89198	0.89579

N	0.1%	0.5%	1%	1.25%	2.5%	5%	10%
745	0.87515	0.88154	0.88449	0.88547	0.88866	0.89210	0.89590
746	0.87528	0.88167	0.88461	0.88559	0.88878	0.89221	0.89601
747	0.87543	0.88180	0.88474	0.88571	0.88890	0.89232	0.89612
748	0.87557	0.88192	0.88486	0.88584	0.88902	0.89244	0.89623
749	0.87570	0.88205	0.88498	0.88596	0.88913	0.89255	0.89634
750	0.87583	0.88218	0.88510	0.88608	0.88925	0.89266	0.89645
751	0.87598	0.88231	0.88522	0.88620	0.88937	0.89278	0.89655
752	0.87611	0.88243	0.88534	0.88632	0.88948	0.89289	0.89666
753	0.87625	0.88255	0.88546	0.88643	0.88960	0.89300	0.89677
754	0.87637	0.88268	0.88558	0.88655	0.88972	0.89311	0.89688
755	0.87651	0.88281	0.88570	0.88667	0.88983	0.89322	0.89698
756	0.87664	0.88292	0.88582	0.88680	0.88995	0.89334	0.89709
757	0.87677	0.88305	0.88594	0.88691	0.89006	0.89345	0.89720
758	0.87690	0.88317	0.88606	0.88703	0.89018	0.89356	0.89730
759	0.87703	0.88330	0.88618	0.88715	0.89029	0.89367	0.89741
760	0.87716	0.88342	0.88630	0.88727	0.89040	0.89378	0.89752
761	0.87730	0.88354	0.88642	0.88739	0.89052	0.89389	0.89762
762	0.87741	0.88366	0.88654	0.88750	0.89063	0.89400	0.89773
763	0.87754	0.88379	0.88665	0.88762	0.89074	0.89411	0.89783
764	0.87766	0.88391	0.88677	0.88773	0.89085	0.89422	0.89794
765	0.87778	0.88403	0.88689	0.88785	0.89097	0.89433	0.89804
766	0.87791	0.88414	0.88701	0.88797	0.89108	0.89444	0.89815
767	0.87802	0.88426	0.88712	0.88808	0.89119	0.89454	0.89825
768	0.87815	0.88438	0.88724	0.88820	0.89130	0.89465	0.89835
769	0.87829	0.88451	0.88736	0.88831	0.89141	0.89476	0.89846
770	0.87842	0.88463	0.88748	0.88843	0.89153	0.89487	0.89856
771	0.87854	0.88475	0.88759	0.88854	0.89164	0.89497	0.89866
772	0.87866	0.88486	0.88771	0.88866	0.89175	0.89508	0.89876
773	0.87879	0.88498	0.88783	0.88877	0.89186	0.89519	0.89887
774	0.87889	0.88510	0.88794	0.88889	0.89197	0.89529	0.89897
775	0.87904	0.88521	0.88805	0.88900	0.89208	0.89540	0.89907
776	0.87916	0.88534	0.88817	0.88911	0.89219	0.89550	0.89917
777	0.87930	0.88545	0.88828	0.88923	0.89230	0.89561	0.89927
778	0.87943	0.88558	0.88840	0.88934	0.89241	0.89572	0.89938
779	0.87956	0.88569	0.88851	0.88945	0.89252	0.89582	0.89948
780	0.87968	0.88581	0.88862	0.88957	0.89263	0.89593	0.89958
781	0.87979	0.88592	0.88873	0.88967	0.89274	0.89603	0.89968
782	0.87992	0.88603	0.88885	0.88979	0.89285	0.89614	0.89978
783	0.88005	0.88615	0.88896	0.88990	0.89296	0.89624	0.89988
784	0.88017	0.88627	0.88907	0.89001	0.89306	0.89635	0.89998
785	0.88029	0.88638	0.88919	0.89013	0.89317	0.89645	0.90008
786	0.88041	0.88649	0.88930	0.89024	0.89328	0.89655	0.90018
787	0.88054	0.88661	0.88942	0.89035	0.89338	0.89666	0.90028
788	0.88066	0.88672	0.88953	0.89046	0.89349	0.89676	0.90038
789	0.88079	0.88684	0.88964	0.89058	0.89360	0.89686	0.90048
790	0.88091	0.88695	0.88975	0.89068	0.89370	0.89697	0.90058
791	0.88102	0.88707	0.88986	0.89079	0.89381	0.89707	0.90068
792	0.88114	0.88718	0.88997	0.89090	0.89392	0.89717	0.90078
793	0.88126	0.88730	0.89008	0.89101	0.89402	0.89727	0.90087
794	0.88138	0.88741	0.89019	0.89112	0.89413	0.89737	0.90097
795	0.88149	0.88753	0.89030	0.89123	0.89423	0.89748	0.90107
796	0.88163	0.88764	0.89040	0.89134	0.89434	0.89758	0.90117
797	0.88174	0.88775	0.89052	0.89145	0.89444	0.89768	0.90126
798	0.88185	0.88786	0.89063	0.89156	0.89455	0.89778	0.90136
799	0.88197	0.88797	0.89073	0.89166	0.89465	0.89788	0.90146
800	0.88208	0.88808	0.89084	0.89177	0.89476	0.89798	0.90155
801	0.88220	0.88819	0.89095	0.89188	0.89486	0.89808	0.90165
802	0.88233	0.88830	0.89105	0.89199	0.89497	0.89818	0.90175
803	0.88245	0.88841	0.89116	0.89209	0.89507	0.89828	0.90184
804	0.88257	0.88852	0.89127	0.89220	0.89517	0.89838	0.90194
805	0.88268	0.88863	0.89138	0.89230	0.89528	0.89848	0.90203
806	0.88281	0.88874	0.89148	0.89241	0.89538	0.89858	0.90213
807	0.88291	0.88885	0.89159	0.89252	0.89548	0.89868	0.90223
808	0.88303	0.88896	0.89170	0.89262	0.89558	0.89878	0.90232
809	0.88314	0.88907	0.89181	0.89273	0.89569	0.89888	0.90241
810	0.88326	0.88918	0.89191	0.89284	0.89579	0.89898	0.90251
811	0.88337	0.88929	0.89202	0.89294	0.89589	0.89908	0.90260
812	0.88349	0.88940	0.89212	0.89305	0.89599	0.89918	0.90270
813	0.88361	0.88951	0.89223	0.89315	0.89609	0.89927	0.90279
814	0.88371	0.88962	0.89233	0.89326	0.89619	0.89937	0.90289
815	0.88383	0.88974	0.89244	0.89336	0.89629	0.89947	0.90298
816	0.88395	0.88985	0.89255	0.89346	0.89639	0.89956	0.90307
817	0.88406	0.88995	0.89265	0.89356	0.89650	0.89966	0.90317
818	0.88418	0.89005	0.89275	0.89367	0.89660	0.89975	0.90326

N	0.1%	0.5%	1%	1.25%	2.5%	5%	10%
819	0.88430	0.89016	0.89286	0.89377	0.89670	0.89985	0.90335
820	0.88441	0.89027	0.89297	0.89387	0.89680	0.89995	0.90345
821	0.88452	0.89038	0.89307	0.89397	0.89690	0.90005	0.90354
822	0.88463	0.89048	0.89318	0.89407	0.89700	0.90014	0.90363
823	0.88474	0.89059	0.89328	0.89418	0.89709	0.90024	0.90372
824	0.88486	0.89069	0.89338	0.89428	0.89719	0.90033	0.90381
825	0.88497	0.89080	0.89348	0.89438	0.89729	0.90043	0.90390
826	0.88509	0.89091	0.89358	0.89448	0.89739	0.90053	0.90400
827	0.88520	0.89101	0.89368	0.89459	0.89749	0.90062	0.90409
828	0.88531	0.89111	0.89379	0.89468	0.89759	0.90072	0.90418
829	0.88543	0.89122	0.89389	0.89478	0.89769	0.90081	0.90427
830	0.88553	0.89132	0.89399	0.89489	0.89778	0.90091	0.90436
831	0.88564	0.89142	0.89409	0.89499	0.89788	0.90100	0.90445
832	0.88575	0.89153	0.89419	0.89509	0.89798	0.90109	0.90454
833	0.88587	0.89163	0.89429	0.89519	0.89808	0.90119	0.90463
834	0.88598	0.89173	0.89439	0.89529	0.89817	0.90128	0.90472
835	0.88608	0.89184	0.89449	0.89539	0.89827	0.90138	0.90481
836	0.88620	0.89195	0.89459	0.89549	0.89836	0.90147	0.90490
837	0.88631	0.89205	0.89470	0.89559	0.89846	0.90156	0.90499
838	0.88641	0.89215	0.89480	0.89568	0.89856	0.90166	0.90508
839	0.88654	0.89226	0.89490	0.89578	0.89866	0.90175	0.90517
840	0.88665	0.89235	0.89500	0.89588	0.89875	0.90184	0.90526
841	0.88676	0.89246	0.89510	0.89598	0.89885	0.90193	0.90534
842	0.88686	0.89257	0.89520	0.89608	0.89894	0.90203	0.90543
843	0.88696	0.89267	0.89530	0.89618	0.89904	0.90212	0.90552
844	0.88707	0.89276	0.89540	0.89628	0.89913	0.90221	0.90561
845	0.88717	0.89287	0.89550	0.89637	0.89923	0.90230	0.90570
846	0.88728	0.89297	0.89559	0.89647	0.89933	0.90239	0.90579
847	0.88739	0.89307	0.89569	0.89657	0.89942	0.90248	0.90587
848	0.88749	0.89317	0.89579	0.89667	0.89952	0.90257	0.90596
849	0.88760	0.89327	0.89589	0.89676	0.89961	0.90267	0.90605
850	0.88770	0.89337	0.89598	0.89686	0.89971	0.90276	0.90614
851	0.88780	0.89347	0.89608	0.89696	0.89980	0.90285	0.90623
852	0.88791	0.89357	0.89618	0.89706	0.89989	0.90294	0.90631
853	0.88801	0.89367	0.89628	0.89715	0.89999	0.90303	0.90640
854	0.88811	0.89376	0.89637	0.89725	0.90008	0.90312	0.90649
855	0.88822	0.89387	0.89647	0.89735	0.90017	0.90321	0.90657
856	0.88833	0.89396	0.89657	0.89744	0.90026	0.90330	0.90666
857	0.88844	0.89406	0.89666	0.89754	0.90035	0.90339	0.90674
858	0.88856	0.89416	0.89676	0.89763	0.90045	0.90348	0.90683
859	0.88866	0.89427	0.89685	0.89773	0.90054	0.90357	0.90691
860	0.88876	0.89436	0.89695	0.89782	0.90063	0.90365	0.90700
861	0.88886	0.89446	0.89705	0.89792	0.90072	0.90374	0.90708
862	0.88896	0.89456	0.89714	0.89801	0.90081	0.90383	0.90717
863	0.88907	0.89466	0.89724	0.89811	0.90091	0.90392	0.90725
864	0.88917	0.89476	0.89734	0.89820	0.90100	0.90401	0.90734
865	0.88927	0.89485	0.89743	0.89830	0.90109	0.90410	0.90743
866	0.88937	0.89495	0.89753	0.89839	0.90118	0.90419	0.90751
867	0.88948	0.89504	0.89762	0.89849	0.90127	0.90427	0.90759
868	0.88958	0.89515	0.89771	0.89858	0.90136	0.90436	0.90768
869	0.88968	0.89524	0.89781	0.89867	0.90145	0.90445	0.90776
870	0.88979	0.89534	0.89790	0.89877	0.90154	0.90454	0.90784
871	0.88989	0.89543	0.89799	0.89886	0.90163	0.90462	0.90793
872	0.88999	0.89553	0.89809	0.89895	0.90172	0.90471	0.90801
873	0.89009	0.89562	0.89818	0.89904	0.90181	0.90479	0.90810
874	0.89019	0.89572	0.89827	0.89914	0.90190	0.90488	0.90818
875	0.89030	0.89582	0.89837	0.89923	0.90199	0.90497	0.90826
876	0.89038	0.89591	0.89846	0.89932	0.90208	0.90505	0.90834
877	0.89048	0.89600	0.89855	0.89941	0.90217	0.90514	0.90843
878	0.89059	0.89610	0.89864	0.89950	0.90226	0.90522	0.90851
879	0.89069	0.89619	0.89874	0.89959	0.90235	0.90531	0.90859
880	0.89080	0.89629	0.89882	0.89968	0.90244	0.90539	0.90867
881	0.89090	0.89638	0.89892	0.89977	0.90253	0.90548	0.90876
882	0.89100	0.89647	0.89901	0.89986	0.90261	0.90556	0.90884
883	0.89109	0.89657	0.89910	0.89995	0.90270	0.90565	0.90892
884	0.89120	0.89666	0.89919	0.90004	0.90279	0.90574	0.90900
885	0.89131	0.89675	0.89929	0.90013	0.90288	0.90582	0.90908
886	0.89139	0.89684	0.89938	0.90022	0.90296	0.90590	0.90917
887	0.89149	0.89694	0.89947	0.90032	0.90305	0.90599	0.90925
888	0.89159	0.89703	0.89956	0.90041	0.90314	0.90607	0.90933
889	0.89169	0.89711	0.89965	0.90049	0.90323	0.90615	0.90941
890	0.89178	0.89721	0.89974	0.90059	0.90331	0.90624	0.90949
891	0.89188	0.89730	0.89983	0.90068	0.90339	0.90632	0.90957
892	0.89198	0.89740	0.89992	0.90077	0.90348	0.90641	0.90965

N	0.1%	0.5%	1%	1.25%	2.5%	5%	10%
893	0.89208	0.89749	0.90001	0.90085	0.90357	0.90649	0.90973
894	0.89217	0.89758	0.90010	0.90094	0.90365	0.90657	0.90981
895	0.89227	0.89768	0.90019	0.90103	0.90374	0.90666	0.90989
896	0.89237	0.89777	0.90028	0.90112	0.90382	0.90674	0.90997
897	0.89247	0.89786	0.90036	0.90121	0.90391	0.90682	0.91005
898	0.89256	0.89795	0.90046	0.90129	0.90400	0.90691	0.91013
899	0.89266	0.89804	0.90055	0.90138	0.90408	0.90699	0.91021
900	0.89276	0.89813	0.90063	0.90147	0.90417	0.90707	0.91028
901	0.89285	0.89823	0.90073	0.90156	0.90425	0.90715	0.91036
902	0.89294	0.89832	0.90081	0.90165	0.90434	0.90723	0.91044
903	0.89304	0.89841	0.90090	0.90173	0.90442	0.90731	0.91052
904	0.89314	0.89850	0.90099	0.90182	0.90450	0.90740	0.91060
905	0.89323	0.89859	0.90108	0.90191	0.90459	0.90748	0.91068
906	0.89332	0.89868	0.90117	0.90199	0.90467	0.90756	0.91075
907	0.89342	0.89877	0.90126	0.90208	0.90476	0.90764	0.91083
908	0.89352	0.89886	0.90134	0.90217	0.90484	0.90772	0.91091
909	0.89361	0.89895	0.90143	0.90225	0.90493	0.90780	0.91099
910	0.89370	0.89904	0.90152	0.90234	0.90501	0.90788	0.91106
911	0.89380	0.89913	0.90160	0.90242	0.90509	0.90796	0.91114
912	0.89389	0.89922	0.90169	0.90251	0.90518	0.90804	0.91122
913	0.89399	0.89931	0.90177	0.90260	0.90526	0.90812	0.91129
914	0.89408	0.89940	0.90186	0.90268	0.90534	0.90820	0.91137
915	0.89417	0.89949	0.90195	0.90277	0.90542	0.90828	0.91145
916	0.89427	0.89958	0.90203	0.90285	0.90550	0.90836	0.91152
917	0.89435	0.89967	0.90212	0.90294	0.90559	0.90844	0.91160
918	0.89444	0.89975	0.90220	0.90302	0.90567	0.90852	0.91168
919	0.89454	0.89984	0.90229	0.90311	0.90575	0.90860	0.91175
920	0.89463	0.89993	0.90237	0.90319	0.90583	0.90868	0.91183
921	0.89473	0.90002	0.90246	0.90328	0.90592	0.90876	0.91190
922	0.89483	0.90010	0.90255	0.90336	0.90600	0.90884	0.91198
923	0.89492	0.90019	0.90263	0.90344	0.90608	0.90892	0.91206
924	0.89502	0.90028	0.90272	0.90353	0.90616	0.90899	0.91213
925	0.89512	0.90037	0.90280	0.90361	0.90624	0.90907	0.91221
926	0.89520	0.90045	0.90288	0.90369	0.90632	0.90915	0.91228
927	0.89530	0.90053	0.90297	0.90378	0.90640	0.90923	0.91236
928	0.89539	0.90062	0.90305	0.90386	0.90648	0.90931	0.91243
929	0.89548	0.90071	0.90313	0.90394	0.90656	0.90938	0.91251
930	0.89557	0.90079	0.90322	0.90403	0.90664	0.90946	0.91258
931	0.89566	0.90088	0.90330	0.90411	0.90672	0.90954	0.91265
932	0.89574	0.90097	0.90338	0.90419	0.90680	0.90962	0.91273
933	0.89584	0.90105	0.90347	0.90427	0.90688	0.90969	0.91280
934	0.89593	0.90114	0.90355	0.90435	0.90696	0.90977	0.91288
935	0.89603	0.90123	0.90364	0.90444	0.90704	0.90985	0.91295
936	0.89612	0.90131	0.90372	0.90452	0.90712	0.90992	0.91302
937	0.89621	0.90139	0.90380	0.90461	0.90720	0.91000	0.91310
938	0.89630	0.90148	0.90388	0.90469	0.90728	0.91008	0.91317
939	0.89639	0.90157	0.90397	0.90477	0.90736	0.91015	0.91325
940	0.89648	0.90165	0.90405	0.90485	0.90743	0.91023	0.91332
941	0.89656	0.90173	0.90413	0.90493	0.90751	0.91030	0.91339
942	0.89665	0.90181	0.90421	0.90501	0.90759	0.91038	0.91346
943	0.89674	0.90189	0.90430	0.90509	0.90767	0.91046	0.91354
944	0.89682	0.90198	0.90437	0.90517	0.90775	0.91053	0.91361
945	0.89690	0.90206	0.90446	0.90525	0.90783	0.91061	0.91368
946	0.89700	0.90215	0.90454	0.90534	0.90790	0.91068	0.91375
947	0.89708	0.90223	0.90462	0.90542	0.90798	0.91076	0.91383
948	0.89717	0.90232	0.90470	0.90550	0.90806	0.91083	0.91390
949	0.89726	0.90240	0.90478	0.90558	0.90814	0.91090	0.91397
950	0.89735	0.90248	0.90486	0.90565	0.90821	0.91098	0.91404
951	0.89744	0.90256	0.90494	0.90573	0.90829	0.91106	0.91411
952	0.89753	0.90264	0.90502	0.90581	0.90837	0.91113	0.91418
953	0.89762	0.90273	0.90510	0.90589	0.90845	0.91120	0.91426
954	0.89771	0.90281	0.90518	0.90597	0.90852	0.91128	0.91433
955	0.89779	0.90289	0.90526	0.90605	0.90860	0.91135	0.91440
956	0.89788	0.90297	0.90534	0.90613	0.90868	0.91143	0.91447
957	0.89797	0.90306	0.90542	0.90620	0.90875	0.91150	0.91454
958	0.89805	0.90314	0.90549	0.90628	0.90883	0.91158	0.91461
959	0.89813	0.90322	0.90558	0.90636	0.90890	0.91165	0.91468
960	0.89821	0.90330	0.90566	0.90644	0.90898	0.91172	0.91475
961	0.89829	0.90338	0.90574	0.90652	0.90905	0.91180	0.91482
962	0.89838	0.90347	0.90581	0.90660	0.90913	0.91187	0.91489
963	0.89847	0.90355	0.90589	0.90667	0.90921	0.91194	0.91496
964	0.89856	0.90362	0.90597	0.90676	0.90928	0.91202	0.91503
965	0.89865	0.90370	0.90605	0.90683	0.90936	0.91209	0.91510
966	0.89873	0.90378	0.90613	0.90691	0.90943	0.91216	0.91517

N	0.1%	0.5%	1%	1.25%	2.5%	5%	10%
967	0.89882	0.90386	0.90621	0.90699	0.90951	0.91223	0.91524
968	0.89890	0.90395	0.90628	0.90706	0.90958	0.91230	0.91531
969	0.89898	0.90402	0.90636	0.90714	0.90966	0.91238	0.91538
970	0.89907	0.90410	0.90644	0.90722	0.90973	0.91245	0.91545
971	0.89916	0.90419	0.90652	0.90729	0.90980	0.91252	0.91552
972	0.89924	0.90427	0.90660	0.90737	0.90988	0.91259	0.91559
973	0.89932	0.90435	0.90667	0.90745	0.90995	0.91266	0.91566
974	0.89941	0.90443	0.90675	0.90752	0.91003	0.91274	0.91573
975	0.89949	0.90450	0.90683	0.90760	0.91010	0.91281	0.91579
976	0.89957	0.90458	0.90690	0.90768	0.91017	0.91288	0.91586
977	0.89966	0.90466	0.90698	0.90775	0.91025	0.91295	0.91593
978	0.89974	0.90474	0.90705	0.90783	0.91032	0.91302	0.91600
979	0.89983	0.90482	0.90713	0.90790	0.91040	0.91309	0.91607
980	0.89992	0.90490	0.90721	0.90798	0.91047	0.91316	0.91614
981	0.90000	0.90497	0.90728	0.90805	0.91054	0.91323	0.91621
982	0.90007	0.90505	0.90736	0.90813	0.91062	0.91330	0.91627
983	0.90016	0.90513	0.90743	0.90820	0.91069	0.91337	0.91634
984	0.90025	0.90521	0.90751	0.90828	0.91076	0.91344	0.91641
985	0.90034	0.90528	0.90759	0.90836	0.91084	0.91351	0.91648
986	0.90042	0.90536	0.90766	0.90843	0.91091	0.91358	0.91654
987	0.90051	0.90544	0.90774	0.90851	0.91098	0.91365	0.91661
988	0.90060	0.90552	0.90781	0.90858	0.91105	0.91372	0.91668
989	0.90068	0.90560	0.90789	0.90866	0.91112	0.91379	0.91674
990	0.90076	0.90567	0.90796	0.90873	0.91120	0.91386	0.91681
991	0.90084	0.90575	0.90804	0.90880	0.91127	0.91393	0.91688
992	0.90092	0.90582	0.90812	0.90888	0.91134	0.91400	0.91694
993	0.90100	0.90590	0.90819	0.90895	0.91141	0.91407	0.91701
994	0.90108	0.90598	0.90827	0.90903	0.91149	0.91414	0.91708
995	0.90116	0.90606	0.90834	0.90910	0.91156	0.91421	0.91714
996	0.90124	0.90613	0.90841	0.90918	0.91163	0.91428	0.91721
997	0.90132	0.90620	0.90849	0.90925	0.91170	0.91435	0.91728
998	0.90140	0.90628	0.90856	0.90932	0.91177	0.91441	0.91734
999	0.90148	0.90636	0.90863	0.90939	0.91184	0.91448	0.91741
1000	0.90155	0.90644	0.90870	0.90947	0.91191	0.91455	0.91747

Table 4 – *GM9(6H3L or 3H6L)*. Critical values of upper significance levels for the Grubbs multiple-9 outlier test, applicable in situations where the six highest and three lowest or three highest and six lowest observations are simultaneously identified as outliers, denoted as *GM9(6H3L)* and *GM9(3H6L)*, respectively.

N	0.1%	0.5%	1%	1.25%	2.5%	5%	10%
11	0.00000	0.00000	0.00000	0.00000	0.00000	0.00001	0.00004
12	0.00001	0.00004	0.00008	0.00010	0.00021	0.00044	0.00097
13	0.00012	0.00038	0.00063	0.00074	0.00124	0.00211	0.00369
14	0.00058	0.00140	0.00206	0.00234	0.00349	0.00531	0.00827
15	0.00161	0.00330	0.00455	0.00505	0.00704	0.00998	0.01447
16	0.00335	0.00618	0.00812	0.00888	0.01181	0.01596	0.02200
17	0.00586	0.00997	0.01268	0.01373	0.01766	0.02302	0.03058
18	0.00914	0.01467	0.01818	0.01950	0.02442	0.03099	0.03996
19	0.01309	0.02011	0.02442	0.02603	0.03194	0.03961	0.04992
20	0.01770	0.02623	0.03133	0.03323	0.04008	0.04879	0.06035
21	0.02291	0.03285	0.03875	0.04090	0.04862	0.05836	0.07104
22	0.02859	0.04007	0.04663	0.04904	0.05758	0.06822	0.08193
23	0.03484	0.04756	0.05481	0.05746	0.06676	0.07827	0.09289
24	0.04135	0.05546	0.06330	0.06615	0.07618	0.08843	0.10390
25	0.04818	0.06348	0.07198	0.07504	0.08571	0.09865	0.11488
26	0.05525	0.07169	0.08081	0.08406	0.09529	0.10891	0.12578
27	0.06253	0.08012	0.08974	0.09311	0.10489	0.11910	0.13658
28	0.07004	0.08864	0.09865	0.10219	0.11450	0.12921	0.14723
29	0.07769	0.09711	0.10756	0.11127	0.12408	0.13922	0.15775
30	0.08531	0.10563	0.11650	0.12032	0.13355	0.14914	0.16806
31	0.09305	0.11416	0.12537	0.12938	0.14288	0.15890	0.17821
32	0.10074	0.12273	0.13428	0.13832	0.15223	0.16857	0.18817
33	0.10860	0.13118	0.14308	0.14722	0.16146	0.17809	0.19795
34	0.11654	0.13967	0.15176	0.15605	0.17052	0.18740	0.20758
35	0.12419	0.14802	0.16045	0.16474	0.17946	0.19657	0.21697
36	0.13201	0.15633	0.16893	0.17336	0.18831	0.20557	0.22613
37	0.13954	0.16451	0.17731	0.18180	0.19699	0.21439	0.23512
38	0.14721	0.17255	0.18562	0.19020	0.20546	0.22303	0.24388
39	0.15479	0.18054	0.19380	0.19836	0.21376	0.23151	0.25252
40	0.16207	0.18840	0.20178	0.20642	0.22199	0.23987	0.26094
41	0.16961	0.19616	0.20967	0.21434	0.23007	0.24801	0.26919

N	0.1%	0.5%	1%	1.25%	2.5%	5%	10%
42	0.17693	0.20381	0.21740	0.22212	0.23801	0.25603	0.27724
43	0.18415	0.21138	0.22508	0.22982	0.24573	0.26388	0.28512
44	0.19132	0.21881	0.23260	0.23737	0.25339	0.27154	0.29283
45	0.19845	0.22610	0.24001	0.24479	0.26087	0.27908	0.30037
46	0.20548	0.23324	0.24723	0.25201	0.26818	0.28644	0.30773
47	0.21231	0.24040	0.25438	0.25917	0.27541	0.29364	0.31495
48	0.21904	0.24734	0.26142	0.26629	0.28242	0.30070	0.32201
49	0.22581	0.25418	0.26830	0.27315	0.28935	0.30762	0.32890
50	0.23244	0.26096	0.27509	0.27993	0.29613	0.31439	0.33567
51	0.23899	0.26757	0.28172	0.28660	0.30280	0.32105	0.34227
52	0.24551	0.27402	0.28822	0.29314	0.30933	0.32756	0.34872
53	0.25195	0.28047	0.29464	0.29952	0.31572	0.33395	0.35506
54	0.25797	0.28681	0.30097	0.30583	0.32199	0.34024	0.36127
55	0.26415	0.29293	0.30714	0.31202	0.32821	0.34640	0.36736
56	0.27008	0.29895	0.31322	0.31808	0.33425	0.35243	0.37332
57	0.27609	0.30499	0.31921	0.32408	0.34020	0.35831	0.37915
58	0.28197	0.31092	0.32502	0.32990	0.34604	0.36408	0.38485
59	0.28764	0.31671	0.33085	0.33569	0.35175	0.36974	0.39047
60	0.29338	0.32237	0.33653	0.34134	0.35737	0.37532	0.39596
61	0.29892	0.32798	0.34210	0.34692	0.36290	0.38079	0.40134
62	0.30445	0.33343	0.34753	0.35236	0.36835	0.38615	0.40664
63	0.30972	0.33880	0.35289	0.35770	0.37368	0.39143	0.41179
64	0.31526	0.34408	0.35815	0.36295	0.37885	0.39658	0.41687
65	0.32045	0.34927	0.36336	0.36813	0.38399	0.40163	0.42186
66	0.32544	0.35443	0.36846	0.37320	0.38902	0.40664	0.42672
67	0.33062	0.35948	0.37348	0.37818	0.39396	0.41151	0.43151
68	0.33573	0.36443	0.37837	0.38310	0.39885	0.41630	0.43621
69	0.34056	0.36936	0.38318	0.38794	0.40364	0.42102	0.44084
70	0.34551	0.37412	0.38797	0.39273	0.40834	0.42567	0.44538
71	0.35030	0.37893	0.39273	0.39743	0.41297	0.43020	0.44984
72	0.35504	0.38356	0.39731	0.40208	0.41750	0.43467	0.45421
73	0.35967	0.38814	0.40190	0.40658	0.42196	0.43905	0.45852
74	0.36426	0.39263	0.40638	0.41101	0.42635	0.44337	0.46274
75	0.36863	0.39703	0.41078	0.41540	0.43069	0.44764	0.46690
76	0.37299	0.40144	0.41508	0.41972	0.43493	0.45181	0.47096
77	0.37755	0.40571	0.41934	0.42396	0.43911	0.45589	0.47500
78	0.38217	0.40992	0.42357	0.42815	0.44321	0.45991	0.47894
79	0.38636	0.41415	0.42769	0.43226	0.44728	0.46392	0.48282
80	0.39045	0.41823	0.43174	0.43630	0.45125	0.46782	0.48663
81	0.39451	0.42229	0.43575	0.44024	0.45515	0.47166	0.49038
82	0.39872	0.42631	0.43966	0.44418	0.45903	0.47544	0.49408
83	0.40277	0.43020	0.44352	0.44804	0.46282	0.47918	0.49770
84	0.40659	0.43405	0.44734	0.45183	0.46659	0.48284	0.50127
85	0.41043	0.43787	0.45112	0.45559	0.47028	0.48646	0.50480
86	0.41436	0.44164	0.45480	0.45927	0.47390	0.49000	0.50825
87	0.41803	0.44531	0.45846	0.46291	0.47747	0.49352	0.51166
88	0.42186	0.44898	0.46210	0.46650	0.48099	0.49694	0.51501
89	0.42544	0.45261	0.46566	0.47004	0.48445	0.50032	0.51828
90	0.42899	0.45624	0.46916	0.47351	0.48788	0.50366	0.52154
91	0.43272	0.45967	0.47264	0.47700	0.49126	0.50698	0.52475
92	0.43636	0.46313	0.47602	0.48035	0.49455	0.51024	0.52791
93	0.43989	0.46657	0.47935	0.48367	0.49783	0.51341	0.53102
94	0.44343	0.46995	0.48265	0.48696	0.50104	0.51654	0.53408
95	0.44665	0.47323	0.48589	0.49018	0.50421	0.51965	0.53710
96	0.45003	0.47656	0.48916	0.49341	0.50738	0.52273	0.54006
97	0.45347	0.47979	0.49231	0.49656	0.51047	0.52574	0.54300
98	0.45674	0.48295	0.49546	0.49968	0.51351	0.52873	0.54590
99	0.45996	0.48607	0.49854	0.50276	0.51651	0.53165	0.54874
100	0.46313	0.48915	0.50158	0.50577	0.51948	0.53455	0.55155
101	0.46621	0.49225	0.50457	0.50874	0.52242	0.53739	0.55433
102	0.46933	0.49525	0.50757	0.51170	0.52527	0.54023	0.55706
103	0.47247	0.49827	0.51049	0.51463	0.52813	0.54300	0.55976
104	0.47544	0.50119	0.51338	0.51751	0.53094	0.54573	0.56242
105	0.47847	0.50412	0.51625	0.52033	0.53372	0.54843	0.56505
106	0.48148	0.50698	0.51910	0.52317	0.53648	0.55108	0.56761
107	0.48430	0.50982	0.52188	0.52593	0.53921	0.55375	0.57017
108	0.48715	0.51263	0.52465	0.52867	0.54187	0.55636	0.57269
109	0.49021	0.51534	0.52737	0.53140	0.54446	0.55893	0.57516
110	0.49303	0.51815	0.53001	0.53400	0.54707	0.56145	0.57762
111	0.49576	0.52087	0.53260	0.53661	0.54963	0.56395	0.58004
112	0.49860	0.52349	0.53527	0.53922	0.55217	0.56642	0.58242
113	0.50143	0.52613	0.53781	0.54177	0.55466	0.56886	0.58479
114	0.50410	0.52871	0.54038	0.54429	0.55715	0.57127	0.58711
115	0.50666	0.53123	0.54287	0.54677	0.55960	0.57364	0.58941

N	0.1%	0.5%	1%	1.25%	2.5%	5%	10%
116	0.50929	0.53375	0.54536	0.54926	0.56199	0.57598	0.59168
117	0.51194	0.53622	0.54784	0.55170	0.56439	0.57829	0.59391
118	0.51442	0.53873	0.55026	0.55414	0.56675	0.58056	0.59613
119	0.51700	0.54119	0.55267	0.55650	0.56907	0.58283	0.59830
120	0.51946	0.54360	0.55505	0.55886	0.57137	0.58506	0.60046
121	0.52206	0.54602	0.55738	0.56118	0.57363	0.58725	0.60260
122	0.52445	0.54842	0.55968	0.56348	0.57588	0.58945	0.60470
123	0.52693	0.55069	0.56195	0.56573	0.57811	0.59160	0.60680
124	0.52940	0.55304	0.56424	0.56799	0.58033	0.59373	0.60886
125	0.53174	0.55530	0.56643	0.57020	0.58249	0.59586	0.61091
126	0.53399	0.55760	0.56865	0.57239	0.58463	0.59793	0.61292
127	0.53632	0.55980	0.57086	0.57456	0.58674	0.59999	0.61492
128	0.53864	0.56203	0.57302	0.57673	0.58881	0.60204	0.61688
129	0.54087	0.56421	0.57515	0.57880	0.59089	0.60405	0.61882
130	0.54310	0.56633	0.57723	0.58091	0.59295	0.60603	0.62073
131	0.54543	0.56847	0.57935	0.58298	0.59497	0.60801	0.62264
132	0.54757	0.57056	0.58143	0.58505	0.59696	0.60996	0.62452
133	0.54980	0.57267	0.58346	0.58708	0.59895	0.61187	0.62637
134	0.55205	0.57472	0.58546	0.58910	0.60090	0.61378	0.62821
135	0.55415	0.57680	0.58750	0.59108	0.60284	0.61566	0.63003
136	0.55623	0.57879	0.58948	0.59306	0.60476	0.61752	0.63181
137	0.55845	0.58081	0.59146	0.59500	0.60664	0.61935	0.63359
138	0.56054	0.58278	0.59338	0.59692	0.60851	0.62117	0.63535
139	0.56253	0.58472	0.59530	0.59883	0.61037	0.62297	0.63709
140	0.56453	0.58666	0.59716	0.60073	0.61221	0.62475	0.63881
141	0.56655	0.58858	0.59905	0.60259	0.61404	0.62652	0.64051
142	0.56856	0.59048	0.60092	0.60443	0.61583	0.62827	0.64219
143	0.57053	0.59234	0.60279	0.60626	0.61762	0.63000	0.64386
144	0.57250	0.59426	0.60460	0.60809	0.61940	0.63172	0.64551
145	0.57447	0.59609	0.60638	0.60985	0.62116	0.63341	0.64713
146	0.57639	0.59794	0.60820	0.61163	0.62287	0.63509	0.64874
147	0.57818	0.59975	0.60996	0.61339	0.62459	0.63673	0.65034
148	0.58010	0.60154	0.61170	0.61511	0.62627	0.63838	0.65192
149	0.58194	0.60333	0.61344	0.61683	0.62795	0.64000	0.65348
150	0.58377	0.60510	0.61517	0.61855	0.62959	0.64161	0.65503
151	0.58553	0.60681	0.61686	0.62022	0.63123	0.64319	0.65656
152	0.58742	0.60854	0.61858	0.62190	0.63286	0.64477	0.65808
153	0.58918	0.61026	0.62022	0.62358	0.63448	0.64633	0.65957
154	0.59093	0.61192	0.62189	0.62521	0.63608	0.64787	0.66106
155	0.59264	0.61364	0.62353	0.62684	0.63765	0.64941	0.66254
156	0.59438	0.61527	0.62515	0.62847	0.63920	0.65092	0.66400
157	0.59620	0.61688	0.62673	0.63007	0.64076	0.65243	0.66545
158	0.59784	0.61851	0.62831	0.63161	0.64229	0.65390	0.66688
159	0.59956	0.62010	0.62989	0.63317	0.64381	0.65538	0.66830
160	0.60127	0.62172	0.63147	0.63471	0.64532	0.65683	0.66971
161	0.60293	0.62332	0.63301	0.63622	0.64681	0.65828	0.67110
162	0.60463	0.62487	0.63451	0.63771	0.64828	0.65971	0.67248
163	0.60626	0.62642	0.63604	0.63923	0.64972	0.66112	0.67385
164	0.60790	0.62798	0.63755	0.64072	0.65119	0.66252	0.67519
165	0.60945	0.62951	0.63902	0.64223	0.65262	0.66392	0.67653
166	0.61098	0.63101	0.64049	0.64368	0.65404	0.66531	0.67786
167	0.61265	0.63250	0.64194	0.64513	0.65546	0.66666	0.67917
168	0.61418	0.63400	0.64338	0.64656	0.65684	0.66802	0.68048
169	0.61574	0.63546	0.64483	0.64799	0.65822	0.66936	0.68178
170	0.61725	0.63692	0.64628	0.64940	0.65959	0.67069	0.68306
171	0.61879	0.63842	0.64768	0.65080	0.66096	0.67200	0.68432
172	0.62031	0.63984	0.64908	0.65220	0.66230	0.67331	0.68557
173	0.62175	0.64126	0.65046	0.65357	0.66365	0.67461	0.68681
174	0.62320	0.64266	0.65185	0.65493	0.66498	0.67590	0.68805
175	0.62468	0.64407	0.65323	0.65628	0.66628	0.67718	0.68928
176	0.62609	0.64543	0.65456	0.65762	0.66759	0.67842	0.69050
177	0.62750	0.64683	0.65591	0.65893	0.66888	0.67968	0.69170
178	0.62890	0.64818	0.65722	0.66023	0.67015	0.68093	0.69289
179	0.63030	0.64955	0.65851	0.66154	0.67141	0.68216	0.69408
180	0.63169	0.65092	0.65984	0.66285	0.67268	0.68338	0.69525
181	0.63307	0.65224	0.66112	0.66413	0.67391	0.68458	0.69639
182	0.63456	0.65354	0.66238	0.66539	0.67515	0.68577	0.69755
183	0.63599	0.65485	0.66368	0.66664	0.67637	0.68696	0.69869
184	0.63732	0.65613	0.66495	0.66791	0.67760	0.68813	0.69982
185	0.63862	0.65740	0.66618	0.66917	0.67880	0.68930	0.70095
186	0.64001	0.65868	0.66742	0.67038	0.67998	0.69045	0.70206
187	0.64131	0.65994	0.66866	0.67158	0.68117	0.69159	0.70315
188	0.64261	0.66123	0.66989	0.67281	0.68234	0.69273	0.70425
189	0.64395	0.66244	0.67108	0.67401	0.68351	0.69385	0.70533

N	0.1%	0.5%	1%	1.25%	2.5%	5%	10%
190	0.64521	0.66369	0.67227	0.67520	0.68467	0.69496	0.70641
191	0.64650	0.66486	0.67348	0.67637	0.68583	0.69608	0.70748
192	0.64770	0.66606	0.67465	0.67755	0.68696	0.69718	0.70853
193	0.64897	0.66726	0.67581	0.67870	0.68808	0.69828	0.70959
194	0.65020	0.66845	0.67697	0.67983	0.68921	0.69936	0.71064
195	0.65149	0.66963	0.67814	0.68099	0.69032	0.70043	0.71167
196	0.65268	0.67078	0.67929	0.68213	0.69142	0.70150	0.71269
197	0.65393	0.67193	0.68044	0.68325	0.69250	0.70255	0.71371
198	0.65515	0.67307	0.68156	0.68438	0.69358	0.70360	0.71472
199	0.65633	0.67419	0.68268	0.68547	0.69466	0.70464	0.71572
200	0.65751	0.67534	0.68378	0.68656	0.69573	0.70568	0.71671
201	0.65861	0.67647	0.68486	0.68766	0.69680	0.70670	0.71769
202	0.65990	0.67761	0.68598	0.68875	0.69784	0.70771	0.71867
203	0.66107	0.67874	0.68703	0.68981	0.69890	0.70873	0.71965
204	0.66228	0.67984	0.68812	0.69089	0.69991	0.70972	0.72062
205	0.66337	0.68093	0.68920	0.69196	0.70094	0.71072	0.72157
206	0.66448	0.68202	0.69024	0.69299	0.70196	0.71171	0.72251
207	0.66562	0.68310	0.69130	0.69403	0.70298	0.71268	0.72345
208	0.66676	0.68419	0.69234	0.69506	0.70398	0.71366	0.72439
209	0.66787	0.68527	0.69338	0.69611	0.70499	0.71463	0.72532
210	0.66898	0.68630	0.69438	0.69713	0.70597	0.71558	0.72624
211	0.67006	0.68734	0.69543	0.69812	0.70695	0.71653	0.72715
212	0.67114	0.68838	0.69646	0.69913	0.70793	0.71747	0.72806
213	0.67225	0.68942	0.69746	0.70013	0.70889	0.71840	0.72895
214	0.67335	0.69045	0.69846	0.70111	0.70986	0.71933	0.72985
215	0.67439	0.69146	0.69945	0.70211	0.71082	0.72026	0.73074
216	0.67544	0.69249	0.70043	0.70308	0.71177	0.72118	0.73161
217	0.67658	0.69350	0.70141	0.70405	0.71270	0.72208	0.73248
218	0.67757	0.69450	0.70238	0.70502	0.71363	0.72299	0.73336
219	0.67865	0.69548	0.70335	0.70598	0.71455	0.72388	0.73421
220	0.67966	0.69645	0.70431	0.70694	0.71548	0.72476	0.73507
221	0.68066	0.69743	0.70524	0.70788	0.71639	0.72564	0.73591
222	0.68169	0.69838	0.70618	0.70880	0.71729	0.72652	0.73676
223	0.68276	0.69935	0.70711	0.70972	0.71820	0.72740	0.73759
224	0.68371	0.70031	0.70803	0.71063	0.71909	0.72826	0.73843
225	0.68474	0.70124	0.70896	0.71155	0.71997	0.72912	0.73925
226	0.68573	0.70220	0.70987	0.71245	0.72086	0.72997	0.74007
227	0.68670	0.70311	0.71078	0.71335	0.72173	0.73082	0.74089
228	0.68770	0.70404	0.71167	0.71425	0.72259	0.73166	0.74169
229	0.68861	0.70495	0.71258	0.71515	0.72345	0.73249	0.74249
230	0.68961	0.70587	0.71347	0.71602	0.72430	0.73332	0.74330
231	0.69051	0.70676	0.71435	0.71690	0.72516	0.73414	0.74408
232	0.69141	0.70767	0.71523	0.71776	0.72599	0.73495	0.74487
233	0.69235	0.70853	0.71613	0.71863	0.72683	0.73576	0.74566
234	0.69328	0.70944	0.71699	0.71950	0.72766	0.73657	0.74643
235	0.69422	0.71032	0.71785	0.72034	0.72849	0.73737	0.74720
236	0.69518	0.71120	0.71871	0.72118	0.72930	0.73816	0.74796
237	0.69612	0.71207	0.71955	0.72203	0.73011	0.73894	0.74872
238	0.69708	0.71293	0.72038	0.72286	0.73092	0.73972	0.74948
239	0.69801	0.71382	0.72121	0.72369	0.73174	0.74050	0.75022
240	0.69888	0.71465	0.72204	0.72451	0.73255	0.74128	0.75097
241	0.69977	0.71551	0.72287	0.72533	0.73334	0.74205	0.75171
242	0.70060	0.71634	0.72368	0.72613	0.73412	0.74281	0.75243
243	0.70157	0.71718	0.72450	0.72694	0.73490	0.74357	0.75316
244	0.70245	0.71803	0.72532	0.72774	0.73568	0.74432	0.75388
245	0.70331	0.71886	0.72614	0.72855	0.73645	0.74507	0.75460
246	0.70414	0.71967	0.72694	0.72935	0.73723	0.74581	0.75531
247	0.70497	0.72047	0.72771	0.73014	0.73800	0.74655	0.75603
248	0.70585	0.72130	0.72851	0.73092	0.73876	0.74729	0.75674
249	0.70666	0.72211	0.72928	0.73168	0.73951	0.74802	0.75744
250	0.70752	0.72291	0.73008	0.73245	0.74026	0.74873	0.75814
251	0.70834	0.72370	0.73084	0.73323	0.74101	0.74946	0.75883
252	0.70922	0.72450	0.73161	0.73399	0.74175	0.75019	0.75951
253	0.71004	0.72531	0.73238	0.73474	0.74249	0.75089	0.76019
254	0.71089	0.72608	0.73313	0.73550	0.74322	0.75160	0.76087
255	0.71172	0.72684	0.73390	0.73624	0.74395	0.75229	0.76154
256	0.71256	0.72762	0.73467	0.73697	0.74467	0.75299	0.76221
257	0.71341	0.72838	0.73539	0.73772	0.74538	0.75368	0.76288
258	0.71423	0.72913	0.73613	0.73845	0.74611	0.75438	0.76354
259	0.71504	0.72989	0.73685	0.73918	0.74681	0.75506	0.76420
260	0.71579	0.73064	0.73758	0.73991	0.74751	0.75574	0.76485
261	0.71659	0.73138	0.73831	0.74063	0.74821	0.75642	0.76551
262	0.71737	0.73212	0.73904	0.74135	0.74889	0.75708	0.76615
263	0.71818	0.73285	0.73977	0.74206	0.74959	0.75775	0.76679

N	0.1%	0.5%	1%	1.25%	2.5%	5%	10%
264	0.71894	0.73358	0.74048	0.74278	0.75029	0.75841	0.76743
265	0.71975	0.73431	0.74118	0.74348	0.75097	0.75907	0.76806
266	0.72052	0.73504	0.74190	0.74418	0.75164	0.75973	0.76870
267	0.72125	0.73576	0.74260	0.74488	0.75232	0.76038	0.76932
268	0.72201	0.73647	0.74329	0.74560	0.75299	0.76103	0.76994
269	0.72275	0.73719	0.74399	0.74626	0.75365	0.76167	0.77056
270	0.72350	0.73790	0.74468	0.74695	0.75431	0.76230	0.77118
271	0.72421	0.73862	0.74536	0.74763	0.75497	0.76294	0.77178
272	0.72496	0.73931	0.74606	0.74830	0.75564	0.76358	0.77240
273	0.72566	0.74000	0.74674	0.74897	0.75628	0.76421	0.77300
274	0.72636	0.74071	0.74740	0.74965	0.75693	0.76483	0.77360
275	0.72709	0.74139	0.74808	0.75030	0.75757	0.76544	0.77419
276	0.72784	0.74208	0.74873	0.75097	0.75821	0.76607	0.77479
277	0.72862	0.74277	0.74940	0.75163	0.75885	0.76668	0.77538
278	0.72932	0.74343	0.75007	0.75228	0.75949	0.76729	0.77597
279	0.73003	0.74411	0.75071	0.75293	0.76012	0.76790	0.77655
280	0.73075	0.74478	0.75135	0.75358	0.76074	0.76851	0.77713
281	0.73143	0.74545	0.75202	0.75421	0.76136	0.76910	0.77771
282	0.73215	0.74611	0.75266	0.75486	0.76197	0.76970	0.77829
283	0.73282	0.74680	0.75330	0.75549	0.76259	0.77030	0.77886
284	0.73355	0.74744	0.75393	0.75611	0.76320	0.77088	0.77942
285	0.73422	0.74811	0.75457	0.75674	0.76381	0.77147	0.77998
286	0.73487	0.74876	0.75520	0.75736	0.76441	0.77205	0.78055
287	0.73553	0.74938	0.75582	0.75798	0.76500	0.77262	0.78110
288	0.73619	0.75003	0.75645	0.75860	0.76560	0.77320	0.78166
289	0.73687	0.75067	0.75707	0.75922	0.76620	0.77378	0.78221
290	0.73749	0.75130	0.75766	0.75983	0.76679	0.77435	0.78276
291	0.73816	0.75192	0.75829	0.76043	0.76739	0.77492	0.78330
292	0.73880	0.75255	0.75891	0.76105	0.76797	0.77549	0.78385
293	0.73945	0.75316	0.75951	0.76164	0.76855	0.77605	0.78438
294	0.74014	0.75377	0.76011	0.76224	0.76913	0.77661	0.78491
295	0.74080	0.75440	0.76073	0.76282	0.76970	0.77716	0.78544
296	0.74141	0.75503	0.76132	0.76341	0.77028	0.77771	0.78598
297	0.74208	0.75563	0.76191	0.76401	0.77085	0.77826	0.78650
298	0.74274	0.75622	0.76248	0.76460	0.77141	0.77880	0.78703
299	0.74341	0.75682	0.76307	0.76518	0.77198	0.77935	0.78755
300	0.74403	0.75742	0.76366	0.76576	0.77253	0.77988	0.78806
301	0.74473	0.75800	0.76424	0.76632	0.77309	0.78042	0.78857
302	0.74533	0.75860	0.76482	0.76689	0.77364	0.78095	0.78908
303	0.74596	0.75919	0.76538	0.76746	0.77418	0.78149	0.78959
304	0.74658	0.75975	0.76595	0.76802	0.77473	0.78201	0.79010
305	0.74715	0.76034	0.76651	0.76858	0.77527	0.78254	0.79061
306	0.74777	0.76091	0.76709	0.76915	0.77582	0.78306	0.79111
307	0.74838	0.76149	0.76764	0.76970	0.77636	0.78358	0.79161
308	0.74892	0.76206	0.76820	0.77025	0.77690	0.78410	0.79210
309	0.74954	0.76262	0.76877	0.77080	0.77743	0.78461	0.79260
310	0.75011	0.76319	0.76931	0.77134	0.77795	0.78512	0.79309
311	0.75073	0.76374	0.76985	0.77188	0.77849	0.78562	0.79357
312	0.75131	0.76431	0.77039	0.77243	0.77901	0.78613	0.79406
313	0.75184	0.76486	0.77094	0.77295	0.77953	0.78663	0.79454
314	0.75247	0.76542	0.77148	0.77348	0.78005	0.78714	0.79503
315	0.75307	0.76598	0.77201	0.77403	0.78056	0.78764	0.79550
316	0.75363	0.76651	0.77255	0.77455	0.78109	0.78813	0.79598
317	0.75420	0.76706	0.77307	0.77508	0.78160	0.78863	0.79645
318	0.75477	0.76761	0.77361	0.77560	0.78210	0.78912	0.79692
319	0.75530	0.76816	0.77413	0.77613	0.78261	0.78961	0.79739
320	0.75593	0.76868	0.77466	0.77666	0.78312	0.79009	0.79786
321	0.75648	0.76923	0.77518	0.77717	0.78362	0.79058	0.79832
322	0.75704	0.76975	0.77570	0.77768	0.78412	0.79106	0.79878
323	0.75759	0.77029	0.77622	0.77819	0.78462	0.79154	0.79924
324	0.75815	0.77081	0.77673	0.77869	0.78510	0.79201	0.79969
325	0.75872	0.77135	0.77723	0.77921	0.78559	0.79248	0.80014
326	0.75926	0.77188	0.77773	0.77970	0.78608	0.79295	0.80060
327	0.75980	0.77240	0.77824	0.78019	0.78655	0.79342	0.80105
328	0.76034	0.77290	0.77875	0.78069	0.78704	0.79389	0.80149
329	0.76091	0.77343	0.77924	0.78118	0.78752	0.79435	0.80194
330	0.76144	0.77393	0.77975	0.78168	0.78799	0.79481	0.80238
331	0.76197	0.77444	0.78023	0.78217	0.78847	0.79527	0.80282
332	0.76251	0.77495	0.78073	0.78267	0.78894	0.79572	0.80326
333	0.76304	0.77545	0.78121	0.78316	0.78941	0.79618	0.80370
334	0.76355	0.77596	0.78171	0.78363	0.78989	0.79664	0.80413
335	0.76408	0.77646	0.78220	0.78411	0.79035	0.79708	0.80456
336	0.76459	0.77695	0.78268	0.78458	0.79082	0.79753	0.80500
337	0.76512	0.77745	0.78316	0.78506	0.79128	0.79798	0.80543

N	0.1%	0.5%	1%	1.25%	2.5%	5%	10%
338	0.76563	0.77793	0.78364	0.78553	0.79174	0.79842	0.80585
339	0.76614	0.77842	0.78411	0.78600	0.79220	0.79886	0.80627
340	0.76667	0.77891	0.78458	0.78647	0.79265	0.79930	0.80669
341	0.76721	0.77939	0.78504	0.78693	0.79310	0.79974	0.80712
342	0.76770	0.77988	0.78551	0.78741	0.79356	0.80017	0.80753
343	0.76821	0.78034	0.78598	0.78787	0.79401	0.80061	0.80795
344	0.76869	0.78084	0.78644	0.78833	0.79445	0.80104	0.80836
345	0.76920	0.78132	0.78690	0.78879	0.79489	0.80147	0.80877
346	0.76968	0.78178	0.78737	0.78925	0.79534	0.80190	0.80918
347	0.77018	0.78226	0.78783	0.78971	0.79578	0.80233	0.80959
348	0.77069	0.78272	0.78830	0.79016	0.79622	0.80275	0.81000
349	0.77118	0.78319	0.78875	0.79061	0.79665	0.80317	0.81040
350	0.77167	0.78367	0.78921	0.79106	0.79708	0.80359	0.81080
351	0.77219	0.78415	0.78965	0.79151	0.79751	0.80400	0.81120
352	0.77263	0.78461	0.79010	0.79195	0.79794	0.80442	0.81160
353	0.77309	0.78505	0.79055	0.79239	0.79836	0.80482	0.81200
354	0.77360	0.78551	0.79100	0.79283	0.79879	0.80524	0.81239
355	0.77409	0.78594	0.79144	0.79327	0.79922	0.80564	0.81279
356	0.77457	0.78638	0.79189	0.79371	0.79964	0.80606	0.81318
357	0.77504	0.78684	0.79232	0.79413	0.80006	0.80646	0.81357
358	0.77549	0.78729	0.79275	0.79458	0.80048	0.80687	0.81395
359	0.77595	0.78774	0.79318	0.79501	0.80089	0.80727	0.81434
360	0.77642	0.78817	0.79361	0.79544	0.80131	0.80767	0.81472
361	0.77688	0.78862	0.79405	0.79587	0.80172	0.80807	0.81510
362	0.77734	0.78906	0.79447	0.79629	0.80213	0.80846	0.81548
363	0.77785	0.78950	0.79490	0.79672	0.80254	0.80886	0.81586
364	0.77830	0.78994	0.79532	0.79713	0.80295	0.80925	0.81624
365	0.77876	0.79038	0.79574	0.79755	0.80334	0.80963	0.81661
366	0.77921	0.79081	0.79617	0.79797	0.80374	0.81002	0.81699
367	0.77967	0.79124	0.79659	0.79837	0.80415	0.81041	0.81736
368	0.78010	0.79165	0.79700	0.79878	0.80455	0.81080	0.81774
369	0.78054	0.79207	0.79742	0.79918	0.80494	0.81119	0.81810
370	0.78097	0.79250	0.79783	0.79960	0.80533	0.81157	0.81847
371	0.78141	0.79292	0.79824	0.80001	0.80573	0.81195	0.81884
372	0.78186	0.79335	0.79865	0.80041	0.80613	0.81233	0.81920
373	0.78229	0.79377	0.79906	0.80081	0.80652	0.81271	0.81956
374	0.78276	0.79419	0.79946	0.80121	0.80691	0.81308	0.81992
375	0.78322	0.79458	0.79986	0.80162	0.80730	0.81346	0.82028
376	0.78364	0.79501	0.80027	0.80203	0.80768	0.81383	0.82064
377	0.78409	0.79541	0.80067	0.80242	0.80806	0.81420	0.82099
378	0.78453	0.79582	0.80106	0.80281	0.80845	0.81456	0.82135
379	0.78497	0.79624	0.80147	0.80321	0.80883	0.81493	0.82170
380	0.78539	0.79663	0.80186	0.80359	0.80921	0.81530	0.82205
381	0.78584	0.79704	0.80226	0.80398	0.80959	0.81567	0.82240
382	0.78625	0.79745	0.80264	0.80437	0.80997	0.81602	0.82274
383	0.78670	0.79783	0.80303	0.80476	0.81034	0.81638	0.82309
384	0.78707	0.79822	0.80342	0.80515	0.81070	0.81674	0.82344
385	0.78755	0.79863	0.80381	0.80553	0.81108	0.81710	0.82378
386	0.78796	0.79902	0.80418	0.80590	0.81145	0.81746	0.82412
387	0.78839	0.79941	0.80456	0.80627	0.81181	0.81782	0.82446
388	0.78879	0.79980	0.80494	0.80664	0.81218	0.81817	0.82480
389	0.78920	0.80020	0.80531	0.80701	0.81254	0.81852	0.82514
390	0.78963	0.80059	0.80570	0.80739	0.81291	0.81887	0.82548
391	0.79003	0.80097	0.80607	0.80776	0.81326	0.81922	0.82581
392	0.79044	0.80134	0.80644	0.80815	0.81362	0.81956	0.82614
393	0.79086	0.80173	0.80680	0.80851	0.81398	0.81991	0.82647
394	0.79128	0.80212	0.80718	0.80887	0.81434	0.82025	0.82680
395	0.79162	0.80249	0.80755	0.80924	0.81469	0.82059	0.82713
396	0.79204	0.80288	0.80792	0.80961	0.81504	0.82094	0.82746
397	0.79242	0.80325	0.80830	0.80997	0.81539	0.82128	0.82778
398	0.79282	0.80363	0.80867	0.81033	0.81574	0.82162	0.82811
399	0.79322	0.80402	0.80903	0.81070	0.81609	0.82195	0.82844
400	0.79358	0.80438	0.80939	0.81106	0.81643	0.82229	0.82876
401	0.79397	0.80474	0.80974	0.81141	0.81679	0.82263	0.82908
402	0.79437	0.80512	0.81011	0.81176	0.81713	0.82296	0.82939
403	0.79475	0.80549	0.81046	0.81213	0.81747	0.82329	0.82971
404	0.79512	0.80586	0.81082	0.81248	0.81782	0.82362	0.83003
405	0.79551	0.80623	0.81117	0.81283	0.81815	0.82395	0.83035
406	0.79590	0.80659	0.81153	0.81318	0.81850	0.82427	0.83066
407	0.79627	0.80695	0.81188	0.81353	0.81884	0.82460	0.83097
408	0.79668	0.80731	0.81224	0.81387	0.81917	0.82492	0.83129
409	0.79705	0.80767	0.81258	0.81422	0.81951	0.82525	0.83160
410	0.79742	0.80802	0.81293	0.81456	0.81984	0.82557	0.83191
411	0.79780	0.80838	0.81328	0.81491	0.82017	0.82589	0.83222

N	0.1%	0.5%	1%	1.25%	2.5%	5%	10%
412	0.79812	0.80874	0.81362	0.81524	0.82050	0.82621	0.83253
413	0.79852	0.80908	0.81396	0.81558	0.82083	0.82652	0.83283
414	0.79886	0.80944	0.81430	0.81592	0.82116	0.82684	0.83314
415	0.79923	0.80979	0.81464	0.81626	0.82149	0.82716	0.83344
416	0.79962	0.81014	0.81497	0.81659	0.82181	0.82747	0.83374
417	0.80002	0.81049	0.81532	0.81692	0.82214	0.82778	0.83404
418	0.80038	0.81083	0.81566	0.81725	0.82246	0.82810	0.83434
419	0.80072	0.81117	0.81599	0.81759	0.82278	0.82841	0.83464
420	0.80109	0.81149	0.81633	0.81792	0.82310	0.82871	0.83494
421	0.80147	0.81184	0.81666	0.81824	0.82342	0.82902	0.83524
422	0.80185	0.81219	0.81699	0.81857	0.82373	0.82933	0.83553
423	0.80220	0.81252	0.81732	0.81890	0.82405	0.82963	0.83582
424	0.80254	0.81286	0.81764	0.81922	0.82436	0.82994	0.83611
425	0.80291	0.81320	0.81797	0.81955	0.82468	0.83024	0.83640
426	0.80329	0.81353	0.81829	0.81988	0.82500	0.83055	0.83670
427	0.80363	0.81386	0.81862	0.82020	0.82531	0.83085	0.83699
428	0.80400	0.81419	0.81894	0.82052	0.82562	0.83115	0.83727
429	0.80437	0.81452	0.81926	0.82084	0.82592	0.83144	0.83756
430	0.80470	0.81486	0.81959	0.82116	0.82623	0.83174	0.83784
431	0.80507	0.81518	0.81991	0.82147	0.82654	0.83204	0.83813
432	0.80541	0.81552	0.82022	0.82178	0.82684	0.83233	0.83841
433	0.80575	0.81584	0.82054	0.82208	0.82715	0.83263	0.83869
434	0.80609	0.81617	0.82085	0.82241	0.82745	0.83292	0.83897
435	0.80640	0.81649	0.82117	0.82272	0.82776	0.83321	0.83925
436	0.80676	0.81681	0.82149	0.82303	0.82806	0.83350	0.83953
437	0.80710	0.81714	0.82180	0.82334	0.82836	0.83378	0.83981
438	0.80744	0.81745	0.82212	0.82364	0.82866	0.83407	0.84009
439	0.80780	0.81777	0.82242	0.82395	0.82896	0.83436	0.84036
440	0.80814	0.81808	0.82273	0.82426	0.82925	0.83465	0.84064
441	0.80848	0.81840	0.82303	0.82456	0.82955	0.83493	0.84091
442	0.80880	0.81871	0.82333	0.82486	0.82984	0.83522	0.84118
443	0.80916	0.81901	0.82364	0.82516	0.83013	0.83550	0.84145
444	0.80946	0.81932	0.82395	0.82546	0.83043	0.83578	0.84172
445	0.80979	0.81963	0.82425	0.82576	0.83072	0.83606	0.84199
446	0.81015	0.81995	0.82455	0.82606	0.83101	0.83634	0.84226
447	0.81046	0.82026	0.82485	0.82636	0.83130	0.83662	0.84253
448	0.81080	0.82057	0.82516	0.82666	0.83158	0.83690	0.84279
449	0.81115	0.82088	0.82545	0.82696	0.83187	0.83718	0.84306
450	0.81150	0.82118	0.82575	0.82725	0.83216	0.83745	0.84332
451	0.81180	0.82148	0.82605	0.82754	0.83244	0.83773	0.84358
452	0.81212	0.82179	0.82634	0.82783	0.83272	0.83800	0.84385
453	0.81242	0.82209	0.82662	0.82812	0.83300	0.83827	0.84411
454	0.81273	0.82240	0.82691	0.82841	0.83328	0.83854	0.84437
455	0.81304	0.82270	0.82720	0.82870	0.83356	0.83881	0.84463
456	0.81337	0.82300	0.82749	0.82898	0.83385	0.83909	0.84489
457	0.81369	0.82329	0.82778	0.82927	0.83413	0.83935	0.84515
458	0.81399	0.82359	0.82807	0.82955	0.83441	0.83961	0.84540
459	0.81431	0.82389	0.82836	0.82984	0.83468	0.83988	0.84566
460	0.81462	0.82418	0.82865	0.83013	0.83496	0.84014	0.84591
461	0.81493	0.82448	0.82894	0.83041	0.83523	0.84041	0.84616
462	0.81524	0.82477	0.82922	0.83069	0.83550	0.84067	0.84642
463	0.81556	0.82506	0.82951	0.83098	0.83577	0.84093	0.84667
464	0.81585	0.82535	0.82979	0.83125	0.83604	0.84119	0.84692
465	0.81618	0.82562	0.83007	0.83153	0.83632	0.84146	0.84718
466	0.81646	0.82592	0.83035	0.83181	0.83658	0.84172	0.84743
467	0.81675	0.82622	0.83062	0.83209	0.83685	0.84198	0.84767
468	0.81706	0.82651	0.83090	0.83236	0.83711	0.84223	0.84792
469	0.81738	0.82678	0.83117	0.83264	0.83738	0.84249	0.84816
470	0.81769	0.82706	0.83145	0.83291	0.83764	0.84274	0.84841
471	0.81800	0.82734	0.83173	0.83318	0.83791	0.84300	0.84866
472	0.81829	0.82761	0.83200	0.83345	0.83817	0.84326	0.84890
473	0.81859	0.82790	0.83227	0.83373	0.83844	0.84351	0.84914
474	0.81889	0.82819	0.83255	0.83400	0.83870	0.84376	0.84938
475	0.81918	0.82848	0.83282	0.83426	0.83896	0.84401	0.84962
476	0.81947	0.82876	0.83309	0.83453	0.83921	0.84426	0.84987
477	0.81977	0.82903	0.83336	0.83479	0.83948	0.84451	0.85011
478	0.82005	0.82929	0.83363	0.83506	0.83973	0.84476	0.85035
479	0.82034	0.82958	0.83390	0.83533	0.83999	0.84501	0.85058
480	0.82065	0.82985	0.83417	0.83560	0.84024	0.84526	0.85082
481	0.82092	0.83013	0.83443	0.83585	0.84050	0.84551	0.85106
482	0.82123	0.83040	0.83469	0.83612	0.84076	0.84575	0.85129
483	0.82150	0.83067	0.83496	0.83638	0.84101	0.84600	0.85153
484	0.82181	0.83094	0.83521	0.83664	0.84125	0.84624	0.85176
485	0.82210	0.83122	0.83547	0.83690	0.84151	0.84648	0.85200

N	0.1%	0.5%	1%	1.25%	2.5%	5%	10%
486	0.82237	0.83148	0.83574	0.83715	0.84176	0.84673	0.85223
487	0.82265	0.83174	0.83600	0.83741	0.84201	0.84697	0.85246
488	0.82295	0.83201	0.83626	0.83767	0.84226	0.84721	0.85269
489	0.82324	0.83227	0.83652	0.83792	0.84250	0.84744	0.85292
490	0.82350	0.83254	0.83678	0.83818	0.84275	0.84768	0.85315
491	0.82377	0.83280	0.83704	0.83843	0.84299	0.84792	0.85338
492	0.82404	0.83305	0.83729	0.83868	0.84324	0.84816	0.85360
493	0.82432	0.83332	0.83755	0.83893	0.84348	0.84840	0.85383
494	0.82460	0.83359	0.83780	0.83919	0.84373	0.84863	0.85406
495	0.82487	0.83384	0.83804	0.83943	0.84397	0.84887	0.85428
496	0.82516	0.83410	0.83829	0.83969	0.84421	0.84910	0.85451
497	0.82544	0.83437	0.83854	0.83993	0.84445	0.84934	0.85473
498	0.82571	0.83462	0.83879	0.84017	0.84469	0.84957	0.85495
499	0.82599	0.83487	0.83904	0.84043	0.84493	0.84980	0.85518
500	0.82627	0.83513	0.83929	0.84067	0.84517	0.85003	0.85540
501	0.82653	0.83539	0.83954	0.84092	0.84541	0.85026	0.85562
502	0.82679	0.83563	0.83979	0.84116	0.84565	0.85049	0.85584
503	0.82705	0.83590	0.84003	0.84141	0.84589	0.85072	0.85606
504	0.82734	0.83615	0.84028	0.84165	0.84613	0.85094	0.85628
505	0.82759	0.83640	0.84053	0.84189	0.84636	0.85117	0.85649
506	0.82786	0.83665	0.84077	0.84213	0.84659	0.85140	0.85671
507	0.82813	0.83690	0.84102	0.84237	0.84683	0.85162	0.85693
508	0.82841	0.83715	0.84126	0.84262	0.84706	0.85184	0.85714
509	0.82867	0.83739	0.84150	0.84286	0.84729	0.85207	0.85736
510	0.82890	0.83765	0.84174	0.84309	0.84753	0.85229	0.85757
511	0.82917	0.83789	0.84198	0.84333	0.84775	0.85252	0.85778
512	0.82943	0.83814	0.84222	0.84357	0.84798	0.85274	0.85800
513	0.82967	0.83839	0.84246	0.84381	0.84820	0.85296	0.85821
514	0.82993	0.83864	0.84270	0.84405	0.84843	0.85317	0.85842
515	0.83018	0.83888	0.84293	0.84429	0.84866	0.85339	0.85863
516	0.83044	0.83911	0.84317	0.84452	0.84888	0.85361	0.85884
517	0.83070	0.83935	0.84341	0.84475	0.84911	0.85383	0.85905
518	0.83095	0.83959	0.84365	0.84498	0.84933	0.85405	0.85926
519	0.83122	0.83983	0.84388	0.84521	0.84956	0.85426	0.85947
520	0.83147	0.84006	0.84410	0.84544	0.84978	0.85448	0.85967
521	0.83173	0.84030	0.84434	0.84567	0.85000	0.85469	0.85988
522	0.83200	0.84055	0.84457	0.84590	0.85022	0.85491	0.86009
523	0.83225	0.84079	0.84480	0.84613	0.85044	0.85512	0.86029
524	0.83248	0.84104	0.84503	0.84636	0.85067	0.85534	0.86050
525	0.83273	0.84127	0.84525	0.84658	0.85088	0.85555	0.86070
526	0.83296	0.84150	0.84549	0.84680	0.85111	0.85576	0.86090
527	0.83320	0.84173	0.84571	0.84703	0.85133	0.85597	0.86111
528	0.83347	0.84196	0.84594	0.84725	0.85154	0.85618	0.86131
529	0.83371	0.84219	0.84617	0.84748	0.85176	0.85639	0.86151
530	0.83395	0.84242	0.84639	0.84770	0.85198	0.85660	0.86171
531	0.83420	0.84266	0.84662	0.84792	0.85219	0.85681	0.86191
532	0.83446	0.84289	0.84685	0.84815	0.85241	0.85701	0.86211
533	0.83472	0.84312	0.84707	0.84837	0.85262	0.85722	0.86231
534	0.83496	0.84335	0.84730	0.84859	0.85284	0.85743	0.86250
535	0.83520	0.84359	0.84752	0.84881	0.85305	0.85763	0.86270
536	0.83543	0.84381	0.84774	0.84903	0.85326	0.85784	0.86290
537	0.83566	0.84404	0.84795	0.84925	0.85347	0.85804	0.86309
538	0.83591	0.84427	0.84818	0.84947	0.85368	0.85825	0.86329
539	0.83614	0.84449	0.84840	0.84968	0.85390	0.85845	0.86348
540	0.83639	0.84471	0.84861	0.84990	0.85411	0.85865	0.86368
541	0.83663	0.84494	0.84883	0.85012	0.85432	0.85885	0.86387
542	0.83685	0.84515	0.84904	0.85033	0.85452	0.85905	0.86407
543	0.83708	0.84537	0.84926	0.85054	0.85473	0.85926	0.86426
544	0.83733	0.84560	0.84947	0.85075	0.85494	0.85946	0.86445
545	0.83756	0.84582	0.84968	0.85097	0.85515	0.85966	0.86464
546	0.83777	0.84604	0.84989	0.85118	0.85535	0.85986	0.86483
547	0.83800	0.84626	0.85010	0.85139	0.85556	0.86005	0.86502
548	0.83823	0.84647	0.85031	0.85160	0.85576	0.86025	0.86521
549	0.83846	0.84669	0.85053	0.85181	0.85597	0.86045	0.86540
550	0.83869	0.84691	0.85074	0.85202	0.85617	0.86065	0.86559
551	0.83893	0.84713	0.85095	0.85223	0.85637	0.86084	0.86578
552	0.83915	0.84735	0.85116	0.85243	0.85658	0.86103	0.86597
553	0.83939	0.84757	0.85137	0.85265	0.85678	0.86123	0.86615
554	0.83961	0.84779	0.85158	0.85285	0.85698	0.86142	0.86634
555	0.83984	0.84800	0.85179	0.85306	0.85718	0.86161	0.86653
556	0.84007	0.84821	0.85200	0.85327	0.85737	0.86181	0.86671
557	0.84032	0.84843	0.85220	0.85347	0.85757	0.86200	0.86689
558	0.84055	0.84864	0.85241	0.85368	0.85777	0.86219	0.86708
559	0.84079	0.84885	0.85261	0.85388	0.85796	0.86238	0.86726

N	0.1%	0.5%	1%	1.25%	2.5%	5%	10%
560	0.84100	0.84907	0.85282	0.85409	0.85816	0.86258	0.86745
561	0.84121	0.84927	0.85303	0.85429	0.85836	0.86276	0.86763
562	0.84142	0.84948	0.85323	0.85449	0.85855	0.86295	0.86781
563	0.84164	0.84969	0.85343	0.85469	0.85875	0.86314	0.86799
564	0.84186	0.84991	0.85364	0.85490	0.85894	0.86333	0.86817
565	0.84208	0.85012	0.85385	0.85510	0.85914	0.86352	0.86835
566	0.84231	0.85032	0.85405	0.85530	0.85933	0.86371	0.86853
567	0.84253	0.85053	0.85425	0.85550	0.85952	0.86389	0.86871
568	0.84276	0.85074	0.85445	0.85569	0.85971	0.86407	0.86889
569	0.84297	0.85094	0.85465	0.85589	0.85991	0.86426	0.86907
570	0.84318	0.85114	0.85485	0.85609	0.86010	0.86444	0.86924
571	0.84340	0.85134	0.85505	0.85628	0.86029	0.86463	0.86942
572	0.84362	0.85155	0.85524	0.85648	0.86048	0.86481	0.86960
573	0.84382	0.85176	0.85544	0.85667	0.86067	0.86499	0.86977
574	0.84404	0.85195	0.85563	0.85686	0.86086	0.86518	0.86994
575	0.84424	0.85215	0.85583	0.85706	0.86105	0.86536	0.87012
576	0.84446	0.85235	0.85602	0.85725	0.86123	0.86554	0.87029
577	0.84468	0.85255	0.85622	0.85745	0.86142	0.86572	0.87046
578	0.84488	0.85276	0.85641	0.85764	0.86161	0.86590	0.87064
579	0.84508	0.85295	0.85661	0.85783	0.86179	0.86608	0.87081
580	0.84530	0.85314	0.85680	0.85802	0.86198	0.86626	0.87098
581	0.84551	0.85334	0.85699	0.85821	0.86216	0.86643	0.87115
582	0.84573	0.85354	0.85718	0.85840	0.86235	0.86661	0.87132
583	0.84593	0.85374	0.85737	0.85859	0.86253	0.86679	0.87149
584	0.84613	0.85393	0.85757	0.85878	0.86272	0.86697	0.87166
585	0.84635	0.85411	0.85776	0.85897	0.86291	0.86715	0.87183
586	0.84654	0.85432	0.85795	0.85915	0.86309	0.86732	0.87200
587	0.84675	0.85450	0.85814	0.85935	0.86327	0.86749	0.87217
588	0.84694	0.85471	0.85833	0.85953	0.86345	0.86767	0.87234
589	0.84715	0.85490	0.85851	0.85972	0.86363	0.86784	0.87251
590	0.84734	0.85510	0.85870	0.85991	0.86381	0.86802	0.87267
591	0.84755	0.85528	0.85889	0.86010	0.86399	0.86819	0.87284
592	0.84775	0.85548	0.85908	0.86028	0.86417	0.86837	0.87300
593	0.84796	0.85567	0.85926	0.86046	0.86435	0.86854	0.87317
594	0.84817	0.85586	0.85945	0.86066	0.86452	0.86871	0.87334
595	0.84835	0.85605	0.85964	0.86084	0.86470	0.86888	0.87350
596	0.84856	0.85625	0.85982	0.86103	0.86487	0.86905	0.87367
597	0.84875	0.85644	0.86001	0.86121	0.86505	0.86922	0.87383
598	0.84895	0.85662	0.86019	0.86139	0.86523	0.86939	0.87399
599	0.84916	0.85681	0.86037	0.86157	0.86541	0.86956	0.87416
600	0.84933	0.85700	0.86055	0.86175	0.86558	0.86973	0.87432
601	0.84951	0.85718	0.86073	0.86193	0.86575	0.86989	0.87448
602	0.84972	0.85736	0.86092	0.86211	0.86593	0.87006	0.87464
603	0.84992	0.85754	0.86110	0.86229	0.86610	0.87023	0.87480
604	0.85012	0.85772	0.86128	0.86246	0.86627	0.87040	0.87496
605	0.85030	0.85791	0.86146	0.86264	0.86645	0.87057	0.87512
606	0.85049	0.85810	0.86164	0.86281	0.86662	0.87073	0.87528
607	0.85072	0.85827	0.86182	0.86299	0.86679	0.87090	0.87544
608	0.85090	0.85846	0.86199	0.86317	0.86696	0.87107	0.87560
609	0.85110	0.85864	0.86217	0.86334	0.86713	0.87123	0.87576
610	0.85128	0.85883	0.86235	0.86352	0.86730	0.87139	0.87592
611	0.85148	0.85901	0.86252	0.86369	0.86747	0.87156	0.87607
612	0.85169	0.85919	0.86271	0.86387	0.86764	0.87172	0.87623
613	0.85186	0.85937	0.86288	0.86405	0.86781	0.87189	0.87639
614	0.85207	0.85955	0.86304	0.86422	0.86798	0.87205	0.87654
615	0.85225	0.85973	0.86322	0.86439	0.86815	0.87221	0.87670
616	0.85243	0.85991	0.86339	0.86456	0.86832	0.87237	0.87685
617	0.85262	0.86009	0.86357	0.86473	0.86848	0.87253	0.87701
618	0.85281	0.86028	0.86375	0.86490	0.86865	0.87269	0.87716
619	0.85301	0.86044	0.86391	0.86508	0.86882	0.87285	0.87732
620	0.85318	0.86063	0.86409	0.86525	0.86899	0.87302	0.87747
621	0.85337	0.86081	0.86427	0.86542	0.86915	0.87318	0.87763
622	0.85358	0.86098	0.86443	0.86559	0.86932	0.87333	0.87778
623	0.85377	0.86116	0.86461	0.86576	0.86948	0.87349	0.87793
624	0.85396	0.86133	0.86478	0.86593	0.86964	0.87365	0.87808
625	0.85414	0.86150	0.86495	0.86610	0.86980	0.87380	0.87823
626	0.85433	0.86167	0.86511	0.86626	0.86997	0.87396	0.87839
627	0.85450	0.86184	0.86529	0.86643	0.87013	0.87412	0.87854
628	0.85469	0.86201	0.86545	0.86660	0.87029	0.87428	0.87869
629	0.85489	0.86219	0.86562	0.86677	0.87045	0.87443	0.87883
630	0.85508	0.86236	0.86579	0.86693	0.87061	0.87459	0.87899
631	0.85525	0.86253	0.86595	0.86709	0.87077	0.87475	0.87913
632	0.85545	0.86270	0.86612	0.86726	0.87093	0.87490	0.87928
633	0.85560	0.86287	0.86628	0.86742	0.87109	0.87506	0.87943

N	0.1%	0.5%	1%	1.25%	2.5%	5%	10%
634	0.85580	0.86304	0.86645	0.86758	0.87125	0.87521	0.87958
635	0.85598	0.86322	0.86661	0.86775	0.87141	0.87536	0.87973
636	0.85615	0.86338	0.86678	0.86791	0.87156	0.87552	0.87988
637	0.85634	0.86356	0.86694	0.86807	0.87172	0.87567	0.88002
638	0.85652	0.86373	0.86711	0.86824	0.87188	0.87582	0.88017
639	0.85668	0.86390	0.86728	0.86840	0.87204	0.87597	0.88032
640	0.85686	0.86407	0.86744	0.86855	0.87219	0.87612	0.88046
641	0.85704	0.86424	0.86760	0.86871	0.87235	0.87628	0.88060
642	0.85720	0.86441	0.86776	0.86888	0.87251	0.87643	0.88075
643	0.85736	0.86457	0.86792	0.86904	0.87267	0.87657	0.88089
644	0.85754	0.86474	0.86808	0.86920	0.87282	0.87672	0.88104
645	0.85773	0.86489	0.86824	0.86936	0.87297	0.87687	0.88118
646	0.85790	0.86506	0.86840	0.86951	0.87313	0.87702	0.88132
647	0.85808	0.86522	0.86855	0.86967	0.87328	0.87717	0.88146
648	0.85823	0.86539	0.86871	0.86983	0.87343	0.87732	0.88161
649	0.85840	0.86555	0.86888	0.86999	0.87359	0.87746	0.88175
650	0.85859	0.86571	0.86903	0.87014	0.87374	0.87761	0.88189
651	0.85877	0.86588	0.86920	0.87030	0.87389	0.87776	0.88203
652	0.85893	0.86605	0.86936	0.87046	0.87405	0.87790	0.88217
653	0.85910	0.86620	0.86951	0.87062	0.87419	0.87805	0.88231
654	0.85928	0.86637	0.86967	0.87077	0.87435	0.87819	0.88246
655	0.85945	0.86653	0.86983	0.87093	0.87450	0.87834	0.88260
656	0.85960	0.86669	0.86998	0.87108	0.87465	0.87848	0.88273
657	0.85978	0.86685	0.87014	0.87124	0.87480	0.87863	0.88287
658	0.85993	0.86700	0.87029	0.87139	0.87495	0.87877	0.88301
659	0.86009	0.86716	0.87045	0.87155	0.87509	0.87892	0.88315
660	0.86026	0.86733	0.87060	0.87169	0.87524	0.87906	0.88329
661	0.86041	0.86749	0.87076	0.87184	0.87539	0.87921	0.88343
662	0.86058	0.86764	0.87091	0.87200	0.87554	0.87935	0.88356
663	0.86075	0.86780	0.87106	0.87215	0.87568	0.87949	0.88370
664	0.86091	0.86795	0.87122	0.87231	0.87583	0.87963	0.88384
665	0.86107	0.86811	0.87137	0.87246	0.87598	0.87977	0.88397
666	0.86124	0.86827	0.87152	0.87261	0.87612	0.87991	0.88411
667	0.86141	0.86842	0.87167	0.87276	0.87627	0.88006	0.88424
668	0.86158	0.86858	0.87183	0.87291	0.87642	0.88020	0.88438
669	0.86176	0.86873	0.87197	0.87306	0.87656	0.88034	0.88451
670	0.86192	0.86889	0.87212	0.87321	0.87671	0.88048	0.88465
671	0.86209	0.86904	0.87228	0.87336	0.87685	0.88061	0.88478
672	0.86224	0.86920	0.87242	0.87351	0.87699	0.88075	0.88491
673	0.86241	0.86936	0.87257	0.87366	0.87714	0.88089	0.88505
674	0.86257	0.86950	0.87272	0.87380	0.87728	0.88103	0.88518
675	0.86276	0.86966	0.87287	0.87395	0.87742	0.88117	0.88531
676	0.86291	0.86982	0.87302	0.87410	0.87757	0.88130	0.88545
677	0.86307	0.86997	0.87318	0.87425	0.87771	0.88144	0.88558
678	0.86323	0.87013	0.87333	0.87439	0.87785	0.88158	0.88571
679	0.86339	0.87028	0.87347	0.87454	0.87799	0.88172	0.88584
680	0.86355	0.87043	0.87362	0.87468	0.87814	0.88185	0.88597
681	0.86370	0.87057	0.87376	0.87483	0.87827	0.88199	0.88610
682	0.86388	0.87072	0.87391	0.87497	0.87841	0.88213	0.88623
683	0.86402	0.87087	0.87406	0.87512	0.87855	0.88226	0.88636
684	0.86417	0.87102	0.87420	0.87526	0.87870	0.88240	0.88649
685	0.86434	0.87118	0.87435	0.87541	0.87883	0.88253	0.88662
686	0.86449	0.87133	0.87449	0.87554	0.87897	0.88266	0.88675
687	0.86465	0.87147	0.87463	0.87569	0.87911	0.88280	0.88688
688	0.86481	0.87162	0.87478	0.87583	0.87925	0.88293	0.88701
689	0.86496	0.87177	0.87492	0.87598	0.87938	0.88306	0.88713
690	0.86512	0.87191	0.87506	0.87612	0.87952	0.88320	0.88726
691	0.86527	0.87206	0.87521	0.87626	0.87966	0.88333	0.88739
692	0.86543	0.87221	0.87535	0.87639	0.87980	0.88346	0.88751
693	0.86559	0.87235	0.87550	0.87654	0.87994	0.88359	0.88764
694	0.86574	0.87250	0.87564	0.87668	0.88007	0.88373	0.88777
695	0.86590	0.87264	0.87578	0.87682	0.88021	0.88386	0.88789
696	0.86605	0.87279	0.87592	0.87696	0.88034	0.88399	0.88802
697	0.86621	0.87294	0.87606	0.87710	0.88047	0.88412	0.88815
698	0.86637	0.87309	0.87619	0.87724	0.88061	0.88425	0.88827
699	0.86652	0.87323	0.87633	0.87738	0.88074	0.88438	0.88839
700	0.86665	0.87337	0.87648	0.87752	0.88088	0.88451	0.88852
701	0.86680	0.87351	0.87662	0.87766	0.88101	0.88464	0.88864
702	0.86697	0.87365	0.87675	0.87779	0.88114	0.88477	0.88877
703	0.86712	0.87380	0.87689	0.87793	0.88127	0.88490	0.88889
704	0.86727	0.87394	0.87703	0.87806	0.88141	0.88503	0.88901
705	0.86741	0.87409	0.87716	0.87820	0.88154	0.88516	0.88914
706	0.86756	0.87423	0.87731	0.87833	0.88167	0.88528	0.88926
707	0.86770	0.87437	0.87745	0.87847	0.88180	0.88541	0.88938

N	0.1%	0.5%	1%	1.25%	2.5%	5%	10%
708	0.86785	0.87451	0.87758	0.87861	0.88194	0.88554	0.88950
709	0.86801	0.87464	0.87772	0.87874	0.88207	0.88566	0.88963
710	0.86816	0.87479	0.87786	0.87888	0.88220	0.88579	0.88975
711	0.86830	0.87493	0.87799	0.87901	0.88233	0.88592	0.88987
712	0.86846	0.87507	0.87812	0.87914	0.88246	0.88604	0.88999
713	0.86860	0.87521	0.87826	0.87928	0.88259	0.88617	0.89011
714	0.86875	0.87535	0.87839	0.87941	0.88272	0.88629	0.89023
715	0.86891	0.87548	0.87852	0.87955	0.88285	0.88642	0.89035
716	0.86904	0.87562	0.87866	0.87968	0.88298	0.88654	0.89047
717	0.86919	0.87576	0.87880	0.87981	0.88311	0.88667	0.89059
718	0.86935	0.87589	0.87893	0.87995	0.88324	0.88679	0.89071
719	0.86949	0.87604	0.87906	0.88008	0.88337	0.88692	0.89083
720	0.86962	0.87618	0.87920	0.88021	0.88350	0.88704	0.89095
721	0.86977	0.87632	0.87933	0.88035	0.88363	0.88717	0.89107
722	0.86991	0.87645	0.87946	0.88048	0.88375	0.88729	0.89118
723	0.87007	0.87659	0.87959	0.88061	0.88387	0.88741	0.89130
724	0.87020	0.87673	0.87973	0.88073	0.88400	0.88753	0.89142
725	0.87034	0.87687	0.87986	0.88086	0.88413	0.88765	0.89153
726	0.87048	0.87701	0.87999	0.88099	0.88426	0.88777	0.89165
727	0.87063	0.87714	0.88012	0.88113	0.88438	0.88789	0.89177
728	0.87077	0.87728	0.88026	0.88126	0.88451	0.88802	0.89188
729	0.87091	0.87740	0.88038	0.88139	0.88463	0.88814	0.89200
730	0.87105	0.87754	0.88052	0.88152	0.88476	0.88826	0.89212
731	0.87121	0.87768	0.88065	0.88165	0.88489	0.88838	0.89223
732	0.87136	0.87781	0.88078	0.88177	0.88501	0.88850	0.89235
733	0.87150	0.87794	0.88090	0.88190	0.88514	0.88862	0.89246
734	0.87165	0.87808	0.88103	0.88203	0.88526	0.88873	0.89258
735	0.87178	0.87820	0.88116	0.88215	0.88538	0.88885	0.89269
736	0.87192	0.87834	0.88129	0.88228	0.88550	0.88897	0.89281
737	0.87206	0.87847	0.88142	0.88241	0.88563	0.88909	0.89292
738	0.87220	0.87860	0.88155	0.88253	0.88575	0.88921	0.89304
739	0.87234	0.87872	0.88168	0.88266	0.88587	0.88933	0.89315
740	0.87248	0.87885	0.88180	0.88278	0.88600	0.88945	0.89326
741	0.87262	0.87899	0.88193	0.88291	0.88612	0.88956	0.89337
742	0.87275	0.87911	0.88206	0.88304	0.88624	0.88968	0.89349
743	0.87288	0.87924	0.88218	0.88316	0.88636	0.88980	0.89360
744	0.87301	0.87937	0.88230	0.88328	0.88648	0.88991	0.89371
745	0.87315	0.87950	0.88243	0.88341	0.88660	0.89003	0.89382
746	0.87329	0.87963	0.88256	0.88353	0.88672	0.89014	0.89393
747	0.87342	0.87976	0.88269	0.88366	0.88684	0.89026	0.89405
748	0.87356	0.87989	0.88281	0.88378	0.88696	0.89038	0.89416
749	0.87368	0.88002	0.88293	0.88390	0.88708	0.89049	0.89427
750	0.87380	0.88014	0.88306	0.88403	0.88720	0.89061	0.89438
751	0.87395	0.88027	0.88319	0.88415	0.88732	0.89072	0.89449
752	0.87408	0.88040	0.88331	0.88427	0.88744	0.89083	0.89460
753	0.87422	0.88052	0.88343	0.88440	0.88755	0.89095	0.89471
754	0.87435	0.88064	0.88355	0.88452	0.88767	0.89106	0.89482
755	0.87448	0.88076	0.88367	0.88464	0.88779	0.89118	0.89493
756	0.87461	0.88089	0.88379	0.88477	0.88791	0.89129	0.89504
757	0.87475	0.88102	0.88392	0.88488	0.88802	0.89141	0.89515
758	0.87488	0.88115	0.88404	0.88500	0.88814	0.89152	0.89526
759	0.87501	0.88127	0.88417	0.88512	0.88826	0.89163	0.89537
760	0.87515	0.88139	0.88429	0.88525	0.88838	0.89174	0.89547
761	0.87528	0.88152	0.88441	0.88537	0.88849	0.89186	0.89558
762	0.87541	0.88164	0.88453	0.88549	0.88861	0.89197	0.89569
763	0.87553	0.88177	0.88465	0.88561	0.88873	0.89208	0.89580
764	0.87566	0.88189	0.88477	0.88572	0.88884	0.89219	0.89590
765	0.87579	0.88201	0.88489	0.88584	0.88896	0.89230	0.89601
766	0.87591	0.88214	0.88501	0.88596	0.88907	0.89241	0.89612
767	0.87605	0.88226	0.88513	0.88607	0.88918	0.89252	0.89622
768	0.87619	0.88239	0.88525	0.88620	0.88930	0.89263	0.89633
769	0.87632	0.88251	0.88537	0.88632	0.88941	0.89274	0.89643
770	0.87643	0.88263	0.88549	0.88643	0.88953	0.89285	0.89654
771	0.87656	0.88275	0.88561	0.88655	0.88964	0.89296	0.89665
772	0.87670	0.88287	0.88572	0.88667	0.88975	0.89307	0.89675
773	0.87681	0.88299	0.88584	0.88679	0.88986	0.89318	0.89686
774	0.87695	0.88311	0.88596	0.88690	0.88998	0.89329	0.89696
775	0.87710	0.88323	0.88608	0.88702	0.89009	0.89340	0.89707
776	0.87721	0.88335	0.88619	0.88714	0.89020	0.89350	0.89717
777	0.87733	0.88348	0.88631	0.88725	0.89032	0.89361	0.89728
778	0.87745	0.88360	0.88643	0.88736	0.89043	0.89372	0.89738
779	0.87758	0.88371	0.88654	0.88748	0.89054	0.89383	0.89748
780	0.87771	0.88383	0.88666	0.88759	0.89065	0.89394	0.89759
781	0.87783	0.88396	0.88677	0.88771	0.89076	0.89405	0.89769

N	0.1%	0.5%	1%	1.25%	2.5%	5%	10%
782	0.87796	0.88407	0.88689	0.88782	0.89088	0.89415	0.89779
783	0.87808	0.88419	0.88700	0.88794	0.89098	0.89426	0.89790
784	0.87821	0.88431	0.88711	0.88805	0.89109	0.89436	0.89800
785	0.87834	0.88443	0.88723	0.88816	0.89121	0.89447	0.89810
786	0.87845	0.88455	0.88734	0.88828	0.89132	0.89458	0.89820
787	0.87858	0.88467	0.88746	0.88840	0.89142	0.89468	0.89830
788	0.87870	0.88478	0.88757	0.88851	0.89153	0.89479	0.89841
789	0.87882	0.88490	0.88769	0.88862	0.89164	0.89489	0.89851
790	0.87895	0.88502	0.88780	0.88873	0.89175	0.89500	0.89861
791	0.87907	0.88513	0.88791	0.88884	0.89186	0.89511	0.89871
792	0.87920	0.88525	0.88802	0.88895	0.89197	0.89521	0.89881
793	0.87933	0.88537	0.88814	0.88907	0.89207	0.89531	0.89891
794	0.87945	0.88548	0.88825	0.88917	0.89218	0.89542	0.89901
795	0.87957	0.88560	0.88836	0.88929	0.89229	0.89552	0.89911
796	0.87969	0.88571	0.88847	0.88940	0.89240	0.89563	0.89921
797	0.87981	0.88582	0.88859	0.88951	0.89251	0.89573	0.89931
798	0.87993	0.88594	0.88870	0.88962	0.89261	0.89584	0.89941
799	0.88005	0.88605	0.88881	0.88973	0.89272	0.89594	0.89951
800	0.88016	0.88617	0.88892	0.88983	0.89283	0.89604	0.89961
801	0.88028	0.88628	0.88903	0.88994	0.89293	0.89614	0.89971
802	0.88040	0.88639	0.88914	0.89005	0.89304	0.89624	0.89980
803	0.88051	0.88650	0.88925	0.89016	0.89315	0.89635	0.89990
804	0.88063	0.88662	0.88935	0.89027	0.89325	0.89645	0.90000
805	0.88075	0.88673	0.88946	0.89038	0.89336	0.89655	0.90010
806	0.88087	0.88684	0.88958	0.89049	0.89346	0.89665	0.90020
807	0.88099	0.88695	0.88968	0.89060	0.89357	0.89675	0.90030
808	0.88112	0.88706	0.88979	0.89070	0.89367	0.89685	0.90039
809	0.88124	0.88718	0.88990	0.89081	0.89378	0.89696	0.90049
810	0.88136	0.88729	0.89001	0.89092	0.89388	0.89705	0.90059
811	0.88149	0.88739	0.89012	0.89103	0.89398	0.89716	0.90068
812	0.88161	0.88750	0.89023	0.89113	0.89409	0.89726	0.90078
813	0.88172	0.88761	0.89034	0.89124	0.89419	0.89736	0.90087
814	0.88184	0.88772	0.89044	0.89134	0.89429	0.89746	0.90097
815	0.88195	0.88784	0.89055	0.89145	0.89440	0.89756	0.90106
816	0.88206	0.88795	0.89066	0.89156	0.89450	0.89766	0.90116
817	0.88218	0.88806	0.89077	0.89166	0.89460	0.89776	0.90126
818	0.88228	0.88817	0.89087	0.89177	0.89471	0.89785	0.90135
819	0.88240	0.88827	0.89098	0.89188	0.89481	0.89795	0.90145
820	0.88252	0.88839	0.89108	0.89198	0.89491	0.89805	0.90154
821	0.88265	0.88850	0.89119	0.89209	0.89501	0.89815	0.90164
822	0.88276	0.88861	0.89130	0.89219	0.89511	0.89825	0.90173
823	0.88288	0.88872	0.89140	0.89230	0.89521	0.89834	0.90182
824	0.88299	0.88883	0.89150	0.89240	0.89531	0.89844	0.90192
825	0.88311	0.88894	0.89161	0.89250	0.89541	0.89854	0.90201
826	0.88322	0.88904	0.89171	0.89261	0.89551	0.89864	0.90211
827	0.88335	0.88914	0.89182	0.89271	0.89562	0.89874	0.90220
828	0.88346	0.88925	0.89192	0.89281	0.89572	0.89884	0.90229
829	0.88358	0.88936	0.89203	0.89292	0.89582	0.89893	0.90239
830	0.88369	0.88947	0.89213	0.89302	0.89592	0.89903	0.90248
831	0.88381	0.88957	0.89224	0.89312	0.89601	0.89912	0.90257
832	0.88391	0.88968	0.89234	0.89323	0.89611	0.89922	0.90266
833	0.88402	0.88979	0.89244	0.89333	0.89621	0.89931	0.90276
834	0.88414	0.88990	0.89254	0.89343	0.89631	0.89941	0.90285
835	0.88425	0.89000	0.89265	0.89354	0.89641	0.89951	0.90294
836	0.88436	0.89011	0.89275	0.89364	0.89651	0.89960	0.90303
837	0.88448	0.89021	0.89286	0.89374	0.89661	0.89970	0.90312
838	0.88458	0.89032	0.89296	0.89384	0.89671	0.89979	0.90321
839	0.88471	0.89043	0.89306	0.89394	0.89680	0.89989	0.90331
840	0.88481	0.89053	0.89316	0.89404	0.89690	0.89998	0.90340
841	0.88492	0.89064	0.89326	0.89415	0.89700	0.90008	0.90349
842	0.88504	0.89075	0.89336	0.89425	0.89710	0.90017	0.90358
843	0.88516	0.89085	0.89346	0.89434	0.89720	0.90026	0.90367
844	0.88526	0.89095	0.89356	0.89445	0.89729	0.90036	0.90376
845	0.88537	0.89105	0.89367	0.89455	0.89739	0.90045	0.90385
846	0.88548	0.89115	0.89377	0.89464	0.89748	0.90054	0.90394
847	0.88559	0.89125	0.89387	0.89475	0.89758	0.90064	0.90403
848	0.88570	0.89135	0.89397	0.89485	0.89768	0.90073	0.90412
849	0.88581	0.89146	0.89407	0.89494	0.89777	0.90082	0.90421
850	0.88592	0.89156	0.89417	0.89504	0.89787	0.90092	0.90430
851	0.88601	0.89165	0.89426	0.89514	0.89796	0.90101	0.90438
852	0.88613	0.89176	0.89436	0.89524	0.89806	0.90110	0.90447
853	0.88624	0.89186	0.89446	0.89533	0.89815	0.90119	0.90456
854	0.88634	0.89196	0.89456	0.89543	0.89825	0.90129	0.90465
855	0.88645	0.89207	0.89466	0.89553	0.89834	0.90138	0.90474

N	0.1%	0.5%	1%	1.25%	2.5%	5%	10%
856	0.88657	0.89217	0.89475	0.89563	0.89844	0.90147	0.90483
857	0.88667	0.89227	0.89485	0.89573	0.89853	0.90156	0.90492
858	0.88679	0.89237	0.89495	0.89582	0.89863	0.90165	0.90500
859	0.88689	0.89247	0.89505	0.89592	0.89872	0.90174	0.90509
860	0.88700	0.89257	0.89515	0.89602	0.89882	0.90183	0.90518
861	0.88710	0.89267	0.89525	0.89612	0.89891	0.90192	0.90526
862	0.88720	0.89277	0.89534	0.89621	0.89901	0.90201	0.90535
863	0.88731	0.89287	0.89544	0.89630	0.89910	0.90211	0.90544
864	0.88740	0.89297	0.89554	0.89640	0.89919	0.90220	0.90553
865	0.88751	0.89307	0.89564	0.89650	0.89929	0.90228	0.90561
866	0.88763	0.89317	0.89573	0.89659	0.89938	0.90237	0.90570
867	0.88772	0.89327	0.89583	0.89669	0.89947	0.90247	0.90578
868	0.88782	0.89337	0.89593	0.89678	0.89956	0.90255	0.90587
869	0.88792	0.89347	0.89602	0.89688	0.89966	0.90264	0.90596
870	0.88803	0.89357	0.89612	0.89697	0.89975	0.90273	0.90604
871	0.88814	0.89367	0.89621	0.89706	0.89984	0.90282	0.90613
872	0.88824	0.89377	0.89631	0.89715	0.89993	0.90291	0.90621
873	0.88835	0.89386	0.89640	0.89725	0.90003	0.90300	0.90630
874	0.88846	0.89396	0.89649	0.89734	0.90011	0.90309	0.90638
875	0.88856	0.89405	0.89659	0.89744	0.90021	0.90317	0.90647
876	0.88865	0.89415	0.89668	0.89754	0.90030	0.90326	0.90655
877	0.88875	0.89425	0.89678	0.89763	0.90039	0.90335	0.90664
878	0.88886	0.89434	0.89688	0.89772	0.90048	0.90344	0.90672
879	0.88896	0.89444	0.89697	0.89781	0.90057	0.90352	0.90681
880	0.88905	0.89453	0.89706	0.89790	0.90066	0.90361	0.90689
881	0.88915	0.89463	0.89715	0.89800	0.90075	0.90370	0.90697
882	0.88926	0.89472	0.89725	0.89809	0.90084	0.90379	0.90706
883	0.88936	0.89482	0.89734	0.89818	0.90093	0.90388	0.90714
884	0.88947	0.89491	0.89743	0.89827	0.90101	0.90396	0.90722
885	0.88957	0.89501	0.89752	0.89836	0.90110	0.90405	0.90731
886	0.88967	0.89510	0.89762	0.89846	0.90119	0.90413	0.90739
887	0.88977	0.89520	0.89771	0.89855	0.90128	0.90422	0.90747
888	0.88987	0.89529	0.89780	0.89864	0.90137	0.90431	0.90756
889	0.88996	0.89538	0.89789	0.89873	0.90146	0.90439	0.90764
890	0.89006	0.89549	0.89799	0.89882	0.90155	0.90447	0.90772
891	0.89016	0.89558	0.89808	0.89891	0.90164	0.90456	0.90780
892	0.89026	0.89568	0.89817	0.89901	0.90173	0.90464	0.90788
893	0.89035	0.89577	0.89826	0.89909	0.90181	0.90473	0.90797
894	0.89046	0.89586	0.89835	0.89918	0.90190	0.90482	0.90805
895	0.89055	0.89596	0.89844	0.89927	0.90199	0.90490	0.90813
896	0.89064	0.89606	0.89853	0.89937	0.90208	0.90499	0.90821
897	0.89073	0.89615	0.89863	0.89946	0.90216	0.90507	0.90829
898	0.89083	0.89624	0.89872	0.89955	0.90225	0.90515	0.90837
899	0.89092	0.89634	0.89880	0.89964	0.90234	0.90524	0.90845
900	0.89103	0.89643	0.89889	0.89973	0.90242	0.90532	0.90853
901	0.89114	0.89652	0.89899	0.89982	0.90251	0.90541	0.90861
902	0.89124	0.89661	0.89908	0.89991	0.90260	0.90549	0.90870
903	0.89133	0.89670	0.89917	0.89999	0.90268	0.90557	0.90878
904	0.89143	0.89679	0.89926	0.90008	0.90277	0.90566	0.90886
905	0.89153	0.89688	0.89935	0.90017	0.90286	0.90574	0.90893
906	0.89162	0.89697	0.89944	0.90026	0.90294	0.90582	0.90901
907	0.89171	0.89706	0.89953	0.90035	0.90302	0.90591	0.90910
908	0.89180	0.89715	0.89962	0.90043	0.90311	0.90599	0.90917
909	0.89191	0.89724	0.89971	0.90053	0.90319	0.90607	0.90925
910	0.89200	0.89734	0.89980	0.90061	0.90328	0.90615	0.90933
911	0.89210	0.89743	0.89989	0.90070	0.90337	0.90624	0.90941
912	0.89220	0.89752	0.89998	0.90079	0.90345	0.90632	0.90949
913	0.89229	0.89761	0.90007	0.90087	0.90354	0.90640	0.90957
914	0.89238	0.89770	0.90015	0.90096	0.90362	0.90648	0.90964
915	0.89247	0.89780	0.90024	0.90105	0.90371	0.90656	0.90972
916	0.89257	0.89789	0.90033	0.90114	0.90379	0.90664	0.90980
917	0.89267	0.89797	0.90042	0.90122	0.90387	0.90673	0.90988
918	0.89276	0.89806	0.90051	0.90131	0.90396	0.90681	0.90996
919	0.89286	0.89816	0.90059	0.90139	0.90404	0.90689	0.91004
920	0.89295	0.89824	0.90068	0.90148	0.90412	0.90697	0.91011
921	0.89305	0.89834	0.90077	0.90157	0.90421	0.90705	0.91019
922	0.89315	0.89842	0.90085	0.90166	0.90429	0.90713	0.91027
923	0.89325	0.89852	0.90094	0.90174	0.90438	0.90721	0.91035
924	0.89333	0.89860	0.90103	0.90183	0.90446	0.90729	0.91042
925	0.89343	0.89869	0.90111	0.90191	0.90454	0.90737	0.91050
926	0.89352	0.89878	0.90120	0.90200	0.90462	0.90745	0.91058
927	0.89361	0.89887	0.90129	0.90208	0.90470	0.90753	0.91065
928	0.89371	0.89896	0.90137	0.90217	0.90479	0.90761	0.91073
929	0.89380	0.89904	0.90145	0.90225	0.90487	0.90768	0.91081

N	0.1%	0.5%	1%	1.25%	2.5%	5%	10%
930	0.89390	0.89913	0.90154	0.90234	0.90495	0.90776	0.91088
931	0.89399	0.89922	0.90162	0.90242	0.90503	0.90784	0.91096
932	0.89408	0.89930	0.90171	0.90250	0.90511	0.90792	0.91103
933	0.89418	0.89939	0.90179	0.90259	0.90519	0.90800	0.91111
934	0.89427	0.89948	0.90187	0.90267	0.90527	0.90808	0.91119
935	0.89436	0.89956	0.90196	0.90276	0.90535	0.90816	0.91126
936	0.89445	0.89965	0.90205	0.90284	0.90543	0.90824	0.91134
937	0.89455	0.89973	0.90213	0.90292	0.90552	0.90831	0.91141
938	0.89464	0.89982	0.90221	0.90301	0.90560	0.90839	0.91149
939	0.89473	0.89991	0.90230	0.90309	0.90568	0.90847	0.91156
940	0.89482	0.90000	0.90238	0.90317	0.90576	0.90855	0.91164
941	0.89492	0.90009	0.90247	0.90326	0.90584	0.90863	0.91171
942	0.89500	0.90017	0.90255	0.90334	0.90592	0.90870	0.91178
943	0.89509	0.90025	0.90263	0.90343	0.90600	0.90878	0.91186
944	0.89519	0.90034	0.90272	0.90351	0.90608	0.90886	0.91193
945	0.89528	0.90043	0.90280	0.90359	0.90616	0.90893	0.91201
946	0.89536	0.90051	0.90288	0.90367	0.90624	0.90901	0.91208
947	0.89545	0.90059	0.90296	0.90375	0.90632	0.90909	0.91216
948	0.89554	0.90068	0.90304	0.90384	0.90640	0.90917	0.91223
949	0.89563	0.90076	0.90313	0.90392	0.90647	0.90924	0.91230
950	0.89572	0.90085	0.90321	0.90400	0.90655	0.90932	0.91238
951	0.89581	0.90093	0.90329	0.90408	0.90663	0.90939	0.91245
952	0.89589	0.90102	0.90337	0.90417	0.90671	0.90947	0.91252
953	0.89598	0.90110	0.90345	0.90424	0.90679	0.90955	0.91260
954	0.89608	0.90119	0.90353	0.90433	0.90687	0.90962	0.91267
955	0.89617	0.90127	0.90361	0.90441	0.90695	0.90970	0.91274
956	0.89625	0.90136	0.90370	0.90449	0.90703	0.90977	0.91281
957	0.89633	0.90144	0.90378	0.90457	0.90710	0.90985	0.91289
958	0.89643	0.90152	0.90386	0.90464	0.90718	0.90993	0.91296
959	0.89651	0.90161	0.90394	0.90473	0.90726	0.91000	0.91303
960	0.89660	0.90169	0.90402	0.90480	0.90734	0.91008	0.91311
961	0.89669	0.90177	0.90411	0.90488	0.90742	0.91015	0.91318
962	0.89678	0.90186	0.90419	0.90496	0.90749	0.91023	0.91325
963	0.89686	0.90194	0.90427	0.90504	0.90757	0.91030	0.91332
964	0.89695	0.90202	0.90435	0.90512	0.90765	0.91037	0.91339
965	0.89703	0.90210	0.90443	0.90520	0.90773	0.91045	0.91346
966	0.89712	0.90219	0.90451	0.90528	0.90780	0.91052	0.91353
967	0.89721	0.90227	0.90458	0.90536	0.90788	0.91060	0.91360
968	0.89731	0.90235	0.90466	0.90544	0.90795	0.91067	0.91367
969	0.89738	0.90244	0.90474	0.90552	0.90803	0.91074	0.91374
970	0.89748	0.90252	0.90482	0.90559	0.90811	0.91082	0.91382
971	0.89756	0.90260	0.90490	0.90567	0.90818	0.91089	0.91389
972	0.89764	0.90267	0.90498	0.90575	0.90826	0.91096	0.91396
973	0.89772	0.90275	0.90506	0.90583	0.90833	0.91104	0.91403
974	0.89782	0.90283	0.90514	0.90591	0.90841	0.91111	0.91410
975	0.89789	0.90292	0.90521	0.90598	0.90849	0.91118	0.91417
976	0.89799	0.90300	0.90529	0.90606	0.90856	0.91126	0.91424
977	0.89806	0.90308	0.90537	0.90614	0.90864	0.91133	0.91431
978	0.89814	0.90316	0.90545	0.90621	0.90871	0.91140	0.91438
979	0.89822	0.90324	0.90553	0.90629	0.90878	0.91147	0.91445
980	0.89831	0.90331	0.90561	0.90637	0.90886	0.91155	0.91452
981	0.89840	0.90339	0.90568	0.90644	0.90893	0.91162	0.91459
982	0.89847	0.90347	0.90576	0.90652	0.90901	0.91169	0.91466
983	0.89857	0.90355	0.90584	0.90660	0.90908	0.91176	0.91473
984	0.89865	0.90363	0.90591	0.90667	0.90916	0.91183	0.91479
985	0.89875	0.90371	0.90599	0.90675	0.90923	0.91191	0.91486
986	0.89883	0.90379	0.90607	0.90683	0.90931	0.91198	0.91493
987	0.89892	0.90387	0.90614	0.90691	0.90938	0.91205	0.91500
988	0.89899	0.90395	0.90622	0.90698	0.90945	0.91212	0.91507
989	0.89908	0.90403	0.90629	0.90706	0.90953	0.91219	0.91514
990	0.89917	0.90411	0.90637	0.90714	0.90960	0.91227	0.91521
991	0.89925	0.90419	0.90645	0.90721	0.90967	0.91234	0.91527
992	0.89934	0.90427	0.90653	0.90729	0.90975	0.91241	0.91534
993	0.89942	0.90435	0.90660	0.90737	0.90982	0.91248	0.91541
994	0.89950	0.90442	0.90668	0.90744	0.90989	0.91255	0.91548
995	0.89958	0.90450	0.90676	0.90752	0.90997	0.91262	0.91555
996	0.89967	0.90457	0.90683	0.90759	0.91004	0.91269	0.91561
997	0.89975	0.90465	0.90691	0.90767	0.91011	0.91276	0.91568
998	0.89983	0.90473	0.90698	0.90774	0.91018	0.91283	0.91575
999	0.89992	0.90481	0.90705	0.90781	0.91025	0.91290	0.91582
1000	0.89999	0.90489	0.90713	0.90789	0.91033	0.91297	0.91588

Table 5 – *GM9(5H4L or 4H5L)*. Critical values of upper significance levels for the Grubbs multiple-9 outlier test, applicable in situations where the five highest and four lowest or four highest and five lowest observations are simultaneously identified as outliers, denoted as *GM9(5H4L)* and *GM9(4H5L)*, respectively.

N	0.1%	0.5%	1%	1.25%	2.5%	5%	10%
11	0.00000	0.00000	0.00000	0.00000	0.00000	0.00001	0.00004
12	0.00001	0.00004	0.00007	0.00009	0.00019	0.00040	0.00088
13	0.00011	0.00035	0.00058	0.00068	0.00114	0.00194	0.00339
14	0.00055	0.00130	0.00191	0.00216	0.00324	0.00492	0.00766
15	0.00150	0.00308	0.00423	0.00470	0.00657	0.00931	0.01352
16	0.00313	0.00579	0.00762	0.00833	0.01109	0.01497	0.02066
17	0.00551	0.00941	0.01197	0.01295	0.01665	0.02170	0.02885
18	0.00861	0.01388	0.01719	0.01847	0.02315	0.02934	0.03787
19	0.01244	0.01911	0.02319	0.02474	0.03036	0.03767	0.04749
20	0.01690	0.02497	0.02986	0.03165	0.03819	0.04654	0.05756
21	0.02191	0.03142	0.03703	0.03908	0.04647	0.05582	0.06796
22	0.02751	0.03837	0.04467	0.04697	0.05515	0.06542	0.07857
23	0.03337	0.04571	0.05268	0.05519	0.06414	0.07521	0.08926
24	0.03977	0.05336	0.06091	0.06367	0.07334	0.08512	0.10004
25	0.04644	0.06119	0.06939	0.07236	0.08266	0.09514	0.11079
26	0.05335	0.06928	0.07802	0.08117	0.09205	0.10519	0.12152
27	0.06037	0.07745	0.08674	0.09004	0.10146	0.11520	0.13215
28	0.06775	0.08576	0.09551	0.09897	0.11092	0.12514	0.14265
29	0.07522	0.09412	0.10430	0.10791	0.12031	0.13502	0.15302
30	0.08282	0.10253	0.11310	0.11683	0.12964	0.14479	0.16323
31	0.09042	0.11092	0.12187	0.12573	0.13895	0.15446	0.17325
32	0.09805	0.11935	0.13066	0.13462	0.14814	0.16402	0.18312
33	0.10560	0.12781	0.13937	0.14342	0.15723	0.17339	0.19284
34	0.11326	0.13617	0.14798	0.15213	0.16623	0.18265	0.20233
35	0.12113	0.14436	0.15653	0.16073	0.17507	0.19172	0.21166
36	0.12875	0.15264	0.16497	0.16925	0.18379	0.20066	0.22080
37	0.13625	0.16071	0.17330	0.17766	0.19241	0.20943	0.22973
38	0.14382	0.16866	0.18145	0.18589	0.20084	0.21807	0.23847
39	0.15132	0.17664	0.18951	0.19397	0.20912	0.22651	0.24706
40	0.15880	0.18441	0.19746	0.20198	0.21729	0.23481	0.25544
41	0.16617	0.19211	0.20527	0.20986	0.22527	0.24293	0.26367
42	0.17347	0.19968	0.21295	0.21765	0.23317	0.25094	0.27172
43	0.18061	0.20715	0.22060	0.22527	0.24090	0.25875	0.27958
44	0.18784	0.21459	0.22814	0.23278	0.24852	0.26636	0.28725
45	0.19481	0.22177	0.23547	0.24016	0.25600	0.27386	0.29479
46	0.20173	0.22891	0.24265	0.24740	0.26329	0.28121	0.30213
47	0.20855	0.23602	0.24984	0.25460	0.27046	0.28841	0.30935
48	0.21525	0.24298	0.25683	0.26157	0.27748	0.29548	0.31642
49	0.22197	0.24981	0.26368	0.26848	0.28437	0.30238	0.32334
50	0.22862	0.25660	0.27050	0.27524	0.29114	0.30915	0.33009
51	0.23512	0.26316	0.27708	0.28189	0.29784	0.31582	0.33672
52	0.24165	0.26967	0.28364	0.28838	0.30437	0.32234	0.34320
53	0.24794	0.27604	0.29001	0.29485	0.31077	0.32873	0.34954
54	0.25413	0.28233	0.29634	0.30115	0.31706	0.33501	0.35576
55	0.26008	0.28847	0.30253	0.30730	0.32325	0.34118	0.36184
56	0.26595	0.29469	0.30861	0.31343	0.32932	0.34719	0.36782
57	0.27184	0.30065	0.31461	0.31937	0.33527	0.35310	0.37367
58	0.27765	0.30653	0.32045	0.32522	0.34113	0.35891	0.37939
59	0.28357	0.31228	0.32622	0.33097	0.34682	0.36461	0.38502
60	0.28928	0.31785	0.33184	0.33662	0.35247	0.37018	0.39056
61	0.29466	0.32348	0.33744	0.34220	0.35799	0.37567	0.39594
62	0.30019	0.32899	0.34290	0.34768	0.36340	0.38106	0.40124
63	0.30572	0.33434	0.34831	0.35302	0.36876	0.38635	0.40643
64	0.31085	0.33958	0.35355	0.35830	0.37397	0.39151	0.41155
65	0.31621	0.34481	0.35872	0.36345	0.37912	0.39658	0.41655
66	0.32136	0.34995	0.36383	0.36858	0.38417	0.40157	0.42143
67	0.32647	0.35502	0.36891	0.37357	0.38917	0.40646	0.42624
68	0.33156	0.36004	0.37382	0.37850	0.39405	0.41127	0.43098
69	0.33645	0.36488	0.37864	0.38337	0.39884	0.41602	0.43563
70	0.34135	0.36975	0.38350	0.38815	0.40357	0.42068	0.44020
71	0.34625	0.37455	0.38821	0.39286	0.40819	0.42524	0.44466
72	0.35099	0.37916	0.39282	0.39747	0.41271	0.42974	0.44907
73	0.35549	0.38377	0.39736	0.40200	0.41721	0.43415	0.45340
74	0.36005	0.38827	0.40188	0.40645	0.42160	0.43849	0.45764
75	0.36464	0.39276	0.40630	0.41090	0.42596	0.44277	0.46182
76	0.36915	0.39714	0.41063	0.41521	0.43023	0.44697	0.46592
77	0.37343	0.40140	0.41489	0.41942	0.43443	0.45107	0.46996
78	0.37805	0.40567	0.41910	0.42363	0.43856	0.45513	0.47393

N	0.1%	0.5%	1%	1.25%	2.5%	5%	10%
79	0.38227	0.40986	0.42324	0.42776	0.44265	0.45914	0.47784
80	0.38628	0.41398	0.42727	0.43175	0.44661	0.46306	0.48169
81	0.39057	0.41807	0.43128	0.43575	0.45056	0.46691	0.48545
82	0.39462	0.42204	0.43523	0.43971	0.45445	0.47074	0.48916
83	0.39879	0.42600	0.43911	0.44354	0.45825	0.47448	0.49282
84	0.40273	0.42988	0.44295	0.44737	0.46199	0.47817	0.49640
85	0.40659	0.43366	0.44672	0.45115	0.46571	0.48181	0.49996
86	0.41042	0.43745	0.45043	0.45486	0.46937	0.48538	0.50345
87	0.41413	0.44122	0.45413	0.45851	0.47296	0.48888	0.50688
88	0.41796	0.44491	0.45775	0.46212	0.47652	0.49233	0.51025
89	0.42168	0.44854	0.46131	0.46566	0.48000	0.49573	0.51358
90	0.42528	0.45210	0.46482	0.46918	0.48346	0.49911	0.51685
91	0.42892	0.45561	0.46833	0.47263	0.48685	0.50242	0.52008
92	0.43266	0.45910	0.47173	0.47603	0.49020	0.50570	0.52325
93	0.43608	0.46253	0.47510	0.47937	0.49347	0.50892	0.52637
94	0.43962	0.46587	0.47846	0.48268	0.49673	0.51207	0.52947
95	0.44299	0.46915	0.48168	0.48593	0.49989	0.51520	0.53250
96	0.44642	0.47245	0.48489	0.48916	0.50308	0.51829	0.53550
97	0.44980	0.47573	0.48811	0.49236	0.50618	0.52134	0.53845
98	0.45310	0.47895	0.49130	0.49548	0.50925	0.52434	0.54138
99	0.45631	0.48212	0.49443	0.49858	0.51226	0.52729	0.54425
100	0.45945	0.48520	0.49744	0.50161	0.51523	0.53021	0.54707
101	0.46256	0.48824	0.50048	0.50463	0.51820	0.53310	0.54986
102	0.46560	0.49132	0.50348	0.50759	0.52110	0.53594	0.55261
103	0.46869	0.49430	0.50644	0.51050	0.52397	0.53874	0.55533
104	0.47182	0.49726	0.50936	0.51342	0.52680	0.54147	0.55801
105	0.47476	0.50013	0.51223	0.51628	0.52959	0.54422	0.56066
106	0.47776	0.50299	0.51507	0.51912	0.53236	0.54690	0.56326
107	0.48066	0.50590	0.51784	0.52188	0.53509	0.54956	0.56584
108	0.48360	0.50873	0.52063	0.52468	0.53781	0.55219	0.56839
109	0.48648	0.51145	0.52335	0.52739	0.54043	0.55478	0.57090
110	0.48931	0.51423	0.52607	0.53003	0.54303	0.55732	0.57337
111	0.49216	0.51692	0.52872	0.53267	0.54562	0.55984	0.57580
112	0.49485	0.51968	0.53135	0.53527	0.54816	0.56234	0.57822
113	0.49759	0.52237	0.53396	0.53783	0.55068	0.56480	0.58059
114	0.50032	0.52490	0.53650	0.54036	0.55317	0.56721	0.58295
115	0.50297	0.52751	0.53902	0.54290	0.55561	0.56960	0.58525
116	0.50563	0.53003	0.54150	0.54536	0.55806	0.57198	0.58754
117	0.50823	0.53253	0.54398	0.54785	0.56045	0.57431	0.58979
118	0.51079	0.53501	0.54644	0.55026	0.56283	0.57660	0.59202
119	0.51346	0.53747	0.54886	0.55269	0.56519	0.57889	0.59424
120	0.51602	0.53994	0.55126	0.55506	0.56751	0.58114	0.59641
121	0.51850	0.54235	0.55360	0.55740	0.56978	0.58337	0.59857
122	0.52089	0.54476	0.55592	0.55974	0.57205	0.58556	0.60070
123	0.52342	0.54712	0.55824	0.56201	0.57427	0.58771	0.60281
124	0.52588	0.54942	0.56055	0.56427	0.57651	0.58987	0.60488
125	0.52821	0.55170	0.56278	0.56651	0.57870	0.59199	0.60694
126	0.53062	0.55394	0.56498	0.56870	0.58084	0.59408	0.60898
127	0.53295	0.55619	0.56718	0.57088	0.58299	0.59616	0.61099
128	0.53534	0.55845	0.56939	0.57306	0.58508	0.59821	0.61298
129	0.53759	0.56066	0.57155	0.57520	0.58717	0.60025	0.61495
130	0.53982	0.56278	0.57368	0.57732	0.58922	0.60225	0.61689
131	0.54203	0.56492	0.57579	0.57941	0.59126	0.60424	0.61880
132	0.54430	0.56703	0.57785	0.58149	0.59328	0.60620	0.62069
133	0.54644	0.56911	0.57992	0.58354	0.59528	0.60814	0.62256
134	0.54871	0.57126	0.58192	0.58556	0.59727	0.61006	0.62442
135	0.55082	0.57332	0.58396	0.58756	0.59921	0.61197	0.62625
136	0.55294	0.57536	0.58596	0.58953	0.60112	0.61385	0.62806
137	0.55500	0.57733	0.58792	0.59150	0.60305	0.61569	0.62985
138	0.55719	0.57935	0.58989	0.59344	0.60495	0.61753	0.63163
139	0.55920	0.58132	0.59180	0.59530	0.60682	0.61936	0.63337
140	0.56122	0.58332	0.59372	0.59721	0.60866	0.62116	0.63512
141	0.56326	0.58524	0.59561	0.59909	0.61048	0.62293	0.63683
142	0.56525	0.58710	0.59747	0.60096	0.61230	0.62469	0.63854
143	0.56729	0.58904	0.59932	0.60281	0.61412	0.62643	0.64022
144	0.56926	0.59094	0.60117	0.60463	0.61590	0.62816	0.64188
145	0.57129	0.59280	0.60300	0.60645	0.61766	0.62988	0.64352
146	0.57323	0.59462	0.60480	0.60824	0.61940	0.63156	0.64517
147	0.57509	0.59640	0.60659	0.60998	0.62112	0.63323	0.64678
148	0.57700	0.59821	0.60836	0.61174	0.62283	0.63487	0.64837
149	0.57885	0.59998	0.61011	0.61349	0.62451	0.63653	0.64996
150	0.58064	0.60178	0.61185	0.61523	0.62618	0.63814	0.65151
151	0.58252	0.60356	0.61355	0.61691	0.62783	0.63973	0.65306
152	0.58435	0.60532	0.61525	0.61860	0.62946	0.64133	0.65460

N	0.1%	0.5%	1%	1.25%	2.5%	5%	10%
153	0.58621	0.60701	0.61693	0.62026	0.63110	0.64292	0.65612
154	0.58791	0.60867	0.61859	0.62194	0.63271	0.64448	0.65762
155	0.58974	0.61036	0.62027	0.62357	0.63431	0.64604	0.65910
156	0.59150	0.61205	0.62189	0.62518	0.63587	0.64756	0.66058
157	0.59317	0.61370	0.62352	0.62677	0.63744	0.64907	0.66204
158	0.59483	0.61535	0.62513	0.62835	0.63897	0.65057	0.66348
159	0.59654	0.61695	0.62671	0.62993	0.64050	0.65206	0.66491
160	0.59820	0.61859	0.62826	0.63150	0.64203	0.65353	0.66633
161	0.59987	0.62019	0.62981	0.63305	0.64352	0.65500	0.66773
162	0.60152	0.62173	0.63136	0.63456	0.64501	0.65643	0.66913
163	0.60324	0.62332	0.63288	0.63608	0.64649	0.65786	0.67051
164	0.60494	0.62489	0.63438	0.63759	0.64794	0.65927	0.67187
165	0.60649	0.62640	0.63590	0.63906	0.64940	0.66067	0.67322
166	0.60804	0.62795	0.63736	0.64052	0.65082	0.66206	0.67456
167	0.60969	0.62945	0.63882	0.64199	0.65225	0.66343	0.67589
168	0.61125	0.63095	0.64028	0.64344	0.65366	0.66479	0.67721
169	0.61278	0.63243	0.64175	0.64488	0.65504	0.66616	0.67852
170	0.61429	0.63389	0.64316	0.64629	0.65643	0.66751	0.67981
171	0.61579	0.63536	0.64461	0.64770	0.65779	0.66883	0.68110
172	0.61732	0.63686	0.64602	0.64910	0.65916	0.67015	0.68236
173	0.61874	0.63829	0.64742	0.65049	0.66051	0.67146	0.68361
174	0.62026	0.63967	0.64881	0.65186	0.66183	0.67273	0.68486
175	0.62174	0.64109	0.65019	0.65323	0.66316	0.67401	0.68609
176	0.62323	0.64248	0.65153	0.65456	0.66446	0.67528	0.68732
177	0.62466	0.64383	0.65290	0.65590	0.66578	0.67654	0.68854
178	0.62607	0.64524	0.65422	0.65724	0.66706	0.67780	0.68974
179	0.62746	0.64660	0.65557	0.65855	0.66834	0.67906	0.69093
180	0.62885	0.64793	0.65688	0.65985	0.66962	0.68028	0.69212
181	0.63029	0.64927	0.65818	0.66113	0.67087	0.68148	0.69329
182	0.63162	0.65061	0.65946	0.66243	0.67211	0.68269	0.69446
183	0.63311	0.65190	0.66076	0.66371	0.67335	0.68389	0.69561
184	0.63446	0.65323	0.66201	0.66496	0.67457	0.68508	0.69674
185	0.63580	0.65454	0.66328	0.66620	0.67580	0.68625	0.69788
186	0.63713	0.65580	0.66452	0.66744	0.67700	0.68742	0.69901
187	0.63853	0.65710	0.66577	0.66869	0.67819	0.68858	0.70013
188	0.63980	0.65833	0.66699	0.66991	0.67938	0.68973	0.70124
189	0.64114	0.65958	0.66820	0.67112	0.68057	0.69086	0.70233
190	0.64247	0.66080	0.66941	0.67232	0.68173	0.69200	0.70342
191	0.64376	0.66201	0.67061	0.67350	0.68289	0.69311	0.70450
192	0.64498	0.66327	0.67179	0.67468	0.68402	0.69423	0.70556
193	0.64617	0.66448	0.67297	0.67586	0.68517	0.69534	0.70663
194	0.64741	0.66564	0.67412	0.67700	0.68631	0.69642	0.70768
195	0.64866	0.66682	0.67532	0.67816	0.68742	0.69751	0.70873
196	0.64992	0.66802	0.67646	0.67928	0.68854	0.69858	0.70977
197	0.65122	0.66915	0.67759	0.68043	0.68964	0.69965	0.71080
198	0.65243	0.67033	0.67872	0.68155	0.69074	0.70070	0.71181
199	0.65363	0.67149	0.67987	0.68269	0.69182	0.70176	0.71283
200	0.65483	0.67264	0.68099	0.68377	0.69291	0.70280	0.71383
201	0.65606	0.67376	0.68209	0.68489	0.69398	0.70383	0.71482
202	0.65719	0.67492	0.68317	0.68598	0.69504	0.70486	0.71581
203	0.65844	0.67602	0.68428	0.68704	0.69609	0.70589	0.71679
204	0.65957	0.67713	0.68537	0.68813	0.69713	0.70689	0.71777
205	0.66070	0.67824	0.68645	0.68920	0.69817	0.70790	0.71873
206	0.66184	0.67936	0.68749	0.69027	0.69919	0.70890	0.71969
207	0.66294	0.68045	0.68857	0.69132	0.70021	0.70989	0.72064
208	0.66409	0.68149	0.68964	0.69236	0.70123	0.71087	0.72159
209	0.66521	0.68259	0.69070	0.69340	0.70224	0.71183	0.72253
210	0.66633	0.68365	0.69173	0.69442	0.70325	0.71280	0.72346
211	0.66738	0.68471	0.69275	0.69544	0.70423	0.71376	0.72438
212	0.66848	0.68575	0.69378	0.69646	0.70521	0.71472	0.72529
213	0.66958	0.68677	0.69477	0.69746	0.70620	0.71566	0.72619
214	0.67070	0.68780	0.69578	0.69846	0.70717	0.71660	0.72710
215	0.67180	0.68884	0.69678	0.69945	0.70814	0.71752	0.72800
216	0.67284	0.68987	0.69778	0.70044	0.70909	0.71845	0.72889
217	0.67386	0.69086	0.69877	0.70143	0.71004	0.71937	0.72977
218	0.67495	0.69187	0.69974	0.70238	0.71099	0.72028	0.73065
219	0.67603	0.69286	0.70071	0.70335	0.71191	0.72119	0.73152
220	0.67708	0.69388	0.70166	0.70431	0.71284	0.72209	0.73238
221	0.67812	0.69487	0.70264	0.70526	0.71376	0.72298	0.73324
222	0.67912	0.69582	0.70359	0.70619	0.71467	0.72386	0.73409
223	0.68016	0.69678	0.70452	0.70714	0.71557	0.72473	0.73493
224	0.68121	0.69773	0.70545	0.70805	0.71647	0.72562	0.73577
225	0.68224	0.69869	0.70639	0.70898	0.71737	0.72648	0.73660
226	0.68320	0.69964	0.70730	0.70990	0.71826	0.72734	0.73743

N	0.1%	0.5%	1%	1.25%	2.5%	5%	10%
227	0.68421	0.70061	0.70822	0.71080	0.71915	0.72819	0.73825
228	0.68519	0.70153	0.70912	0.71170	0.72001	0.72904	0.73907
229	0.68615	0.70247	0.71004	0.71258	0.72089	0.72987	0.73988
230	0.68711	0.70343	0.71094	0.71348	0.72176	0.73071	0.74069
231	0.68811	0.70432	0.71182	0.71437	0.72263	0.73154	0.74149
232	0.68898	0.70520	0.71273	0.71523	0.72348	0.73236	0.74228
233	0.68994	0.70611	0.71362	0.71613	0.72432	0.73319	0.74307
234	0.69088	0.70701	0.71450	0.71699	0.72516	0.73400	0.74385
235	0.69184	0.70787	0.71537	0.71786	0.72599	0.73480	0.74463
236	0.69282	0.70878	0.71624	0.71872	0.72681	0.73560	0.74540
237	0.69371	0.70964	0.71708	0.71956	0.72763	0.73640	0.74617
238	0.69467	0.71052	0.71792	0.72040	0.72846	0.73720	0.74693
239	0.69554	0.71138	0.71877	0.72125	0.72927	0.73798	0.74768
240	0.69642	0.71226	0.71961	0.72209	0.73006	0.73876	0.74843
241	0.69743	0.71310	0.72045	0.72291	0.73087	0.73953	0.74918
242	0.69834	0.71397	0.72128	0.72373	0.73167	0.74030	0.74992
243	0.69919	0.71481	0.72211	0.72454	0.73246	0.74106	0.75066
244	0.70012	0.71565	0.72294	0.72537	0.73325	0.74182	0.75138
245	0.70098	0.71648	0.72377	0.72618	0.73404	0.74259	0.75211
246	0.70183	0.71731	0.72458	0.72699	0.73481	0.74334	0.75283
247	0.70273	0.71814	0.72537	0.72777	0.73559	0.74408	0.75355
248	0.70359	0.71895	0.72615	0.72856	0.73636	0.74483	0.75426
249	0.70442	0.71978	0.72693	0.72933	0.73712	0.74556	0.75497
250	0.70520	0.72059	0.72771	0.73012	0.73787	0.74629	0.75568
251	0.70607	0.72137	0.72849	0.73089	0.73862	0.74703	0.75638
252	0.70693	0.72216	0.72926	0.73165	0.73937	0.74774	0.75707
253	0.70775	0.72295	0.73004	0.73242	0.74010	0.74846	0.75776
254	0.70860	0.72372	0.73080	0.73317	0.74084	0.74918	0.75845
255	0.70941	0.72451	0.73155	0.73392	0.74158	0.74988	0.75914
256	0.71028	0.72528	0.73233	0.73467	0.74231	0.75059	0.75981
257	0.71112	0.72603	0.73309	0.73542	0.74303	0.75130	0.76048
258	0.71190	0.72680	0.73383	0.73616	0.74375	0.75199	0.76115
259	0.71272	0.72757	0.73457	0.73689	0.74447	0.75269	0.76182
260	0.71352	0.72832	0.73530	0.73763	0.74517	0.75337	0.76248
261	0.71430	0.72907	0.73604	0.73835	0.74588	0.75405	0.76313
262	0.71507	0.72981	0.73676	0.73907	0.74659	0.75472	0.76378
263	0.71583	0.73057	0.73749	0.73979	0.74729	0.75539	0.76443
264	0.71661	0.73133	0.73821	0.74050	0.74798	0.75606	0.76507
265	0.71744	0.73205	0.73892	0.74121	0.74868	0.75673	0.76571
266	0.71825	0.73280	0.73965	0.74193	0.74935	0.75740	0.76635
267	0.71902	0.73352	0.74036	0.74264	0.75004	0.75806	0.76698
268	0.71975	0.73424	0.74105	0.74334	0.75071	0.75871	0.76761
269	0.72052	0.73498	0.74175	0.74403	0.75138	0.75935	0.76823
270	0.72127	0.73570	0.74243	0.74473	0.75205	0.76000	0.76886
271	0.72201	0.73640	0.74313	0.74542	0.75271	0.76064	0.76948
272	0.72276	0.73709	0.74383	0.74609	0.75338	0.76130	0.77009
273	0.72350	0.73781	0.74451	0.74677	0.75403	0.76193	0.77070
274	0.72423	0.73851	0.74518	0.74744	0.75468	0.76256	0.77131
275	0.72491	0.73923	0.74586	0.74811	0.75534	0.76318	0.77192
276	0.72565	0.73989	0.74656	0.74877	0.75599	0.76381	0.77251
277	0.72637	0.74058	0.74723	0.74944	0.75662	0.76443	0.77311
278	0.72711	0.74125	0.74789	0.75010	0.75726	0.76505	0.77371
279	0.72781	0.74194	0.74854	0.75075	0.75790	0.76566	0.77429
280	0.72852	0.74260	0.74920	0.75139	0.75854	0.76628	0.77488
281	0.72926	0.74329	0.74984	0.75203	0.75917	0.76688	0.77547
282	0.72995	0.74396	0.75048	0.75269	0.75980	0.76749	0.77604
283	0.73063	0.74463	0.75115	0.75333	0.76042	0.76808	0.77661
284	0.73134	0.74528	0.75178	0.75397	0.76104	0.76868	0.77719
285	0.73206	0.74595	0.75243	0.75459	0.76165	0.76927	0.77776
286	0.73273	0.74658	0.75307	0.75523	0.76226	0.76986	0.77833
287	0.73338	0.74726	0.75370	0.75584	0.76285	0.77044	0.77889
288	0.73406	0.74790	0.75433	0.75647	0.76346	0.77103	0.77945
289	0.73472	0.74854	0.75496	0.75710	0.76406	0.77161	0.78001
290	0.73533	0.74918	0.75557	0.75770	0.76466	0.77219	0.78057
291	0.73602	0.74982	0.75620	0.75832	0.76525	0.77277	0.78112
292	0.73669	0.75044	0.75681	0.75894	0.76584	0.77334	0.78166
293	0.73734	0.75107	0.75741	0.75953	0.76642	0.77390	0.78221
294	0.73802	0.75168	0.75802	0.76012	0.76701	0.77447	0.78275
295	0.73866	0.75229	0.75863	0.76072	0.76759	0.77503	0.78329
296	0.73933	0.75291	0.75924	0.76132	0.76817	0.77558	0.78383
297	0.73998	0.75352	0.75984	0.76191	0.76875	0.77614	0.78435
298	0.74063	0.75411	0.76043	0.76249	0.76931	0.77669	0.78488
299	0.74130	0.75471	0.76101	0.76308	0.76988	0.77724	0.78541
300	0.74195	0.75533	0.76159	0.76367	0.77045	0.77778	0.78593

N	0.1%	0.5%	1%	1.25%	2.5%	5%	10%
301	0.74256	0.75595	0.76217	0.76425	0.77101	0.77832	0.78645
302	0.74323	0.75655	0.76274	0.76483	0.77157	0.77886	0.78697
303	0.74387	0.75713	0.76332	0.76539	0.77212	0.77939	0.78749
304	0.74446	0.75772	0.76390	0.76596	0.77267	0.77993	0.78800
305	0.74509	0.75829	0.76448	0.76653	0.77322	0.78045	0.78851
306	0.74571	0.75886	0.76504	0.76710	0.77377	0.78099	0.78902
307	0.74629	0.75945	0.76562	0.76766	0.77431	0.78151	0.78953
308	0.74689	0.76002	0.76617	0.76822	0.77485	0.78204	0.79002
309	0.74749	0.76058	0.76673	0.76877	0.77539	0.78255	0.79052
310	0.74808	0.76115	0.76730	0.76931	0.77593	0.78306	0.79102
311	0.74867	0.76170	0.76784	0.76987	0.77646	0.78358	0.79150
312	0.74929	0.76227	0.76839	0.77042	0.77698	0.78410	0.79200
313	0.74984	0.76285	0.76894	0.77096	0.77751	0.78461	0.79249
314	0.75046	0.76341	0.76947	0.77149	0.77803	0.78512	0.79298
315	0.75106	0.76397	0.77002	0.77202	0.77856	0.78563	0.79346
316	0.75165	0.76453	0.77055	0.77257	0.77907	0.78612	0.79394
317	0.75222	0.76507	0.77111	0.77310	0.77959	0.78662	0.79442
318	0.75278	0.76561	0.77163	0.77363	0.78011	0.78712	0.79490
319	0.75338	0.76616	0.77216	0.77415	0.78062	0.78761	0.79537
320	0.75399	0.76671	0.77269	0.77467	0.78113	0.78810	0.79585
321	0.75457	0.76725	0.77320	0.77520	0.78163	0.78859	0.79631
322	0.75508	0.76777	0.77373	0.77571	0.78213	0.78907	0.79678
323	0.75563	0.76833	0.77425	0.77623	0.78263	0.78956	0.79725
324	0.75620	0.76885	0.77476	0.77675	0.78313	0.79004	0.79770
325	0.75675	0.76938	0.77527	0.77726	0.78361	0.79052	0.79816
326	0.75734	0.76989	0.77580	0.77777	0.78410	0.79099	0.79862
327	0.75787	0.77042	0.77631	0.77828	0.78459	0.79146	0.79907
328	0.75840	0.77094	0.77682	0.77878	0.78508	0.79194	0.79953
329	0.75898	0.77147	0.77733	0.77928	0.78556	0.79240	0.79997
330	0.75955	0.77198	0.77782	0.77977	0.78605	0.79287	0.80042
331	0.76011	0.77250	0.77831	0.78026	0.78653	0.79333	0.80087
332	0.76063	0.77298	0.77883	0.78077	0.78700	0.79379	0.80131
333	0.76118	0.77351	0.77932	0.78126	0.78748	0.79425	0.80175
334	0.76173	0.77402	0.77982	0.78174	0.78796	0.79470	0.80219
335	0.76223	0.77453	0.78030	0.78223	0.78842	0.79516	0.80263
336	0.76276	0.77503	0.78079	0.78271	0.78889	0.79562	0.80306
337	0.76328	0.77555	0.78127	0.78320	0.78936	0.79607	0.80349
338	0.76379	0.77605	0.78175	0.78368	0.78982	0.79652	0.80393
339	0.76429	0.77655	0.78222	0.78415	0.79029	0.79696	0.80435
340	0.76482	0.77703	0.78271	0.78463	0.79074	0.79741	0.80478
341	0.76533	0.77752	0.78319	0.78509	0.79121	0.79785	0.80520
342	0.76585	0.77800	0.78368	0.78556	0.79166	0.79829	0.80563
343	0.76637	0.77849	0.78414	0.78603	0.79211	0.79873	0.80605
344	0.76689	0.77896	0.78461	0.78649	0.79257	0.79916	0.80646
345	0.76737	0.77944	0.78507	0.78695	0.79301	0.79959	0.80688
346	0.76787	0.77992	0.78554	0.78742	0.79346	0.80003	0.80730
347	0.76836	0.78039	0.78599	0.78787	0.79391	0.80045	0.80772
348	0.76888	0.78087	0.78645	0.78833	0.79436	0.80088	0.80812
349	0.76939	0.78135	0.78693	0.78878	0.79480	0.80131	0.80853
350	0.76987	0.78181	0.78738	0.78923	0.79523	0.80173	0.80893
351	0.77036	0.78227	0.78784	0.78968	0.79567	0.80215	0.80934
352	0.77084	0.78274	0.78829	0.79012	0.79610	0.80257	0.80974
353	0.77130	0.78320	0.78873	0.79057	0.79654	0.80299	0.81015
354	0.77180	0.78365	0.78918	0.79101	0.79698	0.80340	0.81054
355	0.77230	0.78412	0.78963	0.79145	0.79741	0.80381	0.81094
356	0.77278	0.78457	0.79007	0.79189	0.79783	0.80422	0.81134
357	0.77326	0.78502	0.79051	0.79233	0.79826	0.80463	0.81173
358	0.77373	0.78547	0.79096	0.79277	0.79869	0.80504	0.81212
359	0.77420	0.78591	0.79138	0.79321	0.79910	0.80545	0.81251
360	0.77465	0.78636	0.79183	0.79364	0.79952	0.80585	0.81290
361	0.77512	0.78680	0.79226	0.79407	0.79993	0.80626	0.81328
362	0.77559	0.78725	0.79268	0.79449	0.80034	0.80666	0.81366
363	0.77607	0.78768	0.79312	0.79492	0.80075	0.80706	0.81405
364	0.77652	0.78813	0.79355	0.79534	0.80116	0.80745	0.81443
365	0.77695	0.78857	0.79399	0.79576	0.80157	0.80784	0.81480
366	0.77742	0.78901	0.79441	0.79618	0.80197	0.80824	0.81518
367	0.77789	0.78944	0.79483	0.79660	0.80238	0.80863	0.81556
368	0.77835	0.78987	0.79524	0.79701	0.80278	0.80902	0.81594
369	0.77879	0.79031	0.79566	0.79743	0.80318	0.80941	0.81631
370	0.77923	0.79073	0.79608	0.79785	0.80358	0.80979	0.81668
371	0.77967	0.79115	0.79649	0.79825	0.80399	0.81018	0.81705
372	0.78013	0.79156	0.79691	0.79867	0.80438	0.81056	0.81741
373	0.78056	0.79199	0.79732	0.79907	0.80478	0.81094	0.81778
374	0.78102	0.79241	0.79774	0.79947	0.80517	0.81132	0.81815

N	0.1%	0.5%	1%	1.25%	2.5%	5%	10%
375	0.78145	0.79282	0.79814	0.79987	0.80556	0.81169	0.81851
376	0.78191	0.79325	0.79854	0.80027	0.80594	0.81207	0.81887
377	0.78233	0.79366	0.79894	0.80067	0.80633	0.81245	0.81923
378	0.78278	0.79407	0.79934	0.80106	0.80671	0.81282	0.81959
379	0.78319	0.79450	0.79975	0.80146	0.80710	0.81319	0.81995
380	0.78361	0.79489	0.80014	0.80185	0.80748	0.81356	0.82030
381	0.78409	0.79530	0.80053	0.80226	0.80787	0.81392	0.82065
382	0.78451	0.79571	0.80093	0.80265	0.80825	0.81429	0.82100
383	0.78495	0.79611	0.80132	0.80304	0.80862	0.81466	0.82135
384	0.78537	0.79651	0.80171	0.80343	0.80900	0.81502	0.82170
385	0.78580	0.79691	0.80209	0.80381	0.80938	0.81539	0.82205
386	0.78623	0.79731	0.80247	0.80419	0.80975	0.81575	0.82240
387	0.78667	0.79770	0.80286	0.80456	0.81012	0.81610	0.82274
388	0.78710	0.79810	0.80324	0.80495	0.81049	0.81646	0.82309
389	0.78752	0.79848	0.80362	0.80533	0.81085	0.81681	0.82343
390	0.78793	0.79888	0.80401	0.80571	0.81122	0.81717	0.82377
391	0.78835	0.79929	0.80438	0.80608	0.81158	0.81752	0.82411
392	0.78877	0.79967	0.80476	0.80646	0.81194	0.81787	0.82444
393	0.78916	0.80005	0.80513	0.80683	0.81230	0.81822	0.82478
394	0.78956	0.80045	0.80552	0.80719	0.81266	0.81856	0.82511
395	0.78994	0.80084	0.80589	0.80757	0.81301	0.81891	0.82545
396	0.79034	0.80121	0.80626	0.80794	0.81338	0.81926	0.82578
397	0.79074	0.80158	0.80663	0.80831	0.81373	0.81960	0.82611
398	0.79115	0.80196	0.80700	0.80868	0.81408	0.81994	0.82644
399	0.79155	0.80234	0.80737	0.80904	0.81444	0.82028	0.82676
400	0.79196	0.80272	0.80773	0.80940	0.81479	0.82061	0.82709
401	0.79235	0.80310	0.80809	0.80976	0.81513	0.82095	0.82741
402	0.79276	0.80347	0.80846	0.81012	0.81548	0.82129	0.82774
403	0.79315	0.80384	0.80882	0.81047	0.81583	0.82163	0.82806
404	0.79354	0.80422	0.80919	0.81083	0.81618	0.82196	0.82838
405	0.79393	0.80459	0.80955	0.81119	0.81652	0.82230	0.82870
406	0.79433	0.80495	0.80990	0.81154	0.81687	0.82263	0.82902
407	0.79470	0.80532	0.81026	0.81190	0.81722	0.82295	0.82933
408	0.79512	0.80570	0.81061	0.81225	0.81756	0.82329	0.82965
409	0.79548	0.80605	0.81097	0.81261	0.81790	0.82361	0.82996
410	0.79588	0.80641	0.81132	0.81295	0.81823	0.82393	0.83027
411	0.79626	0.80677	0.81167	0.81329	0.81857	0.82426	0.83059
412	0.79662	0.80715	0.81201	0.81364	0.81889	0.82458	0.83089
413	0.79700	0.80748	0.81235	0.81397	0.81923	0.82490	0.83120
414	0.79738	0.80784	0.81269	0.81431	0.81956	0.82522	0.83151
415	0.79777	0.80818	0.81303	0.81466	0.81989	0.82554	0.83182
416	0.79813	0.80854	0.81338	0.81500	0.82022	0.82586	0.83212
417	0.79849	0.80889	0.81372	0.81533	0.82054	0.82617	0.83243
418	0.79887	0.80924	0.81406	0.81567	0.82087	0.82649	0.83273
419	0.79923	0.80958	0.81439	0.81600	0.82119	0.82680	0.83303
420	0.79965	0.80992	0.81474	0.81634	0.82152	0.82711	0.83332
421	0.79998	0.81027	0.81508	0.81666	0.82184	0.82742	0.83363
422	0.80037	0.81061	0.81540	0.81699	0.82216	0.82773	0.83393
423	0.80070	0.81096	0.81574	0.81733	0.82248	0.82804	0.83422
424	0.80107	0.81129	0.81607	0.81765	0.82280	0.82835	0.83451
425	0.80140	0.81164	0.81639	0.81798	0.82311	0.82866	0.83481
426	0.80177	0.81198	0.81673	0.81831	0.82342	0.82896	0.83510
427	0.80212	0.81230	0.81706	0.81862	0.82374	0.82927	0.83539
428	0.80248	0.81263	0.81738	0.81894	0.82405	0.82957	0.83569
429	0.80284	0.81297	0.81771	0.81927	0.82436	0.82987	0.83598
430	0.80318	0.81330	0.81803	0.81959	0.82467	0.83017	0.83626
431	0.80351	0.81363	0.81835	0.81991	0.82498	0.83047	0.83655
432	0.80389	0.81398	0.81867	0.82022	0.82528	0.83077	0.83684
433	0.80425	0.81429	0.81899	0.82054	0.82559	0.83106	0.83712
434	0.80459	0.81463	0.81931	0.82086	0.82590	0.83136	0.83741
435	0.80496	0.81495	0.81963	0.82117	0.82621	0.83166	0.83769
436	0.80529	0.81527	0.81995	0.82149	0.82652	0.83195	0.83797
437	0.80565	0.81560	0.82027	0.82181	0.82682	0.83224	0.83825
438	0.80597	0.81594	0.82058	0.82212	0.82712	0.83253	0.83853
439	0.80629	0.81624	0.82088	0.82242	0.82742	0.83282	0.83881
440	0.80664	0.81656	0.82120	0.82273	0.82772	0.83311	0.83909
441	0.80697	0.81687	0.82149	0.82304	0.82802	0.83340	0.83936
442	0.80735	0.81719	0.82181	0.82334	0.82831	0.83369	0.83964
443	0.80768	0.81750	0.82212	0.82365	0.82861	0.83397	0.83991
444	0.80797	0.81782	0.82242	0.82396	0.82890	0.83425	0.84019
445	0.80832	0.81813	0.82273	0.82426	0.82920	0.83454	0.84046
446	0.80863	0.81844	0.82302	0.82456	0.82949	0.83482	0.84073
447	0.80898	0.81875	0.82332	0.82487	0.82978	0.83511	0.84100
448	0.80931	0.81907	0.82362	0.82516	0.83008	0.83538	0.84127

N	0.1%	0.5%	1%	1.25%	2.5%	5%	10%
449	0.80966	0.81938	0.82394	0.82546	0.83037	0.83566	0.84154
450	0.80999	0.81969	0.82424	0.82576	0.83066	0.83594	0.84180
451	0.81032	0.82000	0.82454	0.82606	0.83094	0.83622	0.84207
452	0.81061	0.82031	0.82484	0.82636	0.83123	0.83650	0.84233
453	0.81094	0.82061	0.82513	0.82664	0.83151	0.83677	0.84260
454	0.81124	0.82092	0.82542	0.82693	0.83180	0.83704	0.84286
455	0.81157	0.82122	0.82571	0.82723	0.83208	0.83732	0.84312
456	0.81192	0.82152	0.82601	0.82751	0.83236	0.83759	0.84338
457	0.81224	0.82183	0.82630	0.82781	0.83264	0.83786	0.84364
458	0.81255	0.82212	0.82658	0.82809	0.83292	0.83813	0.84390
459	0.81286	0.82242	0.82688	0.82838	0.83319	0.83840	0.84416
460	0.81318	0.82273	0.82716	0.82866	0.83347	0.83867	0.84442
461	0.81351	0.82303	0.82746	0.82895	0.83374	0.83894	0.84467
462	0.81382	0.82332	0.82774	0.82924	0.83402	0.83920	0.84493
463	0.81412	0.82360	0.82803	0.82952	0.83429	0.83946	0.84518
464	0.81444	0.82389	0.82831	0.82980	0.83457	0.83973	0.84544
465	0.81473	0.82419	0.82860	0.83009	0.83484	0.84000	0.84569
466	0.81505	0.82448	0.82888	0.83036	0.83512	0.84026	0.84595
467	0.81536	0.82477	0.82916	0.83064	0.83539	0.84052	0.84620
468	0.81567	0.82505	0.82943	0.83092	0.83566	0.84078	0.84645
469	0.81596	0.82534	0.82971	0.83119	0.83592	0.84104	0.84670
470	0.81627	0.82563	0.82999	0.83147	0.83618	0.84130	0.84694
471	0.81657	0.82592	0.83026	0.83174	0.83645	0.84155	0.84719
472	0.81685	0.82620	0.83055	0.83202	0.83672	0.84181	0.84744
473	0.81715	0.82648	0.83082	0.83229	0.83698	0.84207	0.84769
474	0.81745	0.82677	0.83111	0.83256	0.83725	0.84232	0.84793
475	0.81772	0.82706	0.83138	0.83284	0.83751	0.84257	0.84817
476	0.81803	0.82734	0.83165	0.83310	0.83777	0.84283	0.84842
477	0.81834	0.82761	0.83193	0.83338	0.83803	0.84308	0.84866
478	0.81864	0.82788	0.83220	0.83364	0.83829	0.84334	0.84891
479	0.81896	0.82817	0.83247	0.83391	0.83855	0.84359	0.84914
480	0.81925	0.82844	0.83273	0.83417	0.83881	0.84384	0.84938
481	0.81954	0.82872	0.83300	0.83444	0.83907	0.84409	0.84962
482	0.81984	0.82900	0.83327	0.83470	0.83933	0.84434	0.84986
483	0.82014	0.82927	0.83354	0.83496	0.83958	0.84458	0.85010
484	0.82041	0.82954	0.83380	0.83522	0.83983	0.84483	0.85034
485	0.82069	0.82981	0.83407	0.83548	0.84009	0.84507	0.85057
486	0.82098	0.83008	0.83434	0.83574	0.84034	0.84532	0.85080
487	0.82130	0.83034	0.83460	0.83601	0.84059	0.84556	0.85104
488	0.82157	0.83062	0.83485	0.83627	0.84085	0.84581	0.85127
489	0.82187	0.83089	0.83511	0.83653	0.84109	0.84604	0.85150
490	0.82215	0.83116	0.83537	0.83678	0.84134	0.84628	0.85173
491	0.82241	0.83142	0.83563	0.83704	0.84159	0.84652	0.85197
492	0.82270	0.83169	0.83589	0.83730	0.84184	0.84676	0.85220
493	0.82297	0.83195	0.83615	0.83755	0.84208	0.84700	0.85243
494	0.82322	0.83221	0.83641	0.83780	0.84233	0.84724	0.85266
495	0.82349	0.83247	0.83667	0.83806	0.84258	0.84747	0.85288
496	0.82380	0.83273	0.83692	0.83831	0.84282	0.84771	0.85311
497	0.82406	0.83299	0.83717	0.83857	0.84306	0.84794	0.85333
498	0.82434	0.83326	0.83742	0.83882	0.84331	0.84818	0.85356
499	0.82460	0.83352	0.83767	0.83907	0.84355	0.84841	0.85378
500	0.82488	0.83378	0.83792	0.83932	0.84379	0.84865	0.85401
501	0.82515	0.83403	0.83817	0.83957	0.84404	0.84888	0.85423
502	0.82542	0.83428	0.83842	0.83981	0.84427	0.84911	0.85445
503	0.82569	0.83454	0.83867	0.84006	0.84451	0.84934	0.85467
504	0.82595	0.83479	0.83892	0.84030	0.84475	0.84957	0.85490
505	0.82621	0.83504	0.83917	0.84055	0.84499	0.84980	0.85512
506	0.82649	0.83529	0.83941	0.84079	0.84523	0.85002	0.85533
507	0.82676	0.83554	0.83966	0.84103	0.84546	0.85025	0.85555
508	0.82704	0.83579	0.83990	0.84128	0.84570	0.85048	0.85577
509	0.82729	0.83605	0.84015	0.84152	0.84593	0.85070	0.85599
510	0.82756	0.83630	0.84040	0.84176	0.84617	0.85093	0.85620
511	0.82783	0.83654	0.84064	0.84200	0.84640	0.85116	0.85642
512	0.82809	0.83679	0.84088	0.84224	0.84663	0.85138	0.85663
513	0.82835	0.83704	0.84112	0.84248	0.84686	0.85160	0.85685
514	0.82862	0.83729	0.84136	0.84271	0.84709	0.85182	0.85706
515	0.82889	0.83754	0.84159	0.84295	0.84731	0.85204	0.85727
516	0.82914	0.83779	0.84183	0.84318	0.84754	0.85226	0.85749
517	0.82938	0.83803	0.84207	0.84342	0.84777	0.85248	0.85770
518	0.82963	0.83827	0.84231	0.84365	0.84800	0.85271	0.85791
519	0.82988	0.83852	0.84255	0.84388	0.84822	0.85292	0.85812
520	0.83014	0.83875	0.84278	0.84412	0.84845	0.85314	0.85833
521	0.83041	0.83899	0.84301	0.84435	0.84867	0.85336	0.85854
522	0.83068	0.83924	0.84325	0.84458	0.84890	0.85358	0.85875

N	0.1%	0.5%	1%	1.25%	2.5%	5%	10%
523	0.83094	0.83947	0.84348	0.84480	0.84912	0.85379	0.85896
524	0.83118	0.83971	0.84371	0.84504	0.84935	0.85401	0.85916
525	0.83144	0.83994	0.84394	0.84527	0.84957	0.85422	0.85937
526	0.83168	0.84018	0.84417	0.84549	0.84979	0.85443	0.85957
527	0.83193	0.84042	0.84440	0.84572	0.85001	0.85464	0.85977
528	0.83220	0.84065	0.84463	0.84595	0.85023	0.85486	0.85998
529	0.83244	0.84089	0.84486	0.84617	0.85045	0.85507	0.86018
530	0.83270	0.84113	0.84509	0.84640	0.85067	0.85528	0.86039
531	0.83295	0.84136	0.84531	0.84663	0.85089	0.85549	0.86059
532	0.83320	0.84158	0.84554	0.84685	0.85110	0.85570	0.86079
533	0.83344	0.84183	0.84576	0.84707	0.85132	0.85591	0.86099
534	0.83367	0.84206	0.84599	0.84730	0.85153	0.85612	0.86119
535	0.83393	0.84229	0.84621	0.84751	0.85175	0.85633	0.86139
536	0.83416	0.84252	0.84643	0.84774	0.85196	0.85653	0.86159
537	0.83441	0.84275	0.84666	0.84795	0.85217	0.85674	0.86179
538	0.83464	0.84297	0.84687	0.84818	0.85239	0.85695	0.86198
539	0.83489	0.84321	0.84710	0.84840	0.85260	0.85715	0.86218
540	0.83511	0.84344	0.84732	0.84861	0.85281	0.85735	0.86238
541	0.83534	0.84367	0.84754	0.84884	0.85302	0.85756	0.86257
542	0.83559	0.84389	0.84775	0.84905	0.85323	0.85776	0.86277
543	0.83582	0.84411	0.84797	0.84926	0.85345	0.85797	0.86296
544	0.83603	0.84432	0.84819	0.84947	0.85365	0.85817	0.86315
545	0.83628	0.84455	0.84840	0.84969	0.85387	0.85837	0.86335
546	0.83652	0.84478	0.84862	0.84991	0.85407	0.85857	0.86354
547	0.83679	0.84500	0.84884	0.85012	0.85428	0.85877	0.86374
548	0.83700	0.84522	0.84905	0.85033	0.85449	0.85897	0.86393
549	0.83723	0.84544	0.84926	0.85054	0.85469	0.85917	0.86412
550	0.83746	0.84567	0.84948	0.85075	0.85490	0.85937	0.86431
551	0.83770	0.84589	0.84970	0.85097	0.85511	0.85956	0.86450
552	0.83793	0.84611	0.84990	0.85118	0.85531	0.85976	0.86469
553	0.83818	0.84634	0.85012	0.85139	0.85551	0.85996	0.86488
554	0.83841	0.84656	0.85033	0.85160	0.85572	0.86016	0.86507
555	0.83866	0.84678	0.85054	0.85181	0.85592	0.86035	0.86525
556	0.83887	0.84699	0.85075	0.85202	0.85612	0.86055	0.86544
557	0.83910	0.84719	0.85096	0.85222	0.85632	0.86074	0.86562
558	0.83931	0.84742	0.85116	0.85243	0.85652	0.86093	0.86581
559	0.83954	0.84763	0.85138	0.85264	0.85672	0.86113	0.86600
560	0.83977	0.84785	0.85158	0.85284	0.85692	0.86132	0.86618
561	0.83998	0.84806	0.85179	0.85304	0.85712	0.86151	0.86637
562	0.84020	0.84827	0.85199	0.85325	0.85732	0.86170	0.86655
563	0.84042	0.84848	0.85220	0.85346	0.85752	0.86189	0.86673
564	0.84063	0.84869	0.85241	0.85367	0.85772	0.86208	0.86692
565	0.84086	0.84890	0.85262	0.85387	0.85791	0.86227	0.86709
566	0.84107	0.84910	0.85282	0.85407	0.85811	0.86246	0.86728
567	0.84129	0.84932	0.85302	0.85427	0.85830	0.86265	0.86746
568	0.84155	0.84952	0.85323	0.85447	0.85849	0.86284	0.86764
569	0.84173	0.84973	0.85343	0.85467	0.85869	0.86303	0.86782
570	0.84194	0.84994	0.85362	0.85486	0.85888	0.86321	0.86800
571	0.84215	0.85014	0.85383	0.85506	0.85907	0.86340	0.86818
572	0.84237	0.85035	0.85403	0.85526	0.85927	0.86358	0.86835
573	0.84259	0.85055	0.85423	0.85546	0.85946	0.86376	0.86853
574	0.84280	0.85075	0.85442	0.85565	0.85965	0.86395	0.86871
575	0.84299	0.85096	0.85462	0.85585	0.85983	0.86413	0.86889
576	0.84323	0.85116	0.85482	0.85604	0.86002	0.86431	0.86906
577	0.84344	0.85136	0.85502	0.85624	0.86021	0.86450	0.86923
578	0.84364	0.85157	0.85522	0.85644	0.86040	0.86468	0.86941
579	0.84385	0.85177	0.85541	0.85663	0.86059	0.86486	0.86959
580	0.84408	0.85197	0.85560	0.85682	0.86077	0.86504	0.86976
581	0.84430	0.85217	0.85580	0.85701	0.86096	0.86522	0.86993
582	0.84451	0.85236	0.85599	0.85720	0.86114	0.86540	0.87011
583	0.84472	0.85255	0.85618	0.85740	0.86133	0.86558	0.87028
584	0.84493	0.85275	0.85637	0.85759	0.86151	0.86576	0.87045
585	0.84514	0.85295	0.85656	0.85778	0.86170	0.86593	0.87062
586	0.84533	0.85314	0.85676	0.85797	0.86188	0.86611	0.87079
587	0.84553	0.85334	0.85695	0.85816	0.86207	0.86629	0.87096
588	0.84576	0.85353	0.85714	0.85835	0.86226	0.86647	0.87113
589	0.84597	0.85373	0.85733	0.85854	0.86244	0.86664	0.87130
590	0.84617	0.85393	0.85752	0.85873	0.86262	0.86682	0.87147
591	0.84637	0.85412	0.85772	0.85892	0.86280	0.86700	0.87164
592	0.84656	0.85432	0.85791	0.85910	0.86298	0.86717	0.87181
593	0.84676	0.85450	0.85809	0.85929	0.86316	0.86734	0.87198
594	0.84698	0.85470	0.85828	0.85948	0.86334	0.86752	0.87214
595	0.84718	0.85490	0.85847	0.85967	0.86352	0.86769	0.87231
596	0.84739	0.85509	0.85866	0.85985	0.86370	0.86786	0.87247

N	0.1%	0.5%	1%	1.25%	2.5%	5%	10%
597	0.84760	0.85528	0.85884	0.86003	0.86388	0.86803	0.87264
598	0.84780	0.85546	0.85903	0.86022	0.86405	0.86820	0.87280
599	0.84799	0.85565	0.85921	0.86040	0.86423	0.86837	0.87297
600	0.84820	0.85584	0.85939	0.86058	0.86441	0.86855	0.87313
601	0.84840	0.85603	0.85957	0.86076	0.86459	0.86872	0.87330
602	0.84859	0.85621	0.85976	0.86094	0.86476	0.86889	0.87346
603	0.84881	0.85640	0.85994	0.86112	0.86494	0.86906	0.87362
604	0.84900	0.85659	0.86013	0.86130	0.86510	0.86923	0.87378
605	0.84919	0.85678	0.86031	0.86148	0.86528	0.86939	0.87394
606	0.84939	0.85696	0.86049	0.86166	0.86546	0.86956	0.87410
607	0.84958	0.85715	0.86067	0.86184	0.86563	0.86973	0.87426
608	0.84976	0.85733	0.86084	0.86201	0.86580	0.86990	0.87443
609	0.84995	0.85752	0.86102	0.86219	0.86597	0.87006	0.87459
610	0.85015	0.85770	0.86120	0.86237	0.86615	0.87023	0.87475
611	0.85034	0.85788	0.86138	0.86254	0.86632	0.87039	0.87491
612	0.85054	0.85806	0.86156	0.86272	0.86649	0.87056	0.87507
613	0.85074	0.85824	0.86173	0.86290	0.86666	0.87073	0.87522
614	0.85091	0.85841	0.86191	0.86307	0.86683	0.87089	0.87538
615	0.85110	0.85859	0.86208	0.86325	0.86700	0.87106	0.87554
616	0.85132	0.85878	0.86226	0.86342	0.86717	0.87122	0.87570
617	0.85151	0.85896	0.86243	0.86359	0.86734	0.87138	0.87585
618	0.85170	0.85914	0.86261	0.86377	0.86751	0.87154	0.87601
619	0.85190	0.85932	0.86278	0.86394	0.86767	0.87170	0.87617
620	0.85210	0.85950	0.86296	0.86412	0.86784	0.87187	0.87632
621	0.85229	0.85968	0.86313	0.86429	0.86801	0.87203	0.87648
622	0.85247	0.85986	0.86330	0.86445	0.86817	0.87219	0.87663
623	0.85266	0.86004	0.86348	0.86463	0.86834	0.87235	0.87679
624	0.85284	0.86021	0.86365	0.86480	0.86850	0.87251	0.87694
625	0.85302	0.86039	0.86382	0.86497	0.86867	0.87267	0.87709
626	0.85321	0.86056	0.86399	0.86514	0.86883	0.87283	0.87724
627	0.85340	0.86073	0.86417	0.86531	0.86899	0.87299	0.87740
628	0.85356	0.86091	0.86434	0.86547	0.86916	0.87315	0.87755
629	0.85374	0.86109	0.86451	0.86564	0.86932	0.87330	0.87770
630	0.85392	0.86125	0.86468	0.86581	0.86948	0.87346	0.87785
631	0.85409	0.86143	0.86485	0.86598	0.86965	0.87362	0.87800
632	0.85428	0.86160	0.86501	0.86614	0.86981	0.87377	0.87815
633	0.85445	0.86177	0.86518	0.86631	0.86997	0.87393	0.87830
634	0.85465	0.86194	0.86535	0.86648	0.87013	0.87409	0.87845
635	0.85483	0.86212	0.86552	0.86664	0.87029	0.87424	0.87860
636	0.85504	0.86229	0.86568	0.86681	0.87045	0.87440	0.87875
637	0.85521	0.86246	0.86585	0.86697	0.87061	0.87455	0.87890
638	0.85539	0.86264	0.86601	0.86713	0.87077	0.87471	0.87904
639	0.85557	0.86281	0.86618	0.86730	0.87093	0.87486	0.87919
640	0.85574	0.86297	0.86633	0.86746	0.87108	0.87501	0.87934
641	0.85593	0.86314	0.86650	0.86762	0.87124	0.87516	0.87948
642	0.85610	0.86331	0.86667	0.86778	0.87140	0.87531	0.87963
643	0.85627	0.86348	0.86682	0.86794	0.87155	0.87546	0.87977
644	0.85644	0.86365	0.86699	0.86810	0.87171	0.87562	0.87992
645	0.85662	0.86381	0.86715	0.86826	0.87187	0.87577	0.88006
646	0.85676	0.86398	0.86731	0.86842	0.87202	0.87591	0.88021
647	0.85696	0.86415	0.86747	0.86858	0.87218	0.87606	0.88035
648	0.85712	0.86431	0.86763	0.86874	0.87234	0.87621	0.88049
649	0.85730	0.86447	0.86779	0.86889	0.87249	0.87636	0.88064
650	0.85747	0.86463	0.86795	0.86906	0.87265	0.87651	0.88078
651	0.85765	0.86480	0.86811	0.86922	0.87280	0.87666	0.88092
652	0.85783	0.86497	0.86827	0.86937	0.87295	0.87681	0.88107
653	0.85800	0.86513	0.86843	0.86954	0.87310	0.87695	0.88121
654	0.85818	0.86530	0.86859	0.86969	0.87326	0.87710	0.88135
655	0.85833	0.86547	0.86874	0.86985	0.87341	0.87725	0.88149
656	0.85851	0.86563	0.86890	0.87000	0.87356	0.87740	0.88163
657	0.85868	0.86579	0.86906	0.87016	0.87371	0.87754	0.88177
658	0.85885	0.86595	0.86921	0.87032	0.87386	0.87769	0.88191
659	0.85901	0.86611	0.86937	0.87047	0.87401	0.87783	0.88205
660	0.85918	0.86627	0.86953	0.87062	0.87416	0.87798	0.88219
661	0.85935	0.86643	0.86968	0.87078	0.87431	0.87813	0.88233
662	0.85953	0.86658	0.86984	0.87093	0.87446	0.87827	0.88247
663	0.85969	0.86675	0.86999	0.87108	0.87461	0.87841	0.88261
664	0.85985	0.86690	0.87015	0.87123	0.87475	0.87856	0.88275
665	0.86002	0.86707	0.87031	0.87139	0.87490	0.87870	0.88288
666	0.86019	0.86722	0.87046	0.87155	0.87505	0.87884	0.88302
667	0.86036	0.86738	0.87061	0.87170	0.87520	0.87899	0.88316
668	0.86052	0.86753	0.87076	0.87184	0.87534	0.87913	0.88329
669	0.86070	0.86768	0.87091	0.87200	0.87549	0.87927	0.88343
670	0.86088	0.86784	0.87106	0.87215	0.87564	0.87941	0.88357

N	0.1%	0.5%	1%	1.25%	2.5%	5%	10%
671	0.86104	0.86800	0.87121	0.87230	0.87578	0.87955	0.88370
672	0.86120	0.86815	0.87137	0.87245	0.87593	0.87969	0.88384
673	0.86137	0.86831	0.87152	0.87260	0.87608	0.87983	0.88397
674	0.86152	0.86846	0.87167	0.87275	0.87622	0.87997	0.88411
675	0.86169	0.86861	0.87182	0.87289	0.87636	0.88011	0.88424
676	0.86184	0.86877	0.87196	0.87304	0.87650	0.88025	0.88438
677	0.86200	0.86892	0.87211	0.87319	0.87665	0.88039	0.88451
678	0.86217	0.86908	0.87227	0.87334	0.87679	0.88052	0.88464
679	0.86234	0.86923	0.87242	0.87349	0.87693	0.88066	0.88477
680	0.86251	0.86938	0.87257	0.87363	0.87707	0.88080	0.88491
681	0.86266	0.86953	0.87272	0.87378	0.87722	0.88094	0.88504
682	0.86282	0.86968	0.87286	0.87393	0.87736	0.88107	0.88517
683	0.86297	0.86983	0.87301	0.87407	0.87750	0.88121	0.88530
684	0.86313	0.86998	0.87316	0.87422	0.87764	0.88135	0.88543
685	0.86328	0.87013	0.87330	0.87437	0.87779	0.88148	0.88556
686	0.86344	0.87029	0.87345	0.87451	0.87792	0.88162	0.88569
687	0.86361	0.87044	0.87359	0.87465	0.87806	0.88175	0.88582
688	0.86376	0.87059	0.87375	0.87480	0.87820	0.88189	0.88595
689	0.86391	0.87075	0.87389	0.87494	0.87834	0.88202	0.88608
690	0.86406	0.87090	0.87403	0.87508	0.87848	0.88216	0.88621
691	0.86422	0.87105	0.87418	0.87522	0.87862	0.88229	0.88634
692	0.86439	0.87119	0.87432	0.87536	0.87875	0.88243	0.88647
693	0.86456	0.87134	0.87446	0.87550	0.87889	0.88256	0.88660
694	0.86471	0.87149	0.87460	0.87565	0.87903	0.88269	0.88672
695	0.86487	0.87163	0.87474	0.87579	0.87917	0.88283	0.88685
696	0.86502	0.87178	0.87489	0.87593	0.87930	0.88296	0.88698
697	0.86517	0.87193	0.87503	0.87607	0.87944	0.88309	0.88711
698	0.86533	0.87207	0.87517	0.87621	0.87958	0.88322	0.88724
699	0.86547	0.87222	0.87531	0.87635	0.87971	0.88335	0.88736
700	0.86563	0.87236	0.87546	0.87650	0.87984	0.88348	0.88749
701	0.86578	0.87251	0.87560	0.87664	0.87998	0.88361	0.88761
702	0.86592	0.87265	0.87574	0.87677	0.88011	0.88374	0.88774
703	0.86608	0.87279	0.87589	0.87691	0.88025	0.88387	0.88786
704	0.86624	0.87293	0.87602	0.87705	0.88038	0.88400	0.88799
705	0.86639	0.87308	0.87616	0.87719	0.88052	0.88413	0.88811
706	0.86652	0.87322	0.87631	0.87732	0.88065	0.88426	0.88824
707	0.86669	0.87337	0.87644	0.87746	0.88078	0.88439	0.88836
708	0.86683	0.87351	0.87658	0.87760	0.88092	0.88452	0.88848
709	0.86698	0.87365	0.87672	0.87774	0.88105	0.88464	0.88861
710	0.86714	0.87379	0.87685	0.87788	0.88118	0.88477	0.88873
711	0.86729	0.87393	0.87699	0.87801	0.88132	0.88490	0.88885
712	0.86744	0.87407	0.87713	0.87815	0.88145	0.88502	0.88897
713	0.86758	0.87421	0.87726	0.87828	0.88158	0.88515	0.88910
714	0.86773	0.87435	0.87740	0.87842	0.88171	0.88528	0.88922
715	0.86789	0.87449	0.87753	0.87855	0.88184	0.88540	0.88934
716	0.86803	0.87463	0.87767	0.87868	0.88197	0.88553	0.88946
717	0.86818	0.87477	0.87781	0.87881	0.88210	0.88566	0.88958
718	0.86834	0.87491	0.87794	0.87895	0.88223	0.88578	0.88970
719	0.86848	0.87505	0.87808	0.87909	0.88236	0.88591	0.88982
720	0.86863	0.87518	0.87821	0.87922	0.88249	0.88603	0.88994
721	0.86877	0.87532	0.87835	0.87935	0.88262	0.88616	0.89006
722	0.86891	0.87547	0.87848	0.87948	0.88275	0.88628	0.89018
723	0.86906	0.87560	0.87861	0.87961	0.88287	0.88640	0.89030
724	0.86920	0.87574	0.87875	0.87975	0.88300	0.88653	0.89042
725	0.86935	0.87587	0.87888	0.87988	0.88313	0.88665	0.89054
726	0.86949	0.87601	0.87901	0.88001	0.88325	0.88677	0.89065
727	0.86963	0.87615	0.87915	0.88014	0.88338	0.88689	0.89077
728	0.86977	0.87628	0.87927	0.88027	0.88351	0.88701	0.89089
729	0.86990	0.87642	0.87941	0.88041	0.88363	0.88714	0.89101
730	0.87005	0.87655	0.87954	0.88054	0.88376	0.88726	0.89112
731	0.87019	0.87669	0.87967	0.88067	0.88389	0.88738	0.89124
732	0.87033	0.87683	0.87980	0.88080	0.88401	0.88750	0.89136
733	0.87048	0.87696	0.87994	0.88093	0.88414	0.88762	0.89147
734	0.87063	0.87708	0.88007	0.88105	0.88427	0.88774	0.89159
735	0.87077	0.87722	0.88020	0.88118	0.88440	0.88786	0.89171
736	0.87092	0.87735	0.88032	0.88131	0.88452	0.88798	0.89182
737	0.87106	0.87749	0.88045	0.88144	0.88464	0.88810	0.89194
738	0.87120	0.87762	0.88058	0.88157	0.88477	0.88822	0.89205
739	0.87133	0.87775	0.88070	0.88170	0.88489	0.88834	0.89217
740	0.87147	0.87788	0.88083	0.88182	0.88501	0.88846	0.89228
741	0.87162	0.87801	0.88096	0.88195	0.88514	0.88858	0.89240
742	0.87175	0.87815	0.88109	0.88208	0.88526	0.88870	0.89251
743	0.87188	0.87828	0.88122	0.88220	0.88538	0.88882	0.89262
744	0.87202	0.87841	0.88134	0.88232	0.88550	0.88893	0.89274

N	0.1%	0.5%	1%	1.25%	2.5%	5%	10%
745	0.87217	0.87854	0.88147	0.88245	0.88562	0.88905	0.89285
746	0.87230	0.87867	0.88160	0.88257	0.88574	0.88917	0.89296
747	0.87245	0.87880	0.88173	0.88270	0.88587	0.88928	0.89307
748	0.87258	0.87893	0.88185	0.88283	0.88599	0.88940	0.89319
749	0.87272	0.87906	0.88198	0.88295	0.88611	0.88952	0.89330
750	0.87286	0.87918	0.88210	0.88308	0.88623	0.88963	0.89341
751	0.87299	0.87932	0.88223	0.88320	0.88635	0.88975	0.89352
752	0.87314	0.87944	0.88235	0.88333	0.88647	0.88987	0.89363
753	0.87327	0.87957	0.88248	0.88345	0.88659	0.88998	0.89374
754	0.87339	0.87970	0.88261	0.88357	0.88671	0.89010	0.89385
755	0.87354	0.87983	0.88273	0.88370	0.88683	0.89021	0.89396
756	0.87367	0.87995	0.88286	0.88382	0.88695	0.89033	0.89407
757	0.87381	0.88008	0.88298	0.88394	0.88707	0.89044	0.89418
758	0.87395	0.88021	0.88310	0.88406	0.88718	0.89056	0.89429
759	0.87408	0.88033	0.88323	0.88419	0.88731	0.89067	0.89440
760	0.87421	0.88046	0.88335	0.88431	0.88742	0.89078	0.89451
761	0.87434	0.88059	0.88347	0.88443	0.88754	0.89090	0.89462
762	0.87448	0.88072	0.88359	0.88455	0.88766	0.89101	0.89473
763	0.87461	0.88084	0.88371	0.88467	0.88778	0.89112	0.89484
764	0.87473	0.88096	0.88383	0.88479	0.88789	0.89124	0.89495
765	0.87487	0.88108	0.88395	0.88491	0.88801	0.89135	0.89505
766	0.87500	0.88121	0.88407	0.88503	0.88812	0.89146	0.89516
767	0.87512	0.88134	0.88419	0.88514	0.88824	0.89157	0.89527
768	0.87526	0.88146	0.88431	0.88527	0.88836	0.89168	0.89538
769	0.87539	0.88159	0.88443	0.88538	0.88847	0.89179	0.89548
770	0.87552	0.88171	0.88454	0.88550	0.88858	0.89191	0.89559
771	0.87566	0.88183	0.88467	0.88562	0.88870	0.89202	0.89570
772	0.87578	0.88195	0.88479	0.88574	0.88881	0.89213	0.89581
773	0.87591	0.88207	0.88491	0.88586	0.88892	0.89224	0.89591
774	0.87604	0.88218	0.88502	0.88597	0.88904	0.89235	0.89602
775	0.87617	0.88231	0.88514	0.88609	0.88915	0.89246	0.89612
776	0.87630	0.88243	0.88526	0.88621	0.88926	0.89257	0.89623
777	0.87642	0.88255	0.88538	0.88633	0.88937	0.89267	0.89633
778	0.87655	0.88267	0.88549	0.88644	0.88949	0.89278	0.89644
779	0.87668	0.88279	0.88561	0.88656	0.88960	0.89289	0.89654
780	0.87681	0.88292	0.88573	0.88667	0.88971	0.89300	0.89665
781	0.87694	0.88304	0.88584	0.88679	0.88983	0.89311	0.89675
782	0.87707	0.88316	0.88596	0.88690	0.88994	0.89322	0.89685
783	0.87720	0.88328	0.88608	0.88701	0.89005	0.89332	0.89696
784	0.87732	0.88340	0.88619	0.88713	0.89016	0.89343	0.89706
785	0.87746	0.88351	0.88631	0.88724	0.89027	0.89354	0.89717
786	0.87759	0.88363	0.88643	0.88736	0.89038	0.89365	0.89727
787	0.87772	0.88375	0.88655	0.88747	0.89050	0.89375	0.89737
788	0.87784	0.88387	0.88666	0.88759	0.89061	0.89386	0.89748
789	0.87797	0.88399	0.88678	0.88770	0.89072	0.89397	0.89758
790	0.87810	0.88411	0.88689	0.88781	0.89083	0.89407	0.89768
791	0.87822	0.88422	0.88700	0.88792	0.89094	0.89418	0.89778
792	0.87835	0.88434	0.88712	0.88803	0.89105	0.89428	0.89788
793	0.87847	0.88446	0.88723	0.88815	0.89116	0.89439	0.89799
794	0.87859	0.88458	0.88734	0.88826	0.89126	0.89450	0.89809
795	0.87873	0.88469	0.88745	0.88837	0.89137	0.89460	0.89819
796	0.87884	0.88480	0.88756	0.88848	0.89148	0.89471	0.89829
797	0.87897	0.88492	0.88767	0.88859	0.89159	0.89481	0.89839
798	0.87909	0.88503	0.88779	0.88871	0.89170	0.89492	0.89849
799	0.87921	0.88515	0.88790	0.88882	0.89181	0.89502	0.89859
800	0.87933	0.88526	0.88801	0.88893	0.89192	0.89513	0.89869
801	0.87944	0.88537	0.88812	0.88904	0.89203	0.89523	0.89879
802	0.87957	0.88549	0.88823	0.88915	0.89213	0.89533	0.89889
803	0.87970	0.88561	0.88834	0.88926	0.89224	0.89543	0.89899
804	0.87982	0.88572	0.88845	0.88937	0.89234	0.89554	0.89909
805	0.87993	0.88583	0.88856	0.88947	0.89245	0.89564	0.89919
806	0.88006	0.88595	0.88867	0.88958	0.89255	0.89574	0.89929
807	0.88016	0.88606	0.88878	0.88969	0.89266	0.89585	0.89939
808	0.88028	0.88617	0.88889	0.88980	0.89276	0.89595	0.89948
809	0.88040	0.88628	0.88900	0.88991	0.89287	0.89605	0.89958
810	0.88053	0.88640	0.88911	0.89002	0.89297	0.89615	0.89968
811	0.88064	0.88651	0.88922	0.89013	0.89308	0.89625	0.89978
812	0.88076	0.88662	0.88933	0.89023	0.89319	0.89636	0.89987
813	0.88088	0.88673	0.88944	0.89034	0.89329	0.89646	0.89997
814	0.88100	0.88684	0.88955	0.89045	0.89339	0.89656	0.90007
815	0.88113	0.88695	0.88966	0.89056	0.89350	0.89666	0.90016
816	0.88123	0.88706	0.88977	0.89066	0.89360	0.89676	0.90026
817	0.88135	0.88717	0.88987	0.89077	0.89370	0.89686	0.90036
818	0.88148	0.88729	0.88998	0.89088	0.89381	0.89696	0.90045

N	0.1%	0.5%	1%	1.25%	2.5%	5%	10%
819	0.88159	0.88740	0.89008	0.89098	0.89391	0.89706	0.90055
820	0.88172	0.88751	0.89019	0.89109	0.89401	0.89716	0.90064
821	0.88184	0.88761	0.89030	0.89120	0.89411	0.89726	0.90074
822	0.88196	0.88772	0.89041	0.89130	0.89422	0.89736	0.90084
823	0.88206	0.88783	0.89052	0.89141	0.89432	0.89745	0.90093
824	0.88219	0.88794	0.89062	0.89151	0.89442	0.89755	0.90103
825	0.88229	0.88805	0.89073	0.89162	0.89452	0.89765	0.90112
826	0.88241	0.88816	0.89083	0.89172	0.89462	0.89775	0.90122
827	0.88253	0.88827	0.89094	0.89183	0.89473	0.89785	0.90131
828	0.88264	0.88838	0.89104	0.89193	0.89483	0.89795	0.90141
829	0.88276	0.88848	0.89115	0.89204	0.89493	0.89805	0.90150
830	0.88287	0.88860	0.89125	0.89214	0.89503	0.89814	0.90159
831	0.88298	0.88871	0.89136	0.89225	0.89513	0.89824	0.90169
832	0.88310	0.88881	0.89147	0.89235	0.89523	0.89834	0.90178
833	0.88321	0.88892	0.89157	0.89245	0.89533	0.89843	0.90187
834	0.88332	0.88903	0.89167	0.89256	0.89543	0.89853	0.90197
835	0.88343	0.88913	0.89178	0.89266	0.89553	0.89863	0.90206
836	0.88355	0.88924	0.89188	0.89276	0.89563	0.89872	0.90215
837	0.88366	0.88935	0.89198	0.89287	0.89573	0.89882	0.90224
838	0.88376	0.88946	0.89209	0.89297	0.89583	0.89891	0.90234
839	0.88388	0.88956	0.89219	0.89307	0.89593	0.89901	0.90243
840	0.88400	0.88966	0.89229	0.89317	0.89603	0.89911	0.90252
841	0.88410	0.88977	0.89239	0.89327	0.89613	0.89920	0.90261
842	0.88421	0.88988	0.89249	0.89338	0.89622	0.89929	0.90270
843	0.88432	0.88998	0.89260	0.89348	0.89632	0.89939	0.90279
844	0.88442	0.89008	0.89270	0.89358	0.89642	0.89949	0.90289
845	0.88453	0.89019	0.89280	0.89367	0.89652	0.89958	0.90298
846	0.88464	0.89029	0.89291	0.89378	0.89662	0.89967	0.90307
847	0.88475	0.89040	0.89300	0.89388	0.89671	0.89977	0.90316
848	0.88486	0.89050	0.89311	0.89398	0.89681	0.89986	0.90325
849	0.88497	0.89060	0.89321	0.89408	0.89690	0.89996	0.90334
850	0.88508	0.89070	0.89331	0.89418	0.89700	0.90005	0.90343
851	0.88519	0.89081	0.89341	0.89428	0.89709	0.90014	0.90352
852	0.88530	0.89091	0.89351	0.89438	0.89719	0.90023	0.90361
853	0.88539	0.89102	0.89361	0.89448	0.89729	0.90033	0.90370
854	0.88551	0.89112	0.89371	0.89458	0.89739	0.90042	0.90379
855	0.88562	0.89122	0.89381	0.89468	0.89748	0.90051	0.90388
856	0.88573	0.89133	0.89391	0.89477	0.89758	0.90061	0.90396
857	0.88585	0.89143	0.89400	0.89487	0.89767	0.90070	0.90405
858	0.88595	0.89153	0.89411	0.89497	0.89777	0.90079	0.90414
859	0.88606	0.89163	0.89420	0.89507	0.89786	0.90088	0.90423
860	0.88617	0.89173	0.89430	0.89517	0.89796	0.90098	0.90432
861	0.88628	0.89183	0.89441	0.89526	0.89805	0.90107	0.90441
862	0.88640	0.89193	0.89450	0.89536	0.89815	0.90116	0.90449
863	0.88649	0.89203	0.89460	0.89546	0.89824	0.90125	0.90458
864	0.88658	0.89213	0.89470	0.89556	0.89834	0.90134	0.90467
865	0.88668	0.89223	0.89479	0.89565	0.89843	0.90143	0.90476
866	0.88680	0.89233	0.89489	0.89575	0.89852	0.90152	0.90484
867	0.88691	0.89242	0.89499	0.89585	0.89862	0.90161	0.90493
868	0.88700	0.89252	0.89509	0.89594	0.89871	0.90170	0.90502
869	0.88710	0.89262	0.89518	0.89604	0.89880	0.90180	0.90510
870	0.88721	0.89272	0.89528	0.89613	0.89889	0.90189	0.90519
871	0.88731	0.89282	0.89538	0.89623	0.89899	0.90198	0.90528
872	0.88740	0.89292	0.89547	0.89632	0.89908	0.90207	0.90536
873	0.88751	0.89302	0.89557	0.89641	0.89917	0.90215	0.90545
874	0.88762	0.89312	0.89567	0.89651	0.89926	0.90224	0.90553
875	0.88772	0.89322	0.89576	0.89660	0.89935	0.90233	0.90562
876	0.88782	0.89331	0.89586	0.89670	0.89945	0.90242	0.90571
877	0.88792	0.89341	0.89595	0.89679	0.89954	0.90251	0.90579
878	0.88802	0.89351	0.89605	0.89689	0.89963	0.90260	0.90588
879	0.88812	0.89360	0.89614	0.89698	0.89972	0.90269	0.90596
880	0.88823	0.89370	0.89623	0.89707	0.89981	0.90277	0.90605
881	0.88833	0.89380	0.89633	0.89717	0.89990	0.90286	0.90613
882	0.88843	0.89390	0.89642	0.89726	0.90000	0.90295	0.90622
883	0.88853	0.89399	0.89652	0.89735	0.90009	0.90304	0.90630
884	0.88863	0.89409	0.89661	0.89745	0.90018	0.90312	0.90638
885	0.88873	0.89419	0.89670	0.89754	0.90027	0.90321	0.90647
886	0.88884	0.89428	0.89679	0.89763	0.90036	0.90330	0.90655
887	0.88895	0.89438	0.89689	0.89772	0.90045	0.90338	0.90664
888	0.88905	0.89447	0.89698	0.89782	0.90054	0.90347	0.90672
889	0.88915	0.89457	0.89708	0.89791	0.90062	0.90356	0.90680
890	0.88926	0.89466	0.89717	0.89800	0.90071	0.90364	0.90689
891	0.88935	0.89476	0.89726	0.89809	0.90080	0.90373	0.90697
892	0.88946	0.89485	0.89736	0.89818	0.90089	0.90381	0.90705

N	0.1%	0.5%	1%	1.25%	2.5%	5%	10%
893	0.88955	0.89495	0.89745	0.89827	0.90098	0.90390	0.90713
894	0.88964	0.89504	0.89754	0.89836	0.90107	0.90398	0.90722
895	0.88974	0.89514	0.89763	0.89846	0.90116	0.90407	0.90730
896	0.88984	0.89523	0.89772	0.89854	0.90125	0.90416	0.90738
897	0.88994	0.89532	0.89782	0.89864	0.90134	0.90424	0.90746
898	0.89004	0.89542	0.89790	0.89873	0.90142	0.90433	0.90754
899	0.89013	0.89551	0.89800	0.89881	0.90151	0.90441	0.90763
900	0.89024	0.89561	0.89809	0.89891	0.90160	0.90450	0.90771
901	0.89034	0.89570	0.89817	0.89900	0.90169	0.90458	0.90779
902	0.89045	0.89579	0.89826	0.89909	0.90178	0.90467	0.90787
903	0.89053	0.89588	0.89835	0.89918	0.90186	0.90475	0.90795
904	0.89063	0.89598	0.89844	0.89927	0.90195	0.90484	0.90803
905	0.89073	0.89607	0.89854	0.89936	0.90204	0.90492	0.90811
906	0.89083	0.89616	0.89863	0.89945	0.90212	0.90500	0.90820
907	0.89093	0.89626	0.89872	0.89954	0.90221	0.90509	0.90828
908	0.89102	0.89635	0.89881	0.89963	0.90230	0.90517	0.90836
909	0.89111	0.89644	0.89890	0.89972	0.90238	0.90525	0.90844
910	0.89122	0.89653	0.89899	0.89981	0.90247	0.90534	0.90851
911	0.89131	0.89663	0.89907	0.89990	0.90255	0.90542	0.90859
912	0.89143	0.89672	0.89916	0.89998	0.90264	0.90550	0.90867
913	0.89152	0.89681	0.89925	0.90007	0.90272	0.90559	0.90875
914	0.89161	0.89690	0.89934	0.90016	0.90281	0.90567	0.90883
915	0.89171	0.89699	0.89943	0.90025	0.90289	0.90575	0.90891
916	0.89180	0.89708	0.89952	0.90034	0.90298	0.90583	0.90899
917	0.89190	0.89717	0.89960	0.90042	0.90306	0.90591	0.90907
918	0.89200	0.89726	0.89969	0.90051	0.90315	0.90599	0.90915
919	0.89209	0.89735	0.89978	0.90060	0.90323	0.90608	0.90923
920	0.89219	0.89744	0.89987	0.90069	0.90332	0.90615	0.90930
921	0.89229	0.89753	0.89996	0.90077	0.90340	0.90624	0.90938
922	0.89238	0.89762	0.90004	0.90086	0.90349	0.90632	0.90946
923	0.89248	0.89771	0.90013	0.90094	0.90357	0.90640	0.90954
924	0.89257	0.89780	0.90022	0.90103	0.90365	0.90648	0.90962
925	0.89267	0.89789	0.90031	0.90112	0.90373	0.90656	0.90969
926	0.89275	0.89797	0.90040	0.90120	0.90382	0.90664	0.90977
927	0.89285	0.89807	0.90048	0.90129	0.90390	0.90672	0.90985
928	0.89295	0.89815	0.90057	0.90137	0.90398	0.90680	0.90993
929	0.89304	0.89824	0.90065	0.90146	0.90406	0.90688	0.91000
930	0.89314	0.89833	0.90074	0.90154	0.90415	0.90696	0.91008
931	0.89323	0.89842	0.90082	0.90163	0.90423	0.90704	0.91016
932	0.89332	0.89851	0.90091	0.90171	0.90431	0.90712	0.91024
933	0.89342	0.89860	0.90099	0.90180	0.90439	0.90720	0.91031
934	0.89351	0.89868	0.90108	0.90188	0.90448	0.90728	0.91039
935	0.89360	0.89877	0.90117	0.90197	0.90456	0.90736	0.91047
936	0.89370	0.89886	0.90125	0.90205	0.90464	0.90744	0.91054
937	0.89379	0.89894	0.90133	0.90214	0.90472	0.90752	0.91062
938	0.89388	0.89903	0.90142	0.90222	0.90480	0.90760	0.91069
939	0.89398	0.89911	0.90150	0.90231	0.90488	0.90767	0.91077
940	0.89407	0.89920	0.90159	0.90239	0.90496	0.90775	0.91084
941	0.89416	0.89929	0.90168	0.90247	0.90504	0.90783	0.91092
942	0.89426	0.89938	0.90176	0.90256	0.90512	0.90791	0.91100
943	0.89435	0.89947	0.90185	0.90264	0.90520	0.90799	0.91107
944	0.89444	0.89955	0.90193	0.90273	0.90529	0.90807	0.91114
945	0.89452	0.89964	0.90201	0.90281	0.90537	0.90814	0.91122
946	0.89461	0.89972	0.90210	0.90289	0.90545	0.90822	0.91130
947	0.89470	0.89981	0.90218	0.90298	0.90553	0.90830	0.91137
948	0.89479	0.89990	0.90226	0.90306	0.90561	0.90838	0.91144
949	0.89488	0.89998	0.90235	0.90314	0.90569	0.90845	0.91152
950	0.89498	0.90006	0.90243	0.90323	0.90577	0.90853	0.91159
951	0.89506	0.90015	0.90251	0.90331	0.90585	0.90861	0.91167
952	0.89515	0.90024	0.90260	0.90339	0.90593	0.90869	0.91174
953	0.89524	0.90032	0.90268	0.90347	0.90601	0.90876	0.91182
954	0.89533	0.90041	0.90276	0.90355	0.90609	0.90884	0.91189
955	0.89542	0.90049	0.90285	0.90363	0.90617	0.90891	0.91196
956	0.89550	0.90057	0.90293	0.90371	0.90625	0.90899	0.91204
957	0.89560	0.90066	0.90300	0.90379	0.90633	0.90907	0.91211
958	0.89569	0.90074	0.90308	0.90387	0.90641	0.90914	0.91218
959	0.89578	0.90083	0.90317	0.90395	0.90648	0.90922	0.91225
960	0.89587	0.90091	0.90325	0.90404	0.90656	0.90929	0.91233
961	0.89596	0.90099	0.90333	0.90412	0.90664	0.90937	0.91240
962	0.89604	0.90108	0.90341	0.90420	0.90672	0.90945	0.91247
963	0.89613	0.90116	0.90349	0.90428	0.90680	0.90952	0.91255
964	0.89621	0.90125	0.90357	0.90436	0.90687	0.90960	0.91262
965	0.89631	0.90133	0.90366	0.90444	0.90695	0.90967	0.91269
966	0.89640	0.90141	0.90374	0.90452	0.90703	0.90975	0.91276

N	0.1%	0.5%	1%	1.25%	2.5%	5%	10%
967	0.89648	0.90149	0.90381	0.90460	0.90711	0.90982	0.91283
968	0.89657	0.90158	0.90390	0.90468	0.90718	0.90989	0.91290
969	0.89665	0.90166	0.90398	0.90476	0.90726	0.90997	0.91298
970	0.89674	0.90174	0.90406	0.90484	0.90734	0.91004	0.91305
971	0.89683	0.90182	0.90414	0.90491	0.90742	0.91012	0.91312
972	0.89690	0.90190	0.90422	0.90500	0.90749	0.91019	0.91319
973	0.89700	0.90198	0.90430	0.90508	0.90757	0.91027	0.91326
974	0.89708	0.90206	0.90438	0.90515	0.90765	0.91034	0.91333
975	0.89717	0.90214	0.90446	0.90523	0.90772	0.91041	0.91340
976	0.89725	0.90222	0.90454	0.90531	0.90780	0.91049	0.91347
977	0.89734	0.90231	0.90462	0.90539	0.90787	0.91056	0.91354
978	0.89742	0.90239	0.90470	0.90547	0.90795	0.91063	0.91361
979	0.89750	0.90247	0.90478	0.90554	0.90802	0.91070	0.91368
980	0.89758	0.90255	0.90485	0.90562	0.90810	0.91078	0.91376
981	0.89766	0.90263	0.90493	0.90570	0.90818	0.91085	0.91382
982	0.89775	0.90271	0.90501	0.90577	0.90825	0.91093	0.91389
983	0.89783	0.90279	0.90508	0.90585	0.90833	0.91100	0.91396
984	0.89793	0.90287	0.90516	0.90593	0.90840	0.91107	0.91403
985	0.89801	0.90295	0.90524	0.90600	0.90848	0.91114	0.91410
986	0.89809	0.90303	0.90532	0.90608	0.90855	0.91122	0.91417
987	0.89817	0.90311	0.90540	0.90616	0.90862	0.91129	0.91424
988	0.89825	0.90319	0.90547	0.90624	0.90870	0.91136	0.91431
989	0.89835	0.90327	0.90555	0.90631	0.90877	0.91143	0.91438
990	0.89843	0.90334	0.90563	0.90639	0.90885	0.91151	0.91445
991	0.89850	0.90343	0.90570	0.90647	0.90892	0.91158	0.91452
992	0.89860	0.90351	0.90578	0.90654	0.90900	0.91165	0.91459
993	0.89867	0.90358	0.90586	0.90662	0.90907	0.91172	0.91466
994	0.89876	0.90366	0.90594	0.90670	0.90914	0.91179	0.91472
995	0.89884	0.90374	0.90601	0.90677	0.90922	0.91186	0.91479
996	0.89892	0.90382	0.90609	0.90685	0.90929	0.91194	0.91486
997	0.89900	0.90389	0.90616	0.90692	0.90936	0.91201	0.91493
998	0.89909	0.90397	0.90624	0.90700	0.90944	0.91208	0.91500
999	0.89917	0.90405	0.90631	0.90707	0.90951	0.91215	0.91506
1000	0.89925	0.90413	0.90639	0.90715	0.90958	0.91222	0.91513
