# Supplementary material for: A generalized outlier method with an automated test protocol applied to proficiency testing schemes: utilizing Monte Carlo simulation for generating critical values for the extended Grubbs statistic
Source: J AOAC Int. 2026 Apr 29;109(6):974–84. doi: 10.1093/jaoacint/qsag036 (PMC13732683; doi:10.1093/jaoacint/qsag036)
Supplement: Supplementary file 1 [file qsag036_supplementary_data.zip › aoac-25-0307-File022.pdf]

Extended Grubbs Statistics Tables for Multiple-10 Outliers

Table 1 – *GM10(10H0L or 0H10L).* Critical values of upper significance levels for the Grubbs multiple-10 outlier test, applicable in situations where the ten highest or ten lowest observations are simultaneously identified as outliers, denoted as *GM10(10H0L)* and *GM10(0H10L)*, respectively.

N	0.1%	0.5%	1%	1.25%	2.5%	5%	10%
12	0.00000	0.00000	0.00000	0.00000	0.00001	0.00005	0.00021
13	0.00003	0.00014	0.00028	0.00036	0.00074	0.00155	0.00334
14	0.00035	0.00109	0.00178	0.00210	0.00348	0.00589	0.01025
15	0.00143	0.00339	0.00497	0.00564	0.00840	0.01274	0.01982
16	0.00351	0.00712	0.00978	0.01085	0.01514	0.02147	0.03116
17	0.00662	0.01216	0.01602	0.01752	0.02334	0.03157	0.04361
18	0.01078	0.01836	0.02338	0.02530	0.03261	0.04261	0.05677
19	0.01580	0.02549	0.03164	0.03400	0.04269	0.05428	0.07032
20	0.02165	0.03337	0.04062	0.04334	0.05329	0.06633	0.08404
21	0.02817	0.04179	0.05007	0.05314	0.06428	0.07863	0.09771
22	0.03518	0.05076	0.06000	0.06334	0.07549	0.09098	0.11134
23	0.04271	0.06010	0.07011	0.07376	0.08686	0.10334	0.12475
24	0.05055	0.06963	0.08039	0.08428	0.09827	0.11560	0.13793
25	0.05864	0.07928	0.09071	0.09490	0.10959	0.12773	0.15081
26	0.06718	0.08895	0.10114	0.10546	0.12079	0.13962	0.16339
27	0.07570	0.09885	0.11152	0.11604	0.13195	0.15133	0.17565
28	0.08430	0.10862	0.12178	0.12652	0.14290	0.16280	0.18763
29	0.09277	0.11827	0.13194	0.13682	0.15374	0.17407	0.19926
30	0.10144	0.12789	0.14199	0.14697	0.16432	0.18502	0.21058
31	0.11034	0.13748	0.15198	0.15708	0.17477	0.19581	0.22160
32	0.11887	0.14697	0.16171	0.16696	0.18498	0.20631	0.23226
33	0.12750	0.15623	0.17134	0.17667	0.19492	0.21646	0.24267
34	0.13600	0.16542	0.18080	0.18621	0.20472	0.22649	0.25276
35	0.14441	0.17448	0.19008	0.19555	0.21426	0.23612	0.26258
36	0.15295	0.18340	0.19919	0.20474	0.22355	0.24558	0.27210
37	0.16111	0.19215	0.20815	0.21373	0.23267	0.25482	0.28134
38	0.16922	0.20085	0.21689	0.22252	0.24158	0.26384	0.29037
39	0.17756	0.20920	0.22545	0.23110	0.25029	0.27258	0.29915
40	0.18553	0.21749	0.23390	0.23956	0.25882	0.28110	0.30770
41	0.19364	0.22554	0.24212	0.24783	0.26713	0.28946	0.31601
42	0.20124	0.23353	0.25019	0.25592	0.27526	0.29755	0.32407
43	0.20887	0.24141	0.25804	0.26376	0.28319	0.30549	0.33193
44	0.21640	0.24909	0.26571	0.27153	0.29098	0.31327	0.33958
45	0.22370	0.25663	0.27340	0.27921	0.29859	0.32078	0.34703
46	0.23090	0.26409	0.28079	0.28655	0.30594	0.32819	0.35428
47	0.23791	0.27136	0.28806	0.29385	0.31321	0.33533	0.36140
48	0.24485	0.27844	0.29518	0.30093	0.32026	0.34237	0.36826
49	0.25172	0.28532	0.30212	0.30789	0.32716	0.34922	0.37500
50	0.25848	0.29214	0.30884	0.31459	0.33390	0.35592	0.38157
51	0.26535	0.29884	0.31555	0.32127	0.34054	0.36246	0.38798
52	0.27175	0.30542	0.32200	0.32777	0.34702	0.36887	0.39422
53	0.27853	0.31180	0.32847	0.33413	0.35333	0.37509	0.40034
54	0.28459	0.31802	0.33465	0.34037	0.35951	0.38121	0.40632
55	0.29077	0.32423	0.34082	0.34652	0.36559	0.38718	0.41214
56	0.29703	0.33025	0.34678	0.35251	0.37155	0.39305	0.41786
57	0.30271	0.33625	0.35271	0.35842	0.37740	0.39872	0.42345
58	0.30874	0.34205	0.35859	0.36420	0.38309	0.40434	0.42889
59	0.31445	0.34784	0.36428	0.36986	0.38865	0.40981	0.43423
60	0.32001	0.35341	0.36982	0.37545	0.39414	0.41520	0.43946
61	0.32569	0.35905	0.37535	0.38086	0.39954	0.42050	0.44458
62	0.33128	0.36445	0.38068	0.38621	0.40479	0.42560	0.44958
63	0.33650	0.36976	0.38598	0.39150	0.40992	0.43066	0.45446
64	0.34175	0.37491	0.39108	0.39660	0.41497	0.43561	0.45927
65	0.34698	0.38007	0.39607	0.40165	0.41992	0.44043	0.46393
66	0.35234	0.38508	0.40110	0.40655	0.42477	0.44518	0.46856
67	0.35743	0.39004	0.40598	0.41142	0.42951	0.44982	0.47307
68	0.36234	0.39482	0.41078	0.41617	0.43420	0.45439	0.47749
69	0.36718	0.39963	0.41552	0.42090	0.43883	0.45887	0.48183
70	0.37191	0.40433	0.42018	0.42557	0.44334	0.46327	0.48609
71	0.37667	0.40889	0.42472	0.43005	0.44777	0.46759	0.49026
72	0.38140	0.41346	0.42920	0.43449	0.45214	0.47182	0.49435
73	0.38581	0.41799	0.43357	0.43886	0.45642	0.47598	0.49835
74	0.39041	0.42233	0.43791	0.44314	0.46067	0.48006	0.50229
75	0.39473	0.42668	0.44211	0.44738	0.46477	0.48407	0.50617
76	0.39918	0.43087	0.44628	0.45149	0.46880	0.48800	0.50997
77	0.40333	0.43504	0.45038	0.45555	0.47278	0.49186	0.51370

N	0.1%	0.5%	1%	1.25%	2.5%	5%	10%
78	0.40748	0.43918	0.45442	0.45959	0.47667	0.49568	0.51736
79	0.41172	0.44320	0.45840	0.46355	0.48052	0.49942	0.52097
80	0.41581	0.44712	0.46231	0.46744	0.48431	0.50307	0.52451
81	0.41981	0.45105	0.46614	0.47126	0.48799	0.50666	0.52797
82	0.42383	0.45491	0.46993	0.47499	0.49166	0.51023	0.53140
83	0.42764	0.45868	0.47366	0.47870	0.49529	0.51373	0.53476
84	0.43164	0.46242	0.47729	0.48233	0.49881	0.51716	0.53807
85	0.43546	0.46611	0.48091	0.48588	0.50228	0.52056	0.54133
86	0.43914	0.46965	0.48442	0.48939	0.50574	0.52391	0.54452
87	0.44283	0.47323	0.48797	0.49291	0.50909	0.52716	0.54766
88	0.44648	0.47673	0.49138	0.49633	0.51245	0.53039	0.55076
89	0.45005	0.48018	0.49478	0.49966	0.51568	0.53354	0.55380
90	0.45350	0.48357	0.49802	0.50292	0.51891	0.53667	0.55680
91	0.45707	0.48699	0.50131	0.50617	0.52211	0.53972	0.55977
92	0.46054	0.49027	0.50457	0.50937	0.52519	0.54275	0.56267
93	0.46409	0.49352	0.50777	0.51257	0.52827	0.54572	0.56552
94	0.46741	0.49674	0.51097	0.51572	0.53133	0.54864	0.56832
95	0.47055	0.49989	0.51400	0.51873	0.53432	0.55154	0.57110
96	0.47367	0.50303	0.51704	0.52179	0.53725	0.55439	0.57383
97	0.47689	0.50614	0.52002	0.52475	0.54015	0.55722	0.57653
98	0.48012	0.50919	0.52299	0.52767	0.54301	0.55999	0.57919
99	0.48339	0.51215	0.52591	0.53058	0.54587	0.56273	0.58178
100	0.48639	0.51512	0.52878	0.53344	0.54863	0.56541	0.58435
101	0.48936	0.51807	0.53168	0.53626	0.55138	0.56804	0.58690
102	0.49256	0.52098	0.53452	0.53905	0.55410	0.57067	0.58941
103	0.49541	0.52376	0.53724	0.54178	0.55680	0.57322	0.59188
104	0.49842	0.52654	0.53998	0.54453	0.55941	0.57577	0.59431
105	0.50134	0.52932	0.54267	0.54719	0.56201	0.57828	0.59671
106	0.50420	0.53206	0.54534	0.54983	0.56460	0.58077	0.59909
107	0.50709	0.53474	0.54799	0.55245	0.56709	0.58321	0.60143
108	0.50978	0.53741	0.55061	0.55505	0.56958	0.58561	0.60373
109	0.51245	0.54001	0.55318	0.55755	0.57205	0.58798	0.60601
110	0.51503	0.54264	0.55568	0.56004	0.57447	0.59034	0.60825
111	0.51777	0.54518	0.55812	0.56251	0.57686	0.59265	0.61045
112	0.52035	0.54773	0.56059	0.56494	0.57920	0.59494	0.61265
113	0.52311	0.55019	0.56302	0.56737	0.58157	0.59720	0.61481
114	0.52575	0.55263	0.56535	0.56971	0.58388	0.59942	0.61694
115	0.52826	0.55507	0.56776	0.57205	0.58613	0.60161	0.61905
116	0.53075	0.55743	0.57008	0.57434	0.58839	0.60377	0.62112
117	0.53328	0.55978	0.57243	0.57666	0.59059	0.60590	0.62317
118	0.53575	0.56211	0.57467	0.57891	0.59279	0.60803	0.62520
119	0.53825	0.56439	0.57694	0.58114	0.59495	0.61009	0.62719
120	0.54068	0.56670	0.57916	0.58337	0.59710	0.61217	0.62916
121	0.54308	0.56893	0.58138	0.58556	0.59920	0.61421	0.63110
122	0.54521	0.57122	0.58354	0.58770	0.60127	0.61621	0.63304
123	0.54755	0.57346	0.58569	0.58982	0.60333	0.61819	0.63494
124	0.54985	0.57561	0.58782	0.59192	0.60536	0.62014	0.63682
125	0.55214	0.57777	0.58992	0.59399	0.60739	0.62211	0.63869
126	0.55437	0.57991	0.59199	0.59604	0.60938	0.62404	0.64052
127	0.55671	0.58199	0.59403	0.59810	0.61135	0.62595	0.64232
128	0.55885	0.58407	0.59605	0.60009	0.61330	0.62782	0.64412
129	0.56094	0.58616	0.59806	0.60208	0.61525	0.62968	0.64589
130	0.56307	0.58821	0.60003	0.60405	0.61716	0.63152	0.64764
131	0.56519	0.59019	0.60201	0.60598	0.61903	0.63333	0.64937
132	0.56737	0.59216	0.60395	0.60791	0.62090	0.63513	0.65108
133	0.56946	0.59415	0.60587	0.60986	0.62272	0.63690	0.65278
134	0.57151	0.59606	0.60779	0.61172	0.62455	0.63864	0.65447
135	0.57347	0.59802	0.60964	0.61356	0.62636	0.64039	0.65612
136	0.57551	0.59994	0.61152	0.61538	0.62816	0.64209	0.65777
137	0.57750	0.60186	0.61332	0.61719	0.62989	0.64378	0.65937
138	0.57953	0.60372	0.61514	0.61901	0.63164	0.64546	0.66096
139	0.58152	0.60557	0.61697	0.62080	0.63337	0.64713	0.66255
140	0.58351	0.60740	0.61877	0.62258	0.63509	0.64879	0.66411
141	0.58538	0.60918	0.62050	0.62432	0.63676	0.65039	0.66567
142	0.58736	0.61099	0.62226	0.62606	0.63844	0.65199	0.66720
143	0.58918	0.61277	0.62401	0.62777	0.64008	0.65360	0.66872
144	0.59102	0.61450	0.62572	0.62948	0.64171	0.65518	0.67022
145	0.59290	0.61623	0.62741	0.63116	0.64333	0.65673	0.67170
146	0.59472	0.61799	0.62910	0.63280	0.64493	0.65827	0.67318
147	0.59651	0.61972	0.63075	0.63443	0.64651	0.65980	0.67463
148	0.59829	0.62136	0.63237	0.63602	0.64808	0.66130	0.67607
149	0.60001	0.62305	0.63401	0.63765	0.64963	0.66279	0.67749
150	0.60176	0.62468	0.63558	0.63925	0.65117	0.66427	0.67891
151	0.60346	0.62636	0.63718	0.64080	0.65269	0.66572	0.68030

N	0.1%	0.5%	1%	1.25%	2.5%	5%	10%
152	0.60527	0.62795	0.63874	0.64237	0.65422	0.66717	0.68168
153	0.60706	0.62955	0.64033	0.64394	0.65571	0.66861	0.68304
154	0.60864	0.63113	0.64185	0.64546	0.65719	0.67004	0.68441
155	0.61035	0.63271	0.64341	0.64698	0.65865	0.67146	0.68575
156	0.61215	0.63427	0.64490	0.64849	0.66010	0.67285	0.68707
157	0.61367	0.63581	0.64641	0.64999	0.66152	0.67423	0.68840
158	0.61529	0.63729	0.64788	0.65146	0.66295	0.67559	0.68971
159	0.61698	0.63885	0.64935	0.65289	0.66438	0.67694	0.69100
160	0.61857	0.64034	0.65084	0.65434	0.66576	0.67829	0.69229
161	0.62011	0.64186	0.65227	0.65578	0.66713	0.67962	0.69355
162	0.62166	0.64328	0.65370	0.65718	0.66852	0.68094	0.69480
163	0.62316	0.64478	0.65509	0.65858	0.66988	0.68224	0.69606
164	0.62466	0.64621	0.65650	0.65993	0.67121	0.68353	0.69728
165	0.62619	0.64764	0.65791	0.66132	0.67253	0.68481	0.69851
166	0.62769	0.64911	0.65927	0.66269	0.67386	0.68608	0.69972
167	0.62920	0.65050	0.66066	0.66406	0.67516	0.68733	0.70093
168	0.63064	0.65188	0.66201	0.66540	0.67645	0.68857	0.70212
169	0.63212	0.65329	0.66336	0.66673	0.67774	0.68980	0.70330
170	0.63358	0.65466	0.66469	0.66807	0.67902	0.69102	0.70446
171	0.63495	0.65604	0.66601	0.66934	0.68026	0.69223	0.70561
172	0.63645	0.65738	0.66731	0.67063	0.68152	0.69344	0.70675
173	0.63782	0.65872	0.66861	0.67192	0.68277	0.69464	0.70789
174	0.63932	0.66007	0.66989	0.67319	0.68399	0.69581	0.70901
175	0.64071	0.66135	0.67116	0.67446	0.68522	0.69698	0.71012
176	0.64205	0.66267	0.67243	0.67569	0.68642	0.69813	0.71122
177	0.64339	0.66394	0.67366	0.67691	0.68761	0.69928	0.71233
178	0.64473	0.66519	0.67489	0.67813	0.68880	0.70042	0.71342
179	0.64615	0.66650	0.67611	0.67935	0.68996	0.70155	0.71449
180	0.64746	0.66774	0.67732	0.68056	0.69115	0.70268	0.71557
181	0.64877	0.66900	0.67854	0.68174	0.69231	0.70380	0.71662
182	0.65008	0.67025	0.67977	0.68294	0.69346	0.70489	0.71767
183	0.65148	0.67148	0.68094	0.68411	0.69460	0.70597	0.71870
184	0.65270	0.67269	0.68212	0.68529	0.69572	0.70707	0.71974
185	0.65398	0.67387	0.68327	0.68641	0.69684	0.70814	0.72076
186	0.65514	0.67507	0.68442	0.68759	0.69797	0.70920	0.72177
187	0.65643	0.67624	0.68556	0.68871	0.69906	0.71027	0.72278
188	0.65761	0.67742	0.68670	0.68984	0.70016	0.71132	0.72378
189	0.65878	0.67858	0.68782	0.69097	0.70124	0.71236	0.72476
190	0.66010	0.67975	0.68896	0.69207	0.70229	0.71338	0.72574
191	0.66133	0.68086	0.69008	0.69316	0.70334	0.71440	0.72671
192	0.66255	0.68201	0.69120	0.69428	0.70439	0.71540	0.72767
193	0.66374	0.68314	0.69229	0.69538	0.70541	0.71641	0.72863
194	0.66495	0.68427	0.69340	0.69643	0.70646	0.71739	0.72957
195	0.66613	0.68539	0.69445	0.69750	0.70749	0.71838	0.73052
196	0.66726	0.68649	0.69553	0.69855	0.70851	0.71935	0.73146
197	0.66849	0.68761	0.69658	0.69960	0.70952	0.72031	0.73238
198	0.66957	0.68867	0.69763	0.70065	0.71054	0.72128	0.73329
199	0.67067	0.68976	0.69868	0.70168	0.71153	0.72225	0.73422
200	0.67178	0.69084	0.69971	0.70270	0.71251	0.72321	0.73511
201	0.67289	0.69190	0.70074	0.70373	0.71349	0.72416	0.73601
202	0.67398	0.69293	0.70177	0.70475	0.71447	0.72509	0.73690
203	0.67512	0.69394	0.70279	0.70574	0.71544	0.72603	0.73779
204	0.67623	0.69497	0.70377	0.70673	0.71639	0.72693	0.73866
205	0.67731	0.69600	0.70477	0.70772	0.71733	0.72784	0.73954
206	0.67841	0.69698	0.70576	0.70869	0.71827	0.72876	0.74040
207	0.67950	0.69800	0.70672	0.70967	0.71921	0.72965	0.74126
208	0.68052	0.69902	0.70770	0.71063	0.72014	0.73053	0.74211
209	0.68164	0.70000	0.70866	0.71157	0.72106	0.73143	0.74296
210	0.68270	0.70100	0.70963	0.71254	0.72198	0.73232	0.74379
211	0.68377	0.70199	0.71060	0.71347	0.72289	0.73319	0.74463
212	0.68482	0.70297	0.71154	0.71440	0.72378	0.73406	0.74545
213	0.68582	0.70392	0.71247	0.71533	0.72468	0.73492	0.74627
214	0.68682	0.70492	0.71340	0.71627	0.72557	0.73578	0.74708
215	0.68784	0.70585	0.71434	0.71718	0.72646	0.73662	0.74789
216	0.68883	0.70681	0.71525	0.71808	0.72734	0.73746	0.74869
217	0.68984	0.70770	0.71617	0.71898	0.72821	0.73830	0.74949
218	0.69087	0.70865	0.71709	0.71988	0.72909	0.73913	0.75028
219	0.69183	0.70961	0.71799	0.72078	0.72996	0.73995	0.75106
220	0.69280	0.71049	0.71887	0.72166	0.73081	0.74077	0.75185
221	0.69375	0.71141	0.71975	0.72252	0.73166	0.74157	0.75262
222	0.69475	0.71231	0.72063	0.72340	0.73249	0.74239	0.75340
223	0.69572	0.71323	0.72150	0.72427	0.73333	0.74319	0.75415
224	0.69663	0.71410	0.72236	0.72511	0.73417	0.74398	0.75490
225	0.69756	0.71500	0.72320	0.72596	0.73497	0.74477	0.75566

N	0.1%	0.5%	1%	1.25%	2.5%	5%	10%
226	0.69850	0.71587	0.72407	0.72682	0.73580	0.74555	0.75640
227	0.69945	0.71674	0.72489	0.72764	0.73661	0.74632	0.75714
228	0.70031	0.71759	0.72577	0.72849	0.73741	0.74710	0.75788
229	0.70122	0.71844	0.72660	0.72932	0.73821	0.74787	0.75861
230	0.70210	0.71932	0.72744	0.73014	0.73902	0.74864	0.75934
231	0.70297	0.72018	0.72826	0.73097	0.73979	0.74939	0.76006
232	0.70390	0.72103	0.72908	0.73177	0.74057	0.75014	0.76078
233	0.70477	0.72187	0.72991	0.73259	0.74135	0.75089	0.76149
234	0.70572	0.72269	0.73071	0.73339	0.74211	0.75162	0.76220
235	0.70658	0.72351	0.73153	0.73418	0.74289	0.75235	0.76291
236	0.70745	0.72434	0.73233	0.73497	0.74364	0.75309	0.76360
237	0.70829	0.72518	0.73310	0.73578	0.74439	0.75381	0.76429
238	0.70919	0.72600	0.73391	0.73656	0.74515	0.75454	0.76498
239	0.71004	0.72679	0.73470	0.73734	0.74590	0.75526	0.76566
240	0.71087	0.72758	0.73547	0.73811	0.74664	0.75597	0.76635
241	0.71171	0.72838	0.73624	0.73887	0.74739	0.75667	0.76703
242	0.71257	0.72917	0.73700	0.73961	0.74811	0.75738	0.76769
243	0.71338	0.72998	0.73776	0.74037	0.74884	0.75807	0.76836
244	0.71426	0.73075	0.73854	0.74114	0.74957	0.75876	0.76901
245	0.71506	0.73152	0.73928	0.74187	0.75028	0.75946	0.76967
246	0.71590	0.73229	0.74003	0.74261	0.75099	0.76015	0.77032
247	0.71672	0.73308	0.74076	0.74334	0.75170	0.76082	0.77097
248	0.71753	0.73383	0.74150	0.74408	0.75241	0.76150	0.77161
249	0.71837	0.73457	0.74224	0.74481	0.75311	0.76217	0.77225
250	0.71911	0.73530	0.74297	0.74553	0.75380	0.76284	0.77289
251	0.71992	0.73606	0.74369	0.74624	0.75450	0.76351	0.77353
252	0.72070	0.73679	0.74439	0.74695	0.75518	0.76417	0.77415
253	0.72148	0.73752	0.74510	0.74764	0.75586	0.76482	0.77478
254	0.72229	0.73824	0.74582	0.74836	0.75654	0.76547	0.77540
255	0.72310	0.73899	0.74653	0.74908	0.75722	0.76612	0.77601
256	0.72389	0.73972	0.74723	0.74976	0.75789	0.76677	0.77663
257	0.72467	0.74045	0.74792	0.75043	0.75856	0.76740	0.77724
258	0.72544	0.74117	0.74861	0.75112	0.75922	0.76804	0.77784
259	0.72619	0.74188	0.74929	0.75180	0.75988	0.76866	0.77844
260	0.72693	0.74256	0.74997	0.75247	0.76054	0.76929	0.77904
261	0.72764	0.74328	0.75066	0.75314	0.76118	0.76992	0.77963
262	0.72836	0.74396	0.75132	0.75382	0.76182	0.77053	0.78022
263	0.72908	0.74466	0.75198	0.75448	0.76247	0.77115	0.78081
264	0.72981	0.74533	0.75264	0.75513	0.76311	0.77175	0.78139
265	0.73048	0.74601	0.75330	0.75577	0.76374	0.77236	0.78197
266	0.73132	0.74670	0.75397	0.75642	0.76437	0.77296	0.78254
267	0.73201	0.74740	0.75463	0.75707	0.76499	0.77357	0.78311
268	0.73275	0.74807	0.75530	0.75771	0.76561	0.77416	0.78368
269	0.73340	0.74876	0.75594	0.75836	0.76623	0.77476	0.78425
270	0.73411	0.74942	0.75659	0.75899	0.76684	0.77536	0.78481
271	0.73484	0.75009	0.75721	0.75962	0.76746	0.77595	0.78537
272	0.73552	0.75074	0.75786	0.76025	0.76807	0.77653	0.78593
273	0.73618	0.75141	0.75849	0.76089	0.76868	0.77711	0.78648
274	0.73687	0.75206	0.75913	0.76150	0.76927	0.77769	0.78703
275	0.73756	0.75270	0.75975	0.76211	0.76986	0.77826	0.78757
276	0.73824	0.75336	0.76037	0.76273	0.77044	0.77883	0.78812
277	0.73895	0.75399	0.76099	0.76334	0.77103	0.77939	0.78866
278	0.73962	0.75462	0.76159	0.76396	0.77162	0.77996	0.78920
279	0.74031	0.75525	0.76221	0.76456	0.77221	0.78052	0.78973
280	0.74099	0.75587	0.76280	0.76516	0.77279	0.78108	0.79026
281	0.74164	0.75650	0.76341	0.76576	0.77336	0.78163	0.79078
282	0.74230	0.75712	0.76400	0.76636	0.77392	0.78218	0.79131
283	0.74297	0.75776	0.76461	0.76694	0.77449	0.78273	0.79184
284	0.74363	0.75837	0.76521	0.76753	0.77506	0.78328	0.79235
285	0.74430	0.75897	0.76581	0.76810	0.77561	0.78381	0.79286
286	0.74494	0.75960	0.76640	0.76869	0.77619	0.78435	0.79338
287	0.74558	0.76021	0.76698	0.76927	0.77675	0.78488	0.79389
288	0.74625	0.76081	0.76758	0.76985	0.77730	0.78542	0.79440
289	0.74685	0.76140	0.76815	0.77042	0.77785	0.78595	0.79490
290	0.74747	0.76198	0.76872	0.77100	0.77840	0.78647	0.79540
291	0.74807	0.76257	0.76929	0.77157	0.77894	0.78699	0.79590
292	0.74870	0.76315	0.76985	0.77212	0.77949	0.78751	0.79639
293	0.74935	0.76373	0.77041	0.77268	0.78003	0.78803	0.79688
294	0.74994	0.76433	0.77099	0.77324	0.78057	0.78854	0.79737
295	0.75061	0.76489	0.77155	0.77379	0.78111	0.78905	0.79786
296	0.75124	0.76546	0.77211	0.77435	0.78164	0.78956	0.79834
297	0.75183	0.76601	0.77265	0.77489	0.78216	0.79007	0.79883
298	0.75249	0.76660	0.77320	0.77542	0.78267	0.79057	0.79931
299	0.75308	0.76716	0.77375	0.77596	0.78320	0.79107	0.79978

N	0.1%	0.5%	1%	1.25%	2.5%	5%	10%
300	0.75365	0.76772	0.77428	0.77650	0.78372	0.79157	0.80026
301	0.75425	0.76827	0.77481	0.77704	0.78424	0.79207	0.80073
302	0.75487	0.76883	0.77535	0.77756	0.78474	0.79256	0.80119
303	0.75545	0.76938	0.77590	0.77810	0.78525	0.79304	0.80166
304	0.75603	0.76993	0.77642	0.77862	0.78577	0.79353	0.80212
305	0.75664	0.77049	0.77695	0.77914	0.78627	0.79401	0.80258
306	0.75722	0.77104	0.77747	0.77965	0.78678	0.79450	0.80305
307	0.75777	0.77158	0.77801	0.78018	0.78727	0.79497	0.80350
308	0.75837	0.77210	0.77853	0.78069	0.78777	0.79545	0.80396
309	0.75895	0.77263	0.77905	0.78120	0.78826	0.79593	0.80441
310	0.75948	0.77316	0.77957	0.78171	0.78876	0.79640	0.80486
311	0.76008	0.77370	0.78007	0.78223	0.78925	0.79686	0.80531
312	0.76063	0.77423	0.78059	0.78274	0.78974	0.79733	0.80576
313	0.76119	0.77475	0.78109	0.78324	0.79022	0.79780	0.80620
314	0.76173	0.77528	0.78159	0.78373	0.79071	0.79825	0.80664
315	0.76228	0.77580	0.78211	0.78423	0.79119	0.79872	0.80708
316	0.76281	0.77631	0.78259	0.78472	0.79166	0.79917	0.80752
317	0.76338	0.77681	0.78310	0.78520	0.79214	0.79963	0.80796
318	0.76390	0.77732	0.78358	0.78569	0.79261	0.80008	0.80838
319	0.76442	0.77783	0.78408	0.78618	0.79308	0.80053	0.80882
320	0.76501	0.77834	0.78457	0.78667	0.79355	0.80098	0.80924
321	0.76556	0.77884	0.78506	0.78714	0.79400	0.80143	0.80967
322	0.76608	0.77933	0.78555	0.78762	0.79447	0.80188	0.81009
323	0.76661	0.77983	0.78602	0.78810	0.79494	0.80232	0.81051
324	0.76713	0.78031	0.78650	0.78858	0.79540	0.80275	0.81093
325	0.76767	0.78082	0.78699	0.78907	0.79586	0.80320	0.81135
326	0.76820	0.78130	0.78745	0.78953	0.79631	0.80362	0.81177
327	0.76870	0.78180	0.78793	0.79000	0.79676	0.80406	0.81217
328	0.76921	0.78228	0.78839	0.79046	0.79721	0.80449	0.81258
329	0.76973	0.78277	0.78886	0.79092	0.79766	0.80492	0.81299
330	0.77025	0.78325	0.78933	0.79139	0.79810	0.80533	0.81340
331	0.77072	0.78372	0.78979	0.79184	0.79853	0.80577	0.81380
332	0.77125	0.78420	0.79026	0.79230	0.79897	0.80619	0.81420
333	0.77175	0.78467	0.79073	0.79276	0.79941	0.80661	0.81461
334	0.77229	0.78515	0.79119	0.79320	0.79985	0.80702	0.81500
335	0.77275	0.78562	0.79164	0.79366	0.80028	0.80744	0.81540
336	0.77322	0.78608	0.79209	0.79409	0.80070	0.80785	0.81580
337	0.77373	0.78657	0.79253	0.79454	0.80113	0.80827	0.81619
338	0.77420	0.78702	0.79298	0.79499	0.80156	0.80868	0.81658
339	0.77469	0.78747	0.79343	0.79544	0.80199	0.80909	0.81697
340	0.77518	0.78792	0.79388	0.79587	0.80241	0.80950	0.81735
341	0.77569	0.78837	0.79431	0.79632	0.80284	0.80990	0.81774
342	0.77617	0.78881	0.79475	0.79675	0.80326	0.81030	0.81812
343	0.77664	0.78925	0.79520	0.79718	0.80368	0.81071	0.81850
344	0.77710	0.78971	0.79564	0.79761	0.80410	0.81111	0.81888
345	0.77754	0.79016	0.79608	0.79804	0.80450	0.81150	0.81926
346	0.77802	0.79061	0.79651	0.79847	0.80491	0.81190	0.81963
347	0.77846	0.79105	0.79693	0.79891	0.80532	0.81229	0.82001
348	0.77900	0.79150	0.79735	0.79933	0.80573	0.81268	0.82038
349	0.77945	0.79194	0.79777	0.79976	0.80614	0.81307	0.82075
350	0.77992	0.79238	0.79820	0.80017	0.80654	0.81345	0.82112
351	0.78037	0.79281	0.79862	0.80060	0.80694	0.81384	0.82149
352	0.78082	0.79324	0.79905	0.80102	0.80735	0.81422	0.82186
353	0.78127	0.79367	0.79946	0.80143	0.80775	0.81460	0.82222
354	0.78175	0.79410	0.79989	0.80184	0.80815	0.81498	0.82258
355	0.78221	0.79452	0.80031	0.80224	0.80854	0.81536	0.82294
356	0.78267	0.79494	0.80071	0.80265	0.80893	0.81573	0.82330
357	0.78311	0.79539	0.80112	0.80305	0.80932	0.81611	0.82366
358	0.78357	0.79579	0.80153	0.80345	0.80971	0.81649	0.82402
359	0.78403	0.79621	0.80193	0.80386	0.81010	0.81685	0.82437
360	0.78442	0.79663	0.80233	0.80426	0.81048	0.81723	0.82473
361	0.78485	0.79704	0.80273	0.80465	0.81087	0.81760	0.82508
362	0.78534	0.79745	0.80314	0.80504	0.81125	0.81796	0.82543
363	0.78576	0.79786	0.80353	0.80543	0.81163	0.81834	0.82577
364	0.78620	0.79826	0.80393	0.80584	0.81201	0.81870	0.82612
365	0.78663	0.79866	0.80432	0.80622	0.81238	0.81905	0.82646
366	0.78706	0.79904	0.80472	0.80660	0.81275	0.81942	0.82681
367	0.78749	0.79945	0.80511	0.80699	0.81313	0.81978	0.82715
368	0.78792	0.79986	0.80550	0.80738	0.81350	0.82014	0.82749
369	0.78836	0.80026	0.80589	0.80776	0.81387	0.82049	0.82783
370	0.78875	0.80066	0.80628	0.80814	0.81424	0.82085	0.82817
371	0.78919	0.80105	0.80666	0.80853	0.81460	0.82120	0.82851
372	0.78959	0.80145	0.80705	0.80891	0.81497	0.82155	0.82884
373	0.79000	0.80185	0.80742	0.80929	0.81533	0.82190	0.82917

N	0.1%	0.5%	1%	1.25%	2.5%	5%	10%
374	0.79045	0.80224	0.80781	0.80967	0.81570	0.82224	0.82951
375	0.79084	0.80261	0.80819	0.81005	0.81606	0.82260	0.82984
376	0.79127	0.80299	0.80856	0.81043	0.81642	0.82295	0.83016
377	0.79170	0.80340	0.80894	0.81079	0.81677	0.82329	0.83049
378	0.79211	0.80378	0.80931	0.81116	0.81713	0.82363	0.83082
379	0.79248	0.80416	0.80968	0.81152	0.81748	0.82396	0.83114
380	0.79286	0.80456	0.81004	0.81188	0.81784	0.82431	0.83146
381	0.79328	0.80493	0.81041	0.81225	0.81818	0.82464	0.83179
382	0.79365	0.80532	0.81078	0.81260	0.81853	0.82497	0.83210
383	0.79405	0.80571	0.81115	0.81297	0.81887	0.82530	0.83242
384	0.79444	0.80607	0.81151	0.81331	0.81922	0.82563	0.83274
385	0.79482	0.80645	0.81187	0.81367	0.81957	0.82597	0.83305
386	0.79526	0.80683	0.81223	0.81403	0.81991	0.82630	0.83337
387	0.79563	0.80720	0.81258	0.81440	0.82025	0.82663	0.83368
388	0.79600	0.80757	0.81295	0.81475	0.82059	0.82696	0.83399
389	0.79640	0.80794	0.81330	0.81510	0.82093	0.82729	0.83430
390	0.79679	0.80829	0.81365	0.81544	0.82127	0.82761	0.83461
391	0.79715	0.80866	0.81401	0.81578	0.82160	0.82793	0.83492
392	0.79756	0.80902	0.81436	0.81613	0.82194	0.82826	0.83523
393	0.79796	0.80938	0.81471	0.81648	0.82227	0.82857	0.83553
394	0.79836	0.80974	0.81506	0.81683	0.82261	0.82890	0.83583
395	0.79873	0.81010	0.81540	0.81717	0.82294	0.82921	0.83614
396	0.79910	0.81046	0.81575	0.81751	0.82327	0.82952	0.83644
397	0.79949	0.81082	0.81609	0.81785	0.82359	0.82984	0.83674
398	0.79985	0.81117	0.81643	0.81818	0.82392	0.83016	0.83704
399	0.80024	0.81151	0.81678	0.81852	0.82424	0.83046	0.83734
400	0.80059	0.81187	0.81711	0.81886	0.82457	0.83078	0.83764
401	0.80098	0.81222	0.81744	0.81920	0.82489	0.83109	0.83793
402	0.80134	0.81258	0.81778	0.81953	0.82522	0.83139	0.83823
403	0.80172	0.81292	0.81811	0.81986	0.82554	0.83170	0.83852
404	0.80207	0.81326	0.81845	0.82020	0.82586	0.83201	0.83881
405	0.80242	0.81360	0.81878	0.82053	0.82618	0.83231	0.83910
406	0.80276	0.81396	0.81912	0.82085	0.82649	0.83262	0.83939
407	0.80316	0.81430	0.81945	0.82118	0.82681	0.83292	0.83968
408	0.80350	0.81463	0.81978	0.82151	0.82712	0.83322	0.83997
409	0.80388	0.81498	0.82011	0.82183	0.82744	0.83352	0.84025
410	0.80424	0.81531	0.82044	0.82215	0.82774	0.83382	0.84054
411	0.80459	0.81565	0.82076	0.82247	0.82805	0.83412	0.84082
412	0.80494	0.81598	0.82108	0.82279	0.82836	0.83442	0.84111
413	0.80529	0.81631	0.82141	0.82311	0.82867	0.83471	0.84139
414	0.80566	0.81664	0.82173	0.82342	0.82897	0.83500	0.84167
415	0.80600	0.81696	0.82205	0.82375	0.82928	0.83529	0.84194
416	0.80633	0.81728	0.82236	0.82405	0.82958	0.83558	0.84223
417	0.80668	0.81762	0.82268	0.82437	0.82989	0.83587	0.84250
418	0.80702	0.81793	0.82300	0.82468	0.83019	0.83617	0.84278
419	0.80733	0.81828	0.82331	0.82500	0.83048	0.83645	0.84306
420	0.80769	0.81859	0.82362	0.82530	0.83078	0.83674	0.84333
421	0.80803	0.81890	0.82393	0.82561	0.83108	0.83702	0.84360
422	0.80839	0.81921	0.82424	0.82592	0.83138	0.83731	0.84387
423	0.80869	0.81954	0.82455	0.82623	0.83167	0.83759	0.84414
424	0.80905	0.81986	0.82487	0.82652	0.83196	0.83787	0.84441
425	0.80941	0.82019	0.82517	0.82683	0.83226	0.83815	0.84468
426	0.80976	0.82050	0.82548	0.82714	0.83255	0.83843	0.84495
427	0.81009	0.82081	0.82578	0.82744	0.83284	0.83872	0.84522
428	0.81042	0.82111	0.82609	0.82775	0.83313	0.83900	0.84548
429	0.81075	0.82143	0.82640	0.82804	0.83342	0.83927	0.84575
430	0.81109	0.82174	0.82670	0.82835	0.83371	0.83954	0.84601
431	0.81141	0.82205	0.82700	0.82864	0.83399	0.83983	0.84627
432	0.81176	0.82236	0.82730	0.82894	0.83428	0.84009	0.84653
433	0.81210	0.82267	0.82759	0.82922	0.83456	0.84036	0.84679
434	0.81245	0.82298	0.82788	0.82952	0.83485	0.84064	0.84705
435	0.81279	0.82329	0.82818	0.82981	0.83512	0.84091	0.84731
436	0.81309	0.82358	0.82847	0.83011	0.83541	0.84117	0.84757
437	0.81342	0.82388	0.82877	0.83039	0.83569	0.84144	0.84783
438	0.81375	0.82419	0.82905	0.83069	0.83597	0.84171	0.84808
439	0.81405	0.82449	0.82935	0.83097	0.83624	0.84198	0.84834
440	0.81437	0.82480	0.82963	0.83125	0.83652	0.84225	0.84859
441	0.81467	0.82508	0.82992	0.83153	0.83679	0.84251	0.84884
442	0.81496	0.82538	0.83021	0.83182	0.83707	0.84277	0.84909
443	0.81526	0.82567	0.83049	0.83211	0.83735	0.84304	0.84934
444	0.81557	0.82594	0.83078	0.83239	0.83762	0.84330	0.84959
445	0.81590	0.82625	0.83107	0.83267	0.83789	0.84355	0.84984
446	0.81620	0.82654	0.83135	0.83295	0.83815	0.84381	0.85009
447	0.81654	0.82683	0.83164	0.83323	0.83842	0.84407	0.85034

N	0.1%	0.5%	1%	1.25%	2.5%	5%	10%
448	0.81686	0.82713	0.83193	0.83351	0.83868	0.84433	0.85058
449	0.81712	0.82742	0.83220	0.83379	0.83895	0.84459	0.85082
450	0.81745	0.82769	0.83247	0.83407	0.83922	0.84484	0.85107
451	0.81775	0.82797	0.83275	0.83434	0.83949	0.84509	0.85131
452	0.81807	0.82825	0.83304	0.83461	0.83975	0.84535	0.85155
453	0.81838	0.82853	0.83332	0.83488	0.84002	0.84560	0.85180
454	0.81867	0.82882	0.83359	0.83516	0.84028	0.84586	0.85204
455	0.81895	0.82909	0.83387	0.83543	0.84055	0.84610	0.85228
456	0.81926	0.82939	0.83414	0.83570	0.84081	0.84636	0.85252
457	0.81956	0.82967	0.83441	0.83597	0.84107	0.84660	0.85275
458	0.81985	0.82995	0.83469	0.83624	0.84133	0.84686	0.85299
459	0.82012	0.83022	0.83495	0.83651	0.84158	0.84710	0.85323
460	0.82044	0.83050	0.83522	0.83677	0.84185	0.84735	0.85346
461	0.82074	0.83077	0.83549	0.83704	0.84211	0.84760	0.85370
462	0.82104	0.83107	0.83576	0.83730	0.84236	0.84784	0.85394
463	0.82134	0.83134	0.83602	0.83756	0.84261	0.84809	0.85417
464	0.82163	0.83161	0.83630	0.83782	0.84286	0.84833	0.85440
465	0.82191	0.83189	0.83655	0.83809	0.84312	0.84857	0.85463
466	0.82219	0.83217	0.83681	0.83835	0.84336	0.84882	0.85486
467	0.82247	0.83243	0.83708	0.83861	0.84362	0.84906	0.85509
468	0.82273	0.83270	0.83735	0.83888	0.84387	0.84929	0.85532
469	0.82300	0.83297	0.83761	0.83914	0.84412	0.84954	0.85555
470	0.82328	0.83324	0.83787	0.83940	0.84437	0.84977	0.85578
471	0.82359	0.83351	0.83813	0.83965	0.84461	0.85001	0.85600
472	0.82389	0.83378	0.83838	0.83991	0.84486	0.85025	0.85623
473	0.82416	0.83405	0.83864	0.84016	0.84511	0.85048	0.85645
474	0.82445	0.83431	0.83889	0.84041	0.84535	0.85071	0.85668
475	0.82472	0.83456	0.83915	0.84067	0.84559	0.85095	0.85690
476	0.82499	0.83483	0.83941	0.84092	0.84583	0.85118	0.85712
477	0.82528	0.83509	0.83966	0.84117	0.84608	0.85141	0.85735
478	0.82557	0.83536	0.83992	0.84142	0.84632	0.85165	0.85757
479	0.82584	0.83562	0.84016	0.84167	0.84656	0.85188	0.85779
480	0.82611	0.83588	0.84041	0.84192	0.84680	0.85211	0.85801
481	0.82640	0.83614	0.84066	0.84217	0.84704	0.85233	0.85822
482	0.82667	0.83640	0.84091	0.84241	0.84728	0.85256	0.85844
483	0.82696	0.83665	0.84116	0.84266	0.84751	0.85279	0.85866
484	0.82722	0.83691	0.84140	0.84290	0.84775	0.85302	0.85887
485	0.82749	0.83717	0.84165	0.84314	0.84798	0.85325	0.85909
486	0.82777	0.83741	0.84188	0.84338	0.84822	0.85347	0.85930
487	0.82802	0.83766	0.84213	0.84362	0.84846	0.85370	0.85952
488	0.82832	0.83791	0.84237	0.84386	0.84869	0.85392	0.85973
489	0.82859	0.83816	0.84262	0.84410	0.84891	0.85415	0.85994
490	0.82885	0.83841	0.84286	0.84434	0.84915	0.85436	0.86015
491	0.82910	0.83865	0.84311	0.84458	0.84938	0.85459	0.86037
492	0.82939	0.83892	0.84334	0.84481	0.84961	0.85481	0.86057
493	0.82965	0.83915	0.84358	0.84505	0.84983	0.85503	0.86079
494	0.82990	0.83940	0.84382	0.84529	0.85006	0.85525	0.86099
495	0.83015	0.83964	0.84406	0.84552	0.85029	0.85546	0.86120
496	0.83041	0.83988	0.84429	0.84576	0.85052	0.85568	0.86141
497	0.83070	0.84013	0.84453	0.84600	0.85074	0.85590	0.86162
498	0.83095	0.84037	0.84477	0.84624	0.85096	0.85611	0.86182
499	0.83120	0.84062	0.84500	0.84646	0.85119	0.85633	0.86203
500	0.83146	0.84087	0.84523	0.84670	0.85141	0.85654	0.86224
501	0.83173	0.84111	0.84547	0.84693	0.85163	0.85676	0.86244
502	0.83197	0.84133	0.84569	0.84717	0.85186	0.85697	0.86264
503	0.83224	0.84158	0.84593	0.84739	0.85208	0.85719	0.86285
504	0.83250	0.84183	0.84616	0.84762	0.85231	0.85740	0.86305
505	0.83274	0.84207	0.84640	0.84784	0.85252	0.85761	0.86325
506	0.83299	0.84230	0.84663	0.84807	0.85274	0.85782	0.86345
507	0.83324	0.84254	0.84685	0.84830	0.85296	0.85803	0.86365
508	0.83350	0.84278	0.84708	0.84852	0.85318	0.85824	0.86385
509	0.83376	0.84300	0.84731	0.84875	0.85340	0.85845	0.86405
510	0.83400	0.84323	0.84754	0.84897	0.85361	0.85865	0.86425
511	0.83426	0.84347	0.84776	0.84919	0.85383	0.85886	0.86444
512	0.83449	0.84370	0.84798	0.84942	0.85405	0.85906	0.86464
513	0.83474	0.84393	0.84821	0.84963	0.85427	0.85927	0.86484
514	0.83501	0.84417	0.84843	0.84986	0.85448	0.85948	0.86503
515	0.83524	0.84440	0.84865	0.85008	0.85469	0.85968	0.86523
516	0.83549	0.84463	0.84888	0.85029	0.85490	0.85989	0.86542
517	0.83573	0.84486	0.84910	0.85051	0.85512	0.86009	0.86562
518	0.83597	0.84508	0.84931	0.85073	0.85533	0.86029	0.86581
519	0.83620	0.84530	0.84953	0.85095	0.85554	0.86049	0.86600
520	0.83643	0.84553	0.84975	0.85116	0.85575	0.86069	0.86619
521	0.83667	0.84575	0.84996	0.85138	0.85596	0.86089	0.86638

N	0.1%	0.5%	1%	1.25%	2.5%	5%	10%
522	0.83692	0.84598	0.85019	0.85160	0.85617	0.86109	0.86658
523	0.83715	0.84620	0.85040	0.85181	0.85637	0.86129	0.86677
524	0.83739	0.84642	0.85061	0.85203	0.85658	0.86149	0.86696
525	0.83763	0.84665	0.85082	0.85224	0.85678	0.86168	0.86715
526	0.83787	0.84687	0.85104	0.85245	0.85699	0.86188	0.86734
527	0.83809	0.84708	0.85125	0.85266	0.85720	0.86208	0.86753
528	0.83833	0.84731	0.85147	0.85287	0.85740	0.86227	0.86771
529	0.83856	0.84751	0.85168	0.85308	0.85760	0.86247	0.86790
530	0.83880	0.84773	0.85188	0.85329	0.85780	0.86267	0.86808
531	0.83904	0.84795	0.85209	0.85350	0.85800	0.86286	0.86827
532	0.83926	0.84817	0.85231	0.85371	0.85821	0.86306	0.86846
533	0.83951	0.84839	0.85252	0.85391	0.85840	0.86325	0.86864
534	0.83974	0.84861	0.85273	0.85411	0.85860	0.86344	0.86882
535	0.83999	0.84882	0.85294	0.85433	0.85880	0.86363	0.86901
536	0.84022	0.84904	0.85314	0.85453	0.85900	0.86383	0.86919
537	0.84045	0.84925	0.85336	0.85474	0.85920	0.86402	0.86937
538	0.84068	0.84947	0.85356	0.85494	0.85940	0.86421	0.86956
539	0.84090	0.84968	0.85377	0.85515	0.85960	0.86440	0.86974
540	0.84112	0.84989	0.85397	0.85535	0.85979	0.86459	0.86992
541	0.84135	0.85010	0.85417	0.85555	0.85999	0.86478	0.87010
542	0.84158	0.85032	0.85438	0.85575	0.86018	0.86497	0.87027
543	0.84180	0.85052	0.85459	0.85595	0.86037	0.86515	0.87045
544	0.84201	0.85074	0.85479	0.85615	0.86057	0.86534	0.87063
545	0.84224	0.85094	0.85500	0.85635	0.86076	0.86553	0.87081
546	0.84246	0.85115	0.85519	0.85655	0.86096	0.86571	0.87098
547	0.84267	0.85136	0.85539	0.85675	0.86115	0.86590	0.87116
548	0.84291	0.85157	0.85559	0.85695	0.86133	0.86608	0.87133
549	0.84312	0.85177	0.85579	0.85714	0.86153	0.86627	0.87151
550	0.84337	0.85198	0.85599	0.85734	0.86172	0.86645	0.87168
551	0.84359	0.85218	0.85619	0.85754	0.86191	0.86663	0.87186
552	0.84381	0.85240	0.85640	0.85773	0.86209	0.86681	0.87203
553	0.84401	0.85260	0.85659	0.85793	0.86228	0.86699	0.87220
554	0.84425	0.85280	0.85678	0.85812	0.86247	0.86717	0.87237
555	0.84447	0.85300	0.85698	0.85832	0.86266	0.86735	0.87255
556	0.84469	0.85321	0.85718	0.85851	0.86284	0.86753	0.87272
557	0.84489	0.85342	0.85738	0.85870	0.86303	0.86771	0.87289
558	0.84512	0.85361	0.85757	0.85890	0.86321	0.86789	0.87306
559	0.84532	0.85382	0.85776	0.85909	0.86339	0.86807	0.87323
560	0.84552	0.85402	0.85795	0.85928	0.86358	0.86825	0.87340
561	0.84574	0.85421	0.85814	0.85947	0.86376	0.86843	0.87357
562	0.84597	0.85441	0.85834	0.85966	0.86395	0.86860	0.87374
563	0.84617	0.85461	0.85852	0.85985	0.86413	0.86878	0.87391
564	0.84640	0.85481	0.85872	0.86004	0.86431	0.86896	0.87407
565	0.84660	0.85500	0.85891	0.86022	0.86449	0.86913	0.87424
566	0.84682	0.85520	0.85910	0.86042	0.86467	0.86930	0.87441
567	0.84702	0.85540	0.85929	0.86061	0.86486	0.86948	0.87457
568	0.84722	0.85559	0.85949	0.86080	0.86504	0.86965	0.87474
569	0.84743	0.85578	0.85967	0.86098	0.86522	0.86982	0.87490
570	0.84767	0.85597	0.85986	0.86116	0.86539	0.86999	0.87507
571	0.84784	0.85617	0.86005	0.86135	0.86557	0.87016	0.87523
572	0.84805	0.85637	0.86024	0.86154	0.86575	0.87034	0.87540
573	0.84824	0.85655	0.86043	0.86172	0.86593	0.87051	0.87556
574	0.84843	0.85674	0.86061	0.86191	0.86611	0.87068	0.87572
575	0.84865	0.85693	0.86080	0.86209	0.86628	0.87085	0.87588
576	0.84884	0.85712	0.86098	0.86227	0.86646	0.87102	0.87605
577	0.84905	0.85731	0.86116	0.86245	0.86664	0.87119	0.87621
578	0.84925	0.85749	0.86135	0.86263	0.86681	0.87136	0.87637
579	0.84945	0.85768	0.86153	0.86281	0.86699	0.87153	0.87653
580	0.84966	0.85786	0.86171	0.86299	0.86716	0.87170	0.87669
581	0.84986	0.85805	0.86190	0.86317	0.86733	0.87186	0.87685
582	0.85008	0.85825	0.86207	0.86335	0.86750	0.87203	0.87701
583	0.85027	0.85844	0.86226	0.86353	0.86768	0.87220	0.87717
584	0.85045	0.85863	0.86244	0.86371	0.86785	0.87236	0.87733
585	0.85066	0.85882	0.86262	0.86389	0.86802	0.87252	0.87748
586	0.85084	0.85901	0.86280	0.86406	0.86819	0.87269	0.87764
587	0.85102	0.85919	0.86297	0.86424	0.86836	0.87285	0.87779
588	0.85122	0.85937	0.86315	0.86442	0.86853	0.87301	0.87795
589	0.85141	0.85956	0.86333	0.86459	0.86870	0.87318	0.87811
590	0.85162	0.85974	0.86351	0.86477	0.86887	0.87334	0.87826
591	0.85180	0.85991	0.86369	0.86494	0.86904	0.87350	0.87841
592	0.85198	0.86010	0.86386	0.86512	0.86921	0.87367	0.87857
593	0.85220	0.86028	0.86404	0.86529	0.86938	0.87383	0.87872
594	0.85239	0.86045	0.86421	0.86547	0.86955	0.87399	0.87888
595	0.85258	0.86063	0.86439	0.86564	0.86972	0.87415	0.87903

N	0.1%	0.5%	1%	1.25%	2.5%	5%	10%
596	0.85278	0.86081	0.86457	0.86582	0.86988	0.87431	0.87918
597	0.85297	0.86100	0.86474	0.86599	0.87005	0.87447	0.87934
598	0.85316	0.86118	0.86491	0.86616	0.87021	0.87463	0.87949
599	0.85335	0.86135	0.86508	0.86633	0.87038	0.87478	0.87964
600	0.85351	0.86154	0.86526	0.86650	0.87054	0.87494	0.87979
601	0.85372	0.86171	0.86544	0.86667	0.87071	0.87510	0.87994
602	0.85389	0.86189	0.86560	0.86684	0.87087	0.87526	0.88009
603	0.85407	0.86206	0.86578	0.86702	0.87103	0.87541	0.88024
604	0.85425	0.86224	0.86595	0.86718	0.87119	0.87557	0.88039
605	0.85443	0.86241	0.86612	0.86735	0.87135	0.87573	0.88054
606	0.85463	0.86258	0.86629	0.86752	0.87151	0.87588	0.88069
607	0.85481	0.86275	0.86645	0.86768	0.87168	0.87604	0.88084
608	0.85500	0.86293	0.86662	0.86785	0.87184	0.87619	0.88099
609	0.85517	0.86310	0.86678	0.86802	0.87200	0.87634	0.88113
610	0.85535	0.86327	0.86695	0.86818	0.87216	0.87650	0.88128
611	0.85551	0.86345	0.86712	0.86834	0.87232	0.87665	0.88143
612	0.85572	0.86362	0.86728	0.86851	0.87248	0.87680	0.88157
613	0.85591	0.86380	0.86746	0.86867	0.87264	0.87696	0.88172
614	0.85610	0.86396	0.86762	0.86884	0.87280	0.87711	0.88186
615	0.85626	0.86413	0.86778	0.86900	0.87296	0.87726	0.88201
616	0.85644	0.86430	0.86794	0.86916	0.87311	0.87741	0.88216
617	0.85663	0.86447	0.86811	0.86932	0.87326	0.87756	0.88230
618	0.85682	0.86465	0.86828	0.86948	0.87342	0.87771	0.88244
619	0.85701	0.86482	0.86844	0.86965	0.87358	0.87786	0.88259
620	0.85717	0.86499	0.86861	0.86980	0.87374	0.87801	0.88273
621	0.85735	0.86515	0.86877	0.86997	0.87389	0.87816	0.88287
622	0.85754	0.86532	0.86893	0.87013	0.87405	0.87831	0.88301
623	0.85773	0.86548	0.86909	0.87029	0.87420	0.87846	0.88315
624	0.85790	0.86564	0.86925	0.87045	0.87435	0.87861	0.88330
625	0.85809	0.86581	0.86941	0.87061	0.87451	0.87875	0.88344
626	0.85826	0.86598	0.86957	0.87077	0.87466	0.87890	0.88358
627	0.85844	0.86614	0.86973	0.87093	0.87481	0.87905	0.88372
628	0.85861	0.86631	0.86989	0.87108	0.87497	0.87919	0.88386
629	0.85879	0.86647	0.87006	0.87124	0.87512	0.87934	0.88400
630	0.85895	0.86664	0.87021	0.87140	0.87527	0.87948	0.88413
631	0.85913	0.86679	0.87036	0.87155	0.87542	0.87963	0.88427
632	0.85931	0.86696	0.87052	0.87172	0.87557	0.87978	0.88441
633	0.85948	0.86712	0.87067	0.87187	0.87572	0.87992	0.88455
634	0.85964	0.86728	0.87083	0.87202	0.87587	0.88006	0.88469
635	0.85983	0.86744	0.87098	0.87217	0.87602	0.88021	0.88483
636	0.86001	0.86759	0.87114	0.87233	0.87617	0.88035	0.88496
637	0.86017	0.86776	0.87129	0.87248	0.87632	0.88049	0.88510
638	0.86032	0.86792	0.87145	0.87264	0.87647	0.88064	0.88523
639	0.86050	0.86808	0.87161	0.87280	0.87661	0.88078	0.88537
640	0.86067	0.86824	0.87176	0.87295	0.87676	0.88092	0.88551
641	0.86083	0.86840	0.87191	0.87310	0.87691	0.88106	0.88564
642	0.86100	0.86856	0.87206	0.87325	0.87706	0.88120	0.88577
643	0.86116	0.86872	0.87222	0.87340	0.87720	0.88134	0.88591
644	0.86130	0.86887	0.87236	0.87355	0.87735	0.88148	0.88605
645	0.86146	0.86902	0.87252	0.87370	0.87749	0.88162	0.88618
646	0.86163	0.86918	0.87267	0.87385	0.87763	0.88176	0.88631
647	0.86180	0.86934	0.87281	0.87399	0.87778	0.88190	0.88644
648	0.86196	0.86949	0.87297	0.87414	0.87792	0.88203	0.88658
649	0.86214	0.86965	0.87312	0.87429	0.87807	0.88217	0.88671
650	0.86229	0.86980	0.87327	0.87444	0.87822	0.88231	0.88684
651	0.86246	0.86996	0.87342	0.87459	0.87836	0.88245	0.88697
652	0.86263	0.87011	0.87358	0.87474	0.87850	0.88259	0.88710
653	0.86279	0.87026	0.87372	0.87488	0.87864	0.88272	0.88723
654	0.86296	0.87042	0.87387	0.87503	0.87878	0.88286	0.88737
655	0.86312	0.87058	0.87402	0.87518	0.87893	0.88300	0.88749
656	0.86328	0.87073	0.87417	0.87533	0.87907	0.88313	0.88762
657	0.86345	0.87088	0.87432	0.87547	0.87921	0.88327	0.88775
658	0.86360	0.87103	0.87446	0.87562	0.87935	0.88341	0.88788
659	0.86376	0.87118	0.87461	0.87576	0.87949	0.88354	0.88801
660	0.86392	0.87133	0.87476	0.87591	0.87964	0.88367	0.88814
661	0.86407	0.87149	0.87490	0.87605	0.87978	0.88381	0.88827
662	0.86424	0.87164	0.87504	0.87620	0.87991	0.88394	0.88840
663	0.86440	0.87179	0.87519	0.87634	0.88005	0.88407	0.88852
664	0.86456	0.87194	0.87534	0.87648	0.88019	0.88421	0.88865
665	0.86472	0.87209	0.87549	0.87663	0.88033	0.88434	0.88878
666	0.86489	0.87223	0.87563	0.87677	0.88047	0.88447	0.88890
667	0.86506	0.87238	0.87577	0.87691	0.88061	0.88460	0.88903
668	0.86521	0.87253	0.87592	0.87705	0.88074	0.88474	0.88916
669	0.86539	0.87268	0.87606	0.87719	0.88088	0.88487	0.88928

N	0.1%	0.5%	1%	1.25%	2.5%	5%	10%
670	0.86555	0.87283	0.87620	0.87733	0.88101	0.88500	0.88941
671	0.86571	0.87298	0.87635	0.87748	0.88115	0.88513	0.88953
672	0.86584	0.87312	0.87649	0.87762	0.88129	0.88526	0.88965
673	0.86598	0.87328	0.87663	0.87776	0.88142	0.88539	0.88978
674	0.86614	0.87343	0.87678	0.87790	0.88156	0.88552	0.88990
675	0.86629	0.87357	0.87692	0.87804	0.88170	0.88565	0.89003
676	0.86645	0.87371	0.87706	0.87818	0.88183	0.88578	0.89015
677	0.86660	0.87385	0.87719	0.87832	0.88196	0.88591	0.89028
678	0.86675	0.87400	0.87734	0.87846	0.88209	0.88604	0.89040
679	0.86692	0.87415	0.87747	0.87860	0.88223	0.88617	0.89052
680	0.86705	0.87429	0.87761	0.87874	0.88236	0.88629	0.89064
681	0.86721	0.87443	0.87775	0.87887	0.88249	0.88642	0.89077
682	0.86735	0.87457	0.87789	0.87901	0.88262	0.88654	0.89089
683	0.86752	0.87470	0.87803	0.87915	0.88276	0.88667	0.89101
684	0.86767	0.87485	0.87816	0.87928	0.88289	0.88680	0.89113
685	0.86782	0.87499	0.87830	0.87942	0.88302	0.88692	0.89125
686	0.86797	0.87513	0.87844	0.87955	0.88315	0.88705	0.89137
687	0.86813	0.87528	0.87857	0.87969	0.88328	0.88717	0.89149
688	0.86827	0.87541	0.87871	0.87983	0.88341	0.88730	0.89161
689	0.86844	0.87555	0.87885	0.87996	0.88354	0.88742	0.89173
690	0.86859	0.87569	0.87898	0.88009	0.88367	0.88755	0.89185
691	0.86874	0.87583	0.87912	0.88022	0.88380	0.88768	0.89197
692	0.86888	0.87597	0.87926	0.88036	0.88393	0.88780	0.89209
693	0.86903	0.87611	0.87939	0.88049	0.88406	0.88793	0.89221
694	0.86917	0.87625	0.87953	0.88063	0.88419	0.88805	0.89233
695	0.86932	0.87638	0.87967	0.88076	0.88432	0.88817	0.89244
696	0.86946	0.87652	0.87980	0.88090	0.88445	0.88829	0.89256
697	0.86960	0.87666	0.87994	0.88102	0.88457	0.88842	0.89268
698	0.86975	0.87679	0.88006	0.88115	0.88470	0.88854	0.89279
699	0.86990	0.87693	0.88020	0.88129	0.88483	0.88866	0.89291
700	0.87004	0.87708	0.88034	0.88142	0.88495	0.88878	0.89303
701	0.87018	0.87721	0.88047	0.88155	0.88508	0.88890	0.89314
702	0.87033	0.87735	0.88060	0.88168	0.88521	0.88903	0.89326
703	0.87047	0.87749	0.88073	0.88181	0.88533	0.88915	0.89337
704	0.87061	0.87763	0.88087	0.88194	0.88546	0.88926	0.89349
705	0.87075	0.87777	0.88100	0.88207	0.88558	0.88939	0.89360
706	0.87090	0.87790	0.88113	0.88220	0.88571	0.88951	0.89372
707	0.87104	0.87804	0.88126	0.88233	0.88584	0.88963	0.89383
708	0.87120	0.87817	0.88139	0.88246	0.88596	0.88975	0.89394
709	0.87134	0.87830	0.88152	0.88259	0.88608	0.88986	0.89406
710	0.87148	0.87844	0.88164	0.88272	0.88621	0.88998	0.89417
711	0.87161	0.87856	0.88178	0.88284	0.88633	0.89010	0.89428
712	0.87175	0.87870	0.88191	0.88297	0.88645	0.89022	0.89440
713	0.87189	0.87883	0.88203	0.88310	0.88657	0.89034	0.89451
714	0.87203	0.87896	0.88216	0.88322	0.88669	0.89045	0.89462
715	0.87218	0.87910	0.88229	0.88335	0.88682	0.89057	0.89473
716	0.87232	0.87922	0.88242	0.88348	0.88694	0.89069	0.89485
717	0.87247	0.87936	0.88255	0.88361	0.88706	0.89081	0.89496
718	0.87260	0.87949	0.88267	0.88373	0.88718	0.89092	0.89507
719	0.87273	0.87963	0.88280	0.88386	0.88730	0.89104	0.89518
720	0.87288	0.87975	0.88292	0.88398	0.88742	0.89116	0.89530
721	0.87302	0.87988	0.88305	0.88410	0.88754	0.89127	0.89541
722	0.87315	0.88001	0.88317	0.88423	0.88766	0.89139	0.89552
723	0.87329	0.88014	0.88330	0.88435	0.88778	0.89150	0.89563
724	0.87343	0.88028	0.88342	0.88447	0.88790	0.89162	0.89574
725	0.87357	0.88041	0.88355	0.88459	0.88802	0.89173	0.89584
726	0.87370	0.88054	0.88367	0.88472	0.88813	0.89185	0.89596
727	0.87384	0.88067	0.88380	0.88485	0.88825	0.89196	0.89606
728	0.87398	0.88079	0.88392	0.88497	0.88837	0.89207	0.89617
729	0.87412	0.88092	0.88404	0.88509	0.88849	0.89219	0.89628
730	0.87424	0.88105	0.88417	0.88522	0.88861	0.89230	0.89639
731	0.87437	0.88116	0.88429	0.88534	0.88873	0.89241	0.89650
732	0.87451	0.88131	0.88441	0.88546	0.88885	0.89253	0.89661
733	0.87465	0.88143	0.88454	0.88558	0.88896	0.89264	0.89671
734	0.87478	0.88155	0.88466	0.88570	0.88908	0.89275	0.89682
735	0.87491	0.88168	0.88477	0.88582	0.88919	0.89286	0.89693
736	0.87504	0.88180	0.88489	0.88594	0.88931	0.89298	0.89704
737	0.87518	0.88193	0.88502	0.88606	0.88943	0.89309	0.89714
738	0.87531	0.88206	0.88514	0.88618	0.88954	0.89320	0.89725
739	0.87544	0.88218	0.88526	0.88630	0.88966	0.89331	0.89736
740	0.87557	0.88230	0.88539	0.88642	0.88977	0.89342	0.89746
741	0.87569	0.88243	0.88550	0.88654	0.88989	0.89353	0.89757
742	0.87582	0.88255	0.88563	0.88665	0.89000	0.89364	0.89767
743	0.87595	0.88268	0.88574	0.88677	0.89012	0.89375	0.89778

N	0.1%	0.5%	1%	1.25%	2.5%	5%	10%
744	0.87607	0.88279	0.88586	0.88689	0.89023	0.89386	0.89788
745	0.87620	0.88291	0.88598	0.88701	0.89035	0.89397	0.89798
746	0.87633	0.88305	0.88610	0.88712	0.89046	0.89408	0.89809
747	0.87647	0.88317	0.88622	0.88724	0.89057	0.89419	0.89819
748	0.87659	0.88329	0.88635	0.88735	0.89069	0.89430	0.89830
749	0.87672	0.88342	0.88646	0.88747	0.89080	0.89441	0.89840
750	0.87684	0.88354	0.88658	0.88759	0.89091	0.89451	0.89850
751	0.87698	0.88365	0.88670	0.88770	0.89102	0.89462	0.89861
752	0.87711	0.88377	0.88681	0.88782	0.89113	0.89473	0.89871
753	0.87725	0.88389	0.88693	0.88794	0.89125	0.89484	0.89881
754	0.87740	0.88401	0.88705	0.88805	0.89136	0.89494	0.89891
755	0.87752	0.88413	0.88716	0.88817	0.89147	0.89505	0.89902
756	0.87763	0.88426	0.88728	0.88829	0.89158	0.89516	0.89912
757	0.87776	0.88437	0.88739	0.88840	0.89169	0.89526	0.89922
758	0.87789	0.88449	0.88751	0.88852	0.89180	0.89537	0.89933
759	0.87802	0.88460	0.88763	0.88863	0.89191	0.89548	0.89943
760	0.87815	0.88472	0.88774	0.88875	0.89202	0.89558	0.89953
761	0.87829	0.88483	0.88786	0.88886	0.89213	0.89569	0.89963
762	0.87842	0.88496	0.88797	0.88898	0.89224	0.89579	0.89973
763	0.87853	0.88507	0.88808	0.88909	0.89235	0.89590	0.89983
764	0.87866	0.88519	0.88819	0.88920	0.89246	0.89600	0.89993
765	0.87877	0.88530	0.88831	0.88931	0.89257	0.89611	0.90003
766	0.87891	0.88542	0.88842	0.88942	0.89268	0.89621	0.90013
767	0.87902	0.88552	0.88853	0.88953	0.89278	0.89632	0.90023
768	0.87915	0.88564	0.88864	0.88964	0.89289	0.89642	0.90033
769	0.87926	0.88576	0.88875	0.88976	0.89300	0.89653	0.90043
770	0.87939	0.88586	0.88887	0.88987	0.89311	0.89663	0.90053
771	0.87952	0.88598	0.88898	0.88998	0.89322	0.89673	0.90063
772	0.87963	0.88610	0.88909	0.89009	0.89332	0.89684	0.90073
773	0.87976	0.88621	0.88921	0.89020	0.89343	0.89694	0.90082
774	0.87988	0.88633	0.88932	0.89031	0.89354	0.89704	0.90092
775	0.87999	0.88645	0.88943	0.89042	0.89364	0.89714	0.90102
776	0.88012	0.88656	0.88954	0.89053	0.89375	0.89725	0.90112
777	0.88024	0.88667	0.88965	0.89064	0.89385	0.89735	0.90122
778	0.88036	0.88679	0.88977	0.89075	0.89396	0.89745	0.90131
779	0.88049	0.88691	0.88988	0.89086	0.89406	0.89755	0.90141
780	0.88062	0.88702	0.88999	0.89096	0.89417	0.89765	0.90151
781	0.88073	0.88713	0.89009	0.89107	0.89428	0.89775	0.90160
782	0.88086	0.88724	0.89021	0.89118	0.89438	0.89786	0.90170
783	0.88098	0.88735	0.89031	0.89129	0.89449	0.89796	0.90180
784	0.88110	0.88746	0.89042	0.89140	0.89459	0.89806	0.90189
785	0.88122	0.88757	0.89053	0.89151	0.89469	0.89816	0.90199
786	0.88136	0.88769	0.89064	0.89161	0.89480	0.89826	0.90208
787	0.88147	0.88779	0.89074	0.89172	0.89490	0.89836	0.90218
788	0.88159	0.88790	0.89085	0.89182	0.89501	0.89846	0.90227
789	0.88170	0.88801	0.89096	0.89193	0.89511	0.89855	0.90237
790	0.88182	0.88813	0.89107	0.89204	0.89521	0.89865	0.90246
791	0.88194	0.88825	0.89117	0.89214	0.89531	0.89875	0.90256
792	0.88207	0.88836	0.89128	0.89225	0.89542	0.89885	0.90265
793	0.88218	0.88847	0.89139	0.89236	0.89552	0.89895	0.90275
794	0.88231	0.88858	0.89150	0.89246	0.89562	0.89905	0.90284
795	0.88242	0.88869	0.89160	0.89257	0.89572	0.89915	0.90293
796	0.88253	0.88880	0.89171	0.89268	0.89582	0.89924	0.90303
797	0.88263	0.88891	0.89181	0.89278	0.89592	0.89934	0.90312
798	0.88274	0.88902	0.89192	0.89289	0.89603	0.89944	0.90321
799	0.88285	0.88913	0.89203	0.89299	0.89613	0.89954	0.90331
800	0.88297	0.88924	0.89213	0.89309	0.89623	0.89963	0.90340
801	0.88308	0.88934	0.89223	0.89319	0.89633	0.89973	0.90349
802	0.88320	0.88945	0.89234	0.89330	0.89643	0.89982	0.90358
803	0.88331	0.88955	0.89244	0.89340	0.89653	0.89992	0.90367
804	0.88342	0.88966	0.89255	0.89351	0.89662	0.90002	0.90376
805	0.88355	0.88977	0.89265	0.89361	0.89672	0.90011	0.90386
806	0.88366	0.88988	0.89276	0.89372	0.89683	0.90021	0.90395
807	0.88378	0.88998	0.89286	0.89382	0.89692	0.90030	0.90404
808	0.88388	0.89009	0.89296	0.89392	0.89702	0.90040	0.90413
809	0.88400	0.89019	0.89307	0.89402	0.89712	0.90049	0.90422
810	0.88412	0.89030	0.89317	0.89412	0.89722	0.90059	0.90431
811	0.88423	0.89041	0.89327	0.89423	0.89732	0.90068	0.90441
812	0.88435	0.89052	0.89337	0.89433	0.89742	0.90078	0.90450
813	0.88447	0.89062	0.89347	0.89443	0.89752	0.90087	0.90459
814	0.88459	0.89073	0.89358	0.89453	0.89762	0.90097	0.90468
815	0.88469	0.89084	0.89368	0.89463	0.89772	0.90106	0.90477
816	0.88482	0.89094	0.89378	0.89473	0.89781	0.90115	0.90485
817	0.88492	0.89104	0.89388	0.89483	0.89791	0.90124	0.90494

N	0.1%	0.5%	1%	1.25%	2.5%	5%	10%
818	0.88503	0.89115	0.89398	0.89493	0.89801	0.90134	0.90503
819	0.88515	0.89125	0.89408	0.89503	0.89810	0.90143	0.90512
820	0.88526	0.89135	0.89418	0.89513	0.89820	0.90152	0.90521
821	0.88537	0.89146	0.89428	0.89523	0.89829	0.90161	0.90530
822	0.88549	0.89156	0.89438	0.89533	0.89839	0.90171	0.90539
823	0.88559	0.89166	0.89448	0.89542	0.89848	0.90180	0.90547
824	0.88570	0.89176	0.89458	0.89552	0.89858	0.90189	0.90556
825	0.88580	0.89187	0.89468	0.89562	0.89868	0.90199	0.90565
826	0.88591	0.89197	0.89478	0.89572	0.89877	0.90208	0.90574
827	0.88602	0.89207	0.89488	0.89582	0.89887	0.90217	0.90583
828	0.88613	0.89217	0.89498	0.89592	0.89896	0.90226	0.90592
829	0.88623	0.89228	0.89508	0.89602	0.89906	0.90235	0.90600
830	0.88634	0.89238	0.89518	0.89611	0.89915	0.90244	0.90609
831	0.88646	0.89248	0.89528	0.89621	0.89924	0.90253	0.90618
832	0.88657	0.89259	0.89538	0.89631	0.89934	0.90262	0.90626
833	0.88668	0.89269	0.89547	0.89640	0.89943	0.90271	0.90635
834	0.88679	0.89279	0.89557	0.89650	0.89952	0.90280	0.90644
835	0.88689	0.89289	0.89567	0.89660	0.89962	0.90289	0.90652
836	0.88701	0.89299	0.89577	0.89669	0.89971	0.90298	0.90661
837	0.88711	0.89309	0.89586	0.89679	0.89980	0.90307	0.90670
838	0.88722	0.89319	0.89596	0.89689	0.89990	0.90316	0.90678
839	0.88732	0.89328	0.89606	0.89699	0.89999	0.90325	0.90687
840	0.88742	0.89338	0.89616	0.89709	0.90008	0.90334	0.90695
841	0.88751	0.89349	0.89625	0.89718	0.90017	0.90343	0.90704
842	0.88763	0.89358	0.89635	0.89727	0.90026	0.90352	0.90712
843	0.88775	0.89368	0.89644	0.89737	0.90036	0.90361	0.90721
844	0.88785	0.89378	0.89654	0.89747	0.90045	0.90369	0.90729
845	0.88795	0.89388	0.89664	0.89756	0.90054	0.90378	0.90738
846	0.88806	0.89398	0.89673	0.89765	0.90063	0.90387	0.90746
847	0.88816	0.89407	0.89683	0.89775	0.90072	0.90396	0.90754
848	0.88826	0.89417	0.89692	0.89784	0.90082	0.90405	0.90763
849	0.88835	0.89427	0.89702	0.89793	0.90091	0.90413	0.90771
850	0.88846	0.89437	0.89711	0.89803	0.90100	0.90422	0.90779
851	0.88857	0.89446	0.89721	0.89812	0.90109	0.90431	0.90788
852	0.88868	0.89456	0.89730	0.89821	0.90118	0.90439	0.90796
853	0.88878	0.89466	0.89740	0.89831	0.90127	0.90448	0.90804
854	0.88888	0.89476	0.89749	0.89840	0.90136	0.90457	0.90813
855	0.88899	0.89485	0.89759	0.89849	0.90145	0.90466	0.90821
856	0.88909	0.89495	0.89768	0.89859	0.90154	0.90474	0.90829
857	0.88919	0.89505	0.89777	0.89868	0.90163	0.90483	0.90837
858	0.88930	0.89515	0.89786	0.89877	0.90172	0.90492	0.90846
859	0.88940	0.89524	0.89796	0.89887	0.90181	0.90500	0.90854
860	0.88949	0.89534	0.89805	0.89896	0.90190	0.90509	0.90862
861	0.88959	0.89544	0.89814	0.89905	0.90199	0.90517	0.90870
862	0.88969	0.89553	0.89823	0.89914	0.90208	0.90526	0.90878
863	0.88979	0.89563	0.89833	0.89923	0.90217	0.90535	0.90887
864	0.88990	0.89572	0.89842	0.89932	0.90225	0.90543	0.90895
865	0.89000	0.89582	0.89851	0.89941	0.90234	0.90552	0.90903
866	0.89011	0.89591	0.89860	0.89950	0.90243	0.90560	0.90911
867	0.89020	0.89601	0.89869	0.89959	0.90252	0.90569	0.90919
868	0.89029	0.89610	0.89879	0.89968	0.90261	0.90577	0.90927
869	0.89040	0.89620	0.89888	0.89978	0.90269	0.90586	0.90935
870	0.89048	0.89629	0.89898	0.89987	0.90278	0.90594	0.90943
871	0.89058	0.89638	0.89906	0.89996	0.90287	0.90602	0.90951
872	0.89069	0.89648	0.89915	0.90005	0.90295	0.90611	0.90959
873	0.89079	0.89657	0.89924	0.90014	0.90304	0.90619	0.90967
874	0.89089	0.89666	0.89933	0.90023	0.90313	0.90627	0.90975
875	0.89099	0.89676	0.89942	0.90032	0.90321	0.90636	0.90983
876	0.89110	0.89685	0.89951	0.90041	0.90330	0.90644	0.90991
877	0.89120	0.89694	0.89960	0.90049	0.90339	0.90652	0.90999
878	0.89129	0.89703	0.89969	0.90058	0.90347	0.90660	0.91007
879	0.89139	0.89712	0.89978	0.90068	0.90355	0.90669	0.91015
880	0.89148	0.89721	0.89987	0.90076	0.90364	0.90677	0.91023
881	0.89158	0.89730	0.89997	0.90085	0.90373	0.90685	0.91031
882	0.89169	0.89740	0.90005	0.90094	0.90381	0.90694	0.91038
883	0.89178	0.89749	0.90014	0.90103	0.90390	0.90702	0.91046
884	0.89187	0.89758	0.90023	0.90111	0.90398	0.90710	0.91054
885	0.89197	0.89768	0.90032	0.90120	0.90407	0.90718	0.91062
886	0.89207	0.89777	0.90040	0.90129	0.90415	0.90726	0.91070
887	0.89217	0.89786	0.90050	0.90138	0.90423	0.90734	0.91077
888	0.89227	0.89795	0.90058	0.90147	0.90432	0.90742	0.91085
889	0.89237	0.89804	0.90067	0.90155	0.90440	0.90751	0.91093
890	0.89247	0.89813	0.90076	0.90164	0.90449	0.90759	0.91101
891	0.89257	0.89822	0.90085	0.90173	0.90458	0.90767	0.91109

N	0.1%	0.5%	1%	1.25%	2.5%	5%	10%
892	0.89267	0.89832	0.90093	0.90182	0.90466	0.90775	0.91116
893	0.89275	0.89840	0.90102	0.90190	0.90474	0.90783	0.91124
894	0.89285	0.89849	0.90110	0.90199	0.90483	0.90791	0.91132
895	0.89294	0.89858	0.90119	0.90207	0.90491	0.90799	0.91139
896	0.89303	0.89867	0.90128	0.90216	0.90499	0.90807	0.91147
897	0.89313	0.89876	0.90136	0.90224	0.90508	0.90815	0.91155
898	0.89322	0.89885	0.90145	0.90233	0.90516	0.90822	0.91162
899	0.89331	0.89893	0.90153	0.90242	0.90524	0.90830	0.91170
900	0.89340	0.89902	0.90162	0.90250	0.90532	0.90838	0.91177
901	0.89350	0.89912	0.90170	0.90259	0.90541	0.90846	0.91185
902	0.89360	0.89920	0.90179	0.90267	0.90549	0.90854	0.91193
903	0.89369	0.89929	0.90188	0.90275	0.90557	0.90862	0.91200
904	0.89380	0.89938	0.90196	0.90284	0.90565	0.90870	0.91208
905	0.89389	0.89947	0.90205	0.90292	0.90573	0.90878	0.91215
906	0.89397	0.89955	0.90214	0.90301	0.90581	0.90886	0.91223
907	0.89405	0.89964	0.90222	0.90309	0.90590	0.90894	0.91230
908	0.89415	0.89973	0.90230	0.90317	0.90598	0.90901	0.91238
909	0.89424	0.89982	0.90239	0.90326	0.90606	0.90909	0.91245
910	0.89433	0.89991	0.90248	0.90334	0.90614	0.90917	0.91253
911	0.89442	0.89999	0.90256	0.90342	0.90622	0.90925	0.91260
912	0.89452	0.90008	0.90264	0.90351	0.90630	0.90932	0.91267
913	0.89462	0.90017	0.90273	0.90359	0.90638	0.90940	0.91275
914	0.89470	0.90025	0.90282	0.90367	0.90646	0.90948	0.91282
915	0.89479	0.90033	0.90290	0.90376	0.90654	0.90955	0.91289
916	0.89488	0.90042	0.90298	0.90384	0.90662	0.90963	0.91297
917	0.89498	0.90051	0.90306	0.90392	0.90670	0.90971	0.91304
918	0.89507	0.90059	0.90315	0.90400	0.90678	0.90978	0.91311
919	0.89516	0.90068	0.90323	0.90408	0.90686	0.90986	0.91319
920	0.89526	0.90076	0.90331	0.90417	0.90694	0.90993	0.91326
921	0.89535	0.90084	0.90339	0.90425	0.90702	0.91001	0.91333
922	0.89544	0.90093	0.90348	0.90433	0.90710	0.91009	0.91341
923	0.89554	0.90102	0.90356	0.90441	0.90717	0.91016	0.91348
924	0.89562	0.90110	0.90364	0.90449	0.90725	0.91024	0.91355
925	0.89571	0.90119	0.90373	0.90457	0.90733	0.91031	0.91362
926	0.89580	0.90127	0.90381	0.90465	0.90741	0.91039	0.91370
927	0.89589	0.90135	0.90389	0.90474	0.90749	0.91046	0.91377
928	0.89599	0.90143	0.90398	0.90482	0.90757	0.91054	0.91384
929	0.89607	0.90152	0.90405	0.90490	0.90765	0.91061	0.91391
930	0.89616	0.90161	0.90414	0.90497	0.90773	0.91069	0.91398
931	0.89625	0.90169	0.90422	0.90505	0.90780	0.91076	0.91406
932	0.89633	0.90178	0.90430	0.90514	0.90788	0.91084	0.91413
933	0.89641	0.90186	0.90438	0.90522	0.90796	0.91091	0.91420
934	0.89651	0.90194	0.90446	0.90530	0.90803	0.91099	0.91427
935	0.89660	0.90203	0.90454	0.90537	0.90811	0.91106	0.91434
936	0.89669	0.90211	0.90462	0.90546	0.90819	0.91114	0.91441
937	0.89677	0.90220	0.90470	0.90553	0.90826	0.91121	0.91448
938	0.89685	0.90228	0.90478	0.90562	0.90834	0.91128	0.91455
939	0.89695	0.90236	0.90486	0.90569	0.90842	0.91135	0.91462
940	0.89704	0.90244	0.90494	0.90577	0.90849	0.91143	0.91469
941	0.89712	0.90252	0.90502	0.90585	0.90857	0.91150	0.91476
942	0.89721	0.90260	0.90510	0.90593	0.90864	0.91158	0.91483
943	0.89729	0.90268	0.90518	0.90601	0.90872	0.91165	0.91490
944	0.89738	0.90276	0.90526	0.90609	0.90880	0.91172	0.91497
945	0.89746	0.90285	0.90534	0.90617	0.90887	0.91179	0.91504
946	0.89755	0.90293	0.90542	0.90624	0.90895	0.91187	0.91511
947	0.89762	0.90301	0.90550	0.90632	0.90902	0.91194	0.91518
948	0.89771	0.90309	0.90557	0.90640	0.90910	0.91201	0.91525
949	0.89780	0.90317	0.90565	0.90648	0.90917	0.91208	0.91532
950	0.89790	0.90325	0.90573	0.90655	0.90925	0.91215	0.91539
951	0.89798	0.90332	0.90581	0.90663	0.90932	0.91223	0.91546
952	0.89806	0.90340	0.90589	0.90671	0.90939	0.91230	0.91553
953	0.89815	0.90348	0.90596	0.90678	0.90947	0.91237	0.91560
954	0.89824	0.90356	0.90604	0.90686	0.90954	0.91244	0.91566
955	0.89832	0.90364	0.90612	0.90694	0.90962	0.91251	0.91573
956	0.89841	0.90372	0.90620	0.90702	0.90969	0.91259	0.91580
957	0.89850	0.90379	0.90627	0.90709	0.90977	0.91266	0.91587
958	0.89859	0.90388	0.90635	0.90717	0.90984	0.91273	0.91594
959	0.89866	0.90396	0.90643	0.90724	0.90991	0.91280	0.91600
960	0.89874	0.90404	0.90651	0.90732	0.90999	0.91287	0.91607
961	0.89884	0.90412	0.90659	0.90740	0.91006	0.91294	0.91614
962	0.89893	0.90420	0.90666	0.90747	0.91014	0.91301	0.91621
963	0.89900	0.90428	0.90674	0.90755	0.91021	0.91308	0.91628
964	0.89908	0.90436	0.90682	0.90763	0.91028	0.91316	0.91634
965	0.89917	0.90443	0.90689	0.90770	0.91035	0.91323	0.91641

N	0.1%	0.5%	1%	1.25%	2.5%	5%	10%
966	0.89925	0.90451	0.90697	0.90778	0.91043	0.91330	0.91648
967	0.89933	0.90458	0.90704	0.90785	0.91050	0.91336	0.91654
968	0.89941	0.90467	0.90712	0.90793	0.91057	0.91343	0.91661
969	0.89949	0.90474	0.90719	0.90800	0.91064	0.91350	0.91668
970	0.89958	0.90482	0.90727	0.90807	0.91071	0.91357	0.91674
971	0.89967	0.90490	0.90734	0.90815	0.91079	0.91364	0.91681
972	0.89974	0.90497	0.90741	0.90822	0.91086	0.91371	0.91688
973	0.89981	0.90505	0.90749	0.90830	0.91093	0.91378	0.91694
974	0.89991	0.90513	0.90756	0.90837	0.91100	0.91385	0.91701
975	0.89999	0.90521	0.90764	0.90845	0.91107	0.91392	0.91708
976	0.90007	0.90529	0.90772	0.90852	0.91115	0.91399	0.91714
977	0.90016	0.90536	0.90779	0.90859	0.91122	0.91405	0.91721
978	0.90024	0.90544	0.90787	0.90867	0.91129	0.91412	0.91727
979	0.90031	0.90552	0.90794	0.90874	0.91136	0.91419	0.91734
980	0.90039	0.90560	0.90801	0.90881	0.91143	0.91426	0.91740
981	0.90047	0.90567	0.90809	0.90889	0.91150	0.91433	0.91747
982	0.90056	0.90575	0.90816	0.90897	0.91158	0.91440	0.91753
983	0.90064	0.90582	0.90823	0.90904	0.91165	0.91446	0.91760
984	0.90072	0.90589	0.90831	0.90911	0.91171	0.91453	0.91766
985	0.90081	0.90597	0.90838	0.90918	0.91178	0.91460	0.91773
986	0.90089	0.90605	0.90845	0.90926	0.91186	0.91467	0.91779
987	0.90097	0.90613	0.90853	0.90933	0.91193	0.91474	0.91786
988	0.90105	0.90620	0.90860	0.90940	0.91200	0.91480	0.91792
989	0.90113	0.90628	0.90867	0.90947	0.91207	0.91487	0.91799
990	0.90121	0.90636	0.90874	0.90954	0.91214	0.91494	0.91805
991	0.90129	0.90643	0.90881	0.90962	0.91221	0.91501	0.91811
992	0.90137	0.90651	0.90889	0.90969	0.91228	0.91507	0.91818
993	0.90144	0.90658	0.90896	0.90976	0.91235	0.91514	0.91824
994	0.90152	0.90666	0.90903	0.90983	0.91242	0.91521	0.91831
995	0.90160	0.90673	0.90911	0.90991	0.91249	0.91527	0.91837
996	0.90167	0.90680	0.90918	0.90998	0.91255	0.91534	0.91843
997	0.90176	0.90688	0.90925	0.91005	0.91262	0.91540	0.91850
998	0.90183	0.90695	0.90932	0.91012	0.91269	0.91547	0.91856
999	0.90190	0.90702	0.90940	0.91019	0.91276	0.91554	0.91862
1000	0.90199	0.90710	0.90947	0.91026	0.91283	0.91560	0.91869

Table 2 – *GM10(9H1L or 1H9L)*. Critical values of upper significance levels for the Grubbs multiple-10 outlier test, applicable in situations where the nine highest and one lowest or one highest and nine lowest observations are simultaneously identified as outliers, denoted as *GM10(9H1L)* and *GM10(1H9L)*, respectively.

N	0.1%	0.5%	1%	1.25%	2.5%	5%	10%
12	0.00000	0.00000	0.00000	0.00000	0.00000	0.00002	0.00008
13	0.00001	0.00006	0.00013	0.00016	0.00033	0.00069	0.00151
14	0.00018	0.00056	0.00091	0.00107	0.00179	0.00304	0.00531
15	0.00079	0.00190	0.00280	0.00318	0.00476	0.00722	0.01124
16	0.00210	0.00430	0.00592	0.00658	0.00919	0.01302	0.01887
17	0.00420	0.00776	0.01020	0.01117	0.01488	0.02012	0.02779
18	0.00715	0.01221	0.01552	0.01680	0.02167	0.02831	0.03765
19	0.01091	0.01752	0.02176	0.02338	0.02935	0.03729	0.04819
20	0.01534	0.02363	0.02871	0.03062	0.03768	0.04682	0.05919
21	0.02042	0.03033	0.03632	0.03853	0.04654	0.05683	0.07048
22	0.02606	0.03760	0.04438	0.04687	0.05581	0.06712	0.08196
23	0.03220	0.04528	0.05280	0.05555	0.06534	0.07760	0.09347
24	0.03873	0.05323	0.06150	0.06447	0.07506	0.08820	0.10501
25	0.04565	0.06152	0.07038	0.07359	0.08491	0.09883	0.11646
26	0.05274	0.06997	0.07947	0.08287	0.09486	0.10944	0.12777
27	0.06021	0.07857	0.08862	0.09223	0.10476	0.11998	0.13894
28	0.06779	0.08723	0.09783	0.10156	0.11467	0.13043	0.14994
29	0.07554	0.09591	0.10695	0.11090	0.12450	0.14075	0.16078
30	0.08329	0.10461	0.11605	0.12014	0.13421	0.15089	0.17141
31	0.09120	0.11328	0.12520	0.12938	0.14378	0.16094	0.18185
32	0.09897	0.12191	0.13421	0.13852	0.15330	0.17078	0.19203
33	0.10682	0.13057	0.14314	0.14752	0.16266	0.18046	0.20202
34	0.11457	0.13913	0.15199	0.15652	0.17191	0.18998	0.21178
35	0.12247	0.14767	0.16078	0.16539	0.18099	0.19930	0.22134
36	0.13041	0.15605	0.16941	0.17406	0.18994	0.20846	0.23064
37	0.13808	0.16431	0.17789	0.18265	0.19869	0.21741	0.23977
38	0.14598	0.17256	0.18628	0.19107	0.20733	0.22620	0.24865
39	0.15358	0.18065	0.19450	0.19937	0.21578	0.23477	0.25732
40	0.16127	0.18852	0.20264	0.20751	0.22412	0.24319	0.26580
41	0.16878	0.19639	0.21065	0.21559	0.23227	0.25141	0.27409

N	0.1%	0.5%	1%	1.25%	2.5%	5%	10%
42	0.17607	0.20412	0.21844	0.22341	0.24025	0.25946	0.28218
43	0.18340	0.21174	0.22616	0.23113	0.24808	0.26736	0.29009
44	0.19065	0.21919	0.23377	0.23880	0.25571	0.27503	0.29782
45	0.19783	0.22663	0.24121	0.24625	0.26322	0.28261	0.30535
46	0.20480	0.23383	0.24849	0.25358	0.27055	0.28998	0.31272
47	0.21156	0.24095	0.25567	0.26072	0.27780	0.29720	0.31992
48	0.21841	0.24793	0.26266	0.26774	0.28486	0.30425	0.32696
49	0.22507	0.25475	0.26952	0.27469	0.29176	0.31119	0.33386
50	0.23178	0.26148	0.27633	0.28141	0.29854	0.31797	0.34062
51	0.23838	0.26819	0.28302	0.28814	0.30519	0.32460	0.34719
52	0.24497	0.27467	0.28952	0.29467	0.31172	0.33110	0.35361
53	0.25123	0.28113	0.29597	0.30106	0.31814	0.33747	0.35989
54	0.25750	0.28727	0.30217	0.30727	0.32439	0.34373	0.36607
55	0.26356	0.29353	0.30835	0.31352	0.33055	0.34982	0.37211
56	0.26949	0.29957	0.31448	0.31958	0.33659	0.35584	0.37801
57	0.27536	0.30554	0.32044	0.32558	0.34255	0.36169	0.38378
58	0.28097	0.31149	0.32631	0.33142	0.34832	0.36745	0.38948
59	0.28678	0.31728	0.33204	0.33715	0.35405	0.37307	0.39504
60	0.29252	0.32294	0.33774	0.34282	0.35962	0.37862	0.40048
61	0.29800	0.32845	0.34333	0.34834	0.36514	0.38405	0.40582
62	0.30348	0.33396	0.34875	0.35378	0.37056	0.38937	0.41105
63	0.30902	0.33941	0.35411	0.35912	0.37584	0.39462	0.41616
64	0.31425	0.34460	0.35935	0.36433	0.38101	0.39972	0.42118
65	0.31960	0.34981	0.36451	0.36950	0.38609	0.40472	0.42609
66	0.32483	0.35489	0.36957	0.37459	0.39113	0.40962	0.43093
67	0.33003	0.35996	0.37453	0.37952	0.39603	0.41446	0.43564
68	0.33501	0.36491	0.37950	0.38444	0.40083	0.41922	0.44028
69	0.33989	0.36983	0.38432	0.38921	0.40558	0.42390	0.44486
70	0.34474	0.37461	0.38909	0.39400	0.41026	0.42850	0.44934
71	0.34951	0.37930	0.39374	0.39864	0.41484	0.43299	0.45373
72	0.35417	0.38396	0.39831	0.40320	0.41938	0.43741	0.45805
73	0.35872	0.38844	0.40284	0.40769	0.42380	0.44177	0.46230
74	0.36328	0.39294	0.40725	0.41213	0.42814	0.44604	0.46646
75	0.36774	0.39735	0.41162	0.41643	0.43246	0.45022	0.47056
76	0.37217	0.40177	0.41584	0.42065	0.43666	0.45435	0.47456
77	0.37649	0.40601	0.42010	0.42490	0.44078	0.45841	0.47852
78	0.38096	0.41028	0.42428	0.42906	0.44486	0.46241	0.48239
79	0.38503	0.41443	0.42835	0.43310	0.44887	0.46634	0.48621
80	0.38924	0.41846	0.43237	0.43713	0.45281	0.47017	0.48996
81	0.39340	0.42246	0.43635	0.44108	0.45664	0.47397	0.49365
82	0.39756	0.42646	0.44026	0.44497	0.46048	0.47771	0.49729
83	0.40145	0.43030	0.44415	0.44880	0.46423	0.48140	0.50084
84	0.40540	0.43413	0.44793	0.45259	0.46794	0.48500	0.50437
85	0.40937	0.43787	0.45166	0.45626	0.47157	0.48858	0.50783
86	0.41323	0.44161	0.45528	0.45992	0.47519	0.49209	0.51124
87	0.41694	0.44530	0.45891	0.46354	0.47871	0.49555	0.51457
88	0.42071	0.44892	0.46248	0.46706	0.48220	0.49894	0.51788
89	0.42437	0.45249	0.46597	0.47055	0.48562	0.50226	0.52113
90	0.42807	0.45601	0.46945	0.47401	0.48898	0.50557	0.52434
91	0.43156	0.45948	0.47287	0.47742	0.49233	0.50883	0.52748
92	0.43518	0.46286	0.47627	0.48077	0.49560	0.51202	0.53059
93	0.43869	0.46635	0.47954	0.48407	0.49888	0.51518	0.53363
94	0.44204	0.46968	0.48284	0.48729	0.50205	0.51829	0.53664
95	0.44554	0.47295	0.48605	0.49053	0.50518	0.52134	0.53960
96	0.44879	0.47621	0.48930	0.49370	0.50827	0.52435	0.54251
97	0.45206	0.47937	0.49242	0.49683	0.51131	0.52732	0.54539
98	0.45522	0.48252	0.49550	0.49991	0.51432	0.53025	0.54825
99	0.45856	0.48565	0.49855	0.50293	0.51728	0.53314	0.55104
100	0.46158	0.48874	0.50157	0.50596	0.52021	0.53601	0.55380
101	0.46483	0.49173	0.50457	0.50893	0.52311	0.53884	0.55652
102	0.46800	0.49475	0.50753	0.51185	0.52592	0.54160	0.55920
103	0.47097	0.49771	0.51042	0.51470	0.52875	0.54431	0.56186
104	0.47408	0.50055	0.51330	0.51756	0.53155	0.54702	0.56445
105	0.47700	0.50346	0.51614	0.52039	0.53430	0.54968	0.56705
106	0.48003	0.50634	0.51891	0.52315	0.53702	0.55231	0.56958
107	0.48287	0.50917	0.52163	0.52589	0.53968	0.55490	0.57209
108	0.48575	0.51194	0.52439	0.52859	0.54229	0.55746	0.57457
109	0.48862	0.51466	0.52706	0.53124	0.54489	0.55999	0.57700
110	0.49148	0.51733	0.52972	0.53386	0.54743	0.56248	0.57941
111	0.49423	0.52003	0.53228	0.53644	0.54999	0.56492	0.58178
112	0.49689	0.52266	0.53489	0.53901	0.55247	0.56735	0.58412
113	0.49973	0.52526	0.53747	0.54157	0.55494	0.56976	0.58644
114	0.50236	0.52779	0.53996	0.54403	0.55739	0.57213	0.58872
115	0.50497	0.53034	0.54241	0.54646	0.55980	0.57445	0.59098

N	0.1%	0.5%	1%	1.25%	2.5%	5%	10%
116	0.50762	0.53282	0.54486	0.54891	0.56220	0.57678	0.59320
117	0.51021	0.53531	0.54729	0.55134	0.56456	0.57905	0.59540
118	0.51268	0.53776	0.54970	0.55374	0.56687	0.58131	0.59758
119	0.51527	0.54016	0.55209	0.55607	0.56917	0.58352	0.59970
120	0.51770	0.54257	0.55443	0.55841	0.57144	0.58573	0.60183
121	0.52025	0.54496	0.55678	0.56072	0.57368	0.58789	0.60393
122	0.52261	0.54726	0.55911	0.56302	0.57588	0.59002	0.60601
123	0.52508	0.54960	0.56134	0.56526	0.57806	0.59215	0.60805
124	0.52742	0.55188	0.56357	0.56749	0.58022	0.59425	0.61007
125	0.52970	0.55411	0.56576	0.56967	0.58235	0.59633	0.61209
126	0.53205	0.55640	0.56795	0.57185	0.58446	0.59837	0.61406
127	0.53439	0.55862	0.57013	0.57400	0.58656	0.60041	0.61601
128	0.53673	0.56080	0.57226	0.57610	0.58862	0.60241	0.61793
129	0.53890	0.56291	0.57437	0.57820	0.59068	0.60440	0.61984
130	0.54107	0.56507	0.57644	0.58026	0.59269	0.60636	0.62172
131	0.54339	0.56718	0.57851	0.58231	0.59466	0.60829	0.62358
132	0.54554	0.56924	0.58056	0.58433	0.59664	0.61021	0.62542
133	0.54769	0.57139	0.58256	0.58632	0.59861	0.61211	0.62725
134	0.54987	0.57340	0.58456	0.58832	0.60054	0.61398	0.62906
135	0.55203	0.57542	0.58653	0.59028	0.60245	0.61584	0.63085
136	0.55418	0.57741	0.58852	0.59222	0.60432	0.61766	0.63260
137	0.55631	0.57942	0.59049	0.59416	0.60620	0.61948	0.63435
138	0.55836	0.58143	0.59239	0.59604	0.60807	0.62129	0.63608
139	0.56044	0.58333	0.59427	0.59794	0.60991	0.62305	0.63778
140	0.56243	0.58526	0.59616	0.59981	0.61171	0.62481	0.63945
141	0.56439	0.58715	0.59798	0.60164	0.61350	0.62656	0.64113
142	0.56634	0.58903	0.59985	0.60347	0.61530	0.62827	0.64278
143	0.56831	0.59090	0.60169	0.60528	0.61707	0.62997	0.64443
144	0.57030	0.59275	0.60349	0.60707	0.61879	0.63166	0.64605
145	0.57227	0.59459	0.60526	0.60885	0.62050	0.63333	0.64764
146	0.57415	0.59643	0.60704	0.61058	0.62222	0.63499	0.64923
147	0.57606	0.59823	0.60880	0.61233	0.62390	0.63661	0.65080
148	0.57781	0.60003	0.61052	0.61404	0.62556	0.63822	0.65235
149	0.57971	0.60178	0.61221	0.61572	0.62721	0.63983	0.65389
150	0.58165	0.60352	0.61394	0.61743	0.62884	0.64140	0.65541
151	0.58339	0.60528	0.61562	0.61909	0.63047	0.64297	0.65693
152	0.58516	0.60696	0.61729	0.62075	0.63208	0.64453	0.65842
153	0.58696	0.60870	0.61893	0.62240	0.63368	0.64606	0.65991
154	0.58868	0.61033	0.62056	0.62401	0.63524	0.64758	0.66137
155	0.59048	0.61199	0.62220	0.62562	0.63680	0.64909	0.66281
156	0.59219	0.61363	0.62382	0.62721	0.63833	0.65058	0.66425
157	0.59379	0.61525	0.62539	0.62878	0.63988	0.65205	0.66567
158	0.59557	0.61684	0.62696	0.63033	0.64139	0.65352	0.66709
159	0.59725	0.61843	0.62849	0.63187	0.64290	0.65498	0.66848
160	0.59890	0.62007	0.63006	0.63339	0.64438	0.65641	0.66986
161	0.60055	0.62160	0.63157	0.63490	0.64586	0.65783	0.67122
162	0.60214	0.62314	0.63307	0.63641	0.64734	0.65924	0.67258
163	0.60381	0.62471	0.63458	0.63790	0.64878	0.66064	0.67392
164	0.60539	0.62624	0.63606	0.63934	0.65020	0.66202	0.67525
165	0.60703	0.62774	0.63756	0.64082	0.65163	0.66340	0.67657
166	0.60864	0.62926	0.63900	0.64226	0.65304	0.66475	0.67788
167	0.61018	0.63073	0.64044	0.64371	0.65444	0.66611	0.67916
168	0.61165	0.63219	0.64189	0.64513	0.65582	0.66744	0.68044
169	0.61320	0.63369	0.64330	0.64656	0.65719	0.66876	0.68172
170	0.61475	0.63514	0.64472	0.64795	0.65855	0.67007	0.68297
171	0.61625	0.63654	0.64611	0.64935	0.65988	0.67138	0.68422
172	0.61774	0.63802	0.64750	0.65070	0.66120	0.67266	0.68546
173	0.61917	0.63942	0.64887	0.65206	0.66251	0.67395	0.68669
174	0.62067	0.64081	0.65023	0.65341	0.66382	0.67522	0.68791
175	0.62221	0.64218	0.65157	0.65476	0.66511	0.67648	0.68910
176	0.62359	0.64353	0.65291	0.65608	0.66638	0.67769	0.69029
177	0.62502	0.64493	0.65423	0.65739	0.66767	0.67893	0.69148
178	0.62639	0.64627	0.65556	0.65870	0.66894	0.68014	0.69266
179	0.62787	0.64762	0.65687	0.65998	0.67019	0.68136	0.69383
180	0.62920	0.64895	0.65816	0.66126	0.67145	0.68256	0.69498
181	0.63056	0.65024	0.65943	0.66256	0.67267	0.68375	0.69613
182	0.63194	0.65151	0.66073	0.66381	0.67390	0.68494	0.69725
183	0.63330	0.65282	0.66200	0.66507	0.67512	0.68609	0.69837
184	0.63469	0.65408	0.66324	0.66631	0.67632	0.68725	0.69949
185	0.63602	0.65533	0.66445	0.66753	0.67751	0.68841	0.70059
186	0.63736	0.65662	0.66568	0.66877	0.67871	0.68954	0.70169
187	0.63872	0.65783	0.66692	0.66996	0.67988	0.69068	0.70277
188	0.64005	0.65908	0.66812	0.67116	0.68104	0.69179	0.70385
189	0.64125	0.66035	0.66932	0.67233	0.68220	0.69291	0.70491

N	0.1%	0.5%	1%	1.25%	2.5%	5%	10%
190	0.64259	0.66157	0.67051	0.67351	0.68333	0.69401	0.70597
191	0.64382	0.66277	0.67170	0.67468	0.68447	0.69511	0.70703
192	0.64514	0.66396	0.67287	0.67584	0.68559	0.69619	0.70806
193	0.64636	0.66514	0.67403	0.67700	0.68669	0.69727	0.70909
194	0.64763	0.66630	0.67517	0.67815	0.68780	0.69832	0.71012
195	0.64895	0.66747	0.67631	0.67927	0.68889	0.69939	0.71114
196	0.65013	0.66864	0.67745	0.68040	0.69000	0.70044	0.71215
197	0.65131	0.66977	0.67857	0.68153	0.69107	0.70149	0.71315
198	0.65251	0.67094	0.67968	0.68263	0.69214	0.70253	0.71415
199	0.65366	0.67206	0.68079	0.68371	0.69320	0.70355	0.71514
200	0.65488	0.67320	0.68189	0.68481	0.69427	0.70457	0.71612
201	0.65605	0.67432	0.68300	0.68590	0.69532	0.70558	0.71709
202	0.65731	0.67542	0.68407	0.68697	0.69636	0.70659	0.71805
203	0.65842	0.67650	0.68512	0.68802	0.69740	0.70759	0.71900
204	0.65955	0.67761	0.68621	0.68907	0.69840	0.70858	0.71995
205	0.66067	0.67868	0.68727	0.69011	0.69941	0.70957	0.72089
206	0.66183	0.67975	0.68831	0.69115	0.70043	0.71054	0.72182
207	0.66292	0.68083	0.68935	0.69217	0.70144	0.71151	0.72275
208	0.66400	0.68191	0.69037	0.69322	0.70244	0.71246	0.72367
209	0.66514	0.68297	0.69141	0.69424	0.70342	0.71344	0.72458
210	0.66626	0.68401	0.69241	0.69523	0.70439	0.71437	0.72549
211	0.66731	0.68505	0.69344	0.69624	0.70537	0.71530	0.72640
212	0.66849	0.68610	0.69445	0.69724	0.70632	0.71624	0.72729
213	0.66949	0.68710	0.69545	0.69823	0.70725	0.71716	0.72817
214	0.67060	0.68814	0.69644	0.69920	0.70822	0.71807	0.72905
215	0.67168	0.68914	0.69742	0.70017	0.70917	0.71898	0.72992
216	0.67271	0.69015	0.69838	0.70113	0.71010	0.71988	0.73079
217	0.67374	0.69113	0.69935	0.70210	0.71102	0.72078	0.73165
218	0.67479	0.69211	0.70032	0.70304	0.71195	0.72167	0.73251
219	0.67587	0.69313	0.70126	0.70398	0.71287	0.72256	0.73335
220	0.67689	0.69407	0.70222	0.70493	0.71378	0.72343	0.73420
221	0.67786	0.69505	0.70316	0.70585	0.71468	0.72432	0.73503
222	0.67892	0.69604	0.70410	0.70679	0.71560	0.72518	0.73586
223	0.67991	0.69701	0.70502	0.70771	0.71649	0.72604	0.73668
224	0.68091	0.69793	0.70590	0.70862	0.71738	0.72689	0.73750
225	0.68190	0.69886	0.70683	0.70953	0.71826	0.72773	0.73831
226	0.68288	0.69977	0.70772	0.71042	0.71913	0.72857	0.73912
227	0.68385	0.70068	0.70863	0.71129	0.72000	0.72942	0.73991
228	0.68483	0.70160	0.70954	0.71220	0.72085	0.73024	0.74072
229	0.68583	0.70254	0.71043	0.71308	0.72169	0.73106	0.74151
230	0.68677	0.70344	0.71131	0.71395	0.72255	0.73189	0.74229
231	0.68775	0.70433	0.71219	0.71483	0.72339	0.73271	0.74307
232	0.68867	0.70522	0.71308	0.71569	0.72423	0.73352	0.74385
233	0.68958	0.70612	0.71392	0.71653	0.72507	0.73432	0.74462
234	0.69046	0.70701	0.71478	0.71738	0.72589	0.73511	0.74538
235	0.69139	0.70789	0.71565	0.71822	0.72671	0.73591	0.74614
236	0.69236	0.70874	0.71649	0.71907	0.72752	0.73668	0.74689
237	0.69329	0.70964	0.71735	0.71991	0.72835	0.73746	0.74763
238	0.69420	0.71050	0.71816	0.72074	0.72916	0.73825	0.74837
239	0.69506	0.71135	0.71899	0.72156	0.72996	0.73902	0.74912
240	0.69599	0.71219	0.71982	0.72239	0.73075	0.73978	0.74985
241	0.69688	0.71304	0.72064	0.72319	0.73153	0.74054	0.75058
242	0.69785	0.71385	0.72146	0.72403	0.73231	0.74130	0.75130
243	0.69865	0.71473	0.72227	0.72484	0.73309	0.74205	0.75202
244	0.69950	0.71554	0.72308	0.72562	0.73387	0.74279	0.75273
245	0.70033	0.71634	0.72391	0.72641	0.73462	0.74354	0.75344
246	0.70121	0.71716	0.72468	0.72720	0.73538	0.74427	0.75415
247	0.70209	0.71797	0.72547	0.72799	0.73614	0.74500	0.75485
248	0.70291	0.71878	0.72626	0.72875	0.73691	0.74572	0.75555
249	0.70375	0.71959	0.72704	0.72954	0.73766	0.74645	0.75624
250	0.70459	0.72040	0.72780	0.73030	0.73840	0.74716	0.75693
251	0.70545	0.72119	0.72857	0.73108	0.73914	0.74788	0.75762
252	0.70626	0.72197	0.72935	0.73184	0.73987	0.74859	0.75830
253	0.70709	0.72275	0.73010	0.73258	0.74059	0.74929	0.75897
254	0.70795	0.72353	0.73086	0.73331	0.74132	0.74999	0.75964
255	0.70873	0.72430	0.73161	0.73406	0.74205	0.75069	0.76030
256	0.70954	0.72509	0.73236	0.73480	0.74276	0.75138	0.76096
257	0.71036	0.72583	0.73310	0.73553	0.74346	0.75206	0.76162
258	0.71120	0.72658	0.73383	0.73624	0.74417	0.75274	0.76227
259	0.71198	0.72734	0.73456	0.73697	0.74487	0.75343	0.76292
260	0.71277	0.72810	0.73529	0.73769	0.74556	0.75410	0.76357
261	0.71356	0.72883	0.73600	0.73840	0.74625	0.75478	0.76421
262	0.71435	0.72957	0.73673	0.73913	0.74693	0.75543	0.76484
263	0.71521	0.73029	0.73742	0.73982	0.74761	0.75609	0.76548

N	0.1%	0.5%	1%	1.25%	2.5%	5%	10%
264	0.71594	0.73102	0.73815	0.74052	0.74829	0.75674	0.76611
265	0.71672	0.73174	0.73885	0.74122	0.74897	0.75739	0.76673
266	0.71748	0.73246	0.73954	0.74192	0.74965	0.75804	0.76736
267	0.71819	0.73319	0.74023	0.74260	0.75032	0.75868	0.76798
268	0.71894	0.73390	0.74094	0.74329	0.75097	0.75933	0.76859
269	0.71973	0.73461	0.74163	0.74397	0.75164	0.75996	0.76921
270	0.72045	0.73530	0.74232	0.74464	0.75229	0.76060	0.76981
271	0.72118	0.73601	0.74299	0.74532	0.75295	0.76124	0.77042
272	0.72191	0.73673	0.74366	0.74598	0.75360	0.76186	0.77102
273	0.72266	0.73741	0.74432	0.74665	0.75424	0.76248	0.77162
274	0.72342	0.73810	0.74498	0.74731	0.75489	0.76310	0.77221
275	0.72414	0.73877	0.74565	0.74799	0.75552	0.76371	0.77280
276	0.72488	0.73947	0.74633	0.74864	0.75615	0.76433	0.77338
277	0.72560	0.74016	0.74699	0.74930	0.75679	0.76493	0.77396
278	0.72629	0.74085	0.74764	0.74994	0.75741	0.76553	0.77455
279	0.72700	0.74150	0.74830	0.75058	0.75804	0.76613	0.77513
280	0.72777	0.74215	0.74895	0.75123	0.75866	0.76673	0.77570
281	0.72844	0.74283	0.74961	0.75187	0.75928	0.76732	0.77627
282	0.72915	0.74348	0.75025	0.75251	0.75989	0.76791	0.77684
283	0.72983	0.74413	0.75089	0.75313	0.76050	0.76850	0.77740
284	0.73047	0.74480	0.75151	0.75376	0.76111	0.76909	0.77796
285	0.73114	0.74544	0.75213	0.75438	0.76170	0.76967	0.77851
286	0.73184	0.74611	0.75275	0.75499	0.76231	0.77025	0.77907
287	0.73252	0.74674	0.75338	0.75562	0.76290	0.77082	0.77962
288	0.73319	0.74738	0.75400	0.75623	0.76350	0.77139	0.78017
289	0.73389	0.74799	0.75462	0.75684	0.76409	0.77196	0.78071
290	0.73452	0.74863	0.75524	0.75745	0.76469	0.77253	0.78126
291	0.73519	0.74925	0.75585	0.75805	0.76526	0.77308	0.78179
292	0.73582	0.74986	0.75644	0.75865	0.76584	0.77364	0.78233
293	0.73652	0.75048	0.75704	0.75925	0.76642	0.77419	0.78286
294	0.73718	0.75108	0.75763	0.75985	0.76698	0.77475	0.78339
295	0.73785	0.75170	0.75824	0.76044	0.76756	0.77530	0.78391
296	0.73850	0.75232	0.75882	0.76102	0.76813	0.77585	0.78444
297	0.73911	0.75293	0.75941	0.76160	0.76869	0.77639	0.78495
298	0.73977	0.75352	0.76000	0.76217	0.76925	0.77694	0.78547
299	0.74036	0.75413	0.76057	0.76275	0.76980	0.77747	0.78599
300	0.74102	0.75471	0.76115	0.76334	0.77036	0.77801	0.78650
301	0.74165	0.75531	0.76173	0.76390	0.77091	0.77853	0.78702
302	0.74228	0.75591	0.76229	0.76446	0.77146	0.77906	0.78752
303	0.74287	0.75647	0.76286	0.76502	0.77200	0.77958	0.78802
304	0.74346	0.75705	0.76342	0.76558	0.77254	0.78010	0.78853
305	0.74408	0.75763	0.76400	0.76612	0.77309	0.78062	0.78903
306	0.74468	0.75822	0.76457	0.76669	0.77362	0.78114	0.78952
307	0.74528	0.75880	0.76512	0.76725	0.77416	0.78166	0.79001
308	0.74591	0.75936	0.76566	0.76778	0.77470	0.78217	0.79050
309	0.74651	0.75993	0.76622	0.76834	0.77522	0.78268	0.79099
310	0.74707	0.76050	0.76675	0.76889	0.77574	0.78319	0.79147
311	0.74769	0.76106	0.76728	0.76942	0.77627	0.78370	0.79196
312	0.74832	0.76164	0.76785	0.76996	0.77679	0.78419	0.79244
313	0.74885	0.76218	0.76838	0.77048	0.77731	0.78470	0.79291
314	0.74946	0.76273	0.76891	0.77101	0.77781	0.78519	0.79340
315	0.75004	0.76328	0.76946	0.77154	0.77832	0.78569	0.79387
316	0.75062	0.76383	0.76997	0.77207	0.77883	0.78619	0.79434
317	0.75117	0.76438	0.77049	0.77259	0.77934	0.78667	0.79481
318	0.75176	0.76491	0.77104	0.77312	0.77984	0.78716	0.79528
319	0.75235	0.76545	0.77156	0.77363	0.78035	0.78764	0.79574
320	0.75291	0.76600	0.77209	0.77415	0.78086	0.78813	0.79620
321	0.75346	0.76652	0.77260	0.77467	0.78136	0.78860	0.79666
322	0.75399	0.76705	0.77312	0.77518	0.78185	0.78909	0.79712
323	0.75456	0.76759	0.77364	0.77569	0.78234	0.78955	0.79757
324	0.75515	0.76811	0.77414	0.77619	0.78284	0.79003	0.79802
325	0.75573	0.76862	0.77464	0.77669	0.78333	0.79050	0.79847
326	0.75629	0.76913	0.77515	0.77719	0.78381	0.79098	0.79892
327	0.75683	0.76964	0.77565	0.77769	0.78429	0.79144	0.79937
328	0.75740	0.77017	0.77616	0.77819	0.78477	0.79190	0.79980
329	0.75794	0.77068	0.77666	0.77868	0.78525	0.79236	0.80024
330	0.75842	0.77118	0.77715	0.77918	0.78572	0.79282	0.80068
331	0.75895	0.77167	0.77764	0.77965	0.78619	0.79328	0.80112
332	0.75948	0.77219	0.77814	0.78013	0.78666	0.79373	0.80155
333	0.75999	0.77268	0.77861	0.78063	0.78713	0.79418	0.80198
334	0.76053	0.77318	0.77912	0.78111	0.78759	0.79463	0.80241
335	0.76105	0.77368	0.77961	0.78159	0.78805	0.79507	0.80284
336	0.76159	0.77418	0.78009	0.78207	0.78851	0.79552	0.80327
337	0.76211	0.77467	0.78057	0.78254	0.78896	0.79596	0.80370

N	0.1%	0.5%	1%	1.25%	2.5%	5%	10%
338	0.76263	0.77516	0.78104	0.78301	0.78942	0.79640	0.80411
339	0.76318	0.77565	0.78152	0.78348	0.78988	0.79684	0.80454
340	0.76368	0.77613	0.78200	0.78395	0.79032	0.79727	0.80495
341	0.76416	0.77663	0.78247	0.78442	0.79077	0.79771	0.80537
342	0.76466	0.77711	0.78294	0.78488	0.79123	0.79814	0.80579
343	0.76517	0.77759	0.78340	0.78535	0.79167	0.79857	0.80620
344	0.76568	0.77806	0.78386	0.78581	0.79212	0.79900	0.80661
345	0.76619	0.77855	0.78433	0.78626	0.79256	0.79942	0.80702
346	0.76669	0.77903	0.78478	0.78672	0.79300	0.79985	0.80742
347	0.76718	0.77949	0.78524	0.78717	0.79344	0.80026	0.80783
348	0.76769	0.77995	0.78570	0.78762	0.79388	0.80068	0.80823
349	0.76816	0.78042	0.78614	0.78807	0.79431	0.80110	0.80863
350	0.76868	0.78087	0.78661	0.78852	0.79475	0.80152	0.80903
351	0.76918	0.78134	0.78705	0.78897	0.79517	0.80194	0.80942
352	0.76966	0.78182	0.78749	0.78941	0.79561	0.80235	0.80982
353	0.77013	0.78227	0.78794	0.78985	0.79603	0.80276	0.81021
354	0.77061	0.78273	0.78840	0.79029	0.79646	0.80317	0.81060
355	0.77109	0.78320	0.78884	0.79073	0.79688	0.80358	0.81100
356	0.77156	0.78364	0.78928	0.79117	0.79730	0.80398	0.81138
357	0.77202	0.78407	0.78971	0.79160	0.79772	0.80438	0.81177
358	0.77247	0.78453	0.79015	0.79203	0.79814	0.80478	0.81215
359	0.77295	0.78497	0.79057	0.79246	0.79855	0.80518	0.81253
360	0.77339	0.78541	0.79101	0.79288	0.79896	0.80558	0.81291
361	0.77385	0.78585	0.79144	0.79330	0.79937	0.80597	0.81330
362	0.77432	0.78629	0.79186	0.79373	0.79977	0.80637	0.81367
363	0.77482	0.78672	0.79229	0.79414	0.80018	0.80676	0.81405
364	0.77528	0.78715	0.79272	0.79456	0.80058	0.80715	0.81442
365	0.77573	0.78759	0.79312	0.79498	0.80099	0.80754	0.81479
366	0.77617	0.78800	0.79354	0.79538	0.80139	0.80793	0.81516
367	0.77662	0.78843	0.79396	0.79580	0.80179	0.80831	0.81553
368	0.77707	0.78884	0.79436	0.79621	0.80219	0.80870	0.81590
369	0.77754	0.78926	0.79479	0.79662	0.80259	0.80907	0.81626
370	0.77797	0.78970	0.79520	0.79702	0.80299	0.80945	0.81663
371	0.77844	0.79012	0.79560	0.79742	0.80338	0.80983	0.81700
372	0.77888	0.79054	0.79601	0.79783	0.80377	0.81021	0.81735
373	0.77932	0.79096	0.79642	0.79824	0.80415	0.81058	0.81772
374	0.77977	0.79138	0.79683	0.79863	0.80454	0.81096	0.81807
375	0.78021	0.79179	0.79722	0.79903	0.80493	0.81132	0.81843
376	0.78065	0.79221	0.79762	0.79943	0.80531	0.81170	0.81878
377	0.78109	0.79261	0.79802	0.79983	0.80569	0.81206	0.81914
378	0.78153	0.79301	0.79842	0.80022	0.80607	0.81243	0.81949
379	0.78195	0.79341	0.79882	0.80060	0.80646	0.81280	0.81984
380	0.78235	0.79383	0.79922	0.80100	0.80684	0.81316	0.82019
381	0.78278	0.79422	0.79961	0.80139	0.80721	0.81352	0.82054
382	0.78322	0.79462	0.80000	0.80177	0.80758	0.81388	0.82088
383	0.78363	0.79503	0.80040	0.80215	0.80796	0.81424	0.82123
384	0.78403	0.79542	0.80078	0.80254	0.80832	0.81459	0.82156
385	0.78444	0.79583	0.80116	0.80292	0.80869	0.81495	0.82191
386	0.78487	0.79623	0.80154	0.80330	0.80906	0.81530	0.82225
387	0.78529	0.79662	0.80192	0.80368	0.80942	0.81565	0.82258
388	0.78569	0.79700	0.80230	0.80405	0.80978	0.81601	0.82292
389	0.78609	0.79739	0.80268	0.80444	0.81015	0.81636	0.82325
390	0.78646	0.79777	0.80305	0.80481	0.81050	0.81670	0.82359
391	0.78687	0.79816	0.80344	0.80518	0.81087	0.81705	0.82392
392	0.78728	0.79854	0.80381	0.80555	0.81123	0.81740	0.82425
393	0.78768	0.79892	0.80418	0.80592	0.81158	0.81774	0.82458
394	0.78810	0.79930	0.80455	0.80629	0.81194	0.81808	0.82491
395	0.78849	0.79968	0.80492	0.80666	0.81229	0.81842	0.82523
396	0.78893	0.80007	0.80529	0.80703	0.81264	0.81876	0.82556
397	0.78931	0.80045	0.80565	0.80738	0.81300	0.81910	0.82588
398	0.78969	0.80084	0.80601	0.80775	0.81335	0.81944	0.82621
399	0.79008	0.80121	0.80637	0.80811	0.81370	0.81977	0.82653
400	0.79046	0.80158	0.80673	0.80846	0.81404	0.82011	0.82685
401	0.79084	0.80195	0.80709	0.80882	0.81439	0.82044	0.82717
402	0.79125	0.80232	0.80746	0.80918	0.81474	0.82077	0.82749
403	0.79164	0.80268	0.80781	0.80954	0.81508	0.82111	0.82780
404	0.79207	0.80305	0.80816	0.80989	0.81542	0.82143	0.82812
405	0.79244	0.80342	0.80853	0.81023	0.81576	0.82176	0.82843
406	0.79282	0.80379	0.80888	0.81059	0.81610	0.82209	0.82874
407	0.79323	0.80414	0.80923	0.81093	0.81643	0.82241	0.82905
408	0.79362	0.80451	0.80958	0.81128	0.81677	0.82274	0.82936
409	0.79400	0.80487	0.80993	0.81162	0.81710	0.82306	0.82967
410	0.79437	0.80522	0.81027	0.81195	0.81743	0.82337	0.82998
411	0.79473	0.80557	0.81061	0.81230	0.81776	0.82369	0.83028

N	0.1%	0.5%	1%	1.25%	2.5%	5%	10%
412	0.79511	0.80591	0.81096	0.81264	0.81810	0.82401	0.83059
413	0.79551	0.80626	0.81130	0.81298	0.81843	0.82433	0.83090
414	0.79588	0.80661	0.81164	0.81332	0.81876	0.82464	0.83120
415	0.79624	0.80697	0.81198	0.81365	0.81908	0.82496	0.83150
416	0.79662	0.80730	0.81231	0.81398	0.81941	0.82528	0.83180
417	0.79698	0.80765	0.81266	0.81433	0.81973	0.82558	0.83209
418	0.79735	0.80799	0.81300	0.81466	0.82005	0.82589	0.83239
419	0.79771	0.80834	0.81333	0.81499	0.82038	0.82620	0.83269
420	0.79809	0.80869	0.81366	0.81532	0.82069	0.82651	0.83299
421	0.79842	0.80901	0.81400	0.81565	0.82100	0.82682	0.83328
422	0.79883	0.80936	0.81432	0.81599	0.82132	0.82712	0.83357
423	0.79917	0.80971	0.81464	0.81631	0.82164	0.82743	0.83387
424	0.79955	0.81003	0.81497	0.81664	0.82195	0.82773	0.83416
425	0.79990	0.81037	0.81530	0.81696	0.82227	0.82804	0.83445
426	0.80026	0.81072	0.81563	0.81728	0.82258	0.82834	0.83473
427	0.80061	0.81104	0.81596	0.81760	0.82288	0.82864	0.83502
428	0.80096	0.81137	0.81628	0.81792	0.82319	0.82893	0.83531
429	0.80131	0.81170	0.81660	0.81824	0.82350	0.82923	0.83559
430	0.80166	0.81204	0.81692	0.81856	0.82381	0.82953	0.83587
431	0.80203	0.81238	0.81724	0.81887	0.82411	0.82982	0.83616
432	0.80238	0.81269	0.81756	0.81918	0.82442	0.83012	0.83644
433	0.80271	0.81303	0.81787	0.81949	0.82472	0.83041	0.83672
434	0.80304	0.81336	0.81818	0.81980	0.82503	0.83070	0.83700
435	0.80341	0.81368	0.81850	0.82012	0.82533	0.83099	0.83727
436	0.80373	0.81399	0.81881	0.82042	0.82563	0.83129	0.83755
437	0.80407	0.81432	0.81913	0.82073	0.82593	0.83158	0.83783
438	0.80439	0.81464	0.81944	0.82104	0.82623	0.83186	0.83810
439	0.80474	0.81495	0.81975	0.82134	0.82652	0.83215	0.83838
440	0.80507	0.81527	0.82005	0.82164	0.82682	0.83243	0.83865
441	0.80537	0.81558	0.82036	0.82195	0.82712	0.83272	0.83892
442	0.80573	0.81589	0.82066	0.82225	0.82740	0.83300	0.83919
443	0.80606	0.81620	0.82098	0.82255	0.82770	0.83328	0.83946
444	0.80638	0.81652	0.82127	0.82284	0.82799	0.83356	0.83973
445	0.80670	0.81684	0.82158	0.82314	0.82828	0.83384	0.84000
446	0.80700	0.81714	0.82188	0.82344	0.82857	0.83412	0.84027
447	0.80735	0.81745	0.82219	0.82374	0.82886	0.83439	0.84053
448	0.80768	0.81776	0.82248	0.82404	0.82915	0.83467	0.84079
449	0.80803	0.81806	0.82278	0.82434	0.82943	0.83494	0.84106
450	0.80834	0.81837	0.82307	0.82463	0.82972	0.83522	0.84132
451	0.80866	0.81867	0.82337	0.82493	0.83000	0.83549	0.84158
452	0.80897	0.81897	0.82367	0.82522	0.83028	0.83576	0.84184
453	0.80930	0.81928	0.82396	0.82551	0.83056	0.83603	0.84211
454	0.80963	0.81957	0.82425	0.82580	0.83085	0.83631	0.84236
455	0.80992	0.81987	0.82455	0.82609	0.83113	0.83657	0.84262
456	0.81024	0.82017	0.82484	0.82639	0.83140	0.83684	0.84288
457	0.81058	0.82046	0.82512	0.82667	0.83168	0.83711	0.84314
458	0.81091	0.82075	0.82542	0.82695	0.83196	0.83737	0.84339
459	0.81120	0.82104	0.82570	0.82723	0.83224	0.83764	0.84365
460	0.81151	0.82134	0.82599	0.82752	0.83251	0.83791	0.84390
461	0.81182	0.82163	0.82628	0.82781	0.83278	0.83817	0.84415
462	0.81214	0.82192	0.82655	0.82809	0.83305	0.83843	0.84441
463	0.81246	0.82222	0.82684	0.82837	0.83333	0.83869	0.84466
464	0.81276	0.82252	0.82712	0.82865	0.83360	0.83896	0.84491
465	0.81306	0.82281	0.82741	0.82893	0.83387	0.83922	0.84516
466	0.81335	0.82311	0.82768	0.82921	0.83413	0.83947	0.84541
467	0.81366	0.82339	0.82797	0.82949	0.83440	0.83973	0.84565
468	0.81395	0.82369	0.82824	0.82976	0.83467	0.83999	0.84590
469	0.81426	0.82397	0.82851	0.83004	0.83494	0.84024	0.84614
470	0.81458	0.82425	0.82878	0.83031	0.83520	0.84050	0.84639
471	0.81488	0.82453	0.82906	0.83058	0.83547	0.84076	0.84663
472	0.81517	0.82482	0.82933	0.83085	0.83573	0.84101	0.84687
473	0.81545	0.82510	0.82961	0.83112	0.83599	0.84126	0.84712
474	0.81576	0.82538	0.82988	0.83139	0.83625	0.84152	0.84736
475	0.81603	0.82565	0.83015	0.83165	0.83650	0.84177	0.84760
476	0.81633	0.82592	0.83042	0.83192	0.83677	0.84202	0.84784
477	0.81664	0.82620	0.83068	0.83219	0.83702	0.84227	0.84808
478	0.81693	0.82648	0.83096	0.83245	0.83728	0.84252	0.84832
479	0.81723	0.82677	0.83123	0.83272	0.83754	0.84276	0.84856
480	0.81751	0.82704	0.83150	0.83298	0.83780	0.84301	0.84879
481	0.81780	0.82733	0.83176	0.83325	0.83805	0.84326	0.84903
482	0.81811	0.82759	0.83203	0.83351	0.83830	0.84350	0.84926
483	0.81839	0.82786	0.83230	0.83378	0.83856	0.84374	0.84950
484	0.81868	0.82813	0.83256	0.83404	0.83881	0.84398	0.84973
485	0.81898	0.82840	0.83282	0.83431	0.83907	0.84423	0.84996

N	0.1%	0.5%	1%	1.25%	2.5%	5%	10%
486	0.81926	0.82867	0.83308	0.83456	0.83932	0.84447	0.85019
487	0.81954	0.82893	0.83334	0.83482	0.83957	0.84471	0.85042
488	0.81983	0.82919	0.83360	0.83508	0.83982	0.84495	0.85065
489	0.82010	0.82946	0.83385	0.83533	0.84006	0.84518	0.85088
490	0.82037	0.82972	0.83411	0.83559	0.84031	0.84542	0.85111
491	0.82066	0.82999	0.83436	0.83584	0.84055	0.84566	0.85134
492	0.82093	0.83025	0.83461	0.83609	0.84080	0.84590	0.85157
493	0.82123	0.83051	0.83487	0.83634	0.84104	0.84614	0.85179
494	0.82150	0.83078	0.83513	0.83660	0.84129	0.84637	0.85202
495	0.82179	0.83103	0.83539	0.83684	0.84153	0.84660	0.85224
496	0.82208	0.83130	0.83564	0.83710	0.84178	0.84684	0.85246
497	0.82232	0.83155	0.83590	0.83734	0.84202	0.84707	0.85268
498	0.82261	0.83181	0.83614	0.83759	0.84226	0.84730	0.85291
499	0.82287	0.83207	0.83639	0.83784	0.84250	0.84753	0.85313
500	0.82313	0.83233	0.83664	0.83808	0.84273	0.84776	0.85335
501	0.82341	0.83259	0.83689	0.83833	0.84297	0.84800	0.85357
502	0.82371	0.83283	0.83712	0.83858	0.84321	0.84822	0.85379
503	0.82397	0.83309	0.83737	0.83881	0.84345	0.84845	0.85401
504	0.82426	0.83335	0.83763	0.83906	0.84369	0.84868	0.85423
505	0.82452	0.83360	0.83787	0.83930	0.84392	0.84891	0.85444
506	0.82477	0.83385	0.83812	0.83954	0.84416	0.84913	0.85466
507	0.82504	0.83410	0.83836	0.83978	0.84439	0.84936	0.85488
508	0.82530	0.83435	0.83860	0.84001	0.84462	0.84958	0.85509
509	0.82559	0.83460	0.83884	0.84025	0.84485	0.84981	0.85530
510	0.82584	0.83486	0.83908	0.84049	0.84508	0.85003	0.85552
511	0.82609	0.83511	0.83932	0.84073	0.84531	0.85025	0.85573
512	0.82637	0.83535	0.83956	0.84097	0.84554	0.85047	0.85594
513	0.82661	0.83559	0.83980	0.84120	0.84577	0.85069	0.85615
514	0.82687	0.83582	0.84004	0.84144	0.84600	0.85091	0.85636
515	0.82715	0.83607	0.84028	0.84168	0.84622	0.85113	0.85657
516	0.82738	0.83632	0.84051	0.84192	0.84645	0.85135	0.85678
517	0.82762	0.83656	0.84075	0.84215	0.84668	0.85157	0.85699
518	0.82786	0.83681	0.84098	0.84238	0.84690	0.85178	0.85720
519	0.82812	0.83704	0.84121	0.84261	0.84713	0.85200	0.85741
520	0.82838	0.83727	0.84145	0.84283	0.84735	0.85221	0.85761
521	0.82864	0.83751	0.84168	0.84307	0.84758	0.85243	0.85782
522	0.82891	0.83775	0.84191	0.84330	0.84780	0.85264	0.85803
523	0.82918	0.83798	0.84215	0.84352	0.84802	0.85286	0.85823
524	0.82944	0.83823	0.84237	0.84375	0.84824	0.85307	0.85843
525	0.82972	0.83847	0.84260	0.84398	0.84846	0.85328	0.85864
526	0.82995	0.83870	0.84282	0.84421	0.84868	0.85349	0.85884
527	0.83020	0.83893	0.84305	0.84443	0.84889	0.85370	0.85904
528	0.83044	0.83917	0.84327	0.84464	0.84911	0.85391	0.85924
529	0.83068	0.83940	0.84350	0.84487	0.84933	0.85412	0.85944
530	0.83093	0.83964	0.84373	0.84509	0.84954	0.85433	0.85964
531	0.83118	0.83988	0.84395	0.84531	0.84976	0.85453	0.85984
532	0.83142	0.84010	0.84418	0.84554	0.84998	0.85474	0.86004
533	0.83166	0.84033	0.84440	0.84577	0.85019	0.85495	0.86024
534	0.83190	0.84056	0.84462	0.84599	0.85040	0.85516	0.86044
535	0.83215	0.84079	0.84484	0.84620	0.85062	0.85536	0.86063
536	0.83239	0.84103	0.84506	0.84643	0.85083	0.85557	0.86083
537	0.83263	0.84126	0.84528	0.84665	0.85105	0.85577	0.86102
538	0.83285	0.84149	0.84550	0.84686	0.85126	0.85597	0.86122
539	0.83310	0.84172	0.84572	0.84708	0.85147	0.85618	0.86142
540	0.83334	0.84194	0.84594	0.84729	0.85168	0.85638	0.86161
541	0.83355	0.84217	0.84615	0.84751	0.85188	0.85659	0.86180
542	0.83377	0.84239	0.84638	0.84772	0.85209	0.85678	0.86200
543	0.83400	0.84261	0.84659	0.84793	0.85230	0.85699	0.86219
544	0.83424	0.84283	0.84681	0.84815	0.85250	0.85718	0.86238
545	0.83448	0.84306	0.84702	0.84837	0.85271	0.85738	0.86258
546	0.83472	0.84328	0.84723	0.84858	0.85291	0.85758	0.86277
547	0.83494	0.84350	0.84745	0.84879	0.85312	0.85778	0.86296
548	0.83518	0.84371	0.84766	0.84900	0.85332	0.85797	0.86314
549	0.83538	0.84393	0.84787	0.84921	0.85352	0.85817	0.86333
550	0.83562	0.84415	0.84809	0.84942	0.85373	0.85837	0.86352
551	0.83586	0.84437	0.84830	0.84963	0.85393	0.85856	0.86371
552	0.83608	0.84459	0.84851	0.84984	0.85413	0.85876	0.86390
553	0.83633	0.84480	0.84872	0.85005	0.85433	0.85895	0.86408
554	0.83656	0.84501	0.84893	0.85026	0.85453	0.85914	0.86427
555	0.83677	0.84522	0.84914	0.85047	0.85474	0.85934	0.86445
556	0.83701	0.84544	0.84935	0.85067	0.85493	0.85953	0.86464
557	0.83724	0.84565	0.84956	0.85087	0.85513	0.85972	0.86482
558	0.83746	0.84586	0.84976	0.85108	0.85533	0.85991	0.86501
559	0.83771	0.84608	0.84997	0.85128	0.85552	0.86010	0.86519

N	0.1%	0.5%	1%	1.25%	2.5%	5%	10%
560	0.83793	0.84630	0.85018	0.85148	0.85572	0.86029	0.86537
561	0.83815	0.84651	0.85038	0.85168	0.85592	0.86049	0.86556
562	0.83837	0.84673	0.85059	0.85189	0.85611	0.86068	0.86574
563	0.83861	0.84694	0.85079	0.85209	0.85631	0.86087	0.86592
564	0.83882	0.84715	0.85100	0.85230	0.85651	0.86106	0.86610
565	0.83904	0.84736	0.85121	0.85249	0.85670	0.86124	0.86628
566	0.83926	0.84757	0.85141	0.85270	0.85690	0.86143	0.86645
567	0.83948	0.84778	0.85161	0.85290	0.85709	0.86162	0.86663
568	0.83971	0.84798	0.85181	0.85309	0.85728	0.86180	0.86681
569	0.83992	0.84819	0.85201	0.85328	0.85747	0.86199	0.86699
570	0.84014	0.84839	0.85221	0.85348	0.85766	0.86217	0.86717
571	0.84035	0.84860	0.85241	0.85368	0.85785	0.86235	0.86734
572	0.84058	0.84881	0.85260	0.85387	0.85805	0.86254	0.86752
573	0.84078	0.84900	0.85281	0.85407	0.85824	0.86272	0.86769
574	0.84100	0.84920	0.85300	0.85427	0.85842	0.86290	0.86787
575	0.84119	0.84940	0.85320	0.85447	0.85861	0.86308	0.86804
576	0.84141	0.84960	0.85339	0.85467	0.85880	0.86327	0.86822
577	0.84164	0.84981	0.85359	0.85486	0.85899	0.86345	0.86839
578	0.84186	0.85001	0.85378	0.85505	0.85917	0.86363	0.86856
579	0.84206	0.85020	0.85398	0.85524	0.85936	0.86381	0.86874
580	0.84228	0.85040	0.85417	0.85543	0.85955	0.86399	0.86891
581	0.84248	0.85061	0.85437	0.85563	0.85973	0.86416	0.86908
582	0.84270	0.85080	0.85456	0.85582	0.85992	0.86434	0.86925
583	0.84289	0.85100	0.85475	0.85601	0.86010	0.86452	0.86942
584	0.84309	0.85120	0.85494	0.85620	0.86028	0.86470	0.86959
585	0.84330	0.85140	0.85513	0.85639	0.86046	0.86487	0.86976
586	0.84350	0.85160	0.85532	0.85657	0.86064	0.86505	0.86993
587	0.84372	0.85179	0.85552	0.85676	0.86082	0.86523	0.87010
588	0.84394	0.85199	0.85571	0.85695	0.86101	0.86540	0.87026
589	0.84415	0.85218	0.85589	0.85713	0.86119	0.86557	0.87043
590	0.84437	0.85237	0.85608	0.85732	0.86137	0.86575	0.87060
591	0.84455	0.85257	0.85627	0.85751	0.86155	0.86592	0.87076
592	0.84476	0.85276	0.85646	0.85769	0.86173	0.86610	0.87093
593	0.84496	0.85294	0.85665	0.85788	0.86191	0.86627	0.87110
594	0.84516	0.85314	0.85683	0.85806	0.86209	0.86644	0.87126
595	0.84536	0.85333	0.85701	0.85825	0.86227	0.86661	0.87142
596	0.84556	0.85352	0.85720	0.85843	0.86244	0.86678	0.87159
597	0.84575	0.85371	0.85739	0.85862	0.86262	0.86695	0.87175
598	0.84596	0.85390	0.85758	0.85880	0.86280	0.86712	0.87191
599	0.84616	0.85409	0.85776	0.85898	0.86297	0.86729	0.87208
600	0.84636	0.85427	0.85795	0.85916	0.86315	0.86746	0.87224
601	0.84656	0.85446	0.85813	0.85934	0.86332	0.86763	0.87240
602	0.84676	0.85465	0.85831	0.85952	0.86350	0.86780	0.87256
603	0.84695	0.85485	0.85850	0.85970	0.86367	0.86796	0.87272
604	0.84713	0.85503	0.85867	0.85988	0.86384	0.86813	0.87289
605	0.84732	0.85521	0.85885	0.86007	0.86402	0.86830	0.87305
606	0.84750	0.85540	0.85903	0.86024	0.86419	0.86846	0.87321
607	0.84770	0.85558	0.85922	0.86042	0.86436	0.86863	0.87337
608	0.84790	0.85577	0.85940	0.86059	0.86453	0.86880	0.87352
609	0.84809	0.85595	0.85957	0.86077	0.86470	0.86896	0.87368
610	0.84829	0.85613	0.85975	0.86094	0.86488	0.86913	0.87384
611	0.84848	0.85631	0.85993	0.86112	0.86505	0.86929	0.87400
612	0.84867	0.85650	0.86010	0.86130	0.86522	0.86946	0.87416
613	0.84887	0.85668	0.86028	0.86148	0.86538	0.86962	0.87431
614	0.84906	0.85686	0.86045	0.86165	0.86555	0.86978	0.87447
615	0.84925	0.85704	0.86063	0.86183	0.86572	0.86994	0.87462
616	0.84944	0.85722	0.86080	0.86200	0.86589	0.87011	0.87478
617	0.84964	0.85740	0.86098	0.86217	0.86606	0.87027	0.87493
618	0.84983	0.85758	0.86115	0.86235	0.86622	0.87043	0.87509
619	0.85002	0.85776	0.86133	0.86252	0.86639	0.87059	0.87524
620	0.85021	0.85793	0.86150	0.86269	0.86656	0.87075	0.87539
621	0.85040	0.85811	0.86168	0.86287	0.86672	0.87091	0.87555
622	0.85060	0.85829	0.86185	0.86303	0.86688	0.87107	0.87570
623	0.85078	0.85847	0.86202	0.86320	0.86704	0.87123	0.87585
624	0.85097	0.85865	0.86219	0.86337	0.86721	0.87138	0.87600
625	0.85115	0.85882	0.86236	0.86354	0.86737	0.87154	0.87616
626	0.85134	0.85900	0.86253	0.86371	0.86754	0.87170	0.87630
627	0.85151	0.85917	0.86270	0.86388	0.86770	0.87186	0.87646
628	0.85170	0.85935	0.86287	0.86405	0.86786	0.87202	0.87661
629	0.85190	0.85952	0.86303	0.86422	0.86803	0.87217	0.87676
630	0.85207	0.85970	0.86320	0.86438	0.86819	0.87233	0.87691
631	0.85225	0.85986	0.86337	0.86454	0.86835	0.87248	0.87706
632	0.85243	0.86003	0.86353	0.86471	0.86851	0.87264	0.87720
633	0.85261	0.86020	0.86370	0.86488	0.86867	0.87279	0.87735

N	0.1%	0.5%	1%	1.25%	2.5%	5%	10%
634	0.85280	0.86037	0.86387	0.86504	0.86883	0.87295	0.87750
635	0.85299	0.86054	0.86403	0.86521	0.86899	0.87310	0.87765
636	0.85316	0.86071	0.86420	0.86537	0.86915	0.87325	0.87780
637	0.85335	0.86088	0.86436	0.86553	0.86931	0.87340	0.87794
638	0.85353	0.86105	0.86453	0.86570	0.86946	0.87356	0.87809
639	0.85371	0.86122	0.86470	0.86586	0.86962	0.87371	0.87824
640	0.85388	0.86140	0.86486	0.86602	0.86978	0.87386	0.87838
641	0.85404	0.86155	0.86502	0.86618	0.86993	0.87401	0.87852
642	0.85423	0.86172	0.86519	0.86634	0.87009	0.87416	0.87867
643	0.85440	0.86190	0.86535	0.86650	0.87024	0.87431	0.87881
644	0.85457	0.86206	0.86551	0.86666	0.87040	0.87446	0.87896
645	0.85474	0.86222	0.86567	0.86681	0.87056	0.87461	0.87910
646	0.85490	0.86238	0.86583	0.86698	0.87071	0.87476	0.87924
647	0.85509	0.86255	0.86598	0.86714	0.87086	0.87491	0.87939
648	0.85527	0.86271	0.86614	0.86730	0.87102	0.87506	0.87953
649	0.85547	0.86287	0.86630	0.86746	0.87118	0.87520	0.87967
650	0.85564	0.86304	0.86647	0.86761	0.87133	0.87535	0.87981
651	0.85583	0.86320	0.86663	0.86777	0.87148	0.87550	0.87995
652	0.85598	0.86337	0.86679	0.86793	0.87163	0.87565	0.88010
653	0.85616	0.86353	0.86695	0.86808	0.87179	0.87579	0.88024
654	0.85633	0.86368	0.86711	0.86824	0.87194	0.87594	0.88037
655	0.85650	0.86385	0.86726	0.86839	0.87209	0.87609	0.88052
656	0.85668	0.86402	0.86742	0.86855	0.87224	0.87623	0.88065
657	0.85686	0.86418	0.86757	0.86871	0.87239	0.87638	0.88079
658	0.85701	0.86434	0.86773	0.86886	0.87254	0.87652	0.88093
659	0.85718	0.86450	0.86789	0.86902	0.87269	0.87667	0.88107
660	0.85736	0.86465	0.86804	0.86917	0.87283	0.87681	0.88121
661	0.85753	0.86481	0.86820	0.86932	0.87298	0.87695	0.88135
662	0.85770	0.86496	0.86835	0.86947	0.87313	0.87710	0.88149
663	0.85786	0.86513	0.86851	0.86963	0.87327	0.87724	0.88162
664	0.85803	0.86529	0.86866	0.86978	0.87343	0.87738	0.88176
665	0.85818	0.86545	0.86882	0.86993	0.87357	0.87752	0.88190
666	0.85835	0.86561	0.86897	0.87007	0.87372	0.87767	0.88203
667	0.85851	0.86577	0.86912	0.87022	0.87386	0.87781	0.88217
668	0.85868	0.86592	0.86927	0.87038	0.87401	0.87795	0.88230
669	0.85884	0.86608	0.86942	0.87053	0.87415	0.87809	0.88244
670	0.85901	0.86623	0.86958	0.87068	0.87429	0.87823	0.88257
671	0.85919	0.86639	0.86973	0.87083	0.87444	0.87837	0.88271
672	0.85935	0.86654	0.86988	0.87098	0.87459	0.87850	0.88284
673	0.85951	0.86669	0.87003	0.87114	0.87474	0.87864	0.88298
674	0.85967	0.86685	0.87018	0.87128	0.87488	0.87878	0.88311
675	0.85983	0.86701	0.87033	0.87143	0.87502	0.87892	0.88324
676	0.85998	0.86716	0.87048	0.87158	0.87517	0.87906	0.88337
677	0.86015	0.86731	0.87063	0.87173	0.87531	0.87920	0.88350
678	0.86032	0.86747	0.87077	0.87188	0.87545	0.87934	0.88364
679	0.86049	0.86763	0.87093	0.87202	0.87559	0.87947	0.88377
680	0.86064	0.86778	0.87107	0.87217	0.87573	0.87961	0.88390
681	0.86079	0.86793	0.87122	0.87232	0.87588	0.87974	0.88403
682	0.86095	0.86808	0.87136	0.87246	0.87602	0.87988	0.88416
683	0.86110	0.86824	0.87151	0.87261	0.87616	0.88002	0.88429
684	0.86126	0.86838	0.87165	0.87275	0.87630	0.88016	0.88442
685	0.86144	0.86854	0.87180	0.87290	0.87644	0.88029	0.88455
686	0.86161	0.86868	0.87194	0.87304	0.87658	0.88042	0.88468
687	0.86177	0.86883	0.87208	0.87318	0.87671	0.88056	0.88481
688	0.86195	0.86897	0.87223	0.87332	0.87685	0.88069	0.88494
689	0.86210	0.86912	0.87237	0.87347	0.87699	0.88082	0.88507
690	0.86225	0.86927	0.87251	0.87361	0.87713	0.88095	0.88519
691	0.86240	0.86942	0.87266	0.87375	0.87727	0.88109	0.88532
692	0.86257	0.86957	0.87280	0.87390	0.87741	0.88122	0.88545
693	0.86272	0.86971	0.87295	0.87405	0.87755	0.88136	0.88558
694	0.86288	0.86986	0.87309	0.87418	0.87768	0.88149	0.88570
695	0.86303	0.87000	0.87323	0.87432	0.87782	0.88162	0.88583
696	0.86318	0.87015	0.87337	0.87446	0.87796	0.88175	0.88596
697	0.86333	0.87029	0.87351	0.87460	0.87809	0.88188	0.88608
698	0.86348	0.87045	0.87366	0.87474	0.87823	0.88201	0.88621
699	0.86363	0.87059	0.87380	0.87488	0.87836	0.88214	0.88633
700	0.86379	0.87073	0.87394	0.87502	0.87850	0.88227	0.88646
701	0.86394	0.87087	0.87407	0.87516	0.87863	0.88240	0.88658
702	0.86409	0.87102	0.87422	0.87530	0.87877	0.88253	0.88671
703	0.86425	0.87117	0.87436	0.87544	0.87890	0.88266	0.88683
704	0.86441	0.87131	0.87450	0.87558	0.87903	0.88279	0.88695
705	0.86456	0.87146	0.87464	0.87572	0.87917	0.88292	0.88708
706	0.86471	0.87160	0.87478	0.87585	0.87930	0.88305	0.88720
707	0.86484	0.87174	0.87492	0.87598	0.87943	0.88318	0.88732

N	0.1%	0.5%	1%	1.25%	2.5%	5%	10%
708	0.86501	0.87188	0.87506	0.87612	0.87956	0.88330	0.88744
709	0.86515	0.87202	0.87519	0.87626	0.87970	0.88343	0.88757
710	0.86531	0.87216	0.87533	0.87639	0.87983	0.88356	0.88769
711	0.86545	0.87230	0.87546	0.87653	0.87996	0.88368	0.88781
712	0.86561	0.87245	0.87560	0.87666	0.88009	0.88381	0.88793
713	0.86577	0.87258	0.87574	0.87680	0.88022	0.88394	0.88805
714	0.86591	0.87272	0.87587	0.87693	0.88035	0.88406	0.88817
715	0.86605	0.87285	0.87601	0.87707	0.88048	0.88419	0.88830
716	0.86620	0.87299	0.87614	0.87720	0.88061	0.88431	0.88841
717	0.86635	0.87313	0.87628	0.87733	0.88074	0.88444	0.88853
718	0.86649	0.87327	0.87641	0.87746	0.88087	0.88456	0.88865
719	0.86665	0.87341	0.87654	0.87760	0.88100	0.88469	0.88877
720	0.86678	0.87355	0.87668	0.87773	0.88113	0.88481	0.88889
721	0.86692	0.87369	0.87682	0.87786	0.88126	0.88493	0.88901
722	0.86707	0.87382	0.87695	0.87799	0.88138	0.88506	0.88913
723	0.86720	0.87395	0.87708	0.87812	0.88151	0.88518	0.88925
724	0.86735	0.87409	0.87721	0.87825	0.88164	0.88530	0.88936
725	0.86751	0.87422	0.87735	0.87839	0.88177	0.88543	0.88948
726	0.86765	0.87437	0.87748	0.87852	0.88189	0.88555	0.88960
727	0.86779	0.87450	0.87761	0.87865	0.88202	0.88567	0.88972
728	0.86794	0.87464	0.87774	0.87878	0.88214	0.88579	0.88983
729	0.86808	0.87477	0.87788	0.87890	0.88226	0.88591	0.88995
730	0.86824	0.87490	0.87801	0.87904	0.88239	0.88603	0.89006
731	0.86839	0.87504	0.87814	0.87917	0.88252	0.88616	0.89018
732	0.86852	0.87517	0.87827	0.87930	0.88264	0.88628	0.89030
733	0.86867	0.87532	0.87841	0.87943	0.88277	0.88640	0.89041
734	0.86880	0.87545	0.87853	0.87956	0.88289	0.88652	0.89053
735	0.86892	0.87559	0.87867	0.87969	0.88301	0.88664	0.89064
736	0.86905	0.87572	0.87880	0.87982	0.88314	0.88676	0.89076
737	0.86919	0.87585	0.87892	0.87994	0.88326	0.88687	0.89087
738	0.86934	0.87598	0.87905	0.88007	0.88339	0.88699	0.89099
739	0.86948	0.87611	0.87918	0.88020	0.88351	0.88711	0.89110
740	0.86961	0.87624	0.87931	0.88032	0.88363	0.88723	0.89122
741	0.86975	0.87636	0.87944	0.88045	0.88376	0.88735	0.89133
742	0.86990	0.87650	0.87956	0.88057	0.88388	0.88746	0.89144
743	0.87004	0.87663	0.87969	0.88070	0.88400	0.88758	0.89156
744	0.87017	0.87677	0.87982	0.88082	0.88412	0.88770	0.89167
745	0.87030	0.87690	0.87995	0.88095	0.88424	0.88782	0.89178
746	0.87045	0.87703	0.88008	0.88108	0.88437	0.88793	0.89189
747	0.87059	0.87716	0.88019	0.88120	0.88449	0.88805	0.89200
748	0.87073	0.87729	0.88032	0.88133	0.88461	0.88816	0.89211
749	0.87088	0.87743	0.88045	0.88145	0.88473	0.88828	0.89223
750	0.87102	0.87755	0.88057	0.88157	0.88485	0.88840	0.89234
751	0.87115	0.87767	0.88069	0.88170	0.88497	0.88851	0.89245
752	0.87128	0.87780	0.88082	0.88182	0.88509	0.88863	0.89256
753	0.87143	0.87793	0.88094	0.88195	0.88521	0.88874	0.89267
754	0.87157	0.87806	0.88107	0.88207	0.88532	0.88886	0.89278
755	0.87172	0.87819	0.88119	0.88220	0.88545	0.88897	0.89289
756	0.87184	0.87831	0.88131	0.88232	0.88557	0.88909	0.89300
757	0.87199	0.87843	0.88144	0.88244	0.88568	0.88920	0.89311
758	0.87211	0.87855	0.88156	0.88256	0.88580	0.88932	0.89322
759	0.87224	0.87868	0.88168	0.88269	0.88592	0.88943	0.89332
760	0.87236	0.87881	0.88181	0.88281	0.88603	0.88954	0.89343
761	0.87251	0.87893	0.88193	0.88292	0.88615	0.88966	0.89354
762	0.87263	0.87906	0.88205	0.88304	0.88627	0.88977	0.89365
763	0.87277	0.87918	0.88217	0.88316	0.88638	0.88988	0.89376
764	0.87289	0.87930	0.88228	0.88328	0.88650	0.88999	0.89386
765	0.87303	0.87943	0.88240	0.88340	0.88662	0.89010	0.89397
766	0.87315	0.87955	0.88252	0.88352	0.88673	0.89022	0.89408
767	0.87328	0.87968	0.88264	0.88363	0.88684	0.89032	0.89419
768	0.87341	0.87981	0.88276	0.88375	0.88696	0.89044	0.89429
769	0.87353	0.87993	0.88288	0.88387	0.88708	0.89055	0.89440
770	0.87366	0.88005	0.88300	0.88399	0.88719	0.89066	0.89451
771	0.87380	0.88018	0.88312	0.88410	0.88731	0.89077	0.89461
772	0.87392	0.88030	0.88324	0.88422	0.88742	0.89088	0.89472
773	0.87406	0.88042	0.88335	0.88434	0.88753	0.89099	0.89482
774	0.87418	0.88055	0.88347	0.88446	0.88765	0.89109	0.89493
775	0.87432	0.88067	0.88360	0.88457	0.88776	0.89120	0.89503
776	0.87443	0.88079	0.88371	0.88469	0.88787	0.89131	0.89514
777	0.87456	0.88090	0.88383	0.88481	0.88799	0.89142	0.89524
778	0.87468	0.88103	0.88395	0.88492	0.88810	0.89153	0.89535
779	0.87482	0.88115	0.88406	0.88504	0.88821	0.89164	0.89545
780	0.87496	0.88126	0.88418	0.88516	0.88832	0.89174	0.89555
781	0.87510	0.88139	0.88429	0.88527	0.88843	0.89185	0.89566

N	0.1%	0.5%	1%	1.25%	2.5%	5%	10%
782	0.87522	0.88150	0.88441	0.88538	0.88854	0.89196	0.89576
783	0.87536	0.88162	0.88452	0.88550	0.88866	0.89207	0.89587
784	0.87547	0.88174	0.88464	0.88562	0.88877	0.89217	0.89597
785	0.87559	0.88186	0.88476	0.88573	0.88888	0.89228	0.89607
786	0.87570	0.88198	0.88487	0.88584	0.88899	0.89239	0.89617
787	0.87584	0.88210	0.88499	0.88596	0.88910	0.89249	0.89628
788	0.87596	0.88222	0.88510	0.88607	0.88921	0.89260	0.89638
789	0.87609	0.88233	0.88521	0.88619	0.88931	0.89270	0.89648
790	0.87622	0.88245	0.88532	0.88630	0.88942	0.89281	0.89658
791	0.87633	0.88256	0.88543	0.88641	0.88953	0.89292	0.89668
792	0.87646	0.88268	0.88555	0.88652	0.88964	0.89302	0.89678
793	0.87658	0.88279	0.88567	0.88664	0.88975	0.89313	0.89688
794	0.87671	0.88291	0.88578	0.88675	0.88986	0.89323	0.89698
795	0.87684	0.88302	0.88589	0.88686	0.88997	0.89334	0.89708
796	0.87696	0.88314	0.88599	0.88697	0.89008	0.89344	0.89719
797	0.87707	0.88326	0.88611	0.88708	0.89018	0.89355	0.89729
798	0.87719	0.88338	0.88622	0.88719	0.89029	0.89365	0.89739
799	0.87731	0.88349	0.88633	0.88730	0.89040	0.89375	0.89748
800	0.87743	0.88360	0.88645	0.88742	0.89050	0.89386	0.89758
801	0.87755	0.88372	0.88656	0.88752	0.89061	0.89396	0.89768
802	0.87767	0.88383	0.88667	0.88764	0.89072	0.89407	0.89778
803	0.87779	0.88394	0.88678	0.88774	0.89082	0.89417	0.89788
804	0.87791	0.88406	0.88689	0.88785	0.89093	0.89427	0.89798
805	0.87803	0.88417	0.88701	0.88796	0.89104	0.89438	0.89807
806	0.87814	0.88429	0.88712	0.88807	0.89114	0.89448	0.89817
807	0.87826	0.88440	0.88723	0.88818	0.89125	0.89458	0.89827
808	0.87838	0.88451	0.88734	0.88829	0.89136	0.89468	0.89837
809	0.87850	0.88463	0.88744	0.88840	0.89146	0.89478	0.89846
810	0.87862	0.88474	0.88756	0.88850	0.89157	0.89489	0.89856
811	0.87873	0.88485	0.88767	0.88861	0.89167	0.89499	0.89866
812	0.87884	0.88497	0.88778	0.88872	0.89178	0.89509	0.89876
813	0.87897	0.88507	0.88789	0.88883	0.89188	0.89519	0.89885
814	0.87908	0.88518	0.88800	0.88894	0.89198	0.89529	0.89895
815	0.87920	0.88530	0.88810	0.88904	0.89209	0.89539	0.89905
816	0.87933	0.88541	0.88821	0.88915	0.89219	0.89549	0.89914
817	0.87944	0.88552	0.88832	0.88925	0.89230	0.89559	0.89924
818	0.87958	0.88563	0.88843	0.88936	0.89240	0.89568	0.89933
819	0.87969	0.88574	0.88854	0.88947	0.89250	0.89578	0.89943
820	0.87981	0.88585	0.88865	0.88958	0.89261	0.89588	0.89952
821	0.87993	0.88596	0.88875	0.88968	0.89271	0.89598	0.89962
822	0.88004	0.88606	0.88886	0.88979	0.89281	0.89608	0.89971
823	0.88015	0.88617	0.88896	0.88989	0.89291	0.89618	0.89981
824	0.88028	0.88628	0.88907	0.89000	0.89301	0.89628	0.89990
825	0.88039	0.88640	0.88917	0.89010	0.89311	0.89637	0.90000
826	0.88049	0.88651	0.88928	0.89021	0.89321	0.89647	0.90009
827	0.88061	0.88661	0.88939	0.89031	0.89332	0.89657	0.90018
828	0.88073	0.88672	0.88949	0.89042	0.89342	0.89667	0.90028
829	0.88083	0.88683	0.88959	0.89052	0.89352	0.89677	0.90037
830	0.88096	0.88694	0.88970	0.89063	0.89362	0.89686	0.90046
831	0.88108	0.88704	0.88980	0.89073	0.89372	0.89696	0.90055
832	0.88118	0.88715	0.88991	0.89083	0.89382	0.89706	0.90065
833	0.88130	0.88725	0.89001	0.89094	0.89392	0.89716	0.90074
834	0.88140	0.88736	0.89011	0.89104	0.89402	0.89725	0.90083
835	0.88152	0.88747	0.89022	0.89115	0.89412	0.89735	0.90093
836	0.88163	0.88758	0.89032	0.89125	0.89422	0.89744	0.90102
837	0.88175	0.88768	0.89043	0.89135	0.89432	0.89754	0.90111
838	0.88186	0.88779	0.89053	0.89145	0.89442	0.89763	0.90120
839	0.88198	0.88790	0.89063	0.89156	0.89452	0.89773	0.90129
840	0.88209	0.88800	0.89073	0.89166	0.89461	0.89782	0.90138
841	0.88220	0.88811	0.89084	0.89176	0.89472	0.89792	0.90148
842	0.88232	0.88822	0.89094	0.89186	0.89481	0.89801	0.90157
843	0.88243	0.88832	0.89104	0.89196	0.89491	0.89811	0.90166
844	0.88254	0.88843	0.89114	0.89206	0.89501	0.89820	0.90175
845	0.88264	0.88853	0.89124	0.89216	0.89511	0.89830	0.90184
846	0.88276	0.88863	0.89134	0.89226	0.89520	0.89839	0.90193
847	0.88286	0.88873	0.89145	0.89236	0.89530	0.89849	0.90202
848	0.88298	0.88883	0.89155	0.89246	0.89539	0.89858	0.90211
849	0.88309	0.88894	0.89164	0.89255	0.89549	0.89867	0.90220
850	0.88319	0.88904	0.89174	0.89265	0.89559	0.89877	0.90229
851	0.88329	0.88914	0.89185	0.89275	0.89569	0.89886	0.90238
852	0.88340	0.88925	0.89195	0.89285	0.89578	0.89895	0.90247
853	0.88350	0.88935	0.89205	0.89295	0.89588	0.89905	0.90255
854	0.88361	0.88945	0.89215	0.89305	0.89598	0.89914	0.90264
855	0.88373	0.88955	0.89225	0.89315	0.89607	0.89923	0.90273

N	0.1%	0.5%	1%	1.25%	2.5%	5%	10%
856	0.88383	0.88966	0.89235	0.89325	0.89617	0.89932	0.90282
857	0.88394	0.88976	0.89245	0.89335	0.89626	0.89941	0.90291
858	0.88405	0.88986	0.89255	0.89345	0.89636	0.89951	0.90300
859	0.88415	0.88996	0.89265	0.89354	0.89645	0.89960	0.90309
860	0.88426	0.89006	0.89275	0.89364	0.89654	0.89969	0.90317
861	0.88436	0.89017	0.89285	0.89374	0.89664	0.89978	0.90326
862	0.88447	0.89027	0.89295	0.89384	0.89674	0.89987	0.90335
863	0.88458	0.89038	0.89304	0.89394	0.89683	0.89996	0.90344
864	0.88468	0.89047	0.89314	0.89403	0.89692	0.90005	0.90352
865	0.88479	0.89057	0.89324	0.89413	0.89702	0.90014	0.90361
866	0.88489	0.89067	0.89334	0.89423	0.89711	0.90023	0.90370
867	0.88500	0.89077	0.89343	0.89432	0.89720	0.90033	0.90378
868	0.88510	0.89087	0.89353	0.89442	0.89730	0.90042	0.90387
869	0.88521	0.89097	0.89363	0.89452	0.89739	0.90051	0.90396
870	0.88532	0.89107	0.89373	0.89461	0.89748	0.90060	0.90404
871	0.88543	0.89116	0.89382	0.89471	0.89758	0.90068	0.90413
872	0.88554	0.89127	0.89392	0.89480	0.89767	0.90077	0.90422
873	0.88564	0.89137	0.89402	0.89490	0.89776	0.90086	0.90430
874	0.88575	0.89147	0.89411	0.89499	0.89785	0.90095	0.90439
875	0.88585	0.89157	0.89421	0.89509	0.89794	0.90104	0.90447
876	0.88595	0.89166	0.89430	0.89518	0.89804	0.90113	0.90456
877	0.88605	0.89176	0.89440	0.89527	0.89813	0.90122	0.90464
878	0.88615	0.89185	0.89449	0.89537	0.89822	0.90130	0.90473
879	0.88625	0.89195	0.89459	0.89547	0.89831	0.90139	0.90481
880	0.88636	0.89205	0.89468	0.89556	0.89840	0.90148	0.90490
881	0.88647	0.89215	0.89478	0.89566	0.89849	0.90157	0.90498
882	0.88658	0.89224	0.89487	0.89575	0.89858	0.90166	0.90507
883	0.88669	0.89234	0.89496	0.89584	0.89867	0.90174	0.90515
884	0.88679	0.89243	0.89506	0.89593	0.89876	0.90183	0.90523
885	0.88689	0.89253	0.89515	0.89602	0.89886	0.90192	0.90532
886	0.88699	0.89263	0.89525	0.89612	0.89894	0.90200	0.90540
887	0.88708	0.89273	0.89534	0.89621	0.89904	0.90209	0.90548
888	0.88719	0.89282	0.89543	0.89630	0.89913	0.90218	0.90557
889	0.88729	0.89292	0.89552	0.89640	0.89921	0.90227	0.90565
890	0.88738	0.89301	0.89562	0.89649	0.89931	0.90235	0.90573
891	0.88748	0.89311	0.89571	0.89658	0.89939	0.90244	0.90582
892	0.88757	0.89320	0.89581	0.89667	0.89948	0.90253	0.90590
893	0.88767	0.89329	0.89590	0.89676	0.89957	0.90261	0.90598
894	0.88778	0.89339	0.89599	0.89685	0.89966	0.90270	0.90606
895	0.88787	0.89348	0.89608	0.89694	0.89975	0.90278	0.90614
896	0.88798	0.89358	0.89617	0.89703	0.89984	0.90287	0.90623
897	0.88808	0.89367	0.89626	0.89713	0.89993	0.90295	0.90631
898	0.88819	0.89376	0.89635	0.89722	0.90001	0.90304	0.90639
899	0.88828	0.89386	0.89644	0.89731	0.90010	0.90312	0.90647
900	0.88838	0.89395	0.89654	0.89740	0.90019	0.90321	0.90655
901	0.88849	0.89404	0.89663	0.89749	0.90027	0.90329	0.90663
902	0.88858	0.89414	0.89672	0.89758	0.90036	0.90338	0.90671
903	0.88867	0.89423	0.89681	0.89767	0.90045	0.90346	0.90680
904	0.88878	0.89433	0.89690	0.89776	0.90054	0.90354	0.90687
905	0.88887	0.89442	0.89699	0.89785	0.90063	0.90363	0.90696
906	0.88898	0.89452	0.89708	0.89794	0.90072	0.90371	0.90704
907	0.88907	0.89461	0.89717	0.89803	0.90080	0.90379	0.90711
908	0.88917	0.89470	0.89726	0.89812	0.90088	0.90388	0.90719
909	0.88926	0.89479	0.89735	0.89821	0.90097	0.90396	0.90727
910	0.88936	0.89488	0.89744	0.89830	0.90106	0.90404	0.90735
911	0.88945	0.89497	0.89753	0.89839	0.90114	0.90413	0.90743
912	0.88955	0.89507	0.89762	0.89848	0.90123	0.90421	0.90751
913	0.88965	0.89516	0.89771	0.89856	0.90131	0.90429	0.90759
914	0.88975	0.89525	0.89780	0.89865	0.90140	0.90437	0.90767
915	0.88984	0.89534	0.89789	0.89874	0.90148	0.90445	0.90775
916	0.88994	0.89543	0.89798	0.89883	0.90157	0.90454	0.90783
917	0.89004	0.89552	0.89806	0.89891	0.90165	0.90462	0.90791
918	0.89013	0.89561	0.89815	0.89900	0.90173	0.90470	0.90798
919	0.89022	0.89570	0.89824	0.89908	0.90182	0.90478	0.90806
920	0.89032	0.89579	0.89832	0.89917	0.90190	0.90486	0.90814
921	0.89042	0.89588	0.89841	0.89926	0.90199	0.90494	0.90822
922	0.89052	0.89597	0.89850	0.89934	0.90207	0.90503	0.90830
923	0.89061	0.89606	0.89858	0.89943	0.90215	0.90510	0.90838
924	0.89071	0.89615	0.89867	0.89951	0.90224	0.90518	0.90845
925	0.89081	0.89624	0.89876	0.89960	0.90232	0.90526	0.90853
926	0.89090	0.89633	0.89885	0.89969	0.90241	0.90535	0.90861
927	0.89100	0.89642	0.89893	0.89978	0.90249	0.90543	0.90869
928	0.89108	0.89651	0.89902	0.89986	0.90257	0.90551	0.90876
929	0.89116	0.89660	0.89911	0.89995	0.90266	0.90558	0.90884

N	0.1%	0.5%	1%	1.25%	2.5%	5%	10%
930	0.89124	0.89669	0.89919	0.90003	0.90274	0.90566	0.90891
931	0.89135	0.89678	0.89928	0.90012	0.90282	0.90574	0.90899
932	0.89143	0.89686	0.89936	0.90020	0.90290	0.90582	0.90907
933	0.89152	0.89695	0.89945	0.90029	0.90299	0.90590	0.90914
934	0.89163	0.89704	0.89954	0.90037	0.90307	0.90598	0.90922
935	0.89171	0.89713	0.89962	0.90046	0.90315	0.90606	0.90930
936	0.89181	0.89722	0.89971	0.90054	0.90323	0.90614	0.90937
937	0.89190	0.89730	0.89979	0.90063	0.90331	0.90622	0.90945
938	0.89201	0.89739	0.89988	0.90071	0.90339	0.90630	0.90952
939	0.89210	0.89748	0.89997	0.90079	0.90347	0.90638	0.90960
940	0.89219	0.89756	0.90005	0.90088	0.90355	0.90645	0.90967
941	0.89229	0.89766	0.90013	0.90096	0.90364	0.90653	0.90975
942	0.89239	0.89774	0.90022	0.90105	0.90372	0.90661	0.90982
943	0.89248	0.89783	0.90030	0.90113	0.90380	0.90669	0.90990
944	0.89258	0.89792	0.90038	0.90121	0.90388	0.90677	0.90997
945	0.89266	0.89800	0.90047	0.90129	0.90396	0.90684	0.91005
946	0.89275	0.89809	0.90055	0.90138	0.90404	0.90692	0.91012
947	0.89283	0.89817	0.90063	0.90146	0.90412	0.90700	0.91019
948	0.89293	0.89825	0.90072	0.90154	0.90420	0.90708	0.91027
949	0.89302	0.89834	0.90080	0.90163	0.90428	0.90715	0.91034
950	0.89311	0.89843	0.90088	0.90171	0.90436	0.90723	0.91042
951	0.89321	0.89852	0.90096	0.90179	0.90444	0.90731	0.91049
952	0.89330	0.89860	0.90104	0.90187	0.90452	0.90738	0.91056
953	0.89339	0.89868	0.90113	0.90195	0.90460	0.90746	0.91064
954	0.89348	0.89876	0.90121	0.90203	0.90468	0.90754	0.91071
955	0.89356	0.89885	0.90130	0.90211	0.90476	0.90761	0.91078
956	0.89365	0.89894	0.90137	0.90219	0.90483	0.90769	0.91086
957	0.89375	0.89902	0.90145	0.90227	0.90491	0.90777	0.91093
958	0.89383	0.89910	0.90153	0.90235	0.90499	0.90784	0.91100
959	0.89393	0.89919	0.90162	0.90244	0.90507	0.90792	0.91107
960	0.89401	0.89927	0.90170	0.90252	0.90515	0.90800	0.91115
961	0.89410	0.89935	0.90178	0.90259	0.90523	0.90807	0.91122
962	0.89419	0.89944	0.90186	0.90268	0.90530	0.90815	0.91129
963	0.89428	0.89952	0.90194	0.90276	0.90538	0.90822	0.91136
964	0.89437	0.89960	0.90202	0.90284	0.90546	0.90829	0.91144
965	0.89446	0.89968	0.90210	0.90292	0.90554	0.90837	0.91151
966	0.89455	0.89977	0.90218	0.90300	0.90561	0.90844	0.91158
967	0.89464	0.89984	0.90226	0.90308	0.90569	0.90852	0.91165
968	0.89473	0.89993	0.90234	0.90316	0.90577	0.90859	0.91172
969	0.89481	0.90001	0.90242	0.90324	0.90584	0.90867	0.91179
970	0.89490	0.90009	0.90250	0.90332	0.90592	0.90874	0.91186
971	0.89499	0.90017	0.90258	0.90340	0.90600	0.90882	0.91194
972	0.89508	0.90025	0.90266	0.90347	0.90608	0.90889	0.91201
973	0.89516	0.90033	0.90274	0.90355	0.90615	0.90896	0.91208
974	0.89524	0.90042	0.90282	0.90363	0.90623	0.90904	0.91215
975	0.89535	0.90050	0.90290	0.90371	0.90630	0.90911	0.91222
976	0.89544	0.90058	0.90298	0.90378	0.90638	0.90918	0.91229
977	0.89552	0.90066	0.90306	0.90386	0.90645	0.90925	0.91236
978	0.89561	0.90074	0.90314	0.90394	0.90653	0.90933	0.91243
979	0.89569	0.90083	0.90322	0.90402	0.90660	0.90940	0.91250
980	0.89577	0.90091	0.90329	0.90409	0.90668	0.90947	0.91257
981	0.89585	0.90099	0.90337	0.90417	0.90675	0.90955	0.91264
982	0.89593	0.90107	0.90345	0.90425	0.90683	0.90962	0.91271
983	0.89602	0.90115	0.90353	0.90433	0.90690	0.90969	0.91278
984	0.89610	0.90123	0.90361	0.90440	0.90698	0.90977	0.91285
985	0.89619	0.90131	0.90368	0.90448	0.90705	0.90984	0.91292
986	0.89628	0.90139	0.90376	0.90456	0.90713	0.90991	0.91299
987	0.89637	0.90147	0.90384	0.90464	0.90720	0.90998	0.91306
988	0.89646	0.90155	0.90391	0.90471	0.90728	0.91005	0.91313
989	0.89655	0.90163	0.90400	0.90479	0.90735	0.91012	0.91320
990	0.89663	0.90171	0.90407	0.90486	0.90743	0.91019	0.91327
991	0.89671	0.90179	0.90415	0.90494	0.90750	0.91027	0.91334
992	0.89679	0.90187	0.90423	0.90502	0.90757	0.91034	0.91340
993	0.89687	0.90195	0.90431	0.90509	0.90765	0.91041	0.91347
994	0.89696	0.90203	0.90438	0.90517	0.90772	0.91048	0.91354
995	0.89704	0.90210	0.90446	0.90525	0.90780	0.91055	0.91361
996	0.89713	0.90218	0.90453	0.90532	0.90787	0.91062	0.91368
997	0.89721	0.90226	0.90461	0.90540	0.90794	0.91069	0.91375
998	0.89729	0.90234	0.90469	0.90547	0.90802	0.91076	0.91381
999	0.89737	0.90242	0.90476	0.90555	0.90809	0.91083	0.91388
1000	0.89745	0.90249	0.90484	0.90562	0.90816	0.91091	0.91395

Table 3 – *GM10(8H2L or 2H8L)*. Critical values of upper significance levels for the Grubbs multiple-10 outlier test, applicable in situations where the eight highest and two lowest or two highest and eight lowest observations are simultaneously identified as outliers, denoted as *GM10(8H2L)* and *GM10(2H8L)*, respectively.

N	0.1%	0.5%	1%	1.25%	2.5%	5%	10%
12	0.00000	0.00000	0.00000	0.00000	0.00000	0.00001	0.00005
13	0.00001	0.00004	0.00008	0.00010	0.00022	0.00046	0.00099
14	0.00013	0.00039	0.00063	0.00075	0.00125	0.00212	0.00371
15	0.00058	0.00138	0.00204	0.00231	0.00345	0.00525	0.00821
16	0.00157	0.00322	0.00444	0.00494	0.00690	0.00980	0.01425
17	0.00324	0.00598	0.00788	0.00863	0.01150	0.01559	0.02154
18	0.00566	0.00965	0.01228	0.01329	0.01713	0.02239	0.02981
19	0.00878	0.01410	0.01750	0.01879	0.02360	0.03001	0.03883
20	0.01252	0.01927	0.02347	0.02504	0.03077	0.03828	0.04841
21	0.01682	0.02507	0.03006	0.03190	0.03854	0.04706	0.05838
22	0.02175	0.03145	0.03714	0.03924	0.04673	0.05623	0.06865
23	0.02715	0.03823	0.04463	0.04697	0.05527	0.06567	0.07910
24	0.03308	0.04544	0.05248	0.05503	0.06406	0.07528	0.08966
25	0.03921	0.05287	0.06053	0.06330	0.07304	0.08504	0.10021
26	0.04575	0.06055	0.06877	0.07177	0.08217	0.09485	0.11077
27	0.05246	0.06847	0.07721	0.08037	0.09132	0.10463	0.12123
28	0.05943	0.07644	0.08574	0.08901	0.10054	0.11443	0.13162
29	0.06658	0.08453	0.09428	0.09777	0.10979	0.12417	0.14188
30	0.07372	0.09263	0.10284	0.10645	0.11897	0.13380	0.15199
31	0.08102	0.10082	0.11149	0.11522	0.12809	0.14333	0.16201
32	0.08838	0.10901	0.11999	0.12386	0.13714	0.15280	0.17181
33	0.09594	0.11719	0.12849	0.13248	0.14609	0.16208	0.18146
34	0.10353	0.12539	0.13695	0.14101	0.15495	0.17123	0.19094
35	0.11090	0.13352	0.14537	0.14953	0.16375	0.18027	0.20024
36	0.11845	0.14159	0.15371	0.15794	0.17238	0.18914	0.20935
37	0.12588	0.14951	0.16188	0.16621	0.18094	0.19789	0.21827
38	0.13331	0.15740	0.17000	0.17438	0.18926	0.20648	0.22700
39	0.14068	0.16516	0.17797	0.18242	0.19753	0.21489	0.23555
40	0.14795	0.17294	0.18586	0.19036	0.20561	0.22316	0.24393
41	0.15511	0.18054	0.19368	0.19823	0.21357	0.23126	0.25212
42	0.16223	0.18807	0.20137	0.20597	0.22143	0.23917	0.26015
43	0.16933	0.19556	0.20893	0.21358	0.22909	0.24698	0.26801
44	0.17655	0.20286	0.21638	0.22102	0.23669	0.25462	0.27567
45	0.18336	0.21008	0.22365	0.22838	0.24411	0.26207	0.28324
46	0.19024	0.21716	0.23080	0.23555	0.25139	0.26945	0.29059
47	0.19683	0.22410	0.23785	0.24260	0.25854	0.27661	0.29780
48	0.20346	0.23103	0.24484	0.24956	0.26560	0.28365	0.30483
49	0.21007	0.23785	0.25160	0.25639	0.27245	0.29057	0.31174
50	0.21669	0.24452	0.25832	0.26308	0.27919	0.29736	0.31851
51	0.22324	0.25111	0.26497	0.26979	0.28584	0.30396	0.32514
52	0.22945	0.25758	0.27145	0.27626	0.29235	0.31052	0.33162
53	0.23569	0.26388	0.27783	0.28262	0.29871	0.31691	0.33797
54	0.24195	0.27008	0.28404	0.28888	0.30498	0.32317	0.34420
55	0.24800	0.27632	0.29024	0.29508	0.31117	0.32933	0.35030
56	0.25387	0.28235	0.29629	0.30111	0.31722	0.33535	0.35630
57	0.25965	0.28825	0.30230	0.30710	0.32321	0.34127	0.36217
58	0.26553	0.29410	0.30812	0.31298	0.32904	0.34706	0.36791
59	0.27129	0.29990	0.31395	0.31874	0.33478	0.35275	0.37355
60	0.27702	0.30554	0.31959	0.32438	0.34038	0.35833	0.37905
61	0.28242	0.31114	0.32516	0.32996	0.34590	0.36384	0.38448
62	0.28781	0.31659	0.33064	0.33543	0.35135	0.36924	0.38980
63	0.29327	0.32199	0.33601	0.34080	0.35671	0.37452	0.39500
64	0.29857	0.32724	0.34128	0.34603	0.36192	0.37972	0.40012
65	0.30387	0.33254	0.34644	0.35122	0.36709	0.38481	0.40512
66	0.30900	0.33769	0.35154	0.35637	0.37215	0.38980	0.41005
67	0.31410	0.34273	0.35665	0.36138	0.37711	0.39473	0.41490
68	0.31910	0.34775	0.36158	0.36630	0.38202	0.39953	0.41963
69	0.32410	0.35265	0.36642	0.37115	0.38682	0.40431	0.42430
70	0.32902	0.35742	0.37132	0.37602	0.39159	0.40898	0.42888
71	0.33370	0.36224	0.37604	0.38073	0.39625	0.41358	0.43338
72	0.33841	0.36689	0.38063	0.38532	0.40081	0.41808	0.43779
73	0.34293	0.37150	0.38521	0.38990	0.40532	0.42251	0.44213
74	0.34746	0.37603	0.38972	0.39436	0.40974	0.42686	0.44640
75	0.35188	0.38050	0.39415	0.39876	0.41411	0.43113	0.45059
76	0.35642	0.38485	0.39849	0.40307	0.41835	0.43536	0.45473
77	0.36091	0.38921	0.40275	0.40736	0.42257	0.43950	0.45876
78	0.36540	0.39341	0.40699	0.41158	0.42672	0.44358	0.46278
79	0.36967	0.39765	0.41112	0.41569	0.43078	0.44761	0.46669

N	0.1%	0.5%	1%	1.25%	2.5%	5%	10%
80	0.37380	0.40175	0.41521	0.41977	0.43482	0.45155	0.47055
81	0.37789	0.40580	0.41921	0.42378	0.43877	0.45541	0.47435
82	0.38212	0.40982	0.42323	0.42770	0.44266	0.45925	0.47807
83	0.38586	0.41378	0.42711	0.43158	0.44650	0.46301	0.48173
84	0.39003	0.41767	0.43094	0.43545	0.45032	0.46673	0.48535
85	0.39394	0.42151	0.43471	0.43921	0.45402	0.47037	0.48892
86	0.39785	0.42528	0.43848	0.44295	0.45768	0.47397	0.49241
87	0.40164	0.42901	0.44216	0.44663	0.46131	0.47750	0.49586
88	0.40547	0.43269	0.44586	0.45027	0.46485	0.48098	0.49926
89	0.40931	0.43635	0.44942	0.45382	0.46836	0.48441	0.50259
90	0.41294	0.43994	0.45298	0.45737	0.47182	0.48780	0.50589
91	0.41660	0.44348	0.45646	0.46081	0.47522	0.49112	0.50914
92	0.42012	0.44690	0.45984	0.46422	0.47858	0.49442	0.51233
93	0.42364	0.45040	0.46326	0.46765	0.48187	0.49766	0.51550
94	0.42710	0.45381	0.46664	0.47094	0.48517	0.50084	0.51861
95	0.43058	0.45720	0.46991	0.47419	0.48832	0.50399	0.52165
96	0.43401	0.46049	0.47320	0.47745	0.49155	0.50709	0.52467
97	0.43733	0.46374	0.47638	0.48065	0.49468	0.51014	0.52764
98	0.44065	0.46696	0.47955	0.48381	0.49775	0.51315	0.53059
99	0.44384	0.47010	0.48268	0.48691	0.50079	0.51613	0.53350
100	0.44709	0.47329	0.48573	0.48993	0.50381	0.51907	0.53636
101	0.45036	0.47640	0.48878	0.49299	0.50678	0.52196	0.53917
102	0.45363	0.47946	0.49180	0.49596	0.50971	0.52482	0.54193
103	0.45671	0.48247	0.49475	0.49892	0.51258	0.52766	0.54467
104	0.45973	0.48542	0.49770	0.50186	0.51545	0.53044	0.54736
105	0.46262	0.48840	0.50059	0.50471	0.51827	0.53318	0.55002
106	0.46567	0.49132	0.50347	0.50758	0.52105	0.53588	0.55264
107	0.46857	0.49415	0.50630	0.51036	0.52379	0.53857	0.55524
108	0.47146	0.49706	0.50909	0.51313	0.52650	0.54122	0.55782
109	0.47435	0.49981	0.51183	0.51585	0.52915	0.54383	0.56034
110	0.47732	0.50260	0.51453	0.51854	0.53177	0.54639	0.56283
111	0.48015	0.50531	0.51722	0.52126	0.53438	0.54893	0.56529
112	0.48290	0.50802	0.51987	0.52386	0.53696	0.55143	0.56773
113	0.48562	0.51071	0.52247	0.52646	0.53950	0.55391	0.57014
114	0.48841	0.51333	0.52505	0.52901	0.54198	0.55636	0.57250
115	0.49117	0.51588	0.52763	0.53154	0.54447	0.55877	0.57483
116	0.49378	0.51845	0.53014	0.53401	0.54693	0.56115	0.57713
117	0.49637	0.52101	0.53262	0.53651	0.54934	0.56349	0.57941
118	0.49898	0.52355	0.53509	0.53896	0.55173	0.56582	0.58166
119	0.50160	0.52602	0.53751	0.54138	0.55408	0.56810	0.58389
120	0.50411	0.52849	0.53993	0.54378	0.55645	0.57037	0.58607
121	0.50659	0.53087	0.54234	0.54615	0.55877	0.57263	0.58825
122	0.50900	0.53328	0.54470	0.54849	0.56105	0.57485	0.59040
123	0.51144	0.53560	0.54700	0.55079	0.56328	0.57703	0.59254
124	0.51386	0.53794	0.54931	0.55309	0.56554	0.57921	0.59463
125	0.51626	0.54021	0.55154	0.55532	0.56772	0.58136	0.59671
126	0.51865	0.54254	0.55380	0.55752	0.56991	0.58347	0.59876
127	0.52110	0.54475	0.55601	0.55974	0.57207	0.58558	0.60079
128	0.52350	0.54705	0.55817	0.56193	0.57418	0.58766	0.60278
129	0.52568	0.54928	0.56035	0.56408	0.57632	0.58971	0.60476
130	0.52805	0.55143	0.56252	0.56619	0.57837	0.59172	0.60673
131	0.53036	0.55363	0.56462	0.56831	0.58044	0.59373	0.60867
132	0.53264	0.55574	0.56670	0.57040	0.58247	0.59571	0.61058
133	0.53486	0.55786	0.56879	0.57249	0.58449	0.59767	0.61247
134	0.53709	0.55997	0.57079	0.57454	0.58648	0.59963	0.61435
135	0.53920	0.56205	0.57290	0.57657	0.58843	0.60153	0.61620
136	0.54142	0.56410	0.57490	0.57855	0.59040	0.60344	0.61804
137	0.54361	0.56614	0.57692	0.58054	0.59234	0.60530	0.61985
138	0.54573	0.56814	0.57887	0.58247	0.59422	0.60717	0.62164
139	0.54786	0.57011	0.58082	0.58440	0.59612	0.60901	0.62341
140	0.54997	0.57213	0.58275	0.58635	0.59799	0.61082	0.62516
141	0.55191	0.57409	0.58468	0.58823	0.59987	0.61260	0.62690
142	0.55391	0.57602	0.58656	0.59011	0.60173	0.61439	0.62862
143	0.55587	0.57798	0.58844	0.59199	0.60355	0.61616	0.63033
144	0.55790	0.57988	0.59031	0.59380	0.60533	0.61790	0.63200
145	0.55988	0.58175	0.59213	0.59565	0.60712	0.61964	0.63367
146	0.56181	0.58359	0.59393	0.59745	0.60889	0.62134	0.63533
147	0.56375	0.58542	0.59575	0.59924	0.61062	0.62302	0.63695
148	0.56564	0.58726	0.59752	0.60100	0.61235	0.62469	0.63855
149	0.56762	0.58903	0.59928	0.60277	0.61404	0.62636	0.64015
150	0.56945	0.59083	0.60107	0.60451	0.61574	0.62801	0.64174
151	0.57130	0.59260	0.60280	0.60623	0.61742	0.62963	0.64331
152	0.57310	0.59438	0.60454	0.60793	0.61908	0.63124	0.64487
153	0.57501	0.59614	0.60624	0.60962	0.62072	0.63284	0.64640

N	0.1%	0.5%	1%	1.25%	2.5%	5%	10%
154	0.57669	0.59786	0.60793	0.61129	0.62236	0.63441	0.64792
155	0.57851	0.59956	0.60959	0.61296	0.62397	0.63598	0.64943
156	0.58027	0.60126	0.61126	0.61460	0.62555	0.63752	0.65092
157	0.58203	0.60292	0.61289	0.61621	0.62713	0.63905	0.65241
158	0.58372	0.60457	0.61448	0.61782	0.62869	0.64058	0.65387
159	0.58546	0.60615	0.61608	0.61941	0.63025	0.64209	0.65532
160	0.58715	0.60781	0.61769	0.62100	0.63177	0.64357	0.65677
161	0.58887	0.60946	0.61927	0.62255	0.63330	0.64505	0.65818
162	0.59044	0.61105	0.62083	0.62410	0.63482	0.64652	0.65959
163	0.59211	0.61262	0.62240	0.62564	0.63631	0.64796	0.66098
164	0.59379	0.61422	0.62391	0.62713	0.63780	0.64940	0.66236
165	0.59545	0.61576	0.62543	0.62865	0.63928	0.65082	0.66374
166	0.59713	0.61726	0.62693	0.63015	0.64073	0.65222	0.66510
167	0.59870	0.61880	0.62841	0.63164	0.64216	0.65362	0.66645
168	0.60026	0.62034	0.62990	0.63311	0.64358	0.65502	0.66778
169	0.60178	0.62186	0.63138	0.63455	0.64500	0.65640	0.66911
170	0.60336	0.62337	0.63282	0.63603	0.64641	0.65775	0.67041
171	0.60488	0.62486	0.63428	0.63745	0.64778	0.65910	0.67169
172	0.60644	0.62631	0.63569	0.63889	0.64917	0.66042	0.67298
173	0.60791	0.62779	0.63713	0.64029	0.65053	0.66175	0.67427
174	0.60951	0.62923	0.63850	0.64168	0.65189	0.66306	0.67553
175	0.61095	0.63064	0.63989	0.64307	0.65323	0.66438	0.67678
176	0.61237	0.63205	0.64130	0.64442	0.65456	0.66565	0.67802
177	0.61379	0.63346	0.64269	0.64577	0.65588	0.66694	0.67926
178	0.61522	0.63482	0.64400	0.64711	0.65719	0.66821	0.68048
179	0.61669	0.63624	0.64535	0.64844	0.65849	0.66946	0.68169
180	0.61809	0.63759	0.64670	0.64978	0.65978	0.67071	0.68289
181	0.61956	0.63892	0.64802	0.65109	0.66107	0.67194	0.68407
182	0.62102	0.64026	0.64933	0.65240	0.66233	0.67316	0.68526
183	0.62240	0.64161	0.65063	0.65370	0.66358	0.67437	0.68643
184	0.62381	0.64291	0.65193	0.65497	0.66481	0.67559	0.68759
185	0.62513	0.64422	0.65320	0.65621	0.66605	0.67678	0.68874
186	0.62649	0.64556	0.65446	0.65747	0.66728	0.67797	0.68988
187	0.62789	0.64682	0.65573	0.65873	0.66848	0.67914	0.69101
188	0.62912	0.64812	0.65699	0.65995	0.66968	0.68030	0.69214
189	0.63048	0.64939	0.65824	0.66120	0.67087	0.68145	0.69324
190	0.63189	0.65065	0.65947	0.66240	0.67208	0.68259	0.69434
191	0.63321	0.65190	0.66067	0.66361	0.67324	0.68373	0.69544
192	0.63449	0.65310	0.66187	0.66483	0.67440	0.68487	0.69653
193	0.63572	0.65433	0.66308	0.66602	0.67556	0.68598	0.69760
194	0.63702	0.65555	0.66425	0.66719	0.67671	0.68708	0.69868
195	0.63832	0.65677	0.66542	0.66836	0.67785	0.68818	0.69974
196	0.63958	0.65796	0.66661	0.66954	0.67896	0.68928	0.70079
197	0.64086	0.65913	0.66778	0.67067	0.68008	0.69037	0.70183
198	0.64216	0.66033	0.66891	0.67179	0.68119	0.69145	0.70287
199	0.64336	0.66149	0.67007	0.67294	0.68229	0.69252	0.70390
200	0.64457	0.66265	0.67121	0.67407	0.68340	0.69358	0.70491
201	0.64576	0.66381	0.67233	0.67520	0.68450	0.69463	0.70593
202	0.64698	0.66495	0.67345	0.67630	0.68557	0.69568	0.70693
203	0.64817	0.66612	0.67455	0.67740	0.68663	0.69671	0.70792
204	0.64929	0.66722	0.67565	0.67849	0.68767	0.69773	0.70892
205	0.65050	0.66836	0.67676	0.67959	0.68873	0.69875	0.70989
206	0.65168	0.66950	0.67782	0.68065	0.68977	0.69976	0.71086
207	0.65275	0.67062	0.67892	0.68172	0.69082	0.70076	0.71183
208	0.65391	0.67169	0.67997	0.68277	0.69185	0.70176	0.71278
209	0.65501	0.67279	0.68105	0.68383	0.69288	0.70275	0.71373
210	0.65618	0.67385	0.68210	0.68487	0.69388	0.70373	0.71468
211	0.65730	0.67496	0.68313	0.68589	0.69490	0.70471	0.71561
212	0.65842	0.67602	0.68418	0.68694	0.69591	0.70567	0.71654
213	0.65951	0.67707	0.68520	0.68796	0.69688	0.70664	0.71747
214	0.66057	0.67812	0.68624	0.68897	0.69787	0.70759	0.71839
215	0.66168	0.67915	0.68723	0.68998	0.69886	0.70854	0.71930
216	0.66283	0.68017	0.68825	0.69098	0.69983	0.70948	0.72020
217	0.66393	0.68118	0.68926	0.69197	0.70079	0.71041	0.72110
218	0.66503	0.68223	0.69025	0.69297	0.70174	0.71134	0.72199
219	0.66606	0.68323	0.69125	0.69394	0.70270	0.71225	0.72288
220	0.66710	0.68424	0.69224	0.69492	0.70365	0.71316	0.72376
221	0.66816	0.68524	0.69321	0.69589	0.70459	0.71407	0.72463
222	0.66919	0.68623	0.69417	0.69685	0.70551	0.71497	0.72550
223	0.67028	0.68723	0.69512	0.69778	0.70644	0.71586	0.72636
224	0.67120	0.68818	0.69607	0.69873	0.70735	0.71675	0.72721
225	0.67225	0.68915	0.69703	0.69967	0.70826	0.71763	0.72806
226	0.67328	0.69010	0.69794	0.70060	0.70916	0.71851	0.72889
227	0.67430	0.69108	0.69889	0.70152	0.71006	0.71938	0.72974

N	0.1%	0.5%	1%	1.25%	2.5%	5%	10%
228	0.67530	0.69202	0.69983	0.70244	0.71095	0.72023	0.73057
229	0.67629	0.69297	0.70075	0.70335	0.71184	0.72109	0.73139
230	0.67726	0.69390	0.70166	0.70426	0.71272	0.72194	0.73221
231	0.67827	0.69482	0.70258	0.70515	0.71359	0.72277	0.73303
232	0.67928	0.69574	0.70345	0.70605	0.71445	0.72362	0.73384
233	0.68021	0.69665	0.70434	0.70693	0.71532	0.72446	0.73464
234	0.68116	0.69756	0.70524	0.70780	0.71618	0.72529	0.73543
235	0.68212	0.69847	0.70613	0.70867	0.71702	0.72612	0.73623
236	0.68314	0.69937	0.70702	0.70954	0.71786	0.72693	0.73701
237	0.68407	0.70029	0.70788	0.71040	0.71870	0.72774	0.73779
238	0.68499	0.70116	0.70872	0.71126	0.71954	0.72854	0.73856
239	0.68593	0.70205	0.70958	0.71212	0.72038	0.72934	0.73933
240	0.68685	0.70290	0.71043	0.71297	0.72119	0.73013	0.74010
241	0.68779	0.70374	0.71130	0.71382	0.72200	0.73093	0.74085
242	0.68865	0.70458	0.71215	0.71466	0.72280	0.73172	0.74161
243	0.68956	0.70547	0.71299	0.71548	0.72361	0.73250	0.74236
244	0.69046	0.70633	0.71381	0.71629	0.72443	0.73327	0.74310
245	0.69138	0.70718	0.71464	0.71710	0.72521	0.73404	0.74384
246	0.69224	0.70803	0.71547	0.71794	0.72599	0.73481	0.74458
247	0.69317	0.70890	0.71627	0.71875	0.72679	0.73557	0.74531
248	0.69407	0.70972	0.71708	0.71957	0.72758	0.73632	0.74604
249	0.69495	0.71054	0.71789	0.72036	0.72836	0.73707	0.74676
250	0.69578	0.71137	0.71870	0.72114	0.72912	0.73782	0.74748
251	0.69665	0.71221	0.71950	0.72193	0.72989	0.73857	0.74819
252	0.69755	0.71303	0.72026	0.72273	0.73065	0.73930	0.74890
253	0.69839	0.71383	0.72106	0.72351	0.73141	0.74004	0.74960
254	0.69927	0.71461	0.72182	0.72427	0.73216	0.74076	0.75031
255	0.70014	0.71543	0.72261	0.72502	0.73290	0.74148	0.75099
256	0.70098	0.71621	0.72339	0.72579	0.73365	0.74220	0.75168
257	0.70180	0.71698	0.72415	0.72655	0.73438	0.74291	0.75237
258	0.70262	0.71776	0.72490	0.72730	0.73512	0.74362	0.75304
259	0.70347	0.71855	0.72566	0.72805	0.73584	0.74433	0.75373
260	0.70431	0.71931	0.72642	0.72880	0.73656	0.74502	0.75440
261	0.70514	0.72007	0.72716	0.72954	0.73728	0.74572	0.75507
262	0.70591	0.72084	0.72790	0.73029	0.73800	0.74641	0.75573
263	0.70672	0.72162	0.72862	0.73102	0.73871	0.74709	0.75639
264	0.70749	0.72234	0.72938	0.73174	0.73942	0.74778	0.75705
265	0.70828	0.72308	0.73008	0.73245	0.74011	0.74846	0.75771
266	0.70902	0.72381	0.73082	0.73317	0.74082	0.74913	0.75836
267	0.70983	0.72458	0.73154	0.73389	0.74151	0.74980	0.75901
268	0.71060	0.72531	0.73226	0.73461	0.74221	0.75046	0.75965
269	0.71138	0.72604	0.73297	0.73530	0.74291	0.75112	0.76028
270	0.71218	0.72678	0.73367	0.73601	0.74359	0.75178	0.76092
271	0.71289	0.72752	0.73438	0.73670	0.74426	0.75244	0.76155
272	0.71361	0.72824	0.73508	0.73740	0.74493	0.75309	0.76217
273	0.71438	0.72895	0.73577	0.73809	0.74559	0.75373	0.76280
274	0.71512	0.72965	0.73648	0.73878	0.74627	0.75438	0.76342
275	0.71585	0.73036	0.73718	0.73947	0.74693	0.75502	0.76403
276	0.71657	0.73109	0.73786	0.74015	0.74759	0.75566	0.76464
277	0.71733	0.73179	0.73855	0.74084	0.74824	0.75629	0.76525
278	0.71808	0.73246	0.73922	0.74150	0.74889	0.75693	0.76586
279	0.71880	0.73315	0.73990	0.74217	0.74953	0.75755	0.76646
280	0.71954	0.73383	0.74055	0.74283	0.75018	0.75817	0.76705
281	0.72030	0.73452	0.74124	0.74349	0.75081	0.75878	0.76765
282	0.72103	0.73521	0.74190	0.74415	0.75145	0.75940	0.76824
283	0.72169	0.73589	0.74256	0.74481	0.75208	0.76002	0.76883
284	0.72245	0.73657	0.74322	0.74546	0.75271	0.76063	0.76941
285	0.72315	0.73724	0.74388	0.74610	0.75333	0.76123	0.76999
286	0.72385	0.73791	0.74453	0.74674	0.75396	0.76183	0.77057
287	0.72455	0.73856	0.74516	0.74739	0.75458	0.76243	0.77114
288	0.72525	0.73922	0.74580	0.74802	0.75520	0.76303	0.77171
289	0.72594	0.73989	0.74645	0.74865	0.75581	0.76361	0.77228
290	0.72659	0.74054	0.74707	0.74926	0.75643	0.76421	0.77285
291	0.72723	0.74119	0.74771	0.74990	0.75703	0.76479	0.77341
292	0.72794	0.74182	0.74834	0.75052	0.75763	0.76538	0.77397
293	0.72861	0.74248	0.74896	0.75113	0.75823	0.76596	0.77451
294	0.72920	0.74310	0.74958	0.75174	0.75882	0.76653	0.77507
295	0.72988	0.74374	0.75021	0.75236	0.75941	0.76710	0.77561
296	0.73055	0.74437	0.75083	0.75297	0.76000	0.76767	0.77617
297	0.73121	0.74501	0.75143	0.75358	0.76059	0.76824	0.77671
298	0.73186	0.74563	0.75202	0.75418	0.76117	0.76880	0.77725
299	0.73252	0.74624	0.75262	0.75478	0.76175	0.76936	0.77779
300	0.73320	0.74685	0.75323	0.75537	0.76233	0.76990	0.77832
301	0.73385	0.74746	0.75382	0.75597	0.76289	0.77046	0.77885

N	0.1%	0.5%	1%	1.25%	2.5%	5%	10%
302	0.73452	0.74806	0.75441	0.75655	0.76346	0.77100	0.77938
303	0.73514	0.74864	0.75500	0.75713	0.76403	0.77155	0.77990
304	0.73578	0.74927	0.75558	0.75769	0.76459	0.77209	0.78042
305	0.73643	0.74985	0.75617	0.75827	0.76515	0.77263	0.78095
306	0.73706	0.75046	0.75675	0.75886	0.76572	0.77317	0.78147
307	0.73770	0.75106	0.75733	0.75942	0.76627	0.77371	0.78198
308	0.73830	0.75163	0.75790	0.75999	0.76683	0.77424	0.78249
309	0.73891	0.75223	0.75848	0.76057	0.76738	0.77477	0.78300
310	0.73952	0.75281	0.75904	0.76112	0.76792	0.77529	0.78351
311	0.74013	0.75339	0.75961	0.76168	0.76846	0.77582	0.78401
312	0.74077	0.75395	0.76017	0.76225	0.76900	0.77634	0.78451
313	0.74139	0.75452	0.76072	0.76280	0.76953	0.77686	0.78501
314	0.74200	0.75510	0.76129	0.76334	0.77007	0.77738	0.78551
315	0.74258	0.75567	0.76184	0.76389	0.77061	0.77790	0.78600
316	0.74316	0.75622	0.76237	0.76443	0.77114	0.77841	0.78650
317	0.74378	0.75680	0.76292	0.76498	0.77167	0.77892	0.78699
318	0.74436	0.75735	0.76347	0.76552	0.77219	0.77943	0.78747
319	0.74493	0.75792	0.76401	0.76607	0.77271	0.77993	0.78795
320	0.74550	0.75846	0.76455	0.76659	0.77323	0.78043	0.78843
321	0.74609	0.75900	0.76510	0.76712	0.77374	0.78093	0.78891
322	0.74669	0.75955	0.76563	0.76765	0.77425	0.78142	0.78938
323	0.74725	0.76010	0.76616	0.76818	0.77476	0.78192	0.78986
324	0.74783	0.76063	0.76669	0.76870	0.77527	0.78241	0.79033
325	0.74841	0.76118	0.76721	0.76922	0.77577	0.78290	0.79080
326	0.74899	0.76171	0.76773	0.76974	0.77627	0.78339	0.79127
327	0.74954	0.76226	0.76826	0.77027	0.77678	0.78387	0.79173
328	0.75010	0.76279	0.76877	0.77077	0.77727	0.78435	0.79219
329	0.75067	0.76331	0.76930	0.77128	0.77777	0.78483	0.79265
330	0.75120	0.76384	0.76980	0.77179	0.77826	0.78530	0.79310
331	0.75175	0.76435	0.77030	0.77230	0.77875	0.78577	0.79356
332	0.75226	0.76487	0.77081	0.77281	0.77924	0.78625	0.79401
333	0.75281	0.76541	0.77131	0.77330	0.77973	0.78671	0.79447
334	0.75334	0.76593	0.77182	0.77381	0.78021	0.78718	0.79491
335	0.75387	0.76644	0.77233	0.77430	0.78069	0.78764	0.79536
336	0.75442	0.76694	0.77283	0.77480	0.78118	0.78811	0.79581
337	0.75498	0.76746	0.77333	0.77529	0.78165	0.78857	0.79625
338	0.75552	0.76797	0.77383	0.77577	0.78212	0.78903	0.79669
339	0.75607	0.76847	0.77432	0.77626	0.78259	0.78948	0.79712
340	0.75661	0.76898	0.77482	0.77675	0.78307	0.78993	0.79756
341	0.75711	0.76948	0.77531	0.77724	0.78353	0.79039	0.79800
342	0.75763	0.76997	0.77579	0.77772	0.78400	0.79083	0.79843
343	0.75817	0.77048	0.77626	0.77820	0.78446	0.79128	0.79886
344	0.75866	0.77096	0.77675	0.77868	0.78492	0.79173	0.79929
345	0.75919	0.77145	0.77722	0.77915	0.78538	0.79217	0.79971
346	0.75971	0.77193	0.77771	0.77963	0.78583	0.79262	0.80013
347	0.76019	0.77244	0.77818	0.78009	0.78629	0.79305	0.80056
348	0.76071	0.77292	0.77866	0.78056	0.78675	0.79349	0.80098
349	0.76124	0.77342	0.77913	0.78101	0.78719	0.79393	0.80139
350	0.76178	0.77390	0.77959	0.78148	0.78764	0.79436	0.80180
351	0.76228	0.77438	0.78005	0.78195	0.78810	0.79479	0.80222
352	0.76277	0.77486	0.78052	0.78241	0.78854	0.79521	0.80263
353	0.76323	0.77533	0.78098	0.78287	0.78898	0.79565	0.80304
354	0.76374	0.77580	0.78143	0.78333	0.78942	0.79607	0.80345
355	0.76424	0.77628	0.78190	0.78377	0.78986	0.79649	0.80386
356	0.76473	0.77674	0.78235	0.78422	0.79029	0.79692	0.80426
357	0.76521	0.77721	0.78280	0.78467	0.79072	0.79734	0.80466
358	0.76566	0.77766	0.78325	0.78511	0.79115	0.79775	0.80506
359	0.76618	0.77813	0.78369	0.78556	0.79158	0.79817	0.80547
360	0.76665	0.77859	0.78414	0.78600	0.79201	0.79858	0.80586
361	0.76711	0.77905	0.78458	0.78645	0.79244	0.79899	0.80626
362	0.76762	0.77951	0.78503	0.78688	0.79286	0.79941	0.80665
363	0.76809	0.77995	0.78547	0.78732	0.79328	0.79982	0.80704
364	0.76859	0.78039	0.78590	0.78774	0.79371	0.80022	0.80743
365	0.76902	0.78084	0.78634	0.78816	0.79413	0.80062	0.80782
366	0.76952	0.78128	0.78677	0.78859	0.79455	0.80102	0.80821
367	0.77000	0.78172	0.78720	0.78903	0.79496	0.80142	0.80859
368	0.77048	0.78215	0.78762	0.78946	0.79537	0.80181	0.80897
369	0.77093	0.78260	0.78805	0.78988	0.79579	0.80222	0.80936
370	0.77135	0.78302	0.78848	0.79031	0.79620	0.80261	0.80974
371	0.77182	0.78344	0.78891	0.79072	0.79660	0.80300	0.81012
372	0.77229	0.78389	0.78933	0.79114	0.79701	0.80339	0.81049
373	0.77272	0.78432	0.78975	0.79155	0.79741	0.80378	0.81087
374	0.77321	0.78475	0.79017	0.79197	0.79781	0.80417	0.81124
375	0.77363	0.78517	0.79058	0.79238	0.79822	0.80456	0.81161

N	0.1%	0.5%	1%	1.25%	2.5%	5%	10%
376	0.77408	0.78562	0.79101	0.79279	0.79861	0.80495	0.81198
377	0.77454	0.78602	0.79141	0.79320	0.79902	0.80533	0.81235
378	0.77503	0.78644	0.79182	0.79361	0.79941	0.80571	0.81271
379	0.77540	0.78687	0.79223	0.79401	0.79980	0.80609	0.81308
380	0.77586	0.78728	0.79264	0.79442	0.80020	0.80647	0.81344
381	0.77630	0.78770	0.79305	0.79482	0.80058	0.80685	0.81380
382	0.77675	0.78811	0.79345	0.79522	0.80097	0.80722	0.81416
383	0.77717	0.78854	0.79386	0.79562	0.80135	0.80759	0.81452
384	0.77760	0.78895	0.79426	0.79602	0.80174	0.80797	0.81488
385	0.77805	0.78936	0.79466	0.79642	0.80212	0.80833	0.81523
386	0.77848	0.78977	0.79505	0.79680	0.80250	0.80871	0.81559
387	0.77892	0.79018	0.79544	0.79719	0.80288	0.80907	0.81594
388	0.77934	0.79057	0.79583	0.79758	0.80326	0.80944	0.81629
389	0.77973	0.79098	0.79622	0.79796	0.80363	0.80980	0.81664
390	0.78017	0.79138	0.79661	0.79835	0.80401	0.81016	0.81699
391	0.78059	0.79179	0.79700	0.79874	0.80439	0.81052	0.81733
392	0.78099	0.79219	0.79738	0.79911	0.80476	0.81088	0.81768
393	0.78142	0.79257	0.79776	0.79949	0.80514	0.81124	0.81802
394	0.78184	0.79298	0.79815	0.79988	0.80550	0.81159	0.81836
395	0.78226	0.79336	0.79853	0.80025	0.80587	0.81195	0.81870
396	0.78266	0.79375	0.79891	0.80063	0.80623	0.81230	0.81904
397	0.78307	0.79415	0.79929	0.80100	0.80660	0.81265	0.81938
398	0.78347	0.79453	0.79967	0.80138	0.80696	0.81301	0.81972
399	0.78389	0.79491	0.80005	0.80176	0.80733	0.81335	0.82005
400	0.78428	0.79529	0.80041	0.80212	0.80769	0.81370	0.82038
401	0.78469	0.79568	0.80079	0.80249	0.80805	0.81405	0.82072
402	0.78511	0.79607	0.80115	0.80287	0.80841	0.81439	0.82104
403	0.78550	0.79645	0.80153	0.80324	0.80876	0.81473	0.82138
404	0.78591	0.79683	0.80189	0.80360	0.80912	0.81508	0.82171
405	0.78631	0.79719	0.80227	0.80396	0.80947	0.81542	0.82204
406	0.78669	0.79758	0.80264	0.80433	0.80982	0.81575	0.82236
407	0.78711	0.79796	0.80300	0.80468	0.81018	0.81609	0.82268
408	0.78750	0.79833	0.80336	0.80505	0.81052	0.81643	0.82301
409	0.78788	0.79869	0.80372	0.80541	0.81087	0.81676	0.82333
410	0.78826	0.79906	0.80408	0.80576	0.81121	0.81710	0.82365
411	0.78864	0.79943	0.80444	0.80612	0.81156	0.81742	0.82397
412	0.78903	0.79980	0.80481	0.80647	0.81190	0.81776	0.82429
413	0.78946	0.80016	0.80515	0.80682	0.81224	0.81809	0.82461
414	0.78981	0.80053	0.80550	0.80717	0.81258	0.81842	0.82492
415	0.79019	0.80088	0.80586	0.80752	0.81292	0.81874	0.82523
416	0.79054	0.80124	0.80620	0.80786	0.81326	0.81906	0.82555
417	0.79092	0.80159	0.80655	0.80821	0.81359	0.81939	0.82586
418	0.79130	0.80195	0.80691	0.80856	0.81392	0.81971	0.82617
419	0.79169	0.80231	0.80726	0.80890	0.81425	0.82004	0.82648
420	0.79207	0.80267	0.80760	0.80925	0.81458	0.82036	0.82678
421	0.79245	0.80300	0.80794	0.80959	0.81491	0.82068	0.82709
422	0.79286	0.80336	0.80827	0.80993	0.81524	0.82100	0.82740
423	0.79322	0.80371	0.80862	0.81027	0.81556	0.82131	0.82770
424	0.79360	0.80406	0.80896	0.81060	0.81589	0.82163	0.82801
425	0.79395	0.80441	0.80931	0.81095	0.81622	0.82194	0.82831
426	0.79432	0.80477	0.80965	0.81128	0.81654	0.82226	0.82861
427	0.79470	0.80510	0.80998	0.81161	0.81686	0.82257	0.82891
428	0.79505	0.80544	0.81031	0.81194	0.81718	0.82288	0.82921
429	0.79542	0.80578	0.81063	0.81227	0.81750	0.82319	0.82950
430	0.79580	0.80613	0.81097	0.81260	0.81781	0.82350	0.82980
431	0.79616	0.80648	0.81131	0.81293	0.81813	0.82380	0.83009
432	0.79654	0.80681	0.81163	0.81325	0.81845	0.82410	0.83039
433	0.79691	0.80715	0.81196	0.81357	0.81877	0.82441	0.83068
434	0.79723	0.80748	0.81228	0.81390	0.81908	0.82471	0.83097
435	0.79760	0.80781	0.81261	0.81421	0.81939	0.82501	0.83126
436	0.79794	0.80814	0.81293	0.81454	0.81970	0.82532	0.83155
437	0.79830	0.80847	0.81326	0.81486	0.82001	0.82561	0.83184
438	0.79863	0.80880	0.81358	0.81518	0.82033	0.82591	0.83212
439	0.79899	0.80913	0.81390	0.81549	0.82063	0.82621	0.83241
440	0.79934	0.80946	0.81422	0.81581	0.82094	0.82651	0.83269
441	0.79965	0.80978	0.81453	0.81613	0.82124	0.82680	0.83298
442	0.80001	0.81012	0.81485	0.81644	0.82155	0.82710	0.83326
443	0.80034	0.81044	0.81517	0.81675	0.82185	0.82739	0.83354
444	0.80070	0.81077	0.81548	0.81707	0.82216	0.82768	0.83382
445	0.80104	0.81109	0.81580	0.81737	0.82246	0.82797	0.83410
446	0.80136	0.81142	0.81611	0.81768	0.82276	0.82826	0.83438
447	0.80169	0.81174	0.81642	0.81799	0.82306	0.82855	0.83466
448	0.80204	0.81205	0.81672	0.81830	0.82336	0.82884	0.83493
449	0.80240	0.81236	0.81704	0.81861	0.82366	0.82913	0.83521

N	0.1%	0.5%	1%	1.25%	2.5%	5%	10%
450	0.80271	0.81268	0.81735	0.81892	0.82395	0.82941	0.83548
451	0.80307	0.81298	0.81767	0.81922	0.82424	0.82970	0.83575
452	0.80337	0.81331	0.81797	0.81952	0.82453	0.82998	0.83603
453	0.80371	0.81363	0.81827	0.81982	0.82482	0.83026	0.83629
454	0.80404	0.81393	0.81858	0.82011	0.82512	0.83054	0.83656
455	0.80435	0.81423	0.81887	0.82041	0.82541	0.83082	0.83683
456	0.80468	0.81455	0.81917	0.82072	0.82570	0.83110	0.83710
457	0.80499	0.81486	0.81947	0.82102	0.82599	0.83138	0.83737
458	0.80532	0.81517	0.81977	0.82132	0.82628	0.83166	0.83764
459	0.80565	0.81547	0.82007	0.82160	0.82656	0.83193	0.83790
460	0.80597	0.81577	0.82038	0.82190	0.82685	0.83221	0.83817
461	0.80629	0.81608	0.82067	0.82220	0.82713	0.83248	0.83843
462	0.80660	0.81639	0.82097	0.82249	0.82742	0.83275	0.83869
463	0.80692	0.81668	0.82126	0.82278	0.82770	0.83302	0.83895
464	0.80724	0.81697	0.82155	0.82307	0.82798	0.83329	0.83922
465	0.80755	0.81728	0.82184	0.82336	0.82827	0.83356	0.83948
466	0.80787	0.81757	0.82213	0.82365	0.82854	0.83383	0.83974
467	0.80816	0.81788	0.82242	0.82393	0.82882	0.83410	0.83999
468	0.80848	0.81817	0.82271	0.82422	0.82909	0.83437	0.84025
469	0.80879	0.81847	0.82299	0.82450	0.82937	0.83463	0.84050
470	0.80909	0.81876	0.82327	0.82478	0.82965	0.83490	0.84076
471	0.80941	0.81906	0.82356	0.82506	0.82992	0.83517	0.84102
472	0.80971	0.81934	0.82384	0.82535	0.83019	0.83544	0.84127
473	0.81002	0.81963	0.82412	0.82563	0.83046	0.83570	0.84153
474	0.81032	0.81992	0.82441	0.82590	0.83073	0.83596	0.84178
475	0.81065	0.82020	0.82469	0.82618	0.83101	0.83621	0.84203
476	0.81095	0.82050	0.82496	0.82646	0.83128	0.83648	0.84228
477	0.81126	0.82079	0.82524	0.82673	0.83154	0.83673	0.84253
478	0.81155	0.82108	0.82551	0.82701	0.83181	0.83699	0.84278
479	0.81183	0.82137	0.82580	0.82728	0.83208	0.83725	0.84302
480	0.81214	0.82167	0.82608	0.82756	0.83235	0.83751	0.84327
481	0.81246	0.82195	0.82635	0.82782	0.83261	0.83776	0.84351
482	0.81276	0.82222	0.82662	0.82810	0.83287	0.83802	0.84376
483	0.81305	0.82250	0.82690	0.82837	0.83314	0.83827	0.84400
484	0.81336	0.82278	0.82717	0.82864	0.83340	0.83852	0.84424
485	0.81365	0.82306	0.82744	0.82891	0.83366	0.83878	0.84449
486	0.81394	0.82333	0.82772	0.82918	0.83391	0.83903	0.84473
487	0.81426	0.82360	0.82799	0.82945	0.83417	0.83928	0.84497
488	0.81457	0.82389	0.82826	0.82972	0.83443	0.83952	0.84521
489	0.81486	0.82416	0.82852	0.82998	0.83469	0.83977	0.84545
490	0.81515	0.82444	0.82879	0.83024	0.83494	0.84002	0.84568
491	0.81541	0.82472	0.82905	0.83050	0.83519	0.84027	0.84592
492	0.81570	0.82498	0.82932	0.83076	0.83545	0.84051	0.84616
493	0.81599	0.82524	0.82957	0.83102	0.83571	0.84076	0.84639
494	0.81627	0.82551	0.82984	0.83129	0.83596	0.84101	0.84663
495	0.81655	0.82579	0.83010	0.83154	0.83622	0.84125	0.84686
496	0.81684	0.82607	0.83036	0.83180	0.83647	0.84149	0.84709
497	0.81711	0.82634	0.83063	0.83206	0.83672	0.84174	0.84733
498	0.81742	0.82660	0.83089	0.83231	0.83696	0.84198	0.84756
499	0.81770	0.82687	0.83115	0.83257	0.83721	0.84222	0.84779
500	0.81797	0.82714	0.83140	0.83283	0.83746	0.84246	0.84802
501	0.81824	0.82741	0.83166	0.83309	0.83771	0.84269	0.84824
502	0.81853	0.82767	0.83191	0.83333	0.83795	0.84294	0.84847
503	0.81879	0.82794	0.83217	0.83359	0.83820	0.84317	0.84870
504	0.81905	0.82820	0.83243	0.83385	0.83844	0.84341	0.84893
505	0.81933	0.82846	0.83268	0.83409	0.83869	0.84364	0.84916
506	0.81961	0.82873	0.83293	0.83435	0.83893	0.84388	0.84938
507	0.81991	0.82897	0.83318	0.83460	0.83918	0.84412	0.84961
508	0.82020	0.82924	0.83343	0.83484	0.83942	0.84435	0.84983
509	0.82049	0.82949	0.83368	0.83509	0.83966	0.84458	0.85005
510	0.82075	0.82974	0.83393	0.83533	0.83990	0.84481	0.85027
511	0.82103	0.82999	0.83418	0.83558	0.84013	0.84504	0.85050
512	0.82133	0.83025	0.83443	0.83582	0.84037	0.84527	0.85072
513	0.82157	0.83050	0.83468	0.83607	0.84061	0.84550	0.85094
514	0.82185	0.83076	0.83494	0.83632	0.84085	0.84573	0.85115
515	0.82211	0.83101	0.83519	0.83656	0.84108	0.84596	0.85137
516	0.82238	0.83126	0.83542	0.83680	0.84132	0.84619	0.85159
517	0.82264	0.83151	0.83566	0.83704	0.84155	0.84641	0.85181
518	0.82287	0.83176	0.83591	0.83729	0.84179	0.84664	0.85202
519	0.82314	0.83201	0.83615	0.83752	0.84202	0.84686	0.85224
520	0.82342	0.83225	0.83638	0.83776	0.84225	0.84709	0.85245
521	0.82367	0.83249	0.83662	0.83800	0.84247	0.84731	0.85267
522	0.82392	0.83275	0.83687	0.83825	0.84271	0.84753	0.85289
523	0.82419	0.83299	0.83711	0.83847	0.84294	0.84775	0.85310

N	0.1%	0.5%	1%	1.25%	2.5%	5%	10%
524	0.82444	0.83324	0.83735	0.83872	0.84317	0.84797	0.85331
525	0.82471	0.83348	0.83759	0.83895	0.84339	0.84819	0.85352
526	0.82497	0.83373	0.83782	0.83919	0.84362	0.84841	0.85373
527	0.82521	0.83397	0.83805	0.83942	0.84385	0.84863	0.85395
528	0.82546	0.83420	0.83828	0.83965	0.84407	0.84885	0.85416
529	0.82571	0.83444	0.83852	0.83988	0.84430	0.84907	0.85436
530	0.82597	0.83468	0.83875	0.84011	0.84453	0.84928	0.85457
531	0.82623	0.83493	0.83898	0.84034	0.84475	0.84950	0.85478
532	0.82648	0.83517	0.83922	0.84057	0.84498	0.84971	0.85499
533	0.82673	0.83541	0.83945	0.84080	0.84520	0.84993	0.85519
534	0.82698	0.83565	0.83968	0.84102	0.84542	0.85014	0.85540
535	0.82723	0.83589	0.83991	0.84126	0.84563	0.85036	0.85561
536	0.82746	0.83612	0.84013	0.84149	0.84585	0.85057	0.85581
537	0.82771	0.83636	0.84037	0.84172	0.84608	0.85079	0.85601
538	0.82796	0.83660	0.84059	0.84195	0.84629	0.85100	0.85622
539	0.82822	0.83684	0.84083	0.84216	0.84651	0.85121	0.85642
540	0.82847	0.83707	0.84105	0.84239	0.84673	0.85142	0.85662
541	0.82870	0.83729	0.84128	0.84261	0.84694	0.85163	0.85682
542	0.82895	0.83753	0.84150	0.84283	0.84716	0.85184	0.85703
543	0.82920	0.83775	0.84172	0.84305	0.84737	0.85205	0.85722
544	0.82943	0.83799	0.84195	0.84327	0.84759	0.85226	0.85743
545	0.82969	0.83822	0.84217	0.84349	0.84780	0.85246	0.85762
546	0.82994	0.83844	0.84239	0.84372	0.84802	0.85267	0.85782
547	0.83018	0.83867	0.84261	0.84394	0.84823	0.85287	0.85802
548	0.83041	0.83889	0.84283	0.84416	0.84844	0.85308	0.85822
549	0.83064	0.83913	0.84305	0.84437	0.84866	0.85328	0.85842
550	0.83087	0.83934	0.84327	0.84460	0.84887	0.85349	0.85861
551	0.83113	0.83957	0.84350	0.84482	0.84908	0.85369	0.85881
552	0.83137	0.83980	0.84371	0.84503	0.84928	0.85389	0.85900
553	0.83160	0.84002	0.84393	0.84525	0.84950	0.85410	0.85920
554	0.83183	0.84025	0.84415	0.84546	0.84970	0.85430	0.85939
555	0.83208	0.84047	0.84437	0.84568	0.84991	0.85450	0.85958
556	0.83230	0.84069	0.84459	0.84589	0.85012	0.85470	0.85978
557	0.83255	0.84091	0.84480	0.84611	0.85033	0.85490	0.85997
558	0.83278	0.84113	0.84503	0.84632	0.85053	0.85510	0.86016
559	0.83304	0.84136	0.84524	0.84653	0.85074	0.85530	0.86035
560	0.83325	0.84158	0.84545	0.84673	0.85094	0.85549	0.86054
561	0.83347	0.84179	0.84566	0.84694	0.85115	0.85569	0.86073
562	0.83372	0.84201	0.84587	0.84716	0.85135	0.85589	0.86092
563	0.83395	0.84223	0.84608	0.84737	0.85156	0.85609	0.86111
564	0.83418	0.84244	0.84630	0.84758	0.85176	0.85628	0.86130
565	0.83442	0.84267	0.84651	0.84779	0.85196	0.85648	0.86148
566	0.83463	0.84288	0.84672	0.84799	0.85216	0.85667	0.86167
567	0.83484	0.84309	0.84693	0.84820	0.85236	0.85686	0.86186
568	0.83505	0.84330	0.84713	0.84840	0.85256	0.85706	0.86204
569	0.83527	0.84351	0.84733	0.84861	0.85276	0.85725	0.86223
570	0.83551	0.84373	0.84754	0.84881	0.85295	0.85744	0.86241
571	0.83573	0.84394	0.84775	0.84902	0.85315	0.85763	0.86259
572	0.83596	0.84415	0.84796	0.84922	0.85335	0.85782	0.86278
573	0.83617	0.84435	0.84817	0.84943	0.85354	0.85801	0.86296
574	0.83641	0.84457	0.84836	0.84962	0.85374	0.85820	0.86314
575	0.83663	0.84477	0.84856	0.84983	0.85393	0.85839	0.86332
576	0.83683	0.84498	0.84877	0.85003	0.85413	0.85857	0.86350
577	0.83707	0.84519	0.84897	0.85023	0.85432	0.85876	0.86368
578	0.83728	0.84540	0.84917	0.85044	0.85452	0.85895	0.86386
579	0.83750	0.84560	0.84937	0.85063	0.85471	0.85914	0.86404
580	0.83773	0.84581	0.84957	0.85084	0.85490	0.85933	0.86422
581	0.83793	0.84601	0.84977	0.85103	0.85510	0.85951	0.86440
582	0.83815	0.84621	0.84997	0.85123	0.85529	0.85970	0.86458
583	0.83835	0.84641	0.85016	0.85143	0.85548	0.85988	0.86475
584	0.83858	0.84662	0.85036	0.85162	0.85567	0.86006	0.86493
585	0.83878	0.84682	0.85055	0.85181	0.85586	0.86025	0.86511
586	0.83901	0.84703	0.85075	0.85201	0.85605	0.86043	0.86528
587	0.83921	0.84724	0.85095	0.85220	0.85624	0.86061	0.86546
588	0.83943	0.84743	0.85116	0.85239	0.85642	0.86079	0.86563
589	0.83964	0.84763	0.85135	0.85258	0.85661	0.86097	0.86581
590	0.83984	0.84783	0.85154	0.85278	0.85680	0.86115	0.86598
591	0.84007	0.84802	0.85173	0.85297	0.85699	0.86134	0.86616
592	0.84028	0.84823	0.85194	0.85317	0.85717	0.86151	0.86633
593	0.84049	0.84843	0.85213	0.85336	0.85736	0.86170	0.86650
594	0.84071	0.84863	0.85233	0.85356	0.85755	0.86187	0.86667
595	0.84092	0.84882	0.85252	0.85374	0.85773	0.86205	0.86684
596	0.84114	0.84902	0.85270	0.85394	0.85792	0.86223	0.86701
597	0.84133	0.84922	0.85290	0.85414	0.85810	0.86240	0.86718

N	0.1%	0.5%	1%	1.25%	2.5%	5%	10%
598	0.84154	0.84941	0.85309	0.85432	0.85828	0.86258	0.86735
599	0.84175	0.84961	0.85328	0.85451	0.85847	0.86275	0.86752
600	0.84195	0.84980	0.85347	0.85469	0.85865	0.86293	0.86769
601	0.84213	0.84999	0.85365	0.85488	0.85883	0.86311	0.86786
602	0.84234	0.85019	0.85384	0.85506	0.85901	0.86328	0.86803
603	0.84255	0.85039	0.85403	0.85525	0.85919	0.86346	0.86820
604	0.84275	0.85058	0.85421	0.85543	0.85937	0.86363	0.86836
605	0.84291	0.85078	0.85440	0.85562	0.85955	0.86380	0.86853
606	0.84314	0.85097	0.85458	0.85580	0.85973	0.86398	0.86870
607	0.84336	0.85115	0.85478	0.85598	0.85990	0.86415	0.86886
608	0.84356	0.85135	0.85495	0.85617	0.86008	0.86432	0.86903
609	0.84376	0.85153	0.85514	0.85635	0.86026	0.86449	0.86919
610	0.84395	0.85173	0.85533	0.85653	0.86044	0.86466	0.86936
611	0.84415	0.85191	0.85551	0.85671	0.86061	0.86483	0.86952
612	0.84436	0.85210	0.85569	0.85690	0.86079	0.86501	0.86968
613	0.84456	0.85229	0.85587	0.85707	0.86097	0.86518	0.86985
614	0.84475	0.85248	0.85605	0.85725	0.86114	0.86535	0.87001
615	0.84494	0.85266	0.85623	0.85743	0.86132	0.86551	0.87017
616	0.84514	0.85284	0.85641	0.85761	0.86149	0.86568	0.87033
617	0.84534	0.85303	0.85660	0.85779	0.86167	0.86585	0.87049
618	0.84554	0.85322	0.85678	0.85797	0.86184	0.86602	0.87066
619	0.84575	0.85340	0.85696	0.85815	0.86202	0.86618	0.87081
620	0.84595	0.85359	0.85714	0.85833	0.86219	0.86635	0.87097
621	0.84613	0.85377	0.85732	0.85851	0.86236	0.86652	0.87113
622	0.84633	0.85396	0.85750	0.85869	0.86253	0.86668	0.87129
623	0.84653	0.85414	0.85767	0.85887	0.86270	0.86685	0.87145
624	0.84672	0.85432	0.85785	0.85904	0.86287	0.86701	0.87161
625	0.84692	0.85449	0.85803	0.85922	0.86304	0.86717	0.87177
626	0.84711	0.85468	0.85820	0.85939	0.86321	0.86734	0.87193
627	0.84730	0.85486	0.85838	0.85957	0.86338	0.86750	0.87208
628	0.84749	0.85504	0.85855	0.85974	0.86355	0.86767	0.87224
629	0.84768	0.85521	0.85873	0.85992	0.86372	0.86783	0.87240
630	0.84788	0.85539	0.85890	0.86009	0.86388	0.86799	0.87255
631	0.84806	0.85558	0.85907	0.86026	0.86405	0.86815	0.87271
632	0.84823	0.85575	0.85925	0.86043	0.86422	0.86831	0.87286
633	0.84841	0.85593	0.85942	0.86060	0.86438	0.86848	0.87301
634	0.84860	0.85611	0.85959	0.86076	0.86455	0.86864	0.87317
635	0.84880	0.85628	0.85976	0.86093	0.86471	0.86880	0.87332
636	0.84899	0.85645	0.85993	0.86110	0.86488	0.86896	0.87347
637	0.84918	0.85663	0.86011	0.86127	0.86504	0.86912	0.87363
638	0.84936	0.85680	0.86028	0.86144	0.86520	0.86927	0.87378
639	0.84953	0.85698	0.86045	0.86161	0.86537	0.86943	0.87393
640	0.84972	0.85716	0.86062	0.86178	0.86553	0.86959	0.87409
641	0.84990	0.85733	0.86079	0.86194	0.86569	0.86975	0.87424
642	0.85008	0.85750	0.86096	0.86210	0.86586	0.86990	0.87439
643	0.85025	0.85768	0.86112	0.86227	0.86602	0.87006	0.87454
644	0.85044	0.85785	0.86129	0.86245	0.86618	0.87022	0.87468
645	0.85061	0.85801	0.86146	0.86260	0.86633	0.87037	0.87483
646	0.85079	0.85819	0.86162	0.86277	0.86650	0.87052	0.87498
647	0.85097	0.85835	0.86179	0.86294	0.86666	0.87068	0.87513
648	0.85116	0.85853	0.86196	0.86310	0.86681	0.87084	0.87528
649	0.85134	0.85870	0.86212	0.86327	0.86698	0.87099	0.87543
650	0.85152	0.85886	0.86229	0.86343	0.86714	0.87114	0.87558
651	0.85170	0.85904	0.86245	0.86360	0.86729	0.87130	0.87572
652	0.85187	0.85921	0.86262	0.86376	0.86745	0.87145	0.87587
653	0.85207	0.85937	0.86278	0.86392	0.86761	0.87160	0.87602
654	0.85224	0.85954	0.86295	0.86408	0.86777	0.87175	0.87616
655	0.85241	0.85971	0.86311	0.86424	0.86792	0.87190	0.87631
656	0.85259	0.85987	0.86328	0.86440	0.86808	0.87206	0.87645
657	0.85277	0.86004	0.86343	0.86457	0.86824	0.87221	0.87660
658	0.85294	0.86020	0.86359	0.86473	0.86839	0.87236	0.87674
659	0.85312	0.86037	0.86376	0.86489	0.86855	0.87250	0.87689
660	0.85329	0.86054	0.86391	0.86505	0.86870	0.87265	0.87703
661	0.85344	0.86070	0.86407	0.86521	0.86885	0.87280	0.87717
662	0.85362	0.86086	0.86423	0.86537	0.86901	0.87295	0.87732
663	0.85379	0.86103	0.86439	0.86553	0.86916	0.87310	0.87746
664	0.85395	0.86119	0.86455	0.86569	0.86931	0.87325	0.87760
665	0.85413	0.86136	0.86471	0.86585	0.86947	0.87340	0.87775
666	0.85431	0.86152	0.86487	0.86600	0.86962	0.87354	0.87789
667	0.85447	0.86168	0.86503	0.86616	0.86977	0.87369	0.87803
668	0.85465	0.86184	0.86519	0.86631	0.86992	0.87383	0.87817
669	0.85482	0.86201	0.86535	0.86647	0.87007	0.87398	0.87831
670	0.85499	0.86216	0.86550	0.86662	0.87022	0.87413	0.87845
671	0.85517	0.86233	0.86566	0.86678	0.87038	0.87427	0.87859

N	0.1%	0.5%	1%	1.25%	2.5%	5%	10%
672	0.85534	0.86248	0.86582	0.86693	0.87052	0.87441	0.87873
673	0.85549	0.86265	0.86597	0.86709	0.87067	0.87456	0.87886
674	0.85568	0.86280	0.86613	0.86724	0.87082	0.87470	0.87900
675	0.85584	0.86297	0.86628	0.86740	0.87097	0.87485	0.87914
676	0.85601	0.86313	0.86644	0.86755	0.87112	0.87499	0.87928
677	0.85618	0.86328	0.86659	0.86770	0.87127	0.87514	0.87941
678	0.85636	0.86345	0.86675	0.86785	0.87142	0.87528	0.87955
679	0.85653	0.86361	0.86691	0.86801	0.87156	0.87542	0.87969
680	0.85669	0.86377	0.86705	0.86815	0.87171	0.87556	0.87983
681	0.85685	0.86392	0.86721	0.86830	0.87185	0.87571	0.87996
682	0.85700	0.86408	0.86736	0.86845	0.87200	0.87585	0.88010
683	0.85717	0.86424	0.86751	0.86860	0.87215	0.87599	0.88024
684	0.85734	0.86440	0.86766	0.86876	0.87229	0.87613	0.88037
685	0.85750	0.86454	0.86781	0.86891	0.87244	0.87627	0.88051
686	0.85766	0.86469	0.86796	0.86905	0.87258	0.87641	0.88064
687	0.85783	0.86485	0.86811	0.86920	0.87273	0.87654	0.88078
688	0.85799	0.86500	0.86826	0.86935	0.87287	0.87668	0.88091
689	0.85816	0.86515	0.86841	0.86950	0.87302	0.87682	0.88104
690	0.85831	0.86530	0.86856	0.86965	0.87316	0.87696	0.88118
691	0.85846	0.86546	0.86871	0.86980	0.87331	0.87710	0.88131
692	0.85865	0.86561	0.86885	0.86994	0.87345	0.87723	0.88144
693	0.85882	0.86577	0.86900	0.87009	0.87359	0.87737	0.88158
694	0.85899	0.86592	0.86915	0.87023	0.87373	0.87751	0.88171
695	0.85914	0.86607	0.86930	0.87038	0.87388	0.87765	0.88184
696	0.85929	0.86622	0.86945	0.87053	0.87402	0.87778	0.88197
697	0.85944	0.86638	0.86959	0.87068	0.87416	0.87792	0.88210
698	0.85960	0.86653	0.86974	0.87082	0.87430	0.87806	0.88223
699	0.85976	0.86668	0.86989	0.87096	0.87444	0.87819	0.88236
700	0.85991	0.86682	0.87004	0.87111	0.87458	0.87833	0.88249
701	0.86009	0.86697	0.87018	0.87125	0.87472	0.87846	0.88262
702	0.86024	0.86712	0.87032	0.87139	0.87486	0.87860	0.88275
703	0.86039	0.86727	0.87047	0.87153	0.87499	0.87873	0.88288
704	0.86056	0.86742	0.87061	0.87168	0.87513	0.87886	0.88301
705	0.86071	0.86756	0.87075	0.87182	0.87527	0.87900	0.88314
706	0.86086	0.86772	0.87090	0.87196	0.87541	0.87913	0.88326
707	0.86101	0.86786	0.87104	0.87210	0.87555	0.87927	0.88339
708	0.86115	0.86800	0.87118	0.87224	0.87568	0.87940	0.88352
709	0.86131	0.86815	0.87132	0.87238	0.87582	0.87953	0.88365
710	0.86146	0.86830	0.87146	0.87252	0.87596	0.87966	0.88377
711	0.86161	0.86844	0.87160	0.87266	0.87610	0.87979	0.88390
712	0.86178	0.86858	0.87174	0.87280	0.87623	0.87992	0.88402
713	0.86193	0.86873	0.87188	0.87294	0.87637	0.88006	0.88415
714	0.86208	0.86887	0.87202	0.87307	0.87650	0.88019	0.88428
715	0.86224	0.86902	0.87217	0.87321	0.87664	0.88032	0.88440
716	0.86240	0.86917	0.87230	0.87335	0.87677	0.88045	0.88453
717	0.86256	0.86930	0.87244	0.87349	0.87690	0.88058	0.88465
718	0.86272	0.86945	0.87258	0.87363	0.87704	0.88071	0.88477
719	0.86286	0.86959	0.87272	0.87377	0.87717	0.88084	0.88490
720	0.86302	0.86973	0.87286	0.87390	0.87730	0.88097	0.88502
721	0.86318	0.86988	0.87300	0.87404	0.87744	0.88109	0.88515
722	0.86335	0.87002	0.87314	0.87418	0.87757	0.88122	0.88527
723	0.86350	0.87016	0.87327	0.87431	0.87770	0.88135	0.88539
724	0.86362	0.87029	0.87341	0.87445	0.87783	0.88148	0.88551
725	0.86377	0.87043	0.87355	0.87458	0.87797	0.88160	0.88564
726	0.86392	0.87058	0.87368	0.87472	0.87810	0.88173	0.88576
727	0.86407	0.87072	0.87382	0.87486	0.87823	0.88186	0.88588
728	0.86423	0.87086	0.87396	0.87499	0.87836	0.88198	0.88600
729	0.86438	0.87101	0.87410	0.87513	0.87848	0.88211	0.88612
730	0.86451	0.87114	0.87423	0.87526	0.87862	0.88223	0.88624
731	0.86466	0.87128	0.87437	0.87540	0.87875	0.88236	0.88636
732	0.86481	0.87142	0.87451	0.87554	0.87888	0.88248	0.88648
733	0.86495	0.87156	0.87464	0.87567	0.87900	0.88261	0.88661
734	0.86509	0.87169	0.87478	0.87581	0.87913	0.88273	0.88672
735	0.86524	0.87184	0.87491	0.87594	0.87926	0.88286	0.88684
736	0.86539	0.87198	0.87504	0.87607	0.87939	0.88298	0.88696
737	0.86552	0.87211	0.87517	0.87621	0.87952	0.88311	0.88708
738	0.86567	0.87225	0.87531	0.87634	0.87965	0.88323	0.88720
739	0.86582	0.87239	0.87544	0.87647	0.87977	0.88335	0.88732
740	0.86595	0.87252	0.87557	0.87660	0.87990	0.88347	0.88744
741	0.86609	0.87265	0.87570	0.87673	0.88003	0.88360	0.88756
742	0.86623	0.87279	0.87584	0.87686	0.88016	0.88372	0.88767
743	0.86638	0.87293	0.87597	0.87699	0.88029	0.88384	0.88779
744	0.86653	0.87307	0.87610	0.87712	0.88041	0.88396	0.88791
745	0.86666	0.87321	0.87623	0.87725	0.88054	0.88408	0.88802

N	0.1%	0.5%	1%	1.25%	2.5%	5%	10%
746	0.86681	0.87334	0.87636	0.87738	0.88066	0.88421	0.88814
747	0.86696	0.87347	0.87649	0.87750	0.88079	0.88433	0.88825
748	0.86709	0.87361	0.87662	0.87763	0.88091	0.88445	0.88837
749	0.86724	0.87375	0.87675	0.87776	0.88104	0.88457	0.88849
750	0.86738	0.87388	0.87687	0.87788	0.88116	0.88469	0.88860
751	0.86752	0.87401	0.87700	0.87802	0.88129	0.88481	0.88872
752	0.86766	0.87414	0.87713	0.87814	0.88141	0.88492	0.88883
753	0.86782	0.87428	0.87726	0.87827	0.88153	0.88504	0.88895
754	0.86795	0.87440	0.87739	0.87839	0.88165	0.88516	0.88906
755	0.86809	0.87454	0.87751	0.87852	0.88178	0.88528	0.88918
756	0.86823	0.87467	0.87764	0.87865	0.88190	0.88540	0.88929
757	0.86838	0.87480	0.87777	0.87877	0.88202	0.88552	0.88941
758	0.86852	0.87493	0.87789	0.87890	0.88214	0.88564	0.88952
759	0.86865	0.87506	0.87802	0.87903	0.88227	0.88576	0.88963
760	0.86878	0.87519	0.87815	0.87915	0.88239	0.88588	0.88975
761	0.86892	0.87532	0.87828	0.87928	0.88251	0.88599	0.88986
762	0.86907	0.87545	0.87841	0.87940	0.88263	0.88611	0.88997
763	0.86918	0.87558	0.87853	0.87953	0.88275	0.88623	0.89008
764	0.86931	0.87570	0.87866	0.87965	0.88287	0.88634	0.89019
765	0.86944	0.87583	0.87878	0.87978	0.88299	0.88646	0.89031
766	0.86958	0.87596	0.87891	0.87990	0.88310	0.88658	0.89042
767	0.86971	0.87609	0.87903	0.88002	0.88323	0.88669	0.89053
768	0.86983	0.87621	0.87916	0.88014	0.88335	0.88680	0.89064
769	0.86998	0.87634	0.87928	0.88027	0.88346	0.88692	0.89075
770	0.87011	0.87647	0.87940	0.88039	0.88358	0.88703	0.89086
771	0.87024	0.87659	0.87953	0.88051	0.88370	0.88715	0.89097
772	0.87037	0.87672	0.87965	0.88063	0.88382	0.88727	0.89108
773	0.87051	0.87685	0.87977	0.88075	0.88394	0.88738	0.89119
774	0.87063	0.87697	0.87989	0.88088	0.88405	0.88749	0.89130
775	0.87076	0.87711	0.88002	0.88100	0.88417	0.88760	0.89140
776	0.87090	0.87723	0.88014	0.88112	0.88429	0.88772	0.89151
777	0.87104	0.87735	0.88026	0.88124	0.88441	0.88783	0.89162
778	0.87118	0.87748	0.88039	0.88136	0.88452	0.88794	0.89173
779	0.87132	0.87760	0.88051	0.88148	0.88464	0.88806	0.89184
780	0.87144	0.87772	0.88062	0.88160	0.88475	0.88817	0.89195
781	0.87156	0.87785	0.88074	0.88172	0.88487	0.88828	0.89205
782	0.87170	0.87797	0.88086	0.88184	0.88499	0.88839	0.89216
783	0.87182	0.87809	0.88098	0.88196	0.88510	0.88850	0.89227
784	0.87195	0.87821	0.88110	0.88208	0.88522	0.88861	0.89238
785	0.87208	0.87833	0.88122	0.88219	0.88533	0.88872	0.89248
786	0.87221	0.87846	0.88135	0.88232	0.88545	0.88884	0.89259
787	0.87233	0.87858	0.88147	0.88244	0.88556	0.88894	0.89270
788	0.87246	0.87870	0.88158	0.88255	0.88568	0.88906	0.89280
789	0.87260	0.87882	0.88170	0.88267	0.88579	0.88916	0.89291
790	0.87273	0.87895	0.88182	0.88278	0.88590	0.88928	0.89301
791	0.87286	0.87907	0.88194	0.88290	0.88602	0.88938	0.89312
792	0.87297	0.87919	0.88206	0.88302	0.88613	0.88950	0.89323
793	0.87310	0.87931	0.88217	0.88313	0.88625	0.88960	0.89333
794	0.87322	0.87943	0.88229	0.88325	0.88636	0.88971	0.89344
795	0.87335	0.87955	0.88240	0.88337	0.88647	0.88982	0.89354
796	0.87348	0.87967	0.88252	0.88348	0.88658	0.88993	0.89365
797	0.87361	0.87978	0.88264	0.88360	0.88669	0.89004	0.89375
798	0.87373	0.87990	0.88276	0.88371	0.88680	0.89015	0.89385
799	0.87385	0.88002	0.88287	0.88382	0.88691	0.89026	0.89396
800	0.87398	0.88014	0.88298	0.88394	0.88702	0.89036	0.89406
801	0.87410	0.88026	0.88310	0.88406	0.88713	0.89047	0.89416
802	0.87422	0.88038	0.88321	0.88417	0.88724	0.89058	0.89426
803	0.87434	0.88049	0.88332	0.88428	0.88735	0.89068	0.89436
804	0.87447	0.88061	0.88344	0.88440	0.88746	0.89079	0.89447
805	0.87460	0.88072	0.88355	0.88451	0.88757	0.89089	0.89457
806	0.87473	0.88084	0.88367	0.88462	0.88768	0.89100	0.89467
807	0.87484	0.88096	0.88378	0.88473	0.88779	0.89110	0.89477
808	0.87497	0.88108	0.88390	0.88485	0.88790	0.89121	0.89487
809	0.87509	0.88119	0.88401	0.88496	0.88801	0.89131	0.89498
810	0.87521	0.88131	0.88412	0.88508	0.88812	0.89142	0.89508
811	0.87534	0.88142	0.88423	0.88519	0.88823	0.89152	0.89518
812	0.87545	0.88154	0.88435	0.88530	0.88834	0.89163	0.89528
813	0.87557	0.88166	0.88446	0.88541	0.88844	0.89173	0.89538
814	0.87569	0.88177	0.88457	0.88552	0.88855	0.89184	0.89548
815	0.87582	0.88188	0.88469	0.88563	0.88866	0.89194	0.89558
816	0.87595	0.88200	0.88480	0.88574	0.88877	0.89205	0.89568
817	0.87607	0.88212	0.88491	0.88584	0.88887	0.89215	0.89578
818	0.87619	0.88223	0.88502	0.88595	0.88898	0.89225	0.89588
819	0.87631	0.88235	0.88514	0.88606	0.88909	0.89236	0.89598

N	0.1%	0.5%	1%	1.25%	2.5%	5%	10%
820	0.87644	0.88246	0.88525	0.88618	0.88920	0.89246	0.89608
821	0.87656	0.88257	0.88536	0.88628	0.88930	0.89256	0.89618
822	0.87668	0.88268	0.88547	0.88639	0.88940	0.89267	0.89627
823	0.87679	0.88280	0.88558	0.88650	0.88951	0.89277	0.89637
824	0.87691	0.88291	0.88569	0.88661	0.88962	0.89287	0.89647
825	0.87702	0.88303	0.88580	0.88672	0.88972	0.89297	0.89657
826	0.87714	0.88314	0.88591	0.88683	0.88982	0.89308	0.89667
827	0.87726	0.88324	0.88602	0.88694	0.88993	0.89318	0.89676
828	0.87739	0.88335	0.88613	0.88704	0.89004	0.89328	0.89686
829	0.87752	0.88346	0.88624	0.88715	0.89014	0.89338	0.89696
830	0.87764	0.88358	0.88634	0.88726	0.89025	0.89348	0.89706
831	0.87774	0.88368	0.88645	0.88736	0.89035	0.89358	0.89715
832	0.87787	0.88380	0.88656	0.88747	0.89046	0.89368	0.89725
833	0.87798	0.88391	0.88666	0.88758	0.89056	0.89378	0.89735
834	0.87810	0.88402	0.88677	0.88768	0.89066	0.89388	0.89744
835	0.87821	0.88413	0.88688	0.88779	0.89077	0.89398	0.89754
836	0.87833	0.88424	0.88699	0.88790	0.89087	0.89408	0.89763
837	0.87844	0.88435	0.88710	0.88800	0.89097	0.89418	0.89773
838	0.87857	0.88446	0.88720	0.88811	0.89107	0.89428	0.89782
839	0.87869	0.88457	0.88731	0.88822	0.89118	0.89438	0.89792
840	0.87880	0.88468	0.88741	0.88832	0.89128	0.89448	0.89801
841	0.87892	0.88479	0.88752	0.88843	0.89138	0.89458	0.89811
842	0.87904	0.88491	0.88763	0.88854	0.89148	0.89467	0.89820
843	0.87916	0.88501	0.88773	0.88864	0.89158	0.89477	0.89830
844	0.87926	0.88512	0.88784	0.88874	0.89168	0.89487	0.89839
845	0.87937	0.88522	0.88794	0.88885	0.89178	0.89497	0.89849
846	0.87949	0.88533	0.88805	0.88895	0.89189	0.89506	0.89858
847	0.87960	0.88543	0.88815	0.88905	0.89199	0.89516	0.89868
848	0.87972	0.88554	0.88826	0.88915	0.89209	0.89526	0.89877
849	0.87982	0.88565	0.88836	0.88926	0.89219	0.89536	0.89886
850	0.87993	0.88576	0.88846	0.88936	0.89229	0.89545	0.89895
851	0.88005	0.88586	0.88856	0.88946	0.89239	0.89555	0.89905
852	0.88017	0.88596	0.88867	0.88957	0.89249	0.89564	0.89914
853	0.88027	0.88607	0.88878	0.88967	0.89259	0.89574	0.89923
854	0.88038	0.88617	0.88888	0.88977	0.89269	0.89584	0.89933
855	0.88049	0.88628	0.88898	0.88988	0.89279	0.89593	0.89942
856	0.88061	0.88639	0.88909	0.88998	0.89288	0.89603	0.89951
857	0.88073	0.88649	0.88919	0.89008	0.89298	0.89613	0.89960
858	0.88085	0.88660	0.88930	0.89018	0.89308	0.89622	0.89969
859	0.88096	0.88671	0.88939	0.89029	0.89318	0.89632	0.89979
860	0.88106	0.88681	0.88950	0.89039	0.89328	0.89641	0.89988
861	0.88117	0.88692	0.88960	0.89049	0.89337	0.89651	0.89997
862	0.88129	0.88703	0.88970	0.89059	0.89347	0.89660	0.90006
863	0.88138	0.88713	0.88980	0.89069	0.89357	0.89670	0.90015
864	0.88149	0.88723	0.88990	0.89079	0.89367	0.89679	0.90024
865	0.88161	0.88733	0.89001	0.89089	0.89376	0.89688	0.90033
866	0.88171	0.88744	0.89011	0.89100	0.89386	0.89698	0.90042
867	0.88182	0.88754	0.89020	0.89110	0.89396	0.89707	0.90051
868	0.88194	0.88764	0.89031	0.89119	0.89405	0.89717	0.90060
869	0.88205	0.88775	0.89040	0.89130	0.89415	0.89726	0.90069
870	0.88216	0.88786	0.89050	0.89139	0.89425	0.89735	0.90078
871	0.88227	0.88796	0.89060	0.89149	0.89435	0.89744	0.90087
872	0.88238	0.88806	0.89070	0.89159	0.89444	0.89754	0.90096
873	0.88248	0.88816	0.89080	0.89168	0.89454	0.89763	0.90105
874	0.88259	0.88827	0.89089	0.89178	0.89463	0.89772	0.90114
875	0.88269	0.88837	0.89099	0.89188	0.89473	0.89781	0.90123
876	0.88279	0.88847	0.89109	0.89198	0.89482	0.89790	0.90131
877	0.88290	0.88857	0.89119	0.89207	0.89492	0.89800	0.90140
878	0.88301	0.88867	0.89129	0.89217	0.89501	0.89809	0.90149
879	0.88312	0.88877	0.89139	0.89227	0.89511	0.89818	0.90158
880	0.88323	0.88887	0.89149	0.89236	0.89520	0.89827	0.90167
881	0.88334	0.88897	0.89158	0.89246	0.89530	0.89836	0.90176
882	0.88345	0.88907	0.89168	0.89256	0.89539	0.89845	0.90184
883	0.88356	0.88917	0.89178	0.89266	0.89548	0.89854	0.90193
884	0.88366	0.88926	0.89187	0.89275	0.89558	0.89863	0.90202
885	0.88378	0.88936	0.89197	0.89285	0.89567	0.89872	0.90210
886	0.88388	0.88947	0.89207	0.89294	0.89576	0.89881	0.90219
887	0.88398	0.88956	0.89217	0.89304	0.89585	0.89890	0.90228
888	0.88407	0.88966	0.89226	0.89314	0.89595	0.89899	0.90236
889	0.88418	0.88976	0.89236	0.89323	0.89604	0.89908	0.90245
890	0.88428	0.88985	0.89246	0.89333	0.89613	0.89917	0.90254
891	0.88438	0.88995	0.89255	0.89342	0.89622	0.89926	0.90262
892	0.88449	0.89005	0.89265	0.89352	0.89632	0.89935	0.90271
893	0.88460	0.89015	0.89274	0.89361	0.89641	0.89944	0.90279

N	0.1%	0.5%	1%	1.25%	2.5%	5%	10%
894	0.88471	0.89024	0.89283	0.89371	0.89650	0.89953	0.90288
895	0.88481	0.89034	0.89293	0.89380	0.89659	0.89962	0.90296
896	0.88491	0.89044	0.89303	0.89389	0.89668	0.89971	0.90305
897	0.88502	0.89054	0.89312	0.89399	0.89678	0.89980	0.90313
898	0.88512	0.89064	0.89322	0.89408	0.89687	0.89988	0.90322
899	0.88522	0.89073	0.89331	0.89417	0.89696	0.89997	0.90330
900	0.88533	0.89083	0.89341	0.89426	0.89705	0.90006	0.90339
901	0.88543	0.89092	0.89350	0.89436	0.89714	0.90014	0.90347
902	0.88553	0.89102	0.89360	0.89445	0.89723	0.90023	0.90355
903	0.88563	0.89112	0.89369	0.89454	0.89732	0.90032	0.90364
904	0.88572	0.89122	0.89379	0.89464	0.89741	0.90041	0.90372
905	0.88582	0.89132	0.89388	0.89473	0.89750	0.90049	0.90381
906	0.88593	0.89141	0.89397	0.89483	0.89759	0.90058	0.90389
907	0.88603	0.89151	0.89406	0.89492	0.89768	0.90067	0.90397
908	0.88614	0.89161	0.89416	0.89501	0.89777	0.90075	0.90406
909	0.88624	0.89170	0.89425	0.89510	0.89786	0.90084	0.90414
910	0.88634	0.89179	0.89434	0.89519	0.89795	0.90092	0.90422
911	0.88644	0.89189	0.89444	0.89528	0.89804	0.90101	0.90430
912	0.88655	0.89199	0.89453	0.89538	0.89813	0.90110	0.90439
913	0.88664	0.89208	0.89462	0.89547	0.89821	0.90118	0.90447
914	0.88674	0.89217	0.89471	0.89556	0.89830	0.90127	0.90455
915	0.88684	0.89227	0.89480	0.89565	0.89839	0.90135	0.90463
916	0.88693	0.89236	0.89489	0.89574	0.89848	0.90143	0.90471
917	0.88702	0.89246	0.89498	0.89583	0.89857	0.90152	0.90480
918	0.88712	0.89255	0.89507	0.89592	0.89865	0.90160	0.90488
919	0.88722	0.89264	0.89517	0.89601	0.89874	0.90169	0.90496
920	0.88733	0.89274	0.89526	0.89610	0.89883	0.90178	0.90504
921	0.88742	0.89283	0.89535	0.89619	0.89892	0.90186	0.90512
922	0.88752	0.89292	0.89544	0.89628	0.89900	0.90194	0.90520
923	0.88761	0.89302	0.89553	0.89637	0.89909	0.90203	0.90528
924	0.88771	0.89311	0.89562	0.89646	0.89918	0.90211	0.90536
925	0.88781	0.89321	0.89571	0.89655	0.89927	0.90219	0.90544
926	0.88791	0.89329	0.89580	0.89664	0.89935	0.90228	0.90552
927	0.88801	0.89339	0.89589	0.89673	0.89944	0.90236	0.90560
928	0.88811	0.89349	0.89598	0.89682	0.89952	0.90244	0.90568
929	0.88820	0.89357	0.89607	0.89691	0.89961	0.90253	0.90576
930	0.88828	0.89367	0.89615	0.89700	0.89969	0.90261	0.90584
931	0.88839	0.89375	0.89624	0.89708	0.89978	0.90269	0.90592
932	0.88848	0.89385	0.89633	0.89717	0.89986	0.90277	0.90600
933	0.88857	0.89394	0.89642	0.89726	0.89995	0.90285	0.90608
934	0.88868	0.89402	0.89651	0.89735	0.90003	0.90294	0.90616
935	0.88877	0.89412	0.89660	0.89744	0.90012	0.90302	0.90624
936	0.88887	0.89421	0.89669	0.89752	0.90020	0.90310	0.90632
937	0.88897	0.89430	0.89678	0.89761	0.90029	0.90318	0.90640
938	0.88907	0.89438	0.89687	0.89770	0.90037	0.90326	0.90648
939	0.88917	0.89448	0.89696	0.89779	0.90046	0.90334	0.90655
940	0.88926	0.89457	0.89704	0.89787	0.90054	0.90342	0.90663
941	0.88935	0.89466	0.89713	0.89796	0.90062	0.90351	0.90671
942	0.88944	0.89475	0.89722	0.89804	0.90071	0.90359	0.90679
943	0.88953	0.89484	0.89730	0.89813	0.90079	0.90367	0.90687
944	0.88962	0.89493	0.89739	0.89822	0.90088	0.90375	0.90695
945	0.88972	0.89501	0.89747	0.89830	0.90096	0.90383	0.90702
946	0.88982	0.89510	0.89756	0.89839	0.90104	0.90391	0.90710
947	0.88991	0.89519	0.89765	0.89847	0.90112	0.90399	0.90718
948	0.89001	0.89528	0.89774	0.89856	0.90121	0.90407	0.90725
949	0.89009	0.89537	0.89782	0.89864	0.90129	0.90415	0.90733
950	0.89019	0.89546	0.89791	0.89873	0.90137	0.90423	0.90741
951	0.89028	0.89554	0.89799	0.89881	0.90145	0.90431	0.90748
952	0.89038	0.89563	0.89808	0.89890	0.90153	0.90439	0.90756
953	0.89047	0.89572	0.89816	0.89898	0.90162	0.90447	0.90764
954	0.89056	0.89580	0.89825	0.89907	0.90170	0.90455	0.90771
955	0.89065	0.89590	0.89833	0.89915	0.90178	0.90463	0.90779
956	0.89074	0.89598	0.89842	0.89923	0.90186	0.90471	0.90786
957	0.89083	0.89606	0.89850	0.89931	0.90194	0.90479	0.90794
958	0.89092	0.89615	0.89859	0.89940	0.90202	0.90487	0.90802
959	0.89101	0.89624	0.89867	0.89948	0.90211	0.90494	0.90809
960	0.89109	0.89633	0.89875	0.89956	0.90219	0.90502	0.90817
961	0.89120	0.89641	0.89884	0.89965	0.90227	0.90510	0.90824
962	0.89128	0.89650	0.89892	0.89973	0.90235	0.90518	0.90832
963	0.89137	0.89659	0.89901	0.89982	0.90243	0.90526	0.90839
964	0.89146	0.89668	0.89909	0.89990	0.90251	0.90533	0.90847
965	0.89155	0.89676	0.89917	0.89998	0.90259	0.90541	0.90854
966	0.89164	0.89685	0.89925	0.90006	0.90267	0.90549	0.90862
967	0.89174	0.89693	0.89934	0.90014	0.90275	0.90556	0.90869

N	0.1%	0.5%	1%	1.25%	2.5%	5%	10%
968	0.89183	0.89701	0.89942	0.90022	0.90283	0.90564	0.90877
969	0.89192	0.89710	0.89950	0.90031	0.90291	0.90572	0.90884
970	0.89201	0.89718	0.89959	0.90039	0.90299	0.90580	0.90892
971	0.89211	0.89727	0.89967	0.90047	0.90307	0.90587	0.90899
972	0.89219	0.89735	0.89975	0.90055	0.90315	0.90595	0.90906
973	0.89227	0.89744	0.89984	0.90064	0.90323	0.90603	0.90914
974	0.89237	0.89752	0.89991	0.90072	0.90331	0.90610	0.90921
975	0.89244	0.89760	0.90000	0.90080	0.90339	0.90618	0.90928
976	0.89254	0.89769	0.90008	0.90088	0.90346	0.90626	0.90936
977	0.89263	0.89777	0.90016	0.90096	0.90354	0.90633	0.90943
978	0.89272	0.89785	0.90024	0.90104	0.90362	0.90641	0.90950
979	0.89280	0.89794	0.90032	0.90112	0.90370	0.90649	0.90958
980	0.89291	0.89802	0.90041	0.90120	0.90378	0.90656	0.90965
981	0.89298	0.89810	0.90049	0.90128	0.90385	0.90664	0.90972
982	0.89307	0.89819	0.90057	0.90136	0.90393	0.90671	0.90979
983	0.89316	0.89827	0.90065	0.90144	0.90401	0.90679	0.90987
984	0.89325	0.89836	0.90073	0.90152	0.90409	0.90686	0.90994
985	0.89334	0.89844	0.90081	0.90160	0.90417	0.90694	0.91001
986	0.89343	0.89852	0.90089	0.90168	0.90424	0.90701	0.91008
987	0.89353	0.89860	0.90097	0.90176	0.90432	0.90709	0.91015
988	0.89361	0.89869	0.90105	0.90184	0.90440	0.90716	0.91023
989	0.89370	0.89877	0.90113	0.90192	0.90448	0.90724	0.91030
990	0.89378	0.89885	0.90121	0.90200	0.90455	0.90731	0.91037
991	0.89387	0.89893	0.90129	0.90208	0.90463	0.90739	0.91044
992	0.89395	0.89901	0.90137	0.90216	0.90471	0.90746	0.91051
993	0.89404	0.89910	0.90145	0.90224	0.90478	0.90753	0.91058
994	0.89413	0.89918	0.90153	0.90231	0.90486	0.90761	0.91065
995	0.89421	0.89926	0.90161	0.90239	0.90494	0.90768	0.91073
996	0.89429	0.89934	0.90169	0.90247	0.90501	0.90775	0.91080
997	0.89439	0.89942	0.90177	0.90255	0.90509	0.90783	0.91087
998	0.89447	0.89950	0.90185	0.90263	0.90516	0.90790	0.91094
999	0.89455	0.89958	0.90192	0.90271	0.90524	0.90798	0.91101
1000	0.89463	0.89966	0.90200	0.90279	0.90531	0.90805	0.91108

Table 4 – *GM10(7H3L or 3H7L)*. Critical values of upper significance levels for the Grubbs multiple-10 outlier test, applicable in situations where the seven highest and three lowest or three highest and seven lowest observations are simultaneously identified as outliers, denoted as *GM10(7H3L)* and *GM10(3H7L)*, respectively.

N	0.1%	0.5%	1%	1.25%	2.5%	5%	10%
12	0.00000	0.00000	0.00000	0.00000	0.00000	0.00001	0.00003
13	0.00001	0.00003	0.00006	0.00008	0.00017	0.00036	0.00078
14	0.00010	0.00031	0.00051	0.00060	0.00100	0.00171	0.00301
15	0.00048	0.00114	0.00168	0.00191	0.00286	0.00435	0.00681
16	0.00133	0.00272	0.00376	0.00417	0.00582	0.00827	0.01203
17	0.00277	0.00513	0.00675	0.00737	0.00985	0.01333	0.01845
18	0.00487	0.00834	0.01062	0.01151	0.01483	0.01939	0.02585
19	0.00765	0.01231	0.01529	0.01643	0.02064	0.02628	0.03401
20	0.01105	0.01703	0.02071	0.02210	0.02719	0.03383	0.04278
21	0.01501	0.02230	0.02675	0.02838	0.03431	0.04192	0.05202
22	0.01951	0.02818	0.03327	0.03515	0.04188	0.05042	0.06159
23	0.02444	0.03444	0.04023	0.04234	0.04986	0.05923	0.07138
24	0.03000	0.04115	0.04754	0.04988	0.05807	0.06826	0.08134
25	0.03574	0.04813	0.05513	0.05768	0.06657	0.07747	0.09137
26	0.04189	0.05542	0.06297	0.06568	0.07516	0.08680	0.10142
27	0.04819	0.06290	0.07094	0.07382	0.08392	0.09618	0.11147
28	0.05470	0.07051	0.07908	0.08209	0.09275	0.10554	0.12147
29	0.06148	0.07819	0.08723	0.09041	0.10157	0.11489	0.13137
30	0.06836	0.08596	0.09546	0.09882	0.11036	0.12421	0.14116
31	0.07543	0.09379	0.10367	0.10711	0.11916	0.13344	0.15086
32	0.08249	0.10169	0.11191	0.11549	0.12792	0.14261	0.16040
33	0.08977	0.10963	0.12012	0.12386	0.13666	0.15169	0.16981
34	0.09696	0.11753	0.12836	0.13219	0.14526	0.16063	0.17909
35	0.10423	0.12541	0.13653	0.14043	0.15381	0.16946	0.18821
36	0.11146	0.13325	0.14462	0.14864	0.16229	0.17816	0.19714
37	0.11873	0.14099	0.15266	0.15671	0.17063	0.18669	0.20591
38	0.12578	0.14870	0.16056	0.16468	0.17883	0.19508	0.21451
39	0.13300	0.15630	0.16841	0.17264	0.18686	0.20335	0.22296
40	0.13992	0.16386	0.17612	0.18041	0.19486	0.21149	0.23125
41	0.14701	0.17138	0.18379	0.18811	0.20271	0.21949	0.23937
42	0.15398	0.17873	0.19130	0.19567	0.21045	0.22737	0.24736
43	0.16102	0.18603	0.19877	0.20314	0.21805	0.23509	0.25511

N	0.1%	0.5%	1%	1.25%	2.5%	5%	10%
44	0.16785	0.19325	0.20606	0.21053	0.22551	0.24265	0.26278
45	0.17472	0.20037	0.21330	0.21778	0.23285	0.25009	0.27026
46	0.18158	0.20727	0.22038	0.22491	0.24005	0.25739	0.27760
47	0.18810	0.21429	0.22739	0.23190	0.24719	0.26450	0.28480
48	0.19481	0.22116	0.23431	0.23888	0.25414	0.27151	0.29184
49	0.20125	0.22780	0.24106	0.24563	0.26098	0.27841	0.29874
50	0.20773	0.23449	0.24772	0.25231	0.26773	0.28514	0.30554
51	0.21410	0.24093	0.25428	0.25890	0.27436	0.29179	0.31215
52	0.22037	0.24736	0.26073	0.26538	0.28082	0.29830	0.31865
53	0.22664	0.25363	0.26704	0.27169	0.28718	0.30469	0.32500
54	0.23267	0.25984	0.27331	0.27797	0.29346	0.31099	0.33125
55	0.23866	0.26600	0.27948	0.28413	0.29962	0.31715	0.33739
56	0.24463	0.27195	0.28550	0.29020	0.30568	0.32318	0.34340
57	0.25059	0.27787	0.29151	0.29615	0.31164	0.32912	0.34929
58	0.25632	0.28374	0.29738	0.30199	0.31751	0.33492	0.35506
59	0.26198	0.28952	0.30313	0.30778	0.32322	0.34064	0.36076
60	0.26763	0.29522	0.30877	0.31339	0.32885	0.34624	0.36629
61	0.27309	0.30075	0.31436	0.31902	0.33440	0.35177	0.37175
62	0.27863	0.30622	0.31982	0.32449	0.33987	0.35718	0.37713
63	0.28388	0.31162	0.32519	0.32986	0.34526	0.36250	0.38237
64	0.28921	0.31693	0.33045	0.33510	0.35050	0.36771	0.38755
65	0.29438	0.32214	0.33570	0.34032	0.35567	0.37283	0.39260
66	0.29952	0.32722	0.34084	0.34544	0.36079	0.37787	0.39757
67	0.30465	0.33230	0.34590	0.35045	0.36575	0.38282	0.40245
68	0.30968	0.33732	0.35078	0.35537	0.37068	0.38770	0.40724
69	0.31454	0.34222	0.35564	0.36029	0.37550	0.39250	0.41197
70	0.31948	0.34701	0.36049	0.36510	0.38028	0.39722	0.41659
71	0.32434	0.35184	0.36526	0.36989	0.38495	0.40186	0.42115
72	0.32892	0.35655	0.36993	0.37452	0.38955	0.40640	0.42562
73	0.33353	0.36114	0.37452	0.37908	0.39409	0.41087	0.43003
74	0.33813	0.36570	0.37908	0.38359	0.39851	0.41526	0.43432
75	0.34266	0.37015	0.38354	0.38805	0.40295	0.41960	0.43858
76	0.34704	0.37460	0.38790	0.39241	0.40725	0.42386	0.44274
77	0.35169	0.37894	0.39224	0.39671	0.41150	0.42803	0.44687
78	0.35598	0.38325	0.39646	0.40093	0.41569	0.43215	0.45092
79	0.36019	0.38746	0.40066	0.40508	0.41983	0.43622	0.45490
80	0.36434	0.39164	0.40475	0.40919	0.42389	0.44023	0.45881
81	0.36861	0.39570	0.40876	0.41322	0.42788	0.44416	0.46266
82	0.37278	0.39969	0.41284	0.41723	0.43182	0.44802	0.46646
83	0.37677	0.40368	0.41676	0.42116	0.43569	0.45185	0.47019
84	0.38066	0.40761	0.42064	0.42505	0.43955	0.45559	0.47385
85	0.38462	0.41150	0.42447	0.42887	0.44329	0.45930	0.47746
86	0.38853	0.41531	0.42825	0.43260	0.44702	0.46294	0.48103
87	0.39239	0.41901	0.43199	0.43632	0.45066	0.46652	0.48453
88	0.39622	0.42279	0.43565	0.43998	0.45428	0.47006	0.48799
89	0.39991	0.42645	0.43922	0.44356	0.45782	0.47352	0.49136
90	0.40352	0.43012	0.44284	0.44714	0.46134	0.47696	0.49471
91	0.40724	0.43371	0.44637	0.45068	0.46478	0.48036	0.49801
92	0.41097	0.43721	0.44985	0.45412	0.46817	0.48369	0.50127
93	0.41447	0.44065	0.45326	0.45750	0.47152	0.48697	0.50449
94	0.41794	0.44410	0.45664	0.46087	0.47484	0.49019	0.50764
95	0.42130	0.44746	0.45992	0.46419	0.47806	0.49338	0.51077
96	0.42472	0.45078	0.46327	0.46748	0.48131	0.49654	0.51383
97	0.42823	0.45411	0.46647	0.47070	0.48448	0.49963	0.51685
98	0.43145	0.45736	0.46971	0.47389	0.48763	0.50272	0.51984
99	0.43492	0.46058	0.47286	0.47706	0.49070	0.50572	0.52278
100	0.43807	0.46373	0.47597	0.48016	0.49374	0.50872	0.52571
101	0.44124	0.46694	0.47904	0.48321	0.49678	0.51166	0.52856
102	0.44453	0.47000	0.48212	0.48624	0.49972	0.51459	0.53138
103	0.44758	0.47304	0.48513	0.48920	0.50265	0.51745	0.53419
104	0.45063	0.47605	0.48812	0.49216	0.50555	0.52028	0.53694
105	0.45369	0.47904	0.49105	0.49510	0.50841	0.52306	0.53965
106	0.45678	0.48197	0.49398	0.49800	0.51123	0.52582	0.54232
107	0.45968	0.48488	0.49680	0.50084	0.51403	0.52854	0.54497
108	0.46255	0.48779	0.49964	0.50364	0.51677	0.53123	0.54759
109	0.46561	0.49063	0.50242	0.50641	0.51947	0.53388	0.55016
110	0.46855	0.49345	0.50516	0.50914	0.52211	0.53650	0.55270
111	0.47143	0.49621	0.50788	0.51185	0.52475	0.53910	0.55520
112	0.47433	0.49896	0.51057	0.51453	0.52738	0.54165	0.55768
113	0.47717	0.50163	0.51322	0.51714	0.52997	0.54417	0.56014
114	0.47986	0.50428	0.51584	0.51974	0.53254	0.54666	0.56255
115	0.48252	0.50692	0.51842	0.52230	0.53505	0.54910	0.56493
116	0.48521	0.50950	0.52098	0.52488	0.53753	0.55153	0.56728
117	0.48792	0.51205	0.52355	0.52740	0.54001	0.55394	0.56960

N	0.1%	0.5%	1%	1.25%	2.5%	5%	10%
118	0.49053	0.51460	0.52606	0.52988	0.54245	0.55629	0.57191
119	0.49311	0.51712	0.52854	0.53235	0.54483	0.55862	0.57418
120	0.49573	0.51961	0.53097	0.53479	0.54721	0.56095	0.57641
121	0.49834	0.52208	0.53337	0.53717	0.54957	0.56324	0.57864
122	0.50078	0.52450	0.53575	0.53954	0.55189	0.56551	0.58083
123	0.50324	0.52697	0.53812	0.54186	0.55419	0.56773	0.58300
124	0.50576	0.52929	0.54045	0.54422	0.55646	0.56994	0.58514
125	0.50826	0.53158	0.54274	0.54650	0.55871	0.57214	0.58727
126	0.51056	0.53393	0.54503	0.54876	0.56090	0.57429	0.58936
127	0.51296	0.53623	0.54726	0.55099	0.56312	0.57645	0.59143
128	0.51536	0.53852	0.54953	0.55322	0.56526	0.57856	0.59347
129	0.51760	0.54081	0.55171	0.55539	0.56742	0.58065	0.59551
130	0.51981	0.54301	0.55386	0.55753	0.56955	0.58273	0.59750
131	0.52214	0.54518	0.55603	0.55968	0.57166	0.58476	0.59948
132	0.52440	0.54738	0.55816	0.56181	0.57372	0.58677	0.60144
133	0.52671	0.54954	0.56026	0.56392	0.57578	0.58878	0.60339
134	0.52898	0.55169	0.56234	0.56599	0.57780	0.59076	0.60530
135	0.53126	0.55380	0.56444	0.56805	0.57979	0.59270	0.60719
136	0.53339	0.55588	0.56650	0.57009	0.58181	0.59464	0.60906
137	0.53557	0.55796	0.56854	0.57209	0.58377	0.59656	0.61090
138	0.53773	0.55999	0.57055	0.57410	0.58570	0.59845	0.61273
139	0.53978	0.56197	0.57252	0.57609	0.58764	0.60032	0.61455
140	0.54189	0.56399	0.57449	0.57803	0.58956	0.60217	0.61635
141	0.54407	0.56599	0.57644	0.57994	0.59147	0.60401	0.61813
142	0.54608	0.56796	0.57833	0.58185	0.59332	0.60583	0.61988
143	0.54816	0.56992	0.58025	0.58378	0.59519	0.60763	0.62162
144	0.55015	0.57183	0.58216	0.58564	0.59702	0.60942	0.62333
145	0.55222	0.57373	0.58405	0.58749	0.59884	0.61117	0.62503
146	0.55419	0.57565	0.58591	0.58934	0.60060	0.61293	0.62671
147	0.55609	0.57752	0.58774	0.59117	0.60239	0.61467	0.62839
148	0.55807	0.57938	0.58952	0.59295	0.60415	0.61637	0.63004
149	0.55993	0.58121	0.59135	0.59473	0.60589	0.61806	0.63167
150	0.56182	0.58301	0.59313	0.59650	0.60762	0.61974	0.63328
151	0.56362	0.58484	0.59489	0.59825	0.60932	0.62139	0.63488
152	0.56545	0.58663	0.59661	0.59998	0.61101	0.62301	0.63648
153	0.56726	0.58839	0.59835	0.60169	0.61270	0.62466	0.63804
154	0.56915	0.59015	0.60007	0.60341	0.61435	0.62627	0.63961
155	0.57097	0.59188	0.60178	0.60513	0.61601	0.62787	0.64115
156	0.57276	0.59358	0.60346	0.60681	0.61761	0.62945	0.64268
157	0.57455	0.59527	0.60510	0.60847	0.61921	0.63101	0.64420
158	0.57624	0.59694	0.60677	0.61009	0.62081	0.63256	0.64570
159	0.57804	0.59862	0.60841	0.61172	0.62240	0.63410	0.64718
160	0.57974	0.60029	0.61003	0.61331	0.62400	0.63562	0.64866
161	0.58149	0.60195	0.61164	0.61487	0.62556	0.63713	0.65011
162	0.58311	0.60354	0.61323	0.61642	0.62708	0.63861	0.65155
163	0.58486	0.60514	0.61478	0.61800	0.62858	0.64008	0.65297
164	0.58654	0.60677	0.61636	0.61958	0.63012	0.64155	0.65438
165	0.58820	0.60836	0.61791	0.62111	0.63161	0.64300	0.65579
166	0.58982	0.60993	0.61944	0.62264	0.63309	0.64446	0.65719
167	0.59146	0.61146	0.62094	0.62416	0.63457	0.64588	0.65856
168	0.59312	0.61300	0.62245	0.62564	0.63602	0.64729	0.65993
169	0.59465	0.61455	0.62394	0.62713	0.63745	0.64870	0.66129
170	0.59625	0.61606	0.62544	0.62861	0.63889	0.65008	0.66262
171	0.59789	0.61756	0.62690	0.63005	0.64031	0.65146	0.66394
172	0.59946	0.61908	0.62837	0.63147	0.64171	0.65282	0.66527
173	0.60093	0.62057	0.62981	0.63293	0.64312	0.65417	0.66658
174	0.60247	0.62202	0.63124	0.63436	0.64450	0.65553	0.66787
175	0.60403	0.62348	0.63267	0.63577	0.64587	0.65686	0.66915
176	0.60543	0.62488	0.63405	0.63716	0.64722	0.65817	0.67042
177	0.60690	0.62636	0.63548	0.63853	0.64858	0.65948	0.67168
178	0.60832	0.62774	0.63684	0.63988	0.64990	0.66078	0.67293
179	0.60985	0.62913	0.63821	0.64123	0.65122	0.66208	0.67417
180	0.61130	0.63054	0.63956	0.64262	0.65255	0.66336	0.67540
181	0.61273	0.63193	0.64089	0.64394	0.65383	0.66461	0.67661
182	0.61423	0.63330	0.64224	0.64526	0.65512	0.66585	0.67782
183	0.61564	0.63467	0.64359	0.64659	0.65640	0.66709	0.67902
184	0.61701	0.63601	0.64488	0.64789	0.65768	0.66832	0.68021
185	0.61845	0.63730	0.64616	0.64918	0.65893	0.66955	0.68138
186	0.61990	0.63866	0.64746	0.65045	0.66018	0.67077	0.68255
187	0.62123	0.63995	0.64875	0.65173	0.66141	0.67197	0.68370
188	0.62259	0.64129	0.65005	0.65298	0.66264	0.67315	0.68485
189	0.62395	0.64257	0.65131	0.65425	0.66386	0.67433	0.68599
190	0.62525	0.64387	0.65254	0.65548	0.66507	0.67550	0.68712
191	0.62658	0.64510	0.65379	0.65672	0.66628	0.67666	0.68824

N	0.1%	0.5%	1%	1.25%	2.5%	5%	10%
192	0.62790	0.64635	0.65502	0.65795	0.66747	0.67782	0.68936
193	0.62920	0.64761	0.65624	0.65915	0.66865	0.67897	0.69046
194	0.63053	0.64885	0.65745	0.66033	0.66982	0.68010	0.69156
195	0.63185	0.65008	0.65864	0.66152	0.67099	0.68123	0.69264
196	0.63310	0.65131	0.65984	0.66270	0.67213	0.68234	0.69372
197	0.63434	0.65252	0.66104	0.66389	0.67327	0.68346	0.69479
198	0.63565	0.65370	0.66220	0.66505	0.67439	0.68456	0.69584
199	0.63692	0.65488	0.66336	0.66622	0.67553	0.68565	0.69690
200	0.63816	0.65604	0.66452	0.66736	0.67664	0.68673	0.69795
201	0.63937	0.65723	0.66567	0.66850	0.67775	0.68780	0.69898
202	0.64063	0.65841	0.66682	0.66963	0.67886	0.68887	0.70001
203	0.64179	0.65959	0.66793	0.67075	0.67994	0.68993	0.70103
204	0.64298	0.66075	0.66907	0.67187	0.68102	0.69098	0.70204
205	0.64420	0.66188	0.67019	0.67298	0.68209	0.69203	0.70305
206	0.64536	0.66304	0.67130	0.67406	0.68316	0.69306	0.70405
207	0.64653	0.66417	0.67241	0.67515	0.68422	0.69408	0.70504
208	0.64768	0.66530	0.67348	0.67623	0.68528	0.69510	0.70602
209	0.64888	0.66642	0.67459	0.67730	0.68633	0.69612	0.70699
210	0.65002	0.66750	0.67566	0.67837	0.68736	0.69712	0.70797
211	0.65112	0.66860	0.67671	0.67942	0.68838	0.69811	0.70893
212	0.65228	0.66967	0.67779	0.68050	0.68941	0.69910	0.70989
213	0.65338	0.67075	0.67882	0.68153	0.69041	0.70008	0.71083
214	0.65454	0.67183	0.67989	0.68259	0.69143	0.70106	0.71176
215	0.65566	0.67289	0.68091	0.68359	0.69243	0.70204	0.71270
216	0.65675	0.67395	0.68194	0.68462	0.69342	0.70300	0.71363
217	0.65786	0.67497	0.68296	0.68564	0.69441	0.70396	0.71455
218	0.65899	0.67602	0.68398	0.68665	0.69539	0.70490	0.71546
219	0.66003	0.67702	0.68498	0.68766	0.69636	0.70582	0.71636
220	0.66113	0.67804	0.68600	0.68866	0.69732	0.70676	0.71727
221	0.66220	0.67906	0.68699	0.68964	0.69827	0.70769	0.71816
222	0.66326	0.68008	0.68798	0.69060	0.69923	0.70861	0.71905
223	0.66433	0.68108	0.68894	0.69156	0.70018	0.70952	0.71993
224	0.66540	0.68208	0.68990	0.69252	0.70112	0.71043	0.72081
225	0.66641	0.68307	0.69087	0.69346	0.70205	0.71133	0.72167
226	0.66741	0.68405	0.69182	0.69442	0.70298	0.71223	0.72253
227	0.66839	0.68502	0.69276	0.69538	0.70388	0.71312	0.72339
228	0.66943	0.68601	0.69371	0.69632	0.70477	0.71400	0.72424
229	0.67044	0.68695	0.69464	0.69725	0.70568	0.71488	0.72508
230	0.67147	0.68792	0.69557	0.69819	0.70658	0.71574	0.72593
231	0.67246	0.68886	0.69651	0.69910	0.70747	0.71662	0.72676
232	0.67342	0.68981	0.69744	0.70000	0.70835	0.71748	0.72759
233	0.67441	0.69072	0.69837	0.70092	0.70923	0.71833	0.72842
234	0.67538	0.69167	0.69926	0.70181	0.71010	0.71918	0.72923
235	0.67633	0.69260	0.70015	0.70271	0.71097	0.72001	0.73004
236	0.67730	0.69351	0.70106	0.70359	0.71183	0.72085	0.73085
237	0.67825	0.69440	0.70193	0.70448	0.71269	0.72168	0.73165
238	0.67927	0.69529	0.70281	0.70536	0.71353	0.72250	0.73244
239	0.68024	0.69621	0.70369	0.70622	0.71439	0.72333	0.73323
240	0.68119	0.69708	0.70455	0.70707	0.71524	0.72414	0.73401
241	0.68207	0.69799	0.70543	0.70793	0.71607	0.72495	0.73479
242	0.68297	0.69887	0.70628	0.70877	0.71690	0.72575	0.73556
243	0.68392	0.69973	0.70713	0.70963	0.71770	0.72654	0.73632
244	0.68487	0.70061	0.70798	0.71048	0.71853	0.72734	0.73709
245	0.68578	0.70148	0.70882	0.71132	0.71934	0.72812	0.73785
246	0.68671	0.70233	0.70967	0.71216	0.72016	0.72891	0.73861
247	0.68757	0.70318	0.71050	0.71297	0.72097	0.72968	0.73936
248	0.68845	0.70404	0.71133	0.71378	0.72177	0.73046	0.74010
249	0.68930	0.70490	0.71215	0.71460	0.72255	0.73122	0.74084
250	0.69022	0.70573	0.71296	0.71541	0.72334	0.73198	0.74158
251	0.69110	0.70657	0.71377	0.71623	0.72413	0.73274	0.74231
252	0.69199	0.70739	0.71459	0.71702	0.72490	0.73350	0.74303
253	0.69287	0.70821	0.71539	0.71781	0.72568	0.73424	0.74376
254	0.69372	0.70902	0.71617	0.71859	0.72644	0.73498	0.74448
255	0.69459	0.70983	0.71696	0.71939	0.72721	0.73573	0.74518
256	0.69547	0.71064	0.71776	0.72016	0.72797	0.73647	0.74588
257	0.69629	0.71145	0.71855	0.72093	0.72873	0.73719	0.74658
258	0.69714	0.71224	0.71931	0.72170	0.72949	0.73791	0.74728
259	0.69799	0.71303	0.72009	0.72246	0.73023	0.73864	0.74798
260	0.69877	0.71380	0.72085	0.72322	0.73096	0.73935	0.74867
261	0.69960	0.71459	0.72162	0.72397	0.73170	0.74006	0.74936
262	0.70041	0.71536	0.72238	0.72473	0.73242	0.74077	0.75004
263	0.70132	0.71613	0.72314	0.72549	0.73314	0.74148	0.75072
264	0.70209	0.71691	0.72389	0.72623	0.73387	0.74217	0.75139
265	0.70297	0.71768	0.72463	0.72697	0.73458	0.74287	0.75206

N	0.1%	0.5%	1%	1.25%	2.5%	5%	10%
266	0.70373	0.71846	0.72536	0.72770	0.73530	0.74356	0.75273
267	0.70449	0.71921	0.72610	0.72843	0.73601	0.74425	0.75339
268	0.70527	0.71996	0.72684	0.72917	0.73672	0.74493	0.75405
269	0.70608	0.72069	0.72757	0.72990	0.73742	0.74561	0.75470
270	0.70686	0.72147	0.72831	0.73061	0.73812	0.74627	0.75534
271	0.70763	0.72219	0.72903	0.73131	0.73882	0.74695	0.75599
272	0.70837	0.72294	0.72975	0.73203	0.73951	0.74762	0.75664
273	0.70912	0.72365	0.73045	0.73274	0.74019	0.74828	0.75727
274	0.70990	0.72438	0.73117	0.73344	0.74087	0.74894	0.75790
275	0.71064	0.72509	0.73187	0.73413	0.74155	0.74960	0.75853
276	0.71138	0.72580	0.73256	0.73483	0.74222	0.75025	0.75916
277	0.71214	0.72653	0.73326	0.73553	0.74290	0.75090	0.75979
278	0.71291	0.72723	0.73395	0.73623	0.74356	0.75154	0.76041
279	0.71371	0.72795	0.73465	0.73691	0.74422	0.75218	0.76102
280	0.71444	0.72866	0.73534	0.73759	0.74487	0.75281	0.76164
281	0.71518	0.72936	0.73602	0.73827	0.74552	0.75345	0.76225
282	0.71592	0.73004	0.73669	0.73893	0.74617	0.75407	0.76285
283	0.71661	0.73074	0.73736	0.73960	0.74682	0.75470	0.76345
284	0.71732	0.73142	0.73804	0.74026	0.74746	0.75532	0.76405
285	0.71806	0.73209	0.73871	0.74091	0.74810	0.75594	0.76465
286	0.71875	0.73279	0.73937	0.74157	0.74873	0.75656	0.76524
287	0.71945	0.73346	0.74003	0.74224	0.74937	0.75717	0.76584
288	0.72015	0.73412	0.74069	0.74287	0.75000	0.75778	0.76642
289	0.72086	0.73479	0.74135	0.74352	0.75062	0.75838	0.76700
290	0.72149	0.73547	0.74199	0.74417	0.75125	0.75899	0.76759
291	0.72220	0.73613	0.74264	0.74481	0.75187	0.75958	0.76816
292	0.72282	0.73677	0.74328	0.74545	0.75249	0.76018	0.76873
293	0.72359	0.73743	0.74392	0.74609	0.75310	0.76077	0.76930
294	0.72427	0.73807	0.74453	0.74672	0.75371	0.76136	0.76987
295	0.72496	0.73874	0.74518	0.74733	0.75431	0.76194	0.77043
296	0.72563	0.73937	0.74581	0.74795	0.75491	0.76253	0.77099
297	0.72628	0.74002	0.74643	0.74857	0.75551	0.76311	0.77155
298	0.72696	0.74065	0.74704	0.74918	0.75612	0.76368	0.77210
299	0.72764	0.74129	0.74767	0.74981	0.75670	0.76425	0.77265
300	0.72830	0.74192	0.74828	0.75042	0.75729	0.76482	0.77320
301	0.72896	0.74253	0.74889	0.75102	0.75787	0.76539	0.77374
302	0.72963	0.74315	0.74949	0.75160	0.75846	0.76596	0.77428
303	0.73028	0.74377	0.75008	0.75220	0.75903	0.76652	0.77482
304	0.73093	0.74439	0.75068	0.75279	0.75961	0.76707	0.77535
305	0.73156	0.74498	0.75126	0.75338	0.76018	0.76763	0.77589
306	0.73218	0.74557	0.75187	0.75397	0.76076	0.76818	0.77642
307	0.73286	0.74620	0.75246	0.75455	0.76133	0.76872	0.77695
308	0.73346	0.74679	0.75305	0.75514	0.76190	0.76927	0.77748
309	0.73410	0.74738	0.75363	0.75572	0.76246	0.76981	0.77800
310	0.73469	0.74796	0.75421	0.75630	0.76300	0.77035	0.77852
311	0.73534	0.74857	0.75478	0.75688	0.76356	0.77088	0.77903
312	0.73597	0.74917	0.75535	0.75744	0.76411	0.77142	0.77954
313	0.73659	0.74975	0.75590	0.75799	0.76468	0.77195	0.78006
314	0.73720	0.75035	0.75648	0.75855	0.76522	0.77248	0.78057
315	0.73783	0.75092	0.75705	0.75910	0.76577	0.77301	0.78108
316	0.73843	0.75148	0.75761	0.75966	0.76632	0.77354	0.78158
317	0.73903	0.75206	0.75818	0.76021	0.76685	0.77406	0.78208
318	0.73960	0.75262	0.75872	0.76077	0.76739	0.77458	0.78258
319	0.74021	0.75319	0.75926	0.76133	0.76792	0.77510	0.78308
320	0.74080	0.75376	0.75981	0.76187	0.76844	0.77561	0.78357
321	0.74138	0.75434	0.76036	0.76241	0.76898	0.77612	0.78406
322	0.74198	0.75489	0.76091	0.76294	0.76950	0.77662	0.78455
323	0.74255	0.75543	0.76147	0.76348	0.77003	0.77713	0.78503
324	0.74315	0.75599	0.76200	0.76401	0.77055	0.77764	0.78552
325	0.74372	0.75654	0.76254	0.76455	0.77106	0.77813	0.78600
326	0.74429	0.75708	0.76308	0.76508	0.77157	0.77862	0.78648
327	0.74489	0.75765	0.76361	0.76561	0.77209	0.77912	0.78695
328	0.74546	0.75818	0.76414	0.76612	0.77260	0.77961	0.78742
329	0.74605	0.75871	0.76466	0.76664	0.77311	0.78010	0.78789
330	0.74660	0.75924	0.76519	0.76716	0.77361	0.78060	0.78836
331	0.74715	0.75977	0.76570	0.76768	0.77411	0.78108	0.78883
332	0.74771	0.76032	0.76622	0.76819	0.77461	0.78156	0.78929
333	0.74827	0.76085	0.76674	0.76870	0.77511	0.78204	0.78976
334	0.74883	0.76137	0.76725	0.76920	0.77560	0.78252	0.79022
335	0.74937	0.76190	0.76777	0.76971	0.77609	0.78299	0.79067
336	0.74986	0.76244	0.76827	0.77022	0.77659	0.78347	0.79113
337	0.75039	0.76297	0.76878	0.77073	0.77707	0.78394	0.79159
338	0.75095	0.76347	0.76928	0.77122	0.77756	0.78441	0.79204
339	0.75152	0.76398	0.76979	0.77173	0.77805	0.78488	0.79248

N	0.1%	0.5%	1%	1.25%	2.5%	5%	10%
340	0.75202	0.76449	0.77028	0.77222	0.77852	0.78535	0.79292
341	0.75259	0.76501	0.77078	0.77272	0.77900	0.78581	0.79337
342	0.75316	0.76552	0.77127	0.77320	0.77948	0.78627	0.79382
343	0.75368	0.76602	0.77176	0.77369	0.77996	0.78673	0.79426
344	0.75421	0.76653	0.77225	0.77418	0.78043	0.78718	0.79469
345	0.75471	0.76705	0.77274	0.77467	0.78089	0.78764	0.79513
346	0.75525	0.76754	0.77323	0.77515	0.78136	0.78809	0.79557
347	0.75575	0.76804	0.77371	0.77564	0.78183	0.78855	0.79600
348	0.75631	0.76853	0.77420	0.77611	0.78228	0.78899	0.79643
349	0.75684	0.76905	0.77468	0.77659	0.78274	0.78944	0.79685
350	0.75733	0.76953	0.77515	0.77706	0.78319	0.78988	0.79728
351	0.75784	0.77002	0.77564	0.77752	0.78365	0.79032	0.79770
352	0.75835	0.77049	0.77611	0.77800	0.78411	0.79076	0.79813
353	0.75884	0.77097	0.77658	0.77846	0.78456	0.79120	0.79855
354	0.75936	0.77145	0.77705	0.77892	0.78501	0.79163	0.79897
355	0.75986	0.77190	0.77752	0.77939	0.78546	0.79206	0.79939
356	0.76037	0.77238	0.77799	0.77986	0.78590	0.79249	0.79980
357	0.76084	0.77285	0.77844	0.78031	0.78635	0.79292	0.80021
358	0.76130	0.77333	0.77892	0.78077	0.78678	0.79335	0.80062
359	0.76182	0.77379	0.77937	0.78121	0.78723	0.79377	0.80103
360	0.76232	0.77425	0.77982	0.78167	0.78766	0.79419	0.80144
361	0.76280	0.77473	0.78029	0.78212	0.78810	0.79462	0.80184
362	0.76328	0.77519	0.78074	0.78256	0.78854	0.79503	0.80224
363	0.76378	0.77567	0.78118	0.78302	0.78896	0.79545	0.80265
364	0.76424	0.77613	0.78162	0.78345	0.78940	0.79587	0.80305
365	0.76471	0.77658	0.78206	0.78389	0.78983	0.79628	0.80344
366	0.76518	0.77704	0.78251	0.78434	0.79025	0.79669	0.80384
367	0.76565	0.77748	0.78296	0.78477	0.79067	0.79711	0.80424
368	0.76616	0.77793	0.78339	0.78519	0.79110	0.79751	0.80463
369	0.76660	0.77836	0.78382	0.78563	0.79151	0.79792	0.80502
370	0.76706	0.77881	0.78425	0.78607	0.79193	0.79833	0.80541
371	0.76752	0.77925	0.78469	0.78651	0.79235	0.79873	0.80580
372	0.76800	0.77969	0.78513	0.78693	0.79276	0.79913	0.80619
373	0.76848	0.78014	0.78555	0.78736	0.79318	0.79953	0.80656
374	0.76896	0.78059	0.78598	0.78778	0.79359	0.79993	0.80695
375	0.76945	0.78100	0.78639	0.78819	0.79400	0.80032	0.80733
376	0.76987	0.78144	0.78682	0.78863	0.79440	0.80071	0.80771
377	0.77035	0.78186	0.78724	0.78904	0.79481	0.80111	0.80809
378	0.77082	0.78229	0.78765	0.78946	0.79522	0.80149	0.80846
379	0.77126	0.78273	0.78807	0.78987	0.79561	0.80188	0.80884
380	0.77175	0.78315	0.78850	0.79029	0.79601	0.80227	0.80921
381	0.77220	0.78359	0.78892	0.79071	0.79642	0.80266	0.80958
382	0.77262	0.78402	0.78933	0.79111	0.79681	0.80304	0.80995
383	0.77308	0.78444	0.78974	0.79152	0.79721	0.80343	0.81032
384	0.77352	0.78486	0.79014	0.79192	0.79761	0.80380	0.81068
385	0.77398	0.78528	0.79055	0.79233	0.79800	0.80419	0.81105
386	0.77446	0.78569	0.79095	0.79273	0.79839	0.80456	0.81141
387	0.77490	0.78610	0.79136	0.79313	0.79878	0.80493	0.81177
388	0.77533	0.78651	0.79176	0.79352	0.79916	0.80531	0.81213
389	0.77574	0.78692	0.79215	0.79391	0.79955	0.80569	0.81249
390	0.77617	0.78735	0.79256	0.79430	0.79993	0.80606	0.81285
391	0.77662	0.78775	0.79296	0.79470	0.80031	0.80643	0.81320
392	0.77705	0.78817	0.79334	0.79509	0.80070	0.80679	0.81355
393	0.77749	0.78856	0.79373	0.79548	0.80108	0.80715	0.81390
394	0.77790	0.78898	0.79413	0.79586	0.80146	0.80752	0.81426
395	0.77832	0.78937	0.79452	0.79625	0.80183	0.80788	0.81460
396	0.77876	0.78976	0.79491	0.79664	0.80220	0.80825	0.81495
397	0.77917	0.79015	0.79529	0.79702	0.80257	0.80861	0.81530
398	0.77954	0.79054	0.79568	0.79740	0.80294	0.80897	0.81565
399	0.77996	0.79095	0.79607	0.79779	0.80331	0.80932	0.81599
400	0.78037	0.79135	0.79645	0.79816	0.80368	0.80968	0.81633
401	0.78075	0.79172	0.79682	0.79853	0.80405	0.81004	0.81667
402	0.78116	0.79213	0.79720	0.79891	0.80441	0.81039	0.81701
403	0.78156	0.79251	0.79758	0.79929	0.80478	0.81074	0.81735
404	0.78196	0.79291	0.79797	0.79966	0.80514	0.81109	0.81769
405	0.78237	0.79329	0.79834	0.80003	0.80550	0.81143	0.81802
406	0.78278	0.79366	0.79872	0.80040	0.80587	0.81178	0.81836
407	0.78316	0.79405	0.79909	0.80077	0.80623	0.81212	0.81869
408	0.78357	0.79443	0.79946	0.80114	0.80658	0.81247	0.81902
409	0.78399	0.79481	0.79983	0.80150	0.80694	0.81281	0.81935
410	0.78436	0.79519	0.80019	0.80187	0.80729	0.81315	0.81968
411	0.78475	0.79556	0.80056	0.80222	0.80764	0.81349	0.82001
412	0.78513	0.79594	0.80093	0.80259	0.80798	0.81383	0.82034
413	0.78552	0.79631	0.80129	0.80294	0.80833	0.81417	0.82066

N	0.1%	0.5%	1%	1.25%	2.5%	5%	10%
414	0.78591	0.79668	0.80165	0.80330	0.80867	0.81450	0.82099
415	0.78634	0.79704	0.80201	0.80365	0.80902	0.81483	0.82130
416	0.78674	0.79740	0.80236	0.80401	0.80937	0.81517	0.82162
417	0.78712	0.79778	0.80273	0.80437	0.80972	0.81551	0.82194
418	0.78753	0.79814	0.80308	0.80472	0.81005	0.81584	0.82226
419	0.78789	0.79851	0.80344	0.80507	0.81039	0.81617	0.82258
420	0.78830	0.79887	0.80379	0.80542	0.81073	0.81649	0.82290
421	0.78866	0.79923	0.80412	0.80576	0.81107	0.81682	0.82321
422	0.78905	0.79959	0.80448	0.80610	0.81141	0.81714	0.82353
423	0.78940	0.79994	0.80483	0.80645	0.81174	0.81747	0.82384
424	0.78979	0.80030	0.80519	0.80680	0.81208	0.81779	0.82415
425	0.79016	0.80065	0.80552	0.80714	0.81241	0.81811	0.82446
426	0.79053	0.80099	0.80586	0.80749	0.81275	0.81843	0.82477
427	0.79092	0.80134	0.80620	0.80782	0.81308	0.81875	0.82508
428	0.79131	0.80168	0.80654	0.80817	0.81340	0.81907	0.82538
429	0.79165	0.80203	0.80688	0.80850	0.81373	0.81939	0.82569
430	0.79204	0.80240	0.80722	0.80884	0.81406	0.81970	0.82599
431	0.79240	0.80274	0.80756	0.80917	0.81439	0.82001	0.82629
432	0.79277	0.80310	0.80790	0.80949	0.81471	0.82033	0.82659
433	0.79313	0.80345	0.80823	0.80983	0.81503	0.82064	0.82689
434	0.79350	0.80379	0.80857	0.81017	0.81535	0.82095	0.82719
435	0.79387	0.80412	0.80890	0.81050	0.81568	0.82126	0.82748
436	0.79424	0.80445	0.80923	0.81082	0.81599	0.82157	0.82778
437	0.79459	0.80479	0.80957	0.81116	0.81631	0.82187	0.82807
438	0.79494	0.80511	0.80990	0.81148	0.81662	0.82218	0.82837
439	0.79528	0.80545	0.81023	0.81180	0.81694	0.82248	0.82867
440	0.79564	0.80579	0.81055	0.81213	0.81725	0.82278	0.82896
441	0.79600	0.80613	0.81088	0.81245	0.81757	0.82309	0.82925
442	0.79634	0.80645	0.81120	0.81277	0.81787	0.82339	0.82954
443	0.79670	0.80678	0.81152	0.81308	0.81819	0.82369	0.82983
444	0.79705	0.80711	0.81184	0.81341	0.81850	0.82399	0.83011
445	0.79740	0.80744	0.81216	0.81373	0.81880	0.82429	0.83040
446	0.79775	0.80779	0.81248	0.81405	0.81911	0.82458	0.83068
447	0.79809	0.80811	0.81280	0.81436	0.81942	0.82488	0.83097
448	0.79845	0.80843	0.81312	0.81468	0.81972	0.82518	0.83125
449	0.79880	0.80876	0.81344	0.81499	0.82003	0.82547	0.83153
450	0.79917	0.80909	0.81374	0.81531	0.82033	0.82576	0.83181
451	0.79950	0.80940	0.81406	0.81562	0.82063	0.82605	0.83209
452	0.79983	0.80973	0.81437	0.81593	0.82093	0.82634	0.83237
453	0.80014	0.81005	0.81468	0.81623	0.82123	0.82663	0.83265
454	0.80048	0.81036	0.81498	0.81654	0.82153	0.82692	0.83292
455	0.80081	0.81069	0.81529	0.81684	0.82183	0.82721	0.83320
456	0.80114	0.81100	0.81560	0.81714	0.82212	0.82749	0.83347
457	0.80147	0.81132	0.81591	0.81744	0.82241	0.82778	0.83375
458	0.80180	0.81162	0.81621	0.81774	0.82270	0.82807	0.83402
459	0.80212	0.81193	0.81652	0.81804	0.82300	0.82834	0.83429
460	0.80248	0.81224	0.81682	0.81834	0.82329	0.82862	0.83456
461	0.80282	0.81256	0.81713	0.81864	0.82358	0.82890	0.83483
462	0.80314	0.81287	0.81743	0.81895	0.82387	0.82918	0.83510
463	0.80344	0.81316	0.81773	0.81924	0.82416	0.82946	0.83537
464	0.80378	0.81348	0.81803	0.81954	0.82445	0.82974	0.83564
465	0.80411	0.81378	0.81833	0.81984	0.82474	0.83001	0.83591
466	0.80443	0.81408	0.81863	0.82013	0.82502	0.83029	0.83617
467	0.80473	0.81439	0.81892	0.82041	0.82530	0.83057	0.83644
468	0.80504	0.81470	0.81921	0.82071	0.82558	0.83084	0.83670
469	0.80539	0.81499	0.81950	0.82100	0.82586	0.83111	0.83696
470	0.80569	0.81529	0.81979	0.82128	0.82614	0.83138	0.83722
471	0.80603	0.81560	0.82009	0.82158	0.82642	0.83166	0.83749
472	0.80634	0.81589	0.82037	0.82186	0.82670	0.83193	0.83774
473	0.80665	0.81619	0.82066	0.82215	0.82698	0.83220	0.83800
474	0.80695	0.81649	0.82096	0.82243	0.82726	0.83246	0.83826
475	0.80726	0.81678	0.82124	0.82271	0.82753	0.83273	0.83852
476	0.80758	0.81708	0.82153	0.82299	0.82781	0.83299	0.83877
477	0.80787	0.81737	0.82181	0.82328	0.82808	0.83326	0.83903
478	0.80819	0.81767	0.82210	0.82356	0.82836	0.83353	0.83929
479	0.80852	0.81796	0.82239	0.82384	0.82862	0.83379	0.83954
480	0.80883	0.81824	0.82268	0.82413	0.82890	0.83406	0.83979
481	0.80914	0.81854	0.82295	0.82440	0.82917	0.83432	0.84004
482	0.80944	0.81884	0.82322	0.82468	0.82944	0.83458	0.84029
483	0.80973	0.81912	0.82350	0.82496	0.82970	0.83484	0.84054
484	0.81004	0.81940	0.82378	0.82523	0.82997	0.83510	0.84079
485	0.81033	0.81968	0.82405	0.82551	0.83024	0.83536	0.84104
486	0.81064	0.81996	0.82433	0.82578	0.83050	0.83562	0.84128
487	0.81093	0.82025	0.82461	0.82605	0.83077	0.83587	0.84153

N	0.1%	0.5%	1%	1.25%	2.5%	5%	10%
488	0.81126	0.82053	0.82489	0.82633	0.83103	0.83613	0.84178
489	0.81156	0.82080	0.82515	0.82660	0.83129	0.83638	0.84202
490	0.81186	0.82108	0.82542	0.82686	0.83155	0.83663	0.84227
491	0.81215	0.82135	0.82570	0.82714	0.83181	0.83689	0.84251
492	0.81244	0.82164	0.82597	0.82740	0.83208	0.83714	0.84275
493	0.81272	0.82191	0.82623	0.82767	0.83233	0.83739	0.84299
494	0.81299	0.82219	0.82650	0.82794	0.83259	0.83764	0.84323
495	0.81328	0.82248	0.82677	0.82820	0.83285	0.83789	0.84347
496	0.81358	0.82275	0.82704	0.82847	0.83311	0.83814	0.84371
497	0.81388	0.82302	0.82729	0.82874	0.83336	0.83839	0.84395
498	0.81417	0.82330	0.82756	0.82900	0.83361	0.83863	0.84419
499	0.81445	0.82356	0.82782	0.82926	0.83387	0.83888	0.84443
500	0.81473	0.82384	0.82809	0.82952	0.83412	0.83913	0.84466
501	0.81500	0.82411	0.82835	0.82978	0.83437	0.83937	0.84489
502	0.81529	0.82438	0.82862	0.83004	0.83462	0.83961	0.84513
503	0.81557	0.82463	0.82888	0.83030	0.83488	0.83986	0.84536
504	0.81585	0.82490	0.82914	0.83055	0.83513	0.84010	0.84560
505	0.81614	0.82518	0.82941	0.83081	0.83538	0.84034	0.84583
506	0.81641	0.82544	0.82966	0.83107	0.83563	0.84058	0.84606
507	0.81670	0.82570	0.82992	0.83132	0.83588	0.84082	0.84629
508	0.81698	0.82596	0.83018	0.83158	0.83613	0.84106	0.84652
509	0.81728	0.82622	0.83043	0.83183	0.83638	0.84129	0.84675
510	0.81755	0.82649	0.83069	0.83209	0.83663	0.84153	0.84697
511	0.81782	0.82674	0.83095	0.83234	0.83687	0.84177	0.84720
512	0.81810	0.82701	0.83121	0.83259	0.83711	0.84200	0.84743
513	0.81837	0.82727	0.83145	0.83284	0.83735	0.84224	0.84765
514	0.81865	0.82752	0.83171	0.83309	0.83759	0.84247	0.84788
515	0.81892	0.82778	0.83195	0.83334	0.83783	0.84270	0.84810
516	0.81917	0.82803	0.83220	0.83359	0.83807	0.84294	0.84833
517	0.81945	0.82830	0.83245	0.83384	0.83831	0.84316	0.84855
518	0.81969	0.82856	0.83271	0.83408	0.83855	0.84340	0.84877
519	0.81996	0.82880	0.83296	0.83433	0.83878	0.84362	0.84899
520	0.82024	0.82905	0.83319	0.83457	0.83902	0.84385	0.84921
521	0.82050	0.82931	0.83344	0.83481	0.83925	0.84408	0.84943
522	0.82079	0.82956	0.83368	0.83505	0.83949	0.84431	0.84965
523	0.82105	0.82982	0.83393	0.83530	0.83973	0.84454	0.84987
524	0.82131	0.83008	0.83417	0.83554	0.83996	0.84477	0.85009
525	0.82156	0.83033	0.83441	0.83577	0.84020	0.84499	0.85031
526	0.82182	0.83058	0.83466	0.83602	0.84043	0.84522	0.85052
527	0.82209	0.83083	0.83489	0.83625	0.84066	0.84544	0.85074
528	0.82237	0.83107	0.83513	0.83649	0.84089	0.84566	0.85095
529	0.82261	0.83131	0.83537	0.83673	0.84112	0.84588	0.85117
530	0.82290	0.83157	0.83561	0.83696	0.84135	0.84611	0.85138
531	0.82317	0.83181	0.83585	0.83720	0.84157	0.84633	0.85159
532	0.82340	0.83205	0.83609	0.83743	0.84181	0.84655	0.85181
533	0.82367	0.83230	0.83632	0.83766	0.84204	0.84677	0.85202
534	0.82394	0.83254	0.83655	0.83790	0.84226	0.84699	0.85223
535	0.82419	0.83278	0.83679	0.83813	0.84249	0.84720	0.85244
536	0.82443	0.83302	0.83702	0.83836	0.84272	0.84743	0.85265
537	0.82468	0.83326	0.83726	0.83859	0.84294	0.84764	0.85286
538	0.82492	0.83350	0.83749	0.83882	0.84317	0.84786	0.85307
539	0.82518	0.83374	0.83773	0.83906	0.84339	0.84808	0.85328
540	0.82545	0.83397	0.83797	0.83929	0.84361	0.84829	0.85348
541	0.82570	0.83421	0.83819	0.83952	0.84384	0.84851	0.85369
542	0.82593	0.83445	0.83842	0.83974	0.84406	0.84872	0.85390
543	0.82617	0.83468	0.83864	0.83997	0.84428	0.84894	0.85410
544	0.82642	0.83490	0.83887	0.84020	0.84450	0.84915	0.85431
545	0.82664	0.83514	0.83909	0.84042	0.84471	0.84936	0.85451
546	0.82689	0.83537	0.83932	0.84065	0.84493	0.84957	0.85472
547	0.82715	0.83561	0.83954	0.84087	0.84515	0.84978	0.85492
548	0.82739	0.83584	0.83976	0.84109	0.84537	0.84999	0.85512
549	0.82765	0.83607	0.83998	0.84131	0.84559	0.85020	0.85532
550	0.82788	0.83631	0.84021	0.84154	0.84580	0.85041	0.85552
551	0.82811	0.83654	0.84044	0.84176	0.84602	0.85061	0.85572
552	0.82836	0.83677	0.84065	0.84198	0.84623	0.85082	0.85592
553	0.82863	0.83699	0.84088	0.84220	0.84645	0.85103	0.85612
554	0.82886	0.83722	0.84111	0.84242	0.84666	0.85124	0.85632
555	0.82911	0.83745	0.84133	0.84265	0.84687	0.85144	0.85652
556	0.82934	0.83768	0.84155	0.84286	0.84708	0.85165	0.85671
557	0.82960	0.83790	0.84177	0.84308	0.84729	0.85185	0.85691
558	0.82983	0.83813	0.84199	0.84329	0.84750	0.85206	0.85710
559	0.83007	0.83835	0.84221	0.84351	0.84771	0.85226	0.85730
560	0.83032	0.83858	0.84243	0.84372	0.84791	0.85247	0.85749
561	0.83053	0.83880	0.84264	0.84394	0.84812	0.85267	0.85769

N	0.1%	0.5%	1%	1.25%	2.5%	5%	10%
562	0.83078	0.83902	0.84286	0.84416	0.84833	0.85287	0.85788
563	0.83100	0.83924	0.84309	0.84437	0.84854	0.85307	0.85807
564	0.83123	0.83946	0.84330	0.84459	0.84875	0.85327	0.85827
565	0.83146	0.83968	0.84352	0.84480	0.84896	0.85347	0.85846
566	0.83172	0.83991	0.84373	0.84502	0.84917	0.85367	0.85865
567	0.83196	0.84012	0.84395	0.84523	0.84937	0.85387	0.85884
568	0.83219	0.84034	0.84415	0.84544	0.84957	0.85406	0.85903
569	0.83241	0.84055	0.84437	0.84565	0.84978	0.85426	0.85922
570	0.83266	0.84077	0.84458	0.84586	0.84998	0.85446	0.85941
571	0.83286	0.84099	0.84479	0.84607	0.85018	0.85465	0.85960
572	0.83310	0.84121	0.84500	0.84628	0.85039	0.85485	0.85978
573	0.83334	0.84143	0.84521	0.84649	0.85059	0.85504	0.85997
574	0.83355	0.84164	0.84542	0.84669	0.85079	0.85524	0.86015
575	0.83377	0.84184	0.84562	0.84689	0.85098	0.85543	0.86034
576	0.83400	0.84205	0.84583	0.84709	0.85118	0.85562	0.86053
577	0.83423	0.84226	0.84603	0.84730	0.85138	0.85581	0.86071
578	0.83445	0.84247	0.84624	0.84750	0.85158	0.85601	0.86090
579	0.83466	0.84269	0.84645	0.84770	0.85178	0.85620	0.86108
580	0.83488	0.84290	0.84666	0.84791	0.85198	0.85639	0.86127
581	0.83511	0.84311	0.84686	0.84811	0.85218	0.85658	0.86145
582	0.83534	0.84332	0.84706	0.84831	0.85237	0.85676	0.86163
583	0.83554	0.84352	0.84726	0.84850	0.85257	0.85696	0.86181
584	0.83575	0.84373	0.84746	0.84871	0.85276	0.85714	0.86199
585	0.83598	0.84393	0.84767	0.84891	0.85296	0.85733	0.86217
586	0.83621	0.84414	0.84787	0.84911	0.85315	0.85752	0.86235
587	0.83641	0.84435	0.84808	0.84931	0.85335	0.85770	0.86253
588	0.83661	0.84455	0.84828	0.84951	0.85354	0.85789	0.86271
589	0.83684	0.84476	0.84847	0.84971	0.85373	0.85808	0.86289
590	0.83703	0.84498	0.84867	0.84991	0.85392	0.85826	0.86307
591	0.83724	0.84517	0.84886	0.85010	0.85412	0.85845	0.86325
592	0.83747	0.84538	0.84907	0.85030	0.85431	0.85863	0.86343
593	0.83769	0.84559	0.84927	0.85050	0.85450	0.85881	0.86360
594	0.83791	0.84579	0.84946	0.85070	0.85469	0.85900	0.86378
595	0.83812	0.84599	0.84966	0.85090	0.85487	0.85918	0.86396
596	0.83833	0.84619	0.84986	0.85109	0.85506	0.85936	0.86413
597	0.83854	0.84639	0.85006	0.85128	0.85525	0.85954	0.86430
598	0.83875	0.84658	0.85025	0.85147	0.85543	0.85972	0.86448
599	0.83896	0.84679	0.85045	0.85167	0.85562	0.85990	0.86465
600	0.83916	0.84698	0.85064	0.85186	0.85580	0.86008	0.86482
601	0.83938	0.84718	0.85082	0.85205	0.85599	0.86026	0.86500
602	0.83958	0.84738	0.85102	0.85224	0.85618	0.86044	0.86517
603	0.83980	0.84756	0.85121	0.85243	0.85637	0.86062	0.86534
604	0.84000	0.84776	0.85140	0.85261	0.85655	0.86079	0.86551
605	0.84020	0.84796	0.85159	0.85281	0.85674	0.86097	0.86568
606	0.84041	0.84816	0.85178	0.85300	0.85692	0.86115	0.86585
607	0.84061	0.84835	0.85197	0.85318	0.85710	0.86132	0.86602
608	0.84082	0.84855	0.85216	0.85336	0.85728	0.86150	0.86619
609	0.84102	0.84874	0.85234	0.85355	0.85746	0.86168	0.86636
610	0.84123	0.84893	0.85253	0.85374	0.85764	0.86185	0.86653
611	0.84143	0.84912	0.85272	0.85392	0.85782	0.86202	0.86670
612	0.84163	0.84932	0.85291	0.85411	0.85800	0.86220	0.86687
613	0.84183	0.84951	0.85309	0.85430	0.85818	0.86238	0.86703
614	0.84202	0.84970	0.85327	0.85448	0.85836	0.86255	0.86720
615	0.84224	0.84990	0.85346	0.85465	0.85854	0.86272	0.86737
616	0.84242	0.85008	0.85364	0.85484	0.85872	0.86289	0.86753
617	0.84264	0.85027	0.85383	0.85503	0.85890	0.86306	0.86770
618	0.84283	0.85047	0.85401	0.85521	0.85908	0.86324	0.86786
619	0.84303	0.85065	0.85420	0.85539	0.85926	0.86341	0.86803
620	0.84322	0.85084	0.85439	0.85557	0.85943	0.86358	0.86819
621	0.84342	0.85103	0.85457	0.85575	0.85961	0.86375	0.86836
622	0.84362	0.85121	0.85475	0.85593	0.85978	0.86392	0.86852
623	0.84383	0.85140	0.85493	0.85612	0.85996	0.86409	0.86868
624	0.84402	0.85157	0.85511	0.85630	0.86013	0.86426	0.86884
625	0.84421	0.85176	0.85529	0.85648	0.86030	0.86442	0.86900
626	0.84443	0.85195	0.85548	0.85665	0.86047	0.86459	0.86916
627	0.84462	0.85213	0.85565	0.85683	0.86065	0.86476	0.86933
628	0.84482	0.85232	0.85584	0.85701	0.86082	0.86492	0.86949
629	0.84501	0.85251	0.85602	0.85719	0.86099	0.86509	0.86965
630	0.84521	0.85269	0.85619	0.85736	0.86116	0.86526	0.86981
631	0.84540	0.85287	0.85637	0.85754	0.86133	0.86542	0.86997
632	0.84560	0.85305	0.85654	0.85772	0.86150	0.86559	0.87013
633	0.84578	0.85322	0.85672	0.85788	0.86167	0.86575	0.87028
634	0.84598	0.85341	0.85689	0.85806	0.86184	0.86592	0.87044
635	0.84617	0.85359	0.85707	0.85823	0.86201	0.86608	0.87060

N	0.1%	0.5%	1%	1.25%	2.5%	5%	10%
636	0.84633	0.85377	0.85724	0.85841	0.86217	0.86624	0.87076
637	0.84655	0.85396	0.85742	0.85857	0.86234	0.86641	0.87091
638	0.84673	0.85414	0.85760	0.85874	0.86251	0.86657	0.87107
639	0.84691	0.85431	0.85777	0.85892	0.86268	0.86673	0.87123
640	0.84710	0.85449	0.85794	0.85909	0.86284	0.86689	0.87138
641	0.84729	0.85467	0.85811	0.85926	0.86301	0.86705	0.87153
642	0.84746	0.85485	0.85828	0.85944	0.86318	0.86721	0.87169
643	0.84765	0.85502	0.85845	0.85960	0.86334	0.86737	0.87184
644	0.84781	0.85519	0.85862	0.85977	0.86351	0.86753	0.87199
645	0.84800	0.85537	0.85879	0.85993	0.86367	0.86769	0.87214
646	0.84819	0.85554	0.85897	0.86011	0.86383	0.86785	0.87230
647	0.84837	0.85572	0.85914	0.86028	0.86399	0.86801	0.87245
648	0.84854	0.85589	0.85931	0.86044	0.86416	0.86816	0.87260
649	0.84872	0.85607	0.85947	0.86061	0.86432	0.86832	0.87275
650	0.84892	0.85624	0.85964	0.86078	0.86448	0.86848	0.87290
651	0.84910	0.85642	0.85981	0.86095	0.86464	0.86863	0.87305
652	0.84927	0.85658	0.85998	0.86112	0.86481	0.86879	0.87320
653	0.84945	0.85676	0.86015	0.86129	0.86497	0.86895	0.87335
654	0.84963	0.85693	0.86032	0.86145	0.86513	0.86910	0.87350
655	0.84983	0.85710	0.86048	0.86162	0.86529	0.86925	0.87365
656	0.85002	0.85726	0.86065	0.86179	0.86545	0.86941	0.87380
657	0.85018	0.85743	0.86082	0.86195	0.86561	0.86956	0.87395
658	0.85034	0.85760	0.86098	0.86211	0.86577	0.86972	0.87410
659	0.85052	0.85778	0.86115	0.86228	0.86593	0.86987	0.87425
660	0.85069	0.85794	0.86131	0.86244	0.86609	0.87002	0.87439
661	0.85087	0.85810	0.86147	0.86260	0.86624	0.87018	0.87454
662	0.85105	0.85827	0.86163	0.86276	0.86640	0.87033	0.87469
663	0.85122	0.85844	0.86180	0.86293	0.86656	0.87048	0.87484
664	0.85137	0.85860	0.86196	0.86309	0.86672	0.87063	0.87498
665	0.85154	0.85877	0.86212	0.86325	0.86687	0.87078	0.87512
666	0.85173	0.85894	0.86229	0.86341	0.86703	0.87094	0.87527
667	0.85190	0.85911	0.86245	0.86357	0.86719	0.87109	0.87541
668	0.85207	0.85927	0.86261	0.86373	0.86734	0.87124	0.87556
669	0.85225	0.85943	0.86277	0.86389	0.86749	0.87139	0.87570
670	0.85243	0.85960	0.86293	0.86404	0.86765	0.87153	0.87584
671	0.85260	0.85977	0.86309	0.86420	0.86780	0.87168	0.87599
672	0.85277	0.85993	0.86325	0.86436	0.86795	0.87183	0.87613
673	0.85294	0.86009	0.86341	0.86452	0.86811	0.87198	0.87627
674	0.85312	0.86025	0.86357	0.86468	0.86826	0.87213	0.87641
675	0.85329	0.86043	0.86373	0.86483	0.86841	0.87227	0.87656
676	0.85346	0.86059	0.86389	0.86499	0.86856	0.87242	0.87670
677	0.85365	0.86076	0.86404	0.86515	0.86871	0.87257	0.87684
678	0.85381	0.86092	0.86420	0.86530	0.86886	0.87271	0.87698
679	0.85398	0.86108	0.86436	0.86546	0.86902	0.87286	0.87712
680	0.85415	0.86124	0.86451	0.86561	0.86916	0.87301	0.87726
681	0.85433	0.86140	0.86467	0.86577	0.86931	0.87315	0.87740
682	0.85450	0.86156	0.86482	0.86592	0.86946	0.87330	0.87754
683	0.85465	0.86172	0.86498	0.86607	0.86961	0.87344	0.87768
684	0.85482	0.86187	0.86514	0.86622	0.86976	0.87359	0.87781
685	0.85499	0.86203	0.86529	0.86638	0.86991	0.87373	0.87795
686	0.85517	0.86219	0.86545	0.86653	0.87006	0.87387	0.87809
687	0.85534	0.86234	0.86560	0.86668	0.87020	0.87401	0.87823
688	0.85552	0.86250	0.86576	0.86683	0.87035	0.87416	0.87836
689	0.85568	0.86266	0.86591	0.86699	0.87050	0.87430	0.87850
690	0.85584	0.86281	0.86606	0.86714	0.87064	0.87444	0.87864
691	0.85600	0.86297	0.86621	0.86728	0.87079	0.87458	0.87877
692	0.85616	0.86313	0.86636	0.86744	0.87093	0.87472	0.87891
693	0.85633	0.86329	0.86651	0.86758	0.87108	0.87486	0.87904
694	0.85648	0.86344	0.86666	0.86773	0.87122	0.87500	0.87918
695	0.85664	0.86360	0.86681	0.86788	0.87137	0.87514	0.87932
696	0.85681	0.86375	0.86695	0.86803	0.87151	0.87528	0.87945
697	0.85698	0.86390	0.86710	0.86819	0.87166	0.87543	0.87958
698	0.85715	0.86406	0.86725	0.86833	0.87180	0.87556	0.87972
699	0.85732	0.86421	0.86740	0.86847	0.87194	0.87570	0.87985
700	0.85746	0.86436	0.86755	0.86862	0.87208	0.87584	0.87999
701	0.85762	0.86452	0.86770	0.86877	0.87223	0.87598	0.88012
702	0.85778	0.86466	0.86784	0.86891	0.87237	0.87612	0.88025
703	0.85794	0.86482	0.86799	0.86906	0.87251	0.87625	0.88038
704	0.85811	0.86497	0.86814	0.86921	0.87265	0.87639	0.88051
705	0.85826	0.86512	0.86829	0.86935	0.87279	0.87653	0.88064
706	0.85842	0.86528	0.86843	0.86949	0.87294	0.87667	0.88077
707	0.85858	0.86542	0.86858	0.86965	0.87308	0.87680	0.88090
708	0.85874	0.86557	0.86872	0.86978	0.87322	0.87694	0.88103
709	0.85892	0.86572	0.86887	0.86993	0.87336	0.87707	0.88117

N	0.1%	0.5%	1%	1.25%	2.5%	5%	10%
710	0.85907	0.86587	0.86901	0.87007	0.87350	0.87721	0.88130
711	0.85922	0.86602	0.86915	0.87021	0.87363	0.87734	0.88142
712	0.85937	0.86617	0.86930	0.87036	0.87377	0.87748	0.88155
713	0.85953	0.86632	0.86944	0.87050	0.87392	0.87761	0.88168
714	0.85968	0.86646	0.86958	0.87064	0.87405	0.87774	0.88181
715	0.85984	0.86662	0.86973	0.87078	0.87419	0.87788	0.88194
716	0.85999	0.86676	0.86987	0.87093	0.87433	0.87801	0.88207
717	0.86015	0.86690	0.87001	0.87107	0.87446	0.87814	0.88220
718	0.86031	0.86705	0.87015	0.87121	0.87460	0.87827	0.88232
719	0.86046	0.86721	0.87030	0.87135	0.87474	0.87841	0.88245
720	0.86061	0.86735	0.87044	0.87149	0.87487	0.87854	0.88258
721	0.86076	0.86749	0.87059	0.87163	0.87501	0.87867	0.88271
722	0.86092	0.86764	0.87072	0.87177	0.87514	0.87880	0.88283
723	0.86108	0.86778	0.87086	0.87191	0.87528	0.87893	0.88296
724	0.86122	0.86792	0.87100	0.87204	0.87542	0.87906	0.88308
725	0.86139	0.86806	0.87114	0.87219	0.87555	0.87919	0.88321
726	0.86154	0.86821	0.87129	0.87232	0.87569	0.87932	0.88333
727	0.86168	0.86835	0.87142	0.87247	0.87582	0.87945	0.88346
728	0.86184	0.86849	0.87156	0.87260	0.87595	0.87958	0.88358
729	0.86199	0.86864	0.87170	0.87274	0.87608	0.87971	0.88371
730	0.86215	0.86878	0.87184	0.87288	0.87622	0.87983	0.88383
731	0.86230	0.86893	0.87198	0.87301	0.87635	0.87996	0.88395
732	0.86246	0.86907	0.87212	0.87315	0.87649	0.88009	0.88408
733	0.86260	0.86921	0.87226	0.87329	0.87662	0.88022	0.88420
734	0.86275	0.86935	0.87239	0.87343	0.87675	0.88035	0.88432
735	0.86290	0.86949	0.87253	0.87356	0.87688	0.88047	0.88445
736	0.86306	0.86963	0.87266	0.87369	0.87701	0.88060	0.88457
737	0.86320	0.86978	0.87280	0.87383	0.87714	0.88073	0.88469
738	0.86333	0.86991	0.87294	0.87396	0.87728	0.88085	0.88481
739	0.86349	0.87005	0.87307	0.87410	0.87741	0.88098	0.88493
740	0.86363	0.87019	0.87321	0.87423	0.87754	0.88111	0.88505
741	0.86378	0.87033	0.87335	0.87437	0.87767	0.88123	0.88518
742	0.86392	0.87046	0.87348	0.87450	0.87780	0.88136	0.88530
743	0.86407	0.87060	0.87362	0.87463	0.87792	0.88148	0.88542
744	0.86421	0.87074	0.87375	0.87477	0.87805	0.88160	0.88553
745	0.86435	0.87088	0.87388	0.87490	0.87818	0.88173	0.88565
746	0.86449	0.87101	0.87401	0.87503	0.87831	0.88185	0.88577
747	0.86463	0.87115	0.87416	0.87516	0.87844	0.88197	0.88589
748	0.86479	0.87128	0.87428	0.87529	0.87857	0.88210	0.88601
749	0.86492	0.87142	0.87442	0.87542	0.87869	0.88222	0.88613
750	0.86506	0.87155	0.87455	0.87555	0.87882	0.88234	0.88625
751	0.86521	0.87169	0.87468	0.87569	0.87895	0.88246	0.88637
752	0.86535	0.87183	0.87481	0.87582	0.87907	0.88258	0.88648
753	0.86549	0.87196	0.87494	0.87595	0.87920	0.88271	0.88660
754	0.86562	0.87210	0.87508	0.87607	0.87933	0.88283	0.88672
755	0.86577	0.87223	0.87521	0.87621	0.87945	0.88295	0.88683
756	0.86592	0.87236	0.87534	0.87633	0.87958	0.88308	0.88695
757	0.86606	0.87250	0.87547	0.87646	0.87971	0.88320	0.88707
758	0.86621	0.87264	0.87560	0.87659	0.87983	0.88332	0.88718
759	0.86634	0.87277	0.87573	0.87672	0.87996	0.88344	0.88730
760	0.86648	0.87290	0.87585	0.87685	0.88008	0.88356	0.88742
761	0.86662	0.87303	0.87598	0.87698	0.88020	0.88368	0.88753
762	0.86675	0.87316	0.87611	0.87711	0.88033	0.88380	0.88765
763	0.86690	0.87329	0.87624	0.87724	0.88045	0.88392	0.88776
764	0.86705	0.87342	0.87636	0.87736	0.88057	0.88404	0.88788
765	0.86718	0.87355	0.87650	0.87749	0.88069	0.88416	0.88799
766	0.86731	0.87368	0.87662	0.87761	0.88082	0.88427	0.88810
767	0.86745	0.87381	0.87675	0.87773	0.88094	0.88439	0.88822
768	0.86758	0.87395	0.87688	0.87786	0.88106	0.88451	0.88833
769	0.86772	0.87407	0.87700	0.87799	0.88118	0.88462	0.88844
770	0.86783	0.87421	0.87713	0.87812	0.88130	0.88474	0.88856
771	0.86797	0.87433	0.87725	0.87824	0.88142	0.88486	0.88867
772	0.86811	0.87446	0.87738	0.87836	0.88154	0.88498	0.88878
773	0.86824	0.87459	0.87750	0.87849	0.88166	0.88509	0.88890
774	0.86837	0.87471	0.87762	0.87861	0.88178	0.88521	0.88901
775	0.86851	0.87484	0.87775	0.87873	0.88190	0.88532	0.88912
776	0.86864	0.87497	0.87788	0.87885	0.88203	0.88544	0.88923
777	0.86878	0.87510	0.87800	0.87898	0.88214	0.88555	0.88934
778	0.86891	0.87522	0.87813	0.87910	0.88226	0.88567	0.88945
779	0.86905	0.87535	0.87825	0.87922	0.88238	0.88578	0.88956
780	0.86919	0.87548	0.87838	0.87934	0.88250	0.88590	0.88967
781	0.86933	0.87560	0.87849	0.87946	0.88262	0.88601	0.88978
782	0.86945	0.87573	0.87862	0.87958	0.88274	0.88613	0.88989
783	0.86960	0.87586	0.87874	0.87971	0.88286	0.88624	0.89000

N	0.1%	0.5%	1%	1.25%	2.5%	5%	10%
784	0.86974	0.87598	0.87886	0.87983	0.88298	0.88635	0.89011
785	0.86986	0.87611	0.87899	0.87995	0.88309	0.88647	0.89022
786	0.86998	0.87623	0.87911	0.88007	0.88321	0.88658	0.89033
787	0.87012	0.87636	0.87923	0.88019	0.88333	0.88669	0.89044
788	0.87025	0.87648	0.87936	0.88031	0.88345	0.88681	0.89055
789	0.87037	0.87661	0.87947	0.88043	0.88356	0.88692	0.89066
790	0.87052	0.87672	0.87959	0.88056	0.88368	0.88704	0.89077
791	0.87064	0.87685	0.87971	0.88068	0.88379	0.88715	0.89087
792	0.87078	0.87698	0.87983	0.88080	0.88391	0.88726	0.89098
793	0.87091	0.87710	0.87995	0.88091	0.88402	0.88737	0.89109
794	0.87103	0.87723	0.88008	0.88103	0.88414	0.88748	0.89120
795	0.87115	0.87735	0.88020	0.88115	0.88425	0.88759	0.89130
796	0.87128	0.87747	0.88031	0.88127	0.88436	0.88770	0.89141
797	0.87140	0.87759	0.88043	0.88139	0.88448	0.88781	0.89152
798	0.87153	0.87771	0.88055	0.88151	0.88459	0.88792	0.89162
799	0.87164	0.87783	0.88066	0.88162	0.88471	0.88803	0.89173
800	0.87177	0.87796	0.88078	0.88174	0.88482	0.88814	0.89183
801	0.87190	0.87808	0.88090	0.88185	0.88493	0.88825	0.89194
802	0.87203	0.87820	0.88102	0.88197	0.88505	0.88836	0.89205
803	0.87216	0.87831	0.88113	0.88208	0.88516	0.88847	0.89215
804	0.87229	0.87843	0.88125	0.88220	0.88527	0.88858	0.89226
805	0.87241	0.87855	0.88137	0.88231	0.88539	0.88869	0.89236
806	0.87255	0.87867	0.88149	0.88243	0.88550	0.88880	0.89246
807	0.87267	0.87879	0.88160	0.88254	0.88561	0.88890	0.89257
808	0.87280	0.87891	0.88172	0.88266	0.88572	0.88901	0.89268
809	0.87293	0.87903	0.88184	0.88277	0.88583	0.88912	0.89278
810	0.87306	0.87914	0.88195	0.88289	0.88594	0.88923	0.89288
811	0.87320	0.87927	0.88207	0.88300	0.88606	0.88933	0.89298
812	0.87332	0.87938	0.88218	0.88312	0.88616	0.88944	0.89309
813	0.87343	0.87950	0.88230	0.88323	0.88627	0.88955	0.89319
814	0.87356	0.87962	0.88241	0.88334	0.88638	0.88966	0.89329
815	0.87369	0.87974	0.88253	0.88345	0.88650	0.88976	0.89339
816	0.87380	0.87985	0.88264	0.88357	0.88661	0.88987	0.89350
817	0.87393	0.87997	0.88275	0.88368	0.88672	0.88998	0.89360
818	0.87406	0.88009	0.88286	0.88379	0.88683	0.89008	0.89370
819	0.87419	0.88020	0.88298	0.88391	0.88694	0.89019	0.89380
820	0.87431	0.88032	0.88309	0.88402	0.88705	0.89029	0.89390
821	0.87443	0.88044	0.88320	0.88413	0.88715	0.89040	0.89400
822	0.87455	0.88056	0.88332	0.88425	0.88726	0.89050	0.89411
823	0.87468	0.88067	0.88343	0.88436	0.88737	0.89061	0.89421
824	0.87480	0.88079	0.88354	0.88447	0.88748	0.89071	0.89431
825	0.87492	0.88090	0.88365	0.88458	0.88758	0.89082	0.89441
826	0.87505	0.88102	0.88377	0.88469	0.88769	0.89092	0.89451
827	0.87517	0.88113	0.88388	0.88480	0.88780	0.89103	0.89461
828	0.87529	0.88124	0.88399	0.88491	0.88791	0.89113	0.89471
829	0.87541	0.88136	0.88410	0.88502	0.88801	0.89123	0.89481
830	0.87553	0.88147	0.88421	0.88513	0.88812	0.89134	0.89491
831	0.87565	0.88158	0.88432	0.88524	0.88823	0.89144	0.89501
832	0.87576	0.88169	0.88443	0.88535	0.88833	0.89154	0.89510
833	0.87589	0.88180	0.88454	0.88546	0.88844	0.89164	0.89520
834	0.87600	0.88192	0.88465	0.88557	0.88854	0.89174	0.89530
835	0.87612	0.88204	0.88476	0.88568	0.88865	0.89185	0.89540
836	0.87624	0.88215	0.88487	0.88579	0.88876	0.89195	0.89550
837	0.87636	0.88226	0.88497	0.88589	0.88886	0.89205	0.89559
838	0.87646	0.88237	0.88509	0.88600	0.88896	0.89215	0.89569
839	0.87658	0.88248	0.88519	0.88611	0.88907	0.89225	0.89579
840	0.87671	0.88259	0.88530	0.88622	0.88917	0.89235	0.89589
841	0.87684	0.88271	0.88541	0.88632	0.88928	0.89246	0.89598
842	0.87696	0.88282	0.88552	0.88643	0.88938	0.89256	0.89608
843	0.87708	0.88293	0.88563	0.88654	0.88948	0.89266	0.89618
844	0.87719	0.88304	0.88574	0.88664	0.88959	0.89276	0.89628
845	0.87730	0.88315	0.88585	0.88675	0.88969	0.89286	0.89637
846	0.87742	0.88326	0.88595	0.88686	0.88979	0.89296	0.89647
847	0.87754	0.88337	0.88606	0.88697	0.88990	0.89306	0.89656
848	0.87765	0.88348	0.88617	0.88707	0.89000	0.89315	0.89666
849	0.87777	0.88359	0.88627	0.88718	0.89010	0.89325	0.89676
850	0.87788	0.88369	0.88637	0.88728	0.89020	0.89335	0.89685
851	0.87799	0.88380	0.88648	0.88739	0.89030	0.89345	0.89695
852	0.87810	0.88391	0.88658	0.88749	0.89041	0.89355	0.89704
853	0.87822	0.88402	0.88669	0.88760	0.89051	0.89365	0.89714
854	0.87833	0.88413	0.88680	0.88770	0.89061	0.89375	0.89723
855	0.87844	0.88423	0.88690	0.88781	0.89071	0.89385	0.89733
856	0.87856	0.88434	0.88700	0.88791	0.89081	0.89394	0.89742
857	0.87867	0.88445	0.88710	0.88801	0.89091	0.89404	0.89752

N	0.1%	0.5%	1%	1.25%	2.5%	5%	10%
858	0.87879	0.88456	0.88721	0.88812	0.89101	0.89414	0.89761
859	0.87891	0.88467	0.88732	0.88822	0.89111	0.89423	0.89770
860	0.87901	0.88478	0.88742	0.88832	0.89121	0.89433	0.89780
861	0.87912	0.88488	0.88753	0.88843	0.89131	0.89443	0.89789
862	0.87925	0.88498	0.88763	0.88853	0.89141	0.89453	0.89798
863	0.87934	0.88509	0.88774	0.88863	0.89151	0.89462	0.89807
864	0.87945	0.88519	0.88784	0.88873	0.89161	0.89472	0.89817
865	0.87956	0.88530	0.88794	0.88884	0.89171	0.89481	0.89826
866	0.87968	0.88540	0.88804	0.88894	0.89181	0.89491	0.89835
867	0.87979	0.88551	0.88814	0.88904	0.89191	0.89501	0.89845
868	0.87991	0.88562	0.88825	0.88914	0.89200	0.89510	0.89854
869	0.88002	0.88572	0.88835	0.88924	0.89211	0.89520	0.89863
870	0.88013	0.88584	0.88845	0.88934	0.89220	0.89529	0.89872
871	0.88025	0.88594	0.88856	0.88944	0.89230	0.89538	0.89881
872	0.88036	0.88604	0.88865	0.88954	0.89240	0.89548	0.89891
873	0.88045	0.88614	0.88875	0.88964	0.89250	0.89557	0.89900
874	0.88057	0.88625	0.88886	0.88974	0.89260	0.89567	0.89909
875	0.88068	0.88635	0.88896	0.88984	0.89269	0.89576	0.89918
876	0.88079	0.88645	0.88906	0.88994	0.89279	0.89586	0.89927
877	0.88090	0.88655	0.88916	0.89004	0.89289	0.89595	0.89936
878	0.88101	0.88666	0.88926	0.89014	0.89298	0.89604	0.89945
879	0.88112	0.88676	0.88936	0.89024	0.89308	0.89614	0.89954
880	0.88123	0.88686	0.88946	0.89033	0.89317	0.89623	0.89963
881	0.88133	0.88697	0.88955	0.89043	0.89327	0.89632	0.89972
882	0.88144	0.88707	0.88965	0.89053	0.89337	0.89642	0.89981
883	0.88155	0.88717	0.88975	0.89063	0.89346	0.89651	0.89990
884	0.88165	0.88727	0.88985	0.89072	0.89356	0.89660	0.89999
885	0.88175	0.88737	0.88995	0.89082	0.89366	0.89670	0.90008
886	0.88186	0.88747	0.89005	0.89092	0.89375	0.89679	0.90016
887	0.88197	0.88757	0.89015	0.89102	0.89385	0.89688	0.90025
888	0.88207	0.88766	0.89025	0.89112	0.89394	0.89697	0.90034
889	0.88218	0.88777	0.89035	0.89122	0.89403	0.89706	0.90043
890	0.88229	0.88787	0.89045	0.89132	0.89413	0.89715	0.90052
891	0.88240	0.88797	0.89054	0.89141	0.89422	0.89725	0.90061
892	0.88250	0.88807	0.89064	0.89151	0.89432	0.89734	0.90069
893	0.88261	0.88817	0.89074	0.89160	0.89441	0.89743	0.90078
894	0.88271	0.88827	0.89084	0.89170	0.89451	0.89752	0.90087
895	0.88282	0.88837	0.89094	0.89180	0.89460	0.89761	0.90095
896	0.88293	0.88848	0.89103	0.89189	0.89469	0.89770	0.90104
897	0.88303	0.88857	0.89113	0.89199	0.89478	0.89779	0.90113
898	0.88314	0.88867	0.89123	0.89209	0.89488	0.89788	0.90122
899	0.88324	0.88877	0.89132	0.89218	0.89497	0.89797	0.90130
900	0.88335	0.88887	0.89142	0.89228	0.89506	0.89806	0.90139
901	0.88345	0.88897	0.89152	0.89237	0.89516	0.89815	0.90148
902	0.88355	0.88907	0.89161	0.89246	0.89525	0.89824	0.90156
903	0.88364	0.88916	0.89171	0.89256	0.89534	0.89833	0.90165
904	0.88375	0.88926	0.89180	0.89266	0.89543	0.89842	0.90173
905	0.88384	0.88936	0.89190	0.89275	0.89553	0.89851	0.90182
906	0.88395	0.88946	0.89200	0.89285	0.89562	0.89860	0.90190
907	0.88406	0.88955	0.89209	0.89294	0.89571	0.89869	0.90199
908	0.88416	0.88965	0.89219	0.89303	0.89580	0.89877	0.90207
909	0.88427	0.88975	0.89228	0.89313	0.89589	0.89886	0.90216
910	0.88438	0.88984	0.89238	0.89322	0.89598	0.89895	0.90224
911	0.88447	0.88994	0.89247	0.89332	0.89607	0.89904	0.90233
912	0.88458	0.89004	0.89257	0.89341	0.89616	0.89912	0.90241
913	0.88467	0.89014	0.89266	0.89350	0.89625	0.89921	0.90250
914	0.88477	0.89023	0.89275	0.89360	0.89634	0.89930	0.90258
915	0.88487	0.89033	0.89285	0.89369	0.89643	0.89939	0.90266
916	0.88496	0.89042	0.89294	0.89378	0.89652	0.89947	0.90275
917	0.88507	0.89052	0.89304	0.89388	0.89661	0.89956	0.90283
918	0.88517	0.89061	0.89313	0.89397	0.89670	0.89965	0.90292
919	0.88526	0.89071	0.89322	0.89406	0.89679	0.89973	0.90300
920	0.88537	0.89081	0.89332	0.89415	0.89688	0.89982	0.90308
921	0.88548	0.89090	0.89341	0.89424	0.89697	0.89991	0.90316
922	0.88558	0.89100	0.89350	0.89433	0.89706	0.89999	0.90325
923	0.88568	0.89110	0.89359	0.89443	0.89715	0.90008	0.90333
924	0.88579	0.89119	0.89368	0.89452	0.89724	0.90016	0.90341
925	0.88588	0.89129	0.89377	0.89461	0.89733	0.90025	0.90349
926	0.88599	0.89138	0.89387	0.89470	0.89742	0.90033	0.90357
927	0.88608	0.89148	0.89396	0.89479	0.89750	0.90042	0.90366
928	0.88619	0.89157	0.89405	0.89488	0.89759	0.90050	0.90374
929	0.88629	0.89166	0.89414	0.89497	0.89768	0.90059	0.90382
930	0.88638	0.89176	0.89423	0.89506	0.89776	0.90067	0.90390
931	0.88648	0.89185	0.89432	0.89515	0.89785	0.90076	0.90398

N	0.1%	0.5%	1%	1.25%	2.5%	5%	10%
932	0.88657	0.89194	0.89441	0.89524	0.89794	0.90084	0.90407
933	0.88667	0.89204	0.89450	0.89533	0.89803	0.90092	0.90415
934	0.88677	0.89213	0.89460	0.89542	0.89811	0.90101	0.90423
935	0.88686	0.89222	0.89469	0.89551	0.89820	0.90109	0.90431
936	0.88696	0.89231	0.89477	0.89560	0.89828	0.90118	0.90439
937	0.88706	0.89241	0.89487	0.89569	0.89837	0.90126	0.90447
938	0.88715	0.89250	0.89496	0.89578	0.89846	0.90134	0.90455
939	0.88727	0.89259	0.89505	0.89586	0.89854	0.90143	0.90463
940	0.88736	0.89268	0.89513	0.89595	0.89863	0.90151	0.90471
941	0.88746	0.89277	0.89522	0.89604	0.89871	0.90160	0.90479
942	0.88756	0.89286	0.89531	0.89613	0.89880	0.90168	0.90487
943	0.88765	0.89295	0.89540	0.89622	0.89888	0.90176	0.90495
944	0.88775	0.89305	0.89549	0.89631	0.89897	0.90184	0.90503
945	0.88783	0.89313	0.89557	0.89640	0.89905	0.90192	0.90511
946	0.88793	0.89323	0.89566	0.89649	0.89914	0.90201	0.90519
947	0.88803	0.89332	0.89575	0.89657	0.89922	0.90209	0.90527
948	0.88813	0.89341	0.89584	0.89666	0.89931	0.90217	0.90535
949	0.88822	0.89350	0.89593	0.89674	0.89939	0.90225	0.90542
950	0.88833	0.89359	0.89602	0.89683	0.89947	0.90234	0.90550
951	0.88841	0.89368	0.89610	0.89692	0.89956	0.90242	0.90558
952	0.88851	0.89377	0.89619	0.89700	0.89964	0.90250	0.90566
953	0.88860	0.89386	0.89628	0.89709	0.89973	0.90258	0.90574
954	0.88869	0.89395	0.89637	0.89718	0.89981	0.90266	0.90582
955	0.88878	0.89404	0.89645	0.89726	0.89990	0.90274	0.90590
956	0.88887	0.89413	0.89654	0.89735	0.89998	0.90283	0.90597
957	0.88896	0.89421	0.89662	0.89743	0.90006	0.90290	0.90605
958	0.88905	0.89430	0.89671	0.89752	0.90015	0.90298	0.90613
959	0.88916	0.89439	0.89680	0.89761	0.90023	0.90307	0.90620
960	0.88925	0.89448	0.89689	0.89769	0.90031	0.90315	0.90628
961	0.88935	0.89457	0.89697	0.89777	0.90040	0.90323	0.90636
962	0.88944	0.89466	0.89706	0.89786	0.90048	0.90331	0.90643
963	0.88953	0.89475	0.89714	0.89795	0.90056	0.90338	0.90651
964	0.88962	0.89484	0.89722	0.89803	0.90064	0.90346	0.90659
965	0.88970	0.89493	0.89731	0.89812	0.90073	0.90354	0.90666
966	0.88979	0.89501	0.89739	0.89820	0.90081	0.90362	0.90674
967	0.88988	0.89511	0.89748	0.89828	0.90089	0.90370	0.90682
968	0.88998	0.89519	0.89756	0.89837	0.90097	0.90378	0.90689
969	0.89007	0.89528	0.89764	0.89845	0.90105	0.90386	0.90697
970	0.89017	0.89536	0.89773	0.89853	0.90113	0.90394	0.90705
971	0.89026	0.89545	0.89782	0.89861	0.90121	0.90402	0.90712
972	0.89035	0.89553	0.89790	0.89870	0.90129	0.90409	0.90720
973	0.89044	0.89562	0.89799	0.89878	0.90138	0.90417	0.90727
974	0.89054	0.89571	0.89807	0.89887	0.90145	0.90425	0.90735
975	0.89063	0.89579	0.89815	0.89895	0.90154	0.90433	0.90742
976	0.89073	0.89588	0.89823	0.89903	0.90161	0.90441	0.90750
977	0.89082	0.89596	0.89832	0.89912	0.90169	0.90449	0.90757
978	0.89091	0.89604	0.89840	0.89920	0.90177	0.90456	0.90765
979	0.89100	0.89613	0.89848	0.89928	0.90185	0.90464	0.90772
980	0.89108	0.89621	0.89857	0.89936	0.90193	0.90472	0.90780
981	0.89117	0.89630	0.89865	0.89944	0.90202	0.90479	0.90787
982	0.89126	0.89638	0.89873	0.89952	0.90210	0.90487	0.90795
983	0.89135	0.89646	0.89882	0.89961	0.90218	0.90495	0.90802
984	0.89145	0.89655	0.89890	0.89969	0.90225	0.90503	0.90809
985	0.89154	0.89664	0.89898	0.89977	0.90233	0.90510	0.90817
986	0.89163	0.89672	0.89906	0.89985	0.90241	0.90518	0.90824
987	0.89172	0.89681	0.89915	0.89993	0.90249	0.90526	0.90832
988	0.89181	0.89689	0.89923	0.90001	0.90257	0.90533	0.90839
989	0.89190	0.89697	0.89931	0.90010	0.90265	0.90541	0.90846
990	0.89199	0.89706	0.89939	0.90017	0.90273	0.90548	0.90854
991	0.89207	0.89714	0.89947	0.90025	0.90281	0.90556	0.90861
992	0.89216	0.89722	0.89955	0.90033	0.90289	0.90564	0.90868
993	0.89225	0.89731	0.89963	0.90042	0.90296	0.90571	0.90876
994	0.89234	0.89739	0.89972	0.90050	0.90305	0.90579	0.90883
995	0.89243	0.89747	0.89980	0.90058	0.90312	0.90586	0.90890
996	0.89251	0.89755	0.89988	0.90066	0.90320	0.90594	0.90897
997	0.89259	0.89764	0.89996	0.90074	0.90328	0.90601	0.90905
998	0.89268	0.89772	0.90004	0.90082	0.90335	0.90609	0.90912
999	0.89277	0.89780	0.90012	0.90090	0.90343	0.90616	0.90919
1000	0.89286	0.89789	0.90020	0.90098	0.90351	0.90624	0.90926

Table 5 – *GM10(6H4L or 4H6L)*. Critical values of upper significance levels for the Grubbs multiple-10 outlier test, applicable in situations where the six highest and four lowest or four highest and six lowest

observations are simultaneously identified as outliers, denoted as *GM10(6H4L)* and *GM10(4H6L)*, respectively.

N	0.1%	0.5%	1%	1.25%	2.5%	5%	10%
12	0.00000	0.00000	0.00000	0.00000	0.00000	0.00001	0.00003
13	0.00001	0.00003	0.00006	0.00007	0.00015	0.00031	0.00069
14	0.00009	0.00028	0.00046	0.00054	0.00090	0.00153	0.00269
15	0.00043	0.00103	0.00152	0.00172	0.00258	0.00393	0.00615
16	0.00121	0.00248	0.00341	0.00379	0.00530	0.00753	0.01097
17	0.00254	0.00470	0.00619	0.00677	0.00904	0.01224	0.01696
18	0.00450	0.00771	0.00981	0.01063	0.01372	0.01793	0.02391
19	0.00712	0.01147	0.01422	0.01527	0.01918	0.02442	0.03165
20	0.01030	0.01588	0.01935	0.02064	0.02540	0.03161	0.04001
21	0.01407	0.02093	0.02504	0.02659	0.03215	0.03932	0.04883
22	0.01840	0.02654	0.03131	0.03307	0.03943	0.04748	0.05802
23	0.02316	0.03255	0.03799	0.03997	0.04704	0.05596	0.06747
24	0.02842	0.03903	0.04502	0.04722	0.05502	0.06469	0.07710
25	0.03400	0.04574	0.05236	0.05476	0.06320	0.07363	0.08681
26	0.03991	0.05270	0.05992	0.06252	0.07163	0.08268	0.09662
27	0.04604	0.05994	0.06767	0.07043	0.08009	0.09177	0.10640
28	0.05247	0.06735	0.07553	0.07848	0.08863	0.10090	0.11618
29	0.05898	0.07479	0.08348	0.08659	0.09727	0.11007	0.12589
30	0.06573	0.08244	0.09150	0.09471	0.10591	0.11917	0.13551
31	0.07248	0.09013	0.09954	0.10289	0.11451	0.12823	0.14502
32	0.07941	0.09787	0.10767	0.11115	0.12311	0.13724	0.15441
33	0.08639	0.10564	0.11578	0.11936	0.13169	0.14618	0.16370
34	0.09335	0.11340	0.12384	0.12753	0.14016	0.15497	0.17284
35	0.10057	0.12111	0.13191	0.13568	0.14859	0.16365	0.18184
36	0.10766	0.12881	0.13989	0.14376	0.15690	0.17224	0.19068
37	0.11471	0.13648	0.14781	0.15178	0.16514	0.18071	0.19935
38	0.12183	0.14403	0.15561	0.15964	0.17326	0.18904	0.20788
39	0.12889	0.15158	0.16333	0.16738	0.18126	0.19723	0.21626
40	0.13595	0.15904	0.17092	0.17505	0.18912	0.20529	0.22450
41	0.14280	0.16643	0.17850	0.18271	0.19688	0.21320	0.23256
42	0.14977	0.17367	0.18593	0.19020	0.20453	0.22105	0.24050
43	0.15658	0.18089	0.19335	0.19760	0.21208	0.22870	0.24825
44	0.16342	0.18803	0.20061	0.20489	0.21951	0.23622	0.25586
45	0.17018	0.19509	0.20774	0.21211	0.22680	0.24363	0.26335
46	0.17678	0.20202	0.21477	0.21917	0.23397	0.25085	0.27064
47	0.18336	0.20887	0.22179	0.22616	0.24103	0.25798	0.27781
48	0.18998	0.21570	0.22862	0.23306	0.24797	0.26497	0.28486
49	0.19645	0.22232	0.23533	0.23984	0.25479	0.27184	0.29176
50	0.20278	0.22896	0.24199	0.24647	0.26150	0.27856	0.29852
51	0.20918	0.23537	0.24853	0.25309	0.26810	0.28519	0.30514
52	0.21550	0.24183	0.25501	0.25948	0.27459	0.29171	0.31163
53	0.22163	0.24807	0.26129	0.26583	0.28097	0.29811	0.31801
54	0.22774	0.25428	0.26758	0.27209	0.28720	0.30437	0.32427
55	0.23357	0.26043	0.27369	0.27821	0.29338	0.31053	0.33041
56	0.23934	0.26645	0.27973	0.28425	0.29943	0.31657	0.33642
57	0.24514	0.27239	0.28566	0.29021	0.30541	0.32250	0.34233
58	0.25101	0.27819	0.29149	0.29608	0.31125	0.32832	0.34811
59	0.25683	0.28397	0.29722	0.30180	0.31696	0.33405	0.35381
60	0.26252	0.28951	0.30291	0.30745	0.32260	0.33967	0.35939
61	0.26794	0.29503	0.30846	0.31301	0.32819	0.34522	0.36487
62	0.27338	0.30052	0.31390	0.31851	0.33364	0.35065	0.37025
63	0.27873	0.30596	0.31932	0.32386	0.33902	0.35600	0.37550
64	0.28399	0.31120	0.32458	0.32916	0.34427	0.36123	0.38070
65	0.28916	0.31643	0.32976	0.33439	0.34947	0.36637	0.38579
66	0.29434	0.32169	0.33494	0.33946	0.35453	0.37141	0.39077
67	0.29929	0.32667	0.33996	0.34451	0.35956	0.37637	0.39568
68	0.30426	0.33165	0.34492	0.34948	0.36450	0.38126	0.40052
69	0.30934	0.33659	0.34979	0.35436	0.36936	0.38607	0.40524
70	0.31421	0.34139	0.35465	0.35918	0.37414	0.39079	0.40989
71	0.31909	0.34621	0.35943	0.36392	0.37884	0.39544	0.41445
72	0.32373	0.35085	0.36406	0.36857	0.38347	0.40001	0.41897
73	0.32843	0.35550	0.36870	0.37320	0.38799	0.40450	0.42339
74	0.33287	0.36005	0.37329	0.37769	0.39246	0.40891	0.42774
75	0.33742	0.36452	0.37774	0.38218	0.39688	0.41330	0.43202
76	0.34185	0.36898	0.38214	0.38652	0.40120	0.41758	0.43622
77	0.34633	0.37335	0.38639	0.39087	0.40551	0.42177	0.44037
78	0.35092	0.37761	0.39065	0.39511	0.40968	0.42593	0.44443
79	0.35510	0.38187	0.39493	0.39933	0.41384	0.43003	0.44844
80	0.35929	0.38600	0.39903	0.40343	0.41792	0.43405	0.45240
81	0.36344	0.39017	0.40311	0.40746	0.42191	0.43800	0.45626
82	0.36756	0.39423	0.40710	0.41147	0.42586	0.44189	0.46008

N	0.1%	0.5%	1%	1.25%	2.5%	5%	10%
83	0.37171	0.39819	0.41109	0.41543	0.42978	0.44572	0.46385
84	0.37571	0.40211	0.41496	0.41931	0.43359	0.44951	0.46755
85	0.37975	0.40599	0.41881	0.42313	0.43741	0.45325	0.47119
86	0.38364	0.40982	0.42256	0.42688	0.44115	0.45691	0.47479
87	0.38739	0.41365	0.42628	0.43060	0.44481	0.46053	0.47831
88	0.39110	0.41743	0.43003	0.43433	0.44846	0.46408	0.48179
89	0.39479	0.42108	0.43365	0.43790	0.45200	0.46757	0.48522
90	0.39842	0.42474	0.43722	0.44151	0.45556	0.47103	0.48859
91	0.40218	0.42831	0.44079	0.44506	0.45901	0.47445	0.49192
92	0.40588	0.43188	0.44429	0.44855	0.46245	0.47781	0.49520
93	0.40948	0.43537	0.44772	0.45192	0.46582	0.48111	0.49843
94	0.41293	0.43879	0.45117	0.45528	0.46916	0.48437	0.50162
95	0.41647	0.44212	0.45447	0.45864	0.47241	0.48758	0.50477
96	0.41993	0.44551	0.45780	0.46197	0.47566	0.49077	0.50786
97	0.42332	0.44885	0.46109	0.46527	0.47885	0.49390	0.51091
98	0.42665	0.45216	0.46434	0.46845	0.48205	0.49699	0.51394
99	0.43000	0.45534	0.46748	0.47161	0.48514	0.50002	0.51691
100	0.43335	0.45856	0.47067	0.47473	0.48820	0.50303	0.51985
101	0.43645	0.46165	0.47373	0.47783	0.49126	0.50602	0.52274
102	0.43967	0.46481	0.47678	0.48086	0.49422	0.50897	0.52559
103	0.44287	0.46788	0.47984	0.48388	0.49718	0.51185	0.52842
104	0.44584	0.47093	0.48284	0.48684	0.50012	0.51468	0.53119
105	0.44889	0.47384	0.48579	0.48976	0.50299	0.51750	0.53393
106	0.45192	0.47683	0.48871	0.49271	0.50584	0.52027	0.53662
107	0.45497	0.47977	0.49157	0.49556	0.50866	0.52302	0.53930
108	0.45781	0.48266	0.49442	0.49842	0.51145	0.52574	0.54194
109	0.46094	0.48554	0.49724	0.50123	0.51416	0.52842	0.54454
110	0.46378	0.48835	0.50000	0.50394	0.51684	0.53105	0.54710
111	0.46671	0.49109	0.50275	0.50665	0.51950	0.53367	0.54963
112	0.46953	0.49388	0.50544	0.50936	0.52212	0.53626	0.55214
113	0.47231	0.49655	0.50813	0.51200	0.52476	0.53881	0.55462
114	0.47510	0.49925	0.51076	0.51462	0.52733	0.54132	0.55707
115	0.47777	0.50187	0.51336	0.51722	0.52987	0.54378	0.55948
116	0.48049	0.50451	0.51593	0.51977	0.53235	0.54624	0.56185
117	0.48319	0.50709	0.51848	0.52234	0.53485	0.54865	0.56420
118	0.48581	0.50968	0.52102	0.52485	0.53731	0.55104	0.56652
119	0.48847	0.51220	0.52351	0.52731	0.53974	0.55341	0.56881
120	0.49102	0.51473	0.52597	0.52974	0.54213	0.55574	0.57107
121	0.49368	0.51723	0.52840	0.53217	0.54449	0.55805	0.57333
122	0.49614	0.51968	0.53081	0.53459	0.54683	0.56034	0.57556
123	0.49866	0.52212	0.53319	0.53691	0.54917	0.56260	0.57773
124	0.50116	0.52457	0.53556	0.53928	0.55145	0.56484	0.57990
125	0.50361	0.52691	0.53785	0.54155	0.55374	0.56704	0.58204
126	0.50600	0.52922	0.54016	0.54386	0.55597	0.56921	0.58416
127	0.50837	0.53150	0.54239	0.54612	0.55817	0.57138	0.58626
128	0.51076	0.53382	0.54466	0.54835	0.56036	0.57352	0.58834
129	0.51308	0.53609	0.54691	0.55056	0.56253	0.57565	0.59040
130	0.51542	0.53831	0.54910	0.55276	0.56467	0.57772	0.59242
131	0.51784	0.54052	0.55132	0.55493	0.56677	0.57979	0.59441
132	0.52007	0.54270	0.55349	0.55709	0.56887	0.58182	0.59638
133	0.52235	0.54487	0.55560	0.55922	0.57095	0.58385	0.59835
134	0.52461	0.54705	0.55770	0.56127	0.57301	0.58585	0.60028
135	0.52685	0.54916	0.55980	0.56334	0.57504	0.58783	0.60220
136	0.52910	0.55127	0.56185	0.56539	0.57706	0.58979	0.60409
137	0.53123	0.55337	0.56389	0.56743	0.57904	0.59171	0.60595
138	0.53339	0.55541	0.56592	0.56946	0.58100	0.59363	0.60781
139	0.53548	0.55746	0.56792	0.57144	0.58293	0.59551	0.60964
140	0.53755	0.55950	0.56989	0.57342	0.58487	0.59739	0.61146
141	0.53964	0.56149	0.57187	0.57535	0.58676	0.59926	0.61326
142	0.54173	0.56345	0.57381	0.57729	0.58867	0.60110	0.61504
143	0.54381	0.56544	0.57577	0.57921	0.59053	0.60290	0.61680
144	0.54583	0.56741	0.57766	0.58110	0.59239	0.60472	0.61853
145	0.54788	0.56932	0.57953	0.58296	0.59423	0.60651	0.62025
146	0.54992	0.57120	0.58139	0.58484	0.59604	0.60827	0.62196
147	0.55182	0.57310	0.58328	0.58667	0.59784	0.61000	0.62365
148	0.55370	0.57494	0.58511	0.58847	0.59961	0.61172	0.62532
149	0.55560	0.57680	0.58690	0.59030	0.60137	0.61344	0.62698
150	0.55759	0.57867	0.58872	0.59209	0.60311	0.61514	0.62862
151	0.55943	0.58051	0.59050	0.59386	0.60483	0.61681	0.63024
152	0.56134	0.58231	0.59226	0.59562	0.60654	0.61847	0.63185
153	0.56325	0.58408	0.59403	0.59737	0.60823	0.62012	0.63343
154	0.56507	0.58582	0.59576	0.59909	0.60993	0.62175	0.63501
155	0.56690	0.58762	0.59749	0.60080	0.61159	0.62337	0.63657
156	0.56871	0.58935	0.59917	0.60248	0.61322	0.62497	0.63812

N	0.1%	0.5%	1%	1.25%	2.5%	5%	10%
157	0.57047	0.59102	0.60084	0.60415	0.61484	0.62655	0.63964
158	0.57223	0.59272	0.60251	0.60579	0.61646	0.62812	0.64116
159	0.57397	0.59445	0.60414	0.60742	0.61806	0.62967	0.64267
160	0.57575	0.59608	0.60576	0.60904	0.61966	0.63121	0.64415
161	0.57755	0.59776	0.60740	0.61065	0.62121	0.63273	0.64563
162	0.57927	0.59937	0.60899	0.61222	0.62277	0.63424	0.64709
163	0.58097	0.60102	0.61058	0.61380	0.62430	0.63574	0.64855
164	0.58274	0.60265	0.61217	0.61539	0.62583	0.63721	0.64997
165	0.58435	0.60425	0.61373	0.61695	0.62735	0.63868	0.65139
166	0.58596	0.60581	0.61529	0.61844	0.62883	0.64014	0.65280
167	0.58770	0.60738	0.61682	0.61996	0.63031	0.64158	0.65419
168	0.58930	0.60890	0.61832	0.62149	0.63180	0.64301	0.65558
169	0.59099	0.61047	0.61983	0.62299	0.63326	0.64444	0.65695
170	0.59254	0.61198	0.62133	0.62447	0.63469	0.64583	0.65831
171	0.59409	0.61353	0.62283	0.62595	0.63613	0.64723	0.65965
172	0.59567	0.61505	0.62429	0.62741	0.63755	0.64861	0.66098
173	0.59712	0.61655	0.62577	0.62885	0.63896	0.64997	0.66230
174	0.59864	0.61804	0.62722	0.63029	0.64035	0.65133	0.66361
175	0.60021	0.61951	0.62868	0.63173	0.64172	0.65267	0.66490
176	0.60172	0.62092	0.63008	0.63312	0.64310	0.65401	0.66619
177	0.60320	0.62238	0.63149	0.63454	0.64445	0.65533	0.66747
178	0.60467	0.62381	0.63287	0.63591	0.64580	0.65666	0.66874
179	0.60607	0.62521	0.63426	0.63727	0.64714	0.65796	0.67000
180	0.60753	0.62661	0.63564	0.63866	0.64847	0.65925	0.67124
181	0.60898	0.62801	0.63700	0.63998	0.64977	0.66052	0.67247
182	0.61052	0.62937	0.63834	0.64132	0.65108	0.66179	0.67369
183	0.61200	0.63076	0.63969	0.64267	0.65237	0.66303	0.67490
184	0.61338	0.63215	0.64100	0.64397	0.65365	0.66428	0.67610
185	0.61478	0.63346	0.64233	0.64529	0.65493	0.66551	0.67730
186	0.61616	0.63484	0.64363	0.64656	0.65618	0.66675	0.67848
187	0.61757	0.63616	0.64494	0.64786	0.65745	0.66795	0.67965
188	0.61892	0.63748	0.64622	0.64914	0.65868	0.66916	0.68083
189	0.62030	0.63878	0.64747	0.65038	0.65993	0.67034	0.68198
190	0.62169	0.64006	0.64873	0.65163	0.66115	0.67153	0.68313
191	0.62304	0.64133	0.64998	0.65288	0.66237	0.67271	0.68426
192	0.62435	0.64262	0.65123	0.65410	0.66356	0.67389	0.68538
193	0.62561	0.64391	0.65244	0.65533	0.66477	0.67504	0.68651
194	0.62694	0.64514	0.65368	0.65654	0.66594	0.67619	0.68762
195	0.62823	0.64637	0.65490	0.65776	0.66711	0.67734	0.68872
196	0.62951	0.64761	0.65610	0.65895	0.66827	0.67846	0.68981
197	0.63079	0.64879	0.65730	0.66015	0.66945	0.67958	0.69089
198	0.63203	0.65000	0.65848	0.66132	0.67059	0.68069	0.69196
199	0.63332	0.65119	0.65965	0.66249	0.67173	0.68180	0.69303
200	0.63456	0.65242	0.66082	0.66364	0.67286	0.68289	0.69409
201	0.63583	0.65362	0.66200	0.66480	0.67398	0.68398	0.69514
202	0.63703	0.65481	0.66314	0.66595	0.67509	0.68506	0.69618
203	0.63830	0.65597	0.66428	0.66706	0.67621	0.68613	0.69722
204	0.63948	0.65710	0.66540	0.66820	0.67730	0.68720	0.69824
205	0.64060	0.65828	0.66654	0.66930	0.67839	0.68826	0.69926
206	0.64178	0.65942	0.66765	0.67040	0.67947	0.68930	0.70027
207	0.64298	0.66059	0.66875	0.67152	0.68053	0.69033	0.70127
208	0.64415	0.66170	0.66986	0.67261	0.68160	0.69137	0.70227
209	0.64536	0.66284	0.67096	0.67371	0.68266	0.69240	0.70326
210	0.64657	0.66395	0.67204	0.67477	0.68371	0.69342	0.70424
211	0.64769	0.66505	0.67312	0.67586	0.68475	0.69442	0.70522
212	0.64878	0.66615	0.67420	0.67694	0.68578	0.69542	0.70617
213	0.64990	0.66721	0.67525	0.67800	0.68680	0.69641	0.70713
214	0.65104	0.66829	0.67631	0.67903	0.68784	0.69740	0.70808
215	0.65221	0.66938	0.67736	0.68007	0.68884	0.69839	0.70903
216	0.65329	0.67042	0.67840	0.68110	0.68985	0.69937	0.70997
217	0.65438	0.67148	0.67943	0.68212	0.69085	0.70033	0.71091
218	0.65548	0.67254	0.68046	0.68313	0.69184	0.70130	0.71184
219	0.65660	0.67359	0.68148	0.68415	0.69282	0.70224	0.71275
220	0.65772	0.67463	0.68249	0.68516	0.69379	0.70318	0.71366
221	0.65872	0.67564	0.68349	0.68615	0.69476	0.70412	0.71456
222	0.65987	0.67667	0.68449	0.68712	0.69573	0.70506	0.71546
223	0.66092	0.67767	0.68545	0.68809	0.69667	0.70598	0.71635
224	0.66199	0.67866	0.68643	0.68906	0.69762	0.70691	0.71724
225	0.66304	0.67967	0.68741	0.69001	0.69857	0.70782	0.71812
226	0.66410	0.68066	0.68836	0.69099	0.69950	0.70872	0.71899
227	0.66509	0.68166	0.68934	0.69195	0.70042	0.70962	0.71986
228	0.66612	0.68264	0.69029	0.69290	0.70134	0.71051	0.72073
229	0.66717	0.68361	0.69125	0.69386	0.70226	0.71140	0.72158
230	0.66822	0.68457	0.69219	0.69478	0.70316	0.71228	0.72243

N	0.1%	0.5%	1%	1.25%	2.5%	5%	10%
231	0.66924	0.68551	0.69312	0.69571	0.70406	0.71316	0.72328
232	0.67020	0.68647	0.69407	0.69663	0.70494	0.71403	0.72412
233	0.67118	0.68739	0.69500	0.69757	0.70584	0.71489	0.72495
234	0.67218	0.68834	0.69593	0.69847	0.70672	0.71574	0.72578
235	0.67314	0.68926	0.69684	0.69937	0.70760	0.71659	0.72660
236	0.67410	0.69020	0.69774	0.70027	0.70847	0.71744	0.72742
237	0.67510	0.69111	0.69863	0.70114	0.70933	0.71827	0.72822
238	0.67606	0.69201	0.69951	0.70204	0.71020	0.71910	0.72903
239	0.67704	0.69292	0.70042	0.70292	0.71107	0.71994	0.72983
240	0.67798	0.69383	0.70130	0.70380	0.71192	0.72076	0.73063
241	0.67895	0.69473	0.70216	0.70467	0.71275	0.72157	0.73142
242	0.67985	0.69563	0.70302	0.70553	0.71359	0.72238	0.73220
243	0.68077	0.69653	0.70388	0.70640	0.71442	0.72318	0.73298
244	0.68171	0.69742	0.70477	0.70724	0.71525	0.72400	0.73374
245	0.68265	0.69826	0.70563	0.70809	0.71608	0.72479	0.73452
246	0.68352	0.69912	0.70649	0.70894	0.71690	0.72559	0.73528
247	0.68444	0.70000	0.70731	0.70977	0.71772	0.72637	0.73603
248	0.68528	0.70088	0.70814	0.71060	0.71853	0.72716	0.73679
249	0.68625	0.70175	0.70898	0.71142	0.71932	0.72794	0.73754
250	0.68709	0.70260	0.70978	0.71222	0.72012	0.72871	0.73829
251	0.68801	0.70342	0.71062	0.71303	0.72092	0.72948	0.73903
252	0.68891	0.70426	0.71143	0.71386	0.72170	0.73025	0.73976
253	0.68977	0.70507	0.71225	0.71465	0.72248	0.73101	0.74049
254	0.69064	0.70590	0.71304	0.71546	0.72325	0.73175	0.74122
255	0.69151	0.70671	0.71384	0.71625	0.72403	0.73251	0.74194
256	0.69241	0.70754	0.71464	0.71705	0.72481	0.73325	0.74266
257	0.69325	0.70833	0.71545	0.71782	0.72556	0.73399	0.74337
258	0.69412	0.70914	0.71623	0.71861	0.72632	0.73472	0.74408
259	0.69499	0.70994	0.71701	0.71938	0.72707	0.73546	0.74479
260	0.69579	0.71071	0.71777	0.72015	0.72782	0.73618	0.74548
261	0.69663	0.71150	0.71855	0.72090	0.72857	0.73690	0.74617
262	0.69747	0.71230	0.71933	0.72167	0.72932	0.73762	0.74686
263	0.69827	0.71307	0.72008	0.72244	0.73005	0.73832	0.74754
264	0.69904	0.71384	0.72084	0.72319	0.73078	0.73903	0.74822
265	0.69987	0.71462	0.72160	0.72393	0.73151	0.73972	0.74890
266	0.70073	0.71539	0.72236	0.72469	0.73222	0.74042	0.74958
267	0.70153	0.71615	0.72310	0.72542	0.73295	0.74112	0.75025
268	0.70236	0.71693	0.72384	0.72615	0.73366	0.74181	0.75091
269	0.70313	0.71771	0.72457	0.72689	0.73436	0.74250	0.75157
270	0.70392	0.71846	0.72531	0.72761	0.73507	0.74318	0.75224
271	0.70469	0.71918	0.72604	0.72833	0.73577	0.74386	0.75289
272	0.70546	0.71993	0.72676	0.72906	0.73647	0.74454	0.75354
273	0.70622	0.72066	0.72750	0.72977	0.73716	0.74522	0.75419
274	0.70696	0.72142	0.72819	0.73047	0.73786	0.74588	0.75483
275	0.70769	0.72217	0.72891	0.73118	0.73855	0.74655	0.75547
276	0.70844	0.72289	0.72962	0.73189	0.73923	0.74721	0.75611
277	0.70925	0.72360	0.73031	0.73258	0.73991	0.74787	0.75674
278	0.71005	0.72431	0.73103	0.73328	0.74058	0.74852	0.75738
279	0.71079	0.72504	0.73172	0.73397	0.74125	0.74918	0.75800
280	0.71151	0.72574	0.73241	0.73465	0.74192	0.74982	0.75862
281	0.71232	0.72643	0.73309	0.73533	0.74258	0.75046	0.75924
282	0.71302	0.72714	0.73379	0.73601	0.74324	0.75110	0.75985
283	0.71375	0.72785	0.73447	0.73667	0.74389	0.75173	0.76046
284	0.71447	0.72855	0.73515	0.73735	0.74455	0.75236	0.76107
285	0.71522	0.72926	0.73582	0.73802	0.74520	0.75299	0.76167
286	0.71592	0.72993	0.73650	0.73869	0.74584	0.75361	0.76228
287	0.71665	0.73060	0.73717	0.73933	0.74647	0.75423	0.76287
288	0.71734	0.73128	0.73782	0.73999	0.74711	0.75484	0.76347
289	0.71808	0.73197	0.73848	0.74066	0.74775	0.75546	0.76406
290	0.71873	0.73263	0.73914	0.74131	0.74837	0.75607	0.76464
291	0.71945	0.73331	0.73979	0.74195	0.74900	0.75668	0.76523
292	0.72013	0.73396	0.74043	0.74260	0.74962	0.75728	0.76581
293	0.72083	0.73463	0.74107	0.74323	0.75024	0.75789	0.76639
294	0.72153	0.73527	0.74171	0.74387	0.75086	0.75848	0.76696
295	0.72216	0.73591	0.74236	0.74451	0.75147	0.75908	0.76753
296	0.72285	0.73657	0.74299	0.74512	0.75208	0.75966	0.76810
297	0.72352	0.73723	0.74363	0.74575	0.75270	0.76025	0.76866
298	0.72422	0.73784	0.74425	0.74636	0.75329	0.76084	0.76922
299	0.72487	0.73848	0.74487	0.74699	0.75389	0.76142	0.76978
300	0.72555	0.73913	0.74550	0.74760	0.75450	0.76199	0.77034
301	0.72625	0.73975	0.74610	0.74821	0.75510	0.76257	0.77089
302	0.72695	0.74039	0.74672	0.74882	0.75569	0.76313	0.77143
303	0.72763	0.74100	0.74731	0.74941	0.75627	0.76369	0.77198
304	0.72829	0.74161	0.74793	0.75001	0.75685	0.76426	0.77253

N	0.1%	0.5%	1%	1.25%	2.5%	5%	10%
305	0.72893	0.74223	0.74853	0.75062	0.75743	0.76482	0.77307
306	0.72956	0.74286	0.74912	0.75122	0.75801	0.76538	0.77361
307	0.73018	0.74347	0.74973	0.75181	0.75859	0.76594	0.77414
308	0.73079	0.74405	0.75031	0.75240	0.75916	0.76649	0.77467
309	0.73142	0.74465	0.75091	0.75297	0.75972	0.76704	0.77520
310	0.73203	0.74525	0.75150	0.75356	0.76029	0.76759	0.77573
311	0.73265	0.74584	0.75208	0.75413	0.76086	0.76813	0.77625
312	0.73327	0.74643	0.75266	0.75471	0.76141	0.76868	0.77677
313	0.73392	0.74702	0.75323	0.75528	0.76197	0.76921	0.77729
314	0.73455	0.74763	0.75381	0.75585	0.76253	0.76975	0.77781
315	0.73518	0.74821	0.75438	0.75641	0.76307	0.77028	0.77832
316	0.73577	0.74879	0.75494	0.75699	0.76362	0.77082	0.77883
317	0.73635	0.74937	0.75551	0.75755	0.76416	0.77134	0.77934
318	0.73696	0.74995	0.75608	0.75810	0.76471	0.77186	0.77985
319	0.73756	0.75052	0.75663	0.75866	0.76526	0.77239	0.78035
320	0.73818	0.75110	0.75719	0.75921	0.76579	0.77291	0.78085
321	0.73879	0.75168	0.75774	0.75975	0.76632	0.77343	0.78135
322	0.73937	0.75224	0.75828	0.76030	0.76685	0.77395	0.78185
323	0.73997	0.75280	0.75883	0.76085	0.76738	0.77446	0.78234
324	0.74058	0.75336	0.75937	0.76140	0.76791	0.77497	0.78283
325	0.74115	0.75391	0.75992	0.76194	0.76843	0.77547	0.78332
326	0.74173	0.75447	0.76045	0.76247	0.76894	0.77598	0.78380
327	0.74230	0.75501	0.76100	0.76300	0.76945	0.77648	0.78429
328	0.74288	0.75556	0.76153	0.76353	0.76998	0.77698	0.78476
329	0.74347	0.75612	0.76206	0.76406	0.77049	0.77748	0.78524
330	0.74405	0.75666	0.76259	0.76458	0.77100	0.77797	0.78571
331	0.74461	0.75719	0.76312	0.76511	0.77150	0.77846	0.78619
332	0.74516	0.75774	0.76365	0.76563	0.77201	0.77894	0.78666
333	0.74573	0.75827	0.76418	0.76616	0.77251	0.77943	0.78713
334	0.74629	0.75880	0.76469	0.76667	0.77302	0.77992	0.78760
335	0.74683	0.75934	0.76521	0.76717	0.77351	0.78040	0.78806
336	0.74738	0.75987	0.76573	0.76769	0.77402	0.78088	0.78852
337	0.74794	0.76042	0.76624	0.76819	0.77451	0.78136	0.78899
338	0.74847	0.76094	0.76675	0.76869	0.77501	0.78183	0.78945
339	0.74900	0.76146	0.76726	0.76919	0.77549	0.78231	0.78989
340	0.74954	0.76200	0.76776	0.76969	0.77597	0.78278	0.79035
341	0.75010	0.76249	0.76827	0.77020	0.77645	0.78325	0.79080
342	0.75064	0.76301	0.76877	0.77069	0.77694	0.78371	0.79125
343	0.75117	0.76350	0.76925	0.77118	0.77742	0.78418	0.79169
344	0.75173	0.76402	0.76974	0.77167	0.77790	0.78464	0.79214
345	0.75225	0.76454	0.77023	0.77216	0.77838	0.78510	0.79258
346	0.75281	0.76503	0.77072	0.77265	0.77885	0.78555	0.79302
347	0.75330	0.76553	0.77121	0.77314	0.77933	0.78601	0.79346
348	0.75387	0.76602	0.77170	0.77362	0.77981	0.78647	0.79389
349	0.75440	0.76654	0.77219	0.77410	0.78027	0.78692	0.79433
350	0.75488	0.76703	0.77267	0.77458	0.78073	0.78736	0.79476
351	0.75538	0.76754	0.77317	0.77506	0.78119	0.78781	0.79518
352	0.75592	0.76802	0.77364	0.77553	0.78166	0.78825	0.79561
353	0.75641	0.76851	0.77411	0.77601	0.78212	0.78869	0.79605
354	0.75695	0.76898	0.77459	0.77647	0.78257	0.78913	0.79647
355	0.75743	0.76945	0.77506	0.77694	0.78303	0.78957	0.79689
356	0.75796	0.76994	0.77553	0.77741	0.78347	0.79001	0.79731
357	0.75848	0.77042	0.77599	0.77788	0.78392	0.79044	0.79773
358	0.75897	0.77089	0.77647	0.77834	0.78436	0.79088	0.79815
359	0.75942	0.77136	0.77694	0.77879	0.78481	0.79131	0.79856
360	0.75992	0.77183	0.77740	0.77926	0.78526	0.79173	0.79897
361	0.76043	0.77230	0.77785	0.77971	0.78570	0.79217	0.79938
362	0.76094	0.77277	0.77831	0.78015	0.78614	0.79259	0.79979
363	0.76143	0.77324	0.77877	0.78060	0.78657	0.79301	0.80020
364	0.76190	0.77371	0.77921	0.78105	0.78701	0.79344	0.80061
365	0.76238	0.77418	0.77968	0.78149	0.78744	0.79385	0.80100
366	0.76289	0.77464	0.78012	0.78195	0.78787	0.79427	0.80141
367	0.76337	0.77508	0.78056	0.78239	0.78829	0.79469	0.80181
368	0.76382	0.77553	0.78101	0.78283	0.78871	0.79510	0.80220
369	0.76428	0.77598	0.78145	0.78326	0.78914	0.79551	0.80260
370	0.76480	0.77643	0.78188	0.78370	0.78956	0.79592	0.80299
371	0.76527	0.77687	0.78231	0.78413	0.78999	0.79633	0.80339
372	0.76570	0.77732	0.78275	0.78458	0.79041	0.79673	0.80378
373	0.76619	0.77776	0.78319	0.78500	0.79083	0.79714	0.80417
374	0.76666	0.77822	0.78363	0.78543	0.79124	0.79754	0.80456
375	0.76714	0.77866	0.78405	0.78585	0.79165	0.79794	0.80494
376	0.76756	0.77909	0.78449	0.78628	0.79206	0.79834	0.80533
377	0.76803	0.77953	0.78491	0.78670	0.79248	0.79874	0.80571
378	0.76848	0.77996	0.78533	0.78711	0.79288	0.79913	0.80610

N	0.1%	0.5%	1%	1.25%	2.5%	5%	10%
379	0.76893	0.78042	0.78575	0.78753	0.79329	0.79953	0.80648
380	0.76937	0.78084	0.78618	0.78794	0.79370	0.79992	0.80685
381	0.76982	0.78127	0.78660	0.78837	0.79411	0.80031	0.80723
382	0.77031	0.78170	0.78702	0.78878	0.79450	0.80070	0.80760
383	0.77078	0.78212	0.78743	0.78920	0.79490	0.80109	0.80797
384	0.77121	0.78253	0.78785	0.78961	0.79530	0.80148	0.80835
385	0.77167	0.78296	0.78826	0.79002	0.79570	0.80186	0.80871
386	0.77214	0.78340	0.78867	0.79042	0.79610	0.80225	0.80908
387	0.77259	0.78380	0.78907	0.79082	0.79649	0.80262	0.80945
388	0.77303	0.78421	0.78948	0.79122	0.79688	0.80300	0.80981
389	0.77346	0.78464	0.78989	0.79162	0.79726	0.80338	0.81018
390	0.77390	0.78505	0.79030	0.79202	0.79765	0.80376	0.81054
391	0.77430	0.78547	0.79069	0.79241	0.79803	0.80413	0.81090
392	0.77475	0.78588	0.79109	0.79282	0.79842	0.80450	0.81126
393	0.77518	0.78627	0.79148	0.79321	0.79880	0.80487	0.81162
394	0.77561	0.78669	0.79188	0.79361	0.79919	0.80524	0.81197
395	0.77604	0.78709	0.79228	0.79399	0.79957	0.80561	0.81233
396	0.77645	0.78749	0.79267	0.79439	0.79995	0.80598	0.81268
397	0.77684	0.78791	0.79306	0.79478	0.80032	0.80634	0.81303
398	0.77729	0.78831	0.79346	0.79516	0.80070	0.80670	0.81338
399	0.77769	0.78870	0.79385	0.79555	0.80107	0.80706	0.81373
400	0.77814	0.78910	0.79424	0.79593	0.80144	0.80742	0.81407
401	0.77856	0.78950	0.79461	0.79631	0.80182	0.80778	0.81442
402	0.77898	0.78990	0.79500	0.79670	0.80218	0.80814	0.81477
403	0.77939	0.79029	0.79539	0.79708	0.80255	0.80850	0.81510
404	0.77980	0.79070	0.79577	0.79746	0.80292	0.80885	0.81545
405	0.78019	0.79108	0.79614	0.79784	0.80328	0.80921	0.81579
406	0.78061	0.79147	0.79652	0.79821	0.80365	0.80956	0.81612
407	0.78103	0.79186	0.79690	0.79858	0.80401	0.80991	0.81646
408	0.78144	0.79225	0.79728	0.79895	0.80437	0.81026	0.81679
409	0.78183	0.79263	0.79765	0.79933	0.80473	0.81061	0.81713
410	0.78222	0.79300	0.79801	0.79969	0.80508	0.81095	0.81747
411	0.78262	0.79339	0.79838	0.80007	0.80544	0.81130	0.81779
412	0.78302	0.79377	0.79875	0.80043	0.80579	0.81164	0.81813
413	0.78343	0.79414	0.79912	0.80078	0.80615	0.81198	0.81846
414	0.78383	0.79453	0.79948	0.80115	0.80649	0.81232	0.81878
415	0.78424	0.79490	0.79985	0.80151	0.80684	0.81266	0.81911
416	0.78462	0.79528	0.80021	0.80187	0.80719	0.81300	0.81943
417	0.78502	0.79565	0.80057	0.80223	0.80754	0.81334	0.81976
418	0.78541	0.79601	0.80092	0.80258	0.80789	0.81367	0.82008
419	0.78580	0.79637	0.80129	0.80293	0.80824	0.81400	0.82040
420	0.78621	0.79673	0.80164	0.80328	0.80858	0.81434	0.82072
421	0.78657	0.79710	0.80199	0.80363	0.80892	0.81467	0.82104
422	0.78697	0.79745	0.80234	0.80398	0.80926	0.81499	0.82136
423	0.78734	0.79782	0.80270	0.80434	0.80960	0.81532	0.82167
424	0.78770	0.79817	0.80305	0.80468	0.80994	0.81565	0.82199
425	0.78808	0.79853	0.80340	0.80502	0.81027	0.81598	0.82230
426	0.78845	0.79890	0.80375	0.80537	0.81061	0.81630	0.82261
427	0.78885	0.79925	0.80409	0.80571	0.81094	0.81663	0.82292
428	0.78925	0.79961	0.80444	0.80605	0.81127	0.81695	0.82323
429	0.78960	0.79997	0.80479	0.80640	0.81161	0.81728	0.82354
430	0.78999	0.80032	0.80513	0.80674	0.81194	0.81759	0.82385
431	0.79036	0.80067	0.80547	0.80707	0.81226	0.81791	0.82415
432	0.79074	0.80102	0.80581	0.80741	0.81259	0.81822	0.82446
433	0.79111	0.80137	0.80614	0.80774	0.81292	0.81854	0.82476
434	0.79147	0.80171	0.80648	0.80808	0.81324	0.81885	0.82507
435	0.79182	0.80205	0.80682	0.80841	0.81357	0.81916	0.82537
436	0.79217	0.80239	0.80717	0.80875	0.81390	0.81947	0.82567
437	0.79255	0.80274	0.80750	0.80909	0.81421	0.81978	0.82597
438	0.79290	0.80309	0.80783	0.80941	0.81453	0.82009	0.82627
439	0.79325	0.80343	0.80816	0.80973	0.81485	0.82040	0.82657
440	0.79361	0.80376	0.80850	0.81006	0.81517	0.82071	0.82686
441	0.79400	0.80410	0.80882	0.81038	0.81549	0.82101	0.82716
442	0.79434	0.80443	0.80914	0.81071	0.81580	0.82133	0.82745
443	0.79468	0.80476	0.80948	0.81103	0.81611	0.82163	0.82774
444	0.79503	0.80508	0.80980	0.81135	0.81643	0.82193	0.82803
445	0.79541	0.80542	0.81012	0.81167	0.81674	0.82223	0.82832
446	0.79574	0.80575	0.81044	0.81199	0.81705	0.82253	0.82861
447	0.79607	0.80608	0.81077	0.81231	0.81736	0.82283	0.82890
448	0.79643	0.80642	0.81109	0.81263	0.81768	0.82313	0.82919
449	0.79680	0.80674	0.81140	0.81294	0.81798	0.82343	0.82947
450	0.79715	0.80706	0.81172	0.81326	0.81829	0.82372	0.82976
451	0.79750	0.80739	0.81204	0.81358	0.81860	0.82402	0.83004
452	0.79781	0.80772	0.81235	0.81389	0.81890	0.82431	0.83032

N	0.1%	0.5%	1%	1.25%	2.5%	5%	10%
453	0.79816	0.80803	0.81266	0.81419	0.81920	0.82460	0.83060
454	0.79851	0.80836	0.81297	0.81450	0.81950	0.82489	0.83088
455	0.79884	0.80868	0.81329	0.81481	0.81980	0.82519	0.83116
456	0.79918	0.80900	0.81359	0.81512	0.82010	0.82548	0.83144
457	0.79951	0.80931	0.81390	0.81543	0.82040	0.82577	0.83172
458	0.79986	0.80963	0.81421	0.81574	0.82069	0.82605	0.83200
459	0.80016	0.80994	0.81452	0.81604	0.82098	0.82634	0.83227
460	0.80050	0.81027	0.81483	0.81634	0.82128	0.82663	0.83254
461	0.80087	0.81058	0.81514	0.81664	0.82157	0.82691	0.83282
462	0.80122	0.81089	0.81544	0.81695	0.82187	0.82719	0.83309
463	0.80153	0.81120	0.81574	0.81725	0.82216	0.82747	0.83336
464	0.80186	0.81152	0.81605	0.81755	0.82245	0.82776	0.83363
465	0.80218	0.81181	0.81635	0.81785	0.82274	0.82803	0.83391
466	0.80252	0.81213	0.81665	0.81815	0.82303	0.82831	0.83418
467	0.80283	0.81244	0.81694	0.81845	0.82332	0.82859	0.83444
468	0.80313	0.81275	0.81723	0.81873	0.82361	0.82886	0.83471
469	0.80348	0.81305	0.81752	0.81903	0.82389	0.82914	0.83498
470	0.80380	0.81336	0.81783	0.81932	0.82417	0.82942	0.83524
471	0.80411	0.81365	0.81813	0.81961	0.82446	0.82970	0.83551
472	0.80442	0.81395	0.81843	0.81991	0.82474	0.82997	0.83577
473	0.80476	0.81425	0.81872	0.82021	0.82502	0.83024	0.83603
474	0.80505	0.81455	0.81901	0.82050	0.82530	0.83051	0.83630
475	0.80536	0.81486	0.81930	0.82078	0.82558	0.83078	0.83655
476	0.80567	0.81515	0.81959	0.82107	0.82585	0.83105	0.83682
477	0.80599	0.81544	0.81988	0.82136	0.82613	0.83132	0.83707
478	0.80629	0.81574	0.82017	0.82165	0.82641	0.83159	0.83733
479	0.80662	0.81603	0.82046	0.82193	0.82668	0.83186	0.83759
480	0.80692	0.81633	0.82074	0.82222	0.82696	0.83213	0.83784
481	0.80722	0.81662	0.82103	0.82249	0.82724	0.83239	0.83810
482	0.80756	0.81691	0.82131	0.82277	0.82751	0.83266	0.83836
483	0.80785	0.81720	0.82159	0.82305	0.82778	0.83292	0.83861
484	0.80816	0.81750	0.82186	0.82332	0.82805	0.83318	0.83886
485	0.80847	0.81778	0.82215	0.82360	0.82832	0.83344	0.83911
486	0.80877	0.81807	0.82243	0.82388	0.82858	0.83370	0.83936
487	0.80907	0.81835	0.82270	0.82416	0.82885	0.83396	0.83961
488	0.80938	0.81864	0.82298	0.82442	0.82912	0.83422	0.83986
489	0.80967	0.81892	0.82325	0.82471	0.82939	0.83447	0.84011
490	0.80997	0.81922	0.82352	0.82498	0.82966	0.83473	0.84036
491	0.81026	0.81949	0.82380	0.82525	0.82992	0.83499	0.84060
492	0.81056	0.81976	0.82408	0.82552	0.83019	0.83524	0.84085
493	0.81086	0.82005	0.82435	0.82580	0.83045	0.83550	0.84109
494	0.81113	0.82031	0.82463	0.82606	0.83071	0.83574	0.84133
495	0.81142	0.82059	0.82489	0.82633	0.83097	0.83600	0.84158
496	0.81173	0.82088	0.82517	0.82660	0.83123	0.83625	0.84182
497	0.81201	0.82115	0.82544	0.82687	0.83148	0.83650	0.84206
498	0.81231	0.82142	0.82570	0.82713	0.83174	0.83675	0.84230
499	0.81260	0.82170	0.82596	0.82740	0.83200	0.83700	0.84254
500	0.81292	0.82197	0.82623	0.82766	0.83226	0.83725	0.84278
501	0.81320	0.82224	0.82650	0.82792	0.83252	0.83750	0.84302
502	0.81347	0.82252	0.82677	0.82819	0.83278	0.83774	0.84326
503	0.81375	0.82279	0.82703	0.82845	0.83303	0.83799	0.84350
504	0.81404	0.82306	0.82730	0.82871	0.83328	0.83823	0.84373
505	0.81433	0.82333	0.82756	0.82897	0.83354	0.83848	0.84397
506	0.81462	0.82361	0.82782	0.82923	0.83379	0.83872	0.84420
507	0.81491	0.82386	0.82808	0.82949	0.83404	0.83897	0.84443
508	0.81519	0.82413	0.82834	0.82974	0.83429	0.83921	0.84467
509	0.81543	0.82439	0.82860	0.83000	0.83454	0.83945	0.84489
510	0.81573	0.82466	0.82886	0.83026	0.83478	0.83968	0.84513
511	0.81598	0.82492	0.82912	0.83051	0.83503	0.83992	0.84536
512	0.81627	0.82517	0.82938	0.83076	0.83528	0.84016	0.84559
513	0.81656	0.82544	0.82963	0.83102	0.83552	0.84040	0.84582
514	0.81684	0.82570	0.82989	0.83127	0.83577	0.84064	0.84605
515	0.81712	0.82596	0.83014	0.83152	0.83602	0.84087	0.84627
516	0.81737	0.82624	0.83040	0.83177	0.83625	0.84111	0.84650
517	0.81766	0.82649	0.83065	0.83202	0.83649	0.84135	0.84672
518	0.81791	0.82675	0.83091	0.83227	0.83674	0.84158	0.84695
519	0.81818	0.82700	0.83115	0.83252	0.83698	0.84181	0.84717
520	0.81846	0.82726	0.83139	0.83277	0.83721	0.84204	0.84740
521	0.81875	0.82752	0.83164	0.83302	0.83745	0.84227	0.84762
522	0.81901	0.82777	0.83188	0.83326	0.83770	0.84250	0.84784
523	0.81929	0.82802	0.83213	0.83351	0.83794	0.84274	0.84806
524	0.81956	0.82828	0.83238	0.83376	0.83817	0.84296	0.84828
525	0.81983	0.82854	0.83263	0.83399	0.83841	0.84319	0.84850
526	0.82007	0.82879	0.83287	0.83423	0.83864	0.84342	0.84872

N	0.1%	0.5%	1%	1.25%	2.5%	5%	10%
527	0.82033	0.82904	0.83312	0.83447	0.83888	0.84364	0.84894
528	0.82062	0.82929	0.83336	0.83472	0.83911	0.84387	0.84916
529	0.82087	0.82954	0.83360	0.83496	0.83934	0.84410	0.84938
530	0.82115	0.82979	0.83385	0.83520	0.83958	0.84432	0.84959
531	0.82143	0.83003	0.83408	0.83544	0.83981	0.84455	0.84981
532	0.82169	0.83027	0.83433	0.83567	0.84004	0.84477	0.85002
533	0.82196	0.83052	0.83457	0.83592	0.84026	0.84499	0.85024
534	0.82221	0.83077	0.83480	0.83615	0.84050	0.84521	0.85045
535	0.82247	0.83102	0.83505	0.83638	0.84072	0.84543	0.85066
536	0.82271	0.83127	0.83528	0.83662	0.84095	0.84566	0.85088
537	0.82296	0.83151	0.83552	0.83685	0.84119	0.84588	0.85109
538	0.82320	0.83174	0.83575	0.83708	0.84141	0.84610	0.85130
539	0.82346	0.83198	0.83600	0.83731	0.84164	0.84631	0.85151
540	0.82372	0.83221	0.83623	0.83755	0.84186	0.84654	0.85172
541	0.82399	0.83246	0.83646	0.83778	0.84209	0.84675	0.85193
542	0.82423	0.83270	0.83669	0.83800	0.84231	0.84697	0.85214
543	0.82449	0.83294	0.83692	0.83823	0.84253	0.84719	0.85235
544	0.82471	0.83318	0.83714	0.83846	0.84276	0.84740	0.85255
545	0.82497	0.83341	0.83738	0.83869	0.84298	0.84761	0.85276
546	0.82522	0.83365	0.83761	0.83892	0.84321	0.84783	0.85297
547	0.82548	0.83389	0.83783	0.83914	0.84343	0.84804	0.85317
548	0.82571	0.83413	0.83805	0.83937	0.84365	0.84826	0.85338
549	0.82596	0.83436	0.83828	0.83959	0.84386	0.84847	0.85358
550	0.82621	0.83460	0.83850	0.83982	0.84409	0.84868	0.85378
551	0.82645	0.83483	0.83873	0.84005	0.84431	0.84889	0.85399
552	0.82668	0.83506	0.83896	0.84027	0.84452	0.84910	0.85419
553	0.82695	0.83531	0.83918	0.84049	0.84474	0.84931	0.85439
554	0.82721	0.83554	0.83941	0.84071	0.84495	0.84952	0.85459
555	0.82746	0.83577	0.83964	0.84093	0.84517	0.84973	0.85479
556	0.82769	0.83600	0.83986	0.84115	0.84539	0.84994	0.85499
557	0.82793	0.83623	0.84008	0.84137	0.84560	0.85015	0.85519
558	0.82816	0.83646	0.84031	0.84159	0.84581	0.85035	0.85539
559	0.82840	0.83669	0.84053	0.84181	0.84602	0.85056	0.85559
560	0.82863	0.83690	0.84075	0.84204	0.84623	0.85076	0.85579
561	0.82887	0.83713	0.84096	0.84225	0.84645	0.85097	0.85599
562	0.82911	0.83735	0.84118	0.84247	0.84665	0.85117	0.85618
563	0.82936	0.83758	0.84140	0.84269	0.84686	0.85137	0.85638
564	0.82960	0.83781	0.84162	0.84291	0.84707	0.85158	0.85657
565	0.82981	0.83803	0.84184	0.84312	0.84728	0.85178	0.85677
566	0.83006	0.83825	0.84206	0.84334	0.84749	0.85199	0.85696
567	0.83029	0.83847	0.84228	0.84355	0.84770	0.85218	0.85715
568	0.83052	0.83869	0.84249	0.84377	0.84790	0.85238	0.85735
569	0.83073	0.83891	0.84270	0.84399	0.84811	0.85258	0.85754
570	0.83097	0.83913	0.84291	0.84419	0.84831	0.85278	0.85773
571	0.83120	0.83935	0.84313	0.84440	0.84852	0.85298	0.85792
572	0.83146	0.83957	0.84334	0.84461	0.84872	0.85318	0.85811
573	0.83167	0.83979	0.84355	0.84482	0.84893	0.85337	0.85830
574	0.83191	0.84000	0.84376	0.84503	0.84913	0.85357	0.85849
575	0.83212	0.84021	0.84397	0.84524	0.84933	0.85376	0.85868
576	0.83235	0.84043	0.84418	0.84545	0.84953	0.85396	0.85887
577	0.83258	0.84064	0.84439	0.84566	0.84973	0.85415	0.85906
578	0.83280	0.84085	0.84460	0.84586	0.84993	0.85435	0.85924
579	0.83302	0.84107	0.84480	0.84606	0.85013	0.85454	0.85943
580	0.83324	0.84128	0.84502	0.84627	0.85033	0.85474	0.85962
581	0.83346	0.84149	0.84522	0.84648	0.85053	0.85493	0.85980
582	0.83368	0.84171	0.84543	0.84668	0.85073	0.85512	0.85998
583	0.83389	0.84192	0.84563	0.84688	0.85093	0.85531	0.86017
584	0.83412	0.84213	0.84584	0.84709	0.85113	0.85550	0.86035
585	0.83435	0.84234	0.84605	0.84729	0.85133	0.85569	0.86053
586	0.83457	0.84254	0.84626	0.84749	0.85152	0.85588	0.86072
587	0.83478	0.84274	0.84646	0.84770	0.85172	0.85607	0.86090
588	0.83500	0.84295	0.84666	0.84790	0.85192	0.85626	0.86108
589	0.83520	0.84316	0.84685	0.84810	0.85211	0.85645	0.86126
590	0.83543	0.84337	0.84706	0.84829	0.85231	0.85663	0.86144
591	0.83563	0.84357	0.84726	0.84850	0.85250	0.85682	0.86162
592	0.83585	0.84378	0.84746	0.84869	0.85270	0.85701	0.86180
593	0.83606	0.84398	0.84766	0.84889	0.85289	0.85719	0.86198
594	0.83627	0.84419	0.84786	0.84909	0.85308	0.85738	0.86216
595	0.83650	0.84440	0.84806	0.84929	0.85327	0.85756	0.86233
596	0.83671	0.84460	0.84826	0.84949	0.85346	0.85775	0.86251
597	0.83693	0.84480	0.84846	0.84968	0.85365	0.85793	0.86269
598	0.83715	0.84501	0.84866	0.84988	0.85384	0.85812	0.86286
599	0.83738	0.84520	0.84886	0.85007	0.85402	0.85830	0.86304
600	0.83758	0.84540	0.84905	0.85027	0.85421	0.85848	0.86322

N	0.1%	0.5%	1%	1.25%	2.5%	5%	10%
601	0.83778	0.84560	0.84924	0.85047	0.85440	0.85866	0.86339
602	0.83799	0.84580	0.84944	0.85066	0.85459	0.85884	0.86357
603	0.83819	0.84600	0.84963	0.85085	0.85478	0.85902	0.86374
604	0.83840	0.84620	0.84983	0.85104	0.85496	0.85921	0.86391
605	0.83860	0.84640	0.85002	0.85123	0.85515	0.85939	0.86408
606	0.83881	0.84660	0.85022	0.85141	0.85533	0.85957	0.86426
607	0.83902	0.84680	0.85041	0.85161	0.85552	0.85975	0.86443
608	0.83922	0.84699	0.85059	0.85180	0.85570	0.85992	0.86460
609	0.83944	0.84719	0.85079	0.85199	0.85588	0.86010	0.86477
610	0.83965	0.84739	0.85098	0.85218	0.85606	0.86028	0.86495
611	0.83984	0.84758	0.85116	0.85236	0.85625	0.86045	0.86512
612	0.84006	0.84778	0.85136	0.85256	0.85643	0.86063	0.86529
613	0.84026	0.84798	0.85154	0.85274	0.85661	0.86081	0.86546
614	0.84045	0.84817	0.85173	0.85293	0.85679	0.86098	0.86562
615	0.84067	0.84837	0.85192	0.85311	0.85698	0.86116	0.86579
616	0.84086	0.84855	0.85211	0.85329	0.85716	0.86133	0.86596
617	0.84107	0.84874	0.85229	0.85348	0.85734	0.86151	0.86613
618	0.84126	0.84894	0.85247	0.85366	0.85752	0.86168	0.86630
619	0.84146	0.84912	0.85266	0.85385	0.85770	0.86185	0.86646
620	0.84166	0.84931	0.85284	0.85403	0.85788	0.86203	0.86663
621	0.84187	0.84950	0.85303	0.85422	0.85805	0.86220	0.86680
622	0.84207	0.84969	0.85321	0.85440	0.85823	0.86237	0.86696
623	0.84227	0.84988	0.85340	0.85458	0.85841	0.86254	0.86713
624	0.84247	0.85006	0.85358	0.85476	0.85858	0.86271	0.86729
625	0.84266	0.85025	0.85376	0.85495	0.85876	0.86288	0.86745
626	0.84285	0.85043	0.85395	0.85513	0.85893	0.86305	0.86762
627	0.84306	0.85062	0.85413	0.85531	0.85910	0.86322	0.86778
628	0.84325	0.85081	0.85431	0.85548	0.85928	0.86339	0.86794
629	0.84345	0.85099	0.85449	0.85567	0.85945	0.86356	0.86810
630	0.84366	0.85118	0.85468	0.85585	0.85962	0.86373	0.86826
631	0.84383	0.85136	0.85485	0.85602	0.85979	0.86389	0.86842
632	0.84402	0.85154	0.85503	0.85620	0.85997	0.86406	0.86859
633	0.84423	0.85173	0.85520	0.85637	0.86014	0.86423	0.86875
634	0.84444	0.85191	0.85539	0.85655	0.86031	0.86439	0.86890
635	0.84463	0.85209	0.85557	0.85673	0.86048	0.86456	0.86907
636	0.84482	0.85228	0.85574	0.85690	0.86065	0.86472	0.86922
637	0.84500	0.85246	0.85592	0.85707	0.86082	0.86489	0.86938
638	0.84521	0.85264	0.85610	0.85725	0.86099	0.86505	0.86954
639	0.84540	0.85282	0.85627	0.85743	0.86116	0.86522	0.86970
640	0.84558	0.85300	0.85645	0.85760	0.86133	0.86538	0.86986
641	0.84577	0.85318	0.85662	0.85777	0.86150	0.86554	0.87001
642	0.84594	0.85335	0.85680	0.85794	0.86167	0.86570	0.87017
643	0.84614	0.85353	0.85696	0.85811	0.86184	0.86587	0.87033
644	0.84633	0.85371	0.85713	0.85829	0.86200	0.86603	0.87048
645	0.84651	0.85389	0.85731	0.85846	0.86217	0.86619	0.87063
646	0.84668	0.85407	0.85748	0.85862	0.86234	0.86635	0.87079
647	0.84686	0.85424	0.85765	0.85880	0.86250	0.86651	0.87094
648	0.84703	0.85441	0.85782	0.85896	0.86267	0.86667	0.87110
649	0.84723	0.85459	0.85800	0.85914	0.86284	0.86683	0.87125
650	0.84743	0.85477	0.85816	0.85931	0.86300	0.86699	0.87141
651	0.84760	0.85494	0.85833	0.85948	0.86316	0.86715	0.87156
652	0.84779	0.85511	0.85850	0.85964	0.86333	0.86730	0.87171
653	0.84797	0.85530	0.85867	0.85982	0.86349	0.86746	0.87186
654	0.84816	0.85546	0.85884	0.85998	0.86365	0.86762	0.87202
655	0.84834	0.85564	0.85901	0.86014	0.86381	0.86778	0.87217
656	0.84852	0.85581	0.85918	0.86031	0.86397	0.86793	0.87232
657	0.84868	0.85598	0.85935	0.86048	0.86413	0.86809	0.87247
658	0.84888	0.85615	0.85951	0.86065	0.86430	0.86824	0.87262
659	0.84904	0.85632	0.85968	0.86081	0.86445	0.86840	0.87277
660	0.84922	0.85649	0.85985	0.86097	0.86462	0.86856	0.87292
661	0.84941	0.85666	0.86001	0.86113	0.86478	0.86871	0.87306
662	0.84959	0.85683	0.86018	0.86130	0.86494	0.86887	0.87321
663	0.84977	0.85700	0.86034	0.86146	0.86510	0.86902	0.87336
664	0.84994	0.85717	0.86051	0.86163	0.86525	0.86917	0.87351
665	0.85013	0.85734	0.86068	0.86179	0.86541	0.86932	0.87366
666	0.85031	0.85751	0.86084	0.86196	0.86557	0.86948	0.87380
667	0.85050	0.85767	0.86100	0.86212	0.86573	0.86963	0.87395
668	0.85068	0.85784	0.86117	0.86228	0.86588	0.86978	0.87409
669	0.85086	0.85800	0.86133	0.86244	0.86604	0.86993	0.87424
670	0.85104	0.85817	0.86149	0.86260	0.86619	0.87008	0.87439
671	0.85121	0.85833	0.86165	0.86276	0.86635	0.87023	0.87453
672	0.85137	0.85850	0.86182	0.86292	0.86651	0.87038	0.87468
673	0.85155	0.85866	0.86198	0.86308	0.86666	0.87053	0.87482
674	0.85172	0.85883	0.86214	0.86324	0.86681	0.87068	0.87496

N	0.1%	0.5%	1%	1.25%	2.5%	5%	10%
675	0.85189	0.85899	0.86230	0.86340	0.86697	0.87083	0.87511
676	0.85205	0.85916	0.86246	0.86355	0.86712	0.87098	0.87525
677	0.85223	0.85933	0.86262	0.86371	0.86727	0.87113	0.87539
678	0.85241	0.85949	0.86278	0.86388	0.86743	0.87128	0.87553
679	0.85259	0.85965	0.86294	0.86403	0.86758	0.87142	0.87568
680	0.85276	0.85981	0.86309	0.86419	0.86773	0.87157	0.87582
681	0.85291	0.85997	0.86325	0.86435	0.86788	0.87172	0.87596
682	0.85308	0.86014	0.86340	0.86450	0.86803	0.87187	0.87610
683	0.85325	0.86030	0.86356	0.86466	0.86818	0.87201	0.87624
684	0.85342	0.86046	0.86372	0.86481	0.86833	0.87216	0.87638
685	0.85362	0.86062	0.86387	0.86497	0.86849	0.87231	0.87652
686	0.85376	0.86078	0.86403	0.86512	0.86863	0.87245	0.87666
687	0.85393	0.86093	0.86418	0.86528	0.86878	0.87259	0.87680
688	0.85409	0.86110	0.86434	0.86543	0.86893	0.87274	0.87694
689	0.85426	0.86126	0.86449	0.86558	0.86908	0.87288	0.87708
690	0.85442	0.86142	0.86465	0.86573	0.86923	0.87302	0.87722
691	0.85460	0.86158	0.86480	0.86588	0.86937	0.87317	0.87736
692	0.85476	0.86173	0.86495	0.86604	0.86952	0.87331	0.87749
693	0.85494	0.86189	0.86511	0.86619	0.86967	0.87345	0.87763
694	0.85511	0.86205	0.86526	0.86634	0.86982	0.87360	0.87777
695	0.85527	0.86221	0.86541	0.86649	0.86996	0.87374	0.87791
696	0.85544	0.86236	0.86557	0.86664	0.87011	0.87388	0.87804
697	0.85561	0.86251	0.86572	0.86678	0.87026	0.87402	0.87818
698	0.85577	0.86267	0.86586	0.86693	0.87040	0.87416	0.87831
699	0.85594	0.86283	0.86602	0.86708	0.87055	0.87430	0.87845
700	0.85609	0.86299	0.86617	0.86723	0.87069	0.87444	0.87858
701	0.85625	0.86313	0.86632	0.86739	0.87083	0.87458	0.87871
702	0.85641	0.86328	0.86647	0.86753	0.87098	0.87472	0.87885
703	0.85658	0.86344	0.86662	0.86768	0.87112	0.87485	0.87898
704	0.85675	0.86359	0.86677	0.86782	0.87126	0.87500	0.87912
705	0.85690	0.86375	0.86692	0.86797	0.87141	0.87514	0.87925
706	0.85706	0.86390	0.86707	0.86812	0.87155	0.87527	0.87938
707	0.85723	0.86405	0.86722	0.86827	0.87169	0.87541	0.87952
708	0.85739	0.86420	0.86736	0.86841	0.87183	0.87555	0.87965
709	0.85756	0.86435	0.86751	0.86856	0.87198	0.87568	0.87978
710	0.85772	0.86450	0.86765	0.86870	0.87212	0.87582	0.87991
711	0.85787	0.86465	0.86780	0.86885	0.87226	0.87596	0.88004
712	0.85803	0.86480	0.86794	0.86899	0.87240	0.87610	0.88017
713	0.85819	0.86495	0.86809	0.86914	0.87254	0.87623	0.88030
714	0.85834	0.86509	0.86823	0.86928	0.87268	0.87637	0.88043
715	0.85850	0.86524	0.86838	0.86942	0.87282	0.87650	0.88056
716	0.85865	0.86538	0.86852	0.86957	0.87296	0.87663	0.88070
717	0.85883	0.86553	0.86867	0.86971	0.87310	0.87677	0.88083
718	0.85899	0.86569	0.86881	0.86986	0.87324	0.87690	0.88095
719	0.85915	0.86584	0.86896	0.87000	0.87338	0.87704	0.88108
720	0.85930	0.86598	0.86910	0.87014	0.87352	0.87717	0.88121
721	0.85945	0.86613	0.86924	0.87028	0.87365	0.87730	0.88134
722	0.85959	0.86628	0.86938	0.87043	0.87379	0.87743	0.88147
723	0.85973	0.86643	0.86952	0.87056	0.87392	0.87757	0.88159
724	0.85989	0.86657	0.86967	0.87070	0.87406	0.87770	0.88172
725	0.86005	0.86672	0.86981	0.87085	0.87419	0.87783	0.88185
726	0.86020	0.86686	0.86995	0.87099	0.87433	0.87796	0.88197
727	0.86035	0.86701	0.87009	0.87112	0.87447	0.87809	0.88210
728	0.86052	0.86716	0.87024	0.87127	0.87460	0.87822	0.88223
729	0.86066	0.86730	0.87038	0.87141	0.87474	0.87835	0.88235
730	0.86079	0.86744	0.87052	0.87155	0.87487	0.87848	0.88248
731	0.86096	0.86758	0.87066	0.87168	0.87501	0.87861	0.88260
732	0.86112	0.86772	0.87080	0.87182	0.87514	0.87874	0.88273
733	0.86126	0.86787	0.87094	0.87196	0.87528	0.87887	0.88285
734	0.86142	0.86801	0.87107	0.87210	0.87541	0.87900	0.88298
735	0.86156	0.86815	0.87121	0.87223	0.87554	0.87913	0.88310
736	0.86172	0.86829	0.87135	0.87237	0.87568	0.87926	0.88322
737	0.86187	0.86843	0.87149	0.87250	0.87581	0.87939	0.88335
738	0.86202	0.86857	0.87162	0.87264	0.87595	0.87952	0.88347
739	0.86218	0.86871	0.87176	0.87277	0.87607	0.87964	0.88360
740	0.86233	0.86885	0.87190	0.87291	0.87621	0.87977	0.88372
741	0.86249	0.86899	0.87203	0.87305	0.87634	0.87990	0.88384
742	0.86263	0.86913	0.87217	0.87318	0.87647	0.88002	0.88396
743	0.86277	0.86927	0.87231	0.87332	0.87660	0.88015	0.88408
744	0.86291	0.86941	0.87244	0.87345	0.87673	0.88028	0.88421
745	0.86306	0.86955	0.87258	0.87358	0.87686	0.88040	0.88433
746	0.86320	0.86969	0.87271	0.87372	0.87699	0.88052	0.88445
747	0.86335	0.86983	0.87285	0.87385	0.87712	0.88065	0.88457
748	0.86349	0.86998	0.87298	0.87398	0.87725	0.88078	0.88469

N	0.1%	0.5%	1%	1.25%	2.5%	5%	10%
749	0.86365	0.87011	0.87312	0.87412	0.87738	0.88090	0.88481
750	0.86379	0.87025	0.87325	0.87426	0.87751	0.88103	0.88493
751	0.86393	0.87039	0.87338	0.87439	0.87764	0.88115	0.88505
752	0.86408	0.87053	0.87352	0.87452	0.87777	0.88127	0.88517
753	0.86423	0.87066	0.87365	0.87465	0.87789	0.88140	0.88529
754	0.86437	0.87080	0.87378	0.87478	0.87802	0.88152	0.88540
755	0.86451	0.87094	0.87392	0.87491	0.87815	0.88164	0.88552
756	0.86465	0.87108	0.87405	0.87504	0.87827	0.88176	0.88564
757	0.86480	0.87121	0.87418	0.87517	0.87840	0.88189	0.88576
758	0.86493	0.87134	0.87431	0.87530	0.87853	0.88201	0.88588
759	0.86506	0.87147	0.87444	0.87544	0.87865	0.88213	0.88599
760	0.86521	0.87161	0.87457	0.87557	0.87878	0.88225	0.88611
761	0.86534	0.87174	0.87470	0.87569	0.87891	0.88238	0.88623
762	0.86549	0.87187	0.87483	0.87582	0.87903	0.88250	0.88635
763	0.86563	0.87200	0.87496	0.87595	0.87916	0.88262	0.88646
764	0.86576	0.87214	0.87509	0.87608	0.87928	0.88274	0.88658
765	0.86589	0.87228	0.87522	0.87621	0.87940	0.88286	0.88669
766	0.86603	0.87240	0.87534	0.87633	0.87953	0.88298	0.88681
767	0.86618	0.87254	0.87547	0.87646	0.87965	0.88310	0.88692
768	0.86633	0.87267	0.87560	0.87659	0.87977	0.88322	0.88704
769	0.86647	0.87281	0.87573	0.87671	0.87990	0.88334	0.88715
770	0.86661	0.87294	0.87586	0.87684	0.88002	0.88346	0.88727
771	0.86675	0.87306	0.87599	0.87697	0.88014	0.88358	0.88738
772	0.86688	0.87318	0.87611	0.87710	0.88027	0.88369	0.88750
773	0.86702	0.87332	0.87624	0.87722	0.88038	0.88381	0.88761
774	0.86715	0.87345	0.87636	0.87735	0.88051	0.88393	0.88772
775	0.86730	0.87358	0.87649	0.87747	0.88063	0.88404	0.88784
776	0.86743	0.87370	0.87662	0.87759	0.88075	0.88416	0.88795
777	0.86757	0.87383	0.87674	0.87772	0.88087	0.88428	0.88806
778	0.86771	0.87396	0.87687	0.87784	0.88099	0.88439	0.88817
779	0.86785	0.87409	0.87699	0.87797	0.88111	0.88451	0.88829
780	0.86797	0.87422	0.87712	0.87809	0.88123	0.88463	0.88840
781	0.86810	0.87435	0.87724	0.87821	0.88135	0.88474	0.88851
782	0.86825	0.87447	0.87736	0.87834	0.88147	0.88486	0.88862
783	0.86839	0.87460	0.87749	0.87846	0.88159	0.88497	0.88873
784	0.86852	0.87472	0.87761	0.87858	0.88171	0.88509	0.88884
785	0.86866	0.87485	0.87774	0.87870	0.88183	0.88520	0.88895
786	0.86877	0.87498	0.87786	0.87883	0.88195	0.88531	0.88907
787	0.86892	0.87511	0.87799	0.87895	0.88206	0.88543	0.88918
788	0.86904	0.87524	0.87811	0.87907	0.88218	0.88555	0.88928
789	0.86917	0.87537	0.87823	0.87919	0.88230	0.88566	0.88939
790	0.86931	0.87550	0.87835	0.87931	0.88242	0.88578	0.88950
791	0.86943	0.87562	0.87848	0.87943	0.88254	0.88589	0.88961
792	0.86956	0.87575	0.87860	0.87955	0.88265	0.88600	0.88972
793	0.86968	0.87587	0.87872	0.87967	0.88277	0.88611	0.88983
794	0.86981	0.87599	0.87884	0.87979	0.88288	0.88623	0.88994
795	0.86996	0.87612	0.87896	0.87991	0.88300	0.88634	0.89005
796	0.87009	0.87623	0.87908	0.88003	0.88312	0.88645	0.89016
797	0.87023	0.87635	0.87920	0.88015	0.88324	0.88656	0.89026
798	0.87036	0.87648	0.87932	0.88027	0.88335	0.88668	0.89037
799	0.87050	0.87660	0.87944	0.88038	0.88347	0.88679	0.89048
800	0.87061	0.87672	0.87956	0.88050	0.88358	0.88690	0.89059
801	0.87074	0.87685	0.87968	0.88062	0.88370	0.88701	0.89069
802	0.87086	0.87697	0.87979	0.88074	0.88381	0.88712	0.89080
803	0.87099	0.87709	0.87991	0.88085	0.88392	0.88723	0.89091
804	0.87112	0.87721	0.88003	0.88097	0.88404	0.88734	0.89101
805	0.87125	0.87733	0.88015	0.88108	0.88415	0.88745	0.89112
806	0.87138	0.87746	0.88027	0.88120	0.88427	0.88756	0.89123
807	0.87151	0.87757	0.88038	0.88132	0.88438	0.88767	0.89133
808	0.87164	0.87768	0.88050	0.88143	0.88449	0.88778	0.89144
809	0.87177	0.87781	0.88061	0.88155	0.88460	0.88789	0.89154
810	0.87189	0.87793	0.88073	0.88167	0.88471	0.88800	0.89165
811	0.87202	0.87805	0.88085	0.88179	0.88482	0.88811	0.89175
812	0.87215	0.87817	0.88097	0.88191	0.88494	0.88822	0.89186
813	0.87227	0.87828	0.88108	0.88202	0.88505	0.88833	0.89196
814	0.87240	0.87840	0.88119	0.88213	0.88516	0.88844	0.89206
815	0.87252	0.87852	0.88131	0.88225	0.88528	0.88854	0.89217
816	0.87266	0.87864	0.88143	0.88237	0.88539	0.88865	0.89227
817	0.87279	0.87876	0.88155	0.88248	0.88550	0.88876	0.89238
818	0.87290	0.87888	0.88166	0.88259	0.88561	0.88886	0.89248
819	0.87303	0.87900	0.88178	0.88271	0.88572	0.88897	0.89258
820	0.87316	0.87912	0.88189	0.88282	0.88583	0.88908	0.89268
821	0.87328	0.87923	0.88201	0.88293	0.88593	0.88918	0.89279
822	0.87339	0.87935	0.88212	0.88304	0.88605	0.88929	0.89289

N	0.1%	0.5%	1%	1.25%	2.5%	5%	10%
823	0.87353	0.87946	0.88223	0.88316	0.88615	0.88939	0.89299
824	0.87365	0.87958	0.88235	0.88327	0.88626	0.88950	0.89309
825	0.87377	0.87969	0.88246	0.88338	0.88637	0.88961	0.89319
826	0.87390	0.87981	0.88257	0.88349	0.88648	0.88972	0.89330
827	0.87402	0.87993	0.88268	0.88360	0.88659	0.88982	0.89340
828	0.87416	0.88004	0.88279	0.88371	0.88670	0.88993	0.89350
829	0.87427	0.88016	0.88290	0.88383	0.88681	0.89003	0.89360
830	0.87438	0.88027	0.88301	0.88393	0.88691	0.89013	0.89370
831	0.87452	0.88038	0.88313	0.88404	0.88702	0.89024	0.89380
832	0.87464	0.88050	0.88324	0.88416	0.88713	0.89034	0.89390
833	0.87476	0.88061	0.88335	0.88427	0.88724	0.89044	0.89400
834	0.87487	0.88073	0.88346	0.88438	0.88734	0.89055	0.89410
835	0.87499	0.88085	0.88357	0.88449	0.88745	0.89065	0.89420
836	0.87510	0.88096	0.88369	0.88460	0.88756	0.89075	0.89430
837	0.87522	0.88107	0.88380	0.88471	0.88766	0.89086	0.89440
838	0.87534	0.88118	0.88391	0.88481	0.88777	0.89096	0.89450
839	0.87547	0.88130	0.88402	0.88492	0.88788	0.89106	0.89460
840	0.87559	0.88141	0.88413	0.88503	0.88798	0.89116	0.89470
841	0.87571	0.88153	0.88424	0.88514	0.88809	0.89127	0.89479
842	0.87582	0.88165	0.88435	0.88525	0.88819	0.89137	0.89489
843	0.87594	0.88176	0.88445	0.88536	0.88830	0.89147	0.89499
844	0.87606	0.88188	0.88456	0.88547	0.88840	0.89157	0.89509
845	0.87618	0.88198	0.88467	0.88557	0.88851	0.89167	0.89519
846	0.87629	0.88210	0.88478	0.88568	0.88861	0.89178	0.89528
847	0.87641	0.88221	0.88489	0.88578	0.88872	0.89187	0.89538
848	0.87653	0.88232	0.88500	0.88590	0.88882	0.89198	0.89548
849	0.87664	0.88243	0.88511	0.88600	0.88892	0.89208	0.89557
850	0.87676	0.88253	0.88521	0.88611	0.88902	0.89218	0.89567
851	0.87687	0.88263	0.88532	0.88621	0.88913	0.89228	0.89577
852	0.87699	0.88275	0.88543	0.88632	0.88923	0.89238	0.89586
853	0.87711	0.88286	0.88553	0.88643	0.88933	0.89248	0.89596
854	0.87721	0.88297	0.88564	0.88653	0.88944	0.89257	0.89606
855	0.87733	0.88308	0.88575	0.88664	0.88954	0.89267	0.89615
856	0.87745	0.88319	0.88585	0.88674	0.88964	0.89277	0.89625
857	0.87756	0.88329	0.88596	0.88685	0.88974	0.89287	0.89634
858	0.87768	0.88340	0.88606	0.88695	0.88985	0.89297	0.89644
859	0.87779	0.88351	0.88617	0.88706	0.88995	0.89307	0.89653
860	0.87791	0.88362	0.88628	0.88717	0.89005	0.89317	0.89663
861	0.87802	0.88373	0.88638	0.88727	0.89016	0.89327	0.89672
862	0.87813	0.88384	0.88649	0.88737	0.89025	0.89336	0.89682
863	0.87824	0.88394	0.88659	0.88747	0.89035	0.89346	0.89691
864	0.87835	0.88404	0.88669	0.88758	0.89046	0.89356	0.89700
865	0.87845	0.88415	0.88680	0.88768	0.89056	0.89366	0.89710
866	0.87857	0.88426	0.88690	0.88779	0.89066	0.89376	0.89719
867	0.87868	0.88436	0.88700	0.88789	0.89076	0.89385	0.89728
868	0.87879	0.88447	0.88711	0.88799	0.89085	0.89395	0.89738
869	0.87889	0.88458	0.88721	0.88809	0.89095	0.89405	0.89747
870	0.87901	0.88469	0.88731	0.88820	0.89105	0.89414	0.89756
871	0.87911	0.88479	0.88742	0.88830	0.89115	0.89424	0.89766
872	0.87923	0.88490	0.88751	0.88840	0.89125	0.89434	0.89775
873	0.87933	0.88500	0.88762	0.88849	0.89135	0.89443	0.89784
874	0.87944	0.88511	0.88772	0.88860	0.89145	0.89453	0.89793
875	0.87954	0.88521	0.88782	0.88870	0.89154	0.89462	0.89802
876	0.87965	0.88532	0.88793	0.88880	0.89164	0.89472	0.89812
877	0.87976	0.88542	0.88803	0.88890	0.89174	0.89481	0.89821
878	0.87988	0.88553	0.88814	0.88900	0.89184	0.89490	0.89830
879	0.87999	0.88563	0.88824	0.88910	0.89193	0.89500	0.89839
880	0.88010	0.88573	0.88834	0.88920	0.89203	0.89509	0.89848
881	0.88020	0.88583	0.88844	0.88930	0.89213	0.89519	0.89857
882	0.88032	0.88594	0.88853	0.88940	0.89223	0.89528	0.89866
883	0.88042	0.88604	0.88864	0.88950	0.89233	0.89538	0.89876
884	0.88053	0.88614	0.88873	0.88960	0.89242	0.89547	0.89885
885	0.88065	0.88625	0.88883	0.88970	0.89252	0.89556	0.89894
886	0.88075	0.88635	0.88893	0.88980	0.89262	0.89565	0.89902
887	0.88086	0.88645	0.88903	0.88990	0.89271	0.89575	0.89912
888	0.88096	0.88655	0.88913	0.89000	0.89281	0.89584	0.89921
889	0.88107	0.88665	0.88923	0.89009	0.89291	0.89593	0.89930
890	0.88118	0.88676	0.88933	0.89019	0.89300	0.89603	0.89938
891	0.88129	0.88686	0.88943	0.89029	0.89310	0.89612	0.89947
892	0.88139	0.88696	0.88953	0.89038	0.89319	0.89621	0.89956
893	0.88150	0.88706	0.88962	0.89048	0.89329	0.89630	0.89965
894	0.88160	0.88717	0.88972	0.89058	0.89338	0.89639	0.89974
895	0.88171	0.88727	0.88982	0.89067	0.89347	0.89648	0.89983
896	0.88182	0.88737	0.88992	0.89077	0.89357	0.89658	0.89991

N	0.1%	0.5%	1%	1.25%	2.5%	5%	10%
897	0.88191	0.88747	0.89002	0.89087	0.89366	0.89667	0.90000
898	0.88201	0.88756	0.89011	0.89097	0.89376	0.89676	0.90009
899	0.88213	0.88767	0.89021	0.89106	0.89385	0.89685	0.90018
900	0.88224	0.88777	0.89031	0.89116	0.89394	0.89694	0.90027
901	0.88235	0.88786	0.89041	0.89126	0.89404	0.89703	0.90035
902	0.88245	0.88796	0.89050	0.89135	0.89413	0.89712	0.90044
903	0.88256	0.88806	0.89060	0.89145	0.89422	0.89721	0.90053
904	0.88267	0.88816	0.89069	0.89155	0.89432	0.89730	0.90061
905	0.88277	0.88826	0.89079	0.89164	0.89441	0.89739	0.90070
906	0.88287	0.88836	0.89089	0.89174	0.89450	0.89748	0.90079
907	0.88298	0.88845	0.89099	0.89183	0.89459	0.89757	0.90087
908	0.88307	0.88855	0.89109	0.89193	0.89469	0.89766	0.90096
909	0.88317	0.88865	0.89118	0.89202	0.89478	0.89775	0.90105
910	0.88329	0.88875	0.89128	0.89212	0.89487	0.89784	0.90113
911	0.88339	0.88885	0.89137	0.89221	0.89496	0.89793	0.90122
912	0.88350	0.88895	0.89147	0.89231	0.89506	0.89802	0.90130
913	0.88359	0.88905	0.89156	0.89240	0.89515	0.89811	0.90139
914	0.88370	0.88914	0.89166	0.89250	0.89524	0.89819	0.90147
915	0.88380	0.88924	0.89175	0.89259	0.89533	0.89828	0.90156
916	0.88390	0.88934	0.89185	0.89269	0.89542	0.89837	0.90164
917	0.88402	0.88943	0.89194	0.89278	0.89551	0.89846	0.90173
918	0.88411	0.88953	0.89204	0.89287	0.89560	0.89854	0.90181
919	0.88421	0.88962	0.89213	0.89296	0.89569	0.89863	0.90190
920	0.88432	0.88972	0.89222	0.89306	0.89578	0.89872	0.90198
921	0.88441	0.88982	0.89232	0.89315	0.89588	0.89881	0.90206
922	0.88452	0.88991	0.89242	0.89324	0.89596	0.89889	0.90215
923	0.88464	0.89001	0.89251	0.89334	0.89605	0.89898	0.90223
924	0.88472	0.89011	0.89260	0.89343	0.89614	0.89907	0.90231
925	0.88482	0.89020	0.89270	0.89352	0.89623	0.89915	0.90240
926	0.88491	0.89030	0.89279	0.89362	0.89632	0.89924	0.90248
927	0.88502	0.89039	0.89288	0.89371	0.89641	0.89933	0.90257
928	0.88511	0.89049	0.89297	0.89380	0.89650	0.89941	0.90265
929	0.88521	0.89058	0.89306	0.89389	0.89659	0.89950	0.90273
930	0.88531	0.89068	0.89315	0.89398	0.89668	0.89958	0.90281
931	0.88541	0.89077	0.89324	0.89407	0.89676	0.89967	0.90290
932	0.88551	0.89086	0.89333	0.89416	0.89685	0.89975	0.90298
933	0.88561	0.89096	0.89343	0.89426	0.89694	0.89984	0.90306
934	0.88570	0.89105	0.89352	0.89434	0.89703	0.89992	0.90314
935	0.88581	0.89114	0.89361	0.89444	0.89712	0.90001	0.90323
936	0.88592	0.89124	0.89370	0.89453	0.89720	0.90009	0.90331
937	0.88602	0.89133	0.89379	0.89462	0.89729	0.90018	0.90339
938	0.88612	0.89142	0.89389	0.89471	0.89738	0.90026	0.90347
939	0.88623	0.89152	0.89397	0.89480	0.89747	0.90035	0.90355
940	0.88633	0.89161	0.89407	0.89489	0.89755	0.90043	0.90363
941	0.88642	0.89171	0.89416	0.89497	0.89764	0.90051	0.90371
942	0.88652	0.89180	0.89425	0.89506	0.89773	0.90060	0.90379
943	0.88661	0.89189	0.89434	0.89515	0.89782	0.90068	0.90387
944	0.88672	0.89199	0.89443	0.89524	0.89790	0.90077	0.90396
945	0.88681	0.89208	0.89452	0.89533	0.89798	0.90085	0.90404
946	0.88691	0.89217	0.89461	0.89542	0.89807	0.90093	0.90412
947	0.88700	0.89226	0.89470	0.89550	0.89816	0.90102	0.90420
948	0.88710	0.89235	0.89479	0.89560	0.89824	0.90110	0.90428
949	0.88719	0.89244	0.89488	0.89568	0.89833	0.90118	0.90436
950	0.88729	0.89253	0.89497	0.89577	0.89841	0.90126	0.90444
951	0.88738	0.89262	0.89505	0.89586	0.89850	0.90135	0.90452
952	0.88748	0.89271	0.89514	0.89595	0.89859	0.90143	0.90460
953	0.88756	0.89280	0.89523	0.89604	0.89867	0.90151	0.90468
954	0.88765	0.89290	0.89531	0.89612	0.89875	0.90159	0.90475
955	0.88775	0.89298	0.89540	0.89621	0.89884	0.90168	0.90483
956	0.88784	0.89307	0.89549	0.89629	0.89892	0.90176	0.90491
957	0.88794	0.89316	0.89558	0.89638	0.89901	0.90184	0.90499
958	0.88803	0.89325	0.89566	0.89647	0.89909	0.90192	0.90507
959	0.88813	0.89334	0.89575	0.89656	0.89918	0.90200	0.90515
960	0.88823	0.89343	0.89584	0.89665	0.89926	0.90208	0.90523
961	0.88833	0.89352	0.89592	0.89673	0.89934	0.90217	0.90530
962	0.88841	0.89361	0.89601	0.89681	0.89943	0.90225	0.90538
963	0.88851	0.89370	0.89610	0.89690	0.89951	0.90233	0.90546
964	0.88861	0.89379	0.89619	0.89699	0.89960	0.90241	0.90554
965	0.88869	0.89387	0.89627	0.89707	0.89968	0.90249	0.90561
966	0.88879	0.89397	0.89636	0.89716	0.89976	0.90257	0.90569
967	0.88888	0.89405	0.89644	0.89724	0.89984	0.90265	0.90577
968	0.88898	0.89414	0.89653	0.89733	0.89992	0.90273	0.90585
969	0.88907	0.89423	0.89662	0.89742	0.90001	0.90281	0.90592
970	0.88916	0.89431	0.89670	0.89750	0.90009	0.90289	0.90600

N	0.1%	0.5%	1%	1.25%	2.5%	5%	10%
971	0.88925	0.89440	0.89679	0.89759	0.90017	0.90297	0.90608
972	0.88934	0.89449	0.89688	0.89767	0.90025	0.90305	0.90615
973	0.88943	0.89457	0.89696	0.89776	0.90033	0.90313	0.90623
974	0.88952	0.89466	0.89705	0.89784	0.90042	0.90321	0.90630
975	0.88962	0.89475	0.89713	0.89792	0.90050	0.90328	0.90638
976	0.88971	0.89483	0.89722	0.89801	0.90058	0.90336	0.90646
977	0.88980	0.89492	0.89730	0.89809	0.90066	0.90344	0.90653
978	0.88988	0.89500	0.89738	0.89817	0.90074	0.90352	0.90661
979	0.88997	0.89508	0.89747	0.89825	0.90082	0.90360	0.90668
980	0.89006	0.89517	0.89755	0.89834	0.90090	0.90368	0.90676
981	0.89014	0.89526	0.89763	0.89842	0.90098	0.90376	0.90683
982	0.89023	0.89535	0.89771	0.89850	0.90106	0.90384	0.90691
983	0.89033	0.89543	0.89780	0.89858	0.90114	0.90392	0.90698
984	0.89042	0.89552	0.89788	0.89867	0.90123	0.90399	0.90706
985	0.89052	0.89561	0.89796	0.89875	0.90131	0.90407	0.90713
986	0.89061	0.89569	0.89805	0.89883	0.90139	0.90415	0.90721
987	0.89069	0.89577	0.89813	0.89891	0.90147	0.90422	0.90728
988	0.89078	0.89586	0.89821	0.89900	0.90155	0.90430	0.90736
989	0.89088	0.89594	0.89830	0.89908	0.90163	0.90438	0.90743
990	0.89097	0.89603	0.89838	0.89916	0.90171	0.90445	0.90751
991	0.89105	0.89611	0.89846	0.89924	0.90178	0.90453	0.90758
992	0.89115	0.89619	0.89855	0.89932	0.90186	0.90461	0.90765
993	0.89124	0.89628	0.89863	0.89940	0.90194	0.90469	0.90773
994	0.89133	0.89636	0.89871	0.89948	0.90202	0.90476	0.90780
995	0.89142	0.89644	0.89879	0.89957	0.90210	0.90484	0.90787
996	0.89150	0.89653	0.89887	0.89964	0.90218	0.90492	0.90795
997	0.89159	0.89661	0.89895	0.89973	0.90226	0.90499	0.90802
998	0.89167	0.89670	0.89903	0.89981	0.90233	0.90507	0.90809
999	0.89176	0.89678	0.89911	0.89989	0.90241	0.90514	0.90817
1000	0.89185	0.89686	0.89919	0.89997	0.90249	0.90522	0.90824

Table 6 – *GM10(5H5L)*. Critical values of upper significance levels for the Grubbs multiple-10 outlier test, applicable in situations where the five highest and five lowest observations are simultaneously identified as outliers, denoted as *GM10(5H5L)*.

N	0.1%	0.5%	1%	1.25%	2.5%	5%	10%
12	0.00000	0.00000	0.00000	0.00000	0.00000	0.00001	0.00003
13	0.00001	0.00003	0.00005	0.00007	0.00014	0.00030	0.00066
14	0.00009	0.00027	0.00044	0.00052	0.00087	0.00148	0.00259
15	0.00042	0.00100	0.00147	0.00167	0.00250	0.00381	0.00596
16	0.00117	0.00240	0.00331	0.00368	0.00515	0.00732	0.01066
17	0.00248	0.00458	0.00603	0.00660	0.00880	0.01193	0.01652
18	0.00439	0.00752	0.00957	0.01037	0.01338	0.01750	0.02334
19	0.00691	0.01121	0.01392	0.01494	0.01877	0.02388	0.03094
20	0.01008	0.01557	0.01894	0.02021	0.02484	0.03092	0.03916
21	0.01381	0.02052	0.02454	0.02604	0.03152	0.03853	0.04786
22	0.01808	0.02598	0.03070	0.03243	0.03865	0.04660	0.05693
23	0.02276	0.03199	0.03728	0.03925	0.04621	0.05496	0.06625
24	0.02793	0.03835	0.04425	0.04640	0.05409	0.06359	0.07578
25	0.03342	0.04500	0.05148	0.05383	0.06218	0.07243	0.08542
26	0.03928	0.05192	0.05896	0.06151	0.07046	0.08139	0.09514
27	0.04530	0.05906	0.06664	0.06937	0.07887	0.09044	0.10485
28	0.05164	0.06634	0.07445	0.07733	0.08739	0.09947	0.11452
29	0.05811	0.07378	0.08237	0.08540	0.09594	0.10855	0.12415
30	0.06478	0.08134	0.09031	0.09348	0.10449	0.11758	0.13372
31	0.07155	0.08893	0.09828	0.10162	0.11307	0.12662	0.14315
32	0.07853	0.09659	0.10633	0.10978	0.12159	0.13555	0.15254
33	0.08539	0.10434	0.11443	0.11795	0.13010	0.14442	0.16176
34	0.09242	0.11205	0.12242	0.12613	0.13857	0.15317	0.17087
35	0.09948	0.11978	0.13045	0.13416	0.14690	0.16183	0.17983
36	0.10659	0.12743	0.13836	0.14217	0.15520	0.17040	0.18864
37	0.11357	0.13502	0.14628	0.15015	0.16335	0.17882	0.19729
38	0.12071	0.14256	0.15399	0.15794	0.17145	0.18710	0.20581
39	0.12761	0.15011	0.16170	0.16572	0.17939	0.19528	0.21418
40	0.13466	0.15749	0.16929	0.17339	0.18729	0.20332	0.22236
41	0.14155	0.16485	0.17675	0.18096	0.19504	0.21125	0.23041
42	0.14841	0.17212	0.18418	0.18842	0.20272	0.21902	0.23835
43	0.15521	0.17929	0.19148	0.19580	0.21023	0.22669	0.24608
44	0.16207	0.18637	0.19882	0.20312	0.21763	0.23416	0.25369
45	0.16892	0.19334	0.20594	0.21028	0.22492	0.24156	0.26112
46	0.17560	0.20027	0.21296	0.21733	0.23207	0.24881	0.26843
47	0.18208	0.20721	0.21989	0.22433	0.23908	0.25591	0.27562
48	0.18847	0.21397	0.22678	0.23117	0.24604	0.26290	0.28266

N	0.1%	0.5%	1%	1.25%	2.5%	5%	10%
49	0.19496	0.22064	0.23347	0.23797	0.25285	0.26978	0.28952
50	0.20140	0.22721	0.24019	0.24465	0.25953	0.27651	0.29628
51	0.20773	0.23362	0.24667	0.25113	0.26616	0.28314	0.30292
52	0.21389	0.24004	0.25310	0.25763	0.27264	0.28962	0.30940
53	0.22001	0.24632	0.25944	0.26395	0.27901	0.29596	0.31577
54	0.22621	0.25250	0.26566	0.27018	0.28527	0.30225	0.32205
55	0.23197	0.25865	0.27179	0.27634	0.29141	0.30845	0.32816
56	0.23790	0.26459	0.27788	0.28243	0.29745	0.31448	0.33420
57	0.24379	0.27049	0.28379	0.28830	0.30341	0.32039	0.34013
58	0.24950	0.27634	0.28962	0.29411	0.30927	0.32622	0.34589
59	0.25518	0.28213	0.29534	0.29987	0.31500	0.33197	0.35158
60	0.26077	0.28768	0.30095	0.30552	0.32061	0.33762	0.35719
61	0.26629	0.29327	0.30654	0.31107	0.32620	0.34312	0.36267
62	0.27179	0.29878	0.31203	0.31654	0.33165	0.34857	0.36805
63	0.27716	0.30410	0.31737	0.32191	0.33701	0.35393	0.37332
64	0.28232	0.30938	0.32265	0.32724	0.34228	0.35917	0.37853
65	0.28751	0.31458	0.32790	0.33242	0.34748	0.36431	0.38361
66	0.29271	0.31975	0.33303	0.33758	0.35256	0.36937	0.38862
67	0.29776	0.32485	0.33812	0.34267	0.35758	0.37431	0.39351
68	0.30271	0.32979	0.34314	0.34766	0.36252	0.37919	0.39834
69	0.30757	0.33472	0.34803	0.35251	0.36736	0.38401	0.40309
70	0.31243	0.33964	0.35287	0.35735	0.37218	0.38874	0.40773
71	0.31738	0.34451	0.35762	0.36211	0.37688	0.39338	0.41232
72	0.32207	0.34913	0.36223	0.36677	0.38147	0.39799	0.41683
73	0.32661	0.35376	0.36682	0.37131	0.38605	0.40250	0.42125
74	0.33131	0.35826	0.37136	0.37581	0.39051	0.40692	0.42563
75	0.33578	0.36280	0.37584	0.38027	0.39496	0.41128	0.42991
76	0.34023	0.36725	0.38024	0.38465	0.39928	0.41555	0.43412
77	0.34476	0.37163	0.38452	0.38896	0.40354	0.41978	0.43827
78	0.34919	0.37594	0.38884	0.39321	0.40774	0.42392	0.44235
79	0.35357	0.38016	0.39307	0.39743	0.41191	0.42803	0.44636
80	0.35768	0.38435	0.39720	0.40158	0.41597	0.43207	0.45034
81	0.36186	0.38844	0.40127	0.40562	0.42002	0.43600	0.45422
82	0.36595	0.39248	0.40525	0.40960	0.42399	0.43993	0.45805
83	0.37007	0.39646	0.40919	0.41354	0.42787	0.44377	0.46181
84	0.37393	0.40048	0.41310	0.41741	0.43171	0.44754	0.46553
85	0.37805	0.40430	0.41696	0.42127	0.43551	0.45129	0.46917
86	0.38206	0.40814	0.42075	0.42505	0.43927	0.45497	0.47278
87	0.38586	0.41195	0.42451	0.42881	0.44294	0.45858	0.47633
88	0.38968	0.41572	0.42826	0.43253	0.44658	0.46213	0.47980
89	0.39340	0.41936	0.43189	0.43615	0.45015	0.46566	0.48325
90	0.39709	0.42301	0.43550	0.43973	0.45369	0.46913	0.48663
91	0.40063	0.42660	0.43905	0.44323	0.45720	0.47255	0.48996
92	0.40443	0.43016	0.44251	0.44673	0.46064	0.47592	0.49324
93	0.40805	0.43366	0.44598	0.45019	0.46398	0.47924	0.49647
94	0.41148	0.43710	0.44943	0.45357	0.46734	0.48250	0.49967
95	0.41489	0.44045	0.45274	0.45691	0.47062	0.48572	0.50283
96	0.41842	0.44379	0.45606	0.46023	0.47389	0.48892	0.50594
97	0.42180	0.44719	0.45937	0.46348	0.47708	0.49205	0.50900
98	0.42528	0.45049	0.46261	0.46671	0.48022	0.49518	0.51203
99	0.42844	0.45368	0.46578	0.46992	0.48335	0.49823	0.51500
100	0.43179	0.45693	0.46896	0.47304	0.48643	0.50125	0.51795
101	0.43507	0.46002	0.47204	0.47612	0.48950	0.50423	0.52085
102	0.43819	0.46313	0.47511	0.47918	0.49248	0.50717	0.52371
103	0.44125	0.46620	0.47820	0.48220	0.49544	0.51006	0.52654
104	0.44440	0.46920	0.48117	0.48521	0.49836	0.51288	0.52933
105	0.44746	0.47217	0.48414	0.48816	0.50124	0.51572	0.53208
106	0.45047	0.47515	0.48711	0.49107	0.50412	0.51851	0.53477
107	0.45350	0.47807	0.48995	0.49392	0.50691	0.52127	0.53746
108	0.45646	0.48101	0.49279	0.49676	0.50973	0.52400	0.54012
109	0.45937	0.48391	0.49560	0.49956	0.51247	0.52666	0.54272
110	0.46227	0.48673	0.49836	0.50230	0.51516	0.52932	0.54530
111	0.46517	0.48952	0.50111	0.50502	0.51783	0.53195	0.54785
112	0.46790	0.49239	0.50383	0.50772	0.52046	0.53455	0.55034
113	0.47075	0.49511	0.50651	0.51038	0.52308	0.53708	0.55282
114	0.47357	0.49770	0.50915	0.51301	0.52563	0.53961	0.55528
115	0.47637	0.50032	0.51178	0.51562	0.52820	0.54208	0.55769
116	0.47910	0.50291	0.51434	0.51815	0.53071	0.54455	0.56008
117	0.48174	0.50554	0.51690	0.52070	0.53321	0.54699	0.56245
118	0.48445	0.50814	0.51942	0.52320	0.53567	0.54936	0.56477
119	0.48704	0.51067	0.52192	0.52571	0.53812	0.55173	0.56708
120	0.48969	0.51316	0.52440	0.52817	0.54053	0.55408	0.56936
121	0.49224	0.51563	0.52681	0.53059	0.54291	0.55640	0.57161
122	0.49480	0.51808	0.52923	0.53298	0.54525	0.55869	0.57385

N	0.1%	0.5%	1%	1.25%	2.5%	5%	10%
123	0.49739	0.52050	0.53161	0.53538	0.54755	0.56093	0.57604
124	0.49984	0.52297	0.53400	0.53774	0.54986	0.56318	0.57822
125	0.50229	0.52530	0.53633	0.54004	0.55214	0.56540	0.58037
126	0.50472	0.52764	0.53862	0.54235	0.55439	0.56759	0.58251
127	0.50713	0.52993	0.54092	0.54460	0.55658	0.56975	0.58461
128	0.50955	0.53229	0.54318	0.54685	0.55877	0.57190	0.58669
129	0.51178	0.53457	0.54543	0.54907	0.56095	0.57404	0.58875
130	0.51416	0.53677	0.54763	0.55122	0.56309	0.57612	0.59077
131	0.51641	0.53900	0.54981	0.55343	0.56523	0.57818	0.59278
132	0.51881	0.54122	0.55198	0.55557	0.56732	0.58023	0.59477
133	0.52097	0.54341	0.55412	0.55770	0.56939	0.58226	0.59674
134	0.52325	0.54557	0.55622	0.55979	0.57145	0.58427	0.59868
135	0.52544	0.54773	0.55830	0.56186	0.57348	0.58625	0.60060
136	0.52761	0.54980	0.56037	0.56390	0.57551	0.58821	0.60250
137	0.52985	0.55193	0.56239	0.56593	0.57750	0.59014	0.60438
138	0.53201	0.55401	0.56441	0.56796	0.57950	0.59208	0.60624
139	0.53415	0.55605	0.56641	0.56994	0.58143	0.59397	0.60808
140	0.53621	0.55811	0.56842	0.57189	0.58336	0.59587	0.60990
141	0.53841	0.56010	0.57040	0.57385	0.58527	0.59773	0.61171
142	0.54043	0.56209	0.57231	0.57577	0.58717	0.59958	0.61349
143	0.54249	0.56410	0.57428	0.57770	0.58904	0.60140	0.61526
144	0.54452	0.56606	0.57618	0.57963	0.59090	0.60319	0.61701
145	0.54655	0.56796	0.57809	0.58151	0.59274	0.60499	0.61873
146	0.54857	0.56983	0.57997	0.58340	0.59456	0.60675	0.62045
147	0.55061	0.57173	0.58184	0.58525	0.59638	0.60851	0.62215
148	0.55247	0.57358	0.58368	0.58707	0.59815	0.61023	0.62382
149	0.55440	0.57543	0.58554	0.58887	0.59991	0.61194	0.62547
150	0.55629	0.57731	0.58733	0.59068	0.60165	0.61366	0.62713
151	0.55818	0.57914	0.58910	0.59242	0.60339	0.61533	0.62875
152	0.56002	0.58094	0.59085	0.59420	0.60510	0.61701	0.63037
153	0.56198	0.58272	0.59261	0.59594	0.60679	0.61866	0.63195
154	0.56381	0.58450	0.59436	0.59765	0.60848	0.62030	0.63354
155	0.56563	0.58629	0.59609	0.59939	0.61016	0.62192	0.63510
156	0.56751	0.58804	0.59779	0.60106	0.61179	0.62353	0.63666
157	0.56925	0.58973	0.59946	0.60274	0.61343	0.62512	0.63819
158	0.57101	0.59146	0.60114	0.60439	0.61505	0.62668	0.63971
159	0.57269	0.59312	0.60279	0.60604	0.61664	0.62824	0.64123
160	0.57447	0.59479	0.60441	0.60768	0.61824	0.62979	0.64273
161	0.57617	0.59647	0.60605	0.60929	0.61979	0.63131	0.64420
162	0.57790	0.59811	0.60764	0.61087	0.62137	0.63283	0.64567
163	0.57956	0.59974	0.60922	0.61245	0.62292	0.63433	0.64712
164	0.58131	0.60138	0.61083	0.61402	0.62444	0.63580	0.64856
165	0.58291	0.60297	0.61238	0.61558	0.62595	0.63729	0.64998
166	0.58462	0.60456	0.61394	0.61710	0.62743	0.63875	0.65139
167	0.58625	0.60611	0.61545	0.61863	0.62893	0.64020	0.65279
168	0.58790	0.60768	0.61697	0.62015	0.63042	0.64164	0.65417
169	0.58954	0.60922	0.61850	0.62165	0.63189	0.64307	0.65555
170	0.59115	0.61075	0.61999	0.62314	0.63334	0.64447	0.65691
171	0.59270	0.61229	0.62149	0.62461	0.63478	0.64587	0.65826
172	0.59423	0.61378	0.62296	0.62606	0.63621	0.64726	0.65960
173	0.59581	0.61530	0.62444	0.62754	0.63763	0.64863	0.66093
174	0.59732	0.61676	0.62589	0.62897	0.63900	0.65000	0.66225
175	0.59888	0.61825	0.62734	0.63038	0.64040	0.65134	0.66355
176	0.60041	0.61969	0.62876	0.63181	0.64178	0.65268	0.66484
177	0.60197	0.62114	0.63019	0.63322	0.64315	0.65399	0.66612
178	0.60342	0.62258	0.63159	0.63459	0.64450	0.65532	0.66740
179	0.60489	0.62399	0.63297	0.63597	0.64584	0.65663	0.66866
180	0.60636	0.62543	0.63436	0.63732	0.64718	0.65791	0.66991
181	0.60781	0.62682	0.63572	0.63865	0.64848	0.65918	0.67114
182	0.60919	0.62819	0.63706	0.64003	0.64980	0.66045	0.67237
183	0.61065	0.62956	0.63842	0.64136	0.65111	0.66172	0.67358
184	0.61209	0.63093	0.63973	0.64268	0.65239	0.66296	0.67478
185	0.61349	0.63227	0.64105	0.64400	0.65366	0.66422	0.67599
186	0.61495	0.63361	0.64236	0.64530	0.65493	0.66544	0.67718
187	0.61637	0.63496	0.64367	0.64661	0.65618	0.66666	0.67836
188	0.61775	0.63625	0.64493	0.64786	0.65743	0.66787	0.67953
189	0.61915	0.63756	0.64621	0.64914	0.65868	0.66905	0.68069
190	0.62052	0.63884	0.64749	0.65039	0.65989	0.67026	0.68184
191	0.62185	0.64016	0.64872	0.65162	0.66111	0.67144	0.68298
192	0.62314	0.64141	0.64995	0.65285	0.66233	0.67261	0.68411
193	0.62439	0.64269	0.65121	0.65409	0.66352	0.67377	0.68524
194	0.62567	0.64394	0.65243	0.65530	0.66470	0.67493	0.68635
195	0.62702	0.64519	0.65364	0.65652	0.66589	0.67607	0.68746
196	0.62835	0.64640	0.65484	0.65771	0.66705	0.67720	0.68855

N	0.1%	0.5%	1%	1.25%	2.5%	5%	10%
197	0.62964	0.64762	0.65604	0.65889	0.66823	0.67833	0.68964
198	0.63092	0.64885	0.65721	0.66007	0.66937	0.67945	0.69072
199	0.63216	0.65004	0.65842	0.66127	0.67051	0.68056	0.69179
200	0.63342	0.65124	0.65960	0.66244	0.67164	0.68166	0.69284
201	0.63465	0.65242	0.66077	0.66360	0.67277	0.68275	0.69390
202	0.63589	0.65363	0.66192	0.66475	0.67389	0.68384	0.69495
203	0.63713	0.65480	0.66306	0.66589	0.67500	0.68492	0.69598
204	0.63835	0.65597	0.66422	0.66702	0.67610	0.68598	0.69701
205	0.63955	0.65713	0.66537	0.66815	0.67720	0.68704	0.69804
206	0.64075	0.65827	0.66650	0.66924	0.67828	0.68809	0.69906
207	0.64193	0.65937	0.66762	0.67036	0.67934	0.68914	0.70006
208	0.64307	0.66050	0.66872	0.67147	0.68042	0.69017	0.70107
209	0.64423	0.66165	0.66980	0.67256	0.68147	0.69120	0.70206
210	0.64543	0.66276	0.67090	0.67364	0.68254	0.69221	0.70305
211	0.64660	0.66389	0.67196	0.67471	0.68357	0.69322	0.70403
212	0.64770	0.66497	0.67303	0.67578	0.68462	0.69423	0.70499
213	0.64884	0.66606	0.67410	0.67683	0.68565	0.69523	0.70595
214	0.65004	0.66713	0.67518	0.67789	0.68667	0.69623	0.70690
215	0.65115	0.66820	0.67623	0.67892	0.68769	0.69721	0.70785
216	0.65223	0.66930	0.67726	0.67996	0.68869	0.69819	0.70880
217	0.65328	0.67036	0.67830	0.68097	0.68969	0.69916	0.70973
218	0.65443	0.67145	0.67932	0.68199	0.69069	0.70012	0.71066
219	0.65559	0.67247	0.68035	0.68300	0.69167	0.70108	0.71158
220	0.65668	0.67351	0.68135	0.68400	0.69266	0.70203	0.71250
221	0.65768	0.67452	0.68238	0.68500	0.69362	0.70298	0.71340
222	0.65875	0.67555	0.68338	0.68599	0.69458	0.70391	0.71430
223	0.65983	0.67657	0.68437	0.68696	0.69552	0.70483	0.71520
224	0.66095	0.67756	0.68534	0.68794	0.69647	0.70576	0.71609
225	0.66199	0.67858	0.68633	0.68891	0.69743	0.70668	0.71697
226	0.66299	0.67958	0.68729	0.68989	0.69836	0.70760	0.71785
227	0.66401	0.68057	0.68827	0.69086	0.69930	0.70850	0.71872
228	0.66506	0.68154	0.68922	0.69180	0.70020	0.70939	0.71959
229	0.66608	0.68253	0.69018	0.69276	0.70114	0.71029	0.72045
230	0.66710	0.68351	0.69112	0.69369	0.70205	0.71117	0.72130
231	0.66811	0.68447	0.69205	0.69462	0.70296	0.71204	0.72215
232	0.66904	0.68542	0.69297	0.69555	0.70385	0.71292	0.72299
233	0.67007	0.68637	0.69391	0.69647	0.70476	0.71378	0.72383
234	0.67104	0.68733	0.69484	0.69740	0.70563	0.71464	0.72466
235	0.67203	0.68823	0.69576	0.69830	0.70651	0.71549	0.72548
236	0.67305	0.68917	0.69668	0.69921	0.70738	0.71634	0.72630
237	0.67405	0.69011	0.69758	0.70009	0.70824	0.71718	0.72712
238	0.67499	0.69102	0.69845	0.70097	0.70912	0.71802	0.72792
239	0.67597	0.69194	0.69936	0.70185	0.70999	0.71885	0.72873
240	0.67690	0.69282	0.70025	0.70273	0.71085	0.71968	0.72953
241	0.67791	0.69372	0.70114	0.70360	0.71170	0.72049	0.73032
242	0.67883	0.69463	0.70199	0.70448	0.71255	0.72131	0.73111
243	0.67978	0.69552	0.70288	0.70533	0.71337	0.72211	0.73188
244	0.68075	0.69640	0.70376	0.70619	0.71421	0.72292	0.73266
245	0.68164	0.69728	0.70461	0.70706	0.71504	0.72373	0.73343
246	0.68256	0.69813	0.70546	0.70792	0.71585	0.72452	0.73420
247	0.68350	0.69902	0.70630	0.70875	0.71667	0.72532	0.73496
248	0.68440	0.69986	0.70714	0.70957	0.71748	0.72610	0.73572
249	0.68523	0.70072	0.70798	0.71040	0.71829	0.72688	0.73647
250	0.68612	0.70158	0.70880	0.71121	0.71909	0.72766	0.73721
251	0.68702	0.70242	0.70963	0.71203	0.71989	0.72843	0.73796
252	0.68784	0.70324	0.71045	0.71284	0.72068	0.72919	0.73870
253	0.68876	0.70405	0.71127	0.71366	0.72146	0.72995	0.73943
254	0.68968	0.70489	0.71205	0.71445	0.72224	0.73071	0.74016
255	0.69054	0.70570	0.71286	0.71526	0.72301	0.73146	0.74089
256	0.69141	0.70653	0.71366	0.71604	0.72379	0.73221	0.74161
257	0.69225	0.70732	0.71446	0.71682	0.72455	0.73295	0.74232
258	0.69305	0.70814	0.71525	0.71761	0.72531	0.73369	0.74303
259	0.69387	0.70893	0.71603	0.71839	0.72607	0.73443	0.74374
260	0.69469	0.70973	0.71681	0.71916	0.72682	0.73516	0.74445
261	0.69557	0.71052	0.71756	0.71992	0.72755	0.73588	0.74514
262	0.69640	0.71130	0.71836	0.72068	0.72830	0.73660	0.74583
263	0.69723	0.71210	0.71913	0.72145	0.72905	0.73732	0.74653
264	0.69802	0.71289	0.71988	0.72221	0.72979	0.73802	0.74721
265	0.69888	0.71367	0.72064	0.72295	0.73052	0.73873	0.74789
266	0.69974	0.71443	0.72140	0.72371	0.73123	0.73943	0.74857
267	0.70052	0.71520	0.72214	0.72445	0.73195	0.74013	0.74924
268	0.70128	0.71598	0.72288	0.72519	0.73267	0.74082	0.74991
269	0.70209	0.71675	0.72361	0.72592	0.73339	0.74151	0.75058
270	0.70287	0.71752	0.72434	0.72663	0.73409	0.74220	0.75124

N	0.1%	0.5%	1%	1.25%	2.5%	5%	10%
271	0.70364	0.71828	0.72507	0.72737	0.73480	0.74288	0.75190
272	0.70439	0.71900	0.72579	0.72808	0.73551	0.74356	0.75255
273	0.70518	0.71976	0.72652	0.72881	0.73620	0.74424	0.75319
274	0.70596	0.72048	0.72724	0.72951	0.73689	0.74491	0.75384
275	0.70676	0.72121	0.72796	0.73022	0.73757	0.74557	0.75448
276	0.70755	0.72196	0.72868	0.73093	0.73826	0.74624	0.75512
277	0.70832	0.72268	0.72938	0.73163	0.73895	0.74689	0.75576
278	0.70906	0.72339	0.73009	0.73233	0.73963	0.74755	0.75639
279	0.70982	0.72410	0.73077	0.73301	0.74030	0.74822	0.75702
280	0.71052	0.72480	0.73147	0.73371	0.74096	0.74886	0.75764
281	0.71127	0.72552	0.73217	0.73439	0.74163	0.74950	0.75826
282	0.71207	0.72623	0.73285	0.73508	0.74230	0.75014	0.75888
283	0.71279	0.72691	0.73353	0.73576	0.74296	0.75078	0.75949
284	0.71357	0.72760	0.73421	0.73642	0.74361	0.75141	0.76010
285	0.71426	0.72830	0.73489	0.73709	0.74426	0.75204	0.76071
286	0.71493	0.72897	0.73558	0.73776	0.74490	0.75266	0.76131
287	0.71568	0.72969	0.73624	0.73842	0.74554	0.75328	0.76191
288	0.71636	0.73036	0.73691	0.73908	0.74617	0.75390	0.76250
289	0.71706	0.73105	0.73757	0.73973	0.74681	0.75452	0.76310
290	0.71772	0.73172	0.73822	0.74039	0.74745	0.75513	0.76369
291	0.71846	0.73238	0.73888	0.74104	0.74808	0.75575	0.76428
292	0.71912	0.73305	0.73953	0.74167	0.74871	0.75635	0.76486
293	0.71980	0.73370	0.74017	0.74231	0.74933	0.75696	0.76544
294	0.72054	0.73436	0.74079	0.74294	0.74994	0.75755	0.76602
295	0.72121	0.73499	0.74144	0.74359	0.75056	0.75814	0.76659
296	0.72189	0.73565	0.74207	0.74421	0.75117	0.75875	0.76717
297	0.72259	0.73630	0.74270	0.74485	0.75179	0.75934	0.76773
298	0.72329	0.73692	0.74333	0.74546	0.75238	0.75992	0.76829
299	0.72392	0.73756	0.74397	0.74607	0.75299	0.76050	0.76886
300	0.72459	0.73822	0.74458	0.74669	0.75358	0.76107	0.76941
301	0.72527	0.73886	0.74521	0.74731	0.75418	0.76165	0.76997
302	0.72591	0.73950	0.74581	0.74792	0.75477	0.76222	0.77052
303	0.72661	0.74013	0.74642	0.74852	0.75536	0.76279	0.77107
304	0.72725	0.74075	0.74703	0.74912	0.75594	0.76335	0.77161
305	0.72796	0.74136	0.74763	0.74973	0.75653	0.76392	0.77216
306	0.72859	0.74197	0.74824	0.75033	0.75710	0.76449	0.77270
307	0.72921	0.74257	0.74882	0.75093	0.75769	0.76505	0.77324
308	0.72988	0.74320	0.74942	0.75151	0.75826	0.76560	0.77377
309	0.73054	0.74378	0.75001	0.75211	0.75884	0.76615	0.77431
310	0.73116	0.74438	0.75059	0.75269	0.75941	0.76670	0.77483
311	0.73178	0.74498	0.75117	0.75327	0.75997	0.76724	0.77536
312	0.73240	0.74558	0.75176	0.75385	0.76053	0.76779	0.77588
313	0.73300	0.74619	0.75233	0.75441	0.76110	0.76833	0.77640
314	0.73362	0.74678	0.75293	0.75499	0.76165	0.76887	0.77692
315	0.73426	0.74737	0.75350	0.75555	0.76219	0.76941	0.77744
316	0.73488	0.74794	0.75407	0.75612	0.76275	0.76994	0.77795
317	0.73550	0.74853	0.75464	0.75669	0.76330	0.77047	0.77846
318	0.73611	0.74912	0.75519	0.75725	0.76384	0.77100	0.77897
319	0.73673	0.74969	0.75576	0.75780	0.76438	0.77152	0.77947
320	0.73730	0.75029	0.75632	0.75836	0.76493	0.77204	0.77997
321	0.73787	0.75086	0.75689	0.75891	0.76546	0.77256	0.78047
322	0.73846	0.75140	0.75744	0.75945	0.76600	0.77308	0.78097
323	0.73909	0.75196	0.75797	0.75998	0.76653	0.77359	0.78147
324	0.73970	0.75252	0.75852	0.76054	0.76706	0.77411	0.78196
325	0.74025	0.75309	0.75906	0.76109	0.76757	0.77461	0.78245
326	0.74082	0.75362	0.75960	0.76162	0.76809	0.77512	0.78294
327	0.74143	0.75419	0.76015	0.76215	0.76862	0.77562	0.78343
328	0.74201	0.75474	0.76068	0.76269	0.76913	0.77612	0.78390
329	0.74262	0.75530	0.76122	0.76323	0.76963	0.77662	0.78438
330	0.74319	0.75583	0.76174	0.76375	0.77015	0.77712	0.78486
331	0.74377	0.75637	0.76228	0.76428	0.77067	0.77761	0.78534
332	0.74434	0.75692	0.76281	0.76479	0.77117	0.77811	0.78581
333	0.74494	0.75744	0.76333	0.76532	0.77168	0.77859	0.78628
334	0.74546	0.75797	0.76386	0.76583	0.77218	0.77909	0.78675
335	0.74601	0.75852	0.76438	0.76634	0.77268	0.77957	0.78721
336	0.74658	0.75904	0.76490	0.76686	0.77318	0.78005	0.78767
337	0.74713	0.75958	0.76542	0.76736	0.77367	0.78053	0.78814
338	0.74767	0.76011	0.76593	0.76787	0.77417	0.78101	0.78860
339	0.74821	0.76062	0.76643	0.76839	0.77467	0.78148	0.78906
340	0.74874	0.76115	0.76694	0.76889	0.77516	0.78195	0.78952
341	0.74930	0.76166	0.76745	0.76938	0.77564	0.78242	0.78997
342	0.74983	0.76216	0.76796	0.76989	0.77613	0.78289	0.79042
343	0.75038	0.76267	0.76845	0.77039	0.77662	0.78336	0.79087
344	0.75093	0.76320	0.76895	0.77088	0.77709	0.78382	0.79132

N	0.1%	0.5%	1%	1.25%	2.5%	5%	10%
345	0.75148	0.76370	0.76945	0.77138	0.77756	0.78428	0.79176
346	0.75203	0.76419	0.76994	0.77186	0.77803	0.78474	0.79220
347	0.75258	0.76471	0.77042	0.77235	0.77851	0.78520	0.79264
348	0.75310	0.76521	0.77092	0.77284	0.77899	0.78566	0.79308
349	0.75364	0.76570	0.77142	0.77332	0.77946	0.78611	0.79351
350	0.75415	0.76619	0.77190	0.77379	0.77992	0.78656	0.79395
351	0.75466	0.76670	0.77238	0.77428	0.78039	0.78701	0.79438
352	0.75517	0.76719	0.77285	0.77476	0.78085	0.78745	0.79481
353	0.75566	0.76767	0.77332	0.77522	0.78131	0.78790	0.79524
354	0.75619	0.76815	0.77380	0.77570	0.78177	0.78834	0.79567
355	0.75669	0.76864	0.77428	0.77616	0.78222	0.78878	0.79609
356	0.75719	0.76911	0.77475	0.77662	0.78268	0.78922	0.79651
357	0.75768	0.76961	0.77522	0.77709	0.78313	0.78966	0.79693
358	0.75819	0.77008	0.77568	0.77755	0.78357	0.79009	0.79735
359	0.75866	0.77055	0.77614	0.77802	0.78402	0.79052	0.79776
360	0.75916	0.77102	0.77662	0.77847	0.78447	0.79095	0.79818
361	0.75966	0.77151	0.77708	0.77893	0.78492	0.79138	0.79859
362	0.76016	0.77197	0.77752	0.77938	0.78535	0.79181	0.79901
363	0.76067	0.77245	0.77798	0.77984	0.78579	0.79223	0.79941
364	0.76116	0.77291	0.77844	0.78029	0.78622	0.79266	0.79982
365	0.76162	0.77336	0.77890	0.78073	0.78665	0.79308	0.80022
366	0.76214	0.77382	0.77934	0.78118	0.78709	0.79350	0.80063
367	0.76263	0.77430	0.77978	0.78163	0.78752	0.79392	0.80103
368	0.76307	0.77474	0.78022	0.78205	0.78794	0.79434	0.80143
369	0.76353	0.77520	0.78067	0.78249	0.78837	0.79474	0.80183
370	0.76405	0.77566	0.78111	0.78293	0.78879	0.79516	0.80223
371	0.76451	0.77611	0.78154	0.78337	0.78921	0.79557	0.80262
372	0.76501	0.77657	0.78198	0.78380	0.78964	0.79597	0.80301
373	0.76543	0.77700	0.78241	0.78423	0.79006	0.79638	0.80340
374	0.76593	0.77746	0.78285	0.78466	0.79048	0.79678	0.80380
375	0.76638	0.77789	0.78329	0.78509	0.79090	0.79718	0.80418
376	0.76682	0.77834	0.78371	0.78552	0.79131	0.79758	0.80457
377	0.76726	0.77878	0.78415	0.78594	0.79172	0.79798	0.80496
378	0.76772	0.77921	0.78457	0.78636	0.79213	0.79838	0.80534
379	0.76818	0.77966	0.78498	0.78677	0.79254	0.79877	0.80571
380	0.76867	0.78010	0.78541	0.78719	0.79294	0.79917	0.80609
381	0.76913	0.78054	0.78584	0.78762	0.79335	0.79956	0.80647
382	0.76958	0.78097	0.78626	0.78804	0.79375	0.79995	0.80685
383	0.77005	0.78138	0.78667	0.78845	0.79415	0.80034	0.80722
384	0.77050	0.78180	0.78709	0.78887	0.79455	0.80073	0.80760
385	0.77098	0.78224	0.78751	0.78927	0.79496	0.80111	0.80797
386	0.77144	0.78267	0.78791	0.78967	0.79535	0.80149	0.80834
387	0.77186	0.78308	0.78832	0.79007	0.79575	0.80188	0.80871
388	0.77230	0.78350	0.78872	0.79048	0.79614	0.80226	0.80907
389	0.77274	0.78391	0.78913	0.79089	0.79654	0.80264	0.80944
390	0.77315	0.78434	0.78953	0.79128	0.79692	0.80302	0.80980
391	0.77359	0.78476	0.78994	0.79167	0.79731	0.80339	0.81016
392	0.77405	0.78518	0.79034	0.79207	0.79769	0.80377	0.81052
393	0.77449	0.78557	0.79074	0.79246	0.79808	0.80414	0.81088
394	0.77492	0.78600	0.79114	0.79286	0.79846	0.80451	0.81124
395	0.77535	0.78639	0.79154	0.79326	0.79885	0.80488	0.81160
396	0.77578	0.78680	0.79194	0.79365	0.79923	0.80525	0.81195
397	0.77619	0.78720	0.79233	0.79405	0.79960	0.80562	0.81230
398	0.77660	0.78760	0.79272	0.79443	0.79999	0.80598	0.81265
399	0.77700	0.78800	0.79311	0.79482	0.80036	0.80634	0.81300
400	0.77743	0.78840	0.79350	0.79520	0.80074	0.80670	0.81335
401	0.77784	0.78880	0.79388	0.79557	0.80111	0.80707	0.81369
402	0.77828	0.78919	0.79427	0.79597	0.80148	0.80743	0.81404
403	0.77870	0.78958	0.79466	0.79636	0.80185	0.80778	0.81439
404	0.77910	0.78997	0.79504	0.79674	0.80222	0.80814	0.81473
405	0.77950	0.79036	0.79542	0.79712	0.80258	0.80849	0.81507
406	0.77991	0.79075	0.79579	0.79749	0.80295	0.80885	0.81541
407	0.78030	0.79114	0.79617	0.79787	0.80332	0.80919	0.81575
408	0.78073	0.79153	0.79655	0.79824	0.80368	0.80955	0.81608
409	0.78115	0.79192	0.79693	0.79861	0.80405	0.80989	0.81642
410	0.78157	0.79230	0.79730	0.79898	0.80440	0.81024	0.81675
411	0.78196	0.79268	0.79767	0.79934	0.80475	0.81058	0.81708
412	0.78235	0.79306	0.79804	0.79971	0.80511	0.81093	0.81742
413	0.78276	0.79343	0.79840	0.80007	0.80546	0.81127	0.81775
414	0.78315	0.79381	0.79877	0.80044	0.80582	0.81161	0.81808
415	0.78354	0.79420	0.79913	0.80080	0.80616	0.81195	0.81840
416	0.78395	0.79458	0.79950	0.80116	0.80651	0.81229	0.81873
417	0.78435	0.79494	0.79986	0.80152	0.80686	0.81263	0.81905
418	0.78476	0.79530	0.80023	0.80186	0.80721	0.81297	0.81938

N	0.1%	0.5%	1%	1.25%	2.5%	5%	10%
419	0.78514	0.79567	0.80058	0.80223	0.80756	0.81331	0.81970
420	0.78554	0.79603	0.80094	0.80258	0.80790	0.81364	0.82002
421	0.78593	0.79641	0.80130	0.80294	0.80824	0.81397	0.82034
422	0.78631	0.79677	0.80166	0.80329	0.80859	0.81430	0.82066
423	0.78669	0.79713	0.80201	0.80365	0.80892	0.81463	0.82098
424	0.78704	0.79749	0.80236	0.80400	0.80926	0.81495	0.82129
425	0.78745	0.79786	0.80271	0.80434	0.80959	0.81528	0.82161
426	0.78780	0.79821	0.80307	0.80468	0.80993	0.81562	0.82192
427	0.78818	0.79858	0.80341	0.80502	0.81027	0.81594	0.82223
428	0.78857	0.79895	0.80376	0.80536	0.81060	0.81627	0.82254
429	0.78893	0.79930	0.80411	0.80571	0.81093	0.81659	0.82285
430	0.78930	0.79965	0.80445	0.80605	0.81126	0.81690	0.82316
431	0.78968	0.79999	0.80479	0.80639	0.81159	0.81722	0.82347
432	0.79006	0.80036	0.80513	0.80673	0.81192	0.81754	0.82377
433	0.79043	0.80070	0.80547	0.80707	0.81224	0.81785	0.82408
434	0.79078	0.80105	0.80581	0.80740	0.81257	0.81817	0.82438
435	0.79115	0.80139	0.80615	0.80774	0.81290	0.81848	0.82469
436	0.79150	0.80174	0.80648	0.80808	0.81323	0.81880	0.82499
437	0.79189	0.80208	0.80684	0.80841	0.81355	0.81911	0.82529
438	0.79222	0.80241	0.80717	0.80874	0.81387	0.81942	0.82559
439	0.79259	0.80276	0.80750	0.80906	0.81419	0.81973	0.82589
440	0.79299	0.80310	0.80783	0.80939	0.81451	0.82004	0.82618
441	0.79333	0.80343	0.80815	0.80972	0.81483	0.82034	0.82648
442	0.79370	0.80377	0.80849	0.81003	0.81515	0.82065	0.82677
443	0.79403	0.80410	0.80880	0.81036	0.81546	0.82096	0.82707
444	0.79437	0.80442	0.80913	0.81069	0.81577	0.82126	0.82736
445	0.79473	0.80476	0.80946	0.81102	0.81609	0.82157	0.82766
446	0.79509	0.80510	0.80978	0.81134	0.81640	0.82187	0.82795
447	0.79542	0.80543	0.81010	0.81165	0.81671	0.82218	0.82824
448	0.79580	0.80576	0.81043	0.81197	0.81702	0.82248	0.82852
449	0.79613	0.80610	0.81075	0.81229	0.81734	0.82277	0.82881
450	0.79651	0.80642	0.81107	0.81260	0.81764	0.82307	0.82909
451	0.79684	0.80675	0.81139	0.81293	0.81795	0.82336	0.82938
452	0.79717	0.80707	0.81170	0.81324	0.81825	0.82365	0.82966
453	0.79752	0.80739	0.81201	0.81355	0.81855	0.82395	0.82995
454	0.79788	0.80772	0.81232	0.81386	0.81886	0.82424	0.83023
455	0.79822	0.80804	0.81264	0.81416	0.81916	0.82454	0.83051
456	0.79857	0.80836	0.81295	0.81447	0.81946	0.82483	0.83079
457	0.79892	0.80868	0.81326	0.81479	0.81976	0.82512	0.83107
458	0.79925	0.80899	0.81357	0.81510	0.82005	0.82541	0.83134
459	0.79957	0.80931	0.81388	0.81541	0.82035	0.82570	0.83162
460	0.79991	0.80963	0.81419	0.81571	0.82065	0.82598	0.83190
461	0.80025	0.80995	0.81450	0.81601	0.82094	0.82627	0.83217
462	0.80058	0.81025	0.81480	0.81632	0.82123	0.82655	0.83245
463	0.80092	0.81057	0.81511	0.81661	0.82152	0.82684	0.83272
464	0.80124	0.81088	0.81541	0.81692	0.82181	0.82712	0.83299
465	0.80155	0.81119	0.81572	0.81722	0.82210	0.82740	0.83326
466	0.80190	0.81150	0.81601	0.81752	0.82239	0.82768	0.83353
467	0.80224	0.81181	0.81631	0.81781	0.82268	0.82796	0.83380
468	0.80254	0.81212	0.81660	0.81810	0.82297	0.82824	0.83407
469	0.80284	0.81243	0.81690	0.81841	0.82325	0.82852	0.83433
470	0.80316	0.81272	0.81719	0.81869	0.82354	0.82879	0.83460
471	0.80347	0.81302	0.81749	0.81899	0.82382	0.82907	0.83487
472	0.80380	0.81333	0.81778	0.81929	0.82411	0.82934	0.83513
473	0.80411	0.81364	0.81807	0.81957	0.82439	0.82962	0.83540
474	0.80441	0.81394	0.81837	0.81987	0.82467	0.82989	0.83566
475	0.80473	0.81425	0.81866	0.82016	0.82495	0.83016	0.83592
476	0.80507	0.81455	0.81895	0.82044	0.82523	0.83043	0.83618
477	0.80539	0.81483	0.81924	0.82072	0.82551	0.83071	0.83644
478	0.80568	0.81514	0.81953	0.82101	0.82579	0.83097	0.83670
479	0.80599	0.81543	0.81983	0.82130	0.82606	0.83124	0.83696
480	0.80632	0.81573	0.82011	0.82159	0.82634	0.83151	0.83721
481	0.80662	0.81602	0.82040	0.82186	0.82662	0.83177	0.83747
482	0.80693	0.81631	0.82068	0.82214	0.82689	0.83204	0.83772
483	0.80725	0.81661	0.82096	0.82243	0.82716	0.83230	0.83798
484	0.80756	0.81689	0.82125	0.82270	0.82743	0.83257	0.83823
485	0.80789	0.81718	0.82152	0.82298	0.82770	0.83283	0.83848
486	0.80820	0.81748	0.82181	0.82326	0.82797	0.83309	0.83874
487	0.80853	0.81775	0.82209	0.82354	0.82824	0.83335	0.83899
488	0.80881	0.81804	0.82236	0.82381	0.82851	0.83361	0.83924
489	0.80910	0.81832	0.82263	0.82409	0.82877	0.83387	0.83949
490	0.80938	0.81861	0.82291	0.82436	0.82904	0.83412	0.83973
491	0.80968	0.81889	0.82319	0.82463	0.82930	0.83438	0.83998
492	0.80996	0.81917	0.82347	0.82491	0.82957	0.83464	0.84023

N	0.1%	0.5%	1%	1.25%	2.5%	5%	10%
493	0.81027	0.81945	0.82374	0.82518	0.82983	0.83489	0.84047
494	0.81055	0.81972	0.82402	0.82546	0.83009	0.83514	0.84072
495	0.81086	0.82000	0.82428	0.82572	0.83036	0.83539	0.84096
496	0.81114	0.82027	0.82456	0.82599	0.83062	0.83565	0.84121
497	0.81143	0.82054	0.82483	0.82627	0.83088	0.83590	0.84145
498	0.81172	0.82082	0.82510	0.82653	0.83114	0.83615	0.84169
499	0.81203	0.82110	0.82537	0.82679	0.83140	0.83640	0.84193
500	0.81232	0.82137	0.82563	0.82706	0.83166	0.83665	0.84217
501	0.81259	0.82164	0.82589	0.82733	0.83192	0.83690	0.84241
502	0.81288	0.82192	0.82616	0.82759	0.83217	0.83714	0.84265
503	0.81316	0.82219	0.82642	0.82786	0.83243	0.83739	0.84289
504	0.81346	0.82247	0.82669	0.82812	0.83268	0.83764	0.84313
505	0.81373	0.82274	0.82696	0.82838	0.83294	0.83789	0.84336
506	0.81400	0.82302	0.82723	0.82864	0.83320	0.83813	0.84360
507	0.81431	0.82328	0.82748	0.82891	0.83345	0.83837	0.84383
508	0.81460	0.82356	0.82775	0.82917	0.83370	0.83862	0.84406
509	0.81487	0.82381	0.82801	0.82942	0.83395	0.83886	0.84429
510	0.81515	0.82408	0.82827	0.82968	0.83420	0.83910	0.84453
511	0.81543	0.82435	0.82853	0.82994	0.83444	0.83934	0.84476
512	0.81571	0.82460	0.82879	0.83019	0.83469	0.83958	0.84499
513	0.81600	0.82487	0.82904	0.83044	0.83494	0.83981	0.84522
514	0.81627	0.82514	0.82930	0.83069	0.83518	0.84005	0.84545
515	0.81654	0.82540	0.82955	0.83094	0.83542	0.84029	0.84568
516	0.81684	0.82566	0.82980	0.83119	0.83567	0.84053	0.84591
517	0.81710	0.82592	0.83005	0.83145	0.83592	0.84076	0.84613
518	0.81736	0.82619	0.83030	0.83169	0.83616	0.84099	0.84636
519	0.81766	0.82645	0.83056	0.83194	0.83640	0.84122	0.84658
520	0.81792	0.82670	0.83080	0.83219	0.83664	0.84146	0.84681
521	0.81820	0.82695	0.83106	0.83243	0.83689	0.84169	0.84703
522	0.81846	0.82720	0.83131	0.83268	0.83713	0.84192	0.84725
523	0.81876	0.82746	0.83156	0.83293	0.83736	0.84216	0.84748
524	0.81902	0.82772	0.83180	0.83317	0.83760	0.84238	0.84770
525	0.81928	0.82798	0.83205	0.83341	0.83784	0.84261	0.84792
526	0.81954	0.82823	0.83230	0.83367	0.83807	0.84284	0.84814
527	0.81979	0.82849	0.83254	0.83391	0.83831	0.84307	0.84836
528	0.82005	0.82873	0.83279	0.83415	0.83855	0.84330	0.84858
529	0.82032	0.82898	0.83303	0.83439	0.83878	0.84352	0.84880
530	0.82059	0.82924	0.83327	0.83463	0.83901	0.84375	0.84901
531	0.82087	0.82947	0.83351	0.83487	0.83924	0.84397	0.84923
532	0.82113	0.82972	0.83375	0.83511	0.83947	0.84420	0.84945
533	0.82138	0.82997	0.83400	0.83535	0.83970	0.84442	0.84966
534	0.82164	0.83022	0.83422	0.83559	0.83993	0.84465	0.84988
535	0.82188	0.83046	0.83447	0.83582	0.84016	0.84487	0.85009
536	0.82216	0.83071	0.83470	0.83606	0.84039	0.84509	0.85030
537	0.82240	0.83095	0.83494	0.83629	0.84062	0.84531	0.85051
538	0.82265	0.83120	0.83518	0.83653	0.84085	0.84553	0.85073
539	0.82293	0.83145	0.83542	0.83676	0.84108	0.84575	0.85093
540	0.82316	0.83168	0.83565	0.83699	0.84130	0.84597	0.85115
541	0.82343	0.83193	0.83588	0.83722	0.84153	0.84619	0.85136
542	0.82367	0.83217	0.83611	0.83745	0.84175	0.84641	0.85156
543	0.82393	0.83241	0.83635	0.83768	0.84198	0.84663	0.85177
544	0.82417	0.83265	0.83658	0.83791	0.84220	0.84684	0.85198
545	0.82441	0.83289	0.83681	0.83814	0.84242	0.84706	0.85219
546	0.82467	0.83312	0.83704	0.83837	0.84264	0.84727	0.85240
547	0.82491	0.83335	0.83727	0.83860	0.84287	0.84748	0.85261
548	0.82518	0.83359	0.83750	0.83883	0.84309	0.84770	0.85281
549	0.82541	0.83384	0.83773	0.83905	0.84331	0.84792	0.85302
550	0.82566	0.83407	0.83797	0.83928	0.84353	0.84812	0.85322
551	0.82593	0.83430	0.83819	0.83951	0.84375	0.84834	0.85343
552	0.82616	0.83453	0.83842	0.83973	0.84397	0.84855	0.85363
553	0.82642	0.83476	0.83865	0.83996	0.84419	0.84876	0.85383
554	0.82665	0.83500	0.83888	0.84018	0.84440	0.84897	0.85404
555	0.82690	0.83522	0.83910	0.84040	0.84462	0.84918	0.85424
556	0.82714	0.83546	0.83932	0.84062	0.84483	0.84938	0.85444
557	0.82741	0.83568	0.83955	0.84084	0.84505	0.84959	0.85463
558	0.82764	0.83591	0.83977	0.84106	0.84526	0.84980	0.85484
559	0.82789	0.83614	0.83999	0.84128	0.84547	0.85001	0.85503
560	0.82811	0.83637	0.84021	0.84151	0.84569	0.85021	0.85523
561	0.82834	0.83660	0.84043	0.84173	0.84590	0.85042	0.85543
562	0.82857	0.83683	0.84065	0.84194	0.84611	0.85062	0.85563
563	0.82881	0.83705	0.84087	0.84216	0.84632	0.85083	0.85582
564	0.82906	0.83728	0.84109	0.84237	0.84653	0.85103	0.85602
565	0.82928	0.83750	0.84131	0.84259	0.84675	0.85123	0.85621
566	0.82955	0.83773	0.84152	0.84281	0.84696	0.85143	0.85641

N	0.1%	0.5%	1%	1.25%	2.5%	5%	10%
567	0.82976	0.83795	0.84175	0.84303	0.84717	0.85164	0.85660
568	0.82999	0.83817	0.84196	0.84324	0.84737	0.85184	0.85679
569	0.83023	0.83838	0.84217	0.84346	0.84758	0.85204	0.85699
570	0.83045	0.83860	0.84238	0.84366	0.84778	0.85224	0.85718
571	0.83068	0.83883	0.84260	0.84387	0.84799	0.85244	0.85737
572	0.83092	0.83904	0.84282	0.84408	0.84820	0.85264	0.85756
573	0.83113	0.83926	0.84303	0.84429	0.84840	0.85283	0.85775
574	0.83135	0.83948	0.84324	0.84450	0.84860	0.85303	0.85794
575	0.83156	0.83969	0.84345	0.84471	0.84881	0.85323	0.85813
576	0.83181	0.83991	0.84366	0.84492	0.84901	0.85343	0.85832
577	0.83204	0.84012	0.84387	0.84513	0.84921	0.85362	0.85851
578	0.83225	0.84034	0.84408	0.84534	0.84941	0.85382	0.85870
579	0.83249	0.84055	0.84428	0.84554	0.84961	0.85401	0.85889
580	0.83271	0.84077	0.84449	0.84575	0.84981	0.85420	0.85907
581	0.83294	0.84097	0.84470	0.84596	0.85002	0.85439	0.85926
582	0.83317	0.84119	0.84490	0.84616	0.85021	0.85459	0.85945
583	0.83339	0.84140	0.84511	0.84636	0.85041	0.85478	0.85963
584	0.83364	0.84161	0.84532	0.84657	0.85061	0.85497	0.85982
585	0.83385	0.84182	0.84553	0.84677	0.85081	0.85516	0.86000
586	0.83407	0.84202	0.84573	0.84698	0.85101	0.85535	0.86018
587	0.83430	0.84222	0.84594	0.84718	0.85120	0.85554	0.86036
588	0.83451	0.84244	0.84614	0.84738	0.85140	0.85573	0.86054
589	0.83472	0.84265	0.84634	0.84758	0.85160	0.85592	0.86073
590	0.83495	0.84286	0.84655	0.84779	0.85179	0.85611	0.86091
591	0.83516	0.84306	0.84675	0.84799	0.85199	0.85630	0.86109
592	0.83535	0.84327	0.84695	0.84819	0.85218	0.85649	0.86127
593	0.83559	0.84348	0.84715	0.84838	0.85237	0.85667	0.86145
594	0.83581	0.84369	0.84736	0.84858	0.85256	0.85686	0.86163
595	0.83601	0.84389	0.84756	0.84878	0.85275	0.85705	0.86181
596	0.83623	0.84409	0.84776	0.84898	0.85294	0.85723	0.86199
597	0.83645	0.84430	0.84796	0.84918	0.85313	0.85741	0.86217
598	0.83668	0.84450	0.84816	0.84937	0.85333	0.85760	0.86234
599	0.83690	0.84471	0.84835	0.84957	0.85351	0.85778	0.86252
600	0.83710	0.84491	0.84855	0.84977	0.85370	0.85796	0.86270
601	0.83732	0.84510	0.84874	0.84996	0.85389	0.85814	0.86287
602	0.83752	0.84531	0.84894	0.85015	0.85408	0.85833	0.86305
603	0.83774	0.84551	0.84913	0.85034	0.85427	0.85851	0.86322
604	0.83793	0.84570	0.84932	0.85054	0.85445	0.85869	0.86340
605	0.83814	0.84590	0.84952	0.85073	0.85465	0.85887	0.86357
606	0.83836	0.84610	0.84971	0.85092	0.85483	0.85905	0.86374
607	0.83856	0.84630	0.84990	0.85111	0.85502	0.85923	0.86392
608	0.83877	0.84649	0.85009	0.85130	0.85520	0.85941	0.86409
609	0.83896	0.84669	0.85029	0.85149	0.85538	0.85959	0.86426
610	0.83918	0.84689	0.85048	0.85168	0.85556	0.85977	0.86443
611	0.83937	0.84708	0.85066	0.85187	0.85575	0.85994	0.86460
612	0.83959	0.84728	0.85085	0.85205	0.85593	0.86012	0.86477
613	0.83978	0.84748	0.85104	0.85224	0.85611	0.86030	0.86495
614	0.83999	0.84767	0.85123	0.85243	0.85629	0.86047	0.86511
615	0.84018	0.84786	0.85143	0.85262	0.85647	0.86065	0.86528
616	0.84039	0.84805	0.85162	0.85280	0.85665	0.86083	0.86545
617	0.84058	0.84826	0.85180	0.85299	0.85684	0.86100	0.86562
618	0.84079	0.84844	0.85199	0.85317	0.85702	0.86118	0.86579
619	0.84099	0.84864	0.85217	0.85336	0.85720	0.86135	0.86596
620	0.84122	0.84883	0.85236	0.85354	0.85737	0.86153	0.86612
621	0.84140	0.84902	0.85254	0.85372	0.85755	0.86170	0.86629
622	0.84162	0.84921	0.85272	0.85390	0.85773	0.86187	0.86646
623	0.84181	0.84939	0.85291	0.85409	0.85791	0.86204	0.86662
624	0.84200	0.84957	0.85309	0.85427	0.85809	0.86221	0.86679
625	0.84219	0.84977	0.85327	0.85445	0.85826	0.86238	0.86695
626	0.84241	0.84995	0.85346	0.85463	0.85844	0.86256	0.86712
627	0.84261	0.85013	0.85364	0.85482	0.85861	0.86272	0.86728
628	0.84281	0.85031	0.85382	0.85500	0.85879	0.86289	0.86744
629	0.84299	0.85051	0.85401	0.85518	0.85896	0.86306	0.86761
630	0.84318	0.85070	0.85418	0.85536	0.85914	0.86323	0.86777
631	0.84339	0.85088	0.85437	0.85553	0.85931	0.86340	0.86793
632	0.84360	0.85107	0.85455	0.85571	0.85948	0.86357	0.86809
633	0.84378	0.85125	0.85473	0.85589	0.85966	0.86374	0.86825
634	0.84397	0.85144	0.85491	0.85607	0.85983	0.86390	0.86841
635	0.84416	0.85162	0.85509	0.85625	0.86000	0.86406	0.86857
636	0.84436	0.85180	0.85526	0.85643	0.86017	0.86423	0.86873
637	0.84455	0.85198	0.85544	0.85660	0.86034	0.86440	0.86889
638	0.84475	0.85216	0.85562	0.85677	0.86051	0.86456	0.86905
639	0.84493	0.85234	0.85580	0.85694	0.86068	0.86473	0.86921
640	0.84512	0.85253	0.85597	0.85711	0.86085	0.86489	0.86937

N	0.1%	0.5%	1%	1.25%	2.5%	5%	10%
641	0.84530	0.85271	0.85615	0.85729	0.86102	0.86505	0.86952
642	0.84549	0.85289	0.85632	0.85746	0.86118	0.86521	0.86968
643	0.84567	0.85307	0.85650	0.85764	0.86135	0.86538	0.86984
644	0.84586	0.85325	0.85667	0.85781	0.86152	0.86554	0.86999
645	0.84604	0.85342	0.85684	0.85798	0.86169	0.86570	0.87015
646	0.84623	0.85360	0.85701	0.85816	0.86185	0.86586	0.87030
647	0.84640	0.85378	0.85719	0.85832	0.86202	0.86602	0.87046
648	0.84659	0.85395	0.85736	0.85850	0.86219	0.86619	0.87061
649	0.84678	0.85413	0.85753	0.85867	0.86236	0.86634	0.87077
650	0.84698	0.85431	0.85770	0.85884	0.86252	0.86650	0.87092
651	0.84715	0.85448	0.85787	0.85900	0.86269	0.86666	0.87107
652	0.84732	0.85466	0.85803	0.85917	0.86285	0.86682	0.87123
653	0.84751	0.85483	0.85820	0.85934	0.86302	0.86698	0.87138
654	0.84770	0.85501	0.85837	0.85950	0.86318	0.86714	0.87153
655	0.84789	0.85518	0.85854	0.85967	0.86334	0.86730	0.87168
656	0.84806	0.85535	0.85871	0.85984	0.86350	0.86746	0.87183
657	0.84824	0.85552	0.85888	0.86001	0.86367	0.86761	0.87199
658	0.84841	0.85570	0.85905	0.86018	0.86382	0.86777	0.87214
659	0.84859	0.85587	0.85921	0.86034	0.86399	0.86793	0.87229
660	0.84878	0.85604	0.85937	0.86050	0.86414	0.86808	0.87244
661	0.84896	0.85621	0.85954	0.86067	0.86430	0.86824	0.87259
662	0.84913	0.85638	0.85971	0.86083	0.86446	0.86839	0.87274
663	0.84930	0.85655	0.85987	0.86100	0.86462	0.86854	0.87288
664	0.84949	0.85671	0.86004	0.86116	0.86478	0.86870	0.87303
665	0.84967	0.85689	0.86021	0.86132	0.86494	0.86885	0.87318
666	0.84986	0.85706	0.86037	0.86149	0.86510	0.86901	0.87333
667	0.85005	0.85723	0.86054	0.86164	0.86526	0.86916	0.87348
668	0.85020	0.85739	0.86070	0.86181	0.86542	0.86931	0.87362
669	0.85038	0.85756	0.86087	0.86197	0.86558	0.86946	0.87377
670	0.85057	0.85773	0.86103	0.86213	0.86573	0.86961	0.87392
671	0.85074	0.85789	0.86119	0.86229	0.86588	0.86977	0.87406
672	0.85092	0.85806	0.86135	0.86245	0.86604	0.86991	0.87421
673	0.85110	0.85822	0.86151	0.86261	0.86620	0.87007	0.87435
674	0.85128	0.85838	0.86167	0.86277	0.86635	0.87021	0.87450
675	0.85145	0.85854	0.86183	0.86293	0.86650	0.87036	0.87464
676	0.85163	0.85871	0.86198	0.86309	0.86666	0.87051	0.87479
677	0.85181	0.85888	0.86215	0.86325	0.86681	0.87066	0.87493
678	0.85197	0.85905	0.86231	0.86341	0.86696	0.87081	0.87507
679	0.85215	0.85921	0.86247	0.86356	0.86712	0.87096	0.87522
680	0.85232	0.85937	0.86262	0.86372	0.86727	0.87111	0.87536
681	0.85247	0.85953	0.86278	0.86387	0.86742	0.87125	0.87550
682	0.85266	0.85969	0.86294	0.86403	0.86757	0.87140	0.87564
683	0.85281	0.85986	0.86310	0.86418	0.86773	0.87155	0.87578
684	0.85299	0.86002	0.86326	0.86434	0.86788	0.87170	0.87592
685	0.85315	0.86018	0.86341	0.86450	0.86803	0.87184	0.87607
686	0.85331	0.86034	0.86357	0.86465	0.86818	0.87198	0.87620
687	0.85347	0.86051	0.86373	0.86481	0.86833	0.87213	0.87634
688	0.85365	0.86067	0.86389	0.86497	0.86848	0.87227	0.87648
689	0.85381	0.86082	0.86404	0.86512	0.86863	0.87242	0.87662
690	0.85398	0.86098	0.86420	0.86527	0.86878	0.87256	0.87676
691	0.85417	0.86114	0.86435	0.86542	0.86892	0.87271	0.87690
692	0.85434	0.86130	0.86450	0.86558	0.86907	0.87285	0.87704
693	0.85451	0.86146	0.86466	0.86573	0.86922	0.87299	0.87718
694	0.85466	0.86161	0.86481	0.86588	0.86936	0.87314	0.87731
695	0.85484	0.86177	0.86496	0.86603	0.86951	0.87328	0.87745
696	0.85501	0.86193	0.86512	0.86618	0.86966	0.87342	0.87759
697	0.85516	0.86209	0.86527	0.86633	0.86981	0.87356	0.87772
698	0.85532	0.86225	0.86542	0.86648	0.86995	0.87370	0.87786
699	0.85550	0.86241	0.86557	0.86663	0.87010	0.87385	0.87800
700	0.85566	0.86256	0.86572	0.86678	0.87024	0.87399	0.87813
701	0.85582	0.86271	0.86588	0.86692	0.87039	0.87412	0.87827
702	0.85597	0.86286	0.86603	0.86707	0.87053	0.87426	0.87840
703	0.85614	0.86301	0.86618	0.86722	0.87067	0.87440	0.87854
704	0.85630	0.86317	0.86633	0.86737	0.87082	0.87454	0.87867
705	0.85644	0.86333	0.86648	0.86752	0.87096	0.87468	0.87880
706	0.85661	0.86348	0.86662	0.86767	0.87111	0.87482	0.87894
707	0.85677	0.86363	0.86677	0.86782	0.87125	0.87496	0.87907
708	0.85693	0.86378	0.86692	0.86797	0.87140	0.87510	0.87920
709	0.85710	0.86393	0.86707	0.86812	0.87154	0.87524	0.87934
710	0.85727	0.86408	0.86722	0.86826	0.87168	0.87537	0.87947
711	0.85742	0.86423	0.86736	0.86840	0.87181	0.87551	0.87960
712	0.85758	0.86439	0.86751	0.86855	0.87196	0.87565	0.87973
713	0.85774	0.86454	0.86766	0.86869	0.87210	0.87578	0.87986
714	0.85790	0.86468	0.86780	0.86884	0.87224	0.87592	0.87999

N	0.1%	0.5%	1%	1.25%	2.5%	5%	10%
715	0.85805	0.86483	0.86795	0.86898	0.87238	0.87605	0.88012
716	0.85821	0.86497	0.86809	0.86912	0.87252	0.87619	0.88025
717	0.85836	0.86512	0.86824	0.86927	0.87266	0.87633	0.88038
718	0.85853	0.86527	0.86838	0.86942	0.87280	0.87646	0.88051
719	0.85870	0.86542	0.86853	0.86955	0.87293	0.87659	0.88064
720	0.85886	0.86556	0.86866	0.86970	0.87308	0.87673	0.88077
721	0.85901	0.86571	0.86881	0.86984	0.87321	0.87686	0.88090
722	0.85916	0.86586	0.86895	0.86998	0.87335	0.87699	0.88103
723	0.85930	0.86600	0.86910	0.87012	0.87349	0.87713	0.88116
724	0.85944	0.86615	0.86924	0.87027	0.87362	0.87726	0.88128
725	0.85961	0.86630	0.86938	0.87041	0.87376	0.87739	0.88141
726	0.85976	0.86644	0.86953	0.87055	0.87389	0.87752	0.88154
727	0.85991	0.86660	0.86967	0.87069	0.87403	0.87766	0.88167
728	0.86008	0.86674	0.86981	0.87083	0.87417	0.87779	0.88179
729	0.86022	0.86688	0.86996	0.87098	0.87430	0.87792	0.88192
730	0.86039	0.86702	0.87009	0.87111	0.87444	0.87805	0.88204
731	0.86055	0.86717	0.87023	0.87125	0.87458	0.87818	0.88217
732	0.86070	0.86731	0.87037	0.87139	0.87471	0.87831	0.88230
733	0.86084	0.86745	0.87051	0.87154	0.87484	0.87844	0.88242
734	0.86099	0.86759	0.87065	0.87167	0.87498	0.87857	0.88254
735	0.86115	0.86773	0.87079	0.87181	0.87511	0.87870	0.88267
736	0.86130	0.86787	0.87093	0.87195	0.87525	0.87883	0.88280
737	0.86146	0.86802	0.87106	0.87209	0.87538	0.87896	0.88292
738	0.86159	0.86816	0.87120	0.87222	0.87551	0.87909	0.88304
739	0.86174	0.86830	0.87134	0.87236	0.87564	0.87921	0.88317
740	0.86189	0.86845	0.87147	0.87249	0.87578	0.87934	0.88329
741	0.86204	0.86859	0.87161	0.87263	0.87591	0.87947	0.88341
742	0.86219	0.86873	0.87175	0.87276	0.87604	0.87960	0.88353
743	0.86235	0.86887	0.87188	0.87290	0.87617	0.87972	0.88366
744	0.86248	0.86901	0.87201	0.87303	0.87630	0.87985	0.88378
745	0.86263	0.86915	0.87215	0.87316	0.87643	0.87998	0.88390
746	0.86278	0.86929	0.87229	0.87330	0.87656	0.88010	0.88402
747	0.86294	0.86943	0.87242	0.87343	0.87669	0.88023	0.88414
748	0.86307	0.86957	0.87256	0.87357	0.87682	0.88035	0.88426
749	0.86321	0.86970	0.87270	0.87370	0.87695	0.88048	0.88438
750	0.86335	0.86984	0.87283	0.87383	0.87708	0.88060	0.88450
751	0.86350	0.86998	0.87297	0.87396	0.87721	0.88073	0.88462
752	0.86364	0.87012	0.87310	0.87410	0.87734	0.88085	0.88474
753	0.86377	0.87025	0.87323	0.87423	0.87747	0.88098	0.88486
754	0.86391	0.87039	0.87337	0.87436	0.87760	0.88110	0.88498
755	0.86406	0.87052	0.87350	0.87450	0.87772	0.88122	0.88510
756	0.86421	0.87066	0.87364	0.87463	0.87785	0.88135	0.88522
757	0.86435	0.87079	0.87377	0.87476	0.87798	0.88147	0.88534
758	0.86450	0.87093	0.87390	0.87489	0.87810	0.88159	0.88545
759	0.86463	0.87107	0.87403	0.87503	0.87823	0.88171	0.88557
760	0.86478	0.87120	0.87416	0.87516	0.87836	0.88183	0.88569
761	0.86493	0.87133	0.87429	0.87529	0.87848	0.88196	0.88581
762	0.86506	0.87147	0.87442	0.87542	0.87861	0.88208	0.88592
763	0.86520	0.87160	0.87455	0.87555	0.87874	0.88220	0.88604
764	0.86534	0.87174	0.87468	0.87567	0.87886	0.88232	0.88615
765	0.86547	0.87186	0.87481	0.87580	0.87898	0.88244	0.88627
766	0.86562	0.87200	0.87494	0.87593	0.87911	0.88256	0.88639
767	0.86576	0.87213	0.87507	0.87605	0.87923	0.88268	0.88650
768	0.86591	0.87226	0.87520	0.87618	0.87936	0.88280	0.88662
769	0.86605	0.87239	0.87533	0.87630	0.87948	0.88292	0.88673
770	0.86620	0.87252	0.87545	0.87643	0.87960	0.88304	0.88685
771	0.86634	0.87265	0.87558	0.87655	0.87972	0.88316	0.88696
772	0.86648	0.87279	0.87570	0.87668	0.87985	0.88328	0.88708
773	0.86661	0.87292	0.87583	0.87681	0.87997	0.88339	0.88719
774	0.86676	0.87304	0.87596	0.87694	0.88009	0.88351	0.88731
775	0.86688	0.87317	0.87608	0.87707	0.88021	0.88363	0.88742
776	0.86702	0.87330	0.87621	0.87719	0.88033	0.88375	0.88754
777	0.86717	0.87343	0.87634	0.87731	0.88046	0.88387	0.88765
778	0.86730	0.87355	0.87646	0.87744	0.88058	0.88398	0.88776
779	0.86744	0.87368	0.87659	0.87756	0.88070	0.88410	0.88787
780	0.86757	0.87381	0.87671	0.87768	0.88082	0.88422	0.88799
781	0.86770	0.87394	0.87684	0.87781	0.88094	0.88433	0.88810
782	0.86783	0.87407	0.87697	0.87793	0.88106	0.88445	0.88821
783	0.86797	0.87420	0.87709	0.87806	0.88118	0.88457	0.88832
784	0.86811	0.87433	0.87722	0.87818	0.88130	0.88468	0.88843
785	0.86824	0.87445	0.87734	0.87830	0.88142	0.88479	0.88854
786	0.86838	0.87458	0.87747	0.87842	0.88154	0.88491	0.88866
787	0.86851	0.87470	0.87759	0.87855	0.88166	0.88503	0.88877
788	0.86864	0.87484	0.87772	0.87866	0.88178	0.88514	0.88888

N	0.1%	0.5%	1%	1.25%	2.5%	5%	10%
789	0.86877	0.87496	0.87784	0.87879	0.88189	0.88526	0.88899
790	0.86891	0.87509	0.87796	0.87891	0.88201	0.88537	0.88910
791	0.86904	0.87521	0.87809	0.87903	0.88213	0.88549	0.88921
792	0.86918	0.87534	0.87821	0.87915	0.88225	0.88560	0.88932
793	0.86932	0.87547	0.87833	0.87927	0.88236	0.88571	0.88943
794	0.86944	0.87559	0.87845	0.87939	0.88248	0.88583	0.88954
795	0.86958	0.87571	0.87857	0.87951	0.88260	0.88594	0.88965
796	0.86971	0.87583	0.87869	0.87963	0.88272	0.88605	0.88975
797	0.86984	0.87595	0.87881	0.87975	0.88283	0.88617	0.88986
798	0.86998	0.87608	0.87892	0.87987	0.88295	0.88628	0.88997
799	0.87011	0.87620	0.87905	0.87999	0.88306	0.88639	0.89008
800	0.87023	0.87632	0.87916	0.88010	0.88318	0.88650	0.89019
801	0.87036	0.87644	0.87928	0.88022	0.88329	0.88661	0.89029
802	0.87050	0.87656	0.87940	0.88034	0.88341	0.88672	0.89040
803	0.87061	0.87669	0.87952	0.88046	0.88352	0.88683	0.89051
804	0.87075	0.87682	0.87964	0.88058	0.88364	0.88694	0.89061
805	0.87088	0.87694	0.87975	0.88069	0.88375	0.88705	0.89072
806	0.87102	0.87705	0.87987	0.88081	0.88386	0.88716	0.89083
807	0.87115	0.87717	0.87999	0.88093	0.88398	0.88727	0.89093
808	0.87127	0.87730	0.88011	0.88104	0.88409	0.88738	0.89104
809	0.87140	0.87742	0.88022	0.88116	0.88420	0.88749	0.89115
810	0.87153	0.87754	0.88034	0.88128	0.88432	0.88760	0.89125
811	0.87165	0.87766	0.88046	0.88140	0.88443	0.88771	0.89136
812	0.87178	0.87778	0.88058	0.88151	0.88454	0.88782	0.89146
813	0.87190	0.87790	0.88069	0.88162	0.88465	0.88793	0.89156
814	0.87203	0.87802	0.88081	0.88174	0.88476	0.88804	0.89167
815	0.87217	0.87814	0.88092	0.88186	0.88488	0.88815	0.89177
816	0.87229	0.87826	0.88104	0.88197	0.88499	0.88825	0.89188
817	0.87240	0.87838	0.88116	0.88209	0.88510	0.88836	0.89198
818	0.87254	0.87850	0.88127	0.88220	0.88521	0.88847	0.89209
819	0.87266	0.87862	0.88139	0.88231	0.88532	0.88858	0.89219
820	0.87279	0.87873	0.88150	0.88243	0.88543	0.88868	0.89229
821	0.87290	0.87885	0.88162	0.88254	0.88554	0.88879	0.89240
822	0.87303	0.87896	0.88173	0.88265	0.88565	0.88890	0.89250
823	0.87314	0.87908	0.88185	0.88277	0.88576	0.88900	0.89260
824	0.87326	0.87920	0.88196	0.88288	0.88587	0.88911	0.89270
825	0.87338	0.87931	0.88207	0.88300	0.88598	0.88921	0.89281
826	0.87351	0.87943	0.88218	0.88311	0.88609	0.88932	0.89291
827	0.87362	0.87955	0.88230	0.88322	0.88620	0.88942	0.89301
828	0.87375	0.87966	0.88241	0.88333	0.88631	0.88953	0.89311
829	0.87387	0.87978	0.88252	0.88344	0.88642	0.88964	0.89321
830	0.87399	0.87989	0.88264	0.88355	0.88653	0.88974	0.89331
831	0.87411	0.88001	0.88275	0.88366	0.88663	0.88984	0.89342
832	0.87424	0.88012	0.88286	0.88377	0.88674	0.88995	0.89351
833	0.87435	0.88024	0.88297	0.88389	0.88685	0.89005	0.89361
834	0.87448	0.88035	0.88308	0.88400	0.88696	0.89016	0.89371
835	0.87460	0.88046	0.88319	0.88411	0.88706	0.89026	0.89381
836	0.87471	0.88058	0.88331	0.88422	0.88717	0.89037	0.89391
837	0.87483	0.88070	0.88342	0.88433	0.88728	0.89047	0.89401
838	0.87496	0.88081	0.88353	0.88444	0.88738	0.89057	0.89411
839	0.87507	0.88093	0.88364	0.88455	0.88749	0.89067	0.89421
840	0.87521	0.88104	0.88375	0.88466	0.88760	0.89078	0.89431
841	0.87533	0.88115	0.88386	0.88477	0.88770	0.89088	0.89441
842	0.87544	0.88127	0.88397	0.88488	0.88781	0.89098	0.89451
843	0.87555	0.88138	0.88408	0.88499	0.88792	0.89108	0.89461
844	0.87567	0.88149	0.88419	0.88510	0.88802	0.89119	0.89471
845	0.87578	0.88160	0.88430	0.88520	0.88812	0.89129	0.89480
846	0.87590	0.88171	0.88441	0.88531	0.88823	0.89139	0.89490
847	0.87602	0.88182	0.88452	0.88542	0.88833	0.89149	0.89500
848	0.87614	0.88193	0.88463	0.88553	0.88844	0.89159	0.89510
849	0.87626	0.88204	0.88474	0.88563	0.88854	0.89169	0.89520
850	0.87637	0.88215	0.88484	0.88574	0.88864	0.89179	0.89529
851	0.87649	0.88226	0.88495	0.88584	0.88875	0.89189	0.89539
852	0.87661	0.88237	0.88506	0.88595	0.88885	0.89199	0.89549
853	0.87673	0.88248	0.88516	0.88606	0.88895	0.89209	0.89558
854	0.87684	0.88259	0.88527	0.88616	0.88906	0.89219	0.89568
855	0.87696	0.88270	0.88538	0.88627	0.88916	0.89229	0.89578
856	0.87708	0.88281	0.88548	0.88637	0.88926	0.89239	0.89587
857	0.87720	0.88292	0.88559	0.88647	0.88937	0.89249	0.89597
858	0.87731	0.88303	0.88570	0.88658	0.88947	0.89259	0.89606
859	0.87742	0.88314	0.88580	0.88669	0.88957	0.89269	0.89616
860	0.87753	0.88325	0.88591	0.88679	0.88967	0.89279	0.89625
861	0.87764	0.88336	0.88602	0.88690	0.88978	0.89289	0.89635
862	0.87776	0.88346	0.88612	0.88700	0.88988	0.89299	0.89644

N	0.1%	0.5%	1%	1.25%	2.5%	5%	10%
863	0.87787	0.88358	0.88623	0.88711	0.88998	0.89309	0.89654
864	0.87797	0.88368	0.88633	0.88721	0.89008	0.89318	0.89663
865	0.87809	0.88378	0.88644	0.88731	0.89018	0.89328	0.89672
866	0.87820	0.88389	0.88654	0.88742	0.89028	0.89338	0.89682
867	0.87832	0.88400	0.88665	0.88752	0.89038	0.89348	0.89691
868	0.87843	0.88411	0.88675	0.88763	0.89048	0.89357	0.89701
869	0.87853	0.88422	0.88685	0.88773	0.89058	0.89367	0.89710
870	0.87865	0.88433	0.88695	0.88783	0.89068	0.89377	0.89719
871	0.87876	0.88443	0.88705	0.88793	0.89078	0.89386	0.89728
872	0.87887	0.88454	0.88716	0.88803	0.89087	0.89396	0.89738
873	0.87900	0.88464	0.88726	0.88813	0.89097	0.89405	0.89747
874	0.87910	0.88475	0.88736	0.88824	0.89107	0.89415	0.89756
875	0.87922	0.88485	0.88747	0.88834	0.89117	0.89425	0.89765
876	0.87932	0.88495	0.88757	0.88844	0.89127	0.89434	0.89775
877	0.87943	0.88506	0.88767	0.88854	0.89137	0.89444	0.89784
878	0.87954	0.88516	0.88778	0.88864	0.89147	0.89453	0.89793
879	0.87965	0.88527	0.88787	0.88874	0.89157	0.89463	0.89802
880	0.87975	0.88537	0.88797	0.88884	0.89166	0.89472	0.89811
881	0.87986	0.88548	0.88808	0.88894	0.89176	0.89482	0.89821
882	0.87997	0.88558	0.88818	0.88904	0.89186	0.89491	0.89830
883	0.88007	0.88569	0.88828	0.88914	0.89196	0.89501	0.89839
884	0.88018	0.88579	0.88837	0.88924	0.89205	0.89510	0.89848
885	0.88029	0.88589	0.88848	0.88934	0.89215	0.89519	0.89857
886	0.88040	0.88599	0.88857	0.88944	0.89225	0.89529	0.89866
887	0.88050	0.88610	0.88867	0.88954	0.89234	0.89538	0.89875
888	0.88060	0.88620	0.88877	0.88964	0.89244	0.89547	0.89884
889	0.88071	0.88630	0.88887	0.88974	0.89253	0.89557	0.89893
890	0.88083	0.88641	0.88897	0.88984	0.89263	0.89566	0.89902
891	0.88095	0.88651	0.88907	0.88993	0.89273	0.89575	0.89911
892	0.88106	0.88661	0.88917	0.89003	0.89282	0.89584	0.89920
893	0.88115	0.88671	0.88927	0.89013	0.89292	0.89594	0.89929
894	0.88127	0.88681	0.88937	0.89023	0.89301	0.89603	0.89938
895	0.88138	0.88692	0.88947	0.89033	0.89311	0.89612	0.89946
896	0.88147	0.88702	0.88957	0.89043	0.89320	0.89621	0.89955
897	0.88158	0.88712	0.88967	0.89052	0.89330	0.89631	0.89964
898	0.88168	0.88722	0.88976	0.89062	0.89339	0.89640	0.89973
899	0.88179	0.88731	0.88986	0.89071	0.89349	0.89649	0.89982
900	0.88189	0.88742	0.88996	0.89081	0.89358	0.89658	0.89990
901	0.88199	0.88752	0.89006	0.89091	0.89368	0.89667	0.89999
902	0.88211	0.88762	0.89015	0.89100	0.89377	0.89676	0.90008
903	0.88222	0.88771	0.89025	0.89110	0.89386	0.89685	0.90017
904	0.88234	0.88782	0.89035	0.89120	0.89396	0.89694	0.90026
905	0.88243	0.88792	0.89044	0.89129	0.89405	0.89703	0.90034
906	0.88253	0.88802	0.89054	0.89139	0.89414	0.89712	0.90043
907	0.88263	0.88812	0.89064	0.89148	0.89424	0.89721	0.90052
908	0.88273	0.88821	0.89074	0.89158	0.89433	0.89730	0.90060
909	0.88284	0.88831	0.89083	0.89168	0.89442	0.89739	0.90069
910	0.88294	0.88841	0.89093	0.89177	0.89451	0.89748	0.90077
911	0.88305	0.88851	0.89102	0.89187	0.89461	0.89757	0.90086
912	0.88316	0.88861	0.89112	0.89196	0.89470	0.89766	0.90094
913	0.88326	0.88871	0.89121	0.89205	0.89479	0.89775	0.90103
914	0.88335	0.88880	0.89130	0.89215	0.89488	0.89784	0.90111
915	0.88346	0.88889	0.89140	0.89225	0.89497	0.89793	0.90120
916	0.88355	0.88899	0.89150	0.89234	0.89506	0.89801	0.90129
917	0.88367	0.88908	0.89159	0.89243	0.89515	0.89810	0.90137
918	0.88378	0.88918	0.89169	0.89252	0.89524	0.89819	0.90145
919	0.88388	0.88928	0.89178	0.89262	0.89534	0.89828	0.90154
920	0.88399	0.88938	0.89187	0.89271	0.89543	0.89836	0.90162
921	0.88408	0.88947	0.89197	0.89280	0.89552	0.89845	0.90171
922	0.88418	0.88957	0.89206	0.89290	0.89561	0.89854	0.90179
923	0.88428	0.88967	0.89216	0.89299	0.89569	0.89862	0.90188
924	0.88438	0.88977	0.89225	0.89308	0.89579	0.89871	0.90196
925	0.88448	0.88986	0.89234	0.89318	0.89588	0.89880	0.90205
926	0.88458	0.88996	0.89243	0.89327	0.89596	0.89889	0.90213
927	0.88468	0.89005	0.89253	0.89336	0.89605	0.89897	0.90221
928	0.88477	0.89014	0.89262	0.89345	0.89614	0.89906	0.90230
929	0.88488	0.89024	0.89271	0.89354	0.89623	0.89915	0.90238
930	0.88498	0.89033	0.89281	0.89363	0.89632	0.89923	0.90246
931	0.88509	0.89042	0.89290	0.89372	0.89641	0.89932	0.90255
932	0.88519	0.89052	0.89299	0.89381	0.89650	0.89940	0.90263
933	0.88529	0.89061	0.89308	0.89390	0.89659	0.89949	0.90271
934	0.88539	0.89071	0.89317	0.89399	0.89668	0.89957	0.90279
935	0.88549	0.89080	0.89326	0.89409	0.89676	0.89966	0.90288
936	0.88560	0.89089	0.89335	0.89418	0.89685	0.89974	0.90296

N	0.1%	0.5%	1%	1.25%	2.5%	5%	10%
937	0.88570	0.89098	0.89345	0.89427	0.89694	0.89982	0.90304
938	0.88579	0.89108	0.89354	0.89436	0.89703	0.89991	0.90312
939	0.88589	0.89117	0.89363	0.89445	0.89712	0.90000	0.90321
940	0.88598	0.89127	0.89372	0.89454	0.89720	0.90008	0.90329
941	0.88607	0.89136	0.89381	0.89463	0.89729	0.90017	0.90337
942	0.88616	0.89145	0.89390	0.89472	0.89738	0.90025	0.90345
943	0.88626	0.89154	0.89399	0.89481	0.89746	0.90034	0.90353
944	0.88636	0.89164	0.89408	0.89490	0.89755	0.90042	0.90361
945	0.88645	0.89173	0.89417	0.89499	0.89764	0.90050	0.90369
946	0.88655	0.89182	0.89426	0.89508	0.89772	0.90059	0.90377
947	0.88665	0.89191	0.89435	0.89517	0.89781	0.90067	0.90385
948	0.88676	0.89200	0.89444	0.89526	0.89789	0.90075	0.90393
949	0.88685	0.89209	0.89453	0.89535	0.89798	0.90084	0.90401
950	0.88695	0.89219	0.89462	0.89543	0.89807	0.90092	0.90409
951	0.88704	0.89227	0.89471	0.89552	0.89816	0.90100	0.90417
952	0.88714	0.89236	0.89480	0.89561	0.89824	0.90108	0.90425
953	0.88724	0.89245	0.89489	0.89570	0.89833	0.90117	0.90433
954	0.88733	0.89254	0.89498	0.89578	0.89841	0.90125	0.90441
955	0.88743	0.89263	0.89507	0.89587	0.89850	0.90133	0.90449
956	0.88752	0.89272	0.89515	0.89596	0.89858	0.90141	0.90457
957	0.88761	0.89281	0.89524	0.89604	0.89867	0.90149	0.90465
958	0.88771	0.89290	0.89532	0.89613	0.89875	0.90158	0.90473
959	0.88780	0.89299	0.89541	0.89622	0.89884	0.90166	0.90481
960	0.88790	0.89308	0.89550	0.89630	0.89892	0.90174	0.90488
961	0.88799	0.89317	0.89559	0.89639	0.89900	0.90182	0.90496
962	0.88809	0.89326	0.89567	0.89648	0.89909	0.90190	0.90504
963	0.88819	0.89335	0.89576	0.89657	0.89917	0.90198	0.90512
964	0.88828	0.89344	0.89585	0.89665	0.89925	0.90206	0.90520
965	0.88838	0.89353	0.89594	0.89674	0.89934	0.90215	0.90528
966	0.88846	0.89362	0.89602	0.89682	0.89942	0.90223	0.90535
967	0.88857	0.89371	0.89611	0.89691	0.89950	0.90231	0.90543
968	0.88865	0.89379	0.89619	0.89699	0.89958	0.90239	0.90551
969	0.88874	0.89388	0.89628	0.89708	0.89966	0.90247	0.90558
970	0.88884	0.89397	0.89637	0.89717	0.89975	0.90255	0.90566
971	0.88894	0.89406	0.89645	0.89725	0.89983	0.90263	0.90574
972	0.88901	0.89415	0.89654	0.89734	0.89991	0.90271	0.90581
973	0.88910	0.89423	0.89663	0.89742	0.90000	0.90279	0.90589
974	0.88920	0.89432	0.89671	0.89751	0.90008	0.90287	0.90597
975	0.88929	0.89441	0.89680	0.89759	0.90016	0.90294	0.90604
976	0.88937	0.89450	0.89688	0.89767	0.90024	0.90302	0.90612
977	0.88946	0.89458	0.89696	0.89776	0.90032	0.90310	0.90620
978	0.88955	0.89467	0.89705	0.89784	0.90040	0.90318	0.90627
979	0.88964	0.89475	0.89713	0.89792	0.90049	0.90326	0.90635
980	0.88974	0.89484	0.89721	0.89801	0.90057	0.90334	0.90642
981	0.88982	0.89492	0.89730	0.89809	0.90065	0.90342	0.90650
982	0.88991	0.89501	0.89738	0.89817	0.90073	0.90350	0.90658
983	0.89000	0.89510	0.89747	0.89825	0.90081	0.90358	0.90665
984	0.89010	0.89518	0.89755	0.89834	0.90089	0.90365	0.90673
985	0.89018	0.89527	0.89764	0.89842	0.90097	0.90373	0.90680
986	0.89027	0.89535	0.89772	0.89850	0.90106	0.90381	0.90687
987	0.89037	0.89543	0.89780	0.89858	0.90113	0.90389	0.90695
988	0.89045	0.89552	0.89789	0.89866	0.90121	0.90397	0.90703
989	0.89054	0.89561	0.89797	0.89875	0.90129	0.90404	0.90710
990	0.89063	0.89569	0.89805	0.89883	0.90137	0.90412	0.90717
991	0.89071	0.89578	0.89813	0.89891	0.90145	0.90420	0.90725
992	0.89080	0.89586	0.89821	0.89900	0.90153	0.90427	0.90732
993	0.89090	0.89595	0.89830	0.89908	0.90161	0.90435	0.90740
994	0.89098	0.89603	0.89838	0.89916	0.90169	0.90443	0.90747
995	0.89107	0.89612	0.89846	0.89924	0.90177	0.90450	0.90754
996	0.89115	0.89620	0.89854	0.89932	0.90185	0.90458	0.90762
997	0.89124	0.89629	0.89862	0.89941	0.90193	0.90466	0.90769
998	0.89133	0.89637	0.89870	0.89948	0.90201	0.90473	0.90777
999	0.89142	0.89645	0.89879	0.89956	0.90209	0.90481	0.90784
1000	0.89151	0.89654	0.89887	0.89964	0.90217	0.90488	0.90791
